# Comparative genomics of the *Streptomyces* genus: insights into multi-stress-resistant genes for bioremediation

**DOI:** 10.1007/s11274-025-04703-1

**Published:** 2025-12-02

**Authors:** Ajit Kumar Passari, Carlos Caicedo-Montoya, Monserrat Manzo-Ruiz, María Paula Gomez-Roman, Beatriz Ruiz-Villafán, José Fausto Rivero-Cruz, Bhim Pratap Singh, Romina Rodríguez-Sanoja, Sergio Sanchez

**Affiliations:** 1 Instituto de Investigaciones Biomédicas, Universidad Nacional Autónoma de México, Ciudad de México, 04510 México; 2 Grupo de Bioprocesos, Departamento de Ingeniería Química, Universidad de Antioquia, Calle 70 No. 52-21, Medellín, 050010 Colombia; 3 Centro de Innovación para el Desarrollo Apícola Sustentable de Quintana Roo, Universidad Intercultural Maya de Quintana Roo, Carretera Muna-Felipe Carrillo Puerto Km. 137 S/N, Quintana Roo, 03940 México; 4 Department of Agriculture and Environmental Sciences, National Institute of Food Technology Entrepreneurship and Management (NIFTEM), Sonepat, 131028 Haryana India

**Keywords:** *Streptomyces*, Genome, Biosynthetic gene clusters, Secondary metabolites, Stress response

## Abstract

The ability to produce secondary metabolites, enzymes, and other important compounds for various industrial, agricultural, pharmaceutical, and biotechnological applications is a defining characteristic of actinobacteria, one of the most significant microbial phyla among prokaryotes. However, little attention has been given to the relationship between the secondary metabolites of actinobacteria and their stress responses compared to those of other bacteria. In this study, we examined the genomes of two *Streptomyces* species, along with nine reference genomes, to investigate the biosynthetic gene clusters (BGCs) responsible for secondary metabolite production, their potential roles in stress responses, and their functional diversity in this unique genus. Our approach included bioaccumulation and biosorption studies, complemented by analytical techniques such as scanning electron microscopy (SEM), energy-dispersive spectroscopy (EDS), Fourier transform infrared spectroscopy (FT-IR), and inductively coupled plasma-optical emission spectroscopy (ICP-OES). We observed strain-specific variations in tolerance to heavy metals, including zinc, cobalt, copper, and cadmium, which were validated by experimental studies across both genomes. This report represents the first genomic study of heavy metal resistance in *S. thermocarboxydus*, providing valuable insights into its potential applications for bioremediation in metal-contaminated environments.

**Supplementary Information:**

The online version contains supplementary material available at 10.1007/s11274-025-04703-1.

## Key points

**-**Tolerance of *Streptomyces thermocarboxydus* to heavy metals.

- Comparative genome sequences analysis of two *Streptomyces thermocarboxydus* strains.

- Metal biosorption efficiency of two *Streptomyces thermocarboxydus* strains.

**Supplementary Information:**

The online version contains supplementary material available at 10.1007/s11274-025-04703-1.

## Introduction

The Actinomycetes class comprises Gram-positive, filamentous, unicellular microorganisms with a high GC content (over 55%). These microorganisms produce a diverse array of secondary metabolites, many of which are of significant relevance to human health and the clinical industry (Barka et al. 2016). Among Actinobacteria, the genus* Streptomyces* is renowned for producing a wide range of bioactive secondary metabolites and novel antibiotics (Liu et al. 2018). The genome of *Streptomyces coelicolor* was the first reported genome sequence of Actinomycetes (Bentley et al. 2002).

The field of genomics has experienced a remarkable expansion in recent years, driven by advancements in technologies such as next-generation sequencing. In this context, whole-genome sequencing provides an unprecedented wealth of information that can aid in discovering natural products in microorganisms (Rajwani et al. 2021; Majer et al. 2021). Bioinformatics tools have significantly enhanced research on secondary metabolites. Genome mining, a powerful approach for uncovering hidden and novel biosynthetic genes in *Streptomyces* species, is facilitated by tools such as the antiSMASH database, which serves as a comprehensive resource on biosynthetic gene clusters (Blin et al. 2023; Nielsen et al. 2017). This approach enables the identification of multiple gene clusters and supports the prediction of the core chemical structures and functions of the molecules they encode (Kenshole 2021, Nielsen et al. 2017).

In natural environments, secondary metabolites play a critical role as signaling molecules, mediating interactions within microbial communities and contributing to processes such as stress response and biofilm formation (Armes et al. 2022).

Heavy metal pollution is a significant environmental issue in marine, terrestrial, and freshwater habitats (Zaynab, 2022). The human body can be exposed to these metals through food, water, and air, potentially leading to chronic and acute health disorders (Mitra et al. 2022). To address this problem, new, more cost-effective technologies based on microorganisms, such as bioaccumulation and biosorption, have been developed to reduce heavy metal concentrations in industrial wastewater and improve the quality of treated effluent. Bacteria, fungi, and algae are some of the organisms that can mitigate heavy metal toxicity through various mechanisms, including uptake, oxidation, adsorption, and reduction (Singh et al. 2023). Research suggests that due to their metabolic diversity, actinobacteria could be employed as a bioremediation tool (Musani and Das 2024; Devanshi et al. 2022).

In this study, we conducted a comparative analysis of the genome sequences of two *Streptomyces thermocarboxydus* isolates (strains K155 and BPSAC147) from distinct microbial origins, along with nine other *Streptomyces* species. We focused on analyzing the metabolic functions of the genes responsible for producing secondary metabolites. Additionally, we examined the genetic and physiological factors that contribute to these organisms’ adaptability to their environment, including their responses to abiotic stressors such as heavy metals. Our analysis also included the study of genes associated with heavy metal resistance, including those for zinc, cobalt, copper, and cadmium, in *S. thermocarboxydus* strains isolated in Mexico and India. This was done to assess their potential for metal biosorption, which could enhance bioremediation processes.

## Materials and methods

### Sample collection

*S. thermocarboxydus* K155 was originally isolated from the Mexican soil of Valle de Chalco, State of Mexico, and deposited at the (WDCM48) IIBM-UNAM Culture Collection, Cd. MX 04510 (Avalos-García 2010). The strain was re-streaked on tryptic soy agar (TSA) (Sigma Aldrich, St Louis MO) media and incubated at 28 °C for spore production. Similarly, *S. thermocarboxydus* strain BPSAC147 was isolated from the root tissues of *Rhynchotechum ellipticum* plants from the Dampa Tiger Reserve Forest (DTRF) of Mizoram, India (Passari et al. 2019), re-streaked onto the same TSA medium, and incubated at 28 °C for spore production. Fully grown spores from both strains were washed with 0.8% saline solution and stored in 20% glycerol at −20 °C.

### Genomic DNA isolation and whole-genome sequencing

Strain K155 was grown in 250-mL Erlenmeyer baffled flasks containing 50 mL TSB broth at 28 °C for 2 days. The total grown cells were centrifuged at 15600x*g* for 10 min, and the pellet was used to isolate the genomic DNA. Genomic DNA from *S. thermocarboxydus* K155 was obtained using standard phenol: chloroform methods according to Kieser et al. (2000). A triple-sequencing approach was used for the draft genome. Illumina libraries were prepared with MiSeq through BaseClear Company (BaseClear, Neatherlands). Approximately 1.9 Gb and 18.6 million 50 bp-long pair-end reads were obtained. Roche 454 pyrosequencing data were generated by MOgene, USA, using the 454 FLX sequencing system. 454 paired-end sequences contained 318,310 good-quality reads of 368 bp length. The third round of sequencing was performed using Single Molecule, Real-Time (SMRT) sequencing from Pacific Biosciences (PacBio) via the Yale University, Yale Center for Genomic Analysis, USA. A total of 28,547 filtered reads with a mean length of 5,060 bp were obtained. Moreover, genomic DNA extraction and genome sequencing of *S. thermocarboxydus* strain BPSAC147 were performed at Genotypic Private Limited, Bangalore, India using the Illumina MiSeq platform (Passari et al. 2019). After sequencing, the Illumina reads were quality-checked using FastQC (http://www.bioinformatics.babraham.ac.uk/projects/fastqc), and the resulting sequences were filtered for low-quality reads using the default settings in the Trim Galore v0.5.0 program (https://www.bioinformatics.babraham.ac.uk/projects/trim_galore.).

### Genome Assembly, annotation and proteome comparisons

Illumina reads of *S. thermocarboxydus* K155were firstly assembled with an in-house python script using Ray (Boisvert et al. 2010), Edena (Hernandez et al. 2008), and Velvet (Zerbino and Birney 2008). Meanwhile, 454 reads were assembled with Newbler (Margulies et al. 2005). Subsequently, a hybrid assembly of Illumina and 454 assemblies were performed using Newbler, resulting in four scaffolds. Assembly of PacBio sequences was performed using the Hierarchical Genome Assembly Process (HGAP). The draft genome of *S. thermocarboxydus* K155 was polished with PacBio reads using PBJelly version v15.8.24 (https://github.com/esrice/PBJelly) (English et al. 2012), obtaining a single scaffold. On the other hand, de novo genome assembly of *S. thermocarboxydus* BPSAC147 was performed using Unicycler v0.4.8 (Wick et al. 2017). The genomes of *S. thermocarboxydus* K155 and BPSAC147 were annotated using the Rapid Annotations Subsystems Technology (RAST) version 4.0 (https://rast.nmpdr.org/) (Aziz et al. 2008).

Further, the genomes of *S. thermocarboxydus* strains K155 and BPSAC147 were analyzed using the Bioinformatics Resource Center (BRC) version 3.5.43 from the Pathosystems Resource Integration Center (PATRIC) (Wattam et al. 2017). The 16 S rRNA gene sequences of *S. thermocarboxydus* strains K155 and BPSAC147 were analyzed using the BLASTN tool in the PATRIC database to identify closely related reference genomes. Based on this analysis, nine genomes with the highest similarity were selected for further study, which also corresponds to the maximum number of genomes allowed in the PATRIC Proteome Comparison tool. Subsequently, the genome sequences of strains K155 and BPSAC147, along with these selected reference genomes, were analyzed using the Proteome Comparison tool in PATRIC. The strains used for proteome comparison were *Streptomyces* sp. ZS0098, *Streptomyces* sp. BSE7F, *Streptomyces* sp. SMS_SU21, *Actinospica acidiphila* NRRL B-24,431, *Streptomyces* sp. HNS054, *Streptomyces* sp. 4 F, *Streptomyces* sp. ETH9427, *Streptomyces* sp. UNC401CLCol, and *Streptomyces* sp. FxanaD5. Proteome comparisons were performed using the PATRIC Proteome Comparison tool with Pearson pairwise average linkage correlation, applying the default parameters. Orthologous group families were analyzed using FIGFams assignments available in PATRIC, which are based on sequence similarity and functional annotation thresholds defined by the database. No additional cutoffs were applied.

###  Secondary metabolite biosynthetic gene cluster (SMGC) and heavy metal resistance gene detection and identification

The annotated genome sequence of *S. thermocarboxydus* strains K155 and BPSAC147, as well as the reference nine genome sequences, were submitted to antiSMASH version 7.1.0.1 (Blin et al. 2023) for the detection of BGCs and to compare the putative heavy metal resistance genes using PATRIC and RAST databases (Davis et al. 2020; Aziz et al. 2008).

## COG analysis

The genome sequence of *S. thermocarboxydus* strains K155 and BPSAC147, as well as the reference nine genome sequences, were categorized according to their biological function in the cluster of orthologous groups (COG) database (Galperin et al. 2020).

### Core-Pan genome and Kyoto encyclopedia of genes and genomes (KEGG) database analysis

For this purpose, the Bacterial Pan Genome Analysis pipeline (BPGA) (Chaudhari et al. 2016) was used. This analysis consisted of clustering protein sequences that shared more than 50% similarity using USEARCH (Edgar, 2010) as the clustering algorithm, followed by the determination of affiliations of the different gene clusters to the pangenome and core genome. Afterward, using the core gene information, a phylogenetic tree was built from 2644 concatenated genes. For this, we extracted all genes annotated in the core genome and discarded those annotated as hypothetical proteins. Then, we aligned the selected genes using MAFFT (Katoh and Standley, 2013) with the default parameters, and used the Neighbor-Joining (NJ) algorithm implemented in MEGA-X to sketch the final phylogram (Kumar et al. 2018). A pan-genome phylogeny was constructed from the presence/absence matrix generated by BPGA using the NJ algorithm. BPGA reported additional information about the pangenome, such as the size of the core and pangenome, the mathematical equation for the Heap Law, and the determination of whether the pangenome was open or closed.

### Stress response analysis

The genomes were annotated in the KEGG database using the online tool Kofam KOALA (Aramaki et al. 2020). We further analyzed in detail the KEGG annotations for both strains, S. thermocarboxydus K155 and BPSAC147, using the Reconstruct Pathway (https://www.genome.jp/kegg/tool/map_pathway.html) to detect differences across diverse metabolic pathways. On the other hand, to identify stress-response genes in *Streptomyces* strains, we manually reviewed the literature and inspected KEGG annotations for different stresses. We counted the number of proteins that shared a KEGG term as evidence of copy number. The research included genomic responses to oxidative, heavy metal, and hyperosmotic stress, illumination, and cell wall disruption. The results were summarized using heat maps generated with the Python library Seaborn.

### Determination of heavy metal tolerance

#### Effect of heavy metals on ***Streptomyces*** growth

Zinc, cobalt, copper, and cadmium were selected based on the presence of heavy metal resistance genes identified in the whole-genome analysis to assess their impact on bacterial growth. The concentrations of the heavy metals ranged from 25 to 1000 µg/ml. The bacterial inoculum (2.1*10^3^ CFU/ml) was inoculated in 50 ml of tryptic soya broth (TSB) containing individual metals added at various concentrations. The bacterial strain was inoculated in TSB broth as the control without heavy metals. The flasks were then incubated at 28 °C for five days in a continuous orbital shaker (200 rpm). Growth was monitored by measuring optical density (OD₆₂₀ nm) at 0, 24, 48, 72, 96, and 120 h using a Multiscan spectrophotometer (Thermo Fisher Scientific, Germany). All experiments were conducted in triplicate, and results are presented as mean values (Sedláková-Kaduková et al. 2019).

##### Biomass production

*Streptomyces* strains were prepared according to the Saurav and Kannabiran (2011) protocol with slight modifications. Briefly, the strains were inoculated into 500-ml Erlenmeyer flasks containing 150 ml of TSB broth and incubated at 28 °C for 5 days on a continuous orbital shaker at 200 rpm. After growing, each culture was centrifuged at 12,000 rpm for 10 min. For the bioaccumulation study, the biomass was washed three times with saline solution and analyzed directly. For the biosorption study, the biomass was washed three times with saline solution and dried using a freezer-dryer for further analysis.

##### Bioaccumulation study

###### Scanning electron microscope (SEM) and energy dispersive X-ray (EDX) analysis

The metal-treated and untreated bacterial cells were analyzed by scanning electron microscope (SEM). The *Streptomyces* strains were inoculated into TSB broth containing zinc (1000 mg/L), cobalt (500 mg/L), copper (100 mg/L), and cadmium (50 mg/L) in individual flasks. In contrast, each strain was inoculated into TSB broth without heavy metals in a separate flask, serving as a control. All flasks were incubated at 28 °C for five days at 200 rpm. After growing the cells, the cultures were centrifuged at 10,000 rpm for 10 min. The obtained pellet was washed with 100 mM phosphate buffer (pH-7.4) several times. The samples were fixed with 3.0% glutaraldehyde for 5 min and then washed three times with 100 mM phosphate buffer (pH-7.4). After that, the samples were dehydrated by adding ethanol at different gradient levels (30%, 50%, 70%, 90%, and 100%) for 10–15 min. The obtained pellet was dried in a Freeze dryer and used for SEM analysis. The sample was mounted on an aluminum stub and coated with a thin layer of gold for 15 min. All treated and untreated samples were analyzed to observe morphological changes under SEM (JSM-5900LV, JEOL, Japan). The metal concentration was determined by EDX (Oxford ULTIM MX, JEOL, England) at an operating voltage of 20 kV, even though sections were element mapped by an EDX detector (Dhanwal et al. 2018).

##### Biosorption study

###### ICP-MS analysis

A Biosorption study was carried out using 50 ml of TSB broth supplemented with each heavy metal: zinc (1000 mg/L), cobalt (500 mg/L), copper (100 mg/L), and cadmium (50 mg/L). The flasks were inoculated with 2.5 g/L biomass and incubated at 28 °C for 5 days at 200 rpm. The solution was centrifuged at 10,000 rpm for 10 min. The pellet was used to determine the initial and final concentrations of each heavy metal using an inductively coupled plasma mass spectrometer (ICP-MS NexION 2000, Perkin Elmer). The TSB broth, free of heavy metals and bacterial biomass, was used as a control. For each metal, the system was maintained under inert conditions with argon gas at a flow rate of 15 L through the plasma, plus 1.2 L and 0.9 L per minute from an auxiliary nebulizer throughout the analysis. The pressure was controlled between 60 and 70 psi. Plasma formation was carried out at 6,000 °C to separate the samples into distinct atoms, followed by plasma ionization and detection using a mass spectrometer (Saranya et al. 2018).

##### Fourier transform infrared spectrometer (FTIR) analysis

To determine the specific changes in the functional groups of cells grown in the presence and absence of heavy metals, the infrared spectrum was analyzed using a Fourier transform infrared spectrometer (Spectrum Two, Perkin Elmer). Briefly, 2.5 g/L of dried biomass of potential strains K155 and BPSAC147 were inoculated in media with heavy metals as mentioned above. All the flasks were incubated at 28 °C for five days at 200 rpm, and then, the cultures were centrifuged at 10,000 rpm for 10 min at 4 °C. The obtained pellet was washed with 100 mM phosphate buffer (pH-7.4) and dried for three h using a vacuum concentrator (Thermo Scientific). The dried biomass was mixed with KBr in a ratio of about 1:100. The FTIR spectra of the dried biomass sample were recorded using an FT-IR/ATR spectrometer in the range of 400–4,000 cm-1 at a 4 cm^− 1^ (Sedlakova-Kadukova et al. 2019).

## Results

### Phenotypic characterization

Morphological analysis of *S. thermocarboxydus* strain K155 and BPSAC147 was carried out to observe their growth in different media. *S. thermocarboxydus* strain K155 and *S. thermocarboxydus* strain BPSAC147 were grown on tryptic soy agar (TSA) at 28 °C. Both formed brownish mycelia with brown spores at the colony periphery. However, strain BPSAC147 exhibited a more extended growth period, lasting up to two weeks, whereas strain K155 grew for only five days. Interestingly, strain BPSAC147 showed prolonged growth as compared to strain K155.

However, both K155 and BPSAC147 strains showed prolonged growth on ISP7 (tyrosine agar) and starch casein agar (SCA) media and did not produce any pigmentation. The formation of hyphae-like structures and spores was assessed microscopically using a Gram stain. Additionally, the scanning electron microscope (SEM) revealed that both strains have spiral-long spore chain morphology (Fig. S1).

### Genome assembly and annotation

**Fig. 1:**
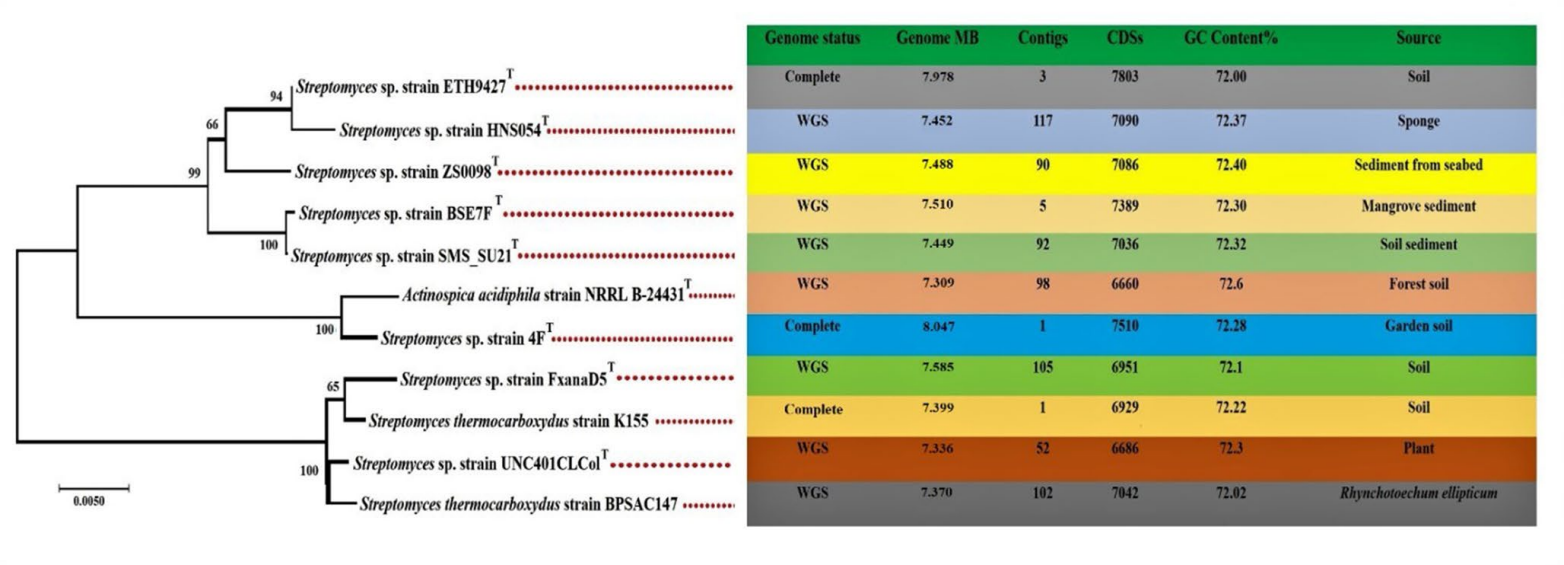
The 16S rRNANeighbor-joining phylogenetic tree constructed with the Tamura 3-parameter (T92+G) model using Mega X and comparative genome characteristics of the Streptomyces strain, along with the reference genome based on the PATRIC database, analyzed in this study. The tree is given 1000 bootstrap replicates, and the scale bar reveals the mean expected substitution per site

The genomes of strains K155 and BPSAC147 were sequenced, and the sequence quality was checked using FastQC. The low-quality sequence reads were removed using Trim Galore. The genome of K155 was assembled using a hybrid assembly approach, whereas the genome of BPSAC147 was assembled solely from short reads. The assembled genome of strain BPSAC147 consists of 7,370,148 bp with a G + C content of 72.03%. In contrast, strain K155 has a total length of 7,399,598 bp and an average G + C content of 72.22% (Fig. 1). The genome annotation of both strains was carried out using Rapid Annotations Subsystems Technology (RAST) and further analyzed with the Pathosystems Resource Integration Center (PATRIC). A total of 102 contigs and 7042 coding DNA sequences (CDS) were found in strain BPSAC147, whereas only one contig and 6929 CDSs were obtained in strain K155. The higher number of contigs in BPSAC147 reflects the use of Illumina short-read sequencing, which generally yields more fragmented assemblies than long-read PacBio sequencing used for strain K155. To assess genome completeness, BUSCO analysis (version 5.8.0) was performed using the *Streptomycetales_odb10* lineage dataset (*n* = 1579 BUSCO groups). For strain K155, the results were as follows: C: 99.4% [S: 99.3%, D: 0.1%], F: 0.1%, M: 0.5%, corresponding to 1570 complete BUSCOs (1568 single-copy [S], 2 duplicated [D]), one fragmented [F], and eight missing [M] BUSCOs. For strain BPSAC147, the results were C: 99.1% [S: 98.7%, D: 0.3%], F: 0.0%, M: 0.9%, corresponding to 1564 complete BUSCOs (1559 single-copy [S], five duplicated [D]), zero fragmented [F], and 15 missing [M] BUSCOs. These results confirm that both genomes are nearly complete, despite the greater fragmentation observed in BPSAC147, justifying its inclusion in downstream comparative analyses. Strain BPSAC147 contained 66 transfer RNA (tRNA) genes, three ribosomal RNA (rRNA) genes, 10 repeat regions, 2,129 genes coding for hypothetical proteins, and 4,913 proteins with functional assignments. In contrast, strain K155 comprises 67 tRNA genes, 18 rRNA genes, 1,964 genes coding for hypothetical proteins, and 4,965 proteins with functional assignments.

### Comparative genome features

The nine reference genomes - *Streptomyces* sp. ZS0098, *Streptomyces* sp. BSE7F, *Streptomyces* sp. SMS_SU21, *Actinospica acidiphila* NRRL B-24,431, *Streptomyces* sp. HNS054, *Streptomyces* sp. 4 F, *Streptomyces* sp. ETH9427, *Streptomyces* sp. UNC401CLCol, and *Streptomyces* sp. FxanaD5 - were selected based on 16 S rRNA sequence similarity in the PATRIC database and analyzed alongside *S. thermocarboxydus* strains K155 and BPSAC147 (Fig. 1). The phylogenetic tree constructed using the Neighbor-Joining method with the Tamura 3-parameter (T92 + G) model according to the lowest BIC (5388.923), and highest AIC (5221.347) values using Mega X, resolved the 11 strains into four clades, confirming the close evolutionary relationship of K155 and BPSAC147 within the *S. thermocarboxydus* cluster.

**Fig. 2:**
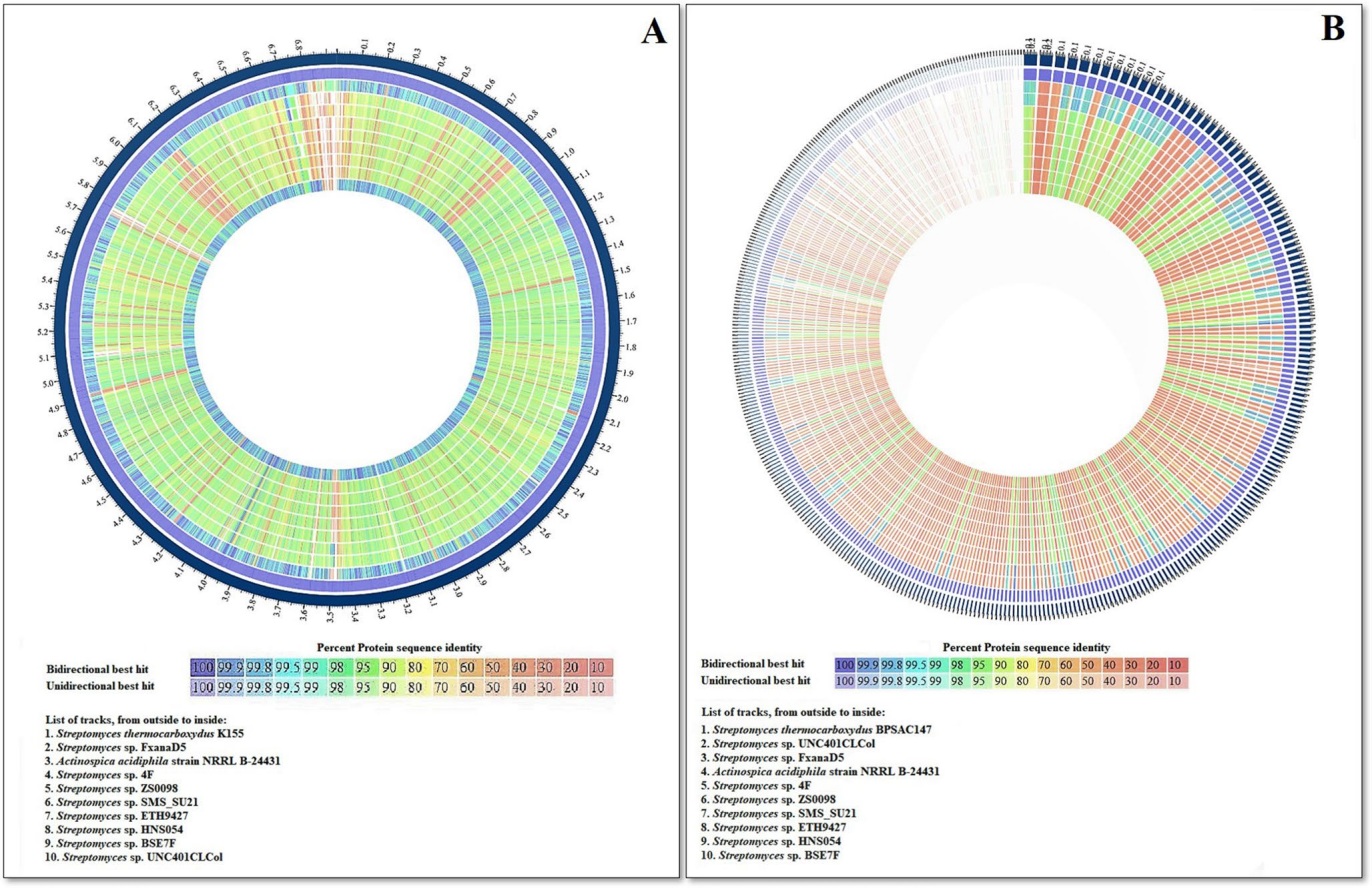
Genome overview for *Streptomyces thermocarboxydus* strain (**A**) K155 and (**B**) BPSAC147 with reference genome strains(*Streptomyces* sp. ZS0098, *Streptomyces* sp. BSE7F, *Streptomyces*sp. SMS_SU21, *Actinospica acidiphila* strain NRRL B-24431, *Streptomyces* sp. HNS054, *Streptomyces* sp. 4 F, *Streptomyces*sp. ETH9427, *Streptomyces* sp. UNC401CLCol and *Streptomyces* sp. FxanaD5), selected based on BlastN analysis using the PATRIC database and carried out the proteome comparative study

**Fig. 3:**
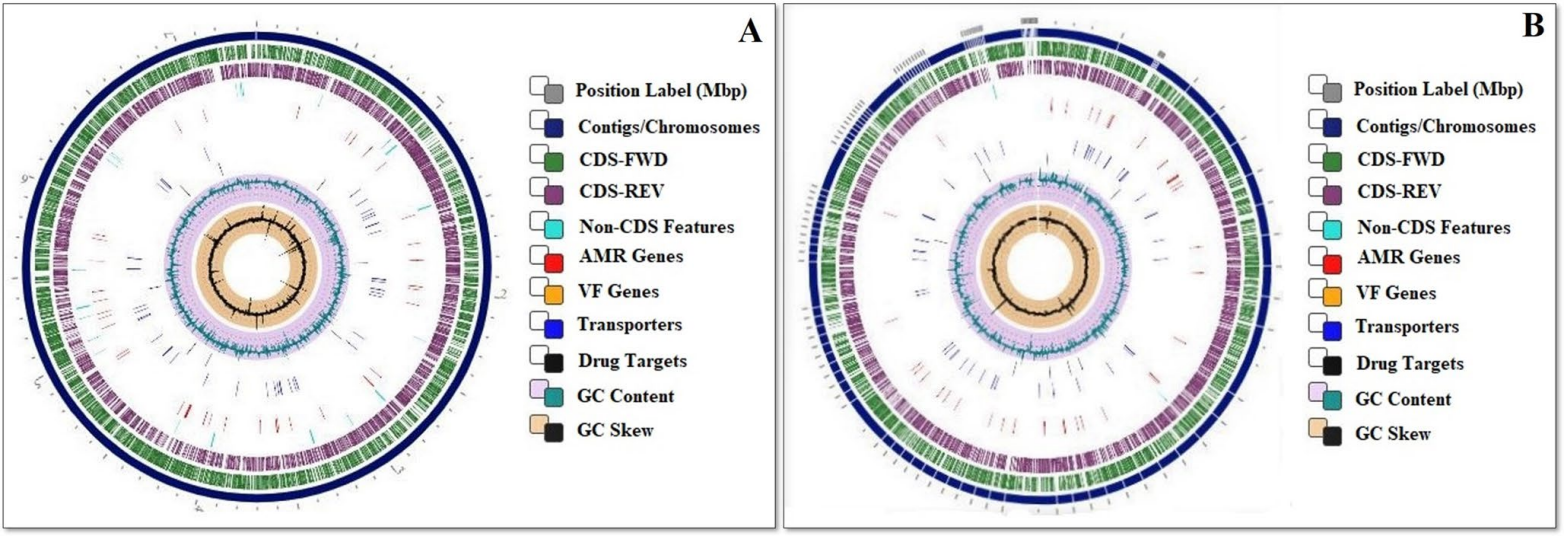
Circular graphical display of the distribution of the genome annotations of (**A**) *Streptomyces thermocarboxydus* strain K155 and (**B) ***Streptomyces thermocarboxydus* strain BPSAC147. From outside to center, the different colors indicate the size of the genome, protein-coding genes on the forward/reverse strands, antimicrobial-resistant (AMR) genes, virulence factor (VF) genes, percentage of G+C content, transporters, and GC skew

Proteome comparison using PATRIC PGfams and PLfams identified 12,573 orthologous gene families (OGFs) across all genomes (Figs. 2 and 3). Of these, 7,033 OGFs were conserved in at least one genome, and 2,897 (41.2%) were shared among all strains, representing the *core proteome*. Strains K155 and BPSAC147 contained 3,874 and 3,900 OGFs, respectively, while 103 and 240 genes were unique (“orphan genes”) to K155 and BPSAC147. Functional annotation of these unique genes indicated enrichment in categories related to heavy-metal resistance, stress adaptation, secondary metabolite biosynthesis, and hypothetical proteins. Notably, K155-specific genes were predominantly associated with metal ion transport and oxidative stress defense (e.g., ABC transporters, oxidoreductases, and catalase-like proteins), whereas BPSAC147-specific genes were linked to cadmium and copper resistance determinants, transcriptional regulators (WhiB-like proteins), and biosynthetic gene clusters for terpenoid and non-ribosomal peptide synthesis.

These comparative analyses highlight that, although both strains share a large, conserved genome backbone, their unique gene sets may underlie strain-specific ecological specialization and heavy-metal adaptation.

### Secondary metabolite biosynthetic gene cluster (SMBGC) analysis

**Fig. 4:**
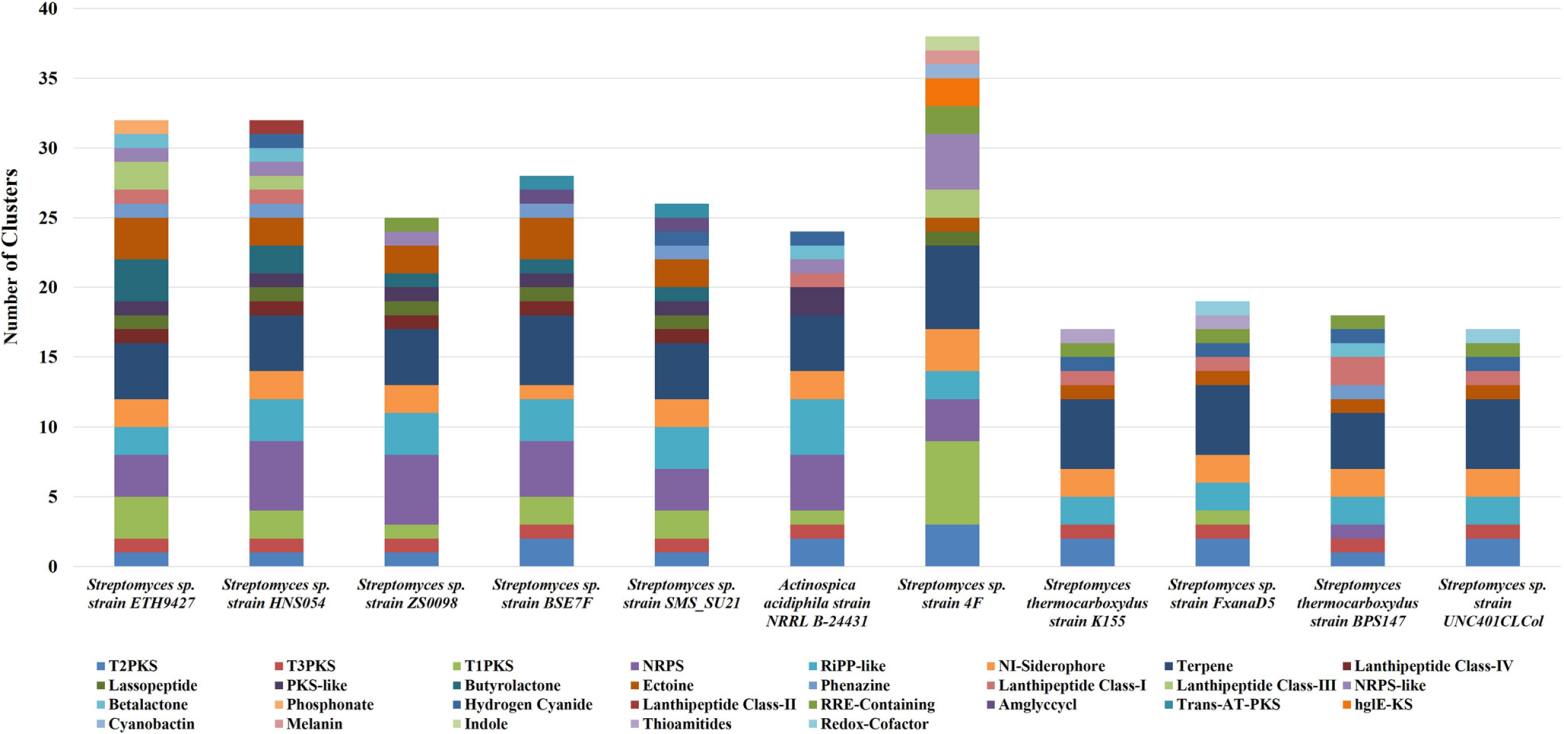
Comparative analysis of secondary metabolite biosynthetic gene clusters (BGCs) among selected *Streptomyces* and *Actinospica* strains using antiSMASH. The right side of the column-stacked bar chart displays the total number and diversity of BGCs predicted in each genome. Each color-coded segment within the bars corresponds to a distinct class of secondary metabolite BGCs, including polyketide synthases (type I, II, III PKS), nonribosomal peptide synthetases (NRPS), RiPPs (ribosomally synthesized and post-translationally modified peptides), terpenes, siderophores, and other specialized metabolite classes. The variation in cluster type and abundance among strains highlights the biosynthetic potential and genomic diversity relevant to secondary metabolite production

We used AntiSMASH version 7.1.0.1 to investigate secondary metabolite BGCs in the 11 genomes. A total of 276 putatively characterized and unknown BGCs were identified, with an average of 25.09 BGCs per genome. The maximum number of BGCs (38 BGCs) was observed in *Streptomyces* sp. 4 F followed by *Streptomyces* sp. ETH9427 (32 BGCs), *Streptomyces* sp. HNS054 (32 BGCs), and *Streptomyces* sp. BSE7F (28 BGCs), respectively. The lowest BGCs were observed in *S. thermocarboxydus* strain K155 (17 BGCs) and *Streptomyces* sp. strain UNC401CLCol (17 BGCs), whereas *S. thermocarboxydus* strain BPS147 was recorded 18 BGCs. The BGCs of all genomes studied covered putative secondary metabolites like terpenes, siderophores, lanthipeptides, lasso peptides, ectoine, PKS type-I, PKS type-II, PKS type-III, NRPS, butyrolactone, phenazines, hydrogen cyanide, cyanobactin, melanin, indole, thioamitides, butyrolactones, and amglyccycl, etc., as shown in Fig. 4.

The number of BGCs secondary metabolites might depend on the completeness of the genome size and its contigs. We detected BGCs of secondary metabolites in all genomes, including 50 terpene clusters, 28 NRPS and RiPP-like clusters, 22 Nl siderophore clusters, 18 PKS type-II and PKS type-I clusters, 17 ectoine clusters, 10 PKS type-III clusters, 08 butyrolactone and NRPS-like clusters, 07 in each strains PKS-like, hydrogen cyanide, and RRE-containing clusters, 06 lasso peptide clusters, 05 each lanthipeptide class-IV, phenazine, and lanthipeptide class-III clusters, respectively.

The most prevalent class of biosynthetic gene clusters (BGCs) identified was terpene BGCs, totaling 50 clusters and averaging 4.54 per genome. The second most common BGC class included non-ribosomal peptide synthetases (NRPS) and ribosomally synthesized and post-translationally modified peptides (RiPPs), accounting for 28 clusters and averaging 2.54 per genome. Finally, the third class comprised non-ribosomal siderophores, with 22 clusters in total and an average of 2 per genome. Interestingly, *S. thermocarboxydus* BPSAC147 harbored unique clusters encoding non-ribosomal peptide synthetase (NRPS) pathways, phenazine biosynthesis, and β-lactone biosynthesis. The NRPS cluster showed the highest similarity (similarity score: 0.73) to the tyrobetaine biosynthetic gene cluster from *Streptomyces* sp. NRRL WC-3703 (MIBiG accession: BGC0001813) (Parkinson et al. 2018). The phenazine cluster exhibited a similarity score of 0.48 to the phenazine SA–SC biosynthetic cluster from *Streptomyces* sp. (MIBiG accession: BGC0002561) (Han et al. 2019). Additionally, a β-lactone cluster with a similarity score of 0.61 was identified, matching the pyreudione A–E biosynthetic gene cluster from *Pseudomonas fluorescens* (MIBiG accession: BGC0002075) (Klapper et al. 2016). These clusters suggest that strain BPSAC147 possesses distinct biosynthetic potential for peptide- and lactone-based metabolites with signaling functions. In contrast, *S. thermocarboxydus* K155 contained a unique thioamitide cluster (similarity score: 0.53) showing homology to the thiovarsolin A–D biosynthetic gene cluster from *Streptomyces varsoviensis* (MIBiG accession: BGC0001849) (Santos-Aberturas et al. 2019). Thioamitides are ribosomally synthesized and post-translationally modified peptides (RiPPs) known for their thioamide-containing backbones and potential cytotoxic activity. Collectively, these findings demonstrate distinct secondary metabolite repertoires between the two *S. thermocarboxydus* strains, reflecting functional diversification in their biosynthetic capabilities and ecological adaptation. Moreover, some of the clusters like PKS type III, RiPP-like, NI siderophore, ectoine, hydrogen cyanide, and RRE-containing, were equally distributed in both the strains K155 and BPS147, respectively (Fig. 4).

### Functional study of heavy metal genes and phylogenetic analysis

All strain genome sequences were analyzed to identify heavy metal resistance genes using the PATRIC and RAST databases (Table S1). The results showed that 433 predicted genes were present in the 11 isolates, providing strong support for heavy metal tolerance. The predominant resistance gene was for mercury, followed by tellurite, copper, arsenic, cobalt, zinc, and cadmium resistance. One hundred thirty-three transcriptional regulators for mercury resistance (MerR family) were found in all strains. The highest number of MerR proteins was found in *Streptomyces* sp. strain ETH9427, and the lowest in *S. thermocarboxydus* strain BPSAC147. The tellurite resistance gene includes sixty-seven *terD* genes, thirty-three *terA* genes, eighteen *terB* genes, and eight for the putative tellurite resistance protein. We also detected eleven genetic determinants for copper tolerance, comprising one for the copper resistance protein *copC*, twenty-two for the copper resistance protein *copC*/*copD*, eleven for the copper chaperone (*copZ*), forty-five for the copper translocating P-type ATPase, sixteen encoding a multicopper oxidase, and eleven for the cytoplasmic copper homeostasis protein *cutC*. Genetic determinants encoding for arsenic resistance genes included eighteen *arsC* genes for arsenate reductases, fourteen for the arsenic resistance protein (*acr3)*, thirty genes for the arsenic operon repressor, and six genes for the flavin-dependent monooxygenase *arsO*, associated with arsenic resistance.

Moreover, we identified 11 genetic determinants for the cobalt-zinc-cadmium tolerance gene, involving 10 genes encoding cobalt-zinc-cadmium resistance proteins and 12 genes encoding the cobalt-zinc-cadmium resistance protein *czc*D. Furthermore, genes for magnesium resistance, a cobalt efflux protein encoded by the *core* gene, and thirty-one genes encoding the magnesium and cobalt transport protein *corA* were found in all eleven strains.

The study indicates that heavy metal genes were identified in isolates K155 and BPSAC147 through genome analysis. Hence, we have attempted to grow the isolates in various heavy metals, including copper, zinc, cadmium, and cobalt, to assess their tolerance.

#### COG analysis

A comparison of orthologous genes (COG) between strains K155 and BPSAC147 revealed a total of 78,407 putative genes (Table S2). The most common COGs were those for transcription (K), carbohydrate transport and metabolism (G), amino acid transport and metabolism (E), general function prediction (R), energy production and conversion (C), and signal transduction mechanisms (T). In contrast, the less common genes were for RNA processing and modification (A), chromatin structure and dynamics (B), cell motility (N), and extracellular structures (W), respectively. The highest number of putative functional genes was found in strain K155 compared to BPSAC147, whereas the category of extracellular structures (W) showed one more gene in strain BPSAC147 (41) than in strain K155 (40). A total of 6,437 genes of unknown function (S) were found.

### Functional annotation of the pangenome

**Fig. 5:**
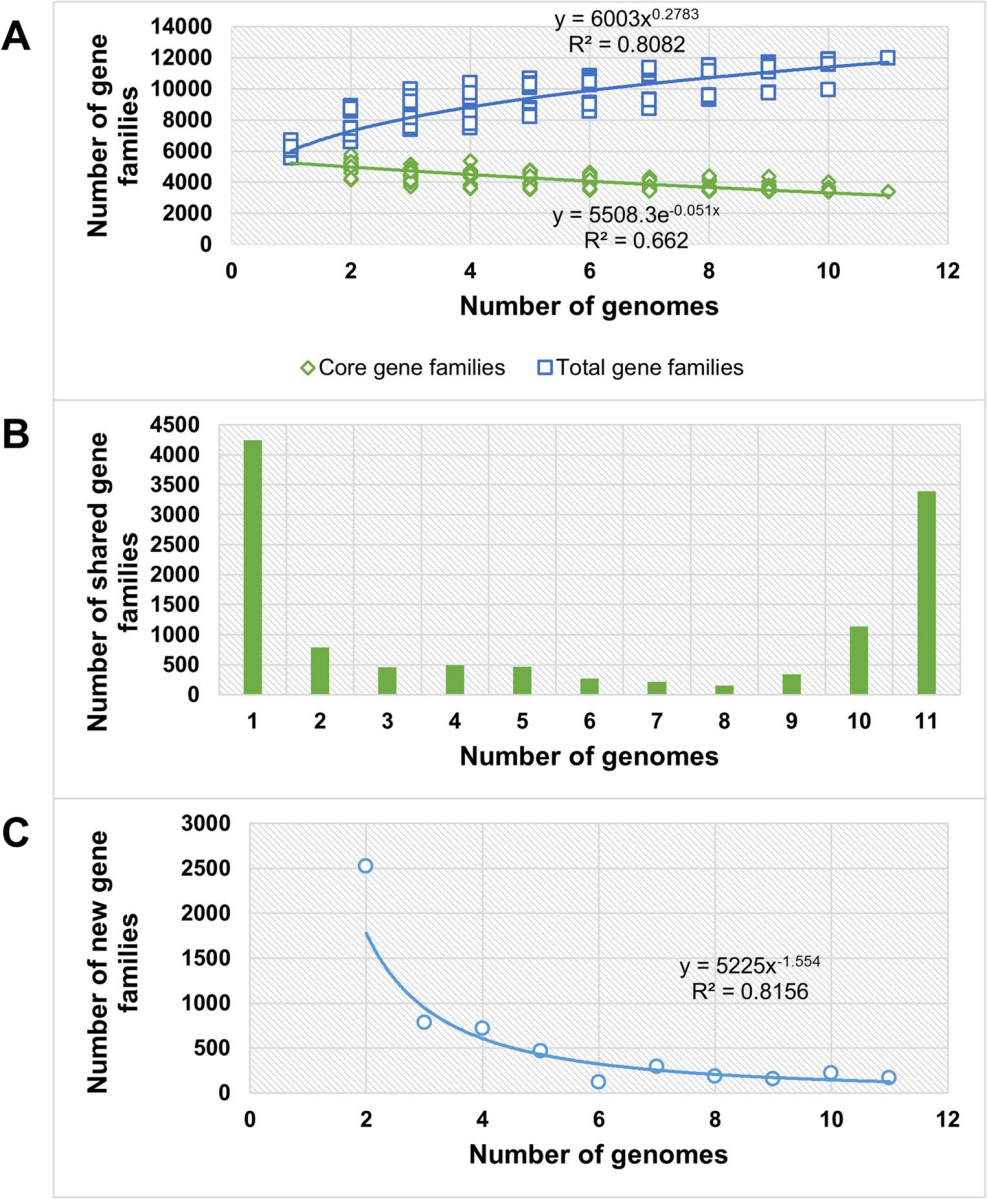
Determination of the pangenome for the analyzed strains. **(A)** Mathematical fit of the core genome size (green line) and the pangenome (blue line). **(B)** A histogram of the number of shared gene families characterizes the pangenome categories: the core genome, the unique genome, and the accessory genome, represented by clusters in one genome, all genomes, and those shared between 2 and 10 genomes, respectively. **(C)** The mathematical fit of the new genes provided for each new genome was added to the analysis

The pan-genome of this *Streptomyces* group comprised 11,969 gene clusters, of which 3390, 4338, and 4241 corresponded to the core, accessory, and unique genomes. Besides, the gamma value (0.2783) showed that the pangenome is open. Considering more recent large-scale genomic studies, it is now well established that the *Streptomyces* pangenome remains open, reflecting the high genomic diversity and adaptability of this genus (Caicedo-Montoya et al. 2021; Mohite et al. 2025; Otani et al. 2022) (Fig. 5A, B). These data are consistent with estimates of new genes, which show a plateau at the end of the plot (Fig. 5C).

**Fig. 6:**
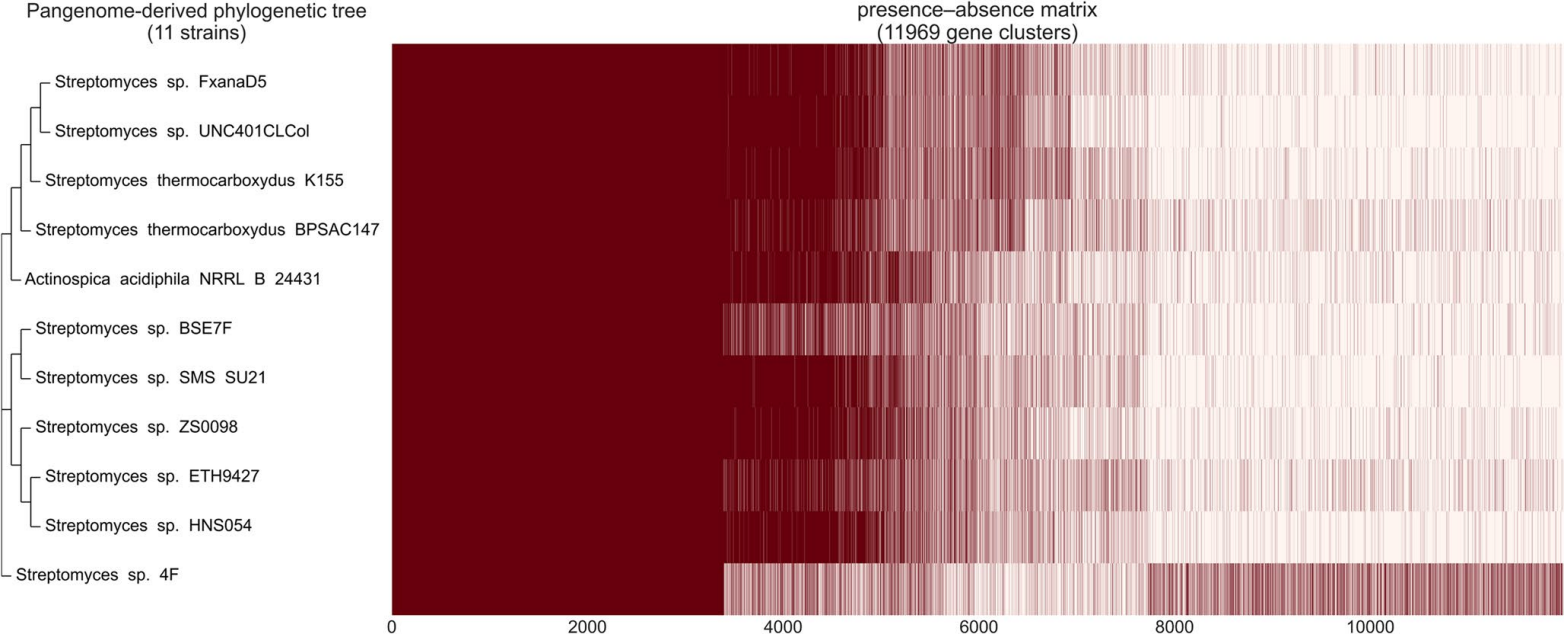
Phylogenetic tree based on the presence-absence of genes across the studied genomes (Red: Presence; White: Absence)

We constructed a phylogenetic tree based on the presence or absence of the different gene clusters in the analyzed genomes of the pangenome (Fig. 6, Table S3). Moreover, we estimated the phylogeny of the selected species based on 2644 core genes (Fig. S2). This tree shows high confidence in the results because all bootstrap values are 100%; interestingly, it exhibits high similarity to the pangenome tree, although the methods used to construct the two phylograms are very different. The differences in the core tree are related to mutations in the proteins encoded, whereas in the pangenome tree, they correspond to mutations in accessory genes (Ceapa et al. 2016).

**Fig. 7:**
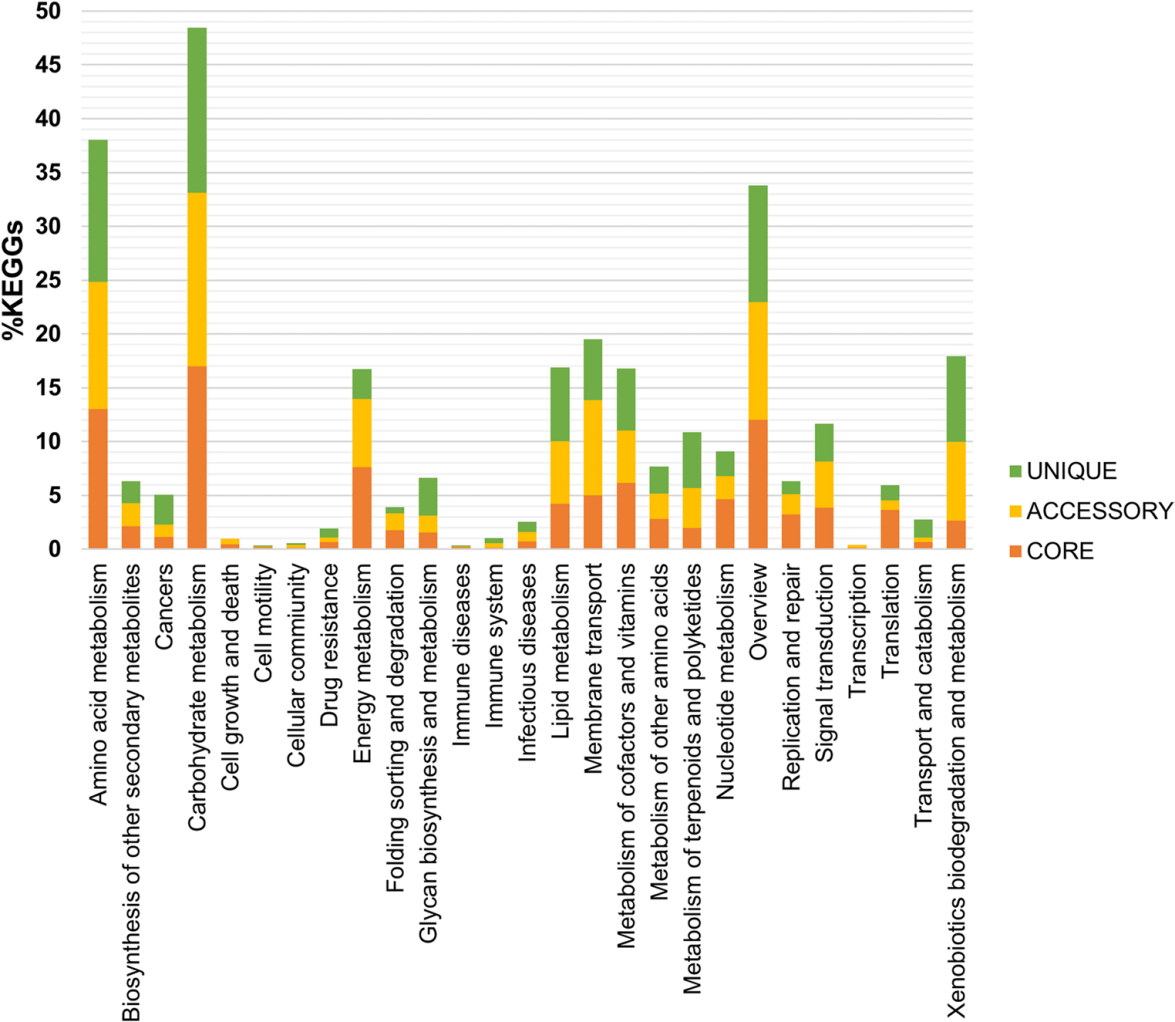
Functional characterization of the pangenome categories

The characterization of pangenome functionality was determined by BPGA using KEGG terms for gene families. As expected, the core genome is enriched in categories related to essential cellular maintenance, including central metabolism, DNA replication, and protein production. The unique and accessory genomes, which represent the diversity in the genomes analyzed, have a higher representation of amino acid metabolism, carbohydrate metabolism, glycan biosynthesis and metabolism, lipid metabolism, vitamin and cofactor metabolism, terpenoids and polyketides metabolism, and xenobiotic degradation and metabolism (Fig. 7, Table S4). On the other hand, the capability of streptomycetes for bioremediation processes is revealed by the enormous quantity of genes annotated in the category of xenobiotic degradation.

#### Stress response analysis

##### Oxidative stress

**Fig. 8:**
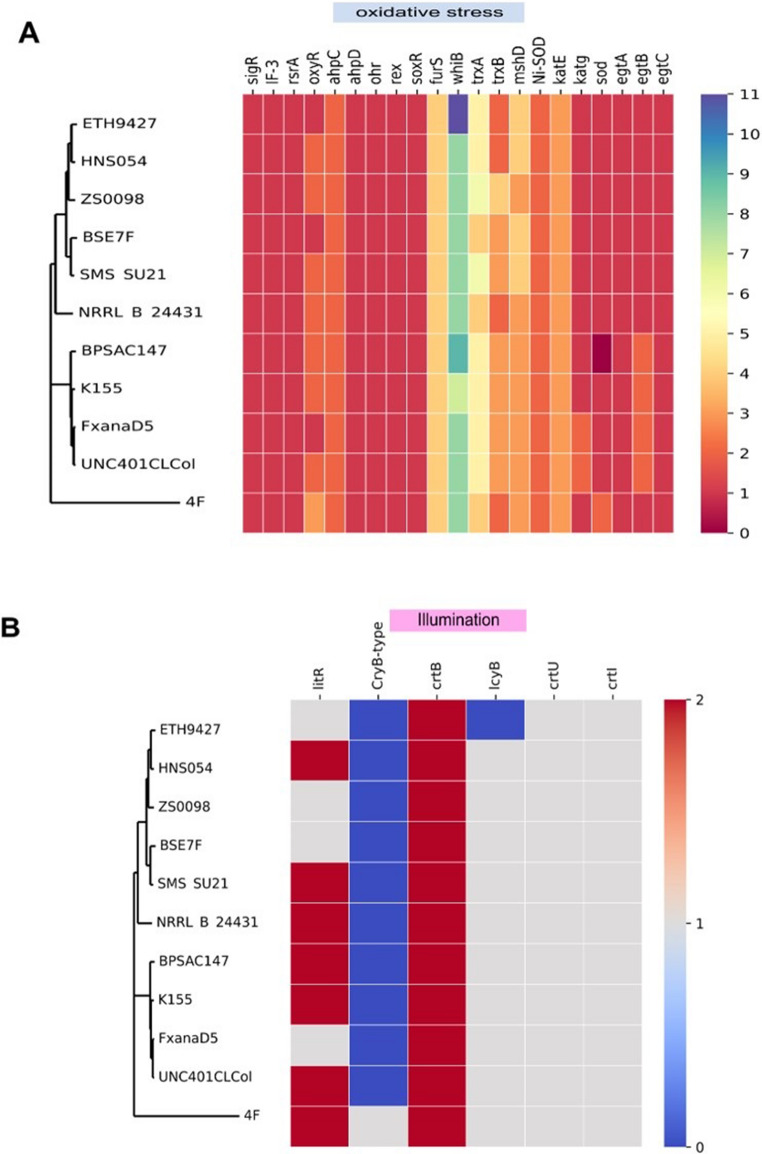
Functional characterization of the pangenome categories

Overall, our analysis revealed that major transcriptional regulators of the oxidative stress response are present in all analyzed species. These included genes encoding the sigma factor SigR and its cognate anti-sigma factor RsrA, which are key players in the global oxidative response in *Streptomyces*, along with the translation initiation factor IF3, which represses SigR translation (Feeney et al. 2017). Moreover, *oxyR*, which mediates the defense system against H_2_O_2_ in *Streptomyces avermitilis*, is a multiple-copy core gene present in most of the strains studied (Fig. 8A). The product of this gene activates the expression of multiple catalases and peroxidases in response to high peroxide concentrations (Liu et al. 2018). OxyR regulates the expression of its structural gene and the alkyl hydroperoxide reductase system (*ahpC* and *ahpD*) (Mitra, 2022). The latter are also core genes with 2 and 1 copies per genome, respectively. Ohr enzymes, which reduce highly toxic organic hydroperoxides (Mongkolsuk et al. 1998), are conserved in the genomes analyzed. FurS is a zinc-containing redox regulator that modulates *cpeB*, a catalase-peroxidase enzyme with five copies in all strains analyzed. Finally, seven and eleven copies of genes encoding WhiB-like proteins were identified in strains K155 and BPSAC147, respectively. WhiB is required for sporulation and aerial hyphae formation but has also been demonstrated to affect genes involved in the oxidative stress response negatively (Chawla et al. 2012).

In addition to the response regulators described above, other genes directly involved in the repair of Reactive Oxygen Species (ROS) damage are conserved. These include various genes encoding thioredoxin, the thioredoxin domain-containing protein, and the TrxB protein, responsible for TrxA reduction (den Hengst and Buttner, 2008), as well as genes of the mycothiol system. The mycothiol system is essential for bioremediation because it is resistant to oxidation by metal-derived molecular oxygen (Mitra, 2014). Nickel-dependent superoxide dismutase is also conserved. Indeed, it has been reported as a general feature in *Streptomyces* (Leclere et al. 1999). Finally, multiple copies of the enzymes catalase, catalase/peroxidase, and superoxide dismutase (SOD), which interact directly with peroxides, were found in the genomes of *Streptomyces* and *Actinospica.* Interestingly, *S. thermocarboxydus* BPSAC147 has no SOD genes. Furthermore, the *egtA*, *egtB*, and *egtC* genes, which are involved in the synthesis of ergothionein, a histidine-derived thiol compound with antioxidant properties (Osawa et al. 2018), are conserved in all *Streptomyces* strains.

#### Genes associated with light response

The light-inducible transcriptional regulator LitR of the *MerR* family has been reported to be widely distributed in the genus *Streptomyces* (Takano, 2016). LitR regulates a cluster for carotenoid biosynthesis in *S. coelicolor* A3(2) (Takano et al. 2005). An alignment of all MerR protein sequences present in the pangenome of *S. thermocarboxydus* K155 and BPSAC147 strains and the LitR sequence of *S. avermitilis* DSM 46,492 was performed (ID: WP_010982652.1). Interestingly, we found proteins in both genomes with 59.65% and 60.51% identity to LitR in strains K155 and BPSAC17, respectively. This result agrees with the fact that the metabolic pathway for the biosynthesis of some carotenoids is complete in both genomes (Fig. S3). On the other hand, the CryB-type cryptochrome, which has been demonstrated to modulate carbohydrate transport in non-phototropic actinobacteria (Maresca et al. 2019), was not detected in any of the *S. thermocarboxydus* strains. However, the cryptochrome/photolyase family protein was found as a unique gene cluster in the genome of *Streptomyces* sp. 4 F (Fig. 8B).

### Hyperosmotic stress

**Fig. 9:**
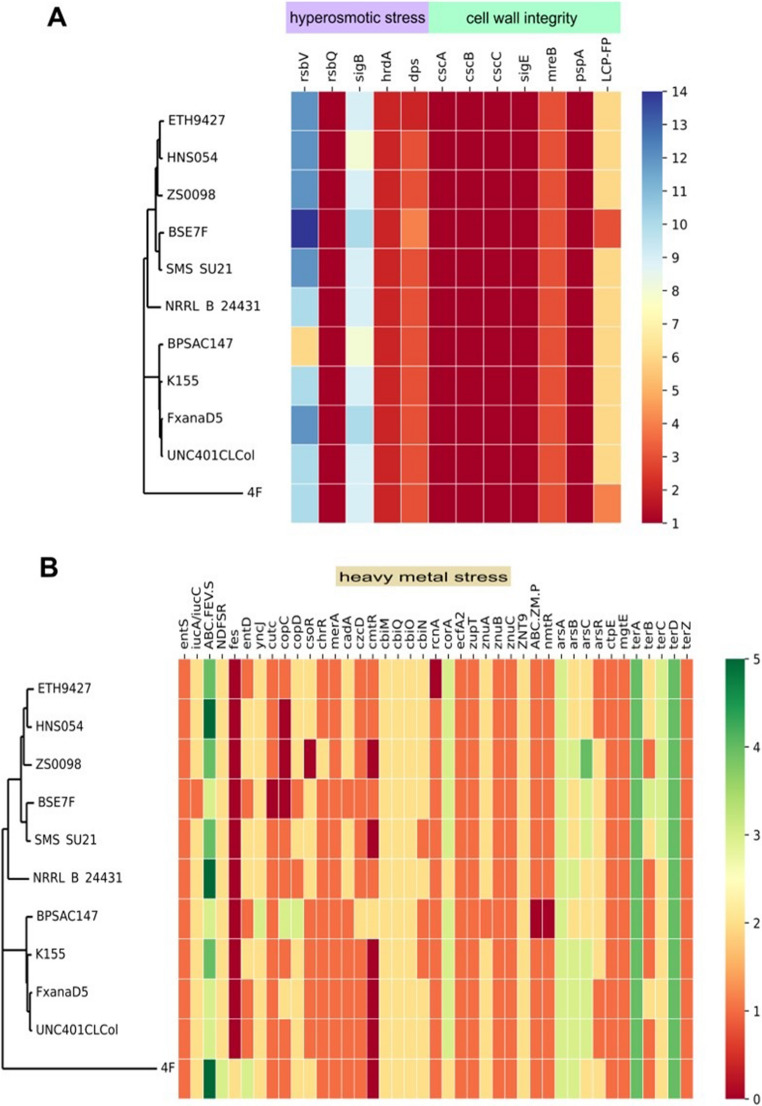
Heat map of the gene copy number of genes involved in **(A)** response to hyperosmotic stress and cell wall damage and (**B**) response to heavy metal presence. The strains are grouped according to the core genome phylogenetic tree

The master regulatory genes involved in the osmotic stress response were conserved in all *Streptomyces* genomes. The general mechanism involves the interaction between the anti-sigma factor RsbA and the sigma-B factor to repress sigma-B biosynthesis. Upon hyperosmotic stress, the anti-anti-sigma factor RsbV binds to RsbA, which initiates sigma-B production, which in turn switches the expression of multiple genes involved in the response to high osmolarity, such as *catB*,* hrdA*, and *whiB*. The latter controls the expression of *dpsA*, whose product is a nucleoid-associated protein that is highly induced by heat and osmotic stress (Cho et al. 2001; Cho et al. 2000; Facey et al. 2011; Lee et al. 2004). Thus, we found multiple anti-sigma B factor antagonists, between seven and ten sigma-B factors, two copies of the *hrdA* gene, and 2 to 4 *dps* genes in all genomes (Fig. 9A).

### Cell wall integrity

*Streptomyces* cell wall integrity is maintained by an operon formed by *cseA*,* cseB*, *cseC*, and sigma E. CseC/CseB form a two-component system that senses cell wall damage and induces SigE production. Induced SigE controls a regulation that regulates several genes involved in maintaining cell wall integrity. These include the penicillin-binding proteins, the cell shape determining MreB protein, the phage shock protein A homologous to PspA, and the LCP family protein (Hong et al. 2019). In this analysis, the operon (*cseA*, *cseB*, *cseC)* and sigma E were detected in the *Streptomyces* genomes. Besides, the peptidoglycan biosynthetic pathway, in which the penicillin-binding proteins catalyze multiple steps, was complete and conserved. The *mreB* gene has two copies, and six to seven copies of LCP family protein genes are present (Fig. 9A).

## Heavy metal stress

Mechanisms used by bacteria to cope with toxic metals include chelator and siderophore production, biomineralization, adsorption to cell walls, and intracellular storage (Kothe et al. 2010). In the genome strains included in this study, there are genes reported for the synthesis of siderophores, which comprise: the *IucA*/*IucC* family of siderophore biosynthesis protein, the siderophore-interacting protein, the NADPH-dependent ferric siderophore reductase, and the siderophore biosynthesis protein. In addition, KEGG annotations revealed an enterobactin synthetase component D for enterochelin synthesis. NADPH-dependent ferric siderophore reductase (which is widespread in bacteria) is essential for the conversion of siderophore-bound ferric iron to ferrous iron (Chillappagari et al. 2010). Siderophores and chelators have been implicated in nickel resistance. Besides, cadmium, copper, aluminum, and nickel have been shown to stimulate siderophore biosynthesis (Dimkpa et al. 2009).

Heavy metal resistance is highly correlated with the presence of metal transporters. Our analysis revealed the presence of multiple metal transporters in the genomes of *Streptomyces* and *Actinospica*. Two copies of the *ycnJ* were found in the genomes analyzed; this gene is responsible for the uptake of copper and its transfer across the membrane in *B. subtilis* (Chillappagari et al. 2010). We also found in all genomes *cutC*, a copper-binding protein that facilitates copper tolerance in *E. coli* (Gupta, et al. 1995), and *copC* that reduces copper resistance by sequestering copper in the periplasm (Cha and Cooksey 1991). We also reported the presence of chromate reductase-NAD(P)H dehydrogenase as a core gene; this enzyme reduces Cr(VI) or Cr(IV) to Cr(III), which is an efficient resistance mechanism because Cr(III) is unable to cross cell membranes (Gonzalez et al. 2005). *CadA* was also present in all genomes studied; it is highly expressed at high cadmium concentrations. It has been suggested that it confers resistance to cadmium and lead in *Pseudomonas putida* (Miller et al. 2009).

Multiple copies of cobalt transporters are present in all genomes, with the presence of ABC transporters standing out. Interestingly, KEGG annotations enabled us to identify zinc-transport genes in the *Streptomyces* genomes common to all strains. However, the permease protein of the zinc/manganese transport system (ABC.ZM.P) was absent from the BPSAC147 genome, explaining the reduced resistance to high zinc concentrations in this strain.

Finally, we found in all genomes the *merA* gene, which encodes the mercuric reductase enzyme responsible for volatilizing mercury as Hg^0^ (Freedman et al. 2012), and several copies of genes for resistance to arsenic and tellurite. Interestingly, *nmtR*, a type of ArsR family transcriptional regulator involved in nickel and cobalt response, was not present in the strain BPSAC 147. The *cmtR*, a different kind of ArsR transcriptional regulator responsible for the control of cadmium and lead response genes, was not present in the clade shared by those of 4 F, FxanaD5, UNC401ClCol, and K155 strains (Fig. 9B).

### Effect of heavy metals on growth

The growth responses of *S. thermocarboxydus* strains K155 and BPSAC147 to zinc, cobalt, copper, and cadmium were evaluated in TSB medium supplemented with varying metal concentrations (zinc, cobalt, and copper: 100–1000 mg/L; cadmium: 10–100 mg/L) (Fig. S4 and Fig. S5) along with metal-free controls (Tables S5, S6). Both strains exhibited distinct growth dynamics depending on the metal type, concentration, and incubation period.

Statistical analysis using two-way ANOVA (strain × concentration × time) followed by Tukey’s post-hoc test revealed significant effects of strain, metal concentration, and incubation time (*p* < 0.05) for all tested metals (Tables S7). The interaction between strain and concentration was significant for zinc, cobalt, and cadmium (*p* < 0.05), confirming strain-specific tolerance responses.

Strain K155 demonstrated significantly higher tolerance to zinc and cobalt (*p* < 0.01), maintaining active growth up to 1000 mg/L at 72–120 h. In contrast, strain BPSAC147 showed greater tolerance to cadmium (*p* < 0.05) and exhibited more rapid early growth under copper exposure (*p* < 0.05). Growth inhibition was concentration-dependent for all metals, with reduced biomass observed at higher concentrations of copper and cadmium compared to metal-free controls.

Overall, these statistical results substantiate that *S. thermocarboxydus* strains K155 and BPSAC147 possess differential heavy-metal tolerance mechanisms, likely reflecting adaptive physiological or genomic variation between the two isolates.

### ICP-MS

The ICP-MS results showed that strain K155 grown without metals had basal concentrations of zinc, cobalt, copper, and cadmium of 0.61, 0.24, 0.44, and 0.10 µg/g, respectively. While K155 grown in the presence of the different metals had final concentrations of zinc, cobalt, copper, and cadmium of 1,316.1, 1,298.8, 551.4, and 447.2 µg/g, respectively. As for strain BPSAC147, its basal concentrations of zinc, cobalt, copper, and cadmium were 0.85, 0.66, 0.77, and 0.16 µg/g, respectively. The final concentrations after growing in the presence of zinc, cobalt, copper, and cadmium were 4677.20, 649.28, 929.38, and 727.96 µg/g, respectively.

#### SEM and EDX analysis

Scanning electron microscopy (SEM) revealed distinct morphological alterations in *S. thermocarboxydus* strains K155 and BPSAC147 under heavy-metal stress (Figs. S6–S15). Control cells displayed smooth, intact hyphae with compact spore chains (0.5–5 μm). Zinc and cobalt exposure caused surface roughness and spore-chain disruption, whereas copper and cadmium induced collapsed filaments and mineral aggregation. Mineralized precipitates frequently formed spore-chain-like structures (~ 1 μm).

Energy-dispersive X-ray (EDX) spectroscopy confirmed metal accumulation and elemental redistribution at the cell surface. Control samples were dominated by carbon, oxygen, and nitrogen peaks. In contrast, metal-treated samples displayed distinct Zn, Co, Cu, and Cd signals together with Na, Mg, P, S, and Ca derived from the cell matrix (Figs. S6–S15). Quantitatively, zinc deposition was highest in K155 (19.3 at%), while BPSAC147 accumulated more cadmium (0.9 at%) than K155 (0.3 at%). Cobalt and copper were detected at trace levels (0.1–0.2 at%) in both strains. These findings indicate that heavy-metal exposure alters hyphal morphology and promotes extracellular biomineralization, with K155 showing enhanced zinc precipitation and BPSAC147 greater cadmium affinity.

#### FTIR analysis

The biosorption capacity of *S. thermocarboxydus* strains K155 and BPSAC147 was evaluated using FTIR spectroscopy to detect functional groups involved in metal binding (Fig. S16–S25). FTIR spectra in the 400–4000 cm⁻¹ range revealed characteristic absorption bands corresponding to O-H (3278–3264 cm⁻¹), -CH (3000–2850 cm⁻¹), C-N (1338–1027 cm⁻¹), -COO (1403–1395 cm⁻¹), C = C (1638–1632 cm⁻¹), -NO₂ (1546–1535 cm⁻¹), C-O (876–852 cm⁻¹), -NH (1582 cm⁻¹), and -CHO/-COOH (1742–1739 cm⁻¹) groups.

Comparative analysis revealed distinct shifts and intensity changes between K155 and BPSAC147 upon metal exposure. In K155, pronounced peak shifts in the –OH (3266.87 cm⁻¹) and –COOH (1739.01 cm⁻¹) regions were observed under zinc and cobalt treatment, indicating strong involvement of hydroxyl and carboxyl groups in metal coordination. In contrast, BPSAC147 showed more pronounced variations in the amide (–NH, 1582.27 cm⁻¹) and nitro (–NO₂, 1535.16 cm⁻¹) regions, particularly under cadmium and copper exposure, suggesting the involvement of proteins and peptides in biosorption. These strain-specific spectral changes highlight differential utilization of functional groups in metal binding, reflecting variations in cell-wall composition and surface chemistry between K155 and BPSAC147.

## Discussion

The eleven strains had genome sizes of 7.309–8.047 Mb and GC contents of 72–72.6%. *S. thermocarboxydus* K155 and BPSAC147 had 7.399 and 7.37 Mb genomes with GC contents of 72.22% and 72.02%, respectively, consistent with *Streptomyces* (Bentley et al. 2002). In contrast, *Ktedonobacteria* and *Amycolatopsis* had lower GC contents (55.1% and 71.4%) (Chater and Chandra 2006; Zheng et al. 2019; Sánchez-Hidalgo et al. 2018). The 0.2% GC difference between K155 and BPSAC147 falls within species-level variation (< 1%) (Meier-Kolthoff et al. 2013).

We constructed a 16 S rRNA phylogenetic tree using the Neighbor-joining method and found that all 11 species clustered into four clades. Interestingly, *S. thermocarboxydus* K155 and BPSAC147 clustered separately with strains of *Streptomyces* sp. FxanaD5 and *Streptomyces* sp. UNC401CLCol in clade IV. Additionally, strains isolated from plants BPSAC147 and *Streptomyces* sp. UNC401CLCol were closely clustered. Tang et al. (2016) reported that the 16 S rRNA phylogenetic tree varies depending on the source from which different strains were isolated. In addition, the 16 S rRNA gene sequence identification for interspecies pairs is much higher than that for intraspecies pairs. This may explain why the two *S. thermocarboxydus* strains are positioned on different branches of the 16 S rRNA gene phylogenetic tree, as phylogenetic variation can depend on the isolation source and interspecies sequence similarity tends to be higher than intraspecies similarity.

According to Genilloud, primary and secondary metabolism were entirely dependent on regulatory signals that provided for the existence and adaptation of the microbial community. The production of several secondary metabolites was entirely dependent on primary metabolism, meaning that many precursors and building blocks were supplied by it. As a result, we understood that the genes strongly related to secondary metabolites BGCs and their heterologous expression is modulated by transcriptional regulators (Genilloud 2017).

In our study, the genomes of the eleven strains were searched for putative BGCs using the antiSMASH program. The BGCs of secondary metabolites from all the genomes were classified into terpene, siderophore, bacteriocin, lanthipeptide, lasso peptide, ectoine, PKS type-I, PKS type-II, PKS type-III, NRPS, beta-lactone, phenazine, butyrolactone, amglyccyclic, etc. Different types of polyketides were found in strains K155 and BPSAC147. In *Streptomyces* sp. ETH9427; *Streptomyces* sp. 4 F; *A. acidiphila* NRRL B-24,431 and *Streptomyces* sp. SMS_SU21, a high number of putative PKS pathways were detected, but they showed highly fragmented PKS genes. This finding was also reported by Sanchez-Hidalgo et al. (2018). They found that the strains *Amycolatopsis kentuckyensis* DSM 44652 T and *Amycolatopsis lexingtoniensis* DSM 44653 T harbor many putative PKS pathways distributed across multiple genome fragments. A type III polyketide BGC was detected in K155 and BPSAC147, showing 100% similarity to alkylresorcinol BGCs from *S. griseus* subsp. *griseus* NBRC 13,350. Moreover, the genomes of *S. thermocarboxydus* K155 and BPSAC147 are consistent with type II PKS, showing 83% similarity to spore pigment BGC from the soil bacterial strain *S. avermitilis*, which is involved in the biosynthesis of type II polyketide-derived melanin pigment (Ômura et al. 2001). Another type II PKS, identified only in strain K155, exhibited 71% similarity to the antibiotic alnumycin. This finding was also reported by Oja et al. (2008), who stated that the alnumycin gene is closely related to the benzoisochromanequinone (BIQ) polyketide actinorhodin, obtained from *Streptomyces* sp. CM020.

Twenty-eight NRPs BGCs from 13 different pathways were found in the 11 strains, as follows: naphthyridinomycin, antimycin, sandarazol, cyclofaulknamycin, polyoxypeptin, auroramycin, omnipeptin, and tyrobetaine. Among these, an NRPS cluster of strain BPSAC147 showed 46% similarity to that of tyrobetaine. This compound was reported by Parkinson et al. (2018), who described tyrobetaine as a new class of NRPs obtained from *Streptomyces* sp. NRRL WC-3703.

To characterize the terpene BGCs of strains K155 and BPSAC147 in comparison to other reference strains, the MIBiG database 20 was used. The predominant terpene BGCs identified in all strains are: geosmin, a carotenoid, hopene, cyslabdan, isorenieratene, and albaflavenone. The terpene BGCs observed in all the strains with 100% similarity were geosmin and albaflavenone (Jiang et al. 2007; Zhao et al. 2008). Geosmin, encoded by the *sco6073* gene in *S. coelicolor* A3(2), can be produced enzymatically from farnesyl diphosphate (PFP, 2), according to Jiang et al. (2007). In contrast, Zhao et al. (2008) reported the biosynthesis of the antibiotic albaflavenone in *S. coelicolor* A3(2) through the coupled action of epi-isozizaene synthase and the cytochrome P450 CYP170A1 gene. All strains showed a terpene cluster with 54% similarity to the carotenoid BGCs of *S. avermitilis*, which contains five genes responsible for their carotenoid biosynthesis (Ômura et al. 2001).

Interestingly, according to the MIBiG database 20, all the strains, including the reference ones, produced ectoine (1,4,5,6-tetrahydro-2-methyl-4-pyrimidinecarboxylic acid). This compound has several biotechnological applications, such as protection against stress under different environmental conditions (Hamedi et al. 2013). Ectoine BGCs were the predominant BGCs in Actinobacterial and Proteobacterial phyla (Hamedi et al. 2013). Ectoine BGCs were widely reported in salt-tolerant *Streptomyces* species (Nett et al. 2009; Zhao et al. 2016). In our study, 17 clusters for ectoine synthesis were detected in all strains with 100% similarity to *Streptomyces chrysomallus* (Prabhu et al. 2004). On the other hand, phenazine BGC was only observed in *S. thermocarboxydus* strain BPSAC147. Phenazine was first investigated in *Streptomyces anulatus* 9663 (Saleh et al. 2012).

Tellurite reduction generates ROS, which are highly harmful to cells, leading to lipid peroxidation and DNA damage (Pérez et al. 2007; Tremaroli et al. 2007). In the genomes of the eleven strains, 133 *merR* genes were found. Moreover, we found the highest number of *merR* genes in the genome of *Streptomyces* sp. ETH9427, while the lowest in *S. thermocarboxydus* strain BPSAC147. In *Streptomyces* sp. H-KF8, isolated from marine sediments of the northern Chilean Patagonia, merR genes were found to regulate the mercury reductase gene *merA* (Undabarrena et al. 2017). MerA is a NADPH-dependent flavoprotein that can reduce mercury (II) to mercury (0), which is less toxic (Barkay et al. 2003). Rodríguez-Rojas et al. (2015) recently reported that mercury resistance may also have cross-resistance to tellurite.

This study identified all *Streptomyces* strains associated with resistance to heavy metals, including copper, arsenic, cobalt, zinc, and cadmium. Copper resistance was mediated by genes encoding c*opC* and *copC/copD*, copper chaperones (*copZ*), multicopper oxidases, and P-type ATPases. Notably, *Streptomyces* sp. H-KF8 exhibited a unique copper resistance phenotype with three *copA* genes encoding multi-copper oxidases responsible for the oxidation of Cu(I) to Cu(II) (Hobman and Crossman 2014; Undabarrena et al. 2017). For arsenic resistance, the *arsC* genes encoding arsenate reductase, the arsenical resistance protein *acr3*, and the flavin-dependent monooxygenase *arsO* were found. *Streptomyces* sp. H-KF8 has a complete *arsRABCD* operon, which could potentially allow the reduction of arsenate reduction to arsenite and its subsequent detoxification (Undabarrena et al. 2017). Resistance to cobalt, zinc, and cadmium includes the presence of cobalt-zinc-cadmium resistance proteins and *czcD* efflux system, consistent with findings in *Microbacterium* spp. strain from contaminated environments (Learman et al. 2019). These findings demonstrate the potential of *Streptomyces* strains to survive in metal-contaminated environments and provide insight into their bioremediation capabilities and resistance mechanisms, which could be exploited for environmental and industrial applications.

*Streptomyces* species exhibit metal tolerance, aiding bioremediation (Hassanein et al. 2012). *S. thermocarboxydus* K155 and BPSAC147 tolerated 1000 mg/L zinc, exceeding *S. werraensis* LD22 (250 mg/L) (Latha et al. 2014). Cobalt tolerance was 1000 mg/L (K155) and 750 mg/L (BPSAC147), comparable to *Streptomyces* sp. H-KF8 (176.79 mg/L) (Schmidt et al. 2005). Copper tolerance was 750 mg/L (K155) and 500 mg/L (BPSAC147), surpassing *S. werraensis* LD22 (250 mg/L) (Latha et al. 2014). Cadmium tolerance was 50 mg/L (K155) and 75 mg/L (BPSAC147), like *S. pactum* Act12 (Cao et al. 2016). These results align with those of metal-resistant Streptomyces strains, including K11 (Sedlakova-Kadukova et al. 2019), and marine sponge-associated species (Joseph et al. 2009).

The pangenome or supra-genome consists of the complete set of genes and provides an overview of the genomes of a given group of species (Tettelin et al. 2008). The pangenome calculation for the species included in the present analysis established an open pangenome, consistent with previous studies showing similar results (Bu et al. 2020). These results reveal the high genetic diversity of this genus and mean that new genes may be discovered as new species are found and sequenced. On the other hand, the low gamma parameter value in the fitted mathematical model suggests that it could close soon, a result that appears to contradict the lifestyle of streptomycetes, which shows a wide variety of niches. However, this could be explained by the selection of closely related species for the present analysis, as supported by the high number of core genes detected (3390). The functional annotation of the pangenome highlighted the enormous potential of Streptomyces as a tool for bioremediation. We identified numerous genes involved in pollutant degradation in accessory and single genomes. These results are consistent with previous studies, e.g., *Streptomyces* sp. Hlh1, which can degrade petroleum hydrocarbons, particularly aromatic compounds, and reduce the cytotoxicity of petroleum end products (Baoune et al. 2018, 2019). Other strains, such as *Streptomyces aureofaciens*, have shown the ability to take up zinc from soils, and *S. mirabilis* P16-B1 can survive in environments contaminated with various metals and favorably promote plant growth (El-Sayed et al. 2011; Schütze et al. 2014).

Bacteria possess sophisticated defense mechanisms that enable them to adapt and survive in highly variable, stressful environments. These include responses to oxidative stress, osmotic fluctuations, the presence of toxic substances, and variations in light exposure. Heavy metals are particularly harmful due to their interactions with biological macromolecules, such as lipids, proteins, and DNA, and their role in generating oxidative stress by producing hydroxyl radicals (Stohs and Bagchi, 1995). Reduction of oxidative stress increases bacterial resistance to heavy metals. For instance, nickel and other metals induce expression of the superoxide dismutase gene (*sodN*) in *S. coelicolor* to counteract oxidative damage (Schmidt et al. 2007). Genes associated with metal resistance, oxidative stress response, and other stress adaptation pathways are conserved in the genus *Streptomyces*. The presence of multiple copies of these genes underscores their critical role in coordinating precise and efficient responses to environmental challenges. These results highlight the genus *Streptomyces* as a robust model for understanding bacterial stress resistance and its potential application in bioremediation and biotechnology.

Stress conditions favor the production of some secondary metabolites; for instance, light can induce the expression of operons involved in metabolite production in some *Streptomyces* species (Takano et al. 2005). Besides, it has been reported that when bacterial cells sense salt stress, they begin producing various solutes, such as glycine betaine, trehalose, and ectoine, to maintain internal osmotic pressure like that of the external environment (He et al. 2018). Kol et al. (2010) showed that the response of *S. coelicolor* to salt stress is characterized by an upregulation of the production of osmoprotectants, such as proline/glycine-containing di- and tripeptides. Our analysis showed that *Streptomyces* strains contain the complete metabolic pathways to synthesize glycine, betaine, and ectoine.

Bacteria regulate mineralization and nucleation processes and influence the type and growth of precipitated minerals (Phillips et al. 2013; Frankel 2013). In this study, SEM-EDX analysis was used to investigate the elemental composition and distribution of zinc, cobalt, copper, and cadmium in the dry biomass of *S. thermocarboxydus* strains K155 and BPSAC147. SEM-EDX revealed high levels of carbon, oxygen, nitrogen, and sodium, consistent with the findings of Zhao et al. (2019), who reported similar results in metal-contaminated bacterial strains. Both strains demonstrated biomineralization capacity for zinc, cobalt, copper, and cadmium, consistent with previous studies on mineralization in several bacterial genera (Ramyakrishna and Sudhamani 2017). The biomineralization of cadmium acetate by the strains was remarkable, producing precipitates within spore chains (~ 1 μm in diameter), similar to the findings of Zhao et al. (2019). Bacterial interactions with the mineral alter mineral morphology and precipitate characteristics (Teng et al. 2006). These results underscore the usefulness of SEM-EDX for elemental mapping, which complements ICP-MS and SEM analyses to provide a complete picture of biosorption and biomineralization processes.

Fourier transform infrared spectroscopy (FT-IR) was used to analyze the functional groups in the dry biomass of *Streptomyces* strains K155 and BPSAC147 treated with zinc, cobalt, copper, and cadmium. Absorption bands were detected at 3084.58–2853.87 cm⁻¹, corresponding to -CH stretches, consistent with previous findings in *Cronobacter muytjensii* treated with metals (Saranya et al. 2018). Peaks at 3283.19–3257.71 cm⁻¹ were assigned to O-H stretching, as in *Streptomyces* sp. strain K11 (Sedlakova-Kadukova et al. 2019). Bands at 1403.24–1395.36 cm⁻¹ indicated vibrations of the -COO, as reported by Zhang et al. (2010). Other significant peaks included 876.50–852.25 cm⁻¹ (C-O group), 1641.08–1632.52 cm⁻¹ (C = C stretch), 1027.01–1338.16 cm⁻¹ (C-N amide II), and 1535.16–1546.46 cm⁻¹ (-NO₂ group), corroborating studies by Movasaghi et al. (2007) and Smith (1999). Metal-treated biomass exhibited significant spectral variations, probably due to the secretion of high-molecular-weight polymers on cell surfaces. This is consistent with findings on zinc-treated *Streptomyces* strain K11 (Sedlakova-Kadukova et al. 2019) and chromium-treated *Streptomyces* sp. VITSVK9 (Saurav and Kannabiran, 2011). This study highlights the functional groups of *S. thermocarboxydus* strains K155 and BPSAC147, which may play a role in metal biosorption. To our knowledge, this is the first report on functional group analysis of these strains under metal treatment.

## Supplementary Information

Below is the link to the electronic supplementary material.

Supplementary Material 1(PDF 6.43 MB)

Supplementary Material 2(PDF 8.05 MB)

## Data Availability

This whole-genome sequence of *S. thermocarboxydus* strain K155 has been deposited at NCBI GenBank under BioProject accession numbers PRJNA312938. The strain sequence BPSAC147 has the BioProject accession number PRJNA527710.
