# Supplementary material for: Comparative genomics of the Streptomyces genus: insights into multi-stress-resistant genes for bioremediation
Source: World J Microbiol Biotechnol. 2025 Dec 2;41(12):492. doi: 10.1007/s11274-025-04703-1 (PMC12672707; doi:10.1007/s11274-025-04703-1)
Supplement: Supplementary file 1 — Supplementary Material 1(PDF 6.43 MB) [file 11274_2025_4703_MOESM1_ESM.pdf]

Supplementary Figures

World Journal of Microbiology and Biotechnology

Comparative Genomics of the *Streptomyces* Genus: Insights into Multi-Stress-Resistant Genes for Bioremediation

Ajit Kumar Passari[1*], Carlos Caicedo-Montoya[2], Monserrat Manzo-Ruiz[1], María Paula Gomez-Roman[1], Beatriz Ruiz-Villafán[1], José Fausto Rivero-Cruz[3], Bhim Pratap Singh[4], Romina Rodríguez-Sanoja[1], and Sergio Sánchez[1]*

[1]Instituto de Investigaciones Biomédicas, Universidad Nacional Autónoma de México, Ciudad de México 04510, México.

[2]Grupo de Bioprocesos, Departamento de Ingeniería Química, Universidad de Antioquia, Calle 70 No. 52-21, Medellín 050010, Colombia.

[3]Centro de Innovación para el Desarrollo Apícola Sustentable de Quintana Roo. Universidad Intercultural Maya de Quintana Roo, QR, 03940, México.

[4]Department of Agriculture & Environmental Sciences, National Institute of Food Technology Entrepreneurship & Management (NIFTEM), Sonepat, Haryana-131028, India

*Corresponding authors: sersan@biomedicas.unam.mx
ajit.passari22@iibiomedicas.unam.mx

Fig.S1 Scanning electron microscope (SEM) indicates strains (**A**) *Streptomyces thermocarboxydus* strain K155; (**B**) *Streptomyces thermocarboxydus* strain BPSAC147 producing spiral long spore chain morphology.

Fig. S2 Core genome-derived phylogenetic tree.

Fig. S3 Biosynthesis of carotenoid pigments present in the strain K155 and BPSAC17. Green indicates strain K155, and pink indicates strain BPS147.

Fig. S4 Effect of different multi-metal concentrations on the growth pattern of *Streptomyces thermocarboxydus* strain K155 growing in tryptone soya broth (TSB). (**A**) Zinc (100 to 1000 mg/L), (**B**) Cobalt (100 to 1000 mg/L), (**C**) Copper (100 to 1000 mg/L), and (**D**) Cadmium (10 to 100 mg/L). Control: Strain K155 inoculated in TSB broth without any heavy metals and incubated at 28 ^{o}C for up to 120 h. The values were performed in a triplicates process, and error bars indicate the mean ± standard deviations (SD).

Fig. S5 Effect of different multi-metal concentrations on the growth pattern of *Streptomyces thermocarboxydus* BPSAC147 growing in tryptone soya broth (TSB). (**A**) Zinc (100 to 1000 mg/L), (**B**) Cobalt (100 to 1000 mg/L), (**C**) Copper (100 to 1000 mg/L), and (**D**) Cadmium (10 to 100 mg/L). Control: Strain BPSAC147 inoculated in TSB broth without any heavy metals and incubated at 28 ^{o}C for up to 120 h. The values were determined in triplicate, and error bars indicate the mean ± standard deviation (SD).

Fig. S6 Scanning electron microscope (SEM) revealing **(A)** the morphology of *S. thermocarboxydus* strain K155 grown in TSB medium in the absence of heavy metals, **(B)** the energy-dispersive X-ray (EDX) spectra depicting the elements (C, N, O, P, K, Na, Ca, Mg, S, Al, Zn, Co, Cu, Cd) distribution with biosorbents in the strain K155 under the same condition.

Fig. S7 Scanning electron microscope (SEM) revealing **(A)** the morphology changes of *S. thermocarboxydus* strain K155 treated with zinc (500 mg/L), **(B)** the energy-dispersive X-ray (EDX) spectra depicting the elements (C, N, O, P, K, Na, Ca, Mg, S, Al, Zn, Co, Cu, Cd) distribution with biosorbents in the strain K155 under the same condition. Bar represents 1-2 µm.

Fig. S8 Scanning electron microscope (SEM) revealing **(A)** the morphology changes of *S. thermocarboxydus* strain K155 treated with cobalt (500 mg/L), **(B)** the energy-dispersive X-ray (EDX) spectra depicting the elements (C, N, O, P, K, Na, Ca, Mg, S, Al, Zn, Co,

Cu, Cd) distribution with biosorbents in the strain K155 under the same condition. Bar represents 1-2 µm.

Fig. S9 Scanning electron microscope (SEM) revealing (A) the morphology changes of *S. thermocarboxydus* strain K155 treated with copper (100 mg/L), (B) the energy-dispersive X-ray (EDX) spectra depicting the elements (C, N, O, P, K, Na, Ca, Mg, S, Al, Zn, Co, Cu, Cd) distribution with biosorbents in the strain K155 under the same condition. Bar represents 1-2 µm.

Fig. S10 Scanning electron microscope (SEM) revealing (A) the morphology changes of *S. thermocarboxydus* strain K155 treated with cadmium (50 mg/L), (B) the energy-dispersive X-ray (EDX) spectra depicting the elements (C, N, O, P, K, Na, Ca, Mg, S, Al, Zn, Co, Cu, Cd) distribution with biosorbents in the strain K155 under the same condition. Bar represents 1-2 µm.

Fig. S11 Scanning electron microscope (SEM) revealing **(A)** the morphology of *S. thermocarboxydus* strain BPSAC147 grown in TSB medium in the absence of heavy metals, **(B)** the energy-dispersive X-ray (EDX) spectra depicting the elements (C, N, O, P, K, Na, Ca, Mg, S, Al, Zn, Co, Cu, Cd) distribution with biosorbents in the strain BPSAC147 under the same condition. Bar represents 1-2 µm.

Fig. S12 Scanning electron microscope (SEM) revealing (A) the morphology changes of *S. thermocarboxydus* strain BPSAC147 treated with zinc (1000 mg/L), (B) the energy-dispersive X-ray (EDX) spectra depicting the elements (C, N, O, P, K, Na, Ca, Mg, S, Al, Zn, Co, Cu, Cd) distribution with biosorbents in the strain BPSAC147 under the same condition. Bar represents 1-2 µm.

Fig. S13 Scanning electron microscope (SEM) revealing (A) the morphology changes of *S. thermocarboxydus* strain BPSAC147 treated with cobalt (500 mg/L), (B) the energy-dispersive X-ray (EDX) spectra depicting the elements (C, N, O, P, K, Na, Ca, Mg, S, Al, Zn, Co, Cu, Cd) distribution with biosorbents in the strain BPSAC147 under the same condition. Bar represents 1-2 µm.

Fig. S14 Scanning electron microscope (SEM) revealing (A) the morphology changes of *S. thermocarboxydus* strain BPSAC147 treated with copper (100 mg/L) (B) the energy-dispersive X-ray (EDX) spectra depicting the elements (C, N, O, P, K, Na, Ca, Mg, S,

Al, Zn, Co, Cu, Cd) distribution with biosorbents in the strain BPSAC147 under the same condition. Bar represents 1-2 µm.

Fig. S15 Scanning electron microscope (SEM) revealing (A) the morphology changes of *S. thermocarboxydus* strain BPSAC147 treated with cadmium (50 mg/L) (B) the energy-dispersive X-ray (EDX) spectra depicting the elements (C, N, O, P, K, Na, Ca, Mg, S, Al, Zn, Co, Cu, Cd) distribution with biosorbents in the strain BPSAC147 under the same condition. Bar represents 1-2 µm.

Fig. S16-S25 Fourier-transform infrared spectroscopy (FT-IR) spectra showed that the biomass of strain K155 and BPSAC147 with and without heavy metals has changed in functional groups' absorption peaks in the 400-4000 cm^{-1} range. FTIR analysis is described as follows:

S16) Control K155 (Absence of metals). The Y-axis represents the percentage of transmission (%T), which means the amount of infrared light absorbed or transmitted by the treated and untreated samples being analyzed.

S17) Strain K155 treated with zinc (1000 mg/L). The Y-axis represents the percentage of transmission (%T), which means the amount of infrared light absorbed or transmitted by the treated and untreated samples being analyzed.

S18) Strain K155 treated with cobalt (500 mg/L), The Y-axis represents the percentage of transmission (%T), which means the amount of infrared light absorbed or transmitted by the treated and untreated samples being analyzed.

S19) Strain K155 treated with copper (100 mg/L). The Y-axis represents the percentage of transmission (%T), which means the amount of infrared light absorbed or transmitted by the treated and untreated samples being analyzed.

S20) Strain K155 treated with cadmium (50 mg/L). The Y-axis represents the percentage of transmission (%T), which means the amount of infrared light absorbed or transmitted by the treated and untreated samples being analyzed.

S21) Control BPSAC147 (Absence of metals). The Y-axis represents the percentage of transmission (%T), which means the amount of infrared light absorbed or transmitted by the treated and untreated samples being analyzed.

S22) Strain BPSAC147 treated with zinc (1000 mg/L). The Y-axis represents the percentage of transmission (%T), which means the amount of infrared light absorbed or transmitted by the treated and untreated samples being analyzed.

S23) Strain BPSAC147 treated with cobalt (500 mg/L). The Y-axis represents the percentage of transmission (%T), which means the amount of infrared light absorbed or transmitted by the treated and untreated samples being analyzed.

S24) Strain BPSAC147 treated with copper (100 mg/L). The Y-axis represents the percentage of transmission (%T), which means the amount of infrared light absorbed or transmitted by the treated and untreated samples being analyzed.

S25) Strain BPSAC147 treated with cadmium (50 mg/L). The Y-axis represents the percentage of transmission (%T), which means the amount of infrared light absorbed or transmitted by the treated and untreated samples being analyzed.

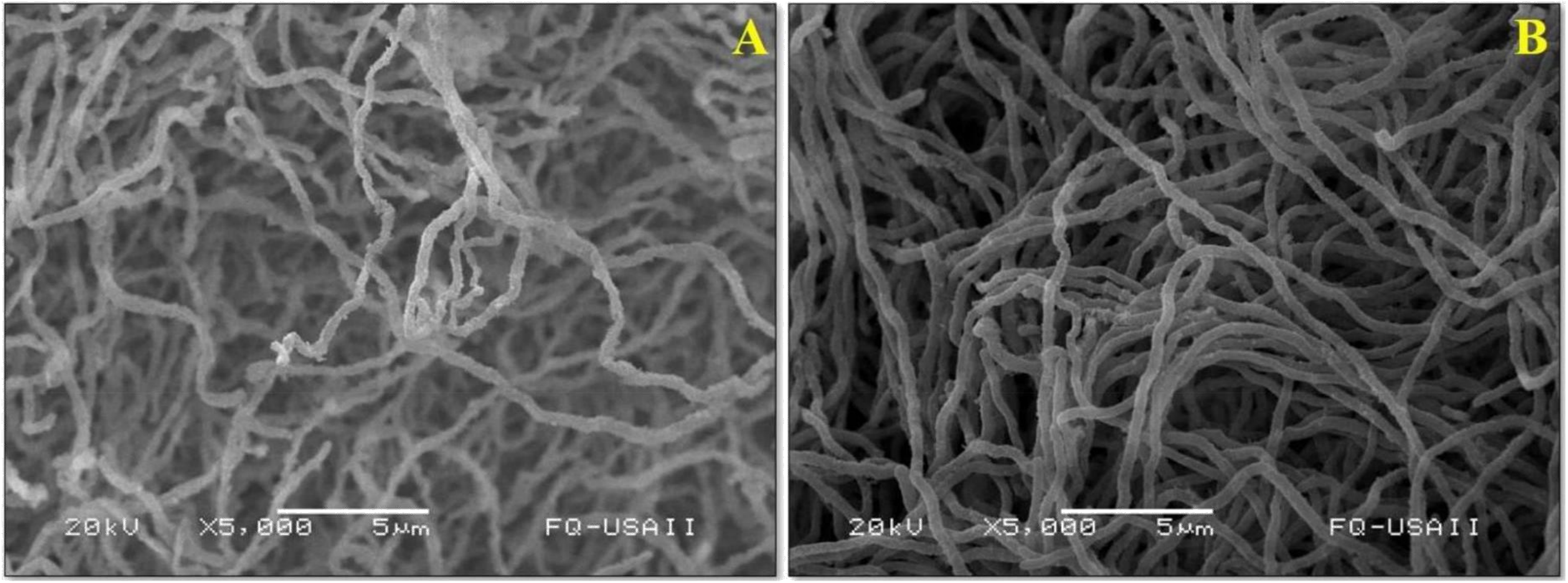

Fig.S1 Scanning electron microscope (SEM) indicates strains (**A**) *Streptomyces thermocarboxydus* strain K155; (**B**) *Streptomyces thermocarboxydus* strain BPSAC147 producing spiral long spore chain morphology.

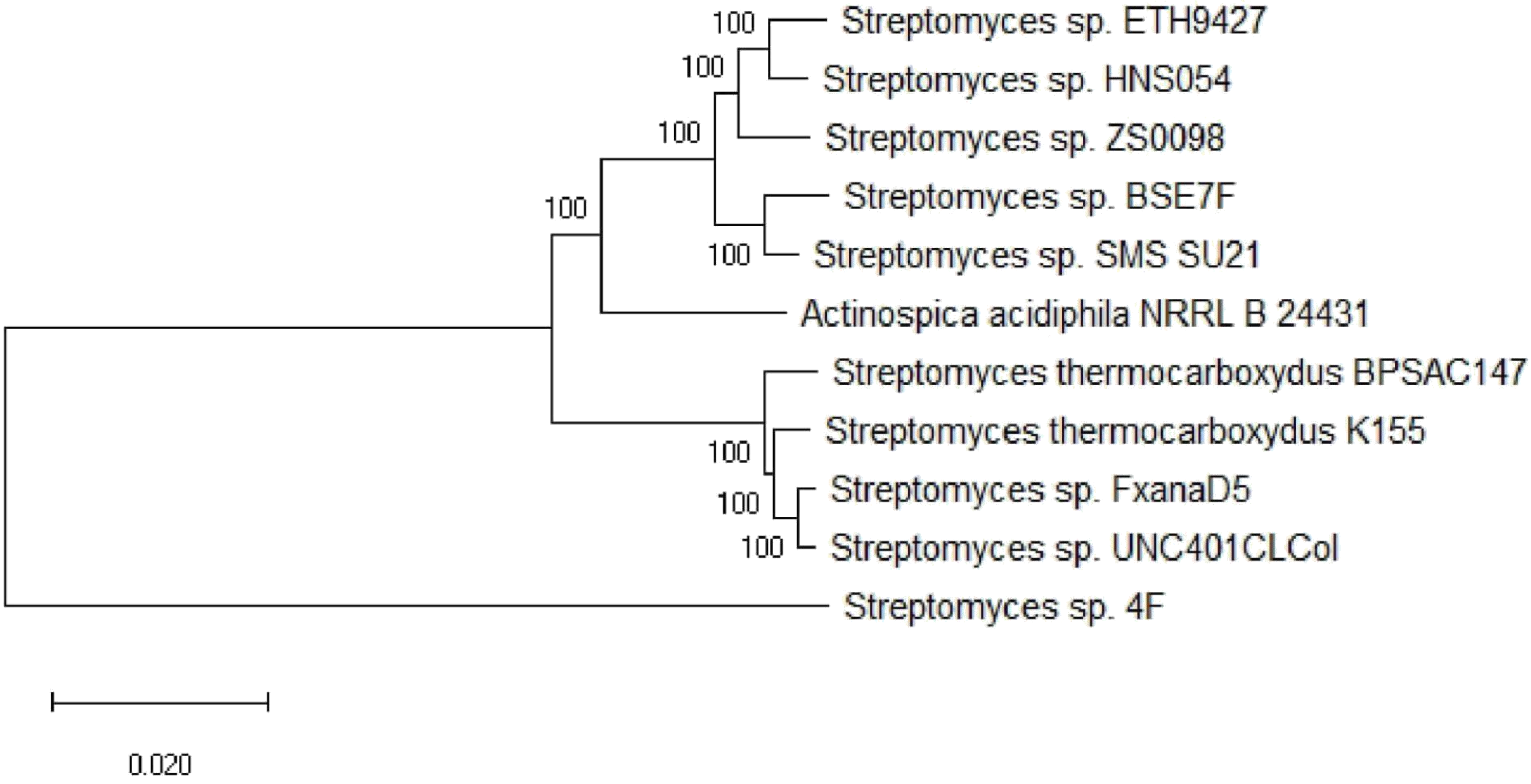

Fig. S2 Core genome-derived phylogenetic tree.

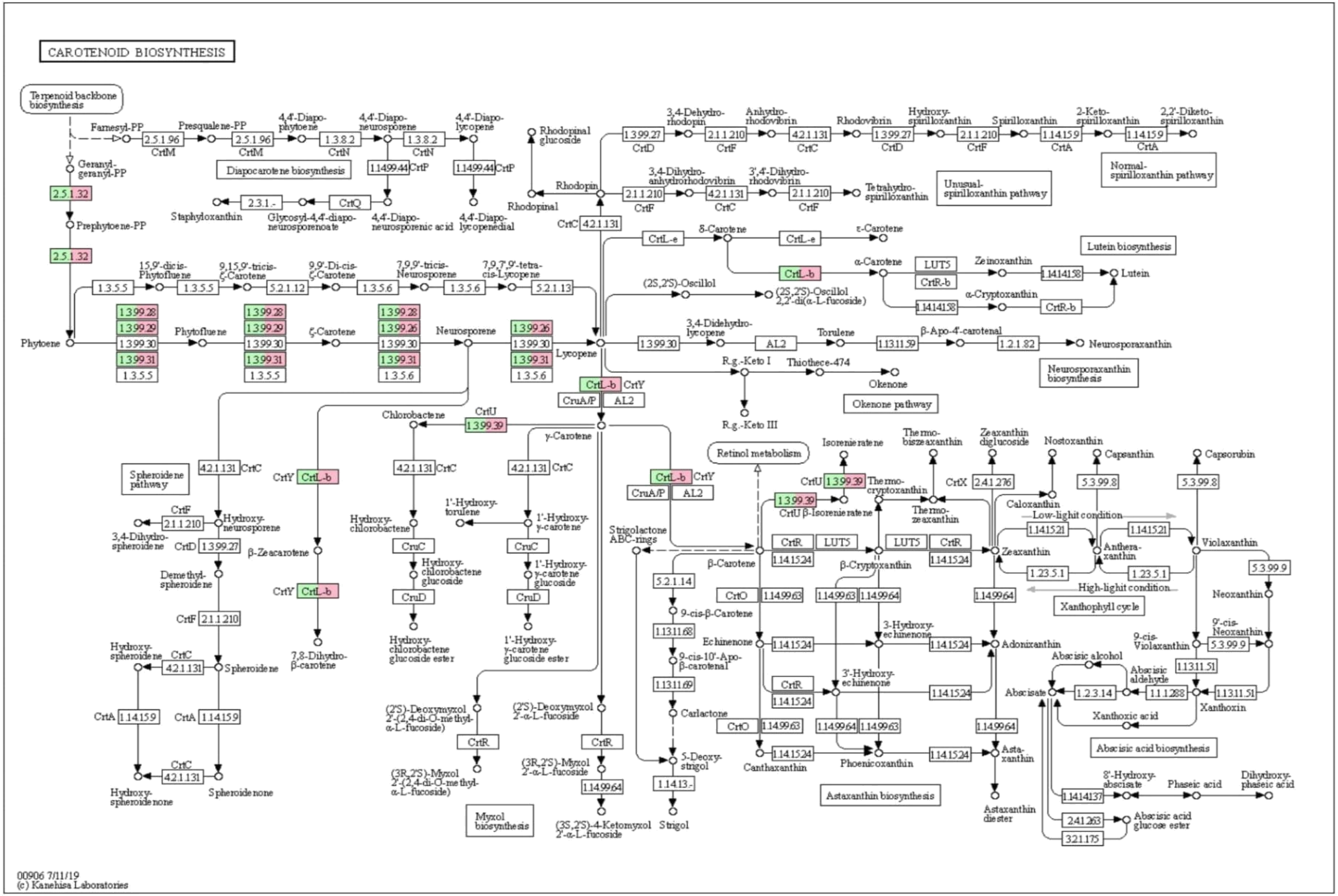

Fig. S3 Biosynthesis of carotenoid pigments present in the strain K155 and BPSAC17. Green indicates strain K155, and pink indicates strain BPS147.

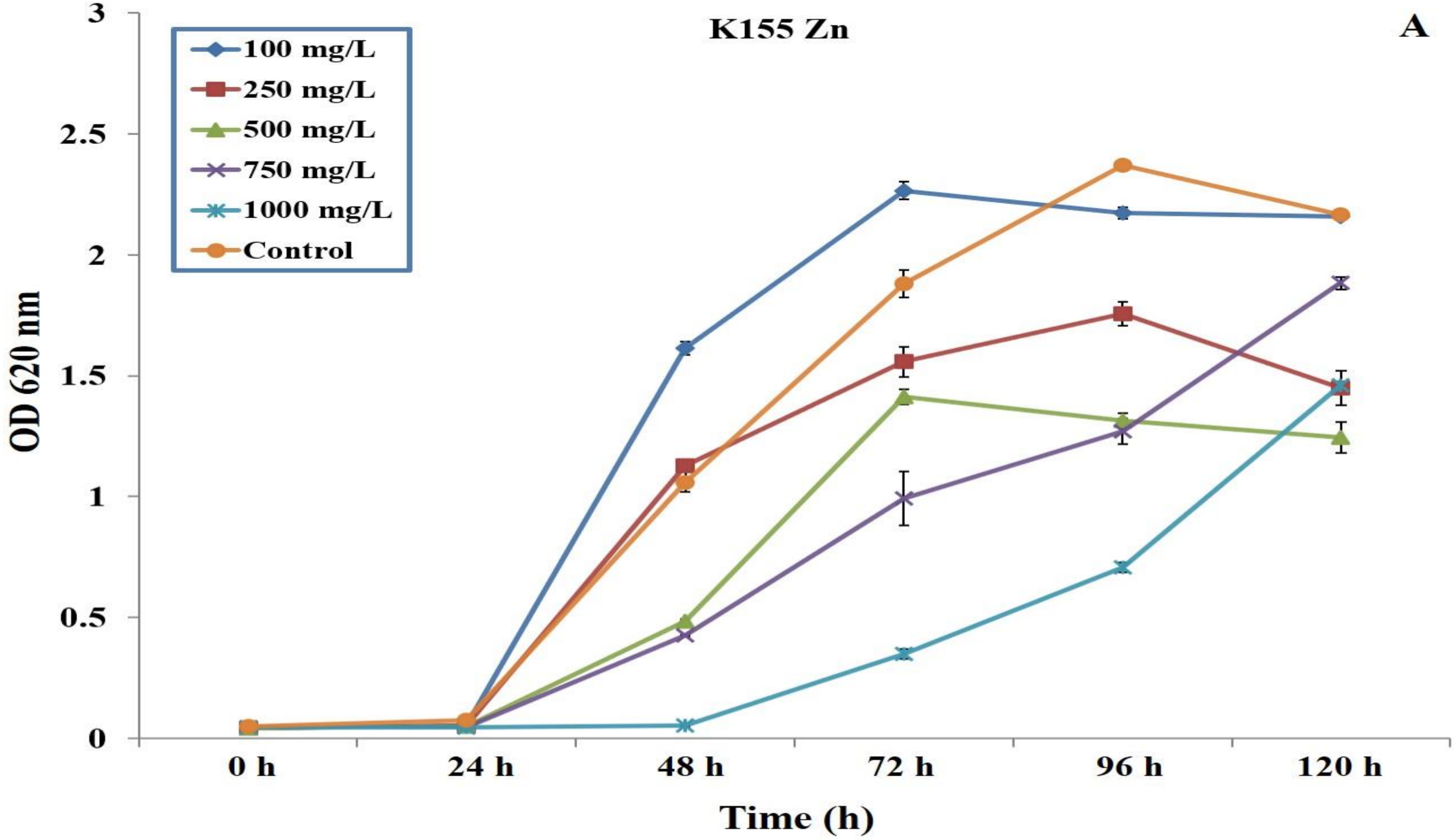

Fig. S4 Effect of different multi-metal concentrations on the growth pattern of *Streptomyces thermocarboxydus* strain K155 growing in tryptone soya broth (TSB). (**A**) Zinc (100 to 1000 mg/L). Control: Strain K155 inoculated in TSB broth without any heavy metals and incubated at 28 ^{o}C for up to 120 h. The values were performed in a triplicates process, and error bars indicate the mean ± standard deviations (SD).

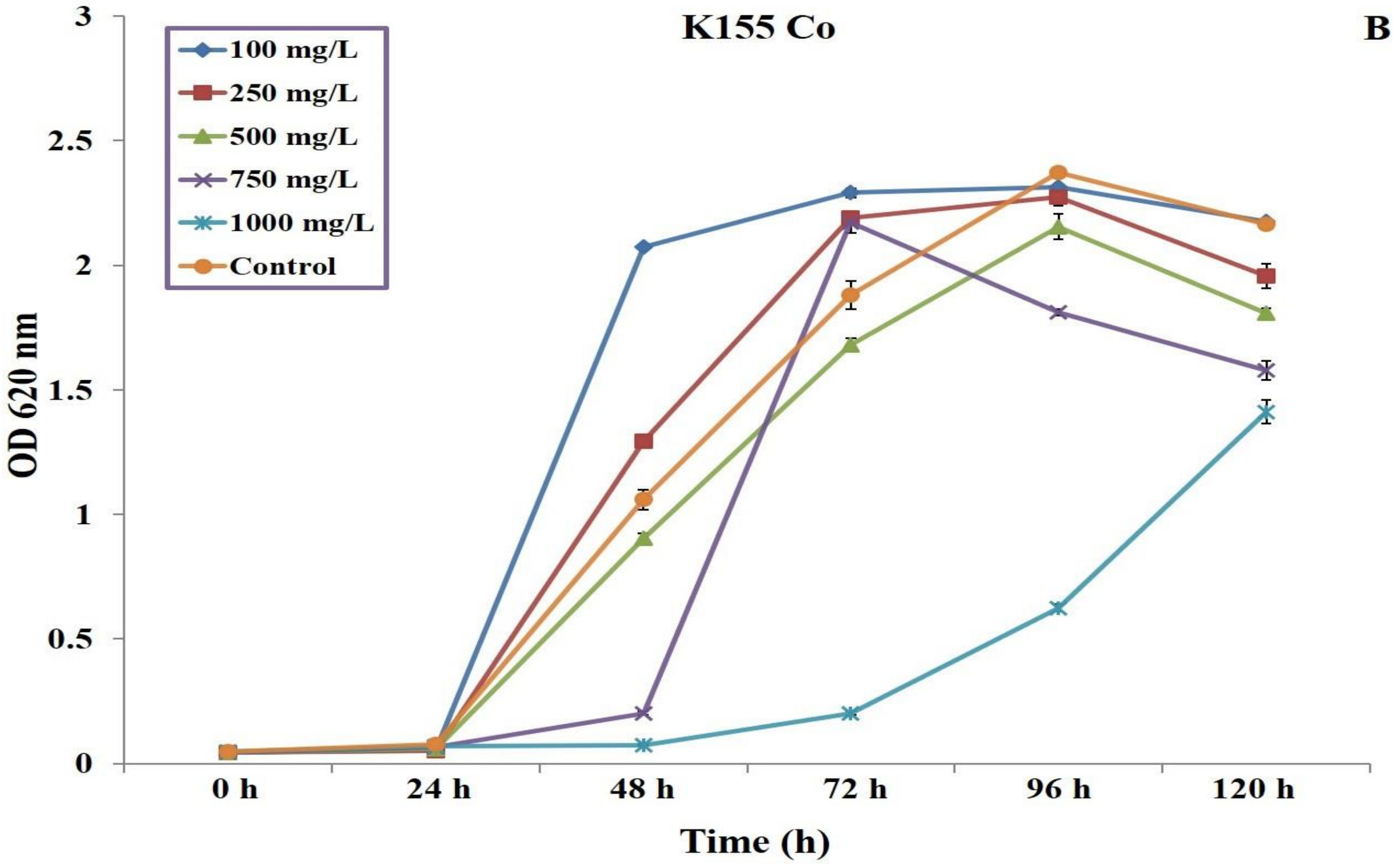

Fig. S4 Effect of different multi-metal concentrations on the growth pattern of *Streptomyces thermocarboxydus* strain K155 growing in tryptone soya broth (TSB). (**B**) Cobalt (100 to 1000 mg/L). Control: Strain K155 inoculated in TSB broth without any heavy metals and incubated at 28 ^{o}C for up to 120 h. The values were performed in a triplicates process, and error bars indicate the mean ± standard deviations (SD).

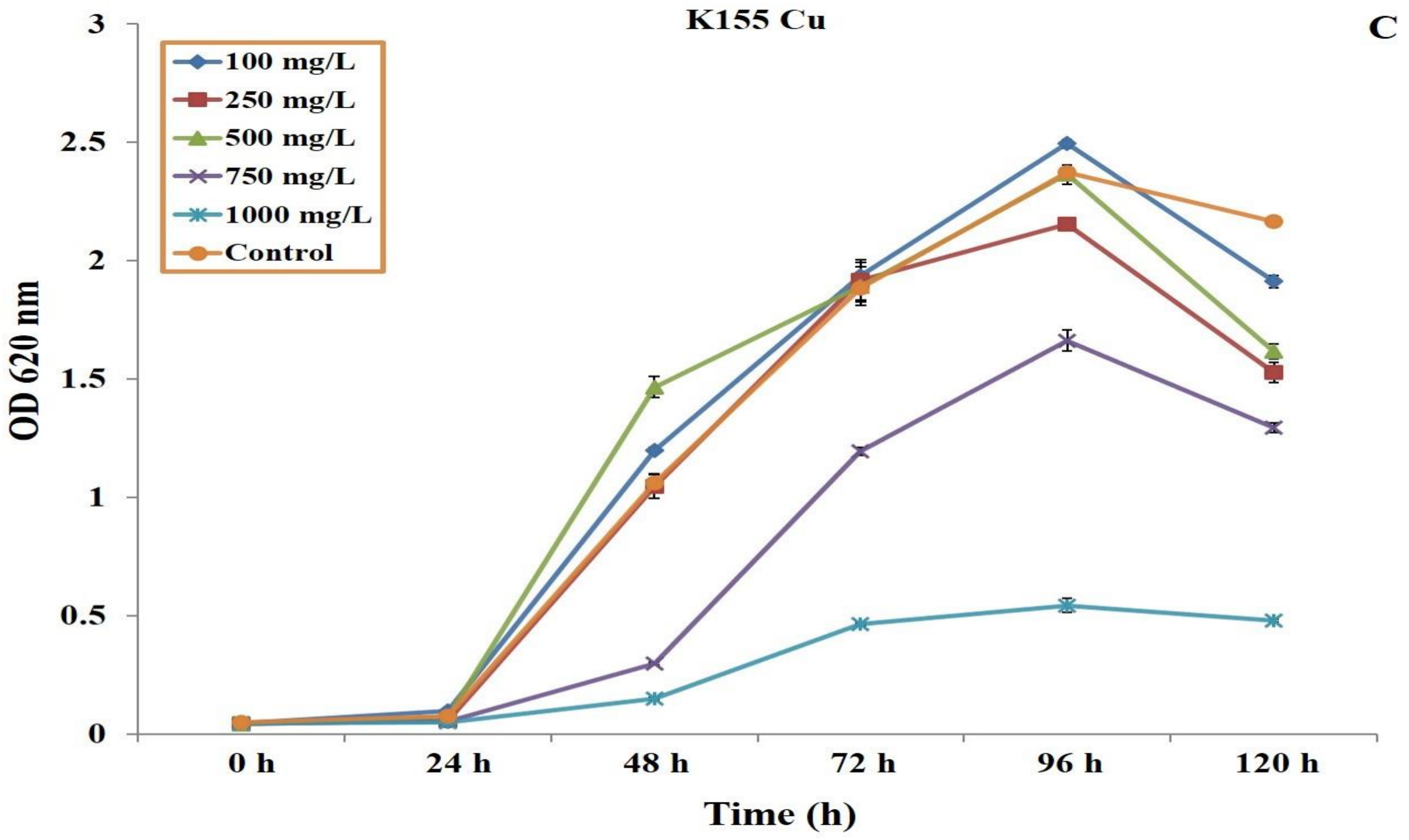

Fig. S4 Effect of different multi-metal concentrations on the growth pattern of *Streptomyces thermocarboxydus* strain K155 growing in tryptone soya broth (TSB). (**C**) Copper (100 to 1000 mg/L). Control: Strain K155 inoculated in TSB broth without any heavy metals and incubated at 28 ^{o}C for up to 120 h. The values were performed in a triplicates process, and error bars indicate the mean ± standard deviations (SD).

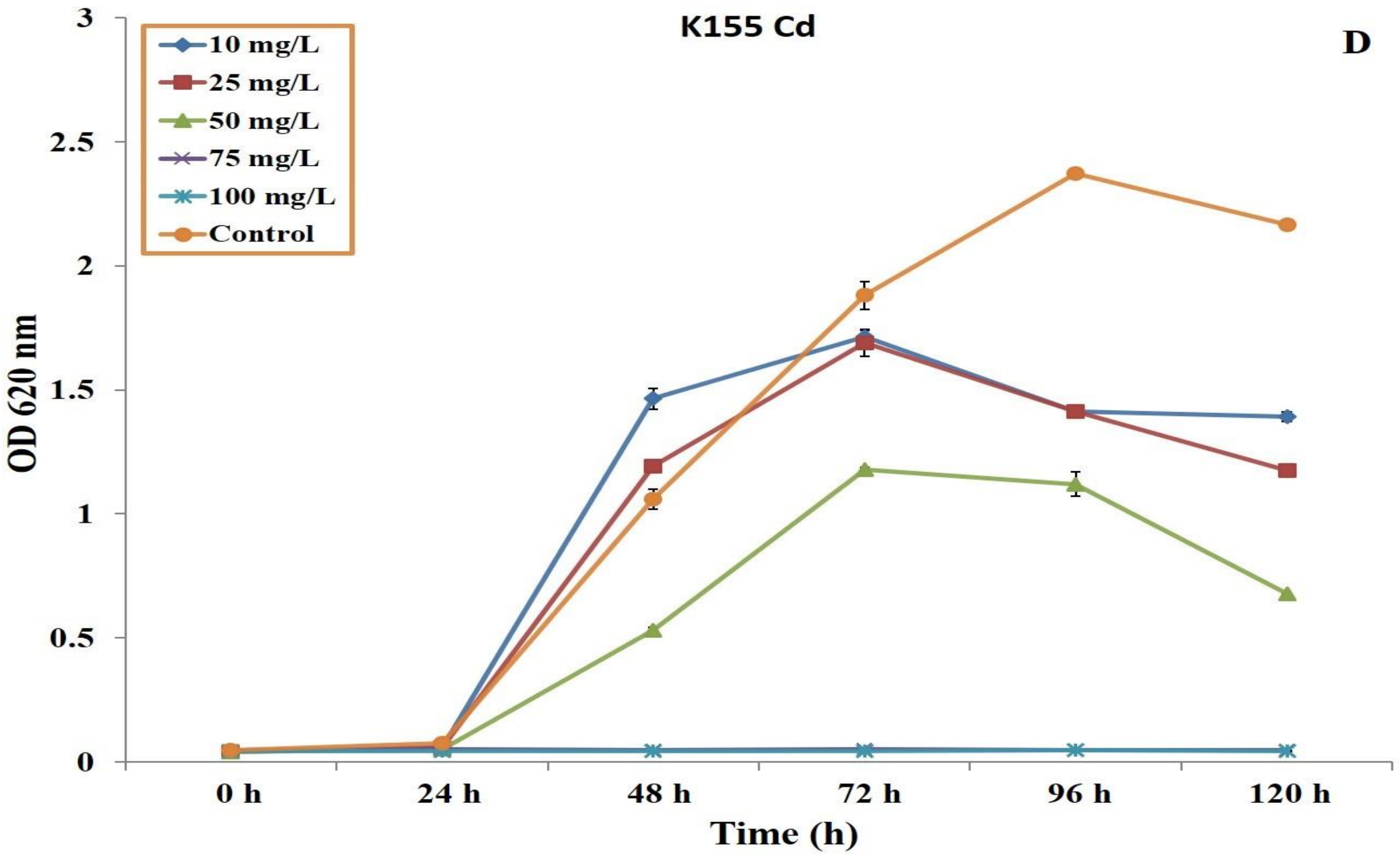

Fig. S4 Effect of different multi-metal concentrations on the growth pattern of *Streptomyces thermocarboxydus* strain K155 growing in tryptone soya broth (TSB). (**D**) Cadmium (10 to 100 mg/L). Control: Strain K155 inoculated in TSB broth without any heavy metals and incubated at 28 ^{o}C for up to 120 h. The values were performed in a triplicates process, and error bars indicate the mean ± standard deviations (SD).

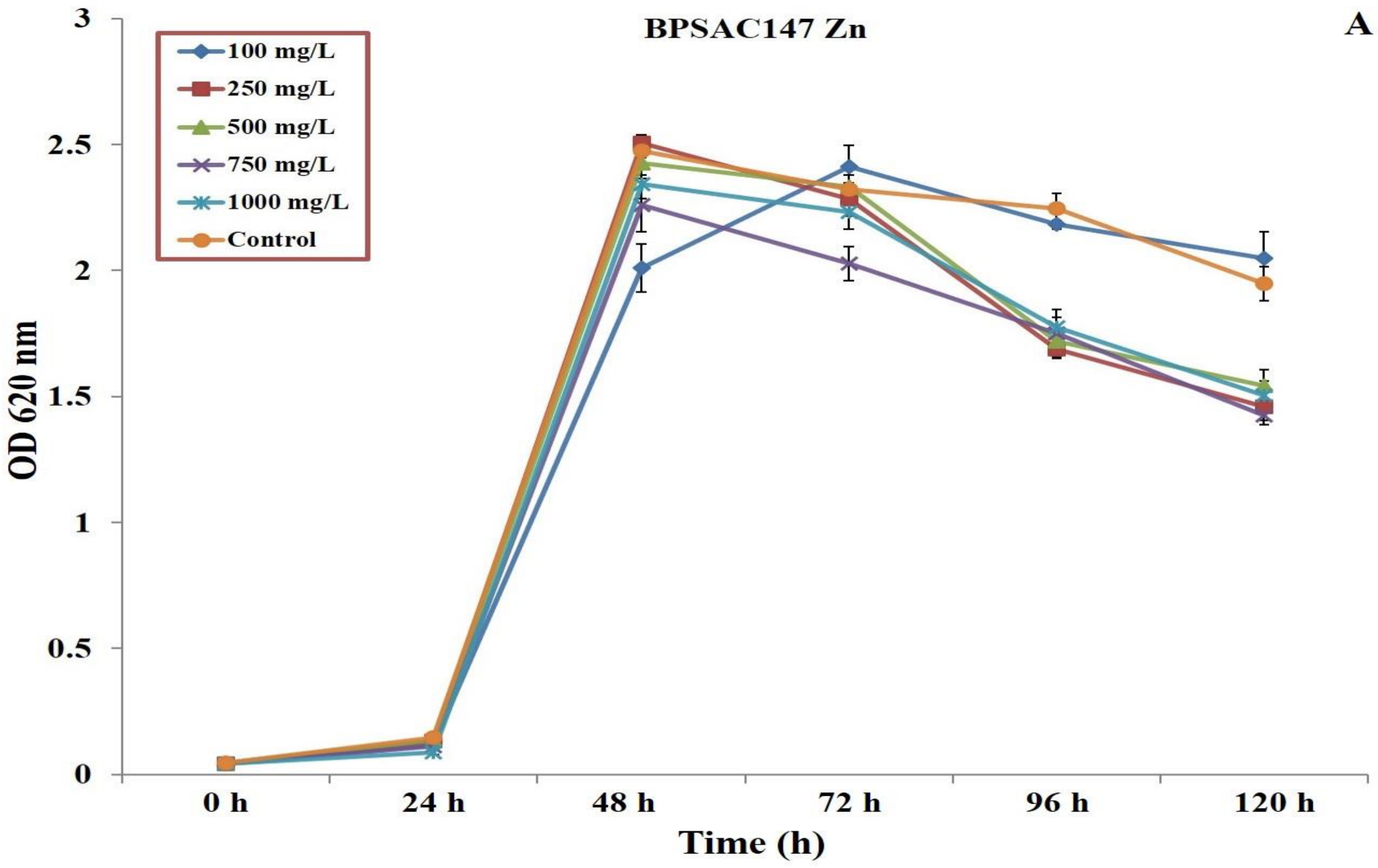

Fig. S5 Effect of different multi-metal concentrations on the growth pattern of *Streptomyces thermocarboxydus* BPSAC147 growing in tryptone soya broth (TSB). (**A**) Zinc (100 to 1000 mg/L). Control: Strain BPSAC147 inoculated in TSB broth without any heavy metals and incubated at 28 ^{o}C for up to 120 h. The values were determined in triplicate, and error bars indicate the mean ± standard deviation (SD).

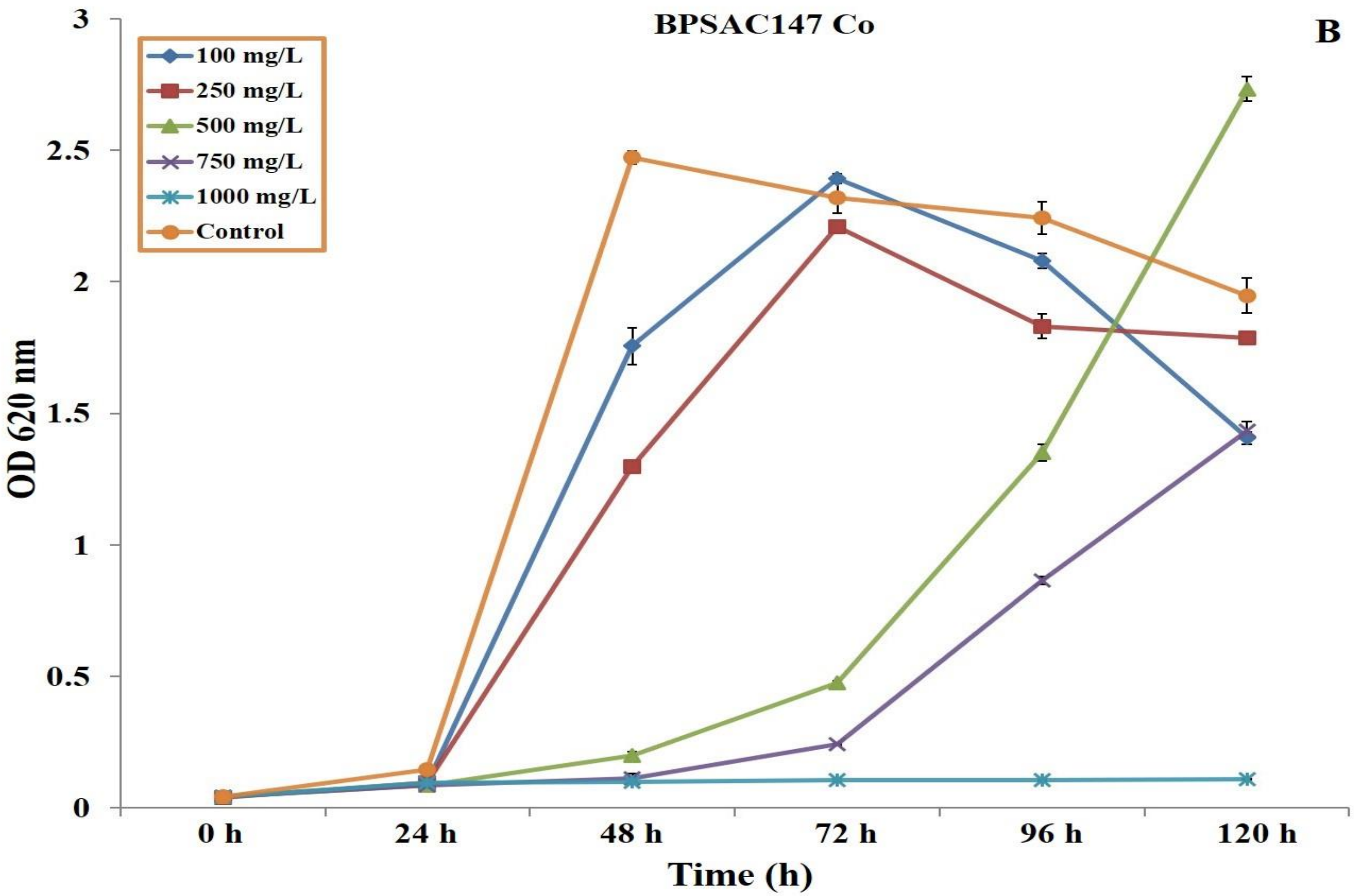

Fig. S5 Effect of different multi-metal concentrations on the growth pattern of *Streptomyces thermocarboxydus* BPSAC147 growing in tryptone soya broth (TSB). (**B**) Cobalt (100 to 1000 mg/L). Control: Strain BPSAC147 inoculated in TSB broth without any heavy metals and incubated at 28 ^{o}C for up to 120 h. The values were determined in triplicate, and error bars indicate the mean ± standard deviation (SD).

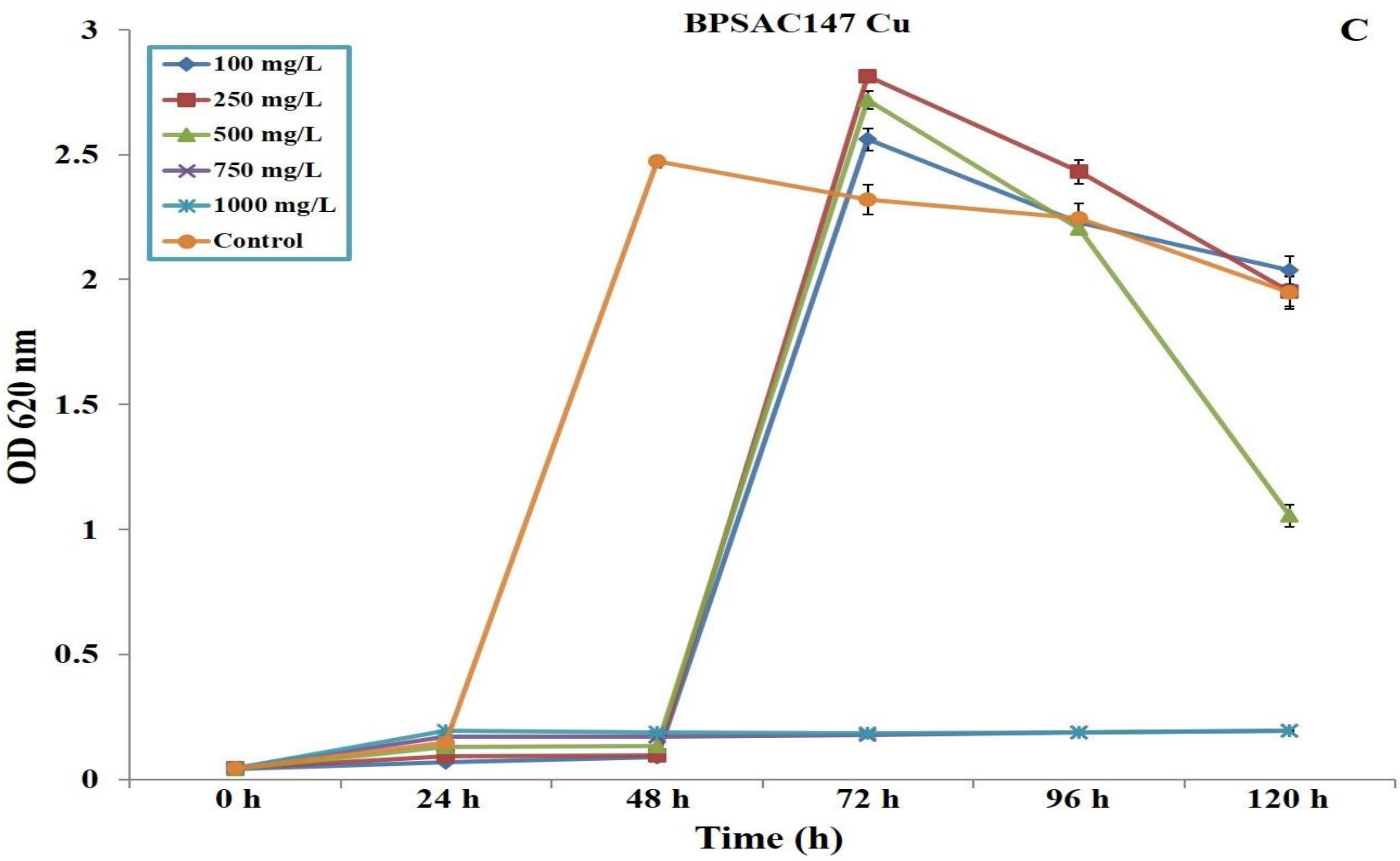

Fig. S5 Effect of different multi-metal concentrations on the growth pattern of *Streptomyces thermocarboxydus* BPSAC147 growing in tryptone soya broth (TSB). (**C**) Copper (100 to 1000 mg/L). Control: Strain BPSAC147 inoculated in TSB broth without any heavy metals and incubated at 28 ^{o}C for up to 120 h. The values were determined in triplicate, and error bars indicate the mean ± standard deviation (SD).

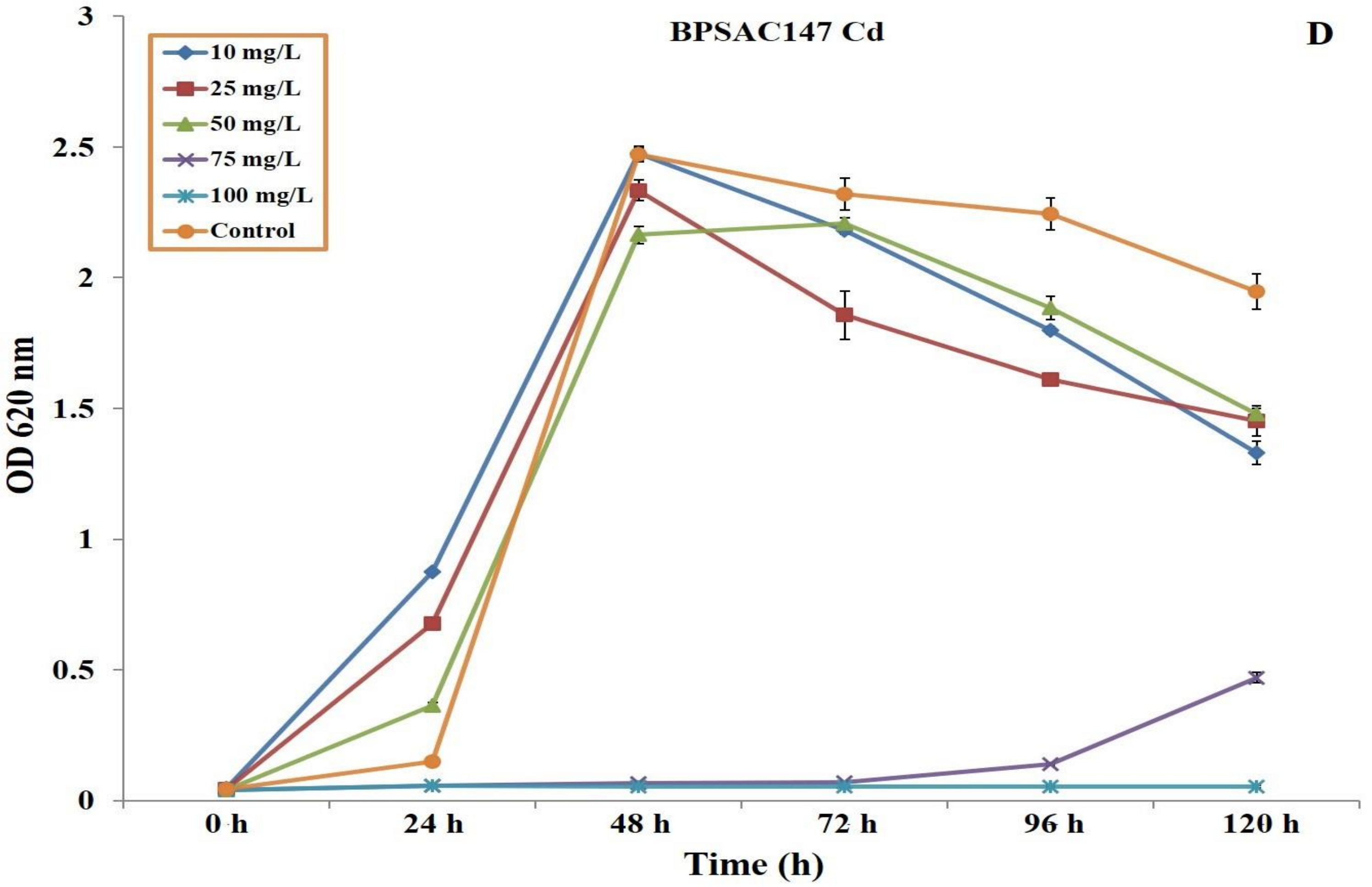

Fig. S5 Effect of different multi-metal concentrations on the growth pattern of *Streptomyces thermocarboxydus* BPSAC147 growing in tryptone soya broth (TSB). (**D**) Cadmium (10 to 100 mg/L). Control: Strain BPSAC147 inoculated in TSB broth without any heavy metals and incubated at 28 ^{o}C for up to 120 h. The values were determined in triplicate, and error bars indicate the mean ± standard deviation (SD).

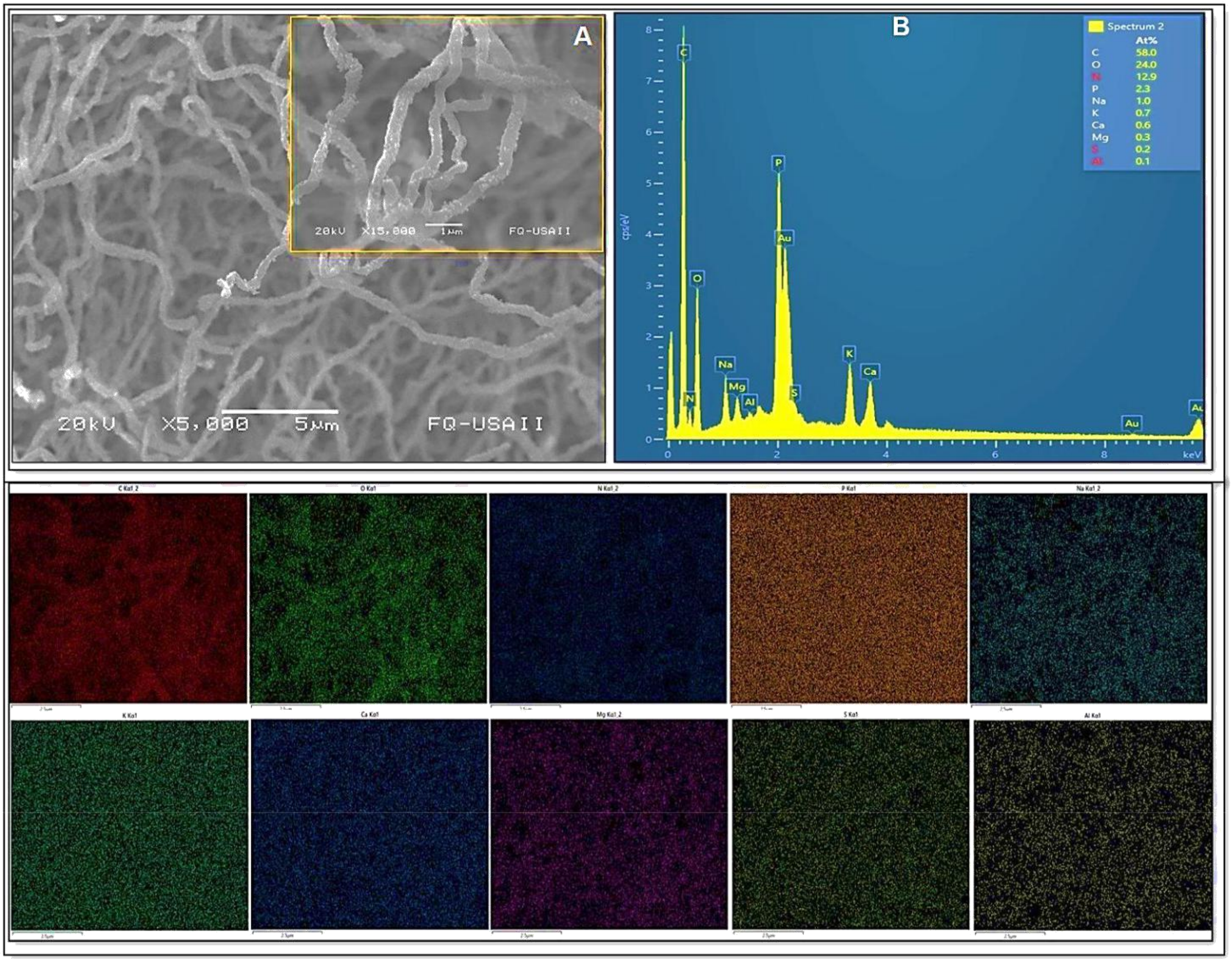

Fig. S6 Scanning electron microscope (SEM) revealing **(A)** the morphology of *S. thermocarboxydus* strain K155 grown in TSB medium in the absence of heavy metals, **(B)** the energy-dispersive X-ray (EDX) spectra depicting the elements (C, N, O, P, K, Na, Ca, Mg, S, Al, Zn, Co, Cu, Cd) distribution with biosorbents in the strain K155 under the same condition.

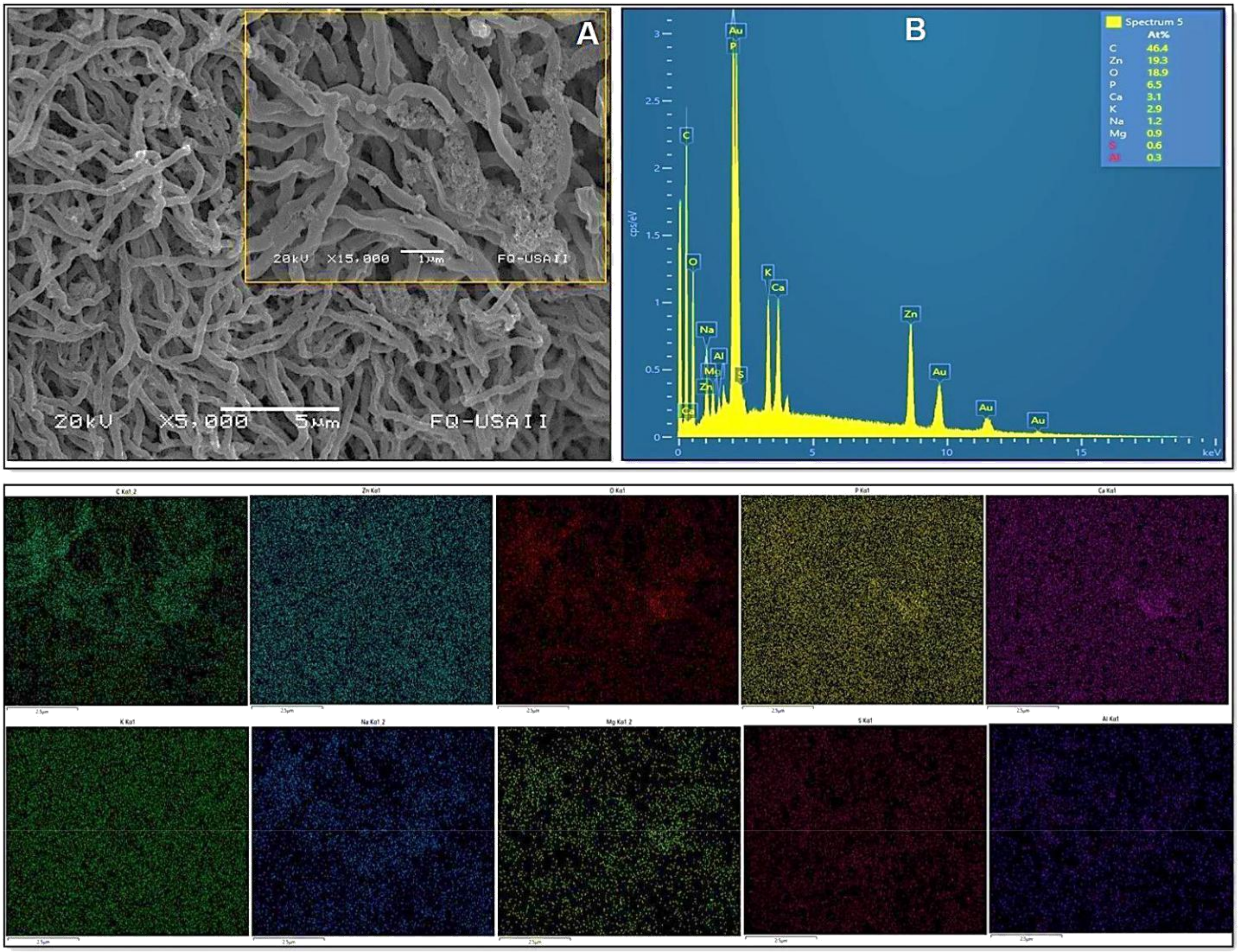

Fig. S7 Scanning electron microscope (SEM) revealing **(A)** the morphology changes of *S. thermocarboxydus* strain K155 treated with zinc (500 mg/L), **(B)** the energy-dispersive X-ray (EDX) spectra depicting the elements (C, N, O, P, K, Na, Ca, Mg, S, Al, Zn, Co, Cu, Cd) distribution with biosorbents in the strain K155 under the same condition. Bar represents 1-2 µm.

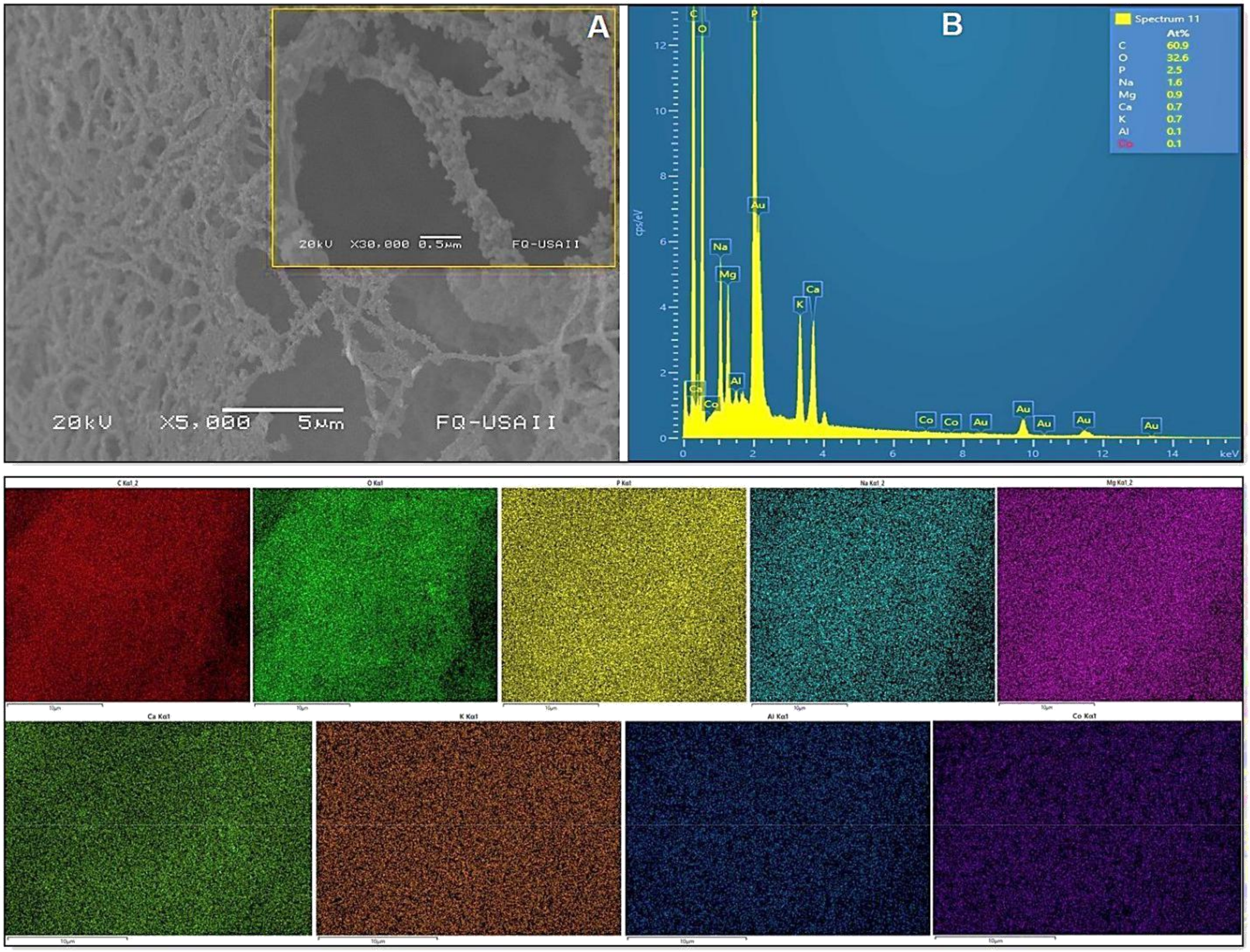

Fig. S8 Scanning electron microscope (SEM) revealing **(A)** the morphology changes of *S. thermocarboxydus* strain K155 treated with cobalt (500 mg/L), **(B)** the energy-dispersive X-ray (EDX) spectra depicting the elements (C, N, O, P, K, Na, Ca, Mg, S, Al, Zn, Co, Cu, Cd) distribution with biosorbents in the strain K155 under the same condition. Bar represents 1-2 µm.

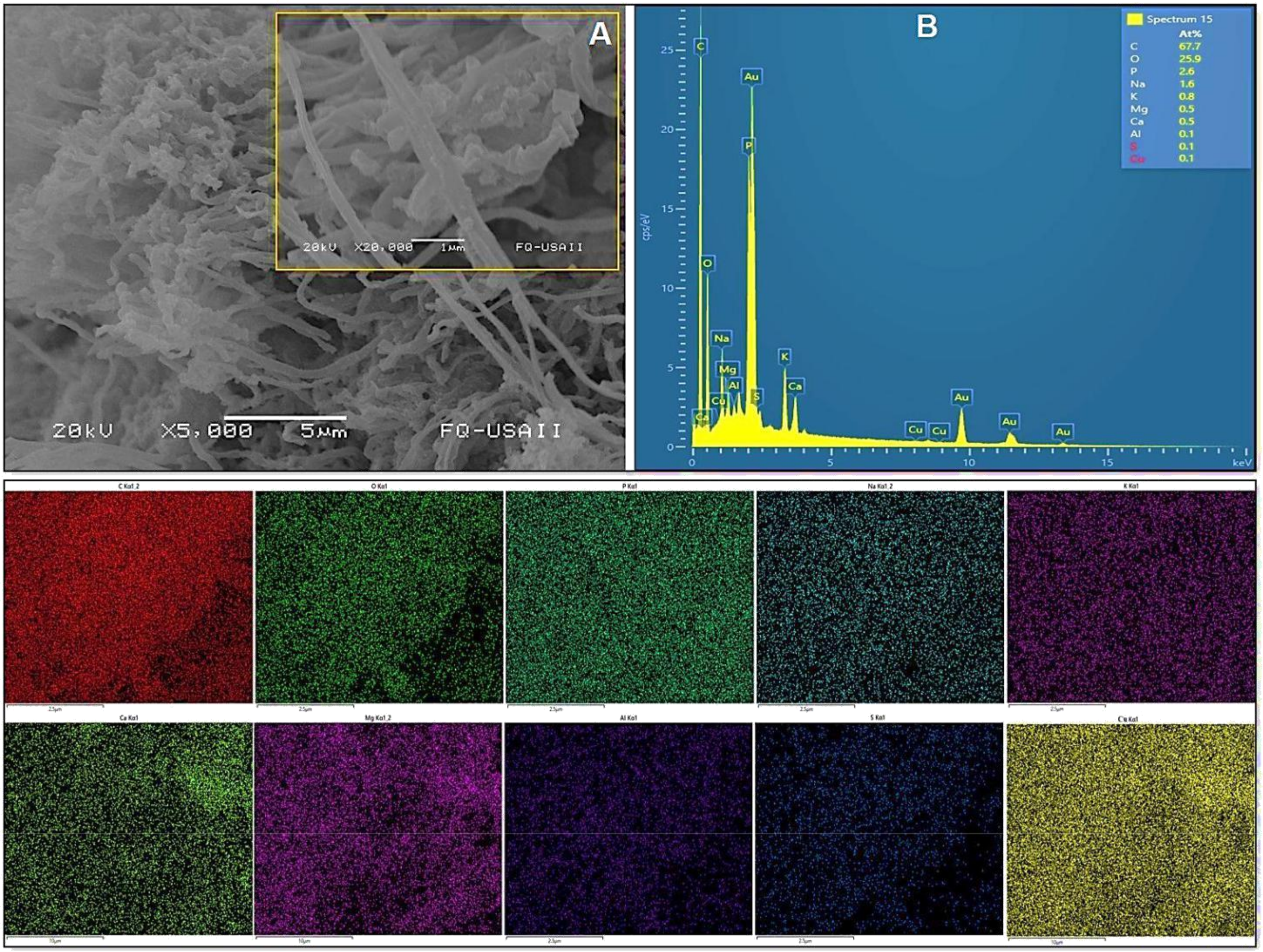

Fig. S9 Scanning electron microscope (SEM) revealing (A) the morphology changes of *S. thermocarboxydus* strain K155 treated with copper (100 mg/L), (B) the energy-dispersive X-ray (EDX) spectra depicting the elements (C, N, O, P, K, Na, Ca, Mg, S, Al, Zn, Co, Cu, Cd) distribution with biosorbents in the strain K155 under the same condition. Bar represents 1-2 µm.

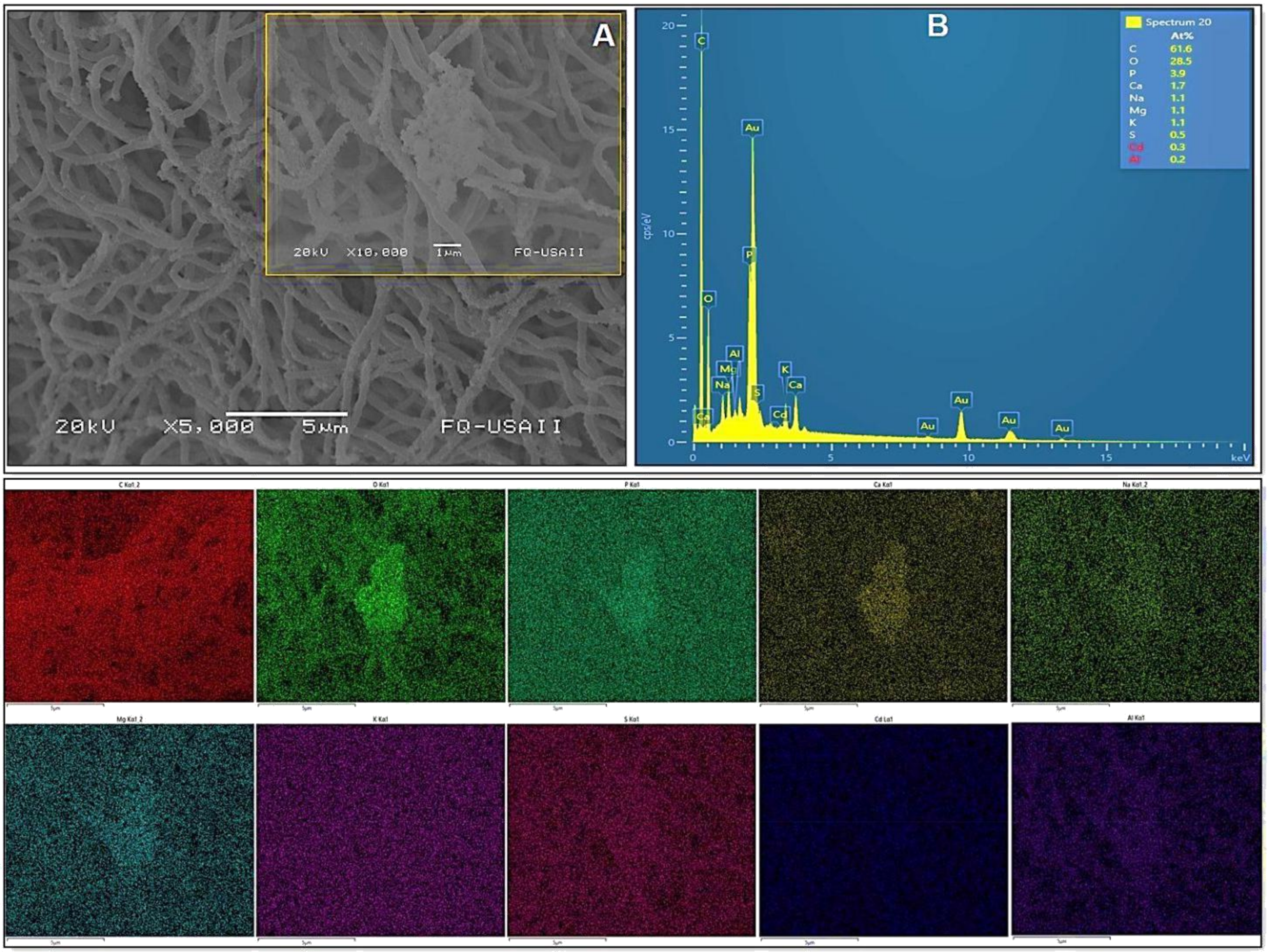

Fig. S10 Scanning electron microscope (SEM) revealing (A) the morphology changes of *S. thermocarboxydus* strain K155 treated with cadmium (50 mg/L), (B) the energy-dispersive X-ray (EDX) spectra depicting the elements (C, N, O, P, K, Na, Ca, Mg, S, Al, Zn, Co, Cu, Cd) distribution with biosorbents in the strain K155 under the same condition. Bar represents 1-2 µm.

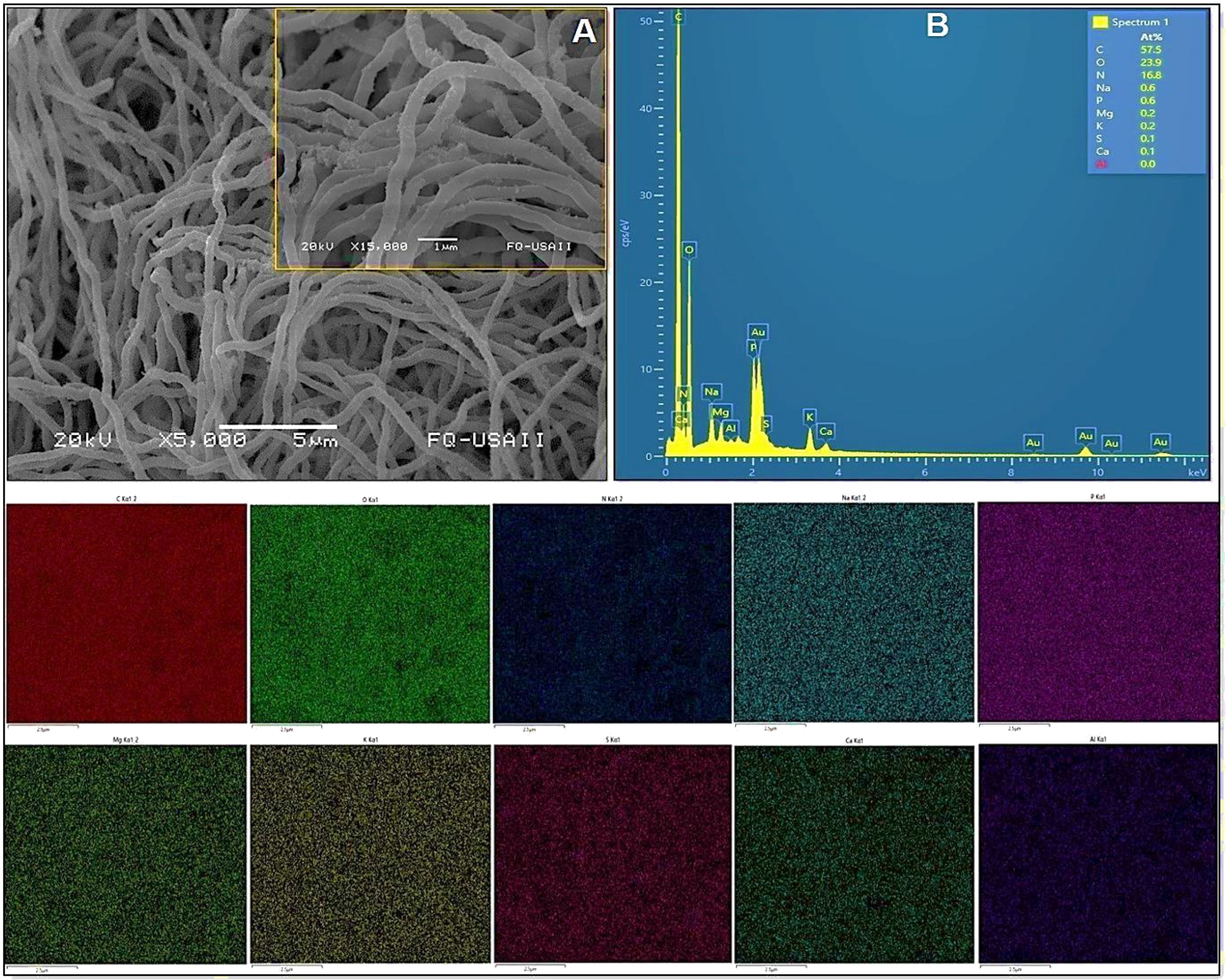

Fig. S11 Scanning electron microscope (SEM) revealing **(A)** the morphology of *S. thermocarboxydus* strain BPSAC147 grown in TSB medium in the absence of heavy metals, **(B)** the energy-dispersive X-ray (EDX) spectra depicting the elements (C, N, O, P, K, Na, Ca, Mg, S, Al, Zn, Co, Cu, Cd) distribution with biosorbents in the strain BPSAC147 under the same condition. Bar represents 1-2 µm.

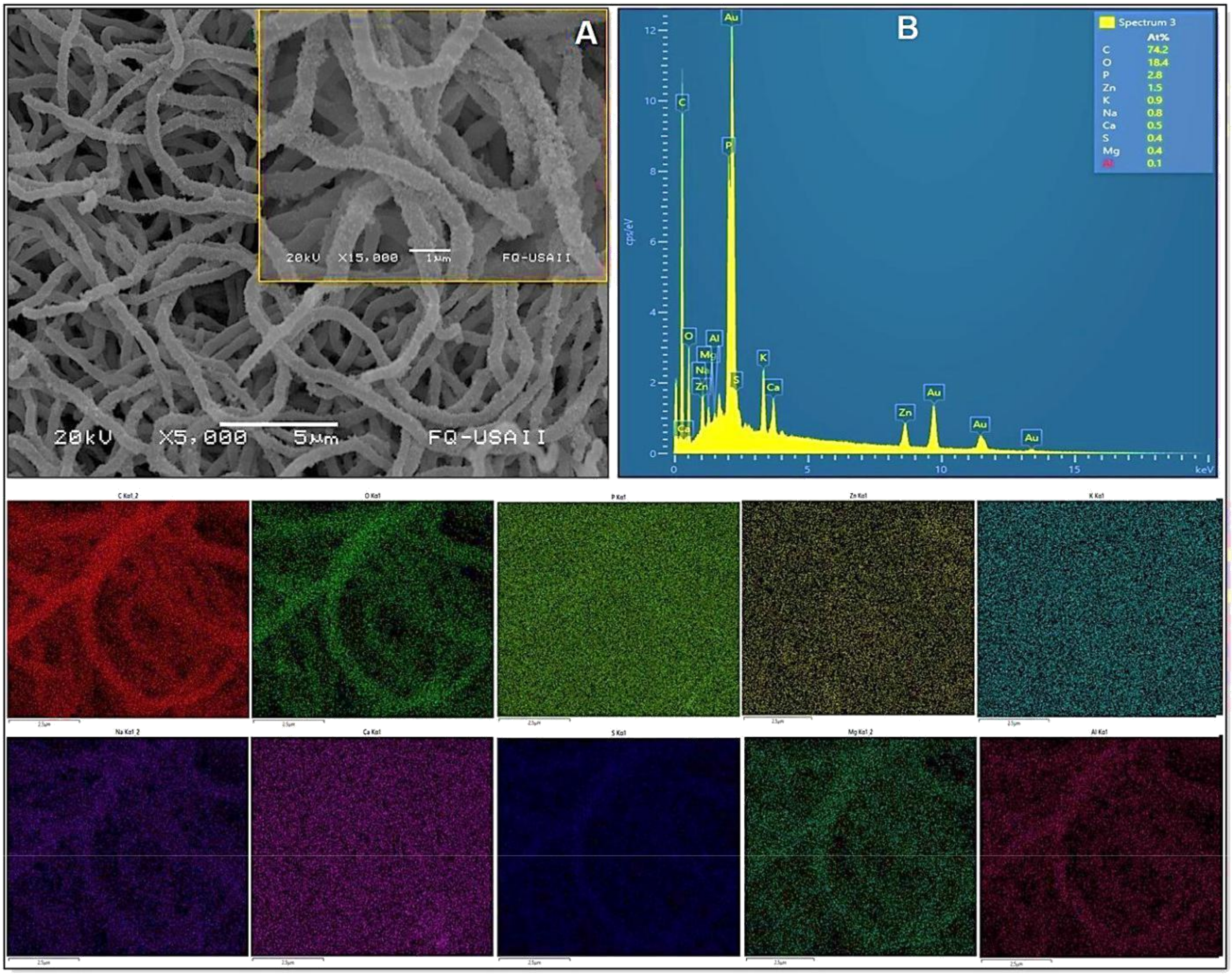

Fig. S12 Scanning electron microscope (SEM) revealing (A) the morphology changes of *S. thermocarboxydus* strain BPSAC147 treated with zinc (1000 mg/L), (B) the energy-dispersive X-ray (EDX) spectra depicting the elements (C, N, O, P, K, Na, Ca, Mg, S, Al, Zn, Co, Cu, Cd) distribution with biosorbents in the strain BPSAC147 under the same condition. Bar represents 1-2 μm.

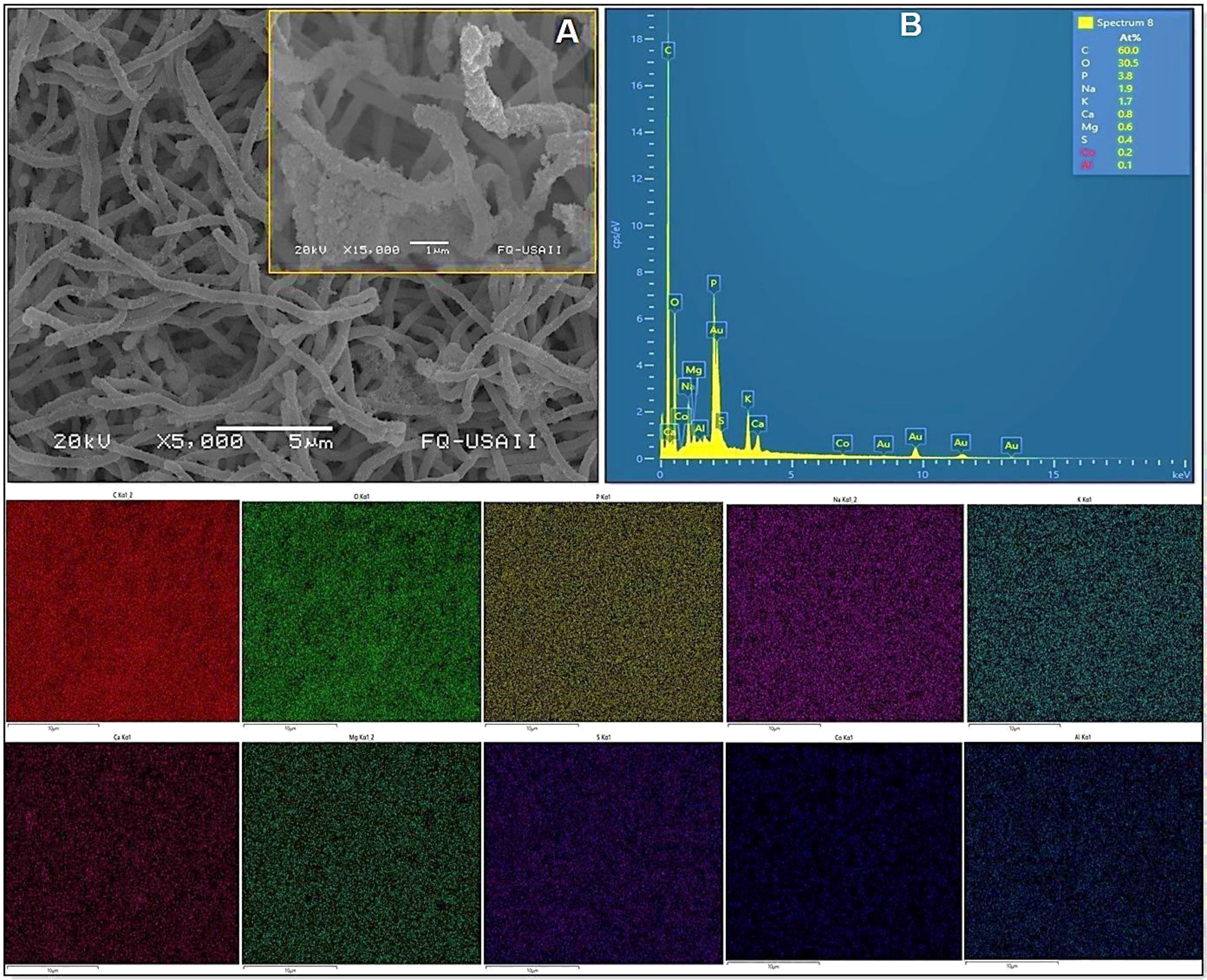

Fig. S13 Scanning electron microscope (SEM) revealing (A) the morphology changes of *S. thermocarboxydus* strain BPSAC147 treated with cobalt (500 mg/L), (B) the energy-dispersive X-ray (EDX) spectra depicting the elements (C, N, O, P, K, Na, Ca, Mg, S, Al, Zn, Co, Cu, Cd) distribution with biosorbents in the strain BPSAC147 under the same condition. Bar represents 1-2 µm.

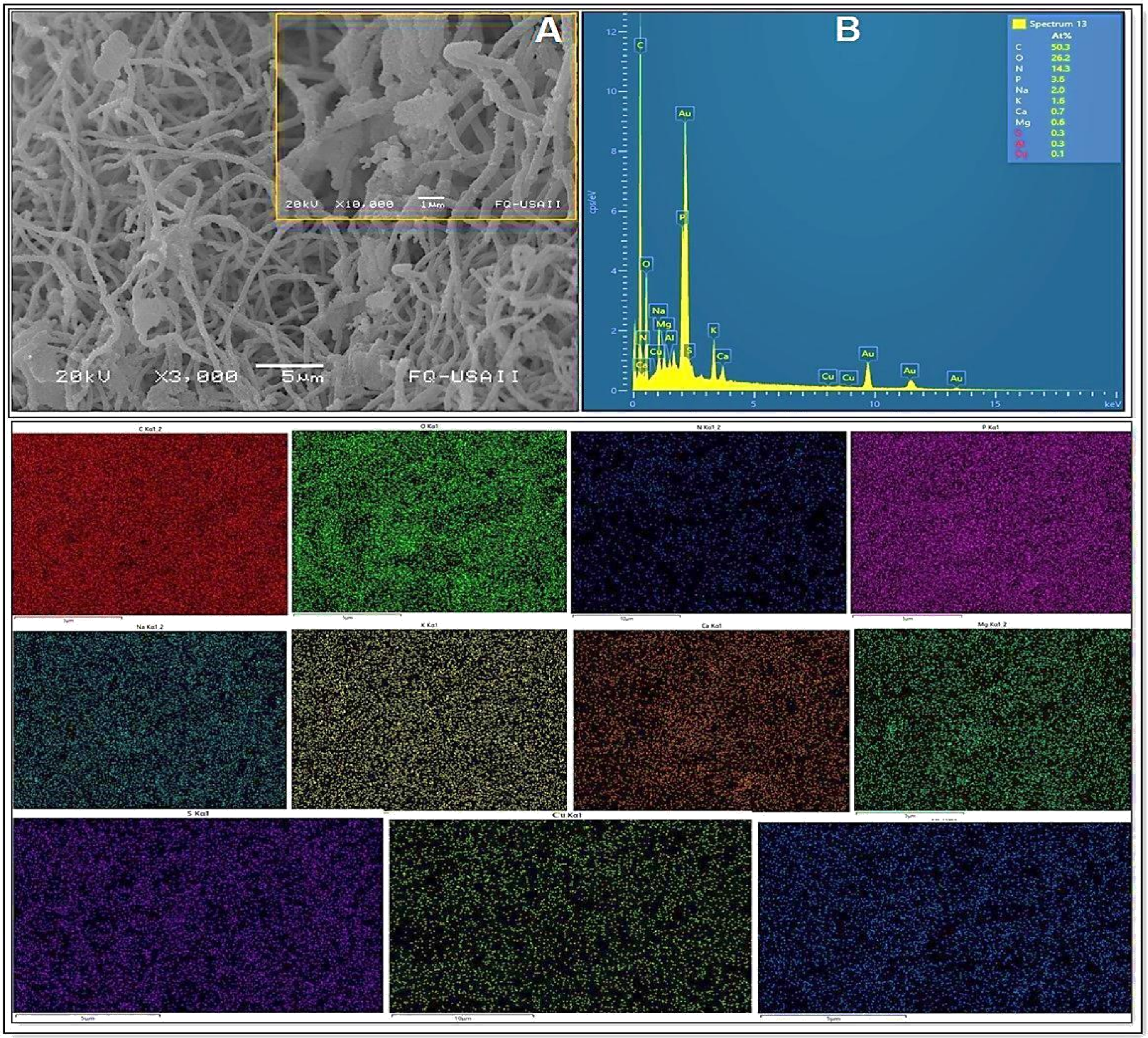

Fig. S14 Scanning electron microscope (SEM) revealing (A) the morphology changes of *S. thermocarboxydus* strain BPSAC147 treated with copper (100 mg/L) (B) the energy-dispersive X-ray (EDX) spectra depicting the elements (C, N, O, P, K, Na, Ca, Mg, S, Al, Zn, Co, Cu, Cd) distribution with biosorbents in the strain BPSAC147 under the same condition. Bar represents 1-2 µm.

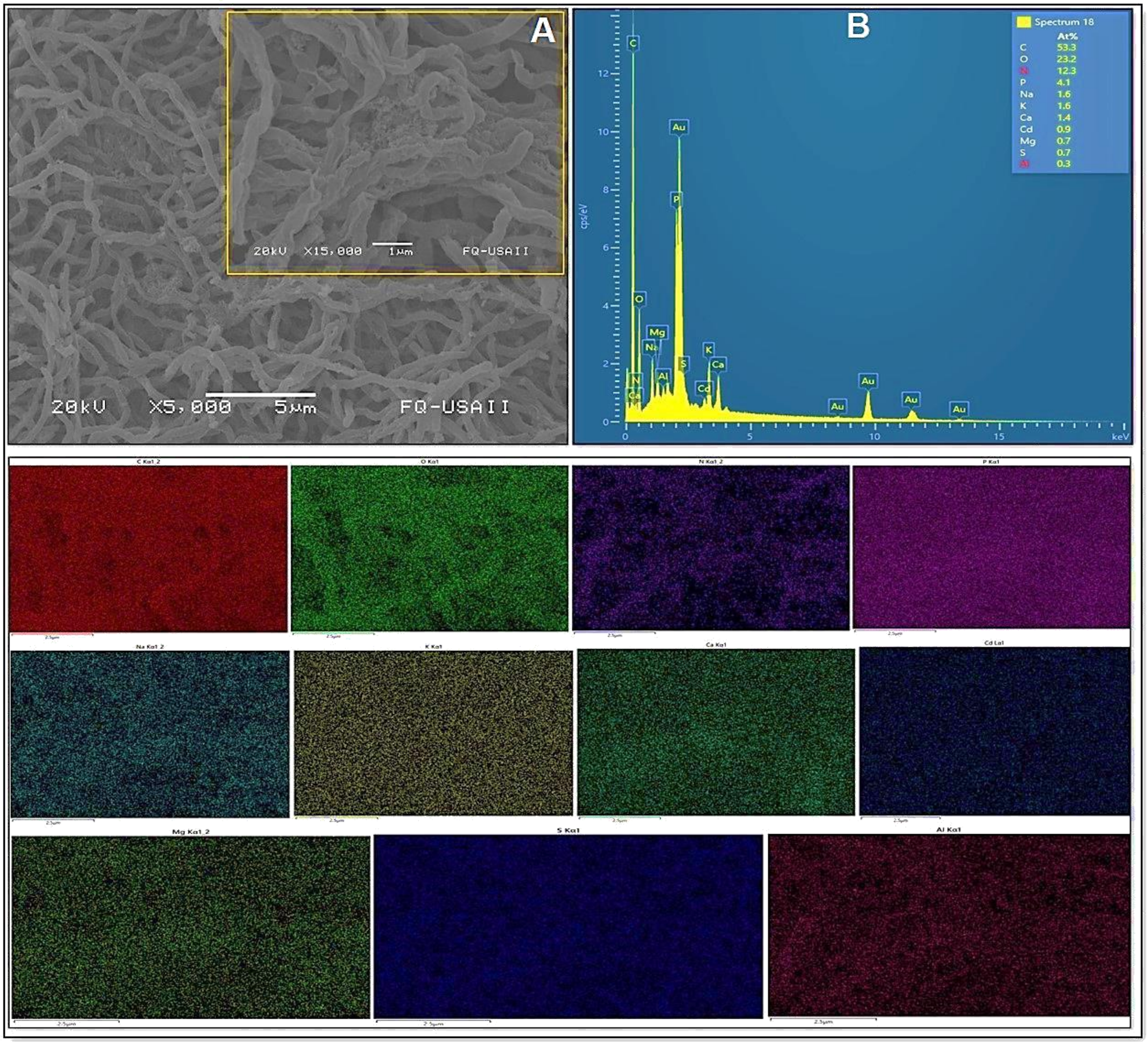

Fig. S15 Scanning electron microscope (SEM) revealing (A) the morphology changes of *S. thermocarboxydus* strain BPSAC147 treated with cadmium (50 mg/L) (B) the energy-dispersive X-ray (EDX) spectra depicting the elements (C, N, O, P, K, Na, Ca, Mg, S, Al, Zn, Co, Cu, Cd) distribution with biosorbents in the strain BPSAC147 under the same condition. Bar represents 1-2 µm.

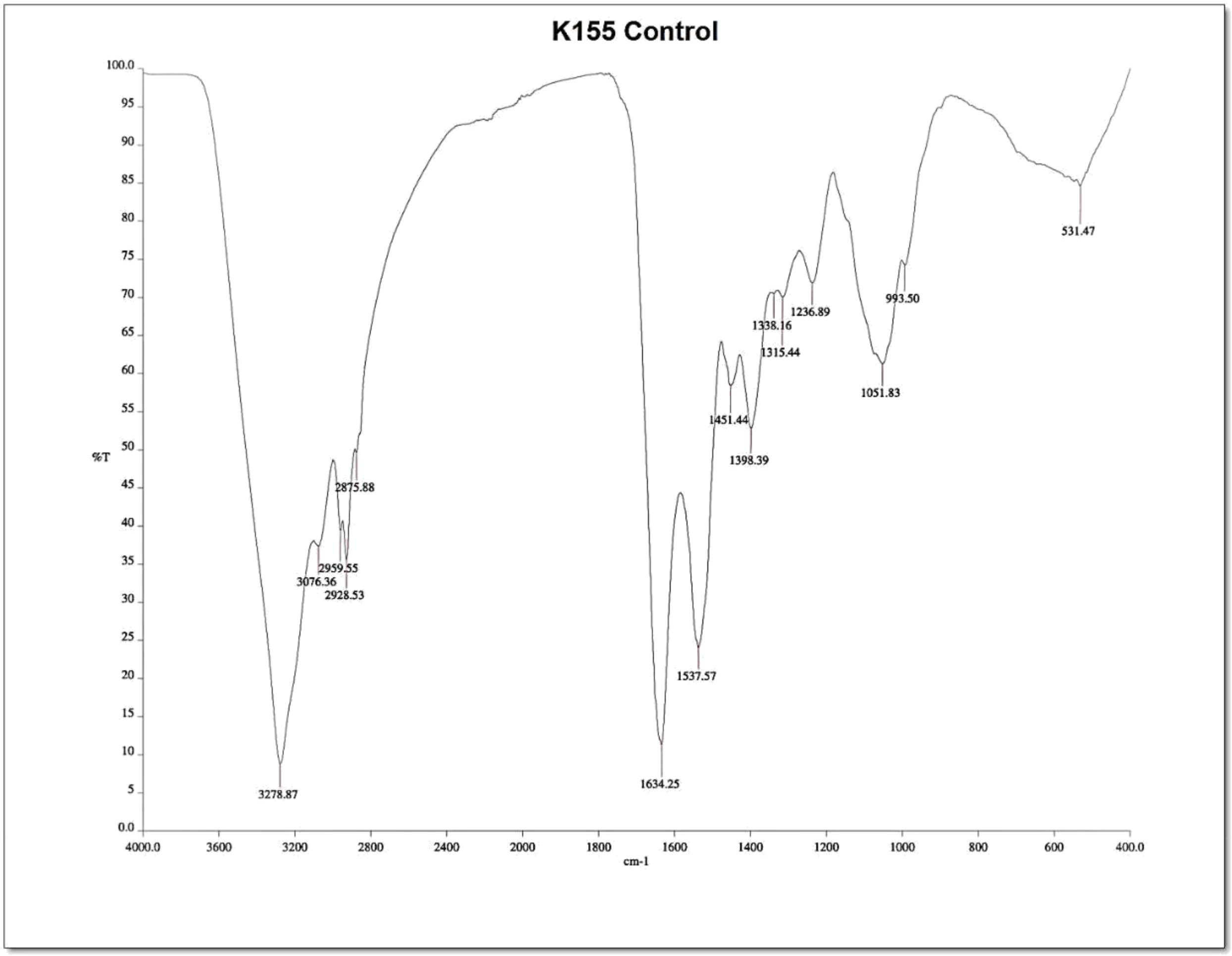

Fig. S16 Fourier-transform infrared spectroscopy (FT-IR) spectra showed that the biomass of strain K155 with and without heavy metals has changed in functional groups' absorption peaks in the 400-4000 cm^{-1} range. Control K155 (Absence of metals). The Y-axis represents the percentage of transmission (%T), which means the amount of infrared light absorbed or transmitted by the treated and untreated samples being analyzed.

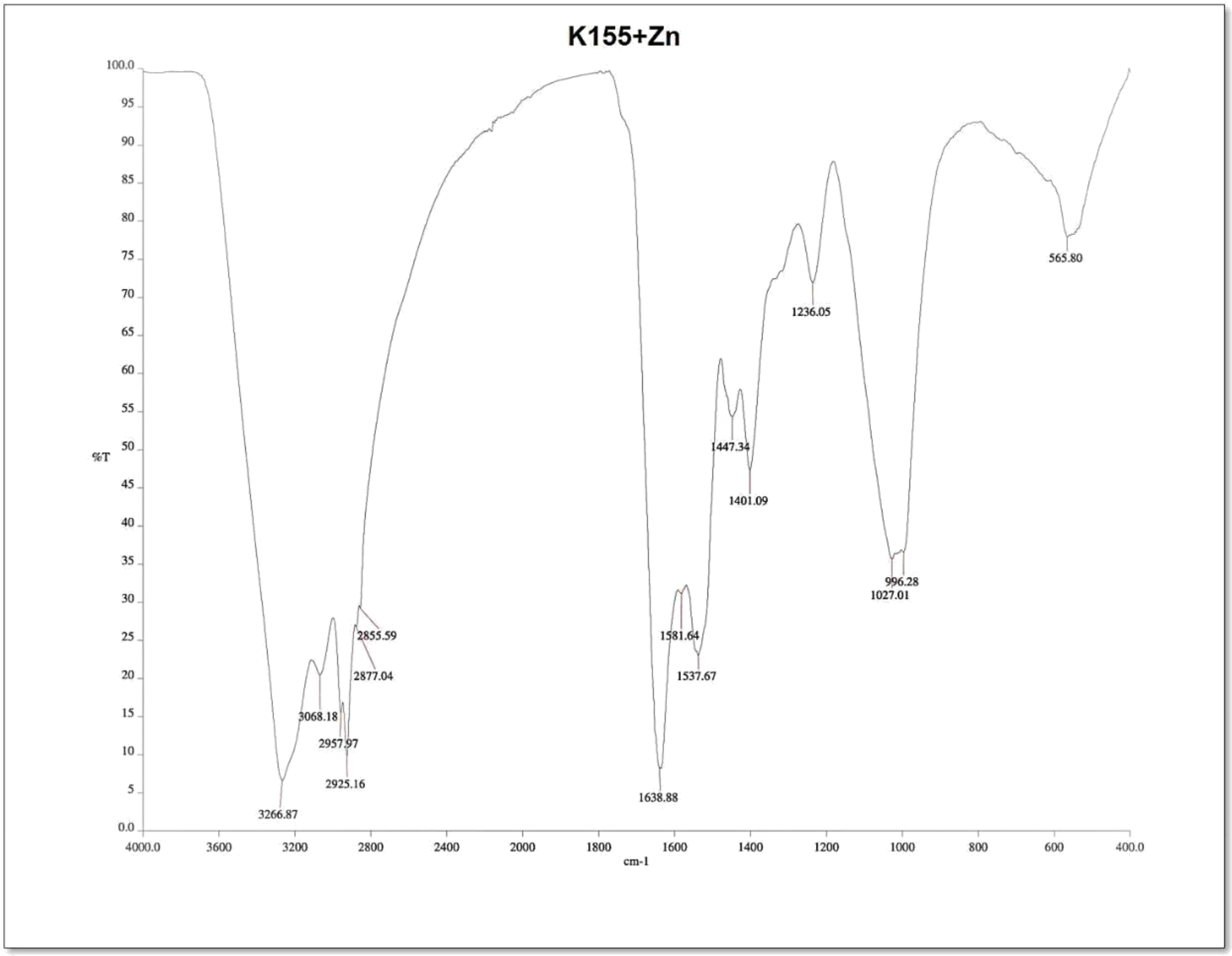

Fig. S17 Fourier-transform infrared spectroscopy (FT-IR) spectra showed that the biomass of strain K155 with and without heavy metals has changed in functional groups' absorption peaks in the 400-4000 cm^{-1} range. Strain K155 treated with zinc (1000 mg/L). The Y-axis represents the percentage of transmission (%T), which means the amount of infrared light absorbed or transmitted by the treated and untreated samples being analyzed.

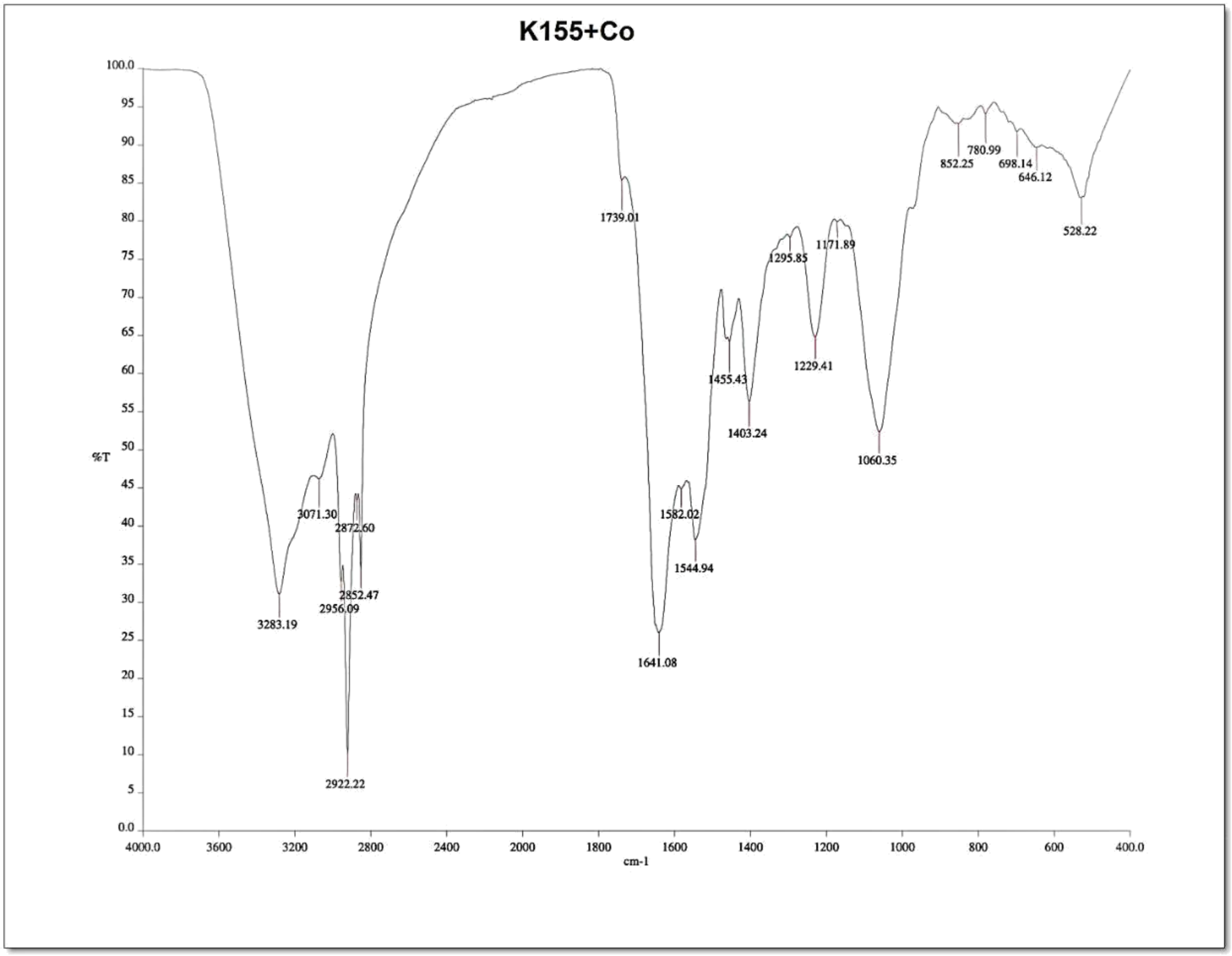

Fig. S18 Fourier-transform infrared spectroscopy (FT-IR) spectra showed that the biomass of strain K155 with and without heavy metals has changed in functional groups' absorption peaks in the 400-4000 cm^{-1} range. Strain K155 treated with cobalt (500 mg/L), The Y-axis represents the percentage of transmission (%T), which means the amount of infrared light absorbed or transmitted by the treated and untreated samples being analyzed.

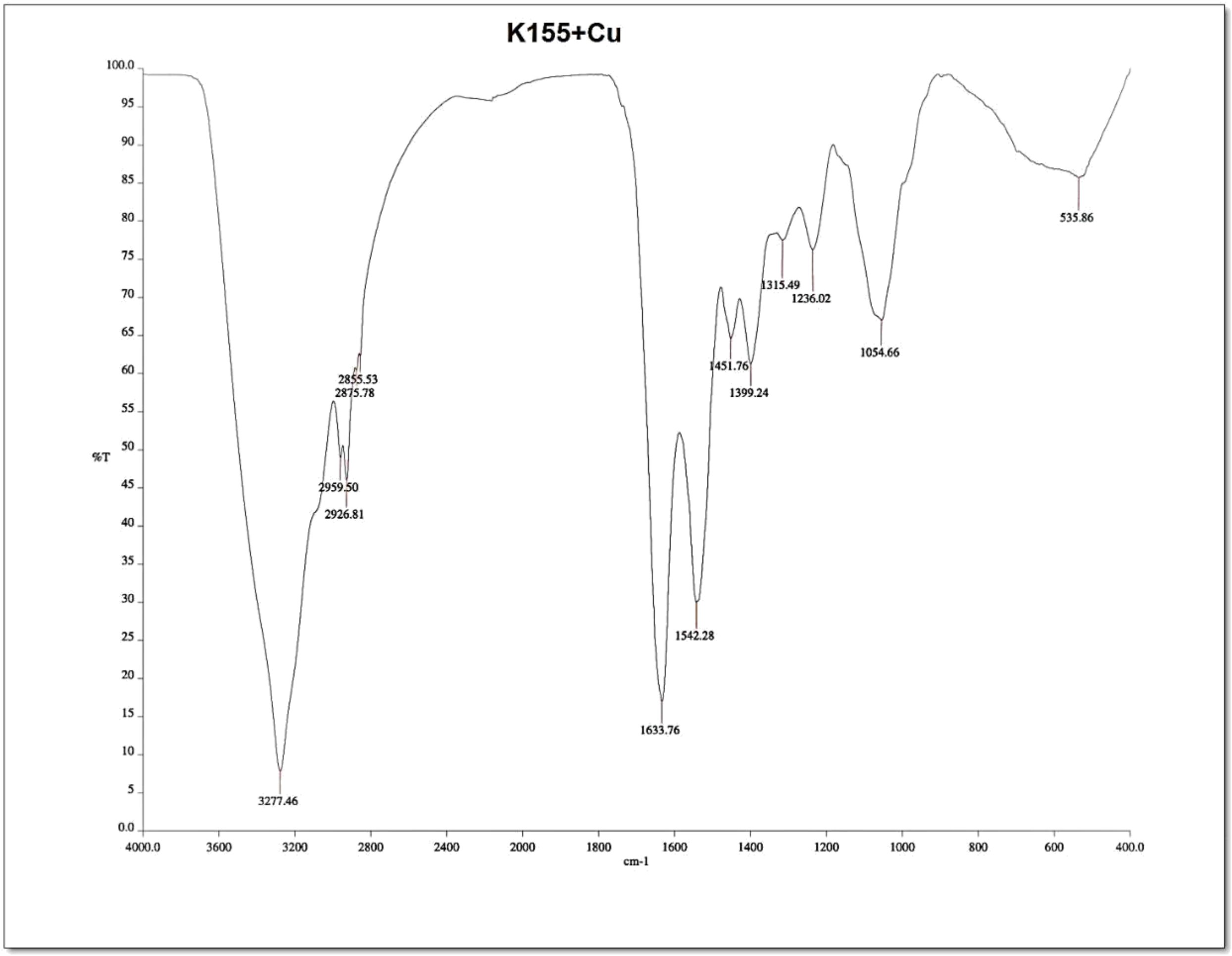

Fig. S19 Fourier-transform infrared spectroscopy (FT-IR) spectra showed that the biomass of strain K155 with and without heavy metals has changed in functional groups' absorption peaks in the 400-4000 cm^{-1} range. Strain K155 treated with copper (100 mg/L). The Y-axis represents the percentage of transmission (%T), which means the amount of infrared light absorbed or transmitted by the treated and untreated samples being analyzed.

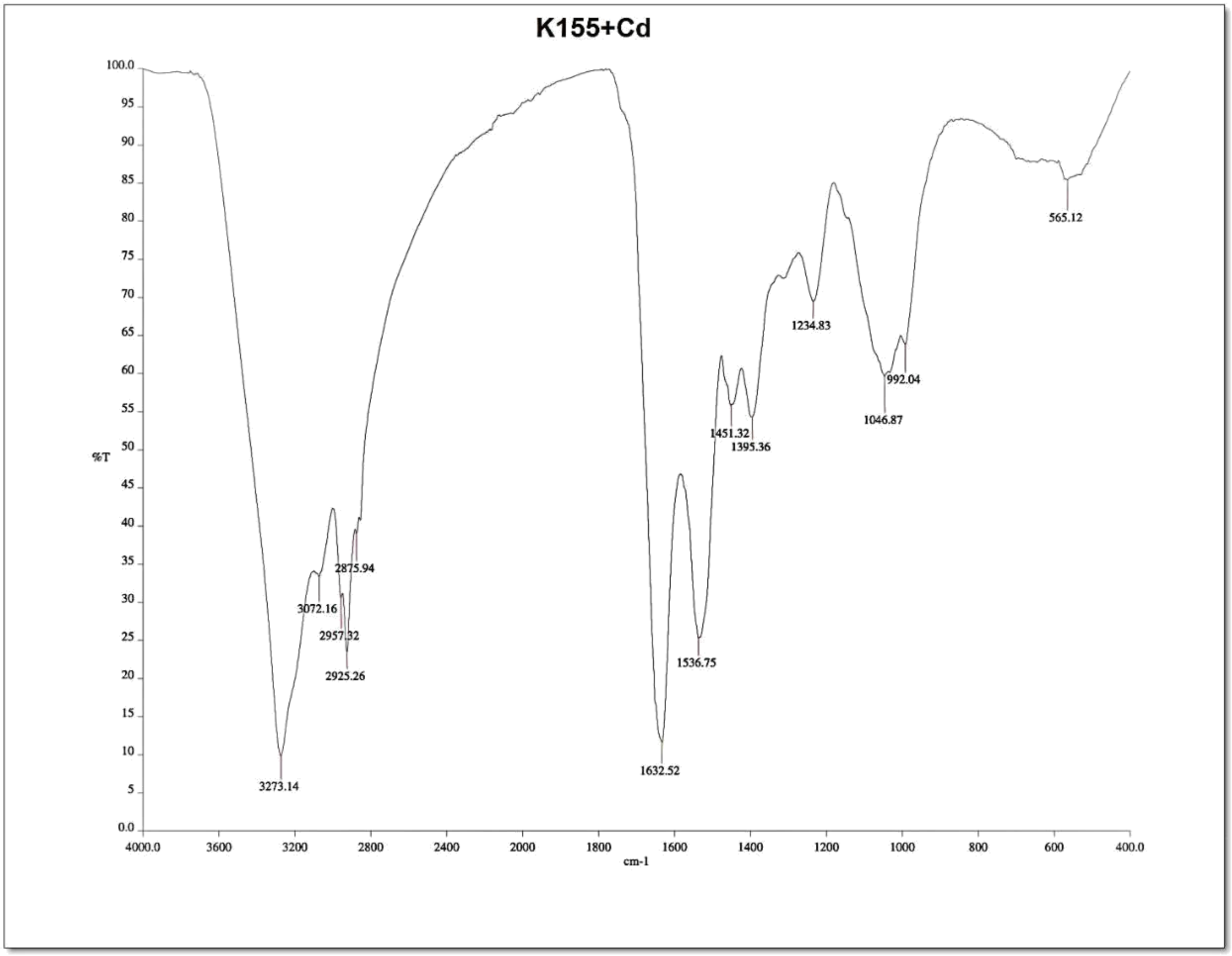

Fig. S20 Fourier-transform infrared spectroscopy (FT-IR) spectra showed that the biomass of strain K155 with and without heavy metals has changed in functional groups' absorption peaks in the 400-4000 cm^{-1} range. Strain K155 treated with cadmium (50 mg/L). The Y-axis represents the percentage of transmission (%T), which means the amount of infrared light absorbed or transmitted by the treated and untreated samples being analyzed.

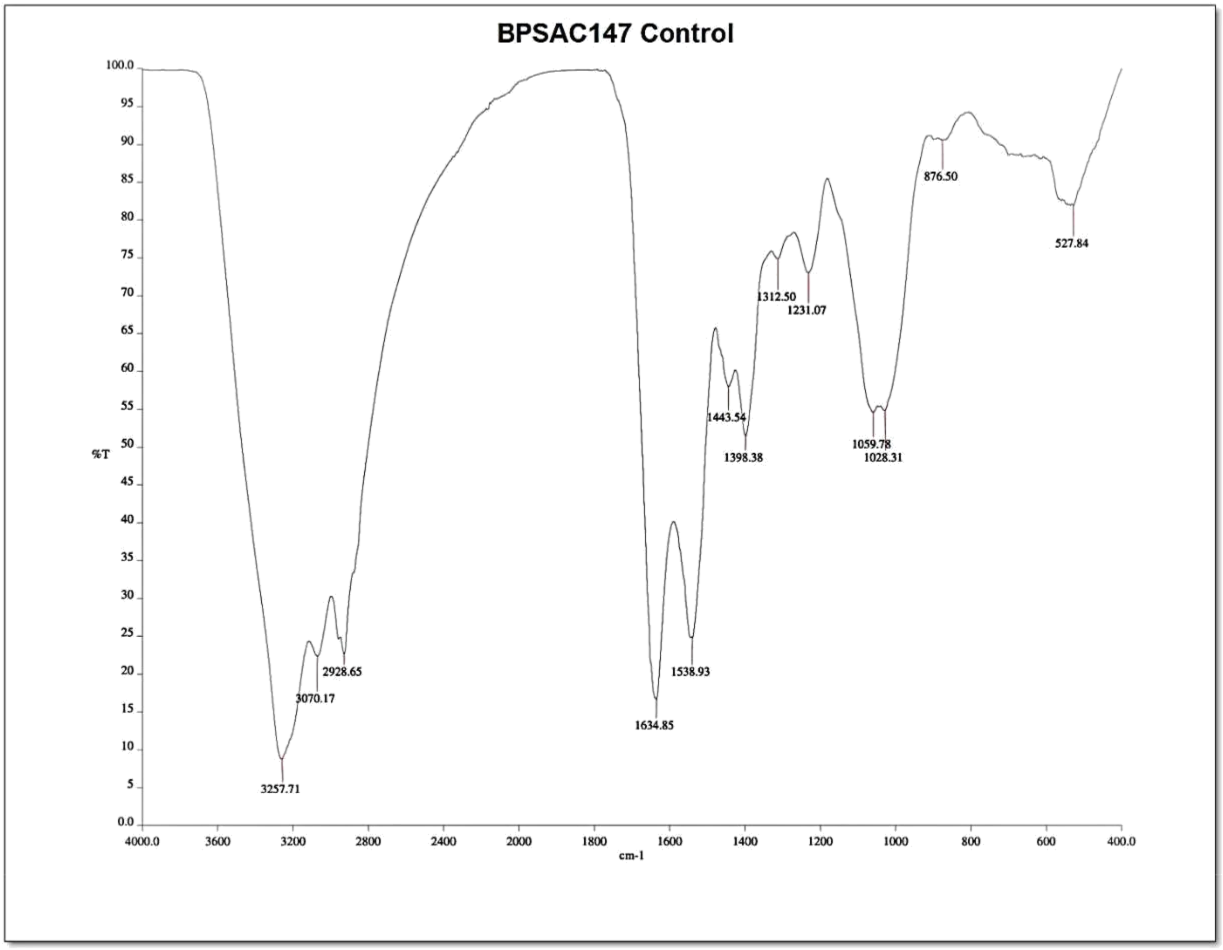

Fig. S21 Fourier-transform infrared spectroscopy (FT-IR) spectra showed that the biomass of strain BPSAC147 with and without heavy metals has changed in functional groups' absorption peaks in the 400-4000 cm^{-1} range. Control BPSAC147 (Absence of metals). The Y-axis represents the percentage of transmission (%T), which means the amount of infrared light absorbed or transmitted by the treated and untreated samples being analyzed.

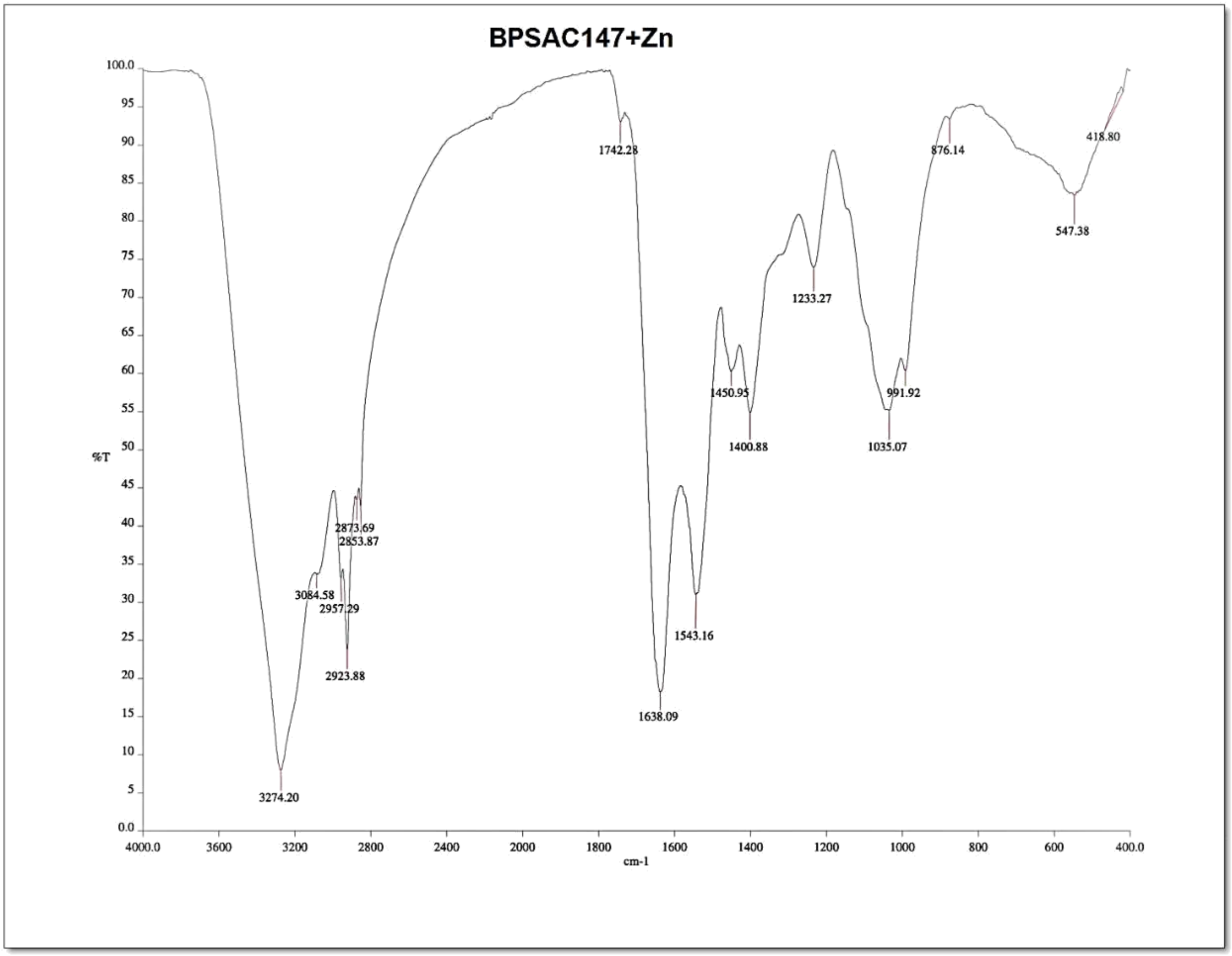

Fig. S22 Fourier-transform infrared spectroscopy (FT-IR) spectra showed that the biomass of strain BPSAC147 with and without heavy metals has changed in functional groups' absorption peaks in the 400-4000 cm^{-1} range. Strain BPSAC147 treated with zinc (1000 mg/L). The Y-axis represents the percentage of transmission (%T), which means the amount of infrared light absorbed or transmitted by the treated and untreated samples being analyzed.

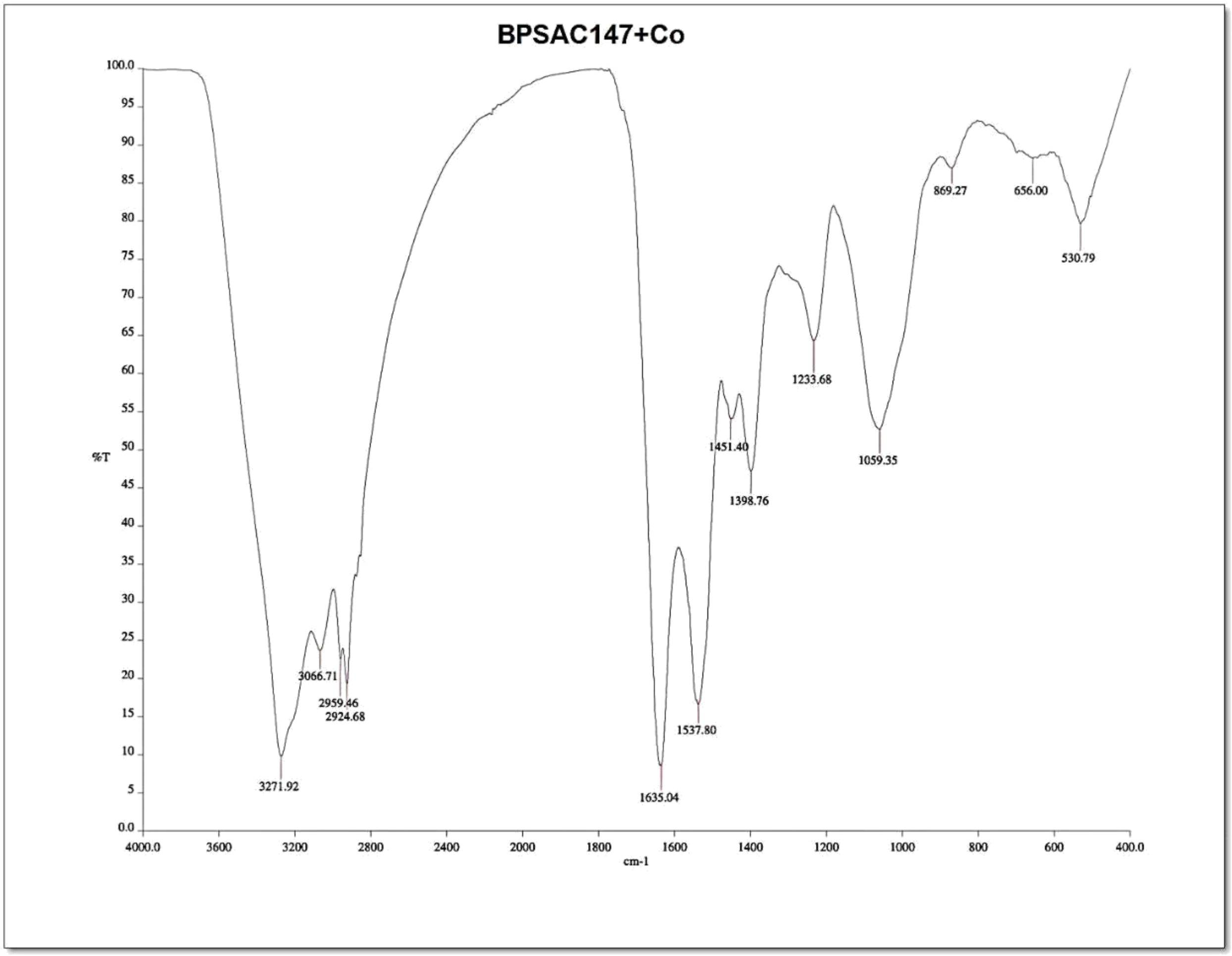

Fig. S23 Fourier-transform infrared spectroscopy (FT-IR) spectra showed that the biomass of strain BPSAC147 with and without heavy metals has changed in functional groups' absorption peaks in the 400-4000 cm^{-1} range. Strain BPSAC147 treated with cobalt (500 mg/L). The Y-axis represents the percentage of transmission (%T), which means the amount of infrared light absorbed or transmitted by the treated and untreated samples being analyzed.

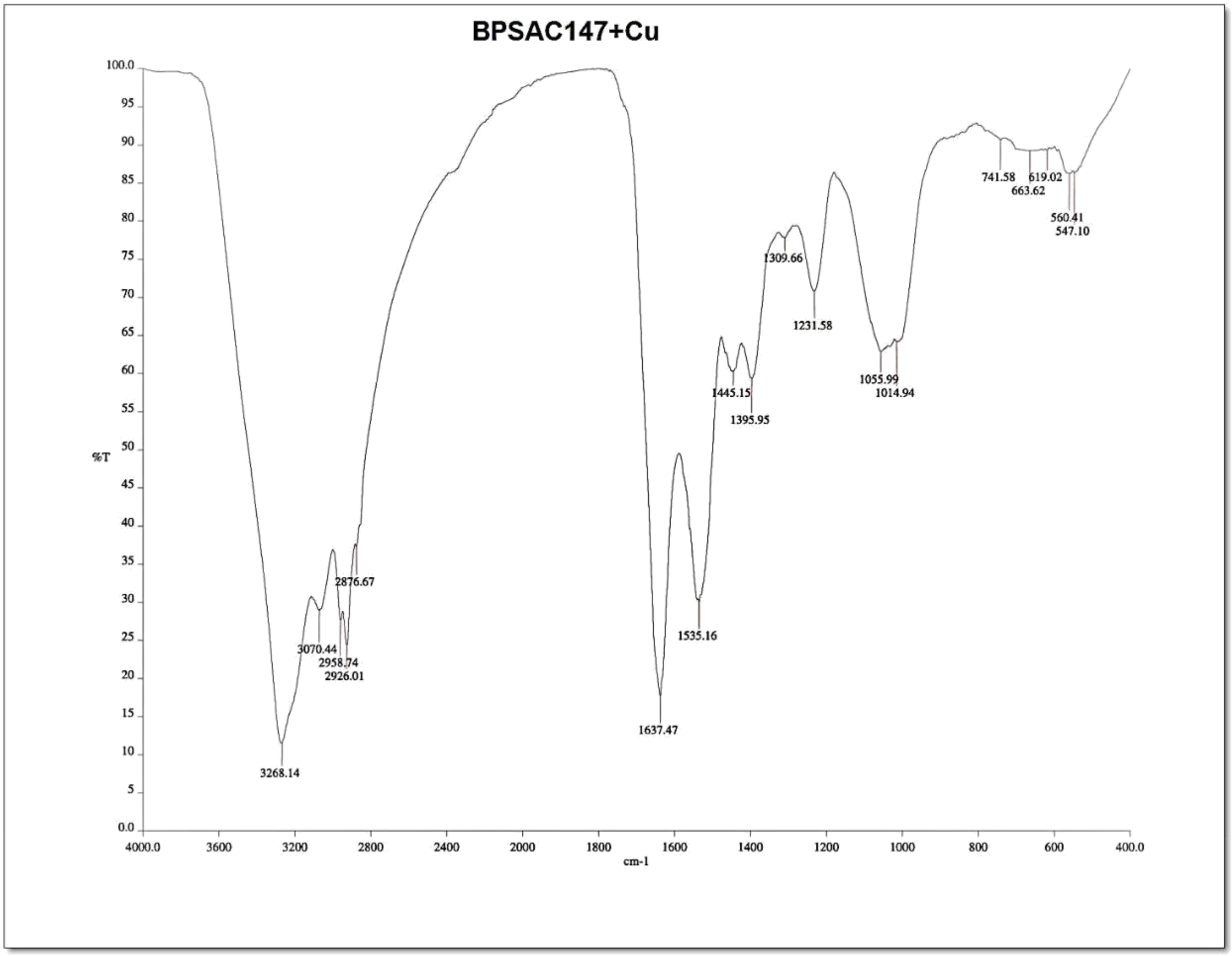

Fig. S24 Fourier-transform infrared spectroscopy (FT-IR) spectra showed that the biomass of strain BPSAC147 with and without heavy metals has changed in functional groups' absorption peaks in the 400-4000 cm^{-1} range. Strain BPSAC147 treated with copper (100 mg/L). The Y-axis represents the percentage of transmission (%T), which means the amount of infrared light absorbed or transmitted by the treated and untreated samples being analyzed.

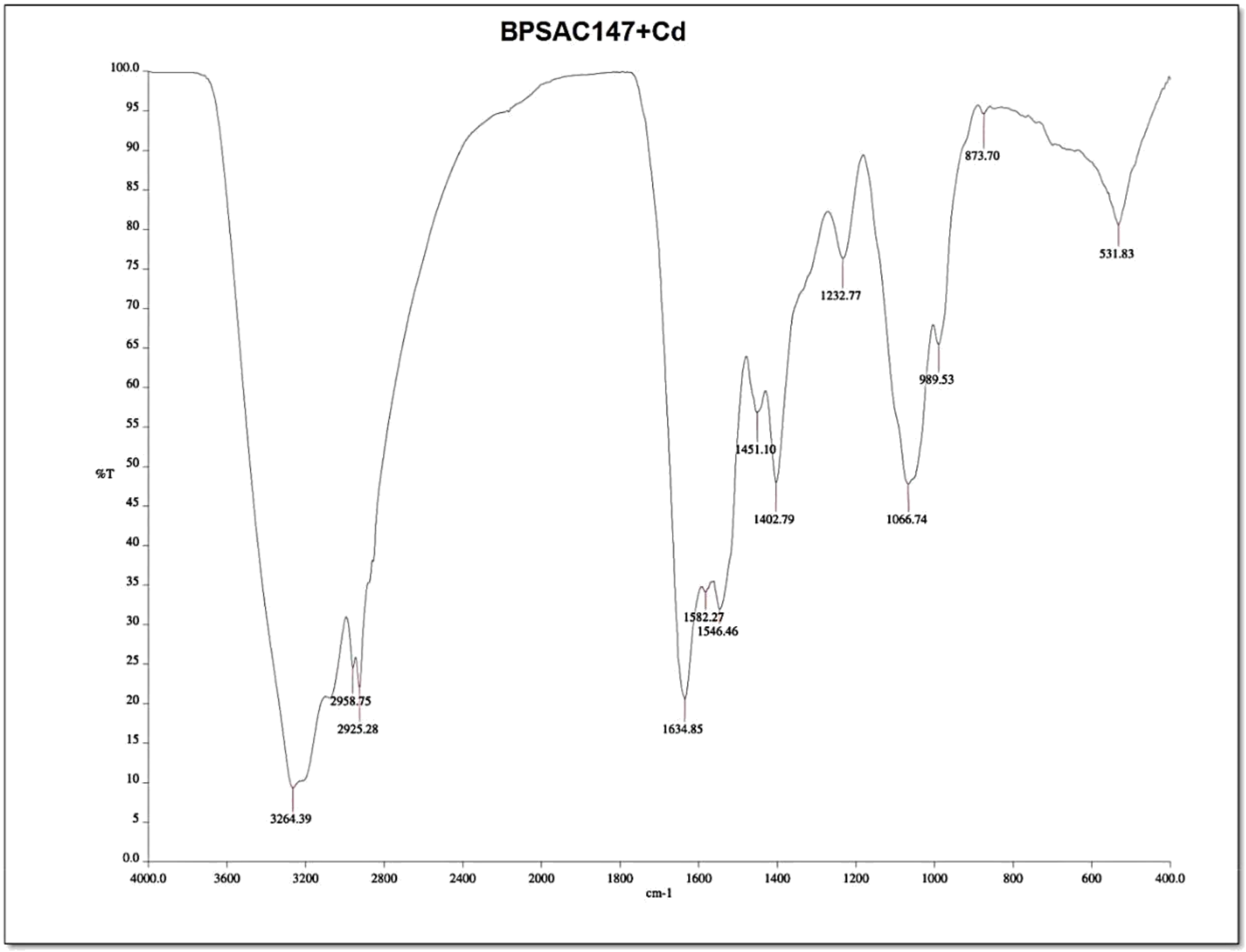

Fig. S25 Fourier-transform infrared spectroscopy (FT-IR) spectra showed that the biomass of strain BPSAC147 with and without heavy metals has changed in functional groups' absorption peaks in the 400-4000 cm^{-1} range. Strain BPSAC147 treated with cadmium (50 mg/L). The Y-axis represents the percentage of transmission (%T), which means the amount of infrared light absorbed or transmitted by the treated and untreated samples being analyzed.
