# Supplementary material for: Comparative genomics of the Streptomyces genus: insights into multi-stress-resistant genes for bioremediation
Source: World J Microbiol Biotechnol. 2025 Dec 2;41(12):492. doi: 10.1007/s11274-025-04703-1 (PMC12672707; doi:10.1007/s11274-025-04703-1)
Supplement: Supplementary file 2 — Supplementary Material 2(PDF 8.05 MB) [file 11274_2025_4703_MOESM2_ESM.pdf]

Supplementary Tables

World Journal of Microbiology and Biotechnology

Comparative Genomics of the *Streptomyces* Genus: Insights into Multi-Stress-Resistant Genes for Bioremediation

Ajit Kumar Passari[1*], Carlos Caicedo-Montoya[2], Monserrat Manzo-Ruiz[1], María Paula Gomez-Roman[1], Beatriz Ruiz-Villafán[1], José Fausto Rivero-Cruz[3], Bhim Pratap Singh[4], Romina Rodríguez-Sanoja[1], and Sergio Sánchez[1]*

[1]Instituto de Investigaciones Biomédicas, Universidad Nacional Autónoma de México, Ciudad de México 04510, México.

[2]Grupo de Bioprocesos, Departamento de Ingeniería Química, Universidad de Antioquia, Calle 70 No. 52-21, Medellín 050010, Colombia.

[3]Centro de Innovación para el Desarrollo Apícola Sustentable de Quintana Roo. Universidad Intercultural Maya de Quintana Roo, QR, 03940, México.

[4]Department of Agriculture & Environmental Sciences, National Institute of Food Technology Entrepreneurship & Management (NIFTEM), Sonepat, Haryana-131028, India

*Corresponding authors: sersan@biomedicas.unam.mx
ajit.passari22@iibiomedicas.unam.mx

Table S1. Genome sequence analysis using the PATRIC and RAST databases was performed to assess the presence and absence of heavy metal-resistant genes in strains K155 and BPSAC147, as well as in reference genomes.

Table S2. COG gene distribution shows the number of coding sequences in all the studied genomes

Table S3. Pangenome matrix with annotations among all the studied genomes

Table S4. Functional characterization of core genes based on KEGG families in all the studied genomes using BPGA

Table S5. Effect of heavy metals on the growth of *Streptomyces thermocarboxydus* K155. Error bars indicate the mean ± standard deviations (SD).

Table S6. Effect of heavy metals on the growth of *Streptomyces thermocarboxydus* BPSAC147. Error bars indicate the mean ± standard deviations (SD).

Table S7. Two-way ANOVA analysis of *Streptomyces thermocarboxydus* strains K155 and BPSAC147 grown under different heavy metal concentrations.

Table S1. Genome sequence analysis using the PATRIC and RAST databases performed to assess the presence and absence of heavy metal-resistant genes in strains K155 and BPSAC147, along with the reference genomes

Gene	PATRIC									
	K155	BPSAC147	FxanaD5	UNC401CLCol	4F	SMS_SU21	JSD-1	NRRLB-24431	BSE7F	ETH9427
Arsenate reductase (EC 1.20.4.1)	6690	2138	4141	4415	6622	1597	6177	4623	6162	3452
	5554	-	6823	6646	-	1596	6821	-	6163	-
	-	-	-	-	-	-	-	-	-	-
Arsenic efflux pump protein	-	-	-	-	-	-	-	-	-	-
	-	-	-	-	-	-	-	-	-	-
Arsenical pump-driving ATPase (EC 3.6.3.16)	-	-	-	-	-	-	-	-	-	-
	-	-	-	-	-	-	-	-	-	-
	-	-	-	-	-	-	-	-	-	-
Arsenical resistance operon repressor	5557	2135	4142	6645	6628	6357	6822	1555	6170	3449
	6691	2137	6822	4416	7174	1591	6178	4617	2546	3451
	5555	4894	-	-	6623	1595	6183	4622	6164	-
	-	-	-	-	-	-	6828	-	-	-
	-	-	-	-	-	-	6846	-	-	-
	-	-	-	-	-	-	6847	-	-	-
	-	-	-	-	-	-	6851	-	-	-
Arsenical-resistance protein ACR3	5556	2136	6821	6644	6624	1594	6179	4621	6165	3450
	-	-	-	-	-	6356	6823	-	2545	-
bacteriophage (phiC31) resistance gene pglY	-	-	-	-	-	2760	-	-	-	-
Beta-lactamase (Cephalosporinase) (EC 3.5.2.6)	-	-	-	-	-	-	-	-	-	-
	-	-	-	-	-	-	-	-	-	-
Beta-lactamase (EC 3.5.2.6)	-	-	-	-	-	-	-	-	-	-
	-	-	-	-	-	-	-	-	-	-
	-	-	-	-	-	-	-	-	-	-

Beta-lactamase class A	3539	4360	5714	2292	3440	6333	3530	4972	2518	3479
Beta-lactamase class A-like and penicillin binding proteins (PBPs) superfamily	3542	4363	5717	2295	3443	6330	3527	4969	2514	3482
Beta-lactamase class C and other penicillin binding proteins	584	2250	2990	909	793	4135	2007	2475	6467	746
	5302	4838	1712	4146	2723	3785	489	5218	6858	2385
	3505	6871	872	5	573	5264	5883	931	6423	6643
	237	524	5429	750	2369	2191	3377	2698	996	4130
	2017	2790	3571	4105	890	3697	676	918	5094	2521
	5853	2800	6693	560	1021	6333	280	4818	878	839
	2368	4519	3023	3035	533	2082	2115	824	2340	210
	2032	5375	1671	2259	2356	3374	307	6244	1261	881
	212	2289	2929	932	2460	6330	584	2510	6887	655
	624	2439	1339	2941	3600	3413	2371	1381	5843	2398
	3689	3931	4568	6188	7098	6463	6637	6208	6558	3638
	393	6911	2169	5597	979	2092	3561	1623	5998	2787
	2127	-	3546	3044	6320	1299	2020	5002	6672	134
	-	-	1242	652	3406	3605	720	4778	885	7423
	-	-	-	-	-	6166	-	-	-	445
	-	-	-	-	-	4590	-	-	-	-
Chloramphenicol resistance, MFS efflux pump => CmlR family	-	-	-	-	7020	5714	6560	1698	5746	7213
	-	-	-	-	-	-	-	-	-	-
Cobalt/zinc/cadmium efflux RND transporter, membrane fusion protein, CzcB family	-	-	-	-	-	-	-	-	-	-
Cobalt-zinc-cadmium resistance protein	2408	6755	2128	1690	2784	6523	-	-	1304	2833
Cobalt-zinc-cadmium resistance protein CzcD	5947	5468	5031	5962	6428	4499	5988	6054	5194	6695
	-	1662	-	-	-	-	-	-	-	-
Copper chaperone	-	-	-	-	-	-	-	-	-	-

Copper chaperone (CopZ)	2444	6668	2077	1657	2822	6562	2448	2843	1337	2871
Copper resistance protein CopC	-	1660	-	-	-	-	-	-	-	-
	-	-	-	-	-	-	-	-	-	-
Copper resistance protein CopC / Copper resistance protein CopD	3730	6162	5822	4674	3726	6123	3336	1422	2233	3678
	3790	1649	6521	4614	3642	6060	3260	1487	2297	3755
	-	-	-	-	-	-	-	-	-	-
Copper resistance protein CopD	-	-	-	-	-	-	-	-	-	-
	-	-	-	-	-	-	-	-	-	-
Copper resistance protein CopD / Cytochrome c oxidase caa3-type assembly factor CtaG_BS (unrelated to Cox11-CtaG family)	-	1659	-	-	-	-	-	-	-	-
Copper-translocating P-type ATPase (EC 3.6.3.4)	758	5631	141	1253	399	4337	156	2351	6289	326
	759	6667	1845	1656	452	5379	207	2401	6978	330
	2445	-	2076	4282	1142	6563	841	2844	1338	1174
	6217	-	4168	4283	2823	530	2449	3703	-	2872
	6720	-	-	4442	6707	-	6240	6081	-	6852
	-	-	-	-	-	-	-	-	-	-
Cytoplasmic copper homeostasis protein CutC	-	-	-	-	-	-	-	-	-	-
Daunorubicin/doxorubicin resistance ABC transporter, permease protein	3601	612	5776	5509	3507	6257	3464	1292	2435	3547
DNA gyrase subunit A (EC 5.99.1.3)	3647	567	6762	5556	3557	6207	3416	1339	2384	3598
	-	-	-	-	-	-	-	-	-	-
DNA gyrase subunit B (EC 5.99.1.3)	3648	566	6763	5557	3558	6206	3415	1340	2383	3599
	-	-	-	-	-	-	-	-	-	-
Ethidium bromide-methyl viologen resistance protein EmrE	1662	273	2711	-	-	1726	-	-	512	2027

Flavin-dependent monooxygenase ArsO associated with arsenic resistance	3478	3903	-	-	6625	-	6825	4620	6167	-
	-	-	-	-	-	-	6180	-	-	-
Glyoxalase/bleomycin resistance protein/dioxygenase	-	-	-	-	2829	-	-	-	-	-
Magnesium and cobalt efflux protein CorC	-	-	-	-	-	-	-	-	-	-
	-	-	-	-	-	-	-	-	-	-
	-	-	-	-	-	-	-	-	-	-
Magnesium and cobalt transport protein CorA	-	-	-	-	-	-	-	-	-	-
	-	-	-	-	-	-	-	-	-	-
	-	-	-	-	-	-	-	-	-	-
Metal-dependent hydrolases of the beta-lactamase superfamily III	-	-	-	-	-	6736	-	-	1523	3049
	-	-	-	-	-	-	-	-	-	2301
MFS-type transporter quinolone resistance protein NorA	-	-	-	-	-	453	-	-	-	-
Multicopper oxidase	5912	5432	5065	5996	6391	4306	5950	6017	5372	6661
	-	-	-	-	-	6756	-	-	5158	3337
	-	-	-	-	-	4534	-	-	-	-
Multidrug resistance transporter, Bcr/CflA family	2033	2801	2930	3034	2370	2093	2021	917	886	2399
Organic hydroperoxide resistance protein	2691	-	-	-	6183	2999	5761	1851	4913	3132
	-	-	-	-	3072	-	-	-	-	6531
Organic hydroperoxide resistance transcriptional regulator	2692	3524	5642	1411	3073	3903	2694	5975	1613	3133
	-	-	-	-	-	-	-	-	-	-
Oxytetracycline resistance protein OtrA	-	-	-	-	-	-	-	-	-	-
	-	-	-	-	-	-	-	-	-	-
Oxytetracycline resistance, ABC-type efflux pump ATP-binding component => Otr(C)	2006	2778	2909	3055	2342	2063	1993	945	857	2373

Oxytetracycline resistance, ABC-type efflux pump permease component => Otr(C)	2007	2779	2910	3054	2343	2064	1994	944	858	2374
Periplasmic divalent cation tolerance protein CutA	-	-	-	-	-	-	-	-	-	-
PF00070 family, FAD-dependent NAD(P)-disulphide oxidoreductase	718	6296	1804	4240	1103	5327	803	5487	6349	6989
	-	-	-	-	-	-	-	-	-	-
PF00070 family, FAD-dependent NAD(P)-disulphide oxidoreductase	6298	6987	60	1172	6795	5958	6331	4420	5496	947
	-	6988	-	-	-	-	-	3610	-	-
Probable Co/Zn/Cd efflux system membrane fusion protein	-	-	-	-	-	-	-	-	-	-
putative tellurium resistance protein	3007	1805	4886	5066	-	606	-	-	-	4039
putative toxic cation resistance protein	-	-	-	-	-	-	4358	281	3441	179
Ribosome protection-type tetracycline resistance related proteins	6384	6385	1873	1083	-	5857	-	-	5603	-
RND multidrug efflux transporter; Acriflavin resistance protein	1919	13	2497	5463	2257	1982	1911	1030	-	-
	-	12	-	5464	-	-	-	-	-	-
small multidrug resistance family (SMR) protein	2600	6005	5168	1811	5030	1018	2600	66	3683	5207
	4595	3591	3392	1503	2982	6724	4572	5883	1511	3037
Tellurite resistance protein TerB	228	2268	3562	918	552	1212	297	2492	5944	7476
	-	-	4976	-	-	3392	78	-	6061	81
	-	-	-	-	-	-	-	-	6869	422
tellurium resistance protein	114	4151	6876	6649	3618	2173	6733	842	978	178
	6860	6499	6935	2958	2442	-	2097	1537	-	2503
	2110	-	3006	-	7193	-	6732	1399	-	2502
	-	-	-	-	-	-	3359	-	-	-

tellurium resistance protein TerA	3707	5612	3008	2956	1121	2174	2098	841	2321	2504
	739	4149	1826	4263	2443	5360	821	6060	6310	3656
	2112	506	5412	5616	-	6147	-	-	6309	1155
	-	-	-	-	-	-	-	-	979	1154
Tellurium resistance protein TerD	2114	1720	3010	5174	1988	1781	1643	838	6872	2506
	2115	2265	3011	4515	2446	1715	294	3980	981	2084
	1718	219	3559	921	3901	2177	3091	1231	500	2507
	1639	4147	5364	2953	2056	2176	2100	4446	568	2014
	3088	298	2687	2954	2445	3389	2101	2489	982	3942
	225	4146	4275	4985	549	4007	1710	839	2038	419
	-	-	-	-	-	-	-	-	-	2015
Topoisomerase IV subunit A (EC 5.99.1.-)	5134	788	1518	3752	5564	5158	5128	6317	4245	5755
Topoisomerase IV subunit B (EC 5.99.1.-)	5125	797	1509	3762	5555	5167	5119	6325	4236	5746
Transcriptional regulator, MerR family	4802	2919	3021	2340	5202	113	2113	4922	4793	7269
	2942	4940	4468	6133	5230	682	5837	1773	5010	2519
	4775	5330	5719	2943	6024	3323	2885	900	1808	333
	2053	4209	4688	2602	4055	1335	4779	4370	1867	6290
	2125	1870	4538	56	6264	2189	2037	3736	2549	6194
	5828	6479	5619	2659	2458	26	3525	2009	915	3445
	6797	4521	4970	3015	4116	4656	2944	826	6024	5428
	3544	4366	4583	6398	3445	6360	5599	5772	3861	7250
	2884	-	406	2297	3348	6328	4750	4967	2513	3484
	4925	-	2949	-	2385	2113	3594	5038	3891	2440
	-	-	3795	-	-	4711	-	-	994	7444
	-	-	4356	-	-	537	-	-	6971	6611
	-	-	-	-	-	3141	-	-	4714	113
	-	-	-	-	-	3171	-	-	5806	5398

	-	-	-	-	-	-	-	-	-	4190
	-	-	-	-	-	-	-	-	-	4116
	-	-	-	-	-	-	-	-	-	-
Translation elongation factor G	4067	1175	5287	3620	4505	2504	4074	554	3112	4650

**Gene**	**RAST**									
	**K155**	**BPSAC147**	**FxanaD5**	**UNC401CLCol**	**4F**	**SMS_SU21**	**JSD-1**	**NRRLB-24431**	**BSE7F**	**ETH9427**
Arsenate reductase (EC 1.20.4.1)	1932	-	-	-	-	-	-	-	-	-
	5485	-	-	-	-	-	-	-	-	-
	6597	-	-	-	-	-	-	-	-	-
Arsenic efflux pump protein	2993	-	-	-	-	-	-	-	-	-
	-	-	-	-	-	-	-	-	-	-
Arsenical pump-driving ATPase (EC 3.6.3.16)	1857	-	-	-	-	-	-	-	-	-
	3301	-	-	-	-	-	-	-	-	-
	3302	-	-	-	-	-	-	-	-	-
Arsenical resistance operon repressor	5486	-	-	-	-	-	-	-	-	-
	-	-	-	-	-	-	-	-	-	-
	-	-	-	-	-	-	-	-	-	-
	-	-	-	-	-	-	-	-	-	-
	-	-	-	-	-	-	-	-	-	-
	-	-	-	-	-	-	-	-	-	-
	-	-	-	-	-	-	-	-	-	-
Arsenical-resistance protein ACR3	5487	-	-	-	-	-	-	-	-	-
	-	-	-	-	-	-	-	-	-	-
bacteriophage (phiC31) resistance gene pglY	-	-	-	-	-	-	-	-	-	-
	597	-	-	-	-	-	-	-	-	-

Beta-lactamase (Cephalosporinase) (EC 3.5.2.6)	-	-	-	-	-	-	-	-	-	-
Beta-lactamase (EC 3.5.2.6)	3450	-	-	-	-	-	-	-	-	-
	3486	-	-	-	-	-	-	-	-	-
	3635	-	-	-	-	-	-	-	-	-
Beta-lactamase class A	3483	-	-	-	-	-	-	-	-	-
Beta-lactamase class A-like and penicillin binding proteins (PBPs) superfamily	-	-	-	-	-	-	-	-	-	-
Beta-lactamase class C and other penicillin binding proteins	556	-	-	-	-	-	-	-	-	-
	2001	-	-	-	-	-	-	-	-	-
	2095	-	-	-	-	-	-	-	-	-
	5232	-	-	-	-	-	-	-	-	-
	5781	-	-	-	-	-	-	-	-	-
	-	-	-	-	-	-	-	-	-	-
	-	-	-	-	-	-	-	-	-	-
	-	-	-	-	-	-	-	-	-	-
	-	-	-	-	-	-	-	-	-	-
	-	-	-	-	-	-	-	-	-	-
	-	-	-	-	-	-	-	-	-	-
	-	-	-	-	-	-	-	-	-	-
	-	-	-	-	-	-	-	-	-	-
	-	-	-	-	-	-	-	-	-	-
	-	-	-	-	-	-	-	-	-	-
	-	-	-	-	-	-	-	-	-	-
Chloramphenicol resistance, MFS efflux pump => CmlR family	-	-	-	-	-	-	-	-	-	-
	-	-	-	-	-	-	-	-	-	-

Cobalt/zinc/cadmium efflux RND transporter, membrane fusion protein, CzcB family	-	-	-	-	-	-	-	-	2066	-
Cobalt-zinc-cadmium resistance protein	2379	2178	2161	1728	2772	3224	-	-	2485	2740
Cobalt-zinc-cadmium resistance protein CzcD	5870	1491	5155	6097	6408	1397	6337	6176	6325	6519
	-	3627	-	-	-	-	-	-	-	-
Copper chaperone	-	-	-	-	-	-	-	-	-	-
Copper chaperone (CopZ)	2414	2091	2108	1694	2810	3260	2602	2892	2517	2776
Copper resistance protein CopC	-	3625	-	-	-	-	-	-	-	-
	-	-	-	-	-	-	-	-	-	-
Copper resistance protein CopC / Copper resistance protein CopD	-	2445	6003	4731	3628	2768	3464	1440	3400	3567
	-	3614	6729	4792	3710	2831	3547	1504	3464	3639
	-	6806	-	-	-	-	-	-	-	-
Copper resistance protein CopD	3676	3624	-	-	-	-	-	-	3399	-
	3736	-	-	-	-	-	-	-	-	-
Copper resistance protein CopD / Cytochrome c oxidase caa3-type assembly factor CtaG_BS (unrelated to Cox11-CtaG family)	-	-	-	-	-	-	-	-	-	-
Copper-translocating P-type ATPase (EC 3.6.3.4)	150	2090	144	1278	395	1233	174	2387	111	317
	731	6507	1871	1693	448	2102	229	2443	790	321
	732	-	2107	4383	1134	3261	894	2893	2518	1143
	2415	-	4242	4384	2811	4238	2604	3755	4260	2777
	6134	-	-	4549	6684	4901	6602	6203	7064	6674
	6623	-	-	-	-	-	-	-	-	-
Cytoplasmic copper homeostasis protein CutC	-	3717	4320	5145	3916	6520	3253	4539	3173	3857

Daunorubicin/doxorubicin resistance ABC transporter, permease protein	-	-	-	-	-	-	-	-	-	-
DNA gyrase subunit A (EC 5.99.1.3)	3693	2536	6989	5682	3543	2916	3633	1356	3552	3488
	-	-	-	-	-	-	-	-	-	-
DNA gyrase subunit B (EC 5.99.1.3)	3594	2535	6990	5683	3544	2915	3632	1357	3551	3489
	-	-	-	-	-	-	-	-	-	-
Ethidium bromide-methyl viologen resistance protein EmrE	-	-	-	-	-	-	-	-	-	-
Flavin-dependent monooxygenase ArsO associated with arsenic resistance	-	-	-	-	-	-	-	-	-	-
	-	-	-	-	-	-	-	-	-	-
Glyoxalase/bleomycin resistance protein/dioxygenase	-	-	-	-	-	-	-	-	-	-
Magnesium and cobalt efflux protein CorC	1108	4221	3735	6388	2606	5548	2409	4175	2334	2595
	2233	-	-	-	-	-	-	-	-	-
	6691	-	-	-	-	-	-	-	-	-
Magnesium and cobalt transport protein CorA	1353	211	2790	1923	1709	187	1495	134	-	1708
	4472	1653	3271	4634	4944	5128	4769	5156	-	2032
	-	-	-	-	-	-	-	-	-	5002
Metal-dependent hydrolases of the beta-lactamase superfamily III	2580	-	-	-	-	3432	-	-	2702	2231
	-	-	-	-	-	-	-	-	-	2946
MFS-type transporter quinolone resistance protein NorA	-	-	-	-	-	-	-	-	-	-
Multicopper oxidase	5836	1455	5190	6132	6371	1203	6301	6140	6290	3229
	-	-	-	-	-	1432	-	-	6501	6483
	-	-	-	-	-	6926	-	-	-	-

Multidrug resistance transporter, Bcr/CflA family	-	4391	2973	3115	2359	489	2154	930	2073	2325
Organic hydroperoxide resistance protein	5651	-	5823	1445	6164	5961	2868	-	6049	3026
	-	-	-	-	-	-	6094	-	-	6355
Organic hydroperoxide resistance transcriptional regulator	2660	5114	5822	1444	3061	6382	2869	-	2791	3027
	-	-	-	-	-	-	-	-	7266	-
Oxytetracycline resistance protein OtrA	6300	-	-	-	-	-	-	-	-	-
	-	-	-	-	-	-	-	-	-	-
Oxytetracycline resistance, ABC-type efflux pump ATP-binding component => Otr(C)	-	-	-	-	-	-	-	-	-	-
Oxytetracycline resistance, ABC-type efflux pump permease component => Otr(C)	-	-	-	-	-	-	-	-	-	-
Periplasmic divalent cation tolerance protein CutA	-	6390	5510	4929	-	5638	-	-	-	6193
PF00070 family, FAD-dependent NAD(P)-disulphide oxidoreductase	691	1830	62	1196	436	2050	215	2430	171	924
	-	-	-	-	-	-	-	3663	-	6806
PF00070 family, FAD-dependent NAD(P)-disulphide oxidoreductase	6215	2364	1828	4340	1095	2675	852	4479	6625	-
	-	2365	1900	-	6771	-	6699	5590	-	-
Probable Co/Zn/Cd efflux system membrane fusion protein	1237	-	-	-	-	-	-	-	-	-
putative tellurium resistance protein	-	-	-	-	-	-	-	-	-	-
putative toxic cation resistance protein	-	-	-	-	-	-	-	-	-	-
Ribosome protection-type tetracycline resistance related proteins	-	1919	-	1106	-	2576	-	-	6731	-

RND multidrug efflux transporter; Acriflavin resistance protein	-	-	-	-	-	-	-	-	-	-
	-	-	-	-	-	-	-	-	-	-
small multidrug resistance family (SMR) protein	-	-	-	-	-	-	-	-	-	-
	-	-	-	-	-	-	-	-	-	-
Tellurite resistance protein TerB	-	-	-	-	-	-	-	-	-	-
	-	-	-	-	-	-	-	-	-	-
	-	-	-	-	-	-	-	-	-	-
tellurium resistance protein	-	-	-	-	-	-	-	-	-	-
	-	-	-	-	-	-	-	-	-	-
	-	-	-	-	-	-	-	-	-	-
	-	-	-	-	-	-	-	-	-	-
tellurium resistance protein TerA	-	-	-	-	-	-	-	-	-	-
	-	-	-	-	-	-	-	-	-	-
	-	-	-	-	-	-	-	-	-	-
	-	-	-	-	-	-	-	-	-	-
Tellurium resistance protein TerD	-	-	-	-	-	-	-	-	-	-
	-	-	-	-	-	-	-	-	-	-
	-	-	-	-	-	-	-	-	-	-
	-	-	-	-	-	-	-	-	-	-
	-	-	-	-	-	-	-	-	-	-
	-	-	-	-	-	-	-	-	-	-
	-	-	-	-	-	-	-	-	-	-
Topoisomerase IV subunit A (EC 5.99.1.-)	5068	2753	1535	3853	5546	1888	5424	6450	5387	5590
Topoisomerase IV subunit B (EC 5.99.1.-)	5059	2762	1526	3863	5537	1897	5415	6458	5378	5581
Transcriptional regulator, MerR family	3113	1353	422	60	2374	509	2169	841	783	111
	5757	3835	2993	2368	2447	584	2250	914	2101	324
	6581	4509	3066	2412	3335	1551	3065	1804	2178	2366

	-	5511	3852	2675	3431	2399	3129	2042	2980	2441
	-	5668	4438	2735	4039	3038	3332	3793	3039	3335
	-	5823	4554	3022	4099	3070	3747	4428	3263	3376
	-	6016	4627	3095	5184	3747	3824	4992	3679	3776
	-	6892	4677	5054	5212	3833	5028	5042	3714	3994
	-	-	4788	6271	6005	4245	5059	5120	5007	4066
	-	-	5083	6545	6244	4386	5928	5884	5037	5249
	-	-	5792	-	-	5015	6175	-	5850	5279
	-	-	5898	-	-	5893	-	-	5929	6023
	-	-	-	-	-	6099	-	-	6910	6118
	-	-	-	-	-	6129	-	-	6933	6434
	-	-	-	-	-	6281	-	-	7146	7059
	-	-	-	-	-	6764	-	-	-	7978
	-	-	-	-	-	-	-	-	-	7249
Translation elongation factor G	4010	3140	5421	3713	-	5468	4316	563	4265	4510

Table S2. COG gene distribution shows the number of coding sequences in all the studied genomes

		7484	7042	7298	7601	7224	7095	6929	6844	6935	6883	7072
**Letter**	**Function**	**4F**	**BPSAC147**	**BSE7F**	**ETH9427**	**FxanaD5**	**HNS054**	**K155**	**NRRLB2**	**SMSSU21**	**UNC401CLCol**	**ZS0098**
J	Translation ribosomal structure and biogenesis	353	322	376	376	377	369	372	344	336	353	358
A	RNA processing and modification	2	1	3	4	2	3	3	1	1	2	2
K	Transcription	856	676	849	839	819	817	793	800	372	776	801
L	Replication recombination and repair	317	250	304	312	265	282	268	247	140	260	262
B	Chromatin structure and dynamics	2	2	2	2	3	2	2	2	1	2	2
D	Cell cycle control cell division chromosome partitioning	87	78	86	79	97	77	83	81	47	79	81
Y	Nuclear structure	0	0	0	0	0	0	0	0	0	0	0
V	Defense mechanisms	209	198	210	209	230	209	213	210	114	216	209
T	Signal transduction mechanisms	455	382	451	459	469	438	448	431	199	442	448
M	Cell wall/membrane/envelope biogenesis	367	305	366	412	391	373	362	366	162	375	366
N	Cell motility	30	24	33	33	30	25	27	23	22	24	27
Z	Cytoskeleton	0	0	0	0	0	0	0	0	0	0	0

W	Extracellular structures	44	41	37	35	42	35	40	31	18	35	31
U	Intracellular trafficking secretion and vesicular transport	66	66	67	76	67	72	70	59	33	68	80
O	Posttranslational modification protein turnover chaperones	311	262	310	312	314	298	305	280	154	298	299
X	Mobilome: prophages transposons	129	67	103	129	128	93	74	50	123	84	93
C	Energy production and conversion	419	325	402	430	405	395	396	376	200	396	436
G	Carbohydrate transport and metabolism	669	578	648	653	696	652	662	657	277	676	654
E	Amino acid transport and metabolism	569	492	540	568	546	541	531	516	255	535	535
F	Nucleotide transport and metabolism	152	134	147	156	156	146	147	148	52	143	142
H	Coenzyme transport and metabolism	336	270	331	323	309	311	305	307	246	294	311
I	Lipid transport and metabolism	319	259	291	299	297	282	289	304	117	301	282
P	Inorganic ion transport and metabolism	310	240	293	312	267	292	276	277	202	272	288

Q	Secondary metabolites biosynthesis transport and catabolism	313	186	326	319	240	281	231	286	155	233	283
R	General function prediction only	621	488	592	615	580	588	544	560	3366	539	576
S	Function unknown	548	1396	531	649	494	514	488	488	343	480	506

Table S3. Pangenome matrix with annotations among all the studied genomes

1	Streptomyces_thermocarboxydus_BPSAC147
2	Streptomyces_sp._4F
3	Streptomyces_sp._BSE7F
4	Streptomyces_sp._ETH9427
5	Streptomyces_sp._FxanaD5
6	Streptomyces_sp._UNC401CLCol
7	Actinospica_acidiphila_NRRL_B_24431
8	Streptomyces_sp._HNS054
9	Streptomyces_sp._SMS_SU21
10	Streptomyces_sp._ZS0098
11	Streptomyces_thermocarboxydus_K155

cluster	GI	gene	Annotation	Streptomyces_thermocarboxydus_BPSAC147	Streptomyces_sp._4F	Streptomyces_sp._BSE7F	Streptomyces_sp._ETH9427	Streptomyces_sp._FxanaD5	Streptomyces_sp._UNC401CLCol	Actinospica_acidiphila_NRRL_B_24431	Streptomyces_sp._HNS054	Streptomyces_sp._SMS_SU21	Streptomyces_sp._ZS0098	Streptomyces_thermocarboxydus_K155	Addition	Core-Accessory-Unique	COG	%Similarity_COG	Score_COG	Evalue_COG	KEGG	%Similarity_KEGG	Score_KEGG	Evalue_KEGG
0	WP_048460437.1	-	lonely Cys domain-containing protein	0	0	0	1	1	1	1	1	1	1	1	8	Accessory	COG2730_G#	31.9	89	8.82E-16	NA	34.1	169	2.34E-39
1	AXI91322.1	-	hypothetical protein	0	0	0	1	0	0	0	0	0	0	0	1	Unique	COG4646_KL#	32.4	410	1.19E-112	NA	25.1	218	1.77E-54
2	WP_109288567.1	-	SDR family NAD(P)-dependent oxidoreductase	0	1	1	0	0	0	0	0	1	0	0	3	Accessory	COG3321_Q#	51.3	476	1.72E-132	K13611	39.8	485	9.9E-135
3	WP_157842213.1	-	amino acid adenylation domain-containing protein	0	1	0	0	0	0	1	0	0	0	0	2	Accessory	COG1020_Q#	34.7	816	3.87E-235	K15654	34.8	1302	0
4	WP_084207100.1	-	non-ribosomal peptide synthetase	0	0	0	0	0	0	1	0	0	0	0	1	Unique	COG1020_Q#	39.5	823	2.89E-237	K15655	32.5	1655	0
5	AXI84712.1	-	hypothetical protein	0	0	1	1	0	0	0	1	1	1	0	5	Accessory	COG1020_Q#	38.7	768	6.96E-221	K15667	32	1009	6.26E-293
6	WP_122216164.1	-	DNRLRE domain-containing protein	0	0	0	0	0	0	0	0	0	1	0	1	Unique	COG3209_M#	23.1	139	2.62E-31	NA	23.5	139	4.11E-31
7	WP_102641535.1	-	DNRLRE domain-containing protein	0	0	1	0	0	0	0	0	1	0	0	2	Accessory	COG3209_M#	24.4	192	2E-47	NA	24.4	192	5.33E-47
8	WP_157842218.1	-	amino acid adenylation domain-containing protein	1	0	0	0	0	0	1	0	0	0	0	2	Accessory	COG1020_Q#	46.2	854	1.21E-246	K04780	43.1	1488	0

cluster	GI	gene	Annotation	Streptomyces_thermocarboxydus_BPSAC147	Streptomyces_sp._4F	Streptomyces_sp._BSE7F	Streptomyces_sp._ETH9427	Streptomyces_sp._FxanaD5	Streptomyces_sp._UNC401CLCol	Actinospica_acidiphila_NRRL_B_24431	Streptomyces_sp._HNS054	Streptomyces_sp._SMS_SU21	Streptomyces_sp._ZS0098	Streptomyces_thermocarboxydus_K155	Addition	Core-Accessory-Unique	COG	%Similarity_COG	Score_COG	Evalue_COG	KEGG	%Similarity_KEGG	Score_KEGG	Evalue_KEGG
9	WP_102641651.1	-	non-ribosomal peptide synthetase	0	0	1	0	0	0	0	0	1	0	0	2	Accessory	COG1020_Q#	38.6	860	1.67E-248	NA	40.4	785	2.38E-225
10	AXI84642.1	-	hypothetical protein	0	0	0	1	0	0	1	1	0	1	0	4	Accessory	COG1020_Q#	44.9	682	6.19E-195	NA	44.9	682	1.65E-194
11	AXI88494.1	-	sugar-binding protein	1	0	0	1	1	0	0	0	0	1	0	4	Accessory	COG3209_M#	28.3	109	2.14E-22				
12	WP_033277978.1	-	non-ribosomal peptide synthetase	0	1	0	1	0	0	1	1	1	1	0	6	Accessory	COG1020_Q#	43.4	1002	3.45E-291	K04780	41.4	1209	0
13	WP_109288721.1	-	sugar-binding protein	1	0	0	1	0	0	0	1	1	1	1	6	Accessory	COG3209_M#	25.8	74	5.25E-12	K03646	34.1	62	5.51E-08
14	WP_048460344.1	-	non-ribosomal peptide synthetase	0	0	0	1	0	0	0	1	1	1	0	4	Accessory	COG1020_Q#	38	874	1.27E-252	K15666	31.3	720	8.22E-206
15	WP_048460602.1	-	non-ribosomal peptide synthetase	0	0	0	1	0	0	0	1	0	0	0	2	Accessory	COG1020_Q#	43.5	779	3.09E-224	K15654	32.2	1005	9.24E-292
16	WP_107080944.1	-	hypothetical protein	0	0	0	0	0	0	0	1	0	0	0	1	Unique	COG3209_M#	27.1	149	1.51E-34				
17	PWE08635.1	-	hypothetical protein	0	0	1	0	0	0	0	0	0	0	0	1	Unique	COG3209_M#	26.1	198	2.14E-49	NA	26.1	218	5.33E-55
18	Prokka:JAPCLKMA_05190	rhlE_5	ATP-dependent RNA helicase RhlE	1	1	1	0	1	1	0	0	0	0	0	5	Accessory	COG1205_R#	31.3	776	2.46E-223	K06877	42.7	57	0.00000266
19	AXI89656.1	-	RNA helicase	0	0	0	1	0	0	0	0	0	0	0	1	Unique	COG1205_R#	32.4	830	1.07E-239	K03724	28.3	72	7.79E-11

cluster	GI	gene	Annotation	Streptomyces_thermocarboxydus_BPSAC147	Streptomyces_sp._4F	Streptomyces_sp._BSE7F	Streptomyces_sp._ETH9427	Streptomyces_sp._FxanaD5	Streptomyces_sp._UNC401CLCol	Actinospica_acidiphila_NRRL_B_24431	Streptomyces_sp._HNS054	Streptomyces_sp._SMS_SU21	Streptomyces_sp._ZS0098	Streptomyces_thermocarboxydus_K155	Addition	Core-Accessory-Unique	COG	%Similarity_COG	Score_COG	Evalue_COG	KEGG	%Similarity_KEGG	Score_KEGG	Evalue_KEGG
20	WP_146217773.1	-	KR domain-containing protein	0	0	1	0	0	0	0	0	1	0	0	2	Accessory	COG3321_Q#	40.4	476	6.66E-133	K13612	37.6	508	2.47E-142
21	WP_109288751.1	-	type I polyketide synthase	0	1	0	1	0	0	0	1	1	1	0	5	Accessory	COG3321_Q#	47.4	1667	0	K12434	47.4	1667	0
22	Prokka:CIODPCDJ_06729	-	hypothetical protein	0	0	0	0	0	0	0	0	0	0	1	1	Unique	COG3209_M#	24	121	5.3E-26	NA	29.6	61	1.33E-07
23	WP_106959411.1	-	RHS repeat-associated core domain-containing protein	0	0	0	0	0	1	0	0	0	0	0	1	Unique	COG3209_M#	26.7	128	2.53E-28				
24	PWE08634.1	-	sugar-binding protein	0	0	1	0	0	0	0	0	0	0	0	1	Unique	COG3209_M#	25.2	142	1.65E-32	NA	25.2	142	4.41E-32
25	WP_033273628.1	-	protein kinase	0	0	0	0	0	0	1	0	0	0	0	1	Unique	COG1205_R#	45.3	869	2.55E-251	K08884	36.5	183	2.25E-44
26	Prokka:JAPCLKMA_01490	-	hypothetical protein	1	0	0	0	0	0	0	0	0	0	0	1	Unique					NA	26.8	256	2.09E-66
27	WP_053077030.1	-	non-ribosomal peptide synthetase	0	0	0	1	0	0	0	1	0	0	0	2	Accessory	COG1020_Q#	41.6	876	2.08E-253	K15667	35	1111	0
28	WP_157842159.1	-	hypothetical protein	0	0	0	0	0	0	1	0	0	0	0	1	Unique								
29	OSC61745.1	-	hypothetical protein	0	1	0	0	0	0	0	0	0	0	0	1	Unique	COG1205_R#	29.6	516	5.06E-145	K06877	33.6	65	8.23E-09

cluster	GI	gene	Annotation	Streptomyces_thermocarboxydus_BPSAC147	Streptomyces_sp._4F	Streptomyces_sp._BSE7F	Streptomyces_sp._ETH9427	Streptomyces_sp._FxanaD5	Streptomyces_sp._UNC401CLCol	Actinospica_acidiphila_NRRL_B_24431	Streptomyces_sp._HNS054	Streptomyces_sp._SMS_SU21	Streptomyces_sp._ZS0098	Streptomyces_thermocarboxydus_K155	Addition	Core-Accessory-Unique	COG	%Similarity_COG	Score_COG	Evalue_COG	KEGG	%Similarity_KEGG	Score_KEGG	Evalue_KEGG
30	WP_052183313.1	-	RHS repeat-associated core domain-containing protein	0	0	0	0	1	1	0	0	0	0	0	2	Accessory	COG3209_M#	28.3	238	1.97E-61	NA	26.1	186	3.1E-45
31	WP_122216015.1	-	SDR family NAD(P)-dependent oxidoreductase	0	1	0	1	0	0	0	1	1	1	0	5	Accessory	COG3321_Q#	50.6	1258	0	K12436	50.6	1258	0
32	OSC61149.1	-	non-ribosomal peptide synthetase	0	1	0	0	0	0	0	0	0	0	0	1	Unique	COG1020_Q#	40.1	1028	2.22E-299	K04788	36.2	530	5.07E-149
33	OSC61544.1	-	hypothetical protein	0	1	0	0	0	0	0	0	0	0	0	1	Unique	COG1002_V#	34.6	407	2.41E-112				
34	PWE09686.1	-	hybrid sensor histidine kinase/response regulator	1	1	1	1	1	1	1	1	1	1	1	11	CORE	COG2770_T#	62.4	988	4.27E-287	K00936	39.4	195	4.96E-48
35	WP_122215774.1	-	DEAD/DEAH box helicase	1	0	0	0	0	0	0	0	0	1	1	3	Accessory	COG1205_R#	33.1	744	1.07E-213	K06877	32.4	62	5.01E-08
36	WP_019527387.1	pulA	pullulanase-type alpha-1,6-glucosidase	1	1	0	1	1	1	1	1	1	1	1	10	Accessory	COG1523_G#	47	753	2.29E-216	K01200	47	753	6.12E-216
37	WP_048455588.1	-	YD repeat-containing protein	0	0	1	1	0	0	0	1	1	1	0	5	Accessory	COG3209_M#	26.5	362	8.43E-99	NA	25.5	176	1.75E-42

cluster	GI	gene	Annotation	Streptomyces_thermocarboxydus_BPSAC147	Streptomyces_sp._4F	Streptomyces_sp._BSE7F	Streptomyces_sp._ETH9427	Streptomyces_sp._FxanaD5	Streptomyces_sp._UNC401CLCol	Actinospica_acidiphila_NRRL_B_24431	Streptomyces_sp._HNS054	Streptomyces_sp._SMS_SU21	Streptomyces_sp._ZS0098	Streptomyces_thermocarboxydus_K155	Addition	Core-Accessory-Unique	COG	%Similarity_COG	Score_COG	Evalue_COG	KEGG	%Similarity_KEGG	Score_KEGG	Evalue_KEGG
38	PWE07256.1	-	restriction endonuclease	1	1	1	0	1	1	0	0	0	0	0	5	Accessory	COG1002_V#	40.3	579	4.33E-164				
39	PWE10568.1	-	hypothetical protein	0	0	1	0	0	0	0	0	1	0	0	2	Accessory	COG1020_Q#	34.9	407	2.94E-112	K04780	39.2	330	1.22E-88
40	Prokka:JAPCLKMA_04917	-	hypothetical protein	1	0	0	0	0	0	0	0	0	0	0	1	Unique								
41	AXI91422.1	-	hypothetical protein	0	0	0	1	0	0	0	0	0	0	0	1	Unique					NA	30	62	3.62E-08
42	WP_078504623.1	-	PAS domain S-box protein	1	1	0	1	1	1	1	1	1	1	1	10	Accessory	COG0642_T#	29.2	105	1.8E-21	NA	29.1	207	8.99E-52
43	Prokka:CIODPCDJ_03589	-	hypothetical protein	0	0	0	0	0	0	0	0	0	0	1	1	Unique	COG1674_D#	35.9	201	1.82E-50	NA	39.1	193	1.73E-47
44	WP_146217802.1	-	amino acid adenylation domain-containing protein	0	0	0	0	0	0	0	0	1	0	0	1	Unique	COG1020_Q#	39.6	612	3.42E-174	K15655	32.6	759	6.05E-218
45	WP_019523536.1	-	AAA domain-containing protein	1	1	1	1	1	1	1	1	1	1	1	11	CORE								
46	WP_122212709.1	-	NAD-glutamate dehydrogenase	1	1	0	1	1	1	1	1	1	1	1	10	Accessory	COG2902_E#	46.3	1369	0	K15371	45	1280	0
47	WP_122216817.1	-	DUF3320 domain-containing protein	0	0	0	0	0	0	0	0	0	1	0	1	Unique	COG1112_L#	40.8	439	5.1E-122	NA	40.7	261	6.64E-68
48	AXI85375.1	-	helicase	0	0	0	1	0	0	0	0	0	0	0	1	Unique	COG1205_R#	27.7	212	1.32E-53	K03724	33.5	89	4.51E-16

cluster	GI	gene	Annotation	Streptomyces_thermocarboxydus_BPSAC147	Streptomyces_sp._4F	Streptomyces_sp._BSE7F	Streptomyces_sp._ETH9427	Streptomyces_sp._FxanaD5	Streptomyces_sp._UNC401CLCol	Actinospica_acidiphila_NRRL_B_24431	Streptomyces_sp._HNS054	Streptomyces_sp._SMS_SU21	Streptomyces_sp._ZS0098	Streptomyces_thermocarboxydus_K155	Addition	Core-Accessory-Unique	COG	%Similarity_COG	Score_COG	Evalue_COG	KEGG	%Similarity_KEGG	Score_KEGG	Evalue_KEGG
49	Prokka:JAPCLKMA_03907	srmB_3	ATP-dependent RNA helicase SrmB	1	1	1	1	1	1	1	1	1	1	1	11	CORE	COG1201_R#	55.6	1589	0	K03724	55.7	1587	0
50	WP_122213241.1	-	hypothetical protein	0	0	0	0	0	0	0	0	0	1	0	1	Unique								
51	WP_033272568.1	-	cell division protein FtsK	1	1	1	1	1	1	1	1	1	1	1	11	CORE	COG1674_D#	28.6	315	1.38E-84	K03466	28.6	315	3.69E-84
52	WP_048458859.1	pglW	BREX system serine/threonine kinase PglW	0	0	0	0	0	0	0	1	0	0	0	1	Unique	COG0515_RTKL#	23.5	62	2.09E-08	NA	29.2	92	5.04E-17
53	WP_028960982.1	-	hypothetical protein	1	1	1	1	1	1	1	1	1	1	1	11	CORE	COG0419_L#	25.6	89	1.21E-16	NA	30.2	78	7.46E-13
54	WP_102640596.1	pglW	BREX system serine/threonine kinase PglW	0	0	0	0	0	0	0	0	1	0	0	1	Unique	COG0515_RTKL#	23	82	1.48E-14	NA	28.6	73	1.84E-11
55	Prokka:CIODPCDJ_03972	-	hypothetical protein	0	0	1	0	1	0	0	0	1	0	1	4	Accessory	COG3209_M#	31.8	500	1.74E-140	NA	25.7	179	3.1E-43
56	OSC72839.1	-	hypothetical protein	0	1	0	0	0	0	0	0	0	0	0	1	Unique	COG3209_M#	31.4	472	5.07E-132	NA	27.4	245	2.69E-63
57	AXI86228.1	-	glutamate synthase large subunit	1	1	1	1	1	1	1	1	1	1	1	11	CORE	COG0069_E#	68.3	1117	0	K00265	64.2	1966	0
58	AXI88245.1	-	hypothetical protein	1	0	1	1	0	0	0	1	1	1	1	7	Accessory	COG3209_M#	31.8	494	1.24E-138	NA	25.3	171	8.41E-41
59	Prokka:JAPCLKMA_04665	-	hypothetical protein	1	0	0	0	0	0	0	0	0	0	1	2	Accessory	COG3209_M#	31.1	439	3.58E-122	NA	27.7	219	2.65E-55

cluster	GI	gene	Annotation	Streptomyces_thermocarboxydus_BPSAC147	Streptomyces_sp._4F	Streptomyces_sp._BSE7F	Streptomyces_sp._ETH9427	Streptomyces_sp._FxanaD5	Streptomyces_sp._UNC401CLCol	Actinospica_acidiphila_NRRL_B_24431	Streptomyces_sp._HNS054	Streptomyces_sp._SMS_SU21	Streptomyces_sp._ZS0098	Streptomyces_thermocarboxydus_K155	Addition	Core-Accessory-Unique	COG	%Similarity_COG	Score_COG	Evalue_COG	KEGG	%Similarity_KEGG	Score_KEGG	Evalue_KEGG
60	Prokka:CIODPCDJ_03158	-	hypothetical protein	0	0	0	0	0	0	0	0	0	0	1	1	Unique								
61	WP_122213583.1	-	DEAD/DEAH box helicase	0	0	0	0	0	0	0	0	0	1	0	1	Unique	COG1205_R#	33.1	57	6.46E-07	K10730	32.3	49	0.000359
62	WP_078895039.1	-	hypothetical protein	0	0	1	0	0	0	0	1	0	0	0	2	Accessory								
63	Prokka:JAPCLKMA_02837	pknD_7	Serine/threonine-protein kinase PknD	1	0	1	0	1	1	0	0	1	0	0	5	Accessory					NA	25.3	62	5.34E-08
64	WP_048460704.1	-	non-ribosomal peptide synthase	0	0	1	1	0	0	0	1	1	1	0	5	Accessory	COG1020_Q#	39.1	641	7.98E-183	NA	32.7	651	2.69E-185
65	WP_020700051.1	-	type I polyketide synthase	0	0	0	0	1	1	0	0	0	0	1	3	Accessory	COG3321_Q#	47.3	500	2.19E-140	K12440	47.3	500	7.65E-140
66	WP_019526980.1	-	hypothetical protein	0	0	0	0	1	0	0	0	0	0	0	1	Unique								
67	WP_107080891.1	-	DUF3367 domain-containing protein	0	0	1	1	0	0	1	1	1	1	0	6	Accessory					K16648	32.8	569	1.34E-160
68	Prokka:JAPCLKMA_05588	dhbF_2	Dimodular nonribosomal peptide synthase	1	0	0	0	0	0	0	0	0	0	0	1	Unique	COG1020_Q#	40.5	556	3.34E-157	K15655	32.2	615	1.24E-174
69	Prokka:JAPCLKMA_02602	-	hypothetical protein	1	0	0	0	0	0	0	0	0	0	0	1	Unique	COG1887_M#	30.4	212	9.03E-54	NA	30.4	212	2.41E-53
70	WP_159020952.1	-	hypothetical protein	0	0	0	0	0	0	0	1	0	0	0	1	Unique								
71	WP_107685365.1	-	ribonuclease E/G	1	0	0	1	1	1	1	1	0	0	1	7	Accessory	COG1530_J#	49.7	740	1.6E-212	K08300	49.7	740	4.28E-212

cluster	GI	gene	Annotation	Streptomyces_thermocarboxydus_BPSAC147	Streptomyces_sp._4F	Streptomyces_sp._BSE7F	Streptomyces_sp._ETH9427	Streptomyces_sp._FxanaD5	Streptomyces_sp._UNC401CLCol	Actinospica_acidiphila_NRRL_B_24431	Streptomyces_sp._HNS054	Streptomyces_sp._SMS_SU21	Streptomyces_sp._ZS0098	Streptomyces_thermocarboxydus_K155	Addition	Core-Accessory-Unique	COG	%Similarity_COG	Score_COG	Evalue_COG	KEGG	%Similarity_KEGG	Score_KEGG	Evalue_KEGG
72	WP_033274542.1	-	HAD-IC family P-type ATPase	0	0	1	1	1	1	1	1	1	1	1	9	Accessory	COG0474_P#	49.4	868	2.99E-251	K12955	49.2	834	2.18E-240
73	OSC70721.1	-	Secreted glycosyl hydrolase	0	1	0	0	0	0	0	0	0	0	0	1	Unique	COG1572_S#	28.6	89	1.13E-16				
74	Prokka:JAPCLKMA_05632	smc_10	Chromosome partition protein Smc	1	0	0	0	0	0	0	0	0	0	0	1	Unique	COG0739_M#	28.6	64	4.99E-09				
75	WP_102637501.1	-	SpoIIE family protein phosphatase	1	0	1	1	1	1	1	1	1	1	1	10	Accessory	COG0642_T#	44.9	209	9.52E-53	NA	34.2	243	9.32E-63
76	AXI89174.1	-	hypothetical protein	0	0	0	1	0	0	0	0	0	0	0	1	Unique	COG5635_T#	25	140	5.43E-32	K14753	28	78	5.18E-13
77	WP_052183308.1	-	hypothetical protein	0	0	0	0	1	1	1	0	0	0	0	3	Accessory	COG3209_M#	29.5	455	9.87E-127	NA	26.4	200	8.96E-50
78	WP_033273630.1	-	N-6 DNA methylase	0	0	0	0	0	0	1	0	0	0	0	1	Unique	COG1002_V#	38.8	361	1.94E-98	NA	25.3	69	2.38E-10
79	WP_048456186.1	-	TIGR02680 family protein	0	0	0	1	0	0	0	1	0	0	0	2	Accessory					K10388	25.8	78	6.68E-13
80	WP_078611371.1	-	LamG domain-containing protein	0	0	0	0	0	1	0	0	0	0	0	1	Unique								
81	OSC62245.1	-	hypothetical protein	0	1	0	0	0	0	0	0	0	0	0	1	Unique					K01190	27	129	3.26E-28
82	WP_052183336.1	eccCa	type VII secretion protein EccCa	0	0	1	1	1	1	1	1	1	1	1	9	Accessory	COG1674_D#	46.2	760	8.25E-219	K03466	46.2	760	2.2E-218
83	OSC65157.1	-	hypothetical protein	0	1	0	0	0	0	0	0	0	0	0	1	Unique	COG5635_T#	31.1	201	1.48E-50				

cluster	GI	gene	Annotation	Streptomyces_thermocarboxydus_BPSAC147	Streptomyces_sp._4F	Streptomyces_sp._BSE7F	Streptomyces_sp._ETH9427	Streptomyces_sp._FxanaD5	Streptomyces_sp._UNC401CLCol	Actinospica_acidiphila_NRRL_B_24431	Streptomyces_sp._HNS054	Streptomyces_sp._SMS_SU21	Streptomyces_sp._ZS0098	Streptomyces_thermocarboxydus_K155	Addition	Core-Accessory-Unique	COG	%Similarity_COG	Score_COG	Evalue_COG	KEGG	%Similarity_KEGG	Score_KEGG	Evalue_KEGG
84	WP_146217787.1	-	hypothetical protein	0	0	1	0	0	0	0	0	1	0	0	2	Accessory	COG1002_V#	36.4	142	1.06E-32	NA	31	110	1.19E-22
85	WP_028961289.1	-	hypothetical protein	0	0	0	0	0	1	0	0	0	0	0	1	Unique	COG5635_T#	29	207	3.5E-52				
86	WP_051101697.1	-	protein kinase	0	1	0	0	1	1	0	0	0	0	0	3	Accessory	COG0515_RTKL#	25.8	59	1.53E-07	K08836	27	53	0.0000224
87	WP_122217373.1	eccCa	type VII secretion protein EccCa	0	0	0	0	0	0	0	0	0	1	0	1	Unique	COG1674_D#	41	650	1.55E-185	K03466	40.9	646	4.57E-184
88	AXI84766.1	-	hypothetical protein	0	0	1	1	1	1	1	0	1	0	1	7	Accessory	COG1530_J#	26.3	57	4.42E-07	NA	26.7	122	2.99E-26
89	WP_019526557.1	hrpA	ATP-dependent RNA helicase HrpA	1	1	1	1	1	1	1	1	1	1	1	11	CORE	COG1643_L#	62.7	1652	0	K03578	62.6	1657	0
90	OSC70368.1	-	hypothetical protein	1	1	1	1	1	1	1	1	1	1	1	11	CORE	COG1196_D#	26.3	149	1.11E-34	K10388	28.4	297	6.74E-79
91	PWE09674.1	-	type VII secretion protein EccC	1	0	1	0	1	1	1	0	1	0	1	7	Accessory	COG1674_D#	42.8	991	3.66E-288	K03466	42.8	991	9.77E-288
92	OSC70440.1	-	beta-ketoacyl synthase	0	1	0	0	0	0	0	0	0	0	0	1	Unique	COG3321_Q#	69.2	454	1.23E-126	K12436	58.4	790	2.53E-227
93	Prokka:JAPCLKMA_02828	-	hypothetical protein	1	0	0	0	0	0	0	0	0	0	0	1	Unique								
94	WP_033273623.1	-	RHS repeat protein	0	0	0	0	0	0	1	0	0	0	0	1	Unique	COG3209_M#	30.6	455	7.16E-127	NA	26.4	212	2.82E-53
95	Prokka:CIODPCDJ_05539	lacZ_4	Beta-galactosidase	1	1	0	0	1	1	1	1	1	1	1	9	Accessory	COG3250_G#	40.4	443	2.81E-123	K01190	40.3	458	2.94E-127

cluster	GI	gene	Annotation	Streptomyces_thermocarboxydus_BPSAC147	Streptomyces_sp._4F	Streptomyces_sp._BSE7F	Streptomyces_sp._ETH9427	Streptomyces_sp._FxanaD5	Streptomyces_sp._UNC401CLCol	Actinospica_acidiphila_NRRL_B_24431	Streptomyces_sp._HNS054	Streptomyces_sp._SMS_SU21	Streptomyces_sp._ZS0098	Streptomyces_thermocarboxydus_K155	Addition	Core-Accessory-Unique	COG	%Similarity_COG	Score_COG	Evalue_COG	KEGG	%Similarity_KEGG	Score_KEGG	Evalue_KEGG
96	Prokka:JAPCLKMA_01150	rpoC	DNA-directed RNA polymerase subunit beta'	1	1	1	1	1	1	1	1	1	1	1	11	CORE	COG0086_K#	71.5	1873	0	K03046	71.5	1873	0
97	WP_019527225.1	-	tetratricopeptide repeat protein	1	0	0	0	1	1	0	0	1	0	1	5	Accessory	COG0457_R#	29.6	156	6.84E-37	NA	27.6	76	1.84E-12
98	OSC62559.1	-	hypothetical protein	0	1	0	0	0	0	0	0	0	0	0	1	Unique	COG0790_R#	36.8	86	1.14E-15				
99	WP_102639909.1	-	phage resistance protein	0	0	0	0	0	0	0	0	1	0	0	1	Unique								
100	WP_048458866.1	-	bacteriophage resistance protein	0	0	0	0	0	0	0	1	0	0	0	1	Unique								
101	WP_102638716.1	-	bifunctional glycosyltransferase family 2 protein/CDP-glycerol:glycerophosphate glycerophosphotransferase	1	0	1	0	0	0	0	0	1	0	0	3	Accessory	COG1887_M#	43.3	307	3.09E-82	K09809	41.6	309	1.27E-82
102	Prokka:JAPCLKMA_00016	-	hypothetical protein	1	0	0	0	0	0	0	0	0	0	0	1	Unique	COG2319_R#	27.3	221	1.31E-56	NA	27.4	141	4.59E-32

cluster	GI	gene	Annotation	Streptomyces_thermocarboxydus_BPSAC147	Streptomyces_sp._4F	Streptomyces_sp._BSE7F	Streptomyces_sp._ETH9427	Streptomyces_sp._FxanaD5	Streptomyces_sp._UNC401CLCol	Actinospica_acidiphila_NRRL_B_24431	Streptomyces_sp._HNS054	Streptomyces_sp._SMS_SU21	Streptomyces_sp._ZS0098	Streptomyces_thermocarboxydus_K155	Addition	Core-Accessory-Unique	COG	%Similarity_COG	Score_COG	Evalue_COG	KEGG	%Similarity_KEGG	Score_KEGG	Evalue_KEGG
103	PWE10968.1	-	non-ribosomal peptide synthetase	0	0	1	1	0	0	0	1	1	1	0	5	Accessory	COG1020_Q#	41.8	761	4.58E-219	K04780	40.5	929	2.59E-269
104	WP_122212593.1	-	hypothetical protein	1	0	0	1	1	1	0	1	0	1	1	7	Accessory					NA	33.2	74	6.87E-12
105	OSC72010.1	-	hypothetical protein	0	1	0	0	0	0	0	0	0	0	0	1	Unique	COG1020_Q#	33.9	581	6.47E-165	K04780	37	776	4.78E-223
106	AXI85374.1	-	restriction endonuclease	0	0	0	1	0	0	0	0	0	0	0	1	Unique	COG1002_V#	31.2	238	1.03E-61				
107	AXI90555.1	-	beta-ketoacyl synthase	0	1	1	1	0	0	1	1	1	1	0	7	Accessory	COG3321_Q#	46.5	724	8.02E-208	K12444	41.7	685	8.44E-196
108	WP_102638318.1	-	hypothetical protein	0	0	0	0	0	0	0	0	1	0	0	1	Unique	COG1196_D#	27.4	114	3.79E-24	NA	32.7	161	7.23E-38
109	Prokka:JAPCLKMA_02831	-	hypothetical protein	1	0	0	0	0	0	0	0	0	0	0	1	Unique								
110	OSC71509.1	-	alpha-mannosidase	1	1	0	1	1	1	1	1	1	1	1	10	Accessory	COG3537_G#	32.6	322	7E-87	NA	35.9	466	1.05E-129
111	WP_078894971.1	-	ATP-dependent helicase	1	1	1	1	1	1	1	1	1	1	1	11	CORE	COG0210_L#	38.8	246	4.87E-64	NA	35	297	8.39E-79

cluster	GI	gene	Annotation	Streptomyces_thermocarboxydus_BPSAC147	Streptomyces_sp._4F	Streptomyces_sp._BSE7F	Streptomyces_sp._ETH9427	Streptomyces_sp._FxanaD5	Streptomyces_sp._UNC401CLCol	Actinospica_acidiphila_NRRL_B_24431	Streptomyces_sp._HNS054	Streptomyces_sp._SMS_SU21	Streptomyces_sp._ZS0098	Streptomyces_thermocarboxydus_K155	Addition	Core-Accessory-Unique	COG	%Similarity_COG	Score_COG	Evalue_COG	KEGG	%Similarity_KEGG	Score_KEGG	Evalue_KEGG
112	WP_019522932.1	-	multifunctional oxoglutarate decarboxylase/oxoglutarate dehydrogenase thiamine pyrophosphate-binding subunit/dihydrolipoyllysine-residue succinyltransferase subunit	1	1	1	1	1	1	1	1	1	1	1	11	CORE	COG0567_C#	58.6	1052	9.77E-307	K01616	58	1433	0
113	WP_109288655.1	-	AAA family ATPase	1	1	1	1	1	1	1	1	1	1	1	11	CORE	COG0455_D#	33	287	3.25E-76	NA	33	287	8.68E-76
114	Prokka:JAPCLKMA_05193	-	hypothetical protein	1	0	0	0	1	0	0	0	0	0	0	2	Accessory								
115	PWE10313.1	-	hypothetical protein	0	0	1	0	1	1	0	0	0	0	0	3	Accessory	COG0568_K#	37.4	205	9.43E-52	K03086	37.4	205	2.52E-51
116	WP_019525694.1	-	S8 family serine peptidase	1	0	0	1	1	1	1	1	1	1	1	9	Accessory	COG1404_O#	41	200	3.03E-50	K17734	41	200	8.08E-50
117	OSC65723.1	-	hypothetical protein	0	1	0	0	0	0	0	0	0	0	0	1	Unique	COG0515_RTKL#	33	136	7E-31	K08884	31.9	135	4.17E-30
118	WP_086020224.1	-	glycosyltransferase family 2 protein	1	1	1	0	1	1	1	1	1	1	1	10	Accessory	COG1216_R#	26.4	65	1.52E-09	NA	26.4	65	4.05E-09

cluster	GI	gene	Annotation	Streptomyces_thermocarboxydus_BPSAC147	Streptomyces_sp._4F	Streptomyces_sp._BSE7F	Streptomyces_sp._ETH9427	Streptomyces_sp._FxanaD5	Streptomyces_sp._UNC401CLCol	Actinospica_acidiphila_NRRL_B_24431	Streptomyces_sp._HNS054	Streptomyces_sp._SMS_SU21	Streptomyces_sp._ZS0098	Streptomyces_thermocarboxydus_K155	Addition	Core-Accessory-Unique	COG	%Similarity_COG	Score_COG	Evalue_COG	KEGG	%Similarity_KEGG	Score_KEGG	Evalue_KEGG
119	WP_109288640.1	cobT	nicotinate-nucleotide--dimethylbenzimidazole phosphoribosyltransferase	1	1	1	1	1	1	1	1	1	1	1	11	CORE	COG2038_H#	45.7	302	7.36E-81	K00768	48	294	6.99E-78
120	OSC66189.1	-	ABC transporter ATP-binding protein	1	1	1	1	1	1	1	1	1	1	1	11	CORE	COG1132_V#	41.6	890	8.24E-258	K18894	53.3	1188	0
121	WP_019521708.1	-	DUF1080 domain-containing protein	1	1	1	1	1	1	1	1	1	1	1	11	CORE	COG5498_M#	29	89	7.45E-17	K15921	28.5	60	1.69E-07
122	Prokka:JAPCLKMA_02633	-	hypothetical protein	1	0	0	0	0	0	0	0	0	0	0	1	Unique	COG1196_D#	26	136	6.94E-31	NA	27.4	102	3.87E-20
123	OSC68917.1	-	nitrate reductase subunit alpha	1	1	0	0	1	1	0	0	0	0	1	5	Accessory	COG5013_C#	66.1	1683	0	K00370	66.1	1683	0
124	WP_026243407.1	cobN	cobaltochelatase subunit CobN	1	1	0	1	1	1	1	1	1	1	1	10	Accessory	COG1429_H#	62.3	1517	0	K02230	62.3	1517	0
125	WP_102639908.1	pglX	BREX-2 system adenine-specific DNA-methyltransferase PglX	0	0	0	0	0	0	0	0	1	0	0	1	Unique								

cluster	GI	gene	Annotation	Streptomyces_thermocarboxydus_BPSAC147	Streptomyces_sp._4F	Streptomyces_sp._BSE7F	Streptomyces_sp._ETH9427	Streptomyces_sp._FxanaD5	Streptomyces_sp._UNC401CLCol	Actinospica_acidiphila_NRRL_B_24431	Streptomyces_sp._HNS054	Streptomyces_sp._SMS_SU21	Streptomyces_sp._ZS0098	Streptomyces_thermocarboxydus_K155	Addition	Core-Accessory-Unique	COG	%Similarity_COG	Score_COG	Evalue_COG	KEGG	%Similarity_KEGG	Score_KEGG	Evalue_KEGG
126	WP_063837932.1	-	HAMP domain-containing protein	1	1	0	0	1	1	0	1	1	1	1	8	Accessory	COG0642_T#	29.7	215	8.88E-55	NA	29.7	215	2.37E-54
127	WP_033275871.1	-	sensor histidine kinase	1	1	1	1	1	1	1	1	1	1	1	11	CORE	COG0642_T#	38.1	257	2.66E-67	NA	38.1	257	7.1E-67
128	WP_019526450.1	-	5-oxoprolinase	1	0	0	1	1	1	1	1	1	1	1	9	Accessory	COG0145_EQ#	73.8	997	4.68E-290	K01469	77.5	1832	0
129	OSC62435.1	-	peptidase S8	0	1	0	0	0	0	0	0	0	0	0	1	Unique	COG1404_O#	38.2	184	2.18E-45	K17734	38.2	184	5.82E-45
130	OSC70556.1	-	DNA polymerase III subunit alpha	1	1	0	1	1	1	1	1	1	1	1	10	Accessory	COG0587_L#	55.6	1276	0	K02337	55.6	1276	0
131	WP_078504589.1	-	LamG domain-containing protein	0	0	0	0	1	0	0	0	0	0	0	1	Unique								
132	WP_102637884.1	smc	chromosome segregation protein SMC	1	1	0	1	1	1	1	1	1	1	1	10	Accessory	COG1196_D#	52.1	1090	0	K03529	52.1	1090	0
133	WP_146217786.1	-	hypothetical protein	0	0	1	0	0	0	0	0	1	0	0	2	Accessory	COG1205_R#	32.3	46	0.000705	K06877	32.3	46	0.00188
134	WP_146217777.1	-	AAA family ATPase	0	0	0	0	0	0	0	0	1	0	0	1	Unique	COG3973_R#	29.8	114	3.59E-24	NA	24.6	74	1.1E-11
135	WP_048458860.1	pglX	BREX-2 system adenine-specific DNA-methyltransferase PglX	0	0	0	0	0	0	0	1	0	0	0	1	Unique	COG1002_V#	20	47	0.00041				

cluster	GI	gene	Annotation	Streptomyces_thermocarboxydus_BPSAC147	Streptomyces_sp._4F	Streptomyces_sp._BSE7F	Streptomyces_sp._ETH9427	Streptomyces_sp._FxanaD5	Streptomyces_sp._UNC401CLCol	Actinospica_acidiphila_NRRL_B_24431	Streptomyces_sp._HNS054	Streptomyces_sp._SMS_SU21	Streptomyces_sp._ZS0098	Streptomyces_thermocarboxydus_K155	Addition	Core-Accessory-Unique	COG	%Similarity_COG	Score_COG	Evalue_COG	KEGG	%Similarity_KEGG	Score_KEGG	Evalue_KEGG
136	Prokka:CIODPCDJ_06730	-	hypothetical protein	0	0	0	0	0	0	0	0	0	0	1	1	Unique								
137	Prokka:CIODPCDJ_04027	-	hypothetical protein	0	0	0	0	0	0	0	0	0	0	1	1	Unique	COG4096_V#	40.9	643	1.69E-183	K01153	34.5	473	8.07E-132
138	Prokka:CIODPCDJ_04448	rep_3	ATP-dependent DNA helicase Rep	1	1	1	1	1	1	1	1	1	1	1	11	CORE	COG0210_L#	41.2	494	9.66E-139	K03657	40	512	9.16E-144
139	WP_102639293.1	-	DNA polymerase III subunit alpha	1	1	0	1	1	1	1	1	1	1	1	10	Accessory	COG0587_L#	33.8	453	3.22E-126	K14162	33.8	453	8.61E-126
140	WP_019521799.1	-	ATP-binding protein	1	0	1	0	1	1	0	0	1	1	1	7	Accessory								
141	WP_033273635.1	-	NACHT domain-containing protein	0	0	0	0	0	0	1	0	0	0	0	1	Unique	COG2319_R#	34.7	338	1.16E-91	NA	35.4	276	1.44E-72
142	OSC69659.1	-	hypothetical protein	0	1	0	0	0	0	0	0	0	0	0	1	Unique	COG2319_R#	26.5	121	2.9E-26	NA	29.5	77	9.82E-13
143	WP_048456660.1	putP	sodium/proline symporter PutP	1	0	0	1	1	1	1	1	1	1	1	9	Accessory	COG0591_ER#	48.8	464	1.39E-129	K11928	46.1	435	1.85E-120
144	WP_102637532.1	-	hypothetical protein	0	0	0	0	0	0	0	1	1	0	1	3	Accessory								
145	PWE07876.1	mfd	transcription-repair coupling factor	1	1	1	0	1	1	1	1	1	1	1	10	Accessory	COG1197_LK#	55.8	1270	0	K03723	55.8	1270	0
146	Prokka:CIODPCDJ_05117	-	hypothetical protein	0	1	1	0	1	1	0	1	0	0	1	6	Accessory	COG3903_R#	38.8	330	2.39E-89	NA	35.4	351	2.68E-95

cluster	GI	gene	Annotation	Streptomyces_thermocarboxydus_BPSAC147	Streptomyces_sp._4F	Streptomyces_sp._BSE7F	Streptomyces_sp._ETH9427	Streptomyces_sp._FxanaD5	Streptomyces_sp._UNC401CLCol	Actinospica_acidiphila_NRRL_B_24431	Streptomyces_sp._HNS054	Streptomyces_sp._SMS_SU21	Streptomyces_sp._ZS0098	Streptomyces_thermocarboxydus_K155	Addition	Core-Accessory-Unique	COG	%Similarity_COG	Score_COG	Evalue_COG	KEGG	%Similarity_KEGG	Score_KEGG	Evalue_KEGG
147	AXI91280.1	-	hypothetical protein	0	0	0	1	0	0	0	0	0	0	0	1	Unique	COG1204_R#	30.2	174	2.19E-42	K03726	29.7	163	1.35E-38
148	WP_102638051.1	-	DEAD/DEAH box helicase	0	0	0	0	0	0	0	0	1	0	0	1	Unique	COG0553_KL#	24	117	4.15E-25	NA	30.6	107	1.15E-21
149	OSC70555.1	-	hypothetical protein	0	1	0	0	0	0	0	0	0	0	0	1	Unique	COG0457_R#	26.9	105	1.25E-21	NA	27.7	67	1.01E-09
150	Prokka:CIODPCDJ_02201	mrdB	Peptidoglycan glycosyltransferase MrdB	1	1	1	1	1	1	1	1	1	1	1	11	CORE	COG0768_M#	26	245	1.31E-63	K05837	35.1	236	9.56E-61
151	Prokka:JAPCLKMA_00705	-	hypothetical protein	1	0	1	1	1	1	0	0	1	0	1	7	Accessory	COG3064_M#	31.5	100	4E-20	K03646	32.1	108	5.12E-22
152	WP_019522710.1	metH	methionine synthase	1	1	1	1	1	1	1	1	1	1	1	11	CORE	COG1410_E#	66.5	1140	0	K00548	65.8	1537	0
153	WP_028961340.1	-	ATP-binding protein	0	0	0	0	0	1	0	0	0	0	0	1	Unique								
154	AXI87436.1	-	ABC transporter	1	1	1	1	1	1	1	1	1	1	1	11	CORE	COG4988_CO#	44.7	393	2.28E-108	K16013	44.7	393	6.08E-108
155	WP_019525218.1	rpoB	DNA-directed RNA polymerase subunit beta	1	1	0	1	1	1	1	1	1	1	1	10	Accessory	COG0085_K#	75.7	1772	0	K03043	75.7	1772	0
156	Prokka:CIODPCDJ_00236	pycA	Pyruvate carboxylase	1	1	1	1	1	0	1	1	1	1	1	10	Accessory	COG1038_C#	75.1	1671	0	K01958	75.1	1671	0
157	WP_158688249.1	-	hypothetical protein	0	0	0	0	1	0	0	0	0	0	0	1	Unique	COG5635_T#	39.5	362	7.3E-99	NA	32.7	284	3.95E-75
158	WP_048458569.1	-	WD40 repeat domain-containing protein	0	0	0	0	0	0	0	1	0	0	0	1	Unique								

cluster	GI	gene	Annotation	Streptomyces_thermocarboxydus_BPSAC147	Streptomyces_sp._4F	Streptomyces_sp._BSE7F	Streptomyces_sp._ETH9427	Streptomyces_sp._FxanaD5	Streptomyces_sp._UNC401CLCol	Actinospica_acidiphila_NRRL_B_24431	Streptomyces_sp._HNS054	Streptomyces_sp._SMS_SU21	Streptomyces_sp._ZS0098	Streptomyces_thermocarboxydus_K155	Addition	Core-Accessory-Unique	COG	%Similarity_COG	Score_COG	Evalue_COG	KEGG	%Similarity_KEGG	Score_KEGG	Evalue_KEGG
159	WP_048458576.1	-	DNRLRE domain-containing protein	0	0	0	0	0	0	0	1	0	0	0	1	Unique	COG3409_M#	32.9	45	0.00115				
160	WP_048459187.1	-	hypothetical protein	0	0	0	0	0	0	0	1	0	0	0	1	Unique	COG1201_R#	27.4	50	0.000061	K03724	27.4	50	0.000163
161	WP_051724534.1	-	S8 family serine peptidase	1	0	1	1	0	0	1	1	1	1	0	7	Accessory	COG1404_O#	29.3	207	2.28E-52	NA	29.3	207	6.08E-52
162	WP_122213578.1	-	hypothetical protein	0	0	0	0	0	0	0	0	0	1	0	1	Unique	COG0553_KL#	32.5	233	3.88E-60	K03580	31.8	76	2.12E-12
163	PWE11168.1	-	cell division protein FtsK	1	1	1	1	1	1	1	1	1	1	1	11	CORE	COG1674_D#	29.8	67	2.82E-10	K03466	29.6	60	9.2E-08
164	WP_019525385.1	-	LysM peptidoglycan-binding domain-containing protein	0	1	0	0	1	0	0	0	0	0	1	3	Accessory	COG3170_NU#	28.3	74	2.3E-12	NA	33.3	54	0.0000112
165	WP_109288767.1	-	hypothetical protein	0	0	1	1	1	1	0	1	1	1	1	8	Accessory	COG1196_D#	24.2	100	3.9E-20	NA	34.3	96	1.97E-18
166	OSC72853.1	-	peptidase S41	1	1	0	1	1	1	1	1	1	1	1	10	Accessory	COG0793_M#	39.6	254	1.61E-66	K08676	23.6	97	6.72E-19
167	WP_028961795.1	-	hypothetical protein	1	1	1	1	1	1	1	1	1	1	1	11	CORE								
168	WP_086020127.1	-	sensor histidine kinase	1	1	0	1	1	1	1	1	1	1	1	10	Accessory	COG0642_T#	42.1	255	9.35E-67	NA	42.1	255	2.5E-66
169	WP_033274505.1	-	APHP domain-containing protein	1	1	0	1	1	1	1	1	0	1	1	9	Accessory	COG1572_S#	30.8	70	3.26E-11				
170	Prokka:JAPCLKMA_01339	-	hypothetical protein	1	0	0	0	0	0	0	0	0	0	0	1	Unique	COG3064_M#	32.2	87	4.4E-16	NA	28.7	117	1.06E-24

cluster	GI	gene	Annotation	Streptomyces_thermocarboxydus_BPSAC147	Streptomyces_sp._4F	Streptomyces_sp._BSE7F	Streptomyces_sp._ETH9427	Streptomyces_sp._FxanaD5	Streptomyces_sp._UNC401CLCol	Actinospica_acidiphila_NRRL_B_24431	Streptomyces_sp._HNS054	Streptomyces_sp._SMS_SU21	Streptomyces_sp._ZS0098	Streptomyces_thermocarboxydus_K155	Addition	Core-Accessory-Unique	COG	%Similarity_COG	Score_COG	Evalue_COG	KEGG	%Similarity_KEGG	Score_KEGG	Evalue_KEGG
171	WP_064743308.1	-	N-6 DNA methylase	0	0	0	0	0	1	0	0	0	0	0	1	Unique								
172	OSC63672.1	-	hypothetical protein	0	1	0	0	0	0	0	0	0	0	0	1	Unique								
173	WP_078504522.1	-	DNRLRE domain-containing protein	0	0	0	0	1	0	0	0	0	0	0	1	Unique								
174	Prokka:JAPCLKMA_04870	-	hypothetical protein	1	0	1	1	1	1	1	1	1	1	1	10	Accessory	COG1404_O#	38.9	192	7.32E-48	K17734	38.9	192	1.95E-47
175	Prokka:CIODPCDJ_00823	afsR_1	Regulatory protein AfsR	1	0	0	0	1	1	0	0	0	0	1	4	Accessory	COG3629_T#	30.7	110	4.76E-23	NA	30.7	109	2.17E-22
176	WP_033276846.1	-	S8 family serine peptidase	1	1	1	1	1	1	1	1	1	1	1	11	CORE	COG1404_O#	30.5	117	2.28E-25	K01280	26.8	153	1E-35
177	WP_122214927.1	carB	carbamoyl-phosphate synthase large subunit	1	1	1	1	1	1	1	1	1	1	1	11	CORE	COG0458_EF#	69.2	1480	0	K01955	69.2	1480	0
178	WP_102637107.1	-	AAA family ATPase	0	0	0	1	1	1	1	1	1	1	1	8	Accessory	COG0464_O#	36.9	327	2.47E-88	K06413	41.4	204	6.46E-51
179	AXI86426.1	-	Wall-associated protein	0	0	1	1	0	0	0	0	1	0	0	3	Accessory	COG3209_M#	23.5	154	2.19E-36	NA	24.2	45	0.00294
180	WP_028960394.1	-	alpha-mannosidase	1	0	1	1	1	1	1	1	1	1	1	10	Accessory	COG3537_G#	33.7	432	5.45E-120	NA	33.7	432	1.46E-119
181	AXI85378.1	-	hypothetical protein	0	0	0	1	0	0	0	0	0	0	0	1	Unique								
182	PWE09796.1	-	DNA methyltransferase	0	0	1	0	0	0	0	0	0	0	0	1	Unique								

cluster	GI	gene	Annotation	Streptomyces_thermocarboxydus_BPSAC147	Streptomyces_sp._4F	Streptomyces_sp._BSE7F	Streptomyces_sp._ETH9427	Streptomyces_sp._FxanaD5	Streptomyces_sp._UNC401CLCol	Actinospica_acidiphila_NRRL_B_24431	Streptomyces_sp._HNS054	Streptomyces_sp._SMS_SU21	Streptomyces_sp._ZS0098	Streptomyces_thermocarboxydus_K155	Addition	Core-Accessory-Unique	COG	%Similarity_COG	Score_COG	Evalue_COG	KEGG	%Similarity_KEGG	Score_KEGG	Evalue_KEGG
183	WP_028959797.1	-	AfsR family transcriptional regulator	1	1	0	1	1	1	1	1	1	1	1	10	Accessory	COG3903_R#	33.7	290	2.54E-77	NA	31.1	315	1.51E-84
184	WP_102638058.1	-	type I restriction endonuclease subunit R	0	1	0	0	0	0	0	0	1	0	0	2	Accessory	COG0610_V#	37.7	687	9.31E-197	K01153	27	211	3.06E-53
185	WP_033274438.1	-	glycoside hydrolase family 2	1	0	1	1	1	1	1	1	1	1	1	10	Accessory					NA	42.1	437	4.48E-121
186	AXI91197.1	-	non-ribosomal peptide synthetase	0	0	0	1	0	0	0	1	1	1	0	4	Accessory	COG1020_Q#	34.6	574	1.15E-162	K04780	34.6	574	3.07E-162
187	WP_167338213.1	-	N-6 DNA methylase	0	0	0	0	0	0	1	0	0	0	0	1	Unique								
188	WP_033274618.1	-	ABC transporter permease	1	1	1	0	1	1	1	0	1	1	1	9	Accessory	COG0577_V#	24.6	50	0.0000577				
189	Prokka:JAPCLKMA_04471	malT	HTH-type transcriptional regulator MalT	1	1	0	0	1	1	1	1	1	1	1	9	Accessory	COG3899_R#	26.3	141	1.9E-32	NA	25.7	74	5.83E-12
190	AXI89652.1	-	hypothetical protein	0	0	0	1	0	0	0	0	0	0	0	1	Unique								
191	WP_086020157.1	-	EAL domain-containing protein	1	1	1	0	1	1	1	1	1	1	1	10	Accessory	COG5001_T#	31.9	235	1.26E-60	NA	32.9	285	2.83E-75
192	AXI91303.1	-	hypothetical protein	0	0	0	1	0	0	0	0	0	0	0	1	Unique								
193	PWE12355.1	-	hypothetical protein	0	0	1	1	0	0	0	0	0	0	0	2	Accessory	COG2931_Q#	29.6	48	0.000167	NA	30.4	178	4.86E-43

cluster	GI	gene	Annotation	Streptomyces_thermocarboxydus_BPSAC147	Streptomyces_sp._4F	Streptomyces_sp._BSE7F	Streptomyces_sp._ETH9427	Streptomyces_sp._FxanaD5	Streptomyces_sp._UNC401CLCol	Actinospica_acidiphila_NRRL_B_24431	Streptomyces_sp._HNS054	Streptomyces_sp._SMS_SU21	Streptomyces_sp._ZS0098	Streptomyces_thermocarboxydus_K155	Addition	Core-Accessory-Unique	COG	%Similarity_COG	Score_COG	Evalue_COG	KEGG	%Similarity_KEGG	Score_KEGG	Evalue_KEGG
194	WP_122216866.1	-	ATP-dependent helicase	1	1	0	0	1	1	1	1	1	1	1	9	Accessory	COG1643_L#	44.7	365	6.14E-100	K03579	46.8	355	1.7E-96
195	WP_026243571.1	-	S41 family peptidase	1	1	0	1	1	1	1	1	1	1	1	10	Accessory	COG0793_M#	44.9	318	8.6E-86				
196	WP_122212777.1	-	DUF11 domain-containing protein	0	0	0	1	0	0	0	1	1	1	0	4	Accessory	COG0443_O#	29.4	65	1.01E-09	NA	25.4	58	4.29E-07
197	WP_028959541.1	-	hypothetical protein	1	0	0	0	0	1	0	0	0	1	1	4	Accessory								
198	Prokka:CIODPCDJ_00975	-	hypothetical protein	1	1	1	1	1	1	1	1	1	1	1	11	CORE	COG0642_T#	30.3	208	1.25E-52	NA	30.3	208	3.34E-52
199	WP_052183377.1	-	hypothetical protein	0	0	0	0	0	1	0	0	0	0	0	1	Unique	COG3209_M#	30.1	414	1.5E-114	NA	27.8	211	5.15E-53
200	WP_102639543.1	-	hypothetical protein	1	0	0	1	1	0	1	0	1	0	0	5	Accessory	COG5394_S#	55.8	45	0.00107				
201	WP_033273447.1	-	family 78 glycoside hydrolase catalytic domain	1	0	0	0	1	1	1	0	0	0	1	5	Accessory					NA	32.5	410	4.41E-113
202	WP_102639228.1	-	tetratricopeptide repeat protein	1	1	1	1	1	1	1	1	1	1	1	11	CORE								
203	WP_078895349.1	-	DNRLRE domain-containing protein	0	0	0	1	0	0	0	1	1	1	1	5	Accessory								
204	WP_102637411.1	-	type I restriction endonuclease subunit R	1	0	0	0	0	0	0	0	1	1	1	4	Accessory	COG0610_V#	37.2	569	2.1E-161				
205	WP_102637602.1	-	DEAD/DEAH box helicase	0	0	1	0	0	0	0	0	1	0	0	2	Accessory	COG0553_KL#	26.8	263	2.47E-69	NA	26.8	96	1.39E-18

cluster	GI	gene	Annotation	Streptomyces_thermocarboxydus_BPSAC147	Streptomyces_sp._4F	Streptomyces_sp._BSE7F	Streptomyces_sp._ETH9427	Streptomyces_sp._FxanaD5	Streptomyces_sp._UNC401CLCol	Actinospica_acidiphila_NRRL_B_24431	Streptomyces_sp._HNS054	Streptomyces_sp._SMS_SU21	Streptomyces_sp._ZS0098	Streptomyces_thermocarboxydus_K155	Addition	Core-Accessory-Unique	COG	%Similarity_COG	Score_COG	Evalue_COG	KEGG	%Similarity_KEGG	Score_KEGG	Evalue_KEGG
206	WP_122215803.1	-	CHAT domain-containing protein	0	0	0	0	0	0	0	0	0	1	0	1	Unique								
207	WP_028960770.1	-	isoleucine--tRNA ligase	1	1	1	1	1	1	1	1	1	1	1	11	CORE	COG0060_J#	41.4	882	1.12E-255	K01870	41.4	882	2.99E-255
208	AXI89655.1	-	helicase	0	1	0	1	0	0	0	0	0	0	0	2	Accessory	COG0553_KL#	43.8	506	2.18E-142	K03580	28	101	4.32E-20
209	WP_019527426.1	-	dTMP kinase	1	0	1	1	1	1	1	1	1	1	1	10	Accessory	COG0125_F#	51.9	175	1.15E-42	K00943	51.9	175	3.07E-42
210	WP_048460597.1	lanM	type 2 lantipeptide synthetase LanM	0	0	0	1	0	0	0	1	0	0	0	2	Accessory	COG4403_V#	28.2	302	6.23E-81				
211	Prokka:JAPCLKMA_03457	srmB_1	ATP-dependent RNA helicase SrmB	1	1	0	0	1	0	1	0	0	1	0	5	Accessory	COG1061_KL#	38.5	258	1.34E-67	K17677	35.3	254	5.18E-66
212	WP_033276487.1	-	hypothetical protein	1	1	1	0	1	1	1	1	1	1	1	10	Accessory								
213	WP_033274296.1	-	lantibiotic dehydratase	1	1	0	0	1	1	1	0	0	0	1	6	Accessory								
214	WP_019526078.1	-	hypothetical protein	0	0	0	0	1	0	0	0	0	0	0	1	Unique								
215	AXI90449.1	-	modular polyketide synthase	0	0	1	1	0	0	0	1	1	1	0	5	Accessory	COG3321_Q#	41.9	678	5.41E-194	K12434	41.9	678	1.45E-193
216	WP_102642063.1	-	helix-turn-helix transcriptional regulator	1	1	0	1	1	1	1	0	1	1	1	9	Accessory	COG2771_K#	38.3	79	6.52E-14	NA	27.1	70	1.38E-10

cluster	GI	gene	Annotation	Streptomyces_thermocarboxydus_BPSAC147	Streptomyces_sp._4F	Streptomyces_sp._BSE7F	Streptomyces_sp._ETH9427	Streptomyces_sp._FxanaD5	Streptomyces_sp._UNC401CLCol	Actinospica_acidiphila_NRRL_B_24431	Streptomyces_sp._HNS054	Streptomyces_sp._SMS_SU21	Streptomyces_sp._ZS0098	Streptomyces_thermocarboxydus_K155	Addition	Core-Accessory-Unique	COG	%Similarity_COG	Score_COG	Evalue_COG	KEGG	%Similarity_KEGG	Score_KEGG	Evalue_KEGG
217	WP_122216965.1	-	efflux RND transporter permease subunit	1	1	0	0	1	1	1	1	1	1	1	9	Accessory	COG0841_V#	33.1	474	1.19E-132	K03296	33.1	474	3.17E-132
218	WP_048460334.1	-	type I polyketide synthase	0	1	1	1	0	0	0	1	1	1	0	6	Accessory	COG3321_Q#	59.7	1020	3.4E-297	K12434	59.7	1020	9.08E-297
219	Prokka:CIODPCDJ_01581	uvrA_1	UvrABC system protein A	1	1	1	1	1	1	1	1	1	1	1	11	CORE	COG0178_L#	72.8	1423	0	K03701	72.8	1421	0
220	WP_019521647.1	-	helicase	0	0	1	0	1	1	1	0	0	0	0	4	Accessory								
221	WP_102637677.1	-	discoidin domain-containing protein	0	0	1	1	0	0	0	1	1	1	0	5	Accessory								
222	PWE10312.1	-	hypothetical protein	0	0	1	0	0	0	0	0	0	0	0	1	Unique								
223	WP_158693578.1	-	N-6 DNA methylase	0	0	0	0	0	1	0	0	0	0	0	1	Unique	COG1002_V#	28.6	311	1.32E-83	NA	28.6	311	3.54E-83
224	WP_078611386.1	-	DEAD/DEAH box helicase	0	0	0	0	0	1	0	0	0	0	0	1	Unique	COG1204_R#	29.4	164	2E-39	K03726	25	134	5.92E-30
225	WP_048460705.1	-	non-ribosomal peptide synthetase/polyketide synthase	0	0	0	1	0	0	0	1	1	1	0	4	Accessory	COG1020_Q#	30.9	372	6.28E-102	K15655	32.4	257	4.61E-67
226	Prokka:JAPCLKMA_01628	nisB_1	Nisin biosynthesis protein NisB	1	0	0	0	0	0	0	0	0	0	0	1	Unique								
227	Prokka:JAPCLKMA_06506	-	hypothetical protein	1	0	0	0	0	0	0	0	0	0	0	1	Unique	COG5280_S#	24.5	149	6.57E-35	NA	23.6	178	2.7E-43

cluster	GI	gene	Annotation	Streptomyces_thermocarboxydus_BPSAC147	Streptomyces_sp._4F	Streptomyces_sp._BSE7F	Streptomyces_sp._ETH9427	Streptomyces_sp._FxanaD5	Streptomyces_sp._UNC401CLCol	Actinospica_acidiphila_NRRL_B_24431	Streptomyces_sp._HNS054	Streptomyces_sp._SMS_SU21	Streptomyces_sp._ZS0098	Streptomyces_thermocarboxydus_K155	Addition	Core-Accessory-Unique	COG	%Similarity_COG	Score_COG	Evalue_COG	KEGG	%Similarity_KEGG	Score_KEGG	Evalue_KEGG
228	PWE08045.1	-	restriction endonuclease	0	0	1	0	0	0	0	0	0	0	0	1	Unique	COG1002_V#	30.5	174	1.46E-42				
229	WP_109288657.1	infB	translation initiation factor IF-2	1	1	1	1	1	1	1	1	1	1	1	11	CORE	COG0532_J#	58.5	1025	1.03E-298	K02519	62	1013	1.85E-294
230	OSC62515.1	-	hypothetical protein	0	1	0	0	0	0	0	0	0	0	0	1	Unique	COG1020_Q#	45.8	807	7.53E-233	K04780	42.7	753	2.64E-216
231	WP_048455381.1	-	FAD-binding oxidoreductase	0	0	0	1	0	0	1	1	0	0	0	3	Accessory	COG0277_C#	36.5	351	8.65E-96	NA	26.8	319	1.66E-85
232	WP_122213581.1	-	hypothetical protein	0	0	0	0	0	0	0	0	0	1	0	1	Unique								
233	OSC70035.1	-	ATP-binding protein	0	1	0	0	0	0	0	0	0	0	0	1	Unique								
234	AXI87795.1	-	D-alanyl-D-alanine carboxypeptidase	0	0	1	1	0	0	0	1	1	0	0	4	Accessory	COG1686_M#	28.3	82	1.27E-14	NA	28.2	116	1.62E-24
235	OSC71392.1	-	ABC transporter	0	1	0	0	0	0	0	0	0	0	0	1	Unique	COG0178_L#	39.6	537	1.44E-151	K03701	39.6	537	3.85E-151
236	Prokka:JAPCLKMA_02219	-	Tn3 family transposase ISNpu13	1	1	0	0	0	0	0	0	0	0	0	2	Accessory	COG4644_L#	44.5	799	2.03E-230				
237	WP_033275405.1	-	alpha-mannosidase	1	1	1	1	1	1	1	1	1	1	1	11	CORE	COG0383_G#	31.7	464	1.18E-129	K01191	37.1	616	4.97E-175
238	WP_102638548.1	-	SMC family ATPase	0	0	0	0	0	0	0	1	1	0	0	2	Accessory	COG0419_L#	29.8	336	2.84E-91	K03546	25	195	2.09E-48
239	OSC64985.1	-	hypothetical protein	1	1	1	1	1	1	1	1	1	1	1	11	CORE	COG1501_G#	39.1	86	8.72E-16				

cluster	GI	gene	Annotation	Streptomyces_thermocarboxydus_BPSAC147	Streptomyces_sp._4F	Streptomyces_sp._BSE7F	Streptomyces_sp._ETH9427	Streptomyces_sp._FxanaD5	Streptomyces_sp._UNC401CLCol	Actinospica_acidiphila_NRRL_B_24431	Streptomyces_sp._HNS054	Streptomyces_sp._SMS_SU21	Streptomyces_sp._ZS0098	Streptomyces_thermocarboxydus_K155	Addition	Core-Accessory-Unique	COG	%Similarity_COG	Score_COG	Evalue_COG	KEGG	%Similarity_KEGG	Score_KEGG	Evalue_KEGG
240	WP_028959803.1	-	bifunctional [glutamine synthetase] adenylyltransferase/[glutamine synthetase]-adenylyl-L-tyrosine phosphorylase	1	1	0	1	1	1	1	1	1	1	1	10	Accessory	COG1391_OT#	47.9	835	2.53E-241	K00982	47.8	834	8.83E-241
241	WP_048459637.1	-	tetratricopeptide repeat protein	1	1	1	1	1	1	1	1	1	1	1	11	CORE								
242	OSC71020.1	-	tetratricopeptide repeat protein	0	1	0	0	0	0	0	0	0	0	0	1	Unique	COG0457_R#	25.5	114	2.28E-24	NA	28.3	64	5.54E-09
243	Prokka:CIODPCDJ_03644	-	hypothetical protein	1	0	0	0	1	1	0	0	0	1	1	5	Accessory	COG3629_T#	28.9	114	1.75E-24	NA	30.9	107	7.44E-22
244	WP_167338214.1	-	hypothetical protein	1	0	0	0	1	1	1	0	0	0	1	5	Accessory	COG3629_T#	34.6	144	2.69E-33	NA	37.3	142	2.09E-32
245	OSC66414.1	-	ATP-binding cassette family protein	1	1	1	1	1	1	1	1	1	1	1	11	CORE	COG0419_L#	29.9	369	3.92E-101	K03546	22.9	202	1.7E-50
246	WP_102639921.1	pglZ	BREX-2 system phosphatase PglZ	0	0	0	0	0	0	0	0	1	0	0	1	Unique								

cluster	GI	gene	Annotation	Streptomyces_thermocarboxydus_BPSAC147	Streptomyces_sp._4F	Streptomyces_sp._BSE7F	Streptomyces_sp._ETH9427	Streptomyces_sp._FxanaD5	Streptomyces_sp._UNC401CLCol	Actinospica_acidiphila_NRRL_B_24431	Streptomyces_sp._HNS054	Streptomyces_sp._SMS_SU21	Streptomyces_sp._ZS0098	Streptomyces_thermocarboxydus_K155	Addition	Core-Accessory-Unique	COG	%Similarity_COG	Score_COG	Evalue_COG	KEGG	%Similarity_KEGG	Score_KEGG	Evalue_KEGG
247	OSC60786.1	-	transcriptional regulator	0	1	0	0	0	0	0	0	0	0	0	1	Unique	COG3629_T#	33.7	148	1.09E-34	NA	33.7	148	2.9E-34
248	OSC68380.1	-	hypothetical protein	1	1	1	1	1	1	1	1	1	1	1	11	CORE	COG1615_S#	38.7	664	7.69E-190	K09118	38.9	664	1.57E-189
249	WP_102639836.1	-	endonuclease	0	0	0	0	0	0	0	0	1	0	0	1	Unique								
250	OSC72237.1	-	AfsR family transcriptional regulator	1	1	1	1	1	1	1	1	1	1	1	11	CORE	COG3629_T#	34.7	136	5.55E-31	NA	32.4	136	1.14E-30
251	WP_033276325.1	-	alpha-amylase	0	0	1	0	0	0	1	0	1	0	0	3	Accessory	COG0366_G#	40.3	106	8.03E-22	K01182	28.1	81	5.65E-14
252	WP_033273548.1	-	alpha-mannosidase	1	0	0	1	1	1	1	1	0	1	1	8	Accessory	COG3537_G#	37.7	481	9.18E-135	NA	37.7	481	2.45E-134
253	WP_033273629.1	-	DEAD/DEAH box helicase	0	0	0	0	0	0	1	0	0	0	0	1	Unique	COG0553_KL#	48.3	753	9.56E-217	NA	25.4	90	1.21E-16
254	AXI85373.1	-	helicase	0	0	0	1	0	0	0	0	0	0	0	1	Unique	COG0553_KL#	28.8	324	1.87E-87	NA	29.2	168	3.5E-40
255	AXI86159.1	-	zinc carboxypeptidase	1	1	0	1	1	1	1	1	1	1	1	10	Accessory	COG4412_S#	35.7	54	0.00000211	NA	32.2	122	2.88E-26
256	OSC67742.1	-	hypothetical protein	0	1	0	0	0	0	0	0	0	0	0	1	Unique								
257	WP_102640321.1	-	hypothetical protein	1	1	0	1	1	1	1	1	1	1	1	10	Accessory								
258	Prokka:JAPCLKMA_04735	rhlB_2	ATP-dependent RNA helicase RhlB	1	1	1	1	1	1	1	1	1	1	1	11	CORE	COG0553_KL#	38.2	520	1.35E-146	NA	38.2	520	3.61E-146
259	OSC64392.1	-	hypothetical protein	0	1	0	0	0	0	0	0	0	0	0	1	Unique	COG3903_R#	33.9	282	8.08E-75	NA	32.3	329	1.54E-88

cluster	GI	gene	Annotation	Streptomyces_thermocarboxydus_BPSAC147	Streptomyces_sp._4F	Streptomyces_sp._BSE7F	Streptomyces_sp._ETH9427	Streptomyces_sp._FxanaD5	Streptomyces_sp._UNC401CLCol	Actinospica_acidiphila_NRRL_B_24431	Streptomyces_sp._HNS054	Streptomyces_sp._SMS_SU21	Streptomyces_sp._ZS0098	Streptomyces_thermocarboxydus_K155	Addition	Core-Accessory-Unique	COG	%Similarity_COG	Score_COG	Evalue_COG	KEGG	%Similarity_KEGG	Score_KEGG	Evalue_KEGG
260	WP_037778539.1	-	CRISPR-associated helicase/endonuclease Cas3	0	0	0	0	1	0	0	0	0	0	0	1	Unique	COG1203_R#	29.4	230	3.64E-59	K07012	25.9	198	2.4E-49
261	WP_048458867.1	pglZ	BREX-2 system phosphatase PglZ	0	0	0	0	0	0	0	1	0	0	0	1	Unique								
262	OSC68927.1	-	cellulose 1,4-beta-cellobiosidase	1	1	1	1	1	1	1	1	1	1	1	11	CORE					NA	57.7	772	6.84E-222
263	WP_048460599.1	-	LuxR family transcriptional regulator	0	0	0	0	0	0	0	1	0	0	0	1	Unique	COG3899_R#	26.5	150	3.64E-35	NA	26.5	74	5.2E-12
264	OSC65883.1	-	Na+/H+ antiporter subunit A	0	1	0	0	0	0	0	0	0	0	0	1	Unique	COG1009_CP#	46.2	422	4.97E-117	K05565	35.5	393	5.05E-108
265	WP_033277864.1	-	GNAT family N-acetyltransferase	0	1	1	1	1	1	1	1	1	1	0	9	Accessory	COG1042_C#	35.6	253	3.98E-66	K09181	32.4	190	8.47E-47
266	AXI87735.1	-	FAD-binding oxidoreductase	1	1	1	1	1	1	1	1	1	1	1	11	CORE	COG0277_C#	42.5	416	2.07E-115	NA	31.7	257	5.62E-67
267	WP_019526310.1	-	vitamin B12-dependent ribonucleotide reductase	1	1	1	1	1	1	1	1	1	1	1	11	CORE	COG0209_F#	37.7	497	9.24E-140	K00525	37.7	497	2.47E-139

cluster	GI	gene	Annotation	Streptomyces_thermocarboxydus_BPSAC147	Streptomyces_sp._4F	Streptomyces_sp._BSE7F	Streptomyces_sp._ETH9427	Streptomyces_sp._FxanaD5	Streptomyces_sp._UNC401CLCol	Actinospica_acidiphila_NRRL_B_24431	Streptomyces_sp._HNS054	Streptomyces_sp._SMS_SU21	Streptomyces_sp._ZS0098	Streptomyces_thermocarboxydus_K155	Addition	Core-Accessory-Unique	COG	%Similarity_COG	Score_COG	Evalue_COG	KEGG	%Similarity_KEGG	Score_KEGG	Evalue_KEGG
268	WP_033278090.1	gcvP	aminomethyl-transferring glycine dehydrogenase	1	1	1	1	1	1	1	1	1	1	1	11	CORE	COG1003_E#	69.9	726	1.22E-208	K00281	65.8	1235	0
269	WP_019521625.1	-	DEAD/DEAH box helicase	1	1	1	0	1	1	0	0	0	0	0	5	Accessory	COG0553_KL#	43	697	6.07E-200	NA	26.9	139	2.22E-31
270	OSC72241.1	-	hypothetical protein	0	1	0	0	0	0	0	0	0	0	0	1	Unique								
271	Prokka:JAPCLKMA_04691	-	hypothetical protein	1	0	1	0	1	1	0	1	1	1	1	8	Accessory					K01197	28.5	229	1.25E-58
272	WP_051724508.1	-	helix-turn-helix transcriptional regulator	0	0	0	0	0	0	1	0	0	0	0	1	Unique	COG2909_K#	30.8	64	2.6E-09	K11618	32.5	48	0.000514
273	WP_028961639.1	-	glycosyltransferase	0	0	0	0	1	1	0	0	0	0	1	3	Accessory	COG0438_M#	32.9	139	8.29E-32	K01784	34.6	291	2.67E-77
274	OSC70646.1	-	hypothetical protein	0	1	0	0	0	0	0	0	0	0	0	1	Unique	COG0433_R#	30.5	44	0.00362				
275	AXI86674.1	-	leucine--tRNA ligase	1	1	1	1	1	1	1	1	1	1	1	11	CORE	COG0495_J#	61.2	1171	0	K01869	61.2	1171	0
276	WP_122215805.1	-	ABC transporter substrate-binding protein	1	0	0	1	1	1	1	1	1	1	1	9	Accessory								
277	WP_048456159.1	-	hypothetical protein	1	0	0	0	1	1	0	1	0	1	1	6	Accessory								
278	OSC70323.1	-	DNA topoisomerase I	1	1	1	1	1	1	1	1	1	1	1	11	CORE	COG0550_L#	63.1	731	3.76E-210	K03168	61.5	1064	6.53e-310
279	WP_028961840.1	-	DEAD/DEAH box helicase	1	1	1	1	1	1	1	1	1	1	1	11	CORE	COG4581_L#	53.9	906	8.51E-263	K03727	53.9	906	2.27E-262

cluster	GI	gene	Annotation	Streptomyces_thermocarboxydus_BPSAC147	Streptomyces_sp._4F	Streptomyces_sp._BSE7F	Streptomyces_sp._ETH9427	Streptomyces_sp._FxanaD5	Streptomyces_sp._UNC401CLCol	Actinospica_acidiphila_NRRL_B_24431	Streptomyces_sp._HNS054	Streptomyces_sp._SMS_SU21	Streptomyces_sp._ZS0098	Streptomyces_thermocarboxydus_K155	Addition	Core-Accessory-Unique	COG	%Similarity_COG	Score_COG	Evalue_COG	KEGG	%Similarity_KEGG	Score_KEGG	Evalue_KEGG
280	OSC61756.1	-	sugar hydrolase	1	1	1	1	1	1	1	1	1	1	1	11	CORE	COG1472_G#	38.2	617	1.03E-175	NA	34.8	259	1.46E-67
281	Prokka:JAPCLKMA_01481	-	hypothetical protein	1	0	0	0	0	0	0	0	0	0	0	1	Unique								
282	WP_048457351.1	-	hypothetical protein	1	1	1	1	1	1	1	1	1	1	1	11	CORE	COG0455_D#	31.9	100	4.23E-20	K06236	34.5	242	2.4E-62
283	OSC69718.1	-	hypothetical protein	0	1	0	0	0	0	0	0	0	0	0	1	Unique	COG2319_R#	26.1	184	1.7E-45	NA	24.6	138	4.88E-31
284	OSC63309.1	-	preprotein translocase subunit SecA	1	1	1	1	1	1	1	1	1	1	1	11	CORE	COG0653_U#	62	1132	0	K03070	62	1132	0
285	OSC62481.1	-	hypothetical protein	0	1	0	0	0	0	0	0	0	0	0	1	Unique	COG1020_Q#	43.7	456	1.78E-127	K04780	43.7	456	4.74E-127
286	WP_019526200.1	-	hypothetical protein	1	1	1	1	1	1	1	1	1	1	1	11	CORE	COG1874_G#	32.9	382	5.55E-105	K12308	32.9	382	1.48E-104
287	OSC61097.1	-	LuxR family transcriptional regulator	0	1	0	0	0	0	0	0	0	0	0	1	Unique	COG2771_K#	33.2	88	1.66E-16	NA	28.5	101	5.05E-20
288	Prokka:CIODPCDJ_02767	quiP	Acyl-homoserine lactone acylase QuiP	1	1	1	1	1	1	1	1	1	1	1	11	CORE	COG2366_R#	45.8	373	1.97E-102	K01434	28.5	158	2.66E-37
289	WP_019524916.1	cas3	CRISPR-associated helicase Cas3'	0	0	0	0	1	0	0	0	0	0	1	2	Accessory	COG1203_R#	28.4	225	8.67E-58	K07012	28.4	225	2.31E-57
290	AXI89538.1	-	Na+/H+ antiporter subunit A	0	0	1	1	0	0	0	0	1	0	0	3	Accessory	COG1009_CP#	44.9	410	1.9E-113	K05565	35.4	433	5.59E-120

cluster	GI	gene	Annotation	Streptomyces_thermocarboxydus_BPSAC147	Streptomyces_sp._4F	Streptomyces_sp._BSE7F	Streptomyces_sp._ETH9427	Streptomyces_sp._FxanaD5	Streptomyces_sp._UNC401CLCol	Actinospica_acidiphila_NRRL_B_24431	Streptomyces_sp._HNS054	Streptomyces_sp._SMS_SU21	Streptomyces_sp._ZS0098	Streptomyces_thermocarboxydus_K155	Addition	Core-Accessory-Unique	COG	%Similarity_COG	Score_COG	Evalue_COG	KEGG	%Similarity_KEGG	Score_KEGG	Evalue_KEGG
291	WP_033275282.1	-	NHLP bacteriocin export ABC transporter permease/ATPase subunit	1	1	1	1	1	1	1	1	1	1	1	11	CORE	COG2274_V#	37.8	485	3.55E-136	K06147	29.1	234	4.97E-60
292	WP_078611351.1	-	cation-transporting P-type ATPase	0	0	0	0	0	1	0	0	1	1	1	4	Accessory	COG0474_P#	44.4	709	1.97E-203	NA	40.7	589	6.07E-167
293	Prokka:JAPCLKMA_04894	-	hypothetical protein	1	0	0	0	0	0	0	0	0	0	0	1	Unique	COG0737_F#	45.9	64	3.32E-09				
294	Prokka:JAPCLKMA_05906	-	hypothetical protein	1	0	0	0	1	0	0	0	0	0	1	3	Accessory								
295	Prokka:JAPCLKMA_00716	-	hypothetical protein	1	0	0	1	1	1	0	1	0	1	1	7	Accessory								
296	WP_102641520.1	-	LuxR family transcriptional regulator	1	1	1	0	1	1	1	1	1	1	1	10	Accessory	COG2771_K#	24.8	131	1.69E-29	NA	28.8	87	7.47E-16
297	OSC67421.1	-	hypothetical protein	1	1	1	1	1	1	1	1	1	1	1	11	CORE	COG0392_S#	34.2	184	2.19E-45	K07027	34.2	184	5.86E-45
298	Prokka:JAPCLKMA_05183	-	hypothetical protein	1	0	1	1	1	1	1	1	1	1	1	10	Accessory	COG5498_M#	36.5	515	5.41E-145	K01180	24.9	165	2.15E-39
299	WP_122213829.1	-	hypothetical protein	0	0	0	0	0	0	0	0	0	1	0	1	Unique								
300	WP_122215328.1	-	tetratricopeptide repeat protein	1	0	1	0	0	0	0	0	0	1	0	3	Accessory	COG3903_R#	25.1	96	4.58E-19				

cluster	GI	gene	Annotation	Streptomyces_thermocarboxydus_BPSAC147	Streptomyces_sp._4F	Streptomyces_sp._BSE7F	Streptomyces_sp._ETH9427	Streptomyces_sp._FxanaD5	Streptomyces_sp._UNC401CLCol	Actinospica_acidiphila_NRRL_B_24431	Streptomyces_sp._HNS054	Streptomyces_sp._SMS_SU21	Streptomyces_sp._ZS0098	Streptomyces_thermocarboxydus_K155	Addition	Core-Accessory-Unique	COG	%Similarity_COG	Score_COG	Evalue_COG	KEGG	%Similarity_KEGG	Score_KEGG	Evalue_KEGG
301	WP_048458873.1	-	hypothetical protein	0	0	0	0	0	0	0	1	0	0	0	1	Unique								
302	OSC61608.1	-	hypothetical protein	0	1	0	0	0	0	0	1	0	0	0	2	Accessory	COG0317_TK#	39.1	400	1.48E-110	K00951	41.1	399	6.75E-110
303	Prokka:JAPCLKMA_06641	-	hypothetical protein	1	0	0	0	1	1	1	0	0	0	1	5	Accessory	COG2771_K#	27.8	109	5.21E-23	NA	27.1	102	2.9E-20
304	Prokka:CIODPCDJ_06761	pknD_18	Serine/threonine-protein kinase PknD	0	0	0	0	0	0	0	0	0	0	1	1	Unique	COG0515_RTKL#	29.8	77	2.86E-13	NA	29.6	68	4.64E-10
305	Prokka:JAPCLKMA_06006	clpC_2	ATP-dependent Clp protease ATP-binding subunit ClpC	1	1	1	1	1	1	1	1	1	1	1	11	CORE	COG0542_O#	49.9	805	1.99E-232	K03696	50.8	759	3.34E-218
306	Prokka:CIODPCDJ_00587	-	hypothetical protein	0	1	0	0	1	1	0	0	0	0	1	4	Accessory	COG3903_R#	31.5	250	2.46E-65	NA	31.7	259	1.42E-67
307	Prokka:CIODPCDJ_05615	-	Xyloglucanase	1	1	1	1	1	1	1	1	1	1	1	11	CORE					NA	51.5	817	1.35E-235
308	Prokka:CIODPCDJ_00725	-	hypothetical protein	1	0	1	0	1	1	0	1	1	1	1	8	Accessory	COG0642_T#	29.4	260	3.1E-68	NA	29.4	260	8.29E-68
309	WP_028960155.1	-	DNA translocase FtsK	1	1	1	1	1	1	1	1	1	1	1	11	CORE	COG1674_D#	49.7	737	5.07E-212	K03466	62.5	676	4.84E-193
310	WP_033274619.1	-	ABC transporter permease	1	1	1	1	1	1	1	0	1	1	1	10	Accessory								
311	OSC70848.1	-	pyruvate, phosphate dikinase	1	1	1	1	1	1	1	1	1	1	1	11	CORE	COG0574_G#	54.9	902	1.55E-261	K01006	51.5	840	1.13E-242

cluster	GI	gene	Annotation	Streptomyces_thermocarboxydus_BPSAC147	Streptomyces_sp._4F	Streptomyces_sp._BSE7F	Streptomyces_sp._ETH9427	Streptomyces_sp._FxanaD5	Streptomyces_sp._UNC401CLCol	Actinospica_acidiphila_NRRL_B_24431	Streptomyces_sp._HNS054	Streptomyces_sp._SMS_SU21	Streptomyces_sp._ZS0098	Streptomyces_thermocarboxydus_K155	Addition	Core-Accessory-Unique	COG	%Similarity_COG	Score_COG	Evalue_COG	KEGG	%Similarity_KEGG	Score_KEGG	Evalue_KEGG
312	WP_122212504.1	-	SpoIIE family protein phosphatase	1	1	0	1	1	1	1	1	1	1	1	10	Accessory	COG2208_TK#	27.9	105	1.27E-21	K02485	24.5	82	2.36E-14
313	OSC71483.1	-	hypothetical protein	0	1	0	0	0	0	0	0	0	0	0	1	Unique								
314	OSC69075.1	-	pyruvate dehydrogenase (acetyl-transferring), homodimeric type	1	1	1	1	1	1	1	1	1	1	1	11	CORE	COG2609_C#	66.1	1218	0	K00163	66.1	1218	0
315	WP_078895235.1	-	Z1 domain-containing protein	0	0	0	0	0	0	0	1	0	0	0	1	Unique								
316	AXI91287.1	-	hypothetical protein	0	0	0	1	0	0	0	0	0	0	0	1	Unique								
317	WP_048457360.1	-	phosphoenolpyruvate carboxylase	1	1	1	1	1	1	1	1	1	1	1	11	CORE	COG2352_C#	39.1	567	1.18E-160	K01595	37.1	535	7.77E-151
318	WP_048454849.1	-	SpoIIE family protein phosphatase	1	1	1	1	1	1	1	1	1	1	1	11	CORE	COG3290_T#	30.6	154	1.39E-36	K07701	29.4	144	6.57E-33
319	Prokka:CIODPCDJ_06795	-	hypothetical protein	0	1	0	0	1	0	0	0	0	0	1	3	Accessory								
320	PWE09463.1	-	1,4-alpha-glucan branching enzyme	1	1	1	1	1	1	1	1	1	1	1	11	CORE	COG0296_G#	59.9	894	2.46E-259	K00700	59.9	894	6.56E-259

cluster	GI	gene	Annotation	Streptomyces_thermocarboxydus_BPSAC147	Streptomyces_sp._4F	Streptomyces_sp._BSE7F	Streptomyces_sp._ETH9427	Streptomyces_sp._FxanaD5	Streptomyces_sp._UNC401CLCol	Actinospica_acidiphila_NRRL_B_24431	Streptomyces_sp._HNS054	Streptomyces_sp._SMS_SU21	Streptomyces_sp._ZS0098	Streptomyces_thermocarboxydus_K155	Addition	Core-Accessory-Unique	COG	%Similarity_COG	Score_COG	Evalue_COG	KEGG	%Similarity_KEGG	Score_KEGG	Evalue_KEGG
321	Prokka:JAPCLKMA_02827	-	hypothetical protein	1	0	0	0	0	0	0	0	0	0	0	1	Unique								
322	Prokka:JAPCLKMA_05601	-	hypothetical protein	1	1	1	1	0	0	1	1	1	1	1	9	Accessory					NA	41.1	367	4.75E-100
323	WP_102640713.1	-	HAMP domain-containing protein	1	1	0	1	1	1	1	1	1	0	1	9	Accessory	COG0642_T#	28	177	2.61E-43	NA	28	177	6.96E-43
324	PWE07035.1	-	DNA polymerase I	1	1	1	1	1	1	1	1	1	1	1	11	CORE	COG0749_L#	56.4	645	3.37E-184	K02335	57.6	997	9.34E-290
325	AXI89298.1	acnA	aconitate hydratase AcnA	1	1	1	1	1	1	1	1	1	1	1	11	CORE	COG1048_C#	71.2	1361	0	K01681	71.2	1361	0
326	OSC57268.1	-	hypothetical protein	1	1	1	1	1	1	1	1	1	1	1	11	CORE	COG0658_R#	37.5	189	5.06E-47	K02238	37.5	189	1.35E-46
327	OSC69892.1	-	hypothetical protein	0	1	0	0	0	0	0	0	0	0	0	1	Unique	COG0515_RTKL#	31.8	194	1.56E-48	NA	27.1	75	3.69E-12
328	OSC73362.1	-	penicillin acylase	0	1	0	0	0	0	0	0	0	0	0	1	Unique								
329	WP_033276144.1	-	DNA-binding protein	1	1	1	1	1	1	1	1	1	1	1	11	CORE					NA	33	250	4.9E-65
330	WP_078895200.1	-	formate dehydrogenase subunit alpha	0	0	0	1	0	0	0	1	1	1	0	4	Accessory	COG3383_R#	40.4	706	1.22E-202	K00123	36.6	492	9.66E-138
331	OSC62771.1	-	hypothetical protein	0	1	0	0	0	0	0	0	0	0	0	1	Unique	COG3227_E#	30	196	4.1E-49	K08777	30	196	1.1E-48
332	Prokka:JAPCLKMA_06241	-	hypothetical protein	1	0	1	1	1	1	1	1	1	1	1	10	Accessory	COG2208_TK#	35	109	6.59E-23	NA	33	114	4.19E-24
333	AXI87307.1	-	hypothetical protein	0	0	0	1	0	0	0	1	0	1	0	3	Accessory					NA	27.8	72	2.38E-11

cluster	GI	gene	Annotation	Streptomyces_thermocarboxydus_BPSAC147	Streptomyces_sp._4F	Streptomyces_sp._BSE7F	Streptomyces_sp._ETH9427	Streptomyces_sp._FxanaD5	Streptomyces_sp._UNC401CLCol	Actinospica_acidiphila_NRRL_B_24431	Streptomyces_sp._HNS054	Streptomyces_sp._SMS_SU21	Streptomyces_sp._ZS0098	Streptomyces_thermocarboxydus_K155	Addition	Core-Accessory-Unique	COG	%Similarity_COG	Score_COG	Evalue_COG	KEGG	%Similarity_KEGG	Score_KEGG	Evalue_KEGG
334	Prokka:CIODPCDJ_00295	-	hypothetical protein	1	0	0	1	1	1	1	1	0	1	1	8	Accessory					NA	33.9	319	1.46E-85
335	Prokka:CIODPCDJ_06797	-	hypothetical protein	0	1	0	0	1	0	0	0	0	0	1	3	Accessory								
336	WP_033273939.1	-	hypothetical protein	0	1	0	1	1	1	1	1	1	1	1	9	Accessory	COG2909_K#	28.7	193	3.46E-48	K13419	28.3	186	1.48E-45
337	Prokka:CIODPCDJ_02431	-	hypothetical protein	0	0	0	0	1	1	0	0	0	0	1	3	Accessory	COG2312_R#	30.4	142	9.14E-33	K06880	29.6	108	2.99E-22
338	AXI89653.1	-	DUF3893 domain-containing protein	0	0	0	1	0	0	0	0	0	0	0	1	Unique								
339	WP_033278145.1	-	AAA family ATPase	1	1	0	0	1	1	1	1	1	1	1	9	Accessory								
340	WP_019523758.1	alaS	alanine--tRNA ligase	1	1	1	1	1	1	1	1	1	1	1	11	CORE	COG0013_J#	53.5	928	2.55E-269	K01872	53.5	928	6.82E-269
341	WP_048457506.1	-	protein kinase/lanthionine synthetase C family protein	0	0	1	1	0	0	0	1	1	1	0	5	Accessory	COG0515_RTKL#	27.5	126	3.95E-28	NA	26	82	1.75E-14
342	Prokka:JAPCLKMA_06050	lacZ_5	Beta-galactosidase	1	1	0	1	1	1	1	1	1	1	1	10	Accessory					NA	43.7	477	2.44E-133
343	PWE10955.1	-	hypothetical protein	0	0	1	1	0	0	0	1	1	1	0	5	Accessory	COG1020_Q#	31.6	133	4.22E-30	NA	31.6	133	1.13E-29
344	WP_106432485.1	-	D-alanyl-D-alanine carboxypeptidase	1	0	0	0	1	1	0	0	0	0	1	4	Accessory	COG1686_M#	27.5	88	1.56E-16	NA	29.8	101	6.2E-20
345	AXI90525.1	-	helicase	0	1	1	1	1	0	1	0	0	0	0	5	Accessory								

cluster	GI	gene	Annotation	Streptomyces_thermocarboxydus_BPSAC147	Streptomyces_sp._4F	Streptomyces_sp._BSE7F	Streptomyces_sp._ETH9427	Streptomyces_sp._FxanaD5	Streptomyces_sp._UNC401CLCol	Actinospica_acidiphila_NRRL_B_24431	Streptomyces_sp._HNS054	Streptomyces_sp._SMS_SU21	Streptomyces_sp._ZS0098	Streptomyces_thermocarboxydus_K155	Addition	Core-Accessory-Unique	COG	%Similarity_COG	Score_COG	Evalue_COG	KEGG	%Similarity_KEGG	Score_KEGG	Evalue_KEGG
346	AXI91262.1	-	ATP/GTP-binding protein	0	0	0	1	0	0	0	0	0	0	0	1	Unique								
347	Prokka:JAPCLKMA_03479	mtgA	Biosynthetic peptidoglycan transglycosylase	1	1	0	1	1	1	1	1	1	1	1	10	Accessory	COG0744_M#	33.8	307	1.63E-82	NA	34.2	306	9.68E-82
348	WP_048458606.1	-	protein kinase/lanthionine synthetase C family protein	0	1	0	1	0	0	0	1	0	0	0	3	Accessory	COG0515_RTKL#	32.7	211	1.59E-53				
349	WP_106432437.1	-	DEAD/DEAH box helicase	1	1	1	1	1	1	1	1	1	1	1	11	CORE	COG0513_LKJ#	54.8	508	4.8E-143	NA	54.8	508	1.28E-142
350	Prokka:JAPCLKMA_02024	-	hypothetical protein	1	1	1	1	1	1	1	1	1	1	1	11	CORE	COG4279_S#	36	148	9.65E-35	NA	36.8	130	7.26E-29
351	WP_019526048.1	-	hypothetical protein	0	0	0	0	1	0	0	0	0	1	0	2	Accessory								
352	OSC61225.1	-	polyketide synthase	0	1	0	0	0	0	0	0	0	0	0	1	Unique	COG3321_Q#	48	373	1.84E-102	NA	48	373	4.93E-102
353	OSC70564.1	-	hypothetical protein	0	1	0	0	0	0	0	0	0	0	0	1	Unique	COG1404_O#	31.2	99	8.76E-20	NA	32.1	98	3.06E-19
354	Prokka:JAPCLKMA_06514	btuD_21	Vitamin B12 import ATP-binding protein BtuD	1	1	1	1	1	1	1	1	1	1	1	11	CORE	COG1131_V#	38.9	196	5.25E-49	K01990	38.9	196	1.4E-48
355	AXI85379.1	-	hypothetical protein	0	0	0	1	0	0	0	0	0	0	0	1	Unique								

cluster	GI	gene	Annotation	Streptomyces_thermocarboxydus_BPSAC147	Streptomyces_sp._4F	Streptomyces_sp._BSE7F	Streptomyces_sp._ETH9427	Streptomyces_sp._FxanaD5	Streptomyces_sp._UNC401CLCol	Actinospica_acidiphila_NRRL_B_24431	Streptomyces_sp._HNS054	Streptomyces_sp._SMS_SU21	Streptomyces_sp._ZS0098	Streptomyces_thermocarboxydus_K155	Addition	Core-Accessory-Unique	COG	%Similarity_COG	Score_COG	Evalue_COG	KEGG	%Similarity_KEGG	Score_KEGG	Evalue_KEGG
356	WP_019522314.1	-	SpoIIE family protein phosphatase	1	1	1	1	1	1	1	1	1	1	1	11	CORE	COG2203_T#	26.3	59	0.0000001	K02485	25.7	80	8.57E-14
357	WP_122215843.1	-	amino acid adenylation domain-containing protein	0	0	1	1	0	0	0	1	1	1	0	5	Accessory	COG1020_Q#	39.1	341	1.32E-92	K04780	35	331	2.79E-89
358	WP_122214996.1	clpB	ATP-dependent chaperone ClpB	1	1	1	1	1	1	1	1	1	1	1	11	CORE	COG0542_O#	61.7	1043	5.38E-304	K03695	57.9	1033	1.49E-300
359	WP_122213280.1	adhE	bifunctional acetaldehyde-CoA/alcohol dehydrogenase	0	0	0	1	0	0	0	1	1	1	0	4	Accessory	COG1012_C#	61.8	567	6.63E-161	K04072	46	762	3.75E-219
360	Prokka:JAPCLKMA_05578	pepN_6	Aminopeptidase N	1	1	1	1	1	1	1	1	1	1	1	11	CORE	COG0308_E#	48.7	768	3.34E-221	K01256	48.7	768	8.91E-221
361	Prokka:JAPCLKMA_04257	-	hypothetical protein	1	1	0	1	1	1	1	1	1	1	1	10	Accessory								
362	Prokka:JAPCLKMA_00846	-	hypothetical protein	1	1	1	1	1	1	1	1	1	1	1	11	CORE								
363	Prokka:CIODPCDJ_06792	-	hypothetical protein	0	1	0	0	1	0	0	0	0	0	1	3	Accessory								
364	WP_019524109.1	-	hypothetical protein	0	0	0	0	1	0	0	0	0	0	0	1	Unique								

cluster	GI	gene	Annotation	Streptomyces_thermocarboxydus_BPSAC147	Streptomyces_sp._4F	Streptomyces_sp._BSE7F	Streptomyces_sp._ETH9427	Streptomyces_sp._FxanaD5	Streptomyces_sp._UNC401CLCol	Actinospica_acidiphila_NRRL_B_24431	Streptomyces_sp._HNS054	Streptomyces_sp._SMS_SU21	Streptomyces_sp._ZS0098	Streptomyces_thermocarboxydus_K155	Addition	Core-Accessory-Unique	COG	%Similarity_COG	Score_COG	Evalue_COG	KEGG	%Similarity_KEGG	Score_KEGG	Evalue_KEGG
365	WP_028961130.1	-	valine--tRNA ligase	1	1	1	1	1	1	1	1	1	1	1	11	CORE	COG0525_J#	60.9	1084	0	K01873	60.9	1084	0
366	OSC72174.1	-	glycogen phosphorylase	1	1	0	1	1	1	1	1	1	1	1	10	Accessory	COG0058_G#	57.8	973	6.8E-283	K00688	57.8	973	1.82E-282
367	WP_102640994.1	-	ABC transporter permease/substrate binding protein	1	1	0	1	1	1	1	1	1	1	1	10	Accessory	COG4176_E#	48.8	270	1.66E-71	K02001	48.7	263	9.26E-69
368	OSC70853.1	-	nitrite reductase (NAD(P)H)	1	1	1	1	1	1	1	1	1	1	1	11	CORE	COG1251_C#	56	925	1.62E-268	K00362	56	925	4.33E-268
369	Prokka:CIODPCDJ_04818	-	hypothetical protein	0	0	0	0	0	0	0	0	0	0	1	1	Unique								
370	Prokka:JAPCLKMA_00532	ponA1_1	Penicillin-binding protein 1A	1	1	0	1	1	1	1	1	1	1	1	10	Accessory	COG0744_M#	32.3	309	4.2E-83	NA	32.7	294	2.86E-78
371	WP_147474338.1	-	DEAD/DEAH box helicase	0	0	0	0	0	0	0	0	0	1	0	1	Unique	COG0514_L#	34.8	152	6.56E-36	K03654	31.3	148	3.31E-34
372	WP_106432453.1	-	cation-transporting P-type ATPase	1	0	0	0	1	1	0	0	1	1	1	6	Accessory	COG0474_P#	40.9	604	8.24E-172	K01537	39.8	583	3.08E-165
373	AXI86507.1	-	phosphoribosyltransferase	1	1	0	1	1	1	1	1	1	1	1	10	Accessory					NA	37.9	200	9.55E-50
374	WP_048457199.1	gyrA	DNA gyrase subunit A	1	1	1	1	1	1	1	1	1	1	1	11	CORE	COG0188_L#	65.2	1094	0	K02469	65.2	1094	0

cluster	GI	gene	Annotation	Streptomyces_thermocarboxydus_BPSAC147	Streptomyces_sp._4F	Streptomyces_sp._BSE7F	Streptomyces_sp._ETH9427	Streptomyces_sp._FxanaD5	Streptomyces_sp._UNC401CLCol	Actinospica_acidiphila_NRRL_B_24431	Streptomyces_sp._HNS054	Streptomyces_sp._SMS_SU21	Streptomyces_sp._ZS0098	Streptomyces_thermocarboxydus_K155	Addition	Core-Accessory-Unique	COG	%Similarity_COG	Score_COG	Evalue_COG	KEGG	%Similarity_KEGG	Score_KEGG	Evalue_KEGG
375	OSC72240.1	fbiC	7,8-didemethyl-8-hydroxy-5-deazariboflavin synthase	1	1	1	1	1	1	1	1	1	1	1	11	CORE	COG1060_HR#	64.7	1099	0	K11779	64.7	1099	0
376	Prokka:JAPCLKMA_04879	-	hypothetical protein	1	0	0	0	0	0	0	0	0	0	0	1	Unique								
377	AXI89195.1	-	VWA domain-containing protein	1	0	0	1	1	1	0	1	0	1	1	7	Accessory								
378	WP_033276318.1	-	DUF262 domain-containing protein	0	0	0	0	0	0	1	0	0	0	0	1	Unique	COG1479_S#	31.5	261	1.3E-68	NA	29	220	6.79E-56
379	OSC64173.1	-	ABC transporter ATP-binding protein	1	1	1	1	1	1	1	1	1	1	1	11	CORE	COG1131_V#	54.3	240	3.1E-62	NA	37.5	508	1.25E-142
380	WP_048457347.1	-	ABC transporter permease	1	1	1	1	1	1	1	1	1	1	1	11	CORE	COG0577_V#	32.9	429	3.6E-119	K02004	20.4	140	8.91E-32
381	Prokka:CIODPCDJ_02238	pepN_2	Aminopeptidase N	1	1	1	1	1	1	1	1	1	1	1	11	CORE	COG0308_E#	49.9	790	4.7E-228	K01256	49.9	790	1.25E-227
382	Prokka:JAPCLKMA_02440	-	hypothetical protein	1	0	0	1	1	1	1	1	1	1	1	9	Accessory	COG2208_TK#	33.5	117	3.02E-25	NA	33	117	4.73E-25
383	WP_019526173.1	-	phenylalanine--tRNA ligase subunit beta	1	1	0	1	1	1	1	1	1	1	1	10	Accessory	COG0072_J#	49.6	614	7.87E-175	K01890	50.5	762	3.66E-219

cluster	GI	gene	Annotation	Streptomyces_thermocarboxydus_BPSAC147	Streptomyces_sp._4F	Streptomyces_sp._BSE7F	Streptomyces_sp._ETH9427	Streptomyces_sp._FxanaD5	Streptomyces_sp._UNC401CLCol	Actinospica_acidiphila_NRRL_B_24431	Streptomyces_sp._HNS054	Streptomyces_sp._SMS_SU21	Streptomyces_sp._ZS0098	Streptomyces_thermocarboxydus_K155	Addition	Core-Accessory-Unique	COG	%Similarity_COG	Score_COG	Evalue_COG	KEGG	%Similarity_KEGG	Score_KEGG	Evalue_KEGG
384	Prokka:CIODPCDJ_02881	-	hypothetical protein	1	1	1	1	1	1	1	1	1	1	1	11	CORE	COG0642_T#	29.8	144	2.3E-33	NA	29.8	144	6.15E-33
385	Prokka:CIODPCDJ_05160	apaH_2	Bis(5'-nucleosyl)-tetraphosphatase, symmetrical	1	1	1	1	1	1	1	1	1	1	1	11	CORE	COG0639_T#	57.1	287	2.2E-76	K01090	49.6	363	6.45E-99
386	PWE11689.1	-	hypothetical protein	1	0	1	1	1	1	0	1	1	1	1	9	Accessory	COG0577_V#	34.1	434	8.52E-121	K02004	24.4	170	6.13E-41
387	PWE09772.1	-	histidine kinase	1	1	1	1	1	1	1	1	1	1	1	11	CORE	COG2205_T#	50.2	777	7E-224	K07646	50.2	776	3.19E-223
388	PWE07541.1	-	serine/threonine protein kinase	1	0	1	0	1	1	1	1	1	1	1	9	Accessory	COG0515_RTKL#	46.2	545	4.47E-154	K14949	46.2	545	1.2E-153
389	AXI91206.1	-	helicase	0	1	0	1	0	0	0	0	0	0	0	2	Accessory	COG4889_R#	31.3	67	2.73E-10				
390	WP_033272903.1	-	FtsX-like permease family protein	1	0	1	0	1	1	1	0	1	0	1	7	Accessory	COG0577_V#	26.8	228	7.06E-59	NA	28.7	65	2.76E-09
391	Prokka:JAPCLKMA_06023	-	hypothetical protein	1	0	0	0	1	1	0	0	0	0	1	4	Accessory	COG0457_R#	28.1	64	2.3E-09	NA	23.7	53	0.0000109
392	Prokka:CIODPCDJ_02440	-	hypothetical protein	0	1	0	0	0	1	0	0	0	0	1	3	Accessory								
393	PWE07596.1	-	hypothetical protein	0	0	1	0	0	0	0	0	0	1	0	2	Accessory					NA	33.3	44	0.00859
394	OSC71514.1	-	hypothetical protein	0	1	0	0	0	0	0	0	0	0	0	1	Unique	COG5635_T#	26.4	77	2.01E-13				
395	WP_086020167.1	-	DEAD/DEAH box helicase	1	1	1	1	1	1	1	1	1	1	1	11	CORE	COG4581_L#	56.1	957	2.87E-278	K03726	30.8	138	2.56E-31
396	WP_028961310.1	pepN	aminopeptidase N	0	0	0	0	1	1	0	0	0	0	0	2	Accessory	COG0308_E#	36.3	484	9.29E-136	K01256	36.3	484	2.48E-135

cluster	GI	gene	Annotation	Streptomyces_thermocarboxydus_BPSAC147	Streptomyces_sp._4F	Streptomyces_sp._BSE7F	Streptomyces_sp._ETH9427	Streptomyces_sp._FxanaD5	Streptomyces_sp._UNC401CLCol	Actinospica_acidiphila_NRRL_B_24431	Streptomyces_sp._HNS054	Streptomyces_sp._SMS_SU21	Streptomyces_sp._ZS0098	Streptomyces_thermocarboxydus_K155	Addition	Core-Accessory-Unique	COG	%Similarity_COG	Score_COG	Evalue_COG	KEGG	%Similarity_KEGG	Score_KEGG	Evalue_KEGG
397	OSC72787.1	-	GTP pyrophosphokinase	1	1	1	1	1	1	1	1	1	1	1	11	CORE	COG0317_TK#	64	1004	2.04E-292	K00951	64	1004	5.46E-292
398	OSC57597.1	-	hypothetical protein	0	1	0	0	0	0	0	0	0	0	0	1	Unique	COG0577_V#	25.1	161	1.8E-38	K09808	28.2	67	5.53E-10
399	WP_084207000.1	-	DEAD/DEAH box helicase	1	0	1	0	1	1	1	1	1	1	1	9	Accessory	COG1205_R#	52.9	757	5.68E-218	K06877	52.9	757	1.52E-217
400	WP_102640083.1	-	MFS transporter	1	1	1	1	1	1	1	1	1	1	1	11	CORE	COG0477_GEPR#	35.1	330	1.71E-89	NA	32.7	289	8.94E-77
401	OSC61651.1	-	ABC transporter permease	0	1	0	0	0	0	0	0	0	0	0	1	Unique	COG0577_V#	25.6	208	7.5E-53	K02004	33.6	48	0.000347
402	WP_102639301.1	-	ABC transporter permease	1	1	1	1	1	1	1	1	1	1	1	11	CORE	COG0577_V#	32.9	454	7.84E-127	K02004	24.5	196	7.87E-49
403	AXI84987.1	-	ABC transporter permease	1	0	1	1	1	1	1	1	1	1	1	10	Accessory	COG0577_V#	25.3	176	4.12E-43				
404	AXI86222.1	pepN	aminopeptidase N	1	1	1	1	1	1	1	1	1	1	1	11	CORE	COG0308_E#	39.2	565	3.15E-160	K01256	39.2	565	8.41E-160
405	WP_033275508.1	-	ATPase AAA	1	1	1	1	1	1	1	1	1	1	1	11	CORE	COG0515_RTKL#	30.5	44	0.00187	NA	27.3	82	2.16E-14
406	WP_019526704.1	-	ATP-dependent Clp protease ATP-binding subunit	1	1	1	1	1	1	1	1	1	1	1	11	CORE	COG0542_O#	81.6	1335	0	K03696	81.6	1334	0
407	PWE10549.1	fabD	[acyl-carrier-protein] S-malonyltransferase	0	0	1	0	0	0	0	0	1	0	0	2	Accessory	COG2070_R#	43.4	352	4.18E-96	K15329	48.9	431	1.45E-119

cluster	GI	gene	Annotation	Streptomyces_thermocarboxydus_BPSAC147	Streptomyces_sp._4F	Streptomyces_sp._BSE7F	Streptomyces_sp._ETH9427	Streptomyces_sp._FxanaD5	Streptomyces_sp._UNC401CLCol	Actinospica_acidiphila_NRRL_B_24431	Streptomyces_sp._HNS054	Streptomyces_sp._SMS_SU21	Streptomyces_sp._ZS0098	Streptomyces_thermocarboxydus_K155	Addition	Core-Accessory-Unique	COG	%Similarity_COG	Score_COG	Evalue_COG	KEGG	%Similarity_KEGG	Score_KEGG	Evalue_KEGG
408	WP_102636907.1	-	SpoIIE family protein phosphatase	1	0	1	1	1	1	1	1	1	1	1	10	Accessory	COG2208_TK#	43.1	80	2.33E-14	NA	30	82	1.64E-14
409	OSC69664.1	-	hypothetical protein	0	1	0	0	0	0	0	0	0	0	0	1	Unique								
410	WP_033272531.1	-	NADH-quinone oxidoreductase subunit G	1	1	1	1	1	1	1	1	1	1	1	11	CORE	COG1034_C#	62.2	977	3.44E-284	K00336	62.2	977	9.19E-284
411	WP_033278098.1	-	NTP transferase domain-containing protein	1	1	1	1	1	1	1	1	1	1	1	11	CORE	COG1208_MJ#	48.4	392	3.61E-108	K16881	40	644	1.41E-183
412	WP_048459338.1	-	[protein-PII] uridylyltransferase	1	1	1	1	1	1	1	1	1	1	1	11	CORE	COG2844_O#	41.2	526	1.6E-148	K00990	41.2	526	4.28E-148
413	WP_109288755.1	-	protein kinase	1	1	1	1	1	1	1	1	1	1	1	11	CORE	COG0515_RTKL#	27.6	50	0.0000336	K08286	27.6	50	0.0000898
414	WP_102638949.1	-	excinuclease ABC subunit UvrA	0	0	0	1	0	0	0	1	1	1	0	4	Accessory	COG0178_L#	41.8	590	9.02E-168	K03701	42	590	2.41E-167
415	PWE06667.1	-	alpha-N-arabinofuranosidase	0	1	1	1	0	0	1	0	0	0	1	5	Accessory	COG3534_G#	41.1	626	1.13E-178	NA	41.1	626	3.03E-178
416	WP_158688203.1	-	hypothetical protein	0	0	0	0	1	0	0	0	0	0	0	1	Unique					NA	29.8	132	2.34E-29
417	PWE10567.1	-	hypothetical protein	0	0	1	0	0	0	0	0	0	0	0	1	Unique	COG3321_Q#	37	255	6.85E-67	K12440	35.7	254	3.12E-66
418	Prokka:CIODPCDJ_04086	uvrD1	ATP-dependent DNA helicase UvrD1	1	1	1	1	1	1	1	1	1	1	1	11	CORE	COG0210_L#	59.4	856	1.14E-247	K03657	59.4	856	3.06E-247

cluster	GI	gene	Annotation	Streptomyces_thermocarboxydus_BPSAC147	Streptomyces_sp._4F	Streptomyces_sp._BSE7F	Streptomyces_sp._ETH9427	Streptomyces_sp._FxanaD5	Streptomyces_sp._UNC401CLCol	Actinospica_acidiphila_NRRL_B_24431	Streptomyces_sp._HNS054	Streptomyces_sp._SMS_SU21	Streptomyces_sp._ZS0098	Streptomyces_thermocarboxydus_K155	Addition	Core-Accessory-Unique	COG	%Similarity_COG	Score_COG	Evalue_COG	KEGG	%Similarity_KEGG	Score_KEGG	Evalue_KEGG
419	WP_028960525.1	-	hypothetical protein	0	0	0	0	0	1	0	0	0	0	0	1	Unique								
420	AXI89524.1	-	nitrate reductase	1	1	0	1	1	1	1	1	0	0	1	8	Accessory	COG0243_C#	37.5	370	1.9E-101	K00372	32	347	4.6E-94
421	WP_019521454.1	uvrA	excinuclease ABC subunit UvrA	1	1	0	0	1	1	0	0	1	1	1	7	Accessory	COG0178_L#	61.9	1002	7.54E-292	K03701	61.9	1002	2.01E-291
422	WP_019521653.1	-	AAA family ATPase	0	0	1	0	1	1	1	0	0	0	0	4	Accessory								
423	WP_033273537.1	-	hypothetical protein	0	0	0	0	1	1	1	0	0	0	1	4	Accessory	COG3533_S#	25.3	83	3.55E-15	NA	38.9	460	3.72E-128
424	OSC69151.1	-	hypothetical protein	0	1	0	0	0	0	0	0	0	0	0	1	Unique	COG3321_Q#	46.3	537	1.17E-151	K12436	46.3	537	3.13E-151
425	AXI91324.1	-	hypothetical protein	0	0	0	1	0	0	0	0	0	0	0	1	Unique	COG4695_S#	27.7	108	1.03E-22	NA	36.8	50	0.000068
426	Prokka:JAPCLKMA_00335	-	hypothetical protein	1	0	0	0	0	0	0	0	0	0	1	2	Accessory								
427	OSC72130.1	-	DNA topoisomerase IV	1	1	1	1	1	1	1	1	1	1	1	11	CORE	COG0188_L#	41.5	547	6.61E-155	K02469	41.3	537	2.38E-151
428	PWE11542.1	-	hypothetical protein	0	0	1	1	0	0	0	1	1	1	0	5	Accessory	COG2234_R#	37.8	209	5.56E-53	NA	23.2	143	7.66E-33
429	WP_037778575.1	-	MMPL family transporter	1	1	1	1	1	1	1	1	1	1	1	11	CORE	COG2409_R#	34.7	427	1.3E-118	K11625	34.7	427	3.47E-118
430	WP_122216046.1	-	hypothetical protein	0	0	1	1	0	0	0	1	1	1	0	5	Accessory	COG3321_Q#	30	160	2.96E-38	NA	30	160	7.9E-38
431	WP_028958996.1	-	SpoIIE family protein phosphatase	1	1	1	1	1	1	1	1	1	1	1	11	CORE	COG2208_TK#	33.9	111	1.21E-23	NA	32.3	128	2.55E-28

cluster	GI	gene	Annotation	Streptomyces_thermocarboxydus_BPSAC147	Streptomyces_sp._4F	Streptomyces_sp._BSE7F	Streptomyces_sp._ETH9427	Streptomyces_sp._FxanaD5	Streptomyces_sp._UNC401CLCol	Actinospica_acidiphila_NRRL_B_24431	Streptomyces_sp._HNS054	Streptomyces_sp._SMS_SU21	Streptomyces_sp._ZS0098	Streptomyces_thermocarboxydus_K155	Addition	Core-Accessory-Unique	COG	%Similarity_COG	Score_COG	Evalue_COG	KEGG	%Similarity_KEGG	Score_KEGG	Evalue_KEGG
432	OSC73035.1	-	magnesium-transporting ATPase	1	1	0	1	1	1	1	1	1	1	1	10	Accessory	COG0474_P#	57.6	851	2.78E-246	K12952	57.6	851	7.43E-246
433	AXI85397.1	-	sporulation protein	1	1	0	1	1	1	1	1	1	1	1	10	Accessory	COG0464_O#	42.6	218	6.99E-56	K06413	41.5	221	3.76E-56
434	WP_102638054.1	-	AAA family ATPase	0	0	0	0	0	0	0	0	1	0	0	1	Unique								
435	WP_102640471.1	-	bifunctional SulP family inorganic anion transporter/carbonic anhydrase	1	1	1	1	1	1	1	1	1	1	1	11	CORE	COG0659_P#	46.8	406	1.81E-112	K01673	43.5	393	5.52E-108
436	OSC65375.1	-	galactose oxidase	0	1	0	0	0	0	0	0	0	0	0	1	Unique					NA	28.4	148	4.04E-34
437	Prokka:CIODPCDJ_01517	cdhA	Caffeine dehydrogenase subunit alpha	1	0	0	0	1	1	0	0	0	0	1	4	Accessory	COG1529_C#	36.7	429	2.6E-119	K03520	37.4	466	6.7E-130
438	AXI89687.1	-	MFS transporter	1	1	1	1	1	1	1	1	1	1	1	11	CORE	COG0477_GEPR#	43.9	461	8.06E-129	NA	43.5	421	1.89E-116
439	OSC52321.1	-	non-ribosomal peptide synthetase	0	1	0	0	0	0	0	0	0	0	0	1	Unique	COG1020_Q#	38	427	1.29E-118	K04789	35.4	382	7.45E-105
440	Prokka:CIODPCDJ_05822	yhgF	Protein YhgF	1	1	1	1	1	1	1	1	1	1	1	11	CORE	COG2183_K#	63.9	934	1.9E-271	K06959	60.1	875	3.66E-253
441	OSC66854.1	-	aminopeptidase N	0	1	0	0	0	0	0	0	0	0	0	1	Unique	COG0308_E#	35.5	453	2.19E-126	K01256	35.5	453	5.86E-126

cluster	GI	gene	Annotation	Streptomyces_thermocarboxydus_BPSAC147	Streptomyces_sp._4F	Streptomyces_sp._BSE7F	Streptomyces_sp._ETH9427	Streptomyces_sp._FxanaD5	Streptomyces_sp._UNC401CLCol	Actinospica_acidiphila_NRRL_B_24431	Streptomyces_sp._HNS054	Streptomyces_sp._SMS_SU21	Streptomyces_sp._ZS0098	Streptomyces_thermocarboxydus_K155	Addition	Core-Accessory-Unique	COG	%Similarity_COG	Score_COG	Evalue_COG	KEGG	%Similarity_KEGG	Score_KEGG	Evalue_KEGG
442	Prokka:JAPCLKMA_05590	-	hypothetical protein	1	0	0	0	0	0	0	0	0	0	0	1	Unique	COG0577_V#	20.2	89	4.86E-17	K02004	24	74	5.65E-12
443	WP_102637463.1	-	ThuA domain-containing protein	1	1	0	1	1	1	1	1	1	1	1	10	Accessory	COG3828_S#	26.8	74	2.11E-12				
444	Prokka:CIODPCDJ_00410	-	hypothetical protein	1	1	1	1	1	1	1	1	1	1	1	11	CORE	COG2208_TK#	34.9	98	1.05E-19	NA	31.4	107	4.61E-22
445	WP_102638549.1	-	DEAD/DEAH box helicase family protein	0	0	0	0	0	0	0	0	1	0	0	1	Unique	COG4889_R#	28.9	186	2.92E-46	K13179	26.6	53	0.0000135
446	AXI85204.1	-	phosphoketolase	1	1	0	1	1	1	1	1	1	1	1	10	Accessory	COG3957_G#	63.6	1094	0	K01621	56.9	968	3.16E-281
447	OSC64140.1	-	endopeptidase La	1	1	1	1	1	1	1	1	1	1	1	11	CORE	COG0466_O#	44.7	619	2.3E-176	K01338	44.7	619	6.15E-176
448	OSC61245.1	-	beta-mannosidase	0	1	0	0	0	0	0	0	0	0	0	1	Unique	COG3250_G#	35.8	404	8.91E-112	K01192	32.1	397	2.91E-109
449	OSC71978.1	-	penicillin amidase	0	1	0	0	0	0	0	0	0	0	0	1	Unique	COG2366_R#	41.1	516	2.1E-145	K07116	41.1	516	5.61E-145
450	OSC65367.1	-	xanthine dehydrogenase molybdopterin binding subunit	0	1	0	0	0	0	0	0	0	0	0	1	Unique	COG4631_F#	69.3	1104	0	K13482	69.3	1104	0
451	WP_033273259.1	-	hypothetical protein	1	1	1	1	1	1	1	1	1	1	1	11	CORE								
452	OSC64152.1	-	hypothetical protein	1	1	1	1	1	1	1	1	1	1	1	11	CORE								

cluster	GI	gene	Annotation	Streptomyces_thermocarboxydus_BPSAC147	Streptomyces_sp._4F	Streptomyces_sp._BSE7F	Streptomyces_sp._ETH9427	Streptomyces_sp._FxanaD5	Streptomyces_sp._UNC401CLCol	Actinospica_acidiphila_NRRL_B_24431	Streptomyces_sp._HNS054	Streptomyces_sp._SMS_SU21	Streptomyces_sp._ZS0098	Streptomyces_thermocarboxydus_K155	Addition	Core-Accessory-Unique	COG	%Similarity_COG	Score_COG	Evalue_COG	KEGG	%Similarity_KEGG	Score_KEGG	Evalue_KEGG
453	WP_026244161.1	-	sodium-translocating pyrophosphatase	1	1	1	1	1	1	1	1	1	1	1	11	CORE	COG3808_C#	45.4	585	3.66E-166	K15987	39.3	505	7.55E-142
454	Prokka:JAPCLKMA_05758	-	hypothetical protein	1	1	1	1	1	1	1	1	1	1	1	11	CORE	COG0265_O#	44.8	291	1.09E-77	K08372	38.6	286	9.4E-76
455	Prokka:CIODPCDJ_00043	-	putative glycosyl hydrolase	1	1	1	1	1	1	1	1	1	1	1	11	CORE	COG1554_G#	51.1	756	1.2E-217	K01194	51.1	756	3.2E-217
456	WP_033272386.1	-	hypothetical protein	1	1	1	0	1	1	1	1	1	1	1	10	Accessory					NA	40.7	533	2.58E-150
457	OSC65756.1	-	ATP-dependent DNA helicase	1	1	1	1	1	1	1	1	1	1	1	11	CORE	COG0210_L#	52.1	680	8.35E-195	K03657	52.1	680	2.23E-194
458	OSC66315.1	-	hyaluronidase	0	1	0	0	0	0	0	0	0	0	0	1	Unique					K01197	30	250	5.69E-65
459	WP_051724704.1	-	FtsX family ABC transporter permease	0	0	0	0	0	0	1	0	0	0	0	1	Unique	COG0577_V#	23.6	133	2.91E-30	K02004	21.2	64	4.43E-09
460	WP_019521959.1	-	transglutaminase domain-containing protein	1	1	1	1	1	1	1	1	1	1	1	11	CORE	COG1305_E#	24.7	159	4.93E-38	NA	31.1	80	7.77E-14
461	WP_033274658.1	-	ATP-dependent RecD-like DNA helicase	1	1	1	1	0	0	1	1	1	1	1	9	Accessory	COG0507_L#	46.3	603	9.88E-172	K03581	38.5	523	2.66E-147
462	WP_157842202.1	-	tetratricopeptide repeat protein	0	0	0	0	0	0	1	0	0	0	0	1	Unique	COG0457_R#	22.7	132	8.43E-30	NA	26.6	91	3.37E-17

cluster	GI	gene	Annotation	Streptomyces_thermocarboxydus_BPSAC147	Streptomyces_sp._4F	Streptomyces_sp._BSE7F	Streptomyces_sp._ETH9427	Streptomyces_sp._FxanaD5	Streptomyces_sp._UNC401CLCol	Actinospica_acidiphila_NRRL_B_24431	Streptomyces_sp._HNS054	Streptomyces_sp._SMS_SU21	Streptomyces_sp._ZS0098	Streptomyces_thermocarboxydus_K155	Addition	Core-Accessory-Unique	COG	%Similarity_COG	Score_COG	Evalue_COG	KEGG	%Similarity_KEGG	Score_KEGG	Evalue_KEGG
463	OSC62519.1	-	ABC transporter permease	0	1	0	0	0	0	0	0	0	0	0	1	Unique	COG0577_V#	24.4	154	1.59E-36	K09808	25.3	56	0.00000157
464	WP_122216699.1	-	DEAD/DEAH box helicase family protein	0	0	0	0	0	0	0	0	0	1	0	1	Unique	COG4889_R#	29.3	81	1.7E-14				
465	PWE10356.1	-	daunorubicin resistance protein DrrC	1	1	1	1	1	1	1	1	1	1	1	11	CORE	COG0178_L#	71.2	1109	0	NA	46.2	620	2.72E-176
466	Prokka:JAPCLKMA_06819	alaS_2	Alanine--tRNA ligase	1	0	0	0	0	0	0	0	0	0	0	1	Unique	COG0013_J#	50.4	773	5.51E-223	K01872	50.2	763	1.52E-219
467	OSC73122.1	-	RNA degradosome polyphosphate kinase	1	1	1	1	1	1	1	1	1	1	1	11	CORE	COG0855_P#	59.6	807	5.87E-233	K00937	59.7	807	1.57E-232
468	OSC57005.1	-	malto-oligosyltrehalose synthase	1	1	0	1	1	1	1	1	1	1	1	10	Accessory	COG3280_G#	41.6	511	6.65E-144	K06044	41.6	511	1.78E-143
469	WP_019526595.1	-	molybdopterin-dependent oxidoreductase	1	1	1	1	1	1	1	1	1	1	1	11	CORE	COG1529_C#	34.2	396	2.39E-109	K00087	34	394	3.16E-108
470	OSC64416.1	-	glycosyl hydrolase	1	1	1	1	1	1	1	1	1	1	1	11	CORE	COG1472_G#	38	490	9.29E-138	K05349	35.2	403	6.8E-111

cluster	GI	gene	Annotation	Streptomyces_thermocarboxydus_BPSAC147	Streptomyces_sp._4F	Streptomyces_sp._BSE7F	Streptomyces_sp._ETH9427	Streptomyces_sp._FxanaD5	Streptomyces_sp._UNC401CLCol	Actinospica_acidiphila_NRRL_B_24431	Streptomyces_sp._HNS054	Streptomyces_sp._SMS_SU21	Streptomyces_sp._ZS0098	Streptomyces_thermocarboxydus_K155	Addition	Core-Accessory-Unique	COG	%Similarity_COG	Score_COG	Evalue_COG	KEGG	%Similarity_KEGG	Score_KEGG	Evalue_KEGG
471	Prokka:CIODPCDJ_04496	nrdA	Ribonucleoside-diphosphate reductase 1 subunit alpha	1	1	1	1	1	1	1	1	1	1	1	11	CORE	COG0209_F#	40.4	570	1.2E-161	K10807	41.3	576	3.44E-163
472	AXI90132.1	-	PAS domain S-box protein	1	1	1	1	1	1	1	1	1	1	1	11	CORE	COG2208_TK#	34.6	131	1.87E-29	NA	31	146	1.15E-33
473	WP_051724435.1	-	bifunctional salicylyl-CoA 5-hydroxylase/oxidoreductase	1	1	1	1	1	1	1	1	1	1	1	11	CORE	COG1902_C#	64.2	461	4.62E-129	NA	48.5	314	3.18E-84
474	OSC62710.1	-	GGDEF-domain containing protein	1	1	1	1	1	1	1	1	1	1	1	11	CORE	COG5001_T#	41.5	283	1.72E-75	NA	42	301	2.78E-80
475	OSC70489.1	-	chitinase	0	1	0	0	0	0	0	0	0	0	0	1	Unique	COG3325_G#	64.9	647	5.92E-185	NA	28	130	8.49E-29
476	Prokka:CIODPCDJ_04925	-	hypothetical protein	0	1	0	0	0	0	0	0	0	0	1	2	Accessory	COG0455_D#	26.2	78	1.43E-13	NA	25.2	198	2.54E-49
477	Prokka:CIODPCDJ_06270	cutL	Carbon monoxide dehydrogenase large chain	1	0	0	0	1	1	1	0	0	0	1	5	Accessory	COG1529_C#	51.9	760	8.17E-219	K03520	51.1	782	5.36E-225
478	WP_102637288.1	cadA	cadmium-translocating P-type ATPase	0	0	0	1	0	0	1	1	1	1	0	5	Accessory	COG2217_P#	47.7	621	4.52E-177	NA	39.2	385	1.46E-105

cluster	GI	gene	Annotation	Streptomyces_thermocarboxydus_BPSAC147	Streptomyces_sp._4F	Streptomyces_sp._BSE7F	Streptomyces_sp._ETH9427	Streptomyces_sp._FxanaD5	Streptomyces_sp._UNC401CLCol	Actinospica_acidiphila_NRRL_B_24431	Streptomyces_sp._HNS054	Streptomyces_sp._SMS_SU21	Streptomyces_sp._ZS0098	Streptomyces_thermocarboxydus_K155	Addition	Core-Accessory-Unique	COG	%Similarity_COG	Score_COG	Evalue_COG	KEGG	%Similarity_KEGG	Score_KEGG	Evalue_KEGG
479	WP_033276553.1	-	PQQ-binding-like beta-propeller repeat protein	1	1	0	1	1	1	1	1	1	1	1	10	Accessory	COG0515_RTKL#	35.1	173	3.25E-42	NA	32.5	145	2.53E-33
480	WP_048458793.1	-	glycoside hydrolase family 2	1	0	0	1	0	1	1	1	0	1	1	7	Accessory					NA	43.3	508	1.14E-142
481	OSC71488.1	-	hypothetical protein	0	1	0	0	0	0	0	0	0	0	0	1	Unique	COG0457_R#	32.5	58	1.52E-07	NA	32.5	58	4.07E-07
482	PWE10740.1	-	glycoside hydrolase family 65 protein	0	1	1	1	0	0	1	1	1	1	0	7	Accessory	COG1554_G#	59.6	899	1.12E-260	NA	59.6	899	2.99E-260
483	PWE12051.1	-	PAS sensor protein	1	1	1	1	1	0	1	1	1	1	1	10	Accessory	COG2208_TK#	34.6	123	2.95E-27	NA	29.5	142	2.14E-32
484	WP_028959163.1	-	M6 family metalloprotease domain-containing protein	1	1	1	1	1	1	1	1	1	1	1	11	CORE	COG4412_S#	31.9	359	2.44E-98	K09607	31.9	359	6.53E-98
485	WP_019524108.1	-	UvrD-helicase domain-containing protein	0	0	0	1	1	0	0	0	0	0	0	2	Accessory	COG0210_L#	30.4	326	2.29E-88				
486	WP_109288775.1	-	AAA family ATPase	1	1	1	1	1	1	1	1	1	1	1	11	CORE	COG3973_R#	32.9	321	7.37E-87	K03657	39.7	109	1.54E-22
487	PWE09672.1	-	hypothetical protein	0	0	1	0	0	0	1	0	1	0	0	3	Accessory								
488	WP_102641640.1	-	ABC transporter permease	1	1	1	1	1	1	1	1	1	1	1	11	CORE								

cluster	GI	gene	Annotation	Streptomyces_thermocarboxydus_BPSAC147	Streptomyces_sp._4F	Streptomyces_sp._BSE7F	Streptomyces_sp._ETH9427	Streptomyces_sp._FxanaD5	Streptomyces_sp._UNC401CLCol	Actinospica_acidiphila_NRRL_B_24431	Streptomyces_sp._HNS054	Streptomyces_sp._SMS_SU21	Streptomyces_sp._ZS0098	Streptomyces_thermocarboxydus_K155	Addition	Core-Accessory-Unique	COG	%Similarity_COG	Score_COG	Evalue_COG	KEGG	%Similarity_KEGG	Score_KEGG	Evalue_KEGG
489	OSC61545.1	-	hypothetical protein	0	1	0	0	0	0	0	0	0	0	0	1	Unique	COG0553_KL#	32	324	1.48E-87	NA	26.9	63	1.26E-08
490	OSC68789.1	-	alkaline phosphatase	0	1	0	0	0	0	0	0	0	0	0	1	Unique					NA	34.1	75	2.45E-12
491	OSC56067.1	-	protein translocase subunit SecD	1	1	1	1	1	1	1	1	1	1	1	11	CORE	COG0342_U#	41.5	313	2.61E-84	K12257	35.3	389	9.99E-107
492	Prokka:JAPCLKMA_04778	hypF	Carbamoyltransferase HypF	1	0	0	1	1	1	0	1	0	1	1	7	Accessory	COG0068_O#	41.9	539	2.23E-152	K04656	41.9	539	5.96E-152
493	WP_033277716.1	-	TOMM precursor leader peptide-binding protein	0	0	0	0	1	1	1	0	0	0	1	4	Accessory								
494	WP_048456160.1	-	hypothetical protein	1	0	0	1	1	1	0	1	0	1	1	7	Accessory					NA	31.9	84	5.25E-15
495	Prokka:CIODPCDJ_03186	-	hypothetical protein	1	1	0	0	1	1	1	1	1	0	1	8	Accessory	COG1807_M#	39.2	204	1.7E-51	NA	39.2	204	4.54E-51
496	PWE08566.1	-	ATP-binding protein	0	0	1	0	0	0	0	0	0	0	0	1	Unique					NA	28.7	53	0.00000991
497	WP_048460389.1	-	DEAD/DEAH box helicase	0	1	1	1	1	1	1	1	1	1	1	10	Accessory	COG0513_LKJ#	47.5	382	2.66E-105	K05592	33.2	280	6.49E-74
498	WP_102638607.1	murJ	murein biosynthesis integral membrane protein MurJ	1	1	0	1	1	1	1	1	1	1	1	10	Accessory	COG0728_R#	31	253	1.87E-66	K03980	30.6	235	1.4E-60

cluster	GI	gene	Annotation	Streptomyces_thermocarboxydus_BPSAC147	Streptomyces_sp._4F	Streptomyces_sp._BSE7F	Streptomyces_sp._ETH9427	Streptomyces_sp._FxanaD5	Streptomyces_sp._UNC401CLCol	Actinospica_acidiphila_NRRL_B_24431	Streptomyces_sp._HNS054	Streptomyces_sp._SMS_SU21	Streptomyces_sp._ZS0098	Streptomyces_thermocarboxydus_K155	Addition	Core-Accessory-Unique	COG	%Similarity_COG	Score_COG	Evalue_COG	KEGG	%Similarity_KEGG	Score_KEGG	Evalue_KEGG
499	OSC65147.1	-	helix-turn-helix transcriptional regulator	0	1	0	0	0	0	0	0	0	0	0	1	Unique								
500	WP_048460594.1	-	lantibiotic dehydratase	0	0	0	1	0	0	0	1	0	0	0	2	Accessory								
501	WP_019523058.1	-	FdhF/YdeP family oxidoreductase	1	1	1	1	1	1	1	1	1	1	1	11	CORE	COG0243_C#	55.9	856	6.27E-248	NA	55.9	856	1.68E-247
502	WP_033275432.1	metE	5-methyltetrahydropteroyltriglutamate--homocysteine S-methyltransferase	0	0	0	0	1	1	1	0	0	0	1	4	Accessory	COG0620_E#	58.8	864	2.3E-250	K00549	58.8	864	6.15E-250
503	OSC56622.1	-	oxidoreductase	1	1	0	1	1	1	1	1	1	1	1	10	Accessory	COG1529_C#	32.1	327	1.01E-88	K00087	32.1	327	2.71E-88
504	OSC61744.1	-	hypothetical protein	0	1	0	0	0	0	0	0	0	0	0	1	Unique	COG0210_L#	30.7	250	2.69E-65	K03657	34.5	54	0.00000578
505	WP_019523027.1	-	M20/M25/M40 family metallo-hydrolase	1	0	0	0	1	1	1	0	0	0	1	5	Accessory	COG0624_E#	39.3	218	6.62E-56	K01295	42.1	208	2.39E-52
506	WP_019523037.1	-	xanthine dehydrogenase family protein molybdopterin-binding subunit	1	0	0	0	1	1	0	0	0	0	1	4	Accessory	COG1529_C#	32.7	280	2.41E-74	K07303	28.9	155	1.83E-36

cluster	GI	gene	Annotation	Streptomyces_thermocarboxydus_BPSAC147	Streptomyces_sp._4F	Streptomyces_sp._BSE7F	Streptomyces_sp._ETH9427	Streptomyces_sp._FxanaD5	Streptomyces_sp._UNC401CLCol	Actinospica_acidiphila_NRRL_B_24431	Streptomyces_sp._HNS054	Streptomyces_sp._SMS_SU21	Streptomyces_sp._ZS0098	Streptomyces_thermocarboxydus_K155	Addition	Core-Accessory-Unique	COG	%Similarity_COG	Score_COG	Evalue_COG	KEGG	%Similarity_KEGG	Score_KEGG	Evalue_KEGG
507	WP_122214071.1	-	hypothetical protein	1	0	1	1	0	1	1	1	1	1	1	9	Accessory	COG0455_D#	31.2	112	8.71E-24	NA	33	170	5.51E-41
508	WP_102640678.1	-	penicillin-binding protein	1	1	1	1	1	1	1	1	1	1	1	11	CORE	COG0744_M#	33	316	3.04E-85	NA	33	316	8.12E-85
509	WP_019524947.1	-	TniQ family protein	0	0	0	0	1	0	0	0	0	0	0	1	Unique								
510	Prokka:JAPCLKMA_05497	lagD_1	Lactococcin-G-processing and transport ATP-binding protein LagD	1	0	0	0	0	0	0	0	0	0	0	1	Unique	COG2274_V#	29.4	319	3.58E-86	K06147	28.2	272	1.34E-71
511	WP_078611532.1	-	CRISPR-associated endonuclease Cas3''	0	0	0	1	0	1	0	0	0	0	0	2	Accessory	COG1203_R#	29.1	195	7.79E-49				
512	WP_048460300.1	-	PQQ-binding-like beta-propeller repeat protein	0	0	0	0	0	0	0	1	0	0	0	1	Unique								
513	PWE09669.1	-	hypothetical protein	0	0	1	0	0	0	0	0	0	0	0	1	Unique								
514	WP_033273359.1	-	DNA polymerase III subunit gamma and tau	1	1	0	1	1	1	1	1	1	1	1	10	Accessory	COG2812_L#	60.3	532	2.69E-150	K02343	60.3	532	7.17E-150
515	WP_028961469.1	-	hypothetical protein	1	0	1	1	1	1	1	1	0	1	1	9	Accessory					NA	29.9	94	4.99E-18

cluster	GI	gene	Annotation	Streptomyces_thermocarboxydus_BPSAC147	Streptomyces_sp._4F	Streptomyces_sp._BSE7F	Streptomyces_sp._ETH9427	Streptomyces_sp._FxanaD5	Streptomyces_sp._UNC401CLCol	Actinospica_acidiphila_NRRL_B_24431	Streptomyces_sp._HNS054	Streptomyces_sp._SMS_SU21	Streptomyces_sp._ZS0098	Streptomyces_thermocarboxydus_K155	Addition	Core-Accessory-Unique	COG	%Similarity_COG	Score_COG	Evalue_COG	KEGG	%Similarity_KEGG	Score_KEGG	Evalue_KEGG
516	PWE07943.1	-	PTS lactose transporter subunit IIC	1	1	1	1	1	1	1	1	1	1	1	11	CORE	COG1299_G#	53.9	381	7.65E-105	K02770	49.5	425	9.44E-118
517	WP_106959481.1	-	tetratricopeptide repeat protein	0	0	0	0	0	1	0	0	0	0	0	1	Unique	COG0457_R#	25.6	75	8.98E-13	NA	24.4	66	1.45E-09
518	OSC68733.1	-	sarcosine dehydrogenase	0	1	0	0	0	0	0	0	0	0	0	1	Unique	COG0404_E#	36.3	253	3.13E-66	K00315	30.6	344	2.11E-93
519	WP_019521398.1	cadA	cadmium-translocating P-type ATPase	1	1	1	1	1	1	1	1	1	1	1	11	CORE	COG2217_P#	47.9	669	1.4E-191	K12949	47.6	614	1.09E-174
520	PWE09191.1	-	penicillin-binding protein	1	1	1	1	1	1	1	1	1	1	1	11	CORE	COG0744_M#	34	321	9.37E-87	NA	34.5	311	2.59E-83
521	PWE10989.1	-	hypothetical protein	1	1	1	1	1	1	1	1	1	1	1	11	CORE	COG2409_R#	41.9	551	4.26E-156	K11625	41.6	551	1.14E-155
522	WP_019527491.1	-	FtsX-like permease family protein	1	1	1	1	1	1	1	1	1	1	1	11	CORE	COG4591_M#	26.9	52	0.00000809				
523	WP_033274783.1	-	(Fe-S)-binding protein	1	1	1	1	1	1	1	1	1	1	1	11	CORE	COG0247_C#	48.2	705	1.76E-202	NA	49.1	698	7.51E-200
524	OSC61030.1	-	catalase HPII	1	1	1	1	1	1	1	1	1	1	1	11	CORE	COG0753_P#	50.3	663	1.31E-189	K03781	49.3	640	2.43E-182
525	WP_048458564.1	-	DEAD/DEAH box helicase family protein	0	0	0	0	0	0	0	1	0	0	0	1	Unique	COG4889_R#	30.8	219	5E-56	K13179	30.3	47	0.000908
526	WP_122213623.1	-	AAA family ATPase	1	1	1	1	1	1	1	1	1	1	1	11	CORE	COG3973_R#	42.9	530	7.75E-150	K03657	28.7	119	1.88E-25

cluster	GI	gene	Annotation	Streptomyces_thermocarboxydus_BPSAC147	Streptomyces_sp._4F	Streptomyces_sp._BSE7F	Streptomyces_sp._ETH9427	Streptomyces_sp._FxanaD5	Streptomyces_sp._UNC401CLCol	Actinospica_acidiphila_NRRL_B_24431	Streptomyces_sp._HNS054	Streptomyces_sp._SMS_SU21	Streptomyces_sp._ZS0098	Streptomyces_thermocarboxydus_K155	Addition	Core-Accessory-Unique	COG	%Similarity_COG	Score_COG	Evalue_COG	KEGG	%Similarity_KEGG	Score_KEGG	Evalue_KEGG
527	AXI88764.1	-	hypothetical protein	0	0	0	1	0	0	0	0	0	0	0	1	Unique								
528	Prokka:CIODPCDJ_06752	pknD_17	Serine/threonine-protein kinase PknD	0	0	0	0	0	0	0	0	0	0	1	1	Unique	COG0515_RTKL#	29.8	69	8.35E-11	NA	23.2	72	2.63E-11
529	WP_028961026.1	yicI	alpha-xylosidase	1	1	1	1	1	1	1	1	1	1	1	11	CORE	COG1501_G#	55	872	8.31E-253	K01811	54.8	863	1.76E-249
530	WP_078894919.1	-	carboxylesterase family protein	0	0	0	1	0	0	0	1	1	1	0	4	Accessory	COG2272_I#	38.1	267	1.6E-70	K03929	33.9	245	1.74E-63
531	WP_048458812.1	pflB	formate C-acetyltransferase	0	0	0	1	0	0	0	1	1	1	0	4	Accessory	COG1882_C#	63.3	970	3.82E-282	K00656	61.6	928	4.45E-269
532	WP_159020934.1	-	hypothetical protein	0	0	0	1	0	0	0	1	0	0	0	2	Accessory								
533	WP_122213828.1	-	hypothetical protein	0	0	0	0	0	0	0	0	0	1	0	1	Unique	COG5280_S#	24.7	86	3.86E-16	NA	24.7	87	6.04E-16
534	OSC70996.1	-	glycosyl transferase	1	1	1	1	1	1	1	1	1	1	1	11	CORE	COG1887_M#	27.9	131	1.37E-29	K19427	30.3	111	5.1E-23
535	WP_158688224.1	-	hypothetical protein	0	0	0	0	1	0	0	0	0	0	0	1	Unique								
536	WP_048459112.1	-	molybdopterin-dependent oxidoreductase	1	1	1	1	1	1	1	1	1	1	1	11	CORE	COG0243_C#	44.2	557	1E-157	NA	29.3	195	2.68E-48
537	OSC66500.1	-	PAS domain S-box protein	0	1	0	0	0	0	0	0	0	0	0	1	Unique	COG2208_TK#	28	74	2.58E-12	K07682	27.7	53	0.0000126
538	OSC71341.1	-	hypothetical protein	1	1	0	1	1	1	1	1	1	1	1	10	Accessory	COG2409_R#	33	325	4.91E-88	K06994	33	325	1.31E-87

cluster	GI	gene	Annotation	Streptomyces_thermocarboxydus_BPSAC147	Streptomyces_sp._4F	Streptomyces_sp._BSE7F	Streptomyces_sp._ETH9427	Streptomyces_sp._FxanaD5	Streptomyces_sp._UNC401CLCol	Actinospica_acidiphila_NRRL_B_24431	Streptomyces_sp._HNS054	Streptomyces_sp._SMS_SU21	Streptomyces_sp._ZS0098	Streptomyces_thermocarboxydus_K155	Addition	Core-Accessory-Unique	COG	%Similarity_COG	Score_COG	Evalue_COG	KEGG	%Similarity_KEGG	Score_KEGG	Evalue_KEGG
539	WP_122216601.1	-	xanthine dehydrogenase family protein molybdopterin-binding subunit	0	0	0	0	0	0	0	0	0	1	0	1	Unique	COG1529_C#	38.8	485	4.84E-136	K11177	39.7	452	9.28E-126
540	PWE06614.1	-	hypothetical protein	0	0	1	0	0	0	0	0	0	0	0	1	Unique								
541	Prokka:JAPCLKMA_05094	purL	Phosphoribosylformylglycinamidine synthase subunit PurL	1	1	1	1	1	1	1	1	1	1	1	11	CORE	COG0046_F#	70.1	1053	4.45E-307	K01952	70.1	1053	1.19E-306
542	WP_019521638.1	-	AAA family ATPase	1	0	1	0	1	1	0	1	0	0	0	5	Accessory	COG0210_L#	31.4	208	8.73E-53				
543	OSC63812.1	-	hypothetical protein	1	1	1	1	1	1	1	1	1	1	1	11	CORE	COG1289_S#	27.6	196	2.63E-49				
544	OSC72728.1	-	daunorubicin resistance protein DrrC	0	1	0	0	0	0	0	0	0	0	0	1	Unique	COG0178_L#	51.1	794	2.85E-229	NA	50.4	756	2.3E-217
545	AXI91266.1	-	transcriptional regulator	0	1	1	1	0	0	1	1	0	0	0	5	Accessory								
546	WP_019527138.1	-	hypothetical protein	0	0	0	0	1	0	0	0	0	0	0	1	Unique								
547	WP_102638739.1	glgX	glycogen debranching protein GlgX	1	1	1	1	1	1	1	1	1	1	1	11	CORE	COG1523_G#	55	786	1.01E-226	K02438	55	786	2.7E-226
548	OSC70809.1	-	hypothetical protein	0	1	0	0	0	0	0	0	1	0	0	2	Accessory								

cluster	GI	gene	Annotation	Streptomyces_thermocarboxydus_BPSAC147	Streptomyces_sp._4F	Streptomyces_sp._BSE7F	Streptomyces_sp._ETH9427	Streptomyces_sp._FxanaD5	Streptomyces_sp._UNC401CLCol	Actinospica_acidiphila_NRRL_B_24431	Streptomyces_sp._HNS054	Streptomyces_sp._SMS_SU21	Streptomyces_sp._ZS0098	Streptomyces_thermocarboxydus_K155	Addition	Core-Accessory-Unique	COG	%Similarity_COG	Score_COG	Evalue_COG	KEGG	%Similarity_KEGG	Score_KEGG	Evalue_KEGG
549	WP_028958786.1	-	MMPL family transporter	1	0	0	0	1	1	1	0	0	1	1	6	Accessory	COG2409_R#	33.3	398	7.74E-110	K11625	33.3	398	2.07E-109
550	AXI88559.1	-	helicase SNF2	0	0	0	1	0	0	0	0	0	0	0	1	Unique	COG0553_KL#	46.1	415	4.68E-115	NA	26.8	156	1.05E-36
551	WP_158688227.1	-	hypothetical protein	0	0	0	0	1	0	0	0	0	0	0	1	Unique								
552	PWE08487.1	-	hypothetical protein	0	0	1	0	0	0	0	0	0	0	0	1	Unique								
553	PWE09218.1	-	hypothetical protein	1	1	1	1	1	1	1	1	1	1	1	11	CORE	COG0747_E#	31.7	155	8.74E-37	K02035	31.7	155	2.33E-36
554	AXI87451.1	-	hypothetical protein	1	1	1	1	1	1	1	1	1	1	1	11	CORE	COG2372_R#	39.7	86	4.98E-16	K14166	26.9	79	1.24E-13
555	WP_122216964.1	-	PQQ-binding-like beta-propeller repeat protein	0	0	0	1	0	0	0	0	0	1	0	2	Accessory	COG0515_RTKL#	33.2	151	1.26E-35	K08884	35.2	150	7.5E-35
556	WP_033272148.1	-	hypothetical protein	1	1	1	1	1	1	1	1	1	0	1	10	Accessory	COG2730_G#	30.4	62	5.89E-09	NA	32	154	5.19E-36
557	WP_102641351.1	-	glycosyl hydrolase family 5	1	1	1	1	1	1	1	1	1	1	1	11	CORE					NA	40.9	560	3.14E-158
558	OSC72480.1	-	glycosyl transferase	1	1	1	1	1	1	0	1	1	1	1	10	Accessory	COG1887_M#	31.2	201	1.38E-50	K09809	31	206	8.8E-52
559	Prokka:JAPCLKMA_00230	uvrC	UvrABC system protein C	1	1	1	1	1	1	1	1	1	1	1	11	CORE	COG0322_L#	60.6	804	3.57E-232	K03703	60.6	804	9.52E-232
560	WP_019527359.1	katG	catalase/peroxidase HPI	1	1	1	1	1	1	1	1	1	1	1	11	CORE	COG0376_P#	63	986	5.07E-287	K03782	63	986	1.35E-286
561	PWE11251.1	-	N-acetylmuramoyl-L-alanine amidase	1	0	1	0	1	1	0	0	0	0	1	5	Accessory	COG5479_M#	28.3	226	2.34E-58	NA	38.3	193	7.67E-48

cluster	GI	gene	Annotation	Streptomyces_thermocarboxydus_BPSAC147	Streptomyces_sp._4F	Streptomyces_sp._BSE7F	Streptomyces_sp._ETH9427	Streptomyces_sp._FxanaD5	Streptomyces_sp._UNC401CLCol	Actinospica_acidiphila_NRRL_B_24431	Streptomyces_sp._HNS054	Streptomyces_sp._SMS_SU21	Streptomyces_sp._ZS0098	Streptomyces_thermocarboxydus_K155	Addition	Core-Accessory-Unique	COG	%Similarity_COG	Score_COG	Evalue_COG	KEGG	%Similarity_KEGG	Score_KEGG	Evalue_KEGG
562	WP_102639205.1	-	hypothetical protein	0	0	0	1	0	0	0	1	1	1	0	4	Accessory								
563	Prokka:JAPCLKMA_06322	-	hypothetical protein	1	0	0	1	1	1	1	1	0	1	1	8	Accessory	COG2310_T#	39.4	135	7.11E-31	K05795	39.4	135	1.9E-30
564	WP_019524114.1	-	hypothetical protein	0	0	0	0	1	0	0	0	0	0	0	1	Unique								
565	Prokka:CIODPCDJ_05102	-	hypothetical protein	1	0	1	1	1	1	0	1	1	1	1	9	Accessory					NA	25.3	61	3.48E-08
566	WP_122217389.1	-	tetratricopeptide repeat protein	0	0	0	0	0	0	1	0	0	1	0	2	Accessory	COG0457_R#	22.9	70	2.14E-11				
567	WP_033275281.1	-	NHLP family bacteriocin export ABC transporter peptidase/permease/ATPase subunit	1	0	1	1	1	1	1	1	1	1	1	10	Accessory	COG2274_V#	46.5	645	2.11E-184	K06147	29.1	280	4.75E-74
568	Prokka:CIODPCDJ_02151	-	hypothetical protein	1	1	1	0	1	1	1	1	1	1	1	10	Accessory								
569	WP_147474341.1	-	DUF2325 domain-containing protein	0	0	0	0	0	0	0	0	0	1	0	1	Unique								
570	AXI89062.1	-	polyribonucleotide nucleotidyltransferase	1	1	1	1	1	1	1	1	1	1	1	11	CORE	COG1185_J#	72.4	1044	2.03E-304	K00962	72.4	1044	5.42E-304

cluster	GI	gene	Annotation	Streptomyces_thermocarboxydus_BPSAC147	Streptomyces_sp._4F	Streptomyces_sp._BSE7F	Streptomyces_sp._ETH9427	Streptomyces_sp._FxanaD5	Streptomyces_sp._UNC401CLCol	Actinospica_acidiphila_NRRL_B_24431	Streptomyces_sp._HNS054	Streptomyces_sp._SMS_SU21	Streptomyces_sp._ZS0098	Streptomyces_thermocarboxydus_K155	Addition	Core-Accessory-Unique	COG	%Similarity_COG	Score_COG	Evalue_COG	KEGG	%Similarity_KEGG	Score_KEGG	Evalue_KEGG
571	WP_122212601.1	-	SpoIIE family protein phosphatase	1	0	0	0	1	1	1	1	1	1	1	8	Accessory	COG2203_T#	45	156	2.96E-37	K07315	25.4	78	3.58E-13
572	WP_048459287.1	-	alpha-L-fucosidase	1	1	1	1	1	1	1	1	1	1	1	11	CORE					K15923	26.5	179	8.73E-44
573	OSC55595.1	-	isocitrate dehydrogenase (NADP(+))	1	1	1	1	1	1	1	1	1	1	1	11	CORE	COG2838_C#	68	994	2.41E-289	K00031	65.3	969	2.22E-281
574	WP_026244253.1	-	S8 family peptidase	0	0	0	0	1	0	0	0	0	0	0	1	Unique								
575	PWE09086.1	-	polysaccharide pyruvyl transferase	0	1	1	1	0	0	0	1	0	1	0	5	Accessory	COG0500_QR#	36.1	177	1.62E-43				
576	OSC63594.1	-	ATP-dependent DNA helicase RecG	1	1	1	1	1	1	1	1	1	1	1	11	CORE	COG1200_LK#	46.7	617	7.98E-176	K03655	46.7	617	2.13E-175
577	WP_019525806.1	-	glycosyltransferase	1	0	0	1	1	1	1	1	1	1	1	9	Accessory	COG1887_M#	31.6	186	4.54E-46	K09809	30.4	186	1.21E-45
578	WP_109288732.1	-	N-6 DNA methylase	1	1	1	1	1	1	1	1	1	1	1	11	CORE	COG0286_V#	24.8	101	1.47E-20	NA	30.6	66	1.4E-09
579	Prokka:CIODPCDJ_05790	-	hypothetical protein	1	1	1	1	1	1	1	1	1	1	1	11	CORE								
580	WP_019524896.1	-	hypothetical protein	0	0	0	0	1	0	0	0	0	0	0	1	Unique								
581	OSC62711.1	-	DNA ligase (NAD(+)) LigA	1	1	1	1	1	1	1	1	1	1	1	11	CORE	COG0272_L#	54.4	730	6.44E-210	K01972	51.7	599	3.5E-170
582	WP_033277531.1	-	glycosyltransferase	1	1	1	1	1	1	1	1	1	1	1	11	CORE	COG1215_M#	31.3	194	1.27E-48	K11936	33.1	163	8.37E-39

cluster	GI	gene	Annotation	Streptomyces_thermocarboxydus_BPSAC147	Streptomyces_sp._4F	Streptomyces_sp._BSE7F	Streptomyces_sp._ETH9427	Streptomyces_sp._FxanaD5	Streptomyces_sp._UNC401CLCol	Actinospica_acidiphila_NRRL_B_24431	Streptomyces_sp._HNS054	Streptomyces_sp._SMS_SU21	Streptomyces_sp._ZS0098	Streptomyces_thermocarboxydus_K155	Addition	Core-Accessory-Unique	COG	%Similarity_COG	Score_COG	Evalue_COG	KEGG	%Similarity_KEGG	Score_KEGG	Evalue_KEGG
583	WP_026244061.1	-	Gfo/Idh/MocA family oxidoreductase	1	0	1	0	1	1	1	1	1	1	1	9	Accessory	COG1063_ER#	40.5	287	1.1E-76	NA	27.3	92	1.82E-17
584	Prokka:CIODPCDJ_05812	fadJ_3	Fatty acid oxidation complex subunit alpha	1	1	1	1	1	1	1	1	1	1	1	11	CORE	COG1250_I#	67.7	536	1.36E-151	K01782	64.8	917	9.9E-266
585	PWE06600.1	-	elongation factor G-like protein EF-G2	1	1	1	1	1	1	1	1	1	1	1	11	CORE	COG0480_J#	62.4	892	1.28E-258	K02355	62.3	891	7.6E-258
586	WP_048458872.1	-	DEAD/DEAH box helicase	1	0	0	0	0	0	0	1	1	0	0	3	Accessory	COG1201_R#	30.4	257	1.22E-67	K03724	30.7	207	3.87E-52
587	WP_102638944.1	-	transketolase	0	0	1	1	0	0	0	1	1	1	0	5	Accessory	COG0022_C#	31.7	156	3.83E-37	K00162	31.2	138	2.21E-31
588	WP_028959136.1	-	CDP-glycerol glycerophosphotransferase family protein	1	1	1	1	1	1	1	1	1	1	1	11	CORE	COG1887_M#	32.7	206	3.23E-52	K09809	32.6	204	4.28E-51
589	WP_107080958.1	-	DUF2075 domain-containing protein	0	0	0	0	0	0	0	1	0	0	0	1	Unique								
590	Prokka:JAPCLKMA_03953	rhlB_1	ATP-dependent RNA helicase RhlB	1	1	1	1	1	1	1	1	1	1	1	11	CORE	COG0514_L#	52.2	685	2.37E-196	K03654	34.8	184	3.51E-45
591	WP_019526060.1	-	DUF3631 domain-containing protein	0	0	0	0	1	0	0	0	0	0	0	1	Unique	COG1674_D#	22.5	48	0.000147	NA	22.5	48	0.000392

cluster	GI	gene	Annotation	Streptomyces_thermocarboxydus_BPSAC147	Streptomyces_sp._4F	Streptomyces_sp._BSE7F	Streptomyces_sp._ETH9427	Streptomyces_sp._FxanaD5	Streptomyces_sp._UNC401CLCol	Actinospica_acidiphila_NRRL_B_24431	Streptomyces_sp._HNS054	Streptomyces_sp._SMS_SU21	Streptomyces_sp._ZS0098	Streptomyces_thermocarboxydus_K155	Addition	Core-Accessory-Unique	COG	%Similarity_COG	Score_COG	Evalue_COG	KEGG	%Similarity_KEGG	Score_KEGG	Evalue_KEGG
592	Prokka:JAPCLKMA_04666	-	hypothetical protein	1	0	0	0	0	1	0	0	0	0	1	3	Accessory								
593	OSC69701.1	-	cell division protein FtsK	0	1	0	0	0	0	0	0	0	0	0	1	Unique	COG1674_D#	24.4	59	8.29E-08	NA	26.2	55	0.0000032
594	WP_028959436.1	-	PQQ-binding-like beta-propeller repeat protein	1	1	1	1	1	1	1	1	1	1	1	11	CORE	COG0515_RTKL#	33.5	164	1.07E-39	K08884	29.5	160	5.4E-38
595	WP_122212544.1	-	ATP-binding cassette domain-containing protein	1	1	0	1	1	1	0	1	1	1	1	9	Accessory	COG1131_V#	32.5	97	2.74E-19	K01990	28.3	100	8.66E-20
596	WP_122214869.1	-	glycosyl hydrolase	1	1	1	0	1	1	1	1	1	1	1	10	Accessory	COG1501_G#	36.7	196	4.34E-49	K05546	38.4	154	5.05E-36
597	WP_019526811.1	-	serine/threonine protein kinase	1	1	1	1	1	1	1	1	1	1	1	11	CORE	COG0515_RTKL#	41	206	2.46E-52	K08884	36.9	196	1.16E-48
598	PWE06622.1	-	hypothetical protein	0	0	1	0	0	0	0	0	0	0	0	1	Unique								
599	PWE08990.1	-	nb-arc domain-containing protein	0	0	1	0	0	0	0	0	0	0	0	1	Unique	COG0457_R#	31.2	85	8.25E-16	NA	24.9	55	0.00000319
600	WP_122215130.1	-	tetratricopeptide repeat protein	1	1	1	1	1	1	1	1	1	1	1	11	CORE	COG0513_LKJ#	41.3	105	7.71E-22	NA	45.9	195	1.51E-48
601	OSC63418.1	-	Geosmin synthase	1	1	1	1	1	1	1	1	1	1	1	11	CORE								

cluster	GI	gene	Annotation	Streptomyces_thermocarboxydus_BPSAC147	Streptomyces_sp._4F	Streptomyces_sp._BSE7F	Streptomyces_sp._ETH9427	Streptomyces_sp._FxanaD5	Streptomyces_sp._UNC401CLCol	Actinospica_acidiphila_NRRL_B_24431	Streptomyces_sp._HNS054	Streptomyces_sp._SMS_SU21	Streptomyces_sp._ZS0098	Streptomyces_thermocarboxydus_K155	Addition	Core-Accessory-Unique	COG	%Similarity_COG	Score_COG	Evalue_COG	KEGG	%Similarity_KEGG	Score_KEGG	Evalue_KEGG
602	WP_122215733.1	-	SpoIIE family protein phosphatase	0	0	0	0	0	0	0	0	0	1	0	1	Unique	COG2208_TK#	31.8	114	2.16E-24	NA	30	131	3.5E-29
603	PWE07480.1	-	acyltransferase	0	1	1	1	1	1	1	1	1	1	1	10	Accessory	COG1835_I#	34.9	349	2.34E-95	NA	30	251	3.02E-65
604	Prokka:JAPCLKMA_03934	-	hypothetical protein	1	1	0	1	1	1	1	0	1	1	1	9	Accessory	COG0317_TK#	43.3	423	1.66E-117	K00951	42.5	417	3.18E-115
605	WP_122216267.1	-	cellobiohydrolase	0	0	0	0	0	0	0	0	0	1	0	1	Unique	COG5297_G#	56	538	4.63E-152	K01179	35	129	1.01E-28
606	WP_122213627.1	-	alpha-1,4-glucan--maltose-1-phosphate maltosyltransferase	1	1	1	1	1	1	1	1	1	1	1	11	CORE	COG0366_G#	53.7	701	4.13E-201	K16147	53.7	701	1.1E-200
607	Prokka:CIODPCDJ_04033	-	hypothetical protein	0	0	0	0	0	0	0	0	0	0	1	1	Unique	COG1479_S#	30.7	299	4.72E-80	NA	21	85	2.86E-15
608	Prokka:JAPCLKMA_02056	-	hypothetical protein	1	0	0	0	1	1	0	0	0	0	1	4	Accessory								
609	OSC61220.1	-	polyketide synthase	0	1	0	0	0	0	0	0	0	0	0	1	Unique	COG3321_Q#	49.3	637	7.32E-182	K12435	48.8	636	4.35E-181
610	WP_019525021.1	-	mycodextranase	1	0	0	0	1	1	1	0	0	0	1	5	Accessory								
611	WP_033274348.1	-	FUSC family protein	1	0	0	0	1	1	1	1	1	1	1	8	Accessory	COG1289_S#	28.3	60	3.69E-08	NA	27.9	59	1.29E-07
612	WP_102637414.1	-	DEAD/DEAH box helicase	1	0	1	0	0	0	1	0	1	1	1	6	Accessory	COG0210_L#	36.9	394	8.25E-109	K03657	28.9	45	0.00192
613	Prokka:CIODPCDJ_00327	thi4_1	Thiamine thiazole synthase	1	1	1	1	1	1	1	1	1	1	1	11	CORE	COG1233_Q#	36.3	270	1.37E-71	NA	36.3	270	3.67E-71

cluster	GI	gene	Annotation	Streptomyces_thermocarboxydus_BPSAC147	Streptomyces_sp._4F	Streptomyces_sp._BSE7F	Streptomyces_sp._ETH9427	Streptomyces_sp._FxanaD5	Streptomyces_sp._UNC401CLCol	Actinospica_acidiphila_NRRL_B_24431	Streptomyces_sp._HNS054	Streptomyces_sp._SMS_SU21	Streptomyces_sp._ZS0098	Streptomyces_thermocarboxydus_K155	Addition	Core-Accessory-Unique	COG	%Similarity_COG	Score_COG	Evalue_COG	KEGG	%Similarity_KEGG	Score_KEGG	Evalue_KEGG
614	WP_122213144.1	-	DUF1996 domain-containing protein	1	1	1	1	1	1	1	1	1	1	1	11	CORE	COG0737_F#	30.1	51	0.0000131	NA	35.3	110	6.34E-23
615	OSC63040.1	-	hypothetical protein	0	1	0	0	0	0	0	0	0	0	0	1	Unique								
616	WP_102637433.1	-	N-6 DNA methylase	1	1	0	0	0	0	0	0	1	1	1	5	Accessory	COG0286_V#	25.3	115	7.39E-25				
617	Prokka:JAPCLKMA_01997	-	hypothetical protein	1	0	0	0	0	0	0	0	0	0	0	1	Unique								
618	Prokka:JAPCLKMA_05003	thiK_1	Thiamine kinase	1	0	1	1	1	1	1	1	0	1	1	9	Accessory								
619	WP_122216842.1	uvrB	excinuclease ABC subunit UvrB	1	1	1	1	1	1	1	1	1	1	1	11	CORE	COG0556_L#	73.4	1028	1.12E-299	K03702	73.7	1023	7.38E-298
620	WP_051724455.1	-	hydantoinase	0	0	0	0	0	0	1	0	0	0	0	1	Unique	COG0145_EQ#	29.8	182	4.91E-45	K01473	28.9	113	7.47E-24
621	WP_048460379.1	-	asparagine synthase	0	1	0	1	1	1	1	1	1	1	1	9	Accessory								
622	WP_102641858.1	-	SpoIIE family protein phosphatase	0	0	0	1	0	0	0	0	1	1	0	3	Accessory	COG2208_TK#	31.1	111	1.06E-23	NA	28.2	127	3.82E-28
623	OSC67540.1	-	transposase	0	1	0	0	0	0	0	0	0	0	0	1	Unique								
624	PWE10792.1	-	alpha-galactosidase	1	0	1	1	1	1	1	1	1	1	1	10	Accessory	COG3345_G#	32.3	367	1.4E-100	K07407	33.7	349	8.06E-95
625	WP_019523736.1	-	primosomal protein N'	1	1	0	1	1	1	1	1	1	1	1	10	Accessory	COG1198_L#	51.6	596	1.09E-169	K04066	51.6	596	2.9E-169
626	WP_048458908.1	-	CoA-binding protein	1	1	1	1	1	1	1	1	1	1	1	11	CORE	COG1042_C#	37.7	393	2.38E-108	K09181	37.9	316	5.79E-85

cluster	GI	gene	Annotation	Streptomyces_thermocarboxydus_BPSAC147	Streptomyces_sp._4F	Streptomyces_sp._BSE7F	Streptomyces_sp._ETH9427	Streptomyces_sp._FxanaD5	Streptomyces_sp._UNC401CLCol	Actinospica_acidiphila_NRRL_B_24431	Streptomyces_sp._HNS054	Streptomyces_sp._SMS_SU21	Streptomyces_sp._ZS0098	Streptomyces_thermocarboxydus_K155	Addition	Core-Accessory-Unique	COG	%Similarity_COG	Score_COG	Evalue_COG	KEGG	%Similarity_KEGG	Score_KEGG	Evalue_KEGG
627	OSC65469.1	-	GGDEF domain-containing protein	1	1	1	1	1	1	1	1	1	1	1	11	CORE	COG5001_T#	43.2	325	4.66E-88	NA	37.5	345	1.52E-93
628	WP_019525903.1	-	SpoIIE family protein phosphatase	1	0	1	1	1	1	1	1	1	1	1	10	Accessory	COG2208_TK#	29.9	106	3.4E-22	NA	30.5	129	1.71E-28
629	OSC61809.1	-	1,4-beta-xylanase	0	1	0	0	0	0	0	0	0	0	0	1	Unique					NA	49.4	292	8.94E-78
630	WP_048455359.1	-	MMPL family transporter	0	0	0	1	0	0	0	1	0	0	0	2	Accessory	COG2409_R#	36.3	393	1.39E-108	K11625	25.5	190	6.25E-47
631	WP_026244179.1	-	FxsB family radical SAM/SPASM domain protein	1	0	0	0	1	1	0	0	0	0	1	4	Accessory	COG0641_R#	24.6	71	1.21E-11	K06871	24.7	81	4.09E-14
632	WP_033275767.1	-	molybdopterin-dependent oxidoreductase	1	1	1	1	1	1	1	1	1	1	1	11	CORE	COG0243_C#	46.6	590	5.95E-168	K00372	43.5	568	8.44E-161
633	WP_019524113.1	-	DEAD/DEAH box helicase family protein	0	0	0	0	1	0	0	0	0	0	0	1	Unique	COG1061_KL#	43.8	366	3.11E-100	NA	30.7	94	6.09E-18
634	WP_146217779.1	-	hypothetical protein	0	0	0	0	0	0	0	0	1	0	0	1	Unique	COG4372_S#	25.4	56	4.03E-07	NA	25.4	56	0.00000108
635	PWE10343.1	-	high-affinity choline transporter BetT	1	0	1	1	1	1	1	1	1	1	1	10	Accessory	COG1292_M#	56	757	6.26E-218	K02168	56	757	1.67E-217

cluster	GI	gene	Annotation	Streptomyces_thermocarboxydus_BPSAC147	Streptomyces_sp._4F	Streptomyces_sp._BSE7F	Streptomyces_sp._ETH9427	Streptomyces_sp._FxanaD5	Streptomyces_sp._UNC401CLCol	Actinospica_acidiphila_NRRL_B_24431	Streptomyces_sp._HNS054	Streptomyces_sp._SMS_SU21	Streptomyces_sp._ZS0098	Streptomyces_thermocarboxydus_K155	Addition	Core-Accessory-Unique	COG	%Similarity_COG	Score_COG	Evalue_COG	KEGG	%Similarity_KEGG	Score_KEGG	Evalue_KEGG
636	WP_020700220.1	-	SpoIIE family protein phosphatase	1	0	1	1	1	1	1	1	1	1	1	10	Accessory	COG2208_TK#	34.6	114	1.63E-24	NA	34.6	114	4.35E-24
637	WP_102638061.1	-	hypothetical protein	0	0	0	0	0	0	0	0	1	0	0	1	Unique								
638	OSC66750.1	-	hypothetical protein	0	1	0	0	0	0	0	0	0	0	0	1	Unique	COG4928_R#	26.2	148	1.02E-34				
639	Prokka:CIODPCDJ_06179	paoC	Aldehyde oxidoreductase molybdenum-binding subunit PaoC	1	1	1	1	1	1	1	1	1	1	1	11	CORE	COG1529_C#	43.6	530	9.51E-150	K11177	43.6	530	2.54E-149
640	WP_019522296.1	-	S9 family peptidase	1	1	0	1	1	1	1	1	1	1	1	10	Accessory	COG1506_E#	30.1	286	3.12E-76	K01278	27.7	232	1.43E-59
641	Prokka:JAPCLKMA_02600	-	hypothetical protein	1	1	1	1	1	1	1	1	1	1	1	11	CORE								
642	WP_019526077.1	-	hypothetical protein	0	0	0	0	1	0	0	0	0	0	0	1	Unique	COG5412_S#	21.3	74	2.43E-12				
643	WP_033275403.1	-	SpoIIE family protein phosphatase	1	0	1	1	1	1	1	1	1	1	1	10	Accessory	COG2208_TK#	35.6	136	3.99E-31	NA	31.2	149	1.22E-34
644	WP_048458865.1	-	tetratricopeptide repeat protein	0	0	0	0	0	0	0	1	0	0	0	1	Unique	COG0457_R#	21	61	1.25E-08	K19360	22.1	56	0.0000014
645	Prokka:CIODPCDJ_05680	-	hypothetical protein	0	0	0	0	0	0	0	0	0	0	1	1	Unique								

cluster	GI	gene	Annotation	Streptomyces_thermocarboxydus_BPSAC147	Streptomyces_sp._4F	Streptomyces_sp._BSE7F	Streptomyces_sp._ETH9427	Streptomyces_sp._FxanaD5	Streptomyces_sp._UNC401CLCol	Actinospica_acidiphila_NRRL_B_24431	Streptomyces_sp._HNS054	Streptomyces_sp._SMS_SU21	Streptomyces_sp._ZS0098	Streptomyces_thermocarboxydus_K155	Addition	Core-Accessory-Unique	COG	%Similarity_COG	Score_COG	Evalue_COG	KEGG	%Similarity_KEGG	Score_KEGG	Evalue_KEGG
646	WP_084207051.1	-	SpoIIE family protein phosphatase	1	1	1	1	1	1	1	1	1	1	1	11	CORE	COG2208_TK#	33.3	106	3.38E-22	NA	31.2	132	1.54E-29
647	Prokka:CIODPCDJ_05050	-	Ferredoxin--NADP reductase	1	0	1	0	1	1	1	0	0	0	1	6	Accessory	COG2072_P#	36	274	9.35E-73	NA	31.8	229	9.22E-59
648	WP_019524949.1	-	DDE-type integrase/transposase/recombinase	0	0	0	0	1	0	0	0	0	0	0	1	Unique	COG2801_L#	25	79	4.42E-14	K07497	25	79	1.18E-13
649	PWE09929.1	-	3-hydroxyacyl-CoA dehydrogenase	1	1	1	1	1	1	1	1	1	1	1	11	CORE	COG1250_I#	35.3	212	4.37E-54	K07516	31.3	312	1.41E-83
650	Prokka:CIODPCDJ_03953	fusA_1	Elongation factor G	1	1	1	1	1	1	1	1	1	1	1	11	CORE	COG0480_J#	70.1	992	8.77E-289	K02355	70.1	992	2.34E-288
651	WP_019525243.1	-	cell division protein FtsK	1	0	0	1	1	0	1	0	0	0	0	4	Accessory								
652	WP_019526328.1	-	type IIA DNA topoisomerase subunit B	1	1	1	1	1	1	1	1	1	1	1	11	CORE	COG0187_L#	45.3	565	2.03E-160	K02470	45.3	565	5.42E-160
653	WP_033273643.1	-	AAA domain-containing protein	0	0	0	0	0	0	1	0	0	0	0	1	Unique	COG1401_V#	36.6	169	3.24E-41	K07452	35.9	159	8.96E-38
654	WP_102640851.1	-	MMPL family transporter	1	0	1	1	1	1	1	1	1	1	1	10	Accessory	COG2409_R#	37.1	439	1.68E-122	K06994	28.3	203	9.21E-51

cluster	GI	gene	Annotation	Streptomyces_thermocarboxydus_BPSAC147	Streptomyces_sp._4F	Streptomyces_sp._BSE7F	Streptomyces_sp._ETH9427	Streptomyces_sp._FxanaD5	Streptomyces_sp._UNC401CLCol	Actinospica_acidiphila_NRRL_B_24431	Streptomyces_sp._HNS054	Streptomyces_sp._SMS_SU21	Streptomyces_sp._ZS0098	Streptomyces_thermocarboxydus_K155	Addition	Core-Accessory-Unique	COG	%Similarity_COG	Score_COG	Evalue_COG	KEGG	%Similarity_KEGG	Score_KEGG	Evalue_KEGG
655	Prokka:JAPCLKMA_03173	-	hypothetical protein	1	1	1	1	1	1	1	1	1	1	1	11	CORE	COG0810_M#	31.7	49	0.0000488	NA	33.3	50	0.0000998
656	Prokka:JAPCLKMA_03885	pta	Phosphate acetyltransferase	1	1	1	1	1	1	1	1	1	1	1	11	CORE	COG0280_C#	69.1	449	2.12E-125	K13788	49	620	1.85E-176
657	Prokka:JAPCLKMA_04693	malQ	4-alpha-glucanotransferase	1	1	1	1	1	1	1	1	1	1	1	11	CORE	COG1640_G#	47.9	631	3.92E-180	K00705	47.9	631	1.05E-179
658	WP_033275953.1	-	hypothetical protein	1	1	0	1	1	1	1	1	1	1	1	10	Accessory								
659	OSC70184.1	-	glycosyl hydrolase	0	1	0	0	0	0	0	0	0	0	0	1	Unique	COG3291_R#	28.3	91	1.12E-17				
660	WP_106432515.1	-	hypothetical protein	1	0	1	1	1	1	1	1	1	1	1	10	Accessory					NA	33.8	99	1.87E-19
661	Prokka:CIODPCDJ_06530	apeX_2	Apo-petrobactin exporter	1	1	1	1	1	1	1	1	1	1	1	11	CORE	COG2409_R#	48.8	629	1.49E-179	K11625	24.6	181	3.74E-44
662	WP_028959132.1	-	HAMP domain-containing histidine kinase	1	1	0	1	1	1	1	1	1	1	1	10	Accessory	COG0642_T#	49.4	451	4.25E-126	K07654	49.4	451	1.14E-125
663	Prokka:JAPCLKMA_05471	-	hypothetical protein	1	0	0	0	0	0	0	0	0	0	0	1	Unique	COG1674_D#	25.2	69	4.53E-11	NA	25.2	69	1.21E-10
664	WP_048459678.1	-	LamG domain-containing protein	0	0	0	1	0	0	0	1	0	1	0	3	Accessory								
665	WP_122216070.1	-	MMPL family transporter	1	1	1	0	0	0	1	0	1	1	1	7	Accessory	COG2409_R#	32.6	309	3.4E-83	K06994	31.3	294	3.02E-78
666	Prokka:CIODPCDJ_04324	-	hypothetical protein	1	1	1	1	1	1	1	1	1	1	1	11	CORE	COG2208_TK#	32.1	121	1.71E-26	NA	28.8	135	1.79E-30

cluster	GI	gene	Annotation	Streptomyces_thermocarboxydus_BPSAC147	Streptomyces_sp._4F	Streptomyces_sp._BSE7F	Streptomyces_sp._ETH9427	Streptomyces_sp._FxanaD5	Streptomyces_sp._UNC401CLCol	Actinospica_acidiphila_NRRL_B_24431	Streptomyces_sp._HNS054	Streptomyces_sp._SMS_SU21	Streptomyces_sp._ZS0098	Streptomyces_thermocarboxydus_K155	Addition	Core-Accessory-Unique	COG	%Similarity_COG	Score_COG	Evalue_COG	KEGG	%Similarity_KEGG	Score_KEGG	Evalue_KEGG
667	Prokka:JAPCLKMA_05587	-	hypothetical protein	1	0	0	0	0	0	0	0	0	0	0	1	Unique	COG3320_Q#	37.4	166	4.63E-40	K13611	42.3	49	0.000169
668	Prokka:JAPCLKMA_00730	kimA_1	Potassium transporter KimA	1	1	1	1	1	1	1	1	1	1	1	11	CORE	COG0531_E#	62.9	802	1.27E-231	NA	62.9	802	3.4E-231
669	Prokka:CIODPCDJ_06523	COQ5_11	2-methoxy-6-polyprenyl-1,4-benzoquinol methylase, mitochondrial	1	0	0	0	1	1	1	0	0	0	1	5	Accessory	COG0747_E#	28.8	215	5.09E-55	K19226	24.3	107	3.99E-22
670	Prokka:JAPCLKMA_05492	tdh_6	L-threonine 3-dehydrogenase	1	0	0	0	0	0	0	0	0	0	0	1	Unique	COG1063_ER#	25.4	61	1.23E-08				
671	OSC61943.1	-	potassium-transporting ATPase subunit B	0	1	1	1	0	0	1	1	1	1	0	7	Accessory	COG2216_P#	70.5	904	2.38E-262	K01547	70.5	904	6.35E-262
672	WP_026243495.1	-	ATP-binding domain-containing protein	1	0	1	0	1	1	1	1	1	1	1	9	Accessory	COG0507_L#	25.4	106	3.33E-22	K03581	25.5	87	5.58E-16
673	WP_122216698.1	-	helix-turn-helix domain-containing protein	0	0	0	0	0	0	0	0	0	1	0	1	Unique								
674	WP_102639647.1	-	S9 family peptidase	1	0	1	1	1	1	1	1	1	1	1	10	Accessory	COG1506_E#	29.3	183	2.8E-45	NA	27.9	175	1.56E-42

cluster	GI	gene	Annotation	Streptomyces_thermocarboxydus_BPSAC147	Streptomyces_sp._4F	Streptomyces_sp._BSE7F	Streptomyces_sp._ETH9427	Streptomyces_sp._FxanaD5	Streptomyces_sp._UNC401CLCol	Actinospica_acidiphila_NRRL_B_24431	Streptomyces_sp._HNS054	Streptomyces_sp._SMS_SU21	Streptomyces_sp._ZS0098	Streptomyces_thermocarboxydus_K155	Addition	Core-Accessory-Unique	COG	%Similarity_COG	Score_COG	Evalue_COG	KEGG	%Similarity_KEGG	Score_KEGG	Evalue_KEGG
675	OSC53421.1	-	LuxR family transcriptional regulator	0	1	0	0	0	0	0	0	0	0	0	1	Unique	COG2771_K#	27.2	80	1.95E-14	NA	24.9	78	3.38E-13
676	Prokka:JAPCLKMA_04878	-	hypothetical protein	1	0	0	0	0	0	0	0	0	0	0	1	Unique					NA	30.9	59	1.25E-07
677	WP_033274043.1	-	glycosyltransferase family 4 protein	0	0	0	0	0	0	1	1	0	1	0	3	Accessory	COG0438_M#	30.1	141	1.22E-32	NA	28.8	156	7.47E-37
678	WP_122213370.1	-	alpha-galactosidase	0	0	0	0	0	0	0	0	0	1	0	1	Unique	COG3345_G#	30.5	309	4.41E-83	K07407	29.9	265	1.95E-69
679	OSC65850.1	-	MFS transporter	0	1	0	0	0	0	0	0	0	0	0	1	Unique	COG0477_GEPR#	49	615	2.87E-175	NA	49	615	7.68E-175
680	WP_048458587.1	-	hypothetical protein	0	0	0	0	0	0	0	1	0	0	0	1	Unique	COG2812_L#	31.2	48	0.00014	NA	28.5	51	0.0000442
681	WP_102641429.1	-	hypothetical protein	0	0	0	1	0	0	0	0	1	1	0	3	Accessory								
682	WP_102640091.1	-	penicillin acylase family protein	1	1	1	1	1	1	1	1	1	1	1	11	CORE	COG2366_R#	36.4	164	1.34E-39	K07116	24.4	83	1.05E-14
683	AXI85404.1	-	N-acetylmuramoyl-L-alanine amidase	0	0	0	1	0	0	1	1	1	1	0	5	Accessory	COG5479_M#	36.1	202	5.8E-51	NA	36.4	200	4.51E-50
684	Prokka:CIODPCDJ_02923	-	hypothetical protein	1	1	0	1	1	1	1	1	1	1	1	10	Accessory	COG3973_R#	32.1	219	4.58E-56	K03657	34.1	127	3.71E-28
685	Prokka:JAPCLKMA_04048	mdtD_6	Putative multidrug resistance protein MdtD	1	1	1	1	1	1	1	1	1	1	1	11	CORE	COG0477_GEPR#	43.5	465	3.67E-130	NA	43.5	465	9.8E-130

cluster	GI	gene	Annotation	Streptomyces_thermocarboxydus_BPSAC147	Streptomyces_sp._4F	Streptomyces_sp._BSE7F	Streptomyces_sp._ETH9427	Streptomyces_sp._FxanaD5	Streptomyces_sp._UNC401CLCol	Actinospica_acidiphila_NRRL_B_24431	Streptomyces_sp._HNS054	Streptomyces_sp._SMS_SU21	Streptomyces_sp._ZS0098	Streptomyces_thermocarboxydus_K155	Addition	Core-Accessory-Unique	COG	%Similarity_COG	Score_COG	Evalue_COG	KEGG	%Similarity_KEGG	Score_KEGG	Evalue_KEGG
686	WP_102639180.1	-	trypsin-like peptidase domain-containing protein	1	0	0	0	0	0	0	0	1	0	0	2	Accessory	COG0265_O#	39.4	231	6.83E-60	NA	39.4	231	1.82E-59
687	Prokka:JAPCLKMA_01626	pcm_2	Protein-L-isoaspartate O-methyltransferase	1	0	0	0	0	0	0	0	0	0	0	1	Unique	COG2518_O#	39.2	140	2.07E-32	K00573	36.7	127	6.32E-28
688	OSC63391.1	-	hypothetical protein	1	1	0	1	0	0	1	1	1	1	0	7	Accessory	COG2409_R#	34.9	376	2.24E-103	K06994	28	186	6.73E-46
689	Prokka:JAPCLKMA_02609	-	hypothetical protein	1	0	0	0	0	0	0	0	0	0	0	1	Unique	COG0438_M#	28.1	101	1.39E-20	NA	26.1	94	5.93E-18
690	WP_019522238.1	-	transketolase	1	0	0	1	1	1	1	1	1	1	1	9	Accessory	COG0021_G#	64	877	2.37E-254	K00615	64	876	1.41E-253
691	WP_102642218.1	-	aminotransferase class I/II-fold pyridoxal phosphate-dependent enzyme	0	0	1	1	0	0	0	1	1	1	0	5	Accessory	COG1167_KE#	39.5	319	2.49E-86	K05825	39.4	293	6.65E-78
692	WP_122216143.1	-	ATP-binding protein	1	1	0	1	1	1	1	1	1	1	1	10	Accessory	COG0642_T#	30.2	197	1.86E-49	NA	30.2	197	4.97E-49
693	PWE07117.1	-	hypothetical protein	1	1	1	1	1	1	1	1	1	1	1	11	CORE	COG1511_S#	27.8	197	1.86E-49	K01421	27.8	197	4.96E-49
694	OSC69217.1	-	DNA topoisomerase (ATP-hydrolyzing) subunit B	1	1	1	1	1	1	1	1	1	1	1	11	CORE	COG0187_L#	64	878	1.39E-254	K02470	64	876	1.84E-253
695	WP_122215460.1	-	hypothetical protein	1	1	0	1	1	1	1	1	1	1	1	10	Accessory								

cluster	GI	gene	Annotation	Streptomyces_thermocarboxydus_BPSAC147	Streptomyces_sp._4F	Streptomyces_sp._BSE7F	Streptomyces_sp._ETH9427	Streptomyces_sp._FxanaD5	Streptomyces_sp._UNC401CLCol	Actinospica_acidiphila_NRRL_B_24431	Streptomyces_sp._HNS054	Streptomyces_sp._SMS_SU21	Streptomyces_sp._ZS0098	Streptomyces_thermocarboxydus_K155	Addition	Core-Accessory-Unique	COG	%Similarity_COG	Score_COG	Evalue_COG	KEGG	%Similarity_KEGG	Score_KEGG	Evalue_KEGG
696	Prokka:JAPCLKMA_02906	-	Beta-galactosidase III	1	1	1	1	1	1	1	1	1	1	1	11	CORE	COG1874_G#	38.2	474	6.03E-133	K12308	33.6	366	6.18E-100
697	OSC70749.1	-	phosphatase	1	1	1	1	1	1	1	1	1	1	1	11	CORE	COG3211_R#	42.4	491	4.77E-138				
698	OSC65438.1	-	PAS sensor protein	0	1	0	0	0	0	0	0	0	0	0	1	Unique	COG2208_TK#	33.6	119	4.92E-26	NA	32.6	120	5.89E-26
699	Prokka:JAPCLKMA_03799	-	hypothetical protein	1	0	0	0	0	0	0	0	0	0	0	1	Unique								
700	WP_051724458.1	-	hypothetical protein	0	0	0	0	0	0	1	0	0	0	0	1	Unique								
701	PWE11241.1	-	hypothetical protein	0	0	1	0	0	0	0	0	0	0	0	1	Unique								
702	OSC70108.1	-	beta keto-acyl synthase	0	1	0	0	0	0	0	0	0	0	0	1	Unique	COG3321_Q#	41	476	2.06E-133	K12444	35.1	307	4.42E-82
703	WP_122216268.1	-	cellulase family glycosylhydrolase	0	0	0	0	0	0	0	0	0	1	0	1	Unique	COG2730_G#	46.2	403	2.22E-111	NA	46.4	416	6.76E-115
704	WP_122213366.1	-	alpha-galactosidase	0	0	0	0	0	0	0	0	0	1	0	1	Unique					K07407	37.6	215	1.34E-54
705	WP_102637025.1	-	SpoIIE family protein phosphatase	1	1	0	1	1	1	1	1	1	1	1	10	Accessory	COG2208_TK#	36	133	2.5E-30	NA	35.5	133	6.69E-30
706	WP_033272803.1	-	DUF2029 domain-containing protein	1	1	1	1	1	1	1	1	1	1	1	11	CORE					K13671	27.4	101	4.8E-20
707	WP_122213461.1	-	SpoIIE family protein phosphatase	0	1	0	1	1	1	1	1	1	1	1	9	Accessory	COG2208_TK#	33.1	108	1.13E-22	NA	30.6	109	1.76E-22

cluster	GI	gene	Annotation	Streptomyces_thermocarboxydus_BPSAC147	Streptomyces_sp._4F	Streptomyces_sp._BSE7F	Streptomyces_sp._ETH9427	Streptomyces_sp._FxanaD5	Streptomyces_sp._UNC401CLCol	Actinospica_acidiphila_NRRL_B_24431	Streptomyces_sp._HNS054	Streptomyces_sp._SMS_SU21	Streptomyces_sp._ZS0098	Streptomyces_thermocarboxydus_K155	Addition	Core-Accessory-Unique	COG	%Similarity_COG	Score_COG	Evalue_COG	KEGG	%Similarity_KEGG	Score_KEGG	Evalue_KEGG
708	OSC73434.1	-	transcription termination factor Rho	1	1	1	1	1	1	1	1	1	1	1	11	CORE	COG1158_K#	70.1	607	7.73E-173	K03628	56.8	631	1.33E-179
709	WP_122215934.1	-	ATP-binding protein	0	0	0	0	0	0	0	0	0	1	0	1	Unique								
710	PWE06821.1	-	hypothetical protein	1	1	1	1	1	1	1	1	1	1	1	11	CORE	COG0457_R#	22	59	7.83E-08	K15837	24.2	66	1E-09
711	PWE10803.1	-	iron ABC transporter permease	1	1	1	1	1	1	1	1	1	1	1	11	CORE	COG0609_P#	35.8	354	9.01E-97	K02015	34.4	339	6.14E-92
712	WP_019523447.1	-	neutral/alkaline ceramidase	1	0	1	1	1	1	1	1	1	1	1	10	Accessory					K12349	53.9	671	8.9E-192
713	OSC68288.1	-	hypothetical protein	0	1	0	0	0	0	0	0	0	0	0	1	Unique								
714	Prokka:CIODPCDJ_03501	-	hypothetical protein	0	0	0	0	0	0	0	0	0	0	1	1	Unique								
715	Prokka:JAPCLKMA_05582	-	hypothetical protein	1	0	0	0	0	0	0	0	0	0	0	1	Unique	COG3882_Q#	30.4	144	1.41E-33	NA	26.6	125	2.37E-27
716	Prokka:JAPCLKMA_02733	-	hypothetical protein	1	0	0	0	0	0	0	0	0	0	0	1	Unique								
717	WP_122215772.1	-	hypothetical protein	1	0	0	0	0	0	0	0	0	1	1	3	Accessory								
718	WP_107080812.1	-	thioredoxin domain-containing protein	1	1	1	1	1	1	1	1	1	1	1	11	CORE	COG1331_O#	52.9	624	3.56E-178	K06888	52.9	624	9.51E-178
719	Prokka:CIODPCDJ_02877	-	hypothetical protein	1	0	0	1	1	1	0	0	1	1	1	7	Accessory	COG1715_V#	30.7	65	1.09E-09	K07448	33.1	60	7.15E-08

cluster	GI	gene	Annotation	Streptomyces_thermocarboxydus_BPSAC147	Streptomyces_sp._4F	Streptomyces_sp._BSE7F	Streptomyces_sp._ETH9427	Streptomyces_sp._FxanaD5	Streptomyces_sp._UNC401CLCol	Actinospica_acidiphila_NRRL_B_24431	Streptomyces_sp._HNS054	Streptomyces_sp._SMS_SU21	Streptomyces_sp._ZS0098	Streptomyces_thermocarboxydus_K155	Addition	Core-Accessory-Unique	COG	%Similarity_COG	Score_COG	Evalue_COG	KEGG	%Similarity_KEGG	Score_KEGG	Evalue_KEGG
720	WP_019524111.1	-	AAA family ATPase	0	0	0	0	1	0	0	0	0	0	0	1	Unique	COG0419_L#	28.8	307	1.64E-82	K03546	20.2	64	6.47E-09
721	OSC71417.1	-	molybdopterin oxidoreductase	0	1	0	0	0	0	0	0	0	0	0	1	Unique	COG0243_C#	34.8	417	8.63E-116	NA	27.7	249	8.31E-65
722	AXI91318.1	-	hypothetical protein	0	0	0	1	0	0	0	0	0	0	0	1	Unique								
723	OSC71192.1	-	flavin-binding oxidoreductase	0	1	0	0	0	0	0	0	0	0	0	1	Unique	COG1231_E#	38.1	104	1.24E-21	K03334	30.1	136	1.02E-30
724	WP_102637404.1	-	Hsp70 family protein	0	0	0	0	0	0	0	0	1	0	0	1	Unique	COG0443_O#	31.2	216	2.23E-55	K04045	31.8	200	7.53E-50
725	PWE12365.1	-	phospholipase C, phosphocholine-specific	0	1	1	1	0	1	0	1	1	1	1	8	Accessory	COG3511_M#	59.7	842	1.08E-243	K01114	40.6	306	7.46E-82
726	WP_102637948.1	-	hypothetical protein	0	0	0	0	0	0	0	0	1	0	0	1	Unique								
727	WP_159020887.1	-	tetratricopeptide repeat protein	0	0	0	0	0	0	0	1	0	0	0	1	Unique	COG0457_R#	24.4	87	2.04E-16	NA	25.6	75	1.64E-12
728	WP_084207002.1	-	serine/threonine-protein kinase	0	0	0	0	0	0	1	0	0	0	0	1	Unique	COG0515_RTKL#	32.8	130	2.1E-29	K08884	28.4	127	4.75E-28
729	AXI89777.1	fusA	elongation factor G	1	1	1	1	1	1	1	1	1	1	1	11	CORE	COG0480_J#	49.9	647	6.67E-185	K02355	49.9	647	1.78E-184
730	PWE09468.1	-	peptidase M4	1	1	1	1	1	1	1	1	1	1	1	11	CORE	COG3227_E#	31.1	191	1E-47	K08777	31.1	191	2.67E-47
731	Prokka:CIODPCDJ_05842	shc	Squalene--hopene cyclase	1	1	1	1	1	1	1	1	1	1	1	11	CORE	COG1657_I#	45.1	526	1.31E-148	K06045	45.1	526	3.5E-148

cluster	GI	gene	Annotation	Streptomyces_thermocarboxydus_BPSAC147	Streptomyces_sp._4F	Streptomyces_sp._BSE7F	Streptomyces_sp._ETH9427	Streptomyces_sp._FxanaD5	Streptomyces_sp._UNC401CLCol	Actinospica_acidiphila_NRRL_B_24431	Streptomyces_sp._HNS054	Streptomyces_sp._SMS_SU21	Streptomyces_sp._ZS0098	Streptomyces_thermocarboxydus_K155	Addition	Core-Accessory-Unique	COG	%Similarity_COG	Score_COG	Evalue_COG	KEGG	%Similarity_KEGG	Score_KEGG	Evalue_KEGG
732	WP_019523537.1	-	flotillin family protein	1	1	1	1	1	1	1	1	1	1	1	11	CORE	COG2268_S#	50.7	518	2.74E-146	NA	27.6	177	5.21E-43
733	AXI85285.1	fxlM	methyltransferase, FxLD system	0	0	0	1	0	0	0	0	0	0	0	1	Unique	COG2518_O#	36	136	2.92E-31	K00573	36.7	128	2.13E-28
734	WP_159020889.1	-	hypothetical protein	0	0	0	0	0	0	0	1	0	0	0	1	Unique								
735	AXI85382.1	-	ATP-binding protein	0	0	0	1	0	0	0	0	0	0	0	1	Unique								
736	Prokka:CIODPCDJ_01915	-	hypothetical protein	1	0	1	0	1	1	1	1	1	1	1	9	Accessory	COG0642_T#	28.7	106	3.24E-22	NA	28.7	106	8.64E-22
737	PWE09789.1	-	hypothetical protein	0	0	1	0	0	0	0	0	0	0	0	1	Unique								
738	OSC70531.1	-	family 31 glucosidase	0	1	0	0	0	0	0	0	0	0	0	1	Unique	COG1501_G#	48.2	646	8.69E-185	K01187	30	237	3.25E-61
739	Prokka:JAPCLKMA_03590	ftsH	ATP-dependent zinc metalloprotease FtsH	1	1	1	1	1	1	1	1	1	1	1	11	CORE	COG0465_O#	57.2	725	1.47E-208	K03798	57.2	725	5.12E-208
740	PWE08141.1	-	AAA family ATPase	1	1	1	1	0	1	1	1	1	1	1	10	Accessory	COG3973_R#	34	166	3.44E-40	K03657	26.2	166	1.2E-39
741	Prokka:JAPCLKMA_01616	-	hypothetical protein	1	0	0	0	0	0	0	0	0	0	0	1	Unique	COG3336_S#	33.7	338	6.6E-92	K02351	33.7	338	1.76E-91
742	WP_122216361.1	-	bifunctional aldolase/short-chain dehydrogenase	1	1	1	1	1	1	1	1	1	1	1	11	CORE	COG3347_S#	50.7	399	3.14E-110	K00540	40	511	1.52E-143

cluster	GI	gene	Annotation	Streptomyces_thermocarboxydus_BPSAC147	Streptomyces_sp._4F	Streptomyces_sp._BSE7F	Streptomyces_sp._ETH9427	Streptomyces_sp._FxanaD5	Streptomyces_sp._UNC401CLCol	Actinospica_acidiphila_NRRL_B_24431	Streptomyces_sp._HNS054	Streptomyces_sp._SMS_SU21	Streptomyces_sp._ZS0098	Streptomyces_thermocarboxydus_K155	Addition	Core-Accessory-Unique	COG	%Similarity_COG	Score_COG	Evalue_COG	KEGG	%Similarity_KEGG	Score_KEGG	Evalue_KEGG
743	PWE07676.1	-	glycosyl transferase	1	1	1	1	1	1	1	1	1	1	1	11	CORE	COG1215_M#	29.5	175	5.66E-43	NA	61.1	45	0.00236
744	Prokka:CIODPCDJ_02363	accA1_1	Acetyl-/propionyl-coenzyme A carboxylase alpha chain	1	1	0	1	1	1	1	1	1	1	1	10	Accessory	COG4770_I#	56.2	677	5.98E-194	K11263	56.2	677	1.6E-193
745	WP_159020821.1	-	hypothetical protein	0	0	0	0	0	0	0	1	0	0	0	1	Unique								
746	WP_147474385.1	-	hypothetical protein	0	0	0	0	0	0	0	0	0	1	0	1	Unique								
747	AXI90261.1	-	ricin-type beta-trefoil lectin domain protein	0	1	1	1	0	0	0	1	0	0	0	4	Accessory					NA	21.8	82	2.27E-14
748	WP_102640752.1	-	protein meaA	1	1	1	1	1	1	1	1	1	1	1	11	CORE	COG1884_I#	63.8	682	1.85E-195	K01847	37.9	406	5.24E-112
749	WP_048457985.1	-	serine/threonine protein kinase	1	1	1	1	1	1	1	1	1	1	1	11	CORE	COG0515_RTKL#	32.6	175	7.35E-43	K08884	32.6	175	1.96E-42
750	Prokka:CIODPCDJ_06759	-	hypothetical protein	0	0	0	0	0	0	0	0	0	0	1	1	Unique								
751	WP_019527712.1	dndD	DNA sulfur modification protein DndD	0	0	0	0	1	0	0	0	0	0	0	1	Unique	COG0419_L#	34	350	1.67E-95				
752	WP_158688225.1	-	AAA family ATPase	0	0	0	0	1	0	0	0	0	0	0	1	Unique	COG3593_L#	31.3	136	4.94E-31				
753	Prokka:CIODPCDJ_06760	-	hypothetical protein	0	0	0	0	0	0	0	0	0	0	1	1	Unique								

cluster	GI	gene	Annotation	Streptomyces_thermocarboxydus_BPSAC147	Streptomyces_sp._4F	Streptomyces_sp._BSE7F	Streptomyces_sp._ETH9427	Streptomyces_sp._FxanaD5	Streptomyces_sp._UNC401CLCol	Actinospica_acidiphila_NRRL_B_24431	Streptomyces_sp._HNS054	Streptomyces_sp._SMS_SU21	Streptomyces_sp._ZS0098	Streptomyces_thermocarboxydus_K155	Addition	Core-Accessory-Unique	COG	%Similarity_COG	Score_COG	Evalue_COG	KEGG	%Similarity_KEGG	Score_KEGG	Evalue_KEGG
754	WP_147474403.1	-	hypothetical protein	0	0	0	0	0	0	0	0	0	1	0	1	Unique								
755	OSC69517.1	-	serine/threonine protein kinase	1	1	1	1	1	1	1	1	1	1	1	11	CORE	COG0515_RTKL#	49.3	393	1.31E-108	K08884	42.8	447	2.67E-124
756	WP_033273175.1	-	VWA domain-containing protein	0	1	0	0	0	0	1	0	1	1	0	4	Accessory	COG1674_D#	34.4	120	2.15E-26	NA	35.6	170	4.83E-41
757	WP_053076985.1	-	M48 family metalloprotease	0	0	0	1	0	0	0	1	0	0	0	2	Accessory								
758	Prokka:CIODPCDJ_00559	-	Beta-galactosidase	1	0	0	0	1	1	0	0	0	0	1	4	Accessory	COG1874_G#	39.1	492	2.71E-138	K12308	32.7	369	9.24E-101
759	WP_102637023.1	-	NADPH-dependent 2,4-dienoyl-CoA reductase	1	1	0	1	1	1	1	1	1	1	1	10	Accessory	COG1902_C#	72.2	521	4.16E-147	K00219	61.8	844	5.73E-244
760	WP_102637157.1	-	SpoIIE family protein phosphatase	0	0	1	1	1	1	0	1	1	1	1	8	Accessory	COG2202_T#	29.5	107	1.87E-22	NA	36.2	89	1.41E-16
761	WP_078492914.1	dnaA	chromosomal replication initiator protein DnaA	1	0	0	1	1	1	1	1	1	1	1	9	Accessory	COG0593_L#	71.9	481	3.65E-135	K02313	71.9	481	9.75E-135
762	WP_051724701.1	-	amino acid adenylation domain-containing protein	0	0	0	0	0	0	1	0	0	0	0	1	Unique	COG1020_Q#	41.3	409	2.3E-113	K04780	41.3	409	6.14E-113

cluster	GI	gene	Annotation	Streptomyces_thermocarboxydus_BPSAC147	Streptomyces_sp._4F	Streptomyces_sp._BSE7F	Streptomyces_sp._ETH9427	Streptomyces_sp._FxanaD5	Streptomyces_sp._UNC401CLCol	Actinospica_acidiphila_NRRL_B_24431	Streptomyces_sp._HNS054	Streptomyces_sp._SMS_SU21	Streptomyces_sp._ZS0098	Streptomyces_thermocarboxydus_K155	Addition	Core-Accessory-Unique	COG	%Similarity_COG	Score_COG	Evalue_COG	KEGG	%Similarity_KEGG	Score_KEGG	Evalue_KEGG
763	OSC71272.1	-	DNA helicase RecQ	1	1	1	1	1	1	1	1	1	1	1	11	CORE	COG0514_L#	59.4	699	1.45E-200	K03654	54	662	5.26E-189
764	WP_122215768.1	-	SAM-dependent DNA methyltransferase	1	1	0	0	0	0	0	0	1	1	1	5	Accessory	COG0286_V#	58.1	535	2.12E-151	K03427	30.7	134	4.97E-30
765	WP_078504706.1	-	AAA family ATPase	0	0	0	0	1	0	0	0	0	0	0	1	Unique	COG3593_L#	28.5	86	3.4E-16				
766	WP_019522142.1	-	putative cobaltochelatase	1	1	1	1	1	1	1	1	1	1	1	11	CORE	COG1239_H#	57.9	380	1.14E-104	K03404	55.1	572	5.45E-162
767	WP_033274780.1	-	ATP-binding protein	0	1	0	0	0	0	1	0	0	0	0	2	Accessory	COG0464_O#	39.9	230	1.9E-59	K13525	31.1	98	2.3E-19
768	WP_048459190.1	-	AIPR family protein	0	0	0	0	0	0	0	1	0	0	0	1	Unique								
769	WP_102639043.1	-	TerD family protein	1	1	1	1	1	1	1	1	1	1	1	11	CORE	COG2310_T#	29.4	70	1.92E-11	NA	27.6	59	1.55E-07
770	OSC72948.1	-	histidine kinase	1	1	1	1	1	1	1	1	1	1	1	11	CORE	COG5001_T#	36.6	263	1.55E-69	NA	37.3	285	1.33E-75
771	Prokka:JAPCLKMA_05488	mhpE	4-hydroxy-2-oxovalerate aldolase	1	0	0	0	0	0	0	0	0	0	0	1	Unique	COG0119_E#	30.3	115	6.81E-25	K01666	30.3	115	1.82E-24
772	Prokka:JAPCLKMA_05441	-	hypothetical protein	1	1	1	1	1	1	1	1	1	1	1	11	CORE	COG2508_TQ#	24.6	115	8.88E-25	K02647	23.8	64	3.68E-09
773	WP_122215267.1	-	NADH-quinone oxidoreductase subunit L	1	1	1	1	1	1	1	1	1	1	1	11	CORE	COG1009_CP#	40.6	327	1.49E-88	K00341	38	306	7.26E-82

cluster	GI	gene	Annotation	Streptomyces_thermocarboxydus_BPSAC147	Streptomyces_sp._4F	Streptomyces_sp._BSE7F	Streptomyces_sp._ETH9427	Streptomyces_sp._FxanaD5	Streptomyces_sp._UNC401CLCol	Actinospica_acidiphila_NRRL_B_24431	Streptomyces_sp._HNS054	Streptomyces_sp._SMS_SU21	Streptomyces_sp._ZS0098	Streptomyces_thermocarboxydus_K155	Addition	Core-Accessory-Unique	COG	%Similarity_COG	Score_COG	Evalue_COG	KEGG	%Similarity_KEGG	Score_KEGG	Evalue_KEGG
774	PWE09721.1	-	ATP-dependent DNA helicase	1	1	1	1	1	1	1	1	1	1	1	11	CORE	COG1199_KL#	53.3	650	5.87E-186	K03722	53.3	650	1.57E-185
775	OSC68287.1	-	B12-binding domain-containing radical SAM protein	1	1	1	1	1	1	1	1	1	1	1	11	CORE	COG1032_C#	41.6	509	1.62E-143	NA	41.6	509	4.32E-143
776	WP_109288709.1	-	serine/threonine protein kinase	1	1	1	1	1	1	1	1	1	1	1	11	CORE	COG0515_RTKL#	33	156	3.48E-37	NA	36	149	8.69E-35
777	AXI91342.1	-	hypothetical protein	0	0	0	1	0	0	0	0	0	0	0	1	Unique	COG1674_D#	32.5	54	0.00000243				
778	Prokka:JAPCLKMA_06813	xerC_5	Tyrosine recombinase XerC	1	0	0	0	0	0	0	0	0	0	0	1	Unique								
779	AXI84713.1	-	long-chain fatty acid--CoA ligase	0	0	1	1	0	0	0	1	1	1	0	5	Accessory	COG0318_IQ#	42.4	382	2.27E-105	NA	33	267	4.86E-70
780	Prokka:JAPCLKMA_03181	btuD_9	Vitamin B12 import ATP-binding protein BtuD	1	0	0	1	0	0	1	1	1	1	1	7	Accessory	COG0488_R#	34	273	1.94E-72	NA	35.1	247	3.04E-64
781	WP_102639968.1	-	DUF5107 domain-containing protein	1	1	1	1	1	1	1	1	1	1	1	11	CORE					NA	25.2	101	2.7E-20
782	OSC57554.1	-	hypothetical protein	0	1	0	0	0	0	0	0	0	0	0	1	Unique								
783	OSC70810.1	-	ABC transporter	0	1	0	0	0	0	1	0	0	0	0	2	Accessory	COG0444_EP#	63.7	375	3.62E-103	K12371	45.2	263	5.35E-69

cluster	GI	gene	Annotation	Streptomyces_thermocarboxydus_BPSAC147	Streptomyces_sp._4F	Streptomyces_sp._BSE7F	Streptomyces_sp._ETH9427	Streptomyces_sp._FxanaD5	Streptomyces_sp._UNC401CLCol	Actinospica_acidiphila_NRRL_B_24431	Streptomyces_sp._HNS054	Streptomyces_sp._SMS_SU21	Streptomyces_sp._ZS0098	Streptomyces_thermocarboxydus_K155	Addition	Core-Accessory-Unique	COG	%Similarity_COG	Score_COG	Evalue_COG	KEGG	%Similarity_KEGG	Score_KEGG	Evalue_KEGG
784	PWE11053.1	-	hypothetical protein	0	0	1	0	0	0	0	0	0	0	0	1	Unique								
785	WP_048458044.1	-	histidine kinase	0	0	0	0	0	0	0	1	0	0	0	1	Unique								
786	Prokka:CIODPCDJ_01097	thrS_1	Threonine--tRNA ligase	1	1	1	1	1	1	1	1	1	1	1	11	CORE	COG0441_J#	66.5	876	8.52E-254	K01868	66.5	876	2.27E-253
787	WP_019526443.1	-	acetoacetate--CoA ligase	1	1	1	1	1	1	1	1	1	1	1	11	CORE	COG0365_I#	45.2	584	6.69E-166	K01907	39	474	1.53E-132
788	PWE09278.1	-	hypothetical protein	1	1	1	1	1	1	1	1	1	1	1	11	CORE	COG0532_J#	29.6	103	3.46E-21	NA	34.6	124	2.97E-27
789	OSC71094.1	-	beta-galactosidase	1	1	1	1	1	1	1	1	1	1	1	11	CORE	COG1874_G#	38	457	9.44E-128	K12308	32	369	5.3E-101
790	Prokka:JAPCLKMA_01426	-	hypothetical protein	1	1	1	1	1	1	1	1	1	1	1	11	CORE	COG1506_E#	40.8	463	1.01E-129	NA	41.1	463	2.69E-129
791	Prokka:JAPCLKMA_06206	tetO	Tetracycline resistance protein TetO	1	0	1	0	1	1	0	0	1	1	1	7	Accessory	COG0480_J#	46.6	559	1.76E-158	K18220	38.8	450	2.36E-125
792	WP_033276297.1	-	FAD/NAD(P)-binding protein	1	1	0	0	1	1	1	0	0	0	1	6	Accessory								
793	WP_102637115.1	-	heavy metal translocating P-type ATPase	0	1	1	0	1	1	1	1	1	1	1	9	Accessory	COG2217_P#	62.3	757	3.37E-218	NA	62.4	757	9E-218
794	WP_048456034.1	dxs	1-deoxy-D-xylulose-5-phosphate synthase	1	1	1	1	1	1	1	1	1	1	1	11	CORE	COG1154_HI#	58	743	5.04E-214	K01662	58	743	1.34E-213
795	WP_122213813.1	-	sporulation protein SsgA	0	0	0	0	0	0	0	0	0	1	0	1	Unique								

cluster	GI	gene	Annotation	Streptomyces_thermocarboxydus_BPSAC147	Streptomyces_sp._4F	Streptomyces_sp._BSE7F	Streptomyces_sp._ETH9427	Streptomyces_sp._FxanaD5	Streptomyces_sp._UNC401CLCol	Actinospica_acidiphila_NRRL_B_24431	Streptomyces_sp._HNS054	Streptomyces_sp._SMS_SU21	Streptomyces_sp._ZS0098	Streptomyces_thermocarboxydus_K155	Addition	Core-Accessory-Unique	COG	%Similarity_COG	Score_COG	Evalue_COG	KEGG	%Similarity_KEGG	Score_KEGG	Evalue_KEGG
796	WP_122216927.1	-	penicillin-binding protein 2	1	1	0	1	1	1	1	1	1	1	1	10	Accessory	COG0768_M#	32.7	321	8.05E-87	K03587	32.7	319	6.26E-86
797	OSC71268.1	-	NADH-quinone oxidoreductase subunit L	1	1	1	1	1	1	1	1	1	1	1	11	CORE	COG1009_CP#	62	759	8.84E-219	K00341	62	759	2.36E-218
798	WP_033276461.1	-	N-acetylmuramoyl-L-alanine amidase	1	1	1	1	1	1	1	1	1	1	1	11	CORE								
799	Prokka:CIODPCDJ_05577	norW	Nitric oxide reductase FlRd-NAD(+) reductase	1	1	1	1	1	1	1	1	1	1	1	11	CORE	COG1252_C#	31.3	169	2.99E-41	K03885	31.3	169	7.98E-41
800	WP_048459424.1	-	putative baseplate assembly protein	0	1	0	1	0	0	1	1	1	1	0	6	Accessory								
801	OSC72274.1	-	hypothetical protein	0	1	0	0	0	0	0	0	0	0	0	1	Unique								
802	AXI84570.1	-	ABC transporter ATP-binding protein	0	1	0	1	0	0	0	1	0	0	0	3	Accessory	COG1132_V#	28.9	176	4.17E-43	K11085	40.7	152	1.72E-35
803	OSC68247.1	-	Xaa-Pro dipeptidyl-peptidase	1	1	1	1	1	1	1	1	0	0	1	9	Accessory					K01281	29.8	156	9.13E-37
804	WP_033274060.1	-	alpha-glucuronidase	1	0	1	1	1	1	1	1	1	1	1	10	Accessory	COG3661_G#	51.1	649	1.68E-185	K01235	37.9	397	2.35E-109
805	OSC58098.1	-	transporter	1	1	1	1	1	1	1	1	1	1	1	11	CORE	COG1172_G#	60.5	357	1.32E-97	K10440	48.3	278	2.08E-73

cluster	GI	gene	Annotation	Streptomyces_thermocarboxydus_BPSAC147	Streptomyces_sp._4F	Streptomyces_sp._BSE7F	Streptomyces_sp._ETH9427	Streptomyces_sp._FxanaD5	Streptomyces_sp._UNC401CLCol	Actinospica_acidiphila_NRRL_B_24431	Streptomyces_sp._HNS054	Streptomyces_sp._SMS_SU21	Streptomyces_sp._ZS0098	Streptomyces_thermocarboxydus_K155	Addition	Core-Accessory-Unique	COG	%Similarity_COG	Score_COG	Evalue_COG	KEGG	%Similarity_KEGG	Score_KEGG	Evalue_KEGG
806	Prokka:JAPCLKMA_03769	-	hypothetical protein	1	0	0	0	0	0	0	0	0	0	0	1	Unique								
807	AXI90637.1	-	lantibiotic dehydratase	0	0	0	1	0	0	0	0	0	0	0	1	Unique								
808	WP_033274875.1	acs	acetate--CoA ligase	1	1	1	1	1	1	1	1	1	1	1	11	CORE	COG0365_I#	66.8	875	1.44E-253	K01895	66.8	875	3.84E-253
809	WP_033277299.1	-	metallophosphoesterase family protein	0	0	0	0	0	0	1	0	0	0	0	1	Unique	COG1409_R#	33.1	261	7.52E-69	NA	33.1	261	2.01E-68
810	Prokka:JAPCLKMA_04029	kimA_2	Potassium transporter KimA	1	0	0	0	1	1	0	0	0	0	1	4	Accessory	COG0531_E#	35.3	361	9.1E-99	NA	33.1	343	5.24E-93
811	WP_078895305.1	-	PQQ-binding-like beta-propeller repeat protein	0	0	0	0	0	0	0	1	0	0	0	1	Unique	COG0515_RTKL#	26.7	111	9.61E-24	K08884	32.2	98	2.94E-19
812	Prokka:CIODPCDJ_02442	-	hypothetical protein	0	0	0	0	0	1	0	0	0	0	1	2	Accessory					NA	36.7	48	0.000267
813	Prokka:CIODPCDJ_04117	-	hypothetical protein	1	1	1	1	1	1	1	1	1	1	1	11	CORE	COG4176_E#	34.4	334	1.19E-90	K02001	40.7	200	5.48E-50
814	WP_019521353.1	sdhA	fumarate reductase/succinate dehydrogenase flavoprotein subunit	1	1	1	1	1	1	1	1	1	1	1	11	CORE	COG1053_C#	50.2	651	2.58E-186	K00239	60.3	800	1.2E-230
815	Prokka:JAPCLKMA_01606	-	hypothetical protein	1	0	0	0	0	0	0	0	0	0	0	1	Unique	COG2372_R#	35	76	3.42E-13				

cluster	GI	gene	Annotation	Streptomyces_thermocarboxydus_BPSAC147	Streptomyces_sp._4F	Streptomyces_sp._BSE7F	Streptomyces_sp._ETH9427	Streptomyces_sp._FxanaD5	Streptomyces_sp._UNC401CLCol	Actinospica_acidiphila_NRRL_B_24431	Streptomyces_sp._HNS054	Streptomyces_sp._SMS_SU21	Streptomyces_sp._ZS0098	Streptomyces_thermocarboxydus_K155	Addition	Core-Accessory-Unique	COG	%Similarity_COG	Score_COG	Evalue_COG	KEGG	%Similarity_KEGG	Score_KEGG	Evalue_KEGG
816	WP_122216563.1	-	cellulase family glycosylhydrolase	1	0	0	1	1	1	1	1	1	1	1	9	Accessory								
817	WP_048459284.1	-	acyl-CoA dehydrogenase	1	1	1	1	1	1	1	1	1	1	1	11	CORE	COG1960_I#	68.8	848	1.88E-245	K00257	68.8	848	5.01E-245
818	WP_122216249.1	-	NADH-quinone oxidoreductase subunit L	0	0	0	1	0	0	0	1	1	1	0	4	Accessory	COG1009_CP#	32.7	241	1.05E-62	K00341	32.7	241	2.8E-62
819	OSC69991.1	-	fumarate reductase/succinate dehydrogenase flavoprotein subunit	1	1	1	1	1	1	1	1	1	1	1	11	CORE	COG1053_C#	71.3	923	3.51E-268	K00239	71.3	923	9.37E-268
820	OSC73331.1	-	cell division protein FtsH	0	1	0	0	0	0	0	0	0	0	0	1	Unique	COG0465_O#	44.9	516	1.69E-145	K03798	45.2	495	8.23E-139
821	Prokka:JAPCLKMA_06283	-	hypothetical protein	1	0	0	0	0	0	0	0	0	0	0	1	Unique								
822	WP_033274902.1	-	AfsR/SARP family transcriptional regulator	1	0	0	0	1	1	1	1	1	1	1	8	Accessory	COG3629_T#	36.1	132	5.24E-30	NA	36.1	132	1.4E-29
823	Prokka:CIODPCDJ_03792	-	hypothetical protein	1	1	1	1	1	1	1	1	1	1	1	11	CORE	COG3629_T#	35.7	131	1.17E-29	NA	35.7	131	3.12E-29
824	Prokka:JAPCLKMA_00680	-	hypothetical protein	1	0	0	0	1	1	1	1	1	1	1	8	Accessory								
825	OSC63952.1	-	acyl-CoA oxidase	0	1	0	0	0	0	0	0	0	0	0	1	Unique					K00232	27.3	184	3.1E-45

cluster	GI	gene	Annotation	Streptomyces_thermocarboxydus_BPSAC147	Streptomyces_sp._4F	Streptomyces_sp._BSE7F	Streptomyces_sp._ETH9427	Streptomyces_sp._FxanaD5	Streptomyces_sp._UNC401CLCol	Actinospica_acidiphila_NRRL_B_24431	Streptomyces_sp._HNS054	Streptomyces_sp._SMS_SU21	Streptomyces_sp._ZS0098	Streptomyces_thermocarboxydus_K155	Addition	Core-Accessory-Unique	COG	%Similarity_COG	Score_COG	Evalue_COG	KEGG	%Similarity_KEGG	Score_KEGG	Evalue_KEGG
826	WP_048460574.1	-	hypothetical protein	1	1	0	0	1	1	1	1	1	1	1	9	Accessory	COG2080_C#	50.8	138	1.25E-31	K03518	44	130	6.94E-29
827	WP_102640523.1	-	DUF1929 domain-containing protein	1	1	1	1	1	1	1	1	1	1	1	11	CORE								
828	AXI85287.1	-	ABC transporter	0	0	0	1	0	0	0	0	0	0	0	1	Unique	COG1132_V#	28.4	216	3.6E-55	K06148	29.2	208	2.62E-52
829	WP_033277164.1	asnB	asparagine synthase (glutamine-hydrolyzing)	1	0	1	0	1	1	1	1	1	1	1	9	Accessory	COG0367_E#	35.7	335	5.31E-91	K01953	29.1	286	5.78E-76
830	WP_048457374.1	-	PQQ-binding-like beta-propeller repeat protein	1	1	1	1	1	1	1	1	1	1	1	11	CORE	COG0810_M#	34.4	60	1.93E-08	NA	42.3	106	8.2E-22
831	Prokka:CIODPCDJ_05530	ywrD_2	Glutathione hydrolase-like YwrD proenzyme	1	1	1	1	1	1	1	1	1	1	1	11	CORE	COG0405_E#	53.5	626	1.15E-178	K00681	32.8	143	6.04E-33
832	WP_019524329.1	-	AAA family ATPase	0	0	0	0	1	0	0	0	0	0	0	1	Unique								
833	WP_019524328.1	-	ATP-dependent endonuclease	0	0	0	0	1	0	0	0	0	0	0	1	Unique	COG1132_V#	40.4	43	0.00319				
834	WP_147474405.1	-	VWA domain-containing protein	1	1	1	1	1	1	1	1	1	1	1	11	CORE	COG1975_O#	60.9	412	3.41E-114	K07402	60.9	412	9.11E-114

cluster	GI	gene	Annotation	Streptomyces_thermocarboxydus_BPSAC147	Streptomyces_sp._4F	Streptomyces_sp._BSE7F	Streptomyces_sp._ETH9427	Streptomyces_sp._FxanaD5	Streptomyces_sp._UNC401CLCol	Actinospica_acidiphila_NRRL_B_24431	Streptomyces_sp._HNS054	Streptomyces_sp._SMS_SU21	Streptomyces_sp._ZS0098	Streptomyces_thermocarboxydus_K155	Addition	Core-Accessory-Unique	COG	%Similarity_COG	Score_COG	Evalue_COG	KEGG	%Similarity_KEGG	Score_KEGG	Evalue_KEGG
835	WP_048459189.1	dcm	DNA cytosine methyltransferase	0	0	0	0	0	0	0	1	0	0	0	1	Unique	COG0270_L#	31.9	166	3.26E-40	K00558	31.9	166	8.7E-40
836	AXI91216.1	cas8c	type I-C CRISPR-associated protein Cas8c/Csd1	0	0	0	1	0	1	0	0	0	0	0	2	Accessory								
837	OSC61898.1	-	hypothetical protein	1	1	1	1	1	1	1	1	1	1	1	11	CORE								
838	OSC72293.1	-	long-chain fatty acid--CoA ligase	1	1	1	1	1	1	1	1	1	1	1	11	CORE	COG1022_I#	44.7	495	3.99E-139	K01897	44.6	493	2.38E-138
839	Prokka:CIODPCDJ_03908	korA	2-oxoglutarate oxidoreductase subunit KorA	1	1	1	1	1	1	1	0	1	1	1	10	Accessory	COG0674_C#	66.1	549	1.37E-155	K00174	66.8	837	1.14E-241
840	WP_102639452.1	-	MFS transporter	1	1	1	1	1	1	1	1	1	1	1	11	CORE	COG0477_GEPR#	27.8	158	6.78E-38	K08369	28.9	145	2.07E-33
841	WP_026243579.1	-	ABC transporter ATP-binding protein	1	1	1	1	1	1	1	1	1	1	1	11	CORE	COG1132_V#	57.4	699	1.07E-200	K06147	57.4	699	2.85E-200
842	WP_019521520.1	-	WD40 repeat domain-containing protein	1	0	0	0	1	1	0	0	0	0	1	4	Accessory								

cluster	GI	gene	Annotation	Streptomyces_thermocarboxydus_BPSAC147	Streptomyces_sp._4F	Streptomyces_sp._BSE7F	Streptomyces_sp._ETH9427	Streptomyces_sp._FxanaD5	Streptomyces_sp._UNC401CLCol	Actinospica_acidiphila_NRRL_B_24431	Streptomyces_sp._HNS054	Streptomyces_sp._SMS_SU21	Streptomyces_sp._ZS0098	Streptomyces_thermocarboxydus_K155	Addition	Core-Accessory-Unique	COG	%Similarity_COG	Score_COG	Evalue_COG	KEGG	%Similarity_KEGG	Score_KEGG	Evalue_KEGG
843	WP_122216939.1	pknB	Stk1 family PASTA domain-containing Ser/Thr kinase	1	1	1	1	1	1	1	1	1	1	1	11	CORE	COG0515_RTKL#	47.2	285	3.67E-76	K08884	32.6	297	3.26E-79
844	OSC72870.1	-	DNA primase	1	1	1	1	1	1	1	1	1	1	1	11	CORE	COG0358_L#	47.3	557	8.54E-158	K02316	47.3	557	2.28E-157
845	WP_028961419.1	-	molybdopterin-dependent oxidoreductase	1	1	1	1	1	1	1	1	1	1	1	11	CORE	COG0243_C#	41.1	467	6.81E-131	K00123	38.1	433	2.91E-120
846	WP_102637435.1	-	DUF262 domain-containing protein	0	0	0	0	0	0	0	0	1	0	1	2	Accessory	COG1479_S#	31.3	110	2.11E-23	NA	32.7	55	0.00000215
847	OSC61151.1	-	thiazolinyl imide reductase	0	1	0	0	0	0	0	0	0	0	0	1	Unique	COG4693_Q#	39.2	233	1.65E-60				
848	WP_102639936.1	-	two-component sensor histidine kinase	0	0	0	0	0	0	0	0	1	0	0	1	Unique	COG4585_T#	30.4	117	1.73E-25	NA	30.4	117	4.61E-25
849	Prokka:CIODPCDJ_05189	-	hypothetical protein	1	0	1	1	1	1	1	1	1	1	1	10	Accessory								
850	WP_106959445.1	-	SMI1/KNR4 family protein	1	0	0	0	1	1	0	0	0	0	1	4	Accessory								
851	AXI89147.1	-	ankyrin repeat domain-containing protein	1	0	1	1	0	1	0	1	0	1	1	7	Accessory	COG1511_S#	45.7	45	0.000833	NA	34.5	62	2.3E-08

cluster	GI	gene	Annotation	Streptomyces_thermocarboxydus_BPSAC147	Streptomyces_sp._4F	Streptomyces_sp._BSE7F	Streptomyces_sp._ETH9427	Streptomyces_sp._FxanaD5	Streptomyces_sp._UNC401CLCol	Actinospica_acidiphila_NRRL_B_24431	Streptomyces_sp._HNS054	Streptomyces_sp._SMS_SU21	Streptomyces_sp._ZS0098	Streptomyces_thermocarboxydus_K155	Addition	Core-Accessory-Unique	COG	%Similarity_COG	Score_COG	Evalue_COG	KEGG	%Similarity_KEGG	Score_KEGG	Evalue_KEGG
852	OSC70876.1	-	type IV secretion protein Rhs	0	1	0	0	0	0	1	0	0	0	0	2	Accessory	COG3500_R#	27.7	112	5.55E-24				
853	WP_122215484.1	-	NADH-quinone oxidoreductase subunit E	1	1	1	1	1	1	1	1	1	1	1	11	CORE	COG1894_C#	44.8	376	2.69E-103	K00335	47	357	2.02E-97
854	WP_019522307.1	typA	translational GTPase TypA	1	1	0	1	1	1	1	1	1	1	1	10	Accessory	COG1217_T#	62.3	787	5.05E-227	K06207	62.3	786	2.3E-226
855	Prokka:CIODPCDJ_06432	-	Long-chain-fatty-acid--CoA ligase FadD15	1	1	1	1	1	1	1	1	1	1	1	11	CORE	COG1022_I#	45.8	505	3.83E-142	K01897	45.8	505	1.02E-141
856	WP_122217070.1	-	reverse transcriptase-like protein	1	0	1	1	1	1	1	1	1	1	1	10	Accessory	COG0406_G#	52.4	202	5.31E-51	K15634	51.1	214	3.61E-54
857	WP_102640686.1	-	potassium transporter TrkA	1	1	0	1	1	1	1	1	1	1	1	10	Accessory	COG1226_P#	25.2	92	4.53E-18	NA	25.2	92	1.21E-17
858	WP_122216479.1	-	aminotransferase	1	0	0	1	1	1	1	0	1	1	1	8	Accessory	COG3408_G#	35	345	5.06E-94				
859	OSC69707.1	-	ABC transporter permease	1	1	1	1	1	1	1	1	1	1	1	11	CORE	COG1132_V#	28.8	154	9.69E-37	K06147	23.3	157	5.21E-37
860	OSC72250.1	-	hypothetical protein	1	1	1	1	1	1	1	1	1	1	1	11	CORE								
861	OSC72657.1	-	long-chain fatty acid--CoA ligase	1	1	1	1	1	1	1	1	1	1	1	11	CORE	COG1022_I#	41.6	431	5.36E-120	K01897	41.6	431	1.43E-119
862	AXI90235.1	-	molecular chaperone HtpG	1	1	1	1	1	1	1	1	1	1	1	11	CORE	COG0326_O#	62.9	815	1.72E-235	K04079	62.8	815	4.6E-235

cluster	GI	gene	Annotation	Streptomyces_thermocarboxydus_BPSAC147	Streptomyces_sp._4F	Streptomyces_sp._BSE7F	Streptomyces_sp._ETH9427	Streptomyces_sp._FxanaD5	Streptomyces_sp._UNC401CLCol	Actinospica_acidiphila_NRRL_B_24431	Streptomyces_sp._HNS054	Streptomyces_sp._SMS_SU21	Streptomyces_sp._ZS0098	Streptomyces_thermocarboxydus_K155	Addition	Core-Accessory-Unique	COG	%Similarity_COG	Score_COG	Evalue_COG	KEGG	%Similarity_KEGG	Score_KEGG	Evalue_KEGG
863	OSC64029.1	-	PAS sensor protein	0	1	0	0	0	0	0	0	0	0	0	1	Unique	COG2208_TK#	34.1	119	3.44E-26	NA	33.3	125	2.19E-27
864	Prokka:CIODPCDJ_02280	hflX_1	GTPase HflX	1	1	1	1	1	1	1	1	1	1	1	11	CORE								
865	Prokka:JAPCLKMA_01733	nhaA_1	Na(+)/H(+) antiporter NhaA	1	0	1	1	1	1	1	1	1	1	1	10	Accessory	COG3004_P#	37.6	265	3.89E-70	K03313	33.5	253	4.08E-66
866	Prokka:JAPCLKMA_02013	menF_2	Isochorismate synthase MenF	1	1	0	0	0	0	0	0	0	0	0	2	Accessory	COG0147_EH#	56.7	483	1.19E-135				
867	OSC64980.1	-	hypothetical protein	0	1	0	0	0	0	0	0	0	0	0	1	Unique	COG2936_R#	26.3	78	8.78E-14	K06978	33.3	54	0.00000474
868	WP_102638996.1	dnaK	molecular chaperone DnaK	1	1	1	1	1	1	1	1	1	1	1	11	CORE	COG0443_O#	70.2	867	2.23E-251	K04043	68.9	861	7.28E-249
869	Prokka:JAPCLKMA_01642	-	Trehalase	1	1	1	1	1	1	1	1	1	1	1	11	CORE	COG3387_G#	50.7	576	1.34E-163	NA	37.2	382	7.59E-105
870	WP_037953287.1	-	ATP-dependent endonuclease	0	0	0	0	0	1	0	0	0	0	0	1	Unique	COG1120_PH#	39.6	42	0.00911				
871	WP_102639208.1	-	hypothetical protein	0	0	1	0	0	0	0	0	1	0	0	2	Accessory					K01127	25.2	80	8.06E-14
872	WP_028958806.1	-	DUF2264 domain-containing protein	1	1	1	1	1	1	1	1	1	1	1	11	CORE	COG4289_S#	29.4	179	4.77E-44	NA	27.6	151	2.84E-35
873	WP_019525847.1	-	hypothetical protein	1	0	0	0	1	1	0	0	0	0	1	4	Accessory	COG5337_M#	42.9	357	9.78E-98	NA	44.2	76	8.9E-13
874	OSC72420.1	-	NAD-binding lipoprotein	0	1	0	0	0	0	0	0	0	0	0	1	Unique	COG1226_P#	37	404	5.34E-112	NA	26.8	173	6.99E-42

cluster	GI	gene	Annotation	Streptomyces_thermocarboxydus_BPSAC147	Streptomyces_sp._4F	Streptomyces_sp._BSE7F	Streptomyces_sp._ETH9427	Streptomyces_sp._FxanaD5	Streptomyces_sp._UNC401CLCol	Actinospica_acidiphila_NRRL_B_24431	Streptomyces_sp._HNS054	Streptomyces_sp._SMS_SU21	Streptomyces_sp._ZS0098	Streptomyces_thermocarboxydus_K155	Addition	Core-Accessory-Unique	COG	%Similarity_COG	Score_COG	Evalue_COG	KEGG	%Similarity_KEGG	Score_KEGG	Evalue_KEGG
875	OSC69059.1	-	GTP-binding protein	0	1	0	0	0	0	0	0	0	0	0	1	Unique	COG0480_J#	43.5	446	1.6E-124	K18220	34.7	375	1.21E-102
876	WP_051724631.1	-	acyl-CoA dehydrogenase	0	0	0	0	0	0	1	0	0	0	0	1	Unique	COG1960_I#	35.2	231	8.08E-60	K00248	25.7	99	9.8E-20
877	OSC72558.1	-	ABC transporter	0	1	0	0	0	0	0	0	0	0	0	1	Unique	COG1132_V#	35.7	307	8.86E-83	K11085	35.7	307	2.37E-82
878	WP_048459188.1	-	DUF1998 domain-containing protein	0	0	0	0	0	0	0	1	0	0	0	1	Unique								
879	OSC56439.1	-	3D-(3,5/4)-trihydroxycyclohexane-1,2-dione acylhydrolase (decyclizing)	1	1	1	1	1	1	1	1	1	1	1	11	CORE	COG3962_E#	56.4	694	4.39E-199	K03336	39.3	460	3.72E-128
880	WP_084207039.1	-	ABC transporter ATP-binding protein	1	1	1	1	1	1	1	1	1	1	1	11	CORE	COG1132_V#	32.8	346	2.24E-94	K11085	32.8	346	5.99E-94
881	Prokka:JAPCLKMA_00406	-	hypothetical protein	1	1	0	1	1	1	1	1	1	1	1	10	Accessory					NA	33.3	59	1.46E-07
882	WP_048458857.1	-	DUF2075 domain-containing protein	0	0	0	0	0	0	0	1	0	0	0	1	Unique	COG3410_S#	30.7	64	2.22E-09				
883	WP_028959512.1	-	serine/threonine protein kinase	1	1	1	1	1	1	1	1	1	1	1	11	CORE	COG0515_RTKL#	32.1	159	5.07E-38	K08884	34.1	146	6.95E-34

cluster	GI	gene	Annotation	Streptomyces_thermocarboxydus_BPSAC147	Streptomyces_sp._4F	Streptomyces_sp._BSE7F	Streptomyces_sp._ETH9427	Streptomyces_sp._FxanaD5	Streptomyces_sp._UNC401CLCol	Actinospica_acidiphila_NRRL_B_24431	Streptomyces_sp._HNS054	Streptomyces_sp._SMS_SU21	Streptomyces_sp._ZS0098	Streptomyces_thermocarboxydus_K155	Addition	Core-Accessory-Unique	COG	%Similarity_COG	Score_COG	Evalue_COG	KEGG	%Similarity_KEGG	Score_KEGG	Evalue_KEGG
884	WP_051101576.1	-	GAF domain-containing protein	1	0	0	0	1	1	0	0	0	0	1	4	Accessory	COG2203_T#	31	82	7.87E-15	NA	28.3	60	6.55E-08
885	WP_158688228.1	-	hypothetical protein	0	0	0	0	1	0	0	0	0	0	0	1	Unique								
886	WP_122216942.1	-	phenazine-specific anthranilate synthase	0	0	0	1	1	1	1	1	1	1	1	8	Accessory	COG0147_EH#	55.1	432	3.1E-120				
887	AXI85292.1	-	radical SAM protein	0	0	0	1	0	0	0	0	0	0	0	1	Unique								
888	Prokka:CIODPCDJ_06779	-	hypothetical protein	1	1	1	1	1	1	1	1	1	1	1	11	CORE								
889	WP_028959120.1	-	repetitive protein	1	0	0	0	1	1	0	0	0	0	1	4	Accessory	COG2319_R#	37.2	86	5.43E-16	K03130	25.1	74	4.37E-12
890	OSC68924.1	-	hypothetical protein	0	1	0	0	0	0	0	0	0	0	0	1	Unique					K18197	57	725	3.61E-208
891	OSC72030.1	-	long-chain fatty acid--CoA ligase	1	1	1	1	1	1	1	1	1	1	1	11	CORE	COG1022_I#	37.9	389	1.76E-107	K01897	37.9	389	4.7E-107
892	Prokka:CIODPCDJ_05292	-	hypothetical protein	0	0	0	0	0	0	0	0	0	0	1	1	Unique								
893	WP_019525729.1	-	ABC transporter ATP-binding protein	1	0	0	0	1	1	1	0	0	0	1	5	Accessory	COG1132_V#	30	215	4.54E-55	K06148	30	215	1.21E-54
894	WP_019527678.1	-	DUF2813 domain-containing protein	0	0	0	0	1	0	0	1	1	0	0	3	Accessory								

cluster	GI	gene	Annotation	Streptomyces_thermocarboxydus_BPSAC147	Streptomyces_sp._4F	Streptomyces_sp._BSE7F	Streptomyces_sp._ETH9427	Streptomyces_sp._FxanaD5	Streptomyces_sp._UNC401CLCol	Actinospica_acidiphila_NRRL_B_24431	Streptomyces_sp._HNS054	Streptomyces_sp._SMS_SU21	Streptomyces_sp._ZS0098	Streptomyces_thermocarboxydus_K155	Addition	Core-Accessory-Unique	COG	%Similarity_COG	Score_COG	Evalue_COG	KEGG	%Similarity_KEGG	Score_KEGG	Evalue_KEGG
895	WP_028960818.1	-	redoxin domain-containing protein	1	1	1	1	1	1	1	1	1	1	1	11	CORE	COG0526_OC#	50.7	162	4.55E-39	NA	35.4	290	5.05E-77
896	OSC72169.1	-	ABC transporter	1	1	1	1	1	1	1	1	1	1	1	11	CORE	COG1132_V#	37.6	347	9.98E-95	K06147	36.5	347	2.04E-94
897	Prokka:CIODPCDJ_03160	-	hypothetical protein	0	0	0	0	0	0	0	0	0	0	1	1	Unique	COG1357_S#	29.3	61	1.43E-08				
898	WP_102636970.1	-	FUSC family protein	1	0	0	0	1	1	0	1	1	1	1	7	Accessory					NA	34.2	78	2.31E-13
899	Prokka:JAPCLKMA_04417	-	hypothetical protein	1	0	0	0	0	0	0	0	0	0	0	1	Unique					NA	35.8	193	4.92E-48
900	WP_048460295.1	lepA	elongation factor 4	1	0	1	1	1	1	1	1	1	1	1	10	Accessory	COG0481_M#	74.4	919	8.29E-267	K03596	74.4	919	2.21E-266
901	OSC71016.1	-	ABC transporter substrate-binding protein	0	1	0	0	0	0	1	0	0	0	0	2	Accessory	COG0747_E#	29	205	6.11E-52	NA	24.9	132	1.76E-29
902	Prokka:CIODPCDJ_01008	-	hypothetical protein	1	1	1	1	1	1	1	1	1	1	1	11	CORE	COG0464_O#	43	209	3.24E-53	NA	41.7	223	5.78E-57
903	OSC72987.1	-	short chain dehydrogenase	1	1	1	1	1	1	1	1	1	1	1	11	CORE	COG1028_IQR#	53.5	268	7.67E-71	NA	43.5	431	1.83E-119
904	Prokka:JAPCLKMA_01991	smc_4	Chromosome partition protein Smc	1	0	0	0	0	0	0	0	0	0	0	1	Unique					NA	22.8	97	4.78E-19
905	WP_028960802.1	-	hypothetical protein	1	1	1	1	1	1	1	1	1	1	1	11	CORE								

cluster	GI	gene	Annotation	Streptomyces_thermocarboxydus_BPSAC147	Streptomyces_sp._4F	Streptomyces_sp._BSE7F	Streptomyces_sp._ETH9427	Streptomyces_sp._FxanaD5	Streptomyces_sp._UNC401CLCol	Actinospica_acidiphila_NRRL_B_24431	Streptomyces_sp._HNS054	Streptomyces_sp._SMS_SU21	Streptomyces_sp._ZS0098	Streptomyces_thermocarboxydus_K155	Addition	Core-Accessory-Unique	COG	%Similarity_COG	Score_COG	Evalue_COG	KEGG	%Similarity_KEGG	Score_KEGG	Evalue_KEGG
906	WP_033273626.1	-	DUF1998 domain-containing protein	0	0	1	0	1	1	1	0	0	0	0	4	Accessory								
907	WP_028960500.1	-	FUSC family protein	1	1	0	0	1	1	0	0	0	0	1	5	Accessory								
908	Prokka:JAPCLKMA_04477	ilvB1	Acetolactate synthase large subunit IlvB1	1	1	1	1	1	1	1	1	1	1	1	11	CORE	COG0028_EH#	66.1	829	1.12E-239	K01652	66.1	829	2.99E-239
909	WP_048460702.1	-	AfsR/SARP family transcriptional regulator	0	0	1	1	0	0	0	1	1	1	0	5	Accessory					NA	27.4	78	2.99E-13
910	PWE11030.1	-	urease subunit alpha	1	1	1	1	1	1	1	1	1	1	1	11	CORE	COG0804_E#	60.3	725	2.28E-208	K01428	60.3	725	6.09E-208
911	Prokka:JAPCLKMA_02010	asnO_1	Asparagine synthetase [glutamine-hydrolyzing] 3	1	0	0	0	0	0	0	0	0	0	0	1	Unique	COG0367_E#	47.9	540	1.04E-152	K01953	40.6	471	1.21E-131
912	WP_019526662.1	ilvD	dihydroxy-acid dehydratase	1	1	1	1	1	1	1	1	1	1	1	11	CORE	COG0129_EG#	67.8	835	1.56E-241	K01687	67.8	835	4.17E-241
913	WP_122212772.1	-	VgrG-related protein	0	0	0	1	0	0	0	1	1	1	0	4	Accessory	COG3501_S#	31.1	84	1.19E-15				

cluster	GI	gene	Annotation	Streptomyces_thermocarboxydus_BPSAC147	Streptomyces_sp._4F	Streptomyces_sp._BSE7F	Streptomyces_sp._ETH9427	Streptomyces_sp._FxanaD5	Streptomyces_sp._UNC401CLCol	Actinospica_acidiphila_NRRL_B_24431	Streptomyces_sp._HNS054	Streptomyces_sp._SMS_SU21	Streptomyces_sp._ZS0098	Streptomyces_thermocarboxydus_K155	Addition	Core-Accessory-Unique	COG	%Similarity_COG	Score_COG	Evalue_COG	KEGG	%Similarity_KEGG	Score_KEGG	Evalue_KEGG
914	WP_102637592.1	-	DUF1998 domain-containing protein	0	0	1	0	0	0	0	0	1	0	0	2	Accessory								
915	WP_122214461.1	-	S9 family peptidase	1	1	1	1	1	1	1	1	1	1	1	11	CORE	COG1506_E#	28.2	149	5.14E-35	NA	28.2	149	1.37E-34
916	WP_028960664.1	-	ATP-grasp domain-containing protein	1	1	1	1	1	1	1	1	1	1	1	11	CORE	COG4770_I#	47.1	495	2.92E-139	K01965	54.4	635	6.32E-181
917	PWE10311.1	-	hypothetical protein	0	0	1	0	0	0	0	0	0	0	0	1	Unique	COG0699_R#	27.4	45	0.000801				
918	WP_048458027.1	glmS	glutamine--fructose-6-phosphate transaminase (isomerizing)	1	1	1	1	1	1	1	1	1	1	1	11	CORE	COG0449_M#	64.2	812	8.28E-235	K00820	64.8	794	8.14E-229
919	WP_122217122.1	-	HSP90 family protein	1	0	1	1	1	0	1	1	1	1	0	8	Accessory	COG0326_O#	36.3	333	1.47E-90	K04079	25.2	148	3.06E-34
920	WP_028961447.1	-	transketolase	1	1	0	1	1	1	1	1	1	1	1	10	Accessory	COG0021_G#	62.5	735	1.68E-211	K00615	49.2	565	6.15E-160
921	WP_033272670.1	-	hypothetical protein	1	0	0	1	1	1	1	0	0	0	1	6	Accessory								
922	WP_033277564.1	-	hypothetical protein	1	1	1	1	1	1	1	1	1	1	1	11	CORE								
923	AXI89604.1	-	hypothetical protein	0	0	0	1	0	0	0	0	0	0	0	1	Unique								
924	AXI89535.1	-	thiamine pyrophosphate-requiring protein	0	1	1	1	0	0	1	1	1	1	0	7	Accessory	COG0028_EH#	53.4	625	1.87E-178	K00158	34.4	360	3E-98

cluster	GI	gene	Annotation	Streptomyces_thermocarboxydus_BPSAC147	Streptomyces_sp._4F	Streptomyces_sp._BSE7F	Streptomyces_sp._ETH9427	Streptomyces_sp._FxanaD5	Streptomyces_sp._UNC401CLCol	Actinospica_acidiphila_NRRL_B_24431	Streptomyces_sp._HNS054	Streptomyces_sp._SMS_SU21	Streptomyces_sp._ZS0098	Streptomyces_thermocarboxydus_K155	Addition	Core-Accessory-Unique	COG	%Similarity_COG	Score_COG	Evalue_COG	KEGG	%Similarity_KEGG	Score_KEGG	Evalue_KEGG
925	AXI86823.1	-	beta-N-acetylhexosaminidase	1	1	1	1	1	1	1	1	1	1	1	11	CORE	COG1472_G#	35.5	351	6.81E-96	K01207	33.9	336	4.64E-91
926	WP_019523168.1	-	hypothetical protein	1	1	1	1	1	1	1	1	1	1	1	11	CORE								
927	AXI85410.1	-	glycosyl hydrolase family 15	1	0	1	1	1	1	1	1	1	1	1	10	Accessory	COG3387_G#	41.5	475	3.12E-133	NA	38.3	415	1.02E-114
928	Prokka:CIODPCDJ_03457	-	hypothetical protein	0	0	0	0	0	0	1	0	0	0	1	2	Accessory					NA	38.3	420	3.18E-116
929	Prokka:CIODPCDJ_04244	bmrA_1	Multidrug resistance ABC transporter ATP-binding/permease protein BmrA	0	0	0	0	0	0	0	0	0	0	1	1	Unique	COG1132_V#	30.2	265	6.4E-70	K18104	30.2	265	1.71E-69
930	WP_033273645.1	-	DUF4357 domain-containing protein	1	0	1	0	1	1	1	0	0	0	0	5	Accessory	COG1401_V#	35.3	164	1.53E-39	K07452	35.3	164	4.1E-39
931	WP_028960877.1	-	AMP-binding protein	1	0	0	0	1	1	1	0	0	0	1	5	Accessory	COG1022_I#	39.3	307	1.12E-82	NA	39.3	307	2.99E-82
932	Prokka:CIODPCDJ_03591	-	hypothetical protein	0	0	0	0	0	0	0	0	0	0	1	1	Unique								
933	WP_122216798.1	-	endonuclease/exonuclease/phosphatase	1	1	1	1	1	1	1	1	1	1	1	11	CORE	COG2374_R#	37.8	340	1.19E-92				
934	AXI87304.1	-	Toxic cation resistance protein	1	0	0	1	1	1	0	1	0	0	1	6	Accessory	COG1674_D#	31.6	117	1.26E-25	NA	38.3	186	5.88E-46

cluster	GI	gene	Annotation	Streptomyces_thermocarboxydus_BPSAC147	Streptomyces_sp._4F	Streptomyces_sp._BSE7F	Streptomyces_sp._ETH9427	Streptomyces_sp._FxanaD5	Streptomyces_sp._UNC401CLCol	Actinospica_acidiphila_NRRL_B_24431	Streptomyces_sp._HNS054	Streptomyces_sp._SMS_SU21	Streptomyces_sp._ZS0098	Streptomyces_thermocarboxydus_K155	Addition	Core-Accessory-Unique	COG	%Similarity_COG	Score_COG	Evalue_COG	KEGG	%Similarity_KEGG	Score_KEGG	Evalue_KEGG
935	AXI91211.1	-	hypothetical protein	0	0	0	1	0	0	0	0	0	0	0	1	Unique								
936	WP_019525262.1	-	hypothetical protein	1	1	0	0	1	1	1	0	1	0	0	6	Accessory	COG1674_D#	32.7	122	3.91E-27	NA	35.1	173	6.73E-42
937	OSC73525.1	-	hypothetical protein	0	1	0	0	0	0	0	0	0	0	0	1	Unique								
938	WP_019525740.1	-	beta-galactosidase	1	1	0	0	1	1	1	0	0	0	1	6	Accessory	COG3250_G#	24.8	121	8.69E-27	NA	38.6	437	1.91E-121
939	Prokka:CIODPCDJ_04607	chtA	Chitinase 63	1	1	1	1	1	1	1	1	1	1	1	11	CORE	COG3325_G#	29.2	194	1.38E-48	K01183	31.7	163	9.08E-39
940	Prokka:CIODPCDJ_03377	-	Butyryl-CoA dehydrogenase	1	1	1	1	1	1	1	1	1	1	1	11	CORE	COG1960_I#	62.3	759	8.22E-219	K00257	61.3	748	3.87E-215
941	PWE07042.1	-	branched-chain amino acid ABC transporter permease	1	1	1	1	1	1	1	1	1	1	1	11	CORE	COG4177_E#	41.8	249	3.61E-65	K01998	37.5	241	2.01E-62
942	AXI87585.1	-	bifunctional metallophosphatase/5'-nucleotidase	1	1	1	1	1	1	1	1	1	1	1	11	CORE	COG0737_F#	38.2	359	1.89E-98	K01081	38.2	359	5.06E-98
943	Prokka:CIODPCDJ_05246	-	hypothetical protein	1	0	1	1	1	1	1	1	1	1	1	10	Accessory					NA	29.8	43	0.00802
944	WP_084207081.1	-	fatty acyl-AMP ligase	0	0	0	0	0	0	1	0	0	0	0	1	Unique	COG0318_IQ#	42.4	393	1.18E-108	K12423	37.4	340	3.17E-92

cluster	GI	gene	Annotation	Streptomyces_thermocarboxydus_BPSAC147	Streptomyces_sp._4F	Streptomyces_sp._BSE7F	Streptomyces_sp._ETH9427	Streptomyces_sp._FxanaD5	Streptomyces_sp._UNC401CLCol	Actinospica_acidiphila_NRRL_B_24431	Streptomyces_sp._HNS054	Streptomyces_sp._SMS_SU21	Streptomyces_sp._ZS0098	Streptomyces_thermocarboxydus_K155	Addition	Core-Accessory-Unique	COG	%Similarity_COG	Score_COG	Evalue_COG	KEGG	%Similarity_KEGG	Score_KEGG	Evalue_KEGG
945	Prokka:CIODPCDJ_04265	pckG	Phosphoenolpyruvate carboxykinase [GTP]	1	1	1	1	1	1	1	1	1	1	1	11	CORE	COG1274_C#	68.5	871	2.52E-252	K01596	68.5	871	6.74E-252
946	WP_122215462.1	-	hypothetical protein	0	1	0	1	0	0	1	1	1	1	0	6	Accessory	COG3864_S#	28.3	92	4.31E-18	NA	28.3	92	1.15E-17
947	Prokka:CIODPCDJ_02444	-	hypothetical protein	0	0	0	0	0	1	0	0	0	0	1	2	Accessory	COG0270_L#	26.8	100	2.07E-20	K00558	26.7	81	2.65E-14
948	WP_122214177.1	ggt	gamma-glutamyltransferase	1	1	1	1	1	1	1	1	1	1	1	11	CORE	COG0405_E#	37.4	352	3.93E-96	K00681	37.3	352	1.05E-95
949	WP_048457759.1	glmS	glutamine--fructose-6-phosphate transaminase (isomerizing)	1	1	1	1	1	1	1	1	1	1	1	11	CORE	COG0449_M#	46.9	546	1.42E-154	K00820	46.9	546	3.8E-154
950	AXI88324.1	-	cholesterol oxidase	1	1	1	1	1	1	1	1	1	1	1	11	CORE	COG2303_E#	60.9	672	1.32E-192	K03333	60.3	670	1.74E-191
951	Prokka:JAPCLKMA_05825	ycf3	Photosystem I assembly protein Ycf3	1	1	1	1	1	1	1	1	1	1	1	11	CORE	COG0790_R#	30.9	71	1.03E-11				
952	WP_028960672.1	-	hypothetical protein	1	0	0	1	1	1	1	1	1	1	1	9	Accessory					K14007	30.2	90	5.7E-17
953	WP_048458869.1	-	DUF262 domain-containing protein	0	0	0	0	0	0	0	1	0	0	0	1	Unique	COG3472_S#	32.6	197	1.62E-49	NA	23.8	148	3.01E-34
954	WP_102641458.1	-	chitinase	0	1	1	1	0	1	1	1	1	1	0	8	Accessory	COG3469_G#	39.8	221	1.05E-56				

cluster	GI	gene	Annotation	Streptomyces_thermocarboxydus_BPSAC147	Streptomyces_sp._4F	Streptomyces_sp._BSE7F	Streptomyces_sp._ETH9427	Streptomyces_sp._FxanaD5	Streptomyces_sp._UNC401CLCol	Actinospica_acidiphila_NRRL_B_24431	Streptomyces_sp._HNS054	Streptomyces_sp._SMS_SU21	Streptomyces_sp._ZS0098	Streptomyces_thermocarboxydus_K155	Addition	Core-Accessory-Unique	COG	%Similarity_COG	Score_COG	Evalue_COG	KEGG	%Similarity_KEGG	Score_KEGG	Evalue_KEGG
955	WP_028958939.1	-	sugar phosphate isomerase/epimerase and 4-hydroxyphenylpyruvate domain-containing protein	1	1	0	0	1	1	0	0	0	0	1	5	Accessory	COG1082_G#	55.8	302	3.57E-81				
956	PWE10283.1	-	coagulation factor 5/8 type domain-containing protein	1	0	1	1	1	1	1	1	1	1	1	10	Accessory								
957	WP_048459588.1	-	cytochrome c biogenesis protein	1	1	0	1	1	1	1	1	1	1	1	10	Accessory	COG1333_O#	38.3	314	1.19E-84	K07399	38.3	314	3.17E-84
958	OSC68522.1	-	DNA helicase	0	1	0	0	0	0	0	1	0	0	0	2	Accessory								
959	WP_048460259.1	thiC	phosphomethylpyrimidine synthase ThiC	1	1	1	1	1	1	1	1	1	1	1	11	CORE	COG0422_H#	69.8	845	1.13E-244	K03147	69.8	846	1.36E-244
960	Prokka:CIODPCDJ_05240	cpsY_3	Exopolysaccharide phosphotransferase CpsY	0	0	0	0	1	1	0	0	0	0	1	3	Accessory					K01784	34.5	285	1.57E-75
961	OSC63116.1	-	ABC transporter	0	1	0	0	0	0	0	0	0	0	0	1	Unique	COG0444_EP#	46	245	5.18E-64	K15583	43.4	239	9.91E-62
962	WP_122215191.1	-	protein kinase	0	1	0	0	0	0	0	0	0	1	0	2	Accessory	COG0515_RTKL#	30.7	141	1.38E-32	K08884	30.7	141	3.67E-32

cluster	GI	gene	Annotation	Streptomyces_thermocarboxydus_BPSAC147	Streptomyces_sp._4F	Streptomyces_sp._BSE7F	Streptomyces_sp._ETH9427	Streptomyces_sp._FxanaD5	Streptomyces_sp._UNC401CLCol	Actinospica_acidiphila_NRRL_B_24431	Streptomyces_sp._HNS054	Streptomyces_sp._SMS_SU21	Streptomyces_sp._ZS0098	Streptomyces_thermocarboxydus_K155	Addition	Core-Accessory-Unique	COG	%Similarity_COG	Score_COG	Evalue_COG	KEGG	%Similarity_KEGG	Score_KEGG	Evalue_KEGG
963	OSC64214.1	-	bifunctional metallophosphatase/5'-nucleotidase	1	1	1	1	1	1	1	1	1	1	1	11	CORE	COG0737_F#	36.2	332	4.21E-90	K01119	36.2	332	1.12E-89
964	PWE08646.1	-	lasso peptide isopeptide bond-forming cyclase	0	0	1	1	0	0	0	1	1	1	0	5	Accessory	COG0367_E#	23.4	58	8.96E-08				
965	AXI87882.1	-	glycerophosphodiester phosphodiesterase	1	1	1	1	1	1	1	1	1	1	1	11	CORE	COG0488_R#	54.3	619	1.32E-176	K15738	35.6	386	6.54E-106
966	WP_026243714.1	-	hypothetical protein	0	0	0	0	1	0	0	0	0	0	0	1	Unique								
967	WP_019524924.1	-	AAA family ATPase	0	0	0	0	1	0	0	0	0	0	0	1	Unique	COG0210_L#	25.5	113	3.06E-24				
968	Prokka:CIODPCDJ_03161	-	hypothetical protein	1	1	0	1	1	1	1	1	1	1	1	10	Accessory	COG2372_R#	37.1	90	2.77E-17				
969	WP_109288541.1	-	hypothetical protein	0	0	1	1	0	0	1	1	1	1	0	6	Accessory	COG3170_NU#	26.6	76	4.15E-13	NA	29.7	152	1.59E-35
970	WP_106959517.1	-	FAD-dependent oxidoreductase	1	0	0	0	1	1	0	0	1	1	1	6	Accessory	COG0492_O#	46.3	280	1.45E-74	K00384	33.2	155	1.44E-36
971	OSC72170.1	-	ABC transporter	1	1	1	1	1	1	1	1	1	1	1	11	CORE	COG2274_V#	26.8	152	5.95E-36	K06147	28	146	1.14E-33

cluster	GI	gene	Annotation	Streptomyces_thermocarboxydus_BPSAC147	Streptomyces_sp._4F	Streptomyces_sp._BSE7F	Streptomyces_sp._ETH9427	Streptomyces_sp._FxanaD5	Streptomyces_sp._UNC401CLCol	Actinospica_acidiphila_NRRL_B_24431	Streptomyces_sp._HNS054	Streptomyces_sp._SMS_SU21	Streptomyces_sp._ZS0098	Streptomyces_thermocarboxydus_K155	Addition	Core-Accessory-Unique	COG	%Similarity_COG	Score_COG	Evalue_COG	KEGG	%Similarity_KEGG	Score_KEGG	Evalue_KEGG
972	WP_019521791.1	-	3-hydroxyacyl-CoA dehydrogenase family protein	1	1	1	1	1	1	1	1	1	1	1	11	CORE	COG1250_I#	47.2	246	2.31E-64	K00074	47.2	246	6.18E-64
973	WP_122215339.1	-	protein kinase	1	1	1	0	1	1	1	1	1	1	1	10	Accessory	COG0515_RTKL#	41.3	185	4.84E-46	K08884	41.3	185	1.29E-45
974	OSC60008.1	-	hypothetical protein	0	1	0	0	0	0	0	0	0	0	0	1	Unique	COG2898_S#	40	378	3.9E-104	K04567	40	378	1.04E-103
975	WP_122213499.1	-	DUF3492 domain-containing protein	1	0	1	1	1	1	1	1	1	1	1	10	Accessory	COG0438_M#	35.9	210	2.4E-53	NA	35.9	210	6.41E-53
976	Prokka:CIODPCDJ_00529	-	hypothetical protein	0	0	0	0	1	1	0	0	0	0	1	3	Accessory	COG1178_P#	30.1	252	5.5E-66	K02011	24.4	151	3.53E-35
977	WP_048456393.1	-	ABC transporter ATP-binding protein	1	1	1	1	1	1	1	1	1	1	1	11	CORE	COG1132_V#	34.1	374	5.62E-103	K11085	34.1	374	1.5E-102
978	WP_102638384.1	-	ABC transporter ATP-binding protein	1	1	1	1	1	1	1	1	1	1	1	11	CORE	COG1132_V#	38.4	326	1.76E-88	K11085	38.4	326	4.69E-88
979	OSC70187.1	-	monooxygenase	0	1	0	0	0	0	0	0	0	0	0	1	Unique	COG2072_P#	32.9	246	3.02E-64	NA	40.9	163	8.96E-39
980	Prokka:CIODPCDJ_03001	-	hypothetical protein	0	0	0	0	0	0	0	0	0	0	1	1	Unique								
981	WP_122215652.1	-	ABC transporter substrate-binding protein	1	1	1	1	1	1	1	1	1	1	1	11	CORE	COG0747_E#	24.9	116	4.71E-25	K02035	24.9	116	1.26E-24

cluster	GI	gene	Annotation	Streptomyces_thermocarboxydus_BPSAC147	Streptomyces_sp._4F	Streptomyces_sp._BSE7F	Streptomyces_sp._ETH9427	Streptomyces_sp._FxanaD5	Streptomyces_sp._UNC401CLCol	Actinospica_acidiphila_NRRL_B_24431	Streptomyces_sp._HNS054	Streptomyces_sp._SMS_SU21	Streptomyces_sp._ZS0098	Streptomyces_thermocarboxydus_K155	Addition	Core-Accessory-Unique	COG	%Similarity_COG	Score_COG	Evalue_COG	KEGG	%Similarity_KEGG	Score_KEGG	Evalue_KEGG
982	PWE11006.1	uppS	di-trans,poly-cis-decaprenylcistransferase	0	0	1	0	0	0	0	0	0	0	0	1	Unique	COG0020_I#	46.2	243	1.95E-63	K14215	46.2	243	5.22E-63
983	OSC65712.1	-	peptide ABC transporter substrate-binding protein	0	1	0	0	0	0	0	0	0	0	0	1	Unique	COG0747_E#	26.4	139	5.18E-32	K02035	25.9	124	4.61E-27
984	WP_084207105.1	-	MFS transporter	0	0	0	0	0	0	1	0	0	0	0	1	Unique	COG0477_GEPR#	35.7	273	2.3E-72	NA	31.7	229	7.79E-59
985	WP_122213424.1	-	ABC transporter ATP-binding protein	0	1	0	0	0	0	1	1	1	1	0	5	Accessory	COG1132_V#	35.3	276	2.72E-73	K11085	33.6	242	1.16E-62
986	WP_122214599.1	-	PQQ-binding-like beta-propeller repeat protein	1	1	1	1	1	1	1	1	1	1	1	11	CORE					NA	40.4	97	4.61E-19
987	WP_048456893.1	-	transglycosylase SLT domain-containing protein	1	1	1	1	1	1	1	1	1	1	1	11	CORE	COG2951_M#	33.6	178	1.01E-43	NA	33.1	176	7.81E-43
988	PWE11166.1	-	hypothetical protein	1	1	1	1	1	1	1	1	1	1	1	11	CORE	COG1061_KL#	56.2	628	2.15E-179	NA	55.7	624	1.08E-177
989	WP_078611500.1	secD	protein translocase subunit SecD	1	1	1	1	1	1	1	1	1	1	1	11	CORE	COG0342_U#	38	353	1.75E-96	K03072	38	353	4.66E-96

cluster	GI	gene	Annotation	Streptomyces_thermocarboxydus_BPSAC147	Streptomyces_sp._4F	Streptomyces_sp._BSE7F	Streptomyces_sp._ETH9427	Streptomyces_sp._FxanaD5	Streptomyces_sp._UNC401CLCol	Actinospica_acidiphila_NRRL_B_24431	Streptomyces_sp._HNS054	Streptomyces_sp._SMS_SU21	Streptomyces_sp._ZS0098	Streptomyces_thermocarboxydus_K155	Addition	Core-Accessory-Unique	COG	%Similarity_COG	Score_COG	Evalue_COG	KEGG	%Similarity_KEGG	Score_KEGG	Evalue_KEGG
990	WP_048456745.1	sucB	2-oxoglutarate dehydrogenase, E2 component, dihydrolipoamide succinyltransferase	1	1	1	1	1	1	1	1	1	1	1	11	CORE	COG0508_C#	41.3	412	2.42E-114	K00658	43.5	367	1.83E-100
991	WP_051724460.1	-	glycoside hydrolase	0	0	0	1	0	0	1	1	0	0	0	3	Accessory	COG3387_G#	29	200	1.89E-50	NA	29	200	6.6E-50
992	Prokka:JAPCLKMA_04769	-	Periplasmic [NiFeSe] hydrogenase large subunit	1	0	0	1	1	1	0	1	0	1	1	7	Accessory	COG0374_C#	26.5	121	1.46E-26	K06281	26.5	121	3.89E-26
993	WP_033277129.1	-	AfsR/SARP family transcriptional regulator	0	1	0	0	0	0	1	0	0	0	0	2	Accessory	COG3629_T#	35.3	141	7.97E-33	NA	35.3	141	2.13E-32
994	WP_107685385.1	-	DUF2510 domain-containing protein	1	0	1	0	1	1	1	1	1	1	1	9	Accessory					NA	32.2	116	1.25E-24
995	WP_048458605.1	-	ABC transporter ATP-binding protein	0	1	0	1	0	0	0	1	0	0	0	3	Accessory	COG1132_V#	28.1	176	2.23E-43	K18894	29.4	188	2.58E-46
996	WP_122212975.1	-	FAD-dependent monooxygenase	1	1	1	1	1	1	1	1	1	1	1	11	CORE	COG0654_HC#	33.3	148	1.11E-34	NA	32.8	173	8.61E-42
997	WP_102637285.1	acsA	acetate--CoA ligase	0	0	0	0	0	0	0	0	1	1	0	2	Accessory	COG0365_I#	47.9	535	1.89E-151	K01895	45.1	500	3.07E-140

cluster	GI	gene	Annotation	Streptomyces_thermocarboxydus_BPSAC147	Streptomyces_sp._4F	Streptomyces_sp._BSE7F	Streptomyces_sp._ETH9427	Streptomyces_sp._FxanaD5	Streptomyces_sp._UNC401CLCol	Actinospica_acidiphila_NRRL_B_24431	Streptomyces_sp._HNS054	Streptomyces_sp._SMS_SU21	Streptomyces_sp._ZS0098	Streptomyces_thermocarboxydus_K155	Addition	Core-Accessory-Unique	COG	%Similarity_COG	Score_COG	Evalue_COG	KEGG	%Similarity_KEGG	Score_KEGG	Evalue_KEGG
998	AXI85352.1	-	hypothetical protein	1	0	0	1	0	0	0	0	0	0	0	2	Accessory								
999	Prokka:JAPCLKMA_01500	-	hypothetical protein	1	0	0	1	0	0	0	0	0	0	0	2	Accessory	COG3505_U#	22	55	0.00000075				
1000	PWE09473.1	-	ABC transporter ATP-binding protein	1	1	1	1	1	1	1	1	1	1	1	11	CORE	COG1132_V#	32.4	310	9.88E-84	K18894	35.5	309	7.68E-83
1001	WP_048458600.1	-	hypothetical protein	0	0	0	0	0	0	0	1	0	0	0	1	Unique								
1002	OSC54260.1	-	hypothetical protein	0	1	0	0	0	0	0	0	0	0	0	1	Unique	COG3321_Q#	41.2	442	2.18E-123	K11628	42.3	434	1.58E-120
1003	OSC58626.1	-	3-oxoacyl-ACP synthase	1	1	1	0	1	0	1	1	1	1	1	9	Accessory	COG0304_IQ#	41.3	308	6.41E-83	K09458	41.4	308	1.71E-82
1004	OSC64906.1	-	serine/threonine protein phosphatase	0	1	0	0	0	0	0	0	0	0	0	1	Unique	COG2208_TK#	34.3	119	5.51E-26	NA	31.1	135	1.52E-30
1005	WP_031020915.1	-	N-acetylglutaminylglutamine amidotransferase	1	0	1	1	1	1	1	1	1	1	1	10	Accessory	COG0367_E#	59.7	718	2.68E-206	K01953	29.8	270	3.02E-71
1006	WP_122213330.1	-	DUF1080 domain-containing protein	1	1	1	1	1	1	1	1	1	1	1	11	CORE	COG3828_S#	23.1	61	1.04E-08				
1007	AXI87133.1	-	two-component sensor histidine kinase	0	0	0	1	0	0	0	1	1	0	0	3	Accessory	COG0642_T#	35.5	184	1.39E-45	K07642	33.3	183	6.35E-45

cluster	GI	gene	Annotation	Streptomyces_thermocarboxydus_BPSAC147	Streptomyces_sp._4F	Streptomyces_sp._BSE7F	Streptomyces_sp._ETH9427	Streptomyces_sp._FxanaD5	Streptomyces_sp._UNC401CLCol	Actinospica_acidiphila_NRRL_B_24431	Streptomyces_sp._HNS054	Streptomyces_sp._SMS_SU21	Streptomyces_sp._ZS0098	Streptomyces_thermocarboxydus_K155	Addition	Core-Accessory-Unique	COG	%Similarity_COG	Score_COG	Evalue_COG	KEGG	%Similarity_KEGG	Score_KEGG	Evalue_KEGG
1008	WP_028961522.1	gcl	glyoxylate carboligase	1	1	1	1	1	1	1	1	1	1	1	11	CORE	COG3960_R#	73.6	890	3.94E-258	K01608	73.4	878	3.17E-254
1009	WP_019524962.1	-	CDP-alcohol phosphatidyltransferase family protein	1	1	1	1	1	1	1	1	1	1	1	11	CORE	COG0558_I#	28.7	52	0.00000825				
1010	WP_122212869.1	-	ABC transporter ATP-binding protein	1	1	1	1	1	1	1	1	1	1	1	11	CORE	COG1132_V#	33.5	285	3.39E-76	K06147	27.1	250	4.22E-65
1011	Prokka:JAPCLKMA_03237	-	hypothetical protein	1	0	0	0	1	1	0	0	1	1	1	6	Accessory	COG3835_KT#	29.9	88	1.04E-16	K02647	29.9	88	2.77E-16
1012	WP_048459562.1	-	alanine-rich protein	1	1	1	1	1	1	1	1	1	1	1	11	CORE	COG0086_K#	37.5	56	4.37E-07	NA	26.3	79	1.29E-13
1013	Prokka:JAPCLKMA_05864	-	putative Na(+)/H(+) exchanger	1	1	1	1	1	1	1	1	1	1	1	11	CORE	COG0025_P#	36.6	292	4.74E-78	K03316	35.3	278	1.89E-73
1014	Prokka:JAPCLKMA_05146	-	hypothetical protein	1	0	0	0	0	0	0	0	0	0	0	1	Unique								
1015	WP_078611434.1	-	NTP transferase domain-containing protein	1	1	1	1	0	1	1	1	1	1	1	10	Accessory	COG1778_R#	39.1	93	3.23E-18	NA	35	93	6.6E-18
1016	WP_122215871.1	-	hydrogenase maturation protein	1	0	0	1	1	1	0	1	0	1	1	7	Accessory	COG0223_J#	48.4	262	4.01E-69	NA	48.4	262	1.07E-68
1017	WP_051724630.1	-	fatty acyl-AMP ligase	0	0	0	0	0	0	1	0	0	0	0	1	Unique	COG0318_IQ#	38.6	346	1.62E-94	NA	36.2	333	3.78E-90

cluster	GI	gene	Annotation	Streptomyces_thermocarboxydus_BPSAC147	Streptomyces_sp._4F	Streptomyces_sp._BSE7F	Streptomyces_sp._ETH9427	Streptomyces_sp._FxanaD5	Streptomyces_sp._UNC401CLCol	Actinospica_acidiphila_NRRL_B_24431	Streptomyces_sp._HNS054	Streptomyces_sp._SMS_SU21	Streptomyces_sp._ZS0098	Streptomyces_thermocarboxydus_K155	Addition	Core-Accessory-Unique	COG	%Similarity_COG	Score_COG	Evalue_COG	KEGG	%Similarity_KEGG	Score_KEGG	Evalue_KEGG
1018	AXI91264.1	-	ATP/GTP-binding protein	0	0	0	1	0	0	0	0	0	0	0	1	Unique								
1019	OSC64736.1	-	2-aminobenzoate-CoA ligase	1	1	1	1	1	1	1	1	1	1	1	11	CORE	COG0365_I#	60.1	426	2.1E-118	K01897	34.4	201	2.24E-50
1020	OSC71378.1	-	carbamoyltransferase	0	1	0	0	0	0	0	0	0	0	0	1	Unique	COG2192_O#	37.4	333	1.85E-90	K00612	35.2	320	4.33E-86
1021	AXI91259.1	-	hypothetical protein	0	0	0	1	0	0	0	0	0	0	0	1	Unique								
1022	WP_026244124.1	-	serine hydrolase	1	1	1	1	1	1	1	1	1	1	1	11	CORE	COG1680_V#	32.8	176	3.77E-43	NA	32.8	176	1.01E-42
1023	WP_048455719.1	-	stress protein	1	1	0	1	1	1	1	1	1	1	1	10	Accessory	COG2310_T#	44.3	154	1.54E-36	K05792	43.5	170	5.53E-41
1024	WP_158693602.1	-	MFS transporter	1	0	0	0	1	1	0	0	0	0	1	4	Accessory	COG0314_H#	69.2	205	4.45E-52	K03635	69.2	205	1.19E-51
1025	Prokka:CIODPCDJ_04589	-	hypothetical protein	1	0	1	1	1	1	1	1	1	1	1	10	Accessory					NA	32.7	128	1.85E-28
1026	WP_048459692.1	-	MOSC domain-containing protein	1	0	1	1	1	0	1	1	1	1	1	9	Accessory	COG1018_C#	40.6	155	5.28E-37	K05916	38.1	164	2.32E-39
1027	WP_033276052.1	-	ABC transporter ATP-binding protein	1	1	1	1	1	1	1	1	1	1	1	11	CORE	COG1132_V#	36.4	319	2.76E-86	K06147	30.3	252	1.11E-65
1028	WP_122215550.1	-	ATP-grasp domain-containing protein	1	1	1	1	1	1	1	1	1	1	1	11	CORE	COG4770_I#	68.5	792	8.5E-229	K11263	68.5	792	2.27E-228

cluster	GI	gene	Annotation	Streptomyces_thermocarboxydus_BPSAC147	Streptomyces_sp._4F	Streptomyces_sp._BSE7F	Streptomyces_sp._ETH9427	Streptomyces_sp._FxanaD5	Streptomyces_sp._UNC401CLCol	Actinospica_acidiphila_NRRL_B_24431	Streptomyces_sp._HNS054	Streptomyces_sp._SMS_SU21	Streptomyces_sp._ZS0098	Streptomyces_thermocarboxydus_K155	Addition	Core-Accessory-Unique	COG	%Similarity_COG	Score_COG	Evalue_COG	KEGG	%Similarity_KEGG	Score_KEGG	Evalue_KEGG
1029	WP_102641628.1	-	IucA/IucC family siderophore biosynthesis protein	1	1	0	1	1	1	1	1	1	1	1	10	Accessory	COG4264_Q#	48.7	581	2.98E-165				
1030	AXI89625.1	-	mobilization protein	1	0	0	1	0	0	0	0	0	0	0	2	Accessory								
1031	Prokka:JAPCLKMA_02984	-	hypothetical protein	1	0	1	1	1	1	1	1	1	1	1	10	Accessory								
1032	PWE10745.1	-	ABC transporter	1	1	1	1	1	1	1	1	1	1	1	11	CORE	COG1132_V#	34.5	283	1.28E-75	NA	34.3	282	9.95E-75
1033	WP_078504678.1	-	LCP family protein	1	1	1	1	1	1	1	1	1	1	1	11	CORE	COG1316_K#	29.3	151	1.3E-35	NA	27.5	142	1.23E-32
1034	OSC70201.1	-	hypothetical protein	1	1	0	1	1	1	1	1	1	1	1	10	Accessory					K06890	40.6	52	0.0000219
1035	PWE10560.1	-	hypothetical protein	0	0	1	0	0	0	0	0	0	0	0	1	Unique	COG3321_Q#	42.6	198	5.42E-50	K13613	37.8	199	8.48E-50
1036	PWE06716.1	arc	proteasome ATPase	1	1	1	1	1	1	1	1	1	1	1	11	CORE	COG0464_O#	66	778	1.65E-224	K13527	66.3	767	1.02E-220
1037	OSC57412.1	-	MFS transporter	1	1	1	1	1	1	1	1	1	1	1	11	CORE	COG0477_GEPR#	47	429	2.46E-119	NA	26.7	149	1.01E-34
1038	WP_033272869.1	-	NAD+ synthase	1	1	1	1	1	1	1	1	1	1	1	11	CORE	COG0171_H#	52.7	278	5.37E-74	K01950	41.9	434	1.2E-120
1039	WP_028959316.1	ctaD	cytochrome c oxidase subunit I	1	1	1	1	1	1	1	1	1	1	1	11	CORE	COG0843_C#	69.6	820	2.91E-237	K02274	69.6	820	7.76E-237
1040	WP_102640348.1	-	lysine--tRNA ligase	1	1	1	1	1	1	1	1	1	1	1	11	CORE	COG1384_J#	31.1	272	5.03E-72	K04566	29	174	2.23E-42
1041	PWE08410.1	-	aspartate--tRNA ligase	1	1	1	1	1	1	1	1	1	1	1	11	CORE	COG0173_J#	57.4	698	2.84E-200	K01876	57.1	688	4.59E-197

cluster	GI	gene	Annotation	Streptomyces_thermocarboxydus_BPSAC147	Streptomyces_sp._4F	Streptomyces_sp._BSE7F	Streptomyces_sp._ETH9427	Streptomyces_sp._FxanaD5	Streptomyces_sp._UNC401CLCol	Actinospica_acidiphila_NRRL_B_24431	Streptomyces_sp._HNS054	Streptomyces_sp._SMS_SU21	Streptomyces_sp._ZS0098	Streptomyces_thermocarboxydus_K155	Addition	Core-Accessory-Unique	COG	%Similarity_COG	Score_COG	Evalue_COG	KEGG	%Similarity_KEGG	Score_KEGG	Evalue_KEGG
1042	Prokka:CIODPCDJ_06413	rhlE_3	ATP-dependent RNA helicase RhlE	1	1	1	1	1	1	1	1	1	1	1	11	CORE	COG0513_LKJ#	49.1	403	1.44E-111	K11927	37.6	284	2.61E-75
1043	WP_026243769.1	casA	type I-E CRISPR-associated protein Cse1/CasA	0	0	0	0	1	0	0	0	0	0	0	1	Unique								
1044	WP_048456026.1	-	sugar phosphotransferase	0	0	0	1	0	0	1	1	0	1	0	4	Accessory					K01784	35.8	270	3.9E-71
1045	OSC68075.1	-	ABC transporter ATP-binding protein	0	1	0	0	0	0	0	0	0	0	0	1	Unique	COG1132_V#	37.2	357	6.96E-98	K14699	37.2	357	1.86E-97
1046	OSC72928.1	-	arginine--tRNA ligase	1	1	1	1	1	1	1	1	1	1	1	11	CORE	COG0018_J#	45.2	490	1.17E-137	K01887	44.8	485	7.7E-136
1047	WP_122215776.1	-	Hsp70 family protein	1	0	0	0	0	0	0	0	0	1	1	3	Accessory	COG0443_O#	31.8	197	1.2E-49	NA	31.8	197	3.21E-49
1048	Prokka:JAPCLKMA_03202	-	hypothetical protein	1	1	1	1	1	1	1	1	1	1	1	11	CORE								
1049	WP_122213284.1	-	GAF domain-containing sensor histidine kinase	1	1	1	1	1	1	1	1	1	1	1	11	CORE	COG2203_T#	43.7	277	1.56E-73	NA	42.1	398	1.62E-109
1050	PWE11071.1	-	recombinase family protein	0	0	1	0	0	0	0	0	0	0	0	1	Unique	COG1961_L#	25	107	2.13E-22	NA	26.4	46	0.00119

cluster	GI	gene	Annotation	Streptomyces_thermocarboxydus_BPSAC147	Streptomyces_sp._4F	Streptomyces_sp._BSE7F	Streptomyces_sp._ETH9427	Streptomyces_sp._FxanaD5	Streptomyces_sp._UNC401CLCol	Actinospica_acidiphila_NRRL_B_24431	Streptomyces_sp._HNS054	Streptomyces_sp._SMS_SU21	Streptomyces_sp._ZS0098	Streptomyces_thermocarboxydus_K155	Addition	Core-Accessory-Unique	COG	%Similarity_COG	Score_COG	Evalue_COG	KEGG	%Similarity_KEGG	Score_KEGG	Evalue_KEGG
1051	WP_086704312.1	-	succinate dehydrogenase flavoprotein subunit	1	1	1	1	1	1	1	1	1	1	1	11	CORE	COG1053_C#	71.5	866	4.59E-251	K00239	71.5	866	1.22E-250
1052	WP_102639505.1	-	hypothetical protein	1	1	1	1	1	1	1	1	1	1	1	11	CORE								
1053	Prokka:JAPCLKMA_05046	mltG_2	Endolytic murein transglycosylase	1	1	0	1	1	1	1	1	1	1	1	10	Accessory	COG1559_R#	31.8	168	7.78E-41	K07082	32.8	129	1.07E-28
1054	WP_028961887.1	-	MFS transporter	1	1	1	1	0	1	1	1	1	1	1	10	Accessory	COG0477_GEPR#	42.7	363	1.65E-99	NA	29.9	211	1.64E-53
1055	WP_146217799.1	-	hypothetical protein	0	0	1	0	0	0	1	0	1	0	0	3	Accessory					NA	33.6	84	3.94E-15
1056	WP_102640364.1	-	hypothetical protein	0	0	0	0	0	0	0	0	1	0	0	1	Unique	COG0737_F#	47.1	58	1.13E-07				
1057	WP_122214059.1	-	histidinol-phosphatase	1	1	1	1	1	1	1	1	1	1	1	11	CORE								
1058	WP_019526905.1	poxB	ubiquinone-dependent pyruvate dehydrogenase	1	0	1	1	1	1	1	1	1	1	1	10	Accessory	COG0028_EH#	56.7	655	1.6E-187	K00156	55.1	648	5.22E-185
1059	WP_033274644.1	-	membrane protein	1	1	1	1	1	1	1	1	1	1	1	11	CORE	COG0728_R#	27.6	149	2.85E-35	K03980	25.3	135	1.49E-30
1060	Prokka:CIODPCDJ_05317	treZ	Malto-oligosyltrehalose trehalohydrolase	1	1	1	1	1	1	1	1	1	1	1	11	CORE	COG0296_G#	53.2	620	5.7E-177	K01236	53.2	620	1.52E-176

cluster	GI	gene	Annotation	Streptomyces_thermocarboxydus_BPSAC147	Streptomyces_sp._4F	Streptomyces_sp._BSE7F	Streptomyces_sp._ETH9427	Streptomyces_sp._FxanaD5	Streptomyces_sp._UNC401CLCol	Actinospica_acidiphila_NRRL_B_24431	Streptomyces_sp._HNS054	Streptomyces_sp._SMS_SU21	Streptomyces_sp._ZS0098	Streptomyces_thermocarboxydus_K155	Addition	Core-Accessory-Unique	COG	%Similarity_COG	Score_COG	Evalue_COG	KEGG	%Similarity_KEGG	Score_KEGG	Evalue_KEGG
1061	Prokka:CIODPCDJ_02733	pmt	putative dolichyl-phosphate-mannose--protein mannosyltransferase	1	1	1	1	1	1	1	1	1	1	1	11	CORE	COG1928_O#	39.1	357	8.99E-98	NA	39.1	357	2.4E-97
1062	Prokka:CIODPCDJ_06096	frc_3	Formyl-CoA:oxalate CoA-transferase	0	0	0	0	1	1	1	0	0	0	1	4	Accessory	COG1804_C#	38.4	120	2.42E-26	NA	34.5	121	3.78E-26
1063	WP_167338198.1	-	serine/threonine-protein phosphatase	1	0	0	0	1	1	1	1	1	1	1	8	Accessory	COG0532_J#	36.3	78	1.05E-13	NA	34.1	92	1.44E-17
1064	OSC56678.1	-	cation acetate symporter	1	1	0	1	1	1	1	1	1	1	1	10	Accessory	COG4147_R#	37.1	139	3.85E-32	K14393	35	122	1.7E-26
1065	PWE07413.1	-	CDP-alcohol phosphatidyltransferase	1	1	1	1	1	1	1	1	1	1	1	11	CORE								
1066	Prokka:CIODPCDJ_05691	-	hypothetical protein	1	0	0	0	0	0	0	0	0	1	1	3	Accessory								
1067	WP_102640197.1	recN	DNA repair protein RecN	1	1	1	0	1	1	1	1	1	1	1	10	Accessory	COG0497_L#	46.2	438	5.23E-122	K03631	46.2	438	1.4E-121
1068	WP_033276648.1	-	relaxase/mobilization nuclease domain-containing protein	0	0	1	1	0	0	1	0	0	0	1	4	Accessory								

cluster	GI	gene	Annotation	Streptomyces_thermocarboxydus_BPSAC147	Streptomyces_sp._4F	Streptomyces_sp._BSE7F	Streptomyces_sp._ETH9427	Streptomyces_sp._FxanaD5	Streptomyces_sp._UNC401CLCol	Actinospica_acidiphila_NRRL_B_24431	Streptomyces_sp._HNS054	Streptomyces_sp._SMS_SU21	Streptomyces_sp._ZS0098	Streptomyces_thermocarboxydus_K155	Addition	Core-Accessory-Unique	COG	%Similarity_COG	Score_COG	Evalue_COG	KEGG	%Similarity_KEGG	Score_KEGG	Evalue_KEGG
1069	WP_028961396.1	-	hypothetical protein	0	0	0	0	0	1	0	0	0	0	0	1	Unique								
1070	WP_109288548.1	-	histidinol-phosphate aminotransferase family protein	1	1	1	1	1	1	1	1	1	0	1	10	Accessory	COG0079_E#	35.2	204	9.73E-52	NA	27.3	117	3.2E-25
1071	PWE11256.1	-	L-aspartate oxidase	1	0	1	1	1	1	1	1	1	1	1	10	Accessory	COG0029_H#	48.6	421	3.87E-117	K00278	48.6	421	1.03E-116
1072	WP_102637131.1	-	ABC transporter ATP-binding protein	0	1	0	1	0	0	1	1	1	1	0	6	Accessory	COG1132_V#	37.8	335	3.65E-91	K11085	33.7	266	5.56E-70
1073	OSC63762.1	-	dihydroxy-acid dehydratase	1	1	1	1	1	1	1	1	1	1	1	11	CORE	COG0129_EG#	61.4	721	2.36E-207	K01687	38.5	359	4.82E-98
1074	AXI84974.1	-	pyruvate dehydrogenase	0	1	0	1	0	0	0	0	0	0	0	2	Accessory	COG0028_EH#	61.4	719	8.98E-207	K00156	48.3	543	3.08E-153
1075	WP_109288705.1	-	hypothetical protein	1	1	0	1	1	1	1	1	1	1	1	10	Accessory	COG1463_Q#	36	74	1.98E-12	NA	27.4	112	1.03E-23
1076	OSC68928.1	-	cellulose 1,4-beta-cellobiosidase	1	1	1	1	1	1	1	1	1	1	1	11	CORE	COG5297_G#	47.1	400	9.21E-111				
1077	Prokka:CIODPCDJ_00395	treS_1	Trehalose synthase/amylase TreS	0	1	1	1	0	0	1	1	1	1	1	8	Accessory	COG0366_G#	70	873	4.85E-253	K05343	70	873	1.3E-252
1078	Prokka:CIODPCDJ_02351	ilvB	Acetolactate synthase isozyme 1 large subunit	1	1	1	1	1	1	1	1	1	1	1	11	CORE	COG0028_EH#	33.8	250	1.54E-65	K01652	31.8	241	2.5E-62

cluster	GI	gene	Annotation	Streptomyces_thermocarboxydus_BPSAC147	Streptomyces_sp._4F	Streptomyces_sp._BSE7F	Streptomyces_sp._ETH9427	Streptomyces_sp._FxanaD5	Streptomyces_sp._UNC401CLCol	Actinospica_acidiphila_NRRL_B_24431	Streptomyces_sp._HNS054	Streptomyces_sp._SMS_SU21	Streptomyces_sp._ZS0098	Streptomyces_thermocarboxydus_K155	Addition	Core-Accessory-Unique	COG	%Similarity_COG	Score_COG	Evalue_COG	KEGG	%Similarity_KEGG	Score_KEGG	Evalue_KEGG
1079	WP_033274031.1	-	CocE/NonD family hydrolase	1	0	1	1	1	1	1	1	1	1	1	10	Accessory	COG2936_R#	34.1	275	5.84E-73	K06978	33.3	190	6.6E-47
1080	WP_106432450.1	-	SpoIIE family protein phosphatase	1	1	1	1	1	1	1	1	1	1	1	11	CORE	COG2208_TK#	34.8	121	8.26E-27	NA	30.1	131	2.78E-29
1081	WP_053077001.1	-	ATP-binding domain-containing protein	0	0	0	0	0	0	0	1	0	0	0	1	Unique	COG0210_L#	27.4	54	0.00000162				
1082	PWE11010.1	-	FAD-linked oxidase	0	0	1	0	0	0	0	0	1	0	0	2	Accessory	COG0277_C#	41.3	186	3.56E-46	NA	26.1	165	1.33E-39
1083	WP_122217182.1	-	ABC transporter ATP-binding protein	1	1	1	1	1	1	1	1	1	1	1	11	CORE	COG1132_V#	56.8	645	2.14E-184	K06147	50.4	580	2.26E-164
1084	OSC56260.1	-	hypothetical protein	1	1	0	1	1	1	1	1	1	1	1	10	Accessory								
1085	WP_102639188.1	-	two-component sensor histidine kinase	1	0	1	1	1	1	1	1	1	1	1	10	Accessory	COG4585_T#	31.6	82	7.24E-15	NA	24.7	79	1.64E-13
1086	WP_122217263.1	-	sugar phosphotransferase	0	0	1	1	1	1	0	1	1	1	1	8	Accessory					K01784	40.7	265	1.23E-69
1087	WP_051724668.1	-	hypothetical protein	0	0	0	0	0	0	1	0	0	0	0	1	Unique	COG1960_I#	25.7	154	1.15E-36				
1088	WP_106959395.1	-	FAD-binding protein	1	0	0	1	1	1	1	1	1	1	1	9	Accessory	COG1053_C#	40.8	377	1.09E-103	K18209	40.8	377	2.9E-103

cluster	GI	gene	Annotation	Streptomyces_thermocarboxydus_BPSAC147	Streptomyces_sp._4F	Streptomyces_sp._BSE7F	Streptomyces_sp._ETH9427	Streptomyces_sp._FxanaD5	Streptomyces_sp._UNC401CLCol	Actinospica_acidiphila_NRRL_B_24431	Streptomyces_sp._HNS054	Streptomyces_sp._SMS_SU21	Streptomyces_sp._ZS0098	Streptomyces_thermocarboxydus_K155	Addition	Core-Accessory-Unique	COG	%Similarity_COG	Score_COG	Evalue_COG	KEGG	%Similarity_KEGG	Score_KEGG	Evalue_KEGG
1089	WP_028958781.1	-	SulP family inorganic anion transporter	1	0	0	0	1	1	1	0	0	0	1	5	Accessory	COG0659_P#	46.9	426	2.04E-118	K03321	35.9	315	7.94E-85
1090	OSC62419.1	-	hypothetical protein	1	1	0	1	1	1	1	1	1	1	1	10	Accessory	COG2730_G#	33.7	46	0.00044	NA	34.9	64	3.19E-09
1091	WP_048457517.1	leuA	2-isopropylmalate synthase	1	1	1	1	1	1	1	1	1	1	1	11	CORE	COG0119_E#	60.6	686	6.42E-197	K01649	60.5	664	6.97E-190
1092	WP_033275299.1	-	SpoIIE family protein phosphatase	1	0	0	0	1	1	1	0	0	0	1	5	Accessory	COG2208_TK#	33	117	2.03E-25	NA	30.6	121	2.87E-26
1093	Prokka:JAPCLKMA_02310	sasA_6	Adaptive-response sensory-kinase SasA	1	0	0	0	0	0	1	0	0	0	1	3	Accessory	COG0642_T#	33.3	167	1.31E-40	NA	33.3	167	3.49E-40
1094	OSC66250.1	-	two-component sensor histidine kinase	0	1	0	0	0	0	0	0	0	0	0	1	Unique	COG4585_T#	28.4	84	1.12E-15	K02480	29.4	71	2.61E-11
1095	WP_033276645.1	-	SpoIIE family protein phosphatase	1	1	0	0	0	0	1	0	0	0	1	4	Accessory	COG2208_TK#	27.4	63	3.47E-09	K07315	27.8	66	1.09E-09
1096	WP_033276189.1	-	LCP family protein	1	0	0	1	1	1	1	1	1	1	1	9	Accessory	COG1316_K#	28.5	131	7.94E-30	NA	28.5	131	2.12E-29
1097	OSC57155.1	-	hypothetical protein	0	1	0	0	0	0	0	0	0	0	0	1	Unique	COG3321_Q#	44.5	383	1.51E-105	NA	44.5	383	4.04E-105
1098	Prokka:JAPCLKMA_04047	-	hypothetical protein	1	0	0	0	1	1	0	0	0	0	1	4	Accessory	COG3409_M#	40.8	54	0.00000161	NA	31.9	124	4.41E-27

cluster	GI	gene	Annotation	Streptomyces_thermocarboxydus_BPSAC147	Streptomyces_sp._4F	Streptomyces_sp._BSE7F	Streptomyces_sp._ETH9427	Streptomyces_sp._FxanaD5	Streptomyces_sp._UNC401CLCol	Actinospica_acidiphila_NRRL_B_24431	Streptomyces_sp._HNS054	Streptomyces_sp._SMS_SU21	Streptomyces_sp._ZS0098	Streptomyces_thermocarboxydus_K155	Addition	Core-Accessory-Unique	COG	%Similarity_COG	Score_COG	Evalue_COG	KEGG	%Similarity_KEGG	Score_KEGG	Evalue_KEGG
1099	WP_051724335.1	-	SPFH domain-containing protein	1	0	1	1	1	1	1	1	1	1	1	10	Accessory								
1100	OSC69968.1	-	glutamyl-tRNA reductase	1	1	1	1	1	1	1	1	1	1	1	11	CORE	COG0373_H#	45.9	237	1.03E-61	K02492	45.9	237	2.74E-61
1101	WP_122213066.1	-	SpoIIE family protein phosphatase	1	1	1	1	1	1	1	1	1	1	1	11	CORE	COG2208_TK#	33.5	121	8.19E-27	NA	31	128	1.79E-28
1102	WP_048460189.1	-	BCCT family transporter	1	1	0	1	1	1	1	1	1	1	1	10	Accessory	COG1292_M#	52.6	534	4.05E-151	K02168	44	429	3.75E-119
1103	PWE09392.1	-	hypothetical protein	0	0	1	0	0	0	0	0	0	0	0	1	Unique					NA	23.4	51	0.0000363
1104	Prokka:JAPCLKMA_06602	-	hypothetical protein	1	1	1	1	1	1	1	1	1	1	1	11	CORE	COG2208_TK#	36	128	6.7E-29	NA	33.1	193	7.73E-48
1105	OSC61203.1	-	glycosylase	0	1	0	0	0	0	0	0	0	0	0	1	Unique	COG3533_S#	33.7	245	3.76E-64	K09955	34.6	263	6.08E-69
1106	AXI89606.1	-	carbamoyltransferase	0	0	0	1	0	0	0	0	0	0	0	1	Unique	COG2192_O#	36	319	2.67E-86	K00612	33.6	305	8.16E-82
1107	Prokka:CIODPCDJ_00826	-	Putative fatty-acid--CoA ligase fadD25	1	0	0	0	1	1	0	0	0	0	1	4	Accessory	COG0318_IQ#	35.3	264	1.02E-69	NA	34.1	236	4.66E-61
1108	WP_019524897.1	-	hypothetical protein	0	0	0	0	1	0	0	0	0	0	0	1	Unique								
1109	WP_102639311.1	-	urocanate hydratase	1	1	1	1	1	1	1	1	1	1	1	11	CORE	COG2987_E#	65.1	726	7.26E-209	K01712	66	725	3.3E-208
1110	WP_051724385.1	-	long-chain fatty acid--CoA ligase	1	1	1	1	1	1	1	1	1	1	1	11	CORE	COG0318_IQ#	54.1	590	8.1E-168	K00666	54.1	590	2.16E-167

cluster	GI	gene	Annotation	Streptomyces_thermocarboxydus_BPSAC147	Streptomyces_sp._4F	Streptomyces_sp._BSE7F	Streptomyces_sp._ETH9427	Streptomyces_sp._FxanaD5	Streptomyces_sp._UNC401CLCol	Actinospica_acidiphila_NRRL_B_24431	Streptomyces_sp._HNS054	Streptomyces_sp._SMS_SU21	Streptomyces_sp._ZS0098	Streptomyces_thermocarboxydus_K155	Addition	Core-Accessory-Unique	COG	%Similarity_COG	Score_COG	Evalue_COG	KEGG	%Similarity_KEGG	Score_KEGG	Evalue_KEGG
1111	Prokka:CIODPCDJ_05343	sir	Sulfite reductase [ferredoxin]	1	1	0	1	1	1	1	1	1	1	1	10	Accessory	COG0155_P#	60.1	694	3.05E-199	K00392	60.1	694	8.14E-199
1112	Prokka:CIODPCDJ_03540	speE_3	Polyamine aminopropyltransferase	1	1	1	1	1	1	1	1	1	1	1	11	CORE	COG4262_R#	43.1	368	5.01E-101	K00797	43.1	368	1.34E-100
1113	PWE08571.1	-	carbamoyltransferase	0	0	1	0	0	0	0	0	0	0	0	1	Unique	COG2192_O#	35.2	265	5.97E-70	K00612	29.6	173	6.31E-42
1114	AXI91247.1	-	hypothetical protein	0	0	0	1	0	0	0	0	0	0	0	1	Unique	COG3468_MU#	35	102	3.92E-21	K01347	35	102	1.05E-20
1115	OSC72814.1	-	serine/threonine protein kinase	1	1	1	1	1	1	1	1	1	1	1	11	CORE	COG0515_RTKL#	27.7	82	4.2E-15	K08884	25.1	71	3.38E-11
1116	WP_019524411.1	-	urease subunit alpha	1	1	0	1	1	1	1	1	0	1	1	9	Accessory	COG0804_E#	48	520	6.03E-147	K01428	45.6	506	4.1E-142
1117	WP_051724438.1	-	N-6 DNA methylase	1	0	1	0	1	1	1	0	0	0	0	5	Accessory	COG0286_V#	27.4	88	9.98E-17				
1118	WP_102640892.1	-	NAD(P)/FAD-dependent oxidoreductase	1	1	1	1	1	1	1	1	1	1	1	11	CORE	COG0665_E#	30.1	47	0.000255	K00231	39.6	55	0.00000191
1119	OSC63839.1	-	serine/threonine protein kinase	1	1	1	1	1	1	1	1	1	1	1	11	CORE	COG0515_RTKL#	34.3	205	5.59E-52	K08884	34.3	205	1.49E-51
1120	WP_033272226.1	-	chitinase	1	1	1	1	1	1	1	1	1	1	1	11	CORE	COG3469_G#	35.1	186	2.06E-46	NA	38.2	59	1.73E-07
1121	OSC68839.1	-	laminin G	0	1	0	0	0	0	0	0	0	0	0	1	Unique	COG4409_G#	28.4	56	3.21E-07	K01186	30	123	7.47E-27

cluster	GI	gene	Annotation	Streptomyces_thermocarboxydus_BPSAC147	Streptomyces_sp._4F	Streptomyces_sp._BSE7F	Streptomyces_sp._ETH9427	Streptomyces_sp._FxanaD5	Streptomyces_sp._UNC401CLCol	Actinospica_acidiphila_NRRL_B_24431	Streptomyces_sp._HNS054	Streptomyces_sp._SMS_SU21	Streptomyces_sp._ZS0098	Streptomyces_thermocarboxydus_K155	Addition	Core-Accessory-Unique	COG	%Similarity_COG	Score_COG	Evalue_COG	KEGG	%Similarity_KEGG	Score_KEGG	Evalue_KEGG
1122	WP_102639526.1	-	sodium:solute symporter family protein	0	0	1	0	0	0	1	0	1	0	0	3	Accessory	COG0591_ER#	24.3	126	3.3E-28	K14392	25.1	122	9.75E-27
1123	AXI89664.1	-	hypothetical protein	0	0	0	1	0	0	0	0	0	0	0	1	Unique								
1124	PWE09006.1	-	glycerol-3-phosphate dehydrogenase/oxidase	1	1	1	1	1	1	1	1	1	1	1	11	CORE	COG0578_C#	60.7	667	5.19E-191	K00111	60.7	667	1.39E-190
1125	Prokka:JAPCLKMA_04442	cysG_3	Siroheme synthase	1	1	0	1	1	1	1	1	1	1	1	10	Accessory	COG1587_H#	68.7	375	4.07E-103	K13542	49.9	493	2.74E-138
1126	WP_019527267.1	-	hypothetical protein	1	1	1	1	1	1	1	1	1	1	1	11	CORE								
1127	OSC70781.1	-	hypothetical protein	1	1	1	1	1	1	1	1	1	1	1	11	CORE	COG2966_S#	32.9	146	4.02E-34	NA	32.2	199	1.07E-49
1128	WP_028959576.1	-	glycoside hydrolase family 13 protein	1	1	1	1	1	1	1	1	1	1	1	11	CORE	COG0366_G#	50.2	539	1.25E-152	K01187	50.3	539	3.33E-152
1129	Prokka:CIODPCDJ_04902	proS_2	Proline--tRNA ligase	1	1	1	1	1	1	1	1	1	1	1	11	CORE	COG0442_J#	57.1	621	4.26E-177	K01881	56.3	619	3.31E-176
1130	OSC72093.1	-	amidohydrolase	0	1	0	0	0	0	0	0	0	0	0	1	Unique	COG1574_R#	34.7	277	1.51E-73	K07047	30.7	208	1.76E-52
1131	WP_102638323.1	-	methylmalonyl-CoA mutase family protein	1	1	1	1	1	1	1	1	1	1	1	11	CORE	COG1884_I#	51.3	570	8.6E-162	K01848	50.9	532	5.31E-150

cluster	GI	gene	Annotation	Streptomyces_thermocarboxydus_BPSAC147	Streptomyces_sp._4F	Streptomyces_sp._BSE7F	Streptomyces_sp._ETH9427	Streptomyces_sp._FxanaD5	Streptomyces_sp._UNC401CLCol	Actinospica_acidiphila_NRRL_B_24431	Streptomyces_sp._HNS054	Streptomyces_sp._SMS_SU21	Streptomyces_sp._ZS0098	Streptomyces_thermocarboxydus_K155	Addition	Core-Accessory-Unique	COG	%Similarity_COG	Score_COG	Evalue_COG	KEGG	%Similarity_KEGG	Score_KEGG	Evalue_KEGG
1132	Prokka:CIODPCDJ_05591	ybiT_2	putative ABC transporter ATP-binding protein YbiT	1	1	1	1	1	1	1	1	1	1	1	11	CORE	COG0488_R#	31.5	215	5.38E-55	NA	31.5	215	1.44E-54
1133	OSC73317.1	-	histidine kinase	1	1	1	1	1	1	1	1	1	1	1	11	CORE	COG3290_T#	30.9	262	3.84E-69	K07701	28.3	231	2.53E-59
1134	PWE09812.1	-	potassium-transporting ATPase subunit KdpA	0	1	1	1	0	0	1	1	1	1	0	7	Accessory	COG2060_P#	49.8	542	1.92E-153	K01546	50.7	534	1.07E-150
1135	WP_028960194.1	-	betaine/proline/choline family ABC transporter ATP-binding protein	1	1	1	1	1	1	0	1	1	1	1	10	Accessory	COG4175_E#	48.4	320	1.55E-86	K02000	53.9	303	4E-81
1136	Prokka:JAPCLKMA_05679	-	hypothetical protein	1	0	0	0	0	0	0	0	0	0	0	1	Unique								
1137	WP_028961542.1	-	PucR family transcriptional regulator	1	1	1	1	1	1	1	1	1	1	1	11	CORE	COG2508_TQ#	29.7	84	1.86E-15	NA	35.6	61	3.45E-08
1138	WP_019523425.1	-	fatty acyl-AMP ligase	0	0	0	0	1	1	1	0	0	0	1	4	Accessory	COG0318_IQ#	40.8	418	5.44E-116	K12428	41	415	7.21E-115
1139	WP_019521433.1	-	hypothetical protein	1	0	0	0	1	1	0	0	0	0	1	4	Accessory	COG2508_TQ#	30.4	147	1.37E-34	NA	30.2	145	1.39E-33

cluster	GI	gene	Annotation	Streptomyces_thermocarboxydus_BPSAC147	Streptomyces_sp._4F	Streptomyces_sp._BSE7F	Streptomyces_sp._ETH9427	Streptomyces_sp._FxanaD5	Streptomyces_sp._UNC401CLCol	Actinospica_acidiphila_NRRL_B_24431	Streptomyces_sp._HNS054	Streptomyces_sp._SMS_SU21	Streptomyces_sp._ZS0098	Streptomyces_thermocarboxydus_K155	Addition	Core-Accessory-Unique	COG	%Similarity_COG	Score_COG	Evalue_COG	KEGG	%Similarity_KEGG	Score_KEGG	Evalue_KEGG
1140	OSC61814.1	-	helix-turn-helix transcriptional regulator	0	1	0	0	0	0	0	0	0	0	0	1	Unique	COG2909_K#	26	57	1.87E-07	K07691	47.1	45	0.0015
1141	WP_028959461.1	-	bifunctional 3'-5' exonuclease/DNA polymerase	1	1	1	1	1	1	1	1	1	1	1	11	CORE	COG0749_L#	24.5	86	4.9E-16	K02349	26.2	80	5.5E-14
1142	WP_158688208.1	-	hypothetical protein	0	0	0	0	1	0	0	0	0	0	0	1	Unique								
1143	WP_158688209.1	-	DUF262 domain-containing protein	0	0	0	0	1	0	0	0	0	0	0	1	Unique	COG1479_S#	30.5	234	8.52E-61				
1144	WP_033276518.1	-	DUF1446 domain-containing protein	1	1	1	1	1	1	1	1	1	1	1	11	CORE					NA	68	747	7.99E-215
1145	WP_033275890.1	-	DAK2 domain-containing protein	1	1	0	1	1	1	1	1	1	1	1	10	Accessory	COG1461_R#	40.6	292	2.64E-78	K07030	40.6	292	7.04E-78
1146	AXI87343.1	paaN	phenylacetic acid degradation protein PaaN	1	1	0	1	1	1	1	1	1	1	1	10	Accessory	COG1012_C#	23.5	94	1.38E-18				
1147	Prokka:JAPCLKMA_04192	pknD_12	Serine/threonine-protein kinase PknD	1	1	1	1	1	1	1	1	1	1	1	11	CORE	COG0515_RTKL#	35.5	183	2.25E-45	K08884	35.5	183	6.01E-45
1148	WP_019522149.1	cobJ	precorrin-3B C(17)-methyltransferase	1	1	1	1	1	1	1	1	1	1	1	11	CORE	COG1010_H#	47.3	207	1.46E-52	K13541	39.2	321	1.42E-86

cluster	GI	gene	Annotation	Streptomyces_thermocarboxydus_BPSAC147	Streptomyces_sp._4F	Streptomyces_sp._BSE7F	Streptomyces_sp._ETH9427	Streptomyces_sp._FxanaD5	Streptomyces_sp._UNC401CLCol	Actinospica_acidiphila_NRRL_B_24431	Streptomyces_sp._HNS054	Streptomyces_sp._SMS_SU21	Streptomyces_sp._ZS0098	Streptomyces_thermocarboxydus_K155	Addition	Core-Accessory-Unique	COG	%Similarity_COG	Score_COG	Evalue_COG	KEGG	%Similarity_KEGG	Score_KEGG	Evalue_KEGG
1149	OSC64973.1	-	multidrug ABC transporter permease	0	1	0	0	0	0	0	0	0	0	0	1	Unique	COG1132_V#	40.4	368	3.78E-101	K06148	31.5	206	6.63E-52
1150	OSC73228.1	-	hypothetical protein	0	1	0	0	0	0	0	0	0	0	0	1	Unique								
1151	AXI86456.1	-	acyl--CoA ligase	0	0	0	1	0	0	0	1	0	0	0	2	Accessory	COG0318_IQ#	29.9	221	5.71E-57	K12507	29.5	219	7.57E-56
1152	OSC73566.1	-	protein-lysine 6-oxidase	0	1	0	0	0	0	0	0	0	0	0	1	Unique								
1153	PWE09629.1	-	Tat pathway signal sequence domain protein	1	1	1	1	1	1	1	1	1	1	1	11	CORE								
1154	Prokka:JAPCLKMA_01899	-	hypothetical protein	1	1	1	1	1	1	1	1	1	1	1	11	CORE	COG4805_S#	35.2	289	3.8E-77				
1155	WP_106959435.1	-	DNA alkylation response protein	1	1	1	1	1	1	1	1	1	1	1	11	CORE	COG1960_I#	62.1	661	2.82E-189	K09456	62.1	661	7.52E-189
1156	Prokka:JAPCLKMA_05511	pknD_18	Serine/threonine-protein kinase PknD	1	0	0	0	0	0	0	0	0	0	0	1	Unique	COG0515_RTKL#	31.1	143	3.37E-33	K08884	31.1	143	8.99E-33
1157	Prokka:CIODPCDJ_01991	stp_2	Multidrug resistance protein Stp	1	1	1	1	1	1	1	1	1	1	1	11	CORE	COG0477_GEPR#	33.2	263	1.71E-69	NA	33.2	263	4.57E-69
1158	OSC64848.1	-	transferase	1	1	1	1	1	1	1	1	1	1	1	11	CORE	COG0463_M#	28	96	2.77E-19	NA	26.3	99	8.73E-20
1159	AXI84917.1	-	choline transporter	0	0	1	1	0	0	1	1	1	1	0	6	Accessory	COG1292_M#	48.8	478	3.38E-134	K05020	44.7	440	2.72E-122

cluster	GI	gene	Annotation	Streptomyces_thermocarboxydus_BPSAC147	Streptomyces_sp._4F	Streptomyces_sp._BSE7F	Streptomyces_sp._ETH9427	Streptomyces_sp._FxanaD5	Streptomyces_sp._UNC401CLCol	Actinospica_acidiphila_NRRL_B_24431	Streptomyces_sp._HNS054	Streptomyces_sp._SMS_SU21	Streptomyces_sp._ZS0098	Streptomyces_thermocarboxydus_K155	Addition	Core-Accessory-Unique	COG	%Similarity_COG	Score_COG	Evalue_COG	KEGG	%Similarity_KEGG	Score_KEGG	Evalue_KEGG
1160	Prokka:CIODPCDJ_04952	rnj	Ribonuclease J	1	1	1	1	1	1	1	1	1	1	1	11	CORE	COG0595_R#	56.2	629	1.55E-179	K12574	55.6	623	1.73E-177
1161	WP_033272537.1	-	hypothetical protein	1	0	1	1	1	1	1	1	1	1	1	10	Accessory	COG2730_G#	41	50	0.0000297	NA	42.2	51	0.0000272
1162	WP_019521408.1	-	acyl--CoA ligase	0	0	0	0	1	1	1	0	0	0	1	4	Accessory	COG0318_IQ#	30.8	197	1.5E-49	K00666	31.8	193	5.79E-48
1163	PWE11370.1	-	hypothetical protein	0	0	1	0	0	0	0	0	0	0	0	1	Unique								
1164	PWE08091.1	-	GGDEF domain-containing protein	1	1	1	1	1	1	1	1	1	1	1	11	CORE	COG2199_T#	45.7	109	5.38E-23	NA	42.5	94	4.78E-18
1165	WP_122213274.1	-	protein disulfide oxidoreductase	0	0	0	1	0	0	0	1	1	1	0	4	Accessory	COG1894_C#	43.6	308	4.63E-83	K05587	40	299	7.49E-80
1166	Prokka:JAPCLKMA_04096	-	hypothetical protein	1	1	1	1	1	1	1	1	1	1	1	11	CORE	COG5479_M#	32.6	178	9.43E-44	NA	32.6	178	2.52E-43
1167	OSC58623.1	-	hypothetical protein	0	1	0	0	0	0	0	0	0	0	0	1	Unique								
1168	WP_102637308.1	-	SulP family inorganic anion transporter	0	0	0	0	0	0	0	0	1	1	0	2	Accessory	COG0659_P#	36.5	339	1.87E-92	NA	37.2	330	3.96E-89
1169	WP_033273676.1	-	acetolactate synthase	1	1	1	1	1	1	1	1	1	1	1	11	CORE	COG0028_EH#	37.6	381	4.29E-105	K01652	37	374	1.83E-102
1170	Prokka:JAPCLKMA_00110	pld	Phospholipase D	1	0	0	1	1	1	1	1	1	1	1	9	Accessory	COG3540_P#	45.2	423	1.29E-117	K01113	34.2	291	1.56E-77

cluster	GI	gene	Annotation	Streptomyces_thermocarboxydus_BPSAC147	Streptomyces_sp._4F	Streptomyces_sp._BSE7F	Streptomyces_sp._ETH9427	Streptomyces_sp._FxanaD5	Streptomyces_sp._UNC401CLCol	Actinospica_acidiphila_NRRL_B_24431	Streptomyces_sp._HNS054	Streptomyces_sp._SMS_SU21	Streptomyces_sp._ZS0098	Streptomyces_thermocarboxydus_K155	Addition	Core-Accessory-Unique	COG	%Similarity_COG	Score_COG	Evalue_COG	KEGG	%Similarity_KEGG	Score_KEGG	Evalue_KEGG
1171	Prokka:CIODPCDJ_04179	mglA_2	Galactose/methyl galactoside import ATP-binding protein MglA	1	1	1	1	1	1	1	1	1	1	1	11	CORE	COG3845_R#	51.3	485	2.76E-136	K02056	46.5	458	1.26E-127
1172	OSC65111.1	-	PucR family transcriptional regulator	1	1	0	1	1	1	1	1	1	1	1	10	Accessory	COG2508_TQ#	31.2	92	5.2E-18	NA	31.2	76	7.89E-13
1173	WP_019526989.1	-	hypothetical protein	0	0	0	0	1	0	0	0	0	0	0	1	Unique								
1174	OSC72993.1	-	histidine kinase	0	1	0	0	0	0	0	0	0	0	0	1	Unique	COG2203_T#	41.9	241	9.05E-63	NA	41.6	371	2.02E-101
1175	Prokka:JAPCLKMA_00782	dcuS_1	Sensor histidine kinase DcuS	1	1	1	1	1	1	1	0	1	1	1	10	Accessory	COG3290_T#	34.4	275	3.32E-73	K11614	30.8	257	4.26E-67
1176	WP_033277124.1	-	acyl-CoA dehydrogenase	0	0	0	0	0	0	1	0	0	0	0	1	Unique	COG1960_I#	33.6	203	2.09E-51	K00248	24.1	69	1.26E-10
1177	PWE07291.1	-	cobalt ABC transporter ATP-binding protein	1	1	1	1	1	1	1	1	1	1	1	11	CORE	COG1122_P#	48.9	508	3.04E-143	K16787	32	133	7.1E-30
1178	WP_019522482.1	-	hypothetical protein	1	1	0	1	1	1	1	1	1	1	1	10	Accessory								
1179	PWE07900.1	-	Na+/galactose cotransporter	1	1	1	1	1	1	1	1	1	1	1	11	CORE	COG4146_R#	26.9	154	8.52E-37	K03307	29.4	229	7.27E-59
1180	WP_019523103.1	-	hypothetical protein	0	0	0	0	1	1	0	0	0	0	1	3	Accessory								

cluster	GI	gene	Annotation	Streptomyces_thermocarboxydus_BPSAC147	Streptomyces_sp._4F	Streptomyces_sp._BSE7F	Streptomyces_sp._ETH9427	Streptomyces_sp._FxanaD5	Streptomyces_sp._UNC401CLCol	Actinospica_acidiphila_NRRL_B_24431	Streptomyces_sp._HNS054	Streptomyces_sp._SMS_SU21	Streptomyces_sp._ZS0098	Streptomyces_thermocarboxydus_K155	Addition	Core-Accessory-Unique	COG	%Similarity_COG	Score_COG	Evalue_COG	KEGG	%Similarity_KEGG	Score_KEGG	Evalue_KEGG
1181	OSC69663.1	-	restriction endonuclease subunit R	0	1	1	0	0	0	0	0	0	0	0	2	Accessory	COG1061_KL#	31.5	131	1.32E-29	K17677	28.2	127	5.08E-28
1182	WP_019527358.1	-	PQQ-dependent dehydrogenase, methanol/ethanol family	0	0	0	0	1	1	0	0	0	0	0	2	Accessory	COG4993_G#	35.4	328	4.3E-89	K00117	29.4	142	1.17E-32
1183	WP_033274982.1	-	cytochrome P450	1	1	1	1	1	1	1	1	1	1	1	11	CORE	COG2124_Q#	25.7	131	1.01E-29	K16046	29.4	126	1.13E-27
1184	WP_102638233.1	-	response regulator	1	1	1	1	1	1	1	1	1	1	1	11	CORE	COG0492_O#	43.7	259	3.2E-68	K00384	33.3	168	2.59E-40
1185	WP_102641247.1	-	ABC-F family ATP-binding cassette domain-containing protein	0	1	0	0	1	1	1	0	1	0	1	6	Accessory	COG0488_R#	36.2	329	1.93E-89	NA	36.6	260	5.01E-68
1186	AXI88543.1	-	fumarate hydratase	1	1	1	1	1	1	1	1	1	1	1	11	CORE	COG1951_C#	64.8	467	5.93E-131	K01676	65	720	1.77E-206
1187	OSC61368.1	-	LuxR family transcriptional regulator	0	1	0	0	0	0	0	0	0	0	0	1	Unique	COG3899_R#	27.2	119	3.96E-26				
1188	Prokka:JAPCLKMA_03028	-	hypothetical protein	1	1	1	1	1	1	1	1	1	1	1	11	CORE	COG3525_G#	26.8	155	4.98E-37	K12373	26.8	155	1.33E-36
1189	WP_019526755.1	-	FAD-binding dehydrogenase	1	0	1	1	1	1	1	1	1	1	1	10	Accessory	COG3573_R#	62.9	711	2.35E-204	K07077	62.6	705	3.45E-202

cluster	GI	gene	Annotation	Streptomyces_thermocarboxydus_BPSAC147	Streptomyces_sp._4F	Streptomyces_sp._BSE7F	Streptomyces_sp._ETH9427	Streptomyces_sp._FxanaD5	Streptomyces_sp._UNC401CLCol	Actinospica_acidiphila_NRRL_B_24431	Streptomyces_sp._HNS054	Streptomyces_sp._SMS_SU21	Streptomyces_sp._ZS0098	Streptomyces_thermocarboxydus_K155	Addition	Core-Accessory-Unique	COG	%Similarity_COG	Score_COG	Evalue_COG	KEGG	%Similarity_KEGG	Score_KEGG	Evalue_KEGG
1190	WP_019521915.1	-	AMP-binding protein	1	1	1	1	1	1	1	1	1	1	1	11	CORE	COG0365_I#	63	707	3.4E-203	K01895	39	357	1.76E-97
1191	WP_048457776.1	-	PucR family transcriptional regulator	1	1	1	1	1	1	1	1	1	1	1	11	CORE	COG2508_TQ#	31.7	54	0.0000012	K09684	31.7	54	0.00000319
1192	PWE11384.1	-	IS1182 family transposase	0	0	1	0	0	0	0	0	0	0	0	1	Unique	COG3666_L#	49.4	527	6.29E-149				
1193	WP_048459377.1	-	iron ABC transporter permease	1	1	1	1	1	1	1	1	1	1	1	11	CORE	COG1178_P#	34.9	297	8.09E-80	K02063	35.8	258	1.45E-67
1194	OSC73359.1	-	acyl-CoA synthetase	0	1	0	0	0	0	0	0	0	0	0	1	Unique	COG0318_IQ#	34.9	317	7.56E-86	K01897	36.3	316	5.87E-85
1195	OSC60505.1	-	beta-mannosidase	1	1	1	1	0	1	1	1	1	1	1	10	Accessory	COG3979_R#	51.8	92	3.95E-18				
1196	WP_102640049.1	-	carbamoyltransferase	1	0	1	0	1	1	1	1	1	1	1	9	Accessory	COG2192_O#	38	333	1.74E-90	K00612	36.5	328	1.14E-88
1197	WP_048458286.1	-	alkaline phosphatase	1	1	1	1	1	1	1	1	1	1	1	11	CORE	COG3540_P#	47.6	454	6.76E-127	K01113	47.2	484	1.63E-135
1198	WP_019526441.1	ptsP	phosphoenolpyruvate--protein phosphotransferase	1	1	1	1	1	1	1	1	1	1	1	11	CORE	COG1080_G#	45.2	420	8.28E-117	K08483	37.5	329	5.13E-89
1199	AXI88871.1	-	ABC transporter substrate-binding protein	1	0	1	1	1	1	1	1	1	1	1	10	Accessory	COG0747_E#	32.6	263	1.69E-69	K02035	32.6	263	4.52E-69

cluster	GI	gene	Annotation	Streptomyces_thermocarboxydus_BPSAC147	Streptomyces_sp._4F	Streptomyces_sp._BSE7F	Streptomyces_sp._ETH9427	Streptomyces_sp._FxanaD5	Streptomyces_sp._UNC401CLCol	Actinospica_acidiphila_NRRL_B_24431	Streptomyces_sp._HNS054	Streptomyces_sp._SMS_SU21	Streptomyces_sp._ZS0098	Streptomyces_thermocarboxydus_K155	Addition	Core-Accessory-Unique	COG	%Similarity_COG	Score_COG	Evalue_COG	KEGG	%Similarity_KEGG	Score_KEGG	Evalue_KEGG
1200	Prokka:CIODPCDJ_05672	-	hypothetical protein	0	0	0	0	0	0	0	0	0	0	1	1	Unique								
1201	Prokka:JAPCLKMA_02005	-	hypothetical protein	1	0	0	0	0	0	0	0	0	0	0	1	Unique	COG0252_EJ#	31.2	67	1.36E-10	K01424	31.2	67	3.64E-10
1202	WP_102641327.1	-	segregation/condensation protein A	1	1	1	1	1	1	1	1	1	1	1	11	CORE	COG1354_S#	56	273	1.25E-72	K05896	54.9	271	2.17E-71
1203	AXI84851.1	-	hypothetical protein	0	0	0	1	0	0	0	0	0	0	0	1	Unique	COG2730_G#	31.2	81	9.1E-15	NA	40	113	7.54E-24
1204	WP_109288734.1	-	CHAD domain-containing protein	1	0	1	1	1	1	1	1	1	1	1	10	Accessory					NA	28.8	70	7.32E-11
1205	WP_122213387.1	-	RecQ family ATP-dependent DNA helicase	1	0	1	1	1	1	1	1	1	1	1	10	Accessory	COG0514_L#	42.7	267	8.95E-71	K03654	36.9	278	1.77E-73
1206	WP_028959356.1	-	amidohydrolase family protein	1	0	0	0	1	1	0	0	0	0	1	4	Accessory	COG1574_R#	28.4	166	2.81E-40	K07047	27.6	154	2.26E-36
1207	OSC63211.1	-	two-component sensor histidine kinase	1	1	1	1	1	1	1	1	1	1	1	11	CORE	COG0642_T#	32	190	1.82E-47	K07654	31.9	190	4.85E-47
1208	WP_102638052.1	-	Hsp70 family protein	0	0	0	0	0	0	0	0	1	0	0	1	Unique	COG0443_O#	26.9	108	6.95E-23				

cluster	GI	gene	Annotation	Streptomyces_thermocarboxydus_BPSAC147	Streptomyces_sp._4F	Streptomyces_sp._BSE7F	Streptomyces_sp._ETH9427	Streptomyces_sp._FxanaD5	Streptomyces_sp._UNC401CLCol	Actinospica_acidiphila_NRRL_B_24431	Streptomyces_sp._HNS054	Streptomyces_sp._SMS_SU21	Streptomyces_sp._ZS0098	Streptomyces_thermocarboxydus_K155	Addition	Core-Accessory-Unique	COG	%Similarity_COG	Score_COG	Evalue_COG	KEGG	%Similarity_KEGG	Score_KEGG	Evalue_KEGG
1209	WP_051724391.1	abc-f	TlrC/CarA/OleB/SrmB family ABC-F type ribosomal protection protein	1	1	1	1	1	1	1	1	1	1	1	11	CORE	COG0488_R#	34.9	289	2.2E-77	NA	37.6	268	1.07E-70
1210	Prokka:JAPCLKMA_06577	ettA_4	Energy-dependent translational throttle protein EttA	1	1	1	1	1	1	1	1	1	1	1	11	CORE	COG0488_R#	80.3	913	3.1E-265	NA	80.1	912	2.41E-264
1211	WP_102637575.1	-	serine/threonine protein kinase	1	0	0	1	1	1	1	1	1	1	1	9	Accessory	COG0515_RTKL#	46	270	1.06E-71	K08884	45	265	1.18E-69
1212	OSC65846.1	-	allantoin permease	0	1	0	0	0	0	0	0	0	0	0	1	Unique					NA	62.3	540	1.91E-152
1213	WP_048460731.1	-	serine/threonine protein kinase	1	1	1	1	1	1	1	1	1	1	1	11	CORE	COG0515_RTKL#	32.9	177	1.59E-43	K08884	32.7	166	7.49E-40
1214	WP_106432514.1	-	SpoIIE family protein phosphatase	1	0	1	1	1	1	1	1	1	1	1	10	Accessory	COG2208_TK#	34.9	79	3.45E-14	K02485	27.7	70	4.28E-11
1215	AXI88145.1	-	recombinase family protein	0	0	0	1	0	0	0	0	0	0	0	1	Unique								
1216	WP_122214609.1	-	penicillin-binding protein	1	1	1	1	1	1	1	1	1	1	1	11	CORE								
1217	OSC65881.1	-	Na+/H+ antiporter subunit D	0	1	0	0	0	0	0	0	0	0	0	1	Unique	COG0651_CP#	49.3	480	1.14E-134	K05568	36.2	289	5.85E-77

cluster	GI	gene	Annotation	Streptomyces_thermocarboxydus_BPSAC147	Streptomyces_sp._4F	Streptomyces_sp._BSE7F	Streptomyces_sp._ETH9427	Streptomyces_sp._FxanaD5	Streptomyces_sp._UNC401CLCol	Actinospica_acidiphila_NRRL_B_24431	Streptomyces_sp._HNS054	Streptomyces_sp._SMS_SU21	Streptomyces_sp._ZS0098	Streptomyces_thermocarboxydus_K155	Addition	Core-Accessory-Unique	COG	%Similarity_COG	Score_COG	Evalue_COG	KEGG	%Similarity_KEGG	Score_KEGG	Evalue_KEGG
1218	WP_028958942.1	narH	nitrate reductase subunit beta	1	1	0	0	1	1	0	0	0	0	1	5	Accessory	COG1140_C#	60.9	690	5.57E-198	K00371	60.9	690	1.49E-197
1219	WP_019526216.1	-	trypsin-like peptidase domain-containing protein	0	1	0	0	1	1	0	0	0	0	0	3	Accessory								
1220	OSC62545.1	-	hypothetical protein	1	1	1	1	1	1	1	1	1	1	1	11	CORE								
1221	WP_048457038.1	-	ABC transporter permease	0	1	1	1	0	0	1	1	1	1	0	7	Accessory	COG3559_M#	42.2	438	3.82E-122	K01992	40.5	371	2E-101
1222	WP_019525845.1	-	HAMP domain-containing histidine kinase	1	0	0	0	1	1	0	0	0	0	1	4	Accessory	COG0642_T#	38.2	263	1.29E-69	K02484	37.8	248	1.95E-64
1223	OSC63820.1	-	catalase	1	1	1	1	1	1	1	1	1	1	1	11	CORE	COG0753_P#	62.4	656	8.9E-188	K03781	61.9	637	1.14E-181
1224	OSC71270.1	-	NADH-quinone oxidoreductase subunit N	1	1	1	1	1	1	1	1	1	1	1	11	CORE	COG1007_C#	56.9	589	1.34E-167	K00343	56.9	589	3.57E-167
1225	Prokka:CIODPCDJ_05221	-	hypothetical protein	1	0	1	1	1	1	1	1	1	1	1	10	Accessory								
1226	Prokka:JAPCLKMA_00240	pgi_1	Glucose-6-phosphate isomerase	1	1	1	1	0	1	1	0	1	1	1	9	Accessory	COG0166_G#	68.5	730	6.32E-210	K01810	68.5	730	1.69E-209

cluster	GI	gene	Annotation	Streptomyces_thermocarboxydus_BPSAC147	Streptomyces_sp._4F	Streptomyces_sp._BSE7F	Streptomyces_sp._ETH9427	Streptomyces_sp._FxanaD5	Streptomyces_sp._UNC401CLCol	Actinospica_acidiphila_NRRL_B_24431	Streptomyces_sp._HNS054	Streptomyces_sp._SMS_SU21	Streptomyces_sp._ZS0098	Streptomyces_thermocarboxydus_K155	Addition	Core-Accessory-Unique	COG	%Similarity_COG	Score_COG	Evalue_COG	KEGG	%Similarity_KEGG	Score_KEGG	Evalue_KEGG
1227	Prokka:CIODPCDJ_01717	murE	UDP-N-acetylmuramoyl-L-alanyl-D-glutamate--2, 6-diaminopimelate ligase	1	1	0	0	1	1	1	1	1	1	1	9	Accessory	COG0769_M#	47.4	441	5.86E-123	K01928	50.6	438	1.02E-121
1228	WP_122216278.1	-	multicopper oxidase family protein	0	0	1	0	0	0	0	0	1	1	0	3	Accessory	COG2132_Q#	35.3	290	1.28E-77	NA	35.1	187	3.12E-46
1229	WP_122215791.1	-	sporulation domain-containing protein	1	1	1	1	1	1	1	1	1	1	1	11	CORE	COG4632_G#	35.8	72	7.14E-12	NA	25.6	76	7.75E-13
1230	WP_028961286.1	-	AAA family ATPase	0	0	0	0	0	1	0	0	0	0	0	1	Unique	COG0507_L#	40	42	0.00606				
1231	AXI87965.1	-	transcriptional regulator	1	1	1	1	1	1	1	1	1	1	1	11	CORE	COG1316_K#	26.5	145	6.64E-34	NA	26.5	145	1.77E-33
1232	Prokka:CIODPCDJ_02362	-	Methylmalonyl-CoA carboxyltransferase 12S subunit	1	1	1	1	1	1	1	1	1	1	1	11	CORE	COG4799_I#	78.3	829	5.85E-240	K01969	78.3	829	1.56E-239
1233	OSC58523.1	-	MFS transporter	0	1	0	0	0	0	0	0	0	0	0	1	Unique	COG0477_GEPR#	41.4	413	1.71E-114	NA	28	166	9.71E-40
1234	OSC70561.1	-	hypothetical protein	0	1	0	0	0	0	0	0	0	0	0	1	Unique					NA	36.2	115	1.5E-24
1235	OSC53641.1	-	hypothetical protein	0	1	0	0	0	0	0	0	0	0	0	1	Unique	COG3321_Q#	30	162	5.25E-39	K13613	29.8	107	4.1E-22
1236	Prokka:JAPCLKMA_05143	-	hypothetical protein	1	0	0	1	1	0	0	0	0	1	0	4	Accessory	COG5479_M#	36.6	191	6.19E-48	NA	36.6	191	1.65E-47

cluster	GI	gene	Annotation	Streptomyces_thermocarboxydus_BPSAC147	Streptomyces_sp._4F	Streptomyces_sp._BSE7F	Streptomyces_sp._ETH9427	Streptomyces_sp._FxanaD5	Streptomyces_sp._UNC401CLCol	Actinospica_acidiphila_NRRL_B_24431	Streptomyces_sp._HNS054	Streptomyces_sp._SMS_SU21	Streptomyces_sp._ZS0098	Streptomyces_thermocarboxydus_K155	Addition	Core-Accessory-Unique	COG	%Similarity_COG	Score_COG	Evalue_COG	KEGG	%Similarity_KEGG	Score_KEGG	Evalue_KEGG
1237	WP_122213839.1	-	metallophosphoesterase	1	1	1	1	1	1	1	1	1	1	1	11	CORE								
1238	Prokka:CIODPCDJ_02281	-	hypothetical protein	1	1	1	1	1	1	1	1	1	1	1	11	CORE								
1239	AXI85992.1	-	CTP synthase	1	1	1	1	1	1	1	1	1	1	1	11	CORE	COG0504_F#	70.7	787	4.34E-227	K01937	70.7	787	1.16E-226
1240	WP_122215548.1	-	phospho-sugar mutase	1	1	1	1	1	1	1	1	1	1	1	11	CORE	COG1109_G#	51.4	486	1.21E-136	K01840	44.2	401	1.79E-110
1241	WP_033272793.1	-	alkaline phosphatase family protein	1	0	0	1	1	1	1	1	1	1	1	9	Accessory								
1242	WP_102637406.1	-	hypothetical protein	0	0	0	0	0	0	0	0	1	0	0	1	Unique								
1243	WP_122213022.1	-	alpha/beta hydrolase	1	1	0	1	1	1	1	1	1	1	1	10	Accessory	COG0596_R#	37	187	1.52E-46	NA	37	187	4.06E-46
1244	OSC61424.1	-	peptide synthetase	0	1	0	0	0	0	0	0	0	0	0	1	Unique	COG1020_Q#	34.5	191	6.17E-48	K04792	34.5	191	1.65E-47
1245	OSC65373.1	-	glycosyltransferase	0	1	0	0	0	0	0	0	0	0	0	1	Unique								
1246	WP_122216387.1	-	ricin-type beta-trefoil lectin domain protein	1	0	1	1	1	1	1	1	1	1	1	10	Accessory								
1247	AXI91334.1	-	hypothetical protein	0	0	0	1	0	1	0	0	0	0	0	2	Accessory								
1248	AXI89545.1	-	alpha-galactosidase	0	0	1	1	0	0	0	1	1	1	0	5	Accessory					K07407	42.9	316	4.43E-85
1249	Prokka:CIODPCDJ_06356	-	hypothetical protein	0	0	0	0	0	0	0	0	0	0	1	1	Unique								

cluster	GI	gene	Annotation	Streptomyces_thermocarboxydus_BPSAC147	Streptomyces_sp._4F	Streptomyces_sp._BSE7F	Streptomyces_sp._ETH9427	Streptomyces_sp._FxanaD5	Streptomyces_sp._UNC401CLCol	Actinospica_acidiphila_NRRL_B_24431	Streptomyces_sp._HNS054	Streptomyces_sp._SMS_SU21	Streptomyces_sp._ZS0098	Streptomyces_thermocarboxydus_K155	Addition	Core-Accessory-Unique	COG	%Similarity_COG	Score_COG	Evalue_COG	KEGG	%Similarity_KEGG	Score_KEGG	Evalue_KEGG
1250	Prokka:CIODPCDJ_02079	-	Transglutaminase-activating metalloprotease	1	1	1	1	1	1	1	1	1	1	1	11	CORE	COG3227_E#	32.3	212	4.41E-54	K08777	32.3	212	1.18E-53
1251	Prokka:JAPCLKMA_03954	-	hypothetical protein	1	1	1	1	1	1	1	1	1	1	1	11	CORE								
1252	OSC61474.1	-	ATPase	0	1	0	0	0	0	0	0	0	0	0	1	Unique	COG3290_T#	32	219	2.76E-56	K11637	29.3	198	2.3E-49
1253	AXI91087.1	-	ubiquinol-cytochrome c reductase cytochrome b subunit	1	1	0	1	0	0	1	1	0	0	0	5	Accessory	COG1290_C#	51.3	499	1.38E-140	K03891	51.6	498	6.3E-140
1254	OSC67743.1	-	hypothetical protein	0	1	0	0	0	0	0	0	0	0	0	1	Unique								
1255	AXI91272.1	-	IS21 family transposase	0	0	0	1	0	0	0	0	0	0	0	1	Unique	COG4584_L#	29.8	190	1.79E-47				
1256	PWE08591.1	-	PE-PGRS family protein	0	0	1	0	0	0	0	0	0	0	0	1	Unique					NA	41.7	52	0.0000119
1257	Prokka:JAPCLKMA_04233	-	hypothetical protein	1	0	0	0	1	1	0	1	0	0	1	5	Accessory					NA	31.5	72	1.9E-11
1258	WP_122212721.1	-	beta-N-acetylhexosaminidase	1	1	1	1	1	1	1	1	1	1	1	11	CORE	COG3525_G#	44	431	6.03E-120	K12373	39.6	406	3.25E-112
1259	WP_048460069.1	-	DEAD/DEAH box helicase	1	1	1	1	1	1	1	1	1	1	1	11	CORE	COG1061_KL#	75.7	832	1.17E-240	K10843	75.7	832	3.13E-240
1260	WP_122215919.1	eccB	type VII secretion protein EccB	0	0	1	0	1	1	1	1	1	1	1	8	Accessory					NA	29.3	150	4.2E-35

cluster	GI	gene	Annotation	Streptomyces_thermocarboxydus_BPSAC147	Streptomyces_sp._4F	Streptomyces_sp._BSE7F	Streptomyces_sp._ETH9427	Streptomyces_sp._FxanaD5	Streptomyces_sp._UNC401CLCol	Actinospica_acidiphila_NRRL_B_24431	Streptomyces_sp._HNS054	Streptomyces_sp._SMS_SU21	Streptomyces_sp._ZS0098	Streptomyces_thermocarboxydus_K155	Addition	Core-Accessory-Unique	COG	%Similarity_COG	Score_COG	Evalue_COG	KEGG	%Similarity_KEGG	Score_KEGG	Evalue_KEGG
1261	PWE11301.1	-	AI-2E family transporter	1	1	1	1	1	1	1	1	1	1	1	11	CORE	COG0628_R#	39.1	257	9.13E-68	NA	39.1	257	2.44E-67
1262	OSC70397.1	-	transcriptional regulator	1	1	1	1	1	1	1	1	1	1	1	11	CORE	COG2508_TQ#	29	103	2.88E-21	NA	28.1	68	2.74E-10
1263	WP_048458315.1	-	alpha-D-glucose phosphate-specific phosphoglucomutase	1	0	1	1	1	1	1	1	1	1	1	10	Accessory	COG0033_G#	70.1	768	2.08E-221	K01835	70.1	768	5.54E-221
1264	Prokka:JAPCLKMA_03918	mhpA_2	3-(3-hydroxy-phenyl)propionate/3-hydroxycinnamic acid hydroxylase	1	1	1	1	1	1	1	1	1	1	1	11	CORE	COG0654_HC#	28.5	155	6.37E-37	K05712	26.2	119	7.92E-26
1265	WP_028960824.1	-	serine/threonine protein kinase	1	1	1	1	1	1	1	1	1	1	1	11	CORE	COG0515_RTKL#	41.3	298	4.66E-80	K08884	41.1	298	1.24E-79
1266	AXI89856.1	-	heme ABC transporter ATP-binding protein	1	0	1	1	1	1	1	1	1	1	1	10	Accessory	COG0488_R#	58.6	607	4.69E-173	NA	38	299	7.29E-80
1267	AXI85307.1	-	hypothetical protein	1	0	0	1	0	0	0	0	0	0	0	2	Accessory								

cluster	GI	gene	Annotation	Streptomyces_thermocarboxydus_BPSAC147	Streptomyces_sp._4F	Streptomyces_sp._BSE7F	Streptomyces_sp._ETH9427	Streptomyces_sp._FxanaD5	Streptomyces_sp._UNC401CLCol	Actinospica_acidiphila_NRRL_B_24431	Streptomyces_sp._HNS054	Streptomyces_sp._SMS_SU21	Streptomyces_sp._ZS0098	Streptomyces_thermocarboxydus_K155	Addition	Core-Accessory-Unique	COG	%Similarity_COG	Score_COG	Evalue_COG	KEGG	%Similarity_KEGG	Score_KEGG	Evalue_KEGG
1268	OSC61607.1	-	2,3-dihydroxybenzoate-AMP ligase	0	1	0	0	0	0	0	0	0	0	0	1	Unique	COG1021_Q#	52	531	4.26E-150	K02363	52.1	531	1.14E-149
1269	WP_122213798.1	-	tyrosine-type recombinase/integrase	0	0	0	0	1	0	0	0	0	1	0	2	Accessory								
1270	OSC66432.1	-	penicillin-binding protein	1	1	1	1	1	1	1	1	1	1	1	11	CORE	COG0768_M#	31.6	156	2.85E-37	K05364	29.1	104	3.44E-21
1271	OSC73181.1	-	ubiquinol-cytochrome c reductase cytochrome b subunit	0	1	1	0	1	1	0	1	1	1	1	8	Accessory	COG1290_C#	47.2	527	8.04E-149	K03891	50.1	525	8.16E-148
1272	OSC61631.1	-	hypothetical protein	0	1	0	0	0	0	0	0	0	0	0	1	Unique	COG3321_Q#	51.5	279	2.24E-74	K12434	51.5	279	5.97E-74
1273	WP_102637306.1	-	DUF1254 domain-containing protein	0	0	0	0	0	0	0	0	1	1	0	2	Accessory	COG5361_S#	44.2	221	5.52E-57				
1274	WP_122215771.1	-	hypothetical protein	1	0	0	0	0	0	0	0	0	1	1	3	Accessory	COG0328_L#	35.4	50	0.0000288				
1275	WP_033272388.1	-	hypothetical protein	1	1	1	1	1	1	1	1	1	1	1	11	CORE								
1276	WP_107685284.1	-	helix-turn-helix domain-containing protein	1	0	0	0	1	1	1	1	1	1	1	8	Accessory	COG1396_K#	39.2	42	0.00783				
1277	OSC60213.1	-	alpha-mannosidase	0	1	0	0	0	0	0	0	0	0	0	1	Unique	COG3537_G#	39.4	348	3.91E-95	NA	39.4	348	1.04E-94

cluster	GI	gene	Annotation	Streptomyces_thermocarboxydus_BPSAC147	Streptomyces_sp._4F	Streptomyces_sp._BSE7F	Streptomyces_sp._ETH9427	Streptomyces_sp._FxanaD5	Streptomyces_sp._UNC401CLCol	Actinospica_acidiphila_NRRL_B_24431	Streptomyces_sp._HNS054	Streptomyces_sp._SMS_SU21	Streptomyces_sp._ZS0098	Streptomyces_thermocarboxydus_K155	Addition	Core-Accessory-Unique	COG	%Similarity_COG	Score_COG	Evalue_COG	KEGG	%Similarity_KEGG	Score_KEGG	Evalue_KEGG
1278	OSC71439.1	-	cation acetate symporter	1	1	1	1	1	1	1	1	1	1	1	11	CORE	COG4147_R#	58	611	3.24E-174	K14393	54.8	535	6.02E-151
1279	OSC59784.1	-	ABC transporter	1	1	1	1	1	1	1	1	1	1	1	11	CORE	COG0488_R#	42.5	377	6.02E-104	NA	32.2	303	5.03E-81
1280	OSC61356.1	-	1-pyrroline-5-carboxylate dehydrogenase	1	1	1	1	1	1	1	1	1	1	1	11	CORE	COG1012_C#	66.1	728	2.37E-209	K00294	66.1	728	6.32E-209
1281	WP_122215884.1	-	hypothetical protein	1	0	0	1	0	1	0	1	0	1	1	6	Accessory								
1282	OSC57543.1	-	FAD-binding oxidoreductase	1	1	1	1	1	1	1	1	1	1	1	11	CORE	COG0277_C#	54.3	558	3.24E-158	K00803	54.3	558	8.66E-158
1283	OSC61737.1	-	acetolactate synthase	0	1	0	0	0	0	0	0	0	0	0	1	Unique	COG0028_EH#	44.7	428	5.06E-119	K01652	30.3	198	2.28E-49
1284	WP_122215323.1	groL	chaperonin GroEL	1	1	1	1	1	1	1	1	1	1	1	11	CORE	COG0459_O#	68.3	706	7.36E-203	K04077	68.1	705	3.35E-202
1285	WP_033272050.1	-	sodium:solute symporter family protein	1	1	1	1	1	1	1	1	1	1	1	11	CORE	COG0591_ER#	42.7	403	1.02E-111	K03307	33.7	283	4.11E-75
1286	Prokka:CIODPCDJ_00705	-	hypothetical protein	1	0	0	0	1	1	0	0	0	0	1	4	Accessory	COG4585_T#	25.5	74	1.85E-12	K02480	24.7	78	2.62E-13
1287	OSC65061.1	-	peptide ABC transporter substrate-binding protein	1	1	1	1	1	1	1	1	1	1	1	11	CORE	COG4166_E#	40.6	388	3.4E-107	K15580	39.1	351	1.61E-95

cluster	GI	gene	Annotation	Streptomyces_thermocarboxydus_BPSAC147	Streptomyces_sp._4F	Streptomyces_sp._BSE7F	Streptomyces_sp._ETH9427	Streptomyces_sp._FxanaD5	Streptomyces_sp._UNC401CLCol	Actinospica_acidiphila_NRRL_B_24431	Streptomyces_sp._HNS054	Streptomyces_sp._SMS_SU21	Streptomyces_sp._ZS0098	Streptomyces_thermocarboxydus_K155	Addition	Core-Accessory-Unique	COG	%Similarity_COG	Score_COG	Evalue_COG	KEGG	%Similarity_KEGG	Score_KEGG	Evalue_KEGG
1288	OSC62169.1	-	hypothetical protein	0	1	0	0	0	0	0	0	0	0	0	1	Unique								
1289	WP_033274260.1	-	FAD-dependent oxidoreductase	0	0	0	0	0	0	1	0	0	0	0	1	Unique	COG0446_R#	30	50	0.0000219	NA	30.9	93	6.03E-18
1290	Prokka:JAPCLKMA_05677	-	hypothetical protein	1	0	0	0	0	0	0	0	0	0	0	1	Unique					NA	40	269	8.01E-71
1291	OSC73346.1	-	Mg chelatase-like protein	1	1	1	1	1	1	1	1	1	1	1	11	CORE	COG0606_O#	51.3	477	5.55E-134	K07391	51.3	477	1.48E-133
1292	AXI89486.1	-	malate synthase A	1	1	1	1	1	1	1	1	1	1	1	11	CORE	COG2225_C#	54.5	554	3.57E-157	K01638	54.5	554	9.54E-157
1293	PWE09318.1	-	ATP-binding protein	0	0	1	0	0	0	1	0	0	0	0	2	Accessory								
1294	WP_019525181.1	-	family 20 glycosylhydrolase	1	0	0	0	1	1	0	0	0	0	1	4	Accessory	COG3525_G#	33	271	7.89E-72	K12373	33.7	277	2.94E-73
1295	WP_019526064.1	-	hypothetical protein	0	0	0	0	1	0	0	0	0	0	0	1	Unique								
1296	WP_122216025.1	-	amino acid adenylation domain-containing protein	0	0	0	1	0	0	0	1	1	1	0	4	Accessory	COG1020_Q#	37.7	303	1.43E-81	K04780	34.3	266	5.19E-70
1297	OSC62851.1	-	glycogen debranching protein	0	1	0	0	0	0	0	0	0	0	0	1	Unique	COG3408_G#	26.9	87	2.11E-16				
1298	WP_033276425.1	-	serine/threonine protein kinase	1	1	1	1	1	1	1	1	1	1	1	11	CORE	COG0515_RTKL#	29.1	104	1.28E-21	K08884	29.1	104	3.41E-21

cluster	GI	gene	Annotation	Streptomyces_thermocarboxydus_BPSAC147	Streptomyces_sp._4F	Streptomyces_sp._BSE7F	Streptomyces_sp._ETH9427	Streptomyces_sp._FxanaD5	Streptomyces_sp._UNC401CLCol	Actinospica_acidiphila_NRRL_B_24431	Streptomyces_sp._HNS054	Streptomyces_sp._SMS_SU21	Streptomyces_sp._ZS0098	Streptomyces_thermocarboxydus_K155	Addition	Core-Accessory-Unique	COG	%Similarity_COG	Score_COG	Evalue_COG	KEGG	%Similarity_KEGG	Score_KEGG	Evalue_KEGG
1299	WP_102637686.1	-	molybdopterin-dependent oxidoreductase	0	1	0	1	0	0	0	1	1	1	0	5	Accessory	COG2041_R#	28.6	134	1.15E-30	K00387	30.3	117	2.98E-25
1300	WP_037778991.1	-	DEAD/DEAH box helicase	0	0	0	0	1	0	0	0	0	1	0	2	Accessory	COG1061_KL#	32.9	217	1.04E-55	K17677	32.9	217	2.77E-55
1301	OSC57508.1	-	diguanylate cyclase	0	1	0	0	0	0	0	0	0	0	0	1	Unique	COG2199_T#	37.6	84	1.79E-15	NA	27.5	73	8.42E-12
1302	OSC70241.1	-	amidohydrolase	0	1	0	0	0	0	0	0	0	0	0	1	Unique	COG1574_R#	34	235	6.27E-61	NA	27.5	183	4.43E-45
1303	Prokka:JAPCLKMA_05634	-	hypothetical protein	1	0	0	0	0	0	0	0	0	0	0	1	Unique								
1304	Prokka:JAPCLKMA_06562	-	hypothetical protein	1	0	0	0	0	0	0	0	0	0	0	1	Unique	COG1961_L#	26.9	107	1.51E-22	NA	32.3	80	6.89E-14
1305	WP_028959140.1	-	glycosyltransferase	1	0	1	1	1	1	1	1	1	1	1	10	Accessory	COG0463_M#	31.3	99	4.1E-20	K16698	39.6	67	3.53E-10
1306	WP_033277705.1	-	citramalate synthase	1	1	0	1	1	1	1	1	1	1	1	10	Accessory	COG0119_E#	47.8	476	9.45E-134	K01649	47.8	476	2.52E-133
1307	WP_033272243.1	-	VWA domain-containing protein	1	1	0	0	1	1	1	0	1	1	1	8	Accessory	COG2425_R#	25.1	111	7.99E-24	NA	30.6	64	5.1E-09
1308	WP_048456027.1	-	sugar phosphotransferase	0	0	0	1	0	0	1	1	0	1	0	4	Accessory								
1309	Prokka:CIODPCDJ_06658	cldB	Copalyl diphosphate synthase	0	0	0	0	1	1	0	0	0	0	1	3	Accessory					K18115	21.1	51	0.0000262
1310	WP_048456028.1	-	sugar phosphotransferase	0	0	0	1	0	0	1	1	0	1	0	4	Accessory					K01784	35	283	3.14E-75

cluster	GI	gene	Annotation	Streptomyces_thermocarboxydus_BPSAC147	Streptomyces_sp._4F	Streptomyces_sp._BSE7F	Streptomyces_sp._ETH9427	Streptomyces_sp._FxanaD5	Streptomyces_sp._UNC401CLCol	Actinospica_acidiphila_NRRL_B_24431	Streptomyces_sp._HNS054	Streptomyces_sp._SMS_SU21	Streptomyces_sp._ZS0098	Streptomyces_thermocarboxydus_K155	Addition	Core-Accessory-Unique	COG	%Similarity_COG	Score_COG	Evalue_COG	KEGG	%Similarity_KEGG	Score_KEGG	Evalue_KEGG
1311	OSC65501.1	-	alpha-glucosidase	1	1	1	1	1	1	1	1	1	1	1	11	CORE	COG0366_G#	51	533	6.52E-151	K01187	51	533	1.74E-150
1312	WP_102641612.1	-	FAD-dependent oxidoreductase	0	0	1	1	0	0	0	1	1	1	0	5	Accessory	COG0277_C#	33.3	235	4.79E-61	NA	25.9	162	1.05E-38
1313	OSC69509.1	-	ABC transporter	0	1	0	0	0	0	0	0	0	0	0	1	Unique	COG1132_V#	29.1	173	2.92E-42	K06147	29.1	173	7.8E-42
1314	Prokka:CIODPCDJ_01006	-	hypothetical protein	1	0	1	0	1	1	1	1	1	1	1	9	Accessory								
1315	WP_102638060.1	-	SAM-dependent DNA methyltransferase	0	0	0	0	0	0	0	0	1	0	0	1	Unique	COG0286_V#	58.8	664	2.44E-190	K03427	58.7	661	9.42E-189
1316	Prokka:CIODPCDJ_02560	-	hypothetical protein	1	1	1	1	0	0	1	1	1	1	1	9	Accessory								
1317	WP_122217427.1	-	recombinase family protein	1	0	0	1	0	1	1	1	1	1	1	8	Accessory	COG1961_L#	22.1	70	1.56E-11				
1318	Prokka:JAPCLKMA_00394	-	hypothetical protein	1	0	0	0	1	1	0	0	0	0	1	4	Accessory	COG1401_V#	32.7	173	2.91E-42	K07452	32.7	173	7.78E-42
1319	WP_102637413.1	-	serine/threonine protein kinase	1	0	0	0	0	0	0	0	1	1	1	4	Accessory	COG0515_RTKL#	33.1	198	6.48E-50	K08884	38.8	196	6.57E-49
1320	Prokka:JAPCLKMA_01885	-	putative GMC-type oxidoreductase	1	0	0	0	1	1	0	0	0	0	1	4	Accessory	COG2303_E#	50.1	463	1.41E-129	NA	50.1	463	3.76E-129

cluster	GI	gene	Annotation	Streptomyces_thermocarboxydus_BPSAC147	Streptomyces_sp._4F	Streptomyces_sp._BSE7F	Streptomyces_sp._ETH9427	Streptomyces_sp._FxanaD5	Streptomyces_sp._UNC401CLCol	Actinospica_acidiphila_NRRL_B_24431	Streptomyces_sp._HNS054	Streptomyces_sp._SMS_SU21	Streptomyces_sp._ZS0098	Streptomyces_thermocarboxydus_K155	Addition	Core-Accessory-Unique	COG	%Similarity_COG	Score_COG	Evalue_COG	KEGG	%Similarity_KEGG	Score_KEGG	Evalue_KEGG
1321	WP_106432464.1	-	ABC transporter ATP-binding protein	1	0	0	0	1	1	1	0	0	0	1	5	Accessory	COG1123_R#	36.6	289	3.64E-77	K02032	36.5	276	4.99E-73
1322	WP_019525047.1	-	glucose-6-phosphate dehydrogenase	1	1	1	1	1	1	1	1	1	1	1	11	CORE	COG0364_G#	71.2	745	1.09E-214	K00036	71.2	745	2.9E-214
1323	WP_019525897.1	-	methionine--tRNA ligase	1	1	1	1	1	1	1	1	1	1	1	11	CORE	COG0143_J#	46.7	481	3.82E-135	K01874	46	463	3.76E-129
1324	WP_019522714.1	-	glycerol-3-phosphate dehydrogenase/oxidase	1	1	0	1	1	1	1	1	1	1	1	10	Accessory	COG0578_C#	33.3	253	1.3E-66	K00111	35.4	275	1.45E-72
1325	WP_102642122.1	-	L-lactate permease	1	1	1	0	1	1	1	0	1	1	1	9	Accessory	COG1620_C#	41.4	416	1.97E-115	K03303	41.4	416	5.27E-115
1326	AXI91404.1	-	hypothetical protein	1	0	0	1	0	0	0	0	0	0	1	3	Accessory	COG1674_D#	25.6	72	6.99E-12	NA	32.1	96	9.24E-19
1327	Prokka:JAPCLKMA_02786	-	hypothetical protein	1	0	0	0	1	0	0	0	0	0	0	2	Accessory								
1328	OSC71188.1	-	MFS transporter	0	1	1	1	0	0	1	1	1	1	0	7	Accessory	COG0477_GEPR#	29.7	231	9E-60	NA	29.7	230	5.35E-59
1329	Prokka:CIODPCDJ_02694	mctC_2	Monocarboxylic acid transporter	1	1	1	1	1	1	1	1	1	1	1	11	CORE	COG4147_R#	34.2	307	7.57E-83	K14393	34.4	287	2.16E-76
1330	OSC70069.1	-	proteinase	1	1	1	1	1	1	1	1	1	1	1	11	CORE	COG0596_R#	37.6	309	1.99E-83	NA	37.6	309	5.32E-83
1331	AXI87699.1	-	hypothetical protein	0	0	0	1	0	0	0	0	0	0	0	1	Unique	COG1961_L#	25.5	96	2.65E-19	NA	25.5	96	7.07E-19

cluster	GI	gene	Annotation	Streptomyces_thermocarboxydus_BPSAC147	Streptomyces_sp._4F	Streptomyces_sp._BSE7F	Streptomyces_sp._ETH9427	Streptomyces_sp._FxanaD5	Streptomyces_sp._UNC401CLCol	Actinospica_acidiphila_NRRL_B_24431	Streptomyces_sp._HNS054	Streptomyces_sp._SMS_SU21	Streptomyces_sp._ZS0098	Streptomyces_thermocarboxydus_K155	Addition	Core-Accessory-Unique	COG	%Similarity_COG	Score_COG	Evalue_COG	KEGG	%Similarity_KEGG	Score_KEGG	Evalue_KEGG
1332	PWE09011.1	gabD2	succinic semialdehyde dehydrogenase	1	1	1	1	1	1	1	1	1	1	1	11	CORE	COG1012_C#	51.2	483	1E-135	K00135	51.2	483	2.68E-135
1333	Prokka:JAPCLKMA_02584	rsxC_2	Ion-translocating oxidoreductase complex subunit C	1	1	1	1	1	1	1	1	1	1	1	11	CORE	COG1894_C#	27.4	156	2.15E-37	K00335	25.9	127	4.88E-28
1334	WP_033275148.1	-	amidohydrolase	1	1	1	1	1	1	1	1	1	1	1	11	CORE	COG1574_R#	52.4	499	1.77E-140	K07047	52.4	499	4.72E-140
1335	OSC71577.1	-	apolipoprotein N-acyltransferase	1	1	1	1	1	1	1	1	1	1	1	11	CORE	COG0815_M#	45.7	414	4.39E-115	K03820	45.7	414	1.17E-114
1336	WP_122216209.1	-	bifunctional 3-(3-hydroxy-phenyl)propionate/3-hydroxycinnamic acid hydroxylase	0	0	0	0	1	1	1	1	0	1	1	6	Accessory	COG0654_HC#	41.8	411	4.85E-114	K05712	41.8	411	1.3E-113
1337	WP_102637111.1	-	ricin-type beta-trefoil lectin domain protein	0	1	0	0	0	1	1	1	1	1	1	7	Accessory					NA	34.3	54	0.00000402
1338	OSC72912.1	-	aminopeptidase	1	1	1	0	1	1	1	0	1	0	1	8	Accessory	COG0596_R#	32.1	219	3.53E-56	NA	32.1	219	9.43E-56
1339	AXI91091.1	-	xylosidase	0	0	1	1	0	0	0	1	1	1	0	5	Accessory	COG3507_G#	34.5	241	6.63E-63	NA	32.7	212	8.81E-54

cluster	GI	gene	Annotation	Streptomyces_thermocarboxydus_BPSAC147	Streptomyces_sp._4F	Streptomyces_sp._BSE7F	Streptomyces_sp._ETH9427	Streptomyces_sp._FxanaD5	Streptomyces_sp._UNC401CLCol	Actinospica_acidiphila_NRRL_B_24431	Streptomyces_sp._HNS054	Streptomyces_sp._SMS_SU21	Streptomyces_sp._ZS0098	Streptomyces_thermocarboxydus_K155	Addition	Core-Accessory-Unique	COG	%Similarity_COG	Score_COG	Evalue_COG	KEGG	%Similarity_KEGG	Score_KEGG	Evalue_KEGG
1340	AXI89481.1	-	iron transporter	1	1	0	1	1	1	1	1	1	1	1	10	Accessory								
1341	WP_033272235.1	-	D-aminoacylase	1	1	1	1	1	1	1	1	1	1	1	11	CORE	COG3653_Q#	41.6	376	1.73E-103	NA	29.5	72	1.42E-11
1342	Prokka:CIODPCDJ_02768	nhaP_1	K(+)/H(+) antiporter NhaP	1	1	1	1	1	1	1	1	1	1	1	11	CORE	COG3263_P#	35.8	314	6.17E-85	K11105	36.5	295	1.35E-78
1343	WP_048456300.1	-	alpha/beta hydrolase	0	0	1	1	0	0	0	1	1	1	0	5	Accessory	COG0596_R#	30.4	191	1.03E-47	NA	30.4	191	2.75E-47
1344	OSC70332.1	-	NAD(P)-dependent oxidoreductase	1	1	1	1	1	1	1	1	1	1	1	11	CORE	COG0702_MG#	45.5	433	1.55E-120	NA	33	263	4.35E-69
1345	PWE06720.1	-	peptidase	1	1	1	0	1	1	1	1	1	1	1	10	Accessory	COG1994_R#	38.8	159	4.33E-38	NA	32.1	182	1.28E-44
1346	OSC61411.1	-	protease	1	1	1	1	1	1	1	1	1	1	1	11	CORE	COG0596_R#	31.6	208	8.13E-53	NA	31.6	208	2.17E-52
1347	WP_106432538.1	-	helix-turn-helix domain-containing protein	1	0	1	1	1	1	1	1	1	1	1	10	Accessory								
1348	WP_019522708.1	-	peptide-binding protein	1	1	0	1	1	1	1	1	1	1	1	10	Accessory	COG0747_E#	31.4	243	2.28E-63	K02035	30.9	234	2.82E-60
1349	Prokka:JAPCLKMA_05968	-	hypothetical protein	1	1	1	1	1	1	0	1	1	1	1	10	Accessory	COG0642_T#	29.8	120	1.7E-26	NA	29.8	120	4.55E-26
1350	OSC72424.1	-	AMP-binding protein	1	1	0	0	1	1	1	0	0	0	1	6	Accessory	COG0318_IQ#	61.6	666	6.38E-191	K00666	61.6	666	1.7E-190
1351	WP_122215363.1	-	hypothetical protein	1	1	0	1	0	0	0	0	0	1	1	5	Accessory								
1352	WP_048457294.1	-	LCP family protein	1	0	0	1	1	1	1	1	1	1	1	9	Accessory	COG1316_K#	28.4	149	2.62E-35	NA	26.7	128	2.18E-28

cluster	GI	gene	Annotation	Streptomyces_thermocarboxydus_BPSAC147	Streptomyces_sp._4F	Streptomyces_sp._BSE7F	Streptomyces_sp._ETH9427	Streptomyces_sp._FxanaD5	Streptomyces_sp._UNC401CLCol	Actinospica_acidiphila_NRRL_B_24431	Streptomyces_sp._HNS054	Streptomyces_sp._SMS_SU21	Streptomyces_sp._ZS0098	Streptomyces_thermocarboxydus_K155	Addition	Core-Accessory-Unique	COG	%Similarity_COG	Score_COG	Evalue_COG	KEGG	%Similarity_KEGG	Score_KEGG	Evalue_KEGG
1353	WP_033276467.1	-	NAD(P)/FAD-dependent oxidoreductase	1	1	1	1	1	1	1	1	1	1	1	11	CORE	COG1233_Q#	52.2	513	6.9E-145	NA	52.2	513	1.84E-144
1354	AXI91260.1	-	hypothetical protein	0	0	0	1	0	0	0	0	0	0	0	1	Unique								
1355	AXI90181.1	-	phospholipid carrier-dependent glycosyltransferase	0	0	1	1	0	0	0	1	1	1	0	5	Accessory								
1356	PWE07658.1	-	hypothetical protein	1	1	1	1	1	1	1	1	1	1	1	11	CORE								
1357	WP_051724506.1	dacB	D-alanyl-D-alanine carboxypeptidase/D-alanyl-D-alanine-endopeptidase	1	1	1	0	1	1	1	1	1	1	1	10	Accessory	COG2027_M#	34.3	231	8.92E-60	K07259	34.3	231	2.38E-59
1358	Prokka:JAPCLKMA_02688	qacA_2	Antiseptic resistance protein	1	0	1	1	1	1	1	1	1	1	1	10	Accessory	COG0477_GEPR#	36.8	297	1.01E-79	NA	32.8	240	5.13E-62
1359	PWE08117.1	-	restriction endonuclease	0	0	1	0	0	0	0	0	0	0	0	1	Unique	COG1715_V#	28.4	62	5.49E-09	K07448	28.4	62	1.46E-08
1360	OSC70032.1	-	MFS transporter	1	1	0	1	1	1	1	1	1	1	1	10	Accessory	COG0477_GEPR#	40.5	398	4.22E-110	NA	39.3	360	3.4E-98
1361	OSC61240.1	-	peptidase M23	1	1	1	1	1	1	1	1	1	1	1	11	CORE	COG0739_M#	30.1	104	1.26E-21	NA	32.1	98	2.41E-19
1362	PWE06887.1	-	ABC transporter	1	1	1	1	1	1	1	1	1	1	1	11	CORE	COG0488_R#	64	689	1.19E-197	NA	64	689	3.19E-197

cluster	GI	gene	Annotation	Streptomyces_thermocarboxydus_BPSAC147	Streptomyces_sp._4F	Streptomyces_sp._BSE7F	Streptomyces_sp._ETH9427	Streptomyces_sp._FxanaD5	Streptomyces_sp._UNC401CLCol	Actinospica_acidiphila_NRRL_B_24431	Streptomyces_sp._HNS054	Streptomyces_sp._SMS_SU21	Streptomyces_sp._ZS0098	Streptomyces_thermocarboxydus_K155	Addition	Core-Accessory-Unique	COG	%Similarity_COG	Score_COG	Evalue_COG	KEGG	%Similarity_KEGG	Score_KEGG	Evalue_KEGG
1363	WP_122216783.1	-	glycosyl hydrolase 43 family protein	1	1	1	1	1	1	1	1	1	1	1	11	CORE	COG3507_G#	31.4	164	7.83E-40	NA	47.8	383	3.74E-105
1364	AXI88484.1	-	hypothetical protein	1	0	0	1	1	0	0	0	0	1	0	4	Accessory								
1365	WP_037953033.1	-	PASTA domain-containing protein	0	0	0	0	0	1	0	0	0	0	0	1	Unique	COG2730_G#	58.1	62	5.48E-09	K07604	43.2	65	1.73E-09
1366	WP_048457021.1	-	hypothetical protein	1	1	1	1	1	1	1	1	1	1	1	11	CORE					K00901	39	51	0.0000258
1367	OSC61981.1	-	transporter	1	1	1	1	1	1	1	1	1	1	1	11	CORE								
1368	OSC72088.1	-	spermidine synthase	0	1	0	0	0	0	0	0	0	0	0	1	Unique	COG4262_R#	45.8	433	9.04E-121	K00797	45.8	433	2.41E-120
1369	WP_102640139.1	-	alpha/beta hydrolase	1	1	1	1	1	1	1	1	1	1	1	11	CORE	COG0596_R#	34.2	275	3.14E-73	NA	34.2	275	8.4E-73
1370	WP_026243838.1	casA	type I-E CRISPR-associated protein Cse1/CasA	0	0	0	0	1	0	0	0	0	0	1	2	Accessory								
1371	WP_048459123.1	-	nickel transporter	1	1	1	1	1	1	1	1	1	1	1	11	CORE	COG2215_R#	33.6	147	1.29E-34	K08970	30.5	122	1.19E-26
1372	OSC69542.1	-	hypothetical protein	0	1	0	0	0	0	0	0	0	0	0	1	Unique	COG3540_P#	46.6	480	1.1E-134	K01113	48.7	503	2.48E-141
1373	WP_048456067.1	-	ABC transporter substrate-binding protein	1	1	1	1	1	1	1	1	1	1	1	11	CORE	COG0747_E#	32.1	243	1.73E-63	K02035	26.5	163	7.92E-39
1374	WP_086020197.1	-	peptide-binding protein	1	1	1	1	1	1	1	1	1	1	1	11	CORE	COG0747_E#	34.2	270	1.01E-71	K02035	34.2	270	2.7E-71
1375	WP_019521934.1	-	Na+/H+ antiporter	1	1	1	1	1	1	1	1	1	1	1	11	CORE	COG0025_P#	37.1	315	2.74E-85	K03316	33.9	253	5.82E-66

cluster	GI	gene	Annotation	Streptomyces_thermocarboxydus_BPSAC147	Streptomyces_sp._4F	Streptomyces_sp._BSE7F	Streptomyces_sp._ETH9427	Streptomyces_sp._FxanaD5	Streptomyces_sp._UNC401CLCol	Actinospica_acidiphila_NRRL_B_24431	Streptomyces_sp._HNS054	Streptomyces_sp._SMS_SU21	Streptomyces_sp._ZS0098	Streptomyces_thermocarboxydus_K155	Addition	Core-Accessory-Unique	COG	%Similarity_COG	Score_COG	Evalue_COG	KEGG	%Similarity_KEGG	Score_KEGG	Evalue_KEGG
1376	WP_033274778.1	-	phage tail sheath family protein	0	1	0	1	0	0	1	1	1	1	0	6	Accessory	COG3497_R#	40.1	349	2.23E-95				
1377	Prokka:JAPCLKMA_00977	dan_1	D-aminoacylase	1	0	0	0	1	1	0	0	0	0	1	4	Accessory	COG3653_Q#	39.2	381	4.06E-105	NA	33.6	107	3.95E-22
1378	PWE10978.1	-	methylmalonyl-CoA mutase	1	1	1	1	1	1	1	0	1	1	1	10	Accessory	COG1884_I#	64	659	1.32E-188	K01848	50.1	529	4.2E-149
1379	Prokka:CIODPCDJ_04216	accD5	putative propionyl-CoA carboxylase beta chain 5	1	1	1	1	1	1	1	1	1	1	1	11	CORE	COG4799_I#	68.3	718	2.39E-206	K01966	68.3	718	6.37E-206
1380	PWE09412.1	-	F0F1 ATP synthase subunit alpha	1	1	1	1	1	1	1	1	1	1	1	11	CORE	COG0056_C#	70.6	745	1.07E-214	K02111	70.6	745	2.85E-214
1381	WP_122215741.1	-	MFS transporter	0	0	1	1	0	0	0	1	1	1	0	5	Accessory	COG0477_GEPR#	41	369	2.08E-101	NA	26.7	179	8.16E-44
1382	WP_033275992.1	-	phosphoglycerate dehydrogenase	1	1	1	1	1	1	1	1	1	1	1	11	CORE	COG0111_HE#	56.1	563	7.52E-160	K00058	57	559	2.9E-158
1383	Prokka:CIODPCDJ_00168	proP_2	Proline/betaine transporter	1	1	1	1	1	1	1	1	1	1	1	11	CORE	COG0477_GEPR#	47.4	455	3.77E-127	K03762	44.8	451	1.45E-125
1384	OSC63024.1	-	phosphatase	1	1	0	1	1	1	1	1	1	1	1	10	Accessory	COG0784_T#	32.4	60	1.58E-08	NA	27.7	62	1.45E-08
1385	WP_048459186.1	-	hypothetical protein	0	0	0	0	0	0	0	1	0	0	0	1	Unique								

cluster	GI	gene	Annotation	Streptomyces_thermocarboxydus_BPSAC147	Streptomyces_sp._4F	Streptomyces_sp._BSE7F	Streptomyces_sp._ETH9427	Streptomyces_sp._FxanaD5	Streptomyces_sp._UNC401CLCol	Actinospica_acidiphila_NRRL_B_24431	Streptomyces_sp._HNS054	Streptomyces_sp._SMS_SU21	Streptomyces_sp._ZS0098	Streptomyces_thermocarboxydus_K155	Addition	Core-Accessory-Unique	COG	%Similarity_COG	Score_COG	Evalue_COG	KEGG	%Similarity_KEGG	Score_KEGG	Evalue_KEGG
1386	Prokka:CIODPCDJ_03333	pstP	PP2C-family Ser/Thr phosphatase	1	1	1	1	1	1	1	1	1	1	1	11	CORE	COG0631_T#	60.1	318	5.5E-86	K01090	44.4	345	8.6E-94
1387	Prokka:CIODPCDJ_04029	-	putative type I restriction enzymeP M protein	0	0	0	0	0	0	0	0	0	0	1	1	Unique	COG0286_V#	49.2	500	7.82E-141	K03427	43.9	379	5.37E-104
1388	OSC66498.1	-	alkyl/aryl-sulfatase	0	1	0	0	0	0	0	0	0	0	0	1	Unique	COG2015_Q#	53.1	560	6.36E-159	NA	53.1	560	1.7E-158
1389	Prokka:JAPCLKMA_05339	ddpA_2	putative D,D-dipeptide-binding periplasmic protein DdpA	1	0	0	0	1	1	1	0	0	0	1	5	Accessory	COG0747_E#	34.5	262	3.58E-69	K02035	32	235	1.63E-60
1390	OSC62577.1	-	ABC transporter substrate-binding protein	0	1	0	0	0	0	0	0	0	0	0	1	Unique	COG0747_E#	29.1	201	7.49E-51	K13889	24	126	1.07E-27
1391	OSC63495.1	-	alkaline phosphatase	1	1	1	1	1	1	1	1	1	1	1	11	CORE	COG3540_P#	57.6	553	7.77E-157				
1392	OSC61291.1	-	hypothetical protein	0	1	0	0	0	0	0	0	0	0	0	1	Unique								
1393	WP_102637211.1	-	M1 family metallopeptidase	1	1	1	1	1	1	1	1	1	1	1	11	CORE	COG0308_E#	31.7	193	2.04E-48	NA	31.7	193	5.45E-48
1394	WP_048454754.1	-	mucoidy inhibitor MuiA family protein	1	1	1	1	1	1	1	1	1	1	1	11	CORE								

cluster	GI	gene	Annotation	Streptomyces_thermocarboxydus_BPSAC147	Streptomyces_sp._4F	Streptomyces_sp._BSE7F	Streptomyces_sp._ETH9427	Streptomyces_sp._FxanaD5	Streptomyces_sp._UNC401CLCol	Actinospica_acidiphila_NRRL_B_24431	Streptomyces_sp._HNS054	Streptomyces_sp._SMS_SU21	Streptomyces_sp._ZS0098	Streptomyces_thermocarboxydus_K155	Addition	Core-Accessory-Unique	COG	%Similarity_COG	Score_COG	Evalue_COG	KEGG	%Similarity_KEGG	Score_KEGG	Evalue_KEGG
1395	WP_020699964.1	-	alpha/beta hydrolase	1	1	1	1	1	1	1	1	1	1	1	11	CORE	COG0596_R#	32.5	250	1.41E-65	NA	32.5	250	3.76E-65
1396	Prokka:JAPCLKMA_05487	ilvG_2	Acetolactate synthase isozyme 2 large subunit	1	0	0	0	0	0	0	0	0	0	0	1	Unique	COG0028_EH#	28	154	8.04E-37	K01652	35.8	87	5.5E-16
1397	WP_033272728.1	-	hypothetical protein	0	0	1	1	1	1	1	1	1	1	1	9	Accessory					NA	32.5	50	0.0000745
1398	OSC55737.1	-	hypothetical protein	0	1	0	0	0	0	0	0	0	0	0	1	Unique	COG1020_Q#	32.3	202	4.39E-51	K15665	23.5	136	6.05E-31
1399	Prokka:CIODPCDJ_04144	mdtD_7	Putative multidrug resistance protein MdtD	1	1	0	0	0	1	1	0	0	0	1	5	Accessory	COG0477_GEPR#	30.1	160	1.91E-38	NA	25.2	130	4.33E-29
1400	OSC70446.1	-	MFS transporter	1	1	1	0	1	1	1	0	1	1	1	9	Accessory	COG0477_GEPR#	42.1	385	2.8E-106	NA	30.1	211	2.52E-53
1401	WP_107081035.1	-	hypothetical protein	1	0	0	0	1	1	0	1	1	0	1	6	Accessory	COG1404_O#	28.1	47	0.000236	K06236	29.1	104	3.32E-21
1402	OSC65695.1	-	RNA-binding protein	0	1	0	0	0	0	0	0	0	0	0	1	Unique					NA	30.3	85	1.6E-15
1403	OSC72249.1	-	Na+:solute symporter	1	1	1	1	1	1	1	1	1	1	1	11	CORE	COG0591_ER#	24.9	119	3.74E-26	K03307	26.1	138	2.71E-31
1404	PWE08011.1	-	4-coumarate--CoA ligase family protein	1	1	1	1	1	1	1	1	1	1	1	11	CORE	COG0318_IQ#	37.6	312	2.3E-84	K01904	42.1	385	9.76E-106
1405	WP_053076979.1	-	DDE-type integrase/transposase/recombinase	0	0	0	0	0	0	0	1	0	0	0	1	Unique								

cluster	GI	gene	Annotation	Streptomyces_thermocarboxydus_BPSAC147	Streptomyces_sp._4F	Streptomyces_sp._BSE7F	Streptomyces_sp._ETH9427	Streptomyces_sp._FxanaD5	Streptomyces_sp._UNC401CLCol	Actinospica_acidiphila_NRRL_B_24431	Streptomyces_sp._HNS054	Streptomyces_sp._SMS_SU21	Streptomyces_sp._ZS0098	Streptomyces_thermocarboxydus_K155	Addition	Core-Accessory-Unique	COG	%Similarity_COG	Score_COG	Evalue_COG	KEGG	%Similarity_KEGG	Score_KEGG	Evalue_KEGG
1406	WP_102641825.1	guaA	glutamine-hydrolyzing GMP synthase	1	1	1	1	1	1	1	1	1	1	1	11	CORE	COG0519_F#	77.9	508	3.73E-143	K01951	70.7	736	2.25E-211
1407	OSC69885.1	-	aldehyde dehydrogenase	1	1	1	1	1	1	1	1	1	1	1	11	CORE	COG1012_C#	29.5	153	3.05E-36	K13821	28.6	137	4.62E-31
1408	OSC61822.1	-	benzoylformate decarboxylase	0	1	0	0	0	0	0	0	0	0	0	1	Unique	COG0028_EH#	58	596	1.36E-169	K01652	32.1	176	6.87E-43
1409	WP_019527713.1	dndC	DNA phosphorothioation system sulfurtransferase DndC	0	0	0	0	1	0	0	0	0	0	0	1	Unique	COG0175_EH#	54	552	1.32E-156				
1410	WP_033276881.1	-	CYTH and CHAD domain-containing protein	1	0	1	1	1	1	1	1	1	1	1	10	Accessory	COG5607_S#	25.4	55	8.63E-07	NA	36.8	263	4.26E-69
1411	WP_048460260.1	-	YibE/F family protein	1	1	1	1	1	1	1	1	1	1	1	11	CORE	COG5438_S#	33.6	207	1.04E-52	NA	28	149	8.98E-35
1412	WP_122213885.1	-	right-handed parallel beta-helix repeat-containing protein	1	0	1	1	1	1	1	1	1	1	1	10	Accessory								
1413	WP_159020907.1	-	tetratricopeptide repeat protein	0	0	0	0	0	0	0	1	0	0	0	1	Unique								
1414	WP_048456849.1	-	hypothetical protein	1	1	1	1	1	1	1	1	1	1	1	11	CORE								

cluster	GI	gene	Annotation	Streptomyces_thermocarboxydus_BPSAC147	Streptomyces_sp._4F	Streptomyces_sp._BSE7F	Streptomyces_sp._ETH9427	Streptomyces_sp._FxanaD5	Streptomyces_sp._UNC401CLCol	Actinospica_acidiphila_NRRL_B_24431	Streptomyces_sp._HNS054	Streptomyces_sp._SMS_SU21	Streptomyces_sp._ZS0098	Streptomyces_thermocarboxydus_K155	Addition	Core-Accessory-Unique	COG	%Similarity_COG	Score_COG	Evalue_COG	KEGG	%Similarity_KEGG	Score_KEGG	Evalue_KEGG
1415	Prokka:CIODPCDJ_00840	afsR_2	Regulatory protein AfsR	1	0	0	0	1	1	0	0	0	0	1	4	Accessory	COG3629_T#	34.1	124	1.16E-27	NA	32.1	124	2.37E-27
1416	WP_157842178.1	-	sigma-70 family RNA polymerase sigma factor	0	0	0	0	0	0	1	0	0	0	0	1	Unique	COG0515_RTKL#	31	42	0.00443	NA	40.4	44	0.00407
1417	WP_037778386.1	-	transporter	1	0	1	1	1	1	1	1	1	1	1	10	Accessory	COG4147_R#	27	197	1.41E-49	K14393	29.2	186	6.63E-46
1418	Prokka:CIODPCDJ_02605	-	hypothetical protein	1	1	1	1	1	1	1	1	1	1	1	11	CORE								
1419	WP_084206995.1	-	DUF4157 domain-containing protein	0	0	0	0	0	0	1	0	0	0	0	1	Unique								
1420	WP_028960059.1	-	NADH-quinone oxidoreductase subunit M	1	1	1	1	1	1	1	1	1	1	1	11	CORE	COG1008_C#	39.2	320	1.1E-86	K00342	35.8	307	1.97E-82
1421	Prokka:CIODPCDJ_00293	glpD1	Glycerol-3-phosphate dehydrogenase 1	1	1	1	1	1	1	1	1	1	1	1	11	CORE	COG0578_C#	55.1	520	5.54E-147	K00111	55.1	520	1.48E-146
1422	WP_028959738.1	-	S8 family peptidase	1	1	1	1	1	1	1	1	1	1	1	11	CORE	COG1404_O#	39.6	328	4.03E-89	K01336	40.3	245	9.18E-64
1423	WP_122213955.1	-	MFS transporter	0	0	0	0	0	0	0	0	0	1	0	1	Unique	COG0477_GEPR#	33.2	248	6.93E-65	NA	33.2	245	9.18E-64
1424	WP_033276280.1	-	NAD(P)-binding domain-containing protein	0	0	0	0	0	0	1	0	0	0	0	1	Unique	COG0644_C#	28.3	51	0.00000952	NA	41.6	395	9.37E-109
1425	OSC71437.1	-	peptidase S8	1	1	1	1	1	1	1	1	1	1	1	11	CORE	COG1404_O#	29.8	117	1.08E-25	K01342	30.1	119	1.3E-25

cluster	GI	gene	Annotation	Streptomyces_thermocarboxydus_BPSAC147	Streptomyces_sp._4F	Streptomyces_sp._BSE7F	Streptomyces_sp._ETH9427	Streptomyces_sp._FxanaD5	Streptomyces_sp._UNC401CLCol	Actinospica_acidiphila_NRRL_B_24431	Streptomyces_sp._HNS054	Streptomyces_sp._SMS_SU21	Streptomyces_sp._ZS0098	Streptomyces_thermocarboxydus_K155	Addition	Core-Accessory-Unique	COG	%Similarity_COG	Score_COG	Evalue_COG	KEGG	%Similarity_KEGG	Score_KEGG	Evalue_KEGG
1426	OSC73225.1	-	silent information regulator protein Sir2	0	1	0	0	0	0	0	0	0	0	0	1	Unique					NA	30.7	110	6.02E-23
1427	Prokka:JAPCLKMA_04873	hutH	Histidine ammonia-lyase	1	1	0	1	1	1	1	1	1	1	1	10	Accessory	COG2986_E#	44.7	384	4.74E-106	K01745	44.7	384	1.27E-105
1428	PWE08856.1	-	NADH-quinone oxidoreductase subunit M	1	1	1	1	1	1	1	1	1	1	1	11	CORE	COG1008_C#	56	575	2.47E-163	K00342	56	575	6.6E-163
1429	WP_033277794.1	-	NAD(P)-binding protein	1	1	1	1	1	1	1	1	1	1	1	11	CORE	COG2303_E#	35	279	2.8E-74	NA	36.5	272	1.19E-71
1430	OSC70506.1	-	apolipoprotein N-acyltransferase	1	1	1	0	1	1	1	1	1	1	1	10	Accessory	COG0815_M#	46.1	399	1.42E-110	NA	49.2	280	2.57E-74
1431	OSC70570.1	-	hypothetical protein	0	1	0	0	0	0	0	0	0	0	0	1	Unique	COG0641_R#	23.8	76	3.6E-13	K06871	22.4	60	5.46E-08
1432	OSC70048.1	-	bifunctional phosphoribosylaminoimidazolecarboxamide formyltransferase/IMP cyclohydrolase	1	1	1	1	1	1	1	1	1	1	1	11	CORE	COG0138_F#	69.1	701	1.75E-201	K00602	67.3	691	8.25E-198

cluster	GI	gene	Annotation	Streptomyces_thermocarboxydus_BPSAC147	Streptomyces_sp._4F	Streptomyces_sp._BSE7F	Streptomyces_sp._ETH9427	Streptomyces_sp._FxanaD5	Streptomyces_sp._UNC401CLCol	Actinospica_acidiphila_NRRL_B_24431	Streptomyces_sp._HNS054	Streptomyces_sp._SMS_SU21	Streptomyces_sp._ZS0098	Streptomyces_thermocarboxydus_K155	Addition	Core-Accessory-Unique	COG	%Similarity_COG	Score_COG	Evalue_COG	KEGG	%Similarity_KEGG	Score_KEGG	Evalue_KEGG
1433	WP_102638544.1	-	helix-turn-helix transcriptional regulator	1	0	0	0	1	1	0	0	1	0	1	5	Accessory								
1434	OSC72945.1	-	pectate lyase	1	1	1	1	1	1	1	1	1	1	1	11	CORE	COG3866_G#	37.7	228	5.66E-59	NA	31.6	121	2.6E-26
1435	Prokka:CIODPCDJ_00866	sasA_3	Adaptive-response sensory-kinase SasA	0	0	0	0	1	1	0	0	0	0	1	3	Accessory	COG0642_T#	40.7	284	8.69E-76	K02484	40	281	1.5E-74
1436	WP_157842161.1	-	acyltransferase domain-containing protein	0	0	0	0	0	0	1	0	0	0	0	1	Unique	COG3319_Q#	31.4	139	3.46E-32	K12437	31.3	142	1.09E-32
1437	WP_146217814.1	-	recombinase family protein	0	0	0	0	0	0	0	0	1	0	0	1	Unique	COG1961_L#	25.5	100	1.78E-20	NA	23.8	72	1.06E-11
1438	WP_028961807.1	-	hypothetical protein	1	0	0	0	1	1	0	0	0	0	1	4	Accessory					NA	29.4	64	3.78E-09
1439	Prokka:CIODPCDJ_00323	carA2	zeta-carotene-forming phytoene desaturase	1	1	1	1	1	1	1	1	1	1	1	11	CORE	COG1233_Q#	29.6	219	2.62E-56	K10027	29.3	187	3.85E-46
1440	WP_051724432.1	-	TrkH family potassium uptake protein	0	0	0	0	0	0	1	0	0	0	0	1	Unique	COG0168_P#	35.9	315	2.69E-85	NA	35.4	285	7.96E-76
1441	OSC57469.1	-	transporter	0	1	0	0	0	0	0	0	0	0	0	1	Unique	COG0596_R#	29.2	165	5.87E-40	NA	33.1	151	1.79E-35

cluster	GI	gene	Annotation	Streptomyces_thermocarboxydus_BPSAC147	Streptomyces_sp._4F	Streptomyces_sp._BSE7F	Streptomyces_sp._ETH9427	Streptomyces_sp._FxanaD5	Streptomyces_sp._UNC401CLCol	Actinospica_acidiphila_NRRL_B_24431	Streptomyces_sp._HNS054	Streptomyces_sp._SMS_SU21	Streptomyces_sp._ZS0098	Streptomyces_thermocarboxydus_K155	Addition	Core-Accessory-Unique	COG	%Similarity_COG	Score_COG	Evalue_COG	KEGG	%Similarity_KEGG	Score_KEGG	Evalue_KEGG
1442	WP_019525638.1	-	FAD-dependent oxidoreductase	1	1	0	1	1	1	1	1	1	1	1	10	Accessory	COG3349_S#	35	116	4.09E-25	K09879	31.3	131	2.51E-29
1443	WP_102641765.1	-	MFS transporter	0	0	1	0	0	0	0	0	1	0	0	2	Accessory	COG0477_GEPR#	37.5	365	3.87E-100	NA	36	317	1.89E-85
1444	WP_048455328.1	-	PucR family transcriptional regulator	1	1	1	1	1	1	1	1	1	1	1	11	CORE	COG2508_TQ#	26.7	119	3.69E-26	NA	20.7	53	0.00000867
1445	WP_019524605.1	-	tripartite tricarboxylate transporter TctA	1	1	1	0	1	1	0	1	1	1	1	9	Accessory	COG3333_S#	58.9	579	1.3E-164				
1446	OSC69446.1	-	MFS transporter	0	1	0	0	0	0	0	0	0	0	0	1	Unique	COG0477_GEPR#	38.6	273	1.17E-72	NA	31.9	266	6.52E-70
1447	WP_159020891.1	-	hypothetical protein	0	0	0	0	0	0	0	1	0	0	0	1	Unique								
1448	OSC63325.1	-	phosphate-binding protein	0	1	0	0	0	0	0	0	0	0	0	1	Unique	COG0226_P#	28.2	119	3.69E-26	K02040	28.2	119	9.85E-26
1449	WP_122213816.1	-	terminase	0	0	0	0	0	0	0	0	0	1	0	1	Unique								
1450	WP_019526881.1	-	arabinogalactan endo-1,4-beta-galactosidase	1	0	0	0	1	1	0	0	0	0	1	4	Accessory	COG3867_G#	45.5	317	9.21E-86	K01224	39.2	222	8.25E-57
1451	WP_122213255.1	-	sugar ABC transporter ATP-binding protein	1	0	1	1	1	1	1	1	1	1	1	10	Accessory	COG1129_G#	50.3	465	3.57E-130	K02056	46.5	431	1.53E-119

cluster	GI	gene	Annotation	Streptomyces_thermocarboxydus_BPSAC147	Streptomyces_sp._4F	Streptomyces_sp._BSE7F	Streptomyces_sp._ETH9427	Streptomyces_sp._FxanaD5	Streptomyces_sp._UNC401CLCol	Actinospica_acidiphila_NRRL_B_24431	Streptomyces_sp._HNS054	Streptomyces_sp._SMS_SU21	Streptomyces_sp._ZS0098	Streptomyces_thermocarboxydus_K155	Addition	Core-Accessory-Unique	COG	%Similarity_COG	Score_COG	Evalue_COG	KEGG	%Similarity_KEGG	Score_KEGG	Evalue_KEGG
1452	WP_048456104.1	-	membrane-bound polysaccharide biosynthesis protein	1	0	1	1	1	1	1	1	1	1	1	10	Accessory								
1453	Prokka:JAPCLKMA_05504	-	hypothetical protein	1	0	1	1	1	1	1	1	1	1	1	10	Accessory								
1454	PWE10319.1	-	hypothetical protein	0	0	1	1	0	0	0	1	1	1	0	5	Accessory								
1455	OSC73212.1	-	hypothetical protein	0	1	0	0	0	0	0	0	0	0	0	1	Unique	COG2304_R#	38.5	319	1.86E-86	NA	22.8	54	0.00000297
1456	WP_048459122.1	-	hypothetical protein	1	1	1	0	0	0	1	1	1	1	1	8	Accessory	COG0457_R#	27.3	52	0.00000553	NA	41.8	44	0.00308
1457	AXI85297.1	-	hypothetical protein	0	0	0	1	0	0	0	0	0	0	0	1	Unique								
1458	WP_051724691.1	-	sensor histidine kinase	0	1	0	0	0	0	1	0	0	0	0	2	Accessory	COG4585_T#	32.5	89	4.08E-17	NA	29.1	71	3.07E-11
1459	OSC61954.1	-	amino acid transporter	0	1	0	0	0	0	0	0	0	0	0	1	Unique	COG0531_E#	30.7	208	6.03E-53	K03294	27.7	97	5.23E-19
1460	WP_122214233.1	-	PH domain-containing protein	1	0	0	1	1	0	1	1	0	1	1	7	Accessory								
1461	WP_028960739.1	-	TldD/PmbA family protein	1	1	1	1	1	1	1	1	1	1	1	11	CORE	COG0312_R#	63.1	641	2.13E-183	K03568	63.1	641	5.68E-183
1462	WP_028960734.1	-	sodium:solute symporter	1	0	1	1	1	1	1	1	1	1	1	10	Accessory	COG0591_ER#	40	317	1.2E-85	K03307	30.4	208	1.61E-52
1463	AXI89984.1	-	hypothetical protein	0	0	0	1	0	0	0	1	1	1	0	4	Accessory	COG3125_C#	54.8	47	0.000232	NA	30.7	63	6.39E-09
1464	Prokka:CIODPCDJ_05357	-	Carboxylesterase	1	0	1	1	1	1	1	1	1	1	1	10	Accessory	COG2272_I#	35.6	255	3.29E-67	K03929	35.8	255	8.77E-67

cluster	GI	gene	Annotation	Streptomyces_thermocarboxydus_BPSAC147	Streptomyces_sp._4F	Streptomyces_sp._BSE7F	Streptomyces_sp._ETH9427	Streptomyces_sp._FxanaD5	Streptomyces_sp._UNC401CLCol	Actinospica_acidiphila_NRRL_B_24431	Streptomyces_sp._HNS054	Streptomyces_sp._SMS_SU21	Streptomyces_sp._ZS0098	Streptomyces_thermocarboxydus_K155	Addition	Core-Accessory-Unique	COG	%Similarity_COG	Score_COG	Evalue_COG	KEGG	%Similarity_KEGG	Score_KEGG	Evalue_KEGG
1465	Prokka:CIODPCDJ_02790	efpA_2	putative MFS-type transporter EfpA	1	1	1	1	1	1	1	1	1	1	1	11	CORE	COG0477_GEPR#	37.8	273	1.52E-72	NA	33.2	264	2.47E-69
1466	WP_051724386.1	-	HAMP domain-containing histidine kinase	1	1	1	1	1	1	1	1	1	1	1	11	CORE	COG0642_T#	50.5	421	3.46E-117	K02484	50.5	397	1.87E-109
1467	OSC61654.1	-	oxidoreductase	0	1	0	0	0	0	0	0	0	0	0	1	Unique								
1468	WP_019527043.1	-	MFS transporter	1	1	1	1	1	1	1	1	1	1	1	11	CORE	COG0477_GEPR#	34.9	265	3.17E-70	NA	32.9	250	4.8E-65
1469	Prokka:CIODPCDJ_00248	efpA_1	putative MFS-type transporter EfpA	1	1	1	1	1	1	1	1	1	1	1	11	CORE	COG0477_GEPR#	40	305	2.76E-82	NA	38.1	305	7.38E-82
1470	WP_051724442.1	-	ABC transporter substrate-binding protein	0	1	0	0	0	0	1	0	0	0	0	2	Accessory	COG0747_E#	47.1	462	1.77E-129	K12368	27.8	152	1.36E-35
1471	WP_048456188.1	-	TIGR02677 family protein	0	0	0	1	0	0	0	1	0	0	0	2	Accessory								
1472	OSC72199.1	-	hypothetical protein	0	1	0	0	0	0	0	0	0	0	0	1	Unique	COG0112_E#	32	163	2.21E-39	K00600	31.7	146	5.73E-34
1473	WP_102639835.1	-	ATP-binding protein	0	0	0	0	0	0	0	0	1	0	0	1	Unique					NA	32.4	50	0.0000559
1474	Prokka:CIODPCDJ_04829	ffh	Signal recognition particle protein	1	1	1	1	1	1	1	1	1	1	1	11	CORE	COG0541_U#	63.7	604	3.75E-172	K03106	63.7	604	1E-171

cluster	GI	gene	Annotation	Streptomyces_thermocarboxydus_BPSAC147	Streptomyces_sp._4F	Streptomyces_sp._BSE7F	Streptomyces_sp._ETH9427	Streptomyces_sp._FxanaD5	Streptomyces_sp._UNC401CLCol	Actinospica_acidiphila_NRRL_B_24431	Streptomyces_sp._HNS054	Streptomyces_sp._SMS_SU21	Streptomyces_sp._ZS0098	Streptomyces_thermocarboxydus_K155	Addition	Core-Accessory-Unique	COG	%Similarity_COG	Score_COG	Evalue_COG	KEGG	%Similarity_KEGG	Score_KEGG	Evalue_KEGG
1475	Prokka:JAPCLKMA_04366	xylG	Xylose import ATP-binding protein XylG	1	1	1	1	1	1	1	1	1	1	1	11	CORE	COG1129_G#	67.6	680	4.12E-195	K10545	46.3	444	1.33E-123
1476	OSC71023.1	-	hypothetical protein	0	1	0	0	0	0	0	0	0	0	0	1	Unique								
1477	AXI90286.1	-	VWA domain-containing protein	1	0	1	1	1	1	1	1	1	1	1	10	Accessory	COG2304_R#	37.7	233	1.74E-60	K07114	39.7	235	1.6E-60
1478	WP_028961284.1	-	hypothetical protein	0	0	0	0	0	1	0	0	0	0	0	1	Unique								
1479	Prokka:JAPCLKMA_05581	dhbF_1	Dimodular nonribosomal peptide synthase	1	0	0	0	0	0	0	0	0	0	0	1	Unique	COG1020_Q#	44.2	355	3.96E-97	K04780	41.1	349	5.81E-95
1480	WP_037954569.1	-	iron transporter	1	1	1	1	1	1	1	1	1	1	1	11	CORE	COG4264_Q#	34.6	169	4.02E-41				
1481	WP_122214246.1	-	glycosyltransferase	1	1	1	1	1	1	1	1	1	1	1	11	CORE	COG0463_M#	43.1	161	6.42E-39	K00729	27.9	79	1.12E-13
1482	WP_033277657.1	-	aldehyde dehydrogenase family protein	1	1	0	0	1	1	1	0	0	0	1	6	Accessory	COG1012_C#	47.7	443	1.44E-123	K00128	32.9	277	3.66E-73
1483	OSC72220.1	-	NADH-quinone oxidoreductase subunit N	1	1	1	1	1	1	1	1	1	1	1	11	CORE	COG1007_C#	32.5	251	8.05E-66	K05573	32.6	245	1.54E-63
1484	WP_033272376.1	-	protein kinase	0	1	0	0	0	0	1	0	0	0	0	2	Accessory	COG0515_RTKL#	29.3	99	3.91E-20	NA	29.2	96	6.77E-19

cluster	GI	gene	Annotation	Streptomyces_thermocarboxydus_BPSAC147	Streptomyces_sp._4F	Streptomyces_sp._BSE7F	Streptomyces_sp._ETH9427	Streptomyces_sp._FxanaD5	Streptomyces_sp._UNC401CLCol	Actinospica_acidiphila_NRRL_B_24431	Streptomyces_sp._HNS054	Streptomyces_sp._SMS_SU21	Streptomyces_sp._ZS0098	Streptomyces_thermocarboxydus_K155	Addition	Core-Accessory-Unique	COG	%Similarity_COG	Score_COG	Evalue_COG	KEGG	%Similarity_KEGG	Score_KEGG	Evalue_KEGG
1485	WP_102640451.1	-	NAD(P)/FAD-dependent oxidoreductase	1	1	0	1	1	1	1	1	1	1	1	10	Accessory	COG2072_P#	48.2	475	2.62E-133	NA	45.6	456	2.58E-127
1486	Prokka:JAPCLKMA_06288	sasA_14	Adaptive-response sensory-kinase SasA	1	0	1	1	1	1	1	1	1	1	1	10	Accessory	COG0642_T#	31.6	200	1.63E-50	K07654	31.4	199	7.42E-50
1487	PWE10557.1	-	D-alanine--poly(phosphoribitol) ligase	0	0	1	0	0	0	0	0	1	0	0	2	Accessory	COG1020_Q#	35.9	222	3.99E-57	K15666	33.3	201	2.54E-50
1488	Prokka:CIODPCDJ_04928	eccB2	ESX-2 secretion system ATPase EccB2	1	0	1	0	1	1	1	0	1	0	1	7	Accessory								
1489	OSC73546.1	-	RNA polymerase sigma factor	1	1	1	1	1	1	1	1	1	1	1	11	CORE	COG0568_K#	63.3	528	3.4E-149	K03086	56.7	508	7.45E-143
1490	OSC73274.1	-	hypothetical protein	0	1	0	0	0	0	0	0	0	0	0	1	Unique	COG3629_T#	35	144	1.38E-33	NA	35	144	3.69E-33
1491	WP_033273854.1	-	alpha/beta fold hydrolase	0	1	0	1	0	0	1	1	1	1	0	6	Accessory								
1492	WP_033278108.1	-	FAD-binding oxidoreductase	1	0	0	1	1	1	1	1	1	1	1	9	Accessory	COG0277_C#	32.2	237	1.2E-61	NA	32.8	245	1.18E-63
1493	WP_028961856.1	-	APC family permease	1	1	0	1	1	1	1	1	1	1	1	10	Accessory	COG0531_E#	36.3	322	2.17E-87				

cluster	GI	gene	Annotation	Streptomyces_thermocarboxydus_BPSAC147	Streptomyces_sp._4F	Streptomyces_sp._BSE7F	Streptomyces_sp._ETH9427	Streptomyces_sp._FxanaD5	Streptomyces_sp._UNC401CLCol	Actinospica_acidiphila_NRRL_B_24431	Streptomyces_sp._HNS054	Streptomyces_sp._SMS_SU21	Streptomyces_sp._ZS0098	Streptomyces_thermocarboxydus_K155	Addition	Core-Accessory-Unique	COG	%Similarity_COG	Score_COG	Evalue_COG	KEGG	%Similarity_KEGG	Score_KEGG	Evalue_KEGG
1494	Prokka:CIODPCDJ_05967	ald_3	4,4'-diapolycopene aldehyde oxidase	1	0	0	0	1	1	1	0	0	0	1	5	Accessory	COG1012_C#	35.1	258	6.56E-68	NA	36.4	268	9.92E-71
1495	OSC71587.1	-	spermidine synthase	1	1	1	1	1	1	1	1	1	1	1	11	CORE	COG0421_E#	32.6	126	2.98E-28	NA	24.4	62	1.41E-08
1496	WP_019527537.1	-	sugar ABC transporter ATP-binding protein	1	1	1	1	1	1	1	1	1	1	1	11	CORE	COG1129_G#	58.3	570	5.97E-162	K10441	46.2	448	1.19E-124
1497	OSC57691.1	-	(2Fe-2S)-binding protein	1	1	1	1	1	1	1	1	1	1	1	11	CORE	COG0665_E#	39.5	270	1.28E-71	NA	34.4	326	4.01E-88
1498	OSC67419.1	-	peptidase	1	1	1	1	1	1	1	1	1	1	1	11	CORE	COG0596_R#	34.9	288	4.53E-77	NA	34.9	288	1.21E-76
1499	OSC66714.1	-	MFS transporter	1	1	1	1	1	1	1	1	1	1	1	11	CORE	COG0477_GEPR#	34	275	3.97E-73	NA	33.2	233	3.54E-60
1500	WP_033278129.1	-	serine/threonine protein kinase	1	1	1	1	1	1	1	1	1	1	1	11	CORE	COG0515_RTKL#	36.6	205	6.57E-52	K08884	36.6	205	1.76E-51
1501	WP_019522036.1	-	leucyl aminopeptidase	1	1	1	1	1	1	1	1	1	1	1	11	CORE	COG0260_E#	45.2	388	4.19E-107	K01255	45.2	388	1.12E-106
1502	WP_051724705.1	-	MFS transporter	0	0	0	0	0	0	1	0	0	0	0	1	Unique	COG0477_GEPR#	34.6	217	1.28E-55	K07001	34.3	191	2.62E-47
1503	AXI85502.1	-	ATP-dependent DNA ligase	1	1	1	1	1	1	1	1	1	1	1	11	CORE	COG1793_L#	55.5	508	2.13E-143	K10747	55.3	507	2.16E-142
1504	WP_048457052.1	-	hypothetical protein	0	1	1	0	0	0	1	1	1	0	0	5	Accessory					NA	29.6	60	6.98E-08

cluster	GI	gene	Annotation	Streptomyces_thermocarboxydus_BPSAC147	Streptomyces_sp._4F	Streptomyces_sp._BSE7F	Streptomyces_sp._ETH9427	Streptomyces_sp._FxanaD5	Streptomyces_sp._UNC401CLCol	Actinospica_acidiphila_NRRL_B_24431	Streptomyces_sp._HNS054	Streptomyces_sp._SMS_SU21	Streptomyces_sp._ZS0098	Streptomyces_thermocarboxydus_K155	Addition	Core-Accessory-Unique	COG	%Similarity_COG	Score_COG	Evalue_COG	KEGG	%Similarity_KEGG	Score_KEGG	Evalue_KEGG
1505	WP_048460550.1	-	alanine:cation symporter family protein	0	1	1	1	0	0	1	1	1	1	0	7	Accessory	COG1115_E#	51	471	4.92E-132	K03310	37.3	288	1.21E-76
1506	OSC63074.1	-	glycerol kinase	1	1	1	1	1	1	1	1	1	1	1	11	CORE	COG0554_C#	58.3	605	2.18E-172	K00864	58.3	605	5.83E-172
1507	AXI86646.1	-	hypothetical protein	0	0	0	1	0	0	0	1	0	0	0	2	Accessory	COG1961_L#	27.3	101	1.34E-20	NA	27.3	101	3.57E-20
1508	Prokka:JAPCLKMA_06824	-	hypothetical protein	1	0	0	0	0	0	0	0	0	0	0	1	Unique	COG3182_S#	38.7	280	1.61E-74				
1509	OSC72628.1	-	gamma-aminobutyraldehyde dehydrogenase	1	1	1	1	1	1	1	1	1	1	1	11	CORE	COG1012_C#	49.8	461	2.98E-129	K00137	49.8	461	7.95E-129
1510	PWE09941.1	-	glycoside hydrolase family 43 protein	1	0	1	1	1	1	1	1	1	1	1	10	Accessory	COG3507_G#	36.9	304	7.95E-82	K01198	36.3	242	9.9E-63
1511	WP_122216756.1	-	cobyric acid synthase	1	1	0	0	1	1	1	1	1	1	1	9	Accessory	COG1492_H#	54.5	483	9.56E-136	K02232	54.5	483	2.55E-135
1512	WP_122214650.1	-	LCP family protein	1	1	0	1	1	1	1	1	1	1	1	10	Accessory	COG1316_K#	28.5	170	1.37E-41	NA	29.3	166	5.29E-40
1513	OSC70574.1	-	DNA primase	0	1	0	0	0	0	0	0	0	0	0	1	Unique	COG3378_R#	36.6	235	5.92E-61	K06919	33.9	215	1.69E-54
1514	OSC66512.1	-	amino acid permease	0	1	0	0	0	0	0	0	0	0	0	1	Unique	COG0531_E#	32.4	282	3.23E-75	NA	44.1	408	7.99E-113
1515	WP_102640299.1	-	PTS transporter subunit EIIC	1	1	0	1	1	1	1	1	1	1	1	10	Accessory	COG1263_G#	48.3	322	2.16E-87	K02810	44.1	388	1.12E-106
1516	PWE10127.1	-	Na+/H+ antiporter subunit D	0	0	1	1	0	0	0	1	1	1	0	5	Accessory	COG0651_CP#	46.5	444	4.91E-124	K05568	36	307	1.92E-82
1517	WP_146217788.1	-	hypothetical protein	0	0	1	1	0	0	0	0	1	1	0	4	Accessory	COG0737_F#	29.2	44	0.00193	NA	26	77	3.22E-13

cluster	GI	gene	Annotation	Streptomyces_thermocarboxydus_BPSAC147	Streptomyces_sp._4F	Streptomyces_sp._BSE7F	Streptomyces_sp._ETH9427	Streptomyces_sp._FxanaD5	Streptomyces_sp._UNC401CLCol	Actinospica_acidiphila_NRRL_B_24431	Streptomyces_sp._HNS054	Streptomyces_sp._SMS_SU21	Streptomyces_sp._ZS0098	Streptomyces_thermocarboxydus_K155	Addition	Core-Accessory-Unique	COG	%Similarity_COG	Score_COG	Evalue_COG	KEGG	%Similarity_KEGG	Score_KEGG	Evalue_KEGG
1518	OSC65129.1	-	MFS transporter	1	1	1	1	1	1	1	1	1	1	1	11	CORE	COG0477_GEPR#	43.3	367	7.63E-101	NA	27.8	197	2.79E-49
1519	WP_147474393.1	-	tetratricopeptide repeat protein	0	0	0	0	0	0	0	0	0	1	0	1	Unique	COG0457_R#	28.7	76	3.51E-13	NA	24.1	67	5.69E-10
1520	OSC71986.1	-	hypothetical protein	0	1	0	0	0	0	0	0	0	0	0	1	Unique	COG4796_U#	31.9	54	0.00000143	NA	27	81	2.92E-14
1521	WP_102639270.1	-	DUF4331 domain-containing protein	1	1	1	1	1	1	1	1	1	1	1	11	CORE								
1522	AXI84684.1	-	thioester reductase	0	0	0	1	0	0	0	1	0	0	0	2	Accessory	COG1020_Q#	35.9	279	2.09E-74	K04780	35.9	279	5.59E-74
1523	OSC71191.1	-	two-component sensor histidine kinase	0	1	0	0	0	0	0	0	0	0	0	1	Unique	COG0642_T#	40	312	3.8E-84	K02484	39.8	309	6.58E-83
1524	OSC68487.1	-	carboxylesterase	0	1	0	0	0	0	0	0	0	0	0	1	Unique	COG2272_I#	39.8	298	5.68E-80	K03929	39.8	298	1.52E-79
1525	Prokka:JAPCLKMA_01529	-	hypothetical protein	1	0	0	1	0	0	0	0	0	0	0	2	Accessory								
1526	OSC71064.1	-	flotillin	0	1	0	0	0	0	0	0	0	0	0	1	Unique	COG2268_S#	36.1	275	3.01E-73	K07192	36.5	274	2.34E-72
1527	PWE06918.1	-	aldehyde dehydrogenase (NADP(+))	1	1	1	1	1	1	1	1	1	1	1	11	CORE	COG1012_C#	46	385	3.53E-106				
1528	Prokka:JAPCLKMA_03621	prmC_1	Release factor glutamine methyltransferase	1	1	1	1	1	1	1	1	1	1	1	11	CORE	COG2890_J#	34.1	232	3.82E-60				

cluster	GI	gene	Annotation	Streptomyces_thermocarboxydus_BPSAC147	Streptomyces_sp._4F	Streptomyces_sp._BSE7F	Streptomyces_sp._ETH9427	Streptomyces_sp._FxanaD5	Streptomyces_sp._UNC401CLCol	Actinospica_acidiphila_NRRL_B_24431	Streptomyces_sp._HNS054	Streptomyces_sp._SMS_SU21	Streptomyces_sp._ZS0098	Streptomyces_thermocarboxydus_K155	Addition	Core-Accessory-Unique	COG	%Similarity_COG	Score_COG	Evalue_COG	KEGG	%Similarity_KEGG	Score_KEGG	Evalue_KEGG
1529	WP_019523500.1	-	UDP-N-acetylglucosamine 1-carboxyvinyltransferase	1	1	1	1	1	1	1	1	1	1	1	11	CORE	COG0766_M#	33.2	214	8.25E-55	K00790	29.5	206	4.59E-52
1530	PWE08589.1	-	LacI family transcriptional regulator	0	0	1	0	0	0	0	0	0	0	1	2	Accessory	COG1609_K#	48.4	54	0.00000143	NA	42.9	45	0.00135
1531	WP_048458582.1	-	ATP-dependent helicase	0	0	0	0	0	0	0	1	0	0	0	1	Unique	COG0210_L#	42.3	57	1.69E-07				
1532	Prokka:JAPCLKMA_03176	fprB	putative ferredoxin/ferredoxin--NADP reductase	1	1	1	1	1	1	1	1	1	1	1	11	CORE	COG0493_ER#	45.8	322	2.15E-87	K00528	46.3	417	1.71E-115
1533	WP_019527762.1	-	bifunctional ADP-dependent NAD(P)H-hydrate dehydratase/NAD(P)H-hydrate epimerase	0	1	1	0	1	1	1	1	1	1	1	9	Accessory	COG0063_G#	49.4	221	5.17E-57	NA	49.4	221	1.38E-56
1534	AXI87531.1	-	amidophosphoribosyltransferase	1	1	1	1	1	1	1	1	1	1	1	11	CORE	COG0034_F#	67.1	657	4.8E-188	K00764	67.1	657	1.28E-187
1535	Prokka:JAPCLKMA_04003	-	hypothetical protein	1	1	1	1	1	1	1	1	1	1	1	11	CORE								
1536	Prokka:JAPCLKMA_04887	-	hypothetical protein	1	0	0	0	0	0	0	0	0	0	0	1	Unique	COG4962_U#	24	103	2.67E-21	K07332	25.4	87	3.1E-16
1537	WP_033277789.1	-	hypothetical protein	0	0	1	0	0	0	1	0	1	0	0	3	Accessory								

cluster	GI	gene	Annotation	Streptomyces_thermocarboxydus_BPSAC147	Streptomyces_sp._4F	Streptomyces_sp._BSE7F	Streptomyces_sp._ETH9427	Streptomyces_sp._FxanaD5	Streptomyces_sp._UNC401CLCol	Actinospica_acidiphila_NRRL_B_24431	Streptomyces_sp._HNS054	Streptomyces_sp._SMS_SU21	Streptomyces_sp._ZS0098	Streptomyces_thermocarboxydus_K155	Addition	Core-Accessory-Unique	COG	%Similarity_COG	Score_COG	Evalue_COG	KEGG	%Similarity_KEGG	Score_KEGG	Evalue_KEGG
1538	OSC57764.1	-	sugar hydrolase	1	1	1	1	1	1	1	1	1	1	1	11	CORE	COG1472_G#	34.4	229	3.23E-59	K01207	35.3	211	1.86E-53
1539	WP_028960148.1	miaB	tRNA (N6-isopentenyladenosine(37)-C2)-methylthiotransferase MiaB	1	1	0	1	1	1	1	1	1	1	1	10	Accessory	COG0621_J#	71.2	704	3.42E-202	K06168	71.2	704	9.15E-202
1540	WP_048456682.1	-	hypothetical protein	0	0	0	0	0	0	1	1	0	0	1	3	Accessory	COG0810_M#	36.9	54	0.00000109	NA	23.9	66	9.67E-10
1541	WP_033276279.1	-	aromatic amino acid lyase	0	0	0	0	0	0	1	0	0	0	0	1	Unique	COG2986_E#	35.5	256	2.47E-67	K01745	35.4	235	1.2E-60
1542	WP_051724665.1	-	alpha/beta fold hydrolase	0	0	0	0	0	0	1	0	0	0	0	1	Unique	COG0596_R#	27.8	131	9.16E-30	NA	27.8	131	2.45E-29
1543	PWE11023.1	-	ATPase	0	0	1	1	0	0	0	0	0	0	0	2	Accessory	COG1672_R#	26.9	116	3.98E-25				
1544	OSC69307.1	-	carboxylesterase	0	1	0	0	0	0	0	0	0	0	0	1	Unique	COG2272_I#	34.3	233	2.24E-60	K03929	34.3	233	5.97E-60
1545	OSC70558.1	-	MFS transporter	0	1	0	0	0	0	0	0	0	0	0	1	Unique	COG0477_GEPR#	63	570	7.72E-162	NA	28.5	179	6E-44
1546	WP_122212674.1	-	MFS transporter	1	1	1	1	1	1	1	1	1	1	1	11	CORE	COG0477_GEPR#	31.2	151	1.12E-35	NA	31.2	151	2.98E-35
1547	Prokka:CIODPCDJ_03067	-	hypothetical protein	0	1	1	1	1	1	1	1	1	1	1	10	Accessory								
1548	WP_026243341.1	-	sugar ABC transporter ATP-binding protein	1	1	0	1	1	1	1	1	1	1	1	10	Accessory	COG1129_G#	45.7	417	8.35E-116	K10539	40.4	376	3.34E-103

cluster	GI	gene	Annotation	Streptomyces_thermocarboxydus_BPSAC147	Streptomyces_sp._4F	Streptomyces_sp._BSE7F	Streptomyces_sp._ETH9427	Streptomyces_sp._FxanaD5	Streptomyces_sp._UNC401CLCol	Actinospica_acidiphila_NRRL_B_24431	Streptomyces_sp._HNS054	Streptomyces_sp._SMS_SU21	Streptomyces_sp._ZS0098	Streptomyces_thermocarboxydus_K155	Addition	Core-Accessory-Unique	COG	%Similarity_COG	Score_COG	Evalue_COG	KEGG	%Similarity_KEGG	Score_KEGG	Evalue_KEGG
1549	WP_033273803.1	-	NCS1 family nucleobase:cation symporter-1	0	1	1	1	1	1	1	1	1	1	1	10	Accessory	COG1953_FH#	47.5	441	4.13E-123	K03457	42.3	414	1.89E-114
1550	WP_019526115.1	-	hypothetical protein	1	1	1	1	1	1	1	1	1	1	1	11	CORE					NA	53.1	508	9.6E-143
1551	Prokka:JAPCLKMA_06146	-	Bifunctional cytochrome P450/NADPH--P450 reductase	1	1	0	1	1	1	1	1	1	1	1	10	Accessory	COG2124_Q#	46	386	1.58E-106	K14338	45.6	386	4.21E-106
1552	Prokka:CIODPCDJ_05210	rox_2	Rifampicin monooxygenase	0	0	0	0	0	0	0	0	0	0	1	1	Unique	COG0654_HC#	53.9	513	6.55E-145	NA	32.5	213	6.38E-54
1553	OSC71871.1	-	aldehyde dehydrogenase	0	1	0	0	0	0	0	0	0	0	0	1	Unique	COG1012_C#	76	800	3.5E-231	K00138	76	800	9.36E-231
1554	Prokka:CIODPCDJ_06688	lysA_3	Diaminopimelate decarboxylase	0	1	1	1	1	1	1	1	1	1	1	10	Accessory	COG0019_E#	29.6	177	1.11E-43				
1555	WP_033274289.1	-	MFS transporter	1	0	0	0	1	1	1	0	0	0	1	5	Accessory	COG0477_GEPR#	43	381	6.63E-105	NA	29	167	3.08E-40
1556	OSC72239.1	-	phosphoesterase	1	1	1	1	1	1	0	1	1	1	1	10	Accessory	COG0613_R#	29.5	48	0.000078	K07053	29.5	48	0.000208
1557	WP_033277696.1	-	hypothetical protein	0	0	0	0	0	0	1	0	0	0	0	1	Unique								
1558	AXI85274.1	-	ATP-binding protein	1	0	0	1	0	0	0	0	0	0	0	2	Accessory								

cluster	GI	gene	Annotation	Streptomyces_thermocarboxydus_BPSAC147	Streptomyces_sp._4F	Streptomyces_sp._BSE7F	Streptomyces_sp._ETH9427	Streptomyces_sp._FxanaD5	Streptomyces_sp._UNC401CLCol	Actinospica_acidiphila_NRRL_B_24431	Streptomyces_sp._HNS054	Streptomyces_sp._SMS_SU21	Streptomyces_sp._ZS0098	Streptomyces_thermocarboxydus_K155	Addition	Core-Accessory-Unique	COG	%Similarity_COG	Score_COG	Evalue_COG	KEGG	%Similarity_KEGG	Score_KEGG	Evalue_KEGG
1559	WP_102642139.1	-	aldehyde dehydrogenase family protein	1	1	1	1	1	1	1	1	1	1	1	11	CORE	COG1012_C#	55.8	567	6.51E-161	K00130	55.8	567	1.74E-160
1560	Prokka:CIODPCDJ_00050	-	hypothetical protein	1	0	0	1	1	1	1	1	1	1	1	9	Accessory	COG2187_S#	47.2	267	1.07E-70	K07028	43.8	373	3.68E-102
1561	PWE07790.1	-	hypothetical protein	1	0	1	1	1	1	1	0	1	1	1	9	Accessory								
1562	Prokka:CIODPCDJ_03833	-	hypothetical protein	0	0	0	0	0	0	0	0	0	0	1	1	Unique								
1563	OSC59412.1	-	monooxygenase	0	1	0	0	0	0	0	0	0	0	0	1	Unique	COG0654_HC#	41.1	314	9.93E-85	NA	36.9	266	6.34E-70
1564	WP_102637939.1	-	bifunctional folylpolyglutamate synthase/dihydrofolate synthase	1	1	1	1	1	1	1	1	1	1	1	11	CORE	COG0285_H#	53.5	497	8.27E-140	K11754	52.7	476	4.03E-133
1565	WP_033273930.1	-	cellulose-binding protein	1	0	1	0	1	1	1	1	1	1	1	9	Accessory					NA	35.2	44	0.00512
1566	Prokka:JAPCLKMA_03861	sasA_10	Adaptive-response sensory-kinase SasA	1	1	1	1	1	1	1	1	1	1	1	11	CORE	COG0642_T#	34.5	168	5.16E-41	K02484	38	171	2.13E-41
1567	WP_122216582.1	-	DUF4246 domain-containing protein	1	1	1	1	1	1	1	1	1	1	1	11	CORE								
1568	WP_122213343.1	-	MFS transporter	1	1	1	1	1	1	1	1	1	1	1	11	CORE	COG0477_GEPR#	33.6	270	9.62E-72	NA	32.1	211	2.42E-53

cluster	GI	gene	Annotation	Streptomyces_thermocarboxydus_BPSAC147	Streptomyces_sp._4F	Streptomyces_sp._BSE7F	Streptomyces_sp._ETH9427	Streptomyces_sp._FxanaD5	Streptomyces_sp._UNC401CLCol	Actinospica_acidiphila_NRRL_B_24431	Streptomyces_sp._HNS054	Streptomyces_sp._SMS_SU21	Streptomyces_sp._ZS0098	Streptomyces_thermocarboxydus_K155	Addition	Core-Accessory-Unique	COG	%Similarity_COG	Score_COG	Evalue_COG	KEGG	%Similarity_KEGG	Score_KEGG	Evalue_KEGG
1569	WP_102638703.1	-	xylan 1,4-beta-xylosidase	1	0	0	1	1	1	1	1	1	1	1	9	Accessory	COG3664_G#	45.7	463	1.01E-129				
1570	WP_102638737.1	-	hypothetical protein	0	0	0	1	0	0	0	1	1	1	0	4	Accessory					NA	32.3	104	3.18E-21
1571	WP_033278115.1	-	type VI secretion protein	1	1	1	0	1	1	1	1	1	1	1	10	Accessory	COG1511_S#	41.6	60	1.51E-08	K00901	48.6	64	2.8E-09
1572	OSC71031.1	-	3-hydroxyacyl-CoA dehydrogenase	1	1	1	1	1	1	1	1	1	1	1	11	CORE	COG1250_I#	42.9	355	3.88E-97	K00074	53.5	278	1.23E-73
1573	OSC68246.1	-	peptidase	0	1	0	0	0	0	0	0	0	0	0	1	Unique	COG0308_E#	34.5	238	4.05E-62	NA	31.4	185	1.09E-45
1574	WP_033276637.1	-	serine hydrolase	0	0	0	0	1	1	1	0	0	0	1	4	Accessory	COG1680_V#	25.8	152	4.99E-36	NA	32	142	1.38E-32
1575	WP_033277434.1	eccD	type VII secretion integral membrane protein EccD	1	0	1	0	1	1	1	0	1	0	1	7	Accessory								
1576	Prokka:JAPCLKMA_05480	tycC	Tyrocidine synthase 3	1	0	0	0	0	0	0	0	0	0	0	1	Unique	COG1020_Q#	27.7	136	3.69E-31	K04788	27.3	124	3.88E-27
1577	OSC70460.1	-	aspartyl/glutamyl-tRNA amidotransferase subunit B	1	1	1	1	1	1	1	1	1	1	1	11	CORE	COG0064_J#	66.3	686	5.61E-197	K02434	66.3	686	1.5E-196
1578	WP_102637906.1	-	hypothetical protein	0	0	1	0	0	0	0	0	1	0	0	2	Accessory								
1579	Prokka:JAPCLKMA_06265	-	hypothetical protein	1	0	0	0	1	1	0	0	0	0	1	4	Accessory								

cluster	GI	gene	Annotation	Streptomyces_thermocarboxydus_BPSAC147	Streptomyces_sp._4F	Streptomyces_sp._BSE7F	Streptomyces_sp._ETH9427	Streptomyces_sp._FxanaD5	Streptomyces_sp._UNC401CLCol	Actinospica_acidiphila_NRRL_B_24431	Streptomyces_sp._HNS054	Streptomyces_sp._SMS_SU21	Streptomyces_sp._ZS0098	Streptomyces_thermocarboxydus_K155	Addition	Core-Accessory-Unique	COG	%Similarity_COG	Score_COG	Evalue_COG	KEGG	%Similarity_KEGG	Score_KEGG	Evalue_KEGG
1580	Prokka:CIODPCDJ_05541	alkA	putative bifunctional transcriptional activator/DNA repair enzyme AlkA	1	1	1	1	1	1	1	1	1	1	1	11	CORE	COG0122_L#	59.3	367	9.84E-101	K13529	64.1	630	1.67E-179
1581	WP_102641284.1	-	serine/threonine-protein phosphatase	1	1	1	1	1	1	1	1	1	1	1	11	CORE	COG2208_TK#	28.1	92	6.1E-18				
1582	Prokka:JAPCLKMA_06367	dop	Depupylase	1	1	0	1	1	1	1	1	1	1	1	10	Accessory					K13571	62.1	616	2.48E-175
1583	WP_122216602.1	-	FAD-dependent oxidoreductase	0	0	1	0	1	1	1	0	1	1	1	7	Accessory	COG0654_HC#	38.4	287	1.29E-76	NA	38.4	287	3.45E-76
1584	WP_033272621.1	-	helix-turn-helix domain-containing protein	1	1	1	0	1	1	1	1	1	1	1	10	Accessory					NA	37.4	79	1.09E-13
1585	OSC57697.1	-	sulfate transporter	1	1	1	1	1	1	1	1	1	1	1	11	CORE	COG0659_P#	48.4	409	1.73E-113	K01673	38.6	323	4.35E-87
1586	Prokka:JAPCLKMA_00961	-	hypothetical protein	1	1	0	0	1	1	0	0	0	0	1	5	Accessory	COG3284_QK#	29.5	75	4.52E-13	K02481	33.1	64	4.74E-09
1587	Prokka:JAPCLKMA_05485	ppsB	Plipastatin synthase subunit B	1	0	0	0	0	0	0	0	0	0	0	1	Unique	COG0318_IQ#	30.5	196	2.99E-49	K15665	29.9	158	1.41E-37

cluster	GI	gene	Annotation	Streptomyces_thermocarboxydus_BPSAC147	Streptomyces_sp._4F	Streptomyces_sp._BSE7F	Streptomyces_sp._ETH9427	Streptomyces_sp._FxanaD5	Streptomyces_sp._UNC401CLCol	Actinospica_acidiphila_NRRL_B_24431	Streptomyces_sp._HNS054	Streptomyces_sp._SMS_SU21	Streptomyces_sp._ZS0098	Streptomyces_thermocarboxydus_K155	Addition	Core-Accessory-Unique	COG	%Similarity_COG	Score_COG	Evalue_COG	KEGG	%Similarity_KEGG	Score_KEGG	Evalue_KEGG
1588	WP_106959473.1	-	DUF2252 domain-containing protein	1	1	1	1	1	1	1	1	1	1	1	11	CORE	COG4320_S#	26.7	134	1.07E-30				
1589	OSC66072.1	-	anthranilate synthase component I	1	1	1	1	1	1	1	1	1	1	1	11	CORE	COG0147_EH#	59.8	568	2.9E-161	K01657	59.8	568	7.74E-161
1590	AXI84782.1	-	sodium-independent anion transporter	1	0	1	1	1	1	1	1	1	1	1	10	Accessory	COG0659_P#	63.7	580	4.32E-165	K03321	53.3	485	5.05E-136
1591	OSC73483.1	-	IMP dehydrogenase	1	1	1	1	1	1	1	1	1	1	1	11	CORE	COG0516_F#	69	403	9.45E-112	K00088	66.8	659	3.33E-188
1592	OSC66537.1	-	hypothetical protein	1	1	1	1	1	1	1	1	1	1	1	11	CORE	COG5650_S#	38	285	3.75E-76	NA	36.5	284	1.71E-75
1593	WP_033273292.1	-	cytochrome ubiquinol oxidase subunit I	1	1	1	1	1	1	1	1	1	1	1	11	CORE	COG1271_C#	45	414	5.35E-115	K00425	41.3	373	3.65E-102
1594	WP_122216250.1	-	NADH-quinone oxidoreductase subunit M	0	0	0	1	0	0	0	1	1	1	0	4	Accessory	COG1008_C#	39	332	3.49E-90	K00342	39	332	9.32E-90
1595	Prokka:CIODPCDJ_00377	-	hypothetical protein	1	0	1	1	1	1	1	1	1	1	1	10	Accessory								
1596	AXI86564.1	-	alpha-N-arabinofuranosidase	1	1	1	1	1	1	1	1	1	1	1	11	CORE	COG3534_G#	54.8	560	7.88E-159	K01209	52.5	541	7.74E-153
1597	WP_102638712.1	-	amino acid permease	1	1	1	1	1	1	1	1	1	1	1	11	CORE	COG0531_E#	52.7	498	2.16E-140	K03294	52.7	498	5.76E-140
1598	WP_102637934.1	-	trigger factor	1	1	1	1	1	1	1	1	1	1	1	11	CORE	COG0544_O#	43	374	8.01E-103	K03545	42.3	374	1.25E-102

cluster	GI	gene	Annotation	Streptomyces_thermocarboxydus_BPSAC147	Streptomyces_sp._4F	Streptomyces_sp._BSE7F	Streptomyces_sp._ETH9427	Streptomyces_sp._FxanaD5	Streptomyces_sp._UNC401CLCol	Actinospica_acidiphila_NRRL_B_24431	Streptomyces_sp._HNS054	Streptomyces_sp._SMS_SU21	Streptomyces_sp._ZS0098	Streptomyces_thermocarboxydus_K155	Addition	Core-Accessory-Unique	COG	%Similarity_COG	Score_COG	Evalue_COG	KEGG	%Similarity_KEGG	Score_KEGG	Evalue_KEGG
1599	OSC62188.1	-	MFS transporter	0	1	0	0	0	0	0	0	0	0	0	1	Unique	COG3104_E#	41	391	4.85E-108	K03305	41	392	7.59E-108
1600	WP_019527551.1	mmsA	CoA-acylating methylmalonate-semialdehyde dehydrogenase	1	1	1	1	1	1	1	1	1	1	1	11	CORE	COG1012_C#	60.2	622	1.68E-177	K00140	51.9	499	4.4E-140
1601	Prokka:JAPCLKMA_03350	-	hypothetical protein	1	1	0	1	1	1	1	1	1	1	1	10	Accessory								
1602	Prokka:JAPCLKMA_02734	-	hypothetical protein	1	0	0	0	0	0	0	0	0	0	0	1	Unique								
1603	AXI90535.1	-	hypothetical protein	0	0	1	1	0	0	0	1	1	1	0	5	Accessory	COG0654_HC#	48.9	458	4.21E-128	NA	41.8	363	2.88E-99
1604	WP_084206908.1	-	inositol phosphorylceramide synthase	1	1	1	1	1	1	1	1	1	1	1	11	CORE								
1605	OSC61659.1	-	monooxygenase	0	1	0	0	0	0	0	0	0	0	0	1	Unique	COG0654_HC#	45.5	414	4.08E-115	NA	45.5	414	1.09E-114
1606	OSC67296.1	-	short-chain dehydrogenase	1	1	0	1	1	1	1	1	1	1	1	10	Accessory								
1607	OSC71190.1	-	hypothetical protein	0	1	0	0	0	0	0	0	0	0	0	1	Unique								
1608	OSC64557.1	-	ornithine decarboxylase	1	1	0	0	1	1	1	0	1	1	1	8	Accessory	COG1982_E#	37.9	347	8.01E-95	K01585	37.1	332	7.12E-90

cluster	GI	gene	Annotation	Streptomyces_thermocarboxydus_BPSAC147	Streptomyces_sp._4F	Streptomyces_sp._BSE7F	Streptomyces_sp._ETH9427	Streptomyces_sp._FxanaD5	Streptomyces_sp._UNC401CLCol	Actinospica_acidiphila_NRRL_B_24431	Streptomyces_sp._HNS054	Streptomyces_sp._SMS_SU21	Streptomyces_sp._ZS0098	Streptomyces_thermocarboxydus_K155	Addition	Core-Accessory-Unique	COG	%Similarity_COG	Score_COG	Evalue_COG	KEGG	%Similarity_KEGG	Score_KEGG	Evalue_KEGG
1609	WP_026243661.1	-	PLP-dependent aminotransferase family protein	1	1	1	1	1	1	1	1	1	1	1	11	CORE	COG1167_KE#	28.9	153	2.89E-36	NA	26.4	153	4.52E-36
1610	Prokka:JAPCLKMA_00616	pdhC	Dihydrolipoyllysine-residue acetyltransferase component of pyruvate dehydrogenase complex	1	1	0	1	1	1	1	1	1	1	1	10	Accessory	COG0508_C#	34	259	2.19E-68	K00627	34	259	5.85E-68
1611	AXI90724.1	-	cobyrinate a,c-diamide synthase	1	1	1	1	1	1	1	1	1	1	1	11	CORE	COG1797_H#	41.3	347	1.04E-94	K02224	46.2	255	8.45E-67
1612	WP_019524417.1	hpnR	hopanoid C-3 methylase HpnR	1	1	1	1	1	1	1	1	1	1	1	11	CORE	COG1032_C#	29.1	159	4.03E-38	NA	25.5	111	2.57E-23
1613	OSC64653.1	-	MFS transporter	0	1	0	0	0	0	0	0	0	0	0	1	Unique	COG0477_GEPR#	45.7	398	3.95E-110	NA	36	260	3.43E-68
1614	OSC71381.1	-	B12-binding domain-containing radical SAM protein	0	1	0	0	0	0	0	0	0	0	0	1	Unique								
1615	OSC71982.1	-	acyl-CoA synthetase	0	1	0	0	0	0	0	0	0	0	0	1	Unique	COG0318_IQ#	43.7	292	2.34E-78	K00666	34.4	255	1.1E-66

cluster	GI	gene	Annotation	Streptomyces_thermocarboxydus_BPSAC147	Streptomyces_sp._4F	Streptomyces_sp._BSE7F	Streptomyces_sp._ETH9427	Streptomyces_sp._FxanaD5	Streptomyces_sp._UNC401CLCol	Actinospica_acidiphila_NRRL_B_24431	Streptomyces_sp._HNS054	Streptomyces_sp._SMS_SU21	Streptomyces_sp._ZS0098	Streptomyces_thermocarboxydus_K155	Addition	Core-Accessory-Unique	COG	%Similarity_COG	Score_COG	Evalue_COG	KEGG	%Similarity_KEGG	Score_KEGG	Evalue_KEGG
1616	WP_102640578.1	-	MFS transporter	1	1	1	1	1	1	1	1	1	1	1	11	CORE	COG3104_E#	44.9	395	3.34E-109	K03305	44.9	395	8.91E-109
1617	PWE09514.1	-	Asp-tRNA(Asn)/Glu-tRNA(Gln) amidotransferase subunit GatA	1	1	1	1	1	1	1	1	1	1	1	11	CORE	COG0154_J#	70.8	668	1.56E-191	K02433	70.8	669	3.2E-191
1618	Prokka:CIODPCDJ_04825	-	hypothetical protein	1	1	1	1	1	1	1	1	1	1	1	11	CORE								
1619	WP_048456487.1	-	hypothetical protein	0	0	1	1	0	0	0	1	1	1	0	5	Accessory								
1620	WP_033275233.1	-	membrane protein	1	1	1	1	1	1	1	1	1	1	1	11	CORE								
1621	OSC61325.1	-	beta-ketoacyl synthase	0	1	0	0	0	0	0	0	0	0	0	1	Unique	COG3321_Q#	45.8	402	2.73E-111	K12436	45.8	402	7.29E-111
1622	PWE11419.1	-	hypothetical protein	0	0	1	0	0	0	0	0	0	0	0	1	Unique	COG0477_GEPR#	36.2	270	1.62E-71	NA	35.2	262	1.18E-68
1623	WP_033273235.1	-	serine/threonine protein kinase	1	1	0	1	1	1	1	1	1	1	1	10	Accessory	COG0515_RTKL#	30.4	146	2.7E-34	K08884	30.4	146	7.21E-34
1624	WP_026243832.1	-	site-specific integrase	0	0	0	0	1	0	0	0	0	0	0	1	Unique								
1625	Prokka:CIODPCDJ_03458	lcfB_3	Long-chain-fatty-acid--CoA ligase	0	0	0	0	0	0	0	0	0	0	1	1	Unique	COG0318_IQ#	37.6	314	5.73E-85	NA	36.5	295	9.61E-79
1626	Prokka:JAPCLKMA_05680	-	hypothetical protein	1	0	0	0	0	0	0	0	0	0	0	1	Unique								
1627	WP_026244042.1	hflX	GTPase HflX	1	1	1	1	1	1	1	1	1	1	1	11	CORE	COG2262_R#	65.3	547	5.24E-155	K03665	65.3	547	1.4E-154

cluster	GI	gene	Annotation	Streptomyces_thermocarboxydus_BPSAC147	Streptomyces_sp._4F	Streptomyces_sp._BSE7F	Streptomyces_sp._ETH9427	Streptomyces_sp._FxanaD5	Streptomyces_sp._UNC401CLCol	Actinospica_acidiphila_NRRL_B_24431	Streptomyces_sp._HNS054	Streptomyces_sp._SMS_SU21	Streptomyces_sp._ZS0098	Streptomyces_thermocarboxydus_K155	Addition	Core-Accessory-Unique	COG	%Similarity_COG	Score_COG	Evalue_COG	KEGG	%Similarity_KEGG	Score_KEGG	Evalue_KEGG
1628	WP_033273080.1	rpsA	30S ribosomal protein S1	1	1	1	1	1	1	1	1	1	1	1	11	CORE	COG0539_J#	79.3	754	2.16E-217	K02945	79.3	754	5.77E-217
1629	OSC71950.1	-	MFS transporter	0	1	0	0	0	0	0	0	0	0	0	1	Unique	COG0477_GEPR#	48.3	457	5.46E-128	K03762	47.4	456	4.24E-127
1630	WP_122213949.1	-	ATP-binding protein	0	0	1	0	0	0	0	0	1	1	0	3	Accessory	COG1672_R#	25.8	101	7.61E-21				
1631	Prokka:JAPCLKMA_03334	-	hypothetical protein	1	1	1	1	1	1	1	1	1	1	1	11	CORE	COG1289_S#	32.6	138	7.33E-32				
1632	OSC63248.1	-	Xaa-Pro aminopeptidase	1	1	1	1	1	1	1	1	1	1	1	11	CORE	COG0006_E#	33.9	235	4.41E-61	K01262	31.8	230	2.9E-59
1633	Prokka:CIODPCDJ_00479	-	55.5 kDa and 49.5 kDa sporulation proteins	1	1	1	1	1	1	1	1	1	1	1	11	CORE								
1634	AXI85975.1	-	ribosome biogenesis GTPase Der	0	1	1	1	1	1	1	1	1	1	1	10	Accessory	COG1160_R#	63.8	586	1.02E-166	K03977	63.8	586	2.71E-166
1635	PWE11305.1	-	hydrolase	1	0	1	1	1	1	1	1	1	1	1	10	Accessory	COG2234_R#	47.1	173	2.68E-42	NA	33.3	149	8.48E-35
1636	WP_048455715.1	-	zinc-dependent metalloprotease	1	1	1	1	1	1	1	1	1	1	1	11	CORE	COG5282_S#	46.1	411	3.43E-114	NA	46.1	411	9.16E-114
1637	PWE12056.1	-	PucR family transcriptional regulator	1	1	1	1	1	1	1	1	1	1	1	11	CORE	COG2508_TQ#	23	48	0.0000763				
1638	PWE09034.1	-	hypothetical protein	0	0	1	1	0	0	0	1	0	0	0	3	Accessory	COG5651_N#	33.3	69	4.18E-11	NA	31.9	73	7.72E-12

cluster	GI	gene	Annotation	Streptomyces_thermocarboxydus_BPSAC147	Streptomyces_sp._4F	Streptomyces_sp._BSE7F	Streptomyces_sp._ETH9427	Streptomyces_sp._FxanaD5	Streptomyces_sp._UNC401CLCol	Actinospica_acidiphila_NRRL_B_24431	Streptomyces_sp._HNS054	Streptomyces_sp._SMS_SU21	Streptomyces_sp._ZS0098	Streptomyces_thermocarboxydus_K155	Addition	Core-Accessory-Unique	COG	%Similarity_COG	Score_COG	Evalue_COG	KEGG	%Similarity_KEGG	Score_KEGG	Evalue_KEGG
1639	AXI86185.1	gltD	glutamate synthase subunit beta	1	1	1	1	1	1	1	1	1	1	1	11	CORE	COG0493_ER#	59.6	581	3.27E-165	K00266	59.6	581	8.73E-165
1640	OSC64816.1	-	hypothetical protein	1	1	0	1	1	1	1	1	1	1	1	10	Accessory								
1641	OSC72546.1	-	ATP/GTP-binding protein	0	1	0	0	1	0	0	0	0	0	1	3	Accessory	COG4962_U#	31.4	146	2.06E-34	K02283	31.7	134	2.82E-30
1642	Prokka:JAPCLKMA_01307	-	hypothetical protein	1	0	0	0	0	0	0	0	1	0	0	2	Accessory								
1643	OSC72428.1	-	esterase	0	1	0	0	0	0	0	0	0	0	0	1	Unique	COG3509_Q#	42.2	235	5.75E-61				
1644	OSC62351.1	-	nitrate reductase	0	1	0	0	0	0	0	0	0	0	0	1	Unique	COG1953_FH#	57.6	574	3.05E-163	K03457	34.2	289	5.24E-77
1645	OSC61789.1	-	aldehyde dehydrogenase	0	1	0	0	0	0	0	0	0	0	0	1	Unique	COG1012_C#	56.4	551	2.77E-156	K00128	38.1	343	3.06E-93
1646	Prokka:JAPCLKMA_05134	ygbN	Inner membrane permease YgbN	1	1	1	1	1	1	1	1	1	1	1	11	CORE	COG2610_GE#	42.4	353	1.11E-96	K03299	42.4	353	2.95E-96
1647	WP_019522472.1	-	glycosyltransferase family 4 protein	1	0	1	1	1	1	1	1	1	1	1	10	Accessory	COG0438_M#	26.6	125	4.9E-28	K15521	26.7	119	1.22E-25
1648	OSC62311.1	-	Cro/Cl family transcriptional regulator	0	1	0	0	0	0	0	0	0	0	0	1	Unique	COG1511_S#	23.9	42	0.00712				
1649	AXI89565.1	-	PAP2 family protein	0	0	0	1	0	0	0	1	0	0	0	2	Accessory	COG0671_I#	25.9	86	4.3E-16	K19302	36.9	77	4.09E-13
1650	Prokka:JAPCLKMA_01129	-	hypothetical protein	1	0	0	0	1	1	1	0	0	0	1	5	Accessory								

cluster	GI	gene	Annotation	Streptomyces_thermocarboxydus_BPSAC147	Streptomyces_sp._4F	Streptomyces_sp._BSE7F	Streptomyces_sp._ETH9427	Streptomyces_sp._FxanaD5	Streptomyces_sp._UNC401CLCol	Actinospica_acidiphila_NRRL_B_24431	Streptomyces_sp._HNS054	Streptomyces_sp._SMS_SU21	Streptomyces_sp._ZS0098	Streptomyces_thermocarboxydus_K155	Addition	Core-Accessory-Unique	COG	%Similarity_COG	Score_COG	Evalue_COG	KEGG	%Similarity_KEGG	Score_KEGG	Evalue_KEGG
1651	OSC64546.1	-	monooxygenase	0	1	0	0	0	0	0	0	0	0	0	1	Unique	COG0654_HC#	42.5	341	4.35E-93	NA	37.5	314	1.99E-84
1652	OSC62579.1	-	ABC transporter substrate-binding protein	0	1	0	0	0	0	0	0	0	0	0	1	Unique	COG0747_E#	35.6	250	1.72E-65	K02035	27	131	1.83E-29
1653	Prokka:JAPCLKMA_01890	emrY	putative multidrug resistance protein EmrY	1	0	0	0	1	1	0	0	0	0	1	4	Accessory	COG0477_GEPR#	40.8	319	2.31E-86	NA	38.9	319	6.17E-86
1654	Prokka:CIODPCDJ_05289	lcfB_4	Long-chain-fatty-acid--CoA ligase	0	0	0	0	1	1	1	0	0	0	1	4	Accessory	COG0318_IQ#	25.8	116	2.97E-25	K01897	26.2	72	1.31E-11
1655	Prokka:JAPCLKMA_02832	-	hypothetical protein	1	0	0	0	0	0	0	0	0	0	0	1	Unique								
1656	PWE11041.1	-	hypothetical protein	0	0	1	0	0	0	0	0	0	0	0	1	Unique	COG2203_T#	37.2	131	6.84E-30	NA	40.8	122	1.11E-26
1657	WP_033273792.1	-	neuramidase	0	1	0	0	0	0	1	0	0	0	0	2	Accessory	COG4409_G#	42.6	280	9.11E-75	K01186	32.1	120	4.21E-26
1658	AXI86295.1	-	NAD(P)/FAD-dependent oxidoreductase	1	1	1	1	1	1	1	1	1	1	1	11	CORE	COG1233_Q#	28.2	141	6.59E-33	K09516	50	46	0.00101
1659	Prokka:CIODPCDJ_04778	gltX	Glutamate--tRNA ligase	1	1	1	1	1	1	1	1	1	1	1	11	CORE	COG0008_J#	57.9	541	2.86E-153	K01885	57.9	541	7.63E-153
1660	WP_122215859.1	-	AMP-binding protein	0	1	1	1	0	0	1	1	1	1	0	7	Accessory	COG0318_IQ#	34.8	290	1.5E-77	K00666	35.2	263	3.07E-69
1661	OSC57733.1	-	1,4-beta-xylanase	1	1	1	1	1	1	1	1	1	1	1	11	CORE	COG3693_G#	35.5	206	2.18E-52	NA	31.4	126	1E-27
1662	OSC63135.1	-	penicillin-binding protein	1	1	1	1	1	1	1	1	1	1	1	11	CORE	COG0768_M#	36.7	304	1E-81	K05364	36.7	304	2.68E-81

cluster	GI	gene	Annotation	Streptomyces_thermocarboxydus_BPSAC147	Streptomyces_sp._4F	Streptomyces_sp._BSE7F	Streptomyces_sp._ETH9427	Streptomyces_sp._FxanaD5	Streptomyces_sp._UNC401CLCol	Actinospica_acidiphila_NRRL_B_24431	Streptomyces_sp._HNS054	Streptomyces_sp._SMS_SU21	Streptomyces_sp._ZS0098	Streptomyces_thermocarboxydus_K155	Addition	Core-Accessory-Unique	COG	%Similarity_COG	Score_COG	Evalue_COG	KEGG	%Similarity_KEGG	Score_KEGG	Evalue_KEGG
1663	AXI89592.1	-	IS21 family transposase	1	0	0	1	1	0	0	0	0	0	0	3	Accessory	COG4584_L#	34.3	179	2.85E-44				
1664	WP_048459741.1	-	CocE/NonD family hydrolase	0	0	1	1	0	0	1	1	1	1	0	6	Accessory	COG2936_R#	30.2	157	1.16E-37	K06978	29.4	121	1.88E-26
1665	Prokka:CIODPCDJ_06476	mleN	Malate-2H(+)/Na(+)-lactate antiporter	1	0	0	0	1	1	1	0	0	0	1	5	Accessory	COG1757_C#	45.9	413	1.53E-114	K03315	35	290	4E-77
1666	AXI87208.1	-	two-component sensor histidine kinase	1	1	1	1	1	1	1	0	1	1	1	10	Accessory	COG4585_T#	26.3	85	7.31E-16	NA	26.3	85	1.95E-15
1667	WP_159020871.1	-	hypothetical protein	0	0	0	0	0	0	0	1	0	0	0	1	Unique								
1668	Prokka:CIODPCDJ_06185	merA_2	Mercuric reductase	1	1	1	1	1	1	1	1	1	1	1	11	CORE	COG1249_C#	61.9	617	4.09E-176	K00382	61.9	617	1.09E-175
1669	WP_026244063.1	-	sugar transferase	1	0	1	1	1	1	1	1	1	1	1	10	Accessory	COG2148_M#	31.7	194	1.11E-48	NA	31.7	194	2.98E-48
1670	WP_122215849.1	-	hypothetical protein	0	0	1	1	0	0	0	1	1	1	0	5	Accessory	COG0654_HC#	38.3	296	1.6E-79	NA	38.3	296	4.26E-79
1671	WP_026243900.1	dnaB	replicative DNA helicase	1	1	1	1	1	1	1	1	1	1	1	11	CORE	COG0305_L#	53.9	525	1.62E-148	K02314	63	517	1.18E-145
1672	OSC54586.1	-	(4Fe-4S)-binding protein	1	1	1	1	1	1	1	1	1	1	1	11	CORE	COG1139_C#	62.6	416	1.38E-115	K18929	62.6	416	3.69E-115
1673	WP_019523727.1	-	amino acid permease	1	1	1	1	1	1	1	1	1	1	1	11	CORE	COG0531_E#	49.5	455	2.68E-127	NA	49.5	455	7.17E-127
1674	Prokka:JAPCLKMA_06452	putP	Sodium/proline symporter	1	1	1	1	1	1	1	1	1	1	1	11	CORE	COG0591_ER#	56.6	518	3.37E-146	K03307	26.4	134	3.66E-30
1675	OSC61413.1	-	protoporphyrinogen oxidase	1	1	1	1	1	1	1	1	1	1	1	11	CORE	COG1232_H#	38.9	257	1.07E-67	K00231	38.9	257	2.86E-67

cluster	GI	gene	Annotation	Streptomyces_thermocarboxydus_BPSAC147	Streptomyces_sp._4F	Streptomyces_sp._BSE7F	Streptomyces_sp._ETH9427	Streptomyces_sp._FxanaD5	Streptomyces_sp._UNC401CLCol	Actinospica_acidiphila_NRRL_B_24431	Streptomyces_sp._HNS054	Streptomyces_sp._SMS_SU21	Streptomyces_sp._ZS0098	Streptomyces_thermocarboxydus_K155	Addition	Core-Accessory-Unique	COG	%Similarity_COG	Score_COG	Evalue_COG	KEGG	%Similarity_KEGG	Score_KEGG	Evalue_KEGG
1676	WP_033277354.1	-	hypothetical protein	0	1	0	1	1	0	1	0	0	0	0	4	Accessory								
1677	WP_033274105.1	-	3' terminal RNA ribose 2'-O-methyltransferase Hen1	1	0	1	1	1	0	1	1	1	1	1	9	Accessory	COG2890_J#	33.3	44	0.00186	NA	30.9	125	1.7E-27
1678	WP_102637244.1	rimO	30S ribosomal protein S12 methylthiotransferase RimO	1	1	1	1	1	1	1	1	1	1	1	11	CORE	COG0621_J#	32.5	240	1.04E-62	K14441	32.5	240	2.77E-62
1679	OSC69327.1	-	ATPase	1	1	1	1	1	1	1	1	1	1	1	11	CORE	COG3920_T#	47.1	393	1.25E-108	K00936	47.1	393	3.34E-108
1680	Prokka:JAPCLKMA_02462	-	hypothetical protein	1	0	0	0	0	0	0	0	0	0	0	1	Unique					K13171	26.7	58	1.95E-07
1681	OSC63345.1	-	hypothetical protein	0	1	0	0	0	0	0	0	0	0	0	1	Unique								
1682	OSC65366.1	-	xanthine dehydrogenase small subunit	0	1	0	0	0	0	0	0	0	0	0	1	Unique	COG4630_F#	64.2	618	2.39E-176	K13481	64.2	618	6.38E-176
1683	Prokka:CIODPCDJ_03492	-	hypothetical protein	0	0	0	0	0	0	1	0	0	0	1	2	Accessory								
1684	WP_019521477.1	-	alpha-L-arabinofuranosidase	1	1	1	1	1	1	1	1	1	1	1	11	CORE	COG2730_G#	29.4	87	1.46E-16	K01179	29.4	87	3.91E-16
1685	AXI88008.1	-	GntR family transcriptional regulator	1	0	1	1	1	1	1	1	1	1	1	10	Accessory	COG1167_KE#	37	245	5.49E-64	K00375	39.4	237	3.05E-61
1686	WP_048457881.1	-	amino acid permease	1	1	1	1	1	1	1	1	1	1	1	11	CORE	COG1113_E#	56.3	508	2.66E-143	K11735	56.3	509	4.16E-143

cluster	GI	gene	Annotation	Streptomyces_thermocarboxydus_BPSAC147	Streptomyces_sp._4F	Streptomyces_sp._BSE7F	Streptomyces_sp._ETH9427	Streptomyces_sp._FxanaD5	Streptomyces_sp._UNC401CLCol	Actinospica_acidiphila_NRRL_B_24431	Streptomyces_sp._HNS054	Streptomyces_sp._SMS_SU21	Streptomyces_sp._ZS0098	Streptomyces_thermocarboxydus_K155	Addition	Core-Accessory-Unique	COG	%Similarity_COG	Score_COG	Evalue_COG	KEGG	%Similarity_KEGG	Score_KEGG	Evalue_KEGG
1687	WP_078504582.1	-	DUF2971 domain-containing protein	0	0	0	0	1	0	0	0	0	0	0	1	Unique								
1688	AXI88386.1	-	sensor histidine kinase	1	0	1	1	0	0	1	1	1	1	1	8	Accessory	COG0642_T#	42.5	236	1.95E-61	K02484	42.2	233	3.38E-60
1689	WP_048460559.1	-	DUF2510 domain-containing protein	1	1	0	1	1	1	1	1	1	1	1	10	Accessory	COG2812_L#	33.3	69	5.39E-11	NA	31	105	1.39E-21
1690	WP_048459193.1	-	ATP-binding protein	0	0	0	0	0	0	0	1	0	0	0	1	Unique								
1691	Prokka:JAPCLKMA_01532	-	hypothetical protein	1	0	0	1	0	0	0	0	0	0	0	2	Accessory								
1692	WP_033275752.1	-	acyl-CoA synthetase	1	1	1	1	1	1	1	1	1	1	1	11	CORE	COG0318_IQ#	52.8	433	1.42E-120	K12429	51	431	1.1E-119
1693	OSC65696.1	-	Tat pathway signal sequence domain protein	0	1	0	0	0	0	0	0	0	0	0	1	Unique	COG3211_R#	38.3	305	2.61E-82				
1694	Prokka:JAPCLKMA_05901	xylB_2	Xylulose kinase	1	1	1	1	1	1	1	1	1	1	1	11	CORE	COG1070_G#	43.7	351	7.08E-96				
1695	WP_033273638.1	-	hypothetical protein	0	0	0	0	1	1	1	0	0	0	0	3	Accessory	COG0840_NT#	30.4	51	0.00000888	NA	23.8	59	8.71E-08
1696	AXI87216.1	-	MFS transporter	1	1	1	1	1	1	1	1	1	1	1	11	CORE	COG0477_GEPR#	36	287	7.36E-77	K07001	34.9	255	1.41E-66
1697	AXI88677.1	-	catalase	1	1	1	1	1	1	1	1	1	1	1	11	CORE	COG0753_P#	62.5	603	6.06E-172	K03781	62.5	605	4.26E-172
1698	Prokka:JAPCLKMA_01338	-	hypothetical protein	1	0	0	0	0	0	0	0	0	0	0	1	Unique					NA	23.8	52	0.0000139

cluster	GI	gene	Annotation	Streptomyces_thermocarboxydus_BPSAC147	Streptomyces_sp._4F	Streptomyces_sp._BSE7F	Streptomyces_sp._ETH9427	Streptomyces_sp._FxanaD5	Streptomyces_sp._UNC401CLCol	Actinospica_acidiphila_NRRL_B_24431	Streptomyces_sp._HNS054	Streptomyces_sp._SMS_SU21	Streptomyces_sp._ZS0098	Streptomyces_thermocarboxydus_K155	Addition	Core-Accessory-Unique	COG	%Similarity_COG	Score_COG	Evalue_COG	KEGG	%Similarity_KEGG	Score_KEGG	Evalue_KEGG
1699	Prokka:JAPCLKMA_06341	mprB	Signal transduction histidine-protein kinase/phosphatase MprB	1	1	1	1	1	1	1	1	1	1	1	11	CORE	COG0642_T#	37.9	284	8.14E-76	K07653	37.9	284	2.17E-75
1700	WP_048456055.1	-	NAD(P)/FAD-dependent oxidoreductase	0	0	0	0	1	1	0	1	0	0	1	4	Accessory	COG1233_Q#	50.3	443	1.05E-123	NA	50.3	443	2.8E-123
1701	WP_028961320.1	-	SAVED domain-containing protein	0	0	0	0	0	1	0	0	0	0	0	1	Unique								
1702	OSC62877.1	-	MFS transporter	0	1	0	0	0	0	0	0	0	0	0	1	Unique	COG0477_GEPR#	54.9	519	1.5E-146	K03290	55.6	517	1.17E-145
1703	OSC70026.1	-	two-component sensor histidine kinase	1	1	0	1	1	1	1	1	1	1	1	10	Accessory	COG0642_T#	27.6	105	5.18E-22	NA	27.3	103	6.87E-21
1704	OSC72138.1	-	CCA tRNA nucleotidyltransferase	1	1	1	1	1	1	1	1	1	1	1	11	CORE	COG0617_J#	60.2	560	7.69E-159	K00970	60.2	560	2.05E-158
1705	OSC71393.1	-	hypothetical protein	0	1	0	0	0	0	0	0	0	0	0	1	Unique								
1706	Prokka:JAPCLKMA_06656	-	hypothetical protein	1	1	1	1	1	1	1	1	1	1	1	11	CORE	COG1252_C#	29.3	183	1.96E-45	K03885	28.9	180	4.42E-44
1707	OSC57746.1	-	hypothetical protein	1	1	1	1	1	1	1	1	1	1	1	11	CORE								

cluster	GI	gene	Annotation	Streptomyces_thermocarboxydus_BPSAC147	Streptomyces_sp._4F	Streptomyces_sp._BSE7F	Streptomyces_sp._ETH9427	Streptomyces_sp._FxanaD5	Streptomyces_sp._UNC401CLCol	Actinospica_acidiphila_NRRL_B_24431	Streptomyces_sp._HNS054	Streptomyces_sp._SMS_SU21	Streptomyces_sp._ZS0098	Streptomyces_thermocarboxydus_K155	Addition	Core-Accessory-Unique	COG	%Similarity_COG	Score_COG	Evalue_COG	KEGG	%Similarity_KEGG	Score_KEGG	Evalue_KEGG
1708	Prokka:CIODPCDJ_02895	adeQ_1	Adenine permease AdeQ	1	1	1	1	1	1	1	1	1	1	1	11	CORE	COG2252_R#	45.6	413	1.16E-114	K06901	38.1	305	6.96E-82
1709	WP_122216609.1	-	aldehyde dehydrogenase family protein	0	0	0	0	0	0	0	0	0	1	0	1	Unique	COG1012_C#	48	374	4.57E-103				
1710	WP_037778998.1	-	phage portal protein	0	0	0	0	1	0	0	0	0	0	0	1	Unique								
1711	OSC71416.1	-	4-hydroxyphenylacetate 3-hydroxylase	0	1	0	0	0	0	0	0	0	0	0	1	Unique	COG2368_Q#	34.2	236	1.94E-61	K00483	34.2	236	5.19E-61
1712	OSC68244.1	-	L-asparagine permease	1	1	0	0	1	1	1	0	0	0	1	6	Accessory	COG1113_E#	60.2	575	1.76E-163	K11738	58.5	566	2.18E-160
1713	OSC66765.1	-	helicase	0	1	0	0	0	0	0	0	0	0	0	1	Unique								
1714	Prokka:JAPCLKMA_02636	-	55.5 kDa and 49.5 kDa sporulation proteins	1	0	0	0	1	1	1	1	1	1	1	8	Accessory								
1715	PWE08611.1	nhaA	Na+/H+ antiporter NhaA	1	1	1	1	1	1	1	1	1	1	1	11	CORE	COG3004_P#	43.3	310	1.06E-83	K03313	39.3	262	8.81E-69
1716	WP_048456598.1	-	cyclic nucleotide-binding domain-containing protein	1	1	1	1	1	1	1	1	1	1	1	11	CORE	COG0642_T#	32	170	1.71E-41	NA	32	82	9.57E-15
1717	Prokka:JAPCLKMA_06499	-	hypothetical protein	1	0	0	0	0	0	0	0	0	0	0	1	Unique								

cluster	GI	gene	Annotation	Streptomyces_thermocarboxydus_BPSAC147	Streptomyces_sp._4F	Streptomyces_sp._BSE7F	Streptomyces_sp._ETH9427	Streptomyces_sp._FxanaD5	Streptomyces_sp._UNC401CLCol	Actinospica_acidiphila_NRRL_B_24431	Streptomyces_sp._HNS054	Streptomyces_sp._SMS_SU21	Streptomyces_sp._ZS0098	Streptomyces_thermocarboxydus_K155	Addition	Core-Accessory-Unique	COG	%Similarity_COG	Score_COG	Evalue_COG	KEGG	%Similarity_KEGG	Score_KEGG	Evalue_KEGG
1718	WP_102641645.1	-	aldehyde dehydrogenase family protein	0	1	0	0	0	0	0	0	1	1	0	3	Accessory	COG1012_C#	53.3	470	6.11E-132	K00128	39.1	319	6.07E-86
1719	Prokka:CIODPCDJ_03356	-	hypothetical protein	1	1	1	1	1	1	1	1	1	1	1	11	CORE	COG0303_H#	34.7	173	2.63E-42	K03750	34.4	154	3.38E-36
1720	OSC69947.1	-	DEAD/DEAH box helicase	0	1	0	0	0	0	0	0	0	0	0	1	Unique								
1721	OSC57786.1	-	LuxR family transcriptional regulator	0	1	0	0	0	0	0	0	0	0	0	1	Unique								
1722	WP_048459515.1	-	aldehyde dehydrogenase family protein	1	1	0	1	1	1	1	1	1	1	1	10	Accessory	COG1012_C#	59.4	602	1.75E-171	K00128	57.7	589	4.1E-167
1723	WP_159020950.1	-	hypothetical protein	0	0	0	0	0	0	0	1	0	0	0	1	Unique								
1724	WP_102641621.1	-	penicillin-binding protein 2	1	1	1	1	1	1	1	1	1	1	1	11	CORE	COG0768_M#	42.5	339	2.77E-92	K05364	42.5	339	7.39E-92
1725	OSC61656.1	-	amidase	0	1	0	0	0	0	0	0	0	0	0	1	Unique	COG0154_J#	40	330	1.29E-89	K01426	40	330	3.43E-89
1726	OSC68570.1	-	membrane-associated oxidoreductase	0	1	0	0	0	0	0	0	0	0	0	1	Unique								
1727	Prokka:JAPCLKMA_02508	stp_2	Multidrug resistance protein Stp	1	1	1	1	1	1	1	1	1	1	1	11	CORE	COG0477_GEPR#	39.9	312	2.12E-84	NA	39.9	312	9.66E-84

cluster	GI	gene	Annotation	Streptomyces_thermocarboxydus_BPSAC147	Streptomyces_sp._4F	Streptomyces_sp._BSE7F	Streptomyces_sp._ETH9427	Streptomyces_sp._FxanaD5	Streptomyces_sp._UNC401CLCol	Actinospica_acidiphila_NRRL_B_24431	Streptomyces_sp._HNS054	Streptomyces_sp._SMS_SU21	Streptomyces_sp._ZS0098	Streptomyces_thermocarboxydus_K155	Addition	Core-Accessory-Unique	COG	%Similarity_COG	Score_COG	Evalue_COG	KEGG	%Similarity_KEGG	Score_KEGG	Evalue_KEGG
1728	Prokka:CIODPCDJ_02596	ahcY	Adenosylhomocysteinase	1	1	1	1	1	1	1	1	1	1	1	11	CORE	COG0499_H#	69.3	684	3.5E-196	K01251	69.3	684	9.35E-196
1729	WP_102639182.1	-	penicillin-binding protein	1	1	1	1	1	1	1	1	1	1	1	11	CORE	COG0768_M#	37.3	288	5.59E-77	K05364	37.3	288	1.49E-76
1730	WP_102637148.1	-	DUF2236 domain-containing protein	0	1	1	1	0	0	1	1	1	1	0	7	Accessory	COG3662_S#	27.7	77	1.96E-13	K13448	31	54	0.00000474
1731	Prokka:JAPCLKMA_01000	ubiD	3-octaprenyl-4-hydroxybenzoate carboxy-lyase	1	1	1	1	1	1	1	1	1	1	1	11	CORE	COG0043_H#	48.3	468	3.02E-131	K03182	48.1	465	8.92E-130
1732	WP_051101619.1	-	Gfo/Idh/MocA family oxidoreductase	1	1	1	1	1	1	1	1	1	1	1	11	CORE	COG0673_R#	30.2	65	7.7E-10	NA	37.5	299	6.48E-80
1733	Prokka:CIODPCDJ_05402	adeQ_2	Adenine permease AdeQ	1	1	1	1	1	1	1	1	1	1	1	11	CORE	COG2252_R#	44	376	2.04E-103	K06901	38.7	295	1.22E-78
1734	OSC61522.1	-	site-specific integrase	0	1	0	0	0	0	0	0	0	0	0	1	Unique								
1735	OSC71052.1	-	hypothetical protein	0	1	0	0	0	0	0	0	0	0	0	1	Unique								
1736	WP_052183339.1	-	TIR domain-containing protein	0	0	0	0	0	1	0	0	0	0	0	1	Unique								
1737	Prokka:CIODPCDJ_05657	-	hypothetical protein	0	0	0	0	0	1	0	0	0	0	1	2	Accessory	COG0642_T#	34	170	1.7E-41	NA	34	170	4.54E-41

cluster	GI	gene	Annotation	Streptomyces_thermocarboxydus_BPSAC147	Streptomyces_sp._4F	Streptomyces_sp._BSE7F	Streptomyces_sp._ETH9427	Streptomyces_sp._FxanaD5	Streptomyces_sp._UNC401CLCol	Actinospica_acidiphila_NRRL_B_24431	Streptomyces_sp._HNS054	Streptomyces_sp._SMS_SU21	Streptomyces_sp._ZS0098	Streptomyces_thermocarboxydus_K155	Addition	Core-Accessory-Unique	COG	%Similarity_COG	Score_COG	Evalue_COG	KEGG	%Similarity_KEGG	Score_KEGG	Evalue_KEGG
1738	Prokka:JAPCLKMA_00901	sasA_2	Adaptive-response sensory-kinase SasA	1	0	0	0	1	1	0	0	0	0	1	4	Accessory	COG0642_T#	30.4	153	2.8E-36	NA	31.6	117	2.67E-25
1739	Prokka:CIODPCDJ_05300	sasA_16	Adaptive-response sensory-kinase SasA	1	1	1	1	1	1	1	1	1	1	1	11	CORE	COG0642_T#	39.2	228	5.25E-59	K02484	38.9	225	1.19E-57
1740	OSC62869.1	-	citrate:proton symporter	1	1	1	1	1	1	1	1	1	1	1	11	CORE	COG2851_C#	48.4	482	1.54E-135	K03300	58.4	265	1.35E-69
1741	OSC63610.1	-	allantoin permease	0	1	0	0	0	0	0	0	0	0	0	1	Unique	COG1457_F#	25.3	134	1.35E-30	K03457	25.3	134	3.6E-30
1742	Prokka:CIODPCDJ_06704	-	hypothetical protein	0	0	0	0	0	0	0	0	0	0	1	1	Unique	COG1484_L#	35.2	98	8.19E-20	NA	28.7	61	2.27E-08
1743	Prokka:CIODPCDJ_01609	mtaD	5-methylthioadenosine/S-adenosylhomocysteine deaminase	1	1	0	1	1	1	1	1	0	1	1	9	Accessory	COG0402_FR#	37.9	282	3.06E-75	K12960	32.2	221	1.31E-56
1744	OSC65416.1	-	beta-glucosidase	1	1	1	1	1	1	1	1	1	1	1	11	CORE	COG2723_G#	46.4	380	1.08E-104	K01223	37.7	316	3.91E-85
1745	WP_048455009.1	-	amino acid permease	1	1	0	1	1	1	1	1	0	0	1	8	Accessory	COG0531_E#	45	410	9.72E-114	K03294	45	410	2.6E-113
1746	WP_102640886.1	-	GuaB1 family IMP dehydrogenase-related protein	1	1	1	1	1	1	1	1	1	1	1	11	CORE	COG0516_F#	72.7	410	7.44E-114	K00088	64.7	626	3.01E-178

cluster	GI	gene	Annotation	Streptomyces_thermocarboxydus_BPSAC147	Streptomyces_sp._4F	Streptomyces_sp._BSE7F	Streptomyces_sp._ETH9427	Streptomyces_sp._FxanaD5	Streptomyces_sp._UNC401CLCol	Actinospica_acidiphila_NRRL_B_24431	Streptomyces_sp._HNS054	Streptomyces_sp._SMS_SU21	Streptomyces_sp._ZS0098	Streptomyces_thermocarboxydus_K155	Addition	Core-Accessory-Unique	COG	%Similarity_COG	Score_COG	Evalue_COG	KEGG	%Similarity_KEGG	Score_KEGG	Evalue_KEGG
1747	Prokka:JAPCLKMA_00459	-	hypothetical protein	1	1	1	1	1	1	1	1	1	1	1	11	CORE	COG0265_O#	38.9	276	2.19E-73	K08372	37.4	261	1.95E-68
1748	WP_053077044.1	-	hypothetical protein	0	0	0	1	0	0	0	1	1	0	0	3	Accessory								
1749	OSC62449.1	-	ergothioneine biosynthesis glutamate--cysteine ligase EgtA	1	1	0	1	1	1	1	1	1	1	1	10	Accessory	COG3572_H#	36.3	203	2.36E-51	K01919	36.3	203	6.29E-51
1750	PWE09053.1	-	glycine hydroxymethyltransferase	1	1	1	1	1	1	1	1	1	1	1	11	CORE	COG0112_E#	56.8	533	5.83E-151	K00600	48.6	461	1.28E-128
1751	Prokka:CIODPCDJ_01524	-	hypothetical protein	1	1	1	1	1	1	1	1	1	1	1	11	CORE								
1752	WP_106963366.1	-	hypothetical protein	1	0	0	0	0	1	1	0	1	0	1	5	Accessory								
1753	Prokka:CIODPCDJ_03590	-	hypothetical protein	0	0	0	0	0	0	0	0	0	0	1	1	Unique								
1754	Prokka:JAPCLKMA_04404	sasA_13	Adaptive-response sensory-kinase SasA	1	1	1	1	1	1	1	1	1	1	1	11	CORE	COG0642_T#	32.9	188	6.01E-47	K07653	32.9	188	1.6E-46
1755	PWE10825.1	-	hydrolase	0	1	1	1	0	0	1	1	1	1	0	7	Accessory	COG2730_G#	42.4	118	7.63E-26	K01179	42.4	118	2.04E-25
1756	AXI90096.1	-	rhamnulokinase	1	0	1	1	1	1	1	1	1	1	1	10	Accessory	COG1070_G#	40.8	334	5.18E-91	K00848	44.3	399	5.97E-110
1757	Prokka:JAPCLKMA_00581	rodA_1	putative FtsW-like protein	1	1	1	1	1	1	1	1	1	1	1	11	CORE	COG0772_D#	46.7	400	1E-110	NA	46.7	400	2.68E-110
1758	WP_037778176.1	-	sensor histidine kinase	1	1	1	1	1	1	1	1	1	1	1	11	CORE	COG4585_T#	26.6	92	3.43E-18	NA	33.2	91	2.66E-17

cluster	GI	gene	Annotation	Streptomyces_thermocarboxydus_BPSAC147	Streptomyces_sp._4F	Streptomyces_sp._BSE7F	Streptomyces_sp._ETH9427	Streptomyces_sp._FxanaD5	Streptomyces_sp._UNC401CLCol	Actinospica_acidiphila_NRRL_B_24431	Streptomyces_sp._HNS054	Streptomyces_sp._SMS_SU21	Streptomyces_sp._ZS0098	Streptomyces_thermocarboxydus_K155	Addition	Core-Accessory-Unique	COG	%Similarity_COG	Score_COG	Evalue_COG	KEGG	%Similarity_KEGG	Score_KEGG	Evalue_KEGG
1759	Prokka:CIODPCDJ_04209	lpdA	NAD(P)H dehydrogenase (quinone)	1	1	1	1	1	1	1	1	1	1	1	11	CORE	COG1249_C#	55.3	488	3.66E-137	K00382	55.1	487	1.67E-136
1760	WP_026244198.1	-	DNA-binding protein	0	0	0	0	1	1	0	0	0	0	0	2	Accessory	COG3284_QK#	27	69	5.3E-11				
1761	OSC70888.1	-	FAD-binding oxidoreductase	1	1	1	1	1	1	1	1	1	1	1	11	CORE	COG0277_C#	48	396	1.45E-109	K00104	48	396	3.87E-109
1762	WP_102640900.1	-	recombinase family protein	1	0	1	0	0	0	0	0	1	1	0	4	Accessory	COG1961_L#	31.2	177	1.38E-43	NA	31	175	1.07E-42
1763	Prokka:CIODPCDJ_02260	-	hypothetical protein	1	1	0	1	1	1	1	1	1	1	1	10	Accessory								
1764	WP_048458066.1	-	hypothetical protein	0	0	0	1	0	0	0	1	0	0	0	2	Accessory	COG0443_O#	38.7	67	1.18E-10	NA	46.1	107	3.59E-22
1765	Prokka:JAPCLKMA_03708	-	hypothetical protein	1	0	0	0	0	0	0	0	0	0	0	1	Unique	COG1961_L#	29.7	192	3.18E-48	NA	29.7	192	8.49E-48
1766	WP_048455360.1	-	HAMP domain-containing histidine kinase	0	0	0	1	0	0	0	1	0	0	0	2	Accessory	COG0642_T#	37.4	237	8.62E-62	K07653	37.4	237	2.3E-61
1767	OSC71508.1	-	carbohydrate ABC transporter, N-acetylglucosamine/diacetylchitobiose-binding protein	0	1	0	0	0	0	0	0	0	0	0	1	Unique	COG1653_G#	23.3	62	3.8E-09				

cluster	GI	gene	Annotation	Streptomyces_thermocarboxydus_BPSAC147	Streptomyces_sp._4F	Streptomyces_sp._BSE7F	Streptomyces_sp._ETH9427	Streptomyces_sp._FxanaD5	Streptomyces_sp._UNC401CLCol	Actinospica_acidiphila_NRRL_B_24431	Streptomyces_sp._HNS054	Streptomyces_sp._SMS_SU21	Streptomyces_sp._ZS0098	Streptomyces_thermocarboxydus_K155	Addition	Core-Accessory-Unique	COG	%Similarity_COG	Score_COG	Evalue_COG	KEGG	%Similarity_KEGG	Score_KEGG	Evalue_KEGG
1768	WP_109288791.1	-	IS1182 family transposase	0	0	0	0	0	0	0	0	1	0	0	1	Unique	COG3666_L#	54.2	524	3.53E-148	NA	37	310	2.14E-83
1769	Prokka:CIODPCDJ_05968	-	hypothetical protein	1	0	0	0	0	0	0	0	0	0	1	2	Accessory					NA	25.5	48	0.000198
1770	PWE07886.1	glmU	bifunctional UDP-N-acetylglucosamine diphosphorylase/glucosamine-1-phosphate N-acetyltransferase GlmU	1	1	1	1	1	1	1	1	1	1	1	11	CORE	COG1207_M#	60.8	565	2.35E-160	K04042	57.3	513	2.17E-144
1771	WP_122215016.1	eat	ethanolamine permease	1	0	1	1	1	1	1	1	1	1	1	10	Accessory	COG0531_E#	58.8	561	2.6E-159	NA	32.5	232	9.65E-60
1772	WP_019523317.1	-	aldehyde dehydrogenase family protein	1	0	1	1	1	1	1	1	1	1	1	10	Accessory	COG1012_C#	39.9	339	1.61E-92	NA	39.9	339	4.29E-92
1773	WP_102637039.1	-	glyceraldehyde-3-phosphate dehydrogenase	1	1	1	1	1	1	1	1	1	1	1	11	CORE	COG0057_G#	61.1	587	3.39E-167	K05298	38.5	233	4.33E-60
1774	Prokka:CIODPCDJ_00732	xylB_1	Xylulose kinase	1	1	1	1	1	1	1	1	1	1	1	11	CORE	COG1070_G#	45	338	4.67E-92	K00854	32.8	211	1.35E-53
1775	AXI86691.1	obgE	GTPase ObgE	1	1	1	1	1	1	1	1	1	1	1	11	CORE	COG0536_R#	60.4	560	5.79E-159	K03979	60.4	560	1.55E-158

cluster	GI	gene	Annotation	Streptomyces_thermocarboxydus_BPSAC147	Streptomyces_sp._4F	Streptomyces_sp._BSE7F	Streptomyces_sp._ETH9427	Streptomyces_sp._FxanaD5	Streptomyces_sp._UNC401CLCol	Actinospica_acidiphila_NRRL_B_24431	Streptomyces_sp._HNS054	Streptomyces_sp._SMS_SU21	Streptomyces_sp._ZS0098	Streptomyces_thermocarboxydus_K155	Addition	Core-Accessory-Unique	COG	%Similarity_COG	Score_COG	Evalue_COG	KEGG	%Similarity_KEGG	Score_KEGG	Evalue_KEGG
1776	OSC69285.1	-	hydrogenase expression protein	0	1	0	0	0	0	1	0	0	0	0	2	Accessory								
1777	WP_102640319.1	-	VWA domain-containing protein	1	1	1	1	1	1	1	1	1	1	1	11	CORE					NA	27.9	145	1.19E-33
1778	OSC60976.1	-	phosphoribosylamine--glycine ligase	1	1	1	1	1	1	1	1	1	1	1	11	CORE								
1779	Prokka:JAPCLKMA_02833	-	hypothetical protein	1	0	0	0	0	0	0	0	0	0	0	1	Unique	COG1192_D#	40.5	51	0.0000114	K03496	26.1	51	0.0000305
1780	WP_019524858.1	-	MFS transporter	0	0	0	0	1	1	0	0	0	0	1	3	Accessory	COG0477_GEPR#	31.1	194	1.09E-48	K08169	27.2	152	9.71E-36
1781	WP_033273875.1	-	MFS transporter	1	0	0	0	0	0	1	0	1	0	1	4	Accessory	COG0477_GEPR#	37.1	255	3.05E-67	NA	34.2	241	1.59E-62
1782	Prokka:CIODPCDJ_00299	-	hypothetical protein	1	1	1	1	1	1	1	1	1	1	1	11	CORE					NA	29.8	71	2.85E-11
1783	OSC69923.1	-	argininosuccinate synthase	0	1	0	0	0	0	0	0	0	0	0	1	Unique	COG0137_E#	67.2	612	9.83E-175	K01940	67.2	612	2.63E-174
1784	PWE08578.1	-	transcriptional regulator	0	0	1	0	0	0	0	0	0	0	0	1	Unique								
1785	WP_033276954.1	-	adenylosuccinate lyase	1	1	1	1	1	1	1	1	1	1	1	11	CORE	COG0015_F#	75.8	685	1.19E-196	K01756	74.7	683	1.58E-195
1786	WP_028959210.1	desA	lysine decarboxylase DesA	1	1	0	1	1	1	1	1	1	1	1	10	Accessory	COG0076_E#	53.2	517	5.61E-146	K01580	30.2	199	6.91E-50

cluster	GI	gene	Annotation	Streptomyces_thermocarboxydus_BPSAC147	Streptomyces_sp._4F	Streptomyces_sp._BSE7F	Streptomyces_sp._ETH9427	Streptomyces_sp._FxanaD5	Streptomyces_sp._UNC401CLCol	Actinospica_acidiphila_NRRL_B_24431	Streptomyces_sp._HNS054	Streptomyces_sp._SMS_SU21	Streptomyces_sp._ZS0098	Streptomyces_thermocarboxydus_K155	Addition	Core-Accessory-Unique	COG	%Similarity_COG	Score_COG	Evalue_COG	KEGG	%Similarity_KEGG	Score_KEGG	Evalue_KEGG
1787	WP_048459270.1	-	cation:dicarboxylase symporter family transporter	1	1	1	1	1	1	1	1	1	1	1	11	CORE	COG1301_C#	60.1	543	9.55E-154	K11103	60.1	543	2.55E-153
1788	AXI91308.1	-	hypothetical protein	0	0	0	1	0	0	0	0	0	0	0	1	Unique	COG1475_K#	30.2	80	1.34E-14	K03497	30	77	5.18E-13
1789	AXI85454.1	-	indole acetimide hydrolase	0	0	1	1	1	1	1	1	1	1	0	8	Accessory	COG0154_J#	36.5	234	7.26E-61	K01426	34.1	214	2.08E-54
1790	PWE09229.1	-	amidase	1	1	1	1	1	1	1	1	1	1	1	11	CORE	COG0154_J#	48.5	389	1.76E-107	K01426	47.2	377	1.85E-103
1791	AXI85385.1	-	MFS transporter	1	1	1	1	1	1	1	1	1	1	1	11	CORE	COG0477_GEPR#	43.6	344	4.98E-94	NA	41	330	3.39E-89
1792	WP_052183389.1	-	MFS transporter	0	0	0	0	0	1	0	0	0	0	0	1	Unique	COG0477_GEPR#	41.6	339	2.73E-92	K08166	36.4	285	7.34E-76
1793	Prokka:CIODPCDJ_01714	murD	UDP-N-acetylmuramoylalanine--D-glutamate ligase	1	1	1	1	1	1	1	1	1	1	1	11	CORE	COG0771_M#	46.1	359	1.96E-98	K01925	46.1	359	5.22E-98
1794	OSC70796.1	-	histidine kinase	1	1	1	1	1	1	1	1	1	1	1	11	CORE	COG0642_T#	28.6	143	2.88E-33	K07642	28.5	143	7.68E-33
1795	WP_033276284.1	-	acyl--CoA ligase	0	0	0	0	0	0	1	0	0	0	0	1	Unique	COG0318_IQ#	28.5	156	1.92E-37	K00666	28.5	156	5.14E-37
1796	OSC56356.1	-	hypothetical protein	0	1	0	0	0	0	0	0	0	0	0	1	Unique								
1797	WP_033275939.1	-	gamma-aminobutyraldehyde dehydrogenase	1	1	1	1	1	1	1	1	1	1	1	11	CORE	COG1012_C#	49.6	473	7.1E-133	K00137	49.1	462	4.37E-129
1798	WP_122217104.1	-	hypothetical protein	1	1	1	1	1	1	1	1	1	1	1	11	CORE								

cluster	GI	gene	Annotation	Streptomyces_thermocarboxydus_BPSAC147	Streptomyces_sp._4F	Streptomyces_sp._BSE7F	Streptomyces_sp._ETH9427	Streptomyces_sp._FxanaD5	Streptomyces_sp._UNC401CLCol	Actinospica_acidiphila_NRRL_B_24431	Streptomyces_sp._HNS054	Streptomyces_sp._SMS_SU21	Streptomyces_sp._ZS0098	Streptomyces_thermocarboxydus_K155	Addition	Core-Accessory-Unique	COG	%Similarity_COG	Score_COG	Evalue_COG	KEGG	%Similarity_KEGG	Score_KEGG	Evalue_KEGG
1799	WP_019521393.1	gndA	NADP-dependent phosphogluconate dehydrogenase	0	0	1	1	1	1	1	1	1	1	1	9	Accessory	COG0362_G#	65.8	640	5.73E-183	K00033	65.5	636	2.88E-181
1800	WP_019521466.1	-	MFS transporter	1	0	0	0	1	1	0	0	0	0	1	4	Accessory	COG0477_GEPR#	38.3	314	5.51E-85	K05819	32.1	180	3.32E-44
1801	WP_019522464.1	-	MFS transporter	1	0	1	1	1	1	1	1	1	1	1	10	Accessory	COG0477_GEPR#	40.1	310	1.04E-83	NA	33.1	256	4.76E-67
1802	OSC70335.1	-	cytochrome P450	0	1	0	0	0	0	0	0	0	0	0	1	Unique	COG2124_Q#	29.4	210	1.46E-53	K00517	26.6	148	1.82E-34
1803	OSC65271.1	-	diaminopimelate decarboxylase	1	1	1	1	1	1	1	1	1	1	1	11	CORE	COG0019_E#	31.8	164	1.2E-39	K01586	31.8	164	3.21E-39
1804	WP_033273634.1	-	hypothetical protein	0	0	0	0	0	0	1	0	0	0	0	1	Unique								
1805	Prokka:JAPCLKMA_06686	-	hypothetical protein	1	0	1	1	1	1	1	1	1	1	1	10	Accessory	COG1680_V#	29.9	118	5.78E-26	NA	25.9	50	0.0000675
1806	WP_102638152.1	pyk	pyruvate kinase	1	1	1	1	1	1	1	1	1	1	1	11	CORE	COG0469_G#	61.4	562	1.16E-159	K00873	61.4	562	3.1E-159
1807	OSC73283.1	-	MFS transporter	0	1	0	0	0	0	0	0	0	0	0	1	Unique	COG0477_GEPR#	33.8	216	1.56E-55	NA	29.8	206	7.36E-52
1808	WP_048455944.1	-	aldehyde dehydrogenase family protein	1	1	1	1	1	1	1	1	1	1	1	11	CORE	COG1012_C#	56.7	545	1.92E-154	K00128	48.1	444	1.61E-123
1809	PWE09414.1	atpD	F0F1 ATP synthase subunit beta	1	1	1	1	1	1	1	1	1	1	1	11	CORE	COG0055_C#	69	651	1.9E-186	K02112	69	651	5.07E-186

cluster	GI	gene	Annotation	Streptomyces_thermocarboxydus_BPSAC147	Streptomyces_sp._4F	Streptomyces_sp._BSE7F	Streptomyces_sp._ETH9427	Streptomyces_sp._FxanaD5	Streptomyces_sp._UNC401CLCol	Actinospica_acidiphila_NRRL_B_24431	Streptomyces_sp._HNS054	Streptomyces_sp._SMS_SU21	Streptomyces_sp._ZS0098	Streptomyces_thermocarboxydus_K155	Addition	Core-Accessory-Unique	COG	%Similarity_COG	Score_COG	Evalue_COG	KEGG	%Similarity_KEGG	Score_KEGG	Evalue_KEGG
1810	OSC57476.1	-	glucose/sorbosone dehydrogenase-like protein	0	1	0	0	0	0	0	0	0	0	0	1	Unique	COG2133_G#	30.4	114	8.35E-25	NA	38.5	230	4.76E-59
1811	WP_019527716.1	-	DNA phosphorothioation-associated putative methyltransferase	0	0	0	0	1	0	0	0	0	0	0	1	Unique	COG0500_QR#	39.1	323	1.18E-87				
1812	WP_019523564.1	-	aldehyde dehydrogenase family protein	1	0	0	0	1	1	0	0	0	0	1	4	Accessory	COG1012_C#	47.2	391	3.54E-108	K00128	42.6	375	7.01E-103
1813	Prokka:CIODPCDJ_06802	-	hypothetical protein	0	0	0	0	1	0	0	0	0	0	1	2	Accessory								
1814	WP_102639438.1	-	PLP-dependent aminotransferase family protein	1	1	0	1	1	1	1	1	1	1	1	10	Accessory	COG1167_KE#	31.4	176	2.34E-43	K00375	34.8	175	1.06E-42
1815	WP_102640449.1	-	PLP-dependent aminotransferase family protein	1	1	0	1	1	1	1	1	1	1	1	10	Accessory	COG1167_KE#	37.3	264	8.52E-70	K00375	39.3	247	2.88E-64
1816	OSC57123.1	-	phytoene dehydrogenase	1	1	1	1	1	1	1	1	1	1	1	11	CORE	COG1233_Q#	28.9	85	7.09E-16	NA	28.9	85	1.89E-15
1817	OSC68521.1	-	hypothetical protein	0	1	0	0	0	0	0	1	0	0	0	2	Accessory								

cluster	GI	gene	Annotation	Streptomyces_thermocarboxydus_BPSAC147	Streptomyces_sp._4F	Streptomyces_sp._BSE7F	Streptomyces_sp._ETH9427	Streptomyces_sp._FxanaD5	Streptomyces_sp._UNC401CLCol	Actinospica_acidiphila_NRRL_B_24431	Streptomyces_sp._HNS054	Streptomyces_sp._SMS_SU21	Streptomyces_sp._ZS0098	Streptomyces_thermocarboxydus_K155	Addition	Core-Accessory-Unique	COG	%Similarity_COG	Score_COG	Evalue_COG	KEGG	%Similarity_KEGG	Score_KEGG	Evalue_KEGG
1818	OSC70485.1	-	nitrate reductase	1	1	1	1	1	1	1	1	1	1	1	11	CORE	COG2233_F#	47.4	386	1.14E-106	K09016	43.4	340	2.5E-92
1819	Prokka:CIODPCDJ_05920	-	hypothetical protein	1	1	1	1	1	1	1	1	1	1	1	11	CORE								
1820	AXI86824.1	-	aminopeptidase	1	1	1	1	1	1	1	1	1	1	1	11	CORE					NA	33.4	228	1.8E-58
1821	WP_033275984.1	-	transporter	1	0	0	1	1	1	1	1	0	1	1	8	Accessory	COG1457_F#	35.9	283	1.77E-75	K03457	24.1	105	1.35E-21
1822	OSC66333.1	-	MFS transporter	0	1	0	0	0	0	0	0	0	0	0	1	Unique	COG0477_GEPR#	62.1	564	3.98E-160	K06609	62.1	564	1.06E-159
1823	WP_033276659.1	-	recombinase family protein	0	0	0	0	0	0	1	0	0	0	0	1	Unique	COG1961_L#	28.8	174	6.79E-43	NA	28.8	174	1.81E-42
1824	WP_028961316.1	-	hypothetical protein	0	0	0	0	0	1	0	0	0	0	0	1	Unique								
1825	WP_122215386.1	-	D-alanyl-D-alanine carboxypeptidase	1	0	1	1	1	1	1	1	1	1	1	10	Accessory	COG1686_M#	31.4	165	5.37E-40	K07258	31.1	164	2.44E-39
1826	PWE06657.1	argH	argininosuccinate lyase	1	1	1	1	1	1	1	1	1	1	1	11	CORE	COG0165_E#	64.7	596	9.42E-170	K01755	64.7	596	3.29E-169
1827	WP_122215749.1	-	OFA family MFS transporter	1	1	1	1	1	1	1	1	1	1	1	11	CORE	COG0477_GEPR#	27	172	4.39E-42	K08177	25.8	109	1.22E-22
1828	WP_048460075.1	-	MFS transporter	1	1	1	1	1	1	1	1	1	1	1	11	CORE	COG0477_GEPR#	37.1	245	5.33E-64	NA	37.1	245	1.42E-63
1829	Prokka:CIODPCDJ_06117	-	Putative diacylglycerol O-acyltransferase	1	1	1	1	1	1	1	1	1	1	1	11	CORE					NA	27.9	143	4.47E-33

cluster	GI	gene	Annotation	Streptomyces_thermocarboxydus_BPSAC147	Streptomyces_sp._4F	Streptomyces_sp._BSE7F	Streptomyces_sp._ETH9427	Streptomyces_sp._FxanaD5	Streptomyces_sp._UNC401CLCol	Actinospica_acidiphila_NRRL_B_24431	Streptomyces_sp._HNS054	Streptomyces_sp._SMS_SU21	Streptomyces_sp._ZS0098	Streptomyces_thermocarboxydus_K155	Addition	Core-Accessory-Unique	COG	%Similarity_COG	Score_COG	Evalue_COG	KEGG	%Similarity_KEGG	Score_KEGG	Evalue_KEGG
1830	OSC71129.1	-	MFS transporter	0	1	0	0	0	0	0	0	0	0	0	1	Unique	COG0477_GEPR#	45.9	366	1.59E-100	NA	34	196	4.44E-49
1831	WP_122216925.1	-	UDP-N-acetylmuramoyl-tripeptide--D-alanyl-D-alanine ligase	1	1	1	1	1	1	1	1	1	1	1	11	CORE	COG0770_M#	49.9	436	1.63E-121	K01929	49.9	436	4.35E-121
1832	WP_033275878.1	leuC	3-isopropylmalate dehydratase large subunit	1	1	1	1	1	1	1	1	1	1	1	11	CORE	COG0065_E#	75.2	728	1.22E-209	K01703	73.9	715	3.72E-205
1833	Prokka:CIODPCDJ_01886	-	hypothetical protein	0	1	0	0	0	0	1	1	0	0	1	4	Accessory					NA	32.4	45	0.00216
1834	OSC61623.1	-	FAD-dependent oxidoreductase	0	1	0	0	0	0	0	0	0	0	0	1	Unique	COG1249_C#	44.6	59	3.17E-08	K00382	47.4	54	0.00000272
1835	WP_033275113.1	-	rRNA cytosine-C5-methyltransferase	1	1	1	1	1	1	1	1	1	1	1	11	CORE	COG0144_J#	54.4	448	3.17E-125	K03500	54.4	448	8.46E-125
1836	WP_102639704.1	-	hypothetical protein	0	0	1	1	0	0	1	1	1	1	0	6	Accessory								
1837	WP_033277543.1	-	extracellular solute-binding protein	1	1	1	1	1	1	1	1	1	1	1	11	CORE	COG1653_G#	34.5	228	6.73E-59	K10232	34.5	228	1.8E-58
1838	OSC64089.1	proY	proline-specific permease ProY	1	1	1	1	1	1	1	1	1	1	1	11	CORE	COG1113_E#	47.9	465	1.92E-130	K03293	48.6	453	2.01E-126

cluster	GI	gene	Annotation	Streptomyces_thermocarboxydus_BPSAC147	Streptomyces_sp._4F	Streptomyces_sp._BSE7F	Streptomyces_sp._ETH9427	Streptomyces_sp._FxanaD5	Streptomyces_sp._UNC401CLCol	Actinospica_acidiphila_NRRL_B_24431	Streptomyces_sp._HNS054	Streptomyces_sp._SMS_SU21	Streptomyces_sp._ZS0098	Streptomyces_thermocarboxydus_K155	Addition	Core-Accessory-Unique	COG	%Similarity_COG	Score_COG	Evalue_COG	KEGG	%Similarity_KEGG	Score_KEGG	Evalue_KEGG
1839	OSC72624.1	-	FAD-dependent oxidoreductase	1	1	0	1	1	1	1	1	1	1	1	10	Accessory	COG0665_E#	30.8	204	1.04E-51				
1840	OSC61082.1	-	phenolic acid decarboxylase	0	1	0	0	0	0	0	0	0	0	0	1	Unique	COG0043_H#	72.8	744	2.79E-214	NA	61.6	616	1.8E-175
1841	OSC70777.1	-	glutamate decarboxylase	1	1	1	1	1	1	1	1	1	1	1	11	CORE	COG0076_E#	60.4	558	2.17E-158	K01580	60.4	558	5.8E-158
1842	AXI87130.1	-	peptidoglycan-binding protein	0	0	0	1	0	0	0	1	1	0	0	3	Accessory								
1843	WP_102641565.1	-	MFS transporter	1	1	1	1	1	1	1	1	1	1	1	11	CORE								
1844	WP_028959249.1	-	exopolysaccharide biosynthesis polyprenyl glycosylphosphotransferase	1	1	1	1	1	1	1	1	1	1	1	11	CORE	COG2148_M#	33.2	151	6.11E-36	K03606	41.8	141	2.21E-32
1845	Prokka:CIODPCDJ_00478	-	hypothetical protein	1	1	0	1	1	1	1	1	1	1	1	10	Accessory	COG2202_T#	33.9	82	3.49E-15	NA	27.3	50	0.0000392
1846	WP_052183382.1	-	hypothetical protein	0	0	0	0	0	1	0	0	0	0	0	1	Unique								
1847	Prokka:JAPCLKMA_04759	-	Ferredoxin--NADP reductase	1	1	1	1	1	1	1	1	1	1	1	11	CORE	COG1232_H#	33.8	44	0.0018	K00231	33.8	44	0.00479
1848	Prokka:CIODPCDJ_06611	-	hypothetical protein	0	0	0	0	1	1	1	1	1	1	1	7	Accessory								

cluster	GI	gene	Annotation	Streptomyces_thermocarboxydus_BPSAC147	Streptomyces_sp._4F	Streptomyces_sp._BSE7F	Streptomyces_sp._ETH9427	Streptomyces_sp._FxanaD5	Streptomyces_sp._UNC401CLCol	Actinospica_acidiphila_NRRL_B_24431	Streptomyces_sp._HNS054	Streptomyces_sp._SMS_SU21	Streptomyces_sp._ZS0098	Streptomyces_thermocarboxydus_K155	Addition	Core-Accessory-Unique	COG	%Similarity_COG	Score_COG	Evalue_COG	KEGG	%Similarity_KEGG	Score_KEGG	Evalue_KEGG
1849	WP_122216251.1	-	NADH-quinone oxidoreductase subunit N	0	0	0	1	0	0	0	1	1	1	0	4	Accessory	COG1007_C#	33.7	248	3.67E-65	K00343	32.6	221	1.68E-56
1850	WP_122216352.1	-	NAD-dependent succinate-semialdehyde dehydrogenase	0	0	0	0	0	0	0	0	1	1	0	2	Accessory	COG1012_C#	63.6	584	2.83E-166	K00135	63.6	584	7.55E-166
1851	WP_102638306.1	-	maltokinase	1	1	1	1	1	1	1	1	1	1	1	11	CORE	COG3281_G#	42.7	255	3.93E-67	K16146	42.7	255	1.05E-66
1852	WP_033273851.1	-	NAD(P)/FAD-dependent oxidoreductase	0	0	0	0	0	0	1	0	0	0	1	2	Accessory	COG1233_Q#	54	518	3.25E-146	NA	54	518	8.67E-146
1853	OSC67163.1	-	ABC transporter substrate-binding protein	1	1	1	1	1	1	1	1	1	1	1	11	CORE	COG1653_G#	23.3	67	1.52E-10				
1854	Prokka:CIODPCDJ_05509	tam_6	Trans-aconitate 2-methyltransferase	1	0	1	1	1	1	1	1	1	1	1	10	Accessory	COG0500_QR#	37.3	62	4.88E-09	K00568	28.6	56	7.15E-07
1855	WP_019523628.1	-	amino acid permease	1	1	1	1	1	1	1	1	1	1	1	11	CORE	COG0531_E#	41.6	316	1.43E-85	K03759	34.3	254	1.78E-66
1856	WP_048458426.1	sufB	Fe-S cluster assembly protein SufB	1	1	1	1	1	1	1	1	1	1	1	11	CORE	COG0719_O#	84	793	4E-229	K09014	64.8	632	4.11E-180

cluster	GI	gene	Annotation	Streptomyces_thermocarboxydus_BPSAC147	Streptomyces_sp._4F	Streptomyces_sp._BSE7F	Streptomyces_sp._ETH9427	Streptomyces_sp._FxanaD5	Streptomyces_sp._UNC401CLCol	Actinospica_acidiphila_NRRL_B_24431	Streptomyces_sp._HNS054	Streptomyces_sp._SMS_SU21	Streptomyces_sp._ZS0098	Streptomyces_thermocarboxydus_K155	Addition	Core-Accessory-Unique	COG	%Similarity_COG	Score_COG	Evalue_COG	KEGG	%Similarity_KEGG	Score_KEGG	Evalue_KEGG
1857	OSC66318.1	-	S-adenosylhomocysteine deaminase	0	1	0	0	0	0	0	0	0	0	0	1	Unique	COG0402_FR#	59.6	546	8.51E-155				
1858	WP_033275459.1	-	lipase maturation factor family protein	1	1	1	1	1	1	1	1	1	1	1	11	CORE					NA	61.7	631	7.01E-180
1859	WP_106432555.1	-	Cys-Gln thioester bond-forming surface protein	1	1	1	1	1	1	1	1	1	1	1	11	CORE	COG3511_M#	31.4	61	8.3E-09				
1860	OSC71776.1	-	Tat pathway signal sequence domain protein	0	1	0	0	0	0	0	0	0	0	0	1	Unique								
1861	WP_028961281.1	-	NAD(P)-binding protein	0	0	0	0	0	1	0	0	0	0	0	1	Unique	COG0493_ER#	46.9	45	0.000616	NA	30.5	212	1.02E-53
1862	WP_102637882.1	-	hypothetical protein	1	0	0	1	1	1	1	1	1	1	1	9	Accessory	COG2340_S#	43.6	122	3.03E-27	NA	43.6	122	8.1E-27
1863	OSC69279.1	-	hypothetical protein	0	1	0	0	0	0	0	0	0	0	0	1	Unique	COG0477_GEPR#	23.3	84	9.16E-16				
1864	OSC57753.1	-	ABC transporter substrate-binding protein	0	1	0	0	0	0	0	0	0	0	0	1	Unique	COG0747_E#	30.2	164	1.19E-39	K02035	30.2	164	3.17E-39
1865	WP_109288623.1	-	FHA domain-containing protein	1	1	1	1	1	1	1	1	1	1	1	11	CORE	COG2931_Q#	32.5	76	2.49E-13	NA	36.3	175	1.38E-42

cluster	GI	gene	Annotation	Streptomyces_thermocarboxydus_BPSAC147	Streptomyces_sp._4F	Streptomyces_sp._BSE7F	Streptomyces_sp._ETH9427	Streptomyces_sp._FxanaD5	Streptomyces_sp._UNC401CLCol	Actinospica_acidiphila_NRRL_B_24431	Streptomyces_sp._HNS054	Streptomyces_sp._SMS_SU21	Streptomyces_sp._ZS0098	Streptomyces_thermocarboxydus_K155	Addition	Core-Accessory-Unique	COG	%Similarity_COG	Score_COG	Evalue_COG	KEGG	%Similarity_KEGG	Score_KEGG	Evalue_KEGG
1866	OSC72417.1	-	hypothetical protein	0	1	0	0	0	0	0	0	0	0	0	1	Unique	COG0477_GEPR#	41.3	351	6.85E-96	NA	41.3	351	1.83E-95
1867	Prokka:JAPCLKMA_06814	-	hypothetical protein	1	0	0	1	0	0	0	0	0	0	0	2	Accessory	COG0477_GEPR#	37.6	186	1.71E-46	NA	37.6	186	4.57E-46
1868	WP_019525928.1	-	2-oxo acid dehydrogenase subunit E2	1	1	1	1	1	1	1	1	1	1	1	11	CORE	COG0508_C#	38.3	267	7.61E-71	K00627	33	228	1.37E-58
1869	WP_122216269.1	-	hypothetical protein	0	0	0	0	0	0	0	0	0	1	0	1	Unique								
1870	OSC56256.1	-	hypothetical protein	1	1	1	1	1	1	1	1	1	1	1	11	CORE								
1871	WP_026243587.1	-	sugar porter family MFS transporter	1	1	1	1	1	1	1	1	1	1	1	11	CORE	COG0477_GEPR#	50.9	437	5.55E-122	K08139	50.9	437	1.48E-121
1872	WP_122213319.1	-	hypothetical protein	1	0	0	0	1	1	1	0	0	1	1	6	Accessory	COG3459_G#	32.3	52	0.00000385	NA	32.3	52	0.0000103
1873	Prokka:JAPCLKMA_02737	-	hypothetical protein	1	0	0	0	0	0	0	0	0	0	0	1	Unique	COG1337_L#	41.9	46	0.00036				
1874	Prokka:JAPCLKMA_03547	radA_2	DNA repair protein RadA	1	0	1	1	1	1	1	1	1	1	1	10	Accessory	COG1066_O#	56.5	483	8.83E-136	K04485	56.5	483	2.36E-135
1875	Prokka:CIODPCDJ_05532	thi4_5	Thiamine thiazole synthase	1	1	1	1	1	1	1	1	1	1	1	11	CORE	COG1233_Q#	48.7	423	1.42E-117	NA	48.7	423	3.78E-117
1876	AXI89645.1	-	ATP-binding protein	1	0	0	1	0	0	0	0	0	0	0	2	Accessory								

cluster	GI	gene	Annotation	Streptomyces_thermocarboxydus_BPSAC147	Streptomyces_sp._4F	Streptomyces_sp._BSE7F	Streptomyces_sp._ETH9427	Streptomyces_sp._FxanaD5	Streptomyces_sp._UNC401CLCol	Actinospica_acidiphila_NRRL_B_24431	Streptomyces_sp._HNS054	Streptomyces_sp._SMS_SU21	Streptomyces_sp._ZS0098	Streptomyces_thermocarboxydus_K155	Addition	Core-Accessory-Unique	COG	%Similarity_COG	Score_COG	Evalue_COG	KEGG	%Similarity_KEGG	Score_KEGG	Evalue_KEGG
1877	WP_122213123.1	rfaE2	D-glycero-beta-D-manno-heptose 1-phosphate adenylyltransferase	1	1	1	1	1	1	1	1	1	1	1	11	CORE	COG2870_M#	37	240	1.3E-62	K03272	35.6	235	1.46E-60
1878	Prokka:JAPCLKMA_02526	-	hypothetical protein	1	1	1	1	0	0	1	1	1	1	0	8	Accessory	COG4585_T#	26.9	107	1.72E-22	NA	26.9	107	4.59E-22
1879	WP_051101579.1	-	allantoin permease	1	0	0	0	1	1	0	0	0	0	1	4	Accessory	COG1457_F#	55.3	528	2.39E-149	K03457	36.5	310	2.73E-83
1880	WP_048455179.1	-	aspartate aminotransferase family protein	1	1	1	1	1	1	1	1	1	1	1	11	CORE	COG4992_E#	44.9	278	5.62E-74	K09251	40.8	219	8.27E-56
1881	WP_048459026.1	-	M1 family metallopeptidase	1	1	1	1	1	1	1	1	1	1	1	11	CORE	COG0308_E#	33	214	7.63E-55	NA	32.1	195	1.28E-48
1882	Prokka:CIODPCDJ_00468	-	hypothetical protein	1	1	1	1	1	1	1	1	1	1	1	11	CORE								
1883	Prokka:JAPCLKMA_00381	frc_1	Formyl-CoA:oxalate CoA-transferase	1	0	1	1	1	1	1	1	1	1	1	10	Accessory	COG1804_C#	41.4	295	2.6E-79	NA	32.4	112	8.35E-24
1884	OSC65725.1	-	MFS transporter	1	1	1	1	1	1	1	1	1	1	1	11	CORE	COG0477_GEPR#	56.9	467	5E-131	NA	56.9	467	1.34E-130
1885	Prokka:CIODPCDJ_05368	-	hypothetical protein	1	1	0	1	1	1	1	1	1	1	1	10	Accessory	COG4320_S#	27.6	140	1.4E-32				
1886	OSC69600.1	-	uracil permease	1	1	1	1	1	1	1	1	1	1	1	11	CORE	COG2233_F#	50.7	426	1.28E-118	NA	46.3	408	7.36E-113
1887	OSC64637.1	-	hypothetical protein	1	1	1	1	1	1	1	1	1	1	1	11	CORE	COG0174_E#	65	373	1.28E-102	K01915	65	373	3.43E-102

cluster	GI	gene	Annotation	Streptomyces_thermocarboxydus_BPSAC147	Streptomyces_sp._4F	Streptomyces_sp._BSE7F	Streptomyces_sp._ETH9427	Streptomyces_sp._FxanaD5	Streptomyces_sp._UNC401CLCol	Actinospica_acidiphila_NRRL_B_24431	Streptomyces_sp._HNS054	Streptomyces_sp._SMS_SU21	Streptomyces_sp._ZS0098	Streptomyces_thermocarboxydus_K155	Addition	Core-Accessory-Unique	COG	%Similarity_COG	Score_COG	Evalue_COG	KEGG	%Similarity_KEGG	Score_KEGG	Evalue_KEGG
1888	Prokka:JAPCLKMA_05689	smc_11	Chromosome partition protein Smc	1	0	0	0	0	0	0	0	0	0	0	1	Unique	COG4637_R#	25.5	71	1.04E-11				
1889	OSC71996.1	-	NAD-dependent malic enzyme	1	1	1	1	1	1	1	1	1	1	1	11	CORE	COG0281_C#	53.5	487	6.1E-137	K00027	52	469	3.52E-131
1890	PWE10016.1	-	hypothetical protein	1	0	1	1	1	1	1	1	1	1	1	10	Accessory	COG2203_T#	49	186	1.7E-46	NA	49	186	4.54E-46
1891	WP_028958872.1	-	proline--tRNA ligase	1	0	0	1	1	1	1	1	0	1	1	8	Accessory	COG0442_J#	48.5	448	3.13E-125	K01881	47.8	453	1.99E-126
1892	WP_102639365.1	-	diacylglycerol kinase	1	1	1	1	1	1	1	1	1	1	1	11	CORE	COG1597_IR#	29.8	81	7.7E-15				
1893	WP_086020177.1	-	NADP-specific glutamate dehydrogenase	1	1	1	1	1	1	1	1	1	1	1	11	CORE	COG0334_E#	67.7	617	2.99E-176	K00262	58	522	4.56E-147
1894	PWE09289.1	-	dipeptidase	1	1	1	1	1	1	1	1	1	1	1	11	CORE	COG0624_E#	42.2	373	9.81E-103	NA	44.9	367	1.44E-100
1895	WP_048454963.1	-	PepSY domain-containing protein	0	0	1	1	0	0	0	1	1	1	0	5	Accessory	COG3182_S#	40	283	1.02E-75				
1896	Prokka:JAPCLKMA_00756	yagG	Putative glycoside/cation symporter YagG	1	1	0	0	1	1	1	0	0	0	1	6	Accessory	COG2211_G#	22.7	87	1.83E-16	K03292	22.8	89	1.29E-16
1897	WP_037778355.1	-	MFS transporter	0	0	0	0	1	1	0	0	0	0	1	3	Accessory	COG0477_GEPR#	36.8	249	2.14E-65	NA	35.7	238	1.01E-61

cluster	GI	gene	Annotation	Streptomyces_thermocarboxydus_BPSAC147	Streptomyces_sp._4F	Streptomyces_sp._BSE7F	Streptomyces_sp._ETH9427	Streptomyces_sp._FxanaD5	Streptomyces_sp._UNC401CLCol	Actinospica_acidiphila_NRRL_B_24431	Streptomyces_sp._HNS054	Streptomyces_sp._SMS_SU21	Streptomyces_sp._ZS0098	Streptomyces_thermocarboxydus_K155	Addition	Core-Accessory-Unique	COG	%Similarity_COG	Score_COG	Evalue_COG	KEGG	%Similarity_KEGG	Score_KEGG	Evalue_KEGG
1898	Prokka:CIODPCDJ_01736	ddmA1_1	Dicamba O-demethylase 1, ferredoxin reductase component	1	1	1	1	1	1	1	1	1	1	1	11	CORE	COG0446_R#	37.3	250	1.64E-65	K00529	37.3	250	4.37E-65
1899	Prokka:CIODPCDJ_02660	emrY_2	putative multidrug resistance protein EmrY	0	0	0	0	1	1	1	0	0	0	1	4	Accessory	COG0477_GEPR#	34.9	280	1.48E-74	NA	34.9	280	3.94E-74
1900	WP_106432563.1	-	NAD(P)/FAD-dependent oxidoreductase	1	1	1	1	1	1	1	1	1	1	1	11	CORE	COG1252_C#	27.7	183	1.88E-45	K03885	28.7	158	1.73E-37
1901	WP_159020850.1	-	hypothetical protein	0	0	1	1	0	0	0	1	0	0	0	3	Accessory								
1902	OSC70627.1	-	ATP/GTP-binding protein	1	1	1	1	1	1	1	1	1	1	1	11	CORE								
1903	WP_026243865.1	-	HAMP domain-containing histidine kinase	1	1	1	1	1	1	1	1	1	1	1	11	CORE	COG0642_T#	29.4	124	7.92E-28	K07640	28.2	103	6.59E-21
1904	WP_107080816.1	ngcE	carbohydrate ABC transporter, N-acetylglucosamine/diacetylchitobiose-binding protein	1	0	1	1	1	1	1	1	1	1	1	10	Accessory	COG1653_G#	22.9	50	0.0000145				

cluster	GI	gene	Annotation	Streptomyces_thermocarboxydus_BPSAC147	Streptomyces_sp._4F	Streptomyces_sp._BSE7F	Streptomyces_sp._ETH9427	Streptomyces_sp._FxanaD5	Streptomyces_sp._UNC401CLCol	Actinospica_acidiphila_NRRL_B_24431	Streptomyces_sp._HNS054	Streptomyces_sp._SMS_SU21	Streptomyces_sp._ZS0098	Streptomyces_thermocarboxydus_K155	Addition	Core-Accessory-Unique	COG	%Similarity_COG	Score_COG	Evalue_COG	KEGG	%Similarity_KEGG	Score_KEGG	Evalue_KEGG
1905	WP_048456730.1	glnA	type I glutamate--ammonia ligase	1	1	1	1	1	1	1	1	1	1	1	11	CORE	COG0174_E#	70.3	712	1.16E-204	K01915	71.4	711	4.05E-204
1906	Prokka:CIODPCDJ_01679	hisD	Histidinol dehydrogenase	1	1	1	1	1	1	1	1	1	1	1	11	CORE	COG0141_E#	64.4	531	2.81E-150	K00013	64.2	530	1.67E-149
1907	WP_102640664.1	-	recombinase family protein	0	0	0	0	0	0	0	0	1	0	0	1	Unique	COG1961_L#	29.7	178	7.88E-44				
1908	OSC73185.1	-	transporter	0	1	0	0	0	0	0	0	0	0	0	1	Unique	COG0471_P#	22.9	84	1.55E-15	K14445	23.4	84	2.43E-15
1909	AXI91257.1	-	replicative DNA helicase	0	1	0	1	0	0	0	0	0	0	0	2	Accessory	COG0305_L#	37.8	265	3.75E-70	K02314	34.8	228	1.77E-58
1910	Prokka:CIODPCDJ_00443	-	hypothetical protein	1	1	1	1	1	1	1	1	1	1	1	11	CORE	COG2340_S#	40.5	122	3.93E-27	NA	40.5	122	1.05E-26
1911	PWE07601.1	-	hypothetical protein	0	0	1	0	0	0	0	0	0	1	0	2	Accessory								
1912	Prokka:CIODPCDJ_06801	-	hypothetical protein	0	1	0	0	1	0	0	0	0	0	1	3	Accessory								
1913	Prokka:JAPCLKMA_05867	-	hypothetical protein	1	1	1	1	1	1	1	1	1	1	1	11	CORE	COG0664_T#	27.3	43	0.00302				
1914	WP_033273578.1	-	extracellular solute-binding protein	1	0	0	0	1	1	1	0	1	1	1	7	Accessory								
1915	AXI88307.1	-	hypothetical protein	0	0	0	1	0	0	0	1	1	0	0	3	Accessory								
1916	WP_159020873.1	-	hypothetical protein	0	0	0	0	0	0	0	1	0	0	0	1	Unique								
1917	WP_033272913.1	-	FAD-dependent oxidoreductase	1	1	1	1	1	1	1	1	1	1	1	11	CORE	COG0446_R#	25.2	58	6.95E-08	NA	40.7	67	3.06E-10

cluster	GI	gene	Annotation	Streptomyces_thermocarboxydus_BPSAC147	Streptomyces_sp._4F	Streptomyces_sp._BSE7F	Streptomyces_sp._ETH9427	Streptomyces_sp._FxanaD5	Streptomyces_sp._UNC401CLCol	Actinospica_acidiphila_NRRL_B_24431	Streptomyces_sp._HNS054	Streptomyces_sp._SMS_SU21	Streptomyces_sp._ZS0098	Streptomyces_thermocarboxydus_K155	Addition	Core-Accessory-Unique	COG	%Similarity_COG	Score_COG	Evalue_COG	KEGG	%Similarity_KEGG	Score_KEGG	Evalue_KEGG
1918	Prokka:JAPCLKMA_02000	-	hypothetical protein	1	0	0	0	0	1	0	0	0	0	0	2	Accessory								
1919	OSC71845.1	-	hypothetical protein	1	1	1	1	1	1	1	1	1	1	1	11	CORE	COG3599_D#	28.4	52	0.00000651	NA	23.6	75	1.12E-12
1920	WP_086020086.1	lpdA	dihydrolipoyl dehydrogenase	1	1	1	1	1	1	1	1	1	1	1	11	CORE	COG1249_C#	40.3	342	1.85E-93	K00382	40.9	342	8.41E-93
1921	WP_019527140.1	mycP	type VII secretion-associated serine protease mycosin	1	0	0	0	1	1	0	0	0	0	1	4	Accessory	COG1404_O#	35.6	132	3.79E-30	K13275	33.7	132	1.01E-29
1922	PWE09315.1	-	VanZ family protein	0	0	1	0	0	0	0	0	0	0	0	1	Unique								
1923	WP_122215926.1	-	hypothetical protein	0	0	0	0	0	0	0	0	0	1	0	1	Unique								
1924	WP_033272198.1	-	class II fumarate hydratase	1	1	1	1	1	1	1	1	1	1	1	11	CORE	COG0114_C#	65.2	571	3.18E-162	K01679	64.3	571	6.51E-162
1925	OSC61488.1	-	trehalose-6-phosphate synthase	1	1	1	1	1	1	1	1	1	1	1	11	CORE	COG0380_G#	32.8	251	7.3E-66	K16055	33.1	224	1.95E-57
1926	WP_086020203.1	-	NAD(P)-binding protein	1	1	0	0	1	1	0	0	0	0	1	5	Accessory					NA	31.9	156	5E-37
1927	WP_122212953.1	-	MBL fold metallo-hydrolase	1	1	1	1	1	1	1	1	1	1	1	11	CORE	COG0491_R#	31.9	171	9.59E-42	NA	32	189	9.09E-47
1928	Prokka:CIODPCDJ_04976	-	hypothetical protein	1	1	1	0	1	1	1	1	1	1	1	10	Accessory	COG0628_R#	25.7	132	4.94E-30	NA	25.7	132	1.32E-29
1929	OSC63494.1	-	hypothetical protein	0	1	0	0	0	0	0	0	0	0	0	1	Unique					NA	31.7	208	1.45E-52

cluster	GI	gene	Annotation	Streptomyces_thermocarboxydus_BPSAC147	Streptomyces_sp._4F	Streptomyces_sp._BSE7F	Streptomyces_sp._ETH9427	Streptomyces_sp._FxanaD5	Streptomyces_sp._UNC401CLCol	Actinospica_acidiphila_NRRL_B_24431	Streptomyces_sp._HNS054	Streptomyces_sp._SMS_SU21	Streptomyces_sp._ZS0098	Streptomyces_thermocarboxydus_K155	Addition	Core-Accessory-Unique	COG	%Similarity_COG	Score_COG	Evalue_COG	KEGG	%Similarity_KEGG	Score_KEGG	Evalue_KEGG
1930	OSC62859.1	-	aldehyde dehydrogenase	0	1	1	1	0	0	0	1	1	1	0	6	Accessory	COG1012_C#	39.1	304	5.56E-82	K00146	39.1	283	2.71E-75
1931	WP_078504540.1	-	MFS transporter	1	1	1	1	1	1	1	1	1	1	1	11	CORE	COG2270_R#	30	219	2.35E-56	K06902	30	219	6.28E-56
1932	WP_028961074.1	-	FAD-dependent oxidoreductase	1	1	0	0	1	1	1	1	1	1	1	9	Accessory	COG0446_R#	37.1	211	6.41E-54	NA	25.7	96	6.14E-19
1933	WP_019521822.1	hydA	dihydropyrimidinase	1	1	1	1	1	1	1	1	1	1	1	11	CORE	COG0044_F#	43.4	370	1.08E-101	K01464	41.5	343	3.77E-93
1934	OSC71640.1	-	hypothetical protein	1	1	1	1	1	1	1	1	1	1	1	11	CORE								
1935	Prokka:JAPCLKMA_01489	-	hypothetical protein	1	0	0	0	0	0	0	0	0	0	0	1	Unique								
1936	PWE07263.1	-	hypothetical protein	0	0	1	0	0	0	0	0	1	0	0	2	Accessory								
1937	Prokka:CIODPCDJ_05884	pcaF	Beta-ketoadipyl-CoA thiolase	1	1	1	1	1	1	1	1	1	1	1	11	CORE	COG0183_I#	58.9	425	2.16E-118	K00626	54.7	409	3.28E-113
1938	OSC66171.1	-	amidase	1	1	1	1	1	1	1	1	1	1	1	11	CORE	COG0154_J#	53.1	456	1.49E-127	K02433	34.9	218	1.07E-55
1939	Prokka:JAPCLKMA_04175	btuD_13	Vitamin B12 import ATP-binding protein BtuD	1	1	1	1	1	1	1	1	1	1	1	11	CORE	COG3839_G#	49.2	308	5.03E-83	K10112	51.6	297	2.37E-79
1940	Prokka:CIODPCDJ_02974	cysS	Cysteine--tRNA ligase	1	1	1	1	1	1	1	1	1	1	1	11	CORE	COG0215_J#	58	552	1.53E-156	K01883	58	551	6.96E-156

cluster	GI	gene	Annotation	Streptomyces_thermocarboxydus_BPSAC147	Streptomyces_sp._4F	Streptomyces_sp._BSE7F	Streptomyces_sp._ETH9427	Streptomyces_sp._FxanaD5	Streptomyces_sp._UNC401CLCol	Actinospica_acidiphila_NRRL_B_24431	Streptomyces_sp._HNS054	Streptomyces_sp._SMS_SU21	Streptomyces_sp._ZS0098	Streptomyces_thermocarboxydus_K155	Addition	Core-Accessory-Unique	COG	%Similarity_COG	Score_COG	Evalue_COG	KEGG	%Similarity_KEGG	Score_KEGG	Evalue_KEGG
1941	Prokka:JAPCLKMA_03039	tetX	Flavin-dependent monooxygenase	1	0	0	0	1	1	1	0	0	0	1	5	Accessory	COG0654_HC#	40.9	326	1.78E-88	NA	31.7	188	2.02E-46
1942	OSC70603.1	-	acyltransferase papA2	0	1	0	0	0	0	0	0	0	0	0	1	Unique	COG1020_Q#	28.4	162	4.45E-39	NA	28.4	162	1.19E-38
1943	OSC71361.1	-	beta-galactosidase	0	1	0	0	0	0	0	0	0	0	0	1	Unique	COG3250_G#	44.2	155	4.16E-37	K01190	33.2	170	4.36E-41
1944	WP_159020960.1	-	hypothetical protein	0	0	0	1	0	0	0	1	1	1	0	4	Accessory								
1945	WP_033272949.1	-	aminotransferase class V-fold PLP-dependent enzyme	1	1	1	1	1	1	1	1	1	1	1	11	CORE	COG0520_E#	33	209	3.17E-53	K11717	35.6	205	9.37E-52
1946	WP_033277459.1	-	MFS transporter	1	0	0	1	1	0	1	1	0	1	1	7	Accessory	COG0477_GEPR#	30.9	185	3.76E-46	K08169	27.5	140	4.83E-32
1947	Prokka:CIODPCDJ_04659	-	hypothetical protein	1	1	0	0	1	1	0	0	0	0	1	5	Accessory	COG1653_G#	29.5	200	1.13E-50				
1948	Prokka:JAPCLKMA_00776	-	putative zinc protease	1	1	1	1	1	1	1	1	1	1	1	11	CORE	COG0612_R#	38.1	287	9.17E-77	NA	35.7	215	1.54E-54
1949	WP_122215935.1	-	hypothetical protein	0	0	0	0	0	0	0	0	0	1	0	1	Unique								
1950	WP_084207108.1	-	DNA polymerase IV	1	1	0	1	0	1	1	1	1	1	1	9	Accessory	COG0389_L#	45.4	342	2.4E-93	K02346	45.4	341	1.09E-92
1951	Prokka:CIODPCDJ_01900	bcr	Bicyclomycin resistance protein	1	1	1	1	1	1	1	1	1	1	1	11	CORE	COG0477_GEPR#	40.3	308	5.01E-83	K07552	40.3	308	1.34E-82
1952	WP_019522734.1	-	hypothetical protein	1	0	1	1	1	1	1	1	1	1	1	10	Accessory	COG2610_GE#	49.4	434	3.55E-121	K06155	43	381	1.62E-104

cluster	GI	gene	Annotation	Streptomyces_thermocarboxydus_BPSAC147	Streptomyces_sp._4F	Streptomyces_sp._BSE7F	Streptomyces_sp._ETH9427	Streptomyces_sp._FxanaD5	Streptomyces_sp._UNC401CLCol	Actinospica_acidiphila_NRRL_B_24431	Streptomyces_sp._HNS054	Streptomyces_sp._SMS_SU21	Streptomyces_sp._ZS0098	Streptomyces_thermocarboxydus_K155	Addition	Core-Accessory-Unique	COG	%Similarity_COG	Score_COG	Evalue_COG	KEGG	%Similarity_KEGG	Score_KEGG	Evalue_KEGG
1953	AXI89347.1	-	Tat pathway signal sequence domain protein	1	1	1	1	1	1	1	1	1	1	1	11	CORE	COG3211_R#	39.7	317	6.32E-86				
1954	OSC65751.1	-	ABC transporter ATP-binding protein	1	1	1	1	1	1	1	1	1	1	1	11	CORE	COG0661_R#	44.8	387	8.48E-107	NA	44.8	386	2.96E-106
1955	OSC68074.1	-	salicylate synthase	0	1	0	0	0	0	0	0	0	0	0	1	Unique	COG0147_EH#	40.4	285	3.48E-76	K04781	40.4	285	9.28E-76
1956	OSC67102.1	-	UDP-N-acetylmuramate--L-alanine ligase	1	1	1	1	1	1	1	1	1	1	1	11	CORE	COG0773_M#	45	380	7.94E-105	K01924	46.3	371	1.29E-101
1957	OSC65252.1	-	replication initiation protein	1	1	0	0	1	0	0	0	1	0	0	4	Accessory								
1958	WP_122214200.1	-	FAD-dependent oxidoreductase	1	1	1	1	1	1	1	1	1	1	1	11	CORE	COG0446_R#	44.2	330	7.21E-90	NA	44.2	330	1.93E-89
1959	WP_033275751.1	-	SLC13 family permease	1	1	1	0	1	1	1	0	0	1	1	8	Accessory	COG0471_P#	24.1	69	3.91E-11	NA	67.9	44	0.00276
1960	WP_078504651.1	-	IS1380 family transposase	0	0	0	1	1	1	0	1	0	1	0	5	Accessory								
1961	OSC71445.1	-	peptidase	1	1	1	1	1	1	1	1	1	1	1	11	CORE	COG0312_R#	53.6	460	1.03E-128	NA	53.6	460	2.75E-128
1962	OSC62116.1	-	1,3-beta-glucanase	1	1	1	1	1	1	1	1	1	1	1	11	CORE	COG5498_M#	44.2	137	1.17E-31				
1963	OSC72007.1	-	hypothetical protein	0	1	0	0	0	0	0	0	0	0	0	1	Unique	COG1020_Q#	30.3	201	5.05E-51	K04789	26.5	154	1.89E-36

cluster	GI	gene	Annotation	Streptomyces_thermocarboxydus_BPSAC147	Streptomyces_sp._4F	Streptomyces_sp._BSE7F	Streptomyces_sp._ETH9427	Streptomyces_sp._FxanaD5	Streptomyces_sp._UNC401CLCol	Actinospica_acidiphila_NRRL_B_24431	Streptomyces_sp._HNS054	Streptomyces_sp._SMS_SU21	Streptomyces_sp._ZS0098	Streptomyces_thermocarboxydus_K155	Addition	Core-Accessory-Unique	COG	%Similarity_COG	Score_COG	Evalue_COG	KEGG	%Similarity_KEGG	Score_KEGG	Evalue_KEGG
1964	OSC63567.1	-	amino acid permease	0	1	0	0	0	0	0	0	0	0	1	2	Accessory	COG0531_E#	33.9	206	2.05E-52	K03294	27.6	157	2.92E-37
1965	Prokka:JAPCLKMA_03267	cysG_2	Siroheme synthase	1	1	1	1	1	1	1	1	1	1	1	11	CORE	COG0007_H#	62.7	281	5.01E-75	K02302	58.3	451	9.76E-126
1966	Prokka:CIODPCDJ_02498	lapB_2	Lipopolysaccharide assembly protein B	1	1	1	1	1	1	1	1	1	1	1	11	CORE	COG0457_R#	24.5	59	3.1E-08	K09667	24.3	64	4.38E-09
1967	Prokka:JAPCLKMA_03474	-	hypothetical protein	1	1	0	1	1	1	1	1	1	1	1	10	Accessory	COG2755_E#	38.4	257	1.01E-67				
1968	OSC61249.1	-	GntR family transcriptional regulator	0	1	0	0	0	0	0	0	0	0	0	1	Unique	COG1167_KE#	37.6	245	5.19E-64	K00375	37.6	245	1.39E-63
1969	Prokka:JAPCLKMA_00519	dap	D-aminopeptidase	1	1	1	1	1	1	1	1	1	1	1	11	CORE	COG1680_V#	31.2	128	5.43E-29	NA	29.3	126	7.19E-28
1970	WP_028960668.1	-	PAS domain-containing protein	1	1	1	1	1	1	1	1	1	1	1	11	CORE	COG2202_T#	23.2	64	1.26E-09	NA	26.9	54	0.00000266
1971	AXI91368.1	-	hypothetical protein	0	0	0	1	0	0	0	0	0	0	0	1	Unique								
1972	OSC64053.1	-	dehydrogenase	0	1	0	0	0	0	0	0	0	0	0	1	Unique	COG0673_R#	35.7	238	3.72E-62	NA	36.9	206	5.47E-52
1973	OSC61086.1	-	MFS transporter	0	1	0	0	0	0	0	0	0	0	0	1	Unique	COG0477_GEPR#	53	459	1.34E-128	K08172	33.3	265	7.59E-70
1974	WP_019524835.1	-	hypothetical protein	0	0	0	0	1	1	0	0	0	0	0	2	Accessory								
1975	Prokka:JAPCLKMA_00441	-	6-hydroxy-D-nicotine oxidase	1	0	1	1	1	1	1	1	1	1	1	10	Accessory	COG0277_C#	43	363	1E-99	NA	38.1	296	4.02E-79

cluster	GI	gene	Annotation	Streptomyces_thermocarboxydus_BPSAC147	Streptomyces_sp._4F	Streptomyces_sp._BSE7F	Streptomyces_sp._ETH9427	Streptomyces_sp._FxanaD5	Streptomyces_sp._UNC401CLCol	Actinospica_acidiphila_NRRL_B_24431	Streptomyces_sp._HNS054	Streptomyces_sp._SMS_SU21	Streptomyces_sp._ZS0098	Streptomyces_thermocarboxydus_K155	Addition	Core-Accessory-Unique	COG	%Similarity_COG	Score_COG	Evalue_COG	KEGG	%Similarity_KEGG	Score_KEGG	Evalue_KEGG
1976	WP_019524101.1	lysA	diaminopimelate decarboxylase	1	1	1	1	1	1	1	1	1	1	1	11	CORE	COG0019_E#	58.5	517	5.41E-146	K01586	59.9	515	4.2E-145
1977	Prokka:JAPCLKMA_02207	yhdG_2	putative amino acid permease YhdG	1	1	1	1	1	1	1	1	1	1	1	11	CORE	COG0531_E#	33.1	217	8.86E-56	NA	29.4	171	1.95E-41
1978	WP_102637526.1	-	amidase	0	1	0	0	0	0	0	1	1	0	0	3	Accessory	COG0154_J#	38.9	295	4.37E-79	K02433	38.9	295	1.17E-78
1979	OSC73414.1	-	hypothetical protein	1	1	1	1	1	1	1	1	1	1	1	11	CORE	COG2733_S#	45.8	387	8.45E-107	NA	45.8	387	2.26E-106
1980	WP_033274125.1	-	hypothetical protein	1	1	1	0	1	1	1	1	1	1	1	10	Accessory								
1981	WP_028960089.1	-	NtaA/DmoA family FMN-dependent monooxygenase	1	0	0	0	1	1	1	0	0	0	1	5	Accessory	COG2141_C#	42.8	322	2.55E-87	NA	39.5	303	4.28E-81
1982	OSC61341.1	-	hypothetical protein	0	1	0	0	0	0	0	0	0	0	0	1	Unique								
1983	Prokka:JAPCLKMA_04597	-	putative aminotransferase	1	1	1	1	1	1	1	1	1	1	1	11	CORE	COG0161_H#	70.8	648	1.56E-185	NA	71.9	641	5.08E-183
1984	WP_033278071.1	-	hypothetical protein	0	0	0	0	0	0	1	0	0	0	0	1	Unique	COG2303_E#	40.3	317	1.07E-85	NA	36.8	227	2.99E-58
1985	WP_026243896.1	-	stress response protein	1	0	1	1	1	1	1	1	1	1	1	10	Accessory	COG2310_T#	46.7	129	4.14E-29	NA	46.7	129	1.1E-28
1986	Prokka:CIODPCDJ_05034	-	hypothetical protein	1	0	1	1	1	1	1	1	1	1	1	10	Accessory	COG0612_R#	30.1	180	1.56E-44	NA	24.8	71	2.09E-11
1987	PWE10436.1	-	MFS transporter	1	0	1	0	1	1	1	0	1	0	1	7	Accessory	COG0477_GEPR#	45.9	422	1.81E-117	K08369	45.9	422	4.83E-117

cluster	GI	gene	Annotation	Streptomyces_thermocarboxydus_BPSAC147	Streptomyces_sp._4F	Streptomyces_sp._BSE7F	Streptomyces_sp._ETH9427	Streptomyces_sp._FxanaD5	Streptomyces_sp._UNC401CLCol	Actinospica_acidiphila_NRRL_B_24431	Streptomyces_sp._HNS054	Streptomyces_sp._SMS_SU21	Streptomyces_sp._ZS0098	Streptomyces_thermocarboxydus_K155	Addition	Core-Accessory-Unique	COG	%Similarity_COG	Score_COG	Evalue_COG	KEGG	%Similarity_KEGG	Score_KEGG	Evalue_KEGG
1988	WP_048457323.1	-	cystathionine beta-synthase	1	1	0	1	1	1	1	1	1	1	1	10	Accessory	COG0031_E#	71.8	502	2.35E-141	K01697	67	618	6E-176
1989	WP_102639153.1	-	aminotransferase class V-fold PLP-dependent enzyme	1	1	1	1	1	1	1	1	1	1	1	11	CORE	COG1104_E#	34	231	5.92E-60	K04487	33.9	207	2.44E-52
1990	OSC72194.1	-	NDP-hexose 2,3-dehydratase	0	1	0	0	0	0	0	0	0	0	0	1	Unique								
1991	Prokka:JAPCLKMA_01644	-	hypothetical protein	1	0	0	1	0	0	0	0	0	0	0	2	Accessory								
1992	Prokka:CIODPCDJ_05243	-	hypothetical protein	0	0	0	0	1	1	0	0	0	0	1	3	Accessory	COG2755_E#	35.2	143	2.77E-33	NA	35.2	143	7.39E-33
1993	PWE10540.1	-	mucin-1	0	0	1	0	0	0	0	1	0	1	0	3	Accessory								
1994	Prokka:CIODPCDJ_05700	bglA_3	Beta-glucosidase A	1	0	1	1	1	1	1	1	1	1	1	10	Accessory	COG2723_G#	49.2	423	1.39E-117	K01188	38.4	316	4.88E-85
1995	OSC54629.1	-	cytosine permease	0	1	0	0	0	0	0	0	0	0	0	1	Unique	COG1457_F#	31	211	6.34E-54	K10974	27.1	158	2.22E-37
1996	AXI85935.1	-	aldehyde dehydrogenase family protein	1	1	1	1	1	1	1	1	1	1	1	11	CORE	COG1012_C#	55.9	510	5.05E-144	K00128	56	497	2.02E-139
1997	Prokka:CIODPCDJ_02876	-	hypothetical protein	1	0	0	1	0	1	1	0	1	0	1	6	Accessory					NA	26.4	81	2.02E-14
1998	Prokka:CIODPCDJ_03000	rep_1	ATP-dependent DNA helicase Rep	0	0	0	0	0	0	0	0	0	0	1	1	Unique	COG0210_L#	44.1	46	0.000353	K03657	33.3	45	0.0021

cluster	GI	gene	Annotation	Streptomyces_thermocarboxydus_BPSAC147	Streptomyces_sp._4F	Streptomyces_sp._BSE7F	Streptomyces_sp._ETH9427	Streptomyces_sp._FxanaD5	Streptomyces_sp._UNC401CLCol	Actinospica_acidiphila_NRRL_B_24431	Streptomyces_sp._HNS054	Streptomyces_sp._SMS_SU21	Streptomyces_sp._ZS0098	Streptomyces_thermocarboxydus_K155	Addition	Core-Accessory-Unique	COG	%Similarity_COG	Score_COG	Evalue_COG	KEGG	%Similarity_KEGG	Score_KEGG	Evalue_KEGG
1999	WP_122216231.1	-	2-oxo acid dehydrogenase subunit E2	0	0	0	0	0	0	0	0	1	1	0	2	Accessory	COG0508_C#	31.6	221	8E-57	K00627	34	179	7.13E-44
2000	OSC69599.1	-	8-oxoguanine deaminase	1	1	1	1	1	1	1	1	1	1	1	11	CORE	COG0402_FR#	45.5	350	1.14E-95	K12960	30.8	190	5.27E-47
2001	WP_033274241.1	-	FAD-dependent oxidoreductase	0	0	0	0	0	0	1	0	0	0	0	1	Unique	COG1249_C#	44.2	351	5.12E-96	NA	41.7	343	2.85E-93
2002	WP_028959479.1	-	ArsA family ATPase	1	1	1	1	1	1	1	1	1	1	1	11	CORE	COG0003_P#	59.7	350	8.72E-96	NA	59.7	350	2.33E-95
2003	WP_019524561.1	-	hypothetical protein	1	1	1	1	1	1	1	1	1	1	1	11	CORE								
2004	Prokka:CIODPCDJ_06652	abaF_2	Fosfomycin resistance protein AbaF	0	0	0	0	1	1	0	0	0	0	1	3	Accessory	COG0477_GEPR#	36.8	278	5.5E-74	NA	39.2	262	8.34E-69
2005	WP_019524718.1	-	NADP-dependent succinic semialdehyde dehydrogenase	1	1	1	1	1	1	1	1	1	1	1	11	CORE	COG1012_C#	63.6	585	2.1E-166	K00135	63.6	585	5.62E-166
2006	OSC61105.1	-	two-component sensor histidine kinase	1	1	1	1	1	1	1	1	1	1	1	11	CORE	COG4585_T#	32.5	95	5.06E-19	NA	32.5	95	1.35E-18
2007	WP_028961596.1	-	dehydrogenase	1	1	0	0	1	1	0	0	0	0	1	5	Accessory								

cluster	GI	gene	Annotation	Streptomyces_thermocarboxydus_BPSAC147	Streptomyces_sp._4F	Streptomyces_sp._BSE7F	Streptomyces_sp._ETH9427	Streptomyces_sp._FxanaD5	Streptomyces_sp._UNC401CLCol	Actinospica_acidiphila_NRRL_B_24431	Streptomyces_sp._HNS054	Streptomyces_sp._SMS_SU21	Streptomyces_sp._ZS0098	Streptomyces_thermocarboxydus_K155	Addition	Core-Accessory-Unique	COG	%Similarity_COG	Score_COG	Evalue_COG	KEGG	%Similarity_KEGG	Score_KEGG	Evalue_KEGG
2008	WP_019527423.1	-	tyrosine-type recombinase/integrase	0	0	0	0	1	0	1	0	0	0	0	2	Accessory	COG0582_L#	29.7	71	1.02E-11	NA	29.7	71	2.73E-11
2009	WP_033277286.1	-	DUF4935 domain-containing protein	0	0	0	0	0	0	1	0	1	0	0	2	Accessory								
2010	Prokka:JAPCLKMA_06126	-	hypothetical protein	1	0	1	1	1	1	1	1	1	1	1	10	Accessory	COG3552_R#	44.7	369	1.39E-101	K07161	44.7	369	3.71E-101
2011	Prokka:JAPCLKMA_02003	-	putative AAA domain-containing protein	1	1	1	1	1	1	1	1	1	1	1	11	CORE	COG2256_L#	63.6	537	6.57E-152	K07478	63.6	537	1.75E-151
2012	WP_122213819.1	-	hypothetical protein	0	0	0	0	0	0	0	0	0	1	0	1	Unique					NA	29.1	45	0.00209
2013	WP_122217371.1	eccD	type VII secretion integral membrane protein EccD	0	0	1	0	0	0	0	0	0	1	0	2	Accessory					NA	28	122	1.35E-26
2014	WP_102638580.1	-	hypothetical protein	1	0	0	0	0	0	0	0	1	0	0	2	Accessory								
2015	WP_048457850.1	-	VWA domain-containing protein	1	1	1	1	1	1	1	1	1	1	1	11	CORE								
2016	WP_033275024.1	-	ferredoxin reductase family protein	1	1	1	1	1	1	1	1	1	1	1	11	CORE	COG4097_P#	27.5	128	7.03E-29				
2017	AXI86612.1	-	glycine--tRNA ligase	1	1	1	1	1	1	1	1	1	1	1	11	CORE	COG0423_J#	70.3	689	1.03E-197	K01880	68.1	662	3.61E-189

cluster	GI	gene	Annotation	Streptomyces_thermocarboxydus_BPSAC147	Streptomyces_sp._4F	Streptomyces_sp._BSE7F	Streptomyces_sp._ETH9427	Streptomyces_sp._FxanaD5	Streptomyces_sp._UNC401CLCol	Actinospica_acidiphila_NRRL_B_24431	Streptomyces_sp._HNS054	Streptomyces_sp._SMS_SU21	Streptomyces_sp._ZS0098	Streptomyces_thermocarboxydus_K155	Addition	Core-Accessory-Unique	COG	%Similarity_COG	Score_COG	Evalue_COG	KEGG	%Similarity_KEGG	Score_KEGG	Evalue_KEGG
2018	Prokka:CIODPCDJ_03159	-	hypothetical protein	0	0	0	0	0	0	0	0	0	0	1	1	Unique								
2019	AXI84626.1	-	hypothetical protein	0	0	0	1	0	0	0	1	0	1	0	3	Accessory	COG0367_E#	29	80	1.68E-14	K01953	29	80	4.49E-14
2020	WP_026244266.1	-	hypothetical protein	0	0	0	0	1	0	0	0	0	0	0	1	Unique								
2021	OSC69287.1	-	hypothetical protein	0	1	0	0	0	0	0	0	0	0	0	1	Unique								
2022	OSC65709.1	-	peptide ABC transporter ATP-binding protein	1	1	1	1	1	1	1	1	1	1	1	11	CORE	COG4608_E#	58.5	387	8.39E-107	K02032	57.1	383	2.48E-105
2023	PWE12322.1	-	hypothetical protein	0	0	1	1	0	0	1	1	1	1	0	6	Accessory								
2024	AXI89063.1	-	insulinase family protein	1	1	1	1	1	1	1	1	1	1	1	11	CORE	COG0612_R#	51.2	418	4.43E-116	NA	51.2	418	1.18E-115
2025	WP_019521701.1	-	alkaline phosphatase family protein	1	1	1	1	1	1	1	1	1	1	1	11	CORE	COG1524_R#	50.1	460	5.96E-129				
2026	WP_033272058.1	-	HD-GYP domain-containing protein	1	1	1	1	1	1	1	1	1	1	1	11	CORE	COG2206_T#	43.4	158	6.33E-38	NA	43.4	160	4.45E-38
2027	WP_019527363.1	-	ATP-dependent 6-phosphofructokinase	0	0	0	0	1	1	0	0	0	0	0	2	Accessory	COG0205_G#	42.8	368	4.02E-101	K00850	41.6	349	6.75E-95
2028	WP_028959274.1	-	conjugal transfer protein TraS	1	1	1	1	1	1	1	1	1	0	1	10	Accessory								

cluster	GI	gene	Annotation	Streptomyces_thermocarboxydus_BPSAC147	Streptomyces_sp._4F	Streptomyces_sp._BSE7F	Streptomyces_sp._ETH9427	Streptomyces_sp._FxanaD5	Streptomyces_sp._UNC401CLCol	Actinospica_acidiphila_NRRL_B_24431	Streptomyces_sp._HNS054	Streptomyces_sp._SMS_SU21	Streptomyces_sp._ZS0098	Streptomyces_thermocarboxydus_K155	Addition	Core-Accessory-Unique	COG	%Similarity_COG	Score_COG	Evalue_COG	KEGG	%Similarity_KEGG	Score_KEGG	Evalue_KEGG
2029	OSC71384.1	-	coproporphyrinogen III oxidase	0	1	0	0	0	0	0	0	0	0	0	1	Unique	COG0635_H#	32.2	201	8.51E-51	K02495	30.4	176	4.59E-43
2030	Prokka:JAPCLKMA_02610	-	hypothetical protein	1	0	0	0	0	0	0	0	0	0	0	1	Unique								
2031	WP_122214199.1	-	hypothetical protein	0	0	0	1	0	0	1	1	0	1	0	4	Accessory	COG2755_E#	38.5	144	9.45E-34	NA	38.5	144	2.52E-33
2032	OSC70908.1	-	sensor histidine kinase	0	1	0	0	0	0	0	0	0	0	0	1	Unique	COG4585_T#	28	88	8.04E-17	NA	28	88	2.15E-16
2033	OSC72954.1	-	peptidase M6	1	1	1	1	1	1	1	1	1	1	1	11	CORE								
2034	WP_048456870.1	-	hypothetical protein	1	1	0	1	1	1	1	1	1	1	1	10	Accessory								
2035	OSC70913.1	-	serine protease	1	1	1	1	1	1	1	1	1	1	1	11	CORE								
2036	PWE06687.1	-	aldehyde dehydrogenase	1	1	1	1	1	1	1	1	1	1	1	11	CORE	COG1012_C#	65.5	569	9.09E-162	K00128	65.5	569	2.43E-161
2037	AXI89218.1	-	deoxyribodipyrimidine photolyase	1	0	1	1	1	1	1	1	1	1	1	10	Accessory	COG0415_L#	37.4	268	4.33E-71	K01669	35	255	1.01E-66
2038	OSC71395.1	-	CoF synthetase	0	1	0	0	0	0	0	0	0	0	0	1	Unique	COG1541_H#	30.2	172	4.22E-42	K01912	27.7	140	4.75E-32
2039	WP_122214007.1	-	hypothetical protein	1	0	1	1	1	1	1	1	1	1	1	10	Accessory					NA	51.5	414	1.3E-114
2040	WP_033272624.1	-	FAD-binding oxidoreductase	0	0	0	0	0	0	1	0	0	0	0	1	Unique	COG0277_C#	27.4	129	4.1E-29	NA	27.4	129	1.1E-28
2041	WP_102637327.1	-	DUF1254 domain-containing protein	0	0	0	0	0	0	0	0	1	1	0	2	Accessory	COG5361_S#	46	429	1.12E-119				

cluster	GI	gene	Annotation	Streptomyces_thermocarboxydus_BPSAC147	Streptomyces_sp._4F	Streptomyces_sp._BSE7F	Streptomyces_sp._ETH9427	Streptomyces_sp._FxanaD5	Streptomyces_sp._UNC401CLCol	Actinospica_acidiphila_NRRL_B_24431	Streptomyces_sp._HNS054	Streptomyces_sp._SMS_SU21	Streptomyces_sp._ZS0098	Streptomyces_thermocarboxydus_K155	Addition	Core-Accessory-Unique	COG	%Similarity_COG	Score_COG	Evalue_COG	KEGG	%Similarity_KEGG	Score_KEGG	Evalue_KEGG
2042	Prokka:CIODPCDJ_06751	-	hypothetical protein	0	0	0	0	0	0	0	0	0	0	1	1	Unique								
2043	Prokka:JAPCLKMA_02184	nisC_2	Nisin biosynthesis protein NisC	1	1	0	0	1	1	1	0	0	0	1	6	Accessory								
2044	WP_048455187.1	-	hypothetical protein	0	0	1	1	0	0	1	1	1	0	0	5	Accessory								
2045	OSC69316.1	-	cytochrome P450	1	1	0	1	1	1	1	1	1	1	1	10	Accessory	COG2124_Q#	29.2	209	3.12E-53	K07427	26.5	156	4.9E-37
2046	OSC57362.1	-	MGT family glycosyltransferase	0	1	0	0	0	0	0	0	0	0	0	1	Unique	COG1819_GC#	26.9	59	3.06E-08	K00699	24.1	98	2.07E-19
2047	OSC71264.1	-	NADH-quinone oxidoreductase subunit H	1	1	1	1	1	1	1	1	1	1	1	11	CORE	COG1005_C#	57.9	485	1.73E-136	K00337	57.9	485	4.61E-136
2048	WP_019524765.1	-	HlyC/CorC family transporter	1	1	1	1	1	1	1	1	1	1	1	11	CORE	COG1253_R#	44.2	322	3.29E-87	NA	44.2	322	8.79E-87
2049	WP_048459204.1	-	tyrosine-type recombinase/integrase	0	1	1	0	0	1	1	1	0	0	0	5	Accessory								
2050	WP_048456716.1	-	glycosidase	1	1	1	1	1	1	1	1	1	1	1	11	CORE	COG0366_G#	28.1	146	2.48E-34	K01176	43.1	369	4.8E-101
2051	WP_028958998.1	-	S1 family peptidase	1	1	1	1	1	1	1	1	1	1	1	11	CORE	COG0265_O#	26.3	58	6.79E-08	NA	27.9	69	7.87E-11
2052	Prokka:CIODPCDJ_06287	-	hypothetical protein	1	1	1	1	1	1	1	1	1	1	1	11	CORE								

cluster	GI	gene	Annotation	Streptomyces_thermocarboxydus_BPSAC147	Streptomyces_sp._4F	Streptomyces_sp._BSE7F	Streptomyces_sp._ETH9427	Streptomyces_sp._FxanaD5	Streptomyces_sp._UNC401CLCol	Actinospica_acidiphila_NRRL_B_24431	Streptomyces_sp._HNS054	Streptomyces_sp._SMS_SU21	Streptomyces_sp._ZS0098	Streptomyces_thermocarboxydus_K155	Addition	Core-Accessory-Unique	COG	%Similarity_COG	Score_COG	Evalue_COG	KEGG	%Similarity_KEGG	Score_KEGG	Evalue_KEGG
2053	OSC69924.1	-	NAD-dependent succinate-semialdehyde dehydrogenase	0	1	0	0	0	0	0	0	0	0	0	1	Unique	COG1012_C#	59.9	532	1.23E-150	K00135	49.5	441	9.95E-123
2054	Prokka:JAPCLKMA_05378	pknD_16	Serine/threonine-protein kinase PknD	1	1	0	1	1	1	1	1	0	1	0	8	Accessory	COG0515_RTKL#	32.8	174	1.11E-42	K08884	32.8	174	2.96E-42
2055	WP_157842221.1	-	hypothetical protein	0	0	0	0	0	0	1	0	0	0	0	1	Unique								
2056	Prokka:JAPCLKMA_03381	-	hypothetical protein	1	0	0	0	0	0	0	0	0	0	0	1	Unique								
2057	WP_106432463.1	-	acyltransferase	1	0	0	0	1	1	0	0	0	0	1	4	Accessory	COG1835_I#	29.8	153	2.03E-36				
2058	OSC65521.1	-	D-inositol-3-phosphate glycosyltransferase	1	1	1	1	1	1	1	1	1	1	1	11	CORE	COG0438_M#	57.3	460	7.75E-129	K15521	57.3	460	2.07E-128
2059	OSC62349.1	-	ABC transporter substrate-binding protein	0	1	0	0	0	0	0	0	0	0	0	1	Unique	COG1653_G#	29.5	206	1.54E-52				
2060	Prokka:JAPCLKMA_00298	-	hypothetical protein	1	1	1	1	1	1	1	1	1	1	1	11	CORE	COG1653_G#	45.3	373	1.24E-102				
2061	Prokka:JAPCLKMA_01765	stp_1	Multidrug resistance protein Stp	1	0	0	0	0	1	0	0	0	0	1	3	Accessory	COG0477_GEPR#	36.9	260	8.99E-69	NA	34.2	220	4.7E-56
2062	WP_122215760.1	-	SMI1/KNR4 family protein	1	0	0	1	0	0	1	1	0	1	0	5	Accessory	COG4282_G#	30.4	56	2.58E-07	NA	30.4	56	6.88E-07

cluster	GI	gene	Annotation	Streptomyces_thermocarboxydus_BPSAC147	Streptomyces_sp._4F	Streptomyces_sp._BSE7F	Streptomyces_sp._ETH9427	Streptomyces_sp._FxanaD5	Streptomyces_sp._UNC401CLCol	Actinospica_acidiphila_NRRL_B_24431	Streptomyces_sp._HNS054	Streptomyces_sp._SMS_SU21	Streptomyces_sp._ZS0098	Streptomyces_thermocarboxydus_K155	Addition	Core-Accessory-Unique	COG	%Similarity_COG	Score_COG	Evalue_COG	KEGG	%Similarity_KEGG	Score_KEGG	Evalue_KEGG
2063	OSC70221.1	-	histidine kinase	1	1	1	1	1	1	1	1	1	1	1	11	CORE	COG4564_T#	29.1	89	2.74E-17	NA	28.9	78	2.21E-13
2064	OSC68773.1	-	putative lipid II flippase FtsW	1	1	1	1	1	1	1	1	1	1	1	11	CORE	COG0772_D#	42.3	297	1.13E-79	K03588	42.3	297	3.02E-79
2065	Prokka:CIODPCDJ_02676	eno	Enolase	1	1	1	1	1	1	1	1	1	1	1	11	CORE	COG0148_G#	74.8	619	1.3E-176	K01689	71.5	597	1.08E-169
2066	WP_052183294.1	-	MATE family efflux transporter	1	0	0	0	0	1	0	0	0	0	1	3	Accessory	COG0534_V#	25.1	100	1.56E-20	NA	26.3	93	5.08E-18
2067	OSC57292.1	-	FAD-binding protein	0	1	0	0	0	0	0	0	0	0	0	1	Unique								
2068	Prokka:JAPCLKMA_02604	-	hypothetical protein	1	0	0	0	0	0	0	0	0	0	0	1	Unique	COG0463_M#	33.9	124	1.01E-27	NA	31.9	125	1.57E-27
2069	WP_078895141.1	-	M48 family metalloprotease	0	0	1	1	0	0	0	1	1	1	0	5	Accessory	COG0737_F#	34.7	60	1.36E-08	NA	42	107	2.6E-22
2070	WP_122216271.1	-	HAMP domain-containing histidine kinase	0	0	0	0	0	0	0	0	0	1	0	1	Unique	COG0642_T#	28.7	111	6.73E-24	K07653	26.6	110	5.23E-23
2071	WP_033276034.1	-	L-serine ammonia-lyase	1	1	1	1	1	1	1	1	1	1	1	11	CORE	COG1760_E#	68.8	616	8.41E-176	K01752	54.5	486	2.06E-136
2072	WP_122213890.1	-	polysaccharide biosynthesis protein	1	0	1	1	1	1	1	1	1	1	1	10	Accessory								
2073	AXI88178.1	-	site-specific integrase	0	0	0	1	1	0	0	0	0	0	0	2	Accessory								

cluster	GI	gene	Annotation	Streptomyces_thermocarboxydus_BPSAC147	Streptomyces_sp._4F	Streptomyces_sp._BSE7F	Streptomyces_sp._ETH9427	Streptomyces_sp._FxanaD5	Streptomyces_sp._UNC401CLCol	Actinospica_acidiphila_NRRL_B_24431	Streptomyces_sp._HNS054	Streptomyces_sp._SMS_SU21	Streptomyces_sp._ZS0098	Streptomyces_thermocarboxydus_K155	Addition	Core-Accessory-Unique	COG	%Similarity_COG	Score_COG	Evalue_COG	KEGG	%Similarity_KEGG	Score_KEGG	Evalue_KEGG
2074	Prokka:JAPCLKMA_02322	-	hypothetical protein	1	0	0	0	1	1	1	0	0	0	1	5	Accessory	COG1653_G#	30.5	204	9.96E-52				
2075	WP_019524945.1	-	hypothetical protein	0	0	0	0	1	0	0	0	0	0	0	1	Unique								
2076	PWE07246.1	-	VanZ family protein	0	0	1	0	0	0	0	0	0	0	0	1	Unique								
2077	OSC61141.1	-	MFS transporter	0	1	0	0	0	0	0	0	0	0	0	1	Unique	COG0477_GEPR#	42.2	345	2.77E-94	NA	37	282	5.89E-75
2078	Prokka:JAPCLKMA_00082	-	hypothetical protein	1	1	1	1	1	1	1	1	1	1	1	11	CORE	COG1721_R#	28.2	83	1.96E-15	NA	28.2	83	5.23E-15
2079	WP_078504447.1	-	Rieske 2Fe-2S domain-containing protein	1	0	0	0	1	1	0	0	0	0	1	4	Accessory	COG4638_PR#	38.4	294	7.31E-79	K05708	33.3	124	3.49E-27
2080	Prokka:JAPCLKMA_05996	-	hypothetical protein	1	0	0	0	0	0	0	0	0	0	0	1	Unique								
2081	WP_048454850.1	-	FAD-dependent oxidoreductase	1	1	1	1	1	1	1	1	1	1	1	11	CORE	COG0493_ER#	63.1	590	3.78E-168	K00528	44.8	379	4.61E-104
2082	WP_019521481.1	-	MFS transporter	0	0	0	0	1	1	1	1	0	1	1	6	Accessory	COG0477_GEPR#	33	219	2.29E-56	K08369	25.7	116	7.28E-25
2083	WP_019521498.1	-	bifunctional o-acetylhomoserine/o-acetylserine sulfhydrylase	1	0	0	0	1	1	1	0	0	0	1	5	Accessory	COG2873_E#	66.5	587	4.17E-167	K01740	66.5	587	1.11E-166

cluster	GI	gene	Annotation	Streptomyces_thermocarboxydus_BPSAC147	Streptomyces_sp._4F	Streptomyces_sp._BSE7F	Streptomyces_sp._ETH9427	Streptomyces_sp._FxanaD5	Streptomyces_sp._UNC401CLCol	Actinospica_acidiphila_NRRL_B_24431	Streptomyces_sp._HNS054	Streptomyces_sp._SMS_SU21	Streptomyces_sp._ZS0098	Streptomyces_thermocarboxydus_K155	Addition	Core-Accessory-Unique	COG	%Similarity_COG	Score_COG	Evalue_COG	KEGG	%Similarity_KEGG	Score_KEGG	Evalue_KEGG
2084	WP_102638966.1	-	ABC transporter permease subunit	1	1	1	1	1	1	1	1	1	1	1	11	CORE	COG1175_G#	42.4	127	9.06E-29	K10233	42.4	127	2.42E-28
2085	PWE07368.1	-	pyridine nucleotide-disulfide oxidoreductase	0	0	1	1	0	0	1	1	1	1	0	6	Accessory					NA	29.8	155	1.41E-36
2086	Prokka:JAPCLKMA_03405	prfB	Peptide chain release factor 2	1	1	1	1	1	1	1	1	1	1	1	11	CORE	COG1186_J#	66.9	505	1.6E-142	K02836	66.9	505	4.27E-142
2087	WP_048460092.1	-	MFS transporter	1	0	1	1	1	1	1	1	1	1	1	10	Accessory	COG0477_GEPR#	32.8	229	2.89E-59	NA	32.8	229	7.71E-59
2088	WP_033276282.1	-	cytochrome P450	0	0	0	0	0	0	1	0	0	0	0	1	Unique					K09832	26.4	53	0.00000757
2089	WP_026244294.1	-	membrane protein	1	1	0	0	1	1	0	0	0	0	1	5	Accessory					NA	29.9	51	0.0000288
2090	PWE07812.1	-	phosphomannomutase/phosphoglucomutase	1	1	1	1	1	1	1	1	1	1	1	11	CORE	COG1109_G#	65	545	2.38E-154	K01840	65	545	6.35E-154
2091	Prokka:CIODPCDJ_01490	-	hypothetical protein	1	1	1	1	1	1	1	1	1	1	1	11	CORE	COG1653_G#	27.9	142	4.64E-33				
2092	OSC69584.1	-	MFS transporter	0	1	0	0	0	0	0	0	0	0	0	1	Unique	COG0477_GEPR#	24.9	139	3.93E-32	NA	25.9	113	4.72E-24
2093	WP_033275544.1	-	hypothetical protein	1	0	1	1	1	1	1	1	1	0	1	9	Accessory								
2094	Prokka:JAPCLKMA_00401	yycB	putative transporter YycB	1	1	1	1	1	1	1	1	1	1	1	11	CORE	COG2807_P#	38.7	264	8.1E-70	K03449	36.5	242	8.8E-63

cluster	GI	gene	Annotation	Streptomyces_thermocarboxydus_BPSAC147	Streptomyces_sp._4F	Streptomyces_sp._BSE7F	Streptomyces_sp._ETH9427	Streptomyces_sp._FxanaD5	Streptomyces_sp._UNC401CLCol	Actinospica_acidiphila_NRRL_B_24431	Streptomyces_sp._HNS054	Streptomyces_sp._SMS_SU21	Streptomyces_sp._ZS0098	Streptomyces_thermocarboxydus_K155	Addition	Core-Accessory-Unique	COG	%Similarity_COG	Score_COG	Evalue_COG	KEGG	%Similarity_KEGG	Score_KEGG	Evalue_KEGG
2095	Prokka:JAPCLKMA_02742	-	hypothetical protein	1	0	0	0	0	0	0	0	0	0	0	1	Unique	COG3344_L#	28.5	108	5.69E-23	NA	28.5	108	1.52E-22
2096	Prokka:CIODPCDJ_06657	ptlI	Pentalenene oxygenase	0	0	0	0	1	1	0	0	0	0	1	3	Accessory	COG2124_Q#	32.2	235	5.26E-61	K15747	27.4	186	4.39E-46
2097	Prokka:JAPCLKMA_02736	-	hypothetical protein	1	0	0	0	0	0	0	0	0	0	0	1	Unique								
2098	WP_146217793.1	-	sugar transferase	0	0	0	0	0	0	0	0	1	0	0	1	Unique	COG2148_M#	44.6	199	2.44E-50	NA	44.6	199	6.53E-50
2099	WP_048456446.1	-	dipeptidase	1	0	1	1	1	1	1	1	1	1	1	10	Accessory	COG0624_E#	47.8	400	7.22E-111	NA	42.4	332	4.94E-90
2100	WP_122215070.1	-	homogentisate 1,2-dioxygenase	1	1	1	1	1	1	1	1	1	1	1	11	CORE	COG3508_Q#	63.8	547	6.24E-155	K00451	52.1	458	7.79E-128
2101	WP_006132297.1	pafA	Pup--protein ligase	1	1	1	1	1	1	1	1	1	1	1	11	CORE					K13571	73.5	697	7.63E-200
2102	WP_033272326.1	-	hypothetical protein	1	1	1	1	1	1	1	1	1	1	1	11	CORE								
2103	WP_048456945.1	glnA	type I glutamate--ammonia ligase	1	1	1	1	1	1	1	1	1	1	1	11	CORE	COG0174_E#	68.6	624	3.08E-178	K01915	68.6	624	8.21E-178
2104	Prokka:JAPCLKMA_04713	ntaA_3	Nitrilotriacetate monooxygenase component A	1	0	0	0	1	1	1	0	0	0	1	5	Accessory	COG2141_C#	53.8	476	1.35E-133	NA	47.5	402	5.08E-111
2105	WP_019525549.1	-	sensor histidine kinase	0	0	0	0	1	1	0	0	0	0	1	3	Accessory								
2106	Prokka:JAPCLKMA_05631	-	hypothetical protein	1	0	0	0	0	0	0	0	0	0	0	1	Unique								

cluster	GI	gene	Annotation	Streptomyces_thermocarboxydus_BPSAC147	Streptomyces_sp._4F	Streptomyces_sp._BSE7F	Streptomyces_sp._ETH9427	Streptomyces_sp._FxanaD5	Streptomyces_sp._UNC401CLCol	Actinospica_acidiphila_NRRL_B_24431	Streptomyces_sp._HNS054	Streptomyces_sp._SMS_SU21	Streptomyces_sp._ZS0098	Streptomyces_thermocarboxydus_K155	Addition	Core-Accessory-Unique	COG	%Similarity_COG	Score_COG	Evalue_COG	KEGG	%Similarity_KEGG	Score_KEGG	Evalue_KEGG
2107	WP_122213450.1	-	nicotinate phosphoribosyltransferase	1	0	0	1	1	1	0	1	1	1	1	8	Accessory	COG1488_H#	46.3	347	9.48E-95	K00763	46.3	347	2.53E-94
2108	WP_078894974.1	-	hypothetical protein	0	0	0	0	0	0	0	1	0	0	0	1	Unique								
2109	OSC61018.1	-	arginine-ornithine antiporter	0	1	0	0	0	0	0	0	0	0	0	1	Unique	COG0531_E#	54.9	486	7.66E-137	K03758	40.7	342	6.23E-93
2110	OSC72001.1	-	MFS transporter	1	1	0	1	1	1	1	1	1	1	1	10	Accessory	COG2223_P#	50.1	437	5.33E-122	K02575	50.1	437	1.42E-121
2111	WP_033275527.1	-	undecaprenyl/decaprenyl-phosphate alpha-N-acetylglucosaminyl 1-phosphate transferase	1	1	1	1	1	1	1	1	1	1	1	11	CORE	COG0472_M#	45.5	295	3.26E-79	K02851	43	290	3.66E-77
2112	WP_019524653.1	-	FAD-dependent oxidoreductase	1	1	1	1	1	1	1	1	1	1	1	11	CORE	COG0493_ER#	46.2	364	4.38E-100	K00528	46.2	364	1.17E-99
2113	WP_033272446.1	-	phosphoglucosamine mutase	1	1	1	1	1	1	1	1	1	1	1	11	CORE	COG1109_G#	61.4	528	2.99E-149	K03431	60.7	514	9.14E-145
2114	WP_033274722.1	-	extracellular solute-binding protein	1	1	0	1	1	1	1	1	1	1	1	10	Accessory	COG1653_G#	31.7	215	5.61E-55				
2115	OSC71402.1	-	MFS transporter	0	1	0	0	0	0	0	0	0	0	0	1	Unique	COG0477_GEPR#	31.8	223	1.58E-57	K08225	26.3	126	7.01E-28

cluster	GI	gene	Annotation	Streptomyces_thermocarboxydus_BPSAC147	Streptomyces_sp._4F	Streptomyces_sp._BSE7F	Streptomyces_sp._ETH9427	Streptomyces_sp._FxanaD5	Streptomyces_sp._UNC401CLCol	Actinospica_acidiphila_NRRL_B_24431	Streptomyces_sp._HNS054	Streptomyces_sp._SMS_SU21	Streptomyces_sp._ZS0098	Streptomyces_thermocarboxydus_K155	Addition	Core-Accessory-Unique	COG	%Similarity_COG	Score_COG	Evalue_COG	KEGG	%Similarity_KEGG	Score_KEGG	Evalue_KEGG
2116	AXI86651.1	-	glucarate dehydratase	1	1	1	1	1	1	1	1	1	1	1	11	CORE	COG4948_MR#	47.5	383	1.19E-105	K13918	36.1	243	3.01E-63
2117	WP_102638821.1	-	acetyl-CoA carboxylase	1	1	1	1	1	1	1	1	1	1	1	11	CORE	COG0825_I#	36.9	139	2.29E-32	K01963	36.8	206	6.96E-52
2118	Prokka:CIODPCDJ_00034	proP_1	Proline/betaine transporter	1	1	0	0	1	1	0	0	0	0	1	5	Accessory	COG0477_GEPR#	51.6	443	1.27E-123	K08172	32.9	243	3.01E-63
2119	OSC55537.1	-	hypothetical protein	0	1	0	0	0	0	0	0	0	0	0	1	Unique								
2120	OSC63331.1	-	sugar transporter	0	1	0	0	0	0	0	0	0	0	0	1	Unique	COG1653_G#	32.4	205	5.8E-52				
2121	WP_102641775.1	-	ATP-binding protein	1	1	1	1	1	1	1	1	1	1	1	11	CORE					NA	23.8	50	0.0000374
2122	OSC73188.1	-	two-component sensor histidine kinase	0	1	0	0	0	0	0	0	0	0	0	1	Unique	COG0642_T#	40.3	319	1.62E-86	NA	32.1	169	5.52E-41
2123	AXI89994.1	-	SGNH/GDSL hydrolase family protein	0	0	0	1	0	0	0	1	0	1	0	3	Accessory								
2124	OSC59664.1	-	hypothetical protein	0	1	1	1	0	0	1	1	1	1	0	7	Accessory								
2125	OSC59835.1	-	hypothetical protein	0	1	0	0	0	0	0	0	0	0	0	1	Unique								
2126	AXI87197.1	-	aspartate aminotransferase family protein	1	1	1	1	1	1	1	1	1	1	1	11	CORE	COG0160_E#	51	444	4.34E-124	K16871	33.2	244	2.3E-63

cluster	GI	gene	Annotation	Streptomyces_thermocarboxydus_BPSAC147	Streptomyces_sp._4F	Streptomyces_sp._BSE7F	Streptomyces_sp._ETH9427	Streptomyces_sp._FxanaD5	Streptomyces_sp._UNC401CLCol	Actinospica_acidiphila_NRRL_B_24431	Streptomyces_sp._HNS054	Streptomyces_sp._SMS_SU21	Streptomyces_sp._ZS0098	Streptomyces_thermocarboxydus_K155	Addition	Core-Accessory-Unique	COG	%Similarity_COG	Score_COG	Evalue_COG	KEGG	%Similarity_KEGG	Score_KEGG	Evalue_KEGG
2127	WP_028960425.1	-	FAD-dependent oxidoreductase	1	0	0	0	1	1	0	0	0	0	1	4	Accessory	COG0446_R#	63.4	54	0.00000165	K00382	37.5	50	0.0000637
2128	Prokka:CIODPCDJ_03273	dinF	DNA damage-inducible protein F	1	1	1	1	1	1	1	1	1	1	1	11	CORE	COG0534_V#	41.2	299	2.95E-80	NA	41	299	6.02E-80
2129	OSC69794.1	-	hypothetical protein	0	1	0	0	0	0	0	0	0	0	0	1	Unique								
2130	OSC57288.1	-	D-alanyl-D-alanine carboxypeptidase/D-alanyl-D-alanine-endopeptidase	0	1	0	0	0	0	0	0	0	0	0	1	Unique	COG2027_M#	29.3	151	5.82E-36	K07259	35.9	105	1.67E-21
2131	Prokka:JAPCLKMA_05685	-	hypothetical protein	1	0	0	0	0	0	0	0	0	0	0	1	Unique								
2132	WP_102640261.1	-	lysine N(6)-hydroxylase/L-ornithine N(5)-oxygenase family protein	0	0	1	1	0	0	0	1	1	1	0	5	Accessory	COG3486_Q#	38.2	278	4.12E-74				
2133	WP_026243387.1	-	3-deoxy-7-phosphoheptulonate synthase class II	1	1	1	1	1	1	1	1	1	1	1	11	CORE	COG3200_E#	57.4	515	2.62E-145	K01626	58	499	3.04E-140
2134	OSC71348.1	-	sulfate adenylyltransferase	1	1	1	1	1	1	1	1	1	1	1	11	CORE	COG2895_P#	56.2	455	2.46E-127	K00955	56.2	455	6.57E-127

cluster	GI	gene	Annotation	Streptomyces_thermocarboxydus_BPSAC147	Streptomyces_sp._4F	Streptomyces_sp._BSE7F	Streptomyces_sp._ETH9427	Streptomyces_sp._FxanaD5	Streptomyces_sp._UNC401CLCol	Actinospica_acidiphila_NRRL_B_24431	Streptomyces_sp._HNS054	Streptomyces_sp._SMS_SU21	Streptomyces_sp._ZS0098	Streptomyces_thermocarboxydus_K155	Addition	Core-Accessory-Unique	COG	%Similarity_COG	Score_COG	Evalue_COG	KEGG	%Similarity_KEGG	Score_KEGG	Evalue_KEGG
2135	Prokka:CIODPCDJ_04572	dasA_3	Diacetylchitobiose binding protein DasA	1	0	1	1	1	1	1	1	1	1	1	10	Accessory	COG1653_G#	40.9	346	1.23E-94	K17318	22.3	70	4.55E-11
2136	AXI89165.1	-	hypothetical protein	0	0	0	1	0	0	0	1	0	1	0	3	Accessory	COG2205_T#	43.3	245	2.96E-64	K07646	43.3	245	7.9E-64
2137	OSC64978.1	-	L-lysine 6-monooxygenase	0	1	0	0	0	0	0	0	0	0	0	1	Unique	COG3486_Q#	38.1	276	1.57E-73				
2138	PWE11324.1	-	HD domain-containing protein	1	1	1	1	1	1	1	1	1	1	1	11	CORE	COG2206_T#	26.6	55	7.39E-07	NA	26.6	55	0.00000197
2139	WP_033273697.1	-	OFA family MFS transporter	1	0	1	1	1	1	1	1	1	1	1	10	Accessory	COG0477_GEPR#	50.5	439	1.39E-122	NA	49.7	435	5.37E-121
2140	WP_053076907.1	-	DUF2254 domain-containing protein	0	0	0	1	0	0	0	1	0	0	0	2	Accessory	COG4325_S#	36.6	217	8.61E-56	NA	36	217	3E-55
2141	Prokka:CIODPCDJ_04350	yeeO	putative FMN/FAD exporter YeeO	1	0	0	0	1	1	0	0	0	0	1	4	Accessory	COG0534_V#	33.2	141	1.02E-32	NA	26.9	120	3.83E-26
2142	WP_102641031.1	-	MFS transporter	1	1	0	1	1	1	1	1	1	1	1	10	Accessory	COG0477_GEPR#	26.3	74	1.18E-12				
2143	Prokka:JAPCLKMA_05687	-	hypothetical protein	1	0	0	0	0	0	0	0	0	0	0	1	Unique								
2144	Prokka:CIODPCDJ_02445	-	hypothetical protein	0	0	0	0	0	1	0	0	0	0	1	2	Accessory	COG0270_L#	35.4	88	7.88E-17	K00558	26.5	101	2.4E-20
2145	WP_033275977.1	-	hypothetical protein	1	1	1	1	1	1	1	1	1	1	1	11	CORE	COG4371_S#	44.7	44	0.000999	NA	39.7	74	4.11E-12

cluster	GI	gene	Annotation	Streptomyces_thermocarboxydus_BPSAC147	Streptomyces_sp._4F	Streptomyces_sp._BSE7F	Streptomyces_sp._ETH9427	Streptomyces_sp._FxanaD5	Streptomyces_sp._UNC401CLCol	Actinospica_acidiphila_NRRL_B_24431	Streptomyces_sp._HNS054	Streptomyces_sp._SMS_SU21	Streptomyces_sp._ZS0098	Streptomyces_thermocarboxydus_K155	Addition	Core-Accessory-Unique	COG	%Similarity_COG	Score_COG	Evalue_COG	KEGG	%Similarity_KEGG	Score_KEGG	Evalue_KEGG
2146	OSC57353.1	-	GNAT family N-acetyltransferase	1	1	1	1	1	1	1	1	1	1	1	11	CORE								
2147	WP_033277652.1	-	hypothetical protein	0	0	0	0	0	0	1	0	0	0	0	1	Unique	COG0666_R#	34.7	44	0.000999	NA	27.4	50	0.0000635
2148	OSC57734.1	-	RNA methyltransferase	1	1	1	1	1	1	1	1	1	1	1	11	CORE	COG2265_J#	37.4	246	2.26E-64	NA	37.4	246	6.04E-64
2149	WP_107080935.1	-	hypothetical protein	1	1	0	1	1	1	0	1	1	1	1	9	Accessory								
2150	AXI88380.1	-	decaprenylphosphoryl-beta-D-ribose oxidase	1	1	1	1	1	1	1	1	1	1	1	11	CORE	COG0277_C#	47.6	397	7.91E-110	K16653	47.6	397	2.11E-109
2151	OSC68523.1	-	DNA (cytosine-5-)-methyltransferase	0	1	0	0	0	0	0	1	0	0	0	2	Accessory	COG0270_L#	27.6	83	2.53E-15	K00558	28.4	71	2.03E-11
2152	WP_102638585.1	ccrA	crotonyl-CoA carboxylase/reductase	0	0	1	0	0	0	0	0	1	0	0	2	Accessory	COG0604_CR#	38.9	253	1.41E-66	NA	28	159	7.42E-38
2153	WP_028958803.1	-	extracellular solute-binding protein	1	1	1	1	1	1	1	1	1	1	1	11	CORE	COG1653_G#	28.4	118	5.43E-26	K10117	19.3	50	0.0000485
2154	OSC71262.1	-	NADH-quinone oxidoreductase subunit F	1	1	1	1	1	1	1	1	1	1	1	11	CORE	COG1894_C#	75.7	698	1.66E-200	K00335	75.7	698	4.44E-200
2155	OSC71286.1	-	ammonia channel protein	1	1	1	1	1	1	1	1	1	1	1	11	CORE	COG0004_P#	56.7	469	1.25E-131	K03320	56.7	470	2.57E-131

cluster	GI	gene	Annotation	Streptomyces_thermocarboxydus_BPSAC147	Streptomyces_sp._4F	Streptomyces_sp._BSE7F	Streptomyces_sp._ETH9427	Streptomyces_sp._FxanaD5	Streptomyces_sp._UNC401CLCol	Actinospica_acidiphila_NRRL_B_24431	Streptomyces_sp._HNS054	Streptomyces_sp._SMS_SU21	Streptomyces_sp._ZS0098	Streptomyces_thermocarboxydus_K155	Addition	Core-Accessory-Unique	COG	%Similarity_COG	Score_COG	Evalue_COG	KEGG	%Similarity_KEGG	Score_KEGG	Evalue_KEGG
2156	WP_147474386.1	-	hypothetical protein	0	0	0	0	0	0	0	0	0	1	0	1	Unique								
2157	WP_026243767.1	-	hypothetical protein	0	0	0	0	1	0	0	0	0	0	0	1	Unique								
2158	Prokka:CIODPCDJ_00267	pksS_1	Polyketide biosynthesis cytochrome P450 PksS	1	0	1	1	1	1	1	1	1	1	1	10	Accessory	COG2124_Q#	30	141	7.83E-33	K00517	26.6	104	2.83E-21
2159	OSC67644.1	-	molybdopterin molybdenumtransferase MoeA	1	1	1	1	1	1	1	1	1	1	1	11	CORE	COG0303_H#	45.9	336	1.66E-91	K03750	45.9	336	4.43E-91
2160	WP_048458871.1	brxD	BREX system ATP-binding protein BrxD	1	0	0	0	0	0	0	1	1	0	0	3	Accessory								
2161	WP_048457853.1	-	hypothetical protein	1	1	0	1	1	1	1	1	1	1	1	10	Accessory								
2162	WP_048457823.1	-	O-antigen ligase family protein	1	1	1	1	1	1	1	1	1	1	1	11	CORE								
2163	WP_048460579.1	murA	UDP-N-acetylglucosamine 1-carboxyvinyltransferase	0	1	1	1	1	1	1	1	1	1	1	10	Accessory	COG0766_M#	44.4	355	3.44E-97	K00790	44.1	346	5.58E-94
2164	OSC69122.1	-	MFS transporter	0	1	0	0	0	0	0	0	0	0	0	1	Unique	COG0477_GEPR#	35.2	210	1.05E-53	NA	29.2	165	1.03E-39

cluster	GI	gene	Annotation	Streptomyces_thermocarboxydus_BPSAC147	Streptomyces_sp._4F	Streptomyces_sp._BSE7F	Streptomyces_sp._ETH9427	Streptomyces_sp._FxanaD5	Streptomyces_sp._UNC401CLCol	Actinospica_acidiphila_NRRL_B_24431	Streptomyces_sp._HNS054	Streptomyces_sp._SMS_SU21	Streptomyces_sp._ZS0098	Streptomyces_thermocarboxydus_K155	Addition	Core-Accessory-Unique	COG	%Similarity_COG	Score_COG	Evalue_COG	KEGG	%Similarity_KEGG	Score_KEGG	Evalue_KEGG
2165	WP_122212664.1	-	nicotinate phosphoribosyltransferase	1	1	1	1	1	1	1	1	1	1	1	11	CORE	COG1488_H#	55.8	447	5.09E-125	K00763	55.8	446	3.03E-124
2166	WP_028960764.1	-	DUF2252 domain-containing protein	1	1	1	1	1	1	1	1	1	1	1	11	CORE	COG4320_S#	29.9	186	2.76E-46				
2167	WP_122214153.1	-	sodium:proton antiporter	0	0	1	1	0	0	0	1	1	1	0	5	Accessory	COG0025_P#	29.8	137	1.13E-31	NA	21.6	102	1.4E-20
2168	OSC70552.1	-	hypothetical protein	0	1	0	0	0	0	0	0	0	0	0	1	Unique								
2169	WP_122215946.1	-	CapA family protein	0	0	1	1	0	0	0	1	1	1	0	5	Accessory	COG2843_M#	31.6	140	1.33E-32	K07282	29.3	50	0.0000632
2170	OSC70622.1	-	1,4-beta-xylanase	0	1	0	0	0	0	0	0	0	0	0	1	Unique	COG3693_G#	42.7	244	8.56E-64	NA	29.7	128	1.4E-28
2171	WP_102642185.1	-	tetratricopeptide repeat protein	1	1	0	1	1	1	1	1	1	1	1	10	Accessory	COG1396_K#	35	44	0.0013	NA	27.9	47	0.000313
2172	WP_122214548.1	-	formimidoylglutamate deiminase	1	1	1	1	1	1	1	1	1	1	1	11	CORE	COG0402_FR#	37.4	248	4.53E-65	NA	31.9	202	9.93E-51
2173	WP_048457303.1	-	UDP-glucose/GDP-mannose dehydrogenase family protein	1	1	1	1	1	1	1	1	1	1	1	11	CORE	COG1004_M#	61.9	541	2.59E-153	K00012	61.1	533	2.46E-150
2174	WP_033273768.1	ccrA	crotonyl-CoA carboxylase/reductase	1	1	0	1	1	1	1	1	1	1	1	10	Accessory	COG0604_CR#	38.5	298	4.98E-80	K00344	27.6	99	6.94E-20

cluster	GI	gene	Annotation	Streptomyces_thermocarboxydus_BPSAC147	Streptomyces_sp._4F	Streptomyces_sp._BSE7F	Streptomyces_sp._ETH9427	Streptomyces_sp._FxanaD5	Streptomyces_sp._UNC401CLCol	Actinospica_acidiphila_NRRL_B_24431	Streptomyces_sp._HNS054	Streptomyces_sp._SMS_SU21	Streptomyces_sp._ZS0098	Streptomyces_thermocarboxydus_K155	Addition	Core-Accessory-Unique	COG	%Similarity_COG	Score_COG	Evalue_COG	KEGG	%Similarity_KEGG	Score_KEGG	Evalue_KEGG
2175	WP_048460152.1	-	sugar ABC transporter substrate-binding protein	1	0	0	0	1	1	1	1	0	1	1	7	Accessory	COG1653_G#	27.7	113	1.74E-24	K05813	23.1	86	1.04E-15
2176	Prokka:CIODPCDJ_01768	-	hypothetical protein	1	1	1	1	1	1	1	1	1	1	1	11	CORE					K16469	28.6	48	0.00024
2177	OSC73224.1	-	iron transporter	1	1	0	1	1	1	1	1	1	1	1	10	Accessory	COG1914_P#	21.7	87	1.34E-16	NA	22.9	82	8.78E-15
2178	Prokka:JAPCLKMA_03771	-	hypothetical protein	1	0	0	0	0	0	0	0	0	0	0	1	Unique								
2179	WP_102637311.1	-	sugar ABC transporter substrate-binding protein	0	0	1	0	0	0	0	1	1	1	0	4	Accessory	COG1653_G#	34.6	235	3.04E-61				
2180	WP_102637410.1	-	hypothetical protein	0	0	0	0	0	0	0	0	1	0	0	1	Unique	COG0732_V#	27.9	138	8.62E-32	K01154	23.2	75	1.4E-12
2181	WP_106432539.1	-	hypothetical protein	1	1	1	1	1	1	1	1	1	1	1	11	CORE	COG1674_D#	35.2	71	9.9E-12	NA	25.3	59	1.04E-07
2182	Prokka:CIODPCDJ_06667	-	hypothetical protein	0	1	1	1	1	1	0	1	1	1	1	9	Accessory	COG1253_R#	38.7	300	1.31E-80	NA	37.1	275	7.07E-73
2183	OSC71050.1	-	aspartate aminotransferase family protein	0	1	0	0	0	0	1	0	0	0	0	2	Accessory	COG0161_H#	31.6	189	2.5E-47	NA	30.4	157	2.81E-37
2184	PWE11498.1	-	aminotransferase	0	0	1	1	0	0	0	1	1	1	0	5	Accessory	COG0520_E#	29.1	80	2.13E-14	K11717	21.8	64	4.21E-09
2185	WP_033275004.1	-	hypothetical protein	1	1	1	1	1	1	1	1	1	1	1	11	CORE								
2186	AXI90895.1	-	IS4 family transposase	0	0	0	1	0	0	0	0	0	0	0	1	Unique	COG3385_L#	26.2	145	4.12E-34	K07495	26.2	145	1.1E-33

cluster	GI	gene	Annotation	Streptomyces_thermocarboxydus_BPSAC147	Streptomyces_sp._4F	Streptomyces_sp._BSE7F	Streptomyces_sp._ETH9427	Streptomyces_sp._FxanaD5	Streptomyces_sp._UNC401CLCol	Actinospica_acidiphila_NRRL_B_24431	Streptomyces_sp._HNS054	Streptomyces_sp._SMS_SU21	Streptomyces_sp._ZS0098	Streptomyces_thermocarboxydus_K155	Addition	Core-Accessory-Unique	COG	%Similarity_COG	Score_COG	Evalue_COG	KEGG	%Similarity_KEGG	Score_KEGG	Evalue_KEGG
2187	PWE09164.1	-	carboxypeptidase	0	0	1	1	0	0	0	1	1	1	0	5	Accessory	COG2866_E#	29.6	101	8.92E-21	NA	33.9	150	3.42E-35
2188	AXI85263.1	-	hypothetical protein	0	0	0	1	0	0	0	0	0	0	0	1	Unique								
2189	WP_122216073.1	-	6-phospho-beta-glucosidase	1	1	0	0	1	1	1	1	1	1	1	9	Accessory	COG1486_G#	36.4	264	6.09E-70	K01222	31.4	208	1.06E-52
2190	AXI90935.1	-	ATPase	0	0	1	1	0	0	0	1	1	1	0	5	Accessory	COG0168_P#	40	302	3.44E-81	K03498	40	302	9.19E-81
2191	WP_084207071.1	-	recombinase family protein	0	0	0	0	0	0	1	0	0	1	0	2	Accessory								
2192	OSC58629.1	-	MFS transporter	0	1	1	1	0	0	0	1	1	1	0	6	Accessory	COG0477_GEPR#	31.1	117	1.57E-25	NA	29.9	110	3.92E-23
2193	Prokka:CIODPCDJ_01015	ribBA_1	Riboflavin biosynthesis protein RibBA	1	1	1	1	1	1	1	1	1	1	1	11	CORE	COG0807_H#	65.7	274	5.88E-73	K14652	62.3	498	8.74E-140
2194	WP_102640283.1	-	sensor histidine kinase	1	0	1	0	1	1	1	0	1	0	1	7	Accessory	COG4585_T#	31.9	110	1.92E-23	NA	31.9	110	5.13E-23
2195	Prokka:JAPCLKMA_02757	lnrJ	Sensor histidine kinase LnrJ	1	1	1	1	1	1	1	1	1	1	1	11	CORE	COG4585_T#	25.2	62	3.51E-09	K07778	34.1	60	4.66E-08
2196	Prokka:JAPCLKMA_04936	-	hypothetical protein	1	0	0	0	0	0	0	0	0	0	0	1	Unique	COG1106_R#	28.5	139	2.26E-32				
2197	OSC67605.1	-	sugar ABC transporter substrate-binding protein	1	1	1	1	1	1	1	1	1	1	1	11	CORE	COG1653_G#	28.6	151	9.81E-36				
2198	WP_033273449.1	-	hypothetical protein	1	0	1	0	1	1	1	1	1	0	1	8	Accessory	COG4279_S#	30.9	110	1.92E-23	NA	29.1	83	6.71E-15
2199	WP_028959450.1	-	PH domain-containing protein	1	1	1	1	1	1	1	1	1	1	1	11	CORE	COG3428_S#	27.3	125	5.75E-28	K08981	28.1	79	9.67E-14

cluster	GI	gene	Annotation	Streptomyces_thermocarboxydus_BPSAC147	Streptomyces_sp._4F	Streptomyces_sp._BSE7F	Streptomyces_sp._ETH9427	Streptomyces_sp._FxanaD5	Streptomyces_sp._UNC401CLCol	Actinospica_acidiphila_NRRL_B_24431	Streptomyces_sp._HNS054	Streptomyces_sp._SMS_SU21	Streptomyces_sp._ZS0098	Streptomyces_thermocarboxydus_K155	Addition	Core-Accessory-Unique	COG	%Similarity_COG	Score_COG	Evalue_COG	KEGG	%Similarity_KEGG	Score_KEGG	Evalue_KEGG
2200	Prokka:JAPCLKMA_02037	allB	Allantoinase	1	1	1	1	1	1	1	1	1	1	1	11	CORE	COG0044_F#	45.3	356	1.17E-97	K01466	44.8	367	1.78E-100
2201	WP_037779189.1	-	hypothetical protein	1	1	1	1	1	1	1	1	1	1	1	11	CORE								
2202	WP_026244005.1	-	aminotransferase class I/II-fold pyridoxal phosphate-dependent enzyme	1	1	1	1	1	1	1	1	1	1	1	11	CORE	COG1167_KE#	41.1	285	4.34E-76	NA	25.3	95	1.7E-18
2203	OSC57813.1	-	FAD-linked oxidoreductase	1	1	1	1	1	1	1	1	1	1	1	11	CORE	COG0277_C#	43.9	347	9.31E-95	K00103	43.9	346	3.25E-94
2204	WP_048459747.1	-	pyridoxal-phosphate dependent enzyme	0	1	0	1	1	1	1	1	1	1	1	9	Accessory	COG0031_E#	36.6	158	8.01E-38	K12339	31.4	132	9.62E-30
2205	WP_102639577.1	-	CpaF family protein	1	1	1	1	1	1	1	1	1	1	1	11	CORE	COG4962_U#	45.7	377	8.4E-104	K02283	36.9	240	4.28E-62
2206	PWE11054.1	-	MFS transporter	0	0	1	0	0	0	0	0	0	0	0	1	Unique	COG0477_GEPR#	32.2	167	1.32E-40	NA	32.2	167	3.53E-40
2207	Prokka:CIODPCDJ_05094	-	hypothetical protein	1	0	0	1	1	1	0	1	0	1	1	7	Accessory								
2208	Prokka:JAPCLKMA_02542	bglA_2	Beta-glucosidase A	1	1	0	1	1	1	1	1	1	1	1	10	Accessory	COG2723_G#	42.9	342	2.99E-93	K01188	35.6	277	2.42E-73
2209	PWE11048.1	-	hypothetical protein	0	0	1	0	0	0	0	0	0	0	0	1	Unique								
2210	WP_147474361.1	-	hypothetical protein	0	0	1	0	0	0	0	0	0	1	0	2	Accessory								

cluster	GI	gene	Annotation	Streptomyces_thermocarboxydus_BPSAC147	Streptomyces_sp._4F	Streptomyces_sp._BSE7F	Streptomyces_sp._ETH9427	Streptomyces_sp._FxanaD5	Streptomyces_sp._UNC401CLCol	Actinospica_acidiphila_NRRL_B_24431	Streptomyces_sp._HNS054	Streptomyces_sp._SMS_SU21	Streptomyces_sp._ZS0098	Streptomyces_thermocarboxydus_K155	Addition	Core-Accessory-Unique	COG	%Similarity_COG	Score_COG	Evalue_COG	KEGG	%Similarity_KEGG	Score_KEGG	Evalue_KEGG
2211	Prokka:JAPCLKMA_01042	-	hypothetical protein	1	0	0	1	0	0	0	0	0	0	0	2	Accessory								
2212	WP_033277530.1	-	acyltransferase	1	1	1	1	1	1	1	1	1	1	1	11	CORE								
2213	OSC72620.1	-	4-aminobutyrate--2-oxoglutarate transaminase	1	1	1	1	1	1	1	1	1	1	1	11	CORE	COG0160_E#	63.9	556	7.72E-158	K07250	63.9	556	2.06E-157
2214	WP_078504703.1	-	glycosyltransferase family 4 protein	1	1	1	1	1	1	1	1	1	1	1	11	CORE	COG0438_M#	30	132	4.7E-30	K13668	31.3	102	1.39E-20
2215	Prokka:JAPCLKMA_05949	-	hypothetical protein	1	1	1	1	1	1	1	0	1	1	1	10	Accessory	COG4585_T#	31.4	120	1.85E-26	K07778	31.4	120	4.93E-26
2216	Prokka:JAPCLKMA_01022	pknD_4	Serine/threonine-protein kinase PknD	1	0	0	0	0	0	0	0	0	0	0	1	Unique	COG0515_RTKL#	33.1	158	6.12E-38	NA	41.8	154	3.08E-36
2217	WP_033274837.1	-	LPXTG cell wall anchor domain-containing protein	0	0	0	0	0	0	1	0	0	0	0	1	Unique								
2218	WP_122216684.1	-	hypothetical protein	0	1	1	1	0	0	0	1	0	1	0	5	Accessory								
2219	OSC66247.1	-	Tat pathway signal protein	0	1	0	0	0	0	0	0	0	0	0	1	Unique								

cluster	GI	gene	Annotation	Streptomyces_thermocarboxydus_BPSAC147	Streptomyces_sp._4F	Streptomyces_sp._BSE7F	Streptomyces_sp._ETH9427	Streptomyces_sp._FxanaD5	Streptomyces_sp._UNC401CLCol	Actinospica_acidiphila_NRRL_B_24431	Streptomyces_sp._HNS054	Streptomyces_sp._SMS_SU21	Streptomyces_sp._ZS0098	Streptomyces_thermocarboxydus_K155	Addition	Core-Accessory-Unique	COG	%Similarity_COG	Score_COG	Evalue_COG	KEGG	%Similarity_KEGG	Score_KEGG	Evalue_KEGG
2220	OSC57756.1	-	manganese transporter	0	1	0	0	0	0	0	0	0	0	0	1	Unique	COG1914_P#	23.7	70	1.68E-11	NA	22	77	3.67E-13
2221	WP_159020955.1	-	alpha/beta hydrolase	0	0	0	0	0	0	0	1	0	0	0	1	Unique	COG4782_S#	41.8	213	1.23E-54				
2222	WP_167338211.1	-	glycosyl hydrolase	1	0	0	1	1	1	1	1	0	0	1	7	Accessory								
2223	WP_033273490.1	-	DUF2254 domain-containing protein	0	0	1	1	0	0	1	1	1	1	0	6	Accessory	COG4325_S#	31.5	160	1.61E-38	NA	31.5	160	4.3E-38
2224	OSC61399.1	-	ABC transporter substrate-binding protein	0	1	0	0	0	0	0	0	0	0	0	1	Unique	COG1653_G#	26.3	92	3.16E-18	K02027	22.8	77	4.79E-13
2225	OSC70935.1	-	ATP-grasp domain-containing protein	1	1	1	1	1	1	1	1	1	1	1	11	CORE								
2226	OSC73383.1	-	pectate lyase	1	1	1	1	1	1	1	1	1	1	1	11	CORE	COG3866_G#	41.6	347	7.11E-95	K01728	41.6	347	1.9E-94
2227	WP_122213726.1	-	hypothetical protein	1	1	1	1	1	1	1	1	1	1	1	11	CORE								
2228	AXI87321.1	-	MFS transporter	1	1	1	1	1	1	1	1	1	1	1	11	CORE	COG0477_GEPR#	34.9	217	8.48E-56	NA	21.3	66	6.46E-10
2229	Prokka:CIODPCDJ_06023	pcaB	3-carboxy-cis,cis-muconate cycloisomerase	1	1	0	0	1	1	1	0	0	0	1	6	Accessory	COG0015_F#	43.9	316	1.75E-85	K01756	30.2	168	1.21E-40
2230	Prokka:CIODPCDJ_03091	-	hypothetical protein	1	0	0	1	1	1	1	1	1	1	1	9	Accessory								
2231	WP_122215601.1	-	sugar kinase	1	1	0	1	1	1	1	1	1	1	1	10	Accessory	COG0524_G#	36.3	140	1.72E-32	K00874	34.9	117	4.17E-25

cluster	GI	gene	Annotation	Streptomyces_thermocarboxydus_BPSAC147	Streptomyces_sp._4F	Streptomyces_sp._BSE7F	Streptomyces_sp._ETH9427	Streptomyces_sp._FxanaD5	Streptomyces_sp._UNC401CLCol	Actinospica_acidiphila_NRRL_B_24431	Streptomyces_sp._HNS054	Streptomyces_sp._SMS_SU21	Streptomyces_sp._ZS0098	Streptomyces_thermocarboxydus_K155	Addition	Core-Accessory-Unique	COG	%Similarity_COG	Score_COG	Evalue_COG	KEGG	%Similarity_KEGG	Score_KEGG	Evalue_KEGG
2232	WP_063833256.1	-	hypothetical protein	0	0	1	0	0	0	1	0	1	0	0	3	Accessory								
2233	OSC72209.1	-	UDP-glucose 6-dehydrogenase	0	1	0	0	0	0	0	0	0	0	0	1	Unique	COG0677_M#	37.2	265	3.55E-70	K02472	32.9	200	2.86E-50
2234	WP_028958934.1	-	SpoIIE family protein phosphatase	1	0	0	0	1	1	0	0	0	0	1	4	Accessory	COG2203_T#	44.3	168	7.7E-41	NA	44.3	168	2.06E-40
2235	WP_033276024.1	-	ATP-binding cassette domain-containing protein	1	0	1	1	1	1	1	1	1	1	1	10	Accessory	COG4608_E#	57.1	377	8.36E-104	K10823	57.2	374	1.11E-102
2236	Prokka:JAPCLKMA_05391	-	hypothetical protein	1	1	1	1	1	1	1	1	1	1	1	11	CORE	COG4585_T#	41	185	3.56E-46				
2237	OSC65500.1	-	sugar ABC transporter substrate-binding protein	0	1	0	0	0	0	0	0	0	0	0	1	Unique	COG1653_G#	31.6	214	9.35E-55	K10232	31.6	214	2.5E-54
2238	OSC65549.1	-	hypothetical protein	0	1	0	0	0	0	0	0	0	0	0	1	Unique								
2239	WP_033277150.1	-	glutamate-1-semialdehyde 2,1-aminomutase	1	0	1	1	1	1	1	1	1	1	1	10	Accessory	COG0001_H#	56	466	1.04E-130	K01845	34	208	1.37E-52
2240	OSC71831.1	-	ribonuclease	1	1	1	1	1	1	1	1	1	1	1	11	CORE	COG1875_T#	72.4	614	3.1E-175	K07175	72.2	613	1.41E-174

cluster	GI	gene	Annotation	Streptomyces_thermocarboxydus_BPSAC147	Streptomyces_sp._4F	Streptomyces_sp._BSE7F	Streptomyces_sp._ETH9427	Streptomyces_sp._FxanaD5	Streptomyces_sp._UNC401CLCol	Actinospica_acidiphila_NRRL_B_24431	Streptomyces_sp._HNS054	Streptomyces_sp._SMS_SU21	Streptomyces_sp._ZS0098	Streptomyces_thermocarboxydus_K155	Addition	Core-Accessory-Unique	COG	%Similarity_COG	Score_COG	Evalue_COG	KEGG	%Similarity_KEGG	Score_KEGG	Evalue_KEGG
2241	WP_048455353.1	-	glutathione-dependent formaldehyde dehydrogenase	0	0	0	1	0	0	0	1	0	0	0	2	Accessory	COG1063_ER#	63.2	529	1.31E-149	NA	58	476	2.06E-133
2242	WP_028960006.1	-	membrane protease subunits stomatin/prohibitin	1	0	1	1	1	1	1	1	1	1	1	10	Accessory	COG0330_O#	25.2	69	2.84E-11				
2243	WP_122213793.1	-	PrsW family intramembrane metalloprotease	1	1	1	0	1	1	1	1	1	1	1	10	Accessory								
2244	WP_019523570.1	-	MFS transporter	1	0	0	0	1	1	0	0	0	0	1	4	Accessory	COG0477_GEPR#	35.3	242	4.18E-63	K03761	33.4	232	6.77E-60
2245	WP_102641958.1	-	M20/M25/M40 family metallo-hydrolase	1	1	1	1	1	1	1	1	1	1	1	11	CORE	COG0624_E#	55.9	471	3.24E-132	NA	56	465	6.21E-130
2246	WP_106432480.1	-	VanZ family protein	0	0	0	0	1	1	0	0	0	0	0	2	Accessory								
2247	WP_019523025.1	-	MFS transporter	1	0	0	0	1	1	1	0	0	0	1	5	Accessory	COG0477_GEPR#	41.2	343	1.33E-93	K03761	37.9	288	1.36E-76
2248	OSC62123.1	-	hypothetical protein	0	1	0	0	0	0	0	0	0	0	0	1	Unique	COG3899_R#	28	50	0.0000178	NA	37	54	0.00000431
2249	WP_167338166.1	-	hypothetical protein	1	1	1	1	1	1	1	1	1	1	1	11	CORE	COG3468_MU#	29.5	50	0.0000233	K00901	39.4	60	4.61E-08
2250	OSC62111.1	-	hypothetical protein	0	1	0	0	0	0	0	0	0	0	0	1	Unique								

cluster	GI	gene	Annotation	Streptomyces_thermocarboxydus_BPSAC147	Streptomyces_sp._4F	Streptomyces_sp._BSE7F	Streptomyces_sp._ETH9427	Streptomyces_sp._FxanaD5	Streptomyces_sp._UNC401CLCol	Actinospica_acidiphila_NRRL_B_24431	Streptomyces_sp._HNS054	Streptomyces_sp._SMS_SU21	Streptomyces_sp._ZS0098	Streptomyces_thermocarboxydus_K155	Addition	Core-Accessory-Unique	COG	%Similarity_COG	Score_COG	Evalue_COG	KEGG	%Similarity_KEGG	Score_KEGG	Evalue_KEGG
2251	WP_033274648.1	-	hypothetical protein	1	1	0	1	1	1	1	1	1	1	1	10	Accessory								
2252	WP_048455495.1	-	hypothetical protein	1	1	1	1	1	1	1	1	1	1	1	11	CORE								
2253	WP_051724466.1	-	hypothetical protein	0	0	0	0	0	0	1	0	0	0	0	1	Unique								
2254	AXI89176.1	-	TIGR02678 family protein	0	0	0	1	0	0	0	1	0	0	0	2	Accessory								
2255	WP_122215225.1	-	hypothetical protein	1	0	0	1	1	1	1	1	1	1	1	9	Accessory								
2256	WP_028960981.1	-	MFS transporter	1	1	1	1	1	1	1	1	1	1	1	11	CORE	COG0477_GEPR#	50.2	418	2.49E-116	K08178	31.1	165	1.01E-39
2257	WP_028960997.1	-	triacylglycerol lipase	1	1	1	1	1	1	1	1	1	1	1	11	CORE					NA	29.1	140	4.56E-32
2258	Prokka:CIODPCDJ_02645	amaB	N-carbamoyl-L-amino acid hydrolase	1	1	1	1	1	1	1	1	1	1	1	11	CORE	COG0624_E#	34.5	204	7.38E-52	K18151	30.5	186	5.55E-46
2259	WP_122216682.1	-	helix-turn-helix domain-containing protein	0	0	0	1	0	0	0	1	0	1	0	3	Accessory	COG3903_R#	25.1	73	1.96E-12				
2260	OSC61246.1	-	sugar ABC transporter substrate-binding protein	0	1	0	0	0	0	0	0	0	0	0	1	Unique								
2261	OSC71260.1	-	NADH dehydrogenase subunit D	1	1	1	1	1	1	1	1	1	1	1	11	CORE	COG0649_C#	73.8	657	4.16E-188	K00333	73.8	657	1.11E-187
2262	Prokka:JAPCLKMA_01061	-	hypothetical protein	1	0	1	0	1	1	0	0	1	0	1	6	Accessory	COG5340_K#	33.9	95	4.83E-19	NA	33.9	95	1.29E-18

cluster	GI	gene	Annotation	Streptomyces_thermocarboxydus_BPSAC147	Streptomyces_sp._4F	Streptomyces_sp._BSE7F	Streptomyces_sp._ETH9427	Streptomyces_sp._FxanaD5	Streptomyces_sp._UNC401CLCol	Actinospica_acidiphila_NRRL_B_24431	Streptomyces_sp._HNS054	Streptomyces_sp._SMS_SU21	Streptomyces_sp._ZS0098	Streptomyces_thermocarboxydus_K155	Addition	Core-Accessory-Unique	COG	%Similarity_COG	Score_COG	Evalue_COG	KEGG	%Similarity_KEGG	Score_KEGG	Evalue_KEGG
2263	WP_102640721.1	-	ergothioneine biosynthesis protein EgtB	1	1	1	1	1	1	1	1	1	1	1	11	CORE	COG1262_S#	54.3	451	3.46E-126	K18912	54.3	451	9.23E-126
2264	WP_122215387.1	-	metallophosphoesterase	1	1	1	1	1	1	1	1	1	1	1	11	CORE	COG1408_R#	36.6	174	6.25E-43	K07098	36.7	171	2.41E-41
2265	OSC69249.1	-	hypothetical protein	0	1	0	0	1	0	1	0	0	0	0	3	Accessory	COG4292_S#	30	115	4.5E-25	NA	30	115	1.2E-24
2266	WP_019526566.1	-	hypothetical protein	1	1	1	1	1	1	1	1	1	1	1	11	CORE	COG0515_RTKL#	25.9	51	0.00000797	NA	35.5	202	7.47E-51
2267	OSC73347.1	-	signal peptidase I	1	1	1	1	1	1	1	1	1	1	1	11	CORE	COG0681_U#	42.3	170	1.18E-41	K03100	42.3	170	3.15E-41
2268	WP_033272578.1	-	dicarboxylate/amino acid:cation symporter	1	1	1	1	1	1	1	1	1	1	1	11	CORE	COG1301_C#	55.3	467	6.09E-131	NA	33.8	234	1.77E-60
2269	Prokka:CIODPCDJ_00560	-	hypothetical protein	1	0	0	0	1	1	0	0	0	0	1	4	Accessory	COG1653_G#	24.2	51	0.0000104				
2270	WP_158688219.1	-	hypothetical protein	1	0	0	0	1	1	0	0	0	0	0	3	Accessory								
2271	AXI84597.1	-	carbohydrate ABC transporter substrate-binding protein	0	0	0	1	0	0	0	1	1	0	0	3	Accessory	COG1653_G#	32.1	250	8.96E-66	K10117	32.1	250	2.39E-65
2272	WP_086020175.1	-	lysine N(6)-hydroxylase/L-ornithine N(5)-oxygenase family protein	1	1	1	1	1	1	1	1	1	1	1	11	CORE	COG3486_Q#	54.2	462	1.5E-129				

cluster	GI	gene	Annotation	Streptomyces_thermocarboxydus_BPSAC147	Streptomyces_sp._4F	Streptomyces_sp._BSE7F	Streptomyces_sp._ETH9427	Streptomyces_sp._FxanaD5	Streptomyces_sp._UNC401CLCol	Actinospica_acidiphila_NRRL_B_24431	Streptomyces_sp._HNS054	Streptomyces_sp._SMS_SU21	Streptomyces_sp._ZS0098	Streptomyces_thermocarboxydus_K155	Addition	Core-Accessory-Unique	COG	%Similarity_COG	Score_COG	Evalue_COG	KEGG	%Similarity_KEGG	Score_KEGG	Evalue_KEGG
2273	OSC66692.1	-	MFS transporter	1	1	1	1	0	1	1	1	1	1	1	10	Accessory	COG0477_GEPR#	25.4	147	1.07E-34	NA	25.4	147	2.85E-34
2274	WP_122216607.1	-	MHS family MFS transporter	0	0	0	0	0	0	0	0	0	1	0	1	Unique	COG0477_GEPR#	46.7	407	5.73E-113	NA	35.7	292	7.17E-78
2275	WP_102637416.1	-	hypothetical protein	0	0	0	0	0	0	0	0	1	0	0	1	Unique								
2276	OSC68711.1	-	hypothetical protein	0	1	0	0	0	0	0	0	0	0	0	1	Unique								
2277	WP_028961402.1	-	sugar ABC transporter substrate-binding protein	1	0	1	1	1	1	1	1	1	1	1	10	Accessory	COG1653_G#	29.1	154	1.14E-36				
2278	WP_122213520.1	aroA	3-phosphoshikimate 1-carboxyvinyltransferase	1	1	0	1	1	1	1	1	1	1	1	10	Accessory	COG0128_E#	57	470	7.18E-132	K00800	51.4	390	2.52E-107
2279	WP_019526437.1	-	nucleotide sugar dehydrogenase	1	0	1	1	1	1	1	1	1	1	1	10	Accessory	COG1004_M#	49.5	438	3.95E-122	K00012	35	225	1.07E-57
2280	AXI88051.1	hemL	glutamate-1-semialdehyde-2,1-aminomutase	1	1	1	1	1	1	1	1	1	1	1	11	CORE	COG0001_H#	62.6	545	2.29E-154	K01845	62.6	545	6.12E-154
2281	OSC71095.1	-	1,4-beta-glucanase	0	1	0	0	0	0	0	0	0	0	0	1	Unique					NA	38.4	295	1.1E-78

cluster	GI	gene	Annotation	Streptomyces_thermocarboxydus_BPSAC147	Streptomyces_sp._4F	Streptomyces_sp._BSE7F	Streptomyces_sp._ETH9427	Streptomyces_sp._FxanaD5	Streptomyces_sp._UNC401CLCol	Actinospica_acidiphila_NRRL_B_24431	Streptomyces_sp._HNS054	Streptomyces_sp._SMS_SU21	Streptomyces_sp._ZS0098	Streptomyces_thermocarboxydus_K155	Addition	Core-Accessory-Unique	COG	%Similarity_COG	Score_COG	Evalue_COG	KEGG	%Similarity_KEGG	Score_KEGG	Evalue_KEGG
2282	WP_106432561.1	mycP	type VII secretion-associated serine protease mycosin	1	0	1	0	1	1	1	0	1	0	1	7	Accessory	COG1404_O#	35.3	178	4.32E-44	K14743	35.3	178	1.15E-43
2283	WP_122214145.1	-	FAD-dependent oxidoreductase	0	0	0	1	0	0	0	1	1	1	0	4	Accessory	COG2907_R#	62.1	480	9.06E-135	K06954	62.1	480	2.42E-134
2284	WP_122214142.1	-	class I SAM-dependent methyltransferase	0	1	0	1	0	0	0	1	1	1	0	5	Accessory	COG2230_M#	39.4	258	3.28E-68	K00574	39.4	258	8.76E-68
2285	WP_102639414.1	eccE	type VII secretion protein EccE	1	0	1	0	1	1	1	0	1	0	1	7	Accessory					NA	27.1	51	0.0000212
2286	OSC64580.1	-	hypothetical protein	1	1	1	1	1	1	1	1	1	1	1	11	CORE								
2287	Prokka:JAPCLKMA_04145	-	putative fatty acid methyltransferase	1	1	1	1	1	1	1	1	1	1	1	11	CORE	COG2230_M#	46.7	330	8.85E-90	K00574	46.7	330	2.36E-89
2288	PWE11845.1	-	aldolase	1	1	1	1	1	1	1	1	1	1	1	11	CORE								
2289	Prokka:CIODPCDJ_02537	-	hypothetical protein	1	1	1	1	1	1	1	1	1	1	1	11	CORE	COG0477_GEPR#	45.2	307	6.15E-83				
2290	WP_114874552.1	secY	preprotein translocase subunit SecY	1	1	0	1	1	1	1	1	1	1	1	10	Accessory	COG0201_U#	60.9	536	8.13E-152	K03076	60.9	536	2.17E-151
2291	WP_048460680.1	-	hypothetical protein	1	0	1	0	1	1	0	1	1	1	1	8	Accessory					K06236	38.7	74	2.34E-12
2292	AXI84978.1	-	glutaminase	0	0	1	1	0	0	0	1	1	1	0	5	Accessory	COG2066_E#	50	375	2.4E-103	K01425	40.9	220	4.5E-56

cluster	GI	gene	Annotation	Streptomyces_thermocarboxydus_BPSAC147	Streptomyces_sp._4F	Streptomyces_sp._BSE7F	Streptomyces_sp._ETH9427	Streptomyces_sp._FxanaD5	Streptomyces_sp._UNC401CLCol	Actinospica_acidiphila_NRRL_B_24431	Streptomyces_sp._HNS054	Streptomyces_sp._SMS_SU21	Streptomyces_sp._ZS0098	Streptomyces_thermocarboxydus_K155	Addition	Core-Accessory-Unique	COG	%Similarity_COG	Score_COG	Evalue_COG	KEGG	%Similarity_KEGG	Score_KEGG	Evalue_KEGG
2293	WP_033274038.1	trkA	Trk system potassium transporter TrkA	0	0	0	0	0	0	1	0	0	0	0	1	Unique	COG0569_P#	28.6	191	6.42E-48	K03499	29.2	65	1.85E-09
2294	OSC69999.1	-	hypothetical protein	0	1	0	0	0	0	0	0	0	0	0	1	Unique					NA	36	67	2.86E-10
2295	AXI85298.1	-	hypothetical protein	0	0	0	1	0	0	0	0	0	0	0	1	Unique								
2296	OSC70129.1	-	hypothetical protein	1	1	1	1	1	1	1	1	1	1	1	11	CORE	COG5542_S#	23.9	58	6.49E-08				
2297	OSC55688.1	-	aspartate aminotransferase family protein	0	1	0	0	0	0	0	0	0	0	0	1	Unique	COG0160_E#	59.4	483	6.26E-136	K00827	36	221	1.18E-56
2298	WP_102641026.1	-	HlyC/CorC family transporter	1	1	1	1	1	1	1	1	1	1	1	11	CORE	COG1253_R#	51.4	404	3.69E-112	NA	51.4	404	9.87E-112
2299	Prokka:JAPCLKMA_01226	-	hypothetical protein	1	1	0	0	1	1	1	0	1	1	1	8	Accessory	COG1653_G#	23.3	94	1.39E-18				
2300	Prokka:JAPCLKMA_06128	-	hypothetical protein	1	1	0	0	1	1	1	0	0	0	1	6	Accessory	COG0577_V#	25	42	0.00481				
2301	Prokka:CIODPCDJ_00193	-	hypothetical protein	1	0	1	1	1	1	1	1	1	1	1	10	Accessory	COG1653_G#	26.8	160	1.58E-38	K10117	25.1	134	2.48E-30
2302	WP_019523449.1	-	DUF692 domain-containing protein	1	1	0	1	1	1	1	1	1	1	1	10	Accessory	COG3220_S#	33.9	148	4.76E-35				
2303	WP_122212728.1	-	DUF58 domain-containing protein	1	1	1	1	1	1	1	1	1	1	1	11	CORE	COG1721_R#	55.7	444	4.2E-124	NA	55.7	444	1.12E-123

cluster	GI	gene	Annotation	Streptomyces_thermocarboxydus_BPSAC147	Streptomyces_sp._4F	Streptomyces_sp._BSE7F	Streptomyces_sp._ETH9427	Streptomyces_sp._FxanaD5	Streptomyces_sp._UNC401CLCol	Actinospica_acidiphila_NRRL_B_24431	Streptomyces_sp._HNS054	Streptomyces_sp._SMS_SU21	Streptomyces_sp._ZS0098	Streptomyces_thermocarboxydus_K155	Addition	Core-Accessory-Unique	COG	%Similarity_COG	Score_COG	Evalue_COG	KEGG	%Similarity_KEGG	Score_KEGG	Evalue_KEGG
2304	WP_019527452.1	-	ATP-grasp domain-containing protein	0	0	0	0	1	0	0	0	0	0	0	1	Unique	COG0439_I#	29.6	144	8.98E-34	K13037	27.3	100	5.19E-20
2305	PWE08576.1	-	glycosyl transferase	0	0	1	0	0	0	0	0	0	0	0	1	Unique	COG0438_M#	25.3	49	0.0000393				
2306	WP_107685380.1	-	MFS transporter	1	1	1	0	1	1	1	1	1	1	1	10	Accessory	COG0477_GEPR#	26.3	140	1.3E-32	NA	26.3	140	3.46E-32
2307	PWE08568.1	-	aspartate--tRNA(Asn) ligase	0	0	1	0	0	0	0	0	0	0	0	1	Unique	COG0017_J#	43.3	342	2.25E-93	K01876	43.6	346	4.15E-94
2308	WP_102641691.1	-	hypothetical protein	1	0	1	1	1	1	1	1	1	1	1	10	Accessory					NA	28.5	105	1.23E-21
2309	PWE10859.1	-	carbohydrate ABC transporter substrate-binding protein	1	1	1	1	1	1	1	1	1	1	1	11	CORE	COG1653_G#	31.4	202	3.62E-51				
2310	Prokka:CIODPCDJ_02479	nagE2_1	PTS system N-acetylglucosamine-specific EIIC component	1	1	1	1	1	1	1	1	1	1	1	11	CORE	COG1263_G#	48.3	384	6.72E-106	K02804	47	357	1.8E-97
2311	WP_048459626.1	-	CpaF family protein	1	1	1	1	1	1	1	1	1	1	1	11	CORE	COG4962_U#	34.1	221	4.41E-57	K02283	32.8	144	1.83E-33
2312	WP_019524572.1	-	MFS transporter	1	1	1	1	1	1	1	1	0	1	1	10	Accessory	COG0477_GEPR#	47.4	386	1.77E-106	NA	31.7	240	4.18E-62

cluster	GI	gene	Annotation	Streptomyces_thermocarboxydus_BPSAC147	Streptomyces_sp._4F	Streptomyces_sp._BSE7F	Streptomyces_sp._ETH9427	Streptomyces_sp._FxanaD5	Streptomyces_sp._UNC401CLCol	Actinospica_acidiphila_NRRL_B_24431	Streptomyces_sp._HNS054	Streptomyces_sp._SMS_SU21	Streptomyces_sp._ZS0098	Streptomyces_thermocarboxydus_K155	Addition	Core-Accessory-Unique	COG	%Similarity_COG	Score_COG	Evalue_COG	KEGG	%Similarity_KEGG	Score_KEGG	Evalue_KEGG
2313	WP_028961269.1	-	deoxyguanosinetriphosphate triphosphohydrolase	1	1	1	1	1	1	1	1	1	1	1	11	CORE	COG0232_F#	50.3	354	5.7E-97	K01129	50.3	354	1.52E-96
2314	PWE07820.1	-	coenzyme F420-0:L-glutamate ligase	1	1	1	1	1	1	1	1	1	1	1	11	CORE	COG1478_S#	55	241	7.03E-63	K12234	52.4	416	2.5E-115
2315	WP_122215203.1	-	3-oxoacyl-ACP reductase	1	1	1	1	1	1	1	1	1	1	1	11	CORE	COG1028_IQR#	51.5	409	1.49E-113	K00059	50.4	399	5.39E-110
2316	AXI90234.1	-	cation transporter	1	1	0	1	1	1	1	1	1	1	1	10	Accessory	COG0025_P#	33.6	219	2.19E-56				
2317	WP_051724338.1	-	ArgE/DapE family deacylase	1	0	1	1	1	1	1	1	1	1	1	10	Accessory	COG0624_E#	33.8	241	5.38E-63	K01438	33.8	241	1.44E-62
2318	WP_033274258.1	-	TRAP transporter large permease subunit	0	0	0	0	0	0	1	0	0	0	0	1	Unique	COG2610_GE#	30.3	58	6.46E-08				
2319	OSC57947.1	-	peptidase	1	1	1	1	1	1	1	1	1	1	1	11	CORE	COG0739_M#	32.5	119	3.09E-26	NA	41.4	133	4.22E-30
2320	OSC61496.1	-	threonine synthase	1	1	1	1	1	1	1	1	1	1	1	11	CORE	COG0498_E#	55.2	461	3.31E-129	K01733	55.3	449	4.54E-125
2321	WP_033273433.1	-	MFS transporter	1	0	0	1	1	1	1	1	1	0	1	8	Accessory	COG0477_GEPR#	31.4	139	3.76E-32	NA	31.4	139	1.01E-31
2322	OSC58481.1	-	MFS transporter	0	1	0	0	0	0	0	0	0	0	0	1	Unique	COG0477_GEPR#	27.8	60	1.7E-08				
2323	WP_019524836.1	rfbH	lipopolysaccharide biosynthesis protein RfbH	0	0	0	0	1	1	0	0	0	0	1	3	Accessory	COG0399_M#	53.5	466	1.03E-130	NA	30.4	164	2.23E-39

cluster	GI	gene	Annotation	Streptomyces_thermocarboxydus_BPSAC147	Streptomyces_sp._4F	Streptomyces_sp._BSE7F	Streptomyces_sp._ETH9427	Streptomyces_sp._FxanaD5	Streptomyces_sp._UNC401CLCol	Actinospica_acidiphila_NRRL_B_24431	Streptomyces_sp._HNS054	Streptomyces_sp._SMS_SU21	Streptomyces_sp._ZS0098	Streptomyces_thermocarboxydus_K155	Addition	Core-Accessory-Unique	COG	%Similarity_COG	Score_COG	Evalue_COG	KEGG	%Similarity_KEGG	Score_KEGG	Evalue_KEGG
2324	WP_086704583.1	-	extracellular solute-binding protein	1	1	1	1	1	1	1	1	1	1	1	11	CORE	COG1653_G#	27.4	166	2.87E-40	K10117	23.8	138	1.31E-31
2325	OSC69275.1	-	phosphoesterase	0	1	0	0	0	0	0	0	0	0	0	1	Unique	COG1597_IR#	32.6	99	4.31E-20	NA	40	59	7.73E-08
2326	WP_048459282.1	-	site-2 protease family protein	1	1	1	1	1	1	1	1	1	1	1	11	CORE	COG0750_M#	33.5	184	1.02E-45	NA	34.8	171	1.4E-41
2327	Prokka:JAPCLKMA_02925	-	hypothetical protein	1	0	1	1	1	1	1	1	1	1	1	10	Accessory	COG2755_E#	41.5	246	2.18E-64				
2328	WP_033277979.1	-	protein kinase	0	1	1	1	0	0	1	1	1	1	0	7	Accessory								
2329	WP_102637711.1	-	alpha/beta fold hydrolase	1	1	1	0	1	1	1	1	1	1	1	10	Accessory	COG0596_R#	38.2	152	3.28E-36	K01607	37.7	94	3.7E-18
2330	Prokka:JAPCLKMA_05904	hrdA	RNA polymerase principal sigma factor HrdA	1	1	1	1	1	1	1	1	1	1	1	11	CORE	COG0568_K#	46.8	358	3.02E-98	K03086	46.8	358	8.05E-98
2331	OSC69321.1	-	sugar transporter	0	1	0	0	0	0	0	0	0	0	0	1	Unique	COG1653_G#	28.9	106	2.07E-22				
2332	Prokka:CIODPCDJ_03797	-	hypothetical protein	1	1	1	1	1	1	1	1	1	1	1	11	CORE								
2333	AXI91317.1	-	phage major capsid protein	0	0	0	1	0	0	0	0	0	0	0	1	Unique								
2334	Prokka:JAPCLKMA_06102	-	hypothetical protein	1	1	1	1	1	1	1	1	1	1	1	11	CORE	COG0477_GEPR#	26.2	87	1.3E-16				

cluster	GI	gene	Annotation	Streptomyces_thermocarboxydus_BPSAC147	Streptomyces_sp._4F	Streptomyces_sp._BSE7F	Streptomyces_sp._ETH9427	Streptomyces_sp._FxanaD5	Streptomyces_sp._UNC401CLCol	Actinospica_acidiphila_NRRL_B_24431	Streptomyces_sp._HNS054	Streptomyces_sp._SMS_SU21	Streptomyces_sp._ZS0098	Streptomyces_thermocarboxydus_K155	Addition	Core-Accessory-Unique	COG	%Similarity_COG	Score_COG	Evalue_COG	KEGG	%Similarity_KEGG	Score_KEGG	Evalue_KEGG
2335	OSC62596.1	-	RNA polymerase subunit sigma-24	1	1	1	1	1	1	1	1	1	1	1	11	CORE	COG4941_K#	65.1	494	3.52E-139				
2336	OSC63343.1	-	hypothetical protein	0	1	0	0	0	0	0	0	0	0	0	1	Unique								
2337	OSC64598.1	-	biotin carboxylase	0	1	0	0	0	0	0	0	0	0	0	1	Unique								
2338	WP_084207074.1	-	S8 family serine peptidase	0	0	0	0	0	0	1	0	1	0	0	2	Accessory	COG1404_O#	33.8	151	9.53E-36	K14645	34.9	131	2.09E-29
2339	Prokka:CIODPCDJ_03834	-	hypothetical protein	0	0	0	0	0	0	0	0	0	0	1	1	Unique								
2340	Prokka:JAPCLKMA_06142	pip_2	Proline iminopeptidase	1	1	1	1	1	1	1	1	1	1	1	11	CORE	COG0596_R#	40.6	271	6.32E-72	NA	46.3	370	2.04E-101
2341	WP_122213016.1	paaF	phenylacetate--CoA ligase	1	1	1	1	1	1	1	1	1	1	1	11	CORE	COG1541_H#	66.1	576	7.03E-164	K01912	60.7	534	8.18E-151
2342	Prokka:JAPCLKMA_03220	-	hypothetical protein	1	1	1	1	1	1	1	1	1	1	1	11	CORE	COG1649_S#	47.7	393	8.44E-109	NA	47.7	393	2.25E-108
2343	WP_122215767.1	-	hypothetical protein	0	0	0	0	0	0	0	0	0	1	0	1	Unique	COG0732_V#	30.7	160	1.2E-38	K01154	32	86	5.89E-16
2344	WP_028961398.1	-	extracellular solute-binding protein	0	0	0	0	0	1	0	0	0	0	0	1	Unique								
2345	OSC73564.1	-	hydrolase	0	1	0	0	0	0	0	0	0	0	0	1	Unique	COG4247_I#	38.4	241	7E-63	K01083	33.3	92	1.4E-17
2346	Prokka:JAPCLKMA_02251	-	hypothetical protein	1	1	0	0	0	0	0	0	0	0	1	3	Accessory	COG3291_R#	29	80	2.07E-14	NA	26.1	57	2.93E-07
2347	WP_033272184.1	-	hydroxyacyl-CoA dehydrogenase	1	0	0	1	1	1	1	1	0	1	1	8	Accessory								

cluster	GI	gene	Annotation	Streptomyces_thermocarboxydus_BPSAC147	Streptomyces_sp._4F	Streptomyces_sp._BSE7F	Streptomyces_sp._ETH9427	Streptomyces_sp._FxanaD5	Streptomyces_sp._UNC401CLCol	Actinospica_acidiphila_NRRL_B_24431	Streptomyces_sp._HNS054	Streptomyces_sp._SMS_SU21	Streptomyces_sp._ZS0098	Streptomyces_thermocarboxydus_K155	Addition	Core-Accessory-Unique	COG	%Similarity_COG	Score_COG	Evalue_COG	KEGG	%Similarity_KEGG	Score_KEGG	Evalue_KEGG
2348	OSC69141.1	-	hypothetical protein	1	1	1	1	1	1	1	1	1	1	1	11	CORE	COG0477_GEPR#	42.1	295	3.13E-79	K08224	39.7	279	4.74E-74
2349	OSC72786.1	-	hypothetical protein	0	1	0	0	0	0	0	0	0	0	0	1	Unique	COG0515_RTKL#	37.7	187	9.18E-47	K08884	37.7	187	2.45E-46
2350	WP_033274851.1	-	ArsB/NhaD family transporter	1	0	0	0	1	1	1	0	0	0	1	5	Accessory	COG1055_P#	51.4	446	1.43E-124	NA	51.2	444	1.11E-123
2351	OSC71991.1	-	ABC transporter substrate-binding protein	1	1	1	1	1	1	1	1	1	1	1	11	CORE	COG1653_G#	27.2	112	4.89E-24	K17244	20.6	63	6.96E-09
2352	WP_048456612.1	-	MFS transporter	1	1	1	1	1	1	1	1	0	1	1	10	Accessory	COG0477_GEPR#	43.1	363	1.22E-99	K08225	27.9	139	7.64E-32
2353	OSC69426.1	-	M18 family aminopeptidase	1	1	1	1	1	1	1	1	1	1	1	11	CORE	COG1362_E#	49.2	403	1.06E-111	NA	49.2	402	6.32E-111
2354	Prokka:CIODPCDJ_05248	rnd	Ribonuclease D	1	1	1	1	1	1	1	1	1	1	1	11	CORE	COG0349_J#	51.4	412	1.75E-114	K03684	51.4	412	4.68E-114
2355	PWE06757.1	-	DUF1343 domain-containing protein	1	1	1	1	1	1	1	1	1	1	1	11	CORE	COG3876_S#	46.6	357	8.73E-98	NA	43.4	340	2.95E-92
2356	WP_102640164.1	-	hypothetical protein	0	0	0	0	0	0	0	0	1	0	0	1	Unique								
2357	WP_051724576.1	-	HlyD family efflux transporter periplasmic adaptor subunit	0	0	0	0	0	0	1	0	0	0	0	1	Unique								
2358	AXI86457.1	-	aminotransferase	0	0	0	1	0	0	0	1	0	0	0	2	Accessory	COG0436_E#	33.2	161	5.38E-39	K00812	33.2	161	1.44E-38

cluster	GI	gene	Annotation	Streptomyces_thermocarboxydus_BPSAC147	Streptomyces_sp._4F	Streptomyces_sp._BSE7F	Streptomyces_sp._ETH9427	Streptomyces_sp._FxanaD5	Streptomyces_sp._UNC401CLCol	Actinospica_acidiphila_NRRL_B_24431	Streptomyces_sp._HNS054	Streptomyces_sp._SMS_SU21	Streptomyces_sp._ZS0098	Streptomyces_thermocarboxydus_K155	Addition	Core-Accessory-Unique	COG	%Similarity_COG	Score_COG	Evalue_COG	KEGG	%Similarity_KEGG	Score_KEGG	Evalue_KEGG
2359	Prokka:JAPCLKMA_04685	nanK_4	N-acetylmannosamine kinase	1	1	1	1	1	1	1	1	1	1	1	11	CORE	COG1940_KG#	27.1	109	2.43E-23	K00845	30.6	91	3.12E-17
2360	WP_051724463.1	-	MFS transporter	1	0	1	0	0	0	1	0	1	0	1	5	Accessory	COG0477_GEPR#	26.8	156	2.95E-37	NA	26.6	92	8.2E-18
2361	OSC63715.1	-	flavin-binding monooxygenase	0	1	0	0	0	0	0	0	0	0	0	1	Unique	COG2072_P#	33.9	247	7.46E-65	NA	32.8	242	1.09E-62
2362	WP_019523483.1	-	ABC transporter permease	1	1	1	1	1	1	1	1	1	1	1	11	CORE	COG4214_G#	41.4	312	3.21E-84	K10544	33.2	210	2.69E-53
2363	AXI87854.1	-	1,3-beta-glucanase	1	1	0	1	1	1	1	1	1	1	1	10	Accessory	COG2273_G#	48.8	255	4.66E-67	NA	34.7	119	1.07E-25
2364	WP_026243700.1	-	sugar ABC transporter substrate-binding protein	1	0	0	1	1	1	1	1	1	1	1	9	Accessory	COG1653_G#	31.4	219	2.84E-56	K10236	27.5	92	1.4E-17
2365	Prokka:CIODPCDJ_05127	-	hypothetical protein	1	0	1	1	1	1	1	1	1	1	1	10	Accessory								
2366	PWE11514.1	ectB	diaminobutyrate--2-oxoglutarate transaminase	1	1	1	1	1	1	1	1	1	1	1	11	CORE	COG0160_E#	55.9	476	7.54E-134	K07250	31.6	185	9.28E-46
2367	OSC72822.1	-	membrane protein insertase YidC	1	1	1	1	1	1	1	1	1	1	1	11	CORE	COG0706_U#	32.4	169	1.97E-41	K03217	32.4	169	5.27E-41
2368	Prokka:JAPCLKMA_05592	-	hypothetical protein	1	0	0	0	0	0	0	0	0	0	0	1	Unique	COG4585_T#	29.5	92	3.06E-18	NA	29.5	92	8.18E-18

cluster	GI	gene	Annotation	Streptomyces_thermocarboxydus_BPSAC147	Streptomyces_sp._4F	Streptomyces_sp._BSE7F	Streptomyces_sp._ETH9427	Streptomyces_sp._FxanaD5	Streptomyces_sp._UNC401CLCol	Actinospica_acidiphila_NRRL_B_24431	Streptomyces_sp._HNS054	Streptomyces_sp._SMS_SU21	Streptomyces_sp._ZS0098	Streptomyces_thermocarboxydus_K155	Addition	Core-Accessory-Unique	COG	%Similarity_COG	Score_COG	Evalue_COG	KEGG	%Similarity_KEGG	Score_KEGG	Evalue_KEGG
2369	OSC63627.1	-	dehydrogenase	1	1	1	1	1	1	1	1	1	1	1	11	CORE	COG0673_R#	49.6	386	1.34E-106	K00540	26.3	102	7.91E-21
2370	WP_048455276.1	-	SRPBCC family protein	1	1	1	1	1	1	1	1	1	1	1	11	CORE	COG3343_K#	35.1	52	0.00000599	NA	35.4	87	3.44E-16
2371	WP_048458571.1	-	hypothetical protein	0	0	0	0	0	0	0	1	0	0	0	1	Unique								
2372	OSC64813.1	-	hypothetical protein	1	1	1	1	1	1	1	1	1	1	1	11	CORE								
2373	WP_048459173.1	-	DUF1015 domain-containing protein	1	1	1	1	1	1	1	1	1	1	1	11	CORE								
2374	PWE07934.1	-	MFS transporter	1	1	1	1	1	1	1	1	1	1	1	11	CORE	COG0477_GEPR#	35.1	235	3.81E-61				
2375	PWE07952.1	-	M6 family metalloprotease domain-containing protein	1	1	1	1	1	1	1	1	1	1	1	11	CORE								
2376	PWE07926.1	-	hypothetical protein	1	0	1	1	1	1	1	1	0	1	1	9	Accessory								
2377	OSC71174.1	-	magnesium transporter	1	1	1	1	1	1	1	1	1	1	1	11	CORE	COG2239_P#	49.5	390	9.27E-108	NA	49.3	386	2.74E-106
2378	AXI88175.1	-	amino acid permease	1	1	1	1	1	1	1	1	1	1	1	11	CORE	COG0531_E#	72.1	574	2.65E-163	K03294	72.1	574	7.08E-163
2379	OSC62172.1	-	threonine--tRNA ligase	1	1	1	1	1	1	0	1	1	1	1	10	Accessory	COG0441_J#	50.7	379	2.14E-104	K01868	47.4	375	6.31E-103
2380	OSC71254.1	-	drug:proton antiporter	1	1	1	1	1	1	1	1	1	1	1	11	CORE	COG0644_C#	34	226	2.31E-58	NA	34	226	6.18E-58
2381	Prokka:JAPCLKMA_01492	-	hypothetical protein	1	0	0	0	0	0	0	0	0	0	0	1	Unique								

cluster	GI	gene	Annotation	Streptomyces_thermocarboxydus_BPSAC147	Streptomyces_sp._4F	Streptomyces_sp._BSE7F	Streptomyces_sp._ETH9427	Streptomyces_sp._FxanaD5	Streptomyces_sp._UNC401CLCol	Actinospica_acidiphila_NRRL_B_24431	Streptomyces_sp._HNS054	Streptomyces_sp._SMS_SU21	Streptomyces_sp._ZS0098	Streptomyces_thermocarboxydus_K155	Addition	Core-Accessory-Unique	COG	%Similarity_COG	Score_COG	Evalue_COG	KEGG	%Similarity_KEGG	Score_KEGG	Evalue_KEGG
2382	WP_033275537.1	-	homoserine dehydrogenase	1	1	1	1	1	1	1	1	1	1	1	11	CORE	COG0460_E#	62.7	500	4.87E-141	K00003	62.7	500	1.3E-140
2383	Prokka:JAPCLKMA_01794	-	IS200/IS605 family transposase ISEc42	1	0	0	0	0	1	0	0	0	1	1	4	Accessory	COG0675_L#	30.3	154	8.56E-37	K07496	30.3	154	2.29E-36
2384	PWE07869.1	-	cytochrome P450	1	0	1	1	1	1	1	1	1	1	1	10	Accessory	COG2124_Q#	38.1	288	4.96E-77	K15468	37.7	295	1.08E-78
2385	OSC56839.1	-	cation/H(+) antiporter	0	1	0	0	0	0	0	0	0	0	0	1	Unique	COG0475_P#	40.7	279	1.76E-74	K03455	40.5	279	4.71E-74
2386	AXI89591.1	-	IS256 family transposase	1	0	0	1	1	1	0	0	0	0	1	5	Accessory	COG3328_L#	59.2	525	2.41E-148	K07493	59.2	525	6.44E-148
2387	Prokka:JAPCLKMA_06154	nagC_5	N-acetylglucosamine repressor	1	0	1	1	1	1	1	1	1	1	1	10	Accessory	COG1940_KG#	31.8	188	4.09E-47	K00845	29.4	136	8.41E-31
2388	OSC69949.1	-	hypothetical protein	0	1	0	0	0	0	0	0	0	0	0	1	Unique								
2389	Prokka:JAPCLKMA_06624	-	hypothetical protein	1	0	0	0	0	0	0	0	0	1	0	2	Accessory								
2390	WP_033274034.1	-	extracellular solute-binding protein	1	0	1	1	1	1	1	1	1	1	1	10	Accessory	COG1653_G#	31.7	200	1.36E-50	K10236	27.6	66	6.27E-10
2391	WP_159020883.1	-	hypothetical protein	0	0	0	0	0	0	0	1	0	0	0	1	Unique								
2392	WP_086703974.1	-	citrate synthase	1	1	1	1	1	1	1	1	1	0	0	9	Accessory	COG0372_C#	68.9	602	9.07E-172	K01647	68.9	602	2.42E-171
2393	AXI85785.1	-	ABC transporter substrate-binding protein	1	1	0	1	1	1	1	1	1	1	1	10	Accessory	COG1653_G#	30.9	186	2.65E-46	K02027	28.4	166	4.44E-40

cluster	GI	gene	Annotation	Streptomyces_thermocarboxydus_BPSAC147	Streptomyces_sp._4F	Streptomyces_sp._BSE7F	Streptomyces_sp._ETH9427	Streptomyces_sp._FxanaD5	Streptomyces_sp._UNC401CLCol	Actinospica_acidiphila_NRRL_B_24431	Streptomyces_sp._HNS054	Streptomyces_sp._SMS_SU21	Streptomyces_sp._ZS0098	Streptomyces_thermocarboxydus_K155	Addition	Core-Accessory-Unique	COG	%Similarity_COG	Score_COG	Evalue_COG	KEGG	%Similarity_KEGG	Score_KEGG	Evalue_KEGG
2394	WP_122214143.1	-	methyltransferase domain-containing protein	0	0	0	1	0	0	0	1	1	1	0	4	Accessory	COG2230_M#	56.3	455	3.06E-127	K00574	56.3	455	8.16E-127
2395	Prokka:CIODPCDJ_05817	-	hypothetical protein	1	0	1	1	1	1	1	1	1	0	1	9	Accessory	COG2866_E#	29.1	47	0.000192				
2396	WP_051724690.1	-	helix-turn-helix transcriptional regulator	0	0	0	0	0	0	1	0	0	0	0	1	Unique								
2397	WP_122217153.1	-	MCE family protein	0	0	0	1	1	1	1	1	1	1	1	8	Accessory	COG1463_Q#	32.3	171	8.81E-42	K02067	32.3	171	2.35E-41
2398	PWE09787.1	-	ABC transporter substrate-binding protein	0	0	1	0	0	0	0	0	0	0	0	1	Unique	COG1653_G#	26.6	123	1.61E-27	K17244	21.3	63	5.29E-09
2399	OSC65551.1	-	plasmid partitioning protein	0	1	0	1	0	0	0	0	0	0	0	2	Accessory	COG1475_K#	26.9	80	1.2E-14				
2400	PWE11247.1	-	hypothetical protein	0	0	1	0	0	0	0	0	0	0	0	1	Unique								
2401	Prokka:JAPCLKMA_01926	bioA	Adenosylmethionine-8-amino-7-oxononanoate aminotransferase	1	1	1	1	1	1	1	1	1	1	1	11	CORE	COG0161_H#	62.4	532	1.15E-150	K00833	61.4	527	1.69E-148
2402	Prokka:CIODPCDJ_01053	pyrC	Dihydroorotase	1	1	1	1	1	1	1	1	1	1	1	11	CORE	COG0044_F#	58.5	476	1.28E-133	K01465	58.5	476	3.41E-133

cluster	GI	gene	Annotation	Streptomyces_thermocarboxydus_BPSAC147	Streptomyces_sp._4F	Streptomyces_sp._BSE7F	Streptomyces_sp._ETH9427	Streptomyces_sp._FxanaD5	Streptomyces_sp._UNC401CLCol	Actinospica_acidiphila_NRRL_B_24431	Streptomyces_sp._HNS054	Streptomyces_sp._SMS_SU21	Streptomyces_sp._ZS0098	Streptomyces_thermocarboxydus_K155	Addition	Core-Accessory-Unique	COG	%Similarity_COG	Score_COG	Evalue_COG	KEGG	%Similarity_KEGG	Score_KEGG	Evalue_KEGG
2403	WP_102639329.1	-	MFS transporter	1	0	1	1	1	1	1	1	1	1	1	10	Accessory	COG0477_GEPR#	28.9	111	8.27E-24	NA	28.9	111	2.21E-23
2404	WP_033272943.1	-	L,D-transpeptidase	1	1	1	1	1	1	1	1	1	1	1	11	CORE	COG1376_S#	34.5	258	5.47E-68	NA	34.5	258	1.46E-67
2405	Prokka:JAPCLKMA_06378	argD_2	Acetylornithine aminotransferase	1	1	1	1	1	1	1	1	1	1	1	11	CORE	COG0160_E#	35.7	234	8.46E-61	K00827	38.2	262	1.01E-68
2406	Prokka:JAPCLKMA_03599	yfeO	Putative ion-transport protein YfeO	1	0	1	1	1	1	1	1	1	1	1	10	Accessory	COG0038_P#	49	382	2.51E-105	NA	49	382	6.71E-105
2407	AXI88072.1	-	acetyl-CoA C-acetyltransferase	1	1	1	1	1	1	1	1	1	1	1	11	CORE	COG0183_I#	64.1	519	1.32E-146	K00626	64.7	513	1.93E-144
2408	WP_033275845.1	-	glutamate-5-semialdehyde dehydrogenase	1	1	1	1	1	1	1	1	1	1	1	11	CORE	COG0014_E#	61.3	465	2.95E-130	K00147	61.3	465	7.87E-130
2409	AXI86713.1	-	ATP-dependent Clp protease ATP-binding subunit ClpX	0	1	1	1	1	1	1	1	1	1	1	10	Accessory	COG1219_O#	77.8	661	2.8E-189	K03544	77.8	661	7.48E-189
2410	WP_033277963.1	-	sulfite oxidase	1	0	0	0	1	1	1	0	0	0	1	5	Accessory	COG2041_R#	38.7	209	2.23E-53	K07147	38.7	209	5.96E-53
2411	WP_019524866.1	-	ketosynthase chain-length factor	0	1	0	0	1	1	1	0	0	0	1	5	Accessory	COG0304_IQ#	32	198	3.94E-50	K09458	31.7	204	1.92E-51

cluster	GI	gene	Annotation	Streptomyces_thermocarboxydus_BPSAC147	Streptomyces_sp._4F	Streptomyces_sp._BSE7F	Streptomyces_sp._ETH9427	Streptomyces_sp._FxanaD5	Streptomyces_sp._UNC401CLCol	Actinospica_acidiphila_NRRL_B_24431	Streptomyces_sp._HNS054	Streptomyces_sp._SMS_SU21	Streptomyces_sp._ZS0098	Streptomyces_thermocarboxydus_K155	Addition	Core-Accessory-Unique	COG	%Similarity_COG	Score_COG	Evalue_COG	KEGG	%Similarity_KEGG	Score_KEGG	Evalue_KEGG
2412	WP_019521458.1	-	aromatic ring-hydroxylating dioxygenase subunit alpha	1	0	0	0	1	1	0	0	0	0	1	4	Accessory	COG4638_PR#	25.7	90	1.97E-17	NA	28	82	1.1E-14
2413	WP_048460410.1	purD	phosphoribosylamine--glycine ligase	1	1	1	1	1	1	1	1	1	1	1	11	CORE	COG0151_F#	58.4	453	6.79E-127	K01945	57.4	452	4.04E-126
2414	Prokka:CIODPCDJ_04246	-	hypothetical protein	0	0	0	0	0	0	0	0	0	0	1	1	Unique					NA	29.5	76	7.87E-13
2415	WP_048459014.1	-	two-component sensor histidine kinase	1	1	0	1	1	1	1	1	1	1	1	10	Accessory	COG0642_T#	45.4	269	2.37E-71	K07768	45.4	269	6.34E-71
2416	OSC71021.1	-	hypothetical protein	0	1	0	0	0	0	0	0	0	0	0	1	Unique								
2417	Prokka:JAPCLKMA_04908	umuC	Protein UmuC	1	0	0	0	0	0	0	0	0	0	0	1	Unique	COG0389_L#	44.4	314	8.4E-85	K03502	44.2	311	1.11E-83
2418	WP_033276283.1	-	cation:proton antiporter	0	0	0	0	0	0	1	0	0	0	0	1	Unique	COG0475_P#	44.3	324	8.12E-88	NA	38.2	265	9.15E-70
2419	WP_078894993.1	-	hypothetical protein	1	0	0	1	1	1	1	1	1	1	1	9	Accessory								
2420	Prokka:CIODPCDJ_03456	-	hypothetical protein	0	0	0	0	0	0	1	0	0	0	1	2	Accessory	COG2508_TQ#	37	234	6.48E-61	NA	37	234	1.73E-60
2421	Prokka:JAPCLKMA_00956	dgoT	D-galactonate transporter	1	0	0	0	1	1	0	0	0	0	1	4	Accessory	COG0477_GEPR#	24.6	125	4.24E-28	K08194	24.6	125	1.13E-27

cluster	GI	gene	Annotation	Streptomyces_thermocarboxydus_BPSAC147	Streptomyces_sp._4F	Streptomyces_sp._BSE7F	Streptomyces_sp._ETH9427	Streptomyces_sp._FxanaD5	Streptomyces_sp._UNC401CLCol	Actinospica_acidiphila_NRRL_B_24431	Streptomyces_sp._HNS054	Streptomyces_sp._SMS_SU21	Streptomyces_sp._ZS0098	Streptomyces_thermocarboxydus_K155	Addition	Core-Accessory-Unique	COG	%Similarity_COG	Score_COG	Evalue_COG	KEGG	%Similarity_KEGG	Score_KEGG	Evalue_KEGG
2422	Prokka:CIODPCDJ_05682	-	hypothetical protein	0	0	0	0	0	0	0	0	0	0	1	1	Unique	COG0732_V#	30.5	151	5.52E-36	K01154	23.2	69	9.62E-11
2423	PWE10269.1	-	beta-ketoacyl-[acyl-carrier-protein] synthase family protein	0	0	1	0	0	0	0	0	0	0	0	1	Unique	COG0304_IQ#	38.8	284	7.13E-76	K09458	37.7	284	1.46E-75
2424	WP_078611395.1	-	site-specific integrase	0	0	0	0	0	1	0	0	0	0	0	1	Unique	COG0582_L#	27.2	128	5E-29	NA	27.4	111	1.69E-23
2425	Prokka:CIODPCDJ_03559	purA	Adenylosuccinate synthetase	1	1	0	1	1	1	1	1	1	1	1	10	Accessory	COG0104_F#	66.3	602	1.18E-171	K01939	67.5	590	1.62E-167
2426	WP_102637617.1	-	NAD(P)-binding protein	0	1	1	1	0	0	1	1	1	1	0	7	Accessory	COG0654_HC#	32.6	147	1.36E-34	NA	29	129	7.82E-29
2427	WP_048456113.1	-	DUF4910 domain-containing protein	1	0	1	1	1	1	1	1	1	1	1	10	Accessory	COG4310_R#	52.5	470	9.14E-132	NA	48	443	1.87E-123
2428	Prokka:CIODPCDJ_01131	-	hypothetical protein	0	0	0	0	0	0	0	0	0	0	1	1	Unique	COG1520_S#	25.9	45	0.000556	NA	25.5	60	5.82E-08
2429	WP_107080913.1	-	methyltransferase domain-containing protein	1	1	1	1	1	1	1	1	1	1	1	11	CORE	COG0500_QR#	27.2	113	1.66E-24				
2430	Prokka:JAPCLKMA_00142	trpB	Tryptophan synthase beta chain	1	1	1	1	1	1	1	1	1	1	1	11	CORE	COG0133_E#	69.2	575	1.18E-163	K01696	69.2	575	3.16E-163
2431	WP_048459481.1	-	hypothetical protein	0	0	0	1	0	0	0	1	1	1	0	4	Accessory								

cluster	GI	gene	Annotation	Streptomyces_thermocarboxydus_BPSAC147	Streptomyces_sp._4F	Streptomyces_sp._BSE7F	Streptomyces_sp._ETH9427	Streptomyces_sp._FxanaD5	Streptomyces_sp._UNC401CLCol	Actinospica_acidiphila_NRRL_B_24431	Streptomyces_sp._HNS054	Streptomyces_sp._SMS_SU21	Streptomyces_sp._ZS0098	Streptomyces_thermocarboxydus_K155	Addition	Core-Accessory-Unique	COG	%Similarity_COG	Score_COG	Evalue_COG	KEGG	%Similarity_KEGG	Score_KEGG	Evalue_KEGG
2432	OSC73218.1	-	amino acid deaminase	1	1	0	1	1	1	1	1	1	1	1	10	Accessory	COG3616_E#	42.7	289	2.21E-77				
2433	OSC57448.1	-	sensor histidine kinase	1	1	0	0	1	1	1	0	0	0	1	6	Accessory	COG4564_T#	28	102	3.83E-21				
2434	OSC71890.1	-	thymidine phosphorylase	1	1	1	1	1	1	1	1	1	1	1	11	CORE	COG0213_F#	63.2	511	2.74E-144	K00758	63.2	511	7.31E-144
2435	WP_028961317.1	-	ATP-binding protein	0	0	0	0	0	1	0	0	0	0	0	1	Unique								
2436	Prokka:JAPCLKMA_00807	lpqY	Trehalose-binding lipoprotein LpqY	1	0	1	1	1	1	1	1	1	1	1	10	Accessory	COG1653_G#	41.7	207	1.1E-52	K02027	41.7	207	2.95E-52
2437	OSC68614.1	-	sugar ABC transporter substrate-binding protein	0	1	0	0	0	0	0	0	0	0	0	1	Unique	COG1653_G#	33	249	2.53E-65	K02027	28	181	1.33E-44
2438	Prokka:JAPCLKMA_02613	wbpA_1	UDP-N-acetyl-D-glucosamine 6-dehydrogenase	1	0	0	0	0	0	0	0	0	0	0	1	Unique	COG0677_M#	37.4	265	2.62E-70	NA	31.5	196	3.99E-49
2439	WP_102638530.1	-	MFS transporter	0	0	1	0	0	0	0	0	1	0	0	2	Accessory	COG2814_G#	38.4	240	1.54E-62	NA	33.8	207	2.26E-52
2440	PWE10081.1	-	MFS transporter	1	0	1	1	1	0	0	1	0	1	0	6	Accessory	COG0477_GEPR#	25.9	105	4.52E-22				
2441	WP_102638450.1	efeB	deferrochelatase/peroxidase EfeB	1	1	1	1	1	1	1	1	1	1	1	11	CORE	COG2837_P#	34.9	233	1.44E-60	K16301	34.9	233	3.83E-60

cluster	GI	gene	Annotation	Streptomyces_thermocarboxydus_BPSAC147	Streptomyces_sp._4F	Streptomyces_sp._BSE7F	Streptomyces_sp._ETH9427	Streptomyces_sp._FxanaD5	Streptomyces_sp._UNC401CLCol	Actinospica_acidiphila_NRRL_B_24431	Streptomyces_sp._HNS054	Streptomyces_sp._SMS_SU21	Streptomyces_sp._ZS0098	Streptomyces_thermocarboxydus_K155	Addition	Core-Accessory-Unique	COG	%Similarity_COG	Score_COG	Evalue_COG	KEGG	%Similarity_KEGG	Score_KEGG	Evalue_KEGG
2442	WP_033278045.1	-	ABC transporter substrate-binding protein	1	1	1	1	1	1	1	1	1	1	1	11	CORE	COG0683_E#	27.2	81	6.98E-15				
2443	Prokka:JAPCLKMA_04214	-	hypothetical protein	1	1	0	1	1	1	1	1	1	1	1	10	Accessory	COG5542_S#	25.8	63	2.57E-09				
2444	Prokka:JAPCLKMA_06225	-	hypothetical protein	1	0	0	0	1	1	1	0	0	0	1	5	Accessory	COG1502_I#	33.3	42	0.00469				
2445	WP_122213367.1	-	extracellular solute-binding protein	0	0	0	0	0	0	0	0	0	1	0	1	Unique	COG1653_G#	21.8	97	9.42E-20	K02027	25.4	70	5.62E-11
2446	Prokka:JAPCLKMA_06601	yhhS	putative MFS-type transporter YhhS	1	1	1	1	1	1	1	1	1	1	1	11	CORE	COG0477_GEPR#	22.9	89	3.35E-17	K08153	22.1	84	3.76E-15
2447	WP_019527965.1	-	tyrosine-type recombinase/integrase	0	0	0	0	1	0	0	0	0	0	0	1	Unique	COG0582_L#	29.3	81	6.98E-15	NA	29.3	81	1.86E-14
2448	OSC61691.1	-	beta-ketoacyl-[acyl-carrier-protein] synthase II	1	1	1	1	1	1	1	1	1	1	1	11	CORE	COG0304_IQ#	45.1	365	3.16E-100	K09458	45.1	365	8.44E-100
2449	Prokka:JAPCLKMA_02097	-	hypothetical protein	1	0	0	0	1	1	0	0	0	0	1	4	Accessory	COG1835_I#	28.1	62	3.36E-09				
2450	WP_019527117.1	-	serine/threonine protein kinase	1	1	0	0	1	1	1	1	1	1	1	9	Accessory	COG0515_RTKL#	30.6	149	3.56E-35	K08884	30.6	149	9.51E-35
2451	PWE07267.1	-	cytochrome	1	1	1	1	1	1	1	1	1	1	1	11	CORE	COG2124_Q#	27	125	5.51E-28	K00517	27	99	6.62E-20

cluster	GI	gene	Annotation	Streptomyces_thermocarboxydus_BPSAC147	Streptomyces_sp._4F	Streptomyces_sp._BSE7F	Streptomyces_sp._ETH9427	Streptomyces_sp._FxanaD5	Streptomyces_sp._UNC401CLCol	Actinospica_acidiphila_NRRL_B_24431	Streptomyces_sp._HNS054	Streptomyces_sp._SMS_SU21	Streptomyces_sp._ZS0098	Streptomyces_thermocarboxydus_K155	Addition	Core-Accessory-Unique	COG	%Similarity_COG	Score_COG	Evalue_COG	KEGG	%Similarity_KEGG	Score_KEGG	Evalue_KEGG
2452	OSC69697.1	-	DNA cytosine methyltransferase	0	1	0	0	0	0	0	0	0	0	0	1	Unique	COG0270_L#	31.3	64	1.15E-09	K00558	31.3	64	3.08E-09
2453	WP_048460765.1	-	dehydrogenase	0	0	0	1	0	0	0	1	0	1	0	3	Accessory	COG0852_C#	40.4	95	4.67E-19	K05581	33.3	79	9.26E-14
2454	WP_033273552.1	-	sugar ABC transporter substrate-binding protein	1	1	1	1	1	1	1	1	1	1	1	11	CORE	COG1653_G#	30.6	183	1.3E-45				
2455	PWE09853.1	-	sugar transporter	1	0	1	1	1	1	1	1	1	1	1	10	Accessory	COG1653_G#	24.6	112	2.83E-24				
2456	AXI85381.1	-	IS701 family transposase	0	0	0	1	0	1	0	0	0	0	1	3	Accessory	COG5659_L#	40.5	286	1.87E-76				
2457	OSC70956.1	-	ketosynthase chain-length factor	1	1	1	1	1	1	1	1	1	1	1	11	CORE	COG0304_IQ#	34.8	213	1.18E-54	K09458	34.8	213	3.14E-54
2458	WP_048457023.1	-	aspartate kinase	1	1	1	1	1	1	1	1	1	1	1	11	CORE	COG0527_E#	64.9	541	3.21E-153	K00928	64.9	541	8.58E-153
2459	OSC65316.1	-	two-component sensor histidine kinase	0	1	0	0	0	0	0	0	0	0	0	1	Unique	COG4585_T#	37.8	198	3.91E-50	NA	35.9	117	4E-25
2460	OSC72981.1	-	serine--tRNA ligase	1	1	1	1	1	1	1	1	1	1	1	11	CORE	COG0172_J#	59.6	489	1.45E-137	K01875	59.8	480	1.8E-134
2461	WP_122217388.1	-	ATP-binding protein	0	0	0	0	0	0	0	0	0	1	0	1	Unique	COG1106_R#	27.7	86	2.17E-16				
2462	OSC69891.1	-	hypothetical protein	0	1	0	0	0	0	0	0	0	0	0	1	Unique								

cluster	GI	gene	Annotation	Streptomyces_thermocarboxydus_BPSAC147	Streptomyces_sp._4F	Streptomyces_sp._BSE7F	Streptomyces_sp._ETH9427	Streptomyces_sp._FxanaD5	Streptomyces_sp._UNC401CLCol	Actinospica_acidiphila_NRRL_B_24431	Streptomyces_sp._HNS054	Streptomyces_sp._SMS_SU21	Streptomyces_sp._ZS0098	Streptomyces_thermocarboxydus_K155	Addition	Core-Accessory-Unique	COG	%Similarity_COG	Score_COG	Evalue_COG	KEGG	%Similarity_KEGG	Score_KEGG	Evalue_KEGG
2463	Prokka:JAPCLKMA_05449	-	hypothetical protein	1	1	0	0	1	1	1	1	1	1	1	9	Accessory	COG4110_R#	40.2	164	6.26E-40	K05792	35.6	218	9.75E-56
2464	OSC63137.1	-	hypothetical protein	0	1	0	0	0	0	0	0	0	0	0	1	Unique	COG3064_M#	39.4	72	3.24E-12	NA	41.3	85	1.68E-15
2465	Prokka:JAPCLKMA_00973	-	hypothetical protein	1	1	0	0	1	1	1	0	0	0	1	6	Accessory	COG2391_R#	43	305	2.27E-82	K07112	32.6	93	4.73E-18
2466	AXI89178.1	-	TIGR02679 family protein	0	0	0	1	0	0	0	1	0	0	0	2	Accessory								
2467	WP_078504715.1	-	sensor histidine kinase	1	0	0	1	1	1	1	1	1	1	1	9	Accessory	COG4585_T#	32.2	105	4.51E-22	NA	32.2	105	1.2E-21
2468	Prokka:JAPCLKMA_01575	-	hypothetical protein	1	0	0	0	0	0	0	0	0	0	0	1	Unique								
2469	WP_033277123.1	-	glycosyl transferase	0	0	0	0	0	0	1	0	0	0	1	2	Accessory	COG1819_GC#	35.1	284	5.42E-76	K00699	28	86	7.55E-16
2470	WP_102640205.1	-	sensor histidine kinase	0	1	1	1	0	0	0	1	1	1	0	6	Accessory	COG4585_T#	28.3	108	5.31E-23	K07778	30.7	101	2.26E-20
2471	WP_019523373.1	-	hypothetical protein	1	1	1	1	1	1	1	1	1	1	1	11	CORE								
2472	Prokka:JAPCLKMA_01210	-	hypothetical protein	1	1	0	0	1	1	1	1	1	1	1	9	Accessory	COG3284_QK#	28.2	88	7.43E-17				
2473	WP_048457310.1	-	HAMP domain-containing histidine kinase	1	1	1	1	1	1	1	1	1	1	1	11	CORE	COG0642_T#	29.5	119	2.3E-26	NA	29.5	119	6.15E-26
2474	WP_026243737.1	-	sensor histidine kinase	1	1	1	1	1	1	1	0	1	1	1	10	Accessory	COG4585_T#	43.5	147	1.35E-34	K02480	43.5	144	1.79E-33

cluster	GI	gene	Annotation	Streptomyces_thermocarboxydus_BPSAC147	Streptomyces_sp._4F	Streptomyces_sp._BSE7F	Streptomyces_sp._ETH9427	Streptomyces_sp._FxanaD5	Streptomyces_sp._UNC401CLCol	Actinospica_acidiphila_NRRL_B_24431	Streptomyces_sp._HNS054	Streptomyces_sp._SMS_SU21	Streptomyces_sp._ZS0098	Streptomyces_thermocarboxydus_K155	Addition	Core-Accessory-Unique	COG	%Similarity_COG	Score_COG	Evalue_COG	KEGG	%Similarity_KEGG	Score_KEGG	Evalue_KEGG
2475	PWE07737.1	-	CBS domain-containing protein	1	1	1	1	1	1	1	1	1	1	1	11	CORE	COG1125_E#	45.9	322	3.06E-87	K05847	46.3	320	3.1E-86
2476	Prokka:JAPCLKMA_02048	-	hypothetical protein	1	1	1	1	1	1	1	1	1	1	1	11	CORE	COG2304_R#	33.1	128	6.48E-29				
2477	WP_033278119.1	-	MFS transporter	0	0	0	0	0	0	1	0	0	0	0	1	Unique	COG0477_GEPR#	23.9	111	6.27E-24	NA	27.1	76	7.8E-13
2478	WP_019527807.1	-	ABC transporter permease	1	1	1	1	1	1	1	1	1	1	1	11	CORE	COG1079_R#	39.2	200	1.03E-50	K02057	39.2	200	2.74E-50
2479	WP_122215818.1	-	glycosyltransferase family 4 protein	1	0	1	1	1	1	1	1	1	1	1	10	Accessory	COG0438_M#	28.6	98	7.18E-20	K15521	30.5	96	7.28E-19
2480	Prokka:JAPCLKMA_00595	-	hypothetical protein	1	0	1	1	1	1	1	1	1	1	1	10	Accessory								
2481	WP_048457576.1	-	condensation protein	0	0	1	1	0	0	1	1	1	1	0	6	Accessory								
2482	WP_019524842.1	-	DUF1205 domain-containing protein	0	0	0	0	1	1	0	0	0	0	1	3	Accessory	COG1819_GC#	26.4	117	1.14E-25				
2483	PWE09014.1	-	hypothetical protein	0	0	1	1	0	0	0	1	1	1	0	5	Accessory								
2484	WP_033273671.1	-	PQQ-binding-like beta-propeller repeat protein	0	1	0	0	1	1	1	0	1	0	0	5	Accessory								
2485	Prokka:CIODPCDJ_04247	-	putative zinc protease	0	0	0	0	0	0	0	0	0	0	1	1	Unique	COG0612_R#	34.1	187	8.99E-47	NA	28.9	138	1.28E-31

cluster	GI	gene	Annotation	Streptomyces_thermocarboxydus_BPSAC147	Streptomyces_sp._4F	Streptomyces_sp._BSE7F	Streptomyces_sp._ETH9427	Streptomyces_sp._FxanaD5	Streptomyces_sp._UNC401CLCol	Actinospica_acidiphila_NRRL_B_24431	Streptomyces_sp._HNS054	Streptomyces_sp._SMS_SU21	Streptomyces_sp._ZS0098	Streptomyces_thermocarboxydus_K155	Addition	Core-Accessory-Unique	COG	%Similarity_COG	Score_COG	Evalue_COG	KEGG	%Similarity_KEGG	Score_KEGG	Evalue_KEGG
2486	OSC69117.1	-	transcriptional regulator	0	1	0	0	0	0	0	0	0	0	0	1	Unique	COG1940_KG#	31.8	154	1.1E-36	K02565	30.1	143	3.98E-33
2487	WP_078895014.1	-	MFS transporter	0	0	0	0	0	0	0	1	1	0	0	2	Accessory	COG0477_GEPR#	29.7	168	5.63E-41	K08169	26	62	1.16E-08
2488	WP_033275722.1	-	ABC transporter substrate-binding protein	1	1	1	1	1	1	1	1	1	1	1	11	CORE	COG1653_G#	37.3	259	1.86E-68	K10236	31.8	185	9.11E-46
2489	Prokka:JAPCLKMA_06831	mdtH_4	Multidrug resistance protein MdtH	1	0	0	1	1	1	1	1	0	1	1	8	Accessory	COG0477_GEPR#	29.6	95	4.64E-19	NA	29.6	95	1.24E-18
2490	Prokka:JAPCLKMA_02351	-	hypothetical protein	1	1	1	1	1	1	1	1	1	1	1	11	CORE	COG4585_T#	30.1	93	2.3E-18	K07778	30.1	93	6.15E-18
2491	PWE10555.1	-	hypothetical protein	0	0	1	0	0	0	0	0	1	0	0	2	Accessory	COG0596_R#	30.8	77	1.31E-13				
2492	AXI84652.1	-	acetylornithine transaminase	1	0	1	1	1	1	1	1	1	1	1	10	Accessory	COG4992_E#	57.6	451	4.35E-126	K00818	57.6	451	1.16E-125
2493	AXI85370.1	-	DNA methylase	1	0	0	1	0	0	0	0	0	0	0	2	Accessory	COG0270_L#	24.5	60	2.16E-08	K00558	47.3	44	0.00251
2494	PWE12005.1	-	DUF4032 domain-containing protein	1	1	1	1	1	1	1	1	1	1	1	11	CORE								
2495	PWE10277.1	-	hypothetical protein	0	0	1	0	0	0	0	0	0	0	0	1	Unique	COG3321_Q#	34.9	200	1.02E-50	K12435	34.9	200	2.74E-50
2496	OSC68516.1	-	transcriptional regulator	0	1	0	0	0	0	0	0	0	0	0	1	Unique								

cluster	GI	gene	Annotation	Streptomyces_thermocarboxydus_BPSAC147	Streptomyces_sp._4F	Streptomyces_sp._BSE7F	Streptomyces_sp._ETH9427	Streptomyces_sp._FxanaD5	Streptomyces_sp._UNC401CLCol	Actinospica_acidiphila_NRRL_B_24431	Streptomyces_sp._HNS054	Streptomyces_sp._SMS_SU21	Streptomyces_sp._ZS0098	Streptomyces_thermocarboxydus_K155	Addition	Core-Accessory-Unique	COG	%Similarity_COG	Score_COG	Evalue_COG	KEGG	%Similarity_KEGG	Score_KEGG	Evalue_KEGG
2497	WP_026244185.1	-	PucR family transcriptional regulator	1	0	1	1	1	1	1	1	1	1	1	10	Accessory	COG2508_TQ#	25.6	66	3.01E-10	NA	25.6	66	8.05E-10
2498	WP_158688253.1	-	TIR domain-containing protein	1	0	0	0	1	1	0	0	1	0	1	5	Accessory								
2499	WP_102640489.1	-	extracellular solute-binding protein	1	1	1	1	1	1	1	1	1	1	1	11	CORE	COG1653_G#	28.1	146	2.29E-34	K10108	25.5	111	2.85E-23
2500	Prokka:CIODPCDJ_06528	purD_2	Phosphoribosylamine--glycine ligase	1	0	0	0	1	1	1	0	0	0	1	5	Accessory	COG0439_I#	31.8	155	4.93E-37	K14755	29.5	107	4.12E-22
2501	WP_106432486.1	-	resuscitation-promoting factor	1	0	1	0	1	1	1	1	1	1	1	9	Accessory	COG3583_S#	28.3	122	3.54E-27	NA	37	170	3.96E-41
2502	OSC70957.1	-	beta-ACP synthase	1	1	1	1	1	1	1	1	1	1	1	11	CORE	COG0304_IQ#	41	322	1.79E-87	K09458	41	322	4.77E-87
2503	OSC71400.1	-	TrpB-like pyridoxal-phosphate dependent enzyme	0	1	0	0	0	0	0	0	0	0	0	1	Unique	COG1350_R#	49.3	372	2.57E-102	K06001	46.7	357	1.75E-97
2504	WP_157842146.1	-	hypothetical protein	0	0	0	0	0	0	1	0	0	0	0	1	Unique								
2505	WP_033275316.1	-	FUSC family protein	1	0	1	1	1	1	1	1	1	1	1	10	Accessory	COG4129_S#	23.7	63	2.55E-09	NA	21.4	60	3.37E-08
2506	WP_033274479.1	-	cytochrome P450	1	0	1	1	1	1	1	1	1	1	1	10	Accessory	COG2124_Q#	32.3	175	4.6E-43	K00517	32.3	175	1.23E-42
2507	Prokka:JAPCLKMA_03089	-	hypothetical protein	1	0	0	1	1	1	1	1	0	0	1	7	Accessory	COG1653_G#	29.6	163	2.36E-39	K17244	22.5	77	3.48E-13

cluster	GI	gene	Annotation	Streptomyces_thermocarboxydus_BPSAC147	Streptomyces_sp._4F	Streptomyces_sp._BSE7F	Streptomyces_sp._ETH9427	Streptomyces_sp._FxanaD5	Streptomyces_sp._UNC401CLCol	Actinospica_acidiphila_NRRL_B_24431	Streptomyces_sp._HNS054	Streptomyces_sp._SMS_SU21	Streptomyces_sp._ZS0098	Streptomyces_thermocarboxydus_K155	Addition	Core-Accessory-Unique	COG	%Similarity_COG	Score_COG	Evalue_COG	KEGG	%Similarity_KEGG	Score_KEGG	Evalue_KEGG
2508	PWE06864.1	-	tyrosine--tRNA ligase	1	1	1	1	1	1	1	1	1	1	1	11	CORE	COG0162_J#	60.6	495	2.01E-139	K01866	60.6	495	5.36E-139
2509	Prokka:CIODPCDJ_02947	-	hypothetical protein	1	1	1	1	1	1	1	1	1	1	1	11	CORE	COG1653_G#	26	131	7.61E-30	K10117	32.8	88	1.51E-16
2510	Prokka:CIODPCDJ_05119	nepI	Purine ribonucleoside efflux pump NepI	0	0	1	1	1	1	0	1	1	0	1	7	Accessory	COG2814_G#	52.4	385	2.93E-106	K03445	27.2	163	6.31E-39
2511	WP_019525231.1	-	amidohydrolase family protein	1	1	0	1	1	1	1	1	1	1	1	10	Accessory								
2512	Prokka:JAPCLKMA_01731	arsB	Arsenical pump membrane protein	1	0	1	1	1	1	1	1	1	1	1	10	Accessory	COG1055_P#	57.9	434	3.22E-121	K03893	57.9	434	8.6E-121
2513	Prokka:CIODPCDJ_02304	-	Ferredoxin--NADP reductase	1	0	1	1	1	1	1	1	1	1	1	10	Accessory	COG2072_P#	34.1	221	4.28E-57				
2514	WP_102638302.1	-	HAMP domain-containing histidine kinase	1	0	1	1	1	1	1	1	1	1	1	10	Accessory	COG0642_T#	34.1	116	3.31E-25	K07645	28.1	94	2.11E-18
2515	Prokka:JAPCLKMA_05131	-	hypothetical protein	1	1	1	1	1	1	0	1	1	1	1	10	Accessory								
2516	OSC72204.1	-	ornithine decarboxylase	0	1	1	1	0	0	0	1	0	1	0	5	Accessory	COG0019_E#	43.8	271	4.71E-72	K01581	33.2	181	2.24E-44
2517	Prokka:JAPCLKMA_01758	-	hypothetical protein	1	0	0	0	0	1	0	0	0	0	1	3	Accessory								
2518	OSC61437.1	-	hypothetical protein	0	1	0	0	0	0	0	0	0	0	0	1	Unique								

cluster	GI	gene	Annotation	Streptomyces_thermocarboxydus_BPSAC147	Streptomyces_sp._4F	Streptomyces_sp._BSE7F	Streptomyces_sp._ETH9427	Streptomyces_sp._FxanaD5	Streptomyces_sp._UNC401CLCol	Actinospica_acidiphila_NRRL_B_24431	Streptomyces_sp._HNS054	Streptomyces_sp._SMS_SU21	Streptomyces_sp._ZS0098	Streptomyces_thermocarboxydus_K155	Addition	Core-Accessory-Unique	COG	%Similarity_COG	Score_COG	Evalue_COG	KEGG	%Similarity_KEGG	Score_KEGG	Evalue_KEGG
2519	WP_028959988.1	-	helix-turn-helix transcriptional regulator	0	0	0	0	0	1	0	0	0	0	0	1	Unique								
2520	Prokka:JAPCLKMA_05495	btrK	L-glutamyl-[BtrI acyl-carrier protein] decarboxylase	1	0	0	0	0	0	0	0	0	0	0	1	Unique	COG0019_E#	32.5	191	6.2E-48	K01586	27.7	116	5.18E-25
2521	WP_158688260.1	-	hypothetical protein	0	0	0	0	1	1	1	0	1	1	1	6	Accessory	COG1426_S#	30.8	62	3.32E-09	K03129	30.7	85	1.67E-15
2522	WP_078611402.1	-	LCP family protein	1	1	1	1	1	1	1	1	1	1	1	11	CORE	COG1316_K#	43.1	208	3.74E-53	NA	32	141	1.96E-32
2523	WP_048458277.1	-	glycosyltransferase	1	1	1	0	1	1	1	1	1	1	1	10	Accessory	COG0438_M#	33.5	212	2.59E-54	K15521	38.2	209	7.65E-53
2524	OSC63175.1	-	sugar transporter	1	1	1	1	1	1	1	1	1	1	1	11	CORE	COG1653_G#	26.6	115	4.31E-25				
2525	WP_122212821.1	bioB	biotin synthase BioB	1	0	1	1	1	1	1	1	1	1	1	10	Accessory	COG0502_H#	50.6	319	2.57E-86	K01012	49.7	317	1.53E-85
2526	WP_048457593.1	-	MCE family protein	0	0	1	1	1	1	1	1	1	1	1	9	Accessory	COG1463_Q#	31.2	176	1.58E-43	K02067	31.2	176	4.21E-43
2527	WP_033277356.1	-	site-specific integrase	1	0	1	0	0	1	1	0	1	0	1	6	Accessory	COG0582_L#	41.7	44	0.00159	NA	41.7	44	0.00426
2528	WP_019522663.1	-	two-component sensor histidine kinase	0	0	0	0	1	1	1	0	0	0	1	4	Accessory	COG4585_T#	33.4	118	5.09E-26	K07778	33.4	118	1.36E-25
2529	AXI87011.1	-	hypothetical protein	1	1	0	1	1	1	1	1	1	1	1	10	Accessory								
2530	OSC69661.1	-	hypothetical protein	0	1	0	0	0	0	0	0	0	0	0	1	Unique	COG0270_L#	29.6	84	8.15E-16	K00558	31.8	106	9.13E-22

cluster	GI	gene	Annotation	Streptomyces_thermocarboxydus_BPSAC147	Streptomyces_sp._4F	Streptomyces_sp._BSE7F	Streptomyces_sp._ETH9427	Streptomyces_sp._FxanaD5	Streptomyces_sp._UNC401CLCol	Actinospica_acidiphila_NRRL_B_24431	Streptomyces_sp._HNS054	Streptomyces_sp._SMS_SU21	Streptomyces_sp._ZS0098	Streptomyces_thermocarboxydus_K155	Addition	Core-Accessory-Unique	COG	%Similarity_COG	Score_COG	Evalue_COG	KEGG	%Similarity_KEGG	Score_KEGG	Evalue_KEGG
2531	PWE11549.1	-	hypothetical protein	0	0	1	1	0	0	0	1	1	1	0	5	Accessory	COG1408_R#	36.3	161	8.95E-39	NA	35.5	165	1.27E-39
2532	Prokka:JAPCLKMA_03105	mdtH_2	Multidrug resistance protein MdtH	1	0	0	0	0	1	0	0	0	0	1	3	Accessory	COG0477_GEPR#	41.3	283	1.56E-75	NA	41.3	283	4.17E-75
2533	WP_048456109.1	-	glycosyltransferase	1	0	0	1	1	1	1	1	1	1	1	9	Accessory	COG0438_M#	33.4	147	1.02E-34	K16150	32.3	91	2.33E-17
2534	WP_048457601.1	-	lytic transglycosylase domain-containing protein	0	0	1	1	1	1	1	1	1	1	1	9	Accessory	COG2951_M#	37.9	200	1.02E-50	NA	37.9	200	2.72E-50
2535	WP_019522137.1	-	inorganic phosphate transporter	1	1	1	1	1	1	1	1	1	1	1	11	CORE	COG0306_P#	54.8	424	5.67E-118	K03306	54.5	413	2.05E-114
2536	AXI91208.1	-	hypothetical protein	0	0	0	1	0	0	0	0	0	0	0	1	Unique								
2537	OSC65719.1	-	serine hydroxymethyltransferase	1	1	1	1	1	1	1	1	1	1	1	11	CORE	COG0112_E#	72.4	602	1.16E-171	K00600	72.4	602	3.11E-171
2538	PWE07393.1	-	FAD-dependent oxidoreductase	1	1	1	1	1	1	1	1	1	1	1	11	CORE	COG0446_R#	51.6	401	5.14E-111	K00529	51.6	401	1.37E-110
2539	WP_122213441.1	-	ricin-type beta-trefoil lectin domain protein	0	0	1	1	1	1	1	1	1	1	1	9	Accessory					NA	36.2	59	1.28E-07
2540	Prokka:JAPCLKMA_02240	-	hypothetical protein	1	0	1	0	1	1	1	1	1	1	1	9	Accessory	COG0142_H#	39.3	238	4.41E-62	K13787	40.9	231	1.44E-59

cluster	GI	gene	Annotation	Streptomyces_thermocarboxydus_BPSAC147	Streptomyces_sp._4F	Streptomyces_sp._BSE7F	Streptomyces_sp._ETH9427	Streptomyces_sp._FxanaD5	Streptomyces_sp._UNC401CLCol	Actinospica_acidiphila_NRRL_B_24431	Streptomyces_sp._HNS054	Streptomyces_sp._SMS_SU21	Streptomyces_sp._ZS0098	Streptomyces_thermocarboxydus_K155	Addition	Core-Accessory-Unique	COG	%Similarity_COG	Score_COG	Evalue_COG	KEGG	%Similarity_KEGG	Score_KEGG	Evalue_KEGG
2541	OSC69505.1	-	phosphopantetheine--protein transferase	0	1	0	0	0	0	0	0	0	0	0	1	Unique	COG1192_D#	32	91	8.71E-18	K03496	30.2	91	3.04E-17
2542	WP_102639183.1	-	histidine kinase	0	1	0	0	0	0	0	0	1	0	0	2	Accessory	COG4585_T#	28.8	81	6.9E-15	NA	28.8	81	1.84E-14
2543	WP_122213617.1	-	acetate kinase	1	1	1	1	1	1	1	1	1	1	1	11	CORE	COG0282_C#	52.1	400	6.71E-111	K00925	52.1	400	1.79E-110
2544	OSC61642.1	-	hypothetical protein	0	1	0	0	0	0	0	0	0	0	0	1	Unique	COG2755_E#	38.2	245	4.71E-64				
2545	OSC57312.1	-	6-phospho-beta-glucosidase	1	1	0	1	1	1	1	1	1	1	1	10	Accessory	COG1486_G#	39.4	301	5.54E-81	K01222	37.2	268	1.39E-70
2546	WP_102637518.1	-	ABC transporter permease	0	0	0	1	0	0	0	1	1	0	0	3	Accessory	COG0577_V#	34.4	160	1.17E-38	K02004	28	144	2.32E-33
2547	OSC62542.1	-	hypothetical protein	1	1	1	1	1	1	1	1	1	1	1	11	CORE								
2548	Prokka:JAPCLKMA_04314	styA	Styrene monooxygenase StyA	1	1	1	1	1	1	1	1	1	1	1	11	CORE								
2549	WP_033274004.1	-	molybdopterin-dependent oxidoreductase	1	0	1	0	1	1	1	1	1	1	1	9	Accessory	COG2041_R#	49.9	370	9.69E-102	K07147	49.9	370	2.59E-101
2550	Prokka:CIODPCDJ_04897	dxr	1-deoxy-D-xylulose 5-phosphate reductoisomerase	1	1	1	1	1	1	1	1	1	1	1	11	CORE	COG0743_I#	52.4	384	6.49E-106	K00099	52.4	384	1.73E-105
2551	PWE07600.1	-	hypothetical protein	0	0	1	0	0	0	0	0	0	1	0	2	Accessory	COG0455_D#	26.6	102	4.92E-21	NA	26.6	102	1.31E-20

cluster	GI	gene	Annotation	Streptomyces_thermocarboxydus_BPSAC147	Streptomyces_sp._4F	Streptomyces_sp._BSE7F	Streptomyces_sp._ETH9427	Streptomyces_sp._FxanaD5	Streptomyces_sp._UNC401CLCol	Actinospica_acidiphila_NRRL_B_24431	Streptomyces_sp._HNS054	Streptomyces_sp._SMS_SU21	Streptomyces_sp._ZS0098	Streptomyces_thermocarboxydus_K155	Addition	Core-Accessory-Unique	COG	%Similarity_COG	Score_COG	Evalue_COG	KEGG	%Similarity_KEGG	Score_KEGG	Evalue_KEGG
2552	OSC65372.1	-	glycosyl transferase	0	1	0	0	0	0	0	0	0	0	0	1	Unique	COG1215_M#	28.2	129	2.88E-29	NA	20.8	47	0.000502
2553	WP_122216751.1	-	hypothetical protein	1	0	0	0	1	1	0	0	1	1	1	6	Accessory					NA	27.3	91	1.78E-17
2554	WP_079031441.1	-	pyroglutamyl peptidase	1	1	1	1	1	1	1	1	1	1	1	11	CORE								
2555	WP_122214302.1	-	MFS transporter	1	1	1	1	1	1	1	1	1	1	1	11	CORE	COG0477_GEPR#	36.1	215	5.21E-55	NA	35.9	212	6.91E-54
2556	WP_033275085.1	-	histidine--tRNA ligase	1	1	1	1	1	1	1	1	1	1	1	11	CORE	COG0124_J#	57.4	470	8.99E-132	K01892	57.4	470	2.4E-131
2557	WP_107685353.1	-	cytochrome P450	1	0	1	1	1	1	1	1	1	1	1	10	Accessory	COG2124_Q#	41.6	269	1.78E-71	K15468	39.4	255	7.11E-67
2558	PWE12423.1	-	FAD-dependent oxidoreductase	1	1	1	1	1	1	1	1	1	1	1	11	CORE	COG0654_HC#	45.4	260	1.08E-68	NA	45.4	260	2.89E-68
2559	Prokka:JAPCLKMA_05062	-	hypothetical protein	1	0	0	0	0	0	0	0	0	0	0	1	Unique								
2560	OSC61017.1	-	arginine deiminase	0	1	0	0	0	0	0	0	0	0	0	1	Unique	COG2235_E#	64.2	568	1.86E-161	K01478	39.9	289	4.45E-77
2561	WP_048459110.1	-	MFS transporter	1	1	1	0	1	1	0	1	1	0	1	8	Accessory	COG0477_GEPR#	26	66	2.99E-10	K08166	23.8	63	6.77E-09
2562	OSC70425.1	-	septum formation initiator	1	1	1	1	1	1	1	1	1	1	1	11	CORE	COG2894_D#	31.5	80	2E-14	K03609	27	74	3.83E-12
2563	Prokka:CIODPCDJ_00832	-	Cyclohex-1-ene-1-carbonyl-CoA dehydrogenase	1	0	0	0	1	1	0	0	0	0	1	4	Accessory	COG1960_I#	35.7	216	2.34E-55	K00249	35.6	210	4.48E-53

cluster	GI	gene	Annotation	Streptomyces_thermocarboxydus_BPSAC147	Streptomyces_sp._4F	Streptomyces_sp._BSE7F	Streptomyces_sp._ETH9427	Streptomyces_sp._FxanaD5	Streptomyces_sp._UNC401CLCol	Actinospica_acidiphila_NRRL_B_24431	Streptomyces_sp._HNS054	Streptomyces_sp._SMS_SU21	Streptomyces_sp._ZS0098	Streptomyces_thermocarboxydus_K155	Addition	Core-Accessory-Unique	COG	%Similarity_COG	Score_COG	Evalue_COG	KEGG	%Similarity_KEGG	Score_KEGG	Evalue_KEGG
2564	OSC60987.1	-	MFS transporter	0	1	0	0	0	0	0	0	0	0	0	1	Unique	COG0477_GEPR#	24.2	109	3.08E-23	NA	27.3	106	9.1E-22
2565	AXI85871.1	-	MFS transporter	1	1	1	1	1	1	1	1	1	1	1	11	CORE	COG0477_GEPR#	29.4	174	1.02E-42				
2566	WP_078504661.1	-	hypothetical protein	1	1	1	1	1	1	1	1	1	1	1	11	CORE								
2567	PWE10807.1	-	hypothetical protein	0	1	1	1	0	0	1	1	1	1	0	7	Accessory								
2568	WP_028960077.1	-	glycosyltransferase family 4 protein	1	0	1	1	1	1	1	1	1	1	1	10	Accessory	COG0438_M#	26	62	5.63E-09				
2569	WP_048460693.1	-	aminotransferase class V-fold PLP-dependent enzyme	0	1	1	1	0	0	1	1	1	1	0	7	Accessory	COG3844_E#	44.4	351	4.65E-96	K01556	49.1	349	3.61E-95
2570	OSC69582.1	-	AMP-dependent synthetase	0	1	0	0	0	0	0	0	0	0	0	1	Unique	COG0318_IQ#	33.7	210	9.8E-54	K00666	33.7	210	2.62E-53
2571	Prokka:CIODPCDJ_01407	-	hypothetical protein	1	0	0	0	1	1	0	0	0	0	1	4	Accessory	COG4268_V#	26.6	77	9.92E-14				
2572	Prokka:JAPCLKMA_01596	mdtH_1	Multidrug resistance protein MdtH	1	0	0	0	0	0	0	0	0	0	0	1	Unique	COG0477_GEPR#	54.5	403	1.03E-111	NA	54.5	403	2.75E-111
2573	WP_102637415.1	-	hypothetical protein	0	0	0	0	0	0	0	0	1	0	0	1	Unique								
2574	Prokka:JAPCLKMA_01559	pknD_6	Serine/threonine-protein kinase PknD	1	0	0	0	0	0	0	0	0	0	0	1	Unique	COG0515_RTKL#	30.2	84	1.38E-15	K08884	30.2	74	3.82E-12

cluster	GI	gene	Annotation	Streptomyces_thermocarboxydus_BPSAC147	Streptomyces_sp._4F	Streptomyces_sp._BSE7F	Streptomyces_sp._ETH9427	Streptomyces_sp._FxanaD5	Streptomyces_sp._UNC401CLCol	Actinospica_acidiphila_NRRL_B_24431	Streptomyces_sp._HNS054	Streptomyces_sp._SMS_SU21	Streptomyces_sp._ZS0098	Streptomyces_thermocarboxydus_K155	Addition	Core-Accessory-Unique	COG	%Similarity_COG	Score_COG	Evalue_COG	KEGG	%Similarity_KEGG	Score_KEGG	Evalue_KEGG
2575	PWE07245.1	-	peptidase	1	0	1	1	1	1	1	1	1	1	1	10	Accessory	COG1680_V#	23.8	67	1.75E-10	NA	23.8	61	1.96E-08
2576	OSC63648.1	-	branched chain amino acid ABC transporter substrate-binding protein	1	1	1	1	1	1	1	1	1	1	1	11	CORE	COG0683_E#	35.3	225	5.02E-58	K01999	35.3	225	1.34E-57
2577	OSC71872.1	-	transcriptional regulator	0	1	0	0	0	0	0	0	0	0	0	1	Unique	COG3284_QK#	35.8	116	2.52E-25	NA	35.8	116	6.72E-25
2578	Prokka:JAPCLKMA_01627	nisC_1	Nisin biosynthesis protein NisC	1	0	0	0	0	0	0	0	0	0	0	1	Unique								
2579	Prokka:CIODPCDJ_05322	-	hypothetical protein	1	0	0	1	1	1	1	1	1	1	1	9	Accessory								
2580	WP_028958849.1	-	two-component sensor histidine kinase	0	1	0	0	0	1	0	0	0	0	1	3	Accessory	COG4585_T#	26.1	92	3.88E-18	NA	26.1	92	1.04E-17
2581	WP_033275704.1	-	cysteine desulfurase	1	1	1	1	1	1	1	1	1	1	1	11	CORE	COG0520_E#	53.4	442	2E-123	K11717	52.2	434	1.45E-120
2582	WP_053077010.1	-	hypothetical protein	0	0	0	0	0	0	0	1	0	0	0	1	Unique								
2583	Prokka:JAPCLKMA_03774	-	hypothetical protein	1	0	0	0	0	0	1	0	0	0	1	3	Accessory								
2584	Prokka:JAPCLKMA_04260	-	hypothetical protein	1	1	0	1	1	1	1	1	1	1	1	10	Accessory								

cluster	GI	gene	Annotation	Streptomyces_thermocarboxydus_BPSAC147	Streptomyces_sp._4F	Streptomyces_sp._BSE7F	Streptomyces_sp._ETH9427	Streptomyces_sp._FxanaD5	Streptomyces_sp._UNC401CLCol	Actinospica_acidiphila_NRRL_B_24431	Streptomyces_sp._HNS054	Streptomyces_sp._SMS_SU21	Streptomyces_sp._ZS0098	Streptomyces_thermocarboxydus_K155	Addition	Core-Accessory-Unique	COG	%Similarity_COG	Score_COG	Evalue_COG	KEGG	%Similarity_KEGG	Score_KEGG	Evalue_KEGG
2585	WP_019524006.1	-	GNAT family N-acetyltransferase	1	1	1	1	1	1	1	1	1	1	1	11	CORE	COG4552_R#	35.5	154	6.37E-37	NA	35.5	154	1.7E-36
2586	WP_122213906.1	-	arginine deiminase	1	1	1	1	1	1	1	1	1	1	1	11	CORE	COG2235_E#	50.7	380	9.32E-105	K01478	45.3	310	1.85E-83
2587	WP_048457993.1	-	caspase family protein	0	0	0	1	0	0	0	1	0	0	0	2	Accessory								
2588	Prokka:JAPCLKMA_01070	-	hypothetical protein	1	1	0	0	1	1	1	0	0	0	1	6	Accessory					K13381	33.3	154	2.9E-36
2589	OSC73441.1	-	serine hydroxymethyltransferase	1	1	1	1	1	1	1	1	1	1	1	11	CORE	COG0112_E#	52.8	414	5.82E-115	K00600	52.8	414	1.56E-114
2590	OSC57814.1	-	MFS transporter	1	1	1	1	1	1	0	1	1	1	1	10	Accessory	COG0477_GEPR#	51.5	387	5.84E-107				
2591	PWE09824.1	urtA	urea ABC transporter substrate-binding protein	0	0	1	1	1	1	1	1	1	1	1	9	Accessory	COG0683_E#	61.8	505	1.92E-142	K11959	53.5	424	1.5E-117
2592	WP_102638436.1	-	serine/threonine-protein phosphatase	1	0	1	1	1	1	1	1	1	1	1	10	Accessory	COG2208_TK#	29.9	85	6.18E-16				
2593	WP_078493019.1	dprA	DNA-protecting protein DprA	1	1	1	1	1	1	1	1	1	1	1	11	CORE	COG0758_LU#	45.9	260	1.4E-68	K04096	45.9	260	3.75E-68
2594	WP_033275267.1	-	PQQ-binding-like beta-propeller repeat protein	1	1	0	1	1	1	1	1	1	1	1	10	Accessory	COG3391_S#	25.9	82	3.07E-15				

cluster	GI	gene	Annotation	Streptomyces_thermocarboxydus_BPSAC147	Streptomyces_sp._4F	Streptomyces_sp._BSE7F	Streptomyces_sp._ETH9427	Streptomyces_sp._FxanaD5	Streptomyces_sp._UNC401CLCol	Actinospica_acidiphila_NRRL_B_24431	Streptomyces_sp._HNS054	Streptomyces_sp._SMS_SU21	Streptomyces_sp._ZS0098	Streptomyces_thermocarboxydus_K155	Addition	Core-Accessory-Unique	COG	%Similarity_COG	Score_COG	Evalue_COG	KEGG	%Similarity_KEGG	Score_KEGG	Evalue_KEGG
2595	WP_051724638.1	-	hypothetical protein	1	0	1	1	1	1	1	1	1	1	1	10	Accessory								
2596	WP_033273932.1	-	FAD-binding protein	1	1	1	1	1	1	1	1	1	1	1	11	CORE	COG0277_C#	31.5	181	4.85E-45	K00103	41	90	3.93E-17
2597	Prokka:JAPCLKMA_04871	ldtB_4	L,D-transpeptidase 2	1	1	1	1	1	1	1	1	1	1	1	11	CORE	COG1376_S#	33.1	214	8.82E-55	NA	32.9	210	4.44E-53
2598	Prokka:JAPCLKMA_05214	-	hypothetical protein	1	1	0	0	1	1	0	0	0	0	1	5	Accessory								
2599	OSC61824.1	-	MFS transporter	0	1	0	0	0	0	0	0	0	0	0	1	Unique	COG0477_GEPR#	41.6	323	1.03E-87	K05819	31	171	1.34E-41
2600	OSC61378.1	-	hypothetical protein	0	1	0	0	0	0	0	0	0	0	0	1	Unique								
2601	WP_019527699.1	-	hypothetical protein	0	0	0	0	1	0	0	0	0	0	1	2	Accessory	COG3482_S#	32.1	141	7.27E-33	NA	32.1	141	1.94E-32
2602	Prokka:JAPCLKMA_00624	-	hypothetical protein	1	0	0	0	1	1	1	0	0	0	0	4	Accessory								
2603	OSC73231.1	-	serine/threonine protein kinase	1	1	0	1	1	1	1	1	1	1	1	10	Accessory	COG0515_RTKL#	32.9	167	1.24E-40	K08884	31.3	159	5.27E-38
2604	WP_037778080.1	-	two-component sensor histidine kinase	1	1	1	1	1	1	1	1	1	1	1	11	CORE	COG4585_T#	31.8	87	9.52E-17	NA	25.6	65	1.76E-09
2605	WP_106432511.1	-	IS3 family transposase	1	0	1	1	1	1	0	1	0	0	1	7	Accessory	COG2801_L#	51.1	321	5.11E-87	NA	51.9	303	2.95E-81
2606	WP_102638806.1	-	hypothetical protein	1	1	1	1	1	1	1	1	1	1	1	11	CORE								

cluster	GI	gene	Annotation	Streptomyces_thermocarboxydus_BPSAC147	Streptomyces_sp._4F	Streptomyces_sp._BSE7F	Streptomyces_sp._ETH9427	Streptomyces_sp._FxanaD5	Streptomyces_sp._UNC401CLCol	Actinospica_acidiphila_NRRL_B_24431	Streptomyces_sp._HNS054	Streptomyces_sp._SMS_SU21	Streptomyces_sp._ZS0098	Streptomyces_thermocarboxydus_K155	Addition	Core-Accessory-Unique	COG	%Similarity_COG	Score_COG	Evalue_COG	KEGG	%Similarity_KEGG	Score_KEGG	Evalue_KEGG
2607	Prokka:CIODPCDJ_02885	ncsB3	2-hydroxy-5-methyl-1-naphthoate 7-hydroxylase	1	1	1	1	1	1	1	1	1	1	1	11	CORE	COG2124_Q#	39.9	276	1.44E-73	NA	33.8	200	2.69E-50
2608	WP_122215888.1	-	metallophosphoesterase	0	0	0	0	0	0	0	0	0	1	0	1	Unique	COG1408_R#	33.2	148	7.75E-35	NA	36.4	173	6.01E-42
2609	Prokka:JAPCLKMA_04999	cefD_2	Isopenicillin N epimerase	1	0	1	1	1	1	1	1	1	1	1	10	Accessory	COG0520_E#	34.3	178	6.99E-44				
2610	WP_048457884.1	-	GNAT family N-acetyltransferase	1	1	1	1	1	1	1	1	1	1	1	11	CORE	COG4552_R#	34	149	2.04E-35	NA	34	149	5.45E-35
2611	WP_122216018.1	-	cytochrome P450	0	0	1	1	0	0	0	1	1	1	0	5	Accessory	COG2124_Q#	35.5	202	3.46E-51	NA	35.5	202	9.25E-51
2612	Prokka:CIODPCDJ_04344	-	hypothetical protein	1	0	1	0	1	1	0	0	1	1	1	7	Accessory								
2613	PWE10338.1	-	serine/threonine protein kinase	1	0	1	0	1	1	0	0	0	0	0	4	Accessory	COG0515_RTKL#	30.1	131	7.5E-30	NA	35.6	134	3.09E-30
2614	PWE09087.1	-	hypothetical protein	0	1	1	0	0	0	0	1	0	1	0	4	Accessory	COG0457_R#	33.7	54	0.00000117				
2615	OSC65100.1	-	hypothetical protein	0	1	0	0	1	1	1	0	0	0	1	5	Accessory	COG0477_GEPR#	54.2	408	2.44E-113	NA	54.4	399	3.02E-110
2616	WP_051724669.1	-	acyltransferase domain-containing protein	0	0	0	0	0	0	1	0	0	0	0	1	Unique	COG3321_Q#	44.3	211	7.45E-54	NA	41.5	184	1.99E-45

cluster	GI	gene	Annotation	Streptomyces_thermocarboxydus_BPSAC147	Streptomyces_sp._4F	Streptomyces_sp._BSE7F	Streptomyces_sp._ETH9427	Streptomyces_sp._FxanaD5	Streptomyces_sp._UNC401CLCol	Actinospica_acidiphila_NRRL_B_24431	Streptomyces_sp._HNS054	Streptomyces_sp._SMS_SU21	Streptomyces_sp._ZS0098	Streptomyces_thermocarboxydus_K155	Addition	Core-Accessory-Unique	COG	%Similarity_COG	Score_COG	Evalue_COG	KEGG	%Similarity_KEGG	Score_KEGG	Evalue_KEGG
2617	WP_102642253.1	-	hydroxymethylglutaryl-CoA synthase family protein	0	0	1	0	0	0	0	0	1	0	0	2	Accessory	COG3425_I#	40.8	260	8.2E-69	K15311	42.6	333	3.47E-90
2618	OSC73464.1	-	sensor histidine kinase	1	1	1	1	1	1	1	1	1	1	1	11	CORE	COG4585_T#	37.4	140	1.62E-32	K07778	37.4	140	4.31E-32
2619	WP_019527291.1	-	SpoIIE family protein phosphatase	1	0	1	1	1	1	1	1	1	1	1	10	Accessory	COG2208_TK#	28.8	102	4.86E-21	K02485	28.4	105	1.53E-21
2620	OSC56313.1	-	cytochrome	0	1	0	0	0	0	0	0	0	0	0	1	Unique	COG2124_Q#	28.2	110	1.79E-23	K17483	27.1	96	9.31E-19
2621	Prokka:CIODPCDJ_00700	khtU_1	K(+)/H(+) antiporter subunit KhtU	1	1	1	1	1	1	1	1	1	1	1	11	CORE	COG0475_P#	55.4	396	1.63E-109	K03455	55.4	396	4.35E-109
2622	AXI84690.1	-	MFS transporter	0	0	0	1	0	0	0	1	0	0	0	2	Accessory	COG0477_GEPR#	29.4	161	6.76E-39	NA	29.7	121	2.07E-26
2623	AXI89601.1	-	transcriptional regulator	0	0	0	1	0	0	0	0	0	0	0	1	Unique								
2624	WP_019522860.1	-	UBA/THIF-type NAD/FAD-binding protein	1	1	1	0	1	1	1	1	1	1	1	10	Accessory					NA	31	79	6.9E-14
2625	PWE08996.1	-	hypothetical protein	0	0	1	0	0	0	0	0	0	0	0	1	Unique								
2626	WP_033277770.1	-	cytochrome P450	0	0	0	1	1	1	1	1	1	1	1	8	Accessory	COG2124_Q#	44.7	352	2.07E-96	K15629	42.5	339	3.7E-92
2627	OSC56165.1	-	histidine kinase	0	1	0	0	0	0	0	0	0	0	0	1	Unique	COG2203_T#	32.1	117	1.46E-25	K07682	34	178	1.86E-43

cluster	GI	gene	Annotation	Streptomyces_thermocarboxydus_BPSAC147	Streptomyces_sp._4F	Streptomyces_sp._BSE7F	Streptomyces_sp._ETH9427	Streptomyces_sp._FxanaD5	Streptomyces_sp._UNC401CLCol	Actinospica_acidiphila_NRRL_B_24431	Streptomyces_sp._HNS054	Streptomyces_sp._SMS_SU21	Streptomyces_sp._ZS0098	Streptomyces_thermocarboxydus_K155	Addition	Core-Accessory-Unique	COG	%Similarity_COG	Score_COG	Evalue_COG	KEGG	%Similarity_KEGG	Score_KEGG	Evalue_KEGG
2628	WP_102641453.1	-	spermidine/putrescine ABC transporter substrate-binding protein	1	1	0	1	1	1	1	1	1	1	1	10	Accessory	COG0687_E#	28.4	155	4.84E-37	K11069	26.8	114	2.53E-24
2629	Prokka:JAPCLKMA_06842	-	IS256 family transposase ISRjo4	1	0	0	0	0	0	0	0	0	0	0	1	Unique	COG3328_L#	47.8	175	4.52E-43	K07493	27.9	139	7.34E-32
2630	OSC69063.1	-	ATP-binding protein	0	1	0	0	0	0	0	0	0	0	0	1	Unique	COG0642_T#	33.1	72	5.39E-12	NA	33.1	72	1.44E-11
2631	PWE11140.1	-	MCE-family lipoprotein LprK (MCE-family lipoprotein Mce1e)	0	0	1	0	1	1	1	1	1	1	1	8	Accessory	COG1463_Q#	37.8	211	7.43E-54	K02067	37.8	211	1.99E-53
2632	OSC73080.1	-	D-alanyl-D-alanine carboxypeptidase	1	1	1	1	1	1	1	1	1	1	1	11	CORE	COG1686_M#	41.2	183	2.17E-45	K07258	41.2	183	5.79E-45
2633	OSC61601.1	-	twin-arginine translocation pathway signal protein	0	1	0	0	0	0	0	0	0	0	0	1	Unique	COG3119_P#	31.4	204	6.96E-52	NA	32.5	201	2.05E-50
2634	OSC57054.1	-	ABC transporter permease	0	1	0	0	0	0	0	0	0	0	0	1	Unique	COG0601_EP#	34.4	139	2.1E-32	K15585	35.2	124	2.44E-27
2635	AXI91417.1	-	hypothetical protein	0	0	0	1	0	0	0	0	0	0	0	1	Unique								

cluster	GI	gene	Annotation	Streptomyces_thermocarboxydus_BPSAC147	Streptomyces_sp._4F	Streptomyces_sp._BSE7F	Streptomyces_sp._ETH9427	Streptomyces_sp._FxanaD5	Streptomyces_sp._UNC401CLCol	Actinospica_acidiphila_NRRL_B_24431	Streptomyces_sp._HNS054	Streptomyces_sp._SMS_SU21	Streptomyces_sp._ZS0098	Streptomyces_thermocarboxydus_K155	Addition	Core-Accessory-Unique	COG	%Similarity_COG	Score_COG	Evalue_COG	KEGG	%Similarity_KEGG	Score_KEGG	Evalue_KEGG
2636	AXI89599.1	-	hypothetical protein	0	0	0	1	0	0	0	0	0	0	0	1	Unique								
2637	WP_019522549.1	-	sugar ABC transporter permease	1	1	1	1	1	1	1	1	1	1	1	11	CORE	COG4214_G#	58.6	444	3.99E-124	K10544	40.3	272	7.24E-72
2638	WP_019526361.1	-	HAMP domain-containing histidine kinase	1	1	0	1	1	1	1	1	1	1	1	10	Accessory	COG0642_T#	34.9	152	3.13E-36	NA	28.2	133	4.02E-30
2639	WP_122216277.1	-	hypothetical protein	0	0	0	0	0	0	0	0	0	1	0	1	Unique								
2640	WP_033273644.1	-	PE-PGRS family protein	0	0	0	0	0	0	1	0	0	0	0	1	Unique	COG4268_V#	25.4	94	1.32E-18				
2641	AXI85278.1	-	acyl-CoA dehydrogenase	1	0	0	1	0	0	0	0	0	0	0	2	Accessory	COG1960_I#	28.6	181	4.82E-45	K00257	28.2	173	4.58E-42
2642	Prokka:JAPCLKMA_01550	purD_1	Phosphoribosylamine--glycine ligase	1	0	0	1	0	0	0	0	0	0	0	2	Accessory	COG0439_I#	30.6	135	5.17E-31	K14755	29.4	95	1.21E-18
2643	WP_048456447.1	-	geranylgeranyl reductase family protein	1	1	1	1	1	1	1	1	1	1	1	11	CORE	COG0644_C#	34.2	139	2.1E-32	NA	31.3	127	2.88E-28
2644	PWE09830.1	-	FAD-dependent oxidoreductase	0	0	1	1	0	0	1	1	1	1	0	6	Accessory	COG1233_Q#	36.8	188	3.94E-47	NA	35	210	2.59E-53
2645	WP_019524860.1	-	hypothetical protein	0	0	0	0	1	1	0	0	0	0	1	3	Accessory	COG1960_I#	32.2	194	9.38E-49	K16047	31.9	156	5.79E-37
2646	Prokka:JAPCLKMA_01398	-	hypothetical protein	1	1	1	1	1	1	1	1	1	1	1	11	CORE								

cluster	GI	gene	Annotation	Streptomyces_thermocarboxydus_BPSAC147	Streptomyces_sp._4F	Streptomyces_sp._BSE7F	Streptomyces_sp._ETH9427	Streptomyces_sp._FxanaD5	Streptomyces_sp._UNC401CLCol	Actinospica_acidiphila_NRRL_B_24431	Streptomyces_sp._HNS054	Streptomyces_sp._SMS_SU21	Streptomyces_sp._ZS0098	Streptomyces_thermocarboxydus_K155	Addition	Core-Accessory-Unique	COG	%Similarity_COG	Score_COG	Evalue_COG	KEGG	%Similarity_KEGG	Score_KEGG	Evalue_KEGG
2647	AXI84933.1	-	cation:proton antiporter	0	0	0	1	0	0	0	1	1	1	0	4	Accessory	COG0475_P#	65.5	475	2.75E-133	K03455	65.5	475	7.35E-133
2648	WP_019526227.1	-	hypothetical protein	1	1	1	1	1	1	1	1	1	1	1	11	CORE								
2649	WP_122215358.1	-	hypothetical protein	1	1	1	1	0	1	0	1	1	1	1	9	Accessory	COG4796_U#	34.7	42	0.00349	NA	29.6	121	2.7E-26
2650	Prokka:JAPCLKMA_00488	-	hypothetical protein	1	0	0	0	0	0	0	0	0	0	0	1	Unique	COG4279_S#	38.7	159	2.56E-38	NA	39.7	142	8.65E-33
2651	OSC67541.1	-	helicase	0	1	0	0	0	0	0	0	0	0	0	1	Unique								
2652	WP_033276217.1	-	ABC transporter permease	0	0	0	0	0	0	1	0	0	0	0	1	Unique	COG0577_V#	29.4	145	5E-34	K02004	29.2	130	3.4E-29
2653	OSC71320.1	-	two-component sensor histidine kinase	0	1	0	0	0	0	0	0	0	0	0	1	Unique	COG0642_T#	31.5	132	3.35E-30	K07654	31.5	132	8.95E-30
2654	OSC61104.1	-	acyltransferase	1	1	1	1	1	1	1	1	1	1	1	11	CORE								
2655	OSC71454.1	-	phosphoserine phosphatase SerB	1	1	1	1	1	1	1	1	1	1	1	11	CORE	COG0560_E#	65.8	286	1.39E-76	K01079	50.5	365	6.28E-100
2656	WP_033273483.1	-	SpoIIE family protein phosphatase	1	0	1	1	1	1	1	1	1	1	1	10	Accessory	COG2208_TK#	31.7	121	5.9E-27	NA	28.7	137	2.78E-31
2657	WP_102639858.1	-	MFS transporter	1	1	1	1	1	1	1	1	1	1	1	11	CORE	COG2814_G#	44.7	328	3.18E-89	K08156	43.2	292	6.74E-78
2658	WP_033275464.1	-	ATP-grasp domain-containing protein	1	0	0	1	1	1	1	1	0	1	1	8	Accessory	COG0439_I#	27.6	131	7.45E-30	K14755	26.1	80	4.03E-14

cluster	GI	gene	Annotation	Streptomyces_thermocarboxydus_BPSAC147	Streptomyces_sp._4F	Streptomyces_sp._BSE7F	Streptomyces_sp._ETH9427	Streptomyces_sp._FxanaD5	Streptomyces_sp._UNC401CLCol	Actinospica_acidiphila_NRRL_B_24431	Streptomyces_sp._HNS054	Streptomyces_sp._SMS_SU21	Streptomyces_sp._ZS0098	Streptomyces_thermocarboxydus_K155	Addition	Core-Accessory-Unique	COG	%Similarity_COG	Score_COG	Evalue_COG	KEGG	%Similarity_KEGG	Score_KEGG	Evalue_KEGG
2659	WP_033274374.1	-	sugar ABC transporter substrate-binding protein	1	0	0	0	1	1	1	0	0	1	1	6	Accessory	COG1653_G#	36.2	262	2.8E-69	K15770	26.6	132	1.17E-29
2660	WP_078493043.1	-	hypothetical protein	1	1	1	1	1	1	1	1	1	1	1	11	CORE								
2661	OSC69911.1	-	two-component sensor histidine kinase	1	1	1	1	1	1	1	1	1	1	1	11	CORE	COG4585_T#	30.5	97	1.56E-19	K07778	30.5	97	4.16E-19
2662	WP_033275741.1	-	helix-turn-helix domain-containing protein	0	0	1	0	0	0	1	1	1	1	0	5	Accessory	COG2508_TQ#	29.5	70	1.2E-11	NA	29.5	70	3.19E-11
2663	OSC61284.1	-	transcriptional regulator	0	1	0	0	0	0	0	0	0	0	0	1	Unique	COG1940_KG#	29.2	168	5.5E-41	NA	26.7	135	1.06E-30
2664	Prokka:CIODPCDJ_02239	-	hypothetical protein	1	1	1	1	1	1	1	1	1	1	1	11	CORE								
2665	Prokka:CIODPCDJ_02332	xdh	D-xylose dehydrogenase	1	1	1	1	1	1	1	1	1	1	1	11	CORE	COG0673_R#	61.7	482	1.72E-135	NA	26	61	1.94E-08
2666	WP_121719763.1	-	helix-turn-helix domain-containing protein	0	0	1	1	1	1	1	1	1	1	1	9	Accessory	COG2508_TQ#	28.1	53	0.00000258	NA	28.1	53	0.00000689
2667	OSC73184.1	-	glycerate kinase	0	1	0	0	0	0	0	0	0	0	0	1	Unique	COG1929_G#	44.8	307	5.81E-83	K00865	44.9	303	3.82E-81
2668	WP_078895225.1	-	hypothetical protein	1	1	1	1	1	1	1	1	1	1	1	11	CORE					NA	39	47	0.000289
2669	Prokka:JAPCLKMA_04548	-	IS256 family transposase IS1245	1	0	1	0	0	0	0	0	0	0	1	3	Accessory	COG3328_L#	58.3	462	1.84E-129	K07493	58.3	462	4.92E-129

cluster	GI	gene	Annotation	Streptomyces_thermocarboxydus_BPSAC147	Streptomyces_sp._4F	Streptomyces_sp._BSE7F	Streptomyces_sp._ETH9427	Streptomyces_sp._FxanaD5	Streptomyces_sp._UNC401CLCol	Actinospica_acidiphila_NRRL_B_24431	Streptomyces_sp._HNS054	Streptomyces_sp._SMS_SU21	Streptomyces_sp._ZS0098	Streptomyces_thermocarboxydus_K155	Addition	Core-Accessory-Unique	COG	%Similarity_COG	Score_COG	Evalue_COG	KEGG	%Similarity_KEGG	Score_KEGG	Evalue_KEGG
2670	OSC72285.1	-	hypothetical protein	1	1	0	0	1	1	1	0	0	0	1	6	Accessory								
2671	OSC71582.1	-	adenylate cyclase	0	1	0	0	0	0	0	0	0	0	0	1	Unique	COG2114_T#	27.5	58	6.14E-08				
2672	Prokka:JAPCLKMA_06329	-	hypothetical protein	1	0	0	1	0	0	0	0	0	0	0	2	Accessory	COG3328_L#	54.3	361	3.39E-99	K07493	54.3	361	9.05E-99
2673	Prokka:CIODPCDJ_00719	-	hypothetical protein	1	1	1	1	1	1	1	1	1	1	1	11	CORE								
2674	Prokka:JAPCLKMA_01498	-	IS110 family transposase IS117	1	0	0	0	0	0	0	0	0	0	0	1	Unique	COG3547_L#	31	65	5.02E-10	NA	31	65	1.34E-09
2675	WP_028959959.1	-	MFS transporter	1	0	1	1	1	1	1	1	1	1	1	10	Accessory								
2676	WP_019524901.1	-	MFS transporter	0	0	0	0	1	0	0	0	0	0	0	1	Unique	COG0477_GEPR#	24.7	117	1.11E-25	K08225	25.9	101	1.69E-20
2677	Prokka:JAPCLKMA_05695	-	hypothetical protein	1	0	0	0	1	1	0	0	0	1	1	5	Accessory	COG0476_H#	32.8	100	1.4E-20	K11996	29.7	92	1.33E-17
2678	WP_122215184.1	-	MFS transporter	1	0	1	0	0	0	1	0	1	1	1	6	Accessory	COG0477_GEPR#	31.4	162	3.93E-39	K08189	25.7	85	1.25E-15
2679	Prokka:CIODPCDJ_00750	murT	Lipid II isoglutaminyl synthase (glutamine-hydrolyzing) subunit MurT	1	1	1	1	1	1	1	1	1	1	1	11	CORE	COG0769_M#	44.6	343	9.53E-94	NA	44.6	343	2.54E-93
2680	Prokka:CIODPCDJ_00615	nanK_1	N-acetylmannosamine kinase	1	1	1	1	1	1	1	1	1	1	1	11	CORE	COG1940_KG#	27.2	111	1.04E-23	K00845	30.4	107	4.01E-22

cluster	GI	gene	Annotation	Streptomyces_thermocarboxydus_BPSAC147	Streptomyces_sp._4F	Streptomyces_sp._BSE7F	Streptomyces_sp._ETH9427	Streptomyces_sp._FxanaD5	Streptomyces_sp._UNC401CLCol	Actinospica_acidiphila_NRRL_B_24431	Streptomyces_sp._HNS054	Streptomyces_sp._SMS_SU21	Streptomyces_sp._ZS0098	Streptomyces_thermocarboxydus_K155	Addition	Core-Accessory-Unique	COG	%Similarity_COG	Score_COG	Evalue_COG	KEGG	%Similarity_KEGG	Score_KEGG	Evalue_KEGG
2681	WP_020699974.1	-	histidine kinase	1	1	1	0	1	1	1	1	1	1	1	10	Accessory	COG4564_T#	26.5	78	5.72E-14				
2682	WP_019525553.1	-	cytochrome P450	0	0	0	0	1	1	0	0	0	0	1	3	Accessory	COG2124_Q#	25.8	110	1.36E-23	K00517	23	74	3.76E-12
2683	WP_019526848.1	-	FAD-dependent oxidoreductase	0	0	0	0	1	1	0	0	0	0	1	3	Accessory	COG0446_R#	45.3	348	2.96E-95	K00529	36.8	227	2.66E-58
2684	WP_048460359.1	-	helix-turn-helix domain-containing protein	0	0	1	1	0	0	0	1	1	1	0	5	Accessory								
2685	WP_122213158.1	-	lycopene cyclase	1	1	1	0	1	1	1	1	1	1	1	10	Accessory								
2686	Prokka:JAPCLKMA_04483	putR	Proline-responsive transcriptional activator PutR	1	1	0	1	1	1	1	1	1	1	1	10	Accessory	COG2508_TQ#	27.4	89	3.24E-17	NA	27.4	89	8.65E-17
2687	AXI90012.1	-	hypothetical protein	0	1	1	1	1	1	1	1	1	1	1	10	Accessory								
2688	PWE10270.1	-	beta-ketoacyl-[acyl-carrier-protein] synthase family protein	0	0	1	0	0	0	0	0	0	0	0	1	Unique	COG0304_IQ#	35.7	243	1.75E-63	K09458	35.7	243	4.68E-63
2689	WP_028961380.1	-	AAA family ATPase	0	0	0	0	0	1	0	0	0	0	0	1	Unique	COG4637_R#	26.8	125	4.08E-28				
2690	WP_122217072.1	-	hypothetical protein	1	1	1	1	1	1	1	1	1	1	1	11	CORE								

cluster	GI	gene	Annotation	Streptomyces_thermocarboxydus_BPSAC147	Streptomyces_sp._4F	Streptomyces_sp._BSE7F	Streptomyces_sp._ETH9427	Streptomyces_sp._FxanaD5	Streptomyces_sp._UNC401CLCol	Actinospica_acidiphila_NRRL_B_24431	Streptomyces_sp._HNS054	Streptomyces_sp._SMS_SU21	Streptomyces_sp._ZS0098	Streptomyces_thermocarboxydus_K155	Addition	Core-Accessory-Unique	COG	%Similarity_COG	Score_COG	Evalue_COG	KEGG	%Similarity_KEGG	Score_KEGG	Evalue_KEGG
2691	WP_033274659.1	-	FAD-dependent oxidoreductase	0	0	0	0	0	0	1	0	0	0	0	1	Unique	COG0446_R#	41.6	254	5.82E-67	NA	41.6	254	1.55E-66
2692	WP_078611383.1	-	IS701 family transposase	0	0	0	0	0	1	0	0	0	0	0	1	Unique	COG5659_L#	41.5	262	2.14E-69	NA	23.9	89	6.62E-17
2693	OSC57409.1	-	hypothetical protein	1	1	1	1	1	1	1	1	1	1	1	11	CORE	COG1376_S#	33.7	220	1.22E-56	NA	33.7	220	3.25E-56
2694	OSC70854.1	-	FAD-dependent oxidoreductase	1	1	1	1	1	1	1	1	1	1	1	11	CORE	COG1251_C#	36.9	236	1.26E-61	K00362	36.8	203	4.11E-51
2695	Prokka:CIODPCDJ_06439	-	hypothetical protein	1	0	1	1	1	1	0	0	1	1	1	8	Accessory	COG0675_L#	49.5	344	4.28E-94	K07496	49.5	344	1.14E-93
2696	OSC60993.1	-	DEAD/DEAH box helicase	0	1	0	0	0	0	0	0	0	0	0	1	Unique	COG1204_R#	26.5	54	0.00000151				
2697	WP_048456303.1	-	serine hydrolase	0	0	0	0	0	0	0	1	0	0	0	1	Unique	COG1680_V#	28.9	140	1.23E-32	NA	26.8	97	2.43E-19
2698	OSC70306.1	-	cation/H(+) antiporter	0	1	0	1	0	0	0	1	0	0	0	3	Accessory	COG0475_P#	26.8	148	4.5E-35	K03455	26.8	148	1.2E-34
2699	WP_033272518.1	-	outer membrane lipoprotein carrier protein LolA	1	1	1	1	1	1	1	1	1	1	1	11	CORE								
2700	AXI86323.1	-	hypothetical protein	1	1	1	1	1	1	1	1	1	1	1	11	CORE								
2701	WP_078611361.1	-	MFS transporter	1	0	0	0	1	1	0	0	0	0	1	4	Accessory	COG0477_GEPR#	30.3	150	1.18E-35	K08224	27.7	74	2.2E-12

cluster	GI	gene	Annotation	Streptomyces_thermocarboxydus_BPSAC147	Streptomyces_sp._4F	Streptomyces_sp._BSE7F	Streptomyces_sp._ETH9427	Streptomyces_sp._FxanaD5	Streptomyces_sp._UNC401CLCol	Actinospica_acidiphila_NRRL_B_24431	Streptomyces_sp._HNS054	Streptomyces_sp._SMS_SU21	Streptomyces_sp._ZS0098	Streptomyces_thermocarboxydus_K155	Addition	Core-Accessory-Unique	COG	%Similarity_COG	Score_COG	Evalue_COG	KEGG	%Similarity_KEGG	Score_KEGG	Evalue_KEGG
2702	AXI89230.1	-	SAM-dependent methyltransferase	1	0	1	1	1	1	1	1	1	1	1	10	Accessory	COG0500_QR#	47.7	412	1.67E-114	NA	45.4	384	1.69E-105
2703	WP_019521467.1	-	MFS transporter	1	0	0	0	1	1	0	0	0	0	1	4	Accessory	COG0477_GEPR#	24.7	86	3.57E-16	K08223	26.5	58	1.63E-07
2704	WP_033275682.1	-	MFS transporter	1	1	1	1	1	1	1	1	1	1	1	11	CORE	COG0477_GEPR#	24.9	85	6.09E-16				
2705	WP_048458489.1	-	bifunctional cobalt-precorrin-7 (C(5))-methyltransferase/cobalt-precorrin-6B (C(15))-methyltransferase	1	1	1	1	1	1	1	1	1	1	1	11	CORE	COG2242_H#	42.6	110	1.77E-23	K00595	31.7	163	3.6E-39
2706	AXI88194.1	-	hypothetical protein	0	0	0	1	0	0	0	0	0	0	0	1	Unique								
2707	WP_102638552.1	-	sedoheptulose 7-phosphate cyclase	0	0	1	0	0	0	0	0	1	0	0	2	Accessory	COG0337_E#	31.7	156	2.15E-37	K01735	28.9	137	3.61E-31
2708	WP_122214612.1	-	serine hydrolase	1	1	1	1	1	1	1	1	1	1	1	11	CORE	COG1680_V#	43.3	284	6.84E-76	NA	37.8	232	8.24E-60
2709	Prokka:CIODPCDJ_00880	-	hypothetical protein	0	0	0	0	1	1	0	0	0	0	1	3	Accessory	COG4585_T#	36.7	134	8.76E-31	K07778	36.7	134	2.34E-30
2710	OSC57752.1	-	hypothetical protein	0	1	0	0	0	0	0	0	0	0	0	1	Unique								

cluster	GI	gene	Annotation	Streptomyces_thermocarboxydus_BPSAC147	Streptomyces_sp._4F	Streptomyces_sp._BSE7F	Streptomyces_sp._ETH9427	Streptomyces_sp._FxanaD5	Streptomyces_sp._UNC401CLCol	Actinospica_acidiphila_NRRL_B_24431	Streptomyces_sp._HNS054	Streptomyces_sp._SMS_SU21	Streptomyces_sp._ZS0098	Streptomyces_thermocarboxydus_K155	Addition	Core-Accessory-Unique	COG	%Similarity_COG	Score_COG	Evalue_COG	KEGG	%Similarity_KEGG	Score_KEGG	Evalue_KEGG
2711	PWE07639.1	-	AraC family transcriptional regulator	1	1	1	1	1	1	1	1	1	1	1	11	CORE	COG4977_K#	47.6	285	3.07E-76	NA	37.1	69	1.21E-10
2712	Prokka:JAPCLKMA_05633	-	hypothetical protein	1	0	0	0	0	0	0	0	0	0	0	1	Unique								
2713	WP_107503754.1	-	hypothetical protein	0	0	0	0	0	0	0	0	1	0	0	1	Unique								
2714	WP_122215316.1	-	alpha/beta fold hydrolase	1	0	1	1	1	1	1	1	1	1	1	10	Accessory	COG0596_R#	30.8	127	8.2E-29	NA	29	103	4.42E-21
2715	AXI85327.1	-	MFS transporter	1	0	0	1	0	0	0	0	0	0	0	2	Accessory								
2716	OSC65038.1	-	N-acyl-L-amino acid amidohydrolase	1	1	0	1	1	1	1	1	1	1	1	10	Accessory	COG1473_R#	50.3	356	1.85E-97	K01436	50.3	356	4.94E-97
2717	OSC65077.1	-	cysteine desulfurase	0	1	0	1	1	1	1	1	1	1	1	9	Accessory	COG0520_E#	32	189	3E-47	K11717	32	189	8E-47
2718	Prokka:CIODPCDJ_06409	lacI	Lactose operon repressor	1	1	0	1	1	1	1	1	1	1	1	10	Accessory	COG1609_K#	36.6	178	6.91E-44	NA	36.6	178	1.84E-43
2719	AXI90792.1	-	coproporphyrinogen III oxidase	1	1	0	1	1	1	1	1	1	1	1	10	Accessory	COG0635_H#	55.5	409	1.08E-113	K02495	56	408	6.41E-113
2720	WP_019524532.1	-	cytochrome P450	1	1	1	1	1	1	1	1	1	1	1	11	CORE	COG2124_Q#	28.5	148	5.85E-35	K16046	26.5	117	2.26E-25
2721	WP_019525472.1	-	CoA transferase	1	1	1	1	1	1	1	1	1	1	1	11	CORE	COG1804_C#	43.4	331	3.73E-90	K18703	30.3	183	5.72E-45
2722	OSC70366.1	-	acetyl-CoA acetyltransferase	1	1	1	1	1	1	1	1	1	1	1	11	CORE	COG0183_I#	67	511	3.43E-144	K00626	67	511	9.17E-144

cluster	GI	gene	Annotation	Streptomyces_thermocarboxydus_BPSAC147	Streptomyces_sp._4F	Streptomyces_sp._BSE7F	Streptomyces_sp._ETH9427	Streptomyces_sp._FxanaD5	Streptomyces_sp._UNC401CLCol	Actinospica_acidiphila_NRRL_B_24431	Streptomyces_sp._HNS054	Streptomyces_sp._SMS_SU21	Streptomyces_sp._ZS0098	Streptomyces_thermocarboxydus_K155	Addition	Core-Accessory-Unique	COG	%Similarity_COG	Score_COG	Evalue_COG	KEGG	%Similarity_KEGG	Score_KEGG	Evalue_KEGG
2723	WP_033273581.1	-	ROK family protein	1	0	0	1	1	1	1	1	1	1	1	9	Accessory	COG1940_KG#	35.1	163	1.76E-39	NA	26.2	134	3.05E-30
2724	WP_033273054.1	-	ADP-ribosylglycohydrolase family protein	1	0	1	0	1	1	1	1	1	1	1	9	Accessory								
2725	WP_122215750.1	frc	formyl-CoA transferase	1	1	1	1	1	1	1	1	1	1	1	11	CORE	COG1804_C#	56.8	460	5.32E-129	K07749	56.8	460	1.42E-128
2726	Prokka:CIODPCDJ_04006	-	hypothetical protein	1	1	1	1	1	1	1	1	1	1	1	11	CORE								
2727	Prokka:CIODPCDJ_05685	-	hypothetical protein	1	0	0	0	0	0	0	0	1	1	1	4	Accessory								
2728	OSC64276.1	-	aldehyde dehydrogenase	0	1	0	0	0	0	0	0	0	0	0	1	Unique	COG1012_C#	41.3	283	1.16E-75	K17761	39.2	285	8.18E-76
2729	OSC57459.1	-	MFS transporter	0	1	1	1	1	1	1	1	1	1	1	10	Accessory	COG0477_GEPR#	29	77	1.27E-13	K18214	24.2	60	4.28E-08
2730	AXI91413.1	-	DUF2637 domain-containing protein	0	0	0	1	0	0	0	0	0	0	0	1	Unique	COG3468_MU#	31.3	83	1.77E-15	NA	34.3	91	2.27E-17
2731	WP_033277961.1	-	acyl-CoA dehydrogenase	1	0	0	0	1	1	1	0	0	0	1	5	Accessory	COG1960_I#	30.9	195	5.45E-49				
2732	Prokka:CIODPCDJ_03136	-	hypothetical protein	0	0	0	0	0	1	0	0	0	0	1	2	Accessory								
2733	OSC69650.1	-	two-component sensor histidine kinase	0	1	0	0	0	0	0	0	0	0	0	1	Unique	COG4585_T#	28.2	82	5.13E-15	K07778	28.2	82	1.37E-14

cluster	GI	gene	Annotation	Streptomyces_thermocarboxydus_BPSAC147	Streptomyces_sp._4F	Streptomyces_sp._BSE7F	Streptomyces_sp._ETH9427	Streptomyces_sp._FxanaD5	Streptomyces_sp._UNC401CLCol	Actinospica_acidiphila_NRRL_B_24431	Streptomyces_sp._HNS054	Streptomyces_sp._SMS_SU21	Streptomyces_sp._ZS0098	Streptomyces_thermocarboxydus_K155	Addition	Core-Accessory-Unique	COG	%Similarity_COG	Score_COG	Evalue_COG	KEGG	%Similarity_KEGG	Score_KEGG	Evalue_KEGG
2734	PWE06584.1	-	DUF349 domain-containing protein	1	1	1	1	1	1	1	1	1	1	1	11	CORE					NA	44.7	320	2.29E-86
2735	WP_020700194.1	-	hypothetical protein	1	0	1	1	1	1	1	1	1	0	1	9	Accessory								
2736	PWE06731.1	mshC	cysteine--1-D-myo-inositol 2-amino-2-deoxy-alpha-D-glucopyranoside ligase	1	1	1	1	1	1	1	1	1	1	1	11	CORE	COG0215_J#	54.5	462	1.4E-129	K15526	54.5	462	3.73E-129
2737	OSC72392.1	-	threonine ammonia-lyase	1	1	1	1	1	1	1	1	1	1	1	11	CORE	COG1171_E#	47.2	363	8.83E-100	K01754	42.8	298	9.32E-80
2738	OSC70239.1	-	lipase	1	1	1	1	1	1	1	1	1	1	1	11	CORE								
2739	WP_107081026.1	-	MFS transporter	1	1	1	1	1	1	1	1	1	1	1	11	CORE	COG0477_GEPR#	34.4	166	1.58E-40	NA	34.4	166	4.23E-40
2740	WP_048460451.1	ccrA	crotonyl-CoA carboxylase/reductase	0	1	1	1	0	0	1	1	1	1	0	7	Accessory	COG0604_CR#	47.8	381	3.13E-105	K13953	27.8	69	1.2E-10
2741	WP_019523092.1	-	hypothetical protein	1	1	1	1	1	1	1	1	1	1	1	11	CORE								
2742	AXI84771.1	-	serine protease	0	0	1	1	1	1	1	1	1	1	1	9	Accessory	COG1404_O#	33.3	132	3.31E-30	K14743	33.3	132	8.84E-30
2743	WP_033276316.1	-	DUF2236 domain-containing protein	1	0	0	0	1	1	1	0	0	0	1	5	Accessory								
2744	OSC63950.1	-	cytochrome	0	1	0	0	0	0	0	0	0	0	0	1	Unique	COG2124_Q#	38.9	272	2.68E-72	K15468	29.8	174	2.65E-42

cluster	GI	gene	Annotation	Streptomyces_thermocarboxydus_BPSAC147	Streptomyces_sp._4F	Streptomyces_sp._BSE7F	Streptomyces_sp._ETH9427	Streptomyces_sp._FxanaD5	Streptomyces_sp._UNC401CLCol	Actinospica_acidiphila_NRRL_B_24431	Streptomyces_sp._HNS054	Streptomyces_sp._SMS_SU21	Streptomyces_sp._ZS0098	Streptomyces_thermocarboxydus_K155	Addition	Core-Accessory-Unique	COG	%Similarity_COG	Score_COG	Evalue_COG	KEGG	%Similarity_KEGG	Score_KEGG	Evalue_KEGG
2745	WP_122212558.1	-	alkaline phosphatase family protein	1	1	1	1	1	1	1	1	1	1	1	11	CORE	COG1524_R#	23.2	82	5.13E-15				
2746	Prokka:JAPCLKMA_03064	add_1	Adenosine deaminase	1	1	1	1	1	1	1	1	1	1	1	11	CORE	COG1816_F#	68.3	482	1.3E-135	K01488	68.3	482	3.48E-135
2747	OSC62681.1	-	hypothetical protein	0	1	0	0	0	0	0	0	0	0	0	1	Unique	COG0477_GEPR#	31.5	198	4.91E-50	NA	31.5	198	1.31E-49
2748	PWE09390.1	-	hypothetical protein	0	0	1	1	0	0	0	1	0	0	1	4	Accessory	COG3440_V#	42.2	107	1.49E-22	K07454	42.2	107	3.98E-22
2749	OSC61879.1	-	serine hydrolase	1	1	1	1	1	1	1	1	1	1	1	11	CORE	COG1680_V#	44.9	327	5.37E-89	NA	40.8	220	3.23E-56
2750	OSC67054.1	-	hypothetical protein	1	1	1	1	1	1	1	1	1	1	1	11	CORE								
2751	WP_048460601.1	-	cytochrome P450	0	0	0	1	0	0	0	1	0	0	0	2	Accessory	COG2124_Q#	34	222	2.43E-57	K16593	34	222	6.5E-57
2752	OSC65374.1	-	dolichol-phosphate mannosyltransferase	0	1	0	0	0	0	0	0	0	0	0	1	Unique	COG0463_M#	43.2	157	9.61E-38	K00721	33.7	169	5E-41
2753	WP_122212591.1	-	hypothetical protein	1	0	0	1	1	1	0	1	0	1	1	7	Accessory								
2754	WP_102637318.1	-	hydroxymethylglutaryl-CoA synthase family protein	0	0	0	0	0	0	0	0	1	0	0	1	Unique	COG3425_I#	43	286	1.37E-76	K15311	42.7	346	2.98E-94
2755	OSC62896.1	-	MFS transporter	0	1	0	0	0	0	0	0	0	0	0	1	Unique	COG0477_GEPR#	32.4	203	1.53E-51	NA	31.1	185	1.15E-45
2756	WP_048458087.1	-	pyridoxal phosphate-dependent aminotransferase	1	1	1	1	1	1	1	1	1	1	1	11	CORE	COG0436_E#	46.9	362	1.96E-99	K00812	46.9	362	5.24E-99

cluster	GI	gene	Annotation	Streptomyces_thermocarboxydus_BPSAC147	Streptomyces_sp._4F	Streptomyces_sp._BSE7F	Streptomyces_sp._ETH9427	Streptomyces_sp._FxanaD5	Streptomyces_sp._UNC401CLCol	Actinospica_acidiphila_NRRL_B_24431	Streptomyces_sp._HNS054	Streptomyces_sp._SMS_SU21	Streptomyces_sp._ZS0098	Streptomyces_thermocarboxydus_K155	Addition	Core-Accessory-Unique	COG	%Similarity_COG	Score_COG	Evalue_COG	KEGG	%Similarity_KEGG	Score_KEGG	Evalue_KEGG
2757	WP_122216615.1	-	cation:proton antiporter	0	0	1	0	0	0	0	0	1	1	0	3	Accessory	COG0475_P#	30	182	2.78E-45	K03455	29.8	183	5.69E-45
2758	WP_019524735.1	rocD	ornithine--oxo-acid transaminase	1	1	1	1	1	1	1	1	1	1	1	11	CORE	COG4992_E#	50.8	414	5.69E-115	K00819	50.8	414	1.52E-114
2759	WP_048460311.1	-	membrane protein	1	1	1	1	1	1	1	1	1	1	1	11	CORE								
2760	WP_019524721.1	-	acyl-CoA dehydrogenase	1	1	1	1	1	1	1	1	1	1	1	11	CORE	COG1960_I#	40.7	272	2.05E-72	NA	39.8	266	5.11E-70
2761	OSC58910.1	-	MFS transporter	0	1	0	0	0	0	0	0	0	0	0	1	Unique	COG0477_GEPR#	34.5	179	3.08E-44	NA	34.5	179	8.22E-44
2762	WP_033273703.1	-	DUF4185 domain-containing protein	0	0	1	1	0	0	1	1	1	1	0	6	Accessory								
2763	WP_019522444.1	-	cytochrome P450	1	1	1	1	1	1	1	1	1	1	1	11	CORE	COG2124_Q#	41.3	312	1.78E-84	K17474	41.3	312	4.76E-84
2764	AXI84811.1	-	hypothetical protein	0	0	0	1	0	0	0	1	0	1	0	3	Accessory					NA	35.8	107	3.97E-22
2765	Prokka:JAPCLKMA_02703	efeN	putative deferrochelatase/peroxidase EfeN	1	0	0	0	1	1	0	0	0	0	1	4	Accessory	COG2837_P#	41.5	317	5.54E-86	K16301	41.5	317	1.48E-85
2766	PWE10565.1	-	polyketide beta-ketoacyl:ACP synthase	0	0	1	0	0	0	0	0	1	0	0	2	Accessory	COG0304_IQ#	32.5	205	5.24E-52	K00646	32.6	208	9.69E-53
2767	WP_033274681.1	-	sensor histidine kinase	0	1	0	0	1	1	1	0	0	0	1	5	Accessory	COG4585_T#	25.9	84	1.03E-15	NA	25.9	84	2.76E-15
2768	AXI91084.1	-	sodium:proton exchanger	0	0	0	1	0	0	0	0	0	0	0	1	Unique	COG0025_P#	40.7	270	1.33E-71	K03316	30.7	161	1.36E-38

cluster	GI	gene	Annotation	Streptomyces_thermocarboxydus_BPSAC147	Streptomyces_sp._4F	Streptomyces_sp._BSE7F	Streptomyces_sp._ETH9427	Streptomyces_sp._FxanaD5	Streptomyces_sp._UNC401CLCol	Actinospica_acidiphila_NRRL_B_24431	Streptomyces_sp._HNS054	Streptomyces_sp._SMS_SU21	Streptomyces_sp._ZS0098	Streptomyces_thermocarboxydus_K155	Addition	Core-Accessory-Unique	COG	%Similarity_COG	Score_COG	Evalue_COG	KEGG	%Similarity_KEGG	Score_KEGG	Evalue_KEGG
2769	WP_026244239.1	-	site-specific integrase	1	0	0	0	1	0	0	0	0	0	0	2	Accessory	COG0582_L#	27.3	117	1.1E-25				
2770	PWE11002.1	-	MFS transporter	0	1	1	1	0	0	0	1	0	1	0	5	Accessory	COG0477_GEPR#	37	196	1.43E-49	NA	37	196	3.81E-49
2771	WP_102642247.1	-	cytochrome P450	0	0	1	0	0	0	0	0	1	0	0	2	Accessory	COG2124_Q#	34.8	202	3.4E-51	K15468	31.8	198	1.31E-49
2772	WP_122214393.1	-	FAD-dependent oxidoreductase	1	1	1	1	1	1	1	1	1	1	1	11	CORE	COG1252_C#	31.2	117	8.42E-26	K03885	27.2	121	2.03E-26
2773	OSC70578.1	-	hypothetical protein	0	1	0	0	0	0	0	0	0	0	0	1	Unique								
2774	WP_106959407.1	-	serine/threonine-protein phosphatase	0	0	1	1	0	1	0	1	1	1	0	6	Accessory								
2775	WP_033278074.1	-	hypothetical protein	0	0	0	0	0	0	1	0	0	0	0	1	Unique								
2776	OSC64411.1	-	hypothetical protein	0	1	0	0	0	0	0	0	0	0	0	1	Unique								
2777	WP_102640434.1	fahA	fumarylacetoacetase	1	1	1	1	1	1	1	1	1	1	1	11	CORE	COG0179_Q#	46.4	353	1.19E-96	K01555	45.2	362	4.01E-99
2778	OSC71049.1	-	hypothetical protein	0	1	0	0	0	0	0	0	0	0	0	1	Unique	COG1454_C#	24.9	72	3.11E-12				
2779	WP_102641564.1	lhgO	L-2-hydroxyglutarate oxidase	1	1	1	1	1	1	1	1	1	1	1	11	CORE	COG0579_R#	52	381	5.32E-105	K15736	49.9	376	3.5E-103
2780	PWE10806.1	-	AAA family ATPase	1	1	1	1	0	1	1	1	0	1	1	9	Accessory	COG0714_R#	32.6	146	2.88E-34	NA	32.6	146	7.69E-34
2781	PWE08873.1	-	ATP/GTP-binding protein	1	0	1	1	1	1	0	1	1	1	1	9	Accessory								

cluster	GI	gene	Annotation	Streptomyces_thermocarboxydus_BPSAC147	Streptomyces_sp._4F	Streptomyces_sp._BSE7F	Streptomyces_sp._ETH9427	Streptomyces_sp._FxanaD5	Streptomyces_sp._UNC401CLCol	Actinospica_acidiphila_NRRL_B_24431	Streptomyces_sp._HNS054	Streptomyces_sp._SMS_SU21	Streptomyces_sp._ZS0098	Streptomyces_thermocarboxydus_K155	Addition	Core-Accessory-Unique	COG	%Similarity_COG	Score_COG	Evalue_COG	KEGG	%Similarity_KEGG	Score_KEGG	Evalue_KEGG
2782	WP_122213492.1	-	MFS transporter	1	1	1	1	1	1	1	1	1	1	1	11	CORE	COG0477_GEPR#	48.4	366	1.04E-100	NA	48.4	366	2.77E-100
2783	Prokka:CIODPCDJ_05179	cbiN	Cobalt transport protein CbiN	1	1	1	1	1	1	1	1	1	1	1	11	CORE	COG1930_P#	52.1	106	2.53E-22	K02008	27.6	92	1.32E-17
2784	OSC71069.1	-	hypothetical protein	0	1	0	0	0	0	0	0	0	0	0	1	Unique	COG0463_M#	36.4	102	3.65E-21	K16698	35.6	67	2.66E-10
2785	OSC59425.1	-	hypothetical protein	0	1	0	0	0	0	0	0	0	0	0	1	Unique								
2786	Prokka:JAPCLKMA_00664	-	hypothetical protein	1	1	1	1	1	1	1	1	1	1	1	11	CORE								
2787	Prokka:CIODPCDJ_01343	-	hypothetical protein	1	1	1	1	1	1	1	1	1	1	1	11	CORE	COG0671_I#	30.2	66	2.22E-10	NA	27.8	60	5.55E-08
2788	Prokka:CIODPCDJ_01798	-	hypothetical protein	1	1	1	1	1	1	1	1	1	1	1	11	CORE	COG2087_H#	44.9	133	1.93E-30	K02231	44.9	133	5.16E-30
2789	OSC57442.1	-	4-hydroxybenzoate 3-monooxygenase	1	1	1	1	1	1	1	1	1	1	1	11	CORE	COG0654_HC#	38.5	280	9.79E-75	NA	48.9	45	0.00108
2790	OSC57354.1	-	cystathionine gamma-synthase	1	1	1	1	1	1	1	1	1	1	1	11	CORE	COG0626_E#	45.1	258	5.2E-68	K01761	45.1	258	1.39E-67
2791	AXI87933.1	-	acetyl-CoA C-acetyltransferase	1	1	1	1	1	1	1	1	1	1	1	11	CORE	COG0183_I#	74.4	602	1.12E-171	K00626	74.4	602	3E-171
2792	Prokka:JAPCLKMA_06669	-	Carboxynorspermidine synthase	1	0	1	1	1	1	1	1	1	1	1	10	Accessory	COG1748_E#	30.5	192	2.68E-48	K00290	30.3	178	1.4E-43

cluster	GI	gene	Annotation	Streptomyces_thermocarboxydus_BPSAC147	Streptomyces_sp._4F	Streptomyces_sp._BSE7F	Streptomyces_sp._ETH9427	Streptomyces_sp._FxanaD5	Streptomyces_sp._UNC401CLCol	Actinospica_acidiphila_NRRL_B_24431	Streptomyces_sp._HNS054	Streptomyces_sp._SMS_SU21	Streptomyces_sp._ZS0098	Streptomyces_thermocarboxydus_K155	Addition	Core-Accessory-Unique	COG	%Similarity_COG	Score_COG	Evalue_COG	KEGG	%Similarity_KEGG	Score_KEGG	Evalue_KEGG
2793	Prokka:JAPCLKMA_03810	-	hypothetical protein	1	0	0	0	1	1	1	0	0	0	1	5	Accessory	COG4585_T#	33.8	102	4.76E-21	NA	33.8	102	1.27E-20
2794	WP_019524290.1	-	cytochrome P450	1	1	1	1	1	1	1	1	1	1	1	11	CORE	COG2124_Q#	42.5	317	5.52E-86	K15468	37.3	262	7.35E-69
2795	WP_122216508.1	-	cysteine desulfurase-like protein	1	0	1	1	1	1	1	1	1	1	1	10	Accessory	COG0520_E#	38.8	276	1.41E-73	NA	40.9	230	4.04E-59
2796	OSC64638.1	-	glucose-1-phosphate adenylyltransferase	1	1	1	1	1	1	1	1	1	1	1	11	CORE	COG0448_G#	64.8	536	7.56E-152	K00975	64.8	536	2.02E-151
2797	Prokka:CIODPCDJ_04016	-	hypothetical protein	1	0	1	1	1	1	1	1	1	1	1	10	Accessory	COG0739_M#	27.6	43	0.00262				
2798	WP_122214780.1	-	alpha/beta hydrolase	0	0	0	0	0	0	0	1	0	1	0	2	Accessory	COG0596_R#	37.3	191	7.79E-48	NA	32.9	168	1.89E-40
2799	OSC65005.1	-	cytochrome	0	1	0	0	0	0	0	0	0	0	0	1	Unique	COG2124_Q#	35.1	223	1.08E-57	K16593	33.4	198	1.7E-49
2800	Prokka:JAPCLKMA_00263	-	hypothetical protein	1	1	1	1	1	1	1	1	1	1	1	11	CORE	COG0719_O#	60.1	453	1.1E-126	K09015	60.1	453	2.93E-126
2801	WP_102638065.1	-	sensor histidine kinase	0	1	0	0	0	0	0	0	1	0	0	2	Accessory	COG4585_T#	34.9	221	5.38E-57	K02480	38.7	127	3.69E-28
2802	OSC73213.1	-	toxic anion resistance protein	0	1	0	0	0	0	0	0	0	0	0	1	Unique	COG3853_P#	43.7	294	6.53E-79	NA	43.7	294	1.74E-78
2803	WP_122213575.1	-	queuine/other tRNA-ribosyltransferase	0	0	0	0	0	0	0	0	0	1	0	1	Unique								
2804	Prokka:CIODPCDJ_05125	-	hypothetical protein	1	0	1	1	1	1	1	1	1	1	1	10	Accessory	COG2327_S#	35.8	220	9.18E-57				

cluster	GI	gene	Annotation	Streptomyces_thermocarboxydus_BPSAC147	Streptomyces_sp._4F	Streptomyces_sp._BSE7F	Streptomyces_sp._ETH9427	Streptomyces_sp._FxanaD5	Streptomyces_sp._UNC401CLCol	Actinospica_acidiphila_NRRL_B_24431	Streptomyces_sp._HNS054	Streptomyces_sp._SMS_SU21	Streptomyces_sp._ZS0098	Streptomyces_thermocarboxydus_K155	Addition	Core-Accessory-Unique	COG	%Similarity_COG	Score_COG	Evalue_COG	KEGG	%Similarity_KEGG	Score_KEGG	Evalue_KEGG
2805	AXI86481.1	-	hypothetical protein	1	1	1	1	1	1	1	1	1	1	1	11	CORE								
2806	OSC66209.1	-	FAD-binding monooxygenase	0	1	0	0	0	0	0	0	0	0	0	1	Unique	COG0654_HC#	42.5	303	1.41E-81	NA	25.9	61	1.9E-08
2807	WP_102638059.1	-	hypothetical protein	0	0	0	0	0	0	0	0	1	0	0	1	Unique	COG0732_V#	26.3	111	6.01E-24				
2808	WP_033274859.1	-	acyltransferase	0	0	0	0	0	0	1	0	0	0	0	1	Unique								
2809	OSC72561.1	-	asparagine synthase	0	1	0	0	0	0	0	0	0	0	0	1	Unique	COG0367_E#	34.6	42	0.00584				
2810	PWE09930.1	-	acetyl-CoA C-acyltransferase	1	1	1	1	1	1	1	1	1	1	1	11	CORE	COG0183_I#	42.5	302	3.13E-81	K02615	42.5	302	8.35E-81
2811	AXI87146.1	-	secretion protein	1	1	1	1	1	1	1	1	1	1	1	11	CORE	COG4962_U#	59.9	386	1.26E-106	K02283	57.9	401	1.01E-110
2812	AXI85002.1	-	serine hydrolase	1	1	1	1	1	1	1	1	1	1	1	11	CORE	COG1680_V#	32.9	168	7.04E-41	K01286	27.3	112	7.18E-24
2813	Prokka:JAPCLKMA_00282	-	hypothetical protein	1	0	0	0	1	1	0	0	0	0	1	4	Accessory	COG3552_R#	31.7	182	2.76E-45				
2814	PWE10259.1	-	Tat (twin-arginine translocation) pathway signal sequence	1	1	1	1	1	1	1	1	1	1	1	11	CORE								
2815	Prokka:CIODPCDJ_05209	-	hypothetical protein	0	0	0	0	0	0	0	0	0	0	1	1	Unique								
2816	Prokka:JAPCLKMA_06675	mdoC	Glucans biosynthesis protein C	1	0	0	0	1	1	1	0	0	0	1	5	Accessory								
2817	PWE08686.1	-	threonine aldolase	1	1	1	1	1	1	1	1	1	1	1	11	CORE	COG2008_E#	37	224	6.34E-58	K01620	28.8	99	1.07E-19

cluster	GI	gene	Annotation	Streptomyces_thermocarboxydus_BPSAC147	Streptomyces_sp._4F	Streptomyces_sp._BSE7F	Streptomyces_sp._ETH9427	Streptomyces_sp._FxanaD5	Streptomyces_sp._UNC401CLCol	Actinospica_acidiphila_NRRL_B_24431	Streptomyces_sp._HNS054	Streptomyces_sp._SMS_SU21	Streptomyces_sp._ZS0098	Streptomyces_thermocarboxydus_K155	Addition	Core-Accessory-Unique	COG	%Similarity_COG	Score_COG	Evalue_COG	KEGG	%Similarity_KEGG	Score_KEGG	Evalue_KEGG
2818	Prokka:JAPCLKMA_04911	-	hypothetical protein	1	0	0	0	0	0	0	0	0	0	0	1	Unique								
2819	WP_019524453.1	-	enoyl-CoA hydratase/isomerase family protein	0	0	0	0	1	1	1	0	1	0	0	4	Accessory								
2820	WP_028959968.1	-	PLP-dependent aminotransferase family protein	1	1	1	1	1	1	1	1	1	1	1	11	CORE	COG1167_KE#	46.9	334	7.42E-91	K05825	44.7	319	3.87E-86
2821	WP_102639550.1	-	hypothetical protein	1	1	1	1	1	1	1	1	1	1	1	11	CORE					NA	26.4	46	0.000825
2822	WP_033278194.1	-	IS110 family transposase	1	0	1	0	1	0	1	0	0	0	0	4	Accessory	COG3547_L#	41.7	301	5.33E-81				
2823	PWE07525.1	-	IS30 family transposase	0	0	1	0	0	0	0	1	0	0	0	2	Accessory	COG2826_L#	49.8	309	1.96E-83	K07482	49.8	309	5.23E-83
2824	Prokka:JAPCLKMA_05024	coaBC_1	Coenzyme A biosynthesis bifunctional protein CoaBC	1	1	1	1	1	1	1	1	1	1	1	11	CORE	COG0452_H#	61.9	460	8.97E-129	K13038	61.9	460	2.39E-128
2825	Prokka:CIODPCDJ_03592	ydiO	putative BsuMI modification methylase subunit YdiO	0	0	0	0	0	0	0	0	0	0	1	1	Unique	COG0270_L#	40.7	287	1.04E-76	K00558	40.7	287	2.78E-76

cluster	GI	gene	Annotation	Streptomyces_thermocarboxydus_BPSAC147	Streptomyces_sp._4F	Streptomyces_sp._BSE7F	Streptomyces_sp._ETH9427	Streptomyces_sp._FxanaD5	Streptomyces_sp._UNC401CLCol	Actinospica_acidiphila_NRRL_B_24431	Streptomyces_sp._HNS054	Streptomyces_sp._SMS_SU21	Streptomyces_sp._ZS0098	Streptomyces_thermocarboxydus_K155	Addition	Core-Accessory-Unique	COG	%Similarity_COG	Score_COG	Evalue_COG	KEGG	%Similarity_KEGG	Score_KEGG	Evalue_KEGG
2826	WP_033277658.1	-	Gfo/Idh/MocA family oxidoreductase	1	1	1	1	1	1	1	1	1	1	1	11	CORE	COG0673_R#	36.2	171	4.88E-42	NA	26.9	73	6.31E-12
2827	OSC71056.1	-	SAM-dependent methyltransferase	0	1	0	0	0	0	0	0	0	0	0	1	Unique	COG0500_QR#	38.6	276	1.84E-73	NA	34.3	242	1.03E-62
2828	WP_122215139.1	-	helix-turn-helix transcriptional regulator	0	0	0	0	0	0	0	0	0	1	0	1	Unique								
2829	Prokka:CIODPCDJ_06099	-	hypothetical protein	0	0	0	0	1	1	1	0	0	0	1	4	Accessory	COG2814_G#	29.4	92	3.76E-18	K18326	26.9	53	0.00000517
2830	WP_033276734.1	-	acyl-CoA dehydrogenase	1	1	1	1	1	1	1	1	1	1	1	11	CORE	COG1960_I#	59.9	480	6.4E-135	K00249	56.5	437	1.66E-121
2831	AXI85816.1	-	argininosuccinate synthase	1	0	1	1	1	1	1	1	1	1	1	10	Accessory	COG0137_E#	69.5	586	8.28E-167	K01940	69.5	586	2.21E-166
2832	WP_048457755.1	-	helix-turn-helix transcriptional regulator	0	0	1	0	0	0	0	1	1	0	0	3	Accessory								
2833	WP_020699971.1	-	NYN domain-containing protein	1	1	1	1	1	1	1	1	1	1	1	11	CORE	COG1432_S#	36.5	77	1.63E-13				
2834	WP_102640755.1	-	acyl-CoA dehydrogenase	1	1	1	1	1	1	1	1	1	1	1	11	CORE	COG1960_I#	44.9	320	8.47E-87	NA	44.9	320	2.26E-86

cluster	GI	gene	Annotation	Streptomyces_thermocarboxydus_BPSAC147	Streptomyces_sp._4F	Streptomyces_sp._BSE7F	Streptomyces_sp._ETH9427	Streptomyces_sp._FxanaD5	Streptomyces_sp._UNC401CLCol	Actinospica_acidiphila_NRRL_B_24431	Streptomyces_sp._HNS054	Streptomyces_sp._SMS_SU21	Streptomyces_sp._ZS0098	Streptomyces_thermocarboxydus_K155	Addition	Core-Accessory-Unique	COG	%Similarity_COG	Score_COG	Evalue_COG	KEGG	%Similarity_KEGG	Score_KEGG	Evalue_KEGG
2835	WP_048457436.1	-	serine/threonine-protein phosphatase	0	0	0	1	0	0	0	1	0	0	0	2	Accessory	COG2208_TK#	32.9	86	2.06E-16	K07315	20.2	56	7.96E-07
2836	WP_048460183.1	-	acetyl-CoA C-acetyltransferase	1	1	1	1	1	1	1	1	1	1	1	11	CORE	COG0183_I#	69.8	570	4.7E-162	K00626	69.6	568	6.23E-161
2837	WP_019523428.1	-	PucR family transcriptional regulator	0	0	0	0	1	1	0	0	0	0	1	3	Accessory	COG2508_TQ#	32.3	56	2.98E-07	NA	32.3	56	7.96E-07
2838	WP_028960102.1	-	glycosyltransferase family 1 protein	0	0	0	0	1	1	0	0	0	0	0	2	Accessory	COG1819_GC#	30.4	143	2.42E-33	K05841	31.2	186	6.66E-46
2839	WP_122215936.1	-	DNA adenine methylase	0	0	0	0	0	0	0	0	0	1	0	1	Unique								
2840	WP_028958948.1	-	hypothetical protein	0	0	0	0	1	1	0	0	0	0	1	3	Accessory								
2841	AXI91304.1	-	hypothetical protein	0	0	0	1	0	0	0	0	0	0	0	1	Unique								
2842	OSC61308.1	-	glycosyl hydrolase	1	1	0	1	1	1	1	1	1	1	1	10	Accessory								
2843	WP_122213817.1	-	phage portal protein	0	0	0	0	0	0	0	0	0	1	0	1	Unique								
2844	WP_048458596.1	-	IS110 family transposase	0	0	0	1	0	0	0	1	0	0	0	2	Accessory	COG3547_L#	33.9	136	2.96E-31	K07486	33.9	136	7.9E-31
2845	OSC58916.1	-	ROK family protein	1	1	1	1	1	1	1	1	1	1	1	11	CORE	COG1940_KG#	33.2	191	5.93E-48	NA	32.6	181	1.26E-44
2846	OSC71163.1	-	cytochrome	0	1	0	0	0	0	0	0	0	0	0	1	Unique	COG2124_Q#	38	202	2.57E-51	K15468	34.4	217	2.7E-55

cluster	GI	gene	Annotation	Streptomyces_thermocarboxydus_BPSAC147	Streptomyces_sp._4F	Streptomyces_sp._BSE7F	Streptomyces_sp._ETH9427	Streptomyces_sp._FxanaD5	Streptomyces_sp._UNC401CLCol	Actinospica_acidiphila_NRRL_B_24431	Streptomyces_sp._HNS054	Streptomyces_sp._SMS_SU21	Streptomyces_sp._ZS0098	Streptomyces_thermocarboxydus_K155	Addition	Core-Accessory-Unique	COG	%Similarity_COG	Score_COG	Evalue_COG	KEGG	%Similarity_KEGG	Score_KEGG	Evalue_KEGG
2847	OSC73266.1	-	3-oxoacyl-ACP synthase	0	1	0	0	0	0	0	0	0	0	0	1	Unique	COG0304_IQ#	40	297	1E-79	K09458	40	297	2.68E-79
2848	Prokka:JAPCLKMA_00614	bkdA_2	3-methyl-2-oxobutanoate dehydrogenase subunit alpha	1	1	1	1	1	1	1	1	1	1	1	11	CORE	COG1071_C#	45.1	283	8.79E-76	K00161	45.1	283	2.35E-75
2849	OSC73121.1	-	hypothetical protein	0	1	0	0	0	0	0	0	0	0	0	1	Unique								
2850	WP_028959892.1	-	SpoIIE family protein phosphatase	0	0	1	0	0	1	0	0	0	0	0	2	Accessory	COG2208_TK#	32.6	111	7.8E-24	NA	30.5	134	3E-30
2851	WP_106963358.1	-	DMT family transporter	1	0	1	1	1	1	1	0	1	1	1	9	Accessory					NA	21.2	62	1.11E-08
2852	Prokka:JAPCLKMA_03265	cbiET_2	Cobalamin biosynthesis bifunctional protein CbiET	1	1	1	1	1	1	1	1	1	1	1	11	CORE	COG2241_H#	34.1	81	6.61E-15	K00595	30.8	72	1.07E-11
2853	WP_102642035.1	-	pyridoxal phosphate-dependent aminotransferase	1	1	1	1	1	1	1	1	1	1	1	11	CORE	COG0436_E#	65.5	555	1.56E-157	K14260	62.1	537	8.99E-152

cluster	GI	gene	Annotation	Streptomyces_thermocarboxydus_BPSAC147	Streptomyces_sp._4F	Streptomyces_sp._BSE7F	Streptomyces_sp._ETH9427	Streptomyces_sp._FxanaD5	Streptomyces_sp._UNC401CLCol	Actinospica_acidiphila_NRRL_B_24431	Streptomyces_sp._HNS054	Streptomyces_sp._SMS_SU21	Streptomyces_sp._ZS0098	Streptomyces_thermocarboxydus_K155	Addition	Core-Accessory-Unique	COG	%Similarity_COG	Score_COG	Evalue_COG	KEGG	%Similarity_KEGG	Score_KEGG	Evalue_KEGG
2854	WP_019526416.1	-	LLM class flavin-dependent oxidoreductase	1	1	0	0	1	1	1	0	0	0	1	6	Accessory	COG2141_C#	31.5	160	1.46E-38	NA	31.5	160	3.9E-38
2855	WP_102638023.1	-	sensor histidine kinase	1	0	1	1	1	1	0	1	1	1	1	9	Accessory	COG4585_T#	37.8	156	2.11E-37	K07778	37.8	156	5.64E-37
2856	WP_033274056.1	-	ROK family transcriptional regulator	1	1	1	1	1	1	1	1	1	1	1	11	CORE	COG1940_KG#	31	206	2.32E-52	NA	28.7	175	1.17E-42
2857	AXI91102.1	-	helicase DnaB	0	0	0	1	0	0	0	0	0	0	0	1	Unique	COG0305_L#	29.6	44	0.00117	NA	33	49	0.000127
2858	AXI89654.1	-	serine/threonine protein kinase	0	0	0	1	0	0	0	0	0	0	0	1	Unique	COG0515_RTKL#	30.7	138	4.55E-32	K08884	29.9	133	5.11E-30
2859	WP_033273129.1	-	phosphoglycerate kinase	1	1	1	1	1	1	1	1	1	1	1	11	CORE	COG0126_G#	55.3	415	1.93E-115	K00927	55.3	415	5.15E-115
2860	Prokka:CIODPCDJ_03593	-	ISAs1 family transposase ISBli27	0	0	0	0	1	0	0	0	0	0	1	2	Accessory	COG5433_L#	25.8	94	1.29E-18				
2861	Prokka:JAPCLKMA_00924	metXA	Homoserine O-acetyltransferase	1	0	0	0	1	1	1	0	0	0	1	5	Accessory	COG2021_E#	59.1	419	1.75E-116	K00641	59.1	419	4.66E-116
2862	WP_048458440.1	-	stress-induced protein	1	1	1	1	1	1	1	1	1	1	1	11	CORE	COG2310_T#	48.8	185	4.24E-46	NA	38.8	234	1.63E-60
2863	OSC57751.1	-	hypothetical protein	0	1	1	1	0	0	1	1	1	1	0	7	Accessory								

cluster	GI	gene	Annotation	Streptomyces_thermocarboxydus_BPSAC147	Streptomyces_sp._4F	Streptomyces_sp._BSE7F	Streptomyces_sp._ETH9427	Streptomyces_sp._FxanaD5	Streptomyces_sp._UNC401CLCol	Actinospica_acidiphila_NRRL_B_24431	Streptomyces_sp._HNS054	Streptomyces_sp._SMS_SU21	Streptomyces_sp._ZS0098	Streptomyces_thermocarboxydus_K155	Addition	Core-Accessory-Unique	COG	%Similarity_COG	Score_COG	Evalue_COG	KEGG	%Similarity_KEGG	Score_KEGG	Evalue_KEGG
2864	OSC70453.1	-	glucose sorbosone dehydrogenase	1	1	1	1	1	1	1	1	1	1	1	11	CORE	COG2133_G#	37.5	231	3.95E-60	NA	28.9	127	3.66E-28
2865	OSC55751.1	-	adenylosuccinate lyase	0	1	0	0	0	0	0	0	0	0	0	1	Unique	COG0015_F#	45	325	3.43E-88	K01756	45	325	9.16E-88
2866	OSC73271.1	-	hypothetical protein	0	1	0	0	0	0	0	0	0	0	0	1	Unique	COG1960_I#	31.5	180	1.36E-44	K00248	30.3	166	4.17E-40
2867	OSC67218.1	-	ABC transporter permease	0	1	0	0	0	0	0	0	0	0	0	1	Unique	COG1173_EP#	38.7	156	2.11E-37	K12370	35.7	133	3.91E-30
2868	Prokka:JAPCLKMA_02369	-	hypothetical protein	1	0	1	1	1	1	1	1	1	1	1	10	Accessory								
2869	WP_019525139.1	-	hypothetical protein	1	1	1	1	1	1	1	1	1	1	1	11	CORE	COG0330_O#	35	125	5.21E-28	NA	25.8	89	1.11E-16
2870	WP_051724382.1	-	hypothetical protein	0	0	0	0	0	0	1	0	0	0	0	1	Unique								
2871	OSC66549.1	-	MFS transporter	1	1	1	1	1	1	1	1	1	1	1	11	CORE	COG2814_G#	52.8	389	1.13E-107	K08156	47.9	334	1.97E-90
2872	Prokka:CIODPCDJ_02220	-	hypothetical protein	1	1	1	1	1	1	1	1	1	1	1	11	CORE	COG3594_G#	33.5	177	8.85E-44	NA	30.5	167	3.19E-40
2873	OSC70487.1	-	MFS transporter	1	1	1	1	1	1	1	1	1	1	1	11	CORE	COG0477_GEPR#	37.4	227	9.74E-59	NA	38.3	210	2.52E-53
2874	WP_048460895.1	-	methionine adenosyltransferase	1	1	1	1	1	1	1	1	1	1	1	11	CORE	COG0192_H#	73.2	572	9.42E-163	K00789	73.9	570	1.25E-161
2875	WP_033278025.1	-	hypothetical protein	1	0	0	0	1	1	1	0	0	0	1	5	Accessory								
2876	Prokka:JAPCLKMA_03228	-	hypothetical protein	1	1	0	0	1	1	0	0	0	0	1	5	Accessory	COG2208_TK#	24.2	81	6.59E-15				

cluster	GI	gene	Annotation	Streptomyces_thermocarboxydus_BPSAC147	Streptomyces_sp._4F	Streptomyces_sp._BSE7F	Streptomyces_sp._ETH9427	Streptomyces_sp._FxanaD5	Streptomyces_sp._UNC401CLCol	Actinospica_acidiphila_NRRL_B_24431	Streptomyces_sp._HNS054	Streptomyces_sp._SMS_SU21	Streptomyces_sp._ZS0098	Streptomyces_thermocarboxydus_K155	Addition	Core-Accessory-Unique	COG	%Similarity_COG	Score_COG	Evalue_COG	KEGG	%Similarity_KEGG	Score_KEGG	Evalue_KEGG
2877	WP_033272189.1	-	exodeoxyribonuclease VII large subunit	1	1	1	1	1	1	1	1	1	1	1	11	CORE	COG1570_L#	53.3	395	2.06E-109	K03601	53.3	395	5.51E-109
2878	WP_048457987.1	-	nucleotide sugar dehydrogenase	1	1	1	1	1	1	1	1	1	1	1	11	CORE	COG0677_M#	38.5	258	3.01E-68	NA	38.9	241	1.33E-62
2879	PWE10674.1	-	hypothetical protein	1	1	1	1	1	1	1	1	1	1	1	11	CORE								
2880	OSC61235.1	-	bifunctional D-altronate/D-mannonate dehydratase	0	1	0	0	0	0	0	0	0	0	0	1	Unique	COG4948_MR#	66	559	1.41E-158	K08323	66	559	3.76E-158
2881	Prokka:CIODPCDJ_02228	-	Transcriptional regulator	1	0	0	0	1	1	1	0	0	0	1	5	Accessory	COG1940_KG#	35.1	188	3.83E-47	NA	35.1	188	1.02E-46
2882	WP_028961096.1	-	ABC transporter ATP-binding protein	1	1	1	1	1	1	1	1	1	1	1	11	CORE	COG1131_V#	56.5	321	3.78E-87	K01990	36.4	179	6.2E-44
2883	Prokka:CIODPCDJ_02615	tagU_1	Polyisoprenyl-teichoic acid--peptidoglycan teichoic acid transferase TagU	1	1	1	1	1	1	1	1	1	1	1	11	CORE	COG1316_K#	33.8	158	4.24E-38	NA	33.8	158	1.13E-37
2884	OSC58911.1	-	chemotaxis protein	0	1	0	0	0	0	0	0	0	0	0	1	Unique	COG2814_G#	40.6	299	1.54E-80	K08156	36.8	245	9.2E-64

cluster	GI	gene	Annotation	Streptomyces_thermocarboxydus_BPSAC147	Streptomyces_sp._4F	Streptomyces_sp._BSE7F	Streptomyces_sp._ETH9427	Streptomyces_sp._FxanaD5	Streptomyces_sp._UNC401CLCol	Actinospica_acidiphila_NRRL_B_24431	Streptomyces_sp._HNS054	Streptomyces_sp._SMS_SU21	Streptomyces_sp._ZS0098	Streptomyces_thermocarboxydus_K155	Addition	Core-Accessory-Unique	COG	%Similarity_COG	Score_COG	Evalue_COG	KEGG	%Similarity_KEGG	Score_KEGG	Evalue_KEGG
2885	PWE11009.1	-	acyltransferase	0	0	1	0	0	0	0	0	1	0	0	2	Accessory	COG1835_I#	31.7	190	1.32E-47	NA	28.8	121	1.53E-26
2886	OSC70960.1	-	protein in whiE locus	1	1	1	1	1	1	1	1	1	1	1	11	CORE								
2887	AXI87135.1	-	DNA polymerase III subunit delta'	1	1	1	1	1	1	1	1	1	1	1	11	CORE	COG0470_L#	47.4	326	1.53E-88	K02341	47.4	326	4.09E-88
2888	WP_086704495.1	-	fatty acid biosynthesis transcriptional regulator FasR	1	1	1	1	1	1	1	1	1	1	1	11	CORE	COG2508_TQ#	43.2	308	3.31E-83	NA	43.2	308	8.83E-83
2889	WP_102637426.1	-	erythromycin esterase family protein	0	1	1	1	1	1	1	1	1	1	1	10	Accessory	COG2312_R#	44.5	285	3.91E-76	NA	44.5	285	1.05E-75
2890	PWE10257.1	-	xylose isomerase	1	1	1	1	1	1	1	1	1	1	1	11	CORE								
2891	WP_019527342.1	-	sugar hydrolase	1	1	1	1	1	1	1	1	1	1	1	11	CORE								
2892	WP_019524729.1	-	signal transduction histidine kinase	1	1	1	1	1	1	1	1	1	1	1	11	CORE	COG3275_T#	33	181	7.96E-45	K02478	33	181	2.13E-44
2893	OSC70000.1	-	proteinase inhibitor I4 serpin	0	1	0	0	0	0	0	0	0	0	0	1	Unique								
2894	WP_019526920.1	-	glycosyltransferase	1	1	1	1	1	1	1	1	1	1	0	10	Accessory	COG0463_M#	47.4	347	6.42E-95	NA	47.7	342	5.52E-93

cluster	GI	gene	Annotation	Streptomyces_thermocarboxydus_BPSAC147	Streptomyces_sp._4F	Streptomyces_sp._BSE7F	Streptomyces_sp._ETH9427	Streptomyces_sp._FxanaD5	Streptomyces_sp._UNC401CLCol	Actinospica_acidiphila_NRRL_B_24431	Streptomyces_sp._HNS054	Streptomyces_sp._SMS_SU21	Streptomyces_sp._ZS0098	Streptomyces_thermocarboxydus_K155	Addition	Core-Accessory-Unique	COG	%Similarity_COG	Score_COG	Evalue_COG	KEGG	%Similarity_KEGG	Score_KEGG	Evalue_KEGG
2895	OSC70077.1	-	two-component sensor histidine kinase	1	1	1	1	1	1	1	1	1	1	1	11	CORE	COG4585_T#	26.6	107	1.12E-22	NA	26.6	107	2.99E-22
2896	Prokka:JAPCLKMA_06840	-	hypothetical protein	1	0	0	0	0	0	0	0	0	0	0	1	Unique								
2897	OSC63419.1	-	hypothetical protein	1	1	1	1	1	1	1	1	1	1	1	11	CORE					NA	27.8	139	7.09E-32
2898	Prokka:CIODPCDJ_02752	ndbB	Demethylphylloquinone reductase NdbB	0	0	0	0	1	0	0	0	0	0	1	2	Accessory	COG1252_C#	33.2	153	1.78E-36	K03885	33.2	153	4.75E-36
2899	WP_122215012.1	-	amino acid deaminase/aldolase	1	1	1	1	1	1	1	1	1	1	1	11	CORE	COG3616_E#	54.8	407	5.22E-113	NA	54.8	407	1.39E-112
2900	WP_019525797.1	-	family 1 glycosylhydrolase	1	1	0	0	1	1	1	0	0	1	1	7	Accessory	COG2723_G#	30	181	7.94E-45	NA	29.6	169	8.34E-41
2901	WP_019524281.1	kynU	kynureninase	1	0	1	1	1	1	1	1	1	1	1	10	Accessory	COG3844_E#	48.2	366	1.33E-100	K01556	50	361	8.77E-99
2902	WP_048458829.1	-	myo-inositol-1-phosphate synthase	1	1	0	1	1	1	1	1	1	1	1	10	Accessory	COG1260_I#	43.1	169	3.12E-41	K01858	31	148	1.17E-34
2903	WP_122216646.1	-	lactonase family protein	1	0	1	1	1	1	1	1	1	1	1	10	Accessory	COG2706_G#	32.1	190	1.31E-47	K07404	32.1	190	3.5E-47
2904	AXI84992.1	-	oleandomycin glycosyltransferase	0	0	0	1	0	0	1	1	1	1	0	5	Accessory	COG1819_GC#	30.7	186	2.47E-46	NA	30.7	186	6.59E-46
2905	WP_122213586.1	-	hypothetical protein	1	1	1	1	1	1	1	1	0	1	1	10	Accessory	COG0018_J#	36	90	1.41E-17	K01887	34.6	88	1.87E-16
2906	WP_048459736.1	-	hypothetical protein	0	0	1	1	0	0	1	1	1	1	0	6	Accessory								

cluster	GI	gene	Annotation	Streptomyces_thermocarboxydus_BPSAC147	Streptomyces_sp._4F	Streptomyces_sp._BSE7F	Streptomyces_sp._ETH9427	Streptomyces_sp._FxanaD5	Streptomyces_sp._UNC401CLCol	Actinospica_acidiphila_NRRL_B_24431	Streptomyces_sp._HNS054	Streptomyces_sp._SMS_SU21	Streptomyces_sp._ZS0098	Streptomyces_thermocarboxydus_K155	Addition	Core-Accessory-Unique	COG	%Similarity_COG	Score_COG	Evalue_COG	KEGG	%Similarity_KEGG	Score_KEGG	Evalue_KEGG
2907	WP_033275905.1	ftsY	signal recognition particle-docking protein FtsY	1	0	1	1	1	1	1	1	1	1	1	10	Accessory	COG0552_U#	60.1	448	2.04E-125	K03110	60.1	448	5.45E-125
2908	WP_028961586.1	mgt	macrolide-inactivating glycosyltransferase	1	1	1	1	1	1	1	1	1	1	1	11	CORE	COG1819_GC#	33.2	226	1.26E-58	NA	33.2	226	3.37E-58
2909	WP_106963209.1	-	IS3 family transposase	0	0	0	0	0	0	1	1	0	0	0	2	Accessory	COG2801_L#	51.7	294	6.44E-79	NA	34.2	167	3.17E-40
2910	OSC55334.1	-	monooxygenase	1	1	1	1	1	1	1	1	1	1	1	11	CORE	COG2072_P#	40.6	249	2.37E-65	K11816	33.7	195	8.32E-49
2911	WP_019523833.1	-	hypothetical protein	0	1	0	1	1	1	1	1	1	1	1	9	Accessory								
2912	Prokka:JAPCLKMA_02926	nanK_2	N-acetylmannosamine kinase	1	0	1	1	1	1	1	1	1	1	1	10	Accessory	COG1940_KG#	33.8	198	6.28E-50	K02565	30	156	4.28E-37
2913	WP_033277702.1	-	oxidoreductase	0	0	0	0	0	0	1	0	0	0	0	1	Unique	COG0446_R#	43.8	296	1.69E-79	NA	39	245	1.2E-63
2914	OSC60330.1	-	alpha/beta hydrolase	0	1	0	0	0	0	0	0	0	0	0	1	Unique	COG4188_R#	34.8	130	1.61E-29				
2915	Prokka:JAPCLKMA_01809	-	hypothetical protein	1	0	0	0	0	1	1	0	0	0	1	4	Accessory								
2916	Prokka:CIODPCDJ_00791	btuD_2	Vitamin B12 import ATP-binding protein BtuD	1	1	1	1	1	1	1	1	1	1	1	11	CORE	COG1136_V#	60.5	315	2.7E-85	K02003	43.9	203	5.21E-51

cluster	GI	gene	Annotation	Streptomyces_thermocarboxydus_BPSAC147	Streptomyces_sp._4F	Streptomyces_sp._BSE7F	Streptomyces_sp._ETH9427	Streptomyces_sp._FxanaD5	Streptomyces_sp._UNC401CLCol	Actinospica_acidiphila_NRRL_B_24431	Streptomyces_sp._HNS054	Streptomyces_sp._SMS_SU21	Streptomyces_sp._ZS0098	Streptomyces_thermocarboxydus_K155	Addition	Core-Accessory-Unique	COG	%Similarity_COG	Score_COG	Evalue_COG	KEGG	%Similarity_KEGG	Score_KEGG	Evalue_KEGG
2917	WP_078493188.1	-	HAMP domain-containing histidine kinase	0	0	0	0	0	0	1	0	0	0	0	1	Unique	COG0642_T#	34.2	126	3.03E-28	K02484	34.2	126	8.1E-28
2918	Prokka:JAPCLKMA_04919	-	hypothetical protein	1	0	0	0	0	0	0	0	0	0	0	1	Unique								
2919	OSC61398.1	-	oxidoreductase	0	1	0	0	0	0	0	0	0	0	0	1	Unique	COG0673_R#	31.1	83	1.73E-15	NA	21.7	79	8.69E-14
2920	WP_028961031.1	-	AI-2E family transporter	1	1	1	0	1	1	1	1	1	1	0	9	Accessory	COG0628_R#	46.9	302	3.09E-81	NA	33.6	201	1.52E-50
2921	Prokka:JAPCLKMA_01207	-	hypothetical protein	1	1	1	1	1	1	1	1	1	1	1	11	CORE	COG1295_S#	34.4	190	1.31E-47	K07058	34.4	190	3.5E-47
2922	OSC69177.1	-	sensor histidine kinase	1	1	1	1	1	1	1	1	1	1	1	11	CORE	COG4585_T#	36.6	122	2.57E-27	NA	32.2	126	8.1E-28
2923	AXI85351.1	-	hypothetical protein	1	0	0	1	0	0	0	0	0	0	0	2	Accessory								
2924	Prokka:CIODPCDJ_03472	-	hypothetical protein	1	0	0	0	0	0	0	0	0	0	1	2	Accessory	COG1396_K#	42.3	44	0.00152				
2925	Prokka:CIODPCDJ_06429	-	hypothetical protein	0	0	0	0	0	0	0	0	0	0	1	1	Unique								
2926	OSC59411.1	-	monooxygenase	0	1	0	0	0	0	0	0	0	0	0	1	Unique	COG0654_HC#	30.3	127	7.98E-29	NA	29.6	114	1.87E-24
2927	WP_084206980.1	-	hypothetical protein	0	0	0	0	0	0	1	0	0	0	0	1	Unique	COG4585_T#	27.1	70	1.16E-11	NA	23.2	57	3.54E-07
2928	PWE08416.1	-	hypothetical protein	0	0	1	0	0	0	0	0	1	0	0	2	Accessory								

cluster	GI	gene	Annotation	Streptomyces_thermocarboxydus_BPSAC147	Streptomyces_sp._4F	Streptomyces_sp._BSE7F	Streptomyces_sp._ETH9427	Streptomyces_sp._FxanaD5	Streptomyces_sp._UNC401CLCol	Actinospica_acidiphila_NRRL_B_24431	Streptomyces_sp._HNS054	Streptomyces_sp._SMS_SU21	Streptomyces_sp._ZS0098	Streptomyces_thermocarboxydus_K155	Addition	Core-Accessory-Unique	COG	%Similarity_COG	Score_COG	Evalue_COG	KEGG	%Similarity_KEGG	Score_KEGG	Evalue_KEGG
2929	WP_028958979.1	-	aminotransferase class I/II-fold pyridoxal phosphate-dependent enzyme	1	1	1	1	1	1	1	1	1	1	1	11	CORE	COG1168_E#	60.6	475	1.56E-133	K14155	60.6	475	4.16E-133
2930	WP_048460850.1	-	TylR regulatory protein	0	0	0	1	0	0	0	1	0	0	0	2	Accessory	COG5659_L#	26.7	102	2.74E-21				
2931	WP_019526205.1	-	hypothetical protein	1	1	0	0	1	1	0	0	0	0	1	5	Accessory								
2932	WP_019527072.1	-	MarP family serine protease	1	1	1	1	1	1	1	1	1	1	1	11	CORE	COG0265_O#	37.7	246	2.01E-64	NA	38.5	243	4.53E-63
2933	WP_028960391.1	-	hypothetical protein	0	1	0	0	1	1	0	0	0	0	1	4	Accessory								
2934	WP_019526015.1	-	AAA domain-containing protein	1	1	1	0	1	1	1	0	1	1	1	9	Accessory	COG0714_R#	35.1	191	5.86E-48	K03924	31.7	188	1.33E-46
2935	WP_028960038.1	mqnC	dehypoxanthine futalosine cyclase	1	1	1	1	1	1	1	1	1	1	1	11	CORE	COG1060_HR#	44.4	319	2.43E-86	K11784	43.9	304	1.66E-81
2936	WP_102641878.1	-	sensor histidine kinase	0	1	0	0	0	0	1	0	1	1	0	4	Accessory	COG4585_T#	30.4	116	3.13E-25	NA	30.4	116	8.36E-25
2937	WP_102639352.1	-	glycine C-acetyltransferase	1	1	1	1	1	1	1	1	1	1	1	11	CORE	COG0156_H#	67.9	510	4.36E-144	K00639	63.4	488	4.74E-137
2938	Prokka:CIODPCDJ_00633	ybjJ_1	Inner membrane protein YbjJ	1	1	1	0	1	1	1	1	1	1	1	10	Accessory	COG0477_GEPR#	39.2	236	1.59E-61	NA	39.2	236	4.24E-61

cluster	GI	gene	Annotation	Streptomyces_thermocarboxydus_BPSAC147	Streptomyces_sp._4F	Streptomyces_sp._BSE7F	Streptomyces_sp._ETH9427	Streptomyces_sp._FxanaD5	Streptomyces_sp._UNC401CLCol	Actinospica_acidiphila_NRRL_B_24431	Streptomyces_sp._HNS054	Streptomyces_sp._SMS_SU21	Streptomyces_sp._ZS0098	Streptomyces_thermocarboxydus_K155	Addition	Core-Accessory-Unique	COG	%Similarity_COG	Score_COG	Evalue_COG	KEGG	%Similarity_KEGG	Score_KEGG	Evalue_KEGG
2939	WP_019524942.1	-	methyltransferase domain-containing protein	0	1	0	0	1	0	0	0	0	0	0	2	Accessory	COG2518_O#	32	72	3.04E-12	K00573	32.2	56	7.86E-07
2940	Prokka:CIODPCDJ_00821	-	hypothetical protein	0	0	0	0	0	0	0	0	0	0	1	1	Unique	COG0419_L#	29.5	157	1.22E-37	K03546	33.1	123	4.01E-27
2941	WP_006135705.1	-	YbdK family carboxylate-amine ligase	1	0	1	1	1	1	1	1	1	1	1	10	Accessory	COG2170_S#	35.5	156	1.6E-37	K06048	28.6	130	4.28E-29
2942	OSC70396.1	-	glycosyltransferase	1	1	1	0	1	1	1	1	1	1	1	10	Accessory	COG0438_M#	29.5	88	6.99E-17	K16697	29.5	88	1.87E-16
2943	WP_033272768.1	-	hypothetical protein	0	0	0	0	0	0	1	0	0	0	0	1	Unique								
2944	AXI85301.1	-	hypothetical protein	0	0	0	1	0	0	0	0	0	0	0	1	Unique								
2945	PWE10279.1	-	hypothetical protein	0	0	1	0	0	0	0	0	0	0	0	1	Unique	COG0654_HC#	47.3	377	4.42E-104	NA	47.9	346	3.8E-94
2946	AXI85289.1	-	methyltransferase	0	0	0	1	0	0	0	0	0	0	0	1	Unique	COG1051_F#	35.1	69	4.39E-11	K03574	35.1	55	0.00000134
2947	WP_048457054.1	-	pyridoxal phosphate-dependent aminotransferase	1	1	1	1	1	1	1	1	1	1	1	11	CORE	COG0436_E#	56.5	421	2.66E-117	K14267	56.5	421	7.1E-117
2948	WP_026243414.1	-	NAD-binding protein	1	0	0	0	1	1	0	0	0	0	1	4	Accessory	COG0654_HC#	41.7	257	1.13E-67				
2949	WP_019521541.1	-	TetR/AcrR family transcriptional regulator	1	0	0	0	1	1	1	0	0	0	1	5	Accessory	COG1309_K#	28.4	136	2.23E-31	NA	28.4	136	5.96E-31
2950	WP_078895325.1	-	serine hydrolase	0	0	0	1	0	0	0	1	0	1	0	3	Accessory	COG1680_V#	37.2	166	2.63E-40	NA	29.9	116	8.34E-25

cluster	GI	gene	Annotation	Streptomyces_thermocarboxydus_BPSAC147	Streptomyces_sp._4F	Streptomyces_sp._BSE7F	Streptomyces_sp._ETH9427	Streptomyces_sp._FxanaD5	Streptomyces_sp._UNC401CLCol	Actinospica_acidiphila_NRRL_B_24431	Streptomyces_sp._HNS054	Streptomyces_sp._SMS_SU21	Streptomyces_sp._ZS0098	Streptomyces_thermocarboxydus_K155	Addition	Core-Accessory-Unique	COG	%Similarity_COG	Score_COG	Evalue_COG	KEGG	%Similarity_KEGG	Score_KEGG	Evalue_KEGG
2951	OSC61093.1	-	two-component sensor histidine kinase	0	1	0	0	0	0	0	0	0	0	0	1	Unique	COG4585_T#	29.5	97	8.8E-20	NA	29.5	97	2.35E-19
2952	Prokka:CIODPCDJ_00177	mhpA_1	3-(3-hydroxy-phenyl)propionate/3-hydroxycinnamic acid hydroxylase	1	1	1	1	1	1	1	1	0	1	1	10	Accessory	COG0644_C#	32.1	93	2.17E-18	K17830	26.1	90	4.9E-17
2953	WP_019521945.1	ftsZ	cell division protein FtsZ	1	0	1	1	1	1	1	1	1	1	1	10	Accessory	COG0206_D#	77.1	510	5.69E-144	K03531	76.7	510	1.98E-143
2954	WP_048460278.1	-	sugar kinase	1	1	1	1	1	1	1	1	1	1	1	11	CORE	COG0524_G#	28.8	111	5.89E-24	K00852	28.3	75	1.63E-12
2955	AXI90289.1	-	glycosyl hydrolase family 5	1	1	1	1	1	1	1	1	1	1	1	11	CORE					NA	53.3	171	1.67E-41
2956	OSC66608.1	-	two-component sensor histidine kinase	1	1	1	1	1	1	1	1	1	1	1	11	CORE	COG4585_T#	31.4	92	4.83E-18	NA	31.4	92	1.29E-17
2957	WP_033277569.1	-	DUF4350 domain-containing protein	1	1	1	1	1	1	1	1	1	1	1	11	CORE					NA	38.7	188	1.73E-46
2958	AXI85266.1	-	hypothetical protein	0	0	0	1	0	0	0	0	0	0	0	1	Unique	COG0582_L#	23.4	63	1.84E-09	K04763	34.1	52	0.0000148

cluster	GI	gene	Annotation	Streptomyces_thermocarboxydus_BPSAC147	Streptomyces_sp._4F	Streptomyces_sp._BSE7F	Streptomyces_sp._ETH9427	Streptomyces_sp._FxanaD5	Streptomyces_sp._UNC401CLCol	Actinospica_acidiphila_NRRL_B_24431	Streptomyces_sp._HNS054	Streptomyces_sp._SMS_SU21	Streptomyces_sp._ZS0098	Streptomyces_thermocarboxydus_K155	Addition	Core-Accessory-Unique	COG	%Similarity_COG	Score_COG	Evalue_COG	KEGG	%Similarity_KEGG	Score_KEGG	Evalue_KEGG
2959	WP_028961278.1	-	helix-turn-helix transcriptional regulator	0	0	0	1	0	1	0	0	0	0	0	2	Accessory								
2960	WP_048458566.1	sbcD	exonuclease SbcCD subunit D	1	1	1	1	1	1	1	1	1	1	1	11	CORE	COG0420_L#	37.6	223	1.81E-57	K03547	27.1	150	3.05E-35
2961	WP_019524588.1	cas7e	type I-E CRISPR-associated protein Cas7/Cse4/CasC	0	0	0	0	1	0	0	0	0	0	0	1	Unique					K19124	29.8	134	1.73E-30
2962	Prokka:JAPCLKMA_04413	rtcB	RNA-splicing ligase RtcB	1	1	1	1	1	1	1	1	1	1	1	11	CORE	COG1690_S#	52.7	390	8.55E-108	K14415	46.9	358	9.62E-98
2963	Prokka:CIODPCDJ_05047	-	hypothetical protein	1	0	1	1	1	1	0	1	0	1	1	8	Accessory								
2964	OSC65155.1	-	hypothetical protein	0	1	0	0	0	0	0	0	0	0	0	1	Unique								
2965	OSC70910.1	-	hypothetical protein	0	1	0	0	0	0	0	0	0	0	0	1	Unique								
2966	WP_033276196.1	-	imidazolonepropionase	1	1	1	1	1	1	1	1	1	1	1	11	CORE	COG1228_Q#	44.4	265	4.15E-70	K01468	37.7	231	1.04E-59
2967	WP_019525214.1	tuf	elongation factor Tu	1	1	1	1	1	1	1	1	1	1	1	11	CORE	COG0050_J#	83.9	684	2.19E-196	K02358	83.9	684	5.86E-196
2968	OSC63605.1	-	hypothetical protein	0	1	0	0	0	0	0	0	0	0	0	1	Unique	COG2340_S#	38.5	109	2.23E-23	NA	38.5	109	5.96E-23
2969	OSC69940.1	-	radical SAM protein	0	1	0	0	0	0	0	0	0	0	0	1	Unique								
2970	WP_028961407.1	-	ABC transporter substrate-binding protein	0	0	0	0	1	1	0	0	0	0	1	3	Accessory	COG0687_E#	44.8	347	8.3E-95	K02055	45.1	340	2.08E-92

cluster	GI	gene	Annotation	Streptomyces_thermocarboxydus_BPSAC147	Streptomyces_sp._4F	Streptomyces_sp._BSE7F	Streptomyces_sp._ETH9427	Streptomyces_sp._FxanaD5	Streptomyces_sp._UNC401CLCol	Actinospica_acidiphila_NRRL_B_24431	Streptomyces_sp._HNS054	Streptomyces_sp._SMS_SU21	Streptomyces_sp._ZS0098	Streptomyces_thermocarboxydus_K155	Addition	Core-Accessory-Unique	COG	%Similarity_COG	Score_COG	Evalue_COG	KEGG	%Similarity_KEGG	Score_KEGG	Evalue_KEGG
2971	OSC70488.1	-	ROK family transcriptional regulator	1	1	1	1	1	1	1	1	1	1	1	11	CORE	COG1940_KG#	28.5	106	3.22E-22	K00845	27.1	97	3.06E-19
2972	WP_078504573.1	-	NADH:flavin oxidoreductase/NADH oxidase	1	1	1	1	1	1	1	1	1	1	1	11	CORE	COG1902_C#	55.3	398	3.14E-110	NA	50	350	2.62E-95
2973	OSC63334.1	-	hypothetical protein	1	1	0	0	1	1	0	0	0	0	1	5	Accessory								
2974	OSC69014.1	-	iron transporter	0	1	0	0	0	0	0	0	0	0	0	1	Unique	COG4264_Q#	45.1	287	7.81E-77				
2975	WP_019525002.1	-	glutathione-dependent formaldehyde dehydrogenase	1	1	1	0	1	1	1	1	1	1	1	10	Accessory	COG1063_ER#	43.3	323	1.28E-87	NA	44.1	318	1.1E-85
2976	WP_033275355.1	-	CoA transferase	1	1	1	1	1	1	1	1	1	1	1	11	CORE	COG1804_C#	57	430	5.7E-120	K18703	33.7	224	2.16E-57
2977	Prokka:CIODPCDJ_05854	-	hypothetical protein	1	1	1	1	1	1	1	1	1	1	1	11	CORE	COG2220_R#	49.5	340	7.75E-93	NA	49.5	340	2.07E-92
2978	WP_019522797.1	-	acyl-CoA dehydrogenase	1	1	1	1	1	1	1	1	1	1	1	11	CORE	COG1960_I#	71.2	576	6.43E-164	K00252	71.3	575	4.99E-163
2979	WP_028960760.1	-	hypothetical protein	1	1	1	1	1	1	1	1	1	1	1	11	CORE	COG1397_O#	28.8	82	3.81E-15	NA	23.9	59	9.21E-08
2980	WP_048455047.1	-	IS110 family transposase	0	0	0	0	0	0	0	1	0	0	0	1	Unique	COG3547_L#	36.6	215	4.91E-55	NA	31	64	2.86E-09

cluster	GI	gene	Annotation	Streptomyces_thermocarboxydus_BPSAC147	Streptomyces_sp._4F	Streptomyces_sp._BSE7F	Streptomyces_sp._ETH9427	Streptomyces_sp._FxanaD5	Streptomyces_sp._UNC401CLCol	Actinospica_acidiphila_NRRL_B_24431	Streptomyces_sp._HNS054	Streptomyces_sp._SMS_SU21	Streptomyces_sp._ZS0098	Streptomyces_thermocarboxydus_K155	Addition	Core-Accessory-Unique	COG	%Similarity_COG	Score_COG	Evalue_COG	KEGG	%Similarity_KEGG	Score_KEGG	Evalue_KEGG
2981	Prokka:JAPCLKMA_04744	frc_3	Formyl-CoA:oxalate CoA-transferase	1	0	0	0	1	1	0	0	0	0	1	4	Accessory	COG1804_C#	37.5	251	6.19E-66	K18703	34.3	233	2.73E-60
2982	Prokka:CIODPCDJ_00203	ynfM_1	Inner membrane transport protein YnfM	1	0	1	1	1	1	1	1	1	1	1	10	Accessory	COG0477_GEPR#	37.9	240	8.36E-63	NA	26.5	88	1.42E-16
2983	OSC63177.1	-	beta-hexosaminidase	0	1	0	0	0	0	0	0	0	0	0	1	Unique	COG3525_G#	39.6	270	7.54E-72	K12373	38.9	288	9.34E-77
2984	PWE11362.1	-	gala protein	1	0	1	1	1	1	0	1	0	1	1	8	Accessory					NA	30.4	94	1.98E-18
2985	Prokka:JAPCLKMA_01520	-	hypothetical protein	1	0	0	1	0	0	0	0	0	0	0	2	Accessory								
2986	Prokka:CIODPCDJ_05633	glcK	Glucokinase	1	1	1	1	1	1	1	1	1	1	1	11	CORE	COG1940_KG#	35.2	192	3.41E-48	K00845	33.8	172	7.47E-42
2987	OSC72600.1	-	molecular chaperone DnaJ	1	1	1	1	1	1	1	1	1	1	1	11	CORE	COG0484_O#	53.4	413	7.21E-115	K03686	51	394	1.58E-108
2988	WP_033277298.1	-	HupE/UreJ family protein	0	0	0	0	0	0	1	0	0	0	0	1	Unique								
2989	OSC72634.1	-	polyamine ABC transporter substrate-binding protein	0	1	0	0	0	0	0	0	0	0	0	1	Unique	COG0687_E#	28.9	139	2.62E-32	K11069	25.9	116	8.29E-25
2990	Prokka:JAPCLKMA_00622	-	hypothetical protein	1	0	0	0	0	0	0	0	0	0	0	1	Unique								

cluster	GI	gene	Annotation	Streptomyces_thermocarboxydus_BPSAC147	Streptomyces_sp._4F	Streptomyces_sp._BSE7F	Streptomyces_sp._ETH9427	Streptomyces_sp._FxanaD5	Streptomyces_sp._UNC401CLCol	Actinospica_acidiphila_NRRL_B_24431	Streptomyces_sp._HNS054	Streptomyces_sp._SMS_SU21	Streptomyces_sp._ZS0098	Streptomyces_thermocarboxydus_K155	Addition	Core-Accessory-Unique	COG	%Similarity_COG	Score_COG	Evalue_COG	KEGG	%Similarity_KEGG	Score_KEGG	Evalue_KEGG
2991	PWE10440.1	-	4-oxalomesaconate tautomerase	1	0	1	0	1	1	1	0	1	0	1	7	Accessory	COG2828_S#	49.4	309	1.91E-83	K09788	43.2	292	6.45E-78
2992	PWE12335.1	-	GTP-binding protein	0	0	1	1	1	1	1	1	1	1	1	9	Accessory	COG0523_R#	54.4	400	8.22E-111	NA	52.5	318	8.4E-86
2993	WP_102638868.1	-	fumarylacetoacetate hydrolase family protein	1	0	0	1	1	1	1	1	1	1	1	9	Accessory	COG3970_R#	52.7	397	6.96E-110				
2994	WP_028961776.1	potA	ABC transporter ATP-binding protein	1	1	1	1	1	1	1	1	1	1	1	11	CORE	COG3842_E#	46.1	312	2.26E-84	K11072	46.7	309	6.66E-83
2995	WP_028959134.1	-	winged helix-turn-helix domain-containing protein	1	1	1	1	1	1	1	1	1	1	1	11	CORE	COG3214_S#	41.5	265	2.42E-70	K09927	29.7	181	1.6E-44
2996	WP_122216980.1	-	MarP family serine protease	1	1	1	1	1	1	1	1	1	1	1	11	CORE	COG0265_O#	38.4	243	1.68E-63	NA	38.4	243	4.49E-63
2997	WP_102641685.1	-	acyltransferase	0	0	1	1	0	0	0	1	1	1	0	5	Accessory								
2998	Prokka:JAPCLKMA_05489	-	hypothetical protein	1	0	0	0	0	0	0	0	0	0	0	1	Unique	COG1541_H#	25.8	97	1.14E-19				
2999	WP_063823446.1	-	hypothetical protein	1	0	0	1	1	1	1	1	1	1	1	9	Accessory								

cluster	GI	gene	Annotation	Streptomyces_thermocarboxydus_BPSAC147	Streptomyces_sp._4F	Streptomyces_sp._BSE7F	Streptomyces_sp._ETH9427	Streptomyces_sp._FxanaD5	Streptomyces_sp._UNC401CLCol	Actinospica_acidiphila_NRRL_B_24431	Streptomyces_sp._HNS054	Streptomyces_sp._SMS_SU21	Streptomyces_sp._ZS0098	Streptomyces_thermocarboxydus_K155	Addition	Core-Accessory-Unique	COG	%Similarity_COG	Score_COG	Evalue_COG	KEGG	%Similarity_KEGG	Score_KEGG	Evalue_KEGG
3000	AXI90441.1	-	FAD-dependent oxidoreductase	0	0	1	1	0	0	0	1	1	1	0	5	Accessory	COG0644_C#	26.3	98	6.69E-20				
3001	WP_122216301.1	-	MFS transporter	0	0	1	0	0	0	0	0	1	1	0	3	Accessory	COG0477_GEPR#	29.2	155	3.53E-37	K08223	27.7	154	1.61E-36
3002	PWE10962.1	-	alkaline D-peptidase	0	0	1	1	0	0	0	1	1	1	0	5	Accessory	COG1680_V#	33.6	151	5.09E-36	NA	27	101	2.11E-20
3003	PWE08988.1	-	hypothetical protein	0	0	1	0	0	0	0	0	0	0	0	1	Unique					NA	29.9	76	5.56E-13
3004	WP_048458956.1	-	membrane protein	1	1	1	0	1	1	1	1	1	0	1	9	Accessory					NA	37.2	58	1.57E-07
3005	WP_019527776.1	-	ImmA/IrrE family metallo-endopeptidase	0	0	0	0	1	0	0	0	0	0	0	1	Unique	COG2856_E#	32.9	99	3.92E-20	NA	30.8	113	5.36E-24
3006	WP_102639852.1	-	DNA (cytosine-5-)-methyltransferase	0	0	0	0	0	0	0	0	1	0	0	1	Unique	COG0270_L#	24.9	105	5.47E-22	K00558	24.9	105	1.46E-21
3007	OSC64821.1	-	glycoside hydrolase	1	1	1	1	1	1	1	1	1	1	1	11	CORE	COG0791_M#	29.5	133	1.88E-30	NA	29.5	133	5.01E-30
3008	WP_033272471.1	-	endo-1,4-beta-xylanase	1	0	1	1	1	1	1	1	1	1	1	10	Accessory	COG3693_G#	45	280	1.24E-74	NA	26.5	91	1.67E-17
3009	WP_102639161.1	nadA	quinolinate synthase NadA	1	1	1	1	1	1	1	1	1	1	1	11	CORE	COG0379_H#	55.3	401	4.81E-111	K03517	52.9	381	8.06E-105
3010	WP_033274870.1	nth	endonuclease III	1	1	1	1	1	1	1	1	1	1	1	11	CORE	COG0177_L#	60	301	3.97E-81	K10773	61.7	293	3.77E-78

cluster	GI	gene	Annotation	Streptomyces_thermocarboxydus_BPSAC147	Streptomyces_sp._4F	Streptomyces_sp._BSE7F	Streptomyces_sp._ETH9427	Streptomyces_sp._FxanaD5	Streptomyces_sp._UNC401CLCol	Actinospica_acidiphila_NRRL_B_24431	Streptomyces_sp._HNS054	Streptomyces_sp._SMS_SU21	Streptomyces_sp._ZS0098	Streptomyces_thermocarboxydus_K155	Addition	Core-Accessory-Unique	COG	%Similarity_COG	Score_COG	Evalue_COG	KEGG	%Similarity_KEGG	Score_KEGG	Evalue_KEGG
3011	AXI89693.1	-	amino acid ABC transporter substrate-binding protein	1	0	1	1	1	1	1	1	1	1	1	10	Accessory	COG0683_E#	26.2	96	1.94E-19	K01999	26.5	92	1.28E-17
3012	Prokka:JAPCLKMA_05043	aroC	Chorismate synthase	1	1	1	1	1	1	1	1	1	1	1	11	CORE	COG0082_E#	65	509	1.25E-143	K01736	65	509	3.35E-143
3013	WP_102639172.1	-	Gfo/Idh/MocA family oxidoreductase	1	1	1	1	1	1	1	1	1	1	1	11	CORE	COG0673_R#	29.1	106	3.2E-22	NA	25.8	43	0.00521
3014	WP_102639522.1	-	AAA family ATPase	0	0	1	0	0	0	0	0	1	0	0	2	Accessory	COG1106_R#	42.9	44	0.00149				
3015	WP_019522476.1	-	cobalt ABC transporter permease	1	1	1	1	1	1	1	1	1	1	1	11	CORE	COG0619_P#	39.7	258	2.95E-68				
3016	PWE09052.1	-	glutathionylspermidine synthase	1	1	1	1	1	1	1	1	1	1	1	11	CORE	COG0754_E#	41.4	277	6.14E-74	NA	38.9	268	7.62E-71
3017	OSC67190.1	-	FAD-dependent oxidoreductase	0	1	0	0	0	0	0	0	0	0	0	1	Unique	COG0446_R#	41.7	268	3.73E-71	NA	41.7	268	9.95E-71
3018	WP_033275428.1	-	acyl-CoA dehydrogenase family protein	0	0	0	0	1	1	1	0	0	0	1	4	Accessory	COG1960_I#	33.4	201	5.59E-51	K00249	34.7	200	3.33E-50
3019	AXI88761.1	-	cupin	0	0	0	1	0	0	0	0	0	0	0	1	Unique	COG2850_S#	27.7	110	1.7E-23	NA	27.7	110	4.53E-23
3020	Prokka:JAPCLKMA_03609	-	hypothetical protein	1	1	1	1	1	1	1	1	1	1	1	11	CORE								

cluster	GI	gene	Annotation	Streptomyces_thermocarboxydus_BPSAC147	Streptomyces_sp._4F	Streptomyces_sp._BSE7F	Streptomyces_sp._ETH9427	Streptomyces_sp._FxanaD5	Streptomyces_sp._UNC401CLCol	Actinospica_acidiphila_NRRL_B_24431	Streptomyces_sp._HNS054	Streptomyces_sp._SMS_SU21	Streptomyces_sp._ZS0098	Streptomyces_thermocarboxydus_K155	Addition	Core-Accessory-Unique	COG	%Similarity_COG	Score_COG	Evalue_COG	KEGG	%Similarity_KEGG	Score_KEGG	Evalue_KEGG
3021	WP_019527453.1	-	ATP-grasp domain-containing protein	0	0	0	0	1	0	0	0	0	0	0	1	Unique	COG0439_I#	26.3	88	5.28E-17	K13037	23.9	78	1.46E-13
3022	WP_033274334.1	-	hypothetical protein	1	0	1	1	1	1	1	1	1	1	1	10	Accessory					NA	27.8	91	2.84E-17
3023	Prokka:JAPCLKMA_02681	hmp_2	Flavohemoprotein	1	1	1	1	1	1	1	1	1	1	1	11	CORE	COG1018_C#	40.6	178	5.06E-44	K05916	40.3	249	6.23E-65
3024	PWE11648.1	-	acyl-CoA dehydrogenase	1	1	1	1	1	1	1	1	1	1	1	11	CORE	COG1960_I#	43.5	302	3.03E-81	NA	44	262	5.45E-69
3025	WP_019527887.1	sucC	ADP-forming succinate--CoA ligase subunit beta	1	1	1	1	1	1	1	1	1	1	1	11	CORE	COG0045_C#	65.5	500	7.59E-141	K01903	65.5	500	2.03E-140
3026	OSC71388.1	-	tyrosine decarboxylase MnfA	0	1	0	0	0	0	0	0	0	0	0	1	Unique	COG0076_E#	40.9	277	6.13E-74	K18933	33.6	211	1.1E-53
3027	OSC64573.1	-	MDMPI N domain containing protein	1	1	1	1	1	1	1	1	1	1	1	11	CORE								
3028	AXI85384.1	-	hypothetical protein	0	0	0	1	0	0	0	0	0	0	0	1	Unique								
3029	OSC57363.1	-	cytochrome	0	1	0	0	0	0	0	0	0	0	0	1	Unique	COG2124_Q#	35.8	203	1.12E-51	K15468	35.3	205	1.03E-51
3030	WP_048456179.1	-	two-component sensor histidine kinase	0	0	0	0	0	0	0	1	0	0	0	1	Unique	COG4585_T#	28.2	83	1.7E-15	K07778	28.2	83	4.53E-15
3031	OSC55885.1	-	hypothetical protein	0	1	0	0	0	0	0	0	0	0	0	1	Unique	COG1595_K#	30.4	60	1.18E-08	K03088	29.3	53	0.00000502
3032	AXI89618.1	-	hypothetical protein	1	0	0	1	0	0	0	0	0	0	0	2	Accessory								

cluster	GI	gene	Annotation	Streptomyces_thermocarboxydus_BPSAC147	Streptomyces_sp._4F	Streptomyces_sp._BSE7F	Streptomyces_sp._ETH9427	Streptomyces_sp._FxanaD5	Streptomyces_sp._UNC401CLCol	Actinospica_acidiphila_NRRL_B_24431	Streptomyces_sp._HNS054	Streptomyces_sp._SMS_SU21	Streptomyces_sp._ZS0098	Streptomyces_thermocarboxydus_K155	Addition	Core-Accessory-Unique	COG	%Similarity_COG	Score_COG	Evalue_COG	KEGG	%Similarity_KEGG	Score_KEGG	Evalue_KEGG
3033	Prokka:CIODPCDJ_04095	-	hypothetical protein	1	1	0	1	1	1	1	1	1	1	1	10	Accessory					NA	56.9	428	7.47E-119
3034	Prokka:CIODPCDJ_06827	-	hypothetical protein	0	0	0	0	0	0	0	0	0	0	1	1	Unique								
3035	WP_048455732.1	moeZ	adenylyltransferase/sulfurtransferase MoeZ	1	1	1	1	1	1	1	1	1	1	1	11	CORE	COG0476_H#	69.2	397	6.91E-110	K11996	64.8	532	2.81E-150
3036	WP_102638559.1	-	CapA family protein	1	1	1	1	1	1	1	1	1	1	1	11	CORE	COG2843_M#	25.9	74	7.86E-13				
3037	WP_102640580.1	-	membrane dipeptidase	1	1	1	1	1	1	1	1	1	1	1	11	CORE	COG2355_E#	46.5	206	1.73E-52	K01273	48.6	257	1.75E-67
3038	Prokka:JAPCLKMA_04723	-	hypothetical protein	1	1	1	1	1	1	1	1	1	1	1	11	CORE	COG2208_TK#	24.8	58	7.61E-08	K07315	24.8	58	2.03E-07
3039	AXI86686.1	-	glutamate 5-kinase	1	1	0	1	1	1	1	1	1	1	1	10	Accessory	COG0263_E#	51.8	345	2.39E-94	K00931	51.8	345	8.32E-94
3040	WP_026244296.1	alr	alanine racemase	1	1	1	1	1	1	1	1	1	1	1	11	CORE	COG0787_M#	48.7	335	3.22E-91	K01775	48.7	335	8.61E-91
3041	WP_048459668.1	-	amidase	1	0	1	1	1	1	1	1	1	1	1	10	Accessory	COG0154_J#	41.5	143	1.8E-33	K01426	41.5	143	4.8E-33
3042	WP_122216155.1	ugpC	sn-glycerol-3-phosphate ABC transporter ATP-binding protein UgpC	1	0	1	1	1	1	1	1	1	1	1	10	Accessory	COG3839_G#	61.6	458	2.53E-128	K10112	61.1	455	5.71E-127

cluster	GI	gene	Annotation	Streptomyces_thermocarboxydus_BPSAC147	Streptomyces_sp._4F	Streptomyces_sp._BSE7F	Streptomyces_sp._ETH9427	Streptomyces_sp._FxanaD5	Streptomyces_sp._UNC401CLCol	Actinospica_acidiphila_NRRL_B_24431	Streptomyces_sp._HNS054	Streptomyces_sp._SMS_SU21	Streptomyces_sp._ZS0098	Streptomyces_thermocarboxydus_K155	Addition	Core-Accessory-Unique	COG	%Similarity_COG	Score_COG	Evalue_COG	KEGG	%Similarity_KEGG	Score_KEGG	Evalue_KEGG
3043	WP_122214980.1	-	glycerophosphodiester phosphodiesterase	1	1	1	1	1	1	1	1	1	1	1	11	CORE	COG0584_C#	50.7	333	1.23E-90	K01126	35.8	182	9.33E-45
3044	WP_028959368.1	-	sensor histidine kinase	1	0	0	0	1	1	1	0	0	0	1	5	Accessory	COG4585_T#	40.6	189	2.19E-47	K07778	40.6	189	5.84E-47
3045	WP_078504629.1	-	helix-turn-helix domain-containing protein	0	0	0	0	1	0	0	0	0	0	0	1	Unique								
3046	WP_033272324.1	-	chromosome condensation regulator	0	0	0	0	0	0	1	0	1	0	0	2	Accessory					NA	26	70	3.96E-11
3047	Prokka:JAPCLKMA_05481	-	hypothetical protein	1	0	0	0	0	0	0	0	0	0	0	1	Unique								
3048	AXI90268.1	-	chloramphenicol efflux pump	0	0	1	1	0	0	1	0	1	0	0	4	Accessory	COG2814_G#	37.1	226	2.11E-58	K08156	33.2	192	6.9E-48
3049	WP_033277610.1	-	saccharopine dehydrogenase	1	1	0	0	1	1	1	1	1	1	1	9	Accessory	COG3268_S#	41.5	231	3.84E-60	NA	41.5	231	1.03E-59
3050	WP_158688215.1	-	DUF262 domain-containing protein	0	0	0	0	1	0	0	0	0	0	0	1	Unique	COG1479_S#	32.4	81	8.39E-15				
3051	AXI85341.1	-	hypothetical protein	0	0	0	1	0	0	0	0	0	0	0	1	Unique								
3052	OSC69364.1	-	carnitine dehydratase	0	1	0	0	0	0	0	0	0	0	0	1	Unique	COG1804_C#	51.4	369	1.18E-101	K18703	43.9	314	2.05E-84

cluster	GI	gene	Annotation	Streptomyces_thermocarboxydus_BPSAC147	Streptomyces_sp._4F	Streptomyces_sp._BSE7F	Streptomyces_sp._ETH9427	Streptomyces_sp._FxanaD5	Streptomyces_sp._UNC401CLCol	Actinospica_acidiphila_NRRL_B_24431	Streptomyces_sp._HNS054	Streptomyces_sp._SMS_SU21	Streptomyces_sp._ZS0098	Streptomyces_thermocarboxydus_K155	Addition	Core-Accessory-Unique	COG	%Similarity_COG	Score_COG	Evalue_COG	KEGG	%Similarity_KEGG	Score_KEGG	Evalue_KEGG
3053	OSC55696.1	-	ATP-binding protein	0	1	0	0	0	0	0	0	0	0	0	1	Unique	COG0642_T#	26.5	50	0.0000207				
3054	PWE11459.1	-	aldehyde dehydrogenase	1	0	1	1	1	1	1	1	1	1	1	10	Accessory	COG1063_ER#	49	359	1.59E-98	K00148	39.4	274	1.8E-72
3055	WP_019523172.1	-	hypothetical protein	1	1	1	1	1	1	1	1	1	1	1	11	CORE								
3056	WP_033276181.1	-	4-hydroxybenzoate 3-monooxygenase	1	1	0	0	1	1	1	0	0	0	1	6	Accessory	COG0654_HC#	58.1	458	3.29E-128	NA	27.6	78	1.89E-13
3057	AXI87956.1	-	integrase	0	1	0	1	0	0	0	0	1	0	0	3	Accessory	COG0582_L#	25.4	47	0.000103				
3058	Prokka:CIODPCDJ_00081	-	IS4 family transposase MICBce2	1	0	1	0	1	1	1	0	1	0	1	7	Accessory	COG1680_V#	31.5	141	5.22E-33	NA	28.2	96	8.77E-19
3059	WP_019523934.1	nagA	N-acetylglucosamine-6-phosphate deacetylase	1	1	1	1	1	1	1	1	1	1	1	11	CORE	COG1820_G#	49.3	330	1.03E-89	K01443	49.3	330	2.76E-89
3060	WP_019525056.1	-	hypothetical protein	1	1	1	1	1	1	1	1	1	1	1	11	CORE								
3061	PWE10479.1	-	phytanoyl-CoA dioxygenase	1	1	1	0	1	1	1	1	1	1	1	10	Accessory	COG5285_Q#	55.5	447	4.45E-125				
3062	WP_019524232.1	-	glutathione-dependent formaldehyde dehydrogenase	1	0	0	0	1	1	0	0	0	0	0	3	Accessory	COG1063_ER#	61.7	501	2.59E-141	NA	57.3	433	3.03E-120

cluster	GI	gene	Annotation	Streptomyces_thermocarboxydus_BPSAC147	Streptomyces_sp._4F	Streptomyces_sp._BSE7F	Streptomyces_sp._ETH9427	Streptomyces_sp._FxanaD5	Streptomyces_sp._UNC401CLCol	Actinospica_acidiphila_NRRL_B_24431	Streptomyces_sp._HNS054	Streptomyces_sp._SMS_SU21	Streptomyces_sp._ZS0098	Streptomyces_thermocarboxydus_K155	Addition	Core-Accessory-Unique	COG	%Similarity_COG	Score_COG	Evalue_COG	KEGG	%Similarity_KEGG	Score_KEGG	Evalue_KEGG
3063	WP_033274340.1	-	glutathione-dependent formaldehyde dehydrogenase	0	0	0	1	0	0	1	1	0	1	0	4	Accessory	COG1063_ER#	53.2	460	6.63E-129	NA	52.4	403	1.97E-111
3064	WP_033272331.1	-	hypothetical protein	1	0	1	1	1	1	1	1	1	1	1	10	Accessory	COG0513_LKJ#	35.1	53	0.00000187	NA	27.9	92	1.27E-17
3065	WP_019521839.1	-	hypothetical protein	0	0	0	0	1	1	0	0	0	0	1	3	Accessory								
3066	AXI87889.1	galK	galactokinase	1	1	1	1	1	1	1	1	1	1	1	11	CORE	COG0153_G#	43	266	1.83E-70	K00849	43	266	4.89E-70
3067	WP_019523699.1	-	acyl-CoA dehydrogenase	1	1	1	1	1	1	1	1	1	1	1	11	CORE	COG1960_I#	51.5	369	1.18E-101	NA	51.5	369	3.14E-101
3068	WP_102641000.1	-	acetoin utilization protein AcuC	1	1	1	1	1	1	1	1	1	1	1	11	CORE	COG0123_BQ#	41	287	5.87E-77	K04768	41	287	1.57E-76
3069	WP_122216458.1	-	S1 family peptidase	1	1	1	1	1	1	1	1	1	1	1	11	CORE								
3070	AXI87745.1	-	acyl-CoA dehydrogenase	1	1	1	1	1	1	1	1	1	1	1	11	CORE	COG1960_I#	64.6	511	3.27E-144	K00257	64.6	511	8.72E-144
3071	WP_122216791.1	-	hypothetical protein	1	1	0	1	1	1	1	1	1	1	1	10	Accessory								
3072	WP_122214544.1	-	5-(carboxyamino)imidazole ribonucleotide synthase	1	1	1	1	1	1	1	1	1	1	1	11	CORE	COG0026_F#	52.9	376	9.61E-104	K01589	52.9	376	2.57E-103

cluster	GI	gene	Annotation	Streptomyces_thermocarboxydus_BPSAC147	Streptomyces_sp._4F	Streptomyces_sp._BSE7F	Streptomyces_sp._ETH9427	Streptomyces_sp._FxanaD5	Streptomyces_sp._UNC401CLCol	Actinospica_acidiphila_NRRL_B_24431	Streptomyces_sp._HNS054	Streptomyces_sp._SMS_SU21	Streptomyces_sp._ZS0098	Streptomyces_thermocarboxydus_K155	Addition	Core-Accessory-Unique	COG	%Similarity_COG	Score_COG	Evalue_COG	KEGG	%Similarity_KEGG	Score_KEGG	Evalue_KEGG
3073	OSC72133.1	-	citrate synthase/methylcitrate synthase	0	1	0	0	0	0	0	0	0	0	0	1	Unique	COG0372_C#	36.7	255	3.23E-67	K01647	36.7	255	6.61E-67
3074	OSC68731.1	-	2Fe-2S ferredoxin	0	1	0	0	0	0	0	0	0	0	0	1	Unique	COG4638_PR#	31.8	201	5.54E-51	NA	29.4	167	3.09E-40
3075	WP_078504464.1	-	ATP-binding cassette domain-containing protein	1	1	1	1	1	1	1	1	1	1	1	11	CORE	COG1136_V#	33.3	80	1.42E-14	K11962	32	74	2.09E-12
3076	Prokka:JAPCLKMA_02611	-	hypothetical protein	1	0	0	0	0	0	0	0	0	0	0	1	Unique	COG1835_I#	27.1	87	8.92E-17				
3077	Prokka:JAPCLKMA_05160	-	hypothetical protein	1	0	0	0	0	0	0	0	0	0	0	1	Unique								
3078	WP_033273799.1	-	hypothetical protein	0	0	0	0	0	0	1	0	0	0	0	1	Unique								
3079	PWE11725.1	-	thiolase family protein	1	0	1	1	1	1	1	1	1	1	1	10	Accessory	COG0183_I#	50.5	368	3.41E-101	K00626	46	327	2.33E-88
3080	AXI86441.1	-	oxidoreductase	0	0	0	1	0	0	0	1	0	0	0	2	Accessory	COG1960_I#	30.7	159	2.41E-38	K16047	32.1	155	9.28E-37
3081	Prokka:CIODPCDJ_06245	-	Lactate 2-monooxygenase	1	1	0	1	1	1	1	1	1	1	1	10	Accessory	COG1304_C#	49	343	1.18E-93	K11517	36.7	240	2.86E-62
3082	PWE06780.1	-	DNA methyltransferase	1	1	1	1	1	1	1	1	1	1	1	11	CORE								
3083	WP_028961695.1	-	hypothetical protein	0	0	0	0	1	1	1	0	0	0	1	4	Accessory								
3084	WP_048456995.1	-	site-specific integrase	0	0	0	0	1	0	0	1	0	0	0	2	Accessory	COG0582_L#	26.5	106	2.42E-22	NA	26.5	106	6.46E-22

cluster	GI	gene	Annotation	Streptomyces_thermocarboxydus_BPSAC147	Streptomyces_sp._4F	Streptomyces_sp._BSE7F	Streptomyces_sp._ETH9427	Streptomyces_sp._FxanaD5	Streptomyces_sp._UNC401CLCol	Actinospica_acidiphila_NRRL_B_24431	Streptomyces_sp._HNS054	Streptomyces_sp._SMS_SU21	Streptomyces_sp._ZS0098	Streptomyces_thermocarboxydus_K155	Addition	Core-Accessory-Unique	COG	%Similarity_COG	Score_COG	Evalue_COG	KEGG	%Similarity_KEGG	Score_KEGG	Evalue_KEGG
3085	PWE12148.1	-	FAD-dependent oxidoreductase	0	0	1	1	0	0	0	1	1	1	0	5	Accessory	COG1252_C#	28.8	122	4.26E-27	K03885	31.6	121	2.53E-26
3086	Prokka:JAPCLKMA_06201	-	hypothetical protein	1	0	1	0	1	1	1	0	1	0	1	7	Accessory	COG3552_R#	36.7	202	2.48E-51	K07161	36.7	193	5.24E-48
3087	Prokka:CIODPCDJ_06039	tdh_3	L-threonine 3-dehydrogenase	1	0	0	0	1	1	0	0	0	0	1	4	Accessory	COG1063_ER#	32.6	161	8.27E-39	K00001	32.6	161	2.21E-38
3088	OSC72784.1	-	hypothetical protein	0	1	0	0	0	0	0	0	0	0	0	1	Unique								
3089	WP_122213653.1	-	cysteine desulfurase	1	1	1	1	1	1	1	1	1	1	1	11	CORE	COG1104_E#	62.2	479	1.37E-134	K04487	62.2	479	3.66E-134
3090	OSC70205.1	-	peptidase M48	1	1	1	1	1	1	1	1	1	1	1	11	CORE	COG0501_O#	46	303	1.03E-81	NA	46	303	2.76E-81
3091	OSC68732.1	-	N-methyltryptophan oxidase	0	1	0	0	0	0	0	0	0	0	0	1	Unique	COG0665_E#	34.6	205	4.99E-52	K02846	34.8	200	3.29E-50
3092	OSC63701.1	-	hypothetical protein	0	1	0	0	0	0	0	0	0	0	0	1	Unique								
3093	PWE09371.1	-	hypothetical protein	1	1	1	1	1	1	1	1	1	1	1	11	CORE	COG0791_M#	25	122	2.5E-27	NA	27.3	110	4.47E-23
3094	PWE08500.1	-	site-specific integrase	1	1	1	0	0	0	1	0	0	0	1	5	Accessory	COG0582_L#	39	234	7.69E-61	NA	39	234	2.05E-60
3095	OSC66916.1	-	D-alanine--D-alanine ligase A	1	1	1	1	1	1	1	1	1	1	1	11	CORE	COG1181_M#	50.4	358	2.7E-98	K01921	50.1	357	2.1E-97
3096	OSC60203.1	-	ATP/GTP-binding protein	1	1	1	1	1	1	1	1	1	1	1	11	CORE	COG2301_G#	32.8	134	6.35E-31				

cluster	GI	gene	Annotation	Streptomyces_thermocarboxydus_BPSAC147	Streptomyces_sp._4F	Streptomyces_sp._BSE7F	Streptomyces_sp._ETH9427	Streptomyces_sp._FxanaD5	Streptomyces_sp._UNC401CLCol	Actinospica_acidiphila_NRRL_B_24431	Streptomyces_sp._HNS054	Streptomyces_sp._SMS_SU21	Streptomyces_sp._ZS0098	Streptomyces_thermocarboxydus_K155	Addition	Core-Accessory-Unique	COG	%Similarity_COG	Score_COG	Evalue_COG	KEGG	%Similarity_KEGG	Score_KEGG	Evalue_KEGG
3097	WP_019526181.1	mycP	type VII secretion-associated serine protease mycosin	1	1	1	1	1	1	1	1	1	1	1	11	CORE	COG1404_O#	36.5	160	1.41E-38	K14645	33.6	139	8.96E-32
3098	WP_086705387.1	-	mandelate racemase/ muconate lactonizing enzyme family protein	1	1	1	1	1	1	1	1	1	1	1	11	CORE	COG4948_MR#	37.6	265	4.06E-70	K01684	35.5	217	1.98E-55
3099	WP_019521972.1	thiO	glycine oxidase ThiO	1	1	1	1	1	1	1	1	1	1	1	11	CORE	COG0665_E#	37	208	4.51E-53	K03149	36.2	209	5.4E-53
3100	PWE11789.1	xylA	xylose isomerase	1	1	1	1	1	1	1	1	1	1	1	11	CORE	COG2115_G#	30.2	127	1.01E-28	K01805	29	123	3.9E-27
3101	PWE06610.1	-	transcriptional regulator	0	0	1	0	0	0	0	0	0	0	0	1	Unique	COG1396_K#	30.4	42	0.00327				
3102	AXI88759.1	-	transcriptional regulator	0	0	0	1	0	0	0	0	0	0	0	1	Unique	COG1396_K#	46.2	42	0.00558				
3103	OSC70714.1	-	FAD-dependent oxidoreductase	1	1	1	0	1	1	1	1	1	1	1	10	Accessory	COG0665_E#	28.1	127	1.32E-28	NA	30.9	152	1.02E-35
3104	WP_122216221.1	-	sensor histidine kinase	0	0	1	1	0	0	0	0	1	1	0	4	Accessory	COG4585_T#	33.7	120	1.24E-26	K07778	30.1	113	4.03E-24
3105	WP_102641432.1	-	hypothetical protein	0	0	1	1	0	0	0	1	1	1	0	5	Accessory	COG3321_Q#	29.4	56	2.19E-07	K12436	32.2	57	4.48E-07
3106	WP_019524840.1	-	DUF1205 domain-containing protein	0	0	0	0	1	1	0	0	0	0	1	3	Accessory	COG1819_GC#	26.9	134	8.27E-31	NA	27.9	73	4.63E-12

cluster	GI	gene	Annotation	Streptomyces_thermocarboxydus_BPSAC147	Streptomyces_sp._4F	Streptomyces_sp._BSE7F	Streptomyces_sp._ETH9427	Streptomyces_sp._FxanaD5	Streptomyces_sp._UNC401CLCol	Actinospica_acidiphila_NRRL_B_24431	Streptomyces_sp._HNS054	Streptomyces_sp._SMS_SU21	Streptomyces_sp._ZS0098	Streptomyces_thermocarboxydus_K155	Addition	Core-Accessory-Unique	COG	%Similarity_COG	Score_COG	Evalue_COG	KEGG	%Similarity_KEGG	Score_KEGG	Evalue_KEGG
3107	WP_122213953.1	-	P1 family peptidase	0	0	0	0	0	0	0	0	0	1	0	1	Unique	COG3191_EQ#	45.9	253	1.6E-66	NA	45.6	236	5.39E-61
3108	OSC62533.1	-	two-component sensor histidine kinase	1	1	1	1	1	1	1	1	1	0	1	10	Accessory	COG4585_T#	26.5	80	1.08E-14	K07778	22.9	68	1.49E-10
3109	PWE11052.1	-	cytochrome P450	0	1	1	0	0	0	0	0	0	0	0	2	Accessory	COG2124_Q#	29.5	75	3.49E-13	NA	27.2	74	3.54E-12
3110	Prokka:JAPCLKMA_02722	-	hypothetical protein	1	0	1	0	0	0	0	0	0	0	0	2	Accessory	COG1479_S#	36.2	166	2.56E-40				
3111	WP_048458198.1	-	glycosyltransferase family 4 protein	1	1	1	1	1	1	1	1	1	1	1	11	CORE	COG0438_M#	52.9	369	1.17E-101	K08256	52.9	369	3.11E-101
3112	OSC55317.1	-	myo-inositol 2-dehydrogenase	1	1	1	1	1	1	1	1	1	1	1	11	CORE	COG0673_R#	37.1	240	8.17E-63	K00540	25.9	115	1.06E-24
3113	PWE09209.1	-	hypothetical protein	1	0	1	1	1	1	1	1	1	1	1	10	Accessory					NA	35.9	66	5.64E-10
3114	AXI87959.1	-	cardiolipin synthase B	1	1	1	1	1	1	1	1	1	1	1	11	CORE	COG1502_I#	29.2	178	4.98E-44	K06131	29.4	170	3.62E-41
3115	Prokka:JAPCLKMA_00888	mmgC_1	Acyl-CoA dehydrogenase	1	0	0	0	1	1	1	0	0	0	1	5	Accessory	COG1960_I#	62.6	476	8.85E-134	K00257	62.6	476	2.36E-133
3116	AXI88064.1	-	aminofutalosine synthase MqnE	1	1	1	1	1	1	1	1	1	1	1	11	CORE	COG1060_HR#	42.5	310	6.43E-84	K18285	42.5	285	1.01E-75
3117	Prokka:JAPCLKMA_02739	-	hypothetical protein	1	0	0	0	0	0	0	0	0	0	0	1	Unique								
3118	WP_084207080.1	-	CoA transferase	0	0	0	0	0	0	1	0	0	0	0	1	Unique	COG1804_C#	50.3	348	3.63E-95	K01796	50.3	348	9.7E-95

cluster	GI	gene	Annotation	Streptomyces_thermocarboxydus_BPSAC147	Streptomyces_sp._4F	Streptomyces_sp._BSE7F	Streptomyces_sp._ETH9427	Streptomyces_sp._FxanaD5	Streptomyces_sp._UNC401CLCol	Actinospica_acidiphila_NRRL_B_24431	Streptomyces_sp._HNS054	Streptomyces_sp._SMS_SU21	Streptomyces_sp._ZS0098	Streptomyces_thermocarboxydus_K155	Addition	Core-Accessory-Unique	COG	%Similarity_COG	Score_COG	Evalue_COG	KEGG	%Similarity_KEGG	Score_KEGG	Evalue_KEGG
3119	WP_052183400.1	zapE	cell division protein ZapE	1	1	1	1	1	1	1	1	1	1	1	11	CORE	COG1485_R#	52.1	335	2.44E-91	K06916	52.1	335	6.51E-91
3120	OSC66076.1	-	MFS transporter	0	1	0	0	0	0	0	0	0	0	0	1	Unique	COG0477_GEPR#	25.2	102	3.47E-21	K08225	23.1	81	2.21E-14
3121	WP_048454951.1	rhaI	L-rhamnose isomerase	1	1	1	1	1	1	1	1	1	1	1	11	CORE	COG4952_M#	50.5	381	2.96E-105				
3122	WP_102637476.1	-	CoA transferase	1	1	1	1	1	1	1	1	1	1	1	11	CORE	COG1804_C#	55.8	390	6.37E-108	K01796	55.8	390	1.7E-107
3123	OSC65313.1	-	FAD-dependent oxidoreductase	1	1	1	1	1	1	1	1	1	1	1	11	CORE	COG1233_Q#	46.7	52	0.00000411	K00266	45.7	44	0.0039
3124	PWE07629.1	-	acyl-CoA dehydrogenase	1	1	1	1	1	1	1	1	1	1	1	11	CORE	COG1960_I#	70.2	527	3.34E-149	NA	70.2	527	8.91E-149
3125	WP_019527421.1	-	DUF3631 domain-containing protein	1	0	0	0	1	0	1	0	0	0	0	3	Accessory					NA	47.5	340	2.02E-92
3126	Prokka:CIODPCDJ_01765	-	hypothetical protein	1	1	1	1	1	1	1	1	1	1	1	11	CORE								
3127	Prokka:CIODPCDJ_05716	-	hypothetical protein	1	1	0	0	1	1	0	0	0	0	1	5	Accessory								
3128	OSC56072.1	-	glycosyl transferase family 1	0	1	0	0	0	0	0	0	0	0	0	1	Unique								
3129	Prokka:CIODPCDJ_06001	msuD	Methanesulfonate monooxygenase	1	1	0	0	1	1	1	0	0	0	1	6	Accessory	COG2141_C#	62.7	463	1.01E-129	K04091	51.3	372	4.79E-102

cluster	GI	gene	Annotation	Streptomyces_thermocarboxydus_BPSAC147	Streptomyces_sp._4F	Streptomyces_sp._BSE7F	Streptomyces_sp._ETH9427	Streptomyces_sp._FxanaD5	Streptomyces_sp._UNC401CLCol	Actinospica_acidiphila_NRRL_B_24431	Streptomyces_sp._HNS054	Streptomyces_sp._SMS_SU21	Streptomyces_sp._ZS0098	Streptomyces_thermocarboxydus_K155	Addition	Core-Accessory-Unique	COG	%Similarity_COG	Score_COG	Evalue_COG	KEGG	%Similarity_KEGG	Score_KEGG	Evalue_KEGG
3130	AXI86449.1	-	isopropylmalate synthase	0	0	0	1	0	0	0	1	0	0	0	2	Accessory	COG0119_E#	34.8	158	4.07E-38	K09011	31	154	2.05E-36
3131	Prokka:CIODPCDJ_02542	ctpA_2	Carboxy-terminal processing protease CtpA	1	1	1	1	1	1	1	1	1	1	1	11	CORE	COG0793_M#	32.1	186	2.38E-46	K03797	35.5	168	1.05E-40
3132	OSC54910.1	-	glycosyl transferase	0	1	0	0	0	0	0	0	0	0	0	1	Unique	COG0438_M#	51.2	319	1.8E-86	K16150	29.6	96	5.08E-19
3133	OSC64048.1	-	MarR family transcriptional regulator	0	1	0	0	0	0	0	0	0	0	0	1	Unique	COG1940_KG#	36.8	166	1.95E-40	K00845	28.9	106	6.41E-22
3134	PWE08114.1	-	hypothetical protein	0	0	1	1	1	1	1	1	1	0	0	7	Accessory	COG0582_L#	46.5	46	0.000226	NA	35.7	43	0.0051
3135	OSC72633.1	-	peptidase	0	1	0	0	0	0	0	0	0	0	0	1	Unique	COG1680_V#	30.3	100	1.72E-20	NA	29.4	83	5.81E-15
3136	OSC72442.1	-	hypothetical protein	0	1	0	0	0	0	0	0	0	0	0	1	Unique								
3137	Prokka:JAPCLKMA_06565	-	hypothetical protein	1	0	0	0	0	0	0	0	1	0	0	2	Accessory								
3138	WP_028959874.1	-	two-component sensor histidine kinase	1	1	1	1	0	1	1	1	1	1	1	10	Accessory	COG4585_T#	30.6	87	1.5E-16	NA	30.6	87	4.01E-16
3139	PWE12416.1	-	serine hydrolase	0	0	1	1	0	0	1	1	1	1	0	6	Accessory	COG1680_V#	30.7	143	2.31E-33	NA	27.3	110	3.39E-23
3140	Prokka:JAPCLKMA_05403	yehL_2	putative protein YehL	1	1	1	1	1	1	1	1	1	1	1	11	CORE	COG0714_R#	38.8	233	9.94E-61	NA	67.5	492	4.14E-138

cluster	GI	gene	Annotation	Streptomyces_thermocarboxydus_BPSAC147	Streptomyces_sp._4F	Streptomyces_sp._BSE7F	Streptomyces_sp._ETH9427	Streptomyces_sp._FxanaD5	Streptomyces_sp._UNC401CLCol	Actinospica_acidiphila_NRRL_B_24431	Streptomyces_sp._HNS054	Streptomyces_sp._SMS_SU21	Streptomyces_sp._ZS0098	Streptomyces_thermocarboxydus_K155	Addition	Core-Accessory-Unique	COG	%Similarity_COG	Score_COG	Evalue_COG	KEGG	%Similarity_KEGG	Score_KEGG	Evalue_KEGG
3141	OSC61506.1	-	C4-dicarboxylate ABC transporter	0	1	0	0	0	0	0	0	0	0	0	1	Unique	COG1275_P#	24.9	143	2.31E-33	NA	24.9	143	6.16E-33
3142	WP_033277527.1	-	uroporphyrinogen-III synthase	1	1	1	1	1	1	1	1	1	1	1	11	CORE	COG1587_H#	55	280	9.26E-75	K01719	55.5	389	4.94E-107
3143	AXI86814.1	-	dihydrodipicolinate synthase family protein	1	1	1	1	1	1	1	1	1	1	1	11	CORE								
3144	AXI91227.1	-	phosphopantetheine--protein transferase	0	1	0	1	0	0	0	0	0	0	0	2	Accessory	COG1192_D#	31	108	6.3E-23	K03496	28.5	99	1.02E-19
3145	PWE07625.1	-	acyl-CoA dehydrogenase	1	1	1	1	1	1	1	1	1	1	1	11	CORE	COG1960_I#	48.3	347	8.05E-95	K00257	47.8	343	3.11E-93
3146	WP_033276207.1	-	acyl-CoA dehydrogenase	1	1	1	1	1	1	1	1	1	1	1	11	CORE	COG1960_I#	67.6	510	4.21E-144	K00257	67.6	510	1.12E-143
3147	Prokka:CIODPCDJ_06772	-	hypothetical protein	0	0	0	0	1	0	0	0	0	0	1	2	Accessory	COG1944_S#	34.5	168	3.93E-41	K09136	34.5	168	1.05E-40
3148	Prokka:JAPCLKMA_01907	nagC_2	N-acetylglucosamine repressor	1	1	1	1	1	1	1	1	1	1	1	11	CORE	COG1940_KG#	27.6	133	1.83E-30	K00845	29.3	101	2.68E-20
3149	Prokka:JAPCLKMA_01254	osmV	Osmoprotectant import ATP-binding protein OsmV	1	1	1	1	1	1	1	1	1	1	1	11	CORE	COG1125_E#	53.8	342	1.52E-93	K05847	53.8	342	4.06E-93

cluster	GI	gene	Annotation	Streptomyces_thermocarboxydus_BPSAC147	Streptomyces_sp._4F	Streptomyces_sp._BSE7F	Streptomyces_sp._ETH9427	Streptomyces_sp._FxanaD5	Streptomyces_sp._UNC401CLCol	Actinospica_acidiphila_NRRL_B_24431	Streptomyces_sp._HNS054	Streptomyces_sp._SMS_SU21	Streptomyces_sp._ZS0098	Streptomyces_thermocarboxydus_K155	Addition	Core-Accessory-Unique	COG	%Similarity_COG	Score_COG	Evalue_COG	KEGG	%Similarity_KEGG	Score_KEGG	Evalue_KEGG
3150	OSC63569.1	-	peptidase	0	1	0	0	0	0	0	0	0	0	0	1	Unique	COG3182_S#	42.1	260	7.59E-69	NA	38.6	44	0.00298
3151	WP_028961017.1	-	spore photoproduct lyase family protein	1	1	1	1	1	1	1	1	1	1	1	11	CORE	COG1533_L#	35.9	122	3.23E-27	K03716	25.5	107	2.87E-22
3152	WP_158688226.1	-	hypothetical protein	0	0	0	0	1	0	0	0	0	0	0	1	Unique								
3153	WP_019522246.1	-	FAD-dependent monooxygenase	1	0	0	0	1	1	0	0	0	0	1	4	Accessory	COG0654_HC#	40.8	242	3.65E-63	NA	28	122	1.13E-26
3154	WP_019525997.1	-	cystathionine gamma-synthase	1	1	1	1	1	1	1	1	1	1	1	11	CORE	COG0626_E#	66.8	501	3.34E-141	K01739	66.8	501	8.91E-141
3155	AXI86602.1	-	translocation protein TolB	0	1	0	1	0	0	0	1	1	1	0	5	Accessory	COG3211_R#	33.4	176	1.44E-43				
3156	WP_102640389.1	ispG	flavodoxin-dependent (E)-4-hydroxy-3-methylbut-2-enyl-diphosphate synthase	1	1	1	1	1	1	1	0	1	1	1	10	Accessory	COG0821_I#	78.9	592	1.1E-168	K03526	78.9	592	2.94E-168
3157	Prokka:CIODPCDJ_02240	-	D-alanyl-D-alanine carboxypeptidase	1	1	1	1	1	1	1	1	1	1	1	11	CORE	COG1680_V#	35.4	177	1.1E-43	K01286	29.8	112	6.83E-24
3158	WP_102638691.1	-	transcription termination factor Rho	0	1	1	0	0	0	1	0	1	0	0	4	Accessory	COG1158_K#	53	371	3.05E-102	K03628	53	371	8.15E-102

cluster	GI	gene	Annotation	Streptomyces_thermocarboxydus_BPSAC147	Streptomyces_sp._4F	Streptomyces_sp._BSE7F	Streptomyces_sp._ETH9427	Streptomyces_sp._FxanaD5	Streptomyces_sp._UNC401CLCol	Actinospica_acidiphila_NRRL_B_24431	Streptomyces_sp._HNS054	Streptomyces_sp._SMS_SU21	Streptomyces_sp._ZS0098	Streptomyces_thermocarboxydus_K155	Addition	Core-Accessory-Unique	COG	%Similarity_COG	Score_COG	Evalue_COG	KEGG	%Similarity_KEGG	Score_KEGG	Evalue_KEGG
3159	WP_106963455.1	-	cytochrome P450	0	0	0	0	0	0	1	0	0	0	0	1	Unique	COG2124_Q#	32.4	189	2.81E-47	K00517	32.4	189	7.5E-47
3160	WP_102637812.1	-	site-specific integrase	0	0	0	1	0	0	0	0	1	0	0	2	Accessory	COG0582_L#	41	250	7.85E-66	NA	41	250	2.1E-65
3161	Prokka:JAPCLKMA_03956	-	hypothetical protein	1	0	0	0	0	0	0	0	0	0	0	1	Unique								
3162	Prokka:CIODPCDJ_05623	-	hypothetical protein	1	1	1	1	1	1	1	1	1	1	1	11	CORE	COG0385_R#	54.4	322	1.63E-87	K14347	46.7	285	1E-75
3163	OSC61431.1	-	glycosyl hydrolase	1	1	0	1	1	1	1	1	1	1	1	10	Accessory								
3164	WP_019521992.1	-	hypothetical protein	1	1	0	1	1	1	1	1	1	1	1	10	Accessory	COG0003_P#	33.1	156	2.63E-37	K01551	34.4	137	2.58E-31
3165	Prokka:JAPCLKMA_02237	-	hypothetical protein	1	0	1	0	1	1	1	1	1	1	1	9	Accessory								
3166	WP_033274646.1	-	glycosyltransferase	1	1	1	1	1	1	1	1	1	1	1	11	CORE	COG0438_M#	30.4	114	1.14E-24	NA	31.2	107	2.19E-22
3167	OSC69935.1	-	hypothetical protein	0	1	0	0	0	0	0	0	0	0	0	1	Unique								
3168	WP_033272686.1	-	protein kinase	1	0	1	1	1	1	1	1	1	1	1	10	Accessory	COG0515_RTKL#	32.7	140	1.49E-32	K08884	35.7	139	6.79E-32
3169	Prokka:JAPCLKMA_01597	cysK2_1	S-sulfocysteine synthase	1	0	0	0	1	1	0	0	0	0	1	4	Accessory	COG0031_E#	66.2	488	2.23E-137	K01738	66.2	488	5.96E-137
3170	WP_048455371.1	-	hypothetical protein	0	1	0	1	0	0	0	1	0	1	0	4	Accessory					NA	28.9	130	4.12E-29
3171	Prokka:CIODPCDJ_02021	mro_2	Aldose 1-epimerase	1	1	1	1	1	1	1	1	1	1	1	11	CORE	COG2017_G#	46.9	337	8.31E-92	K01785	45.6	302	4.64E-81
3172	WP_033275782.1	dusB	tRNA dihydrouridine synthase DusB	1	0	1	1	1	1	1	1	1	1	1	10	Accessory	COG0042_J#	59.8	431	3.24E-120	NA	59.8	431	8.65E-120

cluster	GI	gene	Annotation	Streptomyces_thermocarboxydus_BPSAC147	Streptomyces_sp._4F	Streptomyces_sp._BSE7F	Streptomyces_sp._ETH9427	Streptomyces_sp._FxanaD5	Streptomyces_sp._UNC401CLCol	Actinospica_acidiphila_NRRL_B_24431	Streptomyces_sp._HNS054	Streptomyces_sp._SMS_SU21	Streptomyces_sp._ZS0098	Streptomyces_thermocarboxydus_K155	Addition	Core-Accessory-Unique	COG	%Similarity_COG	Score_COG	Evalue_COG	KEGG	%Similarity_KEGG	Score_KEGG	Evalue_KEGG
3173	WP_048457082.1	arsB	ACR3 family arsenite efflux transporter	1	1	1	1	1	1	1	1	1	1	1	11	CORE	COG0798_P#	74	508	2.08E-143	K03325	72.7	515	5.95E-145
3174	WP_033274216.1	-	GTP-binding protein	0	0	1	1	1	1	1	1	1	1	1	9	Accessory	COG0523_R#	35.3	176	1.88E-43	NA	27.1	137	2.58E-31
3175	WP_033276654.1	-	ImmA/IrrE family metallo-endopeptidase	0	0	0	0	0	0	1	0	0	0	1	2	Accessory	COG2856_E#	43.3	178	4.94E-44	NA	38.8	228	1.11E-58
3176	Prokka:JAPCLKMA_06631	-	hypothetical protein	1	0	0	0	0	0	0	0	0	0	0	1	Unique								
3177	OSC61243.1	-	beta-mannosidase	0	1	0	0	0	0	0	0	0	0	0	1	Unique								
3178	OSC69648.1	-	iron ABC transporter	0	1	1	1	0	0	1	1	0	1	0	6	Accessory	COG0609_P#	36.6	191	7.37E-48	K02015	36.6	191	1.97E-47
3179	OSC71172.1	-	serine/threonine protein phosphatase	0	1	0	0	0	0	0	0	0	0	0	1	Unique	COG0515_RTKL#	29.6	65	6.1E-10	NA	28.2	65	1.25E-09
3180	Prokka:JAPCLKMA_00167	btuD_1	Vitamin B12 import ATP-binding protein BtuD	1	1	1	1	1	1	1	1	1	1	0	10	Accessory	COG0411_E#	50.6	248	5.08E-65	K01995	49.1	247	2.31E-64
3181	WP_033273007.1	-	DivIVA domain-containing protein	1	1	1	1	1	1	1	1	1	1	1	11	CORE	COG3599_D#	48.3	105	4.07E-22	NA	48.3	105	1.09E-21

cluster	GI	gene	Annotation	Streptomyces_thermocarboxydus_BPSAC147	Streptomyces_sp._4F	Streptomyces_sp._BSE7F	Streptomyces_sp._ETH9427	Streptomyces_sp._FxanaD5	Streptomyces_sp._UNC401CLCol	Actinospica_acidiphila_NRRL_B_24431	Streptomyces_sp._HNS054	Streptomyces_sp._SMS_SU21	Streptomyces_sp._ZS0098	Streptomyces_thermocarboxydus_K155	Addition	Core-Accessory-Unique	COG	%Similarity_COG	Score_COG	Evalue_COG	KEGG	%Similarity_KEGG	Score_KEGG	Evalue_KEGG
3182	WP_048459979.1	argJ	bifunctional glutamate N-acetyltransferase/amino-acid acetyltransferase ArgJ	1	1	1	1	1	1	1	1	1	1	1	11	CORE	COG1364_E#	59.9	426	7.96E-119	K00620	58.6	416	2.87E-115
3183	WP_122212936.1	-	PLP-dependent cysteine synthase family protein	1	1	1	1	1	1	1	1	1	1	1	11	CORE	COG0031_E#	62.9	451	3.94E-126	K01738	63.6	441	8.34E-123
3184	WP_033276743.1	-	acyl-CoA dehydrogenase	1	1	1	1	1	1	1	1	1	1	1	11	CORE	COG1960_I#	48.2	349	2.11E-95	NA	48.2	349	5.63E-95
3185	WP_122216294.1	glgA	glycogen synthase	1	0	1	1	1	1	1	1	1	1	1	10	Accessory	COG0438_M#	55.8	421	4.37E-117	K16148	56.7	413	2.43E-114
3186	Prokka:CIODPCDJ_05497	glkA_3	Glucokinase	1	1	1	1	1	1	1	1	1	1	1	11	CORE	COG1940_KG#	46.5	290	8.9E-78	K00845	33.8	181	1.55E-44
3187	OSC61150.1	-	epimerase	0	1	0	0	0	0	0	0	0	0	0	1	Unique	COG3268_S#	28.9	70	1.45E-11				
3188	Prokka:JAPCLKMA_00510	metB_1	Cystathionine gamma-synthase	1	1	1	1	1	1	1	1	1	1	1	11	CORE	COG0626_E#	46.1	314	7.52E-85	K01758	37.2	216	4.36E-55
3189	WP_019524152.1	-	site-specific integrase	0	0	0	0	1	0	0	0	0	0	0	1	Unique	COG0582_L#	30.1	167	8.7E-41	NA	27.6	137	2.57E-31
3190	WP_033276236.1	manA	mannose-6-phosphate isomerase, class I	0	1	0	1	1	1	1	1	1	1	0	8	Accessory	COG1482_G#	45.6	319	2.34E-86	K01809	45.6	319	6.24E-86

cluster	GI	gene	Annotation	Streptomyces_thermocarboxydus_BPSAC147	Streptomyces_sp._4F	Streptomyces_sp._BSE7F	Streptomyces_sp._ETH9427	Streptomyces_sp._FxanaD5	Streptomyces_sp._UNC401CLCol	Actinospica_acidiphila_NRRL_B_24431	Streptomyces_sp._HNS054	Streptomyces_sp._SMS_SU21	Streptomyces_sp._ZS0098	Streptomyces_thermocarboxydus_K155	Addition	Core-Accessory-Unique	COG	%Similarity_COG	Score_COG	Evalue_COG	KEGG	%Similarity_KEGG	Score_KEGG	Evalue_KEGG
3191	Prokka:JAPCLKMA_04874	-	hypothetical protein	1	1	1	1	1	1	1	1	1	1	1	11	CORE	COG2199_T#	39.5	95	4.2E-19	NA	26.2	80	2.86E-14
3192	WP_122215969.1	-	NADH-quinone oxidoreductase subunit D	1	1	1	1	1	1	1	1	1	1	1	11	CORE	COG0649_C#	39.4	287	5.77E-77	K00333	40.2	275	6.06E-73
3193	OSC72192.1	-	hypothetical protein	0	1	0	0	0	0	0	0	0	0	0	1	Unique								
3194	Prokka:JAPCLKMA_05494	salQ	2-epi-5-epi-valiolone synthase	1	0	0	0	0	0	0	0	0	0	0	1	Unique	COG0337_E#	30	162	3.66E-39	K01735	32.5	161	1.67E-38
3195	OSC67248.1	-	alkene reductase	0	1	0	0	0	0	0	0	0	0	0	1	Unique	COG1902_C#	44.7	299	2.5E-80	K10680	41.6	245	1.15E-63
3196	Prokka:JAPCLKMA_01452	garK	Glycerate 2-kinase	1	1	1	0	1	1	1	1	1	1	1	10	Accessory	COG1929_G#	53	341	3.37E-93	K00865	46.4	305	9.32E-82
3197	Prokka:JAPCLKMA_02326	purR_2	HTH-type transcriptional repressor PurR	1	0	1	1	1	1	1	1	1	1	1	10	Accessory	COG1879_G#	41.9	240	8.09E-63	K02058	33.8	171	2.1E-41
3198	Prokka:JAPCLKMA_01311	-	hypothetical protein	1	0	1	0	0	0	0	0	1	0	0	3	Accessory								
3199	OSC72198.1	-	hypothetical protein	0	1	0	0	0	0	0	0	0	0	0	1	Unique								
3200	WP_122215889.1	-	low temperature requirement protein A	0	0	0	0	0	0	0	0	0	1	0	1	Unique	COG4292_S#	25.3	87	1.14E-16	NA	25.3	87	3.06E-16

cluster	GI	gene	Annotation	Streptomyces_thermocarboxydus_BPSAC147	Streptomyces_sp._4F	Streptomyces_sp._BSE7F	Streptomyces_sp._ETH9427	Streptomyces_sp._FxanaD5	Streptomyces_sp._UNC401CLCol	Actinospica_acidiphila_NRRL_B_24431	Streptomyces_sp._HNS054	Streptomyces_sp._SMS_SU21	Streptomyces_sp._ZS0098	Streptomyces_thermocarboxydus_K155	Addition	Core-Accessory-Unique	COG	%Similarity_COG	Score_COG	Evalue_COG	KEGG	%Similarity_KEGG	Score_KEGG	Evalue_KEGG
3201	WP_028959131.1	mtnA	S-methyl-5-thioribose-1-phosphate isomerase	1	1	1	1	1	1	1	1	1	1	1	11	CORE	COG0182_J#	43.1	256	1.42E-67	K08963	41.1	254	1.88E-66
3202	WP_102637567.1	pdhA	pyruvate dehydrogenase (acetyl-transferring) E1 component subunit alpha	1	1	1	1	1	1	1	1	1	1	1	11	CORE	COG1071_C#	47	304	4.55E-82	K00161	47	304	1.21E-81
3203	WP_109288630.1	-	hypothetical protein	1	1	1	1	1	1	1	1	1	1	1	11	CORE								
3204	WP_019526842.1	-	alcohol dehydrogenase catalytic domain-containing protein	0	0	0	0	1	1	0	0	0	0	1	3	Accessory	COG1063_ER#	33.7	186	1.81E-46	K00001	33.7	186	4.82E-46
3205	OSC72472.1	-	hypothetical protein	0	1	0	0	0	0	0	0	0	0	0	1	Unique	COG0438_M#	36.8	138	5.64E-32	NA	31	173	3.23E-42
3206	Prokka:JAPCLKMA_05194	-	hypothetical protein	1	0	1	1	0	1	0	1	1	1	0	7	Accessory								
3207	Prokka:JAPCLKMA_00627	-	hypothetical protein	1	0	0	0	0	0	1	0	0	0	0	2	Accessory	COG2311_S#	30	138	4.32E-32				
3208	Prokka:CIODPCDJ_06700	-	hypothetical protein	0	0	0	0	0	0	0	0	0	0	1	1	Unique	COG3335_L#	58.9	240	1.38E-62				
3209	AXI91213.1	-	hypothetical protein	0	0	0	1	0	0	0	1	0	0	0	2	Accessory								

cluster	GI	gene	Annotation	Streptomyces_thermocarboxydus_BPSAC147	Streptomyces_sp._4F	Streptomyces_sp._BSE7F	Streptomyces_sp._ETH9427	Streptomyces_sp._FxanaD5	Streptomyces_sp._UNC401CLCol	Actinospica_acidiphila_NRRL_B_24431	Streptomyces_sp._HNS054	Streptomyces_sp._SMS_SU21	Streptomyces_sp._ZS0098	Streptomyces_thermocarboxydus_K155	Addition	Core-Accessory-Unique	COG	%Similarity_COG	Score_COG	Evalue_COG	KEGG	%Similarity_KEGG	Score_KEGG	Evalue_KEGG
3210	OSC73435.1	-	transcriptional regulator	0	1	0	0	0	0	0	0	0	0	0	1	Unique	COG1316_K#	32.8	139	2.53E-32	NA	31.1	137	3.35E-31
3211	Prokka:CIODPCDJ_02499	hpd	4-hydroxyphenylpyruvate dioxygenase	1	1	1	1	1	1	1	1	1	1	1	11	CORE	COG3185_ER#	33.8	191	7.31E-48	K00457	47.5	318	8.11E-86
3212	OSC70715.1	-	galactonate dehydratase	0	1	0	0	0	0	0	0	0	1	0	2	Accessory	COG4948_MR#	53.7	421	4.34E-117	K01684	52.9	419	2.58E-116
3213	Prokka:CIODPCDJ_00221	-	hypothetical protein	0	0	0	0	0	1	0	0	0	0	1	2	Accessory								
3214	WP_019522089.1	-	GAF domain-containing sensor histidine kinase	1	1	1	1	1	1	1	1	1	1	1	11	CORE	COG4585_T#	38.9	163	1.25E-39	NA	38.9	163	3.34E-39
3215	Prokka:JAPCLKMA_03212	sasA_8	Adaptive-response sensory-kinase SasA	1	1	1	1	1	1	1	1	1	1	1	11	CORE	COG0642_T#	38.7	150	1.43E-35	NA	38.7	150	3.82E-35
3216	OSC61818.1	-	septum formation initiator	1	1	1	1	1	1	1	1	1	1	1	11	CORE	COG0489_D#	29.6	53	0.00000238	NA	39	176	3.81E-43
3217	WP_122216255.1	-	aromatic ring-hydroxylating dioxygenase subunit alpha	0	0	1	1	0	0	0	1	1	1	0	5	Accessory	COG4638_PR#	37.7	138	7.34E-32	K00517	37.9	121	1.9E-26

cluster	GI	gene	Annotation	Streptomyces_thermocarboxydus_BPSAC147	Streptomyces_sp._4F	Streptomyces_sp._BSE7F	Streptomyces_sp._ETH9427	Streptomyces_sp._FxanaD5	Streptomyces_sp._UNC401CLCol	Actinospica_acidiphila_NRRL_B_24431	Streptomyces_sp._HNS054	Streptomyces_sp._SMS_SU21	Streptomyces_sp._ZS0098	Streptomyces_thermocarboxydus_K155	Addition	Core-Accessory-Unique	COG	%Similarity_COG	Score_COG	Evalue_COG	KEGG	%Similarity_KEGG	Score_KEGG	Evalue_KEGG
3218	WP_122213422.1	-	SagB/ThcOx family dehydrogenase	0	0	0	1	0	0	1	1	1	1	0	5	Accessory	COG0778_C#	37.7	57	1.65E-07	NA	37.7	57	0.00000044
3219	AXI91146.1	-	site-2 protease family protein	0	0	0	1	0	0	0	1	0	1	0	3	Accessory	COG1994_R#	44.1	205	4.89E-52	NA	43.1	289	4.03E-77
3220	AXI89648.1	-	hypothetical protein	1	0	0	1	0	0	0	0	0	0	0	2	Accessory	COG0791_M#	44.4	96	1.88E-19	NA	44.4	96	5.01E-19
3221	WP_037779552.1	dndA	cysteine desulfurase DndA	0	0	0	0	1	0	0	0	0	0	0	1	Unique	COG1104_E#	48	303	1.32E-81	K04487	48	303	3.52E-81
3222	Prokka:JAPCLKMA_05465	rlmG	Ribosomal RNA large subunit methyltransferase G	1	1	1	1	1	1	1	1	1	1	1	11	CORE	COG2813_J#	49.6	325	3.24E-88	K11391	45.2	302	7.85E-81
3223	WP_084207077.1	-	oxidoreductase	1	1	1	0	1	1	1	1	1	1	1	10	Accessory	COG4447_R#	34.8	170	1.34E-41				
3224	WP_028959350.1	-	hypothetical protein	1	1	1	1	1	1	1	1	1	1	1	11	CORE								
3225	OSC56429.1	-	RNA polymerase subunit sigma-24	0	1	0	0	0	0	0	0	0	0	0	1	Unique	COG4941_K#	42.4	265	2.34E-70				
3226	Prokka:CIODPCDJ_01462	-	hypothetical protein	1	1	1	1	1	1	1	1	1	1	1	11	CORE	COG1228_Q#	56.1	402	1.6E-111	NA	56.1	402	4.27E-111
3227	OSC62115.1	-	transaldolase	1	1	1	1	1	1	1	1	1	1	1	11	CORE	COG0176_G#	59.9	419	1.65E-116	K00616	59.6	417	1.28E-115
3228	WP_086020128.1	-	pentapeptide repeat-containing protein	1	0	0	0	1	1	1	0	0	0	1	5	Accessory	COG1357_S#	49.7	110	1.64E-23	NA	41.3	93	4.24E-18
3229	Prokka:JAPCLKMA_01625	-	hypothetical protein	1	0	0	0	0	0	0	0	0	0	0	1	Unique								

cluster	GI	gene	Annotation	Streptomyces_thermocarboxydus_BPSAC147	Streptomyces_sp._4F	Streptomyces_sp._BSE7F	Streptomyces_sp._ETH9427	Streptomyces_sp._FxanaD5	Streptomyces_sp._UNC401CLCol	Actinospica_acidiphila_NRRL_B_24431	Streptomyces_sp._HNS054	Streptomyces_sp._SMS_SU21	Streptomyces_sp._ZS0098	Streptomyces_thermocarboxydus_K155	Addition	Core-Accessory-Unique	COG	%Similarity_COG	Score_COG	Evalue_COG	KEGG	%Similarity_KEGG	Score_KEGG	Evalue_KEGG
3230	AXI85269.1	-	hypothetical protein	0	0	0	1	0	0	0	0	0	0	0	1	Unique								
3231	AXI85735.1	-	carbamoyl-phosphate synthase small subunit	1	1	0	1	1	1	1	1	1	1	1	10	Accessory	COG0505_EF#	63.1	473	7.35E-133	K01956	63.1	473	1.96E-132
3232	WP_033272958.1	-	glycosyltransferase family 4 protein	1	1	1	1	1	1	1	1	1	1	1	11	CORE	COG0438_M#	52.6	379	1.45E-104	K13668	52.6	379	3.86E-104
3233	Prokka:CIODPCDJ_03195	-	hypothetical protein	1	0	1	1	1	0	1	0	0	1	1	7	Accessory								
3234	Prokka:CIODPCDJ_06631	hypD	Hydrogenase maturation factor HypD	1	0	0	1	1	1	0	1	0	1	1	7	Accessory	COG0409_O#	57.3	440	5.28E-123	K04654	57.3	440	1.41E-122
3235	OSC69069.1	-	hypothetical protein	1	1	1	1	1	1	1	1	1	1	1	11	CORE	COG2899_S#	50	354	4.98E-97	K09799	50	354	1.33E-96
3236	WP_102640919.1	-	class E sortase	1	1	1	1	1	1	1	1	1	1	1	11	CORE								
3237	WP_051724671.1	-	two-component sensor histidine kinase	0	0	0	0	0	0	1	0	0	0	0	1	Unique	COG4585_T#	33.2	129	3.4E-29	K07778	33.2	129	9.09E-29
3238	WP_102638412.1	-	DUF418 domain-containing protein	0	1	0	0	0	0	0	0	1	0	0	2	Accessory								
3239	WP_028960992.1	-	branched-chain amino acid ABC transporter permease	1	1	1	1	1	1	1	1	1	1	1	11	CORE	COG4177_E#	35.5	176	1.86E-43	K01998	30.9	131	1.4E-29

cluster	GI	gene	Annotation	Streptomyces_thermocarboxydus_BPSAC147	Streptomyces_sp._4F	Streptomyces_sp._BSE7F	Streptomyces_sp._ETH9427	Streptomyces_sp._FxanaD5	Streptomyces_sp._UNC401CLCol	Actinospica_acidiphila_NRRL_B_24431	Streptomyces_sp._HNS054	Streptomyces_sp._SMS_SU21	Streptomyces_sp._ZS0098	Streptomyces_thermocarboxydus_K155	Addition	Core-Accessory-Unique	COG	%Similarity_COG	Score_COG	Evalue_COG	KEGG	%Similarity_KEGG	Score_KEGG	Evalue_KEGG
3240	WP_107685382.1	-	2Fe-2S iron-sulfur cluster binding domain-containing protein	0	1	1	1	0	0	1	1	1	1	0	7	Accessory	COG1018_C#	36.3	205	2.86E-52	NA	36.3	205	7.64E-52
3241	WP_019523089.1	-	hypothetical protein	1	0	1	1	1	1	1	1	1	1	1	10	Accessory	COG3424_Q#	52.3	360	6.95E-99	K16167	52.3	360	1.86E-98
3242	OSC73157.1	-	acyltransferase	0	1	0	0	0	0	0	0	0	0	0	1	Unique	COG1835_I#	30.9	124	6.42E-28				
3243	WP_157842170.1	-	hypothetical protein	0	0	0	0	0	0	1	0	0	0	0	1	Unique	COG4585_T#	29.9	91	6.02E-18	NA	34.9	84	2.57E-15
3244	OSC62938.1	-	aspartate aminotransferase	1	1	1	1	1	1	1	1	1	1	1	11	CORE	COG0075_E#	42.2	260	7.49E-69	NA	29.9	154	2.64E-36
3245	OSC69235.1	-	monooxygenase	0	1	0	0	0	0	0	0	0	0	0	1	Unique	COG0654_HC#	37.7	233	9.81E-61	NA	37.7	233	2.62E-60
3246	WP_026244252.1	-	AAA family ATPase	0	0	0	0	1	0	0	0	0	0	0	1	Unique	COG0464_O#	30.9	135	4.75E-31	K13525	28.4	111	1.5E-23
3247	OSC61753.1	-	adenylate/guanylate cyclase domain-containing protein	1	1	1	1	1	1	1	1	1	1	1	11	CORE	COG2114_T#	32.6	127	1.29E-28	K01768	32.6	127	3.45E-28
3248	WP_109288598.1	-	XdhC family protein	0	1	1	1	1	1	1	1	1	1	1	10	Accessory	COG1975_O#	55.8	355	2.23E-97	K07402	55.8	355	5.95E-97
3249	WP_019521531.1	-	acyl-CoA/acyl-ACP dehydrogenase	1	0	0	0	1	1	1	0	0	0	1	5	Accessory	COG1960_I#	37	239	2.33E-62	NA	36.7	228	8.4E-59
3250	WP_102641911.1	-	hypothetical protein	1	1	1	1	1	1	1	1	1	1	1	11	CORE								

cluster	GI	gene	Annotation	Streptomyces_thermocarboxydus_BPSAC147	Streptomyces_sp._4F	Streptomyces_sp._BSE7F	Streptomyces_sp._ETH9427	Streptomyces_sp._FxanaD5	Streptomyces_sp._UNC401CLCol	Actinospica_acidiphila_NRRL_B_24431	Streptomyces_sp._HNS054	Streptomyces_sp._SMS_SU21	Streptomyces_sp._ZS0098	Streptomyces_thermocarboxydus_K155	Addition	Core-Accessory-Unique	COG	%Similarity_COG	Score_COG	Evalue_COG	KEGG	%Similarity_KEGG	Score_KEGG	Evalue_KEGG
3251	Prokka:CIODPCDJ_06755	-	hypothetical protein	0	0	0	0	1	0	0	0	0	0	1	2	Accessory								
3252	WP_048460288.1	dnaJ	molecular chaperone DnaJ	1	1	1	1	1	1	1	1	1	1	1	11	CORE	COG0484_O#	55.2	429	9.3E-120	K03686	55.2	429	2.48E-119
3253	WP_102638812.1	-	glycosyltransferase family 4 protein	0	0	1	1	0	0	1	1	1	1	0	6	Accessory	COG0438_M#	31.6	176	1.42E-43	NA	31.6	176	3.79E-43
3254	WP_051724503.1	-	sensor histidine kinase	0	0	1	1	0	0	1	1	1	1	0	6	Accessory	COG4585_T#	30.4	108	6.2E-23	NA	30.5	105	1.07E-21
3255	OSC56992.1	-	Rossmann fold nucleotide-binding protein	0	1	0	0	0	0	0	0	0	0	0	1	Unique	COG1611_R#	44.9	55	4.77E-07	K06966	44.9	55	0.00000127
3256	WP_102637708.1	-	helix-turn-helix domain-containing protein	0	1	1	1	0	0	0	1	1	1	0	6	Accessory	COG2508_TQ#	33.9	47	0.00017	NA	33.9	47	0.000453
3257	OSC68484.1	-	mandelate racemase	0	1	0	0	0	0	1	0	0	0	0	2	Accessory	COG4948_MR#	37	180	9.83E-45	K08323	33.8	73	4.52E-12
3258	WP_019526406.1	urtC	urea ABC transporter permease subunit UrtC	1	0	1	1	1	1	1	1	1	1	1	10	Accessory	COG4177_E#	53.8	366	9.67E-101	K11961	46.1	292	6.19E-78
3259	WP_037779546.1	dndB	DNA sulfur modification protein DndB	0	0	0	0	1	0	0	0	0	0	0	1	Unique								

cluster	GI	gene	Annotation	Streptomyces_thermocarboxydus_BPSAC147	Streptomyces_sp._4F	Streptomyces_sp._BSE7F	Streptomyces_sp._ETH9427	Streptomyces_sp._FxanaD5	Streptomyces_sp._UNC401CLCol	Actinospica_acidiphila_NRRL_B_24431	Streptomyces_sp._HNS054	Streptomyces_sp._SMS_SU21	Streptomyces_sp._ZS0098	Streptomyces_thermocarboxydus_K155	Addition	Core-Accessory-Unique	COG	%Similarity_COG	Score_COG	Evalue_COG	KEGG	%Similarity_KEGG	Score_KEGG	Evalue_KEGG
3260	OSC70604.1	-	c-type cytochrome biogenesis protein CcsB	1	1	1	1	1	1	1	1	1	1	1	11	CORE	COG0755_O#	44.9	212	3.05E-54	NA	44.9	212	8.14E-54
3261	WP_078492773.1	-	ParA family protein	1	1	1	1	1	1	1	1	1	1	1	11	CORE	COG1192_D#	65.3	381	3.78E-105	K03496	65.3	381	1.01E-104
3262	Prokka:CIODPCDJ_00530	potA_1	Spermidine/putrescine import ATP-binding protein PotA	0	0	0	0	1	1	0	0	0	0	1	3	Accessory	COG3839_G#	46.8	272	3.23E-72	NA	46.8	272	8.63E-72
3263	WP_102640227.1	-	aminoglycoside phosphotransferase family protein	1	1	1	1	1	1	1	1	1	1	1	11	CORE								
3264	WP_019522425.1	efeO	iron uptake system protein EfeO	1	0	0	0	1	1	0	0	0	0	1	4	Accessory	COG2822_P#	38.1	248	2.93E-65	K07224	38.1	248	7.83E-65
3265	WP_019523573.1	-	FAD-dependent monooxygenase	1	0	0	0	1	1	1	0	0	0	1	5	Accessory	COG0654_HC#	34.7	67	1.58E-10	NA	60	50	0.0000313
3266	WP_028960389.1	-	hypothetical protein	1	0	1	1	1	1	1	1	1	1	1	10	Accessory								
3267	WP_086020246.1	-	polyprenyl synthetase family protein	1	1	1	1	1	1	1	1	1	1	1	11	CORE	COG0142_H#	48.9	325	2.46E-88	K13787	48.9	325	6.58E-88

cluster	GI	gene	Annotation	Streptomyces_thermocarboxydus_BPSAC147	Streptomyces_sp._4F	Streptomyces_sp._BSE7F	Streptomyces_sp._ETH9427	Streptomyces_sp._FxanaD5	Streptomyces_sp._UNC401CLCol	Actinospica_acidiphila_NRRL_B_24431	Streptomyces_sp._HNS054	Streptomyces_sp._SMS_SU21	Streptomyces_sp._ZS0098	Streptomyces_thermocarboxydus_K155	Addition	Core-Accessory-Unique	COG	%Similarity_COG	Score_COG	Evalue_COG	KEGG	%Similarity_KEGG	Score_KEGG	Evalue_KEGG
3268	WP_019527461.1	-	NUDIX hydrolase	0	0	0	0	1	0	0	0	0	0	0	1	Unique	COG1051_F#	43.1	53	0.00000181	K13988	36.6	44	0.00382
3269	OSC69337.1	-	polyprenyl synthetase	1	1	1	1	1	1	1	1	1	1	1	11	CORE	COG0142_H#	50.6	295	3.57E-79	K13787	50.6	294	1.62E-78
3270	WP_051724459.1	-	mandelate racemase	0	0	0	1	0	0	1	1	0	0	0	3	Accessory	COG4948_MR#	31.5	175	4.12E-43	NA	28.7	75	9.08E-13
3271	Prokka:JAPCLKMA_04881	-	hypothetical protein	1	0	0	0	0	0	0	0	0	0	0	1	Unique								
3272	WP_146217800.1	-	pentapeptide repeat-containing protein	0	0	0	0	0	0	0	0	1	0	0	1	Unique	COG1357_S#	25.8	95	5.42E-19	NA	28.4	62	1.36E-08
3273	OSC58830.1	-	sugar ABC transporter ATP-binding protein	1	1	1	1	1	1	1	1	1	1	1	11	CORE	COG3839_G#	61.4	441	2.36E-123	K10112	61.4	441	6.3E-123
3274	WP_019526038.1	-	5-amino-6-(5-phosphoribosylamino)uracil reductase	1	1	1	1	1	1	1	1	1	1	1	11	CORE	COG1985_H#	36.7	123	1.42E-27	K11752	27.1	75	9.06E-13
3275	AXI85261.1	-	IS3 family transposase	1	0	0	1	0	0	0	0	0	1	1	4	Accessory	COG2801_L#	35.3	148	4.12E-35	NA	33.5	137	2.53E-31
3276	Prokka:CIODPCDJ_05199	-	hypothetical protein	0	0	1	1	0	1	0	1	1	1	1	7	Accessory								
3277	OSC71175.1	-	sodium:proton antiporter	1	1	1	1	1	1	1	1	1	1	1	11	CORE	COG0489_D#	68.2	495	1.79E-139	K03593	68.2	495	4.79E-139
3278	WP_078492963.1	-	zinc-dependent metalloprotease	1	1	1	1	1	1	1	1	1	1	1	11	CORE	COG5282_S#	41.7	257	1.07E-67	NA	41.7	257	2.87E-67

cluster	GI	gene	Annotation	Streptomyces_thermocarboxydus_BPSAC147	Streptomyces_sp._4F	Streptomyces_sp._BSE7F	Streptomyces_sp._ETH9427	Streptomyces_sp._FxanaD5	Streptomyces_sp._UNC401CLCol	Actinospica_acidiphila_NRRL_B_24431	Streptomyces_sp._HNS054	Streptomyces_sp._SMS_SU21	Streptomyces_sp._ZS0098	Streptomyces_thermocarboxydus_K155	Addition	Core-Accessory-Unique	COG	%Similarity_COG	Score_COG	Evalue_COG	KEGG	%Similarity_KEGG	Score_KEGG	Evalue_KEGG
3279	WP_078611416.1	fabH	beta-ketoacyl-ACP synthase III	1	0	0	0	1	1	0	0	0	0	1	4	Accessory	COG0332_I#	42.6	249	2.24E-65	K11608	42.6	249	5.98E-65
3280	WP_086020115.1	-	RNA polymerase sigma factor SigF	1	1	1	1	1	1	1	1	1	1	1	11	CORE	COG1191_K#	54.4	262	2.55E-69	K03090	54.4	262	6.82E-69
3281	PWE10946.1	-	NAD-dependent dehydratase	0	0	1	1	0	0	0	1	1	1	0	5	Accessory	COG0451_MG#	26.5	62	2.97E-09				
3282	OSC56956.1	-	glycosyl transferase	0	1	0	0	0	0	0	0	0	0	0	1	Unique	COG1215_M#	32.6	171	4.54E-42				
3283	PWE07660.1	-	alkanal monooxygenase	1	1	1	1	1	1	1	1	1	1	1	11	CORE	COG2141_C#	49.4	313	1.26E-84	NA	49.4	313	3.37E-84
3284	Prokka:JAPCLKMA_05940	-	hypothetical protein	1	0	1	1	0	1	1	1	1	0	0	7	Accessory								
3285	WP_122214391.1	-	sugar ABC transporter substrate-binding protein	1	1	1	1	1	1	1	1	1	1	1	11	CORE	COG4213_G#	35.5	192	2.49E-48	K10439	25.9	60	3.01E-08
3286	WP_122213574.1	-	DGQHR domain-containing protein	0	0	0	0	0	0	0	0	0	1	0	1	Unique								
3287	OSC69588.1	-	peptidase	0	1	0	0	1	1	1	0	0	0	0	4	Accessory	COG0739_M#	49.2	121	9.19E-27	NA	33.3	119	7.14E-26
3288	WP_048460141.1	-	ATP-grasp domain protein	1	1	0	0	1	1	1	1	1	1	1	9	Accessory					NA	30	118	1.59E-25

cluster	GI	gene	Annotation	Streptomyces_thermocarboxydus_BPSAC147	Streptomyces_sp._4F	Streptomyces_sp._BSE7F	Streptomyces_sp._ETH9427	Streptomyces_sp._FxanaD5	Streptomyces_sp._UNC401CLCol	Actinospica_acidiphila_NRRL_B_24431	Streptomyces_sp._HNS054	Streptomyces_sp._SMS_SU21	Streptomyces_sp._ZS0098	Streptomyces_thermocarboxydus_K155	Addition	Core-Accessory-Unique	COG	%Similarity_COG	Score_COG	Evalue_COG	KEGG	%Similarity_KEGG	Score_KEGG	Evalue_KEGG
3289	OSC61371.1	-	type I polyketide synthase	0	1	0	0	0	0	0	0	0	0	0	1	Unique	COG3320_Q#	29.1	94	1.2E-18	NA	32.6	77	3.11E-13
3290	WP_052183383.1	-	helix-turn-helix transcriptional regulator	0	0	0	0	0	1	0	0	0	0	1	2	Accessory	COG2771_K#	27.4	78	5.22E-14	NA	26.4	56	5.67E-07
3291	Prokka:JAPCLKMA_05927	-	hypothetical protein	1	1	1	1	1	1	1	1	1	1	1	11	CORE								
3292	AXI88882.1	-	tRNA 2-thiouridine(34) synthase MnmA	1	1	1	1	1	1	1	1	1	1	1	11	CORE	COG0482_J#	57.4	404	3.18E-112	K00566	57.4	404	8.49E-112
3293	AXI87380.1	-	DNA polymerase III subunit beta	1	1	1	1	1	1	1	1	1	1	1	11	CORE	COG0592_L#	50.5	378	3.19E-104	K02338	50.5	378	8.51E-104
3294	WP_102639904.1	-	restriction endonuclease	0	0	0	0	0	0	0	0	1	0	0	1	Unique	COG1715_V#	32.8	67	1.57E-10	K07448	32.8	67	4.2E-10
3295	PWE07261.1	-	DUF3696 domain-containing protein	1	0	1	0	0	0	0	0	0	0	0	2	Accessory	COG4938_S#	34.3	204	8.23E-52				
3296	WP_019524948.1	-	AAA family ATPase	0	0	0	0	1	0	0	0	0	0	0	1	Unique								
3297	WP_033277718.1	-	epoxide hydrolase	0	0	0	0	1	1	1	0	0	0	1	4	Accessory	COG0596_R#	42	312	2.15E-84	NA	39.4	300	1.73E-80
3298	OSC71059.1	-	amidinotransferase	0	1	0	0	0	0	0	0	0	0	0	1	Unique					NA	44.1	316	3.97E-85

cluster	GI	gene	Annotation	Streptomyces_thermocarboxydus_BPSAC147	Streptomyces_sp._4F	Streptomyces_sp._BSE7F	Streptomyces_sp._ETH9427	Streptomyces_sp._FxanaD5	Streptomyces_sp._UNC401CLCol	Actinospica_acidiphila_NRRL_B_24431	Streptomyces_sp._HNS054	Streptomyces_sp._SMS_SU21	Streptomyces_sp._ZS0098	Streptomyces_thermocarboxydus_K155	Addition	Core-Accessory-Unique	COG	%Similarity_COG	Score_COG	Evalue_COG	KEGG	%Similarity_KEGG	Score_KEGG	Evalue_KEGG
3299	OSC71866.1	-	2-polyprenyl-6-methoxyphenol hydroxylase	0	1	0	0	0	0	0	0	0	0	0	1	Unique	COG0654_HC#	30.7	153	1.28E-36	NA	30.7	153	3.41E-36
3300	WP_048460840.1	-	Glu/Leu/Phe/Val dehydrogenase	0	0	0	1	0	0	0	1	0	0	0	2	Accessory	COG0334_E#	32.5	131	6.78E-30	K00261	33.3	123	3.78E-27
3301	WP_028961581.1	-	ABC transporter substrate-binding protein	1	1	1	1	1	1	1	1	1	1	1	11	CORE	COG0715_P#	34.7	202	3.13E-51	K02051	34.7	202	8.36E-51
3302	AXI91415.1	-	hypothetical protein	0	0	0	1	0	0	0	0	0	0	0	1	Unique								
3303	OSC73242.1	-	phosphatidate cytidylyltransferase	1	1	1	1	1	1	1	1	1	1	1	11	CORE	COG0575_I#	39.8	215	2.73E-55	K00981	39.8	215	7.3E-55
3304	OSC71911.1	-	allantoicase	1	1	1	1	1	1	1	1	1	1	1	11	CORE	COG4266_F#	41.5	239	2.31E-62	K01477	33.2	191	1.47E-47
3305	WP_019526358.1	-	ferrochelatase	1	1	1	1	1	1	1	1	1	1	1	11	CORE	COG0276_H#	45.7	305	3.42E-82	K01772	44.9	276	4.54E-73
3306	WP_028959681.1	-	membrane dipeptidase	1	1	1	1	1	1	1	1	1	1	1	11	CORE	COG2355_E#	31.9	152	3.7E-36	K01273	40.3	214	2.12E-54
3307	WP_028960726.1	-	alpha/beta hydrolase	1	0	1	1	1	1	1	1	1	1	1	10	Accessory	COG1073_R#	28.6	137	1.23E-31	K06889	28.6	137	3.29E-31
3308	Prokka:JAPCLKMA_01695	-	NADH oxidase	1	1	1	1	1	1	1	1	1	1	1	11	CORE	COG1902_C#	58.4	431	2.42E-120	K10680	31.3	153	3.4E-36
3309	Prokka:CIODPCDJ_00553	-	hypothetical protein	1	1	1	1	1	1	1	1	1	1	1	11	CORE	COG3239_I#	29.9	51	0.0000089	NA	27.2	117	2.07E-25

cluster	GI	gene	Annotation	Streptomyces_thermocarboxydus_BPSAC147	Streptomyces_sp._4F	Streptomyces_sp._BSE7F	Streptomyces_sp._ETH9427	Streptomyces_sp._FxanaD5	Streptomyces_sp._UNC401CLCol	Actinospica_acidiphila_NRRL_B_24431	Streptomyces_sp._HNS054	Streptomyces_sp._SMS_SU21	Streptomyces_sp._ZS0098	Streptomyces_thermocarboxydus_K155	Addition	Core-Accessory-Unique	COG	%Similarity_COG	Score_COG	Evalue_COG	KEGG	%Similarity_KEGG	Score_KEGG	Evalue_KEGG
3310	PWE09983.1	-	peptidase M24 family protein	1	1	1	1	1	1	1	1	1	1	1	11	CORE	COG0006_E#	47.3	319	1.75E-86	K01274	47.3	319	4.68E-86
3311	WP_033273631.1	-	EcsC family protein	0	0	0	0	0	0	1	0	0	0	0	1	Unique					NA	26.6	64	2.08E-09
3312	Prokka:CIODPCDJ_05108	amiC_2	Aliphatic amidase expression-regulating protein	1	0	0	1	1	1	1	1	1	1	1	9	Accessory	COG0683_E#	28.7	148	6.98E-35	K11959	29.1	143	6E-33
3313	WP_102641245.1	-	sensor histidine kinase	1	0	1	1	1	1	1	1	1	1	1	10	Accessory	COG4585_T#	26.4	109	2.11E-23	NA	26.4	109	5.63E-23
3314	Prokka:CIODPCDJ_06740	casC	CRISPR system Cascade subunit CasC	0	0	0	0	1	0	0	0	0	0	1	2	Accessory					K19124	33.1	138	1.48E-31
3315	WP_033276964.1	-	8-amino-7-oxononanoate synthase	1	1	1	1	1	1	1	1	1	1	1	11	CORE	COG0156_H#	58.6	389	1.38E-107	K00652	58.6	389	3.68E-107
3316	PWE09872.1	-	acyl-CoA dehydrogenase	1	1	1	1	1	1	1	1	1	1	1	11	CORE	COG1960_I#	47.9	283	8.16E-76	K00249	34.5	205	7.54E-52
3317	Prokka:CIODPCDJ_01853	degA_3	HTH-type transcriptional regulator DegA	1	1	1	1	1	1	1	1	1	1	1	11	CORE	COG1609_K#	31.3	181	7.44E-45	NA	31.3	181	1.99E-44
3318	OSC72789.1	-	protein translocase subunit SecF	0	1	1	1	1	1	1	1	1	1	0	9	Accessory	COG0341_U#	38.4	248	3.8E-65	K03074	38.4	248	1.32E-64

cluster	GI	gene	Annotation	Streptomyces_thermocarboxydus_BPSAC147	Streptomyces_sp._4F	Streptomyces_sp._BSE7F	Streptomyces_sp._ETH9427	Streptomyces_sp._FxanaD5	Streptomyces_sp._UNC401CLCol	Actinospica_acidiphila_NRRL_B_24431	Streptomyces_sp._HNS054	Streptomyces_sp._SMS_SU21	Streptomyces_sp._ZS0098	Streptomyces_thermocarboxydus_K155	Addition	Core-Accessory-Unique	COG	%Similarity_COG	Score_COG	Evalue_COG	KEGG	%Similarity_KEGG	Score_KEGG	Evalue_KEGG
3319	WP_122213810.1	-	conjugal transfer protein	0	0	0	0	0	0	0	0	0	1	0	1	Unique								
3320	WP_028959091.1	-	ABC transporter permease	0	0	0	0	1	1	1	1	0	0	1	5	Accessory	COG0577_V#	27	143	1.32E-33	K02004	31.6	164	2.52E-39
3321	OSC61174.1	-	magnesium transporter CorA	1	1	1	1	1	1	1	1	1	1	1	11	CORE	COG0598_P#	50.1	352	2.44E-96	K03284	50.1	352	6.51E-96
3322	WP_033274665.1	-	epoxide hydrolase	1	0	0	0	1	1	1	0	0	0	1	5	Accessory	COG0596_R#	37.9	254	6.92E-67	NA	34.3	252	5.38E-66
3323	Prokka:CIODPCDJ_03077	pstS3	Phosphate-binding protein PstS3	1	1	1	1	1	1	1	1	1	1	1	11	CORE	COG0226_P#	43.2	281	5.29E-75	K02040	43.1	281	1.41E-74
3324	WP_033277704.1	-	hypothetical protein	0	0	0	0	0	0	1	0	0	0	0	1	Unique	COG1960_I#	31.6	157	1.15E-37	K16047	30.8	143	4.59E-33
3325	PWE10291.1	-	ATPase	1	1	1	1	1	1	1	1	1	1	1	11	CORE	COG0714_R#	40.7	247	1.1E-64	NA	41.8	274	1.72E-72
3326	Prokka:JAPCLKMA_05421	ald_3	Alanine dehydrogenase	1	1	1	1	1	1	1	1	1	1	1	11	CORE	COG0686_E#	67.4	490	7.47E-138	K00259	67.1	486	1.69E-136
3327	Prokka:JAPCLKMA_00108	-	hypothetical protein	1	1	0	1	1	1	1	1	1	1	1	10	Accessory	COG0668_M#	26.9	96	1.84E-19	NA	23.6	66	5.45E-10
3328	PWE08706.1	-	DNA integrity scanning protein DisA	1	1	1	1	1	1	1	1	1	1	1	11	CORE	COG1623_R#	60	403	9.2E-112	K07067	60	403	2.46E-111
3329	OSC71893.1	-	sugar ABC transporter permease	1	1	1	1	1	1	1	1	1	1	1	11	CORE	COG4603_R#	36.5	198	5.87E-50	K02057	33.9	175	8.33E-43
3330	WP_051724608.1	-	hypothetical protein	0	0	0	0	0	0	1	0	0	0	0	1	Unique								

cluster	GI	gene	Annotation	Streptomyces_thermocarboxydus_BPSAC147	Streptomyces_sp._4F	Streptomyces_sp._BSE7F	Streptomyces_sp._ETH9427	Streptomyces_sp._FxanaD5	Streptomyces_sp._UNC401CLCol	Actinospica_acidiphila_NRRL_B_24431	Streptomyces_sp._HNS054	Streptomyces_sp._SMS_SU21	Streptomyces_sp._ZS0098	Streptomyces_thermocarboxydus_K155	Addition	Core-Accessory-Unique	COG	%Similarity_COG	Score_COG	Evalue_COG	KEGG	%Similarity_KEGG	Score_KEGG	Evalue_KEGG
3331	WP_102641401.1	-	serine hydrolase	1	0	1	0	0	0	1	1	1	0	1	6	Accessory	COG1680_V#	29.1	107	1.36E-22	NA	27.6	102	8.96E-21
3332	Prokka:CIODPCDJ_04260	-	hypothetical protein	0	0	0	0	0	0	0	0	0	0	1	1	Unique								
3333	WP_019527920.1	-	GuaB3 family IMP dehydrogenase-related protein	1	1	1	1	1	1	1	1	1	1	1	11	CORE	COG0516_F#	53.1	382	2.19E-105	K00088	53.1	382	5.86E-105
3334	Prokka:CIODPCDJ_05418	-	hypothetical protein	1	0	1	1	1	1	1	1	1	1	1	10	Accessory								
3335	Prokka:CIODPCDJ_00647	ccpA_4	Catabolite control protein A	1	1	1	1	1	1	1	1	1	1	1	11	CORE	COG1609_K#	36.1	207	9.68E-53	NA	31.1	153	4.43E-36
3336	WP_102640597.1	-	AAA family ATPase	0	0	0	0	0	0	0	0	1	0	0	1	Unique	COG4637_R#	40.3	233	1.26E-60				
3337	OSC73259.1	-	FAD-dependent oxidoreductase	0	1	0	0	0	0	0	0	0	0	0	1	Unique	COG0665_E#	24.5	74	7.49E-13				
3338	WP_033273925.1	rsgA	ribosome small subunit-dependent GTPase A	1	1	1	1	1	1	1	1	1	1	1	11	CORE	COG1162_R#	39.3	245	3.21E-64	K06949	35.5	196	3.49E-49
3339	OSC64377.1	-	lanthionine synthetase	0	1	0	0	0	0	0	0	0	0	0	1	Unique								
3340	OSC70180.1	-	cytochrome	0	1	0	0	0	0	0	0	0	0	0	1	Unique	COG2124_Q#	35.4	194	6.49E-49	K15468	32.1	198	1.57E-49
3341	Prokka:JAPCLKMA_03172	-	hypothetical protein	1	0	0	0	0	1	1	0	0	1	0	4	Accessory	COG0515_RTKL#	30.2	53	0.00000179	NA	28.8	86	8.68E-16

cluster	GI	gene	Annotation	Streptomyces_thermocarboxydus_BPSAC147	Streptomyces_sp._4F	Streptomyces_sp._BSE7F	Streptomyces_sp._ETH9427	Streptomyces_sp._FxanaD5	Streptomyces_sp._UNC401CLCol	Actinospica_acidiphila_NRRL_B_24431	Streptomyces_sp._HNS054	Streptomyces_sp._SMS_SU21	Streptomyces_sp._ZS0098	Streptomyces_thermocarboxydus_K155	Addition	Core-Accessory-Unique	COG	%Similarity_COG	Score_COG	Evalue_COG	KEGG	%Similarity_KEGG	Score_KEGG	Evalue_KEGG
3342	WP_106959516.1	-	adenosine deaminase	1	1	0	1	1	1	1	1	1	1	1	10	Accessory	COG1816_F#	35.1	182	3.33E-45	K01488	30.8	154	2.6E-36
3343	OSC67600.1	-	short-chain dehydrogenase	0	1	0	1	0	0	1	1	0	1	0	5	Accessory	COG1028_IQR#	33.3	148	5.33E-35	NA	31.3	173	5.4E-42
3344	OSC72197.1	-	hypothetical protein	0	1	0	0	0	0	0	0	0	0	0	1	Unique	COG1921_E#	38.5	253	1.54E-66				
3345	WP_048457227.1	femX	peptidoglycan bridge formation glycyltransferase FemX	1	1	1	1	1	1	1	1	1	1	1	11	CORE	COG2348_V#	27.5	74	1.27E-12				
3346	AXI84575.1	-	sigma-70 family RNA polymerase sigma factor	0	0	0	1	0	0	0	1	0	0	0	2	Accessory	COG1595_K#	30.5	79	2.32E-14				
3347	PWE11582.1	-	endoglucanase	1	1	1	1	1	1	1	1	1	1	1	11	CORE	COG5297_G#	38.7	197	7.65E-50	K01179	38.7	197	2.04E-49
3348	OSC56030.1	-	4-carboxymuconolactone decarboxylase	1	1	0	0	1	1	1	0	0	0	1	6	Accessory	COG0596_R#	41.2	159	2.31E-38	K01607	43.2	93	4.15E-18
3349	WP_033273243.1	recF	DNA replication/repair protein RecF	1	1	1	1	1	1	1	1	1	1	1	11	CORE	COG1195_L#	56	382	1.28E-105	K03629	56	382	3.43E-105
3350	Prokka:CIODPCDJ_05379	-	hypothetical protein	1	0	0	0	1	1	1	0	1	1	1	7	Accessory								

cluster	GI	gene	Annotation	Streptomyces_thermocarboxydus_BPSAC147	Streptomyces_sp._4F	Streptomyces_sp._BSE7F	Streptomyces_sp._ETH9427	Streptomyces_sp._FxanaD5	Streptomyces_sp._UNC401CLCol	Actinospica_acidiphila_NRRL_B_24431	Streptomyces_sp._HNS054	Streptomyces_sp._SMS_SU21	Streptomyces_sp._ZS0098	Streptomyces_thermocarboxydus_K155	Addition	Core-Accessory-Unique	COG	%Similarity_COG	Score_COG	Evalue_COG	KEGG	%Similarity_KEGG	Score_KEGG	Evalue_KEGG
3351	AXI89614.1	-	adenosylhomocysteinase	0	0	0	1	0	0	0	0	0	0	0	1	Unique	COG0499_H#	27	86	3.24E-16	K01251	29.2	82	1.25E-14
3352	Prokka:CIODPCDJ_05239	-	hypothetical protein	0	0	0	1	1	1	1	1	0	1	1	7	Accessory								
3353	OSC65058.1	-	peptide ABC transporter ATP-binding protein	1	1	1	1	1	1	1	1	1	1	1	11	CORE	COG4608_E#	55.8	376	1.2E-103	K10823	55.8	376	3.21E-103
3354	OSC70304.1	-	hypothetical protein	1	1	1	1	1	1	1	1	1	1	1	11	CORE	COG0330_O#	44.7	301	3.76E-81	NA	44.7	301	1E-80
3355	Prokka:JAPCLKMA_00285	pucA_1	putative xanthine dehydrogenase subunit A	1	0	0	0	1	1	0	0	0	0	1	4	Accessory	COG1975_O#	32.2	155	3.33E-37	K07402	32.2	155	8.9E-37
3356	Prokka:CIODPCDJ_02119	prtS	Protease PrtS	1	1	1	1	1	1	1	1	1	1	1	11	CORE	COG3227_E#	52.9	360	8.91E-99	NA	47.1	210	2.33E-53
3357	OSC68377.1	-	NAD-dependent dehydratase	1	1	1	1	1	1	1	1	1	1	1	11	CORE	COG0451_MG#	29.8	47	0.000128				
3358	WP_028961825.1	pheS	phenylalanine--tRNA ligase subunit alpha	1	1	1	1	1	1	1	1	1	1	1	11	CORE	COG0016_J#	58.4	402	1.56E-111	K01889	58.4	402	4.18E-111
3359	WP_102638387.1	-	two-component sensor histidine kinase	0	1	0	0	0	0	0	0	1	0	0	2	Accessory	COG4585_T#	27.5	88	5E-17	NA	27.5	88	1.34E-16

cluster	GI	gene	Annotation	Streptomyces_thermocarboxydus_BPSAC147	Streptomyces_sp._4F	Streptomyces_sp._BSE7F	Streptomyces_sp._ETH9427	Streptomyces_sp._FxanaD5	Streptomyces_sp._UNC401CLCol	Actinospica_acidiphila_NRRL_B_24431	Streptomyces_sp._HNS054	Streptomyces_sp._SMS_SU21	Streptomyces_sp._ZS0098	Streptomyces_thermocarboxydus_K155	Addition	Core-Accessory-Unique	COG	%Similarity_COG	Score_COG	Evalue_COG	KEGG	%Similarity_KEGG	Score_KEGG	Evalue_KEGG
3360	Prokka:JAPCLKMA_04877	-	hypothetical protein	1	0	0	0	0	0	0	0	0	0	0	1	Unique								
3361	OSC71409.1	-	two-component sensor histidine kinase	0	1	0	0	0	0	0	0	0	0	0	1	Unique	COG4585_T#	31.9	109	2.74E-23	K07778	31.9	109	7.31E-23
3362	PWE09137.1	-	hypothetical protein	1	1	1	1	1	1	1	1	1	1	1	11	CORE								
3363	WP_102638729.1	gcvT	glycine cleavage system aminomethyltransferase GcvT	1	1	1	1	1	1	1	1	1	1	1	11	CORE	COG0404_E#	46	333	8.9E-91	K00605	46	333	3.11E-90
3364	OSC63688.1	-	sensor histidine kinase	0	1	1	1	0	0	1	1	1	1	0	7	Accessory	COG4585_T#	32.7	97	1.07E-19	NA	32.1	83	5.6E-15
3365	WP_048460101.1	serC	phosphoserine transaminase	1	1	1	1	1	1	1	1	1	1	1	11	CORE	COG1932_HE#	66.5	515	2.16E-145	K00831	65.9	512	3.73E-144
3366	OSC64014.1	-	recombinase RecA	1	1	1	1	1	1	1	1	1	1	1	11	CORE	COG0468_L#	72	512	1.83E-144	K03553	66.2	473	1.92E-132
3367	PWE11190.1	-	ADP-ribosylglycohydrolase family protein	1	1	1	1	1	1	1	1	1	1	1	11	CORE	COG1397_O#	24.6	57	1.61E-07				
3368	WP_048455356.1	-	acyl-CoA desaturase	0	1	1	1	0	0	0	1	1	1	0	6	Accessory	COG3239_I#	48.9	376	9.17E-104	K00508	48.9	376	2.45E-103
3369	WP_028959076.1	menC	o-succinylbenzoate synthase	1	0	0	0	1	1	0	0	0	0	1	4	Accessory	COG4948_MR#	48.5	302	1.68E-81	K02549	45.7	298	1.11E-79
3370	OSC72193.1	-	hypothetical protein	0	1	0	0	0	0	0	0	0	0	0	1	Unique	COG1819_GC#	49.1	50	0.0000115	K00699	32	56	7.33E-07

cluster	GI	gene	Annotation	Streptomyces_thermocarboxydus_BPSAC147	Streptomyces_sp._4F	Streptomyces_sp._BSE7F	Streptomyces_sp._ETH9427	Streptomyces_sp._FxanaD5	Streptomyces_sp._UNC401CLCol	Actinospica_acidiphila_NRRL_B_24431	Streptomyces_sp._HNS054	Streptomyces_sp._SMS_SU21	Streptomyces_sp._ZS0098	Streptomyces_thermocarboxydus_K155	Addition	Core-Accessory-Unique	COG	%Similarity_COG	Score_COG	Evalue_COG	KEGG	%Similarity_KEGG	Score_KEGG	Evalue_KEGG
3371	WP_102637530.1	-	hypothetical protein	0	0	0	0	0	0	0	0	1	0	0	1	Unique								
3372	WP_028958774.1	-	serine hydrolase	0	1	1	1	1	1	1	1	1	1	1	10	Accessory	COG1680_V#	36.1	136	2.72E-31	NA	27	84	3.28E-15
3373	OSC61307.1	-	hypothetical protein	0	1	0	0	0	0	0	0	0	0	0	1	Unique								
3374	Prokka:JAPCLKMA_05674	-	Putative prophage phiRv2 integrase	1	0	0	0	0	0	0	0	0	0	0	1	Unique	COG0582_L#	27.8	112	3.23E-24	NA	25.9	82	9.55E-15
3375	PWE08573.1	-	hypothetical protein	0	0	1	0	0	0	0	0	0	0	0	1	Unique								
3376	Prokka:CIODPCDJ_06416	celE	Endoglucanase E-5	1	0	1	1	1	1	1	1	1	1	1	10	Accessory	COG2730_G#	38.6	241	6.01E-63	K01179	38.6	241	1.61E-62
3377	AXI91049.1	-	MerR family transcriptional regulator	0	0	1	1	0	0	0	1	1	1	0	5	Accessory	COG0789_K#	44.2	97	1.07E-19	NA	35.9	97	2.86E-19
3378	WP_048459156.1	-	rRNA pseudouridine synthase	1	1	1	1	1	1	1	1	1	1	1	11	CORE	COG1187_J#	55.4	255	4.01E-67	K06178	53.1	246	4.98E-64
3379	Prokka:JAPCLKMA_01493	-	hypothetical protein	1	0	0	1	0	0	0	0	0	0	0	2	Accessory	COG0305_L#	29.5	46	0.000283				
3380	AXI90001.1	-	ABC transporter permease	1	0	1	1	1	1	1	1	1	1	1	10	Accessory	COG0609_P#	50.9	312	1.62E-84	K02015	40.9	225	9.09E-58
3381	AXI89662.1	-	hypothetical protein	0	0	0	1	0	0	0	0	0	0	0	1	Unique	COG2856_E#	37	120	1.18E-26	NA	35.6	147	2.41E-34
3382	WP_122217241.1	-	nitronate monooxygenase	1	1	1	1	1	1	1	1	1	1	1	11	CORE	COG2070_R#	44	270	9.23E-72	K00459	44	270	2.47E-71

cluster	GI	gene	Annotation	Streptomyces_thermocarboxydus_BPSAC147	Streptomyces_sp._4F	Streptomyces_sp._BSE7F	Streptomyces_sp._ETH9427	Streptomyces_sp._FxanaD5	Streptomyces_sp._UNC401CLCol	Actinospica_acidiphila_NRRL_B_24431	Streptomyces_sp._HNS054	Streptomyces_sp._SMS_SU21	Streptomyces_sp._ZS0098	Streptomyces_thermocarboxydus_K155	Addition	Core-Accessory-Unique	COG	%Similarity_COG	Score_COG	Evalue_COG	KEGG	%Similarity_KEGG	Score_KEGG	Evalue_KEGG
3383	WP_019524877.1	pqqE	pyrroloquinoline quinone biosynthesis protein PqqE	0	0	0	0	1	1	0	0	0	0	0	2	Accessory	COG0535_R#	47.1	335	3.05E-91	NA	24.3	79	1.05E-13
3384	Prokka:CIODPCDJ_00528	-	hypothetical protein	0	0	0	0	1	1	0	0	0	0	1	3	Accessory								
3385	WP_158688191.1	-	hypothetical protein	1	0	0	0	1	1	0	0	0	0	1	4	Accessory	COG0654_HC#	36.1	208	4.31E-53				
3386	WP_033276070.1	-	glycoside hydrolase	1	1	0	1	1	1	1	1	1	1	1	10	Accessory	COG0791_M#	30	122	2.38E-27	NA	41.2	89	7.79E-17
3387	OSC61091.1	-	hypothetical protein	0	1	0	0	0	0	0	0	0	0	0	1	Unique								
3388	OSC71015.1	-	ABC transporter permease	0	1	0	0	0	0	1	0	0	0	0	2	Accessory	COG0601_EP#	42.3	263	1.13E-69	K12369	37.3	217	2.48E-55
3389	Prokka:JAPCLKMA_03768	-	hypothetical protein	1	0	0	0	1	0	0	1	0	0	0	3	Accessory	COG4034_S#	29.8	113	1.89E-24	NA	29.8	107	2.76E-22
3390	Prokka:JAPCLKMA_02615	desV	dTDP-3-amino-3,4,6-trideoxy-alpha-D-glucose transaminase	1	0	0	0	0	0	0	0	0	0	0	1	Unique	COG0399_M#	48	344	6.57E-94	NA	38.5	252	9.07E-66
3391	OSC63597.1	-	cell division initiation protein	1	1	1	1	1	1	1	1	1	1	1	11	CORE	COG3599_D#	27.9	96	3.12E-19	NA	36.7	85	1.47E-15
3392	OSC55699.1	-	prenyltransferase	0	1	0	0	0	0	0	0	0	0	0	1	Unique								

cluster	GI	gene	Annotation	Streptomyces_thermocarboxydus_BPSAC147	Streptomyces_sp._4F	Streptomyces_sp._BSE7F	Streptomyces_sp._ETH9427	Streptomyces_sp._FxanaD5	Streptomyces_sp._UNC401CLCol	Actinospica_acidiphila_NRRL_B_24431	Streptomyces_sp._HNS054	Streptomyces_sp._SMS_SU21	Streptomyces_sp._ZS0098	Streptomyces_thermocarboxydus_K155	Addition	Core-Accessory-Unique	COG	%Similarity_COG	Score_COG	Evalue_COG	KEGG	%Similarity_KEGG	Score_KEGG	Evalue_KEGG
3393	WP_102638465.1	-	LacI family transcriptional regulator	1	1	1	1	1	1	1	1	1	1	1	11	CORE	COG1609_K#	30	140	1.44E-32	NA	28.2	115	1.01E-24
3394	Prokka:CIODPCDJ_02018	sbpA	Multiple sugar-binding periplasmic protein SbpA	1	1	1	1	1	1	1	1	1	1	1	11	CORE	COG4213_G#	61.5	429	1.55E-119	K10543	38	196	3.45E-49
3395	WP_102638180.1	-	histidinol-phosphate transaminase	1	1	1	1	1	1	1	1	1	1	1	11	CORE	COG0079_E#	58.6	419	1.23E-116	K00817	56.4	401	1.21E-110
3396	Prokka:CIODPCDJ_01487	-	hypothetical protein	1	1	1	1	1	1	1	1	1	1	1	11	CORE	COG4677_G#	32.3	159	2.99E-38	K01051	32.9	132	8E-30
3397	AXI91094.1	-	signal protein PDZ	0	1	0	1	0	0	0	1	0	0	0	3	Accessory	COG0265_O#	39.8	205	4.75E-52	K08372	35.4	193	2.92E-48
3398	WP_086698664.1	-	sugar ABC transporter substrate-binding protein	1	0	1	1	1	1	1	1	1	1	1	10	Accessory	COG4213_G#	39	214	6E-55	K10543	37.1	198	1.19E-49
3399	AXI85286.1	-	hypothetical protein	0	0	0	1	0	0	0	0	0	0	0	1	Unique								
3400	WP_122215706.1	-	FAD-dependent oxidoreductase	0	0	0	0	0	0	0	0	0	1	0	1	Unique					NA	35.7	179	5.71E-44
3401	Prokka:JAPCLKMA_03177	-	hypothetical protein	1	0	0	0	1	1	0	0	0	0	1	4	Accessory								
3402	WP_026243912.1	-	C40 family peptidase	0	1	0	0	1	0	0	0	0	0	1	3	Accessory	COG2951_M#	37.4	135	4.62E-31	NA	37.4	135	1.23E-30

cluster	GI	gene	Annotation	Streptomyces_thermocarboxydus_BPSAC147	Streptomyces_sp._4F	Streptomyces_sp._BSE7F	Streptomyces_sp._ETH9427	Streptomyces_sp._FxanaD5	Streptomyces_sp._UNC401CLCol	Actinospica_acidiphila_NRRL_B_24431	Streptomyces_sp._HNS054	Streptomyces_sp._SMS_SU21	Streptomyces_sp._ZS0098	Streptomyces_thermocarboxydus_K155	Addition	Core-Accessory-Unique	COG	%Similarity_COG	Score_COG	Evalue_COG	KEGG	%Similarity_KEGG	Score_KEGG	Evalue_KEGG
3403	OSC61660.1	-	two-component sensor histidine kinase	1	1	0	1	1	0	1	1	1	1	1	9	Accessory	COG0642_T#	36.2	156	2.53E-37	K07651	35.7	133	3.59E-30
3404	WP_033272017.1	-	HAMP domain-containing histidine kinase	1	0	1	1	1	1	1	1	1	1	1	10	Accessory	COG0642_T#	33.9	166	1.87E-40	NA	33.7	157	3.03E-37
3405	WP_078504705.1	tcmP	three-Cys-motif partner protein TcmP	1	0	0	0	1	0	0	0	0	0	0	2	Accessory								
3406	OSC69118.1	-	hypothetical protein	0	1	0	0	0	0	0	0	0	0	0	1	Unique					NA	32.8	150	3.71E-35
3407	Prokka:CIODPCDJ_00339	-	hypothetical protein	1	1	1	1	1	1	1	1	1	1	1	11	CORE	COG1295_S#	32	165	3.19E-40	K07058	31.7	156	3.96E-37
3408	OSC71054.1	-	daunorubicin biosynthesis sensory transduction protein DnrJ	0	1	0	0	0	0	0	0	0	0	0	1	Unique	COG0399_M#	43.2	294	7.78E-79	NA	40.1	265	1.03E-69
3409	Prokka:CIODPCDJ_02539	-	hypothetical protein	1	0	1	1	1	1	1	1	1	1	1	10	Accessory	COG3509_Q#	44.1	258	3.62E-68				
3410	WP_028960035.1	-	hypothetical protein	1	0	0	0	1	1	1	0	0	0	1	5	Accessory								
3411	WP_019526509.1	-	hypothetical protein	0	0	0	0	1	0	1	0	0	0	0	2	Accessory								
3412	Prokka:JAPCLKMA_01477	-	hypothetical protein	1	0	0	0	0	0	0	0	0	0	0	1	Unique								

cluster	GI	gene	Annotation	Streptomyces_thermocarboxydus_BPSAC147	Streptomyces_sp._4F	Streptomyces_sp._BSE7F	Streptomyces_sp._ETH9427	Streptomyces_sp._FxanaD5	Streptomyces_sp._UNC401CLCol	Actinospica_acidiphila_NRRL_B_24431	Streptomyces_sp._HNS054	Streptomyces_sp._SMS_SU21	Streptomyces_sp._ZS0098	Streptomyces_thermocarboxydus_K155	Addition	Core-Accessory-Unique	COG	%Similarity_COG	Score_COG	Evalue_COG	KEGG	%Similarity_KEGG	Score_KEGG	Evalue_KEGG
3413	PWE08771.1	-	5,10-methylenetetrahydromethanopterin reductase	1	1	1	1	1	1	1	1	1	1	1	11	CORE	COG2141_C#	74.5	554	3.18E-157	NA	26.3	69	6.35E-11
3414	WP_033275397.1	-	DUF3533 domain-containing protein	1	0	0	0	1	1	1	0	0	0	1	5	Accessory	COG1511_S#	24.6	70	1.82E-11	NA	24.6	70	4.86E-11
3415	WP_102637117.1	-	amino acid ABC transporter substrate-binding protein	1	0	1	0	0	0	1	0	1	0	0	4	Accessory	COG0683_E#	24.6	68	6.92E-11				
3416	Prokka:CIODPCDJ_04913	-	hypothetical protein	0	0	0	0	0	0	0	0	0	0	1	1	Unique	COG0532_J#	36	169	2.88E-41	K02519	37.3	137	2.48E-31
3417	WP_159020953.1	-	hypothetical protein	0	0	0	0	0	0	0	1	0	0	0	1	Unique								
3418	WP_078493032.1	-	erythropoiesis-stimulating protein	0	0	0	0	0	0	1	0	0	0	0	1	Unique	COG2771_K#	34.7	50	0.0000195	NA	34.7	50	0.0000521
3419	PWE08574.1	-	molybdenum cofactor biosynthesis protein MoaA	0	0	1	0	0	0	0	0	0	0	0	1	Unique	COG2896_H#	25.7	104	1.14E-21	K03639	29.9	127	3.35E-28
3420	Prokka:JAPCLKMA_05584	Acd	Glutaryl-CoA dehydrogenase	1	0	0	0	0	0	0	0	0	0	0	1	Unique	COG1960_I#	32.7	178	6.2E-44	K00249	30.9	169	7.69E-41
3421	OSC67609.1	-	theronine dehydrogenase	0	1	1	1	0	0	0	1	1	1	0	6	Accessory	COG1063_ER#	39.3	193	1.43E-48	K00001	28.7	79	1.05E-13

cluster	GI	gene	Annotation	Streptomyces_thermocarboxydus_BPSAC147	Streptomyces_sp._4F	Streptomyces_sp._BSE7F	Streptomyces_sp._ETH9427	Streptomyces_sp._FxanaD5	Streptomyces_sp._UNC401CLCol	Actinospica_acidiphila_NRRL_B_24431	Streptomyces_sp._HNS054	Streptomyces_sp._SMS_SU21	Streptomyces_sp._ZS0098	Streptomyces_thermocarboxydus_K155	Addition	Core-Accessory-Unique	COG	%Similarity_COG	Score_COG	Evalue_COG	KEGG	%Similarity_KEGG	Score_KEGG	Evalue_KEGG
3422	WP_102639478.1	tsaD	tRNA (adenosine(37)-N6)-threonylcarbamoyltransferase complex transferase subunit TsaD	1	1	1	1	1	1	1	1	1	1	1	11	CORE	COG0533_O#	60.6	397	4.98E-110	K01409	61	390	1.63E-107
3423	WP_167338184.1	-	amidohydrolase family protein	1	1	1	1	1	1	1	1	1	1	1	11	CORE	COG2159_R#	30.3	68	5.28E-11	NA	34.2	46	0.000574
3424	WP_122214926.1	-	quinone-dependent dihydroorotate dehydrogenase	1	1	1	1	1	1	1	1	1	1	1	11	CORE	COG0167_F#	53.7	364	6.09E-100	K00254	53.7	364	1.63E-99
3425	AXI85347.1	-	hypothetical protein	1	0	0	1	0	0	0	0	0	0	0	2	Accessory								
3426	WP_086700492.1	rlmN	23S rRNA (adenine(2503)-C(2))-methyltransferase RlmN	1	1	0	1	1	1	1	1	1	1	1	10	Accessory	COG0820_R#	60.9	434	4.79E-121	K06941	40.3	236	3.91E-61
3427	WP_048459539.1	-	winged helix-turn-helix transcriptional regulator	1	1	1	1	1	1	1	1	1	1	1	11	CORE	COG0640_K#	31.3	55	3.54E-07	NA	38	50	0.0000305
3428	WP_048457839.1	-	cation transporter	1	1	0	1	1	1	0	1	0	1	1	8	Accessory	COG0053_P#	59.5	354	4.82E-97	NA	59.5	354	1.29E-96

cluster	GI	gene	Annotation	Streptomyces_thermocarboxydus_BPSAC147	Streptomyces_sp._4F	Streptomyces_sp._BSE7F	Streptomyces_sp._ETH9427	Streptomyces_sp._FxanaD5	Streptomyces_sp._UNC401CLCol	Actinospica_acidiphila_NRRL_B_24431	Streptomyces_sp._HNS054	Streptomyces_sp._SMS_SU21	Streptomyces_sp._ZS0098	Streptomyces_thermocarboxydus_K155	Addition	Core-Accessory-Unique	COG	%Similarity_COG	Score_COG	Evalue_COG	KEGG	%Similarity_KEGG	Score_KEGG	Evalue_KEGG
3429	AXI85743.1	-	peptidase M24 family protein	1	1	1	1	1	1	1	1	1	1	1	11	CORE	COG0006_E#	44.4	255	3.98E-67	NA	44.4	255	1.06E-66
3430	WP_102638973.1	-	amidohydrolase family protein	1	1	1	1	1	1	1	1	1	1	1	11	CORE	COG2159_R#	43.3	245	2.42E-64	K07045	43.3	245	6.45E-64
3431	PWE10265.1	-	hypothetical protein	1	0	1	1	1	1	1	1	1	1	1	10	Accessory								
3432	WP_122215897.1	-	NAD(P)-dependent alcohol dehydrogenase	0	1	0	1	0	0	0	1	1	1	0	5	Accessory	COG1062_C#	47.3	310	1.04E-83	K00153	32.3	174	2.39E-42
3433	OSC59245.1	-	ABC transporter substrate-binding protein	0	1	0	0	0	0	0	0	0	0	0	1	Unique	COG0747_E#	25.4	104	1.14E-21	K02035	25.4	104	3.03E-21
3434	WP_107080788.1	-	hypothetical protein	0	0	1	1	0	0	0	1	1	1	0	5	Accessory								
3435	OSC73000.1	-	serine/threonine protein phosphatase	1	1	1	1	1	1	1	1	1	1	1	11	CORE	COG0639_T#	44.4	199	2.59E-50	NA	44.4	199	6.92E-50
3436	WP_122216838.1	-	ionic transporter y4hA	1	0	1	1	1	1	1	1	1	1	1	10	Accessory	COG0387_P#	69.7	457	5.27E-128	K07300	69.7	457	1.41E-127
3437	WP_051724362.1	-	prenyltransferase	1	1	1	1	1	1	1	1	1	1	1	11	CORE								
3438	PWE10290.1	-	hypothetical protein	0	0	1	1	0	0	0	0	1	1	0	4	Accessory					NA	29.9	95	1.4E-18

cluster	GI	gene	Annotation	Streptomyces_thermocarboxydus_BPSAC147	Streptomyces_sp._4F	Streptomyces_sp._BSE7F	Streptomyces_sp._ETH9427	Streptomyces_sp._FxanaD5	Streptomyces_sp._UNC401CLCol	Actinospica_acidiphila_NRRL_B_24431	Streptomyces_sp._HNS054	Streptomyces_sp._SMS_SU21	Streptomyces_sp._ZS0098	Streptomyces_thermocarboxydus_K155	Addition	Core-Accessory-Unique	COG	%Similarity_COG	Score_COG	Evalue_COG	KEGG	%Similarity_KEGG	Score_KEGG	Evalue_KEGG
3439	WP_019524628.1	-	winged helix DNA-binding domain-containing protein	0	0	0	0	1	1	1	0	0	0	1	4	Accessory								
3440	Prokka:JAPCLKMA_04194	-	hypothetical protein	1	1	1	1	1	1	1	1	1	1	1	11	CORE								
3441	PWE06646.1	-	methionine ABC transporter ATP-binding protein	1	1	1	1	1	1	1	1	1	1	1	11	CORE	COG1135_P#	50.7	323	1.19E-87	K02071	49.6	315	6.61E-85
3442	AXI88193.1	-	hypothetical protein	0	0	0	1	0	0	0	0	0	0	0	1	Unique								
3443	WP_019523503.1	-	helix-turn-helix domain-containing protein	0	0	0	0	1	1	0	0	0	0	1	3	Accessory	COG0372_C#	36.7	168	6.38E-41	K01647	26.6	80	2.74E-14
3444	Prokka:JAPCLKMA_00606	kefC_1	Glutathione-regulated potassium-efflux system protein KefC	1	1	0	1	1	1	1	1	1	1	1	10	Accessory	COG1226_P#	50.8	321	4.51E-87	K10716	50.8	321	1.2E-86
3445	WP_122213576.1	-	DGQHR domain-containing protein	0	0	0	0	0	0	0	0	0	1	0	1	Unique								
3446	OSC60029.1	-	Ku protein	1	1	1	1	1	1	1	1	1	1	1	11	CORE	COG1273_S#	37.1	198	5.76E-50	K10979	37.1	198	1.54E-49
3447	WP_078504697.1	-	MFS transporter	0	0	0	0	1	0	0	0	0	0	0	1	Unique	COG0477_GEPR#	25.9	111	5.43E-24	NA	25.9	111	1.45E-23

cluster	GI	gene	Annotation	Streptomyces_thermocarboxydus_BPSAC147	Streptomyces_sp._4F	Streptomyces_sp._BSE7F	Streptomyces_sp._ETH9427	Streptomyces_sp._FxanaD5	Streptomyces_sp._UNC401CLCol	Actinospica_acidiphila_NRRL_B_24431	Streptomyces_sp._HNS054	Streptomyces_sp._SMS_SU21	Streptomyces_sp._ZS0098	Streptomyces_thermocarboxydus_K155	Addition	Core-Accessory-Unique	COG	%Similarity_COG	Score_COG	Evalue_COG	KEGG	%Similarity_KEGG	Score_KEGG	Evalue_KEGG
3448	OSC73261.1	-	2-aminoethylphosphonate ABC transport system ATP-binding subunit PhnT	0	1	0	0	0	0	0	0	0	0	0	1	Unique	COG3842_E#	53.7	365	2.09E-100	NA	43.1	272	8.38E-72
3449	WP_102638214.1	-	citrate synthase 2	1	1	1	1	1	1	1	1	1	1	1	11	CORE	COG0372_C#	74.8	556	6.36E-158	K01647	74.8	556	1.7E-157
3450	AXI91230.1	-	hypothetical protein	0	0	0	1	0	0	0	0	0	0	0	1	Unique								
3451	AXI85933.1	-	alkene reductase	0	0	1	1	0	0	0	1	1	1	0	5	Accessory	COG1902_C#	47.2	323	9.06E-88	K10680	43.9	286	4.28E-76
3452	WP_158688245.1	-	hypothetical protein	1	0	0	1	1	1	0	1	0	1	1	7	Accessory								
3453	Prokka:CIODPCDJ_05925	-	hypothetical protein	1	1	1	1	1	1	1	1	1	1	1	11	CORE	COG0668_M#	47	271	5.34E-72	K03442	29.4	118	1.18E-25
3454	Prokka:CIODPCDJ_03296	parB	putative chromosome-partitioning protein ParB	1	1	1	1	1	1	1	1	1	1	1	11	CORE	COG1475_K#	53.7	324	5.31E-88	K03497	53.7	324	1.42E-87
3455	PWE11005.1	-	amino acid oxidase	0	0	1	1	0	0	0	1	0	1	0	4	Accessory	COG0665_E#	25.9	64	7.59E-10				
3456	WP_048457826.1	-	hypothetical protein	1	0	1	1	1	1	1	1	1	1	1	10	Accessory								
3457	AXI88147.1	-	hypothetical protein	0	0	0	1	0	0	0	0	0	0	0	1	Unique								
3458	WP_102638525.1	-	LacI family transcriptional regulator	0	0	0	1	0	0	0	1	1	0	0	3	Accessory	COG1609_K#	39.6	240	1.32E-62	K05499	31.6	168	1.3E-40
3459	OSC68290.1	-	hypothetical protein	0	1	0	0	0	0	0	0	0	0	0	1	Unique	COG0265_O#	34.3	146	1.98E-34	NA	34.3	146	5.3E-34

cluster	GI	gene	Annotation	Streptomyces_thermocarboxydus_BPSAC147	Streptomyces_sp._4F	Streptomyces_sp._BSE7F	Streptomyces_sp._ETH9427	Streptomyces_sp._FxanaD5	Streptomyces_sp._UNC401CLCol	Actinospica_acidiphila_NRRL_B_24431	Streptomyces_sp._HNS054	Streptomyces_sp._SMS_SU21	Streptomyces_sp._ZS0098	Streptomyces_thermocarboxydus_K155	Addition	Core-Accessory-Unique	COG	%Similarity_COG	Score_COG	Evalue_COG	KEGG	%Similarity_KEGG	Score_KEGG	Evalue_KEGG
3460	WP_122215729.1	-	sensor histidine kinase	0	0	0	0	0	0	0	0	0	1	0	1	Unique	COG4585_T#	28.1	77	8.66E-14	NA	28.1	77	2.31E-13
3461	Prokka:JAPCLKMA_01989	-	hypothetical protein	1	0	0	0	0	0	0	0	0	0	0	1	Unique								
3462	OSC57220.1	-	glycosyl transferase	0	1	0	0	0	0	0	0	0	0	0	1	Unique	COG1887_M#	25.6	94	1.17E-18	NA	40	77	2.31E-13
3463	WP_122216091.1	-	hypothetical protein	1	1	0	0	1	1	1	1	1	1	1	9	Accessory					NA	23.5	52	0.0000136
3464	WP_102641203.1	dapC	succinyldiaminopimelate transaminase	1	1	1	1	1	1	1	1	1	1	1	11	CORE	COG0436_E#	61.3	435	2.13E-121	NA	61.3	435	5.69E-121
3465	WP_122213974.1	-	LCP family protein	1	1	1	1	1	1	1	1	1	1	1	11	CORE	COG1316_K#	28.6	131	6.58E-30	NA	28.6	131	1.76E-29
3466	Prokka:CIODPCDJ_04108	-	hypothetical protein	1	1	1	1	1	1	1	1	1	1	1	11	CORE								
3467	WP_122213378.1	-	protein kinase family protein	1	0	1	1	1	1	1	1	1	1	1	10	Accessory								
3468	OSC59667.1	-	mechanosensitive ion channel protein MscS	1	1	1	1	1	1	1	1	1	1	1	11	CORE	COG0668_M#	33.7	156	2.5E-37	K03442	33.7	156	6.67E-37
3469	WP_086020100.1	-	ATP-binding cassette domain-containing protein	1	1	1	1	1	1	1	1	1	1	1	11	CORE	COG1131_V#	44	260	9.4E-69	K01990	44	260	2.51E-68

cluster	GI	gene	Annotation	Streptomyces_thermocarboxydus_BPSAC147	Streptomyces_sp._4F	Streptomyces_sp._BSE7F	Streptomyces_sp._ETH9427	Streptomyces_sp._FxanaD5	Streptomyces_sp._UNC401CLCol	Actinospica_acidiphila_NRRL_B_24431	Streptomyces_sp._HNS054	Streptomyces_sp._SMS_SU21	Streptomyces_sp._ZS0098	Streptomyces_thermocarboxydus_K155	Addition	Core-Accessory-Unique	COG	%Similarity_COG	Score_COG	Evalue_COG	KEGG	%Similarity_KEGG	Score_KEGG	Evalue_KEGG
3470	OSC65710.1	-	dipeptide/oligopeptide/nickel ABC transporter ATP-binding protein	1	1	1	1	1	1	1	1	1	1	1	11	CORE	COG0444_EP#	53.4	348	3.43E-95	K15583	49	333	3.05E-90
3471	OSC73373.1	-	alcohol dehydrogenase	1	1	1	1	1	1	1	1	1	1	1	11	CORE	COG1062_C#	38.7	248	3.7E-65	K00153	38	236	5.07E-61
3472	WP_019525647.1	-	winged helix DNA-binding domain-containing protein	0	0	0	0	1	0	1	0	0	0	0	2	Accessory								
3473	OSC58943.1	-	hypothetical protein	0	1	0	0	0	0	0	0	0	0	0	1	Unique								
3474	WP_048456563.1	-	hypothetical protein	1	1	0	1	1	1	1	1	1	1	1	10	Accessory					NA	29.6	151	1.64E-35
3475	WP_048456821.1	murG	undecaprenyldiphospho-muramoylpentapeptide beta-N-acetylglucosaminyltransferase	1	1	1	1	1	1	1	1	1	1	1	11	CORE	COG0707_M#	58.1	398	2.21E-110	K02563	58.1	398	5.89E-110
3476	WP_028959335.1	-	PDZ domain-containing protein	1	1	1	1	1	1	1	1	1	1	1	11	CORE	COG3480_T#	40.7	249	2.16E-65	K07177	40.7	249	5.77E-65
3477	Prokka:CIODPCDJ_00888	-	hypothetical protein	0	0	0	0	1	1	0	0	0	0	1	3	Accessory								
3478	WP_051724579.1	-	hypothetical protein	0	0	0	0	0	0	1	0	0	0	0	1	Unique								

cluster	GI	gene	Annotation	Streptomyces_thermocarboxydus_BPSAC147	Streptomyces_sp._4F	Streptomyces_sp._BSE7F	Streptomyces_sp._ETH9427	Streptomyces_sp._FxanaD5	Streptomyces_sp._UNC401CLCol	Actinospica_acidiphila_NRRL_B_24431	Streptomyces_sp._HNS054	Streptomyces_sp._SMS_SU21	Streptomyces_sp._ZS0098	Streptomyces_thermocarboxydus_K155	Addition	Core-Accessory-Unique	COG	%Similarity_COG	Score_COG	Evalue_COG	KEGG	%Similarity_KEGG	Score_KEGG	Evalue_KEGG
3479	WP_019523751.1	-	3-dehydroquinate synthase	1	1	1	1	1	1	1	1	1	1	1	11	CORE	COG0337_E#	63.4	425	2.2E-118	K01735	63.4	425	5.88E-118
3480	WP_048457389.1	-	cobalt ABC transporter permease	1	0	1	1	1	1	1	1	1	1	1	10	Accessory	COG0310_P#	38.2	208	5.52E-53	K02007	30.3	108	1.59E-22
3481	Prokka:CIODPCDJ_01888	hmuU	Hemin transport system permease protein HmuU	0	1	0	0	0	0	1	1	0	0	1	4	Accessory	COG0609_P#	45.1	300	8.17E-81	K02015	42.1	233	4.28E-60
3482	OSC62942.1	-	class V aminotransferase	1	1	1	1	1	1	1	1	1	1	1	11	CORE								
3483	WP_048459981.1	-	LigA protein	0	0	0	0	0	0	0	1	0	0	0	1	Unique					NA	37.3	89	7.64E-17
3484	Prokka:CIODPCDJ_06808	xerC_2	Tyrosine recombinase XerC	0	0	0	0	1	0	0	0	0	0	1	2	Accessory	COG0582_L#	32.9	133	1.73E-30				
3485	Prokka:JAPCLKMA_05382	-	hypothetical protein	1	1	1	1	0	0	1	1	1	1	1	9	Accessory					K09371	35	55	9.36E-07
3486	OSC63508.1	-	valine dehydrogenase	1	1	1	1	1	1	1	1	1	1	1	11	CORE	COG0334_E#	52	342	1.88E-93	K00263	49.3	338	7.23E-92
3487	AXI85265.1	-	hypothetical protein	0	0	0	1	0	0	0	0	0	0	0	1	Unique								
3488	OSC61653.1	-	two-component sensor histidine kinase	0	1	0	0	0	0	0	0	0	0	0	1	Unique	COG0642_T#	37.5	141	6.33E-33	NA	35.1	127	2.53E-28
3489	Prokka:CIODPCDJ_05796	-	IS630 family transposase ISMsm2	0	1	1	0	0	0	0	0	1	0	1	4	Accessory	COG3335_L#	53	374	4.44E-103				

cluster	GI	gene	Annotation	Streptomyces_thermocarboxydus_BPSAC147	Streptomyces_sp._4F	Streptomyces_sp._BSE7F	Streptomyces_sp._ETH9427	Streptomyces_sp._FxanaD5	Streptomyces_sp._UNC401CLCol	Actinospica_acidiphila_NRRL_B_24431	Streptomyces_sp._HNS054	Streptomyces_sp._SMS_SU21	Streptomyces_sp._ZS0098	Streptomyces_thermocarboxydus_K155	Addition	Core-Accessory-Unique	COG	%Similarity_COG	Score_COG	Evalue_COG	KEGG	%Similarity_KEGG	Score_KEGG	Evalue_KEGG
3490	OSC72659.1	-	carboxylate--amine ligase	1	1	1	1	1	1	1	1	1	1	1	11	CORE	COG2170_S#	28.7	123	1.37E-27	K06048	25.3	101	2.53E-20
3491	OSC62898.1	-	chitin-binding protein	1	1	1	1	1	1	1	1	1	1	1	11	CORE								
3492	AXI90397.1	-	hydrogenase expression protein HypE	1	0	0	1	1	1	0	1	0	1	1	7	Accessory	COG1740_C#	31.3	147	8.83E-35	K06282	28.1	127	1.93E-28
3493	PWE10119.1	-	serine/threonine-protein phosphatase	0	1	1	1	0	0	0	1	1	1	0	6	Accessory	COG2208_TK#	29.5	83	2.05E-15				
3494	OSC73155.1	-	hypothetical protein	0	1	0	0	0	0	0	0	0	0	0	1	Unique								
3495	Prokka:CIODPCDJ_04491	cyc1	Epi-isozizaene synthase	1	1	1	1	1	1	1	1	1	1	1	11	CORE								
3496	WP_147474329.1	-	hypothetical protein	0	0	1	1	0	0	1	1	1	1	0	6	Accessory					NA	27.2	57	4.19E-07
3497	WP_019521540.1	-	NAD(P)-dependent alcohol dehydrogenase	1	0	0	0	1	1	1	0	0	0	1	5	Accessory	COG1062_C#	45	306	1.49E-82	K00001	36.9	209	5.05E-53
3498	OSC62697.1	-	hypothetical protein	0	1	0	0	0	0	0	0	0	0	0	1	Unique								
3499	OSC69572.1	-	hypothetical protein	1	1	1	1	1	1	1	1	1	1	1	11	CORE	COG0391_S#	53	311	4.62E-84	NA	54.5	310	2.75E-83
3500	Prokka:CIODPCDJ_04051	-	hypothetical protein	0	0	0	0	1	1	0	0	0	0	1	3	Accessory								
3501	WP_102638087.1	-	ABC transporter permease	0	1	0	0	0	0	0	0	1	0	0	2	Accessory	COG0577_V#	30.1	157	8.53E-38	K02004	30.4	149	6.21E-35

cluster	GI	gene	Annotation	Streptomyces_thermocarboxydus_BPSAC147	Streptomyces_sp._4F	Streptomyces_sp._BSE7F	Streptomyces_sp._ETH9427	Streptomyces_sp._FxanaD5	Streptomyces_sp._UNC401CLCol	Actinospica_acidiphila_NRRL_B_24431	Streptomyces_sp._HNS054	Streptomyces_sp._SMS_SU21	Streptomyces_sp._ZS0098	Streptomyces_thermocarboxydus_K155	Addition	Core-Accessory-Unique	COG	%Similarity_COG	Score_COG	Evalue_COG	KEGG	%Similarity_KEGG	Score_KEGG	Evalue_KEGG
3502	Prokka:JAPCLKMA_00726	-	hypothetical protein	1	0	0	0	1	1	1	0	0	0	1	5	Accessory	COG0789_K#	32.9	127	9.46E-29	NA	32.9	127	2.53E-28
3503	OSC70076.1	-	hypothetical protein	1	1	0	1	1	1	1	1	1	1	1	10	Accessory								
3504	OSC72346.1	-	hypothetical protein	1	1	1	1	1	1	1	1	1	1	1	11	CORE	COG4779_P#	44.8	314	7.12E-85	K02015	45.1	276	4.4E-73
3505	Prokka:JAPCLKMA_02612	wbpI	UDP-2,3-diacetamido-2,3-dideoxy-D-glucuronate 2-epimerase	1	0	0	0	0	0	0	0	0	0	0	1	Unique	COG0381_M#	42.8	285	2.71E-76	K01791	37.3	228	8.05E-59
3506	OSC68776.1	-	phospho-N-acetylmuramoyl-pentapeptide-transferase	1	1	1	1	1	1	1	1	1	1	1	11	CORE	COG0472_M#	50.4	369	1.87E-101	K01000	49.9	363	2.73E-99
3507	WP_078895267.1	-	hypothetical protein	0	1	1	1	0	0	1	1	1	1	0	7	Accessory	COG0701_R#	30.3	156	1.9E-37	K07089	32.2	151	1.63E-35
3508	Prokka:JAPCLKMA_02270	cytR_2	HTH-type transcriptional repressor CytR	1	0	0	0	1	1	1	0	0	1	1	6	Accessory	COG1609_K#	37.4	171	7.44E-42	K05499	31.8	159	4.58E-38
3509	AXI86900.1	-	hypothetical protein	0	1	1	1	1	1	1	1	1	1	1	10	Accessory	COG1940_KG#	35.7	169	2.83E-41	NA	28.3	117	3.4E-25
3510	WP_048459799.1	-	branched-chain amino acid aminotransferase	1	1	1	1	1	1	1	1	1	1	1	11	CORE	COG0115_EH#	61.7	472	1.2E-132	K00826	58.4	448	4.93E-125
3511	WP_048455967.1	-	hypothetical protein	1	1	1	1	1	1	1	1	1	1	1	11	CORE								

cluster	GI	gene	Annotation	Streptomyces_thermocarboxydus_BPSAC147	Streptomyces_sp._4F	Streptomyces_sp._BSE7F	Streptomyces_sp._ETH9427	Streptomyces_sp._FxanaD5	Streptomyces_sp._UNC401CLCol	Actinospica_acidiphila_NRRL_B_24431	Streptomyces_sp._HNS054	Streptomyces_sp._SMS_SU21	Streptomyces_sp._ZS0098	Streptomyces_thermocarboxydus_K155	Addition	Core-Accessory-Unique	COG	%Similarity_COG	Score_COG	Evalue_COG	KEGG	%Similarity_KEGG	Score_KEGG	Evalue_KEGG
3512	Prokka:CIODPCDJ_06659	-	hypothetical protein	0	0	0	0	1	1	0	0	0	0	1	3	Accessory	COG0142_H#	42.5	245	3.1E-64	K13787	39.2	211	1.73E-53
3513	OSC69053.1	-	histidinol phosphatase	1	1	0	1	1	1	1	1	1	1	1	10	Accessory	COG1387_ER#	60.5	395	1.86E-109	K04477	60.5	395	4.96E-109
3514	Prokka:JAPCLKMA_01099	bcrA_1	Bacitracin transport ATP-binding protein BcrA	1	1	1	1	1	1	1	1	1	1	1	11	CORE	COG1131_V#	42.5	220	1.07E-56	K01990	37.3	198	1.52E-49
3515	Prokka:CIODPCDJ_03026	-	hypothetical protein	1	0	1	1	1	1	1	1	1	1	1	10	Accessory	COG0631_T#	53.2	221	6.27E-57	K01090	53.2	221	1.67E-56
3516	WP_122213481.1	-	GNAT family N-acetyltransferase	1	1	1	1	1	1	1	1	1	1	1	11	CORE	COG0454_KR#	34.7	98	6.13E-20				
3517	WP_033274121.1	-	hypothetical protein	0	0	1	0	1	1	1	0	0	0	0	4	Accessory								
3518	WP_102641190.1	ychF	redox-regulated ATPase YchF	1	1	1	1	1	1	1	1	1	1	1	11	CORE	COG0012_J#	70.2	498	1.56E-140	K06942	65.5	470	2.07E-131
3519	Prokka:CIODPCDJ_01681	-	hypothetical protein	1	1	1	1	1	1	1	1	1	1	1	11	CORE								
3520	OSC55819.1	-	hypothetical protein	0	1	0	0	0	0	0	0	0	0	0	1	Unique	COG2319_R#	42.1	249	1.65E-65	NA	32.9	129	8.66E-29
3521	WP_033275930.1	-	thiamine ABC transporter substrate-binding protein	1	1	1	1	1	1	1	1	1	1	1	11	CORE	COG4143_H#	39.4	231	6.06E-60	K02064	39.4	231	1.62E-59
3522	WP_052183375.1	-	hypothetical protein	0	0	0	0	0	1	0	0	0	0	0	1	Unique								

cluster	GI	gene	Annotation	Streptomyces_thermocarboxydus_BPSAC147	Streptomyces_sp._4F	Streptomyces_sp._BSE7F	Streptomyces_sp._ETH9427	Streptomyces_sp._FxanaD5	Streptomyces_sp._UNC401CLCol	Actinospica_acidiphila_NRRL_B_24431	Streptomyces_sp._HNS054	Streptomyces_sp._SMS_SU21	Streptomyces_sp._ZS0098	Streptomyces_thermocarboxydus_K155	Addition	Core-Accessory-Unique	COG	%Similarity_COG	Score_COG	Evalue_COG	KEGG	%Similarity_KEGG	Score_KEGG	Evalue_KEGG
3523	WP_019524886.1	-	HAD-IIA family hydrolase	0	0	0	0	1	1	0	0	0	0	0	2	Accessory	COG0647_G#	42.1	200	8.77E-51	NA	42.1	200	2.34E-50
3524	Prokka:JAPCLKMA_01036	-	hypothetical protein	1	1	0	1	1	1	0	1	1	0	1	8	Accessory								
3525	OSC50232.1	-	hypothetical protein	0	1	0	0	0	0	0	0	0	0	0	1	Unique								
3526	OSC62225.1	-	amino acid permease	0	1	0	0	0	0	0	0	0	0	0	1	Unique	COG0531_E#	41.7	257	1.03E-67	K14052	31	166	6.39E-40
3527	OSC61502.1	-	glycine/betaine ABC transporter ATP-binding protein	1	1	1	1	1	1	1	1	1	1	1	11	CORE	COG4175_E#	59.9	334	3.89E-91	K02000	59.9	335	7.95E-91
3528	OSC61393.1	-	S-(hydroxymethyl)mycothiol dehydrogenase	1	1	1	1	1	1	1	1	1	1	1	11	CORE	COG1062_C#	74	549	7.69E-156	K00153	74	549	2.05E-155
3529	AXI86090.1	-	hydrolase	1	1	1	1	1	1	1	1	1	1	1	11	CORE								
3530	WP_033273625.1	-	DNA helicase	0	0	1	0	1	1	1	0	0	0	0	4	Accessory								
3531	Prokka:JAPCLKMA_05410	mnmC_2	tRNA 5-methylaminomethyl-2-thiouridine biosynthesis bifunctional protein MnmC	1	1	1	1	1	1	1	1	1	1	1	11	CORE	COG0287_E#	34.2	136	2.02E-31	K04517	27.5	119	8.93E-26

cluster	GI	gene	Annotation	Streptomyces_thermocarboxydus_BPSAC147	Streptomyces_sp._4F	Streptomyces_sp._BSE7F	Streptomyces_sp._ETH9427	Streptomyces_sp._FxanaD5	Streptomyces_sp._UNC401CLCol	Actinospica_acidiphila_NRRL_B_24431	Streptomyces_sp._HNS054	Streptomyces_sp._SMS_SU21	Streptomyces_sp._ZS0098	Streptomyces_thermocarboxydus_K155	Addition	Core-Accessory-Unique	COG	%Similarity_COG	Score_COG	Evalue_COG	KEGG	%Similarity_KEGG	Score_KEGG	Evalue_KEGG
3532	OSC62461.1	-	cellulose biosynthesis protein CelD	0	1	0	0	0	0	0	0	0	0	0	1	Unique	COG5653_M#	32.7	181	7.17E-45				
3533	WP_048458319.1	-	DeoR/GlpR family transcriptional regulator	1	0	0	1	1	1	1	1	1	1	1	9	Accessory	COG1609_K#	31.6	94	1.15E-18	K02529	23.9	52	0.00000786
3534	OSC55619.1	-	glycerol acyltransferase	1	1	1	1	1	1	1	1	1	1	1	11	CORE	COG0204_I#	55.5	382	1.62E-105	NA	55.5	382	4.33E-105
3535	OSC71068.1	-	hypothetical protein	0	1	0	0	0	0	0	0	0	0	0	1	Unique								
3536	OSC72131.1	-	cobalamin biosynthesis protein CobW	1	1	1	1	1	1	1	1	1	1	1	11	CORE	COG0523_R#	33.4	151	6.08E-36	NA	35.7	150	2.77E-35
3537	Prokka:CIODPCDJ_00690	-	hypothetical protein	0	0	0	0	0	0	0	0	0	0	1	1	Unique					NA	27	86	6.42E-16
3538	Prokka:JAPCLKMA_01453	-	hypothetical protein	1	0	1	0	1	1	1	1	1	1	1	9	Accessory								
3539	Prokka:JAPCLKMA_05478	-	hypothetical protein	1	0	0	0	0	0	0	0	0	0	0	1	Unique								
3540	PWE08637.1	-	peroxidase	0	0	1	0	0	0	0	0	0	0	0	1	Unique	COG2837_P#	37.9	177	7.92E-44	K16301	39.9	171	1.98E-41
3541	OSC64684.1	-	DNA polymerase III subunit beta	1	1	1	1	1	1	1	1	1	1	1	11	CORE	COG0592_L#	27.3	81	7.73E-15	K02338	27.3	81	2.06E-14
3542	WP_051724675.1	-	hypothetical protein	0	0	0	0	0	0	1	0	0	0	0	1	Unique								
3543	PWE11047.1	-	alkene reductase	0	1	1	0	0	0	0	0	0	0	0	2	Accessory	COG1902_C#	49.5	317	4.91E-86	K10680	42.7	263	2.25E-69

cluster	GI	gene	Annotation	Streptomyces_thermocarboxydus_BPSAC147	Streptomyces_sp._4F	Streptomyces_sp._BSE7F	Streptomyces_sp._ETH9427	Streptomyces_sp._FxanaD5	Streptomyces_sp._UNC401CLCol	Actinospica_acidiphila_NRRL_B_24431	Streptomyces_sp._HNS054	Streptomyces_sp._SMS_SU21	Streptomyces_sp._ZS0098	Streptomyces_thermocarboxydus_K155	Addition	Core-Accessory-Unique	COG	%Similarity_COG	Score_COG	Evalue_COG	KEGG	%Similarity_KEGG	Score_KEGG	Evalue_KEGG
3544	OSC67744.1	-	hypothetical protein	0	1	0	0	0	0	0	0	0	0	0	1	Unique	COG0515_RTKL#	30	107	1.01E-22	K08884	28.4	104	1.74E-21
3545	PWE06619.1	-	hypothetical protein	0	0	1	0	0	0	0	0	0	0	0	1	Unique								
3546	OSC63832.1	-	cell wall protein	0	1	0	0	0	0	0	0	0	0	0	1	Unique					NA	49.4	82	9.26E-15
3547	OSC57766.1	-	phytanoyl-CoA dioxygenase	0	1	1	1	0	0	0	1	0	1	0	5	Accessory								
3548	Prokka:CIODPCDJ_03572	asd_2	Aspartate-semialdehyde dehydrogenase	1	1	1	1	1	1	1	1	1	1	1	11	CORE	COG0136_E#	51.6	311	3.51E-84	K00133	50.1	290	1.71E-77
3549	OSC66289.1	-	GDP-mannose pyrophosphorylase	1	1	1	1	1	1	1	1	1	1	1	11	CORE	COG1208_MJ#	57.6	400	5.74E-111	K00966	57.6	400	1.53E-110
3550	AXI87401.1	-	inositol-3-phosphate synthase	1	1	1	1	1	1	1	1	1	1	1	11	CORE	COG1260_I#	80.2	554	4.06E-157	K01858	79.9	550	9.17E-156
3551	WP_019521298.1	rhaS	rhamnose ABC transporter substrate-binding protein	1	1	1	1	1	1	1	0	1	0	1	9	Accessory	COG1879_G#	36.7	225	4.32E-58	K10555	34.7	190	3.15E-47
3552	AXI85962.1	-	S1 family peptidase	1	1	1	1	1	1	1	1	1	1	1	11	CORE								
3553	WP_122216256.1	-	AI-2E family transporter	0	1	0	1	0	0	0	1	0	1	0	4	Accessory	COG0628_R#	32.5	183	1.44E-45	NA	31.4	171	1.98E-41
3554	AXI91394.1	-	hypothetical protein	0	0	0	1	0	0	0	0	0	0	0	1	Unique								

cluster	GI	gene	Annotation	Streptomyces_thermocarboxydus_BPSAC147	Streptomyces_sp._4F	Streptomyces_sp._BSE7F	Streptomyces_sp._ETH9427	Streptomyces_sp._FxanaD5	Streptomyces_sp._UNC401CLCol	Actinospica_acidiphila_NRRL_B_24431	Streptomyces_sp._HNS054	Streptomyces_sp._SMS_SU21	Streptomyces_sp._ZS0098	Streptomyces_thermocarboxydus_K155	Addition	Core-Accessory-Unique	COG	%Similarity_COG	Score_COG	Evalue_COG	KEGG	%Similarity_KEGG	Score_KEGG	Evalue_KEGG
3555	PWE06973.1	opcA	glucose-6-phosphate dehydrogenase assembly protein OpcA	1	1	1	1	1	1	1	1	1	1	1	11	CORE	COG3429_G#	46.1	250	9.59E-66	NA	46.1	250	2.56E-65
3556	OSC64976.1	-	iron ABC transporter	0	1	0	0	0	0	0	0	0	0	0	1	Unique	COG4779_P#	35.9	235	4.17E-61	K02015	38.6	229	6.11E-59
3557	OSC71155.1	-	acyl-CoA dehydrogenase	1	1	1	1	1	1	1	1	1	1	1	11	CORE	COG1960_I#	38.1	199	1.94E-50	K00257	38.1	199	5.19E-50
3558	WP_033277293.1	-	ABC transporter permease	1	1	1	1	1	1	1	1	1	1	1	11	CORE	COG1172_G#	59.6	405	1.78E-112	K10541	30.4	143	3.37E-33
3559	WP_033277300.1	-	DUF916 domain-containing protein	0	0	0	0	0	0	1	0	0	0	0	1	Unique								
3560	WP_122213160.1	-	cobalamin-binding protein	1	0	1	1	1	1	1	1	1	1	1	10	Accessory								
3561	OSC57289.1	-	DUF58 domain-containing protein	0	1	0	0	0	0	0	0	0	0	0	1	Unique	COG1721_R#	32.1	166	1.82E-40	NA	32.1	166	4.85E-40
3562	WP_019525332.1	hisC	histidinol-phosphate transaminase	1	1	1	1	1	1	1	1	1	1	1	11	CORE	COG0079_E#	52.8	350	6.79E-96	K00817	52.8	350	1.81E-95
3563	WP_102641355.1	-	aldehyde reductase	0	0	1	1	0	0	0	1	1	1	0	5	Accessory	COG0451_MG#	43.8	264	4.9E-70	NA	39.3	236	4.99E-61
3564	WP_033272138.1	-	succinyl-diaminopimelate desuccinylase	1	1	1	1	1	1	1	1	1	1	1	11	CORE	COG0624_E#	58.1	389	1.32E-107	K01439	56.4	381	1.25E-104

cluster	GI	gene	Annotation	Streptomyces_thermocarboxydus_BPSAC147	Streptomyces_sp._4F	Streptomyces_sp._BSE7F	Streptomyces_sp._ETH9427	Streptomyces_sp._FxanaD5	Streptomyces_sp._UNC401CLCol	Actinospica_acidiphila_NRRL_B_24431	Streptomyces_sp._HNS054	Streptomyces_sp._SMS_SU21	Streptomyces_sp._ZS0098	Streptomyces_thermocarboxydus_K155	Addition	Core-Accessory-Unique	COG	%Similarity_COG	Score_COG	Evalue_COG	KEGG	%Similarity_KEGG	Score_KEGG	Evalue_KEGG
3565	OSC73214.1	-	hypothetical protein	0	1	0	0	0	0	0	0	0	0	0	1	Unique								
3566	OSC67134.1	-	phosphate starvation-inducible protein PhoH	1	1	1	1	1	1	1	1	1	1	1	11	CORE	COG1702_T#	63.9	431	3.95E-120	K06217	63.9	431	1.06E-119
3567	Prokka:JAPCLKMA_06187	ppx1_2	Exopolyphosphatase 1	1	1	1	1	1	1	1	1	1	1	1	11	CORE	COG0248_FP#	46.3	252	1.93E-66	K01524	45.1	235	8.5E-61
3568	OSC71063.1	-	regulator	1	1	1	1	1	1	1	1	1	1	1	11	CORE	COG2508_TQ#	27.3	64	7.44E-10	NA	26.9	61	2.2E-08
3569	AXI85343.1	-	integrase	0	0	0	1	0	0	0	0	0	0	0	1	Unique	COG4974_L#	27.2	81	5.88E-15	K04763	24.7	66	6.83E-10
3570	OSC69896.1	-	5-dehydro-2-deoxygluconokinase	1	1	0	1	1	1	1	1	1	1	1	10	Accessory	COG0524_G#	61.3	362	1.72E-99	K03338	29.5	144	2.57E-33
3571	PWE11596.1	-	hypothetical protein	1	1	1	1	1	1	1	1	1	1	1	11	CORE	COG1253_R#	44.1	263	1.42E-69	NA	44.1	263	3.8E-69
3572	WP_078895025.1	-	NAD(P)/FAD-dependent oxidoreductase	1	1	1	1	1	1	1	1	1	1	1	11	CORE	COG2072_P#	44.1	229	1.74E-59	K07222	27.9	102	8.58E-21
3573	OSC73437.1	-	peptide chain release factor 1	1	1	1	1	1	1	1	1	1	1	1	11	CORE	COG0216_J#	60.7	433	6.08E-121	K02835	60.5	433	2.77E-120
3574	AXI86901.1	-	oxidoreductase	1	1	1	1	1	1	1	1	1	1	1	11	CORE	COG0673_R#	44.3	269	1.52E-71	NA	44	267	2.01E-70
3575	Prokka:JAPCLKMA_01526	-	hypothetical protein	1	0	0	1	0	0	0	0	0	0	0	2	Accessory								
3576	WP_033275843.1	-	hypothetical protein	1	1	0	1	1	1	1	1	1	1	1	10	Accessory								
3577	OSC57673.1	-	hypothetical protein	0	1	0	0	0	0	0	0	0	0	0	1	Unique								

cluster	GI	gene	Annotation	Streptomyces_thermocarboxydus_BPSAC147	Streptomyces_sp._4F	Streptomyces_sp._BSE7F	Streptomyces_sp._ETH9427	Streptomyces_sp._FxanaD5	Streptomyces_sp._UNC401CLCol	Actinospica_acidiphila_NRRL_B_24431	Streptomyces_sp._HNS054	Streptomyces_sp._SMS_SU21	Streptomyces_sp._ZS0098	Streptomyces_thermocarboxydus_K155	Addition	Core-Accessory-Unique	COG	%Similarity_COG	Score_COG	Evalue_COG	KEGG	%Similarity_KEGG	Score_KEGG	Evalue_KEGG
3578	WP_084206932.1	-	ParA family protein	1	1	1	1	1	1	1	1	1	1	1	11	CORE	COG1192_D#	60.1	361	2.94E-99	K03496	60.1	361	7.85E-99
3579	PWE07973.1	-	multidrug ABC transporter ATP-binding protein	0	0	1	1	0	0	0	1	1	1	0	5	Accessory	COG1131_V#	44.6	268	3.39E-71	K01990	42	233	3.22E-60
3580	AXI86137.1	-	cytochrome B	1	1	1	1	1	1	1	1	1	1	1	11	CORE	COG1612_O#	34.5	151	4.62E-36	K02259	32.4	145	8.84E-34
3581	OSC65009.1	-	hypothetical protein	1	1	1	1	1	1	1	1	1	1	1	11	CORE	COG0053_P#	31.4	163	1.53E-39	K14696	33	144	2.57E-33
3582	OSC67562.1	-	hypothetical protein	0	1	0	0	0	0	0	0	0	0	0	1	Unique								
3583	OSC61036.1	-	polyprenyl synthetase	0	1	0	0	0	0	0	0	0	0	0	1	Unique	COG0142_H#	33.1	132	2.9E-30	K13787	31.1	119	5.19E-26
3584	Prokka:CIODPCDJ_06525	-	L-Ala-D/L-Glu epimerase	1	0	0	0	1	1	0	0	0	0	1	4	Accessory	COG4948_MR#	38.2	201	5.08E-51	NA	31.4	158	1.32E-37
3585	PWE09791.1	-	hypothetical protein	0	0	1	0	0	0	0	0	0	0	0	1	Unique					NA	24.1	67	2.34E-10
3586	WP_033277694.1	-	hypothetical protein	0	0	0	0	0	0	1	0	0	0	0	1	Unique								
3587	OSC69004.1	-	glycosyl transferase	0	1	0	0	0	0	0	0	0	0	0	1	Unique	COG0463_M#	34	111	5.3E-24	K16698	40.2	92	1.16E-17
3588	AXI91289.1	-	hypothetical protein	0	0	0	1	0	0	0	0	0	0	0	1	Unique	COG1674_D#	48.2	95	3.93E-19	K03466	48.2	95	1.05E-18
3589	OSC72090.1	-	hypothetical protein	0	1	0	0	0	0	0	0	0	0	0	1	Unique								
3590	OSC67362.1	-	chitin-binding protein	1	1	1	0	1	1	1	1	1	1	1	10	Accessory	COG3397_S#	52.2	234	5.4E-61	NA	30	59	1.08E-07

cluster	GI	gene	Annotation	Streptomyces_thermocarboxydus_BPSAC147	Streptomyces_sp._4F	Streptomyces_sp._BSE7F	Streptomyces_sp._ETH9427	Streptomyces_sp._FxanaD5	Streptomyces_sp._UNC401CLCol	Actinospica_acidiphila_NRRL_B_24431	Streptomyces_sp._HNS054	Streptomyces_sp._SMS_SU21	Streptomyces_sp._ZS0098	Streptomyces_thermocarboxydus_K155	Addition	Core-Accessory-Unique	COG	%Similarity_COG	Score_COG	Evalue_COG	KEGG	%Similarity_KEGG	Score_KEGG	Evalue_KEGG
3591	WP_033274541.1	-	zinc-dependent alcohol dehydrogenase family protein	1	1	1	1	1	1	1	1	1	1	1	11	CORE	COG1063_ER#	53.5	385	2.47E-106	K18369	46.2	332	3.89E-90
3592	WP_019522212.1	moaA	GTP 3',8-cyclase MoaA	1	1	1	1	1	1	1	1	1	1	1	11	CORE	COG2896_H#	62.3	417	5.88E-116	K03639	62.3	417	1.57E-115
3593	Prokka:JAPCLKMA_01139	add2_1	Aminodeoxyfutalosine deaminase	1	1	1	1	1	1	1	1	1	1	1	11	CORE	COG1816_F#	36.4	182	2.44E-45	K01488	27.5	118	1.15E-25
3594	OSC64977.1	-	iron ABC transporter permease	0	1	0	0	0	0	0	0	0	0	0	1	Unique	COG0609_P#	44.2	294	5.74E-79	K02015	43.2	257	1.59E-67
3595	OSC72495.1	-	hypothetical protein	1	1	1	1	1	1	1	1	1	1	1	11	CORE								
3596	WP_157842160.1	-	hypothetical protein	0	0	0	0	0	0	1	0	0	0	0	1	Unique								
3597	Prokka:CIODPCDJ_02125	xerC_1	Tyrosine recombinase XerC	0	0	1	0	0	0	0	0	1	0	1	3	Accessory	COG0582_L#	32.3	142	3.65E-33	NA	28.1	88	1.67E-16
3598	OSC71619.1	-	Rieske (2Fe-2S) protein	0	1	0	0	0	0	0	0	0	0	0	1	Unique	COG4638_PR#	38.3	232	2.05E-60				
3599	OSC70340.1	-	hypothetical protein	1	1	0	1	1	1	1	1	1	1	1	10	Accessory								
3600	AXI91269.1	-	hypothetical protein	0	0	0	1	0	0	0	0	0	0	0	1	Unique								
3601	AXI89663.1	-	hypothetical protein	0	0	0	1	0	0	0	0	0	0	0	1	Unique								

cluster	GI	gene	Annotation	Streptomyces_thermocarboxydus_BPSAC147	Streptomyces_sp._4F	Streptomyces_sp._BSE7F	Streptomyces_sp._ETH9427	Streptomyces_sp._FxanaD5	Streptomyces_sp._UNC401CLCol	Actinospica_acidiphila_NRRL_B_24431	Streptomyces_sp._HNS054	Streptomyces_sp._SMS_SU21	Streptomyces_sp._ZS0098	Streptomyces_thermocarboxydus_K155	Addition	Core-Accessory-Unique	COG	%Similarity_COG	Score_COG	Evalue_COG	KEGG	%Similarity_KEGG	Score_KEGG	Evalue_KEGG
3602	Prokka:CIODPCDJ_01083	ruvB_1	Holliday junction ATP-dependent DNA helicase RuvB	1	1	1	1	1	1	1	1	1	1	1	11	CORE	COG2255_L#	68.8	468	1.7E-131	K03551	68.8	468	4.53E-131
3603	WP_078895195.1	-	sulfite oxidase	1	0	1	0	1	1	1	1	1	1	1	9	Accessory	COG2041_R#	51.8	385	1.89E-106	K00387	41.3	252	5.1E-66
3604	WP_037778549.1	-	hypothetical protein	0	0	0	0	1	1	0	0	0	0	1	3	Accessory								
3605	AXI85284.1	-	lanthionine synthetase	0	0	0	1	0	0	0	0	0	0	0	1	Unique								
3606	Prokka:CIODPCDJ_00662	ltaE	Low specificity L-threonine aldolase	1	1	1	1	1	1	1	1	1	1	1	11	CORE	COG2008_E#	47.2	272	2.33E-72	K01620	43.7	304	1.48E-81
3607	WP_158688194.1	-	DUF3068 domain-containing protein	0	0	0	0	1	1	0	0	0	0	1	3	Accessory								
3608	WP_028960467.1	hypE	hydrogenase expression/formation protein HypE	1	0	0	1	1	1	0	1	0	1	1	7	Accessory	COG0309_O#	49.1	337	4.52E-92	K04655	49.1	337	1.21E-91
3609	Prokka:JAPCLKMA_00854	-	hypothetical protein	1	1	1	1	1	1	1	1	1	1	1	11	CORE	COG0392_S#	27.8	99	2.7E-20	K07027	27.8	99	7.22E-20
3610	Prokka:JAPCLKMA_02766	-	hypothetical protein	1	1	1	1	1	1	1	1	1	1	1	11	CORE	COG1398_I#	38.4	212	2.86E-54	K00507	37.5	195	1.26E-48

cluster	GI	gene	Annotation	Streptomyces_thermocarboxydus_BPSAC147	Streptomyces_sp._4F	Streptomyces_sp._BSE7F	Streptomyces_sp._ETH9427	Streptomyces_sp._FxanaD5	Streptomyces_sp._UNC401CLCol	Actinospica_acidiphila_NRRL_B_24431	Streptomyces_sp._HNS054	Streptomyces_sp._SMS_SU21	Streptomyces_sp._ZS0098	Streptomyces_thermocarboxydus_K155	Addition	Core-Accessory-Unique	COG	%Similarity_COG	Score_COG	Evalue_COG	KEGG	%Similarity_KEGG	Score_KEGG	Evalue_KEGG
3611	WP_102638167.1	-	prolipoprotein diacylglyceryl transferase	1	1	1	1	1	1	1	1	1	1	1	11	CORE	COG0682_M#	48.3	325	1.78E-88	NA	48.3	325	4.74E-88
3612	Prokka:CIODPCDJ_00384	ligD_2	Multifunctional non-homologous end joining protein LigD	1	1	1	1	1	1	1	1	1	1	1	11	CORE	COG1793_L#	41.2	216	1.52E-55	K01971	40.9	216	4.05E-55
3613	Prokka:JAPCLKMA_01488	-	hypothetical protein	1	0	0	0	0	0	0	0	0	0	0	1	Unique								
3614	WP_033276281.1	-	alpha-hydroxy-acid oxidizing protein	0	0	0	0	0	0	1	0	0	0	0	1	Unique	COG1304_C#	42.5	254	6.57E-67	K11517	45	269	4.04E-71
3615	WP_019523464.1	hemE	uroporphyrinogen decarboxylase	1	1	1	1	1	1	1	1	1	1	1	11	CORE	COG0407_H#	57.9	382	1.22E-105	K01599	57.9	382	3.26E-105
3616	WP_102638252.1	-	SpoIIE family protein phosphatase	1	1	1	1	1	1	1	1	1	1	1	11	CORE	COG2172_T#	39.1	86	3.09E-16				
3617	Prokka:JAPCLKMA_00083	-	hypothetical protein	1	1	1	1	1	1	1	1	1	1	1	11	CORE	COG0714_R#	51.5	324	5.15E-88	K03924	49.5	307	1.74E-82

cluster	GI	gene	Annotation	Streptomyces_thermocarboxydus_BPSAC147	Streptomyces_sp._4F	Streptomyces_sp._BSE7F	Streptomyces_sp._ETH9427	Streptomyces_sp._FxanaD5	Streptomyces_sp._UNC401CLCol	Actinospica_acidiphila_NRRL_B_24431	Streptomyces_sp._HNS054	Streptomyces_sp._SMS_SU21	Streptomyces_sp._ZS0098	Streptomyces_thermocarboxydus_K155	Addition	Core-Accessory-Unique	COG	%Similarity_COG	Score_COG	Evalue_COG	KEGG	%Similarity_KEGG	Score_KEGG	Evalue_KEGG
3618	WP_102641106.1	-	branched-chain amino acid ABC transporter permease	1	0	1	1	1	1	1	1	1	1	1	10	Accessory	COG4177_E#	31.7	139	2.35E-32	K01998	30.5	101	2.48E-20
3619	OSC63510.1	-	phosphoribosylformylglycinamidine cyclo-ligase	1	1	1	1	1	1	1	1	1	1	1	11	CORE	COG0150_F#	63.5	437	5.46E-122	K01933	59.7	426	2.57E-118
3620	OSC67444.1	-	PadR family transcriptional regulator	1	1	1	1	1	1	1	1	1	1	1	11	CORE	COG1695_K#	36.2	94	6.64E-19	NA	37.2	73	4.23E-12
3621	PWE06759.1	-	RNA ligase (ATP)	0	0	1	1	0	0	1	1	1	1	0	6	Accessory								
3622	WP_102639284.1	-	DUF3533 domain-containing protein	1	1	0	1	1	1	1	1	1	1	1	10	Accessory	COG1511_S#	24.5	59	3.09E-08				
3623	Prokka:JAPCLKMA_02139	-	hypothetical protein	1	0	0	0	1	1	0	0	0	0	1	4	Accessory	COG1275_P#	51.7	158	4.89E-38	NA	50.8	123	4.66E-27
3624	Prokka:JAPCLKMA_02215	xerC_1	Tyrosine recombinase XerC	1	0	0	0	0	0	0	0	0	0	0	1	Unique								
3625	WP_102637460.1	-	ATP-dependent DNA ligase	1	1	1	1	1	1	1	1	1	1	1	11	CORE	COG1793_L#	61.3	430	5.11E-120	NA	61	427	1.15E-118
3626	WP_019524032.1	-	hypothetical protein	1	0	0	0	1	0	0	0	0	0	0	2	Accessory								
3627	WP_102637417.1	-	GNAT family N-acetyltransferase	0	0	1	1	0	0	1	1	1	1	0	6	Accessory	COG0431_R#	34.2	120	1.48E-26	NA	32.3	77	2.93E-13

cluster	GI	gene	Annotation	Streptomyces_thermocarboxydus_BPSAC147	Streptomyces_sp._4F	Streptomyces_sp._BSE7F	Streptomyces_sp._ETH9427	Streptomyces_sp._FxanaD5	Streptomyces_sp._UNC401CLCol	Actinospica_acidiphila_NRRL_B_24431	Streptomyces_sp._HNS054	Streptomyces_sp._SMS_SU21	Streptomyces_sp._ZS0098	Streptomyces_thermocarboxydus_K155	Addition	Core-Accessory-Unique	COG	%Similarity_COG	Score_COG	Evalue_COG	KEGG	%Similarity_KEGG	Score_KEGG	Evalue_KEGG
3628	OSC69398.1	-	LacI family transcriptional regulator	1	1	1	1	1	1	1	1	1	1	1	11	CORE	COG1609_K#	36	194	6.16E-49	K03604	36.2	183	4.95E-45
3629	Prokka:JAPCLKMA_02982	-	hypothetical protein	1	0	0	0	0	0	0	0	0	0	1	2	Accessory								
3630	OSC72911.1	-	reductase	0	1	0	0	0	0	0	0	0	0	0	1	Unique								
3631	WP_033277132.1	-	glucose-1-phosphate thymidylyltransferase	0	1	0	0	1	1	1	0	0	0	1	5	Accessory	COG1209_M#	49.2	350	6.72E-96	K00973	49.2	350	1.79E-95
3632	Prokka:JAPCLKMA_03770	-	hypothetical protein	1	0	0	0	0	0	0	0	0	0	0	1	Unique								
3633	OSC73393.1	-	GNAT family N-acetyltransferase	1	1	1	1	1	1	1	1	1	1	1	11	CORE	COG1670_J#	30	55	5.83E-07	K03790	30	43	0.00469
3634	OSC62535.1	-	alpha-N-arabinofuranosidase	0	1	0	0	0	0	0	0	0	0	0	1	Unique	COG3940_R#	59.8	414	3.79E-115	NA	54.8	381	9.49E-105
3635	OSC71407.1	-	lipoate--protein ligase	1	1	1	1	1	1	1	1	1	1	1	11	CORE	COG0095_H#	53.4	383	9.32E-106	K03800	32.3	116	4.35E-25
3636	WP_122213312.1	-	NAD(+)/NADH kinase	1	0	1	1	1	1	1	1	1	1	1	10	Accessory	COG0061_G#	33.1	138	4E-32	K00858	32.8	119	8.76E-26
3637	WP_086702214.1	-	sugar ABC transporter permease	1	1	1	1	1	1	1	1	1	1	1	11	CORE	COG4209_G#	38.9	264	4.83E-70	K17319	42.1	268	6.84E-71
3638	Prokka:CIODPCDJ_05994	limB	Limonene 1,2-monooxygenase	1	0	0	0	1	1	1	0	0	0	1	5	Accessory	COG2141_C#	31.7	180	9.18E-45	NA	26.8	69	7.96E-11

cluster	GI	gene	Annotation	Streptomyces_thermocarboxydus_BPSAC147	Streptomyces_sp._4F	Streptomyces_sp._BSE7F	Streptomyces_sp._ETH9427	Streptomyces_sp._FxanaD5	Streptomyces_sp._UNC401CLCol	Actinospica_acidiphila_NRRL_B_24431	Streptomyces_sp._HNS054	Streptomyces_sp._SMS_SU21	Streptomyces_sp._ZS0098	Streptomyces_thermocarboxydus_K155	Addition	Core-Accessory-Unique	COG	%Similarity_COG	Score_COG	Evalue_COG	KEGG	%Similarity_KEGG	Score_KEGG	Evalue_KEGG
3639	WP_026244249.1	-	ABC transporter substrate-binding protein	1	0	0	0	1	1	1	0	0	0	1	5	Accessory	COG0715_P#	39.4	219	1.78E-56	K15553	29.2	86	8.22E-16
3640	AXI84709.1	-	methyltransferase	0	0	1	1	0	0	0	1	1	1	0	5	Accessory	COG0500_QR#	22.5	75	5.43E-13				
3641	OSC55204.1	-	LuxR family transcriptional regulator	0	1	0	0	0	0	0	0	0	0	0	1	Unique	COG2197_TK#	41.5	59	3.08E-08	K11618	41.5	59	8.24E-08
3642	WP_019522031.1	cobT	nicotinate-nucleotide--dimethylbenzimidazole phosphoribosyltransferase	0	1	1	0	1	1	1	1	1	1	1	9	Accessory	COG2038_H#	47.6	291	3.69E-78	K00768	47.6	291	9.86E-78
3643	OSC64974.1	-	iron siderophore-binding protein	0	1	0	0	0	0	0	0	0	0	0	1	Unique	COG0614_P#	30.5	164	5.21E-40	K02016	30.2	135	1.18E-30
3644	WP_019522006.1	trpD	anthranilate phosphoribosyltransferase	1	1	1	1	1	1	1	1	1	1	1	11	CORE	COG0547_E#	50.7	333	8.47E-91	K00766	50.7	333	2.26E-90
3645	OSC56950.1	-	two-component sensor histidine kinase	0	1	0	0	0	0	0	0	0	0	0	1	Unique	COG3850_T#	30.9	67	1.13E-10	K07673	30.9	67	3.02E-10
3646	Prokka:JAPCLKMA_03215	-	hypothetical protein	1	0	1	1	1	1	1	1	1	1	1	10	Accessory	COG2208_TK#	29.2	78	4.91E-14	NA	27.1	68	1.77E-10

cluster	GI	gene	Annotation	Streptomyces_thermocarboxydus_BPSAC147	Streptomyces_sp._4F	Streptomyces_sp._BSE7F	Streptomyces_sp._ETH9427	Streptomyces_sp._FxanaD5	Streptomyces_sp._UNC401CLCol	Actinospica_acidiphila_NRRL_B_24431	Streptomyces_sp._HNS054	Streptomyces_sp._SMS_SU21	Streptomyces_sp._ZS0098	Streptomyces_thermocarboxydus_K155	Addition	Core-Accessory-Unique	COG	%Similarity_COG	Score_COG	Evalue_COG	KEGG	%Similarity_KEGG	Score_KEGG	Evalue_KEGG
3647	AXI89607.1	-	hypothetical protein	0	0	0	1	0	0	0	0	0	0	0	1	Unique								
3648	OSC68291.1	-	hypothetical protein	1	1	0	0	1	1	0	0	0	0	1	5	Accessory								
3649	WP_102637427.1	-	hypothetical protein	1	1	0	1	1	1	1	1	1	1	1	10	Accessory	COG0627_R#	29.6	149	2.27E-35	NA	29.1	77	2.24E-13
3650	OSC69113.1	-	sulfatase	0	1	0	0	0	0	0	0	0	0	0	1	Unique	COG3119_P#	32.3	186	1.67E-46	K01138	32.4	140	2.81E-32
3651	WP_078895323.1	-	pyridoxal-phosphate dependent enzyme	0	0	0	1	0	0	0	1	0	0	0	2	Accessory	COG1171_E#	38.6	186	2.18E-46	K12235	38.7	189	6.89E-47
3652	OSC61404.1	-	hypothetical protein	0	1	0	0	0	0	0	0	0	0	0	1	Unique								
3653	WP_051724318.1	-	hypothetical protein	0	0	0	0	0	0	1	0	0	0	0	1	Unique					K18886	28.2	111	1.4E-23
3654	WP_122215725.1	-	hypothetical protein	0	0	0	0	0	0	0	0	0	1	0	1	Unique								
3655	OSC57393.1	-	LacI family transcriptional regulator	0	1	0	0	0	0	0	0	0	0	0	1	Unique	COG1609_K#	33.3	169	2.76E-41	NA	29.8	157	2.22E-37
3656	WP_122215179.1	-	NAD(P)/FAD-dependent oxidoreductase	0	0	1	0	0	0	1	0	0	1	0	3	Accessory	COG2072_P#	37.8	181	7.03E-45	K07222	34.3	117	2.55E-25
3657	OSC59580.1	-	LacI family transcriptional regulator	1	1	1	1	1	1	1	1	1	1	1	11	CORE	COG1609_K#	49.7	328	3.56E-89	K03604	34.5	185	9.95E-46
3658	WP_048460824.1	-	iron-containing alcohol dehydrogenase family protein	1	1	1	1	1	1	1	1	1	1	1	11	CORE	COG0371_C#	37.2	199	2.49E-50	K00096	32.7	164	2.37E-39

cluster	GI	gene	Annotation	Streptomyces_thermocarboxydus_BPSAC147	Streptomyces_sp._4F	Streptomyces_sp._BSE7F	Streptomyces_sp._ETH9427	Streptomyces_sp._FxanaD5	Streptomyces_sp._UNC401CLCol	Actinospica_acidiphila_NRRL_B_24431	Streptomyces_sp._HNS054	Streptomyces_sp._SMS_SU21	Streptomyces_sp._ZS0098	Streptomyces_thermocarboxydus_K155	Addition	Core-Accessory-Unique	COG	%Similarity_COG	Score_COG	Evalue_COG	KEGG	%Similarity_KEGG	Score_KEGG	Evalue_KEGG
3659	WP_028959648.1	galT	galactose-1-phosphate uridylyltransferase	1	1	1	1	1	1	1	1	1	1	1	11	CORE	COG1085_C#	37.6	206	2.03E-52	NA	50.8	175	1.03E-42
3660	WP_048456039.1	-	sugar ABC transporter permease	1	0	1	1	1	1	1	1	1	1	1	10	Accessory	COG1175_G#	35.5	177	7.75E-44	K10118	35.5	177	2.07E-43
3661	AXI87350.1	paaK	phenylacetate-CoA oxygenase/reductase subunit PaaK	1	0	1	1	1	1	1	1	1	1	1	10	Accessory	COG1018_C#	44.9	291	3.68E-78	NA	44.9	291	9.83E-78
3662	WP_028958770.1	-	glycosyltransferase	1	1	1	1	1	1	1	1	1	1	1	11	CORE								
3663	PWE09786.1	-	LacI family transcriptional regulator	0	0	1	0	0	0	0	0	0	0	0	1	Unique	COG1609_K#	34.9	199	2.49E-50	NA	34.9	199	6.64E-50
3664	Prokka:JAPCLKMA_01484	-	IS110 family transposase IS1547	1	0	0	0	0	0	0	0	0	0	0	1	Unique	COG3547_L#	49.3	304	4.2E-82	K07486	49.3	304	1.12E-81
3665	Prokka:JAPCLKMA_02234	ccr	Crotonyl-CoA carboxylase/reductase	1	0	1	1	1	1	1	1	1	1	1	10	Accessory	COG0604_CR#	44.5	278	4.21E-74	NA	32.3	168	9.61E-41
3666	WP_048459975.1	-	FMN-binding protein	1	0	1	1	1	1	1	1	1	1	1	10	Accessory	COG3976_S#	26.4	69	3.88E-11	NA	27.3	53	0.00000589

cluster	GI	gene	Annotation	Streptomyces_thermocarboxydus_BPSAC147	Streptomyces_sp._4F	Streptomyces_sp._BSE7F	Streptomyces_sp._ETH9427	Streptomyces_sp._FxanaD5	Streptomyces_sp._UNC401CLCol	Actinospica_acidiphila_NRRL_B_24431	Streptomyces_sp._HNS054	Streptomyces_sp._SMS_SU21	Streptomyces_sp._ZS0098	Streptomyces_thermocarboxydus_K155	Addition	Core-Accessory-Unique	COG	%Similarity_COG	Score_COG	Evalue_COG	KEGG	%Similarity_KEGG	Score_KEGG	Evalue_KEGG
3667	WP_019526225.1	-	NAD-dependent epimerase/dehydratase family protein	1	1	1	1	1	1	1	1	1	1	1	11	CORE	COG0451_MG#	45.5	297	6.71E-80	K01784	45.5	297	1.79E-79
3668	WP_033273724.1	-	AAA domain-containing protein	0	1	1	1	0	0	1	1	1	1	0	7	Accessory	COG0714_R#	31.3	137	1.16E-31	NA	31.3	137	3.1E-31
3669	WP_122216763.1	-	threonine-phosphate decarboxylase	1	0	1	1	1	1	1	1	1	1	1	10	Accessory	COG0079_E#	50.9	278	2.47E-74	NA	50.9	278	6.6E-74
3670	OSC57811.1	-	DNA-binding protein	1	1	1	1	1	1	1	1	1	1	1	11	CORE					NA	35.6	132	7.63E-30
3671	OSC71736.1	-	peptigoglycan-binding protein LysM	0	1	0	0	0	0	0	0	0	0	0	1	Unique					NA	35.5	127	3.21E-28
3672	AXI88353.1	-	hypothetical protein	0	0	0	1	0	0	0	0	0	0	0	1	Unique	COG0532_J#	25.2	73	1.58E-12	NA	29.5	77	3.81E-13
3673	WP_122217281.1	-	hypothetical protein	0	0	0	0	0	0	0	0	0	1	0	1	Unique								
3674	WP_033277706.1	-	3-oxoacyl-ACP synthase	0	0	0	0	0	0	1	0	0	0	0	1	Unique								
3675	WP_048454996.1	-	PRC and DUF2382 domain-containing protein	1	1	1	1	1	1	1	1	1	1	1	11	CORE	COG3861_S#	34.6	60	1.8E-08	NA	39.5	70	4.65E-11
3676	OSC62463.1	-	glycosyl transferase family 2	0	1	0	0	0	0	0	0	0	0	0	1	Unique	COG0463_M#	32.8	111	8.91E-24	NA	25.1	55	9.08E-07

cluster	GI	gene	Annotation	Streptomyces_thermocarboxydus_BPSAC147	Streptomyces_sp._4F	Streptomyces_sp._BSE7F	Streptomyces_sp._ETH9427	Streptomyces_sp._FxanaD5	Streptomyces_sp._UNC401CLCol	Actinospica_acidiphila_NRRL_B_24431	Streptomyces_sp._HNS054	Streptomyces_sp._SMS_SU21	Streptomyces_sp._ZS0098	Streptomyces_thermocarboxydus_K155	Addition	Core-Accessory-Unique	COG	%Similarity_COG	Score_COG	Evalue_COG	KEGG	%Similarity_KEGG	Score_KEGG	Evalue_KEGG
3677	Prokka:JAPCLKMA_03796	mshA_3	D-inositol-3-phosphate glycosyltransferase	1	0	0	0	0	0	0	0	0	0	1	2	Accessory	COG0438_M#	34	89	2.12E-17	NA	34.3	64	3.33E-09
3678	OSC56455.1	-	GDP-mannose 4,6-dehydratase	0	1	0	0	0	0	0	0	0	0	0	1	Unique	COG1089_M#	55.4	351	3.92E-96	K01711	51.5	329	5.56E-89
3679	OSC69508.1	-	peptide synthetase	0	1	0	0	0	0	0	0	0	0	0	1	Unique	COG1020_Q#	45.4	265	3.68E-70	K04780	45.4	265	9.83E-70
3680	OSC65902.1	-	type II secretion system protein	1	1	1	1	1	1	1	1	1	1	1	11	CORE								
3681	AXI91352.1	-	hypothetical protein	0	0	0	1	0	0	0	0	0	0	0	1	Unique								
3682	Prokka:CIODPCDJ_04586	thrC	Threonine synthase	1	1	1	1	1	1	1	1	1	1	1	11	CORE	COG0498_E#	66.9	470	7.53E-132	K01733	65.7	462	4.19E-129
3683	AXI88642.1	-	alpha/beta hydrolase	1	1	1	1	1	1	1	1	1	1	1	11	CORE	COG0596_R#	27.9	84	6.82E-16	K02170	27.8	79	5.86E-14
3684	AXI85635.1	-	ABC transporter ATP-binding protein	1	1	1	1	1	1	1	1	1	1	1	11	CORE	COG1118_P#	41.2	234	5.33E-61	K02017	41.2	234	1.42E-60
3685	WP_122214830.1	pstA	phosphate ABC transporter permease PstA	1	1	1	1	1	1	1	1	1	1	1	11	CORE	COG0581_P#	51.5	275	2.72E-73	K02038	51.5	275	7.27E-73
3686	AXI86334.1	-	Rieske (2Fe-2S) protein	1	1	1	1	1	1	1	1	1	1	1	11	CORE	COG0723_C#	45.5	129	2.41E-29	K03890	32.1	154	2.44E-36

cluster	GI	gene	Annotation	Streptomyces_thermocarboxydus_BPSAC147	Streptomyces_sp._4F	Streptomyces_sp._BSE7F	Streptomyces_sp._ETH9427	Streptomyces_sp._FxanaD5	Streptomyces_sp._UNC401CLCol	Actinospica_acidiphila_NRRL_B_24431	Streptomyces_sp._HNS054	Streptomyces_sp._SMS_SU21	Streptomyces_sp._ZS0098	Streptomyces_thermocarboxydus_K155	Addition	Core-Accessory-Unique	COG	%Similarity_COG	Score_COG	Evalue_COG	KEGG	%Similarity_KEGG	Score_KEGG	Evalue_KEGG
3687	AXI91405.1	-	hypothetical protein	0	0	0	1	0	0	0	0	0	0	0	1	Unique					K01446	35.9	90	4.33E-17
3688	OSC69586.1	-	aldo/keto reductase	0	1	0	0	0	0	0	0	0	0	0	1	Unique	COG0667_C#	82.3	577	2.56E-164	NA	36.8	209	4.9E-53
3689	Prokka:JAPCLKMA_06306	-	hypothetical protein	1	0	0	0	0	0	0	0	0	0	0	1	Unique								
3690	Prokka:CIODPCDJ_02033	-	hypothetical protein	0	0	1	1	1	1	1	1	1	1	1	9	Accessory	COG1463_Q#	36.3	203	1.31E-51	K02067	36.3	203	3.51E-51
3691	Prokka:CIODPCDJ_03907	korB	2-oxoglutarate oxidoreductase subunit KorB	1	1	1	1	1	1	1	0	1	1	1	10	Accessory	COG1013_C#	65.2	438	2.43E-122	K00175	65.2	438	6.49E-122
3692	WP_019524856.1	-	aldo/keto reductase	0	0	0	0	1	1	0	0	0	0	1	3	Accessory	COG0667_C#	47.3	289	2.38E-77	NA	38.3	213	2.6E-54
3693	Prokka:JAPCLKMA_06590	-	hypothetical protein	1	1	1	1	1	1	1	1	1	1	1	11	CORE	COG0834_ET#	33.2	161	7.48E-39	K02030	33.2	161	2E-38
3694	OSC61233.1	-	hypothetical protein	0	1	0	0	0	0	0	0	0	0	0	1	Unique								
3695	WP_033276271.1	-	NAD(P)/FAD-dependent oxidoreductase	0	0	1	0	0	0	1	0	0	1	0	3	Accessory	COG2072_P#	32.9	192	2.32E-48	K11816	30.2	113	4.78E-24
3696	Prokka:CIODPCDJ_05207	-	hypothetical protein	0	0	0	0	1	0	0	0	0	0	1	2	Accessory					NA	31	90	3.32E-17

cluster	GI	gene	Annotation	Streptomyces_thermocarboxydus_BPSAC147	Streptomyces_sp._4F	Streptomyces_sp._BSE7F	Streptomyces_sp._ETH9427	Streptomyces_sp._FxanaD5	Streptomyces_sp._UNC401CLCol	Actinospica_acidiphila_NRRL_B_24431	Streptomyces_sp._HNS054	Streptomyces_sp._SMS_SU21	Streptomyces_sp._ZS0098	Streptomyces_thermocarboxydus_K155	Addition	Core-Accessory-Unique	COG	%Similarity_COG	Score_COG	Evalue_COG	KEGG	%Similarity_KEGG	Score_KEGG	Evalue_KEGG
3697	WP_122216671.1	-	alcohol dehydrogenase catalytic domain-containing protein	0	0	0	0	0	0	0	0	0	1	0	1	Unique	COG1062_C#	40.5	261	5.31E-69	K00121	38.6	231	1.2E-59
3698	OSC57366.1	-	aminotransferase	0	1	0	0	0	0	0	0	0	0	0	1	Unique	COG0399_M#	44.4	279	1.44E-74	K13010	42.9	242	5.22E-63
3699	PWE08297.1	-	peptidase M23	1	1	1	1	1	1	1	1	1	1	1	11	CORE	COG0739_M#	42.4	99	2.04E-20	NA	33	97	3.53E-19
3700	WP_048458078.1	-	UDP-N-acetylmuramate dehydrogenase	1	1	0	1	1	1	1	1	1	1	1	10	Accessory	COG0812_M#	50.4	312	2E-84	K00075	50.1	312	6.99E-84
3701	Prokka:CIODPCDJ_02381	rbsR_1	Ribose operon repressor	1	1	1	1	1	1	1	1	1	1	1	11	CORE	COG1609_K#	35.6	194	7.95E-49	K02529	34.9	189	5.23E-47
3702	WP_102638114.1	-	MerR family transcriptional regulator	0	1	0	1	0	0	1	1	1	1	0	6	Accessory	COG0789_K#	37.1	69	2.96E-11	NA	34.5	68	1.76E-10
3703	WP_048454784.1	ppk2	polyphosphate kinase 2	1	0	1	1	1	1	1	1	1	1	1	10	Accessory	COG2326_S#	63.9	383	9.25E-106	NA	57	290	2.84E-77
3704	WP_102637227.1	-	TerC family protein	0	0	1	1	0	0	0	1	1	1	0	5	Accessory	COG0861_P#	44.4	270	6.69E-72	K05794	43.1	255	7.76E-67
3705	OSC69237.1	-	stearoyl-CoA 9-desaturase	0	1	1	1	0	0	0	1	1	1	0	6	Accessory	COG1018_C#	50.6	333	1.1E-90	NA	50.6	333	2.93E-90
3706	WP_048460843.1	argC	N-acetyl-gamma-glutamyl-phosphate reductase	0	0	0	1	0	0	0	1	0	0	0	2	Accessory	COG0002_E#	39	247	7.92E-65	K05829	38.6	229	5.96E-59

cluster	GI	gene	Annotation	Streptomyces_thermocarboxydus_BPSAC147	Streptomyces_sp._4F	Streptomyces_sp._BSE7F	Streptomyces_sp._ETH9427	Streptomyces_sp._FxanaD5	Streptomyces_sp._UNC401CLCol	Actinospica_acidiphila_NRRL_B_24431	Streptomyces_sp._HNS054	Streptomyces_sp._SMS_SU21	Streptomyces_sp._ZS0098	Streptomyces_thermocarboxydus_K155	Addition	Core-Accessory-Unique	COG	%Similarity_COG	Score_COG	Evalue_COG	KEGG	%Similarity_KEGG	Score_KEGG	Evalue_KEGG
3707	WP_048460779.1	-	hypothetical protein	0	0	1	1	0	0	0	1	1	1	0	5	Accessory								
3708	PWE06737.1	-	LLM class F420-dependent oxidoreductase	1	1	1	1	1	1	1	1	1	1	1	11	CORE	COG2141_C#	40.6	253	1.1E-66	NA	40.6	253	2.95E-66
3709	WP_078895347.1	-	gamma-butyrolactone biosynthesis protein	0	0	0	1	0	0	0	1	0	0	0	2	Accessory								
3710	Prokka:CIODPCDJ_06694	dmlA	D-malate dehydrogenase [decarboxylating]	0	0	0	0	1	1	0	0	0	0	1	3	Accessory	COG0473_CE#	54.9	391	3.39E-108	K07246	54.6	390	2.02E-107
3711	Prokka:JAPCLKMA_02015	aroH_2	Phospho-2-dehydro-3-deoxyheptonate aldolase	1	0	0	0	0	0	0	0	0	0	0	1	Unique	COG3200_E#	58.7	386	1.09E-106	K01626	37.7	198	1.13E-49
3712	OSC64818.1	-	hypothetical protein	1	1	1	1	1	1	1	1	1	1	1	11	CORE	COG0791_M#	31.3	162	2.56E-39	NA	30.6	136	4.02E-31
3713	Prokka:CIODPCDJ_04423	-	hypothetical protein	1	0	0	0	1	1	0	0	0	0	1	4	Accessory	COG1522_K#	36.8	91	5.56E-18	K03718	27.5	58	1.82E-07
3714	WP_033274995.1	-	DUF2510 domain-containing protein	1	1	1	1	1	1	1	1	1	1	1	11	CORE	COG0532_J#	44.3	60	1.79E-08	NA	47.3	75	8.43E-13
3715	OSC62350.1	-	LacI family transcriptional regulator	1	1	0	0	1	1	1	0	0	0	1	6	Accessory	COG1609_K#	38.2	184	4.83E-46	K03487	33.5	158	1.29E-37

cluster	GI	gene	Annotation	Streptomyces_thermocarboxydus_BPSAC147	Streptomyces_sp._4F	Streptomyces_sp._BSE7F	Streptomyces_sp._ETH9427	Streptomyces_sp._FxanaD5	Streptomyces_sp._UNC401CLCol	Actinospica_acidiphila_NRRL_B_24431	Streptomyces_sp._HNS054	Streptomyces_sp._SMS_SU21	Streptomyces_sp._ZS0098	Streptomyces_thermocarboxydus_K155	Addition	Core-Accessory-Unique	COG	%Similarity_COG	Score_COG	Evalue_COG	KEGG	%Similarity_KEGG	Score_KEGG	Evalue_KEGG
3716	WP_033274261.1	-	LacI family DNA-binding transcriptional regulator	0	0	0	0	0	0	1	0	0	0	0	1	Unique	COG1609_K#	35	184	6.3E-46	K02529	36.9	52	0.00000764
3717	OSC62302.1	-	hypothetical protein	0	1	0	0	0	0	0	0	0	0	0	1	Unique								
3718	PWE09655.1	-	tRNA pseudouridine(55) synthase TruB	1	1	1	1	1	1	1	1	1	1	1	11	CORE	COG0130_J#	53.4	272	2.3E-72	K03177	49.3	266	3.37E-70
3719	Prokka:CIODPCDJ_01117	menF_1	Isochorismate synthase MenF	1	1	1	1	1	1	1	1	1	1	1	11	CORE	COG0147_EH#	42.4	183	1.4E-45	K01665	42.4	183	3.74E-45
3720	WP_048458868.1	-	CBS domain-containing protein	0	0	0	0	0	0	0	1	0	0	0	1	Unique								
3721	WP_122213946.1	-	class I fructose-bisphosphate aldolase	0	0	1	0	0	0	0	0	1	1	0	3	Accessory	COG1830_G#	68.5	491	3.14E-138	K11645	66.3	484	7.85E-136
3722	WP_019525923.1	-	ATP-binding cassette domain-containing protein	1	1	1	1	1	1	1	1	1	1	1	11	CORE	COG1131_V#	46.1	248	2.71E-65	K01990	46.1	248	7.25E-65
3723	WP_048457891.1	-	acyltransferase	1	0	1	1	1	1	1	1	1	1	1	10	Accessory								
3724	OSC73386.1	-	pectin esterase	0	1	0	0	0	0	0	0	0	0	0	1	Unique	COG3866_G#	41.5	218	3.93E-56	K01728	30.8	84	2.36E-15

cluster	GI	gene	Annotation	Streptomyces_thermocarboxydus_BPSAC147	Streptomyces_sp._4F	Streptomyces_sp._BSE7F	Streptomyces_sp._ETH9427	Streptomyces_sp._FxanaD5	Streptomyces_sp._UNC401CLCol	Actinospica_acidiphila_NRRL_B_24431	Streptomyces_sp._HNS054	Streptomyces_sp._SMS_SU21	Streptomyces_sp._ZS0098	Streptomyces_thermocarboxydus_K155	Addition	Core-Accessory-Unique	COG	%Similarity_COG	Score_COG	Evalue_COG	KEGG	%Similarity_KEGG	Score_KEGG	Evalue_KEGG
3725	WP_102637237.1	-	AraC family transcriptional regulator	1	1	0	1	0	0	0	0	1	0	1	5	Accessory	COG2207_K#	39	186	1.65E-46	NA	26.5	88	1.64E-16
3726	WP_078895256.1	-	hypothetical protein	1	1	1	0	1	0	1	1	1	1	1	9	Accessory								
3727	OSC71391.1	-	hypothetical protein	0	1	0	0	0	0	0	0	0	0	0	1	Unique								
3728	OSC60609.1	-	hypothetical protein	1	1	1	1	1	1	1	1	1	1	1	11	CORE								
3729	Prokka:CIODPCDJ_04043	-	hypothetical protein	1	0	0	0	0	0	1	0	0	0	1	3	Accessory	COG1512_R#	70.3	52	0.00000487	NA	44	59	6.24E-08
3730	AXI89623.1	-	hypothetical protein	0	0	0	1	0	0	0	0	0	0	0	1	Unique								
3731	OSC59787.1	-	iron ABC transporter permease	0	1	0	0	0	0	0	0	0	0	0	1	Unique	COG0609_P#	49.6	325	2.28E-88	K02015	37.5	219	4.71E-56
3732	OSC73264.1	-	2-aminoethylphosphonate ABC transporter substrate-binding protein	0	1	0	0	0	0	0	0	0	0	0	1	Unique	COG1840_P#	49.3	325	2.98E-88				
3733	WP_019527810.1	-	BMP family ABC transporter substrate-binding protein	1	1	1	1	1	1	1	1	1	1	1	11	CORE	COG1744_R#	40.5	225	2.45E-58	K07335	36.8	203	2.67E-51

cluster	GI	gene	Annotation	Streptomyces_thermocarboxydus_BPSAC147	Streptomyces_sp._4F	Streptomyces_sp._BSE7F	Streptomyces_sp._ETH9427	Streptomyces_sp._FxanaD5	Streptomyces_sp._UNC401CLCol	Actinospica_acidiphila_NRRL_B_24431	Streptomyces_sp._HNS054	Streptomyces_sp._SMS_SU21	Streptomyces_sp._ZS0098	Streptomyces_thermocarboxydus_K155	Addition	Core-Accessory-Unique	COG	%Similarity_COG	Score_COG	Evalue_COG	KEGG	%Similarity_KEGG	Score_KEGG	Evalue_KEGG
3734	WP_122215718.1	-	LLM class flavin-dependent oxidoreductase	0	0	0	0	0	0	0	0	0	1	0	1	Unique	COG2141_C#	48.4	327	5.99E-89	NA	30.3	71	2.06E-11
3735	WP_033273690.1	-	substrate-binding domain-containing protein	1	1	1	1	1	1	1	1	1	1	1	11	CORE	COG1879_G#	31.1	133	1.66E-30	NA	28.5	126	4.14E-28
3736	WP_048455265.1	-	membrane protein	1	1	0	1	1	1	1	1	1	1	1	10	Accessory	COG2220_R#	38.9	208	5.29E-53	NA	43.3	204	2.04E-51
3737	WP_019524412.1	-	agmatine deiminase family protein	1	1	0	1	1	1	1	1	0	1	1	9	Accessory	COG2957_E#	41.8	275	3.53E-73	K10536	38.5	260	3.13E-68
3738	WP_019522030.1	-	class I SAM-dependent methyltransferase	1	0	0	0	1	1	1	1	1	1	1	8	Accessory								
3739	PWE07647.1	-	chitinase	1	1	1	1	1	1	1	1	1	1	1	11	CORE					K13381	37.9	186	5.75E-46
3740	Prokka:JAPCLKMA_06116	rbsR_3	Ribose operon repressor	1	1	1	1	1	1	1	1	1	1	1	11	CORE	COG1609_K#	32.4	122	2.24E-27	K02529	29.1	107	3.4E-22
3741	Prokka:CIODPCDJ_04860	potA_2	Spermidine/putrescine import ATP-binding protein PotA	1	1	1	1	1	1	1	1	1	1	1	11	CORE	COG3842_E#	48.3	253	8.41E-67	NA	44.2	225	1.12E-57
3742	OSC50870.1	-	glutamine synthetase	0	1	0	0	0	0	0	0	0	0	0	1	Unique	COG0174_E#	33.1	116	2.74E-25				

cluster	GI	gene	Annotation	Streptomyces_thermocarboxydus_BPSAC147	Streptomyces_sp._4F	Streptomyces_sp._BSE7F	Streptomyces_sp._ETH9427	Streptomyces_sp._FxanaD5	Streptomyces_sp._UNC401CLCol	Actinospica_acidiphila_NRRL_B_24431	Streptomyces_sp._HNS054	Streptomyces_sp._SMS_SU21	Streptomyces_sp._ZS0098	Streptomyces_thermocarboxydus_K155	Addition	Core-Accessory-Unique	COG	%Similarity_COG	Score_COG	Evalue_COG	KEGG	%Similarity_KEGG	Score_KEGG	Evalue_KEGG
3743	Prokka:CIODPCDJ_04921	oppD_3	Oligopeptide transport ATP-binding protein OppD	1	1	0	0	0	1	1	0	0	0	1	5	Accessory	COG0444_EP#	48.5	294	5.62E-79	K02031	47.8	282	5.9E-75
3744	AXI91411.1	-	hypothetical protein	0	0	0	1	0	0	0	0	0	0	0	1	Unique	COG2267_I#	42.4	57	1.51E-07	K02890	43.2	50	0.0000377
3745	OSC59417.1	-	ABC transporter ATP-binding protein	0	1	1	1	0	0	1	1	1	1	0	7	Accessory	COG1131_V#	62.8	361	3.74E-99	K01990	62.8	361	9.99E-99
3746	OSC56164.1	-	rod shape-determining protein	1	1	1	1	1	1	1	1	1	1	1	11	CORE	COG1077_D#	36.3	233	9.01E-61	K03569	36.3	233	2.41E-60
3747	WP_033278096.1	-	DUF881 domain-containing protein	1	1	1	1	1	1	1	1	1	1	1	11	CORE	COG3879_S#	33.3	134	9.71E-31	NA	33.3	134	2.59E-30
3748	OSC71405.1	-	ABC transporter substrate-binding protein	0	1	0	0	0	0	0	0	0	0	0	1	Unique	COG0614_P#	59.9	357	5.4E-98	K02016	27.9	81	2E-14
3749	Prokka:JAPCLKMA_06468	galS	HTH-type transcriptional regulator GalS	1	1	0	1	1	1	1	1	1	1	1	10	Accessory	COG1609_K#	36.5	178	5.85E-44	K02529	36.5	178	1.56E-43
3750	WP_033275427.1	-	hypothetical protein	0	0	0	0	1	1	1	0	0	0	1	4	Accessory								
3751	WP_048458898.1	-	class I SAM-dependent methyltransferase	1	1	1	1	1	1	1	1	1	1	1	11	CORE								

cluster	GI	gene	Annotation	Streptomyces_thermocarboxydus_BPSAC147	Streptomyces_sp._4F	Streptomyces_sp._BSE7F	Streptomyces_sp._ETH9427	Streptomyces_sp._FxanaD5	Streptomyces_sp._UNC401CLCol	Actinospica_acidiphila_NRRL_B_24431	Streptomyces_sp._HNS054	Streptomyces_sp._SMS_SU21	Streptomyces_sp._ZS0098	Streptomyces_thermocarboxydus_K155	Addition	Core-Accessory-Unique	COG	%Similarity_COG	Score_COG	Evalue_COG	KEGG	%Similarity_KEGG	Score_KEGG	Evalue_KEGG
3752	PWE11407.1	-	IS110 family transposase	1	0	1	0	0	0	0	1	1	0	0	4	Accessory								
3753	OSC61156.1	-	WYL domain-containing protein	1	1	1	0	1	1	1	1	1	1	1	10	Accessory	COG2378_K#	35	178	3.43E-44	K13573	35	178	9.16E-44
3754	WP_033278073.1	-	hypothetical protein	0	0	0	0	0	0	1	0	0	0	0	1	Unique								
3755	OSC73280.1	-	hypothetical protein	0	1	0	0	0	0	0	0	0	0	0	1	Unique								
3756	WP_048455049.1	-	NAD(P)-dependent alcohol dehydrogenase	0	1	0	0	0	0	0	1	0	0	0	2	Accessory	COG1064_R#	50.9	354	5.96E-97	K13979	45.2	297	1.77E-79
3757	WP_019526588.1	-	DUF2637 domain-containing protein	1	0	1	1	1	1	1	1	1	1	1	10	Accessory								
3758	OSC69766.1	-	tRNA lysidine(34) synthetase TilS	1	1	0	1	1	1	1	1	1	1	1	10	Accessory	COG0037_D#	45.9	214	5.64E-55	K04075	45.9	214	1.51E-54
3759	WP_048455817.1	-	ABC transporter permeases	1	1	1	1	1	1	1	1	1	1	1	11	CORE	COG1173_EP#	33.5	196	2.08E-49	K02034	33.1	185	9.78E-46
3760	Prokka:JAPCLKMA_02947	gpr_1	L-glyceraldehyde 3-phosphate reductase	1	1	1	1	1	1	1	1	1	1	1	11	CORE	COG0667_C#	65	439	1.41E-122	K19265	65	438	4.91E-122
3761	Prokka:JAPCLKMA_02918	adh_3	Alcohol dehydrogenase	1	0	0	0	1	1	1	0	1	1	1	7	Accessory	COG1063_ER#	32.3	154	6.92E-37	K00001	30	85	1.38E-15

cluster	GI	gene	Annotation	Streptomyces_thermocarboxydus_BPSAC147	Streptomyces_sp._4F	Streptomyces_sp._BSE7F	Streptomyces_sp._ETH9427	Streptomyces_sp._FxanaD5	Streptomyces_sp._UNC401CLCol	Actinospica_acidiphila_NRRL_B_24431	Streptomyces_sp._HNS054	Streptomyces_sp._SMS_SU21	Streptomyces_sp._ZS0098	Streptomyces_thermocarboxydus_K155	Addition	Core-Accessory-Unique	COG	%Similarity_COG	Score_COG	Evalue_COG	KEGG	%Similarity_KEGG	Score_KEGG	Evalue_KEGG
3762	Prokka:CIODPCDJ_00885	-	hypothetical protein	0	0	0	0	1	1	0	0	0	0	1	3	Accessory								
3763	OSC61740.1	-	3-phenylpropionate dioxygenase	0	1	0	0	0	0	0	0	0	0	0	1	Unique	COG4638_PR#	52.5	351	5.04E-96	K00517	27.2	92	1.13E-17
3764	OSC72045.1	-	ABC transporter substrate-binding protein	1	1	1	1	1	1	1	1	1	1	1	11	CORE								
3765	OSC61743.1	-	5,10-methylenetetrahydromethanopterin reductase	0	1	0	0	0	0	0	0	0	0	0	1	Unique	COG2141_C#	27.9	164	6.69E-40	K00320	27.9	164	1.79E-39
3766	WP_019524926.1	-	hypothetical protein	0	0	0	0	1	0	0	0	0	0	0	1	Unique								
3767	OSC64763.1	-	AraC family transcriptional regulator	0	1	0	0	0	0	0	0	0	0	0	1	Unique	COG4977_K#	40.8	219	2.29E-56	NA	45.2	85	1.06E-15
3768	OSC72342.1	-	iron ABC transporter permease	1	1	0	1	1	1	1	1	1	1	1	10	Accessory	COG0609_P#	49	324	5.05E-88	K02015	46	270	3.02E-71
3769	WP_048456057.1	-	HTTM domain-containing protein	0	0	0	0	1	1	0	1	0	0	1	4	Accessory								
3770	OSC69624.1	-	LD-carboxypeptidase	0	1	0	0	0	0	0	0	0	0	0	1	Unique	COG1619_V#	40.4	196	1.59E-49	NA	40.4	196	4.24E-49

cluster	GI	gene	Annotation	Streptomyces_thermocarboxydus_BPSAC147	Streptomyces_sp._4F	Streptomyces_sp._BSE7F	Streptomyces_sp._ETH9427	Streptomyces_sp._FxanaD5	Streptomyces_sp._UNC401CLCol	Actinospica_acidiphila_NRRL_B_24431	Streptomyces_sp._HNS054	Streptomyces_sp._SMS_SU21	Streptomyces_sp._ZS0098	Streptomyces_thermocarboxydus_K155	Addition	Core-Accessory-Unique	COG	%Similarity_COG	Score_COG	Evalue_COG	KEGG	%Similarity_KEGG	Score_KEGG	Evalue_KEGG
3771	OSC60095.1	-	hypothetical protein	0	1	0	0	0	0	0	0	0	0	0	1	Unique	COG0013_J#	40.7	260	6.86E-69	K01872	36.4	215	1.15E-54
3772	WP_048458153.1	-	transcriptional regulator	1	1	1	1	1	1	1	1	1	1	1	11	CORE	COG2390_K#	26.3	115	3.55E-25	NA	28.3	120	2.95E-26
3773	PWE09807.1	-	hypothetical protein	1	0	1	1	1	1	1	1	1	1	1	10	Accessory	COG0614_P#	34.3	182	2.37E-45				
3774	WP_019521464.1	-	hypothetical protein	1	0	0	0	1	1	0	0	0	0	1	4	Accessory	COG1995_H#	39.6	191	6.66E-48	K00097	35.9	156	3.72E-37
3775	WP_026243622.1	-	LacI family transcriptional regulator	1	0	1	1	1	1	1	1	1	1	1	10	Accessory	COG1609_K#	33.7	145	3.21E-34	NA	29.1	130	2.85E-29
3776	Prokka:CIODPCDJ_04752	leuB	3-isopropylmalate dehydrogenase	1	1	1	1	1	1	1	1	1	1	1	11	CORE	COG0473_CE#	69.3	452	1.23E-126	K00052	69.3	452	3.28E-126
3777	OSC66191.1	-	hypothetical protein	0	1	0	0	0	0	0	0	0	0	0	1	Unique	COG4222_S#	30.3	104	1.07E-21				
3778	WP_102640909.1	-	S-adenosylmethionine:tRNA ribosyltransferase-isomerase	1	1	1	1	0	1	1	1	1	1	1	10	Accessory	COG0809_J#	34.7	168	6.04E-41	K07568	31.4	149	5.93E-35
3779	WP_048458602.1	-	hypothetical protein	0	0	0	0	0	0	0	1	0	0	0	1	Unique								
3780	AXI86797.1	-	alcohol dehydrogenase	1	1	1	1	1	1	1	1	1	1	1	11	CORE	COG1064_R#	59.5	434	2.65E-121	K13953	32.8	173	3.84E-42
3781	WP_028959719.1	-	MCE family protein	0	0	1	1	1	1	1	1	1	1	1	9	Accessory	COG1463_Q#	33.5	198	5.45E-50	K02067	33.5	196	4.23E-49
3782	OSC71653.1	-	ATP/GTP-binding protein	0	1	1	1	1	1	1	1	1	1	1	10	Accessory	COG0494_LR#	38.4	52	0.0000037	NA	36	90	3.27E-17

cluster	GI	gene	Annotation	Streptomyces_thermocarboxydus_BPSAC147	Streptomyces_sp._4F	Streptomyces_sp._BSE7F	Streptomyces_sp._ETH9427	Streptomyces_sp._FxanaD5	Streptomyces_sp._UNC401CLCol	Actinospica_acidiphila_NRRL_B_24431	Streptomyces_sp._HNS054	Streptomyces_sp._SMS_SU21	Streptomyces_sp._ZS0098	Streptomyces_thermocarboxydus_K155	Addition	Core-Accessory-Unique	COG	%Similarity_COG	Score_COG	Evalue_COG	KEGG	%Similarity_KEGG	Score_KEGG	Evalue_KEGG
3783	Prokka:CIODPCDJ_03462	thrB_1	Homoserine kinase	1	0	0	0	0	0	0	0	0	0	1	2	Accessory								
3784	OSC71401.1	-	transcriptional regulator	0	1	0	0	0	0	0	0	0	0	0	1	Unique								
3785	OSC71404.1	-	iron ABC transporter permease	0	1	0	0	0	0	0	0	0	0	0	1	Unique	COG0609_P#	66	440	6.3E-123	K02015	44.8	267	1.95E-70
3786	Prokka:CIODPCDJ_05535	-	hypothetical protein	0	1	0	0	1	1	0	0	0	0	1	4	Accessory	COG3268_S#	31	121	8.46E-27				
3787	OSC65435.1	-	radical SAM protein	1	1	1	1	1	1	1	1	1	1	1	11	CORE	COG1533_L#	47.2	302	2.05E-81	NA	47.2	302	5.48E-81
3788	WP_052183381.1	-	helix-turn-helix domain-containing protein	0	0	0	0	0	1	0	0	0	0	0	1	Unique								
3789	PWE10268.1	-	hypothetical protein	0	0	1	0	0	0	0	0	0	0	0	1	Unique	COG0500_QR#	28.9	54	7.45E-07				
3790	WP_033276545.1	-	helix-turn-helix domain-containing protein	1	1	0	1	1	1	1	1	1	1	1	10	Accessory	COG4977_K#	44.6	261	4.01E-69	NA	30.6	85	1.05E-15
3791	WP_033274784.1	-	glycosyl transferase	1	1	1	1	1	1	1	1	1	1	1	11	CORE	COG0472_M#	26.8	90	1.22E-17				
3792	OSC66316.1	-	LacI family transcriptional regulator	0	1	0	0	0	0	0	0	0	0	0	1	Unique	COG1609_K#	49.5	285	3.38E-76	NA	27.7	112	1.05E-23
3793	WP_122216101.1	-	LacI family transcriptional regulator	1	1	1	1	1	1	1	1	1	1	1	11	CORE	COG1609_K#	39	179	2E-44	K02529	34.4	147	2.25E-34

cluster	GI	gene	Annotation	Streptomyces_thermocarboxydus_BPSAC147	Streptomyces_sp._4F	Streptomyces_sp._BSE7F	Streptomyces_sp._ETH9427	Streptomyces_sp._FxanaD5	Streptomyces_sp._UNC401CLCol	Actinospica_acidiphila_NRRL_B_24431	Streptomyces_sp._HNS054	Streptomyces_sp._SMS_SU21	Streptomyces_sp._ZS0098	Streptomyces_thermocarboxydus_K155	Addition	Core-Accessory-Unique	COG	%Similarity_COG	Score_COG	Evalue_COG	KEGG	%Similarity_KEGG	Score_KEGG	Evalue_KEGG
3794	WP_122214535.1	-	glycosyltransferase family 2 protein	1	1	1	1	1	1	1	1	1	1	1	11	CORE	COG0463_M#	33.4	145	4.18E-34				
3795	Prokka:CIODPCDJ_06204	rbsC_3	Ribose import permease protein RbsC	1	1	1	1	1	1	1	0	1	0	1	9	Accessory	COG4158_R#	36	195	2.69E-49	K10440	34.5	186	5.7E-46
3796	Prokka:JAPCLKMA_06476	cytR_7	HTH-type transcriptional repressor CytR	1	1	1	1	1	1	1	1	1	1	1	11	CORE	COG1609_K#	39.7	249	1.57E-65	K05499	33.9	208	1.4E-52
3797	WP_019522000.1	-	C40 family peptidase	1	1	1	1	1	1	1	1	1	1	1	11	CORE	COG0791_M#	30.5	144	5.46E-34	NA	24.6	113	3.6E-24
3798	WP_033278120.1	-	LLM class flavin-dependent oxidoreductase	0	0	0	0	0	0	1	0	0	0	0	1	Unique	COG2141_C#	43.7	292	1.62E-78				
3799	WP_019525993.1	-	NAD(P)-dependent alcohol dehydrogenase	1	1	1	1	1	1	1	1	1	1	1	11	CORE	COG1064_R#	65.6	455	1.44E-127	K13979	65.6	455	3.86E-127
3800	WP_019526188.1	-	MCE family protein	0	0	1	1	1	1	1	1	1	1	1	9	Accessory	COG1463_Q#	35.2	190	1.48E-47	K02067	35.2	190	3.95E-47
3801	PWE11509.1	-	glycosyl transferase	0	0	1	0	0	0	0	0	0	0	0	1	Unique	COG0438_M#	29.6	99	3.43E-20	NA	32.7	54	0.00000259

cluster	GI	gene	Annotation	Streptomyces_thermocarboxydus_BPSAC147	Streptomyces_sp._4F	Streptomyces_sp._BSE7F	Streptomyces_sp._ETH9427	Streptomyces_sp._FxanaD5	Streptomyces_sp._UNC401CLCol	Actinospica_acidiphila_NRRL_B_24431	Streptomyces_sp._HNS054	Streptomyces_sp._SMS_SU21	Streptomyces_sp._ZS0098	Streptomyces_thermocarboxydus_K155	Addition	Core-Accessory-Unique	COG	%Similarity_COG	Score_COG	Evalue_COG	KEGG	%Similarity_KEGG	Score_KEGG	Evalue_KEGG
3802	Prokka:CIODPCDJ_06567	hcaB_8	3-phenylpropionate-dihydrodiol/cinnamic acid-dihydrodiol dehydrogenase	1	0	0	0	1	1	0	0	0	0	1	4	Accessory	COG1028_IQR#	40.2	196	1.21E-49	NA	34.6	153	4.1E-36
3803	Prokka:JAPCLKMA_05802	nudC_4	NADH pyrophosphatase	1	0	1	1	1	1	1	1	1	1	1	10	Accessory	COG2816_L#	37.5	208	5.25E-53	K03426	36.9	181	1.4E-44
3804	WP_026243720.1	-	hypothetical protein	0	0	0	0	1	0	0	0	0	0	0	1	Unique								
3805	WP_078895319.1	-	XRE family transcriptional regulator	0	0	0	1	0	0	1	1	0	0	0	3	Accessory								
3806	OSC62244.1	-	LacI family transcriptional regulator	0	1	0	0	0	0	0	0	0	0	0	1	Unique	COG1609_K#	36.1	167	1.03E-40	K02529	33.3	160	3.35E-38
3807	AXI85367.1	-	tryptophan synthase subunit alpha	0	0	0	1	0	0	0	0	0	0	0	1	Unique	COG0159_E#	33.3	123	1.7E-27	K01695	33.3	123	4.54E-27
3808	WP_019521682.1	-	ABC transporter ATP-binding protein	0	0	0	0	1	1	0	0	0	0	1	3	Accessory	COG3842_E#	57.1	366	1.5E-100	K02052	57.1	366	4.01E-100
3809	WP_048456536.1	-	hypothetical protein	1	1	1	1	1	1	1	1	1	1	1	11	CORE	COG4796_U#	31.6	63	2.08E-09	NA	43.4	45	0.0012
3810	WP_033274361.1	-	DNA topoisomerase IB	0	0	0	1	0	0	1	1	0	1	0	4	Accessory	COG3569_L#	39.4	203	1.29E-51	NA	35	178	1.55E-43
3811	AXI86624.1	-	hypothetical protein	1	1	1	1	1	1	1	1	1	1	1	11	CORE								

cluster	GI	gene	Annotation	Streptomyces_thermocarboxydus_BPSAC147	Streptomyces_sp._4F	Streptomyces_sp._BSE7F	Streptomyces_sp._ETH9427	Streptomyces_sp._FxanaD5	Streptomyces_sp._UNC401CLCol	Actinospica_acidiphila_NRRL_B_24431	Streptomyces_sp._HNS054	Streptomyces_sp._SMS_SU21	Streptomyces_sp._ZS0098	Streptomyces_thermocarboxydus_K155	Addition	Core-Accessory-Unique	COG	%Similarity_COG	Score_COG	Evalue_COG	KEGG	%Similarity_KEGG	Score_KEGG	Evalue_KEGG
3812	OSC71380.1	-	hypothetical protein	0	1	0	0	0	0	0	0	0	0	0	1	Unique								
3813	OSC72596.1	-	helix-turn-helix transcriptional regulator	1	1	1	1	1	1	1	1	1	1	1	11	CORE	COG2771_K#	31	51	0.00000819	NA	37.5	42	0.00777
3814	OSC62424.1	-	alcohol dehydrogenase	1	1	0	0	1	1	0	0	0	0	1	5	Accessory	COG1064_R#	50.4	345	2.74E-94	K13953	48.6	319	3.3E-86
3815	Prokka:JAPCLKMA_05111	-	hypothetical protein	1	1	1	1	1	1	1	1	1	1	1	11	CORE								
3816	WP_020700103.1	rarD	EamA family transporter RarD	1	0	1	1	1	1	1	1	1	1	1	10	Accessory	COG2962_R#	47.3	269	1.91E-71	K05786	41.7	236	4.79E-61
3817	WP_033277133.1	-	glycosyltransferase family 2 protein	0	0	0	0	0	0	1	0	0	0	0	1	Unique	COG0463_M#	47.3	93	1.44E-18	NA	23.8	71	2.04E-11
3818	PWE10559.1	-	hypothetical protein	0	0	1	0	0	0	0	0	0	0	0	1	Unique	COG3321_Q#	54.9	284	4.4E-76	K12441	51.8	261	1.39E-68
3819	OSC69544.1	-	ABC transporter ATP-binding protein	1	1	0	1	1	1	1	1	1	1	1	10	Accessory	COG0444_EP#	40.2	203	1.29E-51	K02031	36.7	198	1.11E-49
3820	OSC73268.1	-	hypothetical protein	0	1	0	0	0	0	0	0	0	0	0	1	Unique								
3821	WP_033277326.1	-	SGNH/GDSL hydrolase family protein	1	0	0	0	1	1	1	0	0	1	1	6	Accessory								
3822	OSC68710.1	-	peptidase	1	1	0	1	1	1	1	1	1	1	1	10	Accessory	COG0739_M#	31	103	1.82E-21	NA	31	103	4.85E-21
3823	OSC63166.1	-	oxidoreductase	1	1	1	1	1	1	1	1	1	1	1	11	CORE								

cluster	GI	gene	Annotation	Streptomyces_thermocarboxydus_BPSAC147	Streptomyces_sp._4F	Streptomyces_sp._BSE7F	Streptomyces_sp._ETH9427	Streptomyces_sp._FxanaD5	Streptomyces_sp._UNC401CLCol	Actinospica_acidiphila_NRRL_B_24431	Streptomyces_sp._HNS054	Streptomyces_sp._SMS_SU21	Streptomyces_sp._ZS0098	Streptomyces_thermocarboxydus_K155	Addition	Core-Accessory-Unique	COG	%Similarity_COG	Score_COG	Evalue_COG	KEGG	%Similarity_KEGG	Score_KEGG	Evalue_KEGG
3824	OSC69957.1	-	hypothetical protein	0	1	0	0	0	0	0	0	0	0	0	1	Unique								
3825	WP_028958852.1	-	ABC transporter permease	1	0	1	1	1	1	1	1	1	1	1	10	Accessory	COG1172_G#	41.5	223	9.19E-58	K10440	41.5	219	6.04E-56
3826	WP_033276352.1	-	LacI family DNA-binding transcriptional regulator	1	0	1	1	1	1	1	1	1	1	1	10	Accessory	COG1879_G#	54.1	348	2.47E-95	K02529	25.9	49	0.000108
3827	Prokka:JAPCLKMA_05665	glpX	Fructose-1,6-bisphosphatase class 2	1	1	1	1	1	1	1	1	1	1	1	11	CORE	COG1494_G#	70	448	2.29E-125	K02446	69.7	465	6.32E-130
3828	PWE11122.1	-	oxidoreductase	1	1	1	1	1	1	1	1	1	1	1	11	CORE	COG0673_R#	33.3	196	2.05E-49	K00078	36.7	206	5.29E-52
3829	Prokka:JAPCLKMA_06797	mmcR	Mitomycin biosynthesis 6-O-methyltransferase	1	1	1	1	1	1	1	1	1	1	1	11	CORE	COG0500_QR#	29.4	144	7.08E-34	NA	24	88	1.23E-16
3830	OSC71399.1	-	hypothetical protein	0	1	0	0	0	0	0	0	0	0	0	1	Unique								
3831	WP_048460306.1	-	trypsin-like peptidase domain-containing protein	0	0	0	0	0	0	0	1	0	0	0	1	Unique	COG0265_O#	28.8	102	3.09E-21	NA	35.1	98	1.55E-19
3832	WP_019521706.1	-	UbiA family prenyltransferase	1	1	1	1	1	1	1	1	1	1	1	11	CORE	COG0382_H#	34	89	3.53E-17	K04040	26.6	49	0.0000828
3833	WP_078504529.1	-	hypothetical protein	1	0	1	1	1	1	1	1	1	1	1	10	Accessory								

cluster	GI	gene	Annotation	Streptomyces_thermocarboxydus_BPSAC147	Streptomyces_sp._4F	Streptomyces_sp._BSE7F	Streptomyces_sp._ETH9427	Streptomyces_sp._FxanaD5	Streptomyces_sp._UNC401CLCol	Actinospica_acidiphila_NRRL_B_24431	Streptomyces_sp._HNS054	Streptomyces_sp._SMS_SU21	Streptomyces_sp._ZS0098	Streptomyces_thermocarboxydus_K155	Addition	Core-Accessory-Unique	COG	%Similarity_COG	Score_COG	Evalue_COG	KEGG	%Similarity_KEGG	Score_KEGG	Evalue_KEGG
3834	WP_048458849.1	-	winged helix-turn-helix transcriptional regulator	0	0	0	1	0	0	0	1	0	0	0	2	Accessory	COG0640_K#	35.7	49	0.0000237	K03892	35.7	49	0.0000634
3835	Prokka:JAPCLKMA_00864	xlnB	Endo-1,4-beta-xylanase B	1	1	1	1	1	1	1	1	1	1	1	11	CORE	COG2730_G#	33.6	70	1.7E-11	K01181	60.8	255	7.61E-67
3836	OSC61021.1	-	hypothetical protein	0	1	0	0	0	0	0	0	0	0	0	1	Unique					K06274	24.2	47	0.000315
3837	WP_053076981.1	-	hypothetical protein	0	0	0	0	0	0	0	1	0	0	0	1	Unique								
3838	OSC61085.1	-	L-idonate 5-dehydrogenase	0	1	0	0	0	0	0	0	0	0	0	1	Unique	COG1063_ER#	42.3	230	7.52E-60	K00098	36.4	203	2.63E-51
3839	WP_078504668.1	-	bifunctional riboflavin kinase/FAD synthetase	1	1	1	1	1	1	1	1	1	1	1	11	CORE	COG0196_H#	52.6	326	1.31E-88	K11753	52.6	326	3.51E-88
3840	WP_122214509.1	-	cation transporter	0	1	1	1	0	0	0	1	1	1	0	6	Accessory	COG0530_P#	27.5	56	1.94E-07				
3841	WP_033277972.1	paaA	1,2-phenylacetyl-CoA epoxidase subunit A	0	1	0	1	1	0	1	1	1	1	1	8	Accessory	COG3396_S#	56	398	2.71E-110	K02609	54.2	356	2.42E-97
3842	WP_102641141.1	-	SDR family oxidoreductase	1	1	1	1	1	1	1	1	1	1	1	11	CORE								
3843	Prokka:CIODPCDJ_03544	fba	Fructose-bisphosphate aldolase	1	1	1	1	1	1	1	1	1	1	1	11	CORE	COG0191_G#	68.1	473	5.08E-133	K01624	68.1	473	1.36E-132

cluster	GI	gene	Annotation	Streptomyces_thermocarboxydus_BPSAC147	Streptomyces_sp._4F	Streptomyces_sp._BSE7F	Streptomyces_sp._ETH9427	Streptomyces_sp._FxanaD5	Streptomyces_sp._UNC401CLCol	Actinospica_acidiphila_NRRL_B_24431	Streptomyces_sp._HNS054	Streptomyces_sp._SMS_SU21	Streptomyces_sp._ZS0098	Streptomyces_thermocarboxydus_K155	Addition	Core-Accessory-Unique	COG	%Similarity_COG	Score_COG	Evalue_COG	KEGG	%Similarity_KEGG	Score_KEGG	Evalue_KEGG
3844	WP_024883727.1	tdh	L-threonine 3-dehydrogenase	1	1	1	1	1	1	1	1	1	1	1	11	CORE	COG1063_ER#	61.5	441	3.65E-123	K00060	61.5	440	1.27E-122
3845	WP_033274756.1	-	glycoside hydrolase family 6 protein	1	1	0	1	1	1	1	1	1	1	1	10	Accessory	COG5297_G#	41.3	244	6.55E-64	K01179	41.3	244	1.75E-63
3846	OSC72343.1	-	HAD family hydrolase	1	1	0	1	1	1	1	1	1	1	1	10	Accessory	COG0647_G#	41.5	233	8.85E-61	NA	41.5	233	2.36E-60
3847	OSC69318.1	-	ribonucleoside-diphosphate reductase	1	1	1	1	1	1	1	1	1	1	1	11	CORE	COG0208_F#	35.6	213	9.48E-55	NA	37.8	215	6.66E-55
3848	WP_019526886.1	-	LacI family transcriptional regulator	1	0	0	0	1	1	0	0	0	0	1	4	Accessory	COG1609_K#	37.4	174	6.37E-43	NA	29.9	153	4.06E-36
3849	WP_019525110.1	-	hypothetical protein	1	0	0	0	1	1	0	0	0	0	1	4	Accessory	COG3409_M#	32.9	62	4.61E-09				
3850	Prokka:JAPCLKMA_00530	-	Alanine racemase	1	1	1	1	1	1	1	1	1	1	1	11	CORE								
3851	Prokka:JAPCLKMA_06623	-	hypothetical protein	1	0	0	0	0	0	0	0	0	0	0	1	Unique								
3852	OSC56614.1	-	hypothetical protein	1	1	1	1	1	1	1	1	1	1	1	11	CORE								
3853	WP_122215717.1	-	LLM class flavin-dependent oxidoreductase	0	0	0	0	0	0	0	0	0	1	0	1	Unique	COG2141_C#	54.6	379	1.7E-104	NA	25.1	67	2.93E-10

cluster	GI	gene	Annotation	Streptomyces_thermocarboxydus_BPSAC147	Streptomyces_sp._4F	Streptomyces_sp._BSE7F	Streptomyces_sp._ETH9427	Streptomyces_sp._FxanaD5	Streptomyces_sp._UNC401CLCol	Actinospica_acidiphila_NRRL_B_24431	Streptomyces_sp._HNS054	Streptomyces_sp._SMS_SU21	Streptomyces_sp._ZS0098	Streptomyces_thermocarboxydus_K155	Addition	Core-Accessory-Unique	COG	%Similarity_COG	Score_COG	Evalue_COG	KEGG	%Similarity_KEGG	Score_KEGG	Evalue_KEGG
3854	OSC71216.1	-	hydrogenase expression/formation protein HypE	0	1	0	0	0	0	0	0	0	0	0	1	Unique	COG0309_O#	43	257	5.72E-68	K04655	42.9	250	2.44E-65
3855	Prokka:JAPCLKMA_00933	-	hypothetical protein	1	1	1	1	1	1	1	1	1	1	1	11	CORE								
3856	PWE07334.1	-	ketoacyl-ACP synthase III	1	1	1	1	1	1	1	1	1	1	1	11	CORE	COG0332_I#	50.1	324	4.98E-88	K11608	50.1	324	1.33E-87
3857	WP_019523994.1	-	sugar ABC transporter permease	1	0	1	1	1	1	1	1	1	1	1	10	Accessory	COG1175_G#	39.7	215	3.26E-55	K02025	33.8	183	4.79E-45
3858	PWE10267.1	-	hypothetical protein	0	0	1	0	0	0	0	0	0	0	0	1	Unique	COG0500_QR#	29.6	132	2.78E-30	NA	29.6	132	7.42E-30
3859	OSC56312.1	-	terpene cyclase	0	1	0	0	0	0	0	0	0	0	0	1	Unique								
3860	WP_033277993.1	trpS	tryptophan--tRNA ligase	0	1	1	1	1	1	1	1	1	1	1	10	Accessory	COG0180_J#	47.4	300	1.01E-80	K01867	48.5	297	2.27E-79
3861	OSC62224.1	-	4-hydroxy-3-methylbut-2-enyl diphosphate reductase	1	1	1	1	1	1	1	1	1	1	1	11	CORE	COG0761_IM#	71	433	5.83E-121	K03527	71	433	1.56E-120
3862	WP_122215709.1	-	nitronate monooxygenase	0	0	0	0	0	0	0	0	0	1	0	1	Unique	COG2070_R#	52	325	2.24E-88	K00459	45.2	265	5.61E-70
3863	WP_159020913.1	-	hypothetical protein	0	0	0	0	0	0	0	1	0	0	0	1	Unique								

cluster	GI	gene	Annotation	Streptomyces_thermocarboxydus_BPSAC147	Streptomyces_sp._4F	Streptomyces_sp._BSE7F	Streptomyces_sp._ETH9427	Streptomyces_sp._FxanaD5	Streptomyces_sp._UNC401CLCol	Actinospica_acidiphila_NRRL_B_24431	Streptomyces_sp._HNS054	Streptomyces_sp._SMS_SU21	Streptomyces_sp._ZS0098	Streptomyces_thermocarboxydus_K155	Addition	Core-Accessory-Unique	COG	%Similarity_COG	Score_COG	Evalue_COG	KEGG	%Similarity_KEGG	Score_KEGG	Evalue_KEGG
3864	OSC61027.1	-	hydroxyacid dehydrogenase	1	1	0	1	1	1	1	1	1	1	1	10	Accessory	COG0111_HE#	45	275	2.03E-73	K00058	32	146	3.8E-34
3865	OSC59936.1	-	glutamine synthetase	1	1	1	1	1	1	1	1	1	1	1	11	CORE	COG0174_E#	51.6	348	2.47E-95	K01915	53	354	1.2E-96
3866	OSC71773.1	-	ATPase	1	1	1	0	1	1	1	0	1	0	1	8	Accessory	COG1172_G#	38.8	202	2.85E-51	K10440	34.2	168	1.59E-40
3867	PWE07606.1	-	hypothetical protein	0	0	1	1	0	0	0	1	0	0	0	3	Accessory								
3868	AXI84838.1	-	LD-carboxypeptidase	1	0	1	1	1	1	1	1	1	1	1	10	Accessory	COG1619_V#	33.9	195	4.54E-49				
3869	AXI85981.1	-	hypothetical protein	1	1	1	1	1	1	1	1	1	1	1	11	CORE								
3870	Prokka:JAPCLKMA_02254	xlnD	3-hydroxybenzoate 6-hydroxylase 1	1	0	0	1	1	1	1	1	1	1	1	9	Accessory	COG0644_C#	52.5	310	7.42E-84	NA	52.5	310	1.98E-83
3871	WP_122216028.1	-	N-acetyl-gamma-glutamyl-phosphate reductase	1	1	0	1	1	1	1	1	1	1	1	10	Accessory	COG0002_E#	64.5	433	9.91E-121	K00145	64.5	433	2.65E-120
3872	AXI88802.1	-	LLM class flavin-dependent oxidoreductase	1	1	1	1	1	1	1	1	1	1	1	11	CORE	COG2141_C#	27.7	101	5.24E-21	NA	28.3	73	5.32E-12
3873	Prokka:JAPCLKMA_04696	-	hypothetical protein	1	1	1	1	1	1	0	1	1	1	1	10	Accessory	COG0697_GER#	47.4	263	1.36E-69	NA	27.8	96	5.87E-19

cluster	GI	gene	Annotation	Streptomyces_thermocarboxydus_BPSAC147	Streptomyces_sp._4F	Streptomyces_sp._BSE7F	Streptomyces_sp._ETH9427	Streptomyces_sp._FxanaD5	Streptomyces_sp._UNC401CLCol	Actinospica_acidiphila_NRRL_B_24431	Streptomyces_sp._HNS054	Streptomyces_sp._SMS_SU21	Streptomyces_sp._ZS0098	Streptomyces_thermocarboxydus_K155	Addition	Core-Accessory-Unique	COG	%Similarity_COG	Score_COG	Evalue_COG	KEGG	%Similarity_KEGG	Score_KEGG	Evalue_KEGG
3874	WP_122214653.1	-	SGNH/GDSL hydrolase family protein	1	1	0	1	1	1	1	1	1	1	1	10	Accessory	COG2755_E#	39.3	165	3.86E-40	NA	38.7	162	6.67E-39
3875	WP_033272969.1	-	6-phosphofructokinase	1	1	1	1	1	1	1	1	1	1	1	11	CORE	COG0205_G#	56.5	373	9.32E-103	K00850	56.5	373	2.49E-102
3876	WP_048454885.1	-	hypothetical protein	1	0	1	1	1	1	1	1	1	1	1	10	Accessory								
3877	WP_048454924.1	-	glutathione S-transferase	1	1	1	1	1	1	1	1	1	1	1	11	CORE	COG0435_O#	56.6	393	1.14E-108	K07393	45.4	258	1.17E-67
3878	WP_048455193.1	-	helix-turn-helix domain-containing protein	1	1	1	1	1	1	1	1	1	1	1	11	CORE	COG4977_K#	44.9	255	2.17E-67	K02855	38	72	1.19E-11
3879	WP_122214629.1	-	P1 family peptidase	1	1	1	1	1	1	1	1	1	1	1	11	CORE	COG3191_EQ#	45.9	262	2.32E-69	NA	45.9	262	6.19E-69
3880	OSC70303.1	-	hypothetical protein	0	1	0	0	0	0	0	0	0	0	0	1	Unique								
3881	WP_020700073.1	-	LysR family transcriptional regulator	1	0	0	0	1	1	1	0	0	0	1	5	Accessory	COG0583_K#	31.1	94	6.4E-19	NA	28.5	76	4.82E-13
3882	WP_019521315.1	-	threonine/serine dehydratase	1	1	0	1	1	1	1	1	0	1	1	9	Accessory	COG1171_E#	58.4	355	2.01E-97	NA	51	318	7.28E-86
3883	WP_048458801.1	-	zinc-binding dehydrogenase	0	0	1	1	0	0	0	1	1	1	0	5	Accessory	COG0604_CR#	56.6	349	1.1E-95	NA	31.9	154	1.82E-36
3884	WP_019524283.1	-	hypothetical protein	1	1	1	1	1	1	1	1	1	1	1	11	CORE								

cluster	GI	gene	Annotation	Streptomyces_thermocarboxydus_BPSAC147	Streptomyces_sp._4F	Streptomyces_sp._BSE7F	Streptomyces_sp._ETH9427	Streptomyces_sp._FxanaD5	Streptomyces_sp._UNC401CLCol	Actinospica_acidiphila_NRRL_B_24431	Streptomyces_sp._HNS054	Streptomyces_sp._SMS_SU21	Streptomyces_sp._ZS0098	Streptomyces_thermocarboxydus_K155	Addition	Core-Accessory-Unique	COG	%Similarity_COG	Score_COG	Evalue_COG	KEGG	%Similarity_KEGG	Score_KEGG	Evalue_KEGG
3885	WP_051724582.1	-	amino acid ABC transporter permease	1	1	0	0	1	1	1	0	0	0	1	6	Accessory	COG0765_E#	45.6	267	5.52E-71	K02029	32.8	151	1.54E-35
3886	Prokka:CIODPCDJ_05904	iolG_6	Inositol 2-dehydrogenase/D-chiro-inositol 3-dehydrogenase	1	1	1	1	1	1	1	1	1	1	1	11	CORE	COG0673_R#	46.7	292	1.6E-78	K00010	42.1	283	2.59E-75
3887	OSC61244.1	-	LacI family transcriptional regulator	1	1	0	0	0	0	0	0	0	0	0	2	Accessory	COG1609_K#	33.8	165	2.95E-40	K02529	28.2	137	3E-31
3888	WP_033276925.1	-	hypothetical protein	1	1	1	1	1	1	1	1	1	1	1	11	CORE					K13098	36.1	47	0.000409
3889	WP_019526262.1	-	DUF2637 domain-containing protein	0	0	0	0	1	0	0	0	0	0	0	1	Unique								
3890	OSC64820.1	-	hypothetical protein	0	1	0	0	0	0	0	0	0	0	0	1	Unique	COG1275_P#	27.5	59	3.89E-08	NA	26.7	52	0.0000127
3891	OSC65724.1	-	AraC family transcriptional regulator	0	1	0	0	0	0	0	0	0	0	0	1	Unique	COG2207_K#	29.1	101	6.84E-21	K13652	34.1	51	0.0000166
3892	Prokka:CIODPCDJ_06645	-	hypothetical protein	0	0	0	0	0	0	0	0	0	0	1	1	Unique								
3893	WP_084207087.1	-	acyltransferase domain-containing protein	0	0	0	0	0	0	1	0	0	0	0	1	Unique	COG3321_Q#	33.3	167	7.77E-41	K12436	36.3	155	1.07E-36

cluster	GI	gene	Annotation	Streptomyces_thermocarboxydus_BPSAC147	Streptomyces_sp._4F	Streptomyces_sp._BSE7F	Streptomyces_sp._ETH9427	Streptomyces_sp._FxanaD5	Streptomyces_sp._UNC401CLCol	Actinospica_acidiphila_NRRL_B_24431	Streptomyces_sp._HNS054	Streptomyces_sp._SMS_SU21	Streptomyces_sp._ZS0098	Streptomyces_thermocarboxydus_K155	Addition	Core-Accessory-Unique	COG	%Similarity_COG	Score_COG	Evalue_COG	KEGG	%Similarity_KEGG	Score_KEGG	Evalue_KEGG
3894	WP_033277703.1	-	NADP-dependent oxidoreductase	0	0	0	0	0	0	1	0	0	0	0	1	Unique	COG2130_R#	39.3	207	8.85E-53	K07119	32.2	174	1.7E-42
3895	WP_019522564.1	-	amidohydrolase	0	0	1	0	1	1	1	0	1	0	1	6	Accessory								
3896	WP_019526424.1	-	SDR family oxidoreductase	1	0	1	1	1	1	1	1	1	1	1	10	Accessory	COG0451_MG#	49.9	329	1.54E-89	NA	31.9	125	9.01E-28
3897	OSC71777.1	-	LacI family transcriptional regulator	1	1	1	1	1	1	1	1	1	1	1	11	CORE	COG1609_K#	38.8	212	2.1E-54	NA	34.3	193	2.7E-48
3898	Prokka:JAPCLKMA_01593	smc_3	Chromosome partition protein Smc	1	0	0	0	0	0	0	0	0	0	0	1	Unique	COG0791_M#	29.8	142	2.67E-33	NA	24.6	111	1.76E-23
3899	Prokka:JAPCLKMA_02614	iolG_8	Inositol 2-dehydrogenase/D-chiro-inositol 3-dehydrogenase	1	0	0	0	0	0	0	0	0	0	0	1	Unique	COG0673_R#	34.1	178	3.36E-44	K03810	30.7	137	2.99E-31
3900	Prokka:CIODPCDJ_02441	-	hypothetical protein	0	0	0	0	0	1	0	0	0	0	1	2	Accessory								
3901	OSC59424.1	-	MerR family transcriptional regulator	1	1	1	1	1	1	1	1	1	1	1	11	CORE	COG0789_K#	43.8	235	3.95E-61	NA	32	71	2.02E-11
3902	OSC70186.1	-	hypothetical protein	0	1	0	0	0	0	0	0	0	0	0	1	Unique	COG3386_G#	27.9	111	8.6E-24				

cluster	GI	gene	Annotation	Streptomyces_thermocarboxydus_BPSAC147	Streptomyces_sp._4F	Streptomyces_sp._BSE7F	Streptomyces_sp._ETH9427	Streptomyces_sp._FxanaD5	Streptomyces_sp._UNC401CLCol	Actinospica_acidiphila_NRRL_B_24431	Streptomyces_sp._HNS054	Streptomyces_sp._SMS_SU21	Streptomyces_sp._ZS0098	Streptomyces_thermocarboxydus_K155	Addition	Core-Accessory-Unique	COG	%Similarity_COG	Score_COG	Evalue_COG	KEGG	%Similarity_KEGG	Score_KEGG	Evalue_KEGG
3903	Prokka:JAPCLKMA_03476	btuD_11	Vitamin B12 import ATP-binding protein BtuD	1	1	1	1	1	1	1	1	1	1	1	11	CORE	COG4586_R#	54.4	304	4.06E-82	K01990	54.4	304	1.08E-81
3904	WP_122212636.1	-	hypothetical protein	1	1	1	1	1	1	1	1	1	1	1	11	CORE					NA	31	62	7.18E-09
3905	OSC57446.1	-	hypothetical protein	0	1	0	0	0	0	0	0	0	0	0	1	Unique								
3906	WP_019521536.1	-	LLM class F420-dependent oxidoreductase	1	0	0	0	1	1	1	0	0	0	1	5	Accessory	COG2141_C#	39.9	237	7.96E-62	NA	39.9	237	2.13E-61
3907	OSC69114.1	-	bicyclomycin resistance protein	0	1	0	0	0	0	0	0	0	0	0	1	Unique	COG0715_P#	59	356	1.18E-97	K02051	26.9	107	2.54E-22
3908	Prokka:CIODPCDJ_05983	sigJ_4	ECF RNA polymerase sigma factor SigJ	1	1	1	1	1	1	1	1	1	1	1	11	CORE	COG1595_K#	44.9	232	1.96E-60	K03088	36.8	171	1.43E-41
3909	OSC71905.1	-	dehydrogenase	1	1	1	1	1	1	1	1	1	1	1	11	CORE	COG0673_R#	44.4	300	7.66E-81	K00010	33.1	191	1.02E-47
3910	OSC66754.1	-	hypothetical protein	0	1	0	0	0	0	0	0	0	0	0	1	Unique	COG2207_K#	28	95	3.74E-19				
3911	OSC60426.1	-	hypothetical protein	0	1	0	0	0	0	0	0	0	0	0	1	Unique								
3912	OSC58452.1	-	ABC transporter substrate-binding protein	0	1	0	0	0	0	1	1	0	0	1	4	Accessory	COG4558_P#	35.8	203	1.66E-51	K02016	24.3	102	1.07E-20
3913	OSC63275.1	-	ATP-dependent DNA ligase	1	1	1	1	1	1	1	1	1	1	1	11	CORE	COG3285_L#	60	395	1.75E-109	NA	60	395	4.67E-109

cluster	GI	gene	Annotation	Streptomyces_thermocarboxydus_BPSAC147	Streptomyces_sp._4F	Streptomyces_sp._BSE7F	Streptomyces_sp._ETH9427	Streptomyces_sp._FxanaD5	Streptomyces_sp._UNC401CLCol	Actinospica_acidiphila_NRRL_B_24431	Streptomyces_sp._HNS054	Streptomyces_sp._SMS_SU21	Streptomyces_sp._ZS0098	Streptomyces_thermocarboxydus_K155	Addition	Core-Accessory-Unique	COG	%Similarity_COG	Score_COG	Evalue_COG	KEGG	%Similarity_KEGG	Score_KEGG	Evalue_KEGG
3914	PWE12190.1	-	LacI family transcriptional regulator	1	0	1	1	1	1	1	1	1	1	1	10	Accessory	COG1609_K#	33.7	193	1.31E-48	NA	27.1	166	3.52E-40
3915	OSC59898.1	-	sugar ABC transporter substrate-binding protein	1	1	1	1	1	1	1	1	1	1	1	11	CORE	COG1879_G#	54.1	299	1.7E-80				
3916	WP_048456501.1	-	alcohol dehydrogenase catalytic domain-containing protein	1	0	1	1	1	1	1	1	1	1	1	10	Accessory	COG1064_R#	43.6	288	3.92E-77	K12957	35	194	2.06E-48
3917	PWE08556.1	-	IS481 family transposase	0	0	1	1	1	1	1	1	1	0	1	8	Accessory	COG2801_L#	43.5	229	2.16E-59	NA	30.6	119	6.44E-26
3918	OSC71519.1	-	AraC family transcriptional regulator	1	1	1	1	1	1	1	1	1	1	1	11	CORE	COG4977_K#	47.1	267	7.16E-71	NA	33.3	83	3.92E-15
3919	WP_102638630.1	adhP	alcohol dehydrogenase AdhP	0	0	1	1	0	0	0	1	1	1	0	5	Accessory	COG1064_R#	54.6	364	3.3E-100	K13953	45.8	291	9.47E-78
3920	Prokka:JAPCLKMA_01556	-	hypothetical protein	1	0	0	1	0	0	0	0	0	0	0	2	Accessory	COG0515_RTKL#	33.3	44	0.000755				
3921	Prokka:CIODPCDJ_04002	rpoA	DNA-directed RNA polymerase subunit alpha	1	1	0	1	1	1	1	1	1	1	1	10	Accessory	COG0202_K#	75.6	513	4.39E-145	K03040	75.6	513	1.17E-144

cluster	GI	gene	Annotation	Streptomyces_thermocarboxydus_BPSAC147	Streptomyces_sp._4F	Streptomyces_sp._BSE7F	Streptomyces_sp._ETH9427	Streptomyces_sp._FxanaD5	Streptomyces_sp._UNC401CLCol	Actinospica_acidiphila_NRRL_B_24431	Streptomyces_sp._HNS054	Streptomyces_sp._SMS_SU21	Streptomyces_sp._ZS0098	Streptomyces_thermocarboxydus_K155	Addition	Core-Accessory-Unique	COG	%Similarity_COG	Score_COG	Evalue_COG	KEGG	%Similarity_KEGG	Score_KEGG	Evalue_KEGG
3922	WP_102641158.1	-	NADP-dependent oxidoreductase	1	1	0	1	1	1	1	1	1	1	1	10	Accessory	COG2130_R#	51.8	324	6.45E-88	K07119	50.7	322	5.01E-87
3923	WP_048455041.1	-	cysteine synthase family protein	1	1	0	1	1	1	1	1	0	1	1	9	Accessory	COG0031_E#	33.8	179	2.57E-44	K01738	28	109	6.67E-23
3924	AXI85931.1	-	alcohol dehydrogenase	0	0	1	1	0	0	0	1	1	0	0	4	Accessory	COG0604_CR#	40.9	229	1.65E-59	NA	31	89	7.14E-17
3925	PWE10425.1	hpnH	adenosyl-hopene transferase HpnH	1	1	1	1	1	1	1	1	1	1	1	11	CORE	COG0535_R#	54.9	388	2.13E-107	NA	52.8	385	4.82E-106
3926	WP_122215530.1	-	sigma-70 family RNA polymerase sigma factor	1	1	0	1	1	1	1	1	1	1	1	10	Accessory	COG1595_K#	44.1	241	4.21E-63	K03088	44.1	241	1.12E-62
3927	PWE06753.1	-	acyl-CoA dehydrogenase	1	1	1	1	1	1	1	1	1	1	1	11	CORE	COG3173_R#	43.2	264	6.06E-70	K00257	43.2	264	1.62E-69
3928	Prokka:CIODPCDJ_04128	trpS_2	Tryptophan--tRNA ligase	1	1	1	1	1	1	1	1	1	1	1	11	CORE	COG0180_J#	62.5	429	1.42E-119	K01867	61	408	6.94E-113
3929	WP_033277829.1	-	LacI family DNA-binding transcriptional regulator	1	1	1	1	1	1	1	1	1	1	1	11	CORE	COG1609_K#	37.9	197	9.1E-50	NA	33.5	182	8.09E-45
3930	WP_048458057.1	-	aldo/keto reductase	1	1	1	1	1	1	1	1	1	1	1	11	CORE	COG0667_C#	40.1	228	2.82E-59	NA	39.8	216	5.06E-55

cluster	GI	gene	Annotation	Streptomyces_thermocarboxydus_BPSAC147	Streptomyces_sp._4F	Streptomyces_sp._BSE7F	Streptomyces_sp._ETH9427	Streptomyces_sp._FxanaD5	Streptomyces_sp._UNC401CLCol	Actinospica_acidiphila_NRRL_B_24431	Streptomyces_sp._HNS054	Streptomyces_sp._SMS_SU21	Streptomyces_sp._ZS0098	Streptomyces_thermocarboxydus_K155	Addition	Core-Accessory-Unique	COG	%Similarity_COG	Score_COG	Evalue_COG	KEGG	%Similarity_KEGG	Score_KEGG	Evalue_KEGG
3931	OSC72009.1	-	2,3-diaminopropionate biosynthesis protein SbnB	0	1	0	0	0	0	0	0	0	0	0	1	Unique	COG2423_E#	27.5	126	1.51E-28				
3932	WP_026243621.1	-	DUF4349 domain-containing protein	1	1	1	1	1	1	1	1	1	1	1	11	CORE	COG0845_M#	25	46	0.000259				
3933	WP_033277064.1	-	fibronectin type III domain-containing protein	1	1	1	1	1	1	1	1	1	1	1	11	CORE								
3934	OSC61315.1	-	adenosine deaminase	0	1	0	0	0	0	0	0	0	0	0	1	Unique	COG1816_F#	56	363	7.34E-100	K01488	44.1	267	1.91E-70
3935	OSC66332.1	-	inositol 2-dehydrogenase	0	1	0	0	0	0	0	0	0	0	0	1	Unique	COG0673_R#	60.4	417	5.6E-116	K00010	53	379	3.45E-104
3936	OSC62909.1	-	LLM class F420-dependent oxidoreductase	1	1	1	1	1	1	1	1	1	1	1	11	CORE	COG2141_C#	30.2	124	9.79E-28	K00320	34.2	113	4.62E-24
3937	OSC61918.1	-	hypothetical protein	1	1	1	1	1	1	1	1	1	1	1	11	CORE					NA	32.2	50	0.0000281
3938	WP_086020089.1	-	protoheme IX farnesyltransferase	1	1	1	1	1	1	1	1	1	1	1	11	CORE	COG0109_O#	48.3	290	6.05E-78	K02301	48	283	2.58E-75
3939	WP_167338167.1	-	LysR family transcriptional regulator	1	1	1	1	0	1	1	1	1	1	1	10	Accessory	COG0583_K#	50.3	279	1.82E-74	K04761	42.9	220	2.05E-56

cluster	GI	gene	Annotation	Streptomyces_thermocarboxydus_BPSAC147	Streptomyces_sp._4F	Streptomyces_sp._BSE7F	Streptomyces_sp._ETH9427	Streptomyces_sp._FxanaD5	Streptomyces_sp._UNC401CLCol	Actinospica_acidiphila_NRRL_B_24431	Streptomyces_sp._HNS054	Streptomyces_sp._SMS_SU21	Streptomyces_sp._ZS0098	Streptomyces_thermocarboxydus_K155	Addition	Core-Accessory-Unique	COG	%Similarity_COG	Score_COG	Evalue_COG	KEGG	%Similarity_KEGG	Score_KEGG	Evalue_KEGG
3940	Prokka:JAPCLKMA_02286	-	Beta-peptidyl aminopeptidase BapA	1	1	1	1	1	1	1	1	1	1	1	11	CORE	COG3191_EQ#	50.9	296	1.44E-79				
3941	WP_033275725.1	-	hypothetical protein	0	0	0	0	1	1	1	0	0	0	1	4	Accessory					K11651	36.4	72	1.18E-11
3942	WP_019523479.1	-	hypothetical protein	1	0	1	1	1	1	1	1	1	1	1	10	Accessory								
3943	Prokka:CIODPCDJ_04259	-	hypothetical protein	0	0	0	0	0	0	0	0	0	0	1	1	Unique								
3944	AXI86705.1	-	rod shape-determining protein	1	1	1	1	1	1	1	1	1	1	1	11	CORE	COG1077_D#	65.1	430	6.37E-120	K03569	65.1	430	1.7E-119
3945	WP_019521350.1	-	LLM class flavin-dependent oxidoreductase	1	1	1	1	1	1	1	1	1	1	1	11	CORE	COG2141_C#	42.1	224	6.93E-58	NA	38.9	215	6.58E-55
3946	AXI89285.1	-	hypothetical protein	1	0	0	1	0	0	0	1	0	0	0	3	Accessory	COG5464_S#	36.2	50	0.0000105	NA	34.7	45	0.00118
3947	WP_167338203.1	-	helix-turn-helix transcriptional regulator	1	1	1	1	1	1	1	1	1	1	1	11	CORE								
3948	WP_019521445.1	-	DUF2891 domain-containing protein	1	0	0	0	1	1	0	0	0	0	1	4	Accessory								
3949	WP_102637312.1	-	LacI family transcriptional regulator	0	0	1	0	0	0	0	1	1	1	0	4	Accessory	COG1609_K#	33.9	184	7.95E-46	K05499	36.1	178	1.17E-43
3950	WP_028961611.1	-	esterase	0	0	0	0	1	1	1	0	0	0	1	4	Accessory	COG0627_R#	30.3	172	3.13E-42	NA	31.4	105	1.25E-21

cluster	GI	gene	Annotation	Streptomyces_thermocarboxydus_BPSAC147	Streptomyces_sp._4F	Streptomyces_sp._BSE7F	Streptomyces_sp._ETH9427	Streptomyces_sp._FxanaD5	Streptomyces_sp._UNC401CLCol	Actinospica_acidiphila_NRRL_B_24431	Streptomyces_sp._HNS054	Streptomyces_sp._SMS_SU21	Streptomyces_sp._ZS0098	Streptomyces_thermocarboxydus_K155	Addition	Core-Accessory-Unique	COG	%Similarity_COG	Score_COG	Evalue_COG	KEGG	%Similarity_KEGG	Score_KEGG	Evalue_KEGG
3951	WP_019527304.1	-	sugar ABC transporter permease	1	0	0	0	1	1	1	0	1	1	1	7	Accessory	COG1175_G#	31.8	158	3.58E-38	K02025	35.8	150	2.6E-35
3952	WP_122214414.1	-	HlyC/CorC family transporter	0	1	1	1	1	1	0	1	1	1	1	9	Accessory	COG1253_R#	33.8	195	4.5E-49	NA	34.6	186	5.59E-46
3953	WP_033273691.1	-	ABC transporter permease	1	1	1	1	1	1	1	1	1	1	1	11	CORE	COG1172_G#	40.7	218	3.8E-56	K10440	40.7	211	1.24E-53
3954	WP_078895083.1	-	cell wall assembly protein Knr4	1	1	1	1	1	1	1	1	1	1	1	11	CORE								
3955	Prokka:CIODPCDJ_06371	-	hypothetical protein	1	0	1	1	1	0	0	1	0	1	1	7	Accessory	COG4372_S#	25.4	53	0.00000212	NA	33.6	62	1.22E-08
3956	OSC56247.1	-	peptidase P60	1	1	1	1	1	1	1	1	1	1	1	11	CORE	COG0791_M#	38.9	98	4.39E-20	NA	38.9	98	1.17E-19
3957	WP_028961283.1	-	glycosyltransferase family 4 protein	0	0	0	0	0	1	0	0	0	0	0	1	Unique	COG0438_M#	24	70	9.81E-12	K16150	28	58	1.35E-07
3958	OSC69951.1	-	nucleoside-diphosphate kinase	0	1	0	0	0	0	0	0	0	0	0	1	Unique	COG0105_F#	27.7	65	5.38E-10	K00940	35.6	75	1.06E-12
3959	Prokka:CIODPCDJ_01995	iolS_2	Aldo-keto reductase IolS	1	1	1	1	1	1	1	1	1	1	1	11	CORE	COG0667_C#	50.8	299	1.3E-80	NA	50.8	299	3.47E-80
3960	OSC71865.1	-	amidohydrolase	0	1	0	0	0	0	0	0	0	0	0	1	Unique					NA	34.2	187	2.51E-46
3961	Prokka:JAPCLKMA_01647	-	55.5 kDa and 49.5 kDa sporulation proteins	1	0	0	0	0	0	0	0	0	0	0	1	Unique								

cluster	GI	gene	Annotation	Streptomyces_thermocarboxydus_BPSAC147	Streptomyces_sp._4F	Streptomyces_sp._BSE7F	Streptomyces_sp._ETH9427	Streptomyces_sp._FxanaD5	Streptomyces_sp._UNC401CLCol	Actinospica_acidiphila_NRRL_B_24431	Streptomyces_sp._HNS054	Streptomyces_sp._SMS_SU21	Streptomyces_sp._ZS0098	Streptomyces_thermocarboxydus_K155	Addition	Core-Accessory-Unique	COG	%Similarity_COG	Score_COG	Evalue_COG	KEGG	%Similarity_KEGG	Score_KEGG	Evalue_KEGG
3962	WP_033273793.1	-	prolyl oligopeptidase family serine peptidase	1	1	1	1	1	1	1	1	1	1	1	11	CORE	COG3458_Q#	47.5	314	5.09E-85	K01060	37.6	176	5.78E-43
3963	OSC61859.1	-	sugar ABC transporter substrate-binding protein	0	1	0	0	0	0	0	0	0	0	0	1	Unique	COG0609_P#	43.5	276	1.54E-73	K02015	39.1	208	1.05E-52
3964	WP_122215722.1	-	alcohol dehydrogenase catalytic domain-containing protein	0	0	0	0	0	0	0	0	0	1	0	1	Unique	COG1064_R#	50.1	333	8.11E-91	K12957	34.6	198	8.33E-50
3965	OSC67539.1	-	ATP/GTP-binding protein	0	1	0	0	0	0	0	0	0	0	0	1	Unique								
3966	Prokka:JAPCLKMA_02007	phzM	Phenazine-1-carboxylate N-methyltransferase	1	0	0	0	0	0	0	0	0	0	0	1	Unique	COG0500_QR#	42.6	286	1.49E-76				
3967	WP_048458562.1	-	hypothetical protein	0	0	0	0	0	0	0	1	0	0	0	1	Unique								
3968	OSC62452.1	-	TIGR02452 family protein	1	1	1	0	1	1	1	1	1	1	1	10	Accessory	COG4295_S#	41.7	213	1.6E-54	NA	35.2	155	8.09E-37
3969	Prokka:CIODPCDJ_00357	-	hypothetical protein	0	1	1	1	1	1	1	1	1	1	1	10	Accessory								

cluster	GI	gene	Annotation	Streptomyces_thermocarboxydus_BPSAC147	Streptomyces_sp._4F	Streptomyces_sp._BSE7F	Streptomyces_sp._ETH9427	Streptomyces_sp._FxanaD5	Streptomyces_sp._UNC401CLCol	Actinospica_acidiphila_NRRL_B_24431	Streptomyces_sp._HNS054	Streptomyces_sp._SMS_SU21	Streptomyces_sp._ZS0098	Streptomyces_thermocarboxydus_K155	Addition	Core-Accessory-Unique	COG	%Similarity_COG	Score_COG	Evalue_COG	KEGG	%Similarity_KEGG	Score_KEGG	Evalue_KEGG
3970	WP_048457872.1	-	aspartate-semialdehyde dehydrogenase	1	1	1	1	1	1	1	1	1	1	1	11	CORE	COG0136_E#	67.1	428	3.15E-119	K00133	67.1	428	8.42E-119
3971	WP_051724474.1	-	phytoene/squalene synthase family protein	1	1	1	1	1	1	1	1	1	1	1	11	CORE	COG1562_I#	40.7	154	6.73E-37	K02291	36	150	2.59E-35
3972	WP_048456657.1	-	asparaginase	1	0	1	1	1	1	1	1	1	1	1	10	Accessory	COG4448_E#	36.9	171	4.07E-42	K01424	32.5	127	2.35E-28
3973	WP_019524031.1	-	hypothetical protein	1	0	0	0	1	0	0	0	0	0	0	2	Accessory								
3974	WP_019521588.1	-	Gfo/Idh/MocA family oxidoreductase	1	0	0	1	1	1	1	1	1	0	1	8	Accessory	COG0673_R#	34.1	179	2.55E-44	K00078	36.2	133	3.28E-30
3975	Prokka:JAPCLKMA_00916	-	hypothetical protein	1	0	0	0	1	1	1	0	0	0	1	5	Accessory	COG0765_E#	52.1	279	1.81E-74	K02029	31.8	141	2.06E-32
3976	OSC69571.1	-	RNase adaptor protein RapZ	1	1	1	1	1	1	1	1	1	1	1	11	CORE	COG1660_R#	54.9	320	9.26E-87	K06958	54.9	320	2.47E-86
3977	WP_019525755.1	-	LacI family transcriptional regulator	1	0	1	1	1	1	1	1	1	1	1	10	Accessory	COG1609_K#	34.9	185	3.56E-46	NA	34.9	185	9.5E-46
3978	AXI86662.1	-	heat-inducible transcriptional repressor HrcA	1	1	1	1	1	1	1	1	1	1	1	11	CORE	COG1420_K#	58.2	370	5.97E-102	K03705	58.2	370	1.59E-101

cluster	GI	gene	Annotation	Streptomyces_thermocarboxydus_BPSAC147	Streptomyces_sp._4F	Streptomyces_sp._BSE7F	Streptomyces_sp._ETH9427	Streptomyces_sp._FxanaD5	Streptomyces_sp._UNC401CLCol	Actinospica_acidiphila_NRRL_B_24431	Streptomyces_sp._HNS054	Streptomyces_sp._SMS_SU21	Streptomyces_sp._ZS0098	Streptomyces_thermocarboxydus_K155	Addition	Core-Accessory-Unique	COG	%Similarity_COG	Score_COG	Evalue_COG	KEGG	%Similarity_KEGG	Score_KEGG	Evalue_KEGG
3979	WP_114872613.1	-	thiamine pyrophosphate-dependent dehydrogenase E1 component subunit alpha	0	0	1	1	0	0	0	1	1	1	0	5	Accessory	COG1071_C#	37.6	168	5.88E-41	K00161	35.6	160	3.27E-38
3980	WP_053076987.1	-	hypothetical protein	0	0	0	0	0	0	0	1	0	0	0	1	Unique								
3981	OSC72367.1	-	hypothetical protein	0	1	0	0	0	0	0	0	0	0	0	1	Unique								
3982	WP_028959253.1	-	alpha/beta hydrolase	1	0	1	1	1	1	1	1	1	1	1	10	Accessory	COG0596_R#	31.3	64	1.2E-09				
3983	OSC66255.1	-	3-hydroxybutyryl-CoA dehydrogenase	0	1	0	0	0	0	0	0	0	0	0	1	Unique	COG1250_I#	54.6	314	3.89E-85	K13247	31.6	141	1.57E-32
3984	WP_048457172.1	paaA	1,2-phenylacetyl-CoA epoxidase subunit A	1	1	1	1	0	1	1	1	1	1	0	9	Accessory	COG3396_S#	70.6	481	2.4E-135	K02609	68.7	445	3.9E-124
3985	OSC70130.1	-	glycosyltransferase	0	1	0	0	0	0	0	0	0	0	0	1	Unique	COG0463_M#	42.1	245	3.78E-64	K00721	42.5	242	8.55E-63
3986	Prokka:CIODPCDJ_00904	ppsE	Phthiocerol synthesis polyketide synthase type I PpsE	0	0	0	0	1	1	0	0	0	0	1	3	Accessory	COG3321_Q#	36.5	149	2.83E-35	K12430	32.5	130	3.63E-29
3987	WP_028961035.1	-	TIGR01777 family protein	0	0	0	1	1	1	1	1	0	1	1	7	Accessory	COG1090_R#	41	240	7.14E-63	K07071	31.7	149	5.78E-35

cluster	GI	gene	Annotation	Streptomyces_thermocarboxydus_BPSAC147	Streptomyces_sp._4F	Streptomyces_sp._BSE7F	Streptomyces_sp._ETH9427	Streptomyces_sp._FxanaD5	Streptomyces_sp._UNC401CLCol	Actinospica_acidiphila_NRRL_B_24431	Streptomyces_sp._HNS054	Streptomyces_sp._SMS_SU21	Streptomyces_sp._ZS0098	Streptomyces_thermocarboxydus_K155	Addition	Core-Accessory-Unique	COG	%Similarity_COG	Score_COG	Evalue_COG	KEGG	%Similarity_KEGG	Score_KEGG	Evalue_KEGG
3988	OSC58009.1	-	glycosylase	0	1	0	0	0	0	0	0	0	0	0	1	Unique					K09955	33.7	75	1.38E-12
3989	WP_028959591.1	-	helix-turn-helix transcriptional regulator	1	0	0	0	1	1	1	0	0	0	1	5	Accessory								
3990	WP_048460479.1	-	hypothetical protein	1	0	1	1	1	1	1	1	0	1	1	9	Accessory					NA	27.7	51	0.0000214
3991	OSC69690.1	-	hypothetical protein	0	1	0	0	0	0	0	0	0	0	0	1	Unique								
3992	OSC66884.1	-	hypothetical protein	0	1	0	0	0	0	0	0	0	0	0	1	Unique	COG3321_Q#	31.7	72	4.39E-12				
3993	OSC56431.1	-	phosphotransferase	0	1	0	0	0	0	0	0	0	0	0	1	Unique								
3994	OSC54334.1	-	RNA polymerase subunit sigma	0	1	0	0	0	0	0	0	0	0	0	1	Unique	COG0568_K#	52.6	307	8.11E-83	K03087	52.6	307	2.17E-82
3995	WP_048456323.1	-	HlyC/CorC family transporter	1	1	1	1	1	1	1	1	1	1	1	11	CORE	COG1253_R#	42.7	246	1.3E-64	NA	42.7	245	5.91E-64
3996	WP_019521465.1	-	2Fe-2S iron-sulfur cluster binding domain-containing protein	1	0	0	0	1	1	0	0	0	0	1	4	Accessory	COG0543_HC#	41.5	285	3.29E-76	K00523	37.8	224	1.41E-57
3997	WP_102641602.1	-	C40 family peptidase	1	1	1	1	1	1	1	1	1	1	1	11	CORE	COG0791_M#	31.3	156	1.35E-37	NA	48.7	124	2.59E-27
3998	OSC72952.1	-	transcriptional regulator	0	1	0	0	0	0	0	0	0	0	0	1	Unique	COG2378_K#	26.3	73	1.5E-12	NA	23.8	62	1.21E-08
3999	OSC62743.1	-	hypothetical protein	0	1	0	0	0	0	0	0	0	0	0	1	Unique								

cluster	GI	gene	Annotation	Streptomyces_thermocarboxydus_BPSAC147	Streptomyces_sp._4F	Streptomyces_sp._BSE7F	Streptomyces_sp._ETH9427	Streptomyces_sp._FxanaD5	Streptomyces_sp._UNC401CLCol	Actinospica_acidiphila_NRRL_B_24431	Streptomyces_sp._HNS054	Streptomyces_sp._SMS_SU21	Streptomyces_sp._ZS0098	Streptomyces_thermocarboxydus_K155	Addition	Core-Accessory-Unique	COG	%Similarity_COG	Score_COG	Evalue_COG	KEGG	%Similarity_KEGG	Score_KEGG	Evalue_KEGG
4000	OSC72008.1	-	2,3-diaminopropionate biosynthesis protein SbnA	0	1	0	0	0	0	0	0	0	0	0	1	Unique	COG0031_E#	47.3	270	1.1E-71	K01738	40	190	2.95E-47
4001	AXI91291.1	-	ATPase	0	0	0	1	0	0	0	0	0	0	0	1	Unique					NA	32.4	84	2.28E-15
4002	OSC70418.1	-	epoxide hydrolase	1	1	1	1	1	1	1	1	1	1	1	11	CORE	COG0596_R#	44.4	287	5.08E-77	NA	44.4	287	1.36E-76
4003	OSC66967.1	-	ribosome small subunit-dependent GTPase A	1	1	1	1	1	1	1	1	1	1	1	11	CORE	COG1162_R#	53.8	315	1.74E-85	K06949	53.1	313	2.31E-84
4004	AXI85300.1	-	glycerol-3-phosphate acyltransferase	1	1	1	1	1	1	1	1	1	1	1	11	CORE	COG0240_C#	53.8	329	1.52E-89	K00057	50.9	327	1.18E-88
4005	OSC54489.1	-	serine/threonine protein kinase	0	1	0	0	0	0	0	0	0	0	0	1	Unique	COG0515_RTKL#	33.4	142	2.64E-33	K08884	35.1	138	1.33E-31
4006	Prokka:CIODPCDJ_06277	adh_4	Alcohol dehydrogenase	0	0	0	0	0	0	1	0	0	0	1	2	Accessory	COG1064_R#	39.5	233	1.48E-60	K00083	34.6	198	8.28E-50
4007	OSC61214.1	-	zinc-binding dehydrogenase	0	1	0	0	0	0	0	0	0	0	0	1	Unique	COG1064_R#	33.7	134	9.38E-31	NA	27.7	146	6.37E-34
4008	WP_051724567.1	-	WYL domain-containing protein	1	0	0	0	1	1	1	0	0	1	1	6	Accessory	COG2378_K#	28.7	152	3.33E-36	NA	25.4	102	6.18E-21

cluster	GI	gene	Annotation	Streptomyces_thermocarboxydus_BPSAC147	Streptomyces_sp._4F	Streptomyces_sp._BSE7F	Streptomyces_sp._ETH9427	Streptomyces_sp._FxanaD5	Streptomyces_sp._UNC401CLCol	Actinospica_acidiphila_NRRL_B_24431	Streptomyces_sp._HNS054	Streptomyces_sp._SMS_SU21	Streptomyces_sp._ZS0098	Streptomyces_thermocarboxydus_K155	Addition	Core-Accessory-Unique	COG	%Similarity_COG	Score_COG	Evalue_COG	KEGG	%Similarity_KEGG	Score_KEGG	Evalue_KEGG
4009	OSC63662.1	-	phosphate ABC transporter permease subunit PstC	1	1	1	1	1	1	1	1	1	1	1	11	CORE	COG0573_P#	47.1	274	5.81E-73	K02037	45.5	260	2.32E-68
4010	Prokka:CIODPCDJ_04895	ccpA_7	Catabolite control protein A	1	1	1	1	1	1	1	1	1	1	1	11	CORE	COG1609_K#	30.9	130	1.35E-29	K02529	29.2	116	7.04E-25
4011	WP_106432551.1	-	hypothetical protein	0	0	0	0	1	0	1	0	0	0	0	2	Accessory								
4012	WP_028959810.1	-	NAD-dependent epimerase/dehydratase family protein	1	1	1	1	1	1	1	1	1	1	1	11	CORE								
4013	WP_048457534.1	-	zinc ABC transporter substrate-binding protein	1	1	1	1	1	1	1	1	1	1	1	11	CORE	COG0803_P#	36.5	205	2.53E-52	K09815	33.5	178	8.84E-44
4014	Prokka:JAPCLKMA_05791	tal_2	dTDP-6-deoxy-L-talose 4-dehydrogenase (NAD(P)(+))	1	1	1	1	1	1	1	1	1	1	1	11	CORE	COG0451_MG#	38.5	155	3E-37	NA	38.5	155	8.02E-37
4015	WP_019524500.1	-	polyprenyl synthetase family protein	1	1	1	1	1	1	1	1	1	1	1	11	CORE	COG0142_H#	47.6	285	1.92E-76	K00805	47.6	285	5.14E-76
4016	WP_078895336.1	-	methyltransferase	0	0	1	1	0	0	0	1	1	1	0	5	Accessory	COG0500_QR#	28.9	122	3.68E-27	NA	30.1	123	4.41E-27

cluster	GI	gene	Annotation	Streptomyces_thermocarboxydus_BPSAC147	Streptomyces_sp._4F	Streptomyces_sp._BSE7F	Streptomyces_sp._ETH9427	Streptomyces_sp._FxanaD5	Streptomyces_sp._UNC401CLCol	Actinospica_acidiphila_NRRL_B_24431	Streptomyces_sp._HNS054	Streptomyces_sp._SMS_SU21	Streptomyces_sp._ZS0098	Streptomyces_thermocarboxydus_K155	Addition	Core-Accessory-Unique	COG	%Similarity_COG	Score_COG	Evalue_COG	KEGG	%Similarity_KEGG	Score_KEGG	Evalue_KEGG
4017	Prokka:CIODPCDJ_06536	glmS_3	Glutamine--fructose-6-phosphate aminotransferase [isomerizing]	1	1	1	1	1	1	1	1	1	1	1	11	CORE	COG0449_M#	29.9	97	7.43E-20	K00820	29.9	97	1.98E-19
4018	WP_033277676.1	-	transporter	0	0	0	0	0	0	1	0	0	0	0	1	Unique								
4019	Prokka:CIODPCDJ_00129	nlhH_1	Carboxylesterase NlhH	1	0	0	0	1	1	0	0	0	0	1	4	Accessory	COG0657_I#	39.1	177	9.63E-44	NA	39.1	177	2.57E-43
4020	WP_019525171.1	-	sugar ABC transporter permease	1	1	1	1	1	1	1	1	1	1	1	11	CORE	COG1175_G#	39.8	235	2.28E-61	K17245	34.4	174	1.28E-42
4021	WP_122217208.1	-	aldo/keto reductase	1	0	1	1	1	1	1	1	1	1	1	10	Accessory	COG0667_C#	48.5	293	9.22E-79	NA	48.5	293	2.46E-78
4022	Prokka:CIODPCDJ_05979	egtB_1	Hercynine oxygenase	1	0	0	0	1	1	0	0	0	0	1	4	Accessory	COG1262_S#	31.2	99	1.95E-20	K18912	31.2	99	8.9E-20
4023	OSC71302.1	-	transcription termination /antitermination protein NusA	1	1	1	1	1	1	1	1	1	1	1	11	CORE	COG0195_K#	63.6	400	7E-111	K02600	63.6	400	1.87E-110
4024	WP_033276427.1	-	aldo/keto reductase	0	0	0	0	0	0	1	0	0	1	0	2	Accessory	COG0667_C#	46.7	276	1.17E-73	NA	26.5	105	9.51E-22
4025	Prokka:JAPCLKMA_04654	-	hypothetical protein	1	0	0	0	1	1	0	0	0	0	1	4	Accessory	COG0601_EP#	53.9	112	2.91E-24	K12369	34.8	94	2.19E-18
4026	AXI88190.1	-	regulator	0	0	0	1	1	0	0	0	0	0	0	2	Accessory								
4027	OSC62772.1	-	hypothetical protein	0	1	0	0	0	0	0	0	0	0	0	1	Unique	COG2771_K#	26.4	42	0.00484				

cluster	GI	gene	Annotation	Streptomyces_thermocarboxydus_BPSAC147	Streptomyces_sp._4F	Streptomyces_sp._BSE7F	Streptomyces_sp._ETH9427	Streptomyces_sp._FxanaD5	Streptomyces_sp._UNC401CLCol	Actinospica_acidiphila_NRRL_B_24431	Streptomyces_sp._HNS054	Streptomyces_sp._SMS_SU21	Streptomyces_sp._ZS0098	Streptomyces_thermocarboxydus_K155	Addition	Core-Accessory-Unique	COG	%Similarity_COG	Score_COG	Evalue_COG	KEGG	%Similarity_KEGG	Score_KEGG	Evalue_KEGG
4028	OSC61856.1	-	iron transporter	0	1	0	0	0	0	0	0	0	0	0	1	Unique	COG0614_P#	35.5	166	2.22E-40				
4029	WP_084207082.1	-	4'-phosphopantetheinyl transferase superfamily protein	0	0	0	0	0	0	1	0	0	0	0	1	Unique								
4030	OSC70748.1	-	hypothetical protein	0	1	1	1	0	0	1	1	1	1	0	7	Accessory								
4031	Prokka:CIODPCDJ_00386	hemC_1	Porphobilinogen deaminase	1	1	1	1	1	1	1	1	1	1	1	11	CORE	COG0181_H#	43	226	1.06E-58	K01749	43	225	8.24E-58
4032	OSC57270.1	-	DNA polymerase III subunit delta	1	1	1	1	1	1	1	1	1	1	1	11	CORE	COG1466_L#	42.5	246	1.69E-64	K02340	42.6	243	2.24E-63
4033	WP_048460070.1	-	membrane protein	1	0	1	1	1	1	1	1	1	1	1	10	Accessory								
4034	WP_033274231.1	-	iron ABC transporter permease	0	1	1	1	0	0	1	1	1	1	0	7	Accessory	COG0609_P#	34.4	181	5.09E-45	K02015	34.9	173	2.84E-42
4035	OSC64610.1	-	hypothetical protein	1	1	1	1	1	1	1	1	1	1	1	11	CORE	COG1300_S#	51.8	343	7.75E-94	NA	51.8	343	2.07E-93
4036	Prokka:JAPCLKMA_05527	-	hypothetical protein	1	0	0	1	1	1	1	1	1	1	1	9	Accessory								
4037	WP_033277918.1	-	ATPase	1	1	0	0	1	1	1	1	1	1	1	9	Accessory	COG2971_G#	34.6	108	5.48E-23				
4038	WP_019523548.1	argF	ornithine carbamoyltransferase	1	1	1	1	1	1	1	1	1	1	1	11	CORE	COG0078_E#	66.9	450	7.68E-126	K00611	66.8	442	3.27E-123

cluster	GI	gene	Annotation	Streptomyces_thermocarboxydus_BPSAC147	Streptomyces_sp._4F	Streptomyces_sp._BSE7F	Streptomyces_sp._ETH9427	Streptomyces_sp._FxanaD5	Streptomyces_sp._UNC401CLCol	Actinospica_acidiphila_NRRL_B_24431	Streptomyces_sp._HNS054	Streptomyces_sp._SMS_SU21	Streptomyces_sp._ZS0098	Streptomyces_thermocarboxydus_K155	Addition	Core-Accessory-Unique	COG	%Similarity_COG	Score_COG	Evalue_COG	KEGG	%Similarity_KEGG	Score_KEGG	Evalue_KEGG
4039	WP_048456941.1	gap	type I glyceraldehyde-3-phosphate dehydrogenase	1	1	1	1	1	1	1	1	1	1	1	11	CORE	COG0057_G#	64.8	435	1.96E-121	K00134	64.8	435	5.23E-121
4040	Prokka:CIODPCDJ_06743	cas1	CRISPR-associated endonuclease Cas1	0	0	0	0	1	0	0	0	0	0	1	2	Accessory	COG1518_L#	34.8	165	2.89E-40	K15342	34.8	165	7.72E-40
4041	OSC68283.1	-	rod shape-determining protein MreC	1	1	1	1	1	1	1	1	1	1	1	11	CORE	COG1792_M#	27	79	2.73E-14	K03570	24.5	75	1.37E-12
4042	WP_048455113.1	-	polysaccharide deacetylase family protein	1	1	1	1	1	1	1	1	1	1	1	11	CORE	COG0726_G#	41.3	139	2.22E-32	NA	35.6	117	3.15E-25
4043	WP_048459823.1	-	methionine synthase	1	1	1	1	1	1	1	1	1	1	1	11	CORE					NA	43.5	233	3.94E-60
4044	PWE08165.1	-	serine protease	1	1	1	1	1	1	1	1	1	1	1	11	CORE	COG0265_O#	38.1	195	4.45E-49	K08372	38.1	197	1.83E-49
4045	Prokka:JAPCLKMA_01581	-	hypothetical protein	1	0	0	0	0	0	0	0	0	0	0	1	Unique	COG1961_L#	35.1	93	1.4E-18				
4046	WP_033273819.1	-	MerR family transcriptional regulator	1	0	0	1	1	1	1	1	1	1	1	9	Accessory	COG0789_K#	39.3	57	1.45E-07	NA	38	54	0.00000327
4047	WP_158693579.1	-	hypothetical protein	0	0	0	0	0	1	0	0	0	0	0	1	Unique								
4048	OSC67445.1	-	hypothetical protein	1	1	1	1	1	1	1	1	1	1	1	11	CORE								

cluster	GI	gene	Annotation	Streptomyces_thermocarboxydus_BPSAC147	Streptomyces_sp._4F	Streptomyces_sp._BSE7F	Streptomyces_sp._ETH9427	Streptomyces_sp._FxanaD5	Streptomyces_sp._UNC401CLCol	Actinospica_acidiphila_NRRL_B_24431	Streptomyces_sp._HNS054	Streptomyces_sp._SMS_SU21	Streptomyces_sp._ZS0098	Streptomyces_thermocarboxydus_K155	Addition	Core-Accessory-Unique	COG	%Similarity_COG	Score_COG	Evalue_COG	KEGG	%Similarity_KEGG	Score_KEGG	Evalue_KEGG
4049	OSC59788.1	-	ABC transporter substrate-binding protein	0	1	0	0	0	0	0	0	0	0	0	1	Unique	COG0614_P#	49.1	306	1.05E-82				
4050	OSC65713.1	-	peptide ABC transporter permease	0	1	0	0	0	0	0	0	0	0	0	1	Unique	COG1173_EP#	40.1	248	3.39E-65	K13891	42.7	231	1.15E-59
4051	WP_107080945.1	-	ATP-binding protein	0	0	0	0	0	0	0	1	0	0	0	1	Unique								
4052	WP_028961312.1	-	LysR family transcriptional regulator	0	0	0	0	1	1	0	0	0	0	0	2	Accessory	COG0583_K#	31.3	141	7.63E-33	NA	32.4	127	1.78E-28
4053	AXI86123.1	-	hypothetical protein	1	0	1	1	1	1	0	1	1	1	1	9	Accessory								
4054	OSC71435.1	-	hypothetical protein	1	1	1	1	1	1	1	1	1	1	1	11	CORE	COG1276_P#	30.1	52	0.00000273				
4055	Prokka:CIODPCDJ_03353	bkdB_1	3-methyl-2-oxobutanoate dehydrogenase subunit beta	1	1	1	1	1	1	1	1	1	1	1	11	CORE	COG0022_C#	53.5	352	2.17E-96	K00162	53.5	352	5.8E-96
4056	AXI86173.1	-	tellurium resistance protein TerC	1	1	1	1	1	1	1	1	1	1	1	11	CORE	COG0861_P#	45.8	299	2.18E-80	K05794	47.5	285	5.1E-76
4057	WP_033276154.1	-	hypothetical protein	1	0	0	0	1	1	1	0	0	1	1	6	Accessory	COG1292_M#	39.3	48	0.0000671				
4058	PWE11544.1	-	3-oxoacyl-ACP synthase	0	0	1	1	0	0	0	1	1	1	0	5	Accessory								

cluster	GI	gene	Annotation	Streptomyces_thermocarboxydus_BPSAC147	Streptomyces_sp._4F	Streptomyces_sp._BSE7F	Streptomyces_sp._ETH9427	Streptomyces_sp._FxanaD5	Streptomyces_sp._UNC401CLCol	Actinospica_acidiphila_NRRL_B_24431	Streptomyces_sp._HNS054	Streptomyces_sp._SMS_SU21	Streptomyces_sp._ZS0098	Streptomyces_thermocarboxydus_K155	Addition	Core-Accessory-Unique	COG	%Similarity_COG	Score_COG	Evalue_COG	KEGG	%Similarity_KEGG	Score_KEGG	Evalue_KEGG
4059	WP_019522545.1	-	alcohol dehydrogenase catalytic domain-containing protein	1	1	1	1	1	1	1	1	1	1	1	11	CORE	COG1063_ER#	31.2	152	3.3E-36	K00008	29.6	136	5E-31
4060	AXI86558.1	-	ABC transporter ATP-binding protein	0	0	0	1	1	1	1	1	1	1	1	8	Accessory	COG1127_Q#	68.8	442	1.22E-123	K02065	68.8	442	3.26E-123
4061	WP_048460703.1	-	hypothetical protein	0	0	1	1	0	0	0	1	1	1	0	5	Accessory								
4062	WP_019521587.1	-	Gfo/Idh/MocA family oxidoreductase	1	0	0	1	1	1	1	1	1	1	1	9	Accessory	COG0673_R#	41.2	281	4.71E-75	K00010	35.7	219	5.87E-56
4063	OSC63082.1	-	magnesium and cobalt transport protein CorA	1	1	1	1	1	1	1	1	1	1	1	11	CORE	COG0598_P#	43.7	282	2.11E-75	K03284	43.7	282	5.64E-75
4064	Prokka:CIODPCDJ_02611	-	hypothetical protein	1	1	1	1	1	1	1	1	1	1	1	11	CORE	COG0122_L#	51.9	287	6.57E-77	NA	51.9	287	1.75E-76
4065	OSC66117.1	-	EamA family transporter	1	1	1	1	1	1	1	1	1	1	1	11	CORE	COG0697_GER#	29.6	86	2.22E-16	NA	29.6	86	5.94E-16
4066	Prokka:JAPCLKMA_00151	draG_1	ADP-ribosyl-[dinitrogen reductase] glycohydrolase	1	1	1	1	1	1	1	1	1	1	1	11	CORE	COG1397_O#	29.4	107	1.22E-22				

cluster	GI	gene	Annotation	Streptomyces_thermocarboxydus_BPSAC147	Streptomyces_sp._4F	Streptomyces_sp._BSE7F	Streptomyces_sp._ETH9427	Streptomyces_sp._FxanaD5	Streptomyces_sp._UNC401CLCol	Actinospica_acidiphila_NRRL_B_24431	Streptomyces_sp._HNS054	Streptomyces_sp._SMS_SU21	Streptomyces_sp._ZS0098	Streptomyces_thermocarboxydus_K155	Addition	Core-Accessory-Unique	COG	%Similarity_COG	Score_COG	Evalue_COG	KEGG	%Similarity_KEGG	Score_KEGG	Evalue_KEGG
4067	WP_121719549.1	-	ABC transporter permease	1	0	1	1	1	1	1	1	1	1	1	10	Accessory	COG0601_EP#	45	304	6.78E-82	K02033	45.1	303	2.37E-81
4068	Prokka:CIODPCDJ_01851	-	hypothetical protein	1	1	1	1	1	1	1	1	1	1	1	11	CORE	COG1175_G#	34.7	187	7.08E-47	K15771	33.1	178	1.5E-43
4069	WP_102637772.1	-	TrmB family transcriptional regulator	1	0	1	1	1	1	1	1	1	1	1	10	Accessory	COG2771_K#	31.9	44	0.000742	NA	31.9	44	0.00198
4070	PWE08299.1	cydB	cytochrome d ubiquinol oxidase subunit II	1	1	1	1	1	1	1	1	1	1	1	11	CORE	COG1294_C#	49.6	322	1.84E-87	K00426	49.6	322	4.92E-87
4071	WP_109288556.1	-	Rieske (2Fe-2S) protein	1	0	0	1	1	1	1	1	1	1	1	9	Accessory	COG4638_PR#	31.4	67	1.07E-10	NA	32.4	47	0.000399
4072	OSC69546.1	-	ABC transporter permease	1	1	1	1	1	1	1	1	1	1	1	11	CORE	COG0601_EP#	35.8	195	2.6E-49	K02033	35.1	203	2.55E-51
4073	PWE07111.1	-	16S rRNA (cytosine(1402)-N(4))-methyltransferase	1	0	1	1	1	1	1	1	1	1	1	10	Accessory	COG0275_M#	53.7	326	9.77E-89	K03438	54.4	320	1.87E-86
4074	WP_122217060.1	-	aldo/keto reductase	1	0	1	1	0	0	1	1	1	1	0	7	Accessory	COG0667_C#	50.2	325	1.67E-88	NA	38.4	175	9.72E-43
4075	WP_019526217.1	-	MoxR family ATPase	0	1	0	0	1	1	0	0	0	0	0	3	Accessory	COG0714_R#	30.5	105	3.54E-22	NA	30.9	78	1.62E-13

cluster	GI	gene	Annotation	Streptomyces_thermocarboxydus_BPSAC147	Streptomyces_sp._4F	Streptomyces_sp._BSE7F	Streptomyces_sp._ETH9427	Streptomyces_sp._FxanaD5	Streptomyces_sp._UNC401CLCol	Actinospica_acidiphila_NRRL_B_24431	Streptomyces_sp._HNS054	Streptomyces_sp._SMS_SU21	Streptomyces_sp._ZS0098	Streptomyces_thermocarboxydus_K155	Addition	Core-Accessory-Unique	COG	%Similarity_COG	Score_COG	Evalue_COG	KEGG	%Similarity_KEGG	Score_KEGG	Evalue_KEGG
4076	OSC57154.1	-	daunorubicin/doxorubicin resistance ABC transporter ATP-binding protein DrrA	0	1	0	0	0	0	1	0	0	0	0	2	Accessory	COG1131_V#	44.6	248	2.59E-65	K01990	44	242	4.96E-63
4077	WP_102637936.1	-	hypothetical protein	1	0	1	1	1	1	1	1	1	1	1	10	Accessory					K14007	44.1	67	2.85E-10
4078	WP_019524833.1	fabH	ketoacyl-ACP synthase III	0	0	0	0	1	1	0	0	0	0	1	3	Accessory	COG0332_I#	48.1	304	3.98E-82	K11608	48.1	304	1.06E-81
4079	WP_048459191.1	-	PD-(D/E)XK motif protein	0	0	0	0	0	0	0	1	0	0	0	1	Unique								
4080	WP_078493056.1	-	GNAT family N-acetyltransferase	1	1	1	1	1	1	1	1	1	1	1	11	CORE	COG0454_KR#	40.5	117	6.89E-26				
4081	WP_033277420.1	-	ABC transporter ATP-binding protein	1	1	0	0	0	1	1	0	0	0	1	5	Accessory	COG4608_E#	47.5	290	5.94E-78	K02032	45.5	259	3.92E-68
4082	OSC62773.1	-	fuconate dehydratase	0	1	0	0	0	0	0	0	0	0	0	1	Unique	COG4948_MR#	56.7	384	3.95E-106	NA	52.7	356	3.08E-97
4083	AXI85532.1	-	hypothetical protein	0	1	1	1	0	0	1	1	1	1	0	7	Accessory								

cluster	GI	gene	Annotation	Streptomyces_thermocarboxydus_BPSAC147	Streptomyces_sp._4F	Streptomyces_sp._BSE7F	Streptomyces_sp._ETH9427	Streptomyces_sp._FxanaD5	Streptomyces_sp._UNC401CLCol	Actinospica_acidiphila_NRRL_B_24431	Streptomyces_sp._HNS054	Streptomyces_sp._SMS_SU21	Streptomyces_sp._ZS0098	Streptomyces_thermocarboxydus_K155	Addition	Core-Accessory-Unique	COG	%Similarity_COG	Score_COG	Evalue_COG	KEGG	%Similarity_KEGG	Score_KEGG	Evalue_KEGG
4084	OSC71628.1	-	mechanosensitive ion channel protein MscS	0	1	0	0	0	0	0	0	0	0	0	1	Unique	COG0668_M#	36.3	185	2.68E-46	K03442	42.1	173	2.82E-42
4085	PWE11007.1	-	TIGR00341 family protein	0	0	1	1	0	0	0	1	0	0	0	3	Accessory	COG1808_S#	36.6	135	4.16E-31	NA	32.4	93	3.71E-18
4086	WP_048460025.1	-	FKBP-type peptidyl-prolyl cis-trans isomerase	1	1	1	1	1	1	1	1	1	1	1	11	CORE	COG0545_O#	40.3	91	5.27E-18	K03772	35.2	87	2.03E-16
4087	WP_051724392.1	-	SDR family NAD(P)-dependent oxidoreductase	1	0	0	1	1	1	1	1	1	1	1	9	Accessory	COG1028_IQR#	47.1	254	4.71E-67	NA	47.1	253	2.14E-66
4088	WP_037952889.1	-	membrane protein	1	1	1	1	1	1	1	1	1	1	1	11	CORE	COG3447_T#	25.1	62	4.48E-09	NA	37.3	44	0.00258
4089	AXI88874.1	-	ABC transporter ATP-binding protein	1	0	1	1	1	1	1	1	1	1	1	10	Accessory	COG0444_EP#	53.7	311	4.24E-84	K16202	49.4	297	1.29E-79
4090	WP_102637445.1	-	proline racemase family protein	1	1	1	0	1	1	1	1	1	1	1	10	Accessory	COG3938_E#	44.6	264	5.94E-70	K18384	33.9	187	2.46E-46
4091	WP_033274290.1	-	Lrp/AsnC family transcriptional regulator	0	0	0	0	1	1	1	0	0	0	1	4	Accessory	COG1522_K#	23.8	72	3.31E-12	K03718	25	53	0.00000555
4092	AXI86430.1	-	rRNA methyltransferase	1	1	1	1	1	1	1	1	1	1	1	11	CORE	COG5459_J#	40.9	230	9.5E-60				

cluster	GI	gene	Annotation	Streptomyces_thermocarboxydus_BPSAC147	Streptomyces_sp._4F	Streptomyces_sp._BSE7F	Streptomyces_sp._ETH9427	Streptomyces_sp._FxanaD5	Streptomyces_sp._UNC401CLCol	Actinospica_acidiphila_NRRL_B_24431	Streptomyces_sp._HNS054	Streptomyces_sp._SMS_SU21	Streptomyces_sp._ZS0098	Streptomyces_thermocarboxydus_K155	Addition	Core-Accessory-Unique	COG	%Similarity_COG	Score_COG	Evalue_COG	KEGG	%Similarity_KEGG	Score_KEGG	Evalue_KEGG
4093	WP_122215029.1	-	LacI family transcriptional regulator	1	1	1	1	1	1	1	1	1	1	1	11	CORE	COG1609_K#	34.8	151	4.3E-36	NA	35	146	4.82E-34
4094	OSC73042.1	-	asparaginase	1	1	0	0	1	1	0	0	0	0	1	5	Accessory	COG4448_E#	36.7	182	2.97E-45				
4095	Prokka:CIODPCDJ_00606	ulaR_1	HTH-type transcriptional regulator UlaR	1	1	1	1	1	1	1	1	1	1	1	11	CORE	COG1349_KG#	34.8	166	1.69E-40	K02081	34.8	166	4.5E-40
4096	AXI89620.1	-	hypothetical protein	0	0	0	1	0	0	0	0	0	0	0	1	Unique								
4097	Prokka:JAPCLKMA_02464	rffG	dTDP-glucose 4,6-dehydratase 2	1	1	1	1	1	1	1	1	1	1	1	11	CORE	COG0451_MG#	47.9	297	4.85E-80	K08678	47.4	284	1.13E-75
4098	WP_019525557.1	-	hypothetical protein	0	0	0	0	1	1	0	0	0	0	1	3	Accessory								
4099	Prokka:CIODPCDJ_01106	araP_1	L-arabinose transport system permease protein AraP	1	1	1	1	1	1	1	1	1	1	1	11	CORE	COG1175_G#	46.6	306	1.36E-82	K02025	48.2	302	4.02E-81
4100	Prokka:CIODPCDJ_06049	-	hypothetical protein	1	1	0	0	1	1	0	0	0	0	1	5	Accessory	COG2159_R#	52.3	315	2.93E-85	K07045	52.3	315	7.83E-85
4101	WP_102638527.1	-	SIS domain-containing protein	0	0	0	1	0	0	0	1	1	0	0	3	Accessory	COG2222_M#	42.4	274	5.74E-73	K10708	28.4	153	5.15E-36
4102	OSC71868.1	-	hydrolase	0	1	0	0	0	0	0	0	0	0	0	1	Unique	COG0179_Q#	46.4	164	4.9E-40	NA	34.1	143	4.08E-33

cluster	GI	gene	Annotation	Streptomyces_thermocarboxydus_BPSAC147	Streptomyces_sp._4F	Streptomyces_sp._BSE7F	Streptomyces_sp._ETH9427	Streptomyces_sp._FxanaD5	Streptomyces_sp._UNC401CLCol	Actinospica_acidiphila_NRRL_B_24431	Streptomyces_sp._HNS054	Streptomyces_sp._SMS_SU21	Streptomyces_sp._ZS0098	Streptomyces_thermocarboxydus_K155	Addition	Core-Accessory-Unique	COG	%Similarity_COG	Score_COG	Evalue_COG	KEGG	%Similarity_KEGG	Score_KEGG	Evalue_KEGG
4103	WP_078504617.1	-	pentapeptide repeat-containing protein	1	1	1	1	1	1	1	1	1	1	1	11	CORE	COG1357_S#	58.9	314	6.53E-85	NA	58.9	314	1.75E-84
4104	WP_048457460.1	-	hypothetical protein	0	0	0	0	0	0	0	1	0	0	0	1	Unique								
4105	OSC71410.1	-	DNA-binding protein	0	1	0	0	0	0	0	0	0	0	0	1	Unique					NA	36.2	172	6.28E-42
4106	OSC57424.1	-	ATPase	0	1	0	0	0	0	0	0	0	0	0	1	Unique	COG0714_R#	59	371	3.45E-102	K03924	59	371	9.21E-102
4107	AXI91106.1	-	IS630 family transposase	0	1	0	1	0	0	0	0	0	0	0	2	Accessory	COG3335_L#	28.3	70	9.64E-12	K07494	32.7	84	2.25E-15
4108	WP_048456804.1	-	membrane protein	1	1	1	1	1	1	1	1	1	1	1	11	CORE								
4109	Prokka:JAPCLKMA_04474	iolS_2	Aldo-keto reductase IolS	1	1	1	1	1	1	1	1	1	1	1	11	CORE	COG0667_C#	42.3	258	2.49E-68	NA	43.4	244	1.69E-63
4110	WP_019524161.1	-	sigma-70 family RNA polymerase sigma factor	1	1	1	1	1	1	1	1	1	1	1	11	CORE	COG0568_K#	50.5	294	6.98E-79	K03087	50.5	294	1.86E-78
4111	WP_019526690.1	-	inorganic phosphate transporter	1	1	1	1	1	1	1	1	1	1	1	11	CORE	COG0306_P#	50.8	318	2.65E-86	K03306	50.8	318	7.06E-86
4112	OSC65711.1	-	ABC transporter permease	0	1	0	0	0	0	0	0	0	0	0	1	Unique	COG0601_EP#	37.8	227	8.02E-59	K12369	36.8	221	1.18E-56

cluster	GI	gene	Annotation	Streptomyces_thermocarboxydus_BPSAC147	Streptomyces_sp._4F	Streptomyces_sp._BSE7F	Streptomyces_sp._ETH9427	Streptomyces_sp._FxanaD5	Streptomyces_sp._UNC401CLCol	Actinospica_acidiphila_NRRL_B_24431	Streptomyces_sp._HNS054	Streptomyces_sp._SMS_SU21	Streptomyces_sp._ZS0098	Streptomyces_thermocarboxydus_K155	Addition	Core-Accessory-Unique	COG	%Similarity_COG	Score_COG	Evalue_COG	KEGG	%Similarity_KEGG	Score_KEGG	Evalue_KEGG
4113	Prokka:CIODPCDJ_00869	-	dTDP-3,4-didehydro-2,6-dideoxy-alpha-D-glucose 3-reductase	0	0	0	0	1	1	0	0	0	0	1	3	Accessory	COG0673_R#	32.6	151	7.3E-36	NA	27.8	119	4.82E-26
4114	WP_122215976.1	-	pantoate--beta-alanine ligase	1	0	1	1	1	1	1	1	1	1	1	10	Accessory	COG0414_H#	47	226	1.37E-58	K01918	47	226	3.65E-58
4115	WP_102641842.1	-	hypothetical protein	0	0	0	1	0	0	0	1	1	1	0	4	Accessory								
4116	OSC71274.1	-	acyltransferase	0	1	0	0	0	0	0	0	0	0	0	1	Unique								
4117	OSC56475.1	-	hypothetical protein	1	1	1	1	1	1	1	1	1	1	1	11	CORE					NA	40.8	47	0.000304
4118	OSC61473.1	-	C4-dicarboxylate ABC transporter substrate-binding protein	1	1	1	1	1	1	1	1	1	1	1	11	CORE	COG3181_S#	45.5	259	1.9E-68	NA	24	121	2.16E-26
4119	PWE08575.1	-	hydroxyacid dehydrogenase	0	0	1	0	0	0	0	0	0	0	0	1	Unique	COG0111_HE#	29.6	124	9.56E-28	NA	31.1	111	1.71E-23
4120	Prokka:JAPCLKMA_04479	ilvC	Ketol-acid reductoisomerase (NADP(+))	1	1	1	1	1	1	1	1	1	1	1	11	CORE	COG0059_EH#	67.4	455	2.36E-127	K00053	67.3	453	2.4E-126
4121	Prokka:JAPCLKMA_02316	-	hypothetical protein	1	1	1	1	1	1	1	1	1	1	1	11	CORE	COG1741_R#	61	246	1.28E-64	K06911	28	114	2.64E-24

cluster	GI	gene	Annotation	Streptomyces_thermocarboxydus_BPSAC147	Streptomyces_sp._4F	Streptomyces_sp._BSE7F	Streptomyces_sp._ETH9427	Streptomyces_sp._FxanaD5	Streptomyces_sp._UNC401CLCol	Actinospica_acidiphila_NRRL_B_24431	Streptomyces_sp._HNS054	Streptomyces_sp._SMS_SU21	Streptomyces_sp._ZS0098	Streptomyces_thermocarboxydus_K155	Addition	Core-Accessory-Unique	COG	%Similarity_COG	Score_COG	Evalue_COG	KEGG	%Similarity_KEGG	Score_KEGG	Evalue_KEGG
4122	WP_019521709.1	-	sugar phosphate isomerase/epimerase	1	1	1	1	1	1	1	1	1	1	1	11	CORE	COG1082_G#	33	157	7.8E-38				
4123	WP_033274790.1	-	helix-turn-helix transcriptional regulator	1	1	1	1	1	1	1	1	1	1	1	11	CORE	COG2197_TK#	27.8	41	0.00815	NA	27.9	43	0.00573
4124	Prokka:CIODPCDJ_02948	glkA_2	Glucokinase	1	1	1	1	1	1	1	1	1	1	1	11	CORE	COG1940_KG#	38	183	1.02E-45	K00845	38	183	2.72E-45
4125	WP_033274257.1	-	hypothetical protein	0	0	0	0	0	0	1	0	0	0	0	1	Unique								
4126	OSC66433.1	-	hypothetical protein	0	1	0	0	0	0	0	0	0	0	0	1	Unique	COG3509_Q#	34.6	134	9.24E-31	NA	35.8	44	0.00336
4127	OSC72201.1	-	epimerase	0	1	0	0	0	0	0	0	0	0	0	1	Unique	COG0451_MG#	34.9	181	5.05E-45	K08678	34.7	165	1E-39
4128	WP_033272847.1	-	hypothetical protein	1	1	1	1	1	1	1	1	1	1	1	11	CORE								
4129	WP_033275404.1	-	aldo/keto reductase	1	1	1	1	1	1	1	1	1	1	1	11	CORE	COG0667_C#	42.2	240	7.01E-63	K17744	34.1	140	2.64E-32
4130	OSC67563.1	-	ATP/GTP-binding protein	0	1	0	0	0	0	0	0	0	0	0	1	Unique								
4131	AXI91295.1	-	hypothetical protein	0	0	0	1	0	0	0	0	0	0	0	1	Unique								
4132	WP_102641144.1	-	aminoglycoside phosphotransferase family protein	1	0	1	1	0	1	1	1	1	1	1	9	Accessory								
4133	WP_122215707.1	-	D-cysteine desulfhydrase family protein	0	0	0	0	0	0	0	0	0	1	0	1	Unique	COG2515_E#	44.9	235	2.25E-61	K05396	40.4	193	4.46E-48

cluster	GI	gene	Annotation	Streptomyces_thermocarboxydus_BPSAC147	Streptomyces_sp._4F	Streptomyces_sp._BSE7F	Streptomyces_sp._ETH9427	Streptomyces_sp._FxanaD5	Streptomyces_sp._UNC401CLCol	Actinospica_acidiphila_NRRL_B_24431	Streptomyces_sp._HNS054	Streptomyces_sp._SMS_SU21	Streptomyces_sp._ZS0098	Streptomyces_thermocarboxydus_K155	Addition	Core-Accessory-Unique	COG	%Similarity_COG	Score_COG	Evalue_COG	KEGG	%Similarity_KEGG	Score_KEGG	Evalue_KEGG
4134	AXI85738.1	-	aspartate carbamoyltransferase	1	1	1	1	1	1	1	1	1	1	1	11	CORE	COG0540_F#	64.8	389	1.22E-107	K00609	64.8	389	3.25E-107
4135	WP_122215773.1	-	hypothetical protein	1	0	0	0	0	0	0	0	0	1	1	3	Accessory								
4136	Prokka:CIODPCDJ_06650	cytR_9	HTH-type transcriptional repressor CytR	0	0	0	0	1	1	0	0	0	0	1	3	Accessory	COG1609_K#	32.8	176	1.62E-43	K02529	28.6	148	1.65E-34
4137	PWE10347.1	-	dehydrogenase	1	1	1	1	1	1	1	1	1	1	1	11	CORE	COG1063_ER#	36.1	168	4.41E-41				
4138	WP_033277118.1	-	hypothetical protein	1	0	1	1	1	1	1	1	1	1	1	10	Accessory								
4139	OSC70757.1	-	sporulation protein	1	1	1	1	1	1	1	1	1	1	1	11	CORE	COG4326_R#	36.6	131	7.8E-30	K06377	36.6	129	4.64E-29
4140	WP_048456791.1	-	2-hydroxyacid dehydrogenase	1	1	1	1	1	1	1	1	1	1	1	11	CORE	COG1052_CHR#	56.3	314	6.5E-85	K03778	43.6	262	7.82E-69
4141	OSC70797.1	-	hypothetical protein	1	1	1	1	1	1	1	1	1	1	1	11	CORE	COG2358_R#	31	140	9.84E-33	K07080	27.2	108	1.11E-22
4142	PWE07898.1	galE	UDP-glucose 4-epimerase GalE	1	1	1	1	1	1	1	1	1	1	1	11	CORE	COG1087_M#	59.2	386	1.35E-106	K01784	52.5	312	5.05E-84
4143	Prokka:CIODPCDJ_02318	-	hypothetical protein	1	0	1	1	1	1	1	1	1	1	1	10	Accessory								
4144	WP_028961848.1	-	PAC2 family protein	1	1	1	1	1	1	1	1	1	1	1	11	CORE					NA	55.5	320	1.42E-86
4145	AXI89680.1	-	TetR family transcriptional regulator	1	1	1	1	1	1	1	1	1	1	1	11	CORE	COG1309_K#	29.4	79	2.06E-14	NA	36.4	69	7.44E-11

cluster	GI	gene	Annotation	Streptomyces_thermocarboxydus_BPSAC147	Streptomyces_sp._4F	Streptomyces_sp._BSE7F	Streptomyces_sp._ETH9427	Streptomyces_sp._FxanaD5	Streptomyces_sp._UNC401CLCol	Actinospica_acidiphila_NRRL_B_24431	Streptomyces_sp._HNS054	Streptomyces_sp._SMS_SU21	Streptomyces_sp._ZS0098	Streptomyces_thermocarboxydus_K155	Addition	Core-Accessory-Unique	COG	%Similarity_COG	Score_COG	Evalue_COG	KEGG	%Similarity_KEGG	Score_KEGG	Evalue_KEGG
4146	OSC61292.1	-	peptidase P60	0	1	0	0	0	0	0	0	0	0	0	1	Unique	COG0791_M#	37.5	127	8.62E-29	NA	37.5	127	2.3E-28
4147	WP_102637284.1	-	alpha-ketoacid dehydrogenase subunit beta	0	0	0	0	0	0	0	0	1	1	0	2	Accessory	COG0022_C#	43.8	292	2.64E-78	K00162	47.9	299	4.42E-80
4148	OSC72869.1	-	hypothetical protein	0	1	0	0	0	0	0	0	0	0	0	1	Unique								
4149	Prokka:CIODPCDJ_01963	-	hypothetical protein	0	0	0	0	0	0	0	0	0	0	1	1	Unique	COG1314_U#	37.7	43	0.00164				
4150	WP_147474327.1	-	hypothetical protein	1	0	0	0	0	0	0	0	0	1	1	3	Accessory								
4151	Prokka:JAPCLKMA_05583	fabH_4	3-oxoacyl-[acyl-carrier-protein] synthase 3	1	0	0	0	0	0	0	0	0	0	0	1	Unique								
4152	OSC70553.1	-	hypothetical protein	0	1	0	0	0	0	0	0	0	0	0	1	Unique								
4153	OSC70554.1	-	AAA family ATPase	0	1	0	0	0	0	0	0	0	0	0	1	Unique	COG0714_R#	35.8	134	7.05E-31	NA	27.1	45	0.0015
4154	OSC71434.1	-	DNA polymerase III subunit epsilon	1	1	1	1	1	1	1	1	1	1	1	11	CORE	COG0847_L#	41.5	198	3.04E-50	K02342	41.5	198	8.11E-50
4155	Prokka:JAPCLKMA_02460	-	hypothetical protein	1	0	0	0	0	0	0	0	0	0	0	1	Unique	COG5651_N#	25.1	52	0.00000269	NA	27.3	69	5.68E-11
4156	OSC63550.1	-	dihydroxyacetone kinase subunit DhaK	1	1	1	1	1	1	1	1	1	1	1	11	CORE	COG2376_G#	55.7	366	1.44E-100	K05878	46.5	288	1.02E-76

cluster	GI	gene	Annotation	Streptomyces_thermocarboxydus_BPSAC147	Streptomyces_sp._4F	Streptomyces_sp._BSE7F	Streptomyces_sp._ETH9427	Streptomyces_sp._FxanaD5	Streptomyces_sp._UNC401CLCol	Actinospica_acidiphila_NRRL_B_24431	Streptomyces_sp._HNS054	Streptomyces_sp._SMS_SU21	Streptomyces_sp._ZS0098	Streptomyces_thermocarboxydus_K155	Addition	Core-Accessory-Unique	COG	%Similarity_COG	Score_COG	Evalue_COG	KEGG	%Similarity_KEGG	Score_KEGG	Evalue_KEGG
4157	WP_048460487.1	hemB	porphobilinogen synthase	1	1	1	1	1	1	1	1	1	1	1	11	CORE	COG0113_H#	67	450	5.79E-126	K01698	67	450	1.55E-125
4158	WP_033276495.1	-	aldo/keto reductase	1	1	1	1	1	1	1	1	1	1	1	11	CORE	COG0667_C#	59.1	367	6.45E-101	NA	55.7	354	1.15E-96
4159	WP_102641303.1	-	xanthine dehydrogenase family protein subunit M	1	1	1	1	1	1	1	1	1	1	1	11	CORE	COG1319_C#	58.2	358	2.29E-98	K11178	56.5	346	3.14E-94
4160	Prokka:JAPCLKMA_01744	-	hypothetical protein	1	1	1	1	1	1	1	1	1	1	1	11	CORE								
4161	OSC70893.1	-	glycine/betaine ABC transporter substrate-binding protein	1	1	1	1	1	1	1	1	1	1	1	11	CORE	COG1732_M#	39.1	193	1.67E-48	K05845	30.4	148	9.62E-35
4162	PWE07870.1	-	nucleoside triphosphate pyrophosphohydrolase	1	1	1	1	1	1	1	1	1	1	1	11	CORE	COG1694_R#	41.8	185	2.66E-46	K02428	41.8	185	7.1E-46
4163	Prokka:JAPCLKMA_00023	ctaC	Cytochrome c oxidase subunit 2	1	1	1	1	1	1	1	1	1	1	1	11	CORE	COG1622_C#	37.7	214	6.98E-55	K02275	37.7	214	1.86E-54
4164	Prokka:JAPCLKMA_05493	acbM	2-epi-5-epi-valiolone 7-kinase	1	0	0	0	0	0	0	0	0	0	0	1	Unique	COG1940_KG#	24.9	59	2.88E-08				
4165	WP_033277570.1	-	MoxR family ATPase	1	0	1	1	1	1	1	1	1	1	1	10	Accessory	COG0714_R#	70.6	466	5.98E-131	K03924	70.6	466	1.6E-130

cluster	GI	gene	Annotation	Streptomyces_thermocarboxydus_BPSAC147	Streptomyces_sp._4F	Streptomyces_sp._BSE7F	Streptomyces_sp._ETH9427	Streptomyces_sp._FxanaD5	Streptomyces_sp._UNC401CLCol	Actinospica_acidiphila_NRRL_B_24431	Streptomyces_sp._HNS054	Streptomyces_sp._SMS_SU21	Streptomyces_sp._ZS0098	Streptomyces_thermocarboxydus_K155	Addition	Core-Accessory-Unique	COG	%Similarity_COG	Score_COG	Evalue_COG	KEGG	%Similarity_KEGG	Score_KEGG	Evalue_KEGG
4166	WP_048456046.1	-	sugar ABC transporter permease	1	0	1	1	1	1	1	1	1	1	1	10	Accessory	COG4209_G#	49.2	299	1.65E-80	K17319	44.3	282	5.58E-75
4167	Prokka:JAPCLKMA_02001	-	hypothetical protein	1	1	1	1	0	0	1	1	1	1	0	8	Accessory	COG2378_K#	30.1	112	3.74E-24	NA	19.6	72	8.76E-12
4168	PWE09668.1	-	hypothetical protein	0	0	1	0	0	0	0	0	1	0	0	2	Accessory								
4169	WP_102638584.1	-	ketoacyl-ACP synthase III	0	0	1	0	0	0	0	0	1	0	0	2	Accessory	COG0332_I#	41.7	216	1.84E-55	K00648	37.3	206	3.89E-52
4170	OSC72557.1	-	alcohol dehydrogenase	1	1	1	1	1	1	1	1	1	1	1	11	CORE	COG1063_ER#	35.5	210	1.32E-53	K00060	32.1	169	6.88E-41
4171	OSC70987.1	-	hypothetical protein	1	1	1	1	1	1	1	1	1	1	1	11	CORE								
4172	WP_033274138.1	-	ABC transporter ATP-binding protein	0	1	0	0	0	0	1	0	0	0	0	2	Accessory	COG4608_E#	56.6	285	1.89E-76	K02032	45.1	262	7.8E-69
4173	WP_122216262.1	-	sugar ABC transporter permease	0	0	1	0	0	0	0	1	1	1	0	4	Accessory	COG1175_G#	44.7	206	1.9E-52	K10118	35.5	166	5.82E-40
4174	PWE12113.1	-	hypothetical protein	1	0	1	1	1	1	1	1	1	1	1	10	Accessory	COG2267_I#	33.9	73	1.47E-12	NA	28.5	70	2.55E-11
4175	OSC69132.1	-	AraC family transcriptional regulator	0	1	0	1	0	0	0	1	0	0	0	3	Accessory	COG2207_K#	32.5	144	8.88E-34	NA	36.2	59	7.68E-08
4176	OSC69573.1	-	DNA-binding protein WhiA	1	1	1	1	1	1	1	1	1	1	1	11	CORE	COG1481_S#	71.2	460	7.31E-129	K09762	71	456	2.16E-127

cluster	GI	gene	Annotation	Streptomyces_thermocarboxydus_BPSAC147	Streptomyces_sp._4F	Streptomyces_sp._BSE7F	Streptomyces_sp._ETH9427	Streptomyces_sp._FxanaD5	Streptomyces_sp._UNC401CLCol	Actinospica_acidiphila_NRRL_B_24431	Streptomyces_sp._HNS054	Streptomyces_sp._SMS_SU21	Streptomyces_sp._ZS0098	Streptomyces_thermocarboxydus_K155	Addition	Core-Accessory-Unique	COG	%Similarity_COG	Score_COG	Evalue_COG	KEGG	%Similarity_KEGG	Score_KEGG	Evalue_KEGG
4177	OSC69322.1	-	sugar ABC transporter permease	0	1	0	0	0	0	0	0	0	0	0	1	Unique	COG1175_G#	34	163	1.41E-39	K02025	28.6	137	2.22E-31
4178	WP_033275701.1	dapD	2,3,4,5-tetrahydropyridine-2,6-dicarboxylate N-succinyltransferase	1	1	1	1	1	1	1	1	1	1	1	11	CORE	COG2171_E#	64.9	399	8.95E-111	K00674	64.9	399	2.39E-110
4179	WP_122214012.1	-	NAD-dependent epimerase/dehydratase family protein	1	0	1	1	1	1	1	1	1	1	1	10	Accessory	COG0451_MG#	56.4	355	2.53E-97	K17947	56.4	355	6.75E-97
4180	OSC69418.1	-	DUF1648 domain-containing protein	0	1	0	0	0	0	0	0	0	0	0	1	Unique								
4181	AXI89781.1	-	ABC transporter permease	1	0	1	1	1	1	1	1	1	1	1	10	Accessory	COG1175_G#	34.8	158	3.47E-38	K10237	34.8	158	9.27E-38
4182	WP_102641583.1	-	type I pantothenate kinase	1	1	1	1	1	1	1	1	1	1	1	11	CORE	COG1072_H#	60.6	375	2.36E-103	K00867	60.8	365	6.52E-100
4183	Prokka:JAPCLKMA_05747	-	hypothetical protein	1	1	1	1	1	1	1	1	1	1	1	11	CORE								

cluster	GI	gene	Annotation	Streptomyces_thermocarboxydus_BPSAC147	Streptomyces_sp._4F	Streptomyces_sp._BSE7F	Streptomyces_sp._ETH9427	Streptomyces_sp._FxanaD5	Streptomyces_sp._UNC401CLCol	Actinospica_acidiphila_NRRL_B_24431	Streptomyces_sp._HNS054	Streptomyces_sp._SMS_SU21	Streptomyces_sp._ZS0098	Streptomyces_thermocarboxydus_K155	Addition	Core-Accessory-Unique	COG	%Similarity_COG	Score_COG	Evalue_COG	KEGG	%Similarity_KEGG	Score_KEGG	Evalue_KEGG
4184	WP_048460881.1	-	CDP-alcohol phosphatidyltransferase family protein	1	0	0	1	1	1	1	1	1	1	1	9	Accessory	COG0558_I#	38.1	54	9.22E-07	K17103	31.3	48	0.000135
4185	PWE10380.1	-	DUF3048 domain-containing protein	1	1	1	0	1	1	1	1	1	1	1	10	Accessory					NA	48.5	67	2.81E-10
4186	PWE09043.1	-	malate dehydrogenase	1	1	1	1	1	1	1	1	1	1	1	11	CORE	COG0039_C#	71.4	470	5.39E-132	K00024	71.4	470	1.44E-131
4187	WP_033272322.1	-	glycosyltransferase family 2 protein	1	1	1	1	1	1	1	1	1	1	1	11	CORE	COG0463_M#	31.5	106	1.57E-22	K19427	31.5	106	4.18E-22
4188	WP_048460169.1	-	multicopper oxidase domain-containing protein	1	1	1	1	1	1	1	1	1	1	1	11	CORE	COG2132_Q#	29.3	79	3.5E-14	NA	29.1	72	8.74E-12
4189	OSC62381.1	-	topoisomerase II	1	1	1	1	1	1	1	1	1	1	1	11	CORE	COG0653_U#	46	52	0.00000458	NA	43.2	45	0.00114
4190	WP_122212677.1	-	sugar ABC transporter permease	1	1	1	1	1	1	1	1	1	1	1	11	CORE	COG1175_G#	36	158	3.47E-38	K02025	32.1	148	1.25E-34
4191	WP_048455466.1	-	glycosyltransferase family 9 protein	1	1	1	0	1	1	1	1	1	1	1	10	Accessory	COG0859_M#	26.2	84	6.37E-16				
4192	Prokka:CIODPCDJ_02905	-	hypothetical protein	1	0	0	0	1	1	1	0	0	0	1	5	Accessory	COG2378_K#	41.3	196	1.96E-49	NA	39.2	174	1.63E-42
4193	OSC62167.1	-	nucleoside hydrolase	1	1	1	1	1	1	1	1	1	1	1	11	CORE	COG1957_F#	39.3	199	1.77E-50	K01250	38.3	197	1.8E-49

cluster	GI	gene	Annotation	Streptomyces_thermocarboxydus_BPSAC147	Streptomyces_sp._4F	Streptomyces_sp._BSE7F	Streptomyces_sp._ETH9427	Streptomyces_sp._FxanaD5	Streptomyces_sp._UNC401CLCol	Actinospica_acidiphila_NRRL_B_24431	Streptomyces_sp._HNS054	Streptomyces_sp._SMS_SU21	Streptomyces_sp._ZS0098	Streptomyces_thermocarboxydus_K155	Addition	Core-Accessory-Unique	COG	%Similarity_COG	Score_COG	Evalue_COG	KEGG	%Similarity_KEGG	Score_KEGG	Evalue_KEGG
4194	OSC70339.1	-	hypothetical protein	0	1	0	0	0	0	0	0	0	0	0	1	Unique	COG3321_Q#	36	151	4.24E-36	K12430	31.2	121	2.14E-26
4195	Prokka:CIODPCDJ_03157	-	hypothetical protein	0	0	0	0	0	0	0	0	0	0	1	1	Unique								
4196	OSC61826.1	-	LysR family transcriptional regulator	1	1	1	1	1	1	1	1	1	1	1	11	CORE	COG0583_K#	33.8	113	1.28E-24	NA	29.3	70	4.34E-11
4197	OSC70575.1	-	integrase	0	1	0	0	0	0	0	0	0	0	0	1	Unique								
4198	Prokka:JAPCLKMA_03675	ppsC_3	Phthiocerol synthesis polyketide synthase type I PpsC	1	0	0	0	1	1	1	0	0	0	1	5	Accessory	COG0604_CR#	36.6	177	7.22E-44	NA	35.8	157	2.07E-37
4199	OSC66859.1	-	AraC family transcriptional regulator	1	1	1	0	1	1	1	1	1	1	1	10	Accessory	COG4977_K#	41.7	217	8.22E-56	NA	29.3	89	6.91E-17
4200	PWE11369.1	-	sugar ABC transporter permease	0	0	1	0	0	0	0	0	0	0	0	1	Unique	COG1175_G#	41	242	2.39E-63	K02025	38.7	217	1.68E-55
4201	OSC68515.1	-	hypothetical protein	0	1	0	0	1	0	0	0	0	0	0	2	Accessory								
4202	WP_033277707.1	-	cyclase	0	0	0	0	0	0	1	0	0	0	0	1	Unique								
4203	WP_102637467.1	-	acyl-ACP desaturase	1	1	1	1	1	1	1	1	1	1	1	11	CORE					K03921	48.4	320	1.41E-86

cluster	GI	gene	Annotation	Streptomyces_thermocarboxydus_BPSAC147	Streptomyces_sp._4F	Streptomyces_sp._BSE7F	Streptomyces_sp._ETH9427	Streptomyces_sp._FxanaD5	Streptomyces_sp._UNC401CLCol	Actinospica_acidiphila_NRRL_B_24431	Streptomyces_sp._HNS054	Streptomyces_sp._SMS_SU21	Streptomyces_sp._ZS0098	Streptomyces_thermocarboxydus_K155	Addition	Core-Accessory-Unique	COG	%Similarity_COG	Score_COG	Evalue_COG	KEGG	%Similarity_KEGG	Score_KEGG	Evalue_KEGG
4204	WP_102641114.1	-	YafY family transcriptional regulator	1	1	1	1	1	1	1	1	1	1	1	11	CORE	COG2378_K#	28.5	154	6.53E-37	NA	26.4	104	1.58E-21
4205	OSC71396.1	-	hypothetical protein	0	1	0	0	0	0	0	0	0	0	0	1	Unique								
4206	Prokka:CIODPCDJ_01263	yajO_2	1-deoxyxylulose-5-phosphate synthase YajO	1	1	1	1	1	1	1	1	1	1	1	11	CORE	COG0667_C#	49.7	272	1.64E-72	NA	34.1	183	3.5E-45
4207	AXI84679.1	-	quinone oxidoreductase	0	0	0	1	0	0	0	1	0	1	0	3	Accessory	COG0604_CR#	40.2	216	1.83E-55	K18980	36.6	164	2.2E-39
4208	WP_048460797.1	-	hypothetical protein	0	0	1	1	0	0	0	1	1	1	0	5	Accessory								
4209	OSC71377.1	-	hypothetical protein	0	1	0	0	0	0	0	0	0	0	0	1	Unique								
4210	WP_033274197.1	-	mannose-binding protein	0	0	0	1	1	1	1	1	1	1	1	8	Accessory					NA	27.1	65	1.39E-09
4211	WP_052183407.1	-	LacI family transcriptional regulator	1	0	0	0	0	1	0	0	0	0	1	3	Accessory	COG1609_K#	32.7	176	1.23E-43	NA	32.7	176	3.28E-43
4212	WP_026243309.1	-	NADPH:quinone reductase	1	0	0	0	1	1	0	0	0	0	1	4	Accessory	COG0604_CR#	41.5	227	7.92E-59	K00344	31.3	114	2E-24
4213	WP_033274113.1	-	sugar ABC transporter permease	1	0	1	1	1	1	1	1	1	1	1	10	Accessory	COG1175_G#	31.3	163	1.84E-39	K10237	29.1	153	5.07E-36
4214	OSC70301.1	-	hypothetical protein	0	1	0	0	0	0	0	0	0	0	0	1	Unique								
4215	OSC70759.1	-	hypothetical protein	1	1	1	1	1	1	1	1	1	1	1	11	CORE								

cluster	GI	gene	Annotation	Streptomyces_thermocarboxydus_BPSAC147	Streptomyces_sp._4F	Streptomyces_sp._BSE7F	Streptomyces_sp._ETH9427	Streptomyces_sp._FxanaD5	Streptomyces_sp._UNC401CLCol	Actinospica_acidiphila_NRRL_B_24431	Streptomyces_sp._HNS054	Streptomyces_sp._SMS_SU21	Streptomyces_sp._ZS0098	Streptomyces_thermocarboxydus_K155	Addition	Core-Accessory-Unique	COG	%Similarity_COG	Score_COG	Evalue_COG	KEGG	%Similarity_KEGG	Score_KEGG	Evalue_KEGG
4216	WP_122212639.1	-	hypothetical protein	1	0	0	0	1	0	1	0	0	1	0	4	Accessory	COG3440_V#	37.3	192	2.82E-48	K07454	37.3	192	7.54E-48
4217	WP_106432503.1	glpX	class II fructose-bisphosphatase	0	0	0	0	1	1	0	0	0	0	0	2	Accessory	COG1494_G#	52.8	319	1.17E-86	K02446	52.7	327	1.15E-88
4218	OSC71365.1	-	hypothetical protein	0	1	1	0	0	0	0	0	1	0	0	3	Accessory								
4219	OSC55250.1	-	hypothetical protein	1	1	1	1	1	1	1	1	1	1	1	11	CORE	COG0189_HJ#	26.9	52	0.00000456				
4220	Prokka:CIODPCDJ_03541	-	hypothetical protein	1	0	0	0	1	1	0	0	0	0	1	4	Accessory					NA	27.4	49	0.000079
4221	Prokka:CIODPCDJ_02648	-	hypothetical protein	1	1	1	1	1	1	1	1	1	1	1	11	CORE								
4222	AXI91393.1	-	hypothetical protein	0	0	0	1	0	0	0	0	0	0	0	1	Unique								
4223	PWE08569.1	-	glycosyltransferase	0	0	1	0	0	0	0	0	0	0	0	1	Unique	COG0438_M#	24.2	50	0.0000133				
4224	Prokka:CIODPCDJ_02536	hcaR_1	Hca operon transcriptional activator HcaR	1	0	1	1	1	1	1	1	1	1	1	10	Accessory	COG0583_K#	32.8	110	1.41E-23	NA	32.8	110	3.76E-23
4225	PWE10982.1	-	carbohydrate kinase family protein	1	1	1	1	1	1	1	1	1	1	1	11	CORE	COG0524_G#	49.7	292	2E-78	K00856	49.1	289	4.52E-77
4226	AXI90045.1	-	transcriptional regulator	0	0	1	1	0	0	1	1	1	1	0	6	Accessory	COG2197_TK#	29.2	54	9.16E-07	NA	29.2	54	0.00000245
4227	WP_026243981.1	-	NAD(P)H-quinone oxidoreductase	1	1	1	1	1	1	1	1	1	1	1	11	CORE	COG0604_CR#	60.4	386	1.33E-106	NA	60.1	384	1.03E-105

cluster	GI	gene	Annotation	Streptomyces_thermocarboxydus_BPSAC147	Streptomyces_sp._4F	Streptomyces_sp._BSE7F	Streptomyces_sp._ETH9427	Streptomyces_sp._FxanaD5	Streptomyces_sp._UNC401CLCol	Actinospica_acidiphila_NRRL_B_24431	Streptomyces_sp._HNS054	Streptomyces_sp._SMS_SU21	Streptomyces_sp._ZS0098	Streptomyces_thermocarboxydus_K155	Addition	Core-Accessory-Unique	COG	%Similarity_COG	Score_COG	Evalue_COG	KEGG	%Similarity_KEGG	Score_KEGG	Evalue_KEGG
4228	OSC59248.1	-	ABC transporter substrate-binding protein	1	1	0	0	1	1	1	0	0	0	1	6	Accessory	COG0834_ET#	45.6	239	1.54E-62	K02424	32.9	115	8.95E-25
4229	OSC61427.1	-	ABC transporter substrate-binding protein	1	1	1	1	1	1	1	1	1	1	1	11	CORE	COG0614_P#	30.5	164	4.81E-40	K02016	29.1	114	1.99E-24
4230	WP_048456588.1	-	aldo/keto reductase	1	1	0	1	1	1	1	1	1	1	1	10	Accessory	COG0667_C#	54	331	2.97E-90	NA	35.3	134	1.42E-30
4231	Prokka:CIODPCDJ_06042	pobB	Phenoxybenzoate dioxygenase subunit beta	1	0	0	0	1	1	0	0	0	0	1	4	Accessory	COG1018_C#	42.4	220	7.39E-57	NA	38.9	202	5.56E-51
4232	WP_122215172.1	-	aminoglycoside phosphotransferase family protein	1	0	1	1	1	1	1	1	1	1	1	10	Accessory								
4233	WP_019524838.1	rfbB	dTDP-glucose 4,6-dehydratase	0	1	0	0	1	1	1	0	0	0	1	5	Accessory	COG1088_M#	55.8	367	4.89E-101	K01710	47.3	307	1.23E-82
4234	Prokka:CIODPCDJ_00928	oxyR_1	Hydrogen peroxide-inducible genes activator	1	1	0	0	1	1	1	1	1	1	1	9	Accessory	COG0583_K#	31.1	129	2.93E-29	K04761	28.6	115	1.17E-24
4235	OSC62079.1	-	ATP-dependent DNA ligase	0	1	0	1	0	0	0	0	0	0	1	3	Accessory	COG1793_L#	33.1	90	1.51E-17				
4236	WP_122215407.1	-	hypothetical protein	0	0	1	1	0	0	0	1	1	1	1	6	Accessory					NA	29	48	0.000175

cluster	GI	gene	Annotation	Streptomyces_thermocarboxydus_BPSAC147	Streptomyces_sp._4F	Streptomyces_sp._BSE7F	Streptomyces_sp._ETH9427	Streptomyces_sp._FxanaD5	Streptomyces_sp._UNC401CLCol	Actinospica_acidiphila_NRRL_B_24431	Streptomyces_sp._HNS054	Streptomyces_sp._SMS_SU21	Streptomyces_sp._ZS0098	Streptomyces_thermocarboxydus_K155	Addition	Core-Accessory-Unique	COG	%Similarity_COG	Score_COG	Evalue_COG	KEGG	%Similarity_KEGG	Score_KEGG	Evalue_KEGG
4237	WP_048455956.1	-	LysR family transcriptional regulator	1	1	1	1	1	1	1	1	1	1	1	11	CORE	COG0583_K#	39.9	206	1.88E-52	NA	39.8	213	4.12E-54
4238	WP_122214242.1	-	alpha/beta hydrolase	0	0	0	1	1	1	0	1	0	1	1	6	Accessory	COG0596_R#	47.4	209	1.7E-53				
4239	Prokka:JAPCLKMA_02140	-	hypothetical protein	1	0	0	0	0	0	0	0	0	0	1	2	Accessory								
4240	AXI86474.1	-	N-acetylmuramoyl-L-alanine amidase	1	1	1	1	1	1	1	1	1	1	1	11	CORE	COG0860_M#	41.8	170	8.77E-42	K01448	41.8	170	2.34E-41
4241	OSC73466.1	-	multidrug ABC transporter ATP-binding protein	1	1	1	1	1	1	1	1	1	1	1	11	CORE	COG1131_V#	52.2	291	3.41E-78	K01990	44.4	203	3.26E-51
4242	Prokka:CIODPCDJ_04255	fgd_3	F420-dependent glucose-6-phosphate dehydrogenase	0	0	0	0	0	0	0	0	0	0	1	1	Unique	COG2141_C#	23.8	61	7.5E-09	NA	23.8	61	0.00000002
4243	WP_028959124.1	-	DUF2804 domain-containing protein	1	0	0	0	1	1	1	0	0	0	1	5	Accessory								
4244	OSC71053.1	-	NAD-dependent epimerase/dehydratase	0	1	0	0	0	0	0	0	0	0	0	1	Unique	COG0451_MG#	29.7	92	2.33E-18	K01784	28	86	5.81E-16

cluster	GI	gene	Annotation	Streptomyces_thermocarboxydus_BPSAC147	Streptomyces_sp._4F	Streptomyces_sp._BSE7F	Streptomyces_sp._ETH9427	Streptomyces_sp._FxanaD5	Streptomyces_sp._UNC401CLCol	Actinospica_acidiphila_NRRL_B_24431	Streptomyces_sp._HNS054	Streptomyces_sp._SMS_SU21	Streptomyces_sp._ZS0098	Streptomyces_thermocarboxydus_K155	Addition	Core-Accessory-Unique	COG	%Similarity_COG	Score_COG	Evalue_COG	KEGG	%Similarity_KEGG	Score_KEGG	Evalue_KEGG
4245	WP_033273181.1	-	pirin family protein	1	1	1	1	1	1	1	1	1	1	1	11	CORE	COG1741_R#	39.7	210	9.96E-54	K06911	39.6	206	5.02E-52
4246	OSC61643.1	-	UDP-glucose 4-epimerase GalE	1	1	1	1	1	1	1	1	1	1	1	11	CORE	COG1087_M#	42.9	252	1.75E-66	K01784	42.9	252	4.67E-66
4247	Prokka:JAPCLKMA_05842	-	hypothetical protein	1	1	1	1	1	1	1	1	1	1	1	11	CORE								
4248	AXI87901.1	-	ribose-phosphate diphosphokinase	1	1	1	1	1	1	1	1	1	1	1	11	CORE	COG0462_FE#	60.6	403	8.02E-112	K00948	61.9	389	2.45E-107
4249	WP_026244200.1	glpX	class II fructose-bisphosphatase	0	0	0	0	1	1	0	0	0	0	0	2	Accessory	COG1494_G#	47	254	4.61E-67	K02446	41.8	236	4.53E-61
4250	WP_109288782.1	-	cation diffusion facilitator family transporter	1	1	1	1	1	1	1	1	1	1	1	11	CORE	COG0053_P#	37.5	193	9.65E-49	NA	37.5	193	2.58E-48
4251	WP_122214479.1	-	ATP-binding protein	1	1	1	1	1	1	1	1	1	1	1	11	CORE	COG3640_D#	30.5	79	2.03E-14	K07321	30.5	94	2.78E-18
4252	WP_048456053.1	-	hypothetical protein	0	0	1	1	0	0	0	1	1	1	0	5	Accessory								
4253	WP_122215175.1	trxB	thioredoxin-disulfide reductase	1	1	1	1	1	1	1	1	1	1	1	11	CORE	COG0492_O#	56.7	351	4.72E-96	K00384	56.7	351	1.26E-95
4254	OSC71157.1	-	alcohol dehydrogenase	1	1	1	1	1	1	1	1	1	1	1	11	CORE	COG0604_CR#	42.9	244	6.23E-64	K00344	39.3	223	2.33E-57

cluster	GI	gene	Annotation	Streptomyces_thermocarboxydus_BPSAC147	Streptomyces_sp._4F	Streptomyces_sp._BSE7F	Streptomyces_sp._ETH9427	Streptomyces_sp._FxanaD5	Streptomyces_sp._UNC401CLCol	Actinospica_acidiphila_NRRL_B_24431	Streptomyces_sp._HNS054	Streptomyces_sp._SMS_SU21	Streptomyces_sp._ZS0098	Streptomyces_thermocarboxydus_K155	Addition	Core-Accessory-Unique	COG	%Similarity_COG	Score_COG	Evalue_COG	KEGG	%Similarity_KEGG	Score_KEGG	Evalue_KEGG
4255	WP_122215073.1	-	ATP-binding cassette domain-containing protein	1	0	1	1	0	0	1	1	1	1	0	7	Accessory	COG1131_V#	46.3	247	7.35E-65	K01990	46.3	240	1.84E-62
4256	OSC63557.1	-	LysR family transcriptional regulator	0	1	0	0	0	0	0	0	0	0	0	1	Unique	COG0583_K#	30.2	91	5.16E-18	NA	31.7	80	4.15E-14
4257	WP_102639545.1	-	hypothetical protein	1	0	1	1	1	0	1	1	1	1	0	8	Accessory								
4258	WP_019522299.1	-	ABC transporter permease	1	1	0	1	1	1	1	1	1	1	1	10	Accessory	COG1173_EP#	55	335	2.05E-91	K15582	54.3	302	5.14E-81
4259	PWE09794.1	-	sugar kinase	0	0	1	0	0	0	0	0	0	0	0	1	Unique	COG0524_G#	39.4	186	1.54E-46	K00874	36.2	169	6.8E-41
4260	OSC73269.1	-	hypothetical protein	0	1	0	0	0	0	0	0	0	0	0	1	Unique	COG0476_H#	25.5	82	4.09E-15	NA	31.8	66	4.75E-10
4261	WP_122214469.1	-	ArsA family ATPase	1	1	1	1	1	1	1	1	1	1	1	11	CORE	COG0003_P#	53.4	310	5.4E-84	NA	53.1	304	1.35E-81
4262	WP_028960501.1	pip	prolyl aminopeptidase	1	1	1	1	1	1	1	1	1	1	1	11	CORE	COG0596_R#	45.8	266	1.17E-70	K01259	43.4	240	3.13E-62
4263	WP_028961313.1	-	fructose-bisphosphate aldolase class I	0	0	0	0	1	1	0	0	0	0	0	2	Accessory	COG3588_G#	54.8	351	4.71E-96	K01623	51.5	312	6.47E-84
4264	WP_122217327.1	-	ABC transporter ATP-binding protein	1	0	1	0	1	1	1	0	1	1	1	8	Accessory	COG1136_V#	44.2	186	1.54E-46	K05685	45.1	181	1.01E-44

cluster	GI	gene	Annotation	Streptomyces_thermocarboxydus_BPSAC147	Streptomyces_sp._4F	Streptomyces_sp._BSE7F	Streptomyces_sp._ETH9427	Streptomyces_sp._FxanaD5	Streptomyces_sp._UNC401CLCol	Actinospica_acidiphila_NRRL_B_24431	Streptomyces_sp._HNS054	Streptomyces_sp._SMS_SU21	Streptomyces_sp._ZS0098	Streptomyces_thermocarboxydus_K155	Addition	Core-Accessory-Unique	COG	%Similarity_COG	Score_COG	Evalue_COG	KEGG	%Similarity_KEGG	Score_KEGG	Evalue_KEGG
4265	WP_048459737.1	-	ABC transporter substrate-binding protein	0	1	0	1	0	0	1	1	1	1	0	6	Accessory	COG4594_P#	24.5	72	2.47E-12	K02016	25.3	79	5.41E-14
4266	WP_019526712.1	-	carboxylating nicotinate-nucleotide diphosphorylase	1	1	1	1	1	1	1	1	1	1	1	11	CORE	COG0157_H#	57.8	289	1.68E-77	K00767	57.8	289	4.49E-77
4267	WP_033274236.1	-	zinc ABC transporter substrate-binding protein	0	1	1	1	1	1	1	1	1	1	1	10	Accessory	COG0803_P#	45.8	251	3.89E-66	K11707	29.5	120	3.61E-26
4268	Prokka:JAPCLKMA_02268	lacF_1	Lactose transport system permease protein LacF	1	1	0	0	1	1	1	0	0	1	1	7	Accessory	COG1175_G#	41.9	251	5.08E-66	K17245	37.5	186	3.14E-46
4269	WP_048458241.1	-	aldo/keto reductase	1	1	1	1	1	1	1	1	1	1	1	11	CORE	COG0667_C#	55.1	319	1.52E-86	NA	55.1	319	4.05E-86
4270	Prokka:CIODPCDJ_00827	-	hypothetical protein	1	0	0	0	1	1	0	0	0	0	1	4	Accessory								
4271	WP_048460352.1	argB	acetylglutamate kinase	1	1	1	1	1	1	1	1	1	1	1	11	CORE	COG0548_E#	57.3	334	4.56E-91	K00930	55.7	320	1.82E-86
4272	Prokka:JAPCLKMA_05716	dppB_3	Dipeptide transport system permease protein DppB	1	1	1	1	1	1	1	1	1	1	1	11	CORE	COG0601_EP#	33.4	199	2.29E-50	K13890	32.4	177	1.91E-43
4273	Prokka:JAPCLKMA_06571	-	hypothetical protein	1	0	0	0	0	0	0	0	0	0	0	1	Unique								

cluster	GI	gene	Annotation	Streptomyces_thermocarboxydus_BPSAC147	Streptomyces_sp._4F	Streptomyces_sp._BSE7F	Streptomyces_sp._ETH9427	Streptomyces_sp._FxanaD5	Streptomyces_sp._UNC401CLCol	Actinospica_acidiphila_NRRL_B_24431	Streptomyces_sp._HNS054	Streptomyces_sp._SMS_SU21	Streptomyces_sp._ZS0098	Streptomyces_thermocarboxydus_K155	Addition	Core-Accessory-Unique	COG	%Similarity_COG	Score_COG	Evalue_COG	KEGG	%Similarity_KEGG	Score_KEGG	Evalue_KEGG
4274	WP_122216599.1	-	xanthine dehydrogenase family protein subunit M	0	0	0	0	0	0	0	0	0	1	0	1	Unique	COG1319_C#	55.1	354	4.26E-97	K11178	46.9	295	4.8E-79
4275	Prokka:CIODPCDJ_06046	hdfR_2	HTH-type transcriptional regulator HdfR	1	0	0	0	1	1	0	0	0	0	1	4	Accessory	COG0583_K#	38.9	158	4.48E-38	NA	38.9	158	1.2E-37
4276	OSC69431.1	-	D-alanyl-D-alanine carboxypeptidase	1	1	1	1	1	1	1	1	1	1	1	11	CORE	COG1686_M#	35	126	1.44E-28	K07258	32.7	131	2.04E-29
4277	Prokka:CIODPCDJ_06602	ywlC	Threonylcarbamoyl-AMP synthase	0	0	0	0	0	0	0	0	0	0	1	1	Unique	COG0009_J#	46.6	262	1.69E-69	K07566	42.2	240	3.13E-62
4278	OSC60423.1	-	taurine dioxygenase	0	1	1	1	0	0	1	1	1	1	0	7	Accessory	COG2175_Q#	42.7	208	4.93E-53	K03119	40.9	193	3.36E-48
4279	Prokka:CIODPCDJ_00306	ngcF_2	Diacetylchitobiose uptake system permease protein NgcF	1	1	1	1	1	1	1	1	1	1	1	11	CORE	COG1175_G#	28.2	140	1.26E-32	K10118	28.5	122	7.25E-27
4280	OSC61248.1	-	oxidoreductase	0	1	0	0	0	0	0	0	0	0	0	1	Unique	COG0667_C#	36	180	8.4E-45	NA	35	148	9.45E-35
4281	PWE07087.1	-	ABC transporter ATP-binding protein	1	1	1	1	1	1	1	1	1	1	1	11	CORE	COG1131_V#	38.7	171	6.66E-42	K01990	38.7	171	1.78E-41

cluster	GI	gene	Annotation	Streptomyces_thermocarboxydus_BPSAC147	Streptomyces_sp._4F	Streptomyces_sp._BSE7F	Streptomyces_sp._ETH9427	Streptomyces_sp._FxanaD5	Streptomyces_sp._UNC401CLCol	Actinospica_acidiphila_NRRL_B_24431	Streptomyces_sp._HNS054	Streptomyces_sp._SMS_SU21	Streptomyces_sp._ZS0098	Streptomyces_thermocarboxydus_K155	Addition	Core-Accessory-Unique	COG	%Similarity_COG	Score_COG	Evalue_COG	KEGG	%Similarity_KEGG	Score_KEGG	Evalue_KEGG
4282	WP_106432554.1	-	DUF320 domain-containing protein	1	0	1	1	1	1	1	1	1	1	1	10	Accessory								
4283	WP_086704379.1	-	ABC transporter ATP-binding protein	1	0	1	1	1	1	1	1	1	1	1	10	Accessory	COG0444_EP#	52.8	347	3.97E-95	K02031	52.7	346	2.36E-94
4284	Prokka:CIODPCDJ_00684	-	hypothetical protein	1	0	0	0	1	1	1	0	0	0	1	5	Accessory								
4285	Prokka:JAPCLKMA_00968	-	hypothetical protein	1	1	0	0	1	1	0	0	0	0	1	5	Accessory	COG3817_S#	45.8	270	6.17E-72				
4286	OSC67055.1	-	diaminobutyrate--2-oxoglutarate aminotransferase	0	1	0	0	0	0	0	0	0	0	0	1	Unique	COG0160_E#	53.9	176	1.58E-43	K05830	34	126	3.84E-28
4287	Prokka:CIODPCDJ_02963	-	2-haloacrylate reductase	1	0	1	1	1	1	1	1	1	1	1	10	Accessory	COG0604_CR#	38.4	182	2.89E-45	NA	35.1	148	9.45E-35
4288	Prokka:JAPCLKMA_01985	-	hypothetical protein	1	0	0	0	0	0	0	0	0	0	0	1	Unique								
4289	WP_048459041.1	-	glucosyl-3-phosphoglycerate synthase	1	1	1	1	1	1	1	1	1	1	1	11	CORE	COG0463_M#	49.7	258	3.17E-68	K13693	47.2	242	8.2E-63
4290	Prokka:CIODPCDJ_06749	-	hypothetical protein	0	0	0	0	0	0	0	0	0	0	1	1	Unique								
4291	Prokka:CIODPCDJ_04647	-	hypothetical protein	1	1	1	1	1	1	1	1	1	1	1	11	CORE	COG3118_O#	38	179	2.44E-44	K05838	33.2	159	6.99E-38

cluster	GI	gene	Annotation	Streptomyces_thermocarboxydus_BPSAC147	Streptomyces_sp._4F	Streptomyces_sp._BSE7F	Streptomyces_sp._ETH9427	Streptomyces_sp._FxanaD5	Streptomyces_sp._UNC401CLCol	Actinospica_acidiphila_NRRL_B_24431	Streptomyces_sp._HNS054	Streptomyces_sp._SMS_SU21	Streptomyces_sp._ZS0098	Streptomyces_thermocarboxydus_K155	Addition	Core-Accessory-Unique	COG	%Similarity_COG	Score_COG	Evalue_COG	KEGG	%Similarity_KEGG	Score_KEGG	Evalue_KEGG
4292	OSC72004.1	-	hypothetical protein	0	1	0	0	0	0	0	0	0	0	0	1	Unique								
4293	OSC57227.1	-	cation transporter	0	1	0	0	0	0	0	0	0	0	0	1	Unique	COG0053_P#	38.8	192	2.14E-48	K14696	29.6	128	1.01E-28
4294	WP_051101607.1	-	5-dehydro-4-deoxyglucarate dehydratase	1	1	0	0	1	1	0	0	0	0	1	5	Accessory	COG0329_EM#	39.7	218	2.78E-56	K01707	32.2	172	6.11E-42
4295	WP_048459405.1	-	DMT family transporter	1	1	1	1	1	1	1	1	1	1	1	11	CORE	COG0697_GER#	34.7	146	2.29E-34				
4296	OSC69007.1	-	hypothetical protein	0	1	0	0	0	0	0	0	0	0	0	1	Unique								
4297	OSC69305.1	-	ABC transporter permease	0	1	0	0	0	0	0	0	0	0	0	1	Unique	COG1175_G#	34.3	168	4.31E-41	K10118	34.3	168	1.15E-40
4298	WP_122215303.1	-	hypothetical protein	0	0	0	0	0	0	0	0	0	1	0	1	Unique								
4299	OSC71385.1	-	hypothetical protein	0	1	0	0	0	0	0	0	0	0	0	1	Unique	COG1131_V#	34.9	153	1.88E-36	K01990	29.9	131	2.04E-29
4300	OSC56037.1	-	peptidase M28	0	1	0	0	0	0	0	0	0	0	0	1	Unique	COG2234_R#	53.6	225	2.28E-58	NA	49.8	212	5.33E-54
4301	OSC69317.1	-	AraC family transcriptional regulator	1	1	0	1	1	1	1	1	1	1	1	10	Accessory	COG4977_K#	47.9	282	1.57E-75	NA	46.4	77	3.49E-13
4302	Prokka:CIODPCDJ_06764	-	hypothetical protein	0	0	0	0	1	0	0	0	0	0	1	2	Accessory								

cluster	GI	gene	Annotation	Streptomyces_thermocarboxydus_BPSAC147	Streptomyces_sp._4F	Streptomyces_sp._BSE7F	Streptomyces_sp._ETH9427	Streptomyces_sp._FxanaD5	Streptomyces_sp._UNC401CLCol	Actinospica_acidiphila_NRRL_B_24431	Streptomyces_sp._HNS054	Streptomyces_sp._SMS_SU21	Streptomyces_sp._ZS0098	Streptomyces_thermocarboxydus_K155	Addition	Core-Accessory-Unique	COG	%Similarity_COG	Score_COG	Evalue_COG	KEGG	%Similarity_KEGG	Score_KEGG	Evalue_KEGG
4303	WP_033274701.1	-	NAD(P)-dependent alcohol dehydrogenase	0	1	1	0	1	1	1	0	1	0	1	7	Accessory	COG0604_CR#	40.8	198	2.97E-50	NA	33.8	150	3.24E-35
4304	WP_078611393.1	-	LysR family transcriptional regulator	1	1	1	1	1	1	1	0	1	1	1	10	Accessory	COG0583_K#	37.3	158	4.45E-38	NA	32.8	141	1.15E-32
4305	WP_033272026.1	-	amino acid ABC transporter permease	1	1	1	1	1	1	1	1	1	1	1	11	CORE	COG0765_E#	49.1	278	2.95E-74	K02029	41	160	3.13E-38
4306	PWE10244.1	-	2-dehydropantoate 2-reductase	1	1	1	1	1	1	1	1	1	1	1	11	CORE	COG1893_H#	46.4	295	1.79E-79	K00077	24.1	96	5.55E-19
4307	WP_019524853.1	-	hypothetical protein	0	0	0	0	1	1	0	0	0	0	1	3	Accessory								
4308	WP_026243919.1	-	aminoglycoside phosphotransferase family protein	0	0	0	0	1	0	0	0	0	0	1	2	Accessory								
4309	AXI88811.1	-	methylmalonyl Co-A mutase-associated GTPase MeaB	1	1	1	1	1	1	1	1	1	1	1	11	CORE	COG1703_E#	49.1	307	5.93E-83	K07588	50.3	274	1.14E-72
4310	WP_048456592.1	-	LLM class F420-dependent oxidoreductase	1	0	1	1	1	1	1	1	1	1	1	10	Accessory	COG2141_C#	38.4	188	4.02E-47	K15510	32.7	174	1.23E-42

cluster	GI	gene	Annotation	Streptomyces_thermocarboxydus_BPSAC147	Streptomyces_sp._4F	Streptomyces_sp._BSE7F	Streptomyces_sp._ETH9427	Streptomyces_sp._FxanaD5	Streptomyces_sp._UNC401CLCol	Actinospica_acidiphila_NRRL_B_24431	Streptomyces_sp._HNS054	Streptomyces_sp._SMS_SU21	Streptomyces_sp._ZS0098	Streptomyces_thermocarboxydus_K155	Addition	Core-Accessory-Unique	COG	%Similarity_COG	Score_COG	Evalue_COG	KEGG	%Similarity_KEGG	Score_KEGG	Evalue_KEGG
4311	OSC63110.1	-	hypothetical protein	0	1	0	0	0	0	0	0	0	0	0	1	Unique								
4312	WP_033273532.1	-	alpha/beta hydrolase	0	0	0	0	1	1	1	0	0	0	1	4	Accessory	COG0657_I#	50.9	300	7.25E-81	NA	35.6	170	2.31E-41
4313	AXI89677.1	-	CoA ester lyase	1	1	1	1	1	1	1	1	1	1	1	11	CORE	COG2301_G#	43.3	238	4.42E-62	K01644	30.4	147	2.74E-34
4314	WP_102638811.1	-	DUF3068 domain-containing protein	0	0	1	1	0	0	1	1	1	1	0	6	Accessory								
4315	OSC71615.1	-	nitrate ABC transporter substrate-binding protein	1	1	0	0	1	1	1	0	0	0	1	6	Accessory	COG4521_P#	26.1	84	8.17E-16	K11950	24.7	76	4.55E-13
4316	WP_122216792.1	-	5-dehydro-4-deoxyglucarate dehydratase	1	1	1	1	1	1	1	1	1	1	1	11	CORE	COG0329_EM#	45.4	234	4.89E-61	K01707	38.4	210	3.44E-53
4317	OSC71194.1	-	oxidoreductase	0	1	0	0	0	0	0	0	0	0	0	1	Unique	COG1028_IQR#	45.2	239	1.98E-62	NA	45.2	239	5.3E-62
4318	PWE07947.1	-	transcriptional regulator	1	1	1	1	1	1	1	1	1	1	1	11	CORE	COG2378_K#	35.1	150	1.21E-35	NA	35.1	150	3.24E-35
4319	OSC63090.1	-	oxidoreductase	0	1	0	0	0	0	1	0	0	0	0	2	Accessory	COG1028_IQR#	47.2	252	2.96E-66	NA	47.2	252	7.9E-66
4320	WP_033277649.1	-	hypothetical protein	1	1	1	1	1	1	1	1	1	1	1	11	CORE								
4321	WP_167338237.1	-	NAD-binding protein	1	1	1	1	1	1	1	1	1	1	1	11	CORE	COG2084_I#	32	129	2.21E-29	K18121	32.2	134	1.84E-30

cluster	GI	gene	Annotation	Streptomyces_thermocarboxydus_BPSAC147	Streptomyces_sp._4F	Streptomyces_sp._BSE7F	Streptomyces_sp._ETH9427	Streptomyces_sp._FxanaD5	Streptomyces_sp._UNC401CLCol	Actinospica_acidiphila_NRRL_B_24431	Streptomyces_sp._HNS054	Streptomyces_sp._SMS_SU21	Streptomyces_sp._ZS0098	Streptomyces_thermocarboxydus_K155	Addition	Core-Accessory-Unique	COG	%Similarity_COG	Score_COG	Evalue_COG	KEGG	%Similarity_KEGG	Score_KEGG	Evalue_KEGG
4322	WP_052183396.1	-	NADP-dependent oxidoreductase	1	0	0	0	1	1	0	0	0	0	0	3	Accessory	COG0604_CR#	32.9	158	5.81E-38	K12433	29.4	107	2.41E-22
4323	AXI91090.1	-	LacI family transcriptional regulator	0	0	1	1	0	0	0	1	1	1	0	5	Accessory	COG1609_K#	37.8	173	1.02E-42	K02529	34.4	155	7.7E-37
4324	OSC70947.1	-	hypothetical protein	1	1	1	1	1	1	1	1	1	1	1	11	CORE								
4325	OSC69950.1	-	thymidylate synthase	0	1	0	0	0	0	0	0	0	0	0	1	Unique								
4326	OSC71863.1	-	hydroxylase	0	1	0	0	0	0	0	0	0	0	0	1	Unique	COG0179_Q#	43.6	231	3.17E-60	NA	43.1	236	2.63E-61
4327	OSC59826.1	-	hypothetical protein	0	1	0	0	0	0	0	0	0	0	0	1	Unique	COG3321_Q#	38.8	155	3.77E-37	K12434	38.8	155	1.01E-36
4328	Prokka:JAPCLKMA_02986	-	hypothetical protein	1	0	0	1	1	1	1	1	1	1	1	9	Accessory								
4329	WP_051724416.1	-	ROK family protein	0	1	0	0	0	0	1	0	0	0	0	2	Accessory	COG1940_KG#	36.8	151	5.44E-36	K00845	36.8	151	1.45E-35
4330	WP_019524591.1	cas1e	type I-E CRISPR-associated endonuclease Cas1	0	0	0	0	1	0	0	0	0	0	1	2	Accessory	COG1518_L#	36.8	191	4.74E-48	K15342	36.8	191	1.27E-47
4331	OSC57311.1	-	ATPase	1	1	1	1	1	1	1	1	1	1	1	11	CORE					NA	27.1	103	4.54E-21
4332	AXI91305.1	-	hypothetical protein	0	0	0	1	0	0	0	0	0	0	0	1	Unique								
4333	WP_122213716.1	lepB	signal peptidase I	1	1	1	1	1	1	1	1	1	1	1	11	CORE	COG0681_U#	41.3	165	3.63E-40	K03100	38.9	165	9.69E-40
4334	AXI86831.1	speB	agmatinase	1	1	1	1	1	1	1	1	1	1	1	11	CORE	COG0010_E#	44.8	276	1.12E-73	K01480	41.4	256	4.18E-67
4335	WP_048454919.1	-	aldo/keto reductase	1	1	1	1	1	1	1	1	1	1	1	11	CORE	COG0667_C#	59.7	409	1.44E-113	NA	61.1	403	2.76E-111

cluster	GI	gene	Annotation	Streptomyces_thermocarboxydus_BPSAC147	Streptomyces_sp._4F	Streptomyces_sp._BSE7F	Streptomyces_sp._ETH9427	Streptomyces_sp._FxanaD5	Streptomyces_sp._UNC401CLCol	Actinospica_acidiphila_NRRL_B_24431	Streptomyces_sp._HNS054	Streptomyces_sp._SMS_SU21	Streptomyces_sp._ZS0098	Streptomyces_thermocarboxydus_K155	Addition	Core-Accessory-Unique	COG	%Similarity_COG	Score_COG	Evalue_COG	KEGG	%Similarity_KEGG	Score_KEGG	Evalue_KEGG
4336	OSC63098.1	-	hypothetical protein	1	1	1	1	1	1	1	1	1	1	1	11	CORE	COG1295_S#	30.4	132	2E-30	K07058	27.6	118	1.36E-25
4337	AXI88166.1	-	NADH-quinone oxidoreductase subunit H	1	1	1	1	1	1	1	1	1	1	1	11	CORE	COG1005_C#	41.3	253	7.76E-67	K05572	40.4	226	2.71E-58
4338	WP_028961882.1	-	DNA polymerase	1	1	1	1	1	1	1	1	1	1	1	11	CORE	COG0389_L#	29.1	121	7.85E-27	K02346	30.2	98	1.11E-19
4339	AXI87924.1	-	exopolyphosphatase	1	1	1	1	1	1	1	1	1	1	1	11	CORE	COG0248_FP#	53.5	296	1.04E-79	K01524	53.5	296	2.79E-79
4340	Prokka:JAPCLKMA_00481	btuD_3	Vitamin B12 import ATP-binding protein BtuD	1	1	1	1	1	1	1	1	1	1	1	11	CORE	COG1131_V#	41.7	164	4.74E-40	K01990	34.4	160	2.39E-38
4341	WP_019522925.1	-	ABC transporter substrate-binding protein	1	1	1	1	1	1	1	1	1	1	1	11	CORE	COG0834_ET#	31.3	133	1.17E-30	K02030	32.8	129	4.51E-29
4342	Prokka:JAPCLKMA_00604	mobA	Molybdenum cofactor guanylyltransferase	1	1	1	1	1	1	1	1	1	1	1	11	CORE	COG0746_H#	38.1	89	1.94E-17	NA	28.7	52	0.000012
4343	WP_019522904.1	-	hypothetical protein	1	1	1	1	1	1	1	1	1	1	1	11	CORE	COG1597_IR#	41	223	1.12E-57	NA	41	223	3E-57
4344	Prokka:CIODPCDJ_05321	-	hypothetical protein	0	0	0	0	0	0	0	0	0	0	1	1	Unique								
4345	OSC67469.1	-	non-ribosomal peptide synthetase	0	1	0	0	0	0	0	0	0	0	0	1	Unique	COG1020_Q#	41.2	263	1.28E-69	K04780	43.3	230	1.88E-59

cluster	GI	gene	Annotation	Streptomyces_thermocarboxydus_BPSAC147	Streptomyces_sp._4F	Streptomyces_sp._BSE7F	Streptomyces_sp._ETH9427	Streptomyces_sp._FxanaD5	Streptomyces_sp._UNC401CLCol	Actinospica_acidiphila_NRRL_B_24431	Streptomyces_sp._HNS054	Streptomyces_sp._SMS_SU21	Streptomyces_sp._ZS0098	Streptomyces_thermocarboxydus_K155	Addition	Core-Accessory-Unique	COG	%Similarity_COG	Score_COG	Evalue_COG	KEGG	%Similarity_KEGG	Score_KEGG	Evalue_KEGG
4346	AXI89658.1	-	transposase	0	0	0	1	0	0	0	0	0	0	0	1	Unique								
4347	OSC66918.1	-	thiamine-monophosphate kinase	1	1	1	1	1	1	1	1	1	1	1	11	CORE	COG0611_H#	50.3	283	7E-76	K00946	50.3	283	1.87E-75
4348	OSC70419.1	-	NUDIX hydrolase	1	1	1	1	1	1	1	1	1	1	1	11	CORE	COG0494_LR#	45.8	223	8.6E-58	NA	45.8	223	2.3E-57
4349	Prokka:JAPCLKMA_00822	-	hypothetical protein	1	1	1	1	1	1	1	1	1	1	1	11	CORE								
4350	OSC72856.1	-	alpha/beta hydrolase	1	1	1	1	1	1	1	1	1	1	1	11	CORE	COG0596_R#	36.4	158	5.8E-38	NA	37.5	157	2.64E-37
4351	Prokka:JAPCLKMA_06321	-	hypothetical protein	1	0	0	0	0	0	0	0	0	0	0	1	Unique								
4352	OSC70862.1	-	alpha/beta hydrolase	1	1	1	1	1	1	1	1	1	1	1	11	CORE	COG0596_R#	42.1	218	4.72E-56	NA	40.7	213	4.05E-54
4353	WP_102637487.1	-	GlxA family transcriptional regulator	0	1	0	0	0	0	0	1	1	0	0	3	Accessory	COG4977_K#	48.2	260	6.35E-69	NA	30.9	101	1.32E-20
4354	OSC64609.1	-	hypothetical protein	1	1	1	1	1	1	1	1	1	1	1	11	CORE	COG1714_S#	41.9	211	4.42E-54	NA	41.9	211	1.18E-53
4355	AXI91265.1	-	hypothetical protein	0	0	0	1	0	0	0	0	0	0	0	1	Unique								
4356	OSC70692.1	-	carbohydrate-binding protein	1	1	1	0	1	1	1	1	1	1	1	10	Accessory								
4357	PWE09122.1	-	DeoR/GlpR transcriptional regulator	1	1	1	1	1	1	1	1	1	1	1	11	CORE	COG1349_KG#	33.7	149	1.57E-35	K03436	33.7	149	4.2E-35

cluster	GI	gene	Annotation	Streptomyces_thermocarboxydus_BPSAC147	Streptomyces_sp._4F	Streptomyces_sp._BSE7F	Streptomyces_sp._ETH9427	Streptomyces_sp._FxanaD5	Streptomyces_sp._UNC401CLCol	Actinospica_acidiphila_NRRL_B_24431	Streptomyces_sp._HNS054	Streptomyces_sp._SMS_SU21	Streptomyces_sp._ZS0098	Streptomyces_thermocarboxydus_K155	Addition	Core-Accessory-Unique	COG	%Similarity_COG	Score_COG	Evalue_COG	KEGG	%Similarity_KEGG	Score_KEGG	Evalue_KEGG
4358	AXI84827.1	-	NADPH:quinone reductase	0	1	1	1	1	1	1	1	1	1	1	10	Accessory	COG0604_CR#	47.1	256	1.19E-67	NA	35.3	176	4.19E-43
4359	AXI87603.1	-	folate-binding protein YgfZ	1	1	1	1	1	1	1	1	1	1	1	11	CORE	COG0354_R#	43.6	226	1.32E-58	K06980	43.6	226	3.53E-58
4360	WP_028960346.1	-	hypothetical protein	1	0	1	1	1	1	1	1	1	1	1	10	Accessory								
4361	WP_048458677.1	-	YihY/virulence factor BrkB family protein	0	1	1	1	0	0	0	1	1	1	0	6	Accessory								
4362	OSC71867.1	-	cyclase	0	1	0	0	0	0	0	0	0	0	0	1	Unique								
4363	Prokka:CIODPCDJ_04272	-	2-haloacrylate reductase	1	0	0	1	1	1	1	1	0	1	1	8	Accessory	COG0604_CR#	36.4	157	7.55E-38	K00344	36.1	157	2.02E-37
4364	Prokka:CIODPCDJ_01820	lipA	Lipoyl synthase	1	1	1	1	1	1	1	1	1	1	1	11	CORE	COG0320_H#	73.3	470	5.26E-132	K03644	73.3	470	1.4E-131
4365	PWE10130.1	-	endoglucanase	0	0	1	1	0	0	1	1	1	1	0	6	Accessory	COG5297_G#	38.9	208	3.73E-53	K01179	39.3	208	9.95E-53
4366	WP_019522927.1	-	zinc-binding dehydrogenase	1	1	1	1	1	1	1	1	1	1	1	11	CORE	COG0604_CR#	37.6	190	1.37E-47	K13955	37.6	190	3.66E-47
4367	AXI85911.1	-	hypothetical protein	1	1	1	1	1	1	1	1	1	1	1	11	CORE								
4368	AXI84809.1	-	RNA polymerase subunit sigma-24	0	1	1	1	0	0	1	1	1	1	0	7	Accessory	COG1595_K#	40.9	178	5.39E-44	K03088	40.9	178	1.44E-43
4369	WP_102638008.1	-	hypothetical protein	0	0	0	0	0	0	0	0	1	0	0	1	Unique								

cluster	GI	gene	Annotation	Streptomyces_thermocarboxydus_BPSAC147	Streptomyces_sp._4F	Streptomyces_sp._BSE7F	Streptomyces_sp._ETH9427	Streptomyces_sp._FxanaD5	Streptomyces_sp._UNC401CLCol	Actinospica_acidiphila_NRRL_B_24431	Streptomyces_sp._HNS054	Streptomyces_sp._SMS_SU21	Streptomyces_sp._ZS0098	Streptomyces_thermocarboxydus_K155	Addition	Core-Accessory-Unique	COG	%Similarity_COG	Score_COG	Evalue_COG	KEGG	%Similarity_KEGG	Score_KEGG	Evalue_KEGG
4370	OSC64576.1	-	ABC transporter	1	1	1	1	1	1	1	1	1	1	1	11	CORE	COG1131_V#	53.2	243	8.01E-64	K01990	45	243	2.79E-63
4371	OSC71173.1	-	thioredoxin reductase	1	1	1	1	1	1	1	1	1	1	1	11	CORE	COG0492_O#	37.8	203	1.57E-51	NA	38.2	192	5.65E-48
4372	OSC72095.1	-	amidohydrolase	0	1	0	0	0	0	0	0	0	0	0	1	Unique	COG2159_R#	28.3	107	8.95E-23	K14333	28.3	107	2.39E-22
4373	OSC66756.1	-	hypothetical protein	0	1	0	0	0	0	0	0	0	0	0	1	Unique								
4374	OSC72047.1	-	hydroxymethylglutaryl-CoA lyase	1	1	1	1	1	1	1	1	1	1	1	11	CORE	COG0119_E#	46.8	272	2.75E-72	NA	54	312	4.89E-84
4375	WP_019523170.1	-	hypothetical protein	1	1	1	1	1	1	1	1	1	1	1	11	CORE								
4376	WP_026244216.1	-	D-alanine--D-alanine ligase	0	0	0	0	1	0	0	0	0	0	0	1	Unique	COG1181_M#	37.4	170	8.59E-42	K01921	38.3	169	3.91E-41
4377	Prokka:JAPCLKMA_06091	egtD	Histidine N-alpha-methyltransferase	1	1	1	1	1	1	1	1	1	1	1	11	CORE	COG4301_S#	59	345	1.95E-94	K18911	59	345	5.2E-94
4378	Prokka:JAPCLKMA_04030	-	hypothetical protein	1	1	1	1	1	1	1	1	1	1	1	11	CORE	COG2310_T#	34.5	109	2.35E-23	NA	37.6	107	2.38E-22
4379	WP_048456437.1	-	electron transfer flavoprotein subunit alpha/FixB family protein	1	1	1	1	1	1	1	1	1	1	1	11	CORE	COG2025_C#	58.8	349	1.76E-95	K03522	58.8	349	4.7E-95
4380	AXI91421.1	-	radical SAM protein	0	0	0	1	0	0	0	0	0	0	0	1	Unique	COG0535_R#	24.5	73	1.87E-12				

cluster	GI	gene	Annotation	Streptomyces_thermocarboxydus_BPSAC147	Streptomyces_sp._4F	Streptomyces_sp._BSE7F	Streptomyces_sp._ETH9427	Streptomyces_sp._FxanaD5	Streptomyces_sp._UNC401CLCol	Actinospica_acidiphila_NRRL_B_24431	Streptomyces_sp._HNS054	Streptomyces_sp._SMS_SU21	Streptomyces_sp._ZS0098	Streptomyces_thermocarboxydus_K155	Addition	Core-Accessory-Unique	COG	%Similarity_COG	Score_COG	Evalue_COG	KEGG	%Similarity_KEGG	Score_KEGG	Evalue_KEGG
4381	WP_048459401.1	-	EamA family transporter	1	1	1	1	1	1	1	1	1	1	1	11	CORE	COG0697_GER#	29.5	116	1.92E-25	NA	30	72	8.5E-12
4382	AXI86639.1	-	GTPase Era	1	1	1	1	1	1	1	1	1	1	1	11	CORE	COG1159_R#	63.7	381	4.18E-105	K03595	64	381	1.12E-104
4383	WP_033274436.1	-	ABC transporter permease	1	0	1	1	1	1	1	1	1	1	1	10	Accessory	COG1172_G#	40.7	231	3.14E-60	K02057	37.8	195	8.69E-49
4384	WP_102640158.1	-	helix-turn-helix domain-containing protein	0	0	1	1	0	0	0	1	1	1	0	5	Accessory	COG2207_K#	31.5	121	5.97E-27	K14063	25.6	85	9.7E-16
4385	OSC71051.1	-	5,10-methylene tetrahydromethanopterin reductase	0	1	0	0	0	0	0	0	0	0	0	1	Unique	COG2141_C#	25.8	49	0.0000221	NA	25.8	49	0.000059
4386	Prokka:CIODPCDJ_00561	ugpA	sn-glycerol-3-phosphate transport system permease protein UgpA	1	0	0	0	1	1	0	0	0	0	1	4	Accessory	COG1175_G#	32.3	132	1.98E-30	K05814	26.8	124	2.46E-27
4387	Prokka:CIODPCDJ_05953	-	hypothetical protein	1	0	1	1	1	1	1	1	1	1	1	10	Accessory	COG3662_S#	36.2	165	2.76E-40	NA	36.2	165	7.38E-40
4388	WP_033277681.1	-	ROK family protein	1	1	1	1	1	1	1	1	1	1	1	11	CORE	COG1940_KG#	53.6	298	2.73E-80	K00845	53.6	298	7.29E-80
4389	Prokka:CIODPCDJ_01626	-	hypothetical protein	0	0	0	0	0	0	0	0	0	0	1	1	Unique	COG1643_L#	46.1	174	7.77E-43	K03579	44.8	159	6.9E-38

cluster	GI	gene	Annotation	Streptomyces_thermocarboxydus_BPSAC147	Streptomyces_sp._4F	Streptomyces_sp._BSE7F	Streptomyces_sp._ETH9427	Streptomyces_sp._FxanaD5	Streptomyces_sp._UNC401CLCol	Actinospica_acidiphila_NRRL_B_24431	Streptomyces_sp._HNS054	Streptomyces_sp._SMS_SU21	Streptomyces_sp._ZS0098	Streptomyces_thermocarboxydus_K155	Addition	Core-Accessory-Unique	COG	%Similarity_COG	Score_COG	Evalue_COG	KEGG	%Similarity_KEGG	Score_KEGG	Evalue_KEGG
4390	WP_086020073.1	-	pseudouridylate synthase	1	1	0	1	1	1	1	1	1	0	1	9	Accessory	COG0564_J#	54.8	289	2.16E-77	K06177	54.8	289	5.78E-77
4391	WP_051724633.1	-	hypothetical protein	0	0	0	0	0	0	1	0	0	0	0	1	Unique								
4392	AXI91358.1	-	hypothetical protein	0	0	0	1	0	0	0	0	0	0	0	1	Unique								
4393	Prokka:CIODPCDJ_02679	-	hypothetical protein	1	0	1	1	1	1	1	1	1	1	1	10	Accessory	COG5309_G#	29.8	45	0.000542	NA	39.4	133	4.04E-30
4394	WP_028960891.1	-	sugar ABC transporter permease	1	1	1	1	1	1	1	1	1	1	1	11	CORE	COG1175_G#	32.2	153	1.08E-36	K10118	28.8	141	1.94E-32
4395	PWE12258.1	-	(2Fe-2S)-binding protein	1	0	1	1	1	1	1	1	1	1	1	10	Accessory	COG2146_PR#	36.1	63	1.92E-09	NA	41.2	119	7.89E-26
4396	Prokka:JAPCLKMA_05917	fbiA	Phosphoenolpyruvate transferase	1	1	1	1	1	1	1	1	1	1	1	11	CORE	COG0391_S#	55.2	370	7.36E-102	K11212	55.2	370	1.97E-101
4397	WP_048460110.1	-	EamA family transporter	1	1	1	1	1	1	1	1	1	1	1	11	CORE	COG5006_R#	45.9	232	1.83E-60	K11939	43.8	219	4.29E-56
4398	WP_033273791.1	-	dihydrodipicolinate synthase family protein	0	1	0	0	0	0	1	0	0	0	0	2	Accessory	COG0329_EM#	50.8	282	1.55E-75	K01714	29.9	146	4.62E-34
4399	WP_102637676.1	-	family 43 glycosylhydrolase	0	0	1	1	0	0	0	1	1	1	0	5	Accessory								
4400	WP_102638230.1	-	threonine/serine dehydratase	1	1	1	0	1	1	1	1	1	1	1	10	Accessory	COG1171_E#	40.1	188	5.18E-47	K01754	38.4	181	9.92E-45

cluster	GI	gene	Annotation	Streptomyces_thermocarboxydus_BPSAC147	Streptomyces_sp._4F	Streptomyces_sp._BSE7F	Streptomyces_sp._ETH9427	Streptomyces_sp._FxanaD5	Streptomyces_sp._UNC401CLCol	Actinospica_acidiphila_NRRL_B_24431	Streptomyces_sp._HNS054	Streptomyces_sp._SMS_SU21	Streptomyces_sp._ZS0098	Streptomyces_thermocarboxydus_K155	Addition	Core-Accessory-Unique	COG	%Similarity_COG	Score_COG	Evalue_COG	KEGG	%Similarity_KEGG	Score_KEGG	Evalue_KEGG
4401	WP_078611413.1	-	MBL fold metallo-hydrolase	0	0	0	0	1	1	1	0	0	0	1	4	Accessory	COG0491_R#	35.7	198	2.94E-50	NA	33.9	156	3.42E-37
4402	OSC64984.1	-	chlorophyllase	0	1	0	0	0	0	0	0	0	0	0	1	Unique	COG4188_R#	47.2	261	3.69E-69	NA	26.7	72	8.47E-12
4403	WP_019524363.1	-	ABC transporter permease	1	0	1	1	1	1	1	1	1	1	1	10	Accessory	COG1173_EP#	40.7	253	1.31E-66	K12370	40.2	217	1.63E-55
4404	Prokka:JAPCLKMA_04899	-	hypothetical protein	1	0	0	0	0	0	0	0	0	0	0	1	Unique	COG1396_K#	34.8	48	0.0000491				
4405	OSC62668.1	-	epimerase	0	1	0	0	0	0	0	0	0	0	0	1	Unique	COG0451_MG#	31.6	124	9.19E-28				
4406	PWE08734.1	-	hydroxymethylbilane synthase	1	1	1	1	1	1	1	1	1	1	1	11	CORE	COG0181_H#	51.4	276	1.11E-73	K01749	51.4	276	2.96E-73
4407	Prokka:CIODPCDJ_03757	hprA	Glycerate dehydrogenase	1	0	0	0	1	1	0	0	0	0	1	4	Accessory	COG0111_HE#	39.1	216	1.36E-55	K00015	37.6	167	2.53E-40
4408	WP_026243833.1	-	tyrosine-type recombinase/integrase	0	0	0	0	1	0	0	0	0	0	0	1	Unique								
4409	Prokka:JAPCLKMA_02112	-	hypothetical protein	1	1	0	0	1	1	1	1	1	1	1	9	Accessory	COG0859_M#	34.7	60	1.63E-08	K02841	30.7	52	0.0000118
4410	OSC67217.1	-	peptide ABC transporter ATP-binding protein	0	1	0	0	0	0	0	0	0	0	0	1	Unique	COG1123_R#	49.5	262	2.16E-69	K02032	49.5	262	5.77E-69
4411	Prokka:JAPCLKMA_05577	-	hypothetical protein	1	0	0	0	0	0	0	0	0	0	0	1	Unique								

cluster	GI	gene	Annotation	Streptomyces_thermocarboxydus_BPSAC147	Streptomyces_sp._4F	Streptomyces_sp._BSE7F	Streptomyces_sp._ETH9427	Streptomyces_sp._FxanaD5	Streptomyces_sp._UNC401CLCol	Actinospica_acidiphila_NRRL_B_24431	Streptomyces_sp._HNS054	Streptomyces_sp._SMS_SU21	Streptomyces_sp._ZS0098	Streptomyces_thermocarboxydus_K155	Addition	Core-Accessory-Unique	COG	%Similarity_COG	Score_COG	Evalue_COG	KEGG	%Similarity_KEGG	Score_KEGG	Evalue_KEGG
4412	OSC61050.1	-	glycine/betaine ABC transporter substrate-binding protein	0	1	1	1	1	1	1	1	1	1	0	9	Accessory	COG1732_M#	37.5	180	1.08E-44	K05845	37.5	179	4.92E-44
4413	OSC72547.1	-	hypothetical protein	0	1	0	0	1	0	0	0	0	0	1	3	Accessory								
4414	WP_048457007.1	-	hypothetical protein	0	0	0	0	0	0	0	1	0	0	0	1	Unique								
4415	OSC71014.1	-	ABC transporter permease	1	1	0	0	1	1	1	0	0	0	1	6	Accessory	COG1173_EP#	39.6	241	3.95E-63	K02034	35.4	187	2.36E-46
4416	WP_028961498.1	-	TerC family protein	1	1	1	1	1	1	1	1	1	1	1	11	CORE	COG0861_P#	46	263	7.41E-70	K05794	43.9	253	2.67E-66
4417	WP_122215378.1	-	ABC transporter substrate-binding protein	1	1	0	1	1	1	1	1	1	1	1	10	Accessory	COG2113_E#	26.9	110	1.05E-23				
4418	WP_019523321.1	-	carbohydrate ABC transporter permease	1	1	0	1	1	1	1	1	1	1	1	10	Accessory	COG0395_G#	41.9	211	3.34E-54	K17320	41.9	211	8.92E-54
4419	Prokka:CIODPCDJ_03497	-	hypothetical protein	0	0	0	0	0	0	1	0	0	0	1	2	Accessory								
4420	WP_122215121.1	-	LLM class F420-dependent oxidoreductase	0	0	0	1	0	0	0	0	1	1	0	3	Accessory	COG2141_C#	41.4	218	2.73E-56	NA	41.4	218	7.3E-56

cluster	GI	gene	Annotation	Streptomyces_thermocarboxydus_BPSAC147	Streptomyces_sp._4F	Streptomyces_sp._BSE7F	Streptomyces_sp._ETH9427	Streptomyces_sp._FxanaD5	Streptomyces_sp._UNC401CLCol	Actinospica_acidiphila_NRRL_B_24431	Streptomyces_sp._HNS054	Streptomyces_sp._SMS_SU21	Streptomyces_sp._ZS0098	Streptomyces_thermocarboxydus_K155	Addition	Core-Accessory-Unique	COG	%Similarity_COG	Score_COG	Evalue_COG	KEGG	%Similarity_KEGG	Score_KEGG	Evalue_KEGG
4421	WP_033274430.1	-	sugar ABC transporter permease	1	1	0	0	1	1	1	0	0	0	1	6	Accessory	COG1175_G#	39.3	207	6.3E-53	K10118	34.8	191	1.25E-47
4422	Prokka:CIODPCDJ_05534	cdhR_6	HTH-type transcriptional regulator CdhR	0	1	0	0	1	1	0	0	0	0	1	4	Accessory	COG4977_K#	47.6	277	8.45E-74	NA	45.2	71	1.44E-11
4423	OSC64378.1	-	multidrug ABC transporter ATP-binding protein	0	1	0	0	0	0	0	0	0	0	0	1	Unique	COG1131_V#	36.4	213	1.5E-54	K01990	37	188	8.09E-47
4424	PWE11038.1	-	sensor histidine kinase	0	0	1	0	0	0	0	0	0	0	0	1	Unique								
4425	WP_122217289.1	-	hypothetical protein	0	0	0	1	1	1	1	1	1	1	1	8	Accessory					NA	41.5	63	5.12E-09
4426	OSC72005.1	-	3-oxoacyl-ACP synthase	0	1	0	0	0	0	0	0	0	0	0	1	Unique	COG0332_I#	43.9	250	8.47E-66	K11608	43.9	250	2.26E-65
4427	Prokka:CIODPCDJ_02907	-	hypothetical protein	1	1	1	1	1	1	1	1	1	1	1	11	CORE					NA	47.9	134	2.36E-30
4428	Prokka:JAPCLKMA_01608	-	IS3 family transposase ISPfr13	1	0	0	0	0	0	0	0	0	0	0	1	Unique	COG2801_L#	40.1	215	2.31E-55	NA	40.1	215	6.18E-55
4429	OSC72536.1	-	NAD-dependent dehydratase	0	1	0	0	0	0	0	0	0	0	0	1	Unique	COG0451_MG#	63.8	399	1.13E-110	K01784	63.8	399	3.02E-110
4430	OSC58174.1	-	hypothetical protein	0	1	0	0	0	0	0	0	0	0	0	1	Unique								
4431	WP_102639444.1	-	J domain-containing protein	0	0	0	0	0	0	0	0	1	1	0	2	Accessory	COG2214_O#	39.5	223	8.49E-58	K05516	39.3	216	3.62E-55

cluster	GI	gene	Annotation	Streptomyces_thermocarboxydus_BPSAC147	Streptomyces_sp._4F	Streptomyces_sp._BSE7F	Streptomyces_sp._ETH9427	Streptomyces_sp._FxanaD5	Streptomyces_sp._UNC401CLCol	Actinospica_acidiphila_NRRL_B_24431	Streptomyces_sp._HNS054	Streptomyces_sp._SMS_SU21	Streptomyces_sp._ZS0098	Streptomyces_thermocarboxydus_K155	Addition	Core-Accessory-Unique	COG	%Similarity_COG	Score_COG	Evalue_COG	KEGG	%Similarity_KEGG	Score_KEGG	Evalue_KEGG
4432	WP_037954000.1	-	MBL fold metallo-hydrolase	1	0	0	0	1	1	1	0	0	0	1	5	Accessory	COG0491_R#	28.9	97	9.18E-20	NA	28.9	97	2.45E-19
4433	WP_033273454.1	-	AraC family transcriptional regulator	1	0	1	1	1	1	1	1	1	1	1	10	Accessory	COG2207_K#	41.3	236	1.65E-61	K18991	28.3	107	1.81E-22
4434	WP_102637947.1	-	hypothetical protein	0	0	0	0	0	0	0	0	1	0	0	1	Unique								
4435	WP_158688210.1	-	hypothetical protein	0	0	0	0	1	0	0	0	0	0	0	1	Unique								
4436	WP_159020881.1	-	hypothetical protein	0	0	0	0	0	0	0	1	0	0	0	1	Unique								
4437	Prokka:CIODPCDJ_02999	-	IS481 family transposase ISRhba4	0	0	0	0	0	0	0	0	0	0	1	1	Unique	COG2801_L#	34.3	178	4.09E-44				
4438	OSC56509.1	-	cyclase	0	1	0	0	0	0	0	0	0	0	0	1	Unique								
4439	Prokka:JAPCLKMA_06412	-	hypothetical protein	1	0	1	1	1	1	1	1	1	1	1	10	Accessory								
4440	WP_019524972.1	-	FAD-binding oxidoreductase	1	1	1	1	1	1	1	1	1	1	1	11	CORE	COG0665_E#	44.3	250	1.1E-65	K00273	42.9	240	1.79E-62
4441	WP_102641731.1	-	LPXTG cell wall anchor domain-containing protein	1	1	1	1	1	1	1	1	1	1	1	11	CORE								
4442	WP_033273468.1	-	ABC transporter permease	1	0	0	0	0	1	1	0	0	0	1	4	Accessory	COG0601_EP#	39.7	217	7.92E-56	K13890	35.4	184	1.52E-45

cluster	GI	gene	Annotation	Streptomyces_thermocarboxydus_BPSAC147	Streptomyces_sp._4F	Streptomyces_sp._BSE7F	Streptomyces_sp._ETH9427	Streptomyces_sp._FxanaD5	Streptomyces_sp._UNC401CLCol	Actinospica_acidiphila_NRRL_B_24431	Streptomyces_sp._HNS054	Streptomyces_sp._SMS_SU21	Streptomyces_sp._ZS0098	Streptomyces_thermocarboxydus_K155	Addition	Core-Accessory-Unique	COG	%Similarity_COG	Score_COG	Evalue_COG	KEGG	%Similarity_KEGG	Score_KEGG	Evalue_KEGG
4443	Prokka:CIODPCDJ_04658	sugA_2	Trehalose transport system permease protein SugA	1	1	0	0	1	1	0	0	0	0	1	5	Accessory	COG1175_G#	32.2	183	9.71E-46	K17245	34.9	151	1.09E-35
4444	PWE10323.1	-	serine/threonine protein kinase	1	0	1	0	1	1	0	0	0	0	0	4	Accessory	COG0515_RTKL#	34.3	178	3.12E-44	K08884	34.3	178	8.34E-44
4445	Prokka:CIODPCDJ_00753	-	hypothetical protein	1	1	1	1	1	1	1	1	1	1	1	11	CORE	COG3336_S#	44.2	255	2.01E-67	K02351	44.2	255	5.37E-67
4446	AXI88439.1	deoC	deoxyribose-phosphate aldolase	1	1	1	1	1	1	1	1	1	1	1	11	CORE	COG0274_F#	47.8	246	1.59E-64	K01619	45.5	221	1.12E-56
4447	AXI85867.1	-	WYL domain-containing protein	1	1	1	1	1	1	1	1	1	1	1	11	CORE	COG2378_K#	39.3	190	1.35E-47	K13572	39.3	190	3.62E-47
4448	OSC72712.1	-	glucokinase	1	1	1	1	1	1	1	1	1	1	1	11	CORE	COG1940_KG#	42.4	263	1.26E-69	K00845	35	185	8.91E-46
4449	Prokka:JAPCLKMA_02890	melD_5	Melibiose/raffinose/stachyose import permease protein MelD	1	1	1	1	1	1	1	1	1	1	1	11	CORE	COG1175_G#	37.9	199	1.71E-50	K02025	36.2	181	1.29E-44
4450	OSC70717.1	-	sugar kinase	0	1	0	0	0	0	0	0	0	0	0	1	Unique	COG0524_G#	37.9	194	7.18E-49	K00874	35.1	149	5.42E-35
4451	OSC64943.1	-	LLM class F420-dependent oxidoreductase	0	1	0	0	0	0	0	0	0	0	0	1	Unique	COG2141_C#	36	182	2.16E-45	K15510	33.4	181	1.29E-44

cluster	GI	gene	Annotation	Streptomyces_thermocarboxydus_BPSAC147	Streptomyces_sp._4F	Streptomyces_sp._BSE7F	Streptomyces_sp._ETH9427	Streptomyces_sp._FxanaD5	Streptomyces_sp._UNC401CLCol	Actinospica_acidiphila_NRRL_B_24431	Streptomyces_sp._HNS054	Streptomyces_sp._SMS_SU21	Streptomyces_sp._ZS0098	Streptomyces_thermocarboxydus_K155	Addition	Core-Accessory-Unique	COG	%Similarity_COG	Score_COG	Evalue_COG	KEGG	%Similarity_KEGG	Score_KEGG	Evalue_KEGG
4452	AXI84561.1	-	hypothetical protein	0	0	0	1	0	0	0	0	0	0	0	1	Unique								
4453	WP_107080825.1	-	AraC family transcriptional regulator	0	0	0	0	0	0	0	1	0	0	0	1	Unique	COG2207_K#	23.4	41	0.00596	NA	33.7	57	2.15E-07
4454	WP_048458453.1	-	dihydrodipicolinate synthase family protein	1	1	1	1	1	1	1	1	1	1	1	11	CORE	COG0329_EM#	36.5	185	2.56E-46				
4455	WP_048459719.1	-	DUF1918 domain-containing protein	1	1	1	1	1	1	1	1	1	1	1	11	CORE	COG1396_K#	26.9	43	0.00157	NA	42.5	58	1.26E-07
4456	WP_019521652.1	-	hypothetical protein	0	0	1	0	1	1	1	0	0	0	0	4	Accessory								
4457	WP_107685364.1	-	hypothetical protein	0	1	0	0	0	0	1	1	1	0	0	4	Accessory	COG3300_T#	39	136	2.32E-31				
4458	OSC67604.1	-	ABC transporter permease	1	1	1	1	1	1	1	1	1	1	1	11	CORE	COG0395_G#	34.3	170	8.51E-42	K17236	30.4	146	3.51E-34
4459	PWE08579.1	-	amino acid oxidase	0	0	1	0	0	0	0	0	0	0	0	1	Unique	COG0665_E#	48.6	281	3.42E-75	K00273	48.6	281	9.14E-75
4460	OSC70658.1	-	hypothetical protein	0	1	0	0	0	0	0	0	0	0	0	1	Unique								
4461	Prokka:CIODPCDJ_01213	-	5'-3' exonuclease	1	1	0	1	1	1	1	1	1	1	1	10	Accessory	COG0258_L#	53.4	298	2.7E-80	NA	53.4	298	9.43E-80
4462	OSC59244.1	-	ABC transporter permease	0	1	0	0	0	0	0	0	0	0	0	1	Unique	COG0601_EP#	42.1	244	6.05E-64	K15581	37	205	1.09E-51
4463	OSC64188.1	-	EamA family transporter	1	1	0	1	1	1	1	1	1	1	1	10	Accessory	COG0697_GER#	48.4	250	1.1E-65	K15268	37.4	206	2.86E-52

cluster	GI	gene	Annotation	Streptomyces_thermocarboxydus_BPSAC147	Streptomyces_sp._4F	Streptomyces_sp._BSE7F	Streptomyces_sp._ETH9427	Streptomyces_sp._FxanaD5	Streptomyces_sp._UNC401CLCol	Actinospica_acidiphila_NRRL_B_24431	Streptomyces_sp._HNS054	Streptomyces_sp._SMS_SU21	Streptomyces_sp._ZS0098	Streptomyces_thermocarboxydus_K155	Addition	Core-Accessory-Unique	COG	%Similarity_COG	Score_COG	Evalue_COG	KEGG	%Similarity_KEGG	Score_KEGG	Evalue_KEGG
4464	OSC72764.1	-	hypothetical protein	0	1	0	0	0	0	0	0	0	0	0	1	Unique								
4465	WP_048455176.1	-	M23 family metallopeptidase	1	1	1	1	1	1	1	1	1	1	1	11	CORE	COG0739_M#	31.8	105	4.39E-22	NA	32	104	2.61E-21
4466	OSC69060.1	-	ribose-phosphate pyrophosphokinase	0	1	0	0	0	0	0	0	0	0	0	1	Unique	COG0462_FE#	47.3	278	3.78E-74	K00948	47.3	278	1.01E-73
4467	OSC70829.1	-	hypothetical protein	0	1	0	0	0	0	0	0	0	0	0	1	Unique	COG1530_J#	34.4	55	3.05E-07	NA	30.6	70	2.45E-11
4468	WP_122212578.1	-	sensor histidine kinase	0	0	1	1	0	0	0	1	1	1	0	5	Accessory								
4469	WP_048460547.1	-	cysteine synthase	1	1	1	1	1	1	1	1	1	1	1	11	CORE	COG0031_E#	70.5	453	8.55E-127	K12339	70.5	453	2.28E-126
4470	Prokka:JAPCLKMA_02113	-	hypothetical protein	1	1	1	1	1	1	1	1	1	1	1	11	CORE								
4471	Prokka:JAPCLKMA_00195	-	hypothetical protein	1	0	0	1	1	1	1	1	0	1	1	8	Accessory								
4472	WP_122213670.1	-	2-hydroxyacid dehydrogenase	1	1	1	1	1	1	1	1	1	1	1	11	CORE	COG0111_HE#	40.3	141	4.22E-33	NA	39.9	138	9.54E-32
4473	WP_033278151.1	hpnD	presqualene diphosphate synthase HpnD	1	1	1	1	1	1	1	1	1	1	1	11	CORE	COG1562_I#	46.6	230	1.18E-59	K02291	46.6	230	3.15E-59
4474	WP_019523024.1	-	polysaccharide deacetylase	1	0	0	0	1	1	1	0	0	0	1	5	Accessory	COG0726_G#	36.5	182	2.82E-45	NA	31.3	153	2.86E-36

cluster	GI	gene	Annotation	Streptomyces_thermocarboxydus_BPSAC147	Streptomyces_sp._4F	Streptomyces_sp._BSE7F	Streptomyces_sp._ETH9427	Streptomyces_sp._FxanaD5	Streptomyces_sp._UNC401CLCol	Actinospica_acidiphila_NRRL_B_24431	Streptomyces_sp._HNS054	Streptomyces_sp._SMS_SU21	Streptomyces_sp._ZS0098	Streptomyces_thermocarboxydus_K155	Addition	Core-Accessory-Unique	COG	%Similarity_COG	Score_COG	Evalue_COG	KEGG	%Similarity_KEGG	Score_KEGG	Evalue_KEGG
4475	WP_037953460.1	-	NAD(P)-dependent oxidoreductase	0	0	0	0	1	1	0	0	0	0	1	3	Accessory	COG0451_MG#	32.9	135	3.02E-31	NA	32.9	135	8.07E-31
4476	WP_107080774.1	-	MurR/RpiR family transcriptional regulator	1	1	1	1	1	1	1	1	1	1	1	11	CORE	COG1737_K#	29.9	118	3.82E-26	NA	29.9	118	1.02E-25
4477	OSC73282.1	-	3-oxoacyl-ACP synthase	0	1	0	0	0	0	0	0	0	0	0	1	Unique	COG0332_I#	50.8	298	2.7E-80	K00648	42.2	239	5.18E-62
4478	AXI89632.1	-	hypothetical protein	0	0	0	1	0	0	0	0	0	0	0	1	Unique								
4479	PWE09984.1	-	aminoglycoside phosphotransferase family protein	1	1	1	1	1	1	0	1	1	1	1	10	Accessory	COG3173_R#	28.5	51	0.00000574				
4480	WP_033273796.1	-	ABC transporter permease	0	1	0	0	0	0	1	0	0	0	0	2	Accessory	COG0601_EP#	55.4	350	7.81E-96	K02033	34.1	215	8.02E-55
4481	WP_102638264.1	-	hypothetical protein	1	0	0	0	1	1	1	0	1	1	1	7	Accessory								
4482	WP_019525699.1	murQ	N-acetylmuramic acid 6-phosphate etherase	1	1	1	1	1	1	1	1	1	1	1	11	CORE	COG2103_R#	54.2	295	1.75E-79	K07106	53.6	292	3.95E-78
4483	OSC61340.1	-	hypothetical protein	1	1	0	1	1	1	1	1	1	1	1	10	Accessory								

cluster	GI	gene	Annotation	Streptomyces_thermocarboxydus_BPSAC147	Streptomyces_sp._4F	Streptomyces_sp._BSE7F	Streptomyces_sp._ETH9427	Streptomyces_sp._FxanaD5	Streptomyces_sp._UNC401CLCol	Actinospica_acidiphila_NRRL_B_24431	Streptomyces_sp._HNS054	Streptomyces_sp._SMS_SU21	Streptomyces_sp._ZS0098	Streptomyces_thermocarboxydus_K155	Addition	Core-Accessory-Unique	COG	%Similarity_COG	Score_COG	Evalue_COG	KEGG	%Similarity_KEGG	Score_KEGG	Evalue_KEGG
4484	Prokka:JAPCLKMA_02257	menA	1,4-dihydroxy-2-naphthoate octaprenyltransferase	1	0	1	0	1	1	1	1	1	1	1	9	Accessory								
4485	OSC61159.1	-	twin arginine-targeting protein translocase TatC	1	1	1	1	1	1	1	1	1	1	1	11	CORE	COG0805_U#	40.5	195	4.2E-49	K03118	40.5	195	1.12E-48
4486	Prokka:JAPCLKMA_03773	-	hypothetical protein	1	0	0	0	1	0	0	0	0	0	0	2	Accessory								
4487	OSC73372.1	-	EamA family transporter	0	1	0	0	0	0	0	0	0	0	0	1	Unique	COG0697_GER#	42.4	205	4.06E-52	NA	42.4	205	1.08E-51
4488	OSC62576.1	-	metal transporter	0	1	0	0	0	0	0	0	0	0	0	1	Unique	COG0601_EP#	37.3	205	4.06E-52	K15581	35.4	149	4.14E-35
4489	OSC61738.1	-	oxidoreductase	0	1	0	0	0	0	0	0	0	0	0	1	Unique	COG1018_C#	43.1	233	8.16E-61	NA	35.6	185	8.88E-46
4490	WP_028958935.1	-	hypothetical protein	0	0	0	0	0	1	0	0	0	0	0	1	Unique								
4491	OSC63633.1	-	3-oxoacyl-ACP synthase	1	1	1	1	1	1	1	1	1	1	1	11	CORE	COG0332_I#	40.9	232	1.82E-60	K11608	40.9	232	4.85E-60
4492	OSC62153.1	-	hypothetical protein	1	1	1	1	1	1	1	1	1	1	1	11	CORE								
4493	AXI89605.1	-	methyltransferase	0	0	0	1	0	0	0	0	0	0	0	1	Unique	COG0207_F#	32	99	2.4E-20	K00560	32.2	94	2.69E-18
4494	WP_048460153.1	-	sugar ABC transporter permease	1	1	0	0	1	1	1	1	0	1	1	8	Accessory	COG1175_G#	35.5	185	3.33E-46	K10118	33.1	173	4.56E-42

cluster	GI	gene	Annotation	Streptomyces_thermocarboxydus_BPSAC147	Streptomyces_sp._4F	Streptomyces_sp._BSE7F	Streptomyces_sp._ETH9427	Streptomyces_sp._FxanaD5	Streptomyces_sp._UNC401CLCol	Actinospica_acidiphila_NRRL_B_24431	Streptomyces_sp._HNS054	Streptomyces_sp._SMS_SU21	Streptomyces_sp._ZS0098	Streptomyces_thermocarboxydus_K155	Addition	Core-Accessory-Unique	COG	%Similarity_COG	Score_COG	Evalue_COG	KEGG	%Similarity_KEGG	Score_KEGG	Evalue_KEGG
4495	OSC63096.1	-	decaprenyl-phosphate phosphoribosyltransferase	1	1	1	1	1	1	1	1	1	1	1	11	CORE	COG0382_H#	51.9	288	2.79E-77	K14136	51.9	288	7.45E-77
4496	AXI89613.1	-	epimerase	0	0	0	1	0	0	0	0	0	0	0	1	Unique	COG0451_MG#	42.9	228	2.62E-59	NA	40.4	210	1.97E-53
4497	OSC71222.1	-	acetaldehyde dehydrogenase (acetylating)	0	1	0	0	0	0	0	0	0	0	0	1	Unique	COG4569_Q#	64.7	381	2.42E-105	K04073	64.7	381	6.47E-105
4498	Prokka:JAPCLKMA_04905	-	hypothetical protein	1	0	0	0	0	0	0	0	0	0	0	1	Unique								
4499	Prokka:JAPCLKMA_05576	-	hypothetical protein	1	0	0	0	0	0	0	0	0	0	0	1	Unique								
4500	OSC64979.1	-	methionyl-tRNA formyltransferase	0	1	0	0	0	0	0	0	0	0	0	1	Unique	COG0223_J#	40.9	170	1.1E-41	K10011	31.5	148	1.2E-34
4501	WP_033276732.1	-	NADP-dependent oxidoreductase	1	1	0	1	1	1	1	1	1	1	1	10	Accessory	COG0604_CR#	42	218	4.62E-56	K18980	38.8	171	1.73E-41
4502	WP_019525149.1	-	D-2-hydroxyacid dehydrogenase	1	0	0	1	1	1	1	1	1	1	1	9	Accessory	COG0111_HE#	32.8	161	6.7E-39	K00015	33	124	2.42E-27
4503	PWE10254.1	-	xylose isomerase	1	1	1	1	1	1	1	1	1	1	1	11	CORE	COG3623_G#	25.5	59	2.1E-08				
4504	Prokka:CIODPCDJ_01577	-	hypothetical protein	1	1	1	1	1	1	1	1	1	1	1	11	CORE								

cluster	GI	gene	Annotation	Streptomyces_thermocarboxydus_BPSAC147	Streptomyces_sp._4F	Streptomyces_sp._BSE7F	Streptomyces_sp._ETH9427	Streptomyces_sp._FxanaD5	Streptomyces_sp._UNC401CLCol	Actinospica_acidiphila_NRRL_B_24431	Streptomyces_sp._HNS054	Streptomyces_sp._SMS_SU21	Streptomyces_sp._ZS0098	Streptomyces_thermocarboxydus_K155	Addition	Core-Accessory-Unique	COG	%Similarity_COG	Score_COG	Evalue_COG	KEGG	%Similarity_KEGG	Score_KEGG	Evalue_KEGG
4505	WP_028959607.1	pfkB	1-phosphofructokinase	1	0	1	1	1	1	1	1	1	1	1	10	Accessory	COG1105_G#	39.8	191	3.54E-48	K00882	35.8	136	4.72E-31
4506	WP_122215309.1	-	DUF389 domain-containing protein	0	1	1	1	0	0	0	1	1	1	0	6	Accessory					NA	24.8	67	2.69E-10
4507	WP_033277153.1	-	glycosyltransferase family 2 protein	1	0	1	1	1	1	1	1	1	1	1	10	Accessory	COG0463_M#	37.9	174	4.48E-43	NA	30.5	96	4.14E-19
4508	Prokka:CIODPCDJ_05241	-	hypothetical protein	0	0	0	0	1	1	0	0	0	0	1	3	Accessory								
4509	Prokka:JAPCLKMA_04230	sigJ_3	ECF RNA polymerase sigma factor SigJ	1	1	1	1	1	1	1	1	1	1	1	11	CORE	COG1595_K#	37.5	184	5.66E-46	K03088	40.3	180	2.85E-44
4510	AXI87072.1	-	DUF2520 domain-containing protein	1	0	1	1	1	1	1	1	1	1	1	10	Accessory	COG5495_S#	50.3	257	6.87E-68	NA	50.3	257	1.83E-67
4511	PWE12308.1	-	hypothetical protein	0	0	1	0	0	0	0	0	0	0	0	1	Unique								
4512	WP_102638042.1	-	hypothetical protein	1	0	1	1	0	1	0	1	1	1	1	8	Accessory					NA	30	54	0.00000236
4513	WP_122216632.1	pdhA	pyruvate dehydrogenase (acetyl-transferring) E1 component subunit alpha	0	0	0	0	0	0	0	0	1	1	0	2	Accessory	COG1071_C#	47	291	4.29E-78	K00161	43.7	267	1.36E-70
4514	WP_026244130.1	-	hypothetical protein	0	0	0	0	1	1	0	0	0	0	1	3	Accessory								

cluster	GI	gene	Annotation	Streptomyces_thermocarboxydus_BPSAC147	Streptomyces_sp._4F	Streptomyces_sp._BSE7F	Streptomyces_sp._ETH9427	Streptomyces_sp._FxanaD5	Streptomyces_sp._UNC401CLCol	Actinospica_acidiphila_NRRL_B_24431	Streptomyces_sp._HNS054	Streptomyces_sp._SMS_SU21	Streptomyces_sp._ZS0098	Streptomyces_thermocarboxydus_K155	Addition	Core-Accessory-Unique	COG	%Similarity_COG	Score_COG	Evalue_COG	KEGG	%Similarity_KEGG	Score_KEGG	Evalue_KEGG
4515	OSC61098.1	-	MBL fold metallo-hydrolase	1	1	1	0	1	1	1	1	1	1	1	10	Accessory	COG0491_R#	28.6	112	2.73E-24	NA	28.2	95	1.21E-18
4516	OSC71338.1	-	transporter	1	1	1	0	1	1	1	0	1	1	1	9	Accessory	COG1175_G#	39	184	5.66E-46	K17245	39	184	1.51E-45
4517	WP_122213474.1	-	LysR family transcriptional regulator	0	0	0	0	0	0	1	1	0	1	0	3	Accessory	COG0583_K#	33.1	139	2.73E-32	NA	31.2	136	4.72E-31
4518	PWE11525.1	-	short-chain dehydrogenase	1	1	1	1	1	1	1	1	1	1	1	11	CORE	COG1028_IQR#	73.2	443	5.17E-124	K11163	33.8	144	2.26E-33
4519	OSC70851.1	-	superoxide dismutase	0	1	0	0	0	0	0	0	0	0	0	1	Unique								
4520	AXI88192.1	dprA	DNA-protecting protein DprA	0	0	0	1	0	0	0	0	0	0	0	1	Unique	COG0758_LU#	42.5	156	1.26E-37	K04096	38.5	138	9.48E-32
4521	Prokka:CIODPCDJ_06297	-	hypothetical protein	1	0	1	1	1	1	1	1	1	1	1	10	Accessory	COG1686_M#	30.9	88	4.21E-17	K07258	30	85	1.24E-15
4522	WP_019521508.1	-	ABC transporter substrate-binding protein	1	1	0	0	1	1	1	0	0	0	1	6	Accessory	COG0834_ET#	34.3	124	5.3E-28	K02030	26.6	89	5.05E-17
4523	Prokka:CIODPCDJ_04295	-	hypothetical protein	1	1	1	1	1	1	1	1	1	1	1	11	CORE	COG4965_U#	27.4	95	4.5E-19				
4524	Prokka:JAPCLKMA_05337	-	hypothetical protein	1	1	1	1	1	1	1	1	1	1	1	11	CORE	COG2887_L#	53.3	278	3.75E-74	K07465	53.3	278	1E-73
4525	Prokka:CIODPCDJ_02152	-	2-haloacrylate reductase	1	0	1	1	1	1	1	1	1	1	1	10	Accessory	COG0604_CR#	36.9	156	2.15E-37	K00344	31.5	119	4.55E-26

cluster	GI	gene	Annotation	Streptomyces_thermocarboxydus_BPSAC147	Streptomyces_sp._4F	Streptomyces_sp._BSE7F	Streptomyces_sp._ETH9427	Streptomyces_sp._FxanaD5	Streptomyces_sp._UNC401CLCol	Actinospica_acidiphila_NRRL_B_24431	Streptomyces_sp._HNS054	Streptomyces_sp._SMS_SU21	Streptomyces_sp._ZS0098	Streptomyces_thermocarboxydus_K155	Addition	Core-Accessory-Unique	COG	%Similarity_COG	Score_COG	Evalue_COG	KEGG	%Similarity_KEGG	Score_KEGG	Evalue_KEGG
4526	WP_109288764.1	-	ComF family protein	1	0	1	0	1	1	1	1	1	1	1	9	Accessory	COG1040_R#	37.4	108	3.93E-23				
4527	WP_019525548.1	-	hypothetical protein	1	0	0	0	1	1	1	0	0	0	1	5	Accessory	COG1226_P#	30.8	142	3.21E-33	K16891	25.6	95	1.2E-18
4528	WP_093547247.1	-	hypothetical protein	0	1	0	0	0	0	1	0	0	0	0	2	Accessory								
4529	PWE07096.1	-	RluA family pseudouridine synthase	1	1	1	1	1	1	1	1	1	1	1	11	CORE	COG0564_J#	64.1	407	4.1E-113	K06180	66.4	405	4.16E-112
4530	WP_019523047.1	-	M56 family metallopeptidase	1	0	1	1	1	1	1	1	1	1	1	10	Accessory	COG0501_O#	31.3	80	8.78E-15	NA	31.3	80	2.34E-14
4531	OSC68709.1	-	alcohol dehydrogenase	0	1	0	0	0	0	0	0	0	0	0	1	Unique	COG0667_C#	54.7	315	2.77E-85	NA	38.2	179	3.71E-44
4532	WP_079031440.1	-	SGNH/GDSL hydrolase family protein	1	1	1	1	1	1	1	1	1	1	1	11	CORE								
4533	WP_033274072.1	-	SDR family oxidoreductase	0	1	0	0	0	0	1	0	0	1	0	3	Accessory	COG1028_IQR#	54.5	312	1.37E-84	NA	54.5	312	3.67E-84
4534	OSC55689.1	-	LysR family transcriptional regulator	0	1	0	0	0	0	0	0	0	0	0	1	Unique	COG0583_K#	30.2	99	1.83E-20	K04761	30.2	92	5.96E-18
4535	OSC55795.1	-	hydroxyurea phosphotransferase	0	1	0	0	0	0	0	0	0	0	0	1	Unique	COG3570_V#	30	94	5.88E-19				
4536	Prokka:JAPCLKMA_04896	-	hypothetical protein	1	0	0	0	0	0	0	0	0	0	0	1	Unique					NA	47.1	43	0.00415

cluster	GI	gene	Annotation	Streptomyces_thermocarboxydus_BPSAC147	Streptomyces_sp._4F	Streptomyces_sp._BSE7F	Streptomyces_sp._ETH9427	Streptomyces_sp._FxanaD5	Streptomyces_sp._UNC401CLCol	Actinospica_acidiphila_NRRL_B_24431	Streptomyces_sp._HNS054	Streptomyces_sp._SMS_SU21	Streptomyces_sp._ZS0098	Streptomyces_thermocarboxydus_K155	Addition	Core-Accessory-Unique	COG	%Similarity_COG	Score_COG	Evalue_COG	KEGG	%Similarity_KEGG	Score_KEGG	Evalue_KEGG
4537	WP_019526066.1	-	hypothetical protein	0	0	0	0	1	0	0	0	0	0	0	1	Unique								
4538	AXI87125.1	-	dehydrogenase	0	0	1	1	0	0	0	0	1	0	0	3	Accessory	COG1028_IQR#	40.2	164	4.62E-40	NA	36.2	149	5.37E-35
4539	WP_102638079.1	-	sucrase ferredoxin	1	1	1	1	1	1	1	1	1	1	1	11	CORE	COG4759_O#	34.2	156	2.14E-37				
4540	PWE09937.1	-	sugar ABC transporter permease	1	0	1	1	1	1	1	1	1	1	1	10	Accessory	COG1175_G#	43.2	233	1.06E-60	K02025	34.4	166	4.23E-40
4541	OSC63330.1	-	sugar ABC transporter permease	0	1	0	0	0	0	0	0	0	0	0	1	Unique	COG1175_G#	37.6	196	1.09E-49	K02025	32.6	159	6.75E-38
4542	WP_019526197.1	-	hypothetical protein	0	0	1	1	1	1	1	1	1	1	1	9	Accessory								
4543	Prokka:CIODPCDJ_01506	melD_2	Melibiose/raffinose/stachyose import permease protein MelD	1	1	1	1	1	1	1	1	1	1	1	11	CORE	COG1175_G#	36	221	3.18E-57	K02025	33.3	137	2.1E-31
4544	AXI88754.1	-	sugar ABC transporter permease	1	0	1	1	1	1	1	1	1	1	1	10	Accessory	COG1175_G#	43.6	252	2.19E-66	K17245	34.4	177	2.4E-43
4545	WP_052183253.1	-	helix-turn-helix domain-containing protein	0	0	0	0	1	1	0	0	0	0	1	3	Accessory	COG4977_K#	44.8	243	1.33E-63	NA	38.3	69	7.04E-11
4546	WP_048458496.1	-	cobalamin biosynthesis protein	1	0	1	1	1	1	1	1	1	1	1	10	Accessory	COG1270_H#	55	306	9.81E-83	K02227	55	306	2.62E-82
4547	AXI88210.1	-	hypothetical protein	1	0	1	1	1	1	1	1	1	1	1	10	Accessory								

cluster	GI	gene	Annotation	Streptomyces_thermocarboxydus_BPSAC147	Streptomyces_sp._4F	Streptomyces_sp._BSE7F	Streptomyces_sp._ETH9427	Streptomyces_sp._FxanaD5	Streptomyces_sp._UNC401CLCol	Actinospica_acidiphila_NRRL_B_24431	Streptomyces_sp._HNS054	Streptomyces_sp._SMS_SU21	Streptomyces_sp._ZS0098	Streptomyces_thermocarboxydus_K155	Addition	Core-Accessory-Unique	COG	%Similarity_COG	Score_COG	Evalue_COG	KEGG	%Similarity_KEGG	Score_KEGG	Evalue_KEGG
4548	Prokka:JAPCLKMA_01425	ykfA	putative murein peptide carboxypeptidase	1	1	1	1	1	1	1	1	1	1	1	11	CORE	COG1619_V#	37.4	181	4.76E-45	K01297	37.4	181	1.27E-44
4549	Prokka:CIODPCDJ_06311	-	hypothetical protein	0	0	0	0	0	0	0	0	0	0	1	1	Unique								
4550	Prokka:JAPCLKMA_03323	-	Universal stress protein/MSMEI_3859	1	0	1	1	1	1	1	1	1	1	1	10	Accessory	COG0589_T#	31.9	146	1.3E-34	NA	31.4	138	1.61E-31
4551	Prokka:JAPCLKMA_03811	-	hypothetical protein	1	0	0	0	0	0	0	0	0	0	0	1	Unique								
4552	OSC61294.1	-	hypothetical protein	0	1	0	0	0	0	0	0	0	0	0	1	Unique								
4553	WP_026244259.1	-	biotin--[acetyl-CoA-carboxylase] ligase	0	1	1	1	1	1	1	1	1	1	1	10	Accessory	COG0340_H#	42.3	193	1.21E-48	K03524	42.8	184	1.5E-45
4554	OSC70337.1	-	acyl-ACP desaturase	0	1	0	0	0	0	0	0	0	0	0	1	Unique					K03921	45.8	282	5.29E-75
4555	Prokka:CIODPCDJ_04680	-	hypothetical protein	1	0	1	1	1	1	1	1	1	1	1	10	Accessory	COG3001_G#	27.7	72	4.07E-12				
4556	Prokka:CIODPCDJ_02728	ksgA	Ribosomal RNA small subunit methyltransferase A	1	0	1	1	1	1	1	1	1	1	1	10	Accessory	COG0030_J#	58.7	315	2.76E-85	K02528	51.5	270	1.59E-71
4557	OSC63967.1	-	pseudouridine-5-phosphate glycosidase	1	1	1	1	1	1	1	1	1	1	1	11	CORE	COG2313_Q#	49.1	260	8.06E-69	K16329	47.3	258	1.07E-67

cluster	GI	gene	Annotation	Streptomyces_thermocarboxydus_BPSAC147	Streptomyces_sp._4F	Streptomyces_sp._BSE7F	Streptomyces_sp._ETH9427	Streptomyces_sp._FxanaD5	Streptomyces_sp._UNC401CLCol	Actinospica_acidiphila_NRRL_B_24431	Streptomyces_sp._HNS054	Streptomyces_sp._SMS_SU21	Streptomyces_sp._ZS0098	Streptomyces_thermocarboxydus_K155	Addition	Core-Accessory-Unique	COG	%Similarity_COG	Score_COG	Evalue_COG	KEGG	%Similarity_KEGG	Score_KEGG	Evalue_KEGG
4558	WP_033274423.1	-	ADP-ribosylglycohydrolase family protein	0	0	1	0	0	0	1	0	0	0	0	2	Accessory	COG1397_O#	30.9	115	3.21E-25	NA	26.9	107	3.05E-22
4559	PWE11359.1	-	tRNA (adenosine(37)-N6)-dimethylallyltransferase MiaA	1	1	1	1	1	1	1	1	1	1	1	11	CORE	COG0324_J#	56	310	5.2E-84	K00791	56	310	1.39E-83
4560	WP_019526279.1	-	hypothetical protein	1	1	1	1	1	1	1	1	1	1	1	11	CORE								
4561	OSC62420.1	-	23S rRNA (guanosine(2251)-2'-O)-methyltransferase RlmB	1	1	1	1	1	1	1	1	1	1	1	11	CORE	COG0566_J#	56.9	327	5.37E-89	K03218	56.9	327	1.43E-88
4562	WP_102638085.1	-	transcriptional regulator	1	1	1	0	1	1	0	1	1	1	1	9	Accessory								
4563	OSC70070.1	-	glycosyl transferase	1	1	1	1	1	1	1	1	1	1	1	11	CORE	COG1216_R#	33.3	45	0.000312				
4564	OSC56375.1	-	hypothetical protein	0	1	0	0	0	0	0	0	0	0	0	1	Unique								
4565	AXI85344.1	-	hypothetical protein	1	0	0	1	0	0	0	0	0	0	0	2	Accessory								
4566	OSC63117.1	-	ABC transporter permease	0	1	0	0	0	0	0	0	0	0	0	1	Unique	COG0601_EP#	35.9	212	2.52E-54	K13890	34.9	193	3.23E-48
4567	Prokka:CIODPCDJ_03455	aidA	Quorum-quenching protein AidA	0	0	0	0	0	0	1	0	0	0	1	2	Accessory	COG1073_R#	40.9	186	1.48E-46	K06889	26.1	62	1.12E-08

cluster	GI	gene	Annotation	Streptomyces_thermocarboxydus_BPSAC147	Streptomyces_sp._4F	Streptomyces_sp._BSE7F	Streptomyces_sp._ETH9427	Streptomyces_sp._FxanaD5	Streptomyces_sp._UNC401CLCol	Actinospica_acidiphila_NRRL_B_24431	Streptomyces_sp._HNS054	Streptomyces_sp._SMS_SU21	Streptomyces_sp._ZS0098	Streptomyces_thermocarboxydus_K155	Addition	Core-Accessory-Unique	COG	%Similarity_COG	Score_COG	Evalue_COG	KEGG	%Similarity_KEGG	Score_KEGG	Evalue_KEGG
4568	PWE07030.1	-	PAC2 family protein	1	0	1	1	1	1	1	1	1	1	1	10	Accessory					NA	40.6	226	3.43E-58
4569	OSC71864.1	-	oxidoreductase	0	1	0	0	0	0	0	0	0	0	0	1	Unique	COG0346_E#	26.1	80	1.14E-14				
4570	WP_122214047.1	-	AraC family transcriptional regulator	0	1	1	1	0	0	1	1	1	1	1	8	Accessory	COG2207_K#	30.3	145	3.77E-34	K18991	24.7	71	1.85E-11
4571	Prokka:CIODPCDJ_04624	smc_5	Chromosome partition protein Smc	1	1	1	1	1	1	1	1	1	1	1	11	CORE	COG0739_M#	30.6	69	2.63E-11	NA	28.9	85	1.24E-15
4572	WP_122213368.1	-	sugar ABC transporter permease	0	0	0	0	0	0	0	0	0	1	0	1	Unique	COG1175_G#	34.5	199	2.2E-50	K17245	38.3	194	1.45E-48
4573	WP_019524331.1	-	hypothetical protein	0	0	0	0	1	0	0	0	0	0	0	1	Unique								
4574	WP_019524496.1	-	M56 family metallopeptidase	1	1	1	1	1	1	1	1	1	1	1	11	CORE	COG0501_O#	34.2	126	1.81E-28	NA	34.2	126	4.84E-28
4575	OSC62345.1	-	taurine dioxygenase	0	1	0	0	0	0	0	0	0	0	0	1	Unique	COG2175_Q#	45.6	231	3.06E-60	K03119	44.2	231	8.17E-60
4576	WP_048455653.1	-	diiron oxygenase	1	1	1	1	1	1	1	1	1	1	1	11	CORE					NA	24.8	89	5.02E-17
4577	OSC61572.1	-	N-acetylneuraminate synthase	1	1	1	1	1	1	1	1	1	1	1	11	CORE	COG2089_M#	36.2	187	6.62E-47	K05304	40.7	205	6.27E-52
4578	WP_102638672.1	cysD	sulfate adenylyltransferase subunit CysD	1	1	1	1	1	1	1	1	1	1	1	11	CORE	COG0175_EH#	70.5	453	6.47E-127	K00957	66.1	402	4.57E-111

cluster	GI	gene	Annotation	Streptomyces_thermocarboxydus_BPSAC147	Streptomyces_sp._4F	Streptomyces_sp._BSE7F	Streptomyces_sp._ETH9427	Streptomyces_sp._FxanaD5	Streptomyces_sp._UNC401CLCol	Actinospica_acidiphila_NRRL_B_24431	Streptomyces_sp._HNS054	Streptomyces_sp._SMS_SU21	Streptomyces_sp._ZS0098	Streptomyces_thermocarboxydus_K155	Addition	Core-Accessory-Unique	COG	%Similarity_COG	Score_COG	Evalue_COG	KEGG	%Similarity_KEGG	Score_KEGG	Evalue_KEGG
4579	WP_048460714.1	-	membrane protein	1	0	1	1	1	1	1	1	1	1	1	10	Accessory	COG0342_U#	39.5	49	0.0000215	NA	40	72	8.29E-12
4580	WP_033277630.1	-	cation transporter	1	1	1	1	1	1	1	1	1	1	1	11	CORE	COG1230_P#	44.6	247	9.19E-65	K16264	42.8	230	1.82E-59
4581	AXI91306.1	-	hypothetical protein	0	0	0	1	0	0	0	0	0	0	0	1	Unique								
4582	WP_019522047.1	-	TIGR01777 family protein	1	1	1	0	1	1	1	1	1	1	1	10	Accessory	COG1090_R#	46.7	270	1.01E-71	K07071	46.7	270	2.71E-71
4583	OSC61600.1	-	multidrug ABC transporter ATP-binding protein	1	1	1	1	1	1	1	0	1	1	1	10	Accessory	COG1131_V#	39	210	9.53E-54	K01990	40.4	202	6.93E-51
4584	Prokka:CIODPCDJ_03391	-	hypothetical protein	1	1	1	1	1	1	1	1	1	1	1	11	CORE								
4585	OSC55516.1	-	MerR family transcriptional regulator	0	1	0	0	0	0	0	0	0	0	0	1	Unique	COG0789_K#	38.2	65	4.95E-10	NA	28.5	58	1.24E-07
4586	WP_019524231.1	-	SRPBCC family protein	1	0	0	0	1	1	0	0	0	0	0	3	Accessory	COG5637_S#	44	158	5.62E-38	NA	39.1	151	1.83E-35
4587	OSC61196.1	-	short-chain dehydrogenase	1	1	1	1	1	1	1	1	1	1	1	11	CORE	COG0300_R#	33.3	132	2.53E-30	NA	36.8	120	2.65E-26
4588	OSC61821.1	-	LysR family transcriptional regulator	0	1	0	0	0	0	0	0	0	0	0	1	Unique	COG0583_K#	37.5	192	2.69E-48	NA	26.6	88	1.12E-16

cluster	GI	gene	Annotation	Streptomyces_thermocarboxydus_BPSAC147	Streptomyces_sp._4F	Streptomyces_sp._BSE7F	Streptomyces_sp._ETH9427	Streptomyces_sp._FxanaD5	Streptomyces_sp._UNC401CLCol	Actinospica_acidiphila_NRRL_B_24431	Streptomyces_sp._HNS054	Streptomyces_sp._SMS_SU21	Streptomyces_sp._ZS0098	Streptomyces_thermocarboxydus_K155	Addition	Core-Accessory-Unique	COG	%Similarity_COG	Score_COG	Evalue_COG	KEGG	%Similarity_KEGG	Score_KEGG	Evalue_KEGG
4589	WP_122214064.1	-	NUDIX domain-containing protein	1	0	0	0	1	1	1	0	0	1	1	6	Accessory	COG1051_F#	32.8	58	6.06E-08				
4590	WP_019525743.1	-	carbohydrate ABC transporter permease	1	1	0	0	1	1	1	0	0	0	1	6	Accessory	COG0395_G#	38.6	207	8.05E-53	K17246	33.1	172	7.66E-42
4591	AXI87680.1	-	MurR/RpiR family transcriptional regulator	1	1	1	1	1	1	1	1	1	1	1	11	CORE	COG1737_K#	31.9	153	1.8E-36	K19337	33.1	149	6.94E-35
4592	WP_019521597.1	-	LysR family transcriptional regulator	1	1	1	1	1	1	1	1	1	1	1	11	CORE	COG0583_K#	48.5	255	3.36E-67	NA	48.5	255	8.99E-67
4593	WP_048459907.1	-	1-phosphofructokinase family hexose kinase	1	1	1	1	1	1	1	1	1	1	1	11	CORE	COG1105_G#	38.3	161	6.61E-39	K00882	29.4	141	1.11E-32
4594	PWE10848.1	-	hypothetical protein	0	0	1	0	0	0	0	0	1	0	1	3	Accessory					NA	29	74	1.66E-12
4595	WP_048455890.1	-	serine protease	1	0	1	1	1	1	0	1	1	1	1	9	Accessory								
4596	WP_048455853.1	-	SPFH domain-containing protein	1	1	1	1	1	1	1	1	1	1	1	11	CORE	COG0330_O#	49.3	283	1.15E-75	NA	40.9	222	4.94E-57
4597	WP_028960946.1	-	ABC transporter permease	1	1	1	1	1	1	0	1	1	1	1	10	Accessory	COG1682_GM#	24.6	101	4.76E-21	K01992	23.1	64	2.94E-09

cluster	GI	gene	Annotation	Streptomyces_thermocarboxydus_BPSAC147	Streptomyces_sp._4F	Streptomyces_sp._BSE7F	Streptomyces_sp._ETH9427	Streptomyces_sp._FxanaD5	Streptomyces_sp._UNC401CLCol	Actinospica_acidiphila_NRRL_B_24431	Streptomyces_sp._HNS054	Streptomyces_sp._SMS_SU21	Streptomyces_sp._ZS0098	Streptomyces_thermocarboxydus_K155	Addition	Core-Accessory-Unique	COG	%Similarity_COG	Score_COG	Evalue_COG	KEGG	%Similarity_KEGG	Score_KEGG	Evalue_KEGG
4598	WP_122215986.1	-	A/G-specific adenine glycosylase	1	0	1	1	1	1	1	1	1	1	1	10	Accessory	COG1194_L#	50.7	275	1.84E-73	K03575	50.7	275	4.92E-73
4599	WP_048456191.1	-	hypothetical protein	0	0	0	0	0	0	0	1	1	0	1	3	Accessory								
4600	WP_122216449.1	-	LysR family transcriptional regulator	0	1	1	1	0	0	1	1	1	1	0	7	Accessory	COG0583_K#	35.5	121	7.58E-27	NA	28.8	106	6.74E-22
4601	WP_048459905.1	-	sugar ABC transporter permease	1	0	0	1	1	1	1	1	0	0	1	7	Accessory	COG1175_G#	36	210	9.5E-54	K02025	33	152	6.28E-36
4602	WP_147474339.1	-	hypothetical protein	0	0	0	0	0	0	0	0	0	1	0	1	Unique								
4603	WP_019521482.1	-	3-carboxyethylcatechol 2,3-dioxygenase	0	0	0	0	1	1	1	1	0	1	1	6	Accessory					K05713	37.9	235	9.62E-61
4604	OSC63761.1	-	transporter	1	1	1	1	1	1	1	1	1	1	1	11	CORE	COG1175_G#	40.8	206	1.05E-52	K10237	32.7	185	6.69E-46
4605	OSC71390.1	-	carbon-nitrogen hydrolase family protein	0	1	0	0	0	0	0	0	0	0	0	1	Unique	COG0388_R#	30.2	93	1.69E-18	K12251	29.4	105	8.8E-22
4606	AXI89650.1	-	hypothetical protein	0	0	0	1	0	0	0	0	0	0	0	1	Unique	COG3586_S#	39.7	131	7.33E-30				
4607	WP_106432421.1	-	hypothetical protein	1	0	0	1	1	1	1	1	0	1	1	8	Accessory	COG4542_Q#	30.4	101	8.12E-21	K16651	23.3	49	0.0000573
4608	Prokka:CIODPCDJ_06748	-	hypothetical protein	0	0	0	0	0	0	0	0	0	0	1	1	Unique								

cluster	GI	gene	Annotation	Streptomyces_thermocarboxydus_BPSAC147	Streptomyces_sp._4F	Streptomyces_sp._BSE7F	Streptomyces_sp._ETH9427	Streptomyces_sp._FxanaD5	Streptomyces_sp._UNC401CLCol	Actinospica_acidiphila_NRRL_B_24431	Streptomyces_sp._HNS054	Streptomyces_sp._SMS_SU21	Streptomyces_sp._ZS0098	Streptomyces_thermocarboxydus_K155	Addition	Core-Accessory-Unique	COG	%Similarity_COG	Score_COG	Evalue_COG	KEGG	%Similarity_KEGG	Score_KEGG	Evalue_KEGG
4609	OSC65036.1	-	hypothetical protein	0	1	0	0	0	0	0	0	0	0	0	1	Unique								
4610	WP_102637949.1	-	AAA domain-containing protein	0	0	0	0	0	0	0	0	1	0	0	1	Unique	COG0714_R#	36.2	125	4.02E-28				
4611	WP_033275362.1	-	NAD-dependent epimerase/dehydratase family protein	0	0	0	0	0	0	1	0	0	0	0	1	Unique	COG0451_MG#	34	178	3.06E-44	NA	25.4	70	3.14E-11
4612	WP_102637531.1	-	transposase	1	0	0	1	1	1	0	1	1	0	0	6	Accessory								
4613	OSC60795.1	-	rhomboid family intramembrane serine protease	1	1	1	1	1	1	1	1	1	1	1	11	CORE	COG0705_R#	39.9	125	4.02E-28	NA	39.9	125	1.07E-27
4614	OSC62243.1	-	beta-galactosidase	0	1	0	0	0	0	0	0	0	0	0	1	Unique	COG1874_G#	44.4	258	3.04E-68	K12309	44.1	256	4.03E-67
4615	OSC69601.1	-	urate oxidase	1	1	1	1	1	1	1	1	1	1	1	11	CORE	COG3648_Q#	38.3	186	1.92E-46	K00365	38.3	186	5.13E-46
4616	OSC66753.1	-	hypothetical protein	0	1	0	0	0	0	0	0	0	0	0	1	Unique								
4617	OSC71952.1	-	EamA family transporter	0	1	0	0	0	0	0	0	0	0	0	1	Unique	COG0697_GER#	32.6	109	2.97E-23	NA	25.9	53	0.00000396
4618	WP_033273943.1	-	ADP-ribosylglycohydrolase family protein	0	0	0	0	0	0	1	0	0	0	0	1	Unique	COG1397_O#	30	84	1.02E-15				

cluster	GI	gene	Annotation	Streptomyces_thermocarboxydus_BPSAC147	Streptomyces_sp._4F	Streptomyces_sp._BSE7F	Streptomyces_sp._ETH9427	Streptomyces_sp._FxanaD5	Streptomyces_sp._UNC401CLCol	Actinospica_acidiphila_NRRL_B_24431	Streptomyces_sp._HNS054	Streptomyces_sp._SMS_SU21	Streptomyces_sp._ZS0098	Streptomyces_thermocarboxydus_K155	Addition	Core-Accessory-Unique	COG	%Similarity_COG	Score_COG	Evalue_COG	KEGG	%Similarity_KEGG	Score_KEGG	Evalue_KEGG
4619	AXI86213.1	-	branched-chain amino acid ABC transporter permease	1	1	1	1	1	1	1	1	1	1	1	11	CORE	COG0559_E#	51.7	292	1.9E-78	K01997	41.8	234	1.25E-60
4620	WP_019525975.1	pheA	prephenate dehydratase	1	1	1	1	1	1	1	1	1	1	1	11	CORE	COG0077_E#	47.8	260	6.11E-69	K04518	47.8	260	1.63E-68
4621	WP_026243914.1	-	conjugal transfer protein	0	1	0	0	1	0	0	0	0	0	1	3	Accessory								
4622	Prokka:CIODPCDJ_01325	mshD_3	Mycothiol acetyltransferase	1	1	1	1	1	1	1	1	1	1	1	11	CORE	COG0454_KR#	34.6	117	6.4E-26	NA	34.6	117	1.71E-25
4623	AXI85723.1	-	methionyl-tRNA formyltransferase	1	1	1	1	1	1	1	1	1	1	1	11	CORE	COG0223_J#	55.7	329	1.4E-89	K00604	55.7	329	3.74E-89
4624	WP_028959498.1	-	hypothetical protein	0	1	0	0	0	1	0	0	0	0	0	2	Accessory								
4625	WP_122216009.1	-	Ppx/GppA family phosphatase	1	1	1	1	1	1	1	1	1	1	1	11	CORE	COG0248_FP#	57	333	9.69E-91	K01524	54.3	301	8.34E-81
4626	Prokka:JAPCLKMA_01291	-	hypothetical protein	1	0	1	1	0	1	1	1	1	1	1	9	Accessory	COG0705_R#	41.7	180	8.04E-45	NA	45.1	146	3.44E-34
4627	AXI87708.1	-	hypothetical protein	0	0	1	1	0	0	0	1	0	0	0	3	Accessory								
4628	OSC55367.1	-	hypothetical protein	0	1	0	0	0	0	0	0	0	0	0	1	Unique								
4629	PWE11450.1	-	haloacid dehalogenase-like hydrolase	0	0	1	0	0	0	0	0	1	1	0	3	Accessory								

cluster	GI	gene	Annotation	Streptomyces_thermocarboxydus_BPSAC147	Streptomyces_sp._4F	Streptomyces_sp._BSE7F	Streptomyces_sp._ETH9427	Streptomyces_sp._FxanaD5	Streptomyces_sp._UNC401CLCol	Actinospica_acidiphila_NRRL_B_24431	Streptomyces_sp._HNS054	Streptomyces_sp._SMS_SU21	Streptomyces_sp._ZS0098	Streptomyces_thermocarboxydus_K155	Addition	Core-Accessory-Unique	COG	%Similarity_COG	Score_COG	Evalue_COG	KEGG	%Similarity_KEGG	Score_KEGG	Evalue_KEGG
4630	WP_109288717.1	-	hypothetical protein	0	0	1	1	0	0	0	1	1	1	0	5	Accessory					K06236	37.3	63	3.82E-09
4631	Prokka:JAPCLKMA_02724	-	hypothetical protein	1	0	1	0	0	0	0	0	0	0	0	2	Accessory								
4632	Prokka:CIODPCDJ_03853	-	hypothetical protein	1	1	1	1	1	1	1	1	1	1	1	11	CORE								
4633	Prokka:JAPCLKMA_01881	-	hypothetical protein	1	0	0	0	1	1	0	0	0	0	1	4	Accessory								
4634	Prokka:JAPCLKMA_01879	-	hypothetical protein	1	0	0	0	1	1	0	0	0	0	1	4	Accessory					NA	26.1	87	3.23E-16
4635	OSC58755.1	-	NmrA family protein	0	1	0	0	0	0	0	0	0	0	0	1	Unique	COG0702_MG#	36.5	149	1.98E-35	K19267	30	80	3.02E-14
4636	WP_019523782.1	-	phosphatidylinositol mannoside acyltransferase	1	1	1	1	1	1	1	1	1	1	1	11	CORE	COG1560_M#	47.4	249	1.83E-65	K02517	47.9	248	1.09E-64
4637	WP_048459052.1	-	NAD(P)H-binding protein	0	0	0	0	0	0	1	1	0	1	0	3	Accessory	COG0702_MG#	33.2	173	9.79E-43	NA	43.8	44	0.00313
4638	OSC68612.1	-	oxidoreductase	1	1	1	1	1	1	1	1	1	1	1	11	CORE								
4639	WP_019526609.1	-	polysaccharide pyruvyl transferase family protein	1	1	1	1	1	1	1	1	1	1	1	11	CORE								
4640	AXI89903.1	-	ABC transporter permease	1	0	1	1	1	1	1	1	1	1	1	10	Accessory	COG1175_G#	68.4	397	5.45E-110	K02025	68.4	397	1.45E-109

cluster	GI	gene	Annotation	Streptomyces_thermocarboxydus_BPSAC147	Streptomyces_sp._4F	Streptomyces_sp._BSE7F	Streptomyces_sp._ETH9427	Streptomyces_sp._FxanaD5	Streptomyces_sp._UNC401CLCol	Actinospica_acidiphila_NRRL_B_24431	Streptomyces_sp._HNS054	Streptomyces_sp._SMS_SU21	Streptomyces_sp._ZS0098	Streptomyces_thermocarboxydus_K155	Addition	Core-Accessory-Unique	COG	%Similarity_COG	Score_COG	Evalue_COG	KEGG	%Similarity_KEGG	Score_KEGG	Evalue_KEGG
4641	Prokka:CIODPCDJ_05414	-	hypothetical protein	0	0	0	0	0	0	0	0	0	0	1	1	Unique								
4642	WP_019523309.1	-	ABC transporter permease	1	1	1	1	1	1	1	1	1	1	1	11	CORE	COG1176_E#	39.3	187	6.55E-47	K11071	39.3	187	1.75E-46
4643	WP_122216246.1	-	NADH-quinone oxidoreductase subunit H	0	0	0	1	0	0	0	1	1	1	0	4	Accessory	COG1005_C#	29.9	119	2.86E-26	K00337	26.6	110	2.72E-23
4644	OSC63336.1	-	arabinan endo-1,5-alpha-L-arabinosidase	0	1	0	0	0	0	0	0	0	0	0	1	Unique	COG3507_G#	40.7	225	2.17E-58	K06113	42	244	1.21E-63
4645	OSC66169.1	-	ectoine/hydroxyectoine ABC transporter substrate-binding protein EhuB	1	1	1	1	1	1	1	1	1	1	1	11	CORE	COG0834_ET#	36.6	200	7.48E-51	K10013	26	65	1.31E-09
4646	OSC62355.1	-	GNAT family N-acetyltransferase	1	1	1	1	0	0	1	1	1	1	1	9	Accessory	COG0454_KR#	30.8	52	0.00000329				
4647	OSC69263.1	-	ATP-dependent DNA ligase	1	1	0	1	1	1	1	1	1	1	1	10	Accessory	COG3285_L#	35.7	170	1.08E-41	NA	35.4	159	6.67E-38
4648	WP_033275363.1	-	LysR family transcriptional regulator	1	0	0	0	1	1	1	0	0	0	1	5	Accessory	COG0583_K#	30	120	9.84E-27	K11921	33.2	113	4.2E-24

cluster	GI	gene	Annotation	Streptomyces_thermocarboxydus_BPSAC147	Streptomyces_sp._4F	Streptomyces_sp._BSE7F	Streptomyces_sp._ETH9427	Streptomyces_sp._FxanaD5	Streptomyces_sp._UNC401CLCol	Actinospica_acidiphila_NRRL_B_24431	Streptomyces_sp._HNS054	Streptomyces_sp._SMS_SU21	Streptomyces_sp._ZS0098	Streptomyces_thermocarboxydus_K155	Addition	Core-Accessory-Unique	COG	%Similarity_COG	Score_COG	Evalue_COG	KEGG	%Similarity_KEGG	Score_KEGG	Evalue_KEGG
4649	OSC64277.1	-	hypothetical protein	0	1	0	0	0	0	0	0	0	0	0	1	Unique					NA	29.7	152	8.15E-36
4650	OSC73127.1	-	mycothiol synthase	1	1	1	1	1	1	1	1	1	1	1	11	CORE	COG0456_R#	45.9	221	3.14E-57	K15520	43.8	218	7.09E-56
4651	Prokka:CIODPCDJ_06448	panE	2-dehydropantoate 2-reductase	1	0	0	1	1	1	1	1	1	1	1	9	Accessory	COG1893_H#	37	160	8.58E-39	K00077	29.7	144	2.22E-33
4652	WP_019521636.1	-	hypothetical protein	1	0	0	0	1	0	0	0	0	0	0	2	Accessory								
4653	Prokka:JAPCLKMA_04091	yedA	putative inner membrane transporter YedA	1	0	0	0	0	0	0	0	0	0	1	2	Accessory	COG0697_GER#	29.4	109	2.27E-23	NA	23.6	67	2.02E-10
4654	OSC61694.1	-	ACP S-malonyltransferase	1	1	1	1	1	1	1	1	1	1	1	11	CORE	COG0331_I#	53.1	287	6.08E-77	K00645	53.1	287	1.62E-76
4655	WP_048460661.1	-	hypothetical protein	0	0	0	0	0	0	0	1	0	0	0	1	Unique								
4656	WP_122213468.1	-	sugar kinase	0	0	0	0	0	0	0	0	0	1	0	1	Unique	COG0524_G#	39.4	162	2.95E-39	K00874	30.6	134	1.76E-30
4657	WP_048455630.1	-	aminoglycoside phosphotransferase family protein	0	0	0	0	0	0	0	1	0	0	0	1	Unique								
4658	OSC57455.1	-	LLM class F420-dependent oxidoreductase	0	1	0	0	0	0	0	0	0	0	0	1	Unique	COG2141_C#	29.4	80	1.47E-14	K00320	30.9	68	1.55E-10
4659	WP_033276197.1	-	RNA polymerase sigma factor SigF	1	1	1	1	1	1	1	1	1	1	1	11	CORE	COG1191_K#	46.9	212	2.48E-54	K03090	46.9	212	6.61E-54

cluster	GI	gene	Annotation	Streptomyces_thermocarboxydus_BPSAC147	Streptomyces_sp._4F	Streptomyces_sp._BSE7F	Streptomyces_sp._ETH9427	Streptomyces_sp._FxanaD5	Streptomyces_sp._UNC401CLCol	Actinospica_acidiphila_NRRL_B_24431	Streptomyces_sp._HNS054	Streptomyces_sp._SMS_SU21	Streptomyces_sp._ZS0098	Streptomyces_thermocarboxydus_K155	Addition	Core-Accessory-Unique	COG	%Similarity_COG	Score_COG	Evalue_COG	KEGG	%Similarity_KEGG	Score_KEGG	Evalue_KEGG
4660	Prokka:CIODPCDJ_04670	-	hypothetical protein	0	0	0	0	0	0	0	0	0	0	1	1	Unique								
4661	Prokka:CIODPCDJ_06278	hcaR_3	Hca operon transcriptional activator HcaR	1	0	0	0	1	1	1	0	0	0	1	5	Accessory	COG0583_K#	35.1	144	4.86E-34	K05817	35.1	144	1.3E-33
4662	WP_048454791.1	-	sugar ABC transporter permease	1	1	1	1	1	1	1	1	1	1	1	11	CORE	COG1175_G#	31.5	148	5.74E-35	K02025	31.6	146	5.82E-34
4663	WP_102641886.1	-	aminoglycoside phosphotransferase family protein	1	1	1	1	1	1	1	1	1	1	1	11	CORE								
4664	Prokka:CIODPCDJ_06144	-	hypothetical protein	1	1	1	1	1	1	1	1	1	1	1	11	CORE	COG4315_S#	40.3	82	3.87E-15				
4665	Prokka:CIODPCDJ_06148	gltC_5	HTH-type transcriptional regulator GltC	1	1	1	1	1	1	1	1	1	1	1	11	CORE	COG0583_K#	36	158	4.24E-38	K09681	33.9	159	6.65E-38
4666	Prokka:JAPCLKMA_02356	-	hypothetical protein	1	0	1	1	1	1	1	1	1	1	1	10	Accessory	COG1595_K#	43.5	210	9.41E-54	K03088	37.9	157	1.93E-37
4667	Prokka:CIODPCDJ_04748	fadM	Proline dehydrogenase 1	1	1	1	1	1	1	1	1	1	1	1	11	CORE	COG0506_E#	48.6	296	9.99E-80	K00318	48.6	296	2.67E-79
4668	WP_078492761.1	-	FHA domain-containing protein	1	1	1	1	1	1	1	1	1	1	1	11	CORE	COG1716_T#	57.7	156	2.11E-37	NA	57.7	156	5.63E-37

cluster	GI	gene	Annotation	Streptomyces_thermocarboxydus_BPSAC147	Streptomyces_sp._4F	Streptomyces_sp._BSE7F	Streptomyces_sp._ETH9427	Streptomyces_sp._FxanaD5	Streptomyces_sp._UNC401CLCol	Actinospica_acidiphila_NRRL_B_24431	Streptomyces_sp._HNS054	Streptomyces_sp._SMS_SU21	Streptomyces_sp._ZS0098	Streptomyces_thermocarboxydus_K155	Addition	Core-Accessory-Unique	COG	%Similarity_COG	Score_COG	Evalue_COG	KEGG	%Similarity_KEGG	Score_KEGG	Evalue_KEGG
4669	WP_028961452.1	-	branched-chain amino acid ABC transporter permease	1	0	1	1	1	1	1	1	1	1	1	10	Accessory	COG0559_E#	40	168	4.1E-41	K11960	36.9	166	3.19E-40
4670	Prokka:CIODPCDJ_06519	-	hypothetical protein	1	0	1	1	0	1	1	1	1	1	1	9	Accessory	COG2755_E#	43.6	205	3.03E-52				
4671	OSC71507.1	-	ABC transporter permease	0	1	0	0	0	0	0	0	0	0	0	1	Unique	COG1175_G#	32.6	179	1.78E-44	K10118	32.6	179	4.75E-44
4672	WP_122216266.1	-	chitin-binding protein	0	0	0	0	0	0	0	0	0	1	0	1	Unique	COG3397_S#	45.9	193	1.55E-48				
4673	OSC58944.1	-	hydroxyneurosporene methyltransferase	0	1	0	0	0	0	0	0	0	0	0	1	Unique	COG0500_QR#	33.3	141	7.01E-33	K13066	31.8	127	2.8E-28
4674	OSC53229.1	-	anti-sigma regulatory factor	0	1	0	0	0	0	0	0	0	0	0	1	Unique								
4675	OSC72533.1	-	NarL family transcriptional regulator	0	1	0	0	0	0	0	0	0	0	0	1	Unique	COG0451_MG#	40.5	168	3.14E-41	NA	40.5	168	8.39E-41
4676	WP_167338239.1	-	hypothetical protein	1	1	1	1	1	1	1	1	1	1	1	11	CORE	COG2353_S#	36	116	2.41E-25	NA	28	81	1.76E-14
4677	WP_102642032.1	-	helix-turn-helix domain-containing protein	1	1	1	1	1	1	1	1	1	1	1	11	CORE								
4678	AXI84990.1	-	hypothetical protein	0	0	1	1	0	0	1	1	1	1	0	6	Accessory								

cluster	GI	gene	Annotation	Streptomyces_thermocarboxydus_BPSAC147	Streptomyces_sp._4F	Streptomyces_sp._BSE7F	Streptomyces_sp._ETH9427	Streptomyces_sp._FxanaD5	Streptomyces_sp._UNC401CLCol	Actinospica_acidiphila_NRRL_B_24431	Streptomyces_sp._HNS054	Streptomyces_sp._SMS_SU21	Streptomyces_sp._ZS0098	Streptomyces_thermocarboxydus_K155	Addition	Core-Accessory-Unique	COG	%Similarity_COG	Score_COG	Evalue_COG	KEGG	%Similarity_KEGG	Score_KEGG	Evalue_KEGG
4679	WP_028961436.1	-	hypothetical protein	1	0	1	0	1	1	1	1	1	1	1	9	Accessory	COG0515_RTKL#	27.6	53	0.00000147	NA	26.4	66	5.84E-10
4680	WP_107080955.1	-	nitroreductase	0	1	0	1	0	0	0	1	1	1	0	5	Accessory								
4681	Prokka:JAPCLKMA_04394	-	hypothetical protein	1	1	1	1	1	1	1	1	1	1	1	11	CORE								
4682	PWE08478.1	-	NADPH:quinone reductase	0	0	1	1	0	0	0	1	1	1	0	5	Accessory	COG0604_CR#	42.7	218	2.64E-56	K00344	34.9	141	1.09E-32
4683	WP_051101571.1	metF	methylenetetrahydrofolate reductase [NAD(P)H]	1	1	1	1	1	1	1	1	1	1	1	11	CORE	COG0685_E#	46.5	253	9.66E-67	K00297	46.5	253	2.58E-66
4684	Prokka:JAPCLKMA_03158	lacG_1	Lactose transport system permease protein LacG	1	1	1	1	1	1	1	1	1	1	1	11	CORE	COG0395_G#	43.4	246	1.18E-64	K17246	35.2	182	5.59E-45
4685	WP_028959380.1	-	ABC transporter permease	1	1	1	1	1	1	1	1	1	1	1	11	CORE	COG0601_EP#	58.8	350	5.81E-96	K15581	57.3	338	6.1E-92
4686	PWE06965.1	-	ABC transporter ATP-binding protein	1	1	1	1	1	1	1	1	1	1	1	11	CORE	COG1131_V#	59.9	350	5.81E-96	K01990	60	349	3.46E-95
4687	WP_033274862.1	-	metallophosphoesterase	1	1	1	1	1	1	1	1	1	1	1	11	CORE	COG1408_R#	53.7	319	1.43E-86	K07098	54	316	2.48E-85
4688	WP_078895236.1	-	restriction endonuclease	0	0	0	0	0	0	0	1	0	0	0	1	Unique								
4689	Prokka:CIODPCDJ_00689	-	hypothetical protein	1	1	1	1	1	1	1	1	1	1	1	11	CORE					NA	27.9	79	5.11E-14

cluster	GI	gene	Annotation	Streptomyces_thermocarboxydus_BPSAC147	Streptomyces_sp._4F	Streptomyces_sp._BSE7F	Streptomyces_sp._ETH9427	Streptomyces_sp._FxanaD5	Streptomyces_sp._UNC401CLCol	Actinospica_acidiphila_NRRL_B_24431	Streptomyces_sp._HNS054	Streptomyces_sp._SMS_SU21	Streptomyces_sp._ZS0098	Streptomyces_thermocarboxydus_K155	Addition	Core-Accessory-Unique	COG	%Similarity_COG	Score_COG	Evalue_COG	KEGG	%Similarity_KEGG	Score_KEGG	Evalue_KEGG
4690	WP_122213903.1	-	LLM class F420-dependent oxidoreductase	1	1	1	1	1	1	1	1	1	1	1	11	CORE	COG2141_C#	34	139	1.56E-32	NA	34	139	4.16E-32
4691	OSC64213.1	-	lysine 6-monooxygenase	0	1	0	0	0	0	0	0	0	0	0	1	Unique	COG3486_Q#	40	219	2.02E-56				
4692	Prokka:JAPCLKMA_04454	-	putative phosphatase	1	1	1	1	1	1	1	1	1	1	1	11	CORE								
4693	WP_033275007.1	-	LysR family transcriptional regulator	0	0	0	0	0	0	1	0	0	0	0	1	Unique	COG0583_K#	35.8	158	5.53E-38	K09681	36.6	160	2.97E-38
4694	Prokka:JAPCLKMA_05335	pmdD	2-pyrone-4,6-dicarbaxylate hydrolase	1	0	1	0	1	1	1	0	1	0	1	7	Accessory	COG3618_R#	38.8	190	1.31E-47				
4695	OSC73081.1	-	glutaminase	0	1	0	0	0	0	0	0	0	0	0	1	Unique	COG2066_E#	55.3	327	6.88E-89	K01425	48.2	277	1.67E-73
4696	WP_102641874.1	-	AraC family transcriptional regulator	1	1	0	1	1	1	1	1	1	1	1	10	Accessory	COG2207_K#	30	76	1.62E-13	NA	38.2	79	6.67E-14
4697	Prokka:JAPCLKMA_04079	-	hypothetical protein	1	1	0	1	1	1	1	1	1	1	1	10	Accessory	COG2030_I#	50.2	270	9.98E-72	NA	50.2	270	2.66E-71
4698	OSC71506.1	-	sugar ABC transporter permease	0	1	0	0	0	0	0	0	0	0	0	1	Unique	COG0395_G#	33.8	191	5.89E-48	K10119	33.8	191	1.57E-47

cluster	GI	gene	Annotation	Streptomyces_thermocarboxydus_BPSAC147	Streptomyces_sp._4F	Streptomyces_sp._BSE7F	Streptomyces_sp._ETH9427	Streptomyces_sp._FxanaD5	Streptomyces_sp._UNC401CLCol	Actinospica_acidiphila_NRRL_B_24431	Streptomyces_sp._HNS054	Streptomyces_sp._SMS_SU21	Streptomyces_sp._ZS0098	Streptomyces_thermocarboxydus_K155	Addition	Core-Accessory-Unique	COG	%Similarity_COG	Score_COG	Evalue_COG	KEGG	%Similarity_KEGG	Score_KEGG	Evalue_KEGG
4699	AXI91047.1	-	antibiotic ABC transporter ATP-binding protein	0	0	0	1	0	0	0	0	0	0	0	1	Unique								
4700	Prokka:CIODPCDJ_06595	-	hypothetical protein	0	0	0	0	0	0	0	0	0	0	1	1	Unique	COG0227_J#	42	63	1.85E-09	K02902	36.8	60	4.19E-08
4701	OSC71486.1	-	alpha/beta hydrolase	0	1	0	0	0	0	0	0	0	0	0	1	Unique	COG0596_R#	29	116	2.41E-25	NA	29	116	6.43E-25
4702	AXI91309.1	-	hypothetical protein	0	0	0	1	0	0	0	0	0	0	0	1	Unique	COG1192_D#	30.2	62	2.42E-09				
4703	PWE08453.1	-	class A beta-lactamase	1	0	1	1	1	1	1	1	1	1	1	10	Accessory	COG2367_V#	53.6	289	1.58E-77	K17836	53.6	289	4.23E-77
4704	WP_048454755.1	-	diiron oxygenase	1	1	1	1	1	1	1	1	1	1	1	11	CORE					NA	27.7	90	3.77E-17
4705	WP_078894940.1	-	restriction endonuclease	0	0	1	1	0	0	0	1	1	0	0	4	Accessory								
4706	WP_122213132.1	-	hypothetical protein	1	1	0	1	1	1	1	1	1	1	1	10	Accessory								
4707	WP_102639747.1	-	ABC transporter ATP-binding protein	1	1	1	1	1	1	1	1	1	1	1	11	CORE	COG1131_V#	54	304	3.64E-82	K01990	33.6	166	3.17E-40
4708	PWE06995.1	-	carbohydrate kinase	1	1	1	1	1	1	1	1	1	1	1	11	CORE	COG0524_G#	43.6	225	2.15E-58	K00847	32.7	130	2.51E-29
4709	WP_033272306.1	-	AEC family transporter	1	1	1	1	1	1	1	1	1	1	1	11	CORE	COG0679_R#	40.4	220	9.04E-57				
4710	OSC73479.1	-	LysR family transcriptional regulator	1	1	1	1	1	1	1	1	1	1	1	11	CORE	COG0583_K#	31	130	1.23E-29	K11921	30.3	117	1.69E-25

cluster	GI	gene	Annotation	Streptomyces_thermocarboxydus_BPSAC147	Streptomyces_sp._4F	Streptomyces_sp._BSE7F	Streptomyces_sp._ETH9427	Streptomyces_sp._FxanaD5	Streptomyces_sp._UNC401CLCol	Actinospica_acidiphila_NRRL_B_24431	Streptomyces_sp._HNS054	Streptomyces_sp._SMS_SU21	Streptomyces_sp._ZS0098	Streptomyces_thermocarboxydus_K155	Addition	Core-Accessory-Unique	COG	%Similarity_COG	Score_COG	Evalue_COG	KEGG	%Similarity_KEGG	Score_KEGG	Evalue_KEGG
4711	AXI87393.1	-	hypothetical protein	1	1	1	1	1	1	1	1	1	1	1	11	CORE								
4712	PWE07275.1	-	Nif3-like dinuclear metal center hexameric protein	1	1	1	1	1	1	1	1	1	1	1	11	CORE	COG0327_S#	29.4	152	3.02E-36	NA	29.4	152	8.07E-36
4713	AXI84910.1	-	hypothetical protein	1	1	1	1	1	1	1	1	1	1	1	11	CORE					NA	22	45	0.00139
4714	PWE08069.1	-	alpha/beta hydrolase	0	1	1	0	0	0	1	0	1	0	0	4	Accessory	COG0596_R#	31.8	116	2.4E-25	NA	32.9	116	6.41E-25
4715	OSC69206.1	-	4-hydroxy-tetrahydrodipicolinate synthase	1	1	1	1	1	1	1	1	1	1	1	11	CORE	COG0329_EM#	48.9	253	1.26E-66	K01714	48.9	253	3.36E-66
4716	Prokka:JAPCLKMA_03321	pgdA	Peptidoglycan deacetylase	1	0	0	0	1	1	0	0	0	0	1	4	Accessory	COG0726_G#	67.7	452	1.41E-126	NA	37	206	4.71E-52
4717	OSC60333.1	-	cobalamin biosynthesis protein CbiX	1	1	1	1	1	1	1	1	1	1	1	11	CORE	COG2138_S#	42.3	240	8.44E-63	K03795	42.3	240	2.25E-62
4718	WP_033274916.1	-	membrane protein	1	1	1	1	1	1	1	1	1	1	1	11	CORE	COG0586_S#	48.2	221	4.06E-57	K03975	45.5	191	1.2E-47
4719	WP_102637597.1	-	helix-turn-helix domain-containing protein	1	1	1	1	1	1	1	1	1	1	1	11	CORE								
4720	Prokka:JAPCLKMA_04952	-	hypothetical protein	1	1	1	1	1	1	1	1	1	1	1	11	CORE	COG0726_G#	27.4	82	2.94E-15	NA	32.5	75	7.35E-13
4721	WP_019524196.1	-	RNA polymerase sigma-70 factor	0	0	0	1	1	0	0	0	0	0	1	3	Accessory	COG1595_K#	44.7	230	6.69E-60	K03088	43.4	205	1.05E-51

cluster	GI	gene	Annotation	Streptomyces_thermocarboxydus_BPSAC147	Streptomyces_sp._4F	Streptomyces_sp._BSE7F	Streptomyces_sp._ETH9427	Streptomyces_sp._FxanaD5	Streptomyces_sp._UNC401CLCol	Actinospica_acidiphila_NRRL_B_24431	Streptomyces_sp._HNS054	Streptomyces_sp._SMS_SU21	Streptomyces_sp._ZS0098	Streptomyces_thermocarboxydus_K155	Addition	Core-Accessory-Unique	COG	%Similarity_COG	Score_COG	Evalue_COG	KEGG	%Similarity_KEGG	Score_KEGG	Evalue_KEGG
4722	OSC73357.1	-	LysR family transcriptional regulator	0	1	0	0	0	0	0	0	0	0	0	1	Unique	COG0583_K#	37	144	6.3E-34	NA	35	137	2.06E-31
4723	WP_026243749.1	-	nucleotidyl transferase AbiEii/AbiGii toxin family protein	1	0	1	0	1	1	0	0	1	0	1	6	Accessory								
4724	OSC62532.1	-	CPBP family intramembrane metalloprotease domain-containing protein	1	1	1	1	1	1	1	1	1	0	1	10	Accessory	COG1266_R#	26.3	47	0.000105	K07052	28.7	101	2.13E-20
4725	PWE10085.1	-	GNAT family N-acetyltransferase	1	0	1	1	1	1	1	1	1	1	1	10	Accessory								
4726	OSC65146.1	-	alpha/beta hydrolase	1	1	1	1	1	1	1	1	1	1	1	11	CORE	COG4188_R#	32	53	0.00000146	K01062	24.6	42	0.0069
4727	AXI85293.1	-	hypothetical protein	0	0	0	1	0	0	0	0	0	0	0	1	Unique								
4728	OSC70053.1	-	hypothetical protein	0	1	0	0	0	0	0	0	0	0	0	1	Unique								
4729	WP_102639437.1	-	DMT family transporter	1	0	1	1	1	1	1	1	1	1	1	10	Accessory	COG0697_GER#	41	217	5.84E-56	NA	41	217	1.56E-55
4730	WP_159020823.1	-	hypothetical protein	0	0	0	0	0	0	0	1	0	0	0	1	Unique								

cluster	GI	gene	Annotation	Streptomyces_thermocarboxydus_BPSAC147	Streptomyces_sp._4F	Streptomyces_sp._BSE7F	Streptomyces_sp._ETH9427	Streptomyces_sp._FxanaD5	Streptomyces_sp._UNC401CLCol	Actinospica_acidiphila_NRRL_B_24431	Streptomyces_sp._HNS054	Streptomyces_sp._SMS_SU21	Streptomyces_sp._ZS0098	Streptomyces_thermocarboxydus_K155	Addition	Core-Accessory-Unique	COG	%Similarity_COG	Score_COG	Evalue_COG	KEGG	%Similarity_KEGG	Score_KEGG	Evalue_KEGG
4731	WP_122217380.1	-	LysR family transcriptional regulator	1	1	1	1	1	1	1	1	1	1	1	11	CORE	COG0583_K#	29.4	120	1.27E-26	NA	33.2	100	2.78E-20
4732	OSC70997.1	-	sugar ABC transporter permease	1	1	1	1	1	1	1	1	1	1	1	11	CORE	COG0395_G#	44.9	262	1.58E-69	K10234	44.9	262	4.23E-69
4733	Prokka:JAPCLKMA_03833	atpG	ATP synthase gamma chain	1	1	1	1	1	1	1	1	1	1	1	11	CORE	COG0224_C#	45.5	274	5.26E-73	K02115	48.1	273	1.83E-72
4734	WP_086700728.1	-	homoserine kinase	1	1	1	1	1	1	1	1	1	1	1	11	CORE	COG0083_E#	44.4	225	2.8E-58	K00872	43.4	223	2.84E-57
4735	WP_028960925.1	-	transketolase	1	0	1	1	1	1	1	1	1	1	1	10	Accessory	COG3958_G#	28	97	8.8E-20	K00615	29.1	92	7.56E-18
4736	WP_033272562.1	-	hypothetical protein	0	0	0	0	1	1	1	0	1	0	0	4	Accessory								
4737	WP_033275799.1	-	DNA-binding protein	1	1	1	1	1	1	1	1	1	1	1	11	CORE	COG0455_D#	29.4	65	2.84E-10				
4738	Prokka:JAPCLKMA_02261	-	hypothetical protein	1	1	1	1	1	1	1	1	1	1	1	11	CORE	COG0697_GER#	26.5	92	3.7E-18				
4739	OSC71869.1	-	LysR family transcriptional regulator	0	1	0	0	0	0	0	0	0	0	0	1	Unique	COG0583_K#	34.1	152	3.01E-36	K05798	23.8	97	2.35E-19
4740	WP_048460726.1	-	ABC transporter permease	1	0	1	1	1	1	1	1	1	1	1	10	Accessory	COG2177_D#	33.8	175	3.32E-43	K09811	33.8	175	8.87E-43

cluster	GI	gene	Annotation	Streptomyces_thermocarboxydus_BPSAC147	Streptomyces_sp._4F	Streptomyces_sp._BSE7F	Streptomyces_sp._ETH9427	Streptomyces_sp._FxanaD5	Streptomyces_sp._UNC401CLCol	Actinospica_acidiphila_NRRL_B_24431	Streptomyces_sp._HNS054	Streptomyces_sp._SMS_SU21	Streptomyces_sp._ZS0098	Streptomyces_thermocarboxydus_K155	Addition	Core-Accessory-Unique	COG	%Similarity_COG	Score_COG	Evalue_COG	KEGG	%Similarity_KEGG	Score_KEGG	Evalue_KEGG
4741	WP_122216365.1	-	sigma-70 family RNA polymerase sigma factor	1	1	1	1	1	1	1	1	1	1	1	11	CORE	COG1595_K#	39.5	183	1.22E-45	K03088	39.5	183	3.26E-45
4742	PWE08296.1	-	hydrolase	1	1	1	1	1	1	1	1	1	1	1	11	CORE	COG0561_R#	42.4	181	4.64E-45	K07024	42.4	181	1.24E-44
4743	WP_033274661.1	-	pyridoxamine 5'-phosphate oxidase	0	0	0	0	0	0	1	0	0	0	0	1	Unique	COG3576_R#	41.1	220	1.18E-56				
4744	WP_033273607.1	-	NADP-dependent oxidoreductase	1	0	1	1	1	0	1	1	1	1	1	9	Accessory	COG0604_CR#	37	169	2.38E-41	NA	37.1	125	1.05E-27
4745	AXI87263.1	-	hypothetical protein	1	1	1	1	1	1	1	1	1	1	1	11	CORE					NA	32.2	108	1.02E-22
4746	Prokka:CIODPCDJ_03575	-	hypothetical protein	1	1	1	1	1	1	1	1	1	1	1	11	CORE								
4747	Prokka:CIODPCDJ_04406	-	hypothetical protein	0	0	0	0	0	0	0	0	0	0	1	1	Unique								
4748	OSC70665.1	-	cation-binding protein	0	1	0	0	0	0	0	0	0	0	0	1	Unique					NA	42.2	93	4.43E-18
4749	WP_033277393.1	-	hypothetical protein	1	1	1	1	1	1	1	1	1	1	1	11	CORE	COG3468_MU#	30.9	63	1.84E-09	NA	27.5	63	3.76E-09
4750	OSC65566.1	-	hypothetical protein	1	1	1	0	1	1	1	1	1	1	1	10	Accessory	COG3409_M#	29.4	62	2.4E-09				
4751	OSC69280.1	-	ornithine carbamoyltransferase	0	1	0	0	0	0	0	0	0	0	0	1	Unique	COG0078_E#	36.5	195	3.1E-49	K00611	34.6	192	5.37E-48

cluster	GI	gene	Annotation	Streptomyces_thermocarboxydus_BPSAC147	Streptomyces_sp._4F	Streptomyces_sp._BSE7F	Streptomyces_sp._ETH9427	Streptomyces_sp._FxanaD5	Streptomyces_sp._UNC401CLCol	Actinospica_acidiphila_NRRL_B_24431	Streptomyces_sp._HNS054	Streptomyces_sp._SMS_SU21	Streptomyces_sp._ZS0098	Streptomyces_thermocarboxydus_K155	Addition	Core-Accessory-Unique	COG	%Similarity_COG	Score_COG	Evalue_COG	KEGG	%Similarity_KEGG	Score_KEGG	Evalue_KEGG
4752	PWE10554.1	-	methyltransferase domain-containing protein	0	0	1	0	0	0	0	0	1	0	0	2	Accessory	COG0500_QR#	33.1	82	2.24E-15	NA	33.1	82	5.99E-15
4753	OSC61463.1	-	site-specific integrase	0	1	0	0	0	0	0	0	0	0	0	1	Unique								
4754	WP_033273891.1	-	LysR family transcriptional regulator	0	0	0	0	0	0	1	0	0	0	0	1	Unique	COG0583_K#	35.2	136	1.31E-31	K05817	35.2	136	3.5E-31
4755	Prokka:CIODPCDJ_01650	-	hypothetical protein	1	1	1	1	1	1	1	1	1	1	1	11	CORE					K01233	37.3	132	6.57E-30
4756	WP_019527684.1	-	hypothetical protein	0	0	0	0	1	0	0	0	0	0	0	1	Unique								
4757	WP_028961064.1	-	ABC transporter permease	1	1	1	1	1	1	1	1	1	1	1	11	CORE	COG0600_P#	32.2	163	1.7E-39	K02050	32.2	163	4.54E-39
4758	AXI88997.1	rpsB	30S ribosomal protein S2	1	1	1	1	1	1	1	1	1	1	1	11	CORE	COG0052_J#	72.8	403	5.73E-112	K02967	72.8	403	1.53E-111
4759	AXI90836.1	-	zinc metalloprotease HtpX	0	0	0	1	0	0	0	1	0	0	0	2	Accessory	COG0501_O#	47	263	1.21E-69	K03799	38.8	176	3.04E-43
4760	WP_078611464.1	-	FadR family transcriptional regulator	1	1	1	1	1	1	1	1	1	1	1	11	CORE	COG2186_K#	32.6	56	2.24E-07	K11922	37.7	54	0.00000297
4761	WP_048460241.1	-	ABC transporter permease	1	1	1	1	1	1	1	1	1	1	1	11	CORE								

cluster	GI	gene	Annotation	Streptomyces_thermocarboxydus_BPSAC147	Streptomyces_sp._4F	Streptomyces_sp._BSE7F	Streptomyces_sp._ETH9427	Streptomyces_sp._FxanaD5	Streptomyces_sp._UNC401CLCol	Actinospica_acidiphila_NRRL_B_24431	Streptomyces_sp._HNS054	Streptomyces_sp._SMS_SU21	Streptomyces_sp._ZS0098	Streptomyces_thermocarboxydus_K155	Addition	Core-Accessory-Unique	COG	%Similarity_COG	Score_COG	Evalue_COG	KEGG	%Similarity_KEGG	Score_KEGG	Evalue_KEGG
4762	WP_028961708.1	-	MerR family transcriptional regulator	1	1	1	1	1	1	1	1	1	1	1	11	CORE	COG0789_K#	35.2	69	3.34E-11	NA	33.1	57	2.06E-07
4763	WP_122214730.1	-	aldo/keto reductase family oxidoreductase	0	1	0	1	0	0	0	1	1	1	0	5	Accessory	COG0667_C#	64.1	362	1.91E-99	NA	62.6	361	8.7E-99
4764	WP_019523352.1	mmuM	homocysteine S-methyltransferase	1	0	1	1	1	1	1	1	1	1	1	10	Accessory	COG2040_E#	58.9	335	1.91E-91	K00547	58.9	335	5.11E-91
4765	WP_019524263.1	-	helix-turn-helix domain-containing protein	1	0	0	0	1	1	1	0	0	0	1	5	Accessory	COG1396_K#	42.9	43	0.0015				
4766	WP_102639691.1	-	aldehyde dehydrogenase family protein	1	1	1	1	1	1	1	1	1	1	1	11	CORE	COG1012_C#	42.5	195	4.04E-49	K00128	35.9	140	2.41E-32
4767	WP_019522283.1	-	ABC transporter ATP-binding protein	1	0	0	0	1	1	1	0	0	0	1	5	Accessory	COG1131_V#	46.7	251	3.64E-66	K01990	46.7	251	9.71E-66
4768	Prokka:JAPCLKMA_06232	-	hypothetical protein	1	0	0	0	1	1	0	0	0	0	1	4	Accessory								
4769	WP_033272883.1	-	sugar ABC transporter permease	1	1	1	1	1	1	1	1	1	1	1	11	CORE	COG3833_G#	38.4	193	1.18E-48	K15772	36.4	189	5.92E-47
4770	PWE09728.1	-	hypothetical protein	1	1	1	0	1	1	1	1	1	1	1	10	Accessory	COG1397_O#	29.6	86	1.55E-16				

cluster	GI	gene	Annotation	Streptomyces_thermocarboxydus_BPSAC147	Streptomyces_sp._4F	Streptomyces_sp._BSE7F	Streptomyces_sp._ETH9427	Streptomyces_sp._FxanaD5	Streptomyces_sp._UNC401CLCol	Actinospica_acidiphila_NRRL_B_24431	Streptomyces_sp._HNS054	Streptomyces_sp._SMS_SU21	Streptomyces_sp._ZS0098	Streptomyces_thermocarboxydus_K155	Addition	Core-Accessory-Unique	COG	%Similarity_COG	Score_COG	Evalue_COG	KEGG	%Similarity_KEGG	Score_KEGG	Evalue_KEGG
4771	OSC57395.1	-	multidrug ABC transporter ATP-binding protein	0	1	0	0	0	0	0	0	0	0	1	2	Accessory	COG1131_V#	48.4	208	2.7E-53	K01990	49.4	205	7.98E-52
4772	PWE10837.1	-	alpha/beta hydrolase	0	0	1	1	0	0	1	0	1	0	0	4	Accessory	COG1073_R#	43.3	237	7.1E-62	K06889	37.6	194	1.84E-48
4773	WP_019525411.1	-	hypothetical protein	0	1	0	0	1	0	0	0	0	0	1	3	Accessory								
4774	WP_122214219.1	-	hypothetical protein	1	1	1	1	1	1	1	1	1	1	1	11	CORE					NA	38.9	184	1.9E-45
4775	WP_019521729.1	-	class I SAM-dependent methyltransferase	1	1	1	1	1	1	1	1	1	1	1	11	CORE	COG0500_QR#	28.1	53	0.0000019				
4776	WP_158688217.1	-	hypothetical protein	0	0	0	0	1	0	0	0	0	0	0	1	Unique								
4777	OSC72474.1	-	hypothetical protein	1	1	1	1	1	1	1	1	1	1	1	11	CORE	COG4760_S#	39.3	179	2.29E-44	NA	39.3	179	6.12E-44
4778	PWE11045.1	-	NADPH:quinone reductase	0	0	1	0	0	0	0	0	0	0	0	1	Unique	COG0604_CR#	38	176	1.49E-43	K00344	33.4	141	1.08E-32
4779	AXI90236.1	-	2-hydroxy-3-oxopropionate reductase	1	1	1	1	1	1	1	1	1	0	1	10	Accessory	COG2084_I#	34.4	153	1.35E-36	K18121	32.4	163	4.54E-39
4780	AXI87665.1	-	carbohydrate-binding protein	0	0	0	1	0	0	0	0	0	0	0	1	Unique								
4781	WP_048457269.1	-	hypothetical protein	1	0	1	0	0	0	1	1	1	1	1	7	Accessory								
4782	Prokka:JAPCLKMA_05683	-	hypothetical protein	1	0	0	0	0	0	0	0	0	0	0	1	Unique								

cluster	GI	gene	Annotation	Streptomyces_thermocarboxydus_BPSAC147	Streptomyces_sp._4F	Streptomyces_sp._BSE7F	Streptomyces_sp._ETH9427	Streptomyces_sp._FxanaD5	Streptomyces_sp._UNC401CLCol	Actinospica_acidiphila_NRRL_B_24431	Streptomyces_sp._HNS054	Streptomyces_sp._SMS_SU21	Streptomyces_sp._ZS0098	Streptomyces_thermocarboxydus_K155	Addition	Core-Accessory-Unique	COG	%Similarity_COG	Score_COG	Evalue_COG	KEGG	%Similarity_KEGG	Score_KEGG	Evalue_KEGG
4783	PWE07009.1	-	sugar kinase	1	0	1	0	1	1	1	1	1	1	1	9	Accessory	COG0524_G#	36.8	168	4.04E-41	NA	36.8	168	1.08E-40
4784	OSC71218.1	-	universal stress protein UspA	1	1	1	1	1	1	1	1	1	1	1	11	CORE	COG0589_T#	35	142	2.37E-33	NA	35	142	6.33E-33
4785	PWE10475.1	-	sugar ABC transporter ATP-binding protein	1	1	1	1	1	1	1	1	1	0	0	9	Accessory	COG1129_G#	63.6	317	7.02E-86	K10441	32.6	149	5.18E-35
4786	WP_102641481.1	galU	UTP--glucose-1-phosphate uridylyltransferase GalU	1	1	1	1	1	1	1	1	1	1	1	11	CORE	COG1210_M#	50.5	296	1.28E-79	K00963	50.5	296	3.43E-79
4787	WP_019525979.1	-	ABC transporter ATP-binding protein	1	1	1	1	1	1	1	1	1	1	1	11	CORE	COG1131_V#	38	164	4.46E-40	K01990	30.5	153	4.68E-36
4788	WP_102639720.1	nuoE	NADH-quinone oxidoreductase subunit NuoE	1	1	1	1	1	1	1	1	1	1	1	11	CORE	COG1905_C#	70.4	312	1.73E-84	K00334	70.4	312	4.62E-84
4789	WP_028961136.1	-	TauD/TfdA family dioxygenase	1	0	0	0	1	1	1	0	0	0	1	5	Accessory	COG2175_Q#	51.6	284	5.05E-76	K03119	48.5	260	1.6E-68
4790	AXI85770.1	-	pyridoxal 5'-phosphate synthase lyase subunit PdxS	1	1	1	1	1	1	1	1	1	1	1	11	CORE	COG0214_H#	75.2	448	2.64E-125	K06215	77.6	443	1.33E-123

cluster	GI	gene	Annotation	Streptomyces_thermocarboxydus_BPSAC147	Streptomyces_sp._4F	Streptomyces_sp._BSE7F	Streptomyces_sp._ETH9427	Streptomyces_sp._FxanaD5	Streptomyces_sp._UNC401CLCol	Actinospica_acidiphila_NRRL_B_24431	Streptomyces_sp._HNS054	Streptomyces_sp._SMS_SU21	Streptomyces_sp._ZS0098	Streptomyces_thermocarboxydus_K155	Addition	Core-Accessory-Unique	COG	%Similarity_COG	Score_COG	Evalue_COG	KEGG	%Similarity_KEGG	Score_KEGG	Evalue_KEGG
4791	WP_078504467.1	-	peptidoglycan recognition protein	1	0	1	1	1	1	1	1	1	1	1	10	Accessory	COG5479_M#	40.7	150	8.71E-36	NA	40.7	150	3.04E-35
4792	Prokka:CIODPCDJ_05837	hpnC	Hydroxysqualene synthase	1	1	1	1	1	1	1	1	1	1	1	11	CORE	COG1562_I#	38.6	169	1.81E-41	NA	38.6	169	4.84E-41
4793	WP_051101698.1	-	HAD-IB family hydrolase	1	1	1	1	1	1	1	1	1	1	1	11	CORE	COG0560_E#	57.7	309	1.46E-83	NA	57.7	309	3.91E-83
4794	WP_033272266.1	-	helix-turn-helix domain-containing protein	1	1	1	1	1	1	1	1	1	1	1	11	CORE	COG1396_K#	45.2	230	8.65E-60				
4795	Prokka:CIODPCDJ_04513	dcyD	D-cysteine desulfhydrase	1	1	1	1	1	1	1	1	1	1	1	11	CORE	COG2515_E#	40	199	1.63E-50				
4796	Prokka:JAPCLKMA_01594	-	hypothetical protein	1	0	0	0	0	0	0	0	0	0	0	1	Unique	COG0501_O#	35.9	155	3.53E-37	NA	34	147	2.57E-34
4797	AXI85290.1	-	HEXXH motif domain-containing protein	0	0	0	1	0	0	0	0	0	0	0	1	Unique								
4798	OSC70393.1	-	NAD kinase	1	1	1	1	1	1	1	1	1	1	1	11	CORE	COG0061_G#	54.2	324	3.37E-88	K00858	52.5	311	7.88E-84
4799	WP_048457380.1	-	TatD family hydrolase	1	1	1	1	1	1	1	1	1	1	1	11	CORE	COG0084_L#	46.6	172	2.14E-42	K03424	46.6	172	5.71E-42
4800	WP_122216606.1	-	LysR family transcriptional regulator	0	0	0	0	0	0	0	0	0	1	0	1	Unique	COG0583_K#	47.6	275	3.06E-73	NA	35.1	164	1.56E-39
4801	OSC71520.1	-	restriction endonuclease	0	1	0	0	0	0	0	0	0	0	0	1	Unique	COG3440_V#	34.4	163	1.3E-39				

cluster	GI	gene	Annotation	Streptomyces_thermocarboxydus_BPSAC147	Streptomyces_sp._4F	Streptomyces_sp._BSE7F	Streptomyces_sp._ETH9427	Streptomyces_sp._FxanaD5	Streptomyces_sp._UNC401CLCol	Actinospica_acidiphila_NRRL_B_24431	Streptomyces_sp._HNS054	Streptomyces_sp._SMS_SU21	Streptomyces_sp._ZS0098	Streptomyces_thermocarboxydus_K155	Addition	Core-Accessory-Unique	COG	%Similarity_COG	Score_COG	Evalue_COG	KEGG	%Similarity_KEGG	Score_KEGG	Evalue_KEGG
4802	AXI90639.1	-	hydroxyacid dehydrogenase	0	0	0	1	0	0	0	0	0	0	0	1	Unique	COG0111_HE#	28.3	101	4.64E-21	K00058	28.9	97	1.79E-19
4803	OSC66143.1	-	aldo/keto reductase	0	1	0	0	0	0	0	0	0	0	0	1	Unique	COG0667_C#	32.8	127	7.89E-29				
4804	Prokka:JAPCLKMA_01619	czcD_1	Metal cation efflux system protein CzcD	1	0	0	0	0	0	0	0	0	0	0	1	Unique	COG1230_P#	42.8	216	1.69E-55	K16264	41.4	200	2.56E-50
4805	OSC57287.1	-	transcriptional regulator	0	1	0	0	0	0	0	0	0	0	0	1	Unique								
4806	OSC61648.1	-	alcohol dehydrogenase	0	1	0	0	0	0	0	0	0	0	0	1	Unique	COG0604_CR#	37.3	174	5.63E-43	K00344	32.8	121	1.16E-26
4807	OSC72296.1	-	hypothetical protein	1	1	1	0	1	1	1	1	1	1	1	10	Accessory	COG2030_I#	37.4	171	4.77E-42	NA	34.7	144	1.67E-33
4808	WP_033276064.1	-	aminoglycoside phosphotransferase family protein	0	0	1	1	0	0	1	1	1	0	0	5	Accessory	COG3173_R#	39.9	191	3.41E-48	NA	40	208	1.23E-52
4809	WP_122214206.1	-	LysR family transcriptional regulator	0	1	1	1	0	0	1	1	1	1	1	8	Accessory	COG0583_K#	33.2	132	1.87E-30	NA	35.2	109	4.53E-23
4810	Prokka:JAPCLKMA_01320	ngcG_3	Diacetylchitobiose uptake system permease protein NgcG	1	0	1	1	1	1	1	1	1	1	1	10	Accessory	COG0395_G#	34.3	196	1.06E-49	K10119	30.6	175	6.73E-43

cluster	GI	gene	Annotation	Streptomyces_thermocarboxydus_BPSAC147	Streptomyces_sp._4F	Streptomyces_sp._BSE7F	Streptomyces_sp._ETH9427	Streptomyces_sp._FxanaD5	Streptomyces_sp._UNC401CLCol	Actinospica_acidiphila_NRRL_B_24431	Streptomyces_sp._HNS054	Streptomyces_sp._SMS_SU21	Streptomyces_sp._ZS0098	Streptomyces_thermocarboxydus_K155	Addition	Core-Accessory-Unique	COG	%Similarity_COG	Score_COG	Evalue_COG	KEGG	%Similarity_KEGG	Score_KEGG	Evalue_KEGG
4811	OSC66334.1	-	2-keto-myo-inositol dehydratase	1	1	1	1	1	1	1	1	1	1	1	11	CORE	COG1082_G#	52.2	325	1.51E-88				
4812	OSC64991.1	-	gluconolactonase	0	1	0	0	0	0	0	0	0	0	0	1	Unique	COG3386_G#	54.5	333	9.44E-91	K01053	36.8	174	1.5E-42
4813	PWE11015.1	-	NADP-dependent oxidoreductase	0	0	1	0	0	0	0	0	1	0	0	2	Accessory	COG0604_CR#	37.9	170	1.06E-41	NA	29.9	101	2.11E-20
4814	WP_102640102.1	-	hydroxyurea phosphotransferase	1	1	1	1	1	1	1	1	1	1	1	11	CORE								
4815	WP_019523146.1	-	ribonuclease Z	1	1	1	1	1	1	1	1	1	1	1	11	CORE	COG1234_R#	46.7	273	6.8E-73	K00784	46.3	270	2.62E-71
4816	WP_033274416.1	-	sugar ABC transporter permease	1	0	1	1	1	1	1	1	1	1	1	10	Accessory	COG1175_G#	32.7	160	1.1E-38	K10118	32.7	160	2.92E-38
4817	Prokka:JAPCLKMA_00460	glpQ1	putative glycerophosphodiester phosphodiesterase 1	1	1	1	1	1	1	1	1	1	1	1	11	CORE	COG0584_C#	48.3	223	1.05E-57	K01126	48.3	223	2.81E-57
4818	WP_033274927.1	folP	dihydropteroate synthase	1	1	1	0	1	1	1	1	1	1	1	10	Accessory	COG0294_H#	57.6	282	1.91E-75	K00796	57.6	282	5.1E-75
4819	Prokka:CIODPCDJ_02160	-	hypothetical protein	1	1	1	0	1	1	1	1	1	1	1	10	Accessory	COG1555_L#	37.4	152	2.28E-36	K02237	37.4	152	6.1E-36
4820	OSC61975.1	-	glycerophosphodiester phosphodiesterase	1	1	1	1	1	1	1	1	1	1	1	11	CORE	COG0584_C#	33.8	155	3.52E-37	K01126	33.8	155	9.41E-37

cluster	GI	gene	Annotation	Streptomyces_thermocarboxydus_BPSAC147	Streptomyces_sp._4F	Streptomyces_sp._BSE7F	Streptomyces_sp._ETH9427	Streptomyces_sp._FxanaD5	Streptomyces_sp._UNC401CLCol	Actinospica_acidiphila_NRRL_B_24431	Streptomyces_sp._HNS054	Streptomyces_sp._SMS_SU21	Streptomyces_sp._ZS0098	Streptomyces_thermocarboxydus_K155	Addition	Core-Accessory-Unique	COG	%Similarity_COG	Score_COG	Evalue_COG	KEGG	%Similarity_KEGG	Score_KEGG	Evalue_KEGG
4821	AXI85188.1	-	hypothetical protein	1	1	0	1	1	1	1	0	1	1	1	9	Accessory	COG0589_T#	31.6	124	5.1E-28	NA	34.4	114	1.41E-24
4822	OSC57278.1	-	phosphotriesterase	0	1	0	0	0	0	0	0	0	0	0	1	Unique	COG1735_R#	28.5	145	3.65E-34	K07048	32.7	129	7.22E-29
4823	Prokka:JAPCLKMA_06295	-	hypothetical protein	1	0	0	0	1	1	0	0	0	0	1	4	Accessory								
4824	WP_106432482.1	-	IS5 family transposase	1	1	1	0	1	0	0	1	0	0	1	6	Accessory	COG3293_L#	41.2	208	3.51E-53	K07492	43.8	136	4.52E-31
4825	WP_019524850.1	-	pseudouridine-5'-phosphate glycosidase	0	0	0	0	1	1	0	0	0	0	1	3	Accessory	COG2313_Q#	44.4	227	5.59E-59	K16329	44.1	225	5.67E-58
4826	OSC73156.1	-	hypothetical protein	0	1	0	0	0	0	0	0	0	0	0	1	Unique								
4827	Prokka:CIODPCDJ_00223	-	hypothetical protein	0	0	0	0	0	1	0	0	0	0	1	2	Accessory								
4828	Prokka:CIODPCDJ_05212	-	hypothetical protein	0	0	0	0	0	0	0	0	0	0	1	1	Unique								
4829	WP_102641762.1	-	hypothetical protein	0	0	1	0	0	0	0	0	1	0	0	2	Accessory								
4830	Prokka:CIODPCDJ_06765	-	Anti-sigma F factor	0	0	0	0	1	0	0	0	0	0	1	2	Accessory								
4831	WP_037952935.1	-	shikimate dehydrogenase	1	1	0	0	1	1	0	0	0	0	1	5	Accessory	COG0169_E#	63.9	342	1.56E-93	K00014	33.1	132	5E-30
4832	AXI85712.1	-	6-phosphogluconate dehydrogenase	0	0	1	1	0	0	1	1	1	1	0	6	Accessory	COG2084_I#	46.2	133	1.43E-30	NA	25.3	84	2.04E-15
4833	Prokka:CIODPCDJ_05794	-	Putative glyoxylase CFP32	1	0	1	1	1	1	1	1	1	1	1	10	Accessory	COG3324_R#	36.3	142	3.09E-33	K06996	36.3	142	8.24E-33

cluster	GI	gene	Annotation	Streptomyces_thermocarboxydus_BPSAC147	Streptomyces_sp._4F	Streptomyces_sp._BSE7F	Streptomyces_sp._ETH9427	Streptomyces_sp._FxanaD5	Streptomyces_sp._UNC401CLCol	Actinospica_acidiphila_NRRL_B_24431	Streptomyces_sp._HNS054	Streptomyces_sp._SMS_SU21	Streptomyces_sp._ZS0098	Streptomyces_thermocarboxydus_K155	Addition	Core-Accessory-Unique	COG	%Similarity_COG	Score_COG	Evalue_COG	KEGG	%Similarity_KEGG	Score_KEGG	Evalue_KEGG
4834	WP_048456571.1	-	chitinase	1	1	1	1	1	1	1	1	1	1	1	11	CORE	COG3179_R#	30.1	44	0.000668	K01183	43	169	6.28E-41
4835	Prokka:CIODPCDJ_02927	-	hypothetical protein	1	0	0	0	0	0	0	0	0	0	1	2	Accessory								
4836	WP_019526502.1	-	4-hydroxybenzoate octaprenyltransferase	1	1	1	1	1	1	1	1	1	1	1	11	CORE	COG0382_H#	45.6	242	1.67E-63	K03179	45.6	242	4.47E-63
4837	WP_028960868.1	-	LysR family transcriptional regulator	1	1	1	1	1	1	1	1	1	1	1	11	CORE	COG0583_K#	31.1	91	6.23E-18	NA	23	59	0.00000007
4838	OSC72097.1	-	hypothetical protein	0	1	0	0	0	0	0	0	0	0	0	1	Unique	COG2207_K#	32.5	75	3.54E-13	NA	26.5	63	4.85E-09
4839	WP_122216147.1	-	LysR family transcriptional regulator	1	1	1	1	1	1	1	1	1	1	1	11	CORE	COG0583_K#	31	138	5.8E-32	NA	29.9	115	8.23E-25
4840	AXI90411.1	thpD	ectoine hydroxylase	1	1	1	1	1	1	1	1	1	1	1	11	CORE								
4841	OSC63201.1	-	hypothetical protein	1	1	1	1	1	1	1	1	1	1	1	11	CORE	COG3879_S#	34.9	141	4.02E-33	NA	34.9	141	1.07E-32
4842	WP_122215089.1	-	trypsin-like serine protease	1	1	0	1	1	1	1	1	1	1	1	10	Accessory								
4843	OSC71212.1	-	stress-inducible protein	1	1	1	1	1	1	1	1	1	1	1	11	CORE	COG0589_T#	32.4	135	3.76E-31	NA	32.4	135	1E-30
4844	WP_019527002.1	-	kinase	1	1	1	1	1	1	1	1	1	1	1	11	CORE								
4845	Prokka:JAPCLKMA_06473	rbsK_2	Ribokinase	1	1	1	1	1	1	1	1	1	1	1	11	CORE	COG0524_G#	46.1	233	1.33E-60	K00852	42.1	195	1.07E-48

cluster	GI	gene	Annotation	Streptomyces_thermocarboxydus_BPSAC147	Streptomyces_sp._4F	Streptomyces_sp._BSE7F	Streptomyces_sp._ETH9427	Streptomyces_sp._FxanaD5	Streptomyces_sp._UNC401CLCol	Actinospica_acidiphila_NRRL_B_24431	Streptomyces_sp._HNS054	Streptomyces_sp._SMS_SU21	Streptomyces_sp._ZS0098	Streptomyces_thermocarboxydus_K155	Addition	Core-Accessory-Unique	COG	%Similarity_COG	Score_COG	Evalue_COG	KEGG	%Similarity_KEGG	Score_KEGG	Evalue_KEGG
4846	WP_033277983.1	-	class I SAM-dependent methyltransferase	0	1	1	1	0	0	1	1	1	1	0	7	Accessory	COG3315_Q#	41.8	176	1.92E-43	NA	41.8	176	5.13E-43
4847	WP_019524243.1	-	NADP-dependent oxidoreductase	1	1	1	1	1	1	1	0	1	0	1	9	Accessory	COG0604_CR#	34.9	169	1.38E-41	NA	32.3	129	5.51E-29
4848	WP_048460061.1	-	virginiamycin B lyase	1	1	1	1	1	1	1	1	1	1	1	11	CORE	COG4257_V#	27.9	87	8.99E-17				
4849	WP_019526287.1	-	amino acid ABC transporter permease	1	1	1	1	1	1	1	1	1	1	1	11	CORE	COG0765_E#	45.3	242	2.85E-63	K02029	34.3	118	1.27E-25
4850	PWE07403.1	-	hypothetical protein	0	0	1	0	0	0	0	0	0	0	0	1	Unique								
4851	OSC63554.1	-	LysR family transcriptional regulator	0	1	0	0	0	0	0	0	0	0	0	1	Unique	COG0583_K#	30	121	7.34E-27	K02521	29.5	62	8.27E-09
4852	WP_048454973.1	-	LysR family transcriptional regulator	1	0	1	1	1	1	1	1	1	1	1	10	Accessory	COG0583_K#	33.6	108	3.77E-23	NA	23.4	68	1.15E-10
4853	Prokka:CIODPCDJ_06276	-	hypothetical protein	0	0	0	0	0	0	1	0	0	0	1	2	Accessory	COG1028_IQR#	57.3	336	8.51E-92	NA	30.4	102	5.52E-21
4854	WP_019523077.1	-	AraC family transcriptional regulator	1	0	0	0	1	1	1	0	0	0	1	5	Accessory	COG2207_K#	45.5	242	2.18E-63	NA	33.7	141	1.83E-32
4855	OSC70505.1	-	oxidoreductase	1	1	1	1	1	1	1	1	1	1	1	11	CORE	COG0673_R#	42.1	225	2.12E-58	K03810	33.9	135	7.69E-31

cluster	GI	gene	Annotation	Streptomyces_thermocarboxydus_BPSAC147	Streptomyces_sp._4F	Streptomyces_sp._BSE7F	Streptomyces_sp._ETH9427	Streptomyces_sp._FxanaD5	Streptomyces_sp._UNC401CLCol	Actinospica_acidiphila_NRRL_B_24431	Streptomyces_sp._HNS054	Streptomyces_sp._SMS_SU21	Streptomyces_sp._ZS0098	Streptomyces_thermocarboxydus_K155	Addition	Core-Accessory-Unique	COG	%Similarity_COG	Score_COG	Evalue_COG	KEGG	%Similarity_KEGG	Score_KEGG	Evalue_KEGG
4856	OSC72990.1	-	luciferase	1	1	1	1	1	1	1	1	1	1	1	11	CORE	COG2141_C#	37.7	82	2.21E-15	NA	37.7	82	5.91E-15
4857	Prokka:CIODPCDJ_03956	qorB_2	Quinone oxidoreductase 2	0	0	0	0	1	1	0	0	0	0	1	3	Accessory	COG0702_MG#	36	166	1.99E-40	K19267	35.6	157	1.89E-37
4858	AXI87126.1	-	oxidoreductase	0	0	0	1	0	0	0	0	0	0	0	1	Unique	COG1028_IQR#	50.8	260	1.01E-68	NA	50.5	258	6.02E-68
4859	WP_048458679.1	-	helix-turn-helix domain-containing protein	1	0	0	1	1	0	1	1	0	0	0	5	Accessory								
4860	AXI88195.1	-	hypothetical protein	0	0	0	1	0	0	0	0	0	0	0	1	Unique								
4861	Prokka:CIODPCDJ_00019	-	hypothetical protein	0	0	0	0	0	0	0	0	0	0	1	1	Unique								
4862	WP_048458590.1	-	sigma-70 family RNA polymerase sigma factor	0	1	0	0	1	0	0	1	0	0	0	3	Accessory								
4863	AXI89661.1	-	hypothetical protein	0	0	0	1	0	0	0	0	0	0	0	1	Unique					NA	29.4	106	6.52E-22
4864	AXI85268.1	-	aminoglycoside phosphotransferase	0	0	0	1	0	0	0	0	0	0	0	1	Unique								
4865	WP_033273091.1	-	MIP family channel protein	1	0	1	1	1	1	1	1	1	1	1	10	Accessory	COG0580_G#	39.1	186	1.85E-46	K09878	39.3	200	1.94E-50
4866	AXI86572.1	-	hypothetical protein	0	0	1	1	0	0	0	1	1	1	0	5	Accessory								
4867	WP_033275749.1	-	hypothetical protein	1	1	1	1	1	1	1	1	1	1	1	11	CORE								

cluster	GI	gene	Annotation	Streptomyces_thermocarboxydus_BPSAC147	Streptomyces_sp._4F	Streptomyces_sp._BSE7F	Streptomyces_sp._ETH9427	Streptomyces_sp._FxanaD5	Streptomyces_sp._UNC401CLCol	Actinospica_acidiphila_NRRL_B_24431	Streptomyces_sp._HNS054	Streptomyces_sp._SMS_SU21	Streptomyces_sp._ZS0098	Streptomyces_thermocarboxydus_K155	Addition	Core-Accessory-Unique	COG	%Similarity_COG	Score_COG	Evalue_COG	KEGG	%Similarity_KEGG	Score_KEGG	Evalue_KEGG
4868	WP_033275789.1	-	metal ABC transporter permease	1	0	1	1	1	1	1	1	1	1	1	10	Accessory	COG1108_P#	37	185	3.16E-46	K09816	37	185	8.43E-46
4869	WP_019522703.1	-	tRNA (adenine-N1)-methyltransferase	1	1	1	1	1	1	1	1	1	1	1	11	CORE	COG2519_J#	55.8	314	5.89E-85	K07442	56.9	312	3.5E-84
4870	WP_122215931.1	-	radical SAM protein	0	0	0	0	0	0	0	0	0	1	0	1	Unique	COG0535_R#	31.8	93	1.25E-18	NA	26.2	75	1.23E-12
4871	WP_019527419.1	-	bifunctional DNA primase/polymerase	1	0	0	0	1	0	1	0	0	0	0	3	Accessory								
4872	WP_033272474.1	-	hypothetical protein	1	0	0	1	0	0	1	1	0	1	0	5	Accessory								
4873	OSC65847.1	-	NUDIX hydrolase	0	1	0	0	0	0	0	0	0	0	0	1	Unique	COG4043_S#	31.8	45	0.000299	K01515	42.2	50	0.0000423
4874	WP_048458563.1	-	DUF3626 domain-containing protein	1	0	0	0	1	1	0	1	0	1	1	6	Accessory								
4875	AXI85355.1	-	relaxase/mobilization nuclease	1	0	0	1	0	0	0	0	0	0	0	2	Accessory								
4876	Prokka:CIODPCDJ_06537	dasC_10	Diacetylchitobiose uptake system permease protein DasC	1	0	0	1	1	1	1	1	0	0	1	7	Accessory	COG0395_G#	39	211	4.12E-54	K10238	35.3	163	3.43E-39
4877	Prokka:CIODPCDJ_02306	-	hypothetical protein	1	1	1	1	1	1	1	1	1	1	1	11	CORE	COG1277_R#	26.2	58	5.82E-08	K01992	26.2	58	1.55E-07

cluster	GI	gene	Annotation	Streptomyces_thermocarboxydus_BPSAC147	Streptomyces_sp._4F	Streptomyces_sp._BSE7F	Streptomyces_sp._ETH9427	Streptomyces_sp._FxanaD5	Streptomyces_sp._UNC401CLCol	Actinospica_acidiphila_NRRL_B_24431	Streptomyces_sp._HNS054	Streptomyces_sp._SMS_SU21	Streptomyces_sp._ZS0098	Streptomyces_thermocarboxydus_K155	Addition	Core-Accessory-Unique	COG	%Similarity_COG	Score_COG	Evalue_COG	KEGG	%Similarity_KEGG	Score_KEGG	Evalue_KEGG
4878	OSC69884.1	-	succinate--CoA ligase subunit alpha	1	1	1	1	1	1	1	1	1	1	1	11	CORE	COG0074_C#	66.7	401	3.66E-111	K01902	67.3	396	2.41E-109
4879	AXI86458.1	-	fumarylacetoacetate (FAA) hydrolase	0	0	0	1	0	0	0	1	0	0	0	2	Accessory								
4880	WP_102641841.1	-	AfsR/SARP family transcriptional regulator	0	1	0	1	0	0	0	1	1	1	0	5	Accessory	COG3629_T#	34.4	93	1.25E-18	NA	34.4	93	3.34E-18
4881	OSC58937.1	-	hypothetical protein	0	1	0	0	0	0	0	0	0	0	0	1	Unique								
4882	OSC56516.1	-	hypothetical protein	0	1	0	0	0	0	0	0	0	0	0	1	Unique	COG2409_R#	29.1	107	1.09E-22	K06994	29.1	107	2.92E-22
4883	OSC70661.1	-	ABC transporter ATP-binding protein	0	1	0	0	0	0	0	0	0	0	0	1	Unique	COG1131_V#	41.6	202	1.91E-51	K01990	38.2	184	1.88E-45
4884	WP_028961658.1	-	helix-turn-helix domain-containing protein	1	0	0	0	1	1	1	0	0	1	1	6	Accessory	COG1813_K#	36.4	43	0.00194				
4885	OSC73333.1	-	SAM-dependent methyltransferase	1	1	1	1	1	1	1	1	1	1	1	11	CORE								
4886	WP_019527774.1	-	aminoglycoside phosphotransferase family protein	0	0	0	0	1	0	0	0	0	0	0	1	Unique								

cluster	GI	gene	Annotation	Streptomyces_thermocarboxydus_BPSAC147	Streptomyces_sp._4F	Streptomyces_sp._BSE7F	Streptomyces_sp._ETH9427	Streptomyces_sp._FxanaD5	Streptomyces_sp._UNC401CLCol	Actinospica_acidiphila_NRRL_B_24431	Streptomyces_sp._HNS054	Streptomyces_sp._SMS_SU21	Streptomyces_sp._ZS0098	Streptomyces_thermocarboxydus_K155	Addition	Core-Accessory-Unique	COG	%Similarity_COG	Score_COG	Evalue_COG	KEGG	%Similarity_KEGG	Score_KEGG	Evalue_KEGG
4887	OSC54509.1	-	phosphotransferase	0	1	0	0	0	0	0	0	0	0	0	1	Unique	COG3173_R#	50	275	2.32E-73	NA	49.2	285	4.59E-76
4888	OSC67483.1	-	saccharopine dehydrogenase	0	1	0	0	0	0	0	0	0	0	0	1	Unique	COG1748_E#	36.6	82	3.77E-15				
4889	OSC71130.1	-	DNA-binding protein	0	1	0	0	0	0	0	0	0	0	0	1	Unique	COG1396_K#	46.1	254	4.24E-67				
4890	WP_102637152.1	-	class I SAM-dependent methyltransferase	0	1	1	1	0	0	1	1	1	1	0	7	Accessory	COG0500_QR#	27.5	69	2.52E-11	NA	38.1	63	3.69E-09
4891	AXI90736.1	-	hypothetical protein	1	1	1	1	1	1	1	1	1	1	1	11	CORE								
4892	Prokka:JAPCLKMA_05779	menH_5	2-succinyl-6-hydroxy-2, 4-cyclohexadiene-1-carboxylate synthase	1	1	1	1	1	1	1	1	1	1	1	11	CORE	COG2267_I#	24.1	56	1.69E-07				
4893	WP_019526543.1	-	phosphoribosylaminoimidazolesuccinocarboxamide synthase	1	1	1	1	1	1	1	1	1	1	1	11	CORE	COG0152_F#	58.3	336	8.45E-92	K01923	58.3	327	1.05E-88
4894	Prokka:JAPCLKMA_06651	-	hypothetical protein	1	0	0	1	1	1	1	1	1	1	1	9	Accessory	COG1366_T#	64.5	165	2.58E-40	K17763	28.5	122	5.12E-27
4895	OSC66482.1	-	hypothetical protein	1	1	0	1	1	1	0	1	0	1	1	8	Accessory								
4896	Prokka:JAPCLKMA_02061	-	hypothetical protein	1	1	1	1	1	1	1	1	1	1	1	11	CORE	COG1396_K#	40	189	1.67E-47				

cluster	GI	gene	Annotation	Streptomyces_thermocarboxydus_BPSAC147	Streptomyces_sp._4F	Streptomyces_sp._BSE7F	Streptomyces_sp._ETH9427	Streptomyces_sp._FxanaD5	Streptomyces_sp._UNC401CLCol	Actinospica_acidiphila_NRRL_B_24431	Streptomyces_sp._HNS054	Streptomyces_sp._SMS_SU21	Streptomyces_sp._ZS0098	Streptomyces_thermocarboxydus_K155	Addition	Core-Accessory-Unique	COG	%Similarity_COG	Score_COG	Evalue_COG	KEGG	%Similarity_KEGG	Score_KEGG	Evalue_KEGG
4897	WP_122214731.1	-	helix-turn-helix transcriptional regulator	0	0	1	1	0	0	0	1	1	1	0	5	Accessory	COG1396_K#	40.7	179	1.32E-44				
4898	WP_102641468.1	-	LLM class flavin-dependent oxidoreductase	1	0	1	1	1	1	1	1	1	1	1	10	Accessory	COG2141_C#	36.5	144	6.16E-34	NA	36.5	144	1.64E-33
4899	WP_028960974.1	-	polyprenol monophosphomannose synthase	1	1	1	1	1	1	1	1	1	1	1	11	CORE	COG0463_M#	61.3	298	2.55E-80	K03820	54.6	272	6.83E-72
4900	WP_048458757.1	-	carbohydrate ABC transporter permease	1	0	1	1	1	1	1	1	1	1	1	10	Accessory	COG0395_G#	32.1	163	1.28E-39	K10119	27.5	133	3.79E-30
4901	WP_048459969.1	-	NAD-dependent protein deacetylase	1	0	1	1	1	1	1	1	1	1	1	10	Accessory	COG0846_K#	47.2	150	8.59E-36	K11414	46.6	238	8.37E-62
4902	AXI87612.1	-	GntR family transcriptional regulator	0	0	0	1	1	0	0	1	0	1	0	4	Accessory	COG1167_KE#	36.4	55	4.91E-07	K14348	43.7	47	0.000209
4903	OSC62639.1	-	branched-chain amino acid ABC transporter permease	1	1	1	1	1	1	1	1	1	1	1	11	CORE	COG0559_E#	35.4	151	6.58E-36	K01997	34.9	151	1.76E-35

cluster	GI	gene	Annotation	Streptomyces_thermocarboxydus_BPSAC147	Streptomyces_sp._4F	Streptomyces_sp._BSE7F	Streptomyces_sp._ETH9427	Streptomyces_sp._FxanaD5	Streptomyces_sp._UNC401CLCol	Actinospica_acidiphila_NRRL_B_24431	Streptomyces_sp._HNS054	Streptomyces_sp._SMS_SU21	Streptomyces_sp._ZS0098	Streptomyces_thermocarboxydus_K155	Addition	Core-Accessory-Unique	COG	%Similarity_COG	Score_COG	Evalue_COG	KEGG	%Similarity_KEGG	Score_KEGG	Evalue_KEGG
4904	WP_048456184.1	-	MBL fold metallo-hydrolase	0	1	0	0	0	0	0	1	1	0	0	3	Accessory	COG0491_R#	31.7	104	7.06E-22	NA	28.9	65	9.71E-10
4905	Prokka:CIODPCDJ_06268	-	hypothetical protein	1	0	0	0	1	1	1	0	0	0	1	5	Accessory	COG0714_R#	51.8	317	6.93E-86	NA	53.4	283	2.27E-75
4906	OSC56890.1	-	transcriptional regulator ArgP	1	1	1	1	1	1	1	1	1	1	1	11	CORE	COG0583_K#	49.8	262	2.03E-69	K05596	49.8	262	5.41E-69
4907	Prokka:JAPCLKMA_04883	-	Iron-sulfur cluster carrier protein	1	0	0	0	0	0	0	0	0	0	0	1	Unique	COG1192_D#	36	101	5.98E-21	K03496	32.5	93	4.35E-18
4908	OSC63760.1	-	transporter	1	1	1	1	1	1	1	1	1	1	1	11	CORE	COG0395_G#	39	202	2.49E-51	K10119	29.7	164	1.54E-39
4909	WP_033274279.1	-	universal stress protein	1	0	1	1	1	1	1	1	1	1	1	10	Accessory	COG0589_T#	37.1	168	5.2E-41	NA	34.8	152	7.89E-36
4910	OSC70971.1	-	4-(cytidine 5'-diphospho)-2-C-methyl-D-erythritol kinase	1	1	1	1	1	1	1	1	1	1	1	11	CORE	COG1947_I#	54.9	280	9.36E-75	K00919	54.9	280	2.5E-74
4911	WP_019526786.1	-	LysR family transcriptional regulator	1	1	1	1	1	1	1	1	1	1	1	11	CORE	COG0583_K#	36.6	141	3.98E-33	NA	35.7	136	4.46E-31
4912	WP_078504658.1	-	hypothetical protein	1	1	1	1	1	0	1	1	1	1	1	10	Accessory	COG2730_G#	48.3	94	7.28E-19	NA	36	86	5.3E-16
4913	OSC71088.1	-	NYN domain-containing protein	1	1	1	1	1	1	1	1	1	1	1	11	CORE	COG1432_S#	32.3	51	0.00000541				
4914	WP_122213573.1	-	hypothetical protein	0	0	0	0	0	0	0	0	0	1	0	1	Unique								

cluster	GI	gene	Annotation	Streptomyces_thermocarboxydus_BPSAC147	Streptomyces_sp._4F	Streptomyces_sp._BSE7F	Streptomyces_sp._ETH9427	Streptomyces_sp._FxanaD5	Streptomyces_sp._UNC401CLCol	Actinospica_acidiphila_NRRL_B_24431	Streptomyces_sp._HNS054	Streptomyces_sp._SMS_SU21	Streptomyces_sp._ZS0098	Streptomyces_thermocarboxydus_K155	Addition	Core-Accessory-Unique	COG	%Similarity_COG	Score_COG	Evalue_COG	KEGG	%Similarity_KEGG	Score_KEGG	Evalue_KEGG
4915	Prokka:JAPCLKMA_02110	-	hypothetical protein	1	1	1	1	1	1	1	1	1	1	1	11	CORE	COG1216_R#	28.2	89	1.79E-17	K16870	28.2	89	4.79E-17
4916	Prokka:CIODPCDJ_05692	-	hypothetical protein	0	0	0	0	0	0	0	0	0	0	1	1	Unique								
4917	Prokka:CIODPCDJ_06696	-	Fluoroacetate dehalogenase	0	0	0	0	1	1	0	0	0	0	1	3	Accessory	COG0596_R#	53.3	327	6.68E-89	NA	28.6	111	1.18E-23
4918	Prokka:JAPCLKMA_01491	-	hypothetical protein	1	0	0	1	0	0	0	0	0	0	0	2	Accessory	COG3586_S#	41.3	130	9.17E-30				
4919	WP_048455036.1	-	metal ABC transporter permease	0	1	1	1	1	1	1	1	1	1	1	10	Accessory	COG1108_P#	57	294	6.26E-79	K09819	43.1	205	1.02E-51
4920	OSC69323.1	-	sugar ABC transporter permease	0	1	0	0	0	0	0	0	0	0	0	1	Unique	COG0395_G#	36.6	203	1.11E-51	K10238	36.4	200	3.29E-50
4921	OSC69545.1	-	ABC transporter permease	1	1	1	1	1	1	1	1	1	1	1	11	CORE	COG1173_EP#	39.7	196	1.04E-49	K02034	38.7	192	5.25E-48
4922	WP_048458683.1	-	helix-turn-helix domain-containing protein	0	0	0	1	0	0	1	1	0	1	0	4	Accessory	COG1396_K#	41.6	210	6.97E-54				
4923	WP_102638262.1	-	hypothetical protein	1	1	1	1	1	1	1	1	1	1	1	11	CORE								
4924	Prokka:CIODPCDJ_06773	-	hypothetical protein	0	0	0	0	1	0	0	0	0	0	1	2	Accessory	COG0500_QR#	33	54	8.35E-07				
4925	Prokka:CIODPCDJ_03465	-	hypothetical protein	1	0	0	0	0	0	0	0	0	0	1	2	Accessory								

cluster	GI	gene	Annotation	Streptomyces_thermocarboxydus_BPSAC147	Streptomyces_sp._4F	Streptomyces_sp._BSE7F	Streptomyces_sp._ETH9427	Streptomyces_sp._FxanaD5	Streptomyces_sp._UNC401CLCol	Actinospica_acidiphila_NRRL_B_24431	Streptomyces_sp._HNS054	Streptomyces_sp._SMS_SU21	Streptomyces_sp._ZS0098	Streptomyces_thermocarboxydus_K155	Addition	Core-Accessory-Unique	COG	%Similarity_COG	Score_COG	Evalue_COG	KEGG	%Similarity_KEGG	Score_KEGG	Evalue_KEGG
4926	AXI85304.1	-	helix-turn-helix domain-containing protein	1	0	0	1	0	0	0	0	0	0	0	2	Accessory								
4927	WP_122214647.1	-	MerR family transcriptional regulator	1	1	1	1	1	1	1	1	1	1	1	11	CORE	COG0789_K#	50.5	95	3.27E-19	NA	43.4	195	8.1E-49
4928	Prokka:JAPCLKMA_06026	-	hypothetical protein	1	0	1	1	1	1	1	1	1	1	1	10	Accessory	COG2141_C#	50.7	250	1.04E-65	NA	50.7	250	2.77E-65
4929	OSC60512.1	-	dihydrodipicolinate synthase family protein	1	1	0	0	1	1	1	1	1	1	1	9	Accessory	COG0329_EM#	34.6	161	6.34E-39	K01714	26.3	78	1.44E-13
4930	Prokka:CIODPCDJ_06143	-	hypothetical protein	1	0	0	0	0	0	0	0	0	0	1	2	Accessory	COG3474_C#	33.7	41	0.0056	K18260	34.2	43	0.00514
4931	Prokka:CIODPCDJ_05386	-	hypothetical protein	0	0	0	0	0	0	0	0	0	0	1	1	Unique					NA	36.8	83	3.43E-15
4932	WP_033277677.1	-	ABC transporter ATP-binding protein	0	0	1	0	0	0	1	0	0	0	0	2	Accessory	COG1131_V#	32.3	131	4.12E-30	K01990	37.3	128	9.31E-29
4933	WP_026243824.1	pqqB	pyrroloquinoline quinone biosynthesis protein PqqB	0	0	0	0	1	1	0	0	0	0	0	2	Accessory	COG1235_R#	43	226	1.6E-58				

cluster	GI	gene	Annotation	Streptomyces_thermocarboxydus_BPSAC147	Streptomyces_sp._4F	Streptomyces_sp._BSE7F	Streptomyces_sp._ETH9427	Streptomyces_sp._FxanaD5	Streptomyces_sp._UNC401CLCol	Actinospica_acidiphila_NRRL_B_24431	Streptomyces_sp._HNS054	Streptomyces_sp._SMS_SU21	Streptomyces_sp._ZS0098	Streptomyces_thermocarboxydus_K155	Addition	Core-Accessory-Unique	COG	%Similarity_COG	Score_COG	Evalue_COG	KEGG	%Similarity_KEGG	Score_KEGG	Evalue_KEGG
4934	PWE10527.1	-	ABC transporter ATP-binding protein	0	0	1	1	0	0	0	0	1	1	0	4	Accessory	COG1131_V#	44.3	222	1.77E-57	K01990	39	178	1.02E-43
4935	OSC64047.1	-	sugar ABC transporter permease	0	1	0	0	0	0	0	0	0	0	0	1	Unique	COG1175_G#	35.2	208	4.52E-53	K02025	40.7	201	8.65E-51
4936	AXI88762.1	-	aldo/keto reductase	0	0	0	1	0	0	0	0	0	0	0	1	Unique								
4937	WP_106432424.1	-	helix-turn-helix domain-containing protein	1	0	1	1	1	1	1	1	1	1	1	10	Accessory	COG1396_K#	36	40	0.00956				
4938	OSC69821.1	-	sugar ABC transporter permease	1	1	1	1	1	1	1	1	1	1	1	11	CORE	COG0395_G#	50.2	305	1.59E-82	K02026	45.5	267	1.67E-70
4939	WP_048457868.1	-	NADP-dependent oxidoreductase	0	1	1	1	0	0	1	1	1	0	0	6	Accessory	COG0604_CR#	35.9	167	6.75E-41	K00344	32.8	129	4.16E-29
4940	OSC72855.1	-	short-chain dehydrogenase	1	1	1	1	1	1	1	1	1	1	1	11	CORE	COG1028_IQR#	43	198	4.66E-50	NA	43	198	1.24E-49
4941	Prokka:JAPCLKMA_00825	iolB	5-deoxy-glucuronate isomerase	1	1	1	1	1	1	1	1	1	1	1	11	CORE	COG3718_G#	46.6	242	1.65E-63	K03337	35	136	5.81E-31
4942	WP_122217329.1	-	aminoglycoside phosphotransferase family protein	1	0	1	0	1	1	1	1	1	1	1	9	Accessory	COG3173_R#	37.3	165	2.56E-40	NA	38.1	167	2.35E-40

cluster	GI	gene	Annotation	Streptomyces_thermocarboxydus_BPSAC147	Streptomyces_sp._4F	Streptomyces_sp._BSE7F	Streptomyces_sp._ETH9427	Streptomyces_sp._FxanaD5	Streptomyces_sp._UNC401CLCol	Actinospica_acidiphila_NRRL_B_24431	Streptomyces_sp._HNS054	Streptomyces_sp._SMS_SU21	Streptomyces_sp._ZS0098	Streptomyces_thermocarboxydus_K155	Addition	Core-Accessory-Unique	COG	%Similarity_COG	Score_COG	Evalue_COG	KEGG	%Similarity_KEGG	Score_KEGG	Evalue_KEGG
4943	Prokka:CIODPCDJ_02373	-	hypothetical protein	1	1	1	1	1	1	1	1	1	1	1	11	CORE								
4944	AXI88804.1	-	ABC transporter permease	1	1	1	1	1	1	1	1	1	1	1	11	CORE								
4945	AXI88763.1	-	hypothetical protein	0	0	0	1	0	0	0	0	0	0	0	1	Unique								
4946	WP_048460575.1	-	dehydrogenase	1	1	1	1	1	1	1	1	1	1	1	11	CORE	COG4630_F#	34.7	108	3.72E-23	K03519	27.7	80	3.78E-14
4947	Prokka:JAPCLKMA_04746	araD	L-2-keto-3-deoxyarabonate dehydratase	1	0	0	0	1	1	0	0	0	0	1	4	Accessory	COG0329_EM#	25	50	0.0000157				
4948	OSC68656.1	-	EamA family transporter	0	1	0	0	0	0	0	0	0	0	0	1	Unique	COG0697_GER#	36.7	157	9.12E-38				
4949	PWE10743.1	-	oxidoreductase	1	1	1	1	1	1	1	1	1	1	1	11	CORE	COG0673_R#	28.2	94	5.56E-19	NA	28.7	79	4.94E-14
4950	WP_033274462.1	-	metal-dependent hydrolase	1	1	1	1	1	1	1	1	1	1	1	11	CORE	COG3568_R#	26.9	74	1.02E-12				
4951	Prokka:JAPCLKMA_06783	araQ_9	L-arabinose transport system permease protein AraQ	1	0	0	0	1	1	0	0	0	0	1	4	Accessory	COG0395_G#	32.9	159	1.41E-38	K17236	32	148	1.13E-34
4952	Prokka:CIODPCDJ_02443	-	hypothetical protein	0	0	0	0	0	1	0	0	0	0	1	2	Accessory								
4953	OSC70545.1	-	hypothetical protein	0	1	0	0	0	0	0	0	0	0	0	1	Unique								

cluster	GI	gene	Annotation	Streptomyces_thermocarboxydus_BPSAC147	Streptomyces_sp._4F	Streptomyces_sp._BSE7F	Streptomyces_sp._ETH9427	Streptomyces_sp._FxanaD5	Streptomyces_sp._UNC401CLCol	Actinospica_acidiphila_NRRL_B_24431	Streptomyces_sp._HNS054	Streptomyces_sp._SMS_SU21	Streptomyces_sp._ZS0098	Streptomyces_thermocarboxydus_K155	Addition	Core-Accessory-Unique	COG	%Similarity_COG	Score_COG	Evalue_COG	KEGG	%Similarity_KEGG	Score_KEGG	Evalue_KEGG
4954	PWE09928.1	-	endonuclease/exonuclease/phosphatase family protein	1	0	1	0	0	0	0	0	1	0	0	3	Accessory								
4955	OSC61373.1	-	LysR family transcriptional regulator	0	1	0	0	0	0	0	0	0	0	0	1	Unique	COG0583_K#	35.3	145	3.59E-34	NA	37.1	136	4.45E-31
4956	Prokka:CIODPCDJ_05563	menH_7	2-succinyl-6-hydroxy-2, 4-cyclohexadiene-1-carboxylate synthase	1	0	1	1	1	1	1	1	1	1	1	10	Accessory	COG0596_R#	40.4	74	5.95E-13	NA	33.7	60	2.37E-08
4957	OSC70429.1	-	hypothetical protein	1	1	1	1	1	1	1	1	1	1	1	11	CORE	COG2064_NU#	28.7	94	5.56E-19				
4958	OSC64599.1	-	peptidase S15	0	1	0	0	0	0	0	0	0	0	0	1	Unique	COG2936_R#	52	347	3.64E-95				
4959	OSC64578.1	-	formyltetrahydrofolate deformylase	1	1	1	1	1	1	1	1	1	1	1	11	CORE	COG0788_F#	68.4	397	4.01E-110	K01433	47.9	268	7.5E-71
4960	OSC64738.1	-	sulfotransferase family protein	0	1	0	0	0	0	0	0	0	0	0	1	Unique								
4961	OSC66254.1	-	NADPH:quinone reductase	0	1	0	0	0	0	0	0	0	0	0	1	Unique	COG3246_S#	65.4	392	1.68E-108	NA	36.3	169	6.19E-41
4962	OSC72548.1	-	hypothetical protein	0	1	0	0	1	0	0	0	0	0	1	3	Accessory								
4963	AXI87278.1	-	serine hydrolase	1	1	1	1	1	1	1	1	1	1	1	11	CORE	COG2367_V#	26.7	55	2.85E-07	NA	26.7	55	7.61E-07

cluster	GI	gene	Annotation	Streptomyces_thermocarboxydus_BPSAC147	Streptomyces_sp._4F	Streptomyces_sp._BSE7F	Streptomyces_sp._ETH9427	Streptomyces_sp._FxanaD5	Streptomyces_sp._UNC401CLCol	Actinospica_acidiphila_NRRL_B_24431	Streptomyces_sp._HNS054	Streptomyces_sp._SMS_SU21	Streptomyces_sp._ZS0098	Streptomyces_thermocarboxydus_K155	Addition	Core-Accessory-Unique	COG	%Similarity_COG	Score_COG	Evalue_COG	KEGG	%Similarity_KEGG	Score_KEGG	Evalue_KEGG
4964	Prokka:CIODPCDJ_05459	btuD_23	Vitamin B12 import ATP-binding protein BtuD	1	1	0	0	1	1	1	0	0	0	1	6	Accessory	COG1116_P#	47.3	218	3.32E-56	K02049	44.2	210	2.42E-53
4965	OSC63015.1	-	dehydrogenase	0	1	0	0	0	0	0	0	0	0	0	1	Unique	COG2084_I#	43.2	114	8.82E-25	K18121	22.8	67	2.53E-10
4966	Prokka:CIODPCDJ_00965	-	hypothetical protein	1	1	1	1	1	1	1	1	1	1	1	11	CORE								
4967	WP_048455475.1	-	xanthine dehydrogenase family protein subunit M	1	1	1	1	1	1	1	1	1	1	1	11	CORE	COG1319_C#	34.9	153	1.71E-36	K13481	34.2	145	7.31E-34
4968	PWE10068.1	-	2-hydroxy-3-oxopropionate reductase	1	1	1	1	1	1	1	1	1	1	1	11	CORE	COG2084_I#	47.4	256	1.44E-67	K00042	47.8	255	8.55E-67
4969	WP_033272424.1	-	hypothetical protein	1	1	1	0	1	1	1	0	1	0	1	8	Accessory								
4970	PWE10256.1	-	hydrolase TatD	1	1	1	1	1	1	1	1	1	1	1	11	CORE	COG1099_R#	51.4	300	5.09E-81	K07051	27.7	110	2.6E-23
4971	Prokka:JAPCLKMA_05966	dagK_3	Diacylglycerol kinase	1	0	1	1	1	1	1	1	1	1	1	10	Accessory	COG1597_IR#	43.8	250	7.89E-66	K07029	43.8	250	2.11E-65
4972	Prokka:JAPCLKMA_05564	-	hypothetical protein	1	1	0	1	1	1	1	1	1	1	1	10	Accessory								
4973	PWE08822.1	-	hypothetical protein	1	0	1	1	0	0	0	1	1	1	0	6	Accessory								
4974	WP_028959857.1	-	ThiF family adenylyltransferase	1	0	0	0	1	1	0	0	0	0	1	4	Accessory	COG0476_H#	36.9	91	8E-18	NA	33.6	94	1.93E-18

cluster	GI	gene	Annotation	Streptomyces_thermocarboxydus_BPSAC147	Streptomyces_sp._4F	Streptomyces_sp._BSE7F	Streptomyces_sp._ETH9427	Streptomyces_sp._FxanaD5	Streptomyces_sp._UNC401CLCol	Actinospica_acidiphila_NRRL_B_24431	Streptomyces_sp._HNS054	Streptomyces_sp._SMS_SU21	Streptomyces_sp._ZS0098	Streptomyces_thermocarboxydus_K155	Addition	Core-Accessory-Unique	COG	%Similarity_COG	Score_COG	Evalue_COG	KEGG	%Similarity_KEGG	Score_KEGG	Evalue_KEGG
4975	WP_048458740.1	-	class I SAM-dependent methyltransferase	1	0	0	1	1	1	1	1	1	1	1	9	Accessory	COG2226_H#	25.5	54	6.35E-07	K13210	34.9	47	0.000207
4976	WP_028961653.1	-	alpha/beta hydrolase	1	0	1	1	1	1	0	1	1	1	0	8	Accessory	COG0596_R#	36.9	155	2.64E-37	NA	33.7	122	6.62E-27
4977	WP_033272491.1	-	hypothetical protein	0	0	0	0	0	0	1	0	0	0	0	1	Unique								
4978	WP_053076973.1	-	hypothetical protein	0	0	0	0	0	0	0	1	0	0	0	1	Unique								
4979	OSC56474.1	-	DUF3558 domain-containing protein	1	1	1	1	1	1	1	1	1	1	1	11	CORE	COG2730_G#	47.2	48	0.0000595	NA	36.1	53	0.00000494
4980	OSC56661.1	-	1-acyl-sn-glycerol-3-phosphate acyltransferase	0	1	0	0	0	0	0	0	0	0	0	1	Unique	COG0204_I#	38.9	167	8.78E-41	NA	38.9	167	2.35E-40
4981	OSC65973.1	-	F420-dependent oxidoreductase	0	1	0	0	0	0	0	0	0	0	0	1	Unique	COG2141_C#	37.6	175	2.47E-43	NA	37.6	175	6.59E-43
4982	OSC62248.1	-	NAD(P)-dependent dehydrogenase	1	1	1	1	1	1	1	1	1	1	1	11	CORE	COG1028_IQR#	56.8	318	3.08E-86	K00540	50.7	294	9.74E-79
4983	AXI84666.1	-	hypothetical protein	0	0	0	1	0	0	0	1	1	1	0	4	Accessory	COG2091_H#	31	55	3.72E-07	K06133	31	55	9.94E-07
4984	AXI91293.1	-	hypothetical protein	0	0	0	1	0	0	0	0	0	0	0	1	Unique	COG1595_K#	34.4	44	0.00112	NA	29.5	49	0.0000713
4985	Prokka:CIODPCDJ_03323	glpG	Rhomboid protease GlpG	1	1	1	1	1	1	1	1	1	1	1	11	CORE	COG0705_R#	48.4	238	2.37E-62	NA	46.4	195	6.14E-49

cluster	GI	gene	Annotation	Streptomyces_thermocarboxydus_BPSAC147	Streptomyces_sp._4F	Streptomyces_sp._BSE7F	Streptomyces_sp._ETH9427	Streptomyces_sp._FxanaD5	Streptomyces_sp._UNC401CLCol	Actinospica_acidiphila_NRRL_B_24431	Streptomyces_sp._HNS054	Streptomyces_sp._SMS_SU21	Streptomyces_sp._ZS0098	Streptomyces_thermocarboxydus_K155	Addition	Core-Accessory-Unique	COG	%Similarity_COG	Score_COG	Evalue_COG	KEGG	%Similarity_KEGG	Score_KEGG	Evalue_KEGG
4986	OSC56373.1	-	hypothetical protein	0	1	0	0	0	0	0	0	0	0	0	1	Unique	COG0702_MG#	30.8	88	3.96E-17	NA	34.3	49	0.000071
4987	Prokka:CIODPCDJ_03072	btuD_13	Vitamin B12 import ATP-binding protein BtuD	1	0	0	1	1	1	1	0	1	1	1	8	Accessory	COG1131_V#	34.8	145	3.56E-34	K01990	34.2	134	1.29E-30
4988	WP_102637068.1	-	cytochrome c biogenesis protein CcdA	0	1	1	1	0	0	0	1	1	1	0	6	Accessory	COG0785_O#	39.5	180	7.65E-45				
4989	OSC59900.1	-	deoxyribose-phosphate aldolase	1	1	1	1	1	1	1	1	1	1	1	11	CORE								
4990	WP_122212804.1	-	RNA polymerase sigma-70 factor	1	1	1	0	1	1	1	1	1	1	1	10	Accessory	COG1595_K#	45	227	7.13E-59	K03088	39.2	188	1.28E-46
4991	AXI90489.1	-	sugar ABC transporter permease	0	0	0	1	0	0	0	1	1	0	0	3	Accessory	COG1175_G#	47.2	276	1.02E-73	K10118	47.2	276	2.74E-73
4992	AXI88181.1	-	DNA primase	0	1	0	1	1	0	1	0	0	0	0	4	Accessory								
4993	WP_157842192.1	-	hypothetical protein	0	0	0	0	0	0	1	0	0	0	0	1	Unique								
4994	OSC72543.1	-	ATP/GTP-binding protein	0	1	0	0	1	0	0	0	0	0	1	3	Accessory								
4995	WP_019525515.1	-	LysR family transcriptional regulator	0	0	0	0	1	1	0	0	0	0	1	3	Accessory	COG0583_K#	30.3	112	2.56E-24	K11921	27.5	110	3.39E-23

cluster	GI	gene	Annotation	Streptomyces_thermocarboxydus_BPSAC147	Streptomyces_sp._4F	Streptomyces_sp._BSE7F	Streptomyces_sp._ETH9427	Streptomyces_sp._FxanaD5	Streptomyces_sp._UNC401CLCol	Actinospica_acidiphila_NRRL_B_24431	Streptomyces_sp._HNS054	Streptomyces_sp._SMS_SU21	Streptomyces_sp._ZS0098	Streptomyces_thermocarboxydus_K155	Addition	Core-Accessory-Unique	COG	%Similarity_COG	Score_COG	Evalue_COG	KEGG	%Similarity_KEGG	Score_KEGG	Evalue_KEGG
4996	Prokka:CIODPCDJ_00459	sigF_3	RNA polymerase sigma factor SigF	1	1	1	1	1	1	1	1	1	1	1	11	CORE	COG1191_K#	39.6	190	9.65E-48	K03090	39.6	190	2.58E-47
4997	OSC65055.1	-	N-acetyl-1-D-myo-inositol-2-amino-2-deoxy-alpha-D-glucopyranoside deacetylase	1	1	1	1	1	1	1	1	1	1	1	11	CORE	COG2120_S#	43.8	210	1.18E-53	K15525	43.8	210	3.14E-53
4998	WP_122213947.1	-	universal stress protein	0	0	1	0	0	0	0	0	1	1	0	3	Accessory	COG0589_T#	31.6	116	1.77E-25	NA	31.6	116	4.73E-25
4999	Prokka:CIODPCDJ_03468	-	hypothetical protein	1	0	0	0	0	0	0	0	0	0	1	2	Accessory								
5000	OSC63821.1	-	fructosamine kinase	1	1	1	0	1	1	1	1	1	1	1	10	Accessory	COG3001_G#	44.7	187	6.26E-47	NA	35	119	7.3E-26
5001	OSC66687.1	-	AraC family transcriptional regulator	0	1	0	1	1	1	1	1	1	1	1	9	Accessory	COG3708_S#	35.4	112	2.56E-24	K13653	36.7	213	2.18E-54
5002	WP_033275361.1	-	aldo/keto reductase	1	0	1	1	1	1	1	1	1	1	1	10	Accessory	COG0667_C#	43.7	223	1.03E-57	NA	37.1	176	3.85E-43
5003	Prokka:JAPCLKMA_01573	-	hypothetical protein	1	0	0	0	0	0	0	0	0	0	0	1	Unique	COG1396_K#	44.6	49	0.0000204	NA	44.6	49	0.0000544
5004	OSC73541.1	-	GntR family transcriptional regulator	1	1	1	1	1	1	1	1	1	1	1	11	CORE	COG2186_K#	36.7	139	1.5E-32	NA	31.7	79	4.91E-14
5005	OSC57083.1	-	nitrate reductase	0	1	0	0	0	0	0	0	0	0	0	1	Unique	COG1457_F#	29.6	109	2.83E-23	K03457	28	109	4.43E-23

cluster	GI	gene	Annotation	Streptomyces_thermocarboxydus_BPSAC147	Streptomyces_sp._4F	Streptomyces_sp._BSE7F	Streptomyces_sp._ETH9427	Streptomyces_sp._FxanaD5	Streptomyces_sp._UNC401CLCol	Actinospica_acidiphila_NRRL_B_24431	Streptomyces_sp._HNS054	Streptomyces_sp._SMS_SU21	Streptomyces_sp._ZS0098	Streptomyces_thermocarboxydus_K155	Addition	Core-Accessory-Unique	COG	%Similarity_COG	Score_COG	Evalue_COG	KEGG	%Similarity_KEGG	Score_KEGG	Evalue_KEGG
5006	OSC66761.1	-	transcriptional regulator	1	1	1	1	1	1	0	1	1	1	1	10	Accessory	COG1396_K#	33.3	40	0.00946				
5007	WP_048455801.1	-	helix-turn-helix domain-containing protein	1	1	1	1	1	1	1	1	1	1	1	11	CORE								
5008	WP_048458736.1	-	alpha/beta hydrolase	0	0	1	1	0	0	0	1	1	1	0	5	Accessory	COG0596_R#	44.3	244	5.61E-64	NA	44.3	244	1.5E-63
5009	WP_102640488.1	-	phosphatase PAP2 family protein	1	1	1	1	1	1	1	1	1	1	1	11	CORE								
5010	WP_019521483.1	-	alpha/beta fold hydrolase	0	0	0	0	1	1	1	1	0	1	1	6	Accessory	COG0596_R#	31.9	102	2.02E-21	K16050	31.9	102	5.39E-21
5011	WP_122214816.1	-	alpha/beta fold hydrolase	1	1	1	1	1	1	1	1	1	1	1	11	CORE	COG1073_R#	32	68	4.22E-11	K06889	32	68	1.13E-10
5012	AXI89467.1	-	lysozyme	1	0	1	1	1	1	1	1	1	1	1	10	Accessory	COG3757_M#	31.3	135	2.81E-31	K07273	33.2	112	8.89E-24
5013	OSC61677.1	-	transcriptional regulator	0	1	1	1	0	0	1	1	1	1	0	7	Accessory	COG1396_K#	39.2	167	6.68E-41				
5014	WP_033273580.1	-	carbohydrate ABC transporter permease	1	0	0	0	1	1	1	0	1	1	1	7	Accessory	COG0395_G#	36.4	180	7.62E-45	K02026	36.4	178	7.74E-44
5015	WP_102640786.1	urtB	urea ABC transporter permease subunit UrtB	1	0	1	1	1	1	1	1	1	1	1	10	Accessory	COG0559_E#	61.9	363	8.29E-100	K11960	51	288	9.05E-77

cluster	GI	gene	Annotation	Streptomyces_thermocarboxydus_BPSAC147	Streptomyces_sp._4F	Streptomyces_sp._BSE7F	Streptomyces_sp._ETH9427	Streptomyces_sp._FxanaD5	Streptomyces_sp._UNC401CLCol	Actinospica_acidiphila_NRRL_B_24431	Streptomyces_sp._HNS054	Streptomyces_sp._SMS_SU21	Streptomyces_sp._ZS0098	Streptomyces_thermocarboxydus_K155	Addition	Core-Accessory-Unique	COG	%Similarity_COG	Score_COG	Evalue_COG	KEGG	%Similarity_KEGG	Score_KEGG	Evalue_KEGG
5016	WP_033275896.1	mutM	bifunctional DNA-formamidopyrimidine glycosylase/DNA-(apurinic or apyrimidinic site) lyase	1	1	1	1	1	1	1	1	1	1	1	11	CORE	COG0266_L#	57.1	308	2.42E-83	K10563	56.3	304	9.35E-82
5017	OSC51529.1	-	2-nitropropane dioxygenase	0	1	0	0	0	0	0	0	0	0	0	1	Unique	COG2070_R#	55.7	329	1.33E-89	K15329	45.5	249	4.66E-65
5018	Prokka:CIODPCDJ_03899	-	hypothetical protein	1	1	1	1	1	1	1	1	1	1	1	11	CORE	COG1277_R#	27.1	72	3.82E-12	K01992	23.3	65	9.54E-10
5019	OSC61267.1	-	hypothetical protein	0	1	0	0	0	0	0	0	0	0	0	1	Unique								
5020	Prokka:CIODPCDJ_00152	-	hypothetical protein	1	0	0	0	1	1	1	0	0	1	1	6	Accessory	COG0395_G#	44.1	247	5.08E-65	K17246	36.7	206	3.46E-52
5021	PWE07242.1	-	transcriptional regulator	0	0	1	0	0	0	0	0	0	0	0	1	Unique	COG1396_K#	35.6	41	0.00722				
5022	Prokka:CIODPCDJ_04390	-	hypothetical protein	1	1	1	0	1	1	1	1	1	1	1	10	Accessory	COG2912_S#	29.3	97	8.49E-20	K10301	23.7	57	1.99E-07
5023	Prokka:JAPCLKMA_02406	-	hypothetical protein	1	1	1	1	1	1	1	1	1	1	1	11	CORE								
5024	Prokka:JAPCLKMA_00288	cdhB	Caffeine dehydrogenase subunit beta	1	0	0	0	1	1	0	0	0	0	1	4	Accessory	COG1319_C#	37.6	197	6.03E-50	K03519	37.6	197	1.61E-49

cluster	GI	gene	Annotation	Streptomyces_thermocarboxydus_BPSAC147	Streptomyces_sp._4F	Streptomyces_sp._BSE7F	Streptomyces_sp._ETH9427	Streptomyces_sp._FxanaD5	Streptomyces_sp._UNC401CLCol	Actinospica_acidiphila_NRRL_B_24431	Streptomyces_sp._HNS054	Streptomyces_sp._SMS_SU21	Streptomyces_sp._ZS0098	Streptomyces_thermocarboxydus_K155	Addition	Core-Accessory-Unique	COG	%Similarity_COG	Score_COG	Evalue_COG	KEGG	%Similarity_KEGG	Score_KEGG	Evalue_KEGG
5025	WP_019521684.1	-	ABC transporter permease	0	0	0	0	1	1	0	0	0	0	1	3	Accessory	COG1176_E#	45.4	251	4.59E-66	K02054	44.8	248	6.09E-65
5026	AXI89078.1	pgsA	CDP-diacylglycerol--glycerol-3-phosphate 3-phosphatidyltransferase	1	0	1	1	1	1	1	1	1	1	1	10	Accessory	COG0558_I#	56.9	206	9.93E-53	K00995	56.9	206	2.65E-52
5027	OSC62385.1	-	GNAT family N-acetyltransferase	1	1	1	1	1	1	1	1	1	1	1	11	CORE	COG0251_J#	52	121	7.17E-27	NA	38.4	74	1.57E-12
5028	WP_033278163.1	-	hypothetical protein	1	0	0	1	1	0	1	0	0	0	0	4	Accessory								
5029	Prokka:JAPCLKMA_06333	-	IS3 family transposase ISPmi1	1	0	1	0	0	0	0	1	0	0	0	3	Accessory	COG2801_L#	48.8	285	2.87E-76	NA	48.5	284	1E-75
5030	Prokka:JAPCLKMA_02153	-	Universal stress protein	1	1	1	1	1	1	0	1	1	1	1	10	Accessory	COG0589_T#	34.6	147	9.34E-35	NA	34.6	121	1.47E-26
5031	OSC69810.1	-	LysR family transcriptional regulator	1	1	1	0	1	1	1	1	1	1	1	10	Accessory	COG0583_K#	51.6	258	2.88E-68	NA	30.5	130	2.42E-29
5032	OSC72556.1	-	sugar ABC transporter permease	1	1	1	1	1	1	1	1	1	1	1	11	CORE	COG0395_G#	42.2	206	1.69E-52	K10238	30.4	123	2.95E-27
5033	OSC69639.1	-	oxidoreductase	0	1	0	0	0	0	0	0	0	0	0	1	Unique	COG1028_IQR#	41.3	180	7.62E-45	K00540	35.5	139	3.98E-32

cluster	GI	gene	Annotation	Streptomyces_thermocarboxydus_BPSAC147	Streptomyces_sp._4F	Streptomyces_sp._BSE7F	Streptomyces_sp._ETH9427	Streptomyces_sp._FxanaD5	Streptomyces_sp._UNC401CLCol	Actinospica_acidiphila_NRRL_B_24431	Streptomyces_sp._HNS054	Streptomyces_sp._SMS_SU21	Streptomyces_sp._ZS0098	Streptomyces_thermocarboxydus_K155	Addition	Core-Accessory-Unique	COG	%Similarity_COG	Score_COG	Evalue_COG	KEGG	%Similarity_KEGG	Score_KEGG	Evalue_KEGG
5034	WP_086700673.1	gnd	decarboxylating 6-phosphogluconate dehydrogenase	1	1	1	1	1	1	1	1	1	1	1	11	CORE	COG1023_G#	48.2	289	1.52E-77	K00033	48.2	289	4.05E-77
5035	WP_122216633.1	-	universal stress protein	1	0	0	1	1	1	1	1	1	1	1	9	Accessory	COG0589_T#	32.1	126	1.7E-28	NA	33	116	4.7E-25
5036	Prokka:JAPCLKMA_03792	srkA	Stress response kinase A	1	0	0	0	0	0	0	0	0	0	1	2	Accessory								
5037	WP_019526456.1	-	hypothetical protein	1	1	1	1	1	1	1	1	1	1	1	11	CORE								
5038	Prokka:JAPCLKMA_02395	dagK_1	Diacylglycerol kinase	1	1	1	1	1	1	1	1	1	1	1	11	CORE	COG1597_IR#	34.2	143	1.34E-33	K07029	34.2	143	3.59E-33
5039	WP_048455912.1	mca	mycothiol conjugate amidase Mca	1	1	1	1	1	1	1	1	1	1	1	11	CORE	COG2120_S#	59	348	2.75E-95	K18455	51.9	300	2.29E-80
5040	Prokka:CIODPCDJ_03850	-	hypothetical protein	1	1	1	1	1	1	1	1	1	1	1	11	CORE								
5041	WP_122216473.1	-	Gfo/Idh/MocA family oxidoreductase	1	0	0	0	1	1	1	0	1	1	1	7	Accessory	COG0673_R#	31.8	72	2.91E-12				
5042	OSC62358.1	-	NAD(P)-dependent oxidoreductase	0	1	0	0	0	0	0	0	0	0	0	1	Unique	COG1028_IQR#	52.9	279	1.2E-74	NA	46.9	258	5.86E-68
5043	WP_102637988.1	-	winged helix-turn-helix transcriptional regulator	0	0	0	0	0	0	0	0	1	0	0	1	Unique	COG1167_KE#	36.9	66	2.09E-10	K00375	38	56	4.42E-07

cluster	GI	gene	Annotation	Streptomyces_thermocarboxydus_BPSAC147	Streptomyces_sp._4F	Streptomyces_sp._BSE7F	Streptomyces_sp._ETH9427	Streptomyces_sp._FxanaD5	Streptomyces_sp._UNC401CLCol	Actinospica_acidiphila_NRRL_B_24431	Streptomyces_sp._HNS054	Streptomyces_sp._SMS_SU21	Streptomyces_sp._ZS0098	Streptomyces_thermocarboxydus_K155	Addition	Core-Accessory-Unique	COG	%Similarity_COG	Score_COG	Evalue_COG	KEGG	%Similarity_KEGG	Score_KEGG	Evalue_KEGG
5044	WP_048456045.1	-	carbohydrate ABC transporter permease	0	0	1	1	1	1	1	1	1	1	1	9	Accessory	COG0395_G#	42.8	245	3.28E-64	K17320	42.8	245	8.76E-64
5045	WP_048455646.1	-	ATP-dependent DNA ligase	1	1	0	1	1	1	1	1	1	1	1	10	Accessory	COG3285_L#	42.6	215	3.64E-55	K01971	42.6	215	9.71E-55
5046	WP_019524889.1	-	barstar family protein	0	0	0	0	1	0	0	0	0	0	0	1	Unique	COG2732_K#	42.9	41	0.00551				
5047	WP_122213408.1	-	arylamine N-acetyltransferase	0	0	1	1	0	0	0	1	1	1	1	6	Accessory	COG2162_Q#	34.6	143	1.76E-33	K00622	32.8	114	1.79E-24
5048	Prokka:JAPCLKMA_01648	-	hypothetical protein	1	0	0	0	0	0	0	0	0	0	0	1	Unique								
5049	WP_019522430.1	-	glycerophosphodiester phosphodiesterase	1	0	0	0	1	1	1	0	0	0	1	5	Accessory	COG0584_C#	37.5	64	6.08E-10				
5050	OSC72105.1	-	dehydrogenase	0	1	0	0	0	0	0	0	0	0	0	1	Unique	COG2084_I#	55.4	154	7.61E-37	K18121	25.3	73	4.56E-12
5051	PWE10879.1	-	endonuclease	1	1	1	1	1	1	1	1	1	1	1	11	CORE								
5052	Prokka:JAPCLKMA_04889	-	hypothetical protein	1	0	0	0	0	0	0	0	0	0	0	1	Unique								
5053	OSC63689.1	-	DedA family protein	1	1	1	1	1	1	1	1	1	1	1	11	CORE	COG0586_S#	46.4	183	8.97E-46	NA	46.2	178	1.01E-43
5054	AXI87173.1	-	hypothetical protein	0	0	0	1	0	0	0	1	0	0	0	2	Accessory								
5055	Prokka:JAPCLKMA_06542	-	hypothetical protein	1	0	0	0	0	0	0	0	0	0	0	1	Unique								

cluster	GI	gene	Annotation	Streptomyces_thermocarboxydus_BPSAC147	Streptomyces_sp._4F	Streptomyces_sp._BSE7F	Streptomyces_sp._ETH9427	Streptomyces_sp._FxanaD5	Streptomyces_sp._UNC401CLCol	Actinospica_acidiphila_NRRL_B_24431	Streptomyces_sp._HNS054	Streptomyces_sp._SMS_SU21	Streptomyces_sp._ZS0098	Streptomyces_thermocarboxydus_K155	Addition	Core-Accessory-Unique	COG	%Similarity_COG	Score_COG	Evalue_COG	KEGG	%Similarity_KEGG	Score_KEGG	Evalue_KEGG
5056	WP_028961814.1	-	hypothetical protein	1	0	1	1	1	1	0	0	1	1	1	8	Accessory					NA	41.2	52	0.00000833
5057	OSC52338.1	-	hypothetical protein	0	1	0	0	0	0	0	0	0	0	0	1	Unique								
5058	OSC57266.1	-	hypothetical protein	0	1	0	0	0	0	0	0	0	0	0	1	Unique	COG2353_S#	29.8	109	1.65E-23	NA	32.4	89	6.15E-17
5059	OSC55978.1	-	short-chain dehydrogenase	1	1	1	1	1	1	1	1	1	1	1	11	CORE	COG1028_IQR#	38.6	127	5.84E-29	NA	40.1	165	6.75E-40
5060	WP_122215170.1	-	alpha/beta hydrolase	1	1	1	1	1	1	1	1	1	1	1	11	CORE	COG0596_R#	45.8	233	7.54E-61	NA	31.5	115	7.99E-25
5061	Prokka:CIODPCDJ_00703	-	hypothetical protein	1	0	0	0	1	1	0	0	0	0	1	4	Accessory	COG0388_R#	55.5	338	2.84E-92	NA	35.9	179	3.45E-44
5062	Prokka:CIODPCDJ_00986	pxpC_1	5-oxoprolinase subunit C	1	1	1	1	1	1	1	1	1	1	1	11	CORE	COG1984_E#	46.7	228	2.42E-59	NA	46.7	228	6.47E-59
5063	AXI86305.1	-	deoxyribonuclease IV	1	1	1	1	1	1	1	1	1	1	1	11	CORE	COG0648_L#	37.2	169	1.75E-41	K01151	35.9	168	1.36E-40
5064	OSC52294.1	-	arabinan endo-1,5-alpha-L-arabinosidase	0	1	0	0	0	0	1	0	0	0	0	2	Accessory	COG3507_G#	43.4	233	7.54E-61	K06113	45.4	258	7.63E-68
5065	Prokka:JAPCLKMA_02192	-	Lipase 2	1	1	1	1	1	1	1	1	1	1	1	11	CORE								
5066	PWE09387.1	-	methyltransferase type 11	0	0	1	1	0	0	0	1	1	1	0	5	Accessory	COG0500_QR#	41.2	169	1.75E-41	NA	26.7	62	1.05E-08

cluster	GI	gene	Annotation	Streptomyces_thermocarboxydus_BPSAC147	Streptomyces_sp._4F	Streptomyces_sp._BSE7F	Streptomyces_sp._ETH9427	Streptomyces_sp._FxanaD5	Streptomyces_sp._UNC401CLCol	Actinospica_acidiphila_NRRL_B_24431	Streptomyces_sp._HNS054	Streptomyces_sp._SMS_SU21	Streptomyces_sp._ZS0098	Streptomyces_thermocarboxydus_K155	Addition	Core-Accessory-Unique	COG	%Similarity_COG	Score_COG	Evalue_COG	KEGG	%Similarity_KEGG	Score_KEGG	Evalue_KEGG
5067	Prokka:CIODPCDJ_02855	-	2-(acetamidomethylene)succinate hydrolase	1	1	1	1	1	1	1	1	1	1	1	11	CORE	COG0596_R#	33.1	124	8.41E-28	NA	33.1	124	2.25E-27
5068	WP_122215620.1	-	LLM class flavin-dependent oxidoreductase	1	1	1	1	1	1	1	1	1	1	1	11	CORE	COG2141_C#	28.1	91	6.04E-18	K00320	28.1	91	1.61E-17
5069	AXI90565.1	uppS	di-trans,poly-cis-decaprenylcistransferase	0	1	0	1	0	0	1	1	1	1	0	6	Accessory	COG0020_I#	46.8	211	5.23E-54	K14215	47.4	210	3.11E-53
5070	PWE12260.1	-	phenazine biosynthesis protein PhzF	1	1	1	1	1	1	1	1	1	1	1	11	CORE	COG0384_R#	39.8	198	4.58E-50	NA	32.9	119	5.53E-26
5071	OSC63168.1	-	hypothetical protein	0	1	0	0	0	0	0	0	0	0	0	1	Unique								
5072	WP_028961060.1	-	glutathione S-transferase	1	0	0	0	1	1	1	0	0	0	1	5	Accessory	COG0435_O#	30	104	9.01E-22	K07393	25.9	90	3.59E-17
5073	OSC58756.1	-	hydrolase	0	1	0	0	0	0	0	0	0	0	0	1	Unique	COG0596_R#	32.5	156	1.53E-37	NA	31.1	103	3.14E-21
5074	WP_033276916.1	-	undecaprenyl-diphosphatase phosphatase	1	1	1	1	1	1	1	1	1	1	1	11	CORE	COG1968_V#	55.7	303	1.01E-81	K06153	46.2	236	2.38E-61
5075	WP_037778106.1	-	phosphotransferase	1	0	0	0	1	1	1	0	0	0	0	4	Accessory								

cluster	GI	gene	Annotation	Streptomyces_thermocarboxydus_BPSAC147	Streptomyces_sp._4F	Streptomyces_sp._BSE7F	Streptomyces_sp._ETH9427	Streptomyces_sp._FxanaD5	Streptomyces_sp._UNC401CLCol	Actinospica_acidiphila_NRRL_B_24431	Streptomyces_sp._HNS054	Streptomyces_sp._SMS_SU21	Streptomyces_sp._ZS0098	Streptomyces_thermocarboxydus_K155	Addition	Core-Accessory-Unique	COG	%Similarity_COG	Score_COG	Evalue_COG	KEGG	%Similarity_KEGG	Score_KEGG	Evalue_KEGG
5076	WP_019525194.1	tesB	acyl-CoA thioesterase II	1	1	1	1	1	1	1	1	1	1	1	11	CORE	COG1946_I#	50.7	277	7.76E-74	K10805	48.3	258	9.97E-68
5077	OSC72410.1	-	hypothetical protein	1	1	1	0	1	1	1	1	1	1	1	10	Accessory	COG1801_S#	32.9	146	1.58E-34	NA	29.7	132	6.31E-30
5078	OSC70929.1	-	hypothetical protein	0	1	0	0	0	0	0	0	0	0	0	1	Unique								
5079	WP_122214734.1	-	alpha/beta fold hydrolase	0	1	1	1	0	0	1	1	1	1	0	7	Accessory					NA	50	42	0.00856
5080	WP_051101666.1	-	hypothetical protein	1	0	1	0	1	0	0	0	1	0	1	5	Accessory								
5081	WP_033273900.1	-	glycosyltransferase	1	0	1	0	1	1	1	1	1	1	1	9	Accessory	COG1216_R#	25.6	52	0.0000031				
5082	OSC60608.1	-	3-methyl-2-oxobutanoate hydroxymethyltransferase	1	1	1	1	1	1	1	1	1	1	1	11	CORE	COG0413_H#	59.1	309	1.41E-83	K00606	59.1	309	3.76E-83
5083	OSC64529.1	-	LLM class F420-dependent oxidoreductase	0	1	0	0	0	0	0	0	0	0	0	1	Unique	COG2141_C#	47.2	232	1.67E-60	NA	47.2	232	4.47E-60
5084	WP_028959438.1	-	hypothetical protein	1	1	1	1	1	1	1	1	1	1	1	11	CORE					NA	53.7	279	3.19E-74
5085	OSC69299.1	-	ABC transporter permease	1	1	0	0	1	1	1	1	0	1	1	8	Accessory	COG0395_G#	36.6	171	3.5E-42	K17246	31	154	1.55E-36
5086	OSC70559.1	-	transcriptional regulator	0	1	0	0	0	0	0	0	0	0	0	1	Unique	COG1396_K#	40.1	181	5.78E-45				
5087	OSC56635.1	-	oxidoreductase	0	1	0	0	0	0	0	0	0	0	0	1	Unique								

cluster	GI	gene	Annotation	Streptomyces_thermocarboxydus_BPSAC147	Streptomyces_sp._4F	Streptomyces_sp._BSE7F	Streptomyces_sp._ETH9427	Streptomyces_sp._FxanaD5	Streptomyces_sp._UNC401CLCol	Actinospica_acidiphila_NRRL_B_24431	Streptomyces_sp._HNS054	Streptomyces_sp._SMS_SU21	Streptomyces_sp._ZS0098	Streptomyces_thermocarboxydus_K155	Addition	Core-Accessory-Unique	COG	%Similarity_COG	Score_COG	Evalue_COG	KEGG	%Similarity_KEGG	Score_KEGG	Evalue_KEGG
5088	Prokka:JAPCLKMA_06541	-	hypothetical protein	1	0	0	0	0	0	0	0	0	0	0	1	Unique								
5089	AXI85377.1	-	hypothetical protein	0	0	0	1	0	0	0	0	0	0	0	1	Unique								
5090	Prokka:JAPCLKMA_06318	-	hypothetical protein	1	0	0	1	0	0	0	0	1	0	0	3	Accessory	COG3293_L#	40.6	225	3.49E-58	K07492	37	171	1.22E-41
5091	Prokka:CIODPCDJ_04256	-	hypothetical protein	0	0	0	0	0	0	0	0	0	0	1	1	Unique	COG0842_V#	26.7	64	1.03E-09	K01992	26.7	64	2.75E-09
5092	Prokka:JAPCLKMA_01578	-	hypothetical protein	1	0	0	0	0	0	0	0	0	0	0	1	Unique								
5093	OSC61848.1	-	hypothetical protein	0	1	0	0	0	0	0	0	0	0	0	1	Unique					NA	25.3	82	9.75E-15
5094	OSC72459.1	-	transcription termination/antitermination factor NusG	1	1	1	1	1	1	1	1	1	1	1	11	CORE	COG0250_K#	57.1	255	1.85E-67	K02601	57.1	255	4.93E-67
5095	WP_048458861.1	-	hypothetical protein	0	0	0	0	0	0	0	1	1	0	0	2	Accessory								
5096	OSC52778.1	-	beta-galactosidase	0	1	0	0	0	0	0	0	0	0	0	1	Unique	COG3250_G#	40.7	225	2.67E-58	K01190	44.6	262	5.27E-69
5097	OSC65729.1	-	RNA polymerase subunit sigma-70	0	1	0	0	0	0	0	0	0	0	0	1	Unique	COG1595_K#	38.8	157	8.93E-38	K03088	38.8	157	2.39E-37
5098	Prokka:JAPCLKMA_03531	dasC_8	Diacetylchitobiose uptake system permease protein DasC	1	1	1	1	1	1	1	1	1	1	1	11	CORE	COG0395_G#	39.8	191	4.27E-48	K10238	39.8	191	1.14E-47

cluster	GI	gene	Annotation	Streptomyces_thermocarboxydus_BPSAC147	Streptomyces_sp._4F	Streptomyces_sp._BSE7F	Streptomyces_sp._ETH9427	Streptomyces_sp._FxanaD5	Streptomyces_sp._UNC401CLCol	Actinospica_acidiphila_NRRL_B_24431	Streptomyces_sp._HNS054	Streptomyces_sp._SMS_SU21	Streptomyces_sp._ZS0098	Streptomyces_thermocarboxydus_K155	Addition	Core-Accessory-Unique	COG	%Similarity_COG	Score_COG	Evalue_COG	KEGG	%Similarity_KEGG	Score_KEGG	Evalue_KEGG
5099	OSC72337.1	-	hypothetical protein	0	1	0	0	0	0	0	0	0	0	0	1	Unique	COG4312_S#	43.8	165	3.28E-40				
5100	Prokka:CIODPCDJ_03597	-	hypothetical protein	0	0	0	0	0	0	0	0	0	0	1	1	Unique								
5101	Prokka:CIODPCDJ_02701	-	hypothetical protein	1	0	1	1	1	1	1	1	1	1	1	10	Accessory								
5102	OSC73270.1	-	hypothetical protein	0	1	0	0	0	0	0	0	0	0	0	1	Unique								
5103	WP_026243870.1	-	siderophore-interacting protein	1	1	1	1	1	1	1	1	1	1	1	11	CORE	COG2375_P#	33.4	143	1.02E-33	NA	38.1	130	3.11E-29
5104	WP_106432465.1	-	cytochrome c oxidase assembly protein	1	0	0	1	1	1	1	1	1	0	1	8	Accessory	COG3336_S#	33.3	143	1.74E-33	K02351	28.7	93	4.22E-18
5105	PWE08259.1	-	DUF2797 domain-containing protein	1	0	1	1	1	1	1	1	1	1	1	10	Accessory								
5106	WP_048456600.1	-	universal stress protein	1	0	1	1	1	1	1	1	1	1	1	10	Accessory	COG0589_T#	34.4	137	9.53E-32	NA	34.4	137	2.55E-31
5107	OSC56559.1	-	3-hydroxybutyryl-CoA dehydrogenase	1	1	1	1	1	1	1	1	1	1	1	11	CORE	COG1250_I#	59.4	340	7.42E-93	K00074	59.4	340	1.98E-92
5108	WP_052183223.1	-	GNAT family N-acetyltransferase	1	0	0	0	1	1	0	0	0	0	1	4	Accessory	COG3375_S#	35.7	114	8.64E-25	NA	34.2	124	1.31E-27
5109	Prokka:CIODPCDJ_05881	-	hypothetical protein	1	0	1	1	1	1	1	1	1	1	1	10	Accessory								

cluster	GI	gene	Annotation	Streptomyces_thermocarboxydus_BPSAC147	Streptomyces_sp._4F	Streptomyces_sp._BSE7F	Streptomyces_sp._ETH9427	Streptomyces_sp._FxanaD5	Streptomyces_sp._UNC401CLCol	Actinospica_acidiphila_NRRL_B_24431	Streptomyces_sp._HNS054	Streptomyces_sp._SMS_SU21	Streptomyces_sp._ZS0098	Streptomyces_thermocarboxydus_K155	Addition	Core-Accessory-Unique	COG	%Similarity_COG	Score_COG	Evalue_COG	KEGG	%Similarity_KEGG	Score_KEGG	Evalue_KEGG
5110	WP_102638526.1	-	sugar kinase	0	0	0	1	0	0	0	1	1	0	0	3	Accessory	COG0524_G#	34	169	1.73E-41	NA	35.4	166	3.92E-40
5111	Prokka:JAPCLKMA_02128	-	hypothetical protein	1	1	1	1	1	1	1	1	1	1	1	11	CORE								
5112	WP_028960046.1	-	Cof-type HAD-IIB family hydrolase	1	1	1	1	1	1	1	1	1	1	1	11	CORE	COG0561_R#	42.8	176	1.85E-43	K07024	42.8	176	4.95E-43
5113	WP_028959469.1	-	membrane protein	1	1	1	0	1	1	1	1	1	1	1	10	Accessory	COG4965_U#	35.3	102	2.6E-21	K12510	35.3	102	6.95E-21
5114	OSC71456.1	-	hypothetical protein	1	1	1	1	1	1	1	1	1	1	1	11	CORE	COG3346_S#	31.1	70	1.1E-11				
5115	OSC63411.1	-	hypothetical protein	0	1	0	0	0	0	0	0	0	0	0	1	Unique	COG2936_R#	38.9	192	1.91E-48				
5116	PWE11039.1	-	hypothetical protein	0	0	1	0	0	0	0	0	0	0	0	1	Unique	COG2202_T#	24.3	57	9.6E-08				
5117	Prokka:JAPCLKMA_00587	-	hypothetical protein	1	1	1	1	1	1	1	1	1	1	1	11	CORE	COG1076_O#	27.4	79	2.36E-14	NA	27.4	79	6.3E-14
5118	OSC72943.1	-	decarboxylase	1	1	1	1	1	1	1	1	1	1	1	11	CORE	COG3473_Q#	35.4	132	3.06E-30	K01799	35.4	132	8.16E-30
5119	WP_019527894.1	-	alpha/beta fold hydrolase	1	1	1	1	1	1	1	1	1	1	1	11	CORE	COG1075_R#	35.1	91	4.58E-18	K01046	32	80	3.68E-14
5120	Prokka:JAPCLKMA_01029	-	hypothetical protein	1	0	1	1	1	1	1	1	1	1	1	10	Accessory								
5121	WP_033274229.1	-	hypothetical protein	0	0	1	1	0	0	1	1	1	1	0	6	Accessory								
5122	WP_033274529.1	-	DUF2236 domain-containing protein	1	1	1	1	1	1	1	1	1	1	1	11	CORE	COG3662_S#	44	184	6.78E-46				
5123	AXI87876.1	rsmI	16S rRNA (cytidine(1402)-2'-O)-methyltransferase	1	1	1	1	1	1	1	1	1	1	1	11	CORE	COG0313_R#	53.2	268	2.73E-71	K07056	52.6	267	2.12E-70

cluster	GI	gene	Annotation	Streptomyces_thermocarboxydus_BPSAC147	Streptomyces_sp._4F	Streptomyces_sp._BSE7F	Streptomyces_sp._ETH9427	Streptomyces_sp._FxanaD5	Streptomyces_sp._UNC401CLCol	Actinospica_acidiphila_NRRL_B_24431	Streptomyces_sp._HNS054	Streptomyces_sp._SMS_SU21	Streptomyces_sp._ZS0098	Streptomyces_thermocarboxydus_K155	Addition	Core-Accessory-Unique	COG	%Similarity_COG	Score_COG	Evalue_COG	KEGG	%Similarity_KEGG	Score_KEGG	Evalue_KEGG
5124	WP_028960146.1	-	diaminopimelate epimerase	1	1	1	0	1	1	1	1	1	1	1	10	Accessory	COG0253_E#	43.6	210	6.76E-54	K01778	44.2	208	1.17E-52
5125	Prokka:CIODPCDJ_03549	-	hypothetical protein	1	1	1	1	1	1	1	1	1	1	1	11	CORE								
5126	PWE07651.1	-	spermine synthase	1	1	1	1	1	1	1	1	1	1	1	11	CORE	COG0421_E#	35.1	150	1.08E-35				
5127	Prokka:CIODPCDJ_03345	-	hypothetical protein	1	1	1	0	1	1	1	1	1	1	1	10	Accessory								
5128	OSC71522.1	-	hypothetical protein	1	1	1	1	1	1	1	1	1	1	1	11	CORE								
5129	Prokka:JAPCLKMA_00498	-	hypothetical protein	1	1	1	1	1	1	1	1	1	1	1	11	CORE								
5130	AXI86812.1	-	hypothetical protein	0	0	1	1	0	0	0	1	0	1	0	4	Accessory								
5131	PWE08650.1	-	ABC transporter	0	0	1	1	0	0	0	1	1	1	0	5	Accessory	COG0842_V#	36.6	157	6.79E-38	K01992	31.9	92	5.49E-18
5132	WP_019526068.1	-	N4-gp56 family major capsid protein	0	0	0	0	1	0	0	0	0	0	0	1	Unique					NA	34.2	138	1.14E-31
5133	Prokka:JAPCLKMA_04218	-	hypothetical protein	1	1	1	1	1	1	1	1	1	1	1	11	CORE								
5134	WP_019521410.1	-	metal ABC transporter permease	0	0	0	0	1	1	1	0	0	0	1	4	Accessory	COG1108_P#	31.3	124	8.32E-28	K09816	23.8	94	1.89E-18
5135	WP_122215900.1	-	alpha/beta hydrolase	0	0	1	1	0	0	1	1	1	1	0	6	Accessory	COG0596_R#	34.4	69	3.18E-11	NA	34.4	69	8.49E-11
5136	WP_122216229.1	-	universal stress protein	0	0	0	0	0	0	0	0	1	1	0	2	Accessory	COG0589_T#	29.6	108	3.62E-23	NA	31.3	107	2.15E-22

cluster	GI	gene	Annotation	Streptomyces_thermocarboxydus_BPSAC147	Streptomyces_sp._4F	Streptomyces_sp._BSE7F	Streptomyces_sp._ETH9427	Streptomyces_sp._FxanaD5	Streptomyces_sp._UNC401CLCol	Actinospica_acidiphila_NRRL_B_24431	Streptomyces_sp._HNS054	Streptomyces_sp._SMS_SU21	Streptomyces_sp._ZS0098	Streptomyces_thermocarboxydus_K155	Addition	Core-Accessory-Unique	COG	%Similarity_COG	Score_COG	Evalue_COG	KEGG	%Similarity_KEGG	Score_KEGG	Evalue_KEGG
5137	WP_033275834.1	-	arylamine N-acetyltransferase	1	1	0	1	1	1	1	0	0	1	1	8	Accessory	COG2162_Q#	35.5	136	2.12E-31	K00675	30.5	123	3.79E-27
5138	Prokka:CIODPCDJ_06604	-	hypothetical protein	0	0	0	0	1	0	0	0	0	0	1	2	Accessory	COG0456_R#	31.9	42	0.00416				
5139	WP_028961274.1	cas7c	type I-C CRISPR-associated protein Cas7/Csd2	0	0	0	1	0	1	0	0	0	0	0	2	Accessory								
5140	OSC73262.1	-	2-aminoethylphosphonate ABC transporter permease subunit	0	1	0	0	0	0	0	0	0	0	0	1	Unique	COG0555_O#	49.8	272	1.89E-72	K11070	27.4	93	4.2E-18
5141	WP_033276855.1	-	ABC transporter permease	1	0	0	0	1	1	1	0	0	0	1	5	Accessory	COG0600_P#	52.1	300	6.49E-81	K15554	41.9	185	8.12E-46
5142	OSC58177.1	-	glycosyl transferase	0	1	0	0	0	0	0	0	0	0	0	1	Unique	COG0463_M#	38.7	130	1.16E-29	K03820	28.5	86	3.93E-16
5143	WP_122213376.1	-	SMP-30/gluconolactonase/LRE family protein	0	0	0	0	0	0	0	0	0	1	0	1	Unique	COG3386_G#	35.6	153	1.28E-36				
5144	OSC70430.1	-	hypothetical protein	1	1	0	1	1	1	1	1	1	1	1	10	Accessory								
5145	WP_048460921.1	-	IS5 family transposase	0	0	0	0	0	0	0	1	0	0	0	1	Unique	COG3293_L#	43.1	227	6.98E-59	K07492	41.5	209	4.02E-53

cluster	GI	gene	Annotation	Streptomyces_thermocarboxydus_BPSAC147	Streptomyces_sp._4F	Streptomyces_sp._BSE7F	Streptomyces_sp._ETH9427	Streptomyces_sp._FxanaD5	Streptomyces_sp._UNC401CLCol	Actinospica_acidiphila_NRRL_B_24431	Streptomyces_sp._HNS054	Streptomyces_sp._SMS_SU21	Streptomyces_sp._ZS0098	Streptomyces_thermocarboxydus_K155	Addition	Core-Accessory-Unique	COG	%Similarity_COG	Score_COG	Evalue_COG	KEGG	%Similarity_KEGG	Score_KEGG	Evalue_KEGG
5146	OSC71607.1	-	metallophosphoesterase	1	1	0	0	1	1	1	1	1	1	1	9	Accessory	COG1409_R#	60.4	369	8.71E-102	NA	60.4	369	2.33E-101
5147	OSC71585.1	-	hypothetical protein	0	1	0	0	0	0	0	0	0	0	0	1	Unique								
5148	WP_106959393.1	-	restriction endonuclease	1	1	1	1	1	1	1	1	1	1	1	11	CORE	COG1787_V#	31.9	60	1.47E-08	NA	31.9	60	3.93E-08
5149	WP_048458644.1	-	bifunctional 5,10-methylenetetrahydrofolate dehydrogenase/5,10-methenyltetrahydrofolate cyclohydrolase	0	0	1	1	1	0	0	1	1	1	0	6	Accessory	COG0190_H#	50.9	250	7.67E-66	NA	46.4	236	2.34E-61
5150	WP_122215127.1	-	ABC transporter ATP-binding protein	1	1	1	1	1	1	1	1	1	1	1	11	CORE	COG1120_PH#	66.7	366	9.6E-101	K02013	59.7	332	3.14E-90
5151	WP_028960585.1	-	GNAT family N-acetyltransferase	1	0	1	1	1	1	1	1	1	1	1	10	Accessory	COG3393_R#	31.1	100	1.28E-20				
5152	OSC61449.1	-	heme ABC transporter ATP-binding protein	0	1	0	0	0	0	1	1	0	0	1	4	Accessory	COG4559_P#	48.5	208	2.56E-53	K02013	42	168	1.02E-40
5153	WP_019527226.1	-	alpha/beta hydrolase	1	0	0	0	1	1	0	0	0	0	1	4	Accessory	COG0596_R#	33.2	142	2.25E-33	NA	33.2	142	6.02E-33

cluster	GI	gene	Annotation	Streptomyces_thermocarboxydus_BPSAC147	Streptomyces_sp._4F	Streptomyces_sp._BSE7F	Streptomyces_sp._ETH9427	Streptomyces_sp._FxanaD5	Streptomyces_sp._UNC401CLCol	Actinospica_acidiphila_NRRL_B_24431	Streptomyces_sp._HNS054	Streptomyces_sp._SMS_SU21	Streptomyces_sp._ZS0098	Streptomyces_thermocarboxydus_K155	Addition	Core-Accessory-Unique	COG	%Similarity_COG	Score_COG	Evalue_COG	KEGG	%Similarity_KEGG	Score_KEGG	Evalue_KEGG
5154	Prokka:CIODPCDJ_04559	-	hypothetical protein	1	0	0	0	1	1	0	0	0	0	1	4	Accessory	COG4249_R#	28.6	69	2.42E-11	NA	38.4	102	5.28E-21
5155	OSC63178.1	-	sugar ABC transporter permease	0	1	0	0	0	0	0	0	0	0	0	1	Unique	COG1175_G#	33.5	168	2.94E-41	K02025	33.6	146	5.44E-34
5156	WP_048456308.1	-	sulfurtransferase	1	1	1	1	1	1	1	1	1	1	1	11	CORE	COG2897_P#	49.2	256	1.07E-67	K01011	45.9	221	1.02E-56
5157	WP_033273858.1	-	DUF1963 domain-containing protein	0	0	0	0	0	0	1	0	0	0	0	1	Unique								
5158	WP_122217385.1	-	ribonuclease	1	0	1	1	1	1	1	1	1	1	1	10	Accessory	COG2356_L#	63	364	4.76E-100	NA	63	364	1.27E-99
5159	WP_051724536.1	-	alpha/beta fold hydrolase	1	1	0	0	1	1	1	0	0	0	1	6	Accessory	COG0596_R#	33.3	132	2.33E-30	NA	37.3	60	3.93E-08
5160	OSC72394.1	-	ABC transporter	1	1	1	1	1	1	1	1	1	1	1	11	CORE	COG0842_V#	30.9	135	3.6E-31	K01992	25.3	85	8.73E-16
5161	AXI89659.1	-	nucleotidyltransferase	0	0	0	1	0	0	0	0	0	0	0	1	Unique								
5162	OSC66331.1	-	protein iolH	0	1	0	0	0	0	0	0	0	0	0	1	Unique	COG1082_G#	58.4	360	6.88E-99	K06605	57.1	333	1.84E-90
5163	OSC70550.1	-	ergot alkaloid biosynthesis protein	0	1	0	0	0	0	0	0	0	0	0	1	Unique	COG0702_MG#	41.4	196	1.01E-49	NA	41.4	196	2.69E-49
5164	OSC58912.1	-	hypothetical protein	0	1	0	0	0	0	0	0	0	0	0	1	Unique								
5165	OSC67910.1	-	hypothetical protein	0	1	0	0	0	0	0	0	0	0	0	1	Unique								
5166	WP_122213831.1	-	N-acetylmuramoyl-L-alanine amidase	0	0	0	0	1	0	0	0	0	1	0	2	Accessory					K01446	34.5	90	2.71E-17

cluster	GI	gene	Annotation	Streptomyces_thermocarboxydus_BPSAC147	Streptomyces_sp._4F	Streptomyces_sp._BSE7F	Streptomyces_sp._ETH9427	Streptomyces_sp._FxanaD5	Streptomyces_sp._UNC401CLCol	Actinospica_acidiphila_NRRL_B_24431	Streptomyces_sp._HNS054	Streptomyces_sp._SMS_SU21	Streptomyces_sp._ZS0098	Streptomyces_thermocarboxydus_K155	Addition	Core-Accessory-Unique	COG	%Similarity_COG	Score_COG	Evalue_COG	KEGG	%Similarity_KEGG	Score_KEGG	Evalue_KEGG
5167	OSC64554.1	-	B/F/G family RNA polymerase sigma-70 factor	1	1	1	1	1	1	1	1	1	1	1	11	CORE	COG1191_K#	45.8	215	2.09E-55	K03090	45.8	215	5.59E-55
5168	OSC73125.1	-	ABC transporter ATP-binding protein	0	1	1	1	1	1	1	1	1	1	1	10	Accessory	COG1131_V#	42.8	203	1.08E-51	NA	43.6	184	1.38E-45
5169	OSC66551.1	-	GNAT family N-acetyltransferase	1	1	1	1	1	1	1	1	1	1	1	11	CORE	COG0454_KR#	29.2	91	5.96E-18	NA	36.3	47	0.000344
5170	Prokka:JAPCLKMA_01553	-	hypothetical protein	1	0	0	0	0	0	0	0	0	0	0	1	Unique								
5171	OSC70165.1	-	tyrosinase	0	1	0	0	0	0	0	0	0	0	0	1	Unique					K00505	34.6	128	8.99E-29
5172	WP_122214030.1	-	aminoglycoside phosphotransferase family protein	1	1	1	1	1	1	1	1	1	1	1	11	CORE								
5173	WP_102638002.1	-	hypothetical protein	0	0	0	1	0	0	0	0	1	0	0	2	Accessory								
5174	PWE10226.1	-	transcriptional regulator	1	1	1	1	1	1	1	1	1	1	1	11	CORE								
5175	OSC57399.1	-	phosphotransferase	0	1	0	0	0	0	0	0	0	0	0	1	Unique								
5176	Prokka:JAPCLKMA_00177	-	hypothetical protein	1	1	1	1	1	1	1	1	1	1	1	11	CORE								
5177	WP_033276769.1	-	alpha/beta fold hydrolase	1	0	1	1	1	1	1	1	1	1	1	10	Accessory	COG1075_R#	37.2	141	6.53E-33	K01046	32.9	97	1.69E-19

cluster	GI	gene	Annotation	Streptomyces_thermocarboxydus_BPSAC147	Streptomyces_sp._4F	Streptomyces_sp._BSE7F	Streptomyces_sp._ETH9427	Streptomyces_sp._FxanaD5	Streptomyces_sp._UNC401CLCol	Actinospica_acidiphila_NRRL_B_24431	Streptomyces_sp._HNS054	Streptomyces_sp._SMS_SU21	Streptomyces_sp._ZS0098	Streptomyces_thermocarboxydus_K155	Addition	Core-Accessory-Unique	COG	%Similarity_COG	Score_COG	Evalue_COG	KEGG	%Similarity_KEGG	Score_KEGG	Evalue_KEGG
5178	AXI90640.1	-	phosphoenolpyruvate phosphomutase	0	0	0	1	0	0	0	0	0	0	0	1	Unique	COG2513_G#	37.4	171	5.9E-42	K01841	33.2	106	4.76E-22
5179	OSC72219.1	-	zinc metalloprotease HtpX	1	1	1	1	1	1	1	1	1	1	1	11	CORE	COG0501_O#	65	368	1.93E-101	K03799	64.3	364	1.27E-99
5180	PWE10933.1	-	IS5 family transposase	0	0	1	0	0	0	0	0	0	0	0	1	Unique	COG3293_L#	39.4	183	1.5E-45	K07492	36.5	159	6.19E-38
5181	WP_019524291.1	-	hypothetical protein	1	1	0	1	1	1	1	1	1	1	1	10	Accessory								
5182	WP_048455014.1	-	Fpg/Nei family DNA glycosylase	1	1	1	1	1	1	1	1	1	1	1	11	CORE	COG0266_L#	58.7	187	6.09E-47	K10563	62.3	186	2.77E-46
5183	OSC61026.1	-	sugar ABC transporter permease	1	1	1	1	1	1	1	1	1	1	1	11	CORE	COG0395_G#	36.1	164	5.52E-40	K17236	29.7	141	1.02E-32
5184	Prokka:CIODPCDJ_00736	-	hypothetical protein	1	1	1	1	1	1	1	1	0	1	1	10	Accessory								
5185	WP_122214186.1	-	CAP domain-containing protein	0	0	1	1	1	1	1	1	1	1	1	9	Accessory	COG2340_S#	43.8	137	9.44E-32	NA	43.8	137	2.52E-31
5186	WP_048458684.1	-	SDR family NAD(P)-dependent oxidoreductase	0	0	1	1	0	0	1	1	1	1	0	6	Accessory	COG1028_IQR#	40.6	181	5.7E-45	NA	36	154	1.53E-36

cluster	GI	gene	Annotation	Streptomyces_thermocarboxydus_BPSAC147	Streptomyces_sp._4F	Streptomyces_sp._BSE7F	Streptomyces_sp._ETH9427	Streptomyces_sp._FxanaD5	Streptomyces_sp._UNC401CLCol	Actinospica_acidiphila_NRRL_B_24431	Streptomyces_sp._HNS054	Streptomyces_sp._SMS_SU21	Streptomyces_sp._ZS0098	Streptomyces_thermocarboxydus_K155	Addition	Core-Accessory-Unique	COG	%Similarity_COG	Score_COG	Evalue_COG	KEGG	%Similarity_KEGG	Score_KEGG	Evalue_KEGG
5187	WP_102641133.1	-	sugar phosphate isomerase/epimerase	1	0	1	1	1	1	1	1	1	1	1	10	Accessory	COG1082_G#	54.7	285	1.64E-76	NA	54.7	285	4.39E-76
5188	OSC63835.1	-	hypothetical protein	0	1	0	0	0	0	0	0	0	0	0	1	Unique	COG0515_RTKL#	38.7	92	2.04E-18	K13419	34.7	88	1.03E-16
5189	WP_122216454.1	-	ABC transporter ATP-binding protein	1	1	1	1	1	1	1	1	1	1	1	11	CORE	COG0411_E#	47.2	221	3.8E-57	K01995	47.2	221	1.02E-56
5190	AXI86177.1	-	hypothetical protein	1	0	0	1	1	1	1	0	0	1	1	7	Accessory	COG3300_T#	35.2	130	1.15E-29				
5191	OSC72959.1	-	hypothetical protein	1	1	1	1	1	1	1	1	1	1	1	11	CORE								
5192	Prokka:CIODPCDJ_04657	dasC_6	Diacetylchitobiose uptake system permease protein DasC	1	1	0	0	1	1	0	0	0	0	1	5	Accessory	COG0395_G#	37.3	186	1.77E-46	K10238	29.6	128	8.96E-29
5193	WP_102639838.1	-	restriction endonuclease	0	0	0	0	0	0	0	0	1	0	0	1	Unique								
5194	AXI91243.1	-	hypothetical protein	0	0	0	1	0	0	0	0	0	0	0	1	Unique								
5195	OSC63329.1	-	sugar ABC transporter permease	0	1	0	0	0	0	0	0	0	0	0	1	Unique	COG0395_G#	34.9	193	1.45E-48	K10119	32.5	182	5.23E-45
5196	OSC67019.1	-	alpha/beta hydrolase	0	1	0	0	0	0	0	0	0	0	0	1	Unique	COG0596_R#	32.1	107	8.01E-23	K01563	27.8	75	9E-13
5197	OSC57812.1	-	hypothetical protein	0	1	0	0	0	0	0	0	0	0	0	1	Unique	COG1314_U#	35.8	42	0.00316	NA	29.8	58	1.14E-07
5198	WP_146217830.1	-	hypothetical protein	0	1	1	0	0	0	0	0	1	0	0	3	Accessory								

cluster	GI	gene	Annotation	Streptomyces_thermocarboxydus_BPSAC147	Streptomyces_sp._4F	Streptomyces_sp._BSE7F	Streptomyces_sp._ETH9427	Streptomyces_sp._FxanaD5	Streptomyces_sp._UNC401CLCol	Actinospica_acidiphila_NRRL_B_24431	Streptomyces_sp._HNS054	Streptomyces_sp._SMS_SU21	Streptomyces_sp._ZS0098	Streptomyces_thermocarboxydus_K155	Addition	Core-Accessory-Unique	COG	%Similarity_COG	Score_COG	Evalue_COG	KEGG	%Similarity_KEGG	Score_KEGG	Evalue_KEGG
5199	Prokka:JAPCLKMA_04914	-	hypothetical protein	1	0	0	0	0	0	0	0	0	0	0	1	Unique								
5200	OSC69306.1	-	sugar ABC transporter permease	1	1	0	0	0	0	0	0	0	0	1	3	Accessory	COG0395_G#	31.9	159	2.32E-38	K10119	29.1	147	2.43E-34
5201	WP_157842211.1	-	4'-phosphopantetheinyl transferase superfamily protein	0	1	0	0	0	0	1	0	0	0	0	2	Accessory	COG2977_Q#	40.2	89	1.73E-17				
5202	OSC69116.1	-	ABC transporter permease	0	1	0	0	0	0	0	0	0	0	0	1	Unique	COG0600_P#	69.5	334	3.08E-91	K15552	37.3	168	7.81E-41
5203	WP_026244017.1	-	RNA methyltransferase	1	0	1	1	1	1	1	1	1	1	1	10	Accessory	COG0566_J#	47	208	2.54E-53	K03437	46.6	205	9.81E-52
5204	AXI91328.1	-	hypothetical protein	0	0	0	1	0	0	0	0	0	0	0	1	Unique								
5205	WP_114874732.1	folP	dihydropteroate synthase	1	1	1	1	1	1	1	1	1	1	1	11	CORE	COG0294_H#	57	321	3.52E-87	K00796	57	321	9.39E-87
5206	WP_033272663.1	-	PHP domain-containing protein	1	0	0	1	1	1	1	1	1	1	1	9	Accessory	COG0613_R#	41.5	164	4.21E-40	K07053	40.8	159	3.62E-38
5207	Prokka:CIODPCDJ_00782	-	hypothetical protein	1	1	1	1	1	1	1	1	1	1	1	11	CORE								
5208	WP_109288610.1	-	DNA primase	1	1	1	0	1	1	1	1	1	1	1	10	Accessory								
5209	OSC61344.1	-	peptidase	0	1	0	0	0	0	0	0	0	0	0	1	Unique								

cluster	GI	gene	Annotation	Streptomyces_thermocarboxydus_BPSAC147	Streptomyces_sp._4F	Streptomyces_sp._BSE7F	Streptomyces_sp._ETH9427	Streptomyces_sp._FxanaD5	Streptomyces_sp._UNC401CLCol	Actinospica_acidiphila_NRRL_B_24431	Streptomyces_sp._HNS054	Streptomyces_sp._SMS_SU21	Streptomyces_sp._ZS0098	Streptomyces_thermocarboxydus_K155	Addition	Core-Accessory-Unique	COG	%Similarity_COG	Score_COG	Evalue_COG	KEGG	%Similarity_KEGG	Score_KEGG	Evalue_KEGG
5210	WP_078894944.1	-	alpha/beta hydrolase	0	0	0	0	0	0	0	1	1	1	0	3	Accessory								
5211	AXI85253.1	-	sulfite exporter TauE/SafE family protein	0	0	1	1	0	0	0	1	1	1	0	5	Accessory	COG0730_R#	65	308	2.36E-83	K07090	43.5	199	4.12E-50
5212	WP_033274394.1	-	hypothetical protein	1	1	1	1	1	1	1	1	1	1	1	11	CORE								
5213	WP_033276118.1	otsB	trehalose-phosphatase	1	1	1	1	1	1	1	1	1	1	1	11	CORE	COG1877_G#	30.1	102	2.57E-21				
5214	WP_028961676.1	-	ATP-binding cassette domain-containing protein	1	1	1	1	1	1	1	1	1	1	1	11	CORE	COG1122_P#	45.6	213	1.03E-54	K02006	45.6	213	2.76E-54
5215	WP_102640540.1	truA	tRNA pseudouridine(38-40) synthase TruA	1	1	1	1	1	1	1	1	1	1	1	11	CORE	COG0101_J#	50.3	258	2.8E-68	K06173	48.6	220	1.73E-56
5216	WP_019522962.1	-	carbohydrate ABC transporter permease	1	0	1	1	1	1	1	1	1	1	1	10	Accessory	COG0395_G#	48.2	262	1.94E-69	K05815	32	144	2.05E-33
5217	WP_033274403.1	-	helix-turn-helix domain-containing protein	1	0	0	0	1	1	1	0	0	0	1	5	Accessory	COG1396_K#	37.8	156	1.15E-37				

cluster	GI	gene	Annotation	Streptomyces_thermocarboxydus_BPSAC147	Streptomyces_sp._4F	Streptomyces_sp._BSE7F	Streptomyces_sp._ETH9427	Streptomyces_sp._FxanaD5	Streptomyces_sp._UNC401CLCol	Actinospica_acidiphila_NRRL_B_24431	Streptomyces_sp._HNS054	Streptomyces_sp._SMS_SU21	Streptomyces_sp._ZS0098	Streptomyces_thermocarboxydus_K155	Addition	Core-Accessory-Unique	COG	%Similarity_COG	Score_COG	Evalue_COG	KEGG	%Similarity_KEGG	Score_KEGG	Evalue_KEGG
5218	WP_033273701.1	-	4Fe-4S dicluster domain-containing protein	1	1	1	1	1	1	1	1	1	1	1	11	CORE	COG3383_R#	34.1	183	1.14E-45	K00336	37.8	137	1.92E-31
5219	OSC57210.1	-	sugar phosphate isomerase	1	1	1	1	1	1	1	1	1	1	1	11	CORE	COG1082_G#	47	243	9.32E-64				
5220	OSC70911.1	-	hypothetical protein	0	1	0	0	0	0	0	0	0	0	0	1	Unique								
5221	Prokka:CIODPCDJ_05699	ycjP_2	Inner membrane ABC transporter permease protein YcjP	1	0	1	1	1	1	1	1	1	1	1	10	Accessory	COG0395_G#	40.3	203	1.07E-51	K02026	37.5	189	4.26E-47
5222	Prokka:CIODPCDJ_03652	-	hypothetical protein	1	1	1	1	1	1	1	1	1	1	1	11	CORE								
5223	WP_028961715.1	-	thioesterase family protein	1	1	1	1	1	1	1	1	1	1	1	11	CORE					NA	30.9	99	5.8E-20
5224	OSC61193.1	-	type I methionyl aminopeptidase	1	1	1	1	1	1	1	1	1	1	1	11	CORE	COG0024_J#	72.6	427	4.55E-119	K01265	72.6	427	1.21E-118
5225	AXI85257.1	-	disulfide bond formation protein DsbA	0	1	1	1	0	0	0	1	1	1	0	6	Accessory	COG1651_O#	33.8	132	2.32E-30	NA	30.3	70	2.21E-11
5226	OSC63115.1	-	glutathione ABC transporter ATP-binding protein	0	1	0	0	0	0	0	0	0	0	0	1	Unique	COG1123_R#	49.6	257	4.77E-68	K13892	49.3	257	1.66E-67

cluster	GI	gene	Annotation	Streptomyces_thermocarboxydus_BPSAC147	Streptomyces_sp._4F	Streptomyces_sp._BSE7F	Streptomyces_sp._ETH9427	Streptomyces_sp._FxanaD5	Streptomyces_sp._UNC401CLCol	Actinospica_acidiphila_NRRL_B_24431	Streptomyces_sp._HNS054	Streptomyces_sp._SMS_SU21	Streptomyces_sp._ZS0098	Streptomyces_thermocarboxydus_K155	Addition	Core-Accessory-Unique	COG	%Similarity_COG	Score_COG	Evalue_COG	KEGG	%Similarity_KEGG	Score_KEGG	Evalue_KEGG
5227	OSC62575.1	-	transporter	0	1	0	0	0	0	0	0	0	0	0	1	Unique	COG1173_EP#	32	132	3.02E-30	K02034	30.4	119	5.42E-26
5228	AXI84964.1	-	enoyl-CoA hydratase	0	1	0	1	0	0	0	1	0	0	0	3	Accessory	COG1024_I#	31.5	106	1.78E-22	K01715	28.8	100	2.6E-20
5229	WP_128128253.1	-	hypothetical protein	0	0	1	0	0	0	0	0	1	0	0	2	Accessory								
5230	Prokka:JAPCLKMA_05490	iolS_3	Aldo-keto reductase IolS	1	0	0	0	0	0	0	0	0	0	0	1	Unique	COG0667_C#	34.1	163	1.23E-39	NA	35.6	134	1.25E-30
5231	WP_157842156.1	-	hypothetical protein	0	0	0	0	0	0	1	0	0	0	0	1	Unique								
5232	OSC66366.1	-	tRNA (guanosine(46)-N7)-methyltransferase TrmB	1	1	1	1	1	1	1	1	1	1	1	11	CORE	COG0220_R#	50	216	1.59E-55	K03439	49.5	215	7.26E-55
5233	Prokka:JAPCLKMA_04898	-	hypothetical protein	1	0	0	0	0	0	0	0	0	0	0	1	Unique								
5234	OSC58521.1	-	class II glutamine amidotransferase	1	1	1	1	1	1	1	1	1	1	1	11	CORE	COG0121_R#	41.6	209	1.49E-53	K18802	33.3	152	5.78E-36
5235	OSC58691.1	-	precorrin-4 C(11)-methyltransferase	1	1	1	1	1	1	1	1	1	1	1	11	CORE	COG2875_H#	47.7	227	6.91E-59	K05936	42.6	218	1.12E-55
5236	OSC67021.1	-	AraC family transcriptional regulator	0	1	0	0	0	0	0	0	0	0	0	1	Unique	COG2207_K#	31.9	119	2.03E-26				
5237	WP_107081009.1	-	TIGR03084 family protein	1	1	1	1	1	1	1	1	1	1	1	11	CORE					NA	62	310	1.26E-83

cluster	GI	gene	Annotation	Streptomyces_thermocarboxydus_BPSAC147	Streptomyces_sp._4F	Streptomyces_sp._BSE7F	Streptomyces_sp._ETH9427	Streptomyces_sp._FxanaD5	Streptomyces_sp._UNC401CLCol	Actinospica_acidiphila_NRRL_B_24431	Streptomyces_sp._HNS054	Streptomyces_sp._SMS_SU21	Streptomyces_sp._ZS0098	Streptomyces_thermocarboxydus_K155	Addition	Core-Accessory-Unique	COG	%Similarity_COG	Score_COG	Evalue_COG	KEGG	%Similarity_KEGG	Score_KEGG	Evalue_KEGG
5238	PWE08055.1	-	1,4-dihydroxy-6-naphthoate synthase	1	1	1	1	1	1	1	1	1	1	1	11	CORE	COG2107_R#	62.3	345	1.73E-94	K11785	48.9	228	6.32E-59
5239	PWE08567.1	-	hypothetical protein	0	0	1	0	0	0	0	0	0	0	0	1	Unique								
5240	WP_122212717.1	-	fumarylacetoacetate hydrolase family protein	1	1	1	1	1	1	1	1	1	1	1	11	CORE	COG0179_Q#	58.1	319	1.74E-86	NA	44.8	210	2.33E-53
5241	OSC70420.1	-	MBL fold metallo-hydrolase	1	1	1	1	1	1	1	1	1	1	1	11	CORE	COG0491_R#	51.3	226	1.17E-58	NA	51.3	226	3.14E-58
5242	Prokka:JAPCLKMA_05360	chpC	Chaplin-C	1	1	1	1	1	1	1	1	1	1	1	11	CORE					NA	40.1	137	2.5E-31
5243	Prokka:CIODPCDJ_01892	efeU	Ferrous iron permease EfeU	1	0	0	0	1	1	0	0	0	0	1	4	Accessory	COG0672_P#	40.1	204	6.24E-52	K07243	39.1	191	1.46E-47
5244	WP_102636944.1	mltG	endolytic transglycosylase MltG	1	1	1	1	1	1	1	1	1	1	1	11	CORE	COG1559_R#	38.8	132	3.01E-30	K07082	34.9	130	3.06E-29
5245	WP_048454957.1	-	MoxR family ATPase	1	0	1	1	1	1	1	1	1	1	1	10	Accessory	COG0714_R#	69.6	391	3.6E-108	NA	69.6	391	9.61E-108
5246	Prokka:CIODPCDJ_02337	-	hypothetical protein	0	0	0	0	1	1	1	0	0	0	1	4	Accessory	COG1266_R#	31.4	48	0.0000438				
5247	WP_159020877.1	-	hypothetical protein	0	0	0	0	0	0	0	1	0	0	0	1	Unique								
5248	Prokka:JAPCLKMA_05483	cof	HMP-PP phosphatase	1	0	0	0	0	0	0	0	0	0	0	1	Unique	COG0561_R#	21.1	57	1.23E-07	K07024	26	52	0.0000106
5249	WP_078895179.1	-	hypothetical protein	0	0	0	0	0	0	0	1	0	0	0	1	Unique								

cluster	GI	gene	Annotation	Streptomyces_thermocarboxydus_BPSAC147	Streptomyces_sp._4F	Streptomyces_sp._BSE7F	Streptomyces_sp._ETH9427	Streptomyces_sp._FxanaD5	Streptomyces_sp._UNC401CLCol	Actinospica_acidiphila_NRRL_B_24431	Streptomyces_sp._HNS054	Streptomyces_sp._SMS_SU21	Streptomyces_sp._ZS0098	Streptomyces_thermocarboxydus_K155	Addition	Core-Accessory-Unique	COG	%Similarity_COG	Score_COG	Evalue_COG	KEGG	%Similarity_KEGG	Score_KEGG	Evalue_KEGG
5250	WP_033273499.1	-	alpha/beta hydrolase	1	0	1	1	1	1	1	1	1	1	1	10	Accessory	COG0596_R#	34.2	161	3.55E-39	K01563	29.9	109	4.28E-23
5251	Prokka:JAPCLKMA_05198	-	hypothetical protein	1	0	1	1	1	1	0	1	0	1	1	8	Accessory								
5252	AXI91225.1	-	hypothetical protein	0	0	0	1	0	0	0	0	0	0	0	1	Unique								
5253	Prokka:JAPCLKMA_01591	-	hypothetical protein	1	0	0	0	0	0	0	0	0	0	0	1	Unique					NA	29.5	52	0.00000811
5254	AXI86912.1	-	hypothetical protein	0	0	1	1	0	0	0	0	0	0	0	2	Accessory								
5255	WP_019521879.1	-	LysR family transcriptional regulator	1	0	0	0	1	1	1	0	0	0	1	5	Accessory	COG0583_K#	34.1	127	5.68E-29	NA	34	118	1.2E-25
5256	WP_019527694.1	-	hypothetical protein	0	0	0	0	1	0	0	0	0	0	1	2	Accessory								
5257	OSC69640.1	-	hypothetical protein	0	1	0	0	0	0	0	0	0	0	0	1	Unique								
5258	OSC69635.1	-	MerR family transcriptional regulator	0	1	0	0	0	0	0	0	0	0	0	1	Unique	COG0789_K#	32.4	67	1.19E-10	K06042	32	54	0.00000213
5259	AXI89800.1	-	peptidase M23	1	0	1	1	1	1	1	1	1	1	1	10	Accessory	COG0739_M#	41.2	79	2.32E-14	NA	34.3	77	3.07E-13
5260	OSC61099.1	-	carboxylesterase	0	1	0	0	0	0	0	0	0	0	0	1	Unique	COG2272_I#	46.1	195	3.79E-49	K03929	46.1	195	1.01E-48
5261	OSC73542.1	-	serine protease	1	1	0	1	1	1	1	1	1	1	1	10	Accessory	COG5640_O#	29.6	104	8.79E-22	K09640	36.3	144	1.57E-33
5262	OSC71266.1	-	NADH:ubiquinone oxidoreductase subunit J	1	1	0	1	1	1	1	1	1	1	1	10	Accessory	COG0839_C#	63.4	297	5.42E-80	K00339	63.4	297	1.45E-79

cluster	GI	gene	Annotation	Streptomyces_thermocarboxydus_BPSAC147	Streptomyces_sp._4F	Streptomyces_sp._BSE7F	Streptomyces_sp._ETH9427	Streptomyces_sp._FxanaD5	Streptomyces_sp._UNC401CLCol	Actinospica_acidiphila_NRRL_B_24431	Streptomyces_sp._HNS054	Streptomyces_sp._SMS_SU21	Streptomyces_sp._ZS0098	Streptomyces_thermocarboxydus_K155	Addition	Core-Accessory-Unique	COG	%Similarity_COG	Score_COG	Evalue_COG	KEGG	%Similarity_KEGG	Score_KEGG	Evalue_KEGG
5263	OSC63223.1	-	polyketide cyclase	0	1	0	0	0	0	0	0	0	0	0	1	Unique					NA	60	46	0.000445
5264	WP_102642088.1	-	SDR family oxidoreductase	1	1	1	1	1	1	1	1	1	1	1	11	CORE	COG0702_MG#	48.6	258	3.63E-68	K19267	50	255	8.21E-67
5265	Prokka:JAPCLKMA_03204	-	hypothetical protein	1	0	0	0	0	0	0	0	0	0	1	2	Accessory								
5266	WP_028961112.1	-	5'-methylthioadenosine/S-adenosylhomocysteine nucleosidase	0	0	0	0	0	1	0	0	0	0	0	1	Unique								
5267	PWE10801.1	fecE	Fe(3+)-dicitrate ABC transporter ATP-binding protein	1	1	1	1	1	1	1	1	1	1	1	11	CORE	COG1120_PH#	45.2	222	2.21E-57	K02013	42.4	208	8.81E-53
5268	Prokka:CIODPCDJ_00815	fabG_1	3-oxoacyl-[acyl-carrier-protein] reductase FabG	1	0	1	1	1	1	1	1	1	1	1	10	Accessory	COG1028_IQR#	42.4	181	3.31E-45	NA	42.4	181	8.83E-45
5269	WP_033273546.1	-	GntR family transcriptional regulator	1	1	1	1	1	1	1	1	1	1	1	11	CORE	COG2188_K#	36.1	136	1.59E-31	K03710	35.9	129	3.98E-29
5270	OSC70892.1	-	permease	1	1	1	1	1	1	1	1	1	1	1	11	CORE	COG1174_E#	43.6	167	6.45E-41	K05845	37.4	150	2.18E-35

cluster	GI	gene	Annotation	Streptomyces_thermocarboxydus_BPSAC147	Streptomyces_sp._4F	Streptomyces_sp._BSE7F	Streptomyces_sp._ETH9427	Streptomyces_sp._FxanaD5	Streptomyces_sp._UNC401CLCol	Actinospica_acidiphila_NRRL_B_24431	Streptomyces_sp._HNS054	Streptomyces_sp._SMS_SU21	Streptomyces_sp._ZS0098	Streptomyces_thermocarboxydus_K155	Addition	Core-Accessory-Unique	COG	%Similarity_COG	Score_COG	Evalue_COG	KEGG	%Similarity_KEGG	Score_KEGG	Evalue_KEGG
5271	WP_048455896.1	-	helix-turn-helix domain-containing protein	1	0	0	1	1	0	0	1	0	1	0	5	Accessory								
5272	WP_048460716.1	-	helix-turn-helix domain-containing protein	0	1	1	1	0	0	0	1	1	1	0	6	Accessory								
5273	AXI91412.1	-	hypothetical protein	0	0	0	1	0	0	0	0	0	0	0	1	Unique								
5274	Prokka:JAPCLKMA_06203	cutM	Carbon monoxide dehydrogenase medium chain	1	0	0	0	1	1	1	0	0	0	1	5	Accessory	COG1319_C#	44.2	218	4.16E-56	K03519	44.2	218	1.11E-55
5275	WP_033272374.1	-	bifunctional methylenetetrahydrofolate dehydrogenase/methenyltetrahydrofolate cyclohydrolase	1	1	1	1	1	1	1	1	1	1	1	11	CORE	COG0190_H#	68	393	9.44E-109	K01491	68.7	379	2.88E-104
5276	WP_019526837.1	-	alpha/beta hydrolase	0	0	0	0	1	1	0	0	0	0	1	3	Accessory	COG0596_R#	30.2	72	3.69E-12				
5277	Prokka:JAPCLKMA_00908	nit1	Deaminated glutathione amidase	1	0	0	0	1	1	0	0	0	0	1	4	Accessory	COG0388_R#	33.2	114	4.96E-25	NA	26.9	82	9.52E-15

cluster	GI	gene	Annotation	Streptomyces_thermocarboxydus_BPSAC147	Streptomyces_sp._4F	Streptomyces_sp._BSE7F	Streptomyces_sp._ETH9427	Streptomyces_sp._FxanaD5	Streptomyces_sp._UNC401CLCol	Actinospica_acidiphila_NRRL_B_24431	Streptomyces_sp._HNS054	Streptomyces_sp._SMS_SU21	Streptomyces_sp._ZS0098	Streptomyces_thermocarboxydus_K155	Addition	Core-Accessory-Unique	COG	%Similarity_COG	Score_COG	Evalue_COG	KEGG	%Similarity_KEGG	Score_KEGG	Evalue_KEGG
5278	Prokka:CIODPCDJ_04160	-	hypothetical protein	1	0	1	0	0	1	1	1	1	1	1	8	Accessory								
5279	OSC56250.1	-	glycosyl transferase	0	1	0	0	0	0	0	0	0	0	0	1	Unique	COG0438_M#	57	283	6.18E-76	NA	57	283	1.65E-75
5280	WP_102637319.1	-	acyltransferase domain-containing protein	0	0	1	0	0	0	0	0	1	0	0	2	Accessory	COG0331_I#	37.3	165	3.2E-40	K15329	37.3	165	8.55E-40
5281	WP_102637997.1	-	hypothetical protein	0	1	1	0	0	0	0	0	1	1	0	4	Accessory								
5282	Prokka:JAPCLKMA_04006	allR	HTH-type transcriptional repressor AllR	1	1	1	1	1	1	1	1	1	1	1	11	CORE	COG1414_K#	32	143	1.3E-33	K10973	34.6	138	1.12E-31
5283	WP_102637808.1	-	integrase	1	0	0	1	1	1	1	1	1	0	0	7	Accessory								
5284	AXI91348.1	-	hypothetical protein	0	0	0	1	0	0	0	0	0	0	0	1	Unique								
5285	Prokka:JAPCLKMA_05586	hbd_2	3-hydroxybutyryl-CoA dehydrogenase	1	0	0	0	0	0	0	0	0	0	0	1	Unique	COG1250_I#	40.1	186	1.34E-46	K00074	39.3	196	4.52E-49
5286	WP_122213325.1	-	MBL fold metallo-hydrolase	0	1	0	0	0	0	1	1	0	1	0	4	Accessory								
5287	WP_033275541.1	-	NERD domain-containing protein	0	0	0	0	0	0	1	1	0	0	0	2	Accessory								
5288	Prokka:JAPCLKMA_05975	-	hypothetical protein	1	0	0	0	0	0	0	0	0	0	0	1	Unique								
5289	OSC65099.1	-	hypothetical protein	0	1	0	0	0	0	0	0	0	0	0	1	Unique	COG0149_G#	32.9	127	9.66E-29	K01803	30.6	121	1.08E-26

cluster	GI	gene	Annotation	Streptomyces_thermocarboxydus_BPSAC147	Streptomyces_sp._4F	Streptomyces_sp._BSE7F	Streptomyces_sp._ETH9427	Streptomyces_sp._FxanaD5	Streptomyces_sp._UNC401CLCol	Actinospica_acidiphila_NRRL_B_24431	Streptomyces_sp._HNS054	Streptomyces_sp._SMS_SU21	Streptomyces_sp._ZS0098	Streptomyces_thermocarboxydus_K155	Addition	Core-Accessory-Unique	COG	%Similarity_COG	Score_COG	Evalue_COG	KEGG	%Similarity_KEGG	Score_KEGG	Evalue_KEGG
5290	WP_122213771.1	-	hypothetical protein	0	0	0	0	0	0	0	0	0	1	0	1	Unique								
5291	OSC64761.1	-	ECF transporter S component	1	1	1	1	1	1	1	1	1	1	1	11	CORE								
5292	OSC72305.1	-	transcriptional regulator	0	1	0	0	0	0	0	0	0	0	0	1	Unique								
5293	Prokka:JAPCLKMA_01295	ngcG_2	Diacetylchitobiose uptake system permease protein NgcG	1	0	1	1	1	1	1	1	1	1	1	10	Accessory	COG0395_G#	38.2	211	2.97E-54	K02026	34.8	162	7.21E-39
5294	WP_048457866.1	-	helix-turn-helix domain-containing protein	1	1	1	1	0	0	0	1	1	1	1	8	Accessory								
5295	AXI87580.1	-	hypothetical protein	1	1	1	1	1	1	1	1	1	1	1	11	CORE								
5296	AXI89633.1	-	hypothetical protein	0	0	0	1	0	0	0	0	0	0	0	1	Unique								
5297	OSC72200.1	-	hypothetical protein	0	1	0	0	0	0	0	0	0	0	0	1	Unique								
5298	WP_122217164.1	-	SMP-30/gluconolactonase/LRE family protein	1	1	1	1	1	1	1	1	1	1	1	11	CORE	COG3386_G#	43.1	211	5.07E-54	NA	33.4	154	1.15E-36

cluster	GI	gene	Annotation	Streptomyces_thermocarboxydus_BPSAC147	Streptomyces_sp._4F	Streptomyces_sp._BSE7F	Streptomyces_sp._ETH9427	Streptomyces_sp._FxanaD5	Streptomyces_sp._UNC401CLCol	Actinospica_acidiphila_NRRL_B_24431	Streptomyces_sp._HNS054	Streptomyces_sp._SMS_SU21	Streptomyces_sp._ZS0098	Streptomyces_thermocarboxydus_K155	Addition	Core-Accessory-Unique	COG	%Similarity_COG	Score_COG	Evalue_COG	KEGG	%Similarity_KEGG	Score_KEGG	Evalue_KEGG
5299	WP_102637343.1	-	sigma-70 family RNA polymerase sigma factor	1	1	1	1	1	1	1	1	1	1	1	11	CORE	COG1595_K#	60.7	279	1.52E-74	K03088	62.4	275	4.48E-73
5300	Prokka:CIODPCDJ_04575	-	hypothetical protein	1	0	0	0	0	1	1	1	1	1	1	7	Accessory								
5301	AXI91314.1	-	hypothetical protein	0	0	0	1	0	0	0	0	0	0	0	1	Unique								
5302	OSC59426.1	-	hypothetical protein	1	1	1	1	1	1	1	1	1	1	1	11	CORE								
5303	OSC68336.1	-	B/F/G family RNA polymerase sigma-70 factor	1	1	1	1	1	1	1	1	1	1	1	11	CORE	COG1191_K#	44	214	4.59E-55	K03090	44	214	1.22E-54
5304	OSC57396.1	-	multidrug ABC transporter permease	0	1	0	0	0	0	0	0	0	0	0	1	Unique	COG0842_V#	25.1	47	0.0000742				
5305	Prokka:JAPCLKMA_02004	phzF_1	Trans-2,3-dihydro-3-hydroxyanthranilate isomerase	1	0	0	0	0	0	0	0	0	0	0	1	Unique	COG0384_R#	58.4	327	4.85E-89	K06998	35.5	136	4.24E-31
5306	WP_048457004.1	-	sigma-70 family RNA polymerase sigma factor	1	0	0	1	1	1	1	1	1	0	0	7	Accessory								
5307	OSC63507.1	-	hypothetical protein	1	1	1	1	1	1	1	1	1	1	1	11	CORE								

cluster	GI	gene	Annotation	Streptomyces_thermocarboxydus_BPSAC147	Streptomyces_sp._4F	Streptomyces_sp._BSE7F	Streptomyces_sp._ETH9427	Streptomyces_sp._FxanaD5	Streptomyces_sp._UNC401CLCol	Actinospica_acidiphila_NRRL_B_24431	Streptomyces_sp._HNS054	Streptomyces_sp._SMS_SU21	Streptomyces_sp._ZS0098	Streptomyces_thermocarboxydus_K155	Addition	Core-Accessory-Unique	COG	%Similarity_COG	Score_COG	Evalue_COG	KEGG	%Similarity_KEGG	Score_KEGG	Evalue_KEGG
5308	OSC66253.1	-	TetR family transcriptional regulator	0	1	0	0	0	0	0	0	0	0	0	1	Unique	COG1309_K#	22.8	54	6.07E-07	NA	27.5	51	0.0000137
5309	WP_019523065.1	-	phytanoyl-CoA dioxygenase	1	1	0	0	1	1	1	0	0	0	1	6	Accessory								
5310	WP_033273178.1	-	helix-turn-helix domain-containing protein	0	0	0	0	1	1	1	0	0	0	1	4	Accessory	COG1396_K#	39.5	156	1.13E-37				
5311	PWE12381.1	-	glutamate racemase	1	1	1	1	1	1	1	1	1	1	1	11	CORE	COG0796_M#	28.6	84	7.16E-16				
5312	WP_019523515.1	-	serine hydrolase	0	0	0	0	1	1	0	0	0	0	1	3	Accessory	COG1680_V#	35.2	97	8.17E-20	K18988	35	89	4.55E-17
5313	Prokka:CIODPCDJ_01019	-	hypothetical protein	1	0	1	1	1	1	1	1	1	1	1	10	Accessory	COG0702_MG#	34.7	109	2.71E-23	NA	27.6	65	7.03E-10
5314	OSC57222.1	-	hydroxylase	0	1	0	0	0	0	0	0	0	0	0	1	Unique	COG3324_R#	39.3	170	9.92E-42	K06996	39.3	170	2.65E-41
5315	Prokka:JAPCLKMA_02741	-	hypothetical protein	1	0	0	0	0	0	0	0	0	0	0	1	Unique	COG5551_S#	28.6	82	2.72E-15	NA	28.6	82	7.26E-15
5316	AXI86451.1	-	RimK family alpha-L-glutamate ligase	0	0	0	1	0	0	0	1	0	0	0	2	Accessory	COG0189_HJ#	27.6	99	2.15E-20	K05844	27.9	79	8.03E-14
5317	OSC72252.1	-	hypothetical protein	0	1	0	0	0	0	0	0	0	0	0	1	Unique								
5318	Prokka:JAPCLKMA_05498	-	hypothetical protein	1	0	0	0	0	0	0	0	0	0	0	1	Unique					NA	32.1	109	5.55E-23

cluster	GI	gene	Annotation	Streptomyces_thermocarboxydus_BPSAC147	Streptomyces_sp._4F	Streptomyces_sp._BSE7F	Streptomyces_sp._ETH9427	Streptomyces_sp._FxanaD5	Streptomyces_sp._UNC401CLCol	Actinospica_acidiphila_NRRL_B_24431	Streptomyces_sp._HNS054	Streptomyces_sp._SMS_SU21	Streptomyces_sp._ZS0098	Streptomyces_thermocarboxydus_K155	Addition	Core-Accessory-Unique	COG	%Similarity_COG	Score_COG	Evalue_COG	KEGG	%Similarity_KEGG	Score_KEGG	Evalue_KEGG
5319	OSC71387.1	-	carbon-nitrogen hydrolase family protein	0	1	0	0	0	0	0	0	0	0	0	1	Unique	COG0388_R#	28.1	108	3.54E-23	K08590	28	97	1.67E-19
5320	OSC71211.1	-	universal stress protein	1	1	0	0	0	1	0	0	0	0	1	4	Accessory	COG0589_T#	30.3	105	3.92E-22	NA	30.3	105	1.05E-21
5321	PWE07563.1	-	hypothetical protein	0	0	1	0	0	0	0	0	0	0	0	1	Unique	COG3210_U#	27	41	0.00532				
5322	WP_102639590.1	-	class I SAM-dependent methyltransferase	1	1	0	1	1	1	1	1	1	1	1	10	Accessory	COG0500_QR#	56.1	282	1.37E-75	NA	55.7	280	2.37E-74
5323	OSC69120.1	-	hypothetical protein	0	1	0	0	0	0	0	0	0	0	0	1	Unique								
5324	OSC63176.1	-	sugar ABC transporter permease	1	1	1	1	1	1	1	1	1	1	1	11	CORE	COG0395_G#	36	188	4.6E-47	K02026	32.7	168	1.01E-40
5325	OSC62354.1	-	hypothetical protein	0	1	0	0	1	1	0	0	0	0	1	4	Accessory	COG0730_R#	44.2	177	6.21E-44				
5326	WP_122215710.1	-	LysR family transcriptional regulator	0	0	0	0	0	0	0	0	0	1	0	1	Unique	COG0583_K#	29.9	95	3.1E-19	NA	27.4	86	6.57E-16
5327	WP_102637970.1	-	AraC family transcriptional regulator	1	1	1	1	1	1	1	1	1	1	1	11	CORE	COG2207_K#	34.6	146	1.17E-34	NA	24.5	75	1.16E-12
5328	WP_019523709.1	-	ATP phosphoribosyltransferase	1	1	1	1	1	1	1	1	1	1	1	11	CORE	COG0040_E#	56.3	315	1.9E-85	K00765	56.3	315	5.08E-85

cluster	GI	gene	Annotation	Streptomyces_thermocarboxydus_BPSAC147	Streptomyces_sp._4F	Streptomyces_sp._BSE7F	Streptomyces_sp._ETH9427	Streptomyces_sp._FxanaD5	Streptomyces_sp._UNC401CLCol	Actinospica_acidiphila_NRRL_B_24431	Streptomyces_sp._HNS054	Streptomyces_sp._SMS_SU21	Streptomyces_sp._ZS0098	Streptomyces_thermocarboxydus_K155	Addition	Core-Accessory-Unique	COG	%Similarity_COG	Score_COG	Evalue_COG	KEGG	%Similarity_KEGG	Score_KEGG	Evalue_KEGG
5329	WP_122214343.1	-	ABC transporter permease	1	1	1	1	1	1	1	1	1	1	1	11	CORE	COG0842_V#	33.3	145	3.4E-34	K01992	25.3	77	1.78E-13
5330	WP_026244099.1	-	hypothetical protein	1	1	1	1	1	1	1	1	1	1	1	11	CORE								
5331	WP_122212650.1	-	DUF1707 domain-containing protein	1	1	1	1	1	1	1	1	1	1	1	11	CORE					NA	39.9	146	3.12E-34
5332	WP_019521723.1	fdhD	formate dehydrogenase accessory sulfurtransferase FdhD	1	1	1	1	1	1	1	1	1	1	1	11	CORE	COG1526_C#	58.3	306	1.15E-82	K02379	58.3	306	3.08E-82
5333	WP_019524265.1	-	hypothetical protein	1	0	0	1	1	1	1	0	0	0	1	6	Accessory	COG1511_S#	47.3	48	0.0000566				
5334	Prokka:CIODPCDJ_03828	mqnA	Chorismate dehydratase	1	1	1	1	1	1	1	1	1	1	1	11	CORE	COG1427_R#	31.3	132	2.98E-30	K11782	30.5	99	4.38E-20
5335	WP_019524074.1	-	3-hydroxyacyl-CoA dehydrogenase family protein	1	1	1	1	1	1	1	1	1	1	1	11	CORE	COG1250_I#	47.5	259	1.24E-68	K00074	44.6	251	1.18E-65
5336	WP_048460885.1	-	GNAT family N-acetyltransferase	1	1	1	0	1	1	1	1	1	1	1	10	Accessory	COG3393_R#	49.1	238	2.95E-62	K06976	49.1	238	7.89E-62
5337	OSC63244.1	-	hypothetical protein	1	1	1	1	1	1	1	1	1	1	1	11	CORE								
5338	WP_102637524.1	-	oxidoreductase	0	0	0	1	0	0	0	1	1	0	0	3	Accessory	COG1028_IQR#	62.8	339	9.42E-93	NA	38.4	189	4.2E-47

cluster	GI	gene	Annotation	Streptomyces_thermocarboxydus_BPSAC147	Streptomyces_sp._4F	Streptomyces_sp._BSE7F	Streptomyces_sp._ETH9427	Streptomyces_sp._FxanaD5	Streptomyces_sp._UNC401CLCol	Actinospica_acidiphila_NRRL_B_24431	Streptomyces_sp._HNS054	Streptomyces_sp._SMS_SU21	Streptomyces_sp._ZS0098	Streptomyces_thermocarboxydus_K155	Addition	Core-Accessory-Unique	COG	%Similarity_COG	Score_COG	Evalue_COG	KEGG	%Similarity_KEGG	Score_KEGG	Evalue_KEGG
5339	WP_019522159.1	-	transporter substrate-binding domain-containing protein	1	0	1	1	1	1	1	1	1	1	1	10	Accessory	COG0834_ET#	32.6	134	6.01E-31	K02030	34.9	123	2.83E-27
5340	WP_102637807.1	-	helix-turn-helix transcriptional regulator	1	0	0	1	1	1	1	1	1	0	0	7	Accessory	COG1396_K#	40.6	54	0.00000079	NA	41.4	44	0.00218
5341	PWE11551.1	-	alpha/beta hydrolase	0	1	1	1	0	0	1	1	1	1	0	7	Accessory	COG1506_E#	24.5	50	0.0000114	K01303	24.5	50	0.0000305
5342	OSC69554.1	-	inositol monophosphatase	1	1	1	0	1	1	1	1	1	1	1	10	Accessory	COG0483_G#	32.8	118	3.41E-26	K01092	32.8	118	9.11E-26
5343	OSC67845.1	-	TetR family transcriptional regulator	1	1	1	1	0	0	1	1	1	1	0	8	Accessory	COG1309_K#	34	43	0.0014				
5344	OSC57693.1	-	hypothetical protein	0	1	0	0	0	0	0	0	0	0	0	1	Unique	COG0330_O#	50.8	247	6.36E-65	NA	49.8	247	1.7E-64
5345	WP_033277968.1	-	tryptophan 2,3-dioxygenase	1	1	1	1	1	1	1	1	1	1	1	11	CORE	COG3483_E#	38	184	3.88E-46	K00453	38	184	1.04E-45
5346	OSC70031.1	-	transcriptional regulator	0	1	0	0	0	0	0	0	0	0	0	1	Unique								
5347	WP_122213369.1	-	carbohydrate ABC transporter permease	0	0	0	0	0	0	0	0	0	1	0	1	Unique	COG0395_G#	40.8	224	5.77E-58	K02026	35.6	173	4.07E-42
5348	OSC69076.1	-	ion transporter	0	1	1	1	0	0	0	1	1	1	0	6	Accessory	COG1226_P#	30.3	126	1.64E-28	K10716	27.1	93	4.1E-18

cluster	GI	gene	Annotation	Streptomyces_thermocarboxydus_BPSAC147	Streptomyces_sp._4F	Streptomyces_sp._BSE7F	Streptomyces_sp._ETH9427	Streptomyces_sp._FxanaD5	Streptomyces_sp._UNC401CLCol	Actinospica_acidiphila_NRRL_B_24431	Streptomyces_sp._HNS054	Streptomyces_sp._SMS_SU21	Streptomyces_sp._ZS0098	Streptomyces_thermocarboxydus_K155	Addition	Core-Accessory-Unique	COG	%Similarity_COG	Score_COG	Evalue_COG	KEGG	%Similarity_KEGG	Score_KEGG	Evalue_KEGG
5349	WP_048460660.1	-	helix-turn-helix domain-containing protein	0	0	0	0	0	1	0	1	0	0	1	3	Accessory								
5350	OSC70753.1	-	ergot alkaloid biosynthesis protein	0	1	0	0	0	0	0	0	0	0	0	1	Unique	COG0702_MG#	45.4	231	3.61E-60	NA	45.4	231	9.65E-60
5351	WP_159020911.1	-	hypothetical protein	0	0	0	0	0	0	0	1	0	0	0	1	Unique								
5352	OSC70124.1	-	DUF1396 domain-containing protein	0	1	0	0	0	0	0	0	0	0	0	1	Unique								
5353	OSC57156.1	-	ABC transporter permease	0	1	0	0	0	0	0	0	0	0	0	1	Unique	COG0842_V#	29.6	96	2.37E-19	K01992	29.7	87	2.25E-16
5354	OSC59243.1	-	ABC transporter permease	0	1	0	0	0	0	0	0	0	0	0	1	Unique	COG1173_EP#	37.4	187	7.81E-47	K13891	36.3	182	6.71E-45
5355	AXI89609.1	-	class I SAM-dependent methyltransferase	0	0	0	1	0	0	0	0	0	0	0	1	Unique	COG0500_QR#	33.3	55	3.55E-07	NA	33.3	55	9.47E-07
5356	OSC62769.1	-	transcriptional regulator	0	1	0	0	0	0	1	0	0	1	0	3	Accessory	COG1396_K#	35.4	136	2.07E-31				
5357	Prokka:JAPCLKMA_05019	-	Putative thiosulfate sulfurtransferase	1	1	1	1	1	1	1	1	1	1	1	11	CORE	COG2897_P#	72	425	1.7E-118	K01011	72	425	4.54E-118

cluster	GI	gene	Annotation	Streptomyces_thermocarboxydus_BPSAC147	Streptomyces_sp._4F	Streptomyces_sp._BSE7F	Streptomyces_sp._ETH9427	Streptomyces_sp._FxanaD5	Streptomyces_sp._UNC401CLCol	Actinospica_acidiphila_NRRL_B_24431	Streptomyces_sp._HNS054	Streptomyces_sp._SMS_SU21	Streptomyces_sp._ZS0098	Streptomyces_thermocarboxydus_K155	Addition	Core-Accessory-Unique	COG	%Similarity_COG	Score_COG	Evalue_COG	KEGG	%Similarity_KEGG	Score_KEGG	Evalue_KEGG
5358	AXI86914.1	-	XRE family transcriptional regulator	1	0	1	1	1	0	1	0	1	1	0	7	Accessory	COG1396_K#	36	42	0.00404				
5359	Prokka:CIODPCDJ_04592	prmC_4	Release factor glutamine methyltransferase	1	1	1	1	1	1	1	1	1	1	1	11	CORE	COG2890_J#	43.5	208	3.26E-53	K02493	43.5	208	8.71E-53
5360	WP_048460308.1	-	DegV family protein	1	1	1	1	1	1	1	1	1	1	1	11	CORE	COG1307_S#	38	173	1.16E-42	NA	38	173	3.11E-42
5361	OSC65619.1	-	orotidine-5'-phosphate decarboxylase	1	1	1	1	1	1	1	1	1	1	1	11	CORE	COG0284_F#	50.2	252	1.51E-66	K01591	52	245	6.43E-64
5362	Prokka:JAPCLKMA_00652	paaX	Transcriptional repressor PaaX	1	1	1	1	1	1	1	1	1	1	1	11	CORE	COG3327_K#	29.1	131	6.62E-30				
5363	OSC62821.1	-	proteasome subunit beta	1	1	1	1	1	1	1	1	1	1	1	11	CORE	COG0638_O#	51.6	259	1.61E-68	K03433	51.6	259	4.3E-68
5364	WP_122214077.1	-	GntR family transcriptional regulator	1	1	1	1	1	1	1	1	1	1	1	11	CORE	COG1802_K#	30.5	90	9.92E-18	NA	30.3	80	3.58E-14
5365	WP_122216784.1	-	oxidoreductase	1	1	1	1	1	1	1	1	1	1	1	11	CORE								
5366	OSC70551.1	-	transcriptional regulator	0	1	0	0	0	0	0	0	0	0	0	1	Unique	COG1396_K#	38.7	150	1.05E-35				
5367	WP_102642006.1	-	DUF4397 domain-containing protein	1	0	1	1	1	1	1	1	1	1	1	10	Accessory								

cluster	GI	gene	Annotation	Streptomyces_thermocarboxydus_BPSAC147	Streptomyces_sp._4F	Streptomyces_sp._BSE7F	Streptomyces_sp._ETH9427	Streptomyces_sp._FxanaD5	Streptomyces_sp._UNC401CLCol	Actinospica_acidiphila_NRRL_B_24431	Streptomyces_sp._HNS054	Streptomyces_sp._SMS_SU21	Streptomyces_sp._ZS0098	Streptomyces_thermocarboxydus_K155	Addition	Core-Accessory-Unique	COG	%Similarity_COG	Score_COG	Evalue_COG	KEGG	%Similarity_KEGG	Score_KEGG	Evalue_KEGG
5368	WP_048458595.1	-	hypothetical protein	0	0	0	0	0	0	0	1	0	0	0	1	Unique								
5369	WP_102638351.1	-	AAA family ATPase	1	0	0	1	1	1	1	1	1	1	1	9	Accessory					NA	36.1	62	7.72E-09
5370	WP_019526512.1	-	hypothetical protein	1	0	0	0	1	0	1	0	0	0	0	3	Accessory								
5371	Prokka:JAPCLKMA_05482	-	hypothetical protein	1	0	0	0	0	0	0	0	0	0	0	1	Unique								
5372	OSC61501.1	-	NADPH-dependent ferric siderophore reductase	1	1	1	1	1	1	1	1	1	1	1	11	CORE	COG2375_P#	43.3	222	2.18E-57	NA	45.8	220	2.22E-56
5373	OSC66194.1	-	hypothetical protein	0	1	0	0	0	0	0	0	0	0	0	1	Unique	COG1807_M#	41.4	199	1.52E-50	NA	41.4	199	4.05E-50
5374	Prokka:CIODPCDJ_00549	COQ5_2	2-methoxy-6-polyprenyl-1,4-benzoquinol methylase, mitochondrial	1	1	1	1	1	1	1	1	1	1	1	11	CORE	COG0500_QR#	35.3	63	1.7E-09	K03183	34.8	61	1.72E-08
5375	WP_026243957.1	-	NAD(P)H-binding protein	1	0	1	0	1	1	1	0	1	1	1	8	Accessory	COG0702_MG#	31.2	112	1.87E-24				
5376	WP_146217804.1	-	hypothetical protein	0	0	0	0	0	0	0	0	1	0	0	1	Unique								
5377	OSC64987.1	-	aquaporin	0	1	0	0	0	0	0	0	0	0	0	1	Unique	COG0580_G#	44.5	200	6.8E-51	K02440	44.5	200	1.82E-50
5378	PWE08582.1	-	hypothetical protein	1	0	1	1	0	0	0	0	0	0	0	3	Accessory								

cluster	GI	gene	Annotation	Streptomyces_thermocarboxydus_BPSAC147	Streptomyces_sp._4F	Streptomyces_sp._BSE7F	Streptomyces_sp._ETH9427	Streptomyces_sp._FxanaD5	Streptomyces_sp._UNC401CLCol	Actinospica_acidiphila_NRRL_B_24431	Streptomyces_sp._HNS054	Streptomyces_sp._SMS_SU21	Streptomyces_sp._ZS0098	Streptomyces_thermocarboxydus_K155	Addition	Core-Accessory-Unique	COG	%Similarity_COG	Score_COG	Evalue_COG	KEGG	%Similarity_KEGG	Score_KEGG	Evalue_KEGG
5379	Prokka:CIODPCDJ_01234	-	hypothetical protein	0	0	0	0	0	0	0	0	0	0	1	1	Unique								
5380	PWE08616.1	-	HAD-IB family hydrolase	1	1	1	1	1	1	1	1	1	1	1	11	CORE	COG0560_E#	60.5	325	2.39E-88	NA	60.5	325	6.39E-88
5381	WP_033274287.1	-	MBL fold metallo-hydrolase	0	0	0	0	0	0	1	0	0	0	0	1	Unique	COG0491_R#	29.7	111	5.43E-24	K01069	27.5	50	0.0000397
5382	Prokka:CIODPCDJ_00984	-	Putative hydro-lyase	1	1	1	1	1	1	1	1	1	1	1	11	CORE	COG4336_S#	58.8	320	5.89E-87	NA	52.7	283	2.78E-75
5383	AXI85579.1	-	acyltransferase	1	1	1	1	1	1	1	1	1	1	1	11	CORE	COG0110_R#	37.6	91	7.59E-18	NA	36.2	90	3.46E-17
5384	OSC70338.1	-	hypothetical protein	0	1	0	0	0	0	0	0	0	0	0	1	Unique	COG3208_Q#	31.7	122	3.08E-27	NA	26.5	94	2.4E-18
5385	OSC72876.1	-	sirohydrochlorin chelatase	1	1	1	1	1	1	1	1	1	1	1	11	CORE	COG2138_S#	36.6	122	3.08E-27	NA	36.6	122	8.21E-27
5386	OSC72849.1	-	EamA family transporter	0	1	0	0	0	0	0	0	0	0	0	1	Unique	COG0697_GER#	39.6	191	3.16E-48				
5387	WP_122217302.1	-	hypothetical protein	1	0	1	0	0	1	1	1	1	1	1	8	Accessory								
5388	OSC62907.1	-	acyltransferase	1	1	1	1	1	1	1	1	1	1	1	11	CORE	COG0388_R#	40.6	198	4.39E-50	K12251	40.4	202	4.76E-51
5389	Prokka:JAPCLKMA_03732	-	hypothetical protein	1	0	0	0	1	1	1	0	0	0	1	5	Accessory								
5390	WP_033273461.1	-	class II fructose-bisphosphate aldolase family protein	1	1	1	1	1	1	1	1	1	1	1	11	CORE	COG0191_G#	39	201	3.97E-51	K08302	39	201	1.06E-50
5391	OSC73233.1	-	hypothetical protein	0	1	0	0	0	0	0	0	0	0	0	1	Unique	COG5651_N#	36	78	5.07E-14	NA	41.8	111	1.11E-23

cluster	GI	gene	Annotation	Streptomyces_thermocarboxydus_BPSAC147	Streptomyces_sp._4F	Streptomyces_sp._BSE7F	Streptomyces_sp._ETH9427	Streptomyces_sp._FxanaD5	Streptomyces_sp._UNC401CLCol	Actinospica_acidiphila_NRRL_B_24431	Streptomyces_sp._HNS054	Streptomyces_sp._SMS_SU21	Streptomyces_sp._ZS0098	Streptomyces_thermocarboxydus_K155	Addition	Core-Accessory-Unique	COG	%Similarity_COG	Score_COG	Evalue_COG	KEGG	%Similarity_KEGG	Score_KEGG	Evalue_KEGG
5392	WP_102641611.1	-	methyltransferase domain-containing protein	0	0	1	1	0	0	0	1	1	1	0	5	Accessory	COG0500_QR#	32.3	131	5.05E-30	K05928	31.2	121	1.4E-26
5393	WP_019523442.1	-	polysaccharide deacetylase family protein	1	1	1	0	1	1	1	1	1	1	1	10	Accessory	COG0726_G#	26.6	86	1.86E-16				
5394	AXI88994.1	-	RNA polymerase sigma factor WhiG	1	1	1	1	1	1	1	1	1	1	1	11	CORE	COG1191_K#	49	245	2.4E-64	K02405	43	188	7.12E-47
5395	WP_102637245.1	-	helix-turn-helix domain-containing protein	1	1	1	1	1	1	1	1	1	1	1	11	CORE	COG1426_S#	37.8	62	2.21E-09				
5396	Prokka:CIODPCDJ_02649	-	hypothetical protein	1	1	1	1	1	1	1	1	1	1	1	11	CORE	COG1737_K#	25.1	90	1.29E-17	NA	27.3	54	0.0000021
5397	AXI85329.1	-	MarR family transcriptional regulator	1	0	0	1	0	0	0	0	0	0	0	2	Accessory								
5398	Prokka:CIODPCDJ_02771	mtnP	S-methyl-5'-thioadenosine phosphorylase	1	1	1	1	1	1	1	1	1	1	1	11	CORE	COG0005_F#	50.9	264	4.99E-70	K00772	50.9	264	1.33E-69
5399	WP_019521492.1	-	enoyl-CoA hydratase/isomerase family protein	1	0	0	0	1	1	0	0	0	0	1	4	Accessory	COG1024_I#	31.6	112	3.17E-24	K15016	30.3	100	2.55E-20

cluster	GI	gene	Annotation	Streptomyces_thermocarboxydus_BPSAC147	Streptomyces_sp._4F	Streptomyces_sp._BSE7F	Streptomyces_sp._ETH9427	Streptomyces_sp._FxanaD5	Streptomyces_sp._UNC401CLCol	Actinospica_acidiphila_NRRL_B_24431	Streptomyces_sp._HNS054	Streptomyces_sp._SMS_SU21	Streptomyces_sp._ZS0098	Streptomyces_thermocarboxydus_K155	Addition	Core-Accessory-Unique	COG	%Similarity_COG	Score_COG	Evalue_COG	KEGG	%Similarity_KEGG	Score_KEGG	Evalue_KEGG
5400	WP_102641438.1	-	4'-phosphopantetheinyl transferase superfamily protein	0	0	1	1	0	0	0	1	1	1	0	5	Accessory								
5401	WP_078611412.1	-	hypothetical protein	1	1	1	1	1	1	1	1	1	1	1	11	CORE	COG1579_R#	36.5	141	4.88E-33	K07164	36.5	141	1.3E-32
5402	Prokka:JAPCLKMA_03772	-	hypothetical protein	1	0	0	0	0	0	0	0	0	0	0	1	Unique								
5403	OSC71382.1	-	carbon-nitrogen hydrolase family protein	0	1	0	0	0	0	0	0	0	0	0	1	Unique	COG0388_R#	26.5	92	3.4E-18	K13566	30.1	120	2.38E-26
5404	WP_102639906.1	-	helix-turn-helix domain-containing protein	0	0	0	0	0	0	0	0	1	0	0	1	Unique								
5405	Prokka:CIODPCDJ_04673	-	hypothetical protein	0	0	0	0	0	0	0	0	0	0	1	1	Unique					NA	32.3	43	0.0037
5406	AXI91268.1	-	hypothetical protein	0	0	0	1	0	0	0	0	0	0	0	1	Unique								
5407	PWE10210.1	-	gas vesicle protein	1	1	1	1	1	1	1	1	1	1	1	11	CORE								
5408	PWE06885.1	-	enoyl-CoA hydratase	1	1	1	1	1	1	1	1	1	1	1	11	CORE	COG1024_I#	34.4	109	2.68E-23	K13767	31.1	98	1.27E-19
5409	WP_028959119.1	ribA	GTP cyclohydrolase II RibA	1	0	0	0	1	1	0	0	0	0	1	4	Accessory	COG0807_H#	48.2	203	1.37E-51	K14652	48.6	158	1.35E-37

cluster	GI	gene	Annotation	Streptomyces_thermocarboxydus_BPSAC147	Streptomyces_sp._4F	Streptomyces_sp._BSE7F	Streptomyces_sp._ETH9427	Streptomyces_sp._FxanaD5	Streptomyces_sp._UNC401CLCol	Actinospica_acidiphila_NRRL_B_24431	Streptomyces_sp._HNS054	Streptomyces_sp._SMS_SU21	Streptomyces_sp._ZS0098	Streptomyces_thermocarboxydus_K155	Addition	Core-Accessory-Unique	COG	%Similarity_COG	Score_COG	Evalue_COG	KEGG	%Similarity_KEGG	Score_KEGG	Evalue_KEGG
5410	Prokka:CIODPCDJ_00844	mshC_1	L-cysteine:1D-myo-inositol 2-amino-2-deoxy-alpha-D-glucopyranoside ligase	1	1	1	0	1	1	1	1	1	1	1	10	Accessory								
5411	WP_048455054.1	-	carbohydrate ABC transporter permease	0	0	1	0	0	0	0	1	1	1	0	4	Accessory	COG0395_G#	33.9	184	5.03E-46	K17236	31.8	158	1.35E-37
5412	PWE08590.1	-	PE-PGRS family protein	0	0	1	0	0	0	0	0	0	0	0	1	Unique								
5413	WP_033274327.1	-	patatin-like phospholipase family protein	1	0	1	1	1	1	1	1	1	1	1	10	Accessory	COG1752_R#	40.3	169	1.67E-41	K07001	40.3	169	4.45E-41
5414	WP_019521757.1	-	methyltransferase domain-containing protein	1	1	1	1	1	1	1	1	1	1	1	11	CORE	COG0500_QR#	59.2	335	1.76E-91	NA	59.2	335	4.69E-91
5415	WP_048457181.1	-	DUF3662 domain-containing protein	1	0	1	1	1	1	1	1	1	1	1	10	Accessory	COG1716_T#	37.8	57	7.07E-08	NA	34.7	72	9.68E-12
5416	Prokka:CIODPCDJ_02795	-	hypothetical protein	1	0	0	0	0	1	0	0	0	0	1	3	Accessory								
5417	Prokka:CIODPCDJ_06138	-	hypothetical protein	1	1	0	1	1	1	1	1	1	1	1	10	Accessory	COG0726_G#	43.6	163	1.56E-39	NA	41.2	143	4.46E-33

cluster	GI	gene	Annotation	Streptomyces_thermocarboxydus_BPSAC147	Streptomyces_sp._4F	Streptomyces_sp._BSE7F	Streptomyces_sp._ETH9427	Streptomyces_sp._FxanaD5	Streptomyces_sp._UNC401CLCol	Actinospica_acidiphila_NRRL_B_24431	Streptomyces_sp._HNS054	Streptomyces_sp._SMS_SU21	Streptomyces_sp._ZS0098	Streptomyces_thermocarboxydus_K155	Addition	Core-Accessory-Unique	COG	%Similarity_COG	Score_COG	Evalue_COG	KEGG	%Similarity_KEGG	Score_KEGG	Evalue_KEGG
5418	WP_048455513.1	-	NAD(P)H-binding protein	1	1	1	1	0	0	1	1	1	1	1	9	Accessory	COG0702_MG#	44.7	230	7.96E-60	NA	28.1	96	3.67E-19
5419	WP_028960659.1	-	IS5 family transposase	1	0	0	0	0	1	0	0	0	0	1	3	Accessory	COG3293_L#	63.4	358	2.53E-98				
5420	OSC56949.1	-	hypothetical protein	0	1	0	0	0	0	0	0	0	0	0	1	Unique								
5421	WP_033275209.1	-	SDR family NAD(P)-dependent oxidoreductase	0	0	1	1	0	0	1	1	1	1	0	6	Accessory	COG1028_IQR#	44.2	216	1.55E-55	NA	38	182	6.64E-45
5422	Prokka:JAPCLKMA_03921	glnH_2	ABC transporter glutamine-binding protein GlnH	1	1	1	1	1	1	1	1	1	1	1	11	CORE	COG0834_ET#	47.9	226	8.8E-59	K10039	39.6	167	2.21E-40
5423	WP_122216350.1	-	aldo/keto reductase	0	0	1	0	0	0	0	0	1	1	0	3	Accessory	COG0656_R#	59.6	325	1.39E-88	NA	52.5	284	1.24E-75
5424	OSC69606.1	-	hydroxypyruvate isomerase	1	1	1	1	1	1	1	1	1	1	1	11	CORE	COG3622_G#	36.5	164	5.37E-40	K01816	34.2	142	5.83E-33
5425	WP_033276319.1	-	SDR family NAD(P)-dependent oxidoreductase	1	1	1	1	1	1	1	1	1	1	1	11	CORE	COG0300_R#	46.7	201	5.17E-51	K07124	38	151	1.64E-35
5426	OSC61538.1	-	transcriptional regulator	0	1	0	0	0	0	0	0	0	0	0	1	Unique								
5427	OSC63225.1	-	gas vesicle protein	0	1	0	0	0	0	0	0	0	0	0	1	Unique								

cluster	GI	gene	Annotation	Streptomyces_thermocarboxydus_BPSAC147	Streptomyces_sp._4F	Streptomyces_sp._BSE7F	Streptomyces_sp._ETH9427	Streptomyces_sp._FxanaD5	Streptomyces_sp._UNC401CLCol	Actinospica_acidiphila_NRRL_B_24431	Streptomyces_sp._HNS054	Streptomyces_sp._SMS_SU21	Streptomyces_sp._ZS0098	Streptomyces_thermocarboxydus_K155	Addition	Core-Accessory-Unique	COG	%Similarity_COG	Score_COG	Evalue_COG	KEGG	%Similarity_KEGG	Score_KEGG	Evalue_KEGG
5428	Prokka:CIODPCDJ_06812	-	hypothetical protein	0	0	0	0	0	0	0	0	0	0	1	1	Unique								
5429	PWE10276.1	-	hypothetical protein	0	0	1	0	0	0	0	0	0	0	0	1	Unique								
5430	AXI91383.1	-	hypothetical protein	0	0	0	1	0	0	0	0	0	0	0	1	Unique								
5431	WP_102640448.1	xth	exodeoxyribonuclease III	1	1	0	1	1	1	1	1	1	1	1	10	Accessory	COG0708_L#	49.6	275	1.65E-73	K01142	49.6	275	4.41E-73
5432	OSC72196.1	-	glucose-1-phosphate thymidylyltransferase	0	1	0	0	0	0	0	0	0	0	0	1	Unique	COG1209_M#	58.2	320	7.64E-87	K00973	54	313	1.91E-84
5433	AXI88998.1	-	elongation factor Ts	1	1	1	1	1	1	1	1	1	1	1	11	CORE	COG0264_J#	56	283	6.05E-76	K02357	56	283	1.61E-75
5434	WP_048458980.1	-	carbohydrate ABC transporter permease	1	0	1	1	1	1	1	1	1	1	1	10	Accessory	COG0395_G#	38.1	221	4.81E-57	K02026	32.9	185	5.98E-46
5435	PWE12177.1	-	alpha/beta hydrolase	0	1	1	1	0	0	0	0	1	0	0	4	Accessory	COG0596_R#	27.8	85	3.16E-16	NA	26	71	1.64E-11
5436	WP_019526958.1	map	type I methionyl aminopeptidase	1	1	1	1	1	1	1	1	1	1	1	11	CORE	COG0024_J#	52.3	261	3.21E-69	K01265	52.3	261	8.58E-69
5437	OSC73076.1	-	undecaprenyl-diphosphatase	0	1	0	0	0	0	0	0	0	0	0	1	Unique	COG1968_V#	46.6	241	3.44E-63	K06153	44.3	222	4.42E-57
5438	PWE08935.1	-	50S ribosomal protein L2	1	1	1	1	1	1	1	1	1	1	1	11	CORE	COG0090_J#	80.9	476	6.36E-134	K02886	80.9	476	1.7E-133
5439	WP_122215925.1	-	hypothetical protein	0	0	0	0	0	0	0	0	0	1	0	1	Unique								

cluster	GI	gene	Annotation	Streptomyces_thermocarboxydus_BPSAC147	Streptomyces_sp._4F	Streptomyces_sp._BSE7F	Streptomyces_sp._ETH9427	Streptomyces_sp._FxanaD5	Streptomyces_sp._UNC401CLCol	Actinospica_acidiphila_NRRL_B_24431	Streptomyces_sp._HNS054	Streptomyces_sp._SMS_SU21	Streptomyces_sp._ZS0098	Streptomyces_thermocarboxydus_K155	Addition	Core-Accessory-Unique	COG	%Similarity_COG	Score_COG	Evalue_COG	KEGG	%Similarity_KEGG	Score_KEGG	Evalue_KEGG
5440	AXI89612.1	-	aminoglycoside phosphotransferase	0	0	0	1	0	0	0	0	0	0	0	1	Unique								
5441	WP_019523602.1	-	hypothetical protein	1	0	0	0	1	1	1	0	0	0	1	5	Accessory								
5442	AXI87528.1	-	peptidase M23	0	0	1	1	0	0	1	1	1	1	0	6	Accessory	COG0739_M#	31.1	72	2.76E-12	NA	45	53	0.00000464
5443	WP_102641728.1	-	amino acid ABC transporter permease	1	0	1	1	1	1	1	1	1	1	1	10	Accessory	COG0765_E#	41.4	168	4.84E-41	K02029	42.6	139	4.92E-32
5444	OSC71755.1	-	AraC family transcriptional regulator	0	1	0	0	0	0	1	0	0	0	0	2	Accessory	COG2207_K#	37.2	159	2.25E-38	NA	21.2	52	0.0000103
5445	WP_102639717.1	-	C40 family peptidase	1	1	1	1	1	1	1	1	1	1	1	11	CORE	COG0791_M#	34.6	104	6.57E-22	NA	31.2	88	1.3E-16
5446	AXI84580.1	-	NAD(P)-dependent oxidoreductase	0	0	0	1	0	0	0	1	0	0	0	2	Accessory	COG0702_MG#	43.5	203	7.95E-52	NA	31.6	117	2.61E-25
5447	WP_048458626.1	-	carbohydrate ABC transporter permease	0	0	0	1	0	0	0	1	1	0	0	3	Accessory	COG0395_G#	43.3	253	1.14E-66	K10119	43.3	253	3.05E-66
5448	Prokka:CIODPCDJ_03764	-	hypothetical protein	1	0	0	0	1	1	0	0	1	0	1	5	Accessory								
5449	AXI90314.1	-	MerR family transcriptional regulator	0	1	0	1	0	0	0	1	0	1	0	4	Accessory	COG0789_K#	47.5	79	2.95E-14	NA	33.6	99	7.37E-20

cluster	GI	gene	Annotation	Streptomyces_thermocarboxydus_BPSAC147	Streptomyces_sp._4F	Streptomyces_sp._BSE7F	Streptomyces_sp._ETH9427	Streptomyces_sp._FxanaD5	Streptomyces_sp._UNC401CLCol	Actinospica_acidiphila_NRRL_B_24431	Streptomyces_sp._HNS054	Streptomyces_sp._SMS_SU21	Streptomyces_sp._ZS0098	Streptomyces_thermocarboxydus_K155	Addition	Core-Accessory-Unique	COG	%Similarity_COG	Score_COG	Evalue_COG	KEGG	%Similarity_KEGG	Score_KEGG	Evalue_KEGG
5450	WP_122214590.1	-	trans-aconitate 2-methyltransferase	1	1	1	1	1	1	1	1	1	1	1	11	CORE	COG4106_R#	43.8	208	4.22E-53	K00598	41.5	192	4.9E-48
5451	OSC70761.1	-	LysR family transcriptional regulator	0	1	0	0	0	0	0	0	0	1	0	2	Accessory	COG0583_K#	30.2	119	1.97E-26	NA	29.4	116	3.41E-25
5452	Prokka:JAPCLKMA_04951	-	hypothetical protein	1	1	0	1	1	1	0	1	1	1	1	9	Accessory	COG0726_G#	28	57	7.05E-08				
5453	AXI88679.1	hisN	histidinol-phosphatase	1	0	1	1	1	1	1	1	1	1	1	10	Accessory	COG0483_G#	56	305	1.94E-82	K05602	55.7	291	7.74E-78
5454	Prokka:JAPCLKMA_00696	hcaB_2	3-phenylpropionate-dihydrodiol/cinnamic acid-dihydrodiol dehydrogenase	1	1	1	1	1	1	1	1	1	1	1	11	CORE	COG0300_R#	34.6	142	2.84E-33	K07124	35	128	8.68E-29
5455	WP_122216919.1	-	DNA-binding protein	1	1	1	1	1	1	1	1	1	1	1	11	CORE	COG1734_T#	34.1	78	3.86E-14	NA	28.5	73	3.31E-12
5456	WP_122214768.1	-	protein kinase	0	0	0	0	0	0	0	0	0	1	0	1	Unique					NA	23.8	50	0.0000392
5457	AXI91214.1	-	hypothetical protein	0	0	0	1	0	0	0	0	0	0	0	1	Unique								
5458	OSC69812.1	-	oxidoreductase	1	1	1	1	1	1	1	1	1	1	1	11	CORE	COG0656_R#	59.9	349	8.97E-96	NA	54.6	294	1.19E-78
5459	OSC66499.1	-	DNA polymerase subunit beta	0	1	0	0	0	0	0	0	0	0	0	1	Unique								

cluster	GI	gene	Annotation	Streptomyces_thermocarboxydus_BPSAC147	Streptomyces_sp._4F	Streptomyces_sp._BSE7F	Streptomyces_sp._ETH9427	Streptomyces_sp._FxanaD5	Streptomyces_sp._UNC401CLCol	Actinospica_acidiphila_NRRL_B_24431	Streptomyces_sp._HNS054	Streptomyces_sp._SMS_SU21	Streptomyces_sp._ZS0098	Streptomyces_thermocarboxydus_K155	Addition	Core-Accessory-Unique	COG	%Similarity_COG	Score_COG	Evalue_COG	KEGG	%Similarity_KEGG	Score_KEGG	Evalue_KEGG
5460	OSC66144.1	-	hypothetical protein	0	1	0	0	0	0	0	0	0	0	0	1	Unique								
5461	Prokka:CIODPCDJ_01753	-	hypothetical protein	1	1	1	1	1	1	1	1	1	1	1	11	CORE	COG0204_I#	52.4	243	1.18E-63	K00655	52.4	243	3.16E-63
5462	Prokka:JAPCLKMA_05371	msrP_6	Protein-methionine-sulfoxide reductase catalytic subunit MsrP	1	0	1	1	1	1	1	1	1	1	1	10	Accessory	COG2041_R#	37.2	114	6.34E-25	K07147	37.2	114	1.69E-24
5463	OSC62625.1	-	shikimate dehydrogenase	1	1	0	1	1	1	1	1	1	1	1	10	Accessory	COG0169_E#	42.5	183	1.11E-45	K00014	42.5	183	2.97E-45
5464	Prokka:JAPCLKMA_04888	-	hypothetical protein	1	0	0	0	0	0	0	0	0	0	0	1	Unique								
5465	Prokka:CIODPCDJ_02103	-	Trans,polycis-polyprenyl diphosphate synthase ((2Z,6E)-farnesyl diphosphate specific)	1	1	1	1	1	1	1	1	1	1	1	11	CORE	COG0020_I#	62.3	344	3.76E-94	K14215	62.3	344	1E-93
5466	WP_078611572.1	-	sugar ABC transporter permease	0	0	0	0	0	1	0	0	0	0	0	1	Unique	COG1175_G#	36	172	1.96E-42	K10118	33.8	162	5.41E-39
5467	WP_019526274.1	-	Fpg/Nei family DNA glycosylase	1	1	1	1	1	1	1	1	1	1	1	11	CORE	COG0266_L#	50.4	219	1.82E-56	K05522	50.4	219	4.87E-56

cluster	GI	gene	Annotation	Streptomyces_thermocarboxydus_BPSAC147	Streptomyces_sp._4F	Streptomyces_sp._BSE7F	Streptomyces_sp._ETH9427	Streptomyces_sp._FxanaD5	Streptomyces_sp._UNC401CLCol	Actinospica_acidiphila_NRRL_B_24431	Streptomyces_sp._HNS054	Streptomyces_sp._SMS_SU21	Streptomyces_sp._ZS0098	Streptomyces_thermocarboxydus_K155	Addition	Core-Accessory-Unique	COG	%Similarity_COG	Score_COG	Evalue_COG	KEGG	%Similarity_KEGG	Score_KEGG	Evalue_KEGG
5468	AXI86910.1	-	alpha/beta hydrolase	1	0	1	1	1	1	1	1	1	1	1	10	Accessory	COG0596_R#	61.8	361	2.97E-99	K00433	61.8	361	7.93E-99
5469	WP_048459391.1	modB	molybdate ABC transporter permease subunit	1	1	1	1	1	1	1	1	1	1	1	11	CORE	COG4149_P#	49.8	233	7.15E-61	K02018	49.8	233	1.91E-60
5470	OSC61923.1	-	hypothetical protein	0	1	1	0	0	0	0	0	1	1	0	4	Accessory								
5471	Prokka:JAPCLKMA_04566	trmD	tRNA (guanine-N(1)-)-methyltransferase	1	1	1	1	1	1	1	1	1	1	1	11	CORE	COG0336_J#	58.1	271	4.04E-72	K00554	56.8	262	6.55E-69
5472	WP_019522450.1	-	AraC family transcriptional regulator	1	1	1	0	1	1	1	1	1	1	1	10	Accessory	COG2207_K#	38	181	3.22E-45	NA	29.4	114	1.69E-24
5473	Prokka:JAPCLKMA_00809	sugB_1	Trehalose transport system permease protein SugB	1	0	1	1	1	1	1	1	1	1	1	10	Accessory	COG0395_G#	73.3	398	2.19E-110	K02026	73.3	398	5.85E-110
5474	PWE08767.1	-	hypothetical protein	0	0	1	0	0	0	0	0	1	0	0	2	Accessory								
5475	Prokka:JAPCLKMA_05710	-	hypothetical protein	1	1	1	1	1	1	1	1	1	1	1	11	CORE					NA	53.2	298	6.31E-80
5476	WP_033273968.1	-	carbohydrate ABC transporter permease	1	1	1	0	1	1	1	0	1	1	1	9	Accessory	COG0395_G#	35.4	186	1.31E-46	K10238	34.4	174	1.37E-42

cluster	GI	gene	Annotation	Streptomyces_thermocarboxydus_BPSAC147	Streptomyces_sp._4F	Streptomyces_sp._BSE7F	Streptomyces_sp._ETH9427	Streptomyces_sp._FxanaD5	Streptomyces_sp._UNC401CLCol	Actinospica_acidiphila_NRRL_B_24431	Streptomyces_sp._HNS054	Streptomyces_sp._SMS_SU21	Streptomyces_sp._ZS0098	Streptomyces_thermocarboxydus_K155	Addition	Core-Accessory-Unique	COG	%Similarity_COG	Score_COG	Evalue_COG	KEGG	%Similarity_KEGG	Score_KEGG	Evalue_KEGG
5477	WP_019523026.1	-	SDR family oxidoreductase	1	0	0	0	1	1	1	0	0	0	1	5	Accessory	COG1028_IQR#	35.8	142	2.17E-33	NA	36.7	136	5.42E-31
5478	OSC59366.1	-	hypothetical protein	0	1	0	0	0	0	0	0	0	0	0	1	Unique								
5479	OSC57672.1	-	SAM-dependent methyltransferase	1	1	1	1	1	1	1	1	1	1	1	11	CORE	COG2890_J#	35	115	3.71E-25	K02493	34.3	110	2.44E-23
5480	OSC63600.1	-	ribonuclease III	1	1	1	1	1	1	1	1	1	1	1	11	CORE	COG0571_K#	59.5	275	2.14E-73	K03685	59.5	275	5.72E-73
5481	Prokka:JAPCLKMA_01428	dppA_1	D-aminopeptidase	1	1	1	1	1	1	1	1	1	1	1	11	CORE	COG2362_E#	38.5	158	2.92E-38	K16203	38.5	158	7.81E-38
5482	Prokka:CIODPCDJ_01257	-	hypothetical protein	1	1	1	1	1	1	1	1	1	1	1	11	CORE					NA	53.2	268	6.99E-71
5483	WP_019527456.1	-	glutamate racemase	0	0	0	0	1	0	0	0	0	0	0	1	Unique	COG0796_M#	53.3	266	1.3E-70	K01776	53.3	266	3.47E-70
5484	WP_033274326.1	-	DUF4037 domain-containing protein	1	0	1	1	1	1	1	1	1	1	1	10	Accessory								
5485	OSC70760.1	-	capsule biosynthesis protein CapD	0	1	0	0	0	0	0	0	0	0	0	1	Unique	COG0702_MG#	34.6	141	6.31E-33	NA	34.6	141	1.68E-32
5486	Prokka:JAPCLKMA_00985	ybfK	Carboxylesterase YbfK	1	0	0	0	0	0	0	0	0	0	0	1	Unique	COG0596_R#	30.4	129	1.9E-29	NA	30.4	129	5.07E-29
5487	OSC72503.1	-	GlcNAc-PI de-N-acetylase	0	1	0	0	0	0	0	0	0	0	0	1	Unique	COG2120_S#	33.9	135	3.46E-31	K18455	32.2	126	3.29E-28
5488	OSC56500.1	-	lyase	0	1	0	0	0	0	0	0	0	0	0	1	Unique								

cluster	GI	gene	Annotation	Streptomyces_thermocarboxydus_BPSAC147	Streptomyces_sp._4F	Streptomyces_sp._BSE7F	Streptomyces_sp._ETH9427	Streptomyces_sp._FxanaD5	Streptomyces_sp._UNC401CLCol	Actinospica_acidiphila_NRRL_B_24431	Streptomyces_sp._HNS054	Streptomyces_sp._SMS_SU21	Streptomyces_sp._ZS0098	Streptomyces_thermocarboxydus_K155	Addition	Core-Accessory-Unique	COG	%Similarity_COG	Score_COG	Evalue_COG	KEGG	%Similarity_KEGG	Score_KEGG	Evalue_KEGG
5489	AXI91252.1	-	phosphogluconate dehydrogenase	0	0	0	1	0	0	0	0	0	0	0	1	Unique								
5490	PWE10532.1	-	cytochrome c oxidase assembly protein	0	0	1	1	0	0	0	1	0	0	0	3	Accessory	COG3336_S#	36.3	160	1E-38	K02351	31.1	103	3.89E-21
5491	OSC61686.1	-	hypothetical protein	1	1	1	1	1	1	1	1	1	1	1	11	CORE	COG2981_E#	27	84	7.01E-16	K06203	25.1	72	5.63E-12
5492	OSC70178.1	-	thioesterase	0	1	0	0	0	0	0	0	0	0	0	1	Unique	COG3208_Q#	40.8	183	1.45E-45	NA	31.9	136	3.18E-31
5493	WP_122216274.1	-	hypothetical protein	0	0	0	0	0	0	0	0	0	1	0	1	Unique								
5494	OSC56633.1	-	IclR family transcriptional regulator	0	1	0	0	0	0	0	0	0	0	0	1	Unique	COG1414_K#	32.1	99	1.61E-20	NA	27.4	69	8.13E-11
5495	OSC73158.1	-	hypothetical protein	0	1	0	0	0	0	0	0	0	0	0	1	Unique								
5496	OSC72782.1	-	peptidylprolyl isomerase	1	1	1	1	1	1	1	1	1	1	1	11	CORE	COG0652_O#	35.8	158	3.82E-38	K03768	35.8	158	1.02E-37
5497	WP_019521916.1	-	helix-turn-helix transcriptional regulator	1	1	0	0	1	1	1	0	0	0	1	6	Accessory	COG2771_K#	43.8	200	1.14E-50				
5498	OSC66858.1	-	alpha/beta hydrolase	1	1	1	1	1	1	1	1	1	1	1	11	CORE	COG0596_R#	73.6	434	2.75E-121	K00433	39.3	197	1.97E-49
5499	WP_048456868.1	-	CoA ester lyase	1	1	1	1	1	1	1	1	1	1	1	11	CORE	COG2301_G#	40.7	161	5.87E-39	K01644	38.7	155	6.58E-37
5500	Prokka:CIODPCDJ_00297	-	hypothetical protein	1	1	1	1	1	1	1	1	1	1	1	11	CORE	COG2208_TK#	27.1	73	1.61E-12	K07315	33.3	52	0.00000785

cluster	GI	gene	Annotation	Streptomyces_thermocarboxydus_BPSAC147	Streptomyces_sp._4F	Streptomyces_sp._BSE7F	Streptomyces_sp._ETH9427	Streptomyces_sp._FxanaD5	Streptomyces_sp._UNC401CLCol	Actinospica_acidiphila_NRRL_B_24431	Streptomyces_sp._HNS054	Streptomyces_sp._SMS_SU21	Streptomyces_sp._ZS0098	Streptomyces_thermocarboxydus_K155	Addition	Core-Accessory-Unique	COG	%Similarity_COG	Score_COG	Evalue_COG	KEGG	%Similarity_KEGG	Score_KEGG	Evalue_KEGG
5501	WP_033276288.1	-	DUF1684 domain-containing protein	1	0	0	0	1	1	1	0	0	0	1	5	Accessory	COG3358_S#	32.9	138	5.32E-32	K09164	39.5	92	8.94E-18
5502	WP_107080854.1	-	thioredoxin domain-containing protein	1	1	1	1	1	1	1	1	1	1	1	11	CORE								
5503	AXI85563.1	-	alpha/beta hydrolase	1	1	1	1	1	1	1	1	1	1	1	11	CORE	COG0596_R#	25.9	89	2.17E-17	NA	28.9	58	0.00000011
5504	WP_033272014.1	-	lysozyme	1	1	1	1	1	1	1	1	1	1	1	11	CORE	COG3757_M#	30	104	8.51E-22	K07273	28.8	86	3.76E-16
5505	Prokka:JAPCLKMA_06617	-	IS3 family transposase ISBli34	1	0	0	0	0	0	0	0	0	0	0	1	Unique	COG2801_L#	49.6	261	4.17E-69	K07497	49.6	261	1.11E-68
5506	OSC62880.1	-	ABC transporter ATP-binding protein	0	1	0	0	0	0	0	0	0	0	0	1	Unique	COG1119_P#	45.6	204	4.63E-52	K02013	45.6	204	1.24E-51
5507	WP_053076957.1	-	hypothetical protein	1	0	0	1	1	1	1	1	1	1	1	9	Accessory								
5508	WP_048455436.1	-	helix-turn-helix domain-containing protein	1	0	1	1	1	1	1	1	1	1	1	10	Accessory								
5509	Prokka:JAPCLKMA_02258	-	hypothetical protein	1	1	1	1	1	1	1	1	1	1	1	11	CORE								
5510	OSC71616.1	-	IclR family transcriptional regulator	0	1	0	0	0	0	0	0	0	0	0	1	Unique	COG1414_K#	54.2	260	7.11E-69	NA	34.4	131	1.33E-29
5511	Prokka:JAPCLKMA_05594	novG	Transcriptional regulator NovG	1	0	0	0	0	0	0	0	0	0	0	1	Unique								

cluster	GI	gene	Annotation	Streptomyces_thermocarboxydus_BPSAC147	Streptomyces_sp._4F	Streptomyces_sp._BSE7F	Streptomyces_sp._ETH9427	Streptomyces_sp._FxanaD5	Streptomyces_sp._UNC401CLCol	Actinospica_acidiphila_NRRL_B_24431	Streptomyces_sp._HNS054	Streptomyces_sp._SMS_SU21	Streptomyces_sp._ZS0098	Streptomyces_thermocarboxydus_K155	Addition	Core-Accessory-Unique	COG	%Similarity_COG	Score_COG	Evalue_COG	KEGG	%Similarity_KEGG	Score_KEGG	Evalue_KEGG
5512	OSC72677.1	-	30S ribosomal protein S3	1	1	1	1	1	1	1	1	1	1	1	11	CORE	COG0092_J#	71.1	379	1.05E-104	K02982	71.1	379	2.8E-104
5513	WP_037954480.1	-	carbohydrate ABC transporter permease	0	0	0	0	0	1	0	0	0	0	0	1	Unique	COG0395_G#	33.7	175	3.01E-43	K10119	32.1	158	7.78E-38
5514	OSC64986.1	-	NAD(+) synthase	0	1	0	0	0	0	0	0	0	0	0	1	Unique	COG0171_H#	56	278	1.93E-74	K01916	56	278	5.16E-74
5515	WP_122216275.1	-	hypothetical protein	0	0	0	0	0	0	0	0	0	1	0	1	Unique								
5516	WP_158693585.1	-	hypothetical protein	0	0	0	0	0	1	0	0	0	0	0	1	Unique								
5517	OSC55300.1	-	hypothetical protein	0	1	0	0	0	0	0	0	0	0	0	1	Unique								
5518	OSC60607.1	-	ABC transporter	1	1	1	1	1	1	1	1	1	1	1	11	CORE	COG0842_V#	32.3	144	4.35E-34	K01992	26.5	72	5.61E-12
5519	WP_033277098.1	-	enoyl-CoA hydratase/isomerase family protein	0	0	0	0	0	0	1	0	0	1	0	2	Accessory	COG1024_I#	32.7	103	1.11E-21	K15016	29.2	89	5.79E-17
5520	Prokka:JAPCLKMA_02824	-	hypothetical protein	1	0	0	0	0	0	0	0	0	0	0	1	Unique								
5521	WP_019525393.1	-	hypothetical protein	0	1	0	0	1	0	0	0	0	0	1	3	Accessory								
5522	WP_033273615.1	-	carbohydrate ABC transporter permease	1	1	1	1	1	1	1	1	1	1	1	11	CORE	COG0395_G#	34	166	1.82E-40	K10238	34	166	4.85E-40
5523	WP_102641442.1	-	enoyl-CoA hydratase family protein	1	1	1	1	1	1	1	1	1	1	1	11	CORE	COG1024_I#	45.1	225	3.3E-58	K15866	36.7	149	6.14E-35

cluster	GI	gene	Annotation	Streptomyces_thermocarboxydus_BPSAC147	Streptomyces_sp._4F	Streptomyces_sp._BSE7F	Streptomyces_sp._ETH9427	Streptomyces_sp._FxanaD5	Streptomyces_sp._UNC401CLCol	Actinospica_acidiphila_NRRL_B_24431	Streptomyces_sp._HNS054	Streptomyces_sp._SMS_SU21	Streptomyces_sp._ZS0098	Streptomyces_thermocarboxydus_K155	Addition	Core-Accessory-Unique	COG	%Similarity_COG	Score_COG	Evalue_COG	KEGG	%Similarity_KEGG	Score_KEGG	Evalue_KEGG
5524	AXI88781.1	atpB	ATP synthase F0 subunit A	1	1	1	1	1	1	1	1	1	1	1	11	CORE	COG0356_C#	35.6	149	1.76E-35	K02108	32.8	118	1.16E-25
5525	WP_078504656.1	-	hypothetical protein	1	1	1	1	1	1	1	0	1	0	1	9	Accessory					K11650	39.5	48	0.000148
5526	OSC69793.1	-	S-adenosyl methyltransferase	1	1	1	1	1	1	1	1	1	1	1	11	CORE								
5527	WP_026244143.1	-	MOSC domain-containing protein	1	0	1	0	1	1	1	1	1	1	1	9	Accessory	COG3217_R#	41.6	195	3.65E-49	NA	34.3	158	1.32E-37
5528	PWE06645.1	-	ABC transporter substrate-binding protein	1	1	1	1	1	1	1	1	1	1	1	11	CORE	COG1464_P#	54.1	262	2.43E-69	K02073	50.2	259	3.23E-68
5529	AXI85262.1	-	hypothetical protein	0	0	0	1	0	0	0	0	0	0	0	1	Unique								
5530	OSC66121.1	-	CPBP family intramembrane metalloprotease domain-containing protein	1	1	1	1	1	1	1	1	1	1	1	11	CORE	COG1266_R#	42.7	172	1.94E-42				
5531	WP_106959460.1	-	HIT family protein	0	0	0	0	0	1	0	0	0	0	0	1	Unique	COG0537_FGR#	41.4	83	1.55E-15	K02503	39.4	79	5.97E-14
5532	Prokka:CIODPCDJ_00626	citT_1	Transcriptional regulatory protein CitT	1	1	1	1	1	1	1	1	1	1	1	11	CORE	COG4565_KT#	40.2	164	5.29E-40	K11692	35.6	148	8.02E-35
5533	OSC72238.1	-	endonuclease	0	1	0	0	0	0	0	0	0	0	0	1	Unique								

cluster	GI	gene	Annotation	Streptomyces_thermocarboxydus_BPSAC147	Streptomyces_sp._4F	Streptomyces_sp._BSE7F	Streptomyces_sp._ETH9427	Streptomyces_sp._FxanaD5	Streptomyces_sp._UNC401CLCol	Actinospica_acidiphila_NRRL_B_24431	Streptomyces_sp._HNS054	Streptomyces_sp._SMS_SU21	Streptomyces_sp._ZS0098	Streptomyces_thermocarboxydus_K155	Addition	Core-Accessory-Unique	COG	%Similarity_COG	Score_COG	Evalue_COG	KEGG	%Similarity_KEGG	Score_KEGG	Evalue_KEGG
5534	OSC64013.1	-	recombination regulator RecX	1	1	1	1	1	1	1	1	1	1	1	11	CORE	COG2137_R#	44.7	120	1.14E-26	K03565	43.3	119	5.21E-26
5535	Prokka:CIODPCDJ_04906	-	hypothetical protein	1	0	0	0	0	0	0	0	0	0	1	2	Accessory								
5536	WP_019525502.1	-	hypothetical protein	0	0	0	0	1	0	0	0	0	0	0	1	Unique								
5537	OSC72089.1	-	hydrolase	1	1	1	1	1	1	1	1	1	1	1	11	CORE	COG0726_G#	38.8	142	2.15E-33	NA	31.6	102	5.05E-21
5538	Prokka:CIODPCDJ_02102	-	hypothetical protein	1	0	0	0	0	1	1	0	0	0	1	4	Accessory								
5539	PWE06623.1	-	hypothetical protein	0	0	1	0	0	0	0	0	0	0	0	1	Unique								
5540	WP_102641019.1	-	GNAT family N-acetyltransferase	1	1	1	1	1	1	1	1	1	1	1	11	CORE	COG0454_KR#	34.7	53	0.00000131				
5541	WP_146217838.1	-	hypothetical protein	0	0	0	0	0	0	0	0	1	0	0	1	Unique					K08912	96.3	526	1.85E-148
5542	OSC56684.1	-	glyoxalase/bleomycin resistance/extradiol dioxygenase family protein	1	1	1	1	1	1	1	1	1	1	1	11	CORE	COG3324_R#	34.6	160	9.97E-39	K06996	34.6	160	2.66E-38
5543	OSC63990.1	-	alpha/beta hydrolase	1	1	1	1	1	1	1	1	1	1	1	11	CORE	COG0596_R#	34.2	154	7.15E-37	NA	34.2	154	1.91E-36
5544	WP_109288695.1	-	hypothetical protein	0	0	0	0	0	0	0	0	1	0	0	1	Unique								

cluster	GI	gene	Annotation	Streptomyces_thermocarboxydus_BPSAC147	Streptomyces_sp._4F	Streptomyces_sp._BSE7F	Streptomyces_sp._ETH9427	Streptomyces_sp._FxanaD5	Streptomyces_sp._UNC401CLCol	Actinospica_acidiphila_NRRL_B_24431	Streptomyces_sp._HNS054	Streptomyces_sp._SMS_SU21	Streptomyces_sp._ZS0098	Streptomyces_thermocarboxydus_K155	Addition	Core-Accessory-Unique	COG	%Similarity_COG	Score_COG	Evalue_COG	KEGG	%Similarity_KEGG	Score_KEGG	Evalue_KEGG
5545	WP_048455940.1	-	purine-nucleoside phosphorylase	1	1	1	1	1	1	1	1	1	1	1	11	CORE	COG0005_F#	62.8	322	1.97E-87	K03783	62.8	322	5.27E-87
5546	PWE08400.1	-	maleylpyruvate isomerase family mycothiol-dependent enzyme	1	1	1	1	1	1	1	1	1	1	1	11	CORE					NA	32.7	114	2.18E-24
5547	PWE08615.1	-	oxidoreductase	1	1	1	1	1	1	1	1	1	1	1	11	CORE					NA	38.1	158	1.01E-37
5548	WP_048460927.1	-	IS110 family transposase	0	0	0	0	0	0	0	1	0	0	0	1	Unique								
5549	Prokka:JAPCLKMA_06512	-	hypothetical protein	1	0	0	0	0	0	0	0	0	0	0	1	Unique								
5550	WP_019521920.1	-	ABC transporter permease	1	1	1	1	1	1	1	1	1	1	1	11	CORE								
5551	AXI89634.1	-	large ATP-binding protein	0	0	0	1	0	0	0	0	0	0	0	1	Unique								
5552	PWE11013.1	-	cupin domain-containing protein	0	0	1	0	0	0	0	0	1	0	0	2	Accessory								
5553	WP_033277424.1	-	hypothetical protein	1	0	1	0	1	1	1	0	1	0	1	7	Accessory								
5554	Prokka:CIODPCDJ_06823	-	hypothetical protein	0	0	0	0	0	0	0	0	0	0	1	1	Unique								

cluster	GI	gene	Annotation	Streptomyces_thermocarboxydus_BPSAC147	Streptomyces_sp._4F	Streptomyces_sp._BSE7F	Streptomyces_sp._ETH9427	Streptomyces_sp._FxanaD5	Streptomyces_sp._UNC401CLCol	Actinospica_acidiphila_NRRL_B_24431	Streptomyces_sp._HNS054	Streptomyces_sp._SMS_SU21	Streptomyces_sp._ZS0098	Streptomyces_thermocarboxydus_K155	Addition	Core-Accessory-Unique	COG	%Similarity_COG	Score_COG	Evalue_COG	KEGG	%Similarity_KEGG	Score_KEGG	Evalue_KEGG
5555	Prokka:CIODPCDJ_03703	-	hypothetical protein	0	0	0	0	0	0	0	0	0	1	1	2	Accessory								
5556	OSC61947.1	-	molybdate ABC transporter substrate-binding protein	1	1	1	1	1	1	1	1	1	1	1	11	CORE	COG0725_P#	43.2	199	1.48E-50	K02020	43.2	199	3.95E-50
5557	Prokka:CIODPCDJ_00552	nanR_2	HTH-type transcriptional repressor NanR	1	0	1	1	1	1	1	1	1	1	1	10	Accessory	COG2186_K#	41.7	181	3.19E-45	K05799	33.6	97	2.11E-19
5558	Prokka:JAPCLKMA_04886	-	hypothetical protein	1	0	0	0	0	0	0	0	0	0	0	1	Unique								
5559	OSC67843.1	-	multidrug ABC transporter permease	1	1	1	1	0	0	1	1	1	1	0	8	Accessory	COG0842_V#	31.8	118	4.33E-26				
5560	WP_026243645.1	-	ABC transporter permease	1	0	0	0	1	1	1	0	0	0	1	5	Accessory	COG1173_EP#	29.1	134	7.6E-31	K02034	30.1	128	8.53E-29
5561	OSC63269.1	-	glycosyltransferase	0	1	0	0	0	0	0	0	0	0	0	1	Unique								
5562	Prokka:JAPCLKMA_04721	nei1	Endonuclease 8 1	1	1	1	1	1	1	1	1	1	1	1	11	CORE	COG0266_L#	52	282	1.73E-75	K05522	50.9	261	8.43E-69
5563	PWE07705.1	-	transporter	1	1	1	1	1	1	1	1	1	1	1	11	CORE	COG3694_R#	34.6	174	6.63E-43	K01992	34.6	174	1.77E-42
5564	WP_019522525.1	-	serine hydrolase	1	1	1	1	1	1	1	1	1	1	1	11	CORE	COG1680_V#	56.5	300	6.13E-81	NA	56.5	300	1.64E-80

cluster	GI	gene	Annotation	Streptomyces_thermocarboxydus_BPSAC147	Streptomyces_sp._4F	Streptomyces_sp._BSE7F	Streptomyces_sp._ETH9427	Streptomyces_sp._FxanaD5	Streptomyces_sp._UNC401CLCol	Actinospica_acidiphila_NRRL_B_24431	Streptomyces_sp._HNS054	Streptomyces_sp._SMS_SU21	Streptomyces_sp._ZS0098	Streptomyces_thermocarboxydus_K155	Addition	Core-Accessory-Unique	COG	%Similarity_COG	Score_COG	Evalue_COG	KEGG	%Similarity_KEGG	Score_KEGG	Evalue_KEGG
5565	WP_019521409.1	-	zinc ABC transporter solute-binding protein	0	0	0	0	1	1	1	0	0	0	1	4	Accessory								
5566	WP_048458916.1	-	SAM-dependent methyltransferase	0	0	1	1	0	0	0	1	1	1	0	5	Accessory								
5567	AXI90215.1	-	hypothetical protein	1	1	1	1	1	1	1	1	1	1	1	11	CORE								
5568	PWE12319.1	-	ABC transporter ATP-binding protein	0	1	1	1	0	0	1	1	1	1	0	7	Accessory	COG1120_PH#	44.7	223	9.52E-58	K02013	44.7	223	2.54E-57
5569	PWE10952.1	-	hypothetical protein	0	1	1	1	0	0	1	1	1	1	0	7	Accessory								
5570	WP_048459119.1	-	anti-sigma factor	1	1	1	1	1	1	1	1	1	1	1	11	CORE								
5571	Prokka:CIODPCDJ_05013	rnhB	Ribonuclease HII	1	1	0	1	1	1	1	1	1	1	1	10	Accessory	COG0164_L#	44.3	148	3.89E-35	K03470	42.5	136	5.34E-31
5572	Prokka:JAPCLKMA_00428	ycel_1	Protein Ycel	1	1	1	1	1	1	1	0	1	1	1	10	Accessory	COG2353_S#	40.8	153	9.27E-37	NA	40	108	1.19E-22
5573	WP_102641020.1	-	aminodeoxychorismate lyase	1	1	1	1	1	1	1	1	1	1	1	11	CORE	COG0115_EH#	35.8	152	2.06E-36	K00826	34.6	141	1.27E-32
5574	WP_019523234.1	-	1-acyl-sn-glycerol-3-phosphate acyltransferase	1	1	1	1	1	1	1	1	1	1	1	11	CORE	COG0204_I#	40.9	107	5.83E-23	NA	30.9	89	5.73E-17

cluster	GI	gene	Annotation	Streptomyces_thermocarboxydus_BPSAC147	Streptomyces_sp._4F	Streptomyces_sp._BSE7F	Streptomyces_sp._ETH9427	Streptomyces_sp._FxanaD5	Streptomyces_sp._UNC401CLCol	Actinospica_acidiphila_NRRL_B_24431	Streptomyces_sp._HNS054	Streptomyces_sp._SMS_SU21	Streptomyces_sp._ZS0098	Streptomyces_thermocarboxydus_K155	Addition	Core-Accessory-Unique	COG	%Similarity_COG	Score_COG	Evalue_COG	KEGG	%Similarity_KEGG	Score_KEGG	Evalue_KEGG
5575	WP_086020148.1	-	SAM-dependent methyltransferase	1	1	1	1	1	1	1	1	1	1	1	11	CORE								
5576	WP_106432477.1	-	hypothetical protein	1	0	0	0	1	1	1	0	0	0	1	5	Accessory								
5577	WP_122213374.1	-	hypothetical protein	0	0	0	0	0	0	0	0	0	1	0	1	Unique								
5578	WP_048460558.1	-	RDD family protein	1	1	1	1	1	1	1	1	1	1	1	11	CORE	COG0532_J#	35.2	69	3E-11	NA	51.8	81	1.2E-14
5579	OSC72906.1	-	amidinotransferase	1	1	1	1	1	1	1	1	1	1	1	11	CORE	COG1834_E#	49.3	258	2.05E-68	NA	49.3	258	5.46E-68
5580	WP_037778495.1	-	hypothetical protein	0	0	0	0	1	1	0	0	0	0	0	2	Accessory								
5581	Prokka:JAPCLKMA_01503	-	hypothetical protein	1	0	0	1	0	0	0	0	0	0	0	2	Accessory								
5582	WP_143661392.1	-	universal stress protein	0	0	0	0	0	0	1	0	0	0	0	1	Unique								
5583	WP_051724699.1	-	SDR family oxidoreductase	0	0	0	0	0	0	1	0	0	0	0	1	Unique	COG1028_IQR#	39.9	148	2.98E-35	NA	36.1	143	4.37E-33
5584	AXI85260.1	-	serine protease	0	0	0	1	0	0	0	0	0	0	0	1	Unique								
5585	WP_051724634.1	-	4'-phosphopantetheinyl transferase superfamily protein	0	0	0	0	0	0	1	0	0	0	0	1	Unique					K06133	36.9	67	2.33E-10

cluster	GI	gene	Annotation	Streptomyces_thermocarboxydus_BPSAC147	Streptomyces_sp._4F	Streptomyces_sp._BSE7F	Streptomyces_sp._ETH9427	Streptomyces_sp._FxanaD5	Streptomyces_sp._UNC401CLCol	Actinospica_acidiphila_NRRL_B_24431	Streptomyces_sp._HNS054	Streptomyces_sp._SMS_SU21	Streptomyces_sp._ZS0098	Streptomyces_thermocarboxydus_K155	Addition	Core-Accessory-Unique	COG	%Similarity_COG	Score_COG	Evalue_COG	KEGG	%Similarity_KEGG	Score_KEGG	Evalue_KEGG
5586	OSC71067.1	-	SAM-dependent methyltransferase	0	1	0	0	0	0	0	0	0	0	0	1	Unique	COG0500_QR#	28.2	54	9.99E-07				
5587	WP_019521737.1	-	transcriptional regulator	1	1	0	1	1	1	1	1	1	1	1	10	Accessory	COG1396_K#	47.5	232	2.04E-60				
5588	WP_048457120.1	-	hypothetical protein	1	0	1	1	1	1	1	1	1	1	1	10	Accessory								
5589	WP_028959819.1	lipB	lipoyl(octanoyl) transferase LipB	1	1	1	1	1	1	1	1	1	1	1	11	CORE	COG0321_H#	63.1	171	4.28E-42	K03801	63.1	171	1.14E-41
5590	WP_033275044.1	-	RNA methyltransferase	1	1	1	1	1	1	1	1	1	1	1	11	CORE	COG0566_J#	44.9	215	3.37E-55	NA	44.9	210	1.7E-53
5591	WP_033276326.1	-	carbohydrate ABC transporter permease	1	1	1	1	1	1	1	1	1	1	1	11	CORE	COG0395_G#	32.1	158	4.9E-38	K10119	28.4	132	5.88E-30
5592	WP_028959266.1	-	hypothetical protein	0	0	0	0	0	1	0	0	0	0	0	1	Unique								
5593	OSC73098.1	-	transcription antitermination regulator	0	1	0	0	0	0	0	0	0	0	0	1	Unique								
5594	OSC71910.1	-	hypothetical protein	0	1	0	0	0	0	0	0	0	0	0	1	Unique								
5595	WP_086020143.1	-	IS5 family transposase	1	0	0	0	1	1	0	1	0	1	1	6	Accessory	COG3293_L#	33.7	146	1.47E-34	K07492	29.9	97	2.1E-19
5596	OSC70433.1	-	DeoR family transcriptional regulator	0	1	0	0	0	0	0	0	0	0	0	1	Unique	COG1349_KG#	32.4	131	4.91E-30	K03436	30.8	129	4.98E-29

cluster	GI	gene	Annotation	Streptomyces_thermocarboxydus_BPSAC147	Streptomyces_sp._4F	Streptomyces_sp._BSE7F	Streptomyces_sp._ETH9427	Streptomyces_sp._FxanaD5	Streptomyces_sp._UNC401CLCol	Actinospica_acidiphila_NRRL_B_24431	Streptomyces_sp._HNS054	Streptomyces_sp._SMS_SU21	Streptomyces_sp._ZS0098	Streptomyces_thermocarboxydus_K155	Addition	Core-Accessory-Unique	COG	%Similarity_COG	Score_COG	Evalue_COG	KEGG	%Similarity_KEGG	Score_KEGG	Evalue_KEGG
5597	WP_048460301.1	-	VWA domain-containing protein	0	0	0	0	0	0	0	1	0	0	0	1	Unique								
5598	Prokka:CIODPCDJ_00005	-	hypothetical protein	0	0	0	0	0	0	0	0	0	0	1	1	Unique								
5599	WP_026243895.1	-	class I SAM-dependent methyltransferase	1	1	1	1	1	1	1	1	1	1	1	11	CORE	COG0500_QR#	48.4	254	5.02E-67	NA	33.5	59	6.33E-08
5600	WP_033272357.1	-	hypothetical protein	1	0	0	0	1	1	1	0	0	0	1	5	Accessory								
5601	OSC62324.1	-	phenylacetic acid degradation protein	0	1	0	0	0	0	0	0	0	0	0	1	Unique	COG1018_C#	41.2	202	2.26E-51	NA	39.6	196	4.33E-49
5602	Prokka:CIODPCDJ_05461	-	hypothetical protein	1	1	0	0	1	1	1	0	0	0	1	6	Accessory	COG0600_P#	32	131	6.41E-30	K15552	30.4	120	3.02E-26
5603	OSC67777.1	-	CDP-diacylglycerol--serine O-phosphatidyltransferase	1	1	1	1	1	1	1	1	1	1	1	11	CORE	COG1183_I#	29.4	91	5.63E-18	K17103	33.5	71	1.61E-11
5604	PWE11555.1	paaI	phenylacetate-CoA oxygenase subunit PaaI	0	1	1	1	0	0	1	1	1	1	0	7	Accessory	COG3396_S#	44.3	208	2.42E-53	K02611	43.3	203	2.71E-51
5605	OSC60785.1	-	hypothetical protein	0	1	0	0	0	0	0	0	0	0	0	1	Unique								
5606	OSC71654.1	-	hypothetical protein	0	1	1	1	1	1	1	1	1	1	1	10	Accessory								

cluster	GI	gene	Annotation	Streptomyces_thermocarboxydus_BPSAC147	Streptomyces_sp._4F	Streptomyces_sp._BSE7F	Streptomyces_sp._ETH9427	Streptomyces_sp._FxanaD5	Streptomyces_sp._UNC401CLCol	Actinospica_acidiphila_NRRL_B_24431	Streptomyces_sp._HNS054	Streptomyces_sp._SMS_SU21	Streptomyces_sp._ZS0098	Streptomyces_thermocarboxydus_K155	Addition	Core-Accessory-Unique	COG	%Similarity_COG	Score_COG	Evalue_COG	KEGG	%Similarity_KEGG	Score_KEGG	Evalue_KEGG
5607	WP_048458802.1	-	TlpA family protein disulfide reductase	0	0	0	0	0	0	0	1	0	0	0	1	Unique					K17904	30.2	45	0.000946
5608	WP_122213916.1	-	hypothetical protein	0	0	1	1	0	0	1	1	1	1	0	6	Accessory								
5609	AXI86235.1	-	tryptophan synthase subunit alpha	1	1	1	1	1	1	1	1	1	1	1	11	CORE	COG0159_E#	50.6	250	9.43E-66	K01695	50.6	250	2.52E-65
5610	AXI85997.1	-	TlyA family RNA methyltransferase	1	1	1	1	1	1	1	1	1	1	1	11	CORE	COG1189_J#	63.9	309	1E-83	K06442	61.3	292	3.39E-78
5611	WP_028961408.1	-	ABC transporter permease	0	0	0	0	1	1	0	0	0	0	1	3	Accessory	COG1177_E#	49.6	253	6.53E-67	K02053	48.5	234	1.1E-60
5612	WP_019527408.1	-	helix-turn-helix domain-containing protein	1	0	0	0	1	1	1	0	0	0	1	5	Accessory	COG1396_K#	40.4	141	3.62E-33				
5613	PWE09411.1	-	F0F1 ATP synthase subunit delta	1	1	1	1	1	1	1	1	1	1	1	11	CORE	COG0712_C#	33.6	149	1.33E-35	K02113	35.9	162	5.29E-39
5614	WP_019525301.1	-	SAM-dependent methyltransferase	1	1	1	1	1	1	1	1	1	1	1	11	CORE								
5615	Prokka:JAPCLKMA_05836	yedK_2	SOS response-associated protein YedK	1	0	1	1	1	1	1	1	1	1	1	10	Accessory	COG2135_S#	37.8	184	3.73E-46	NA	37.3	171	1.49E-41

cluster	GI	gene	Annotation	Streptomyces_thermocarboxydus_BPSAC147	Streptomyces_sp._4F	Streptomyces_sp._BSE7F	Streptomyces_sp._ETH9427	Streptomyces_sp._FxanaD5	Streptomyces_sp._UNC401CLCol	Actinospica_acidiphila_NRRL_B_24431	Streptomyces_sp._HNS054	Streptomyces_sp._SMS_SU21	Streptomyces_sp._ZS0098	Streptomyces_thermocarboxydus_K155	Addition	Core-Accessory-Unique	COG	%Similarity_COG	Score_COG	Evalue_COG	KEGG	%Similarity_KEGG	Score_KEGG	Evalue_KEGG
5616	WP_122213248.1	-	formate/nitrite transporter family protein	1	0	1	1	1	1	1	1	1	1	1	10	Accessory								
5617	OSC61574.1	-	hypothetical protein	0	1	0	0	0	0	0	0	0	0	0	1	Unique								
5618	OSC73465.1	-	hypothetical protein	1	1	1	1	1	1	1	1	1	1	1	11	CORE	COG0842_V#	23.6	60	8.11E-09				
5619	WP_064743260.1	-	fumarylacetoacetate hydrolase	0	0	0	0	1	1	1	1	0	1	1	6	Accessory	COG3971_Q#	36.9	155	2.42E-37	K02554	36.9	155	6.46E-37
5620	WP_033273863.1	-	inositol monophosphatase family protein	0	0	0	0	0	0	1	0	0	0	0	1	Unique	COG0483_G#	30.9	98	3.51E-20	K01092	30.6	79	5.89E-14
5621	Prokka:CIODPCDJ_06644	-	hypothetical protein	0	0	0	0	0	0	0	0	0	0	1	1	Unique								
5622	Prokka:CIODPCDJ_05887	cobK	Precorrin-6A reductase	1	0	1	1	1	1	1	1	1	1	1	10	Accessory	COG2099_H#	53.8	237	4.84E-62	K05895	52.6	213	3.41E-54
5623	Prokka:CIODPCDJ_06540	glpR_3	Glycerol-3-phosphate regulon repressor	1	1	1	1	1	1	1	1	1	1	1	11	CORE	COG1349_KG#	35.2	147	6.57E-35	K02081	34.2	143	2.53E-33
5624	PWE10605.1	-	hypothetical protein	1	1	1	1	1	1	1	1	1	1	1	11	CORE								
5625	Prokka:CIODPCDJ_01694	-	hypothetical protein	1	1	1	1	1	1	1	1	1	1	1	11	CORE	COG1651_O#	28.1	63	1.25E-09	K08884	27.2	72	9.37E-12
5626	AXI89510.1	-	hypothetical protein	0	0	0	1	0	0	0	1	1	1	0	4	Accessory	COG3752_S#	49.4	240	7.45E-63	NA	49.4	240	1.99E-62
5627	WP_033277994.1	proC	pyrroline-5-carboxylate reductase	0	1	0	1	1	1	1	1	1	1	1	9	Accessory	COG0345_E#	47	228	3.82E-59	K00286	42.6	205	7.09E-52

cluster	GI	gene	Annotation	Streptomyces_thermocarboxydus_BPSAC147	Streptomyces_sp._4F	Streptomyces_sp._BSE7F	Streptomyces_sp._ETH9427	Streptomyces_sp._FxanaD5	Streptomyces_sp._UNC401CLCol	Actinospica_acidiphila_NRRL_B_24431	Streptomyces_sp._HNS054	Streptomyces_sp._SMS_SU21	Streptomyces_sp._ZS0098	Streptomyces_thermocarboxydus_K155	Addition	Core-Accessory-Unique	COG	%Similarity_COG	Score_COG	Evalue_COG	KEGG	%Similarity_KEGG	Score_KEGG	Evalue_KEGG
5628	WP_102639157.1	-	c-type cytochrome	1	1	1	1	1	1	1	1	1	1	1	11	CORE	COG2010_C#	48.8	246	1.04E-64	K03889	48.7	243	1.8E-63
5629	Prokka:JAPCLKMA_04534	thiD	Hydroxymethylpyrimidine/phosphomethylpyrimidine kinase	1	1	0	1	1	1	1	1	1	1	1	10	Accessory	COG0351_H#	50.2	250	5.51E-66	K00941	52.3	248	9.54E-65
5630	AXI88354.1	-	hypothetical protein	1	1	0	1	1	1	1	1	1	1	1	10	Accessory					NA	34.7	56	4.07E-07
5631	OSC63480.1	-	hypothetical protein	0	1	1	1	0	0	1	1	1	1	0	7	Accessory								
5632	OSC62398.1	-	MFS transporter	0	1	0	0	0	0	0	0	0	0	0	1	Unique	COG0477_GEPR#	47.5	257	4.51E-68	NA	38.7	213	3.4E-54
5633	PWE06609.1	-	5-formyltetrahydrofolate cyclo-ligase	0	0	1	1	0	0	0	0	0	0	0	2	Accessory	COG0212_H#	46.4	199	1.46E-50	K01934	46.4	199	3.89E-50
5634	WP_048458663.1	-	hypothetical protein	0	0	0	0	0	0	0	1	0	0	0	1	Unique								
5635	WP_033274367.1	-	polysaccharide deacetylase family protein	1	1	1	1	1	1	1	1	1	1	1	11	CORE	COG0726_G#	39.2	137	8.88E-32	NA	38.6	138	8.15E-32
5636	Prokka:JAPCLKMA_02252	tgnD	(E)-2-((N-methylformamido)methylene)succinate hydrolase	1	0	0	1	1	1	1	1	1	1	1	9	Accessory	COG0596_R#	33.5	127	5.39E-29	K18399	27.3	77	2.91E-13
5637	Prokka:CIODPCDJ_02128	-	hypothetical protein	0	0	0	0	0	0	0	0	0	0	1	1	Unique								

cluster	GI	gene	Annotation	Streptomyces_thermocarboxydus_BPSAC147	Streptomyces_sp._4F	Streptomyces_sp._BSE7F	Streptomyces_sp._ETH9427	Streptomyces_sp._FxanaD5	Streptomyces_sp._UNC401CLCol	Actinospica_acidiphila_NRRL_B_24431	Streptomyces_sp._HNS054	Streptomyces_sp._SMS_SU21	Streptomyces_sp._ZS0098	Streptomyces_thermocarboxydus_K155	Addition	Core-Accessory-Unique	COG	%Similarity_COG	Score_COG	Evalue_COG	KEGG	%Similarity_KEGG	Score_KEGG	Evalue_KEGG
5638	Prokka:JAPCLKMA_05150	-	hypothetical protein	1	0	0	0	0	0	0	0	0	0	0	1	Unique	COG0732_V#	32.4	63	1.63E-09				
5639	Prokka:CIODPCDJ_05949	fbiB_2	Bifunctional F420 biosynthesis protein FbiB	1	0	1	1	1	1	1	1	1	1	1	10	Accessory	COG0778_C#	40.3	49	0.0000186	NA	21	44	0.00273
5640	WP_033273472.1	-	class I SAM-dependent methyltransferase	0	0	0	0	0	0	1	0	0	0	0	1	Unique	COG0500_QR#	28.5	84	8.92E-16	NA	28	81	1.54E-14
5641	OSC63690.1	-	HAD family hydrolase	1	1	1	1	1	1	1	1	1	1	1	11	CORE								
5642	WP_109288677.1	-	hypothetical protein	1	1	1	1	0	0	1	1	1	1	1	9	Accessory								
5643	PWE08116.1	-	hypothetical protein	0	0	1	0	0	0	0	0	0	0	0	1	Unique								
5644	WP_019522193.1	-	NAD(P)-dependent oxidoreductase	1	1	0	1	1	1	1	1	1	1	1	10	Accessory	COG0451_MG#	51.4	241	3.33E-63	K18981	45	209	4.89E-53
5645	OSC73340.1	-	signal peptidase I	1	1	0	1	1	1	1	1	1	1	1	10	Accessory	COG0681_U#	30.7	103	1.42E-21	K03100	36.9	81	1.18E-14
5646	AXI91222.1	-	hypothetical protein	0	0	0	1	0	0	0	0	0	0	0	1	Unique								
5647	OSC65918.1	-	hypothetical protein	0	1	0	0	0	0	0	0	0	0	0	1	Unique								
5648	WP_028960986.1	-	M15 family metallopeptidase	1	1	1	1	1	1	1	1	1	1	1	11	CORE	COG2173_M#	49.6	255	2.91E-67				
5649	Prokka:JAPCLKMA_05082	-	hypothetical protein	1	0	0	0	1	0	1	0	0	0	0	3	Accessory								

cluster	GI	gene	Annotation	Streptomyces_thermocarboxydus_BPSAC147	Streptomyces_sp._4F	Streptomyces_sp._BSE7F	Streptomyces_sp._ETH9427	Streptomyces_sp._FxanaD5	Streptomyces_sp._UNC401CLCol	Actinospica_acidiphila_NRRL_B_24431	Streptomyces_sp._HNS054	Streptomyces_sp._SMS_SU21	Streptomyces_sp._ZS0098	Streptomyces_thermocarboxydus_K155	Addition	Core-Accessory-Unique	COG	%Similarity_COG	Score_COG	Evalue_COG	KEGG	%Similarity_KEGG	Score_KEGG	Evalue_KEGG
5650	WP_033278081.1	-	PhzF family phenazine biosynthesis protein	1	1	1	1	1	1	1	1	1	1	1	11	CORE	COG0384_R#	41.7	205	2.02E-52	NA	42.3	180	2.43E-44
5651	Prokka:CIODPCDJ_03189	tcrX_2	putative transcriptional regulatory protein TcrX	1	1	1	1	1	1	1	1	1	1	1	11	CORE	COG0745_TK#	72	333	6.44E-91	K02483	73.2	332	2.93E-90
5652	WP_102641137.1	-	SDR family oxidoreductase	1	1	1	1	1	1	1	1	1	1	1	11	CORE	COG1028_IQR#	55.2	270	6.69E-72	K00540	54.8	267	1.51E-70
5653	Prokka:JAPCLKMA_00749	suhB	Inositol-1-monophosphatase SuhB	1	1	0	1	1	1	1	1	1	1	1	10	Accessory	COG0483_G#	44.3	205	3.45E-52	K01092	44.3	205	9.22E-52
5654	Prokka:CIODPCDJ_06750	-	hypothetical protein	0	0	0	0	0	0	0	0	0	0	1	1	Unique								
5655	WP_026243816.1	-	AfsR/SARP family transcriptional regulator	0	0	0	0	1	1	0	0	0	0	1	3	Accessory	COG3629_T#	32	127	9.15E-29				
5656	Prokka:JAPCLKMA_04371	-	hypothetical protein	1	0	1	1	1	1	0	1	1	1	1	9	Accessory					NA	36.5	52	0.00000765
5657	WP_122213293.1	-	FadR family transcriptional regulator	1	0	0	1	1	1	1	1	0	1	1	8	Accessory	COG2186_K#	41.3	178	2.65E-44	NA	34.3	122	7.86E-27
5658	OSC70662.1	-	hypothetical protein	0	1	0	0	0	0	0	0	0	0	0	1	Unique								
5659	WP_122215762.1	-	EcsC family protein	1	0	0	0	0	0	0	0	0	1	1	3	Accessory								

cluster	GI	gene	Annotation	Streptomyces_thermocarboxydus_BPSAC147	Streptomyces_sp._4F	Streptomyces_sp._BSE7F	Streptomyces_sp._ETH9427	Streptomyces_sp._FxanaD5	Streptomyces_sp._UNC401CLCol	Actinospica_acidiphila_NRRL_B_24431	Streptomyces_sp._HNS054	Streptomyces_sp._SMS_SU21	Streptomyces_sp._ZS0098	Streptomyces_thermocarboxydus_K155	Addition	Core-Accessory-Unique	COG	%Similarity_COG	Score_COG	Evalue_COG	KEGG	%Similarity_KEGG	Score_KEGG	Evalue_KEGG
5660	WP_033276762.1	-	DUF72 domain-containing protein	1	1	1	1	1	1	1	1	1	1	1	11	CORE	COG1801_S#	32.8	136	1.97E-31	NA	32.8	136	5.26E-31
5661	WP_033275718.1	pgl	6-phosphogluconolactonase	1	1	1	1	1	1	1	1	1	1	1	11	CORE	COG0363_G#	49.6	244	5.14E-64	K01057	49.6	244	1.37E-63
5662	OSC66075.1	-	indole-3-glycerol phosphate synthase	1	1	1	1	1	1	1	1	1	1	1	11	CORE	COG0134_E#	64.5	328	2.07E-89	K01609	64.5	328	5.53E-89
5663	WP_078493084.1	-	hypothetical protein	0	0	0	0	1	1	1	0	0	0	1	4	Accessory	COG0533_O#	44.9	68	5.05E-11	K01409	44.9	68	1.35E-10
5664	Prokka:JAPCLKMA_06593	cobS	Adenosylcobinamide-GDP ribazoletransferase	1	1	0	1	1	1	1	1	1	1	1	10	Accessory	COG0368_H#	39.2	146	1.9E-34	K02233	39.2	146	5.08E-34
5665	OSC69115.1	-	ATP-binding protein	0	1	0	0	0	0	0	0	0	0	0	1	Unique	COG1116_P#	53.5	264	3.67E-70	K02049	53.5	264	9.81E-70
5666	Prokka:CIODPCDJ_04873	-	hypothetical protein	1	0	0	1	1	0	1	1	1	1	1	8	Accessory					NA	31.1	56	4.06E-07
5667	OSC71668.1	-	hypothetical protein	0	1	0	0	0	0	0	0	0	0	0	1	Unique								
5668	AXI89616.1	-	3-deoxy-manno-octulosonate cytidylyltransferase	0	0	0	1	0	0	0	0	0	0	0	1	Unique	COG1212_M#	38.2	154	5.35E-37	K00979	38.2	154	1.43E-36
5669	Prokka:JAPCLKMA_02065	-	hypothetical protein	1	1	0	0	1	1	1	0	0	0	1	6	Accessory	COG0600_P#	34.8	165	2.32E-40	K15552	31.5	144	1.48E-33
5670	Prokka:CIODPCDJ_06113	-	hypothetical protein	1	1	1	1	1	1	1	1	1	1	1	11	CORE								

cluster	GI	gene	Annotation	Streptomyces_thermocarboxydus_BPSAC147	Streptomyces_sp._4F	Streptomyces_sp._BSE7F	Streptomyces_sp._ETH9427	Streptomyces_sp._FxanaD5	Streptomyces_sp._UNC401CLCol	Actinospica_acidiphila_NRRL_B_24431	Streptomyces_sp._HNS054	Streptomyces_sp._SMS_SU21	Streptomyces_sp._ZS0098	Streptomyces_thermocarboxydus_K155	Addition	Core-Accessory-Unique	COG	%Similarity_COG	Score_COG	Evalue_COG	KEGG	%Similarity_KEGG	Score_KEGG	Evalue_KEGG
5671	OSC65368.1	-	xanthine dehydrogenase accessory protein XdhC	0	1	0	0	0	0	0	0	0	0	0	1	Unique	COG1975_O#	54.3	270	6.69E-72	K07402	54.3	270	1.79E-71
5672	WP_122215394.1	-	aminoglycoside phosphotransferase	0	0	0	0	0	0	0	0	0	1	0	1	Unique								
5673	Prokka:CIODPCDJ_00593	bdhA_1	D-beta-hydroxybutyrate dehydrogenase	1	1	1	1	1	1	1	1	1	1	1	11	CORE	COG1028_IQR#	48.2	219	1.77E-56	K00019	42.9	186	4.43E-46
5674	WP_122217428.1	-	IS5 family transposase	1	1	1	0	1	0	0	1	1	1	1	8	Accessory	COG3293_L#	33.7	141	6.12E-33				
5675	Prokka:JAPCLKMA_02934	rsbQ	Sigma factor SigB regulation protein RsbQ	1	0	1	1	1	1	1	1	1	1	1	10	Accessory	COG0596_R#	49.4	273	1.03E-72	NA	49.4	273	2.75E-72
5676	AXI85494.1	-	MBL fold metallo-hydrolase	0	1	1	1	0	1	1	1	1	1	0	8	Accessory								
5677	AXI90360.1	-	hypothetical protein	1	0	0	1	1	1	1	1	1	1	1	9	Accessory	COG2343_S#	52.4	248	2.71E-65	NA	52.4	248	7.25E-65
5678	WP_102638289.1	-	HlyD family efflux transporter periplasmic adaptor subunit	1	1	1	1	1	1	1	1	1	1	1	11	CORE	COG0845_M#	26	78	3.72E-14				
5679	OSC58178.1	-	lipase	1	1	1	1	1	1	1	1	1	1	1	11	CORE								

cluster	GI	gene	Annotation	Streptomyces_thermocarboxydus_BPSAC147	Streptomyces_sp._4F	Streptomyces_sp._BSE7F	Streptomyces_sp._ETH9427	Streptomyces_sp._FxanaD5	Streptomyces_sp._UNC401CLCol	Actinospica_acidiphila_NRRL_B_24431	Streptomyces_sp._HNS054	Streptomyces_sp._SMS_SU21	Streptomyces_sp._ZS0098	Streptomyces_thermocarboxydus_K155	Addition	Core-Accessory-Unique	COG	%Similarity_COG	Score_COG	Evalue_COG	KEGG	%Similarity_KEGG	Score_KEGG	Evalue_KEGG
5680	AXI86504.1	-	hypothetical protein	1	1	1	1	1	1	1	1	1	1	1	11	CORE								
5681	WP_048457592.1	-	ABC transporter permease	0	0	1	1	1	1	1	1	1	1	1	9	Accessory	COG0767_Q#	49.4	279	1.1E-74	K02066	49.4	279	2.94E-74
5682	WP_078895249.1	-	hypothetical protein	0	0	0	1	0	0	0	1	1	1	0	4	Accessory								
5683	AXI89489.1	-	IclR family transcriptional regulator	1	0	1	1	1	1	1	1	1	1	1	10	Accessory	COG1414_K#	37.4	161	5.7E-39	K13641	37.4	161	1.52E-38
5684	Prokka:CIODPCDJ_00220	-	Tropinesterase	0	0	0	0	0	1	0	0	0	0	1	2	Accessory	COG0596_R#	32.8	109	2.57E-23	NA	29.7	67	2.99E-10
5685	AXI85354.1	-	hypothetical protein	1	0	0	1	0	0	0	0	0	0	0	2	Accessory								
5686	Prokka:JAPCLKMA_02715	hcaB_6	3-phenylpropionate-dihydrodiol/cinnamic acid-dihydrodiol dehydrogenase	1	1	1	1	1	1	1	1	1	1	1	11	CORE	COG1028_IQR#	59.1	282	2.21E-75	NA	33.6	118	8.66E-26
5687	WP_033275826.1	-	DUF3097 domain-containing protein	1	1	0	1	1	1	1	1	1	1	1	10	Accessory					NA	62.9	315	3.7E-85
5688	OSC61124.1	-	hypothetical protein	1	1	1	1	1	1	1	1	1	1	1	11	CORE	COG0730_R#	28.6	107	7.48E-23				
5689	Prokka:JAPCLKMA_05154	-	hypothetical protein	1	0	0	0	0	0	0	0	0	1	1	3	Accessory								

cluster	GI	gene	Annotation	Streptomyces_thermocarboxydus_BPSAC147	Streptomyces_sp._4F	Streptomyces_sp._BSE7F	Streptomyces_sp._ETH9427	Streptomyces_sp._FxanaD5	Streptomyces_sp._UNC401CLCol	Actinospica_acidiphila_NRRL_B_24431	Streptomyces_sp._HNS054	Streptomyces_sp._SMS_SU21	Streptomyces_sp._ZS0098	Streptomyces_thermocarboxydus_K155	Addition	Core-Accessory-Unique	COG	%Similarity_COG	Score_COG	Evalue_COG	KEGG	%Similarity_KEGG	Score_KEGG	Evalue_KEGG
5690	AXI85466.1	-	LysR family transcriptional regulator	1	1	1	1	1	1	1	1	1	1	1	11	CORE	COG0583_K#	31.5	105	2.84E-22	NA	28.2	69	6.03E-11
5691	WP_122215711.1	-	MBL fold metallo-hydrolase	0	0	0	0	0	0	0	0	0	1	0	1	Unique								
5692	OSC55884.1	-	TetR family transcriptional regulator	1	1	1	1	1	1	1	1	1	1	1	11	CORE	COG1309_K#	31.7	79	2.18E-14	NA	38.7	71	1.59E-11
5693	PWE11508.1	-	urease accessory protein	0	0	1	0	0	0	0	0	0	0	0	1	Unique	COG0829_O#	30.1	72	2.04E-12				
5694	WP_084207091.1	-	HAD family hydrolase	1	1	0	1	1	1	1	1	1	1	1	10	Accessory	COG0546_R#	23.8	52	0.00000285				
5695	AXI91279.1	-	hypothetical protein	0	0	0	1	0	0	0	0	0	0	0	1	Unique								
5696	PWE11505.1	-	hypothetical protein	0	0	1	0	0	0	0	0	0	0	0	1	Unique								
5697	Prokka:CIODPCDJ_04728	COQ5_8	2-methoxy-6-polyprenyl-1,4-benzoquinol methylase, mitochondrial	1	0	1	1	1	1	1	1	1	1	1	10	Accessory	COG2230_M#	42.5	173	8.46E-43	K05928	32.4	65	8.67E-10
5698	WP_019522664.1	-	ABC transporter permease subunit	0	0	0	0	1	1	1	0	0	0	1	4	Accessory								

cluster	GI	gene	Annotation	Streptomyces_thermocarboxydus_BPSAC147	Streptomyces_sp._4F	Streptomyces_sp._BSE7F	Streptomyces_sp._ETH9427	Streptomyces_sp._FxanaD5	Streptomyces_sp._UNC401CLCol	Actinospica_acidiphila_NRRL_B_24431	Streptomyces_sp._HNS054	Streptomyces_sp._SMS_SU21	Streptomyces_sp._ZS0098	Streptomyces_thermocarboxydus_K155	Addition	Core-Accessory-Unique	COG	%Similarity_COG	Score_COG	Evalue_COG	KEGG	%Similarity_KEGG	Score_KEGG	Evalue_KEGG
5699	WP_102637563.1	-	TrmH family RNA methyltransferase	1	1	0	1	1	1	1	1	1	1	1	10	Accessory	COG0566_J#	34.3	65	3.25E-10				
5700	WP_028959421.1	-	response regulator transcription factor	1	1	1	1	1	1	1	1	1	1	1	11	CORE	COG2197_TK#	38.7	136	1.96E-31	K07692	31.6	118	1.13E-25
5701	WP_033272134.1	-	enoyl-CoA hydratase	1	1	1	1	1	1	1	1	1	1	1	11	CORE	COG1024_I#	41	189	1.95E-47	K15866	38.6	183	3.73E-45
5702	OSC61733.1	-	alpha/beta hydrolase	0	1	0	0	0	0	0	0	0	0	0	1	Unique	COG0596_R#	31.6	112	1.77E-24	NA	30.4	70	3.52E-11
5703	PWE12042.1	-	VOC family protein	1	0	1	1	1	1	1	1	1	1	1	10	Accessory	COG3324_R#	26.1	91	7.21E-18	K06996	26.1	91	1.93E-17
5704	AXI87562.1	-	hypothetical protein	1	1	1	1	1	1	1	1	1	1	1	11	CORE								
5705	Prokka:CIODPCDJ_02099	btuD_7	Vitamin B12 import ATP-binding protein BtuD	1	1	0	1	1	1	1	1	1	1	1	10	Accessory	COG1121_P#	39.4	155	2.38E-37	K11710	38.9	146	2.96E-34
5706	OSC62601.1	-	pectate lyase	1	1	1	1	1	1	1	1	1	1	1	11	CORE					NA	37.2	121	1.33E-26
5707	OSC62037.1	-	ArsR family transcriptional regulator	0	1	0	0	0	0	0	0	0	0	0	1	Unique	COG0640_K#	65	135	2.55E-31	NA	37.9	62	5.62E-09
5708	WP_102640171.1	-	hypothetical protein	1	1	1	1	1	1	1	1	1	1	1	11	CORE								

cluster	GI	gene	Annotation	Streptomyces_thermocarboxydus_BPSAC147	Streptomyces_sp._4F	Streptomyces_sp._BSE7F	Streptomyces_sp._ETH9427	Streptomyces_sp._FxanaD5	Streptomyces_sp._UNC401CLCol	Actinospica_acidiphila_NRRL_B_24431	Streptomyces_sp._HNS054	Streptomyces_sp._SMS_SU21	Streptomyces_sp._ZS0098	Streptomyces_thermocarboxydus_K155	Addition	Core-Accessory-Unique	COG	%Similarity_COG	Score_COG	Evalue_COG	KEGG	%Similarity_KEGG	Score_KEGG	Evalue_KEGG
5709	WP_051724682.1	-	PaaX domain-containing protein, C-domain protein	1	1	1	1	1	1	1	1	1	1	1	11	CORE	COG3327_K#	52.7	213	9.64E-55	K02616	52.7	213	2.57E-54
5710	Prokka:JAPCLKMA_01505	-	hypothetical protein	1	0	0	1	0	0	0	0	0	0	0	2	Accessory								
5711	WP_048456748.1	-	endo alpha-1,4 polygalactosaminidase	1	1	1	1	1	1	1	1	1	1	1	11	CORE	COG3868_S#	39.2	158	2.82E-38	NA	39.2	158	7.52E-38
5712	WP_122213869.1	urtD	urea ABC transporter ATP-binding protein UrtD	1	0	1	1	1	1	1	1	1	1	1	10	Accessory	COG4674_R#	56.8	275	2.7E-73	K11962	46	218	8E-56
5713	Prokka:CIODPCDJ_03761	-	hypothetical protein	1	0	1	1	1	1	1	1	1	1	1	10	Accessory	COG2730_G#	39.6	67	6.54E-11	K07605	33.3	70	3.52E-11
5714	WP_048460302.1	-	hypothetical protein	0	0	0	0	0	0	0	1	0	0	0	1	Unique								
5715	OSC61678.1	-	3-ketoacyl-ACP reductase	0	1	1	1	0	0	1	1	1	1	0	7	Accessory	COG1028_IQR#	57.6	273	6.01E-73	K00059	44.7	205	9.15E-52
5716	WP_033272619.1	-	helix-turn-helix domain-containing protein	1	0	0	0	0	0	1	1	0	0	0	3	Accessory	COG3409_M#	35	56	1.51E-07	K01449	37.3	54	0.00000153
5717	AXI86453.1	-	acetylglutamate kinase	0	0	0	1	0	0	0	1	0	0	0	2	Accessory	COG0548_E#	35.8	101	5.34E-21	K05828	31	90	2.52E-17
5718	Prokka:CIODPCDJ_01470	-	hypothetical protein	0	0	0	0	0	0	0	0	0	0	1	1	Unique								

cluster	GI	gene	Annotation	Streptomyces_thermocarboxydus_BPSAC147	Streptomyces_sp._4F	Streptomyces_sp._BSE7F	Streptomyces_sp._ETH9427	Streptomyces_sp._FxanaD5	Streptomyces_sp._UNC401CLCol	Actinospica_acidiphila_NRRL_B_24431	Streptomyces_sp._HNS054	Streptomyces_sp._SMS_SU21	Streptomyces_sp._ZS0098	Streptomyces_thermocarboxydus_K155	Addition	Core-Accessory-Unique	COG	%Similarity_COG	Score_COG	Evalue_COG	KEGG	%Similarity_KEGG	Score_KEGG	Evalue_KEGG
5719	OSC72715.1	-	esterase	1	1	1	1	1	1	1	1	1	1	1	11	CORE	COG1647_R#	33.2	99	2.03E-20				
5720	OSC54619.1	-	alpha/beta hydrolase	0	1	0	0	0	0	0	0	0	0	0	1	Unique	COG0596_R#	33.5	136	1.15E-31	NA	33.5	136	3.06E-31
5721	OSC55635.1	-	uridine kinase	1	1	1	1	1	1	1	1	1	1	1	11	CORE								
5722	Prokka:JAPCLKMA_03352	-	hypothetical protein	1	1	1	1	1	1	1	1	1	1	1	11	CORE	COG0671_I#	41.8	94	5E-19	NA	29.7	78	1.29E-13
5723	WP_026243927.1	-	AraC family transcriptional regulator	0	1	0	0	1	1	0	0	0	0	1	4	Accessory	COG2207_K#	43.3	211	4.78E-54				
5724	OSC64376.1	-	ABC transporter	0	1	0	0	0	0	0	0	0	0	0	1	Unique	COG0842_V#	31.1	121	3.82E-27	K01992	24.4	62	9.58E-09
5725	Prokka:JAPCLKMA_05491	-	hypothetical protein	1	0	0	0	0	0	0	0	0	0	0	1	Unique								
5726	WP_102638328.1	-	Ser or Arg-related nuclear matrix protein	1	1	0	1	1	1	1	1	1	1	1	10	Accessory								
5727	WP_048457792.1	-	sugar phosphate isomerase/epimerase	1	1	1	1	1	1	1	1	1	1	1	11	CORE								
5728	WP_048460332.1	-	alpha/beta fold hydrolase	0	0	1	1	0	0	0	1	1	1	0	5	Accessory	COG0596_R#	36.9	68	4.99E-11	K19311	25.8	45	0.00121
5729	OSC61732.1	-	aspartate dehydrogenase	0	1	0	0	0	0	0	0	0	0	0	1	Unique	COG1712_R#	37.4	124	4.49E-28				
5730	WP_033273846.1	-	hypothetical protein	1	1	1	1	1	1	1	1	1	1	1	11	CORE								

cluster	GI	gene	Annotation	Streptomyces_thermocarboxydus_BPSAC147	Streptomyces_sp._4F	Streptomyces_sp._BSE7F	Streptomyces_sp._ETH9427	Streptomyces_sp._FxanaD5	Streptomyces_sp._UNC401CLCol	Actinospica_acidiphila_NRRL_B_24431	Streptomyces_sp._HNS054	Streptomyces_sp._SMS_SU21	Streptomyces_sp._ZS0098	Streptomyces_thermocarboxydus_K155	Addition	Core-Accessory-Unique	COG	%Similarity_COG	Score_COG	Evalue_COG	KEGG	%Similarity_KEGG	Score_KEGG	Evalue_KEGG
5731	Prokka:JAPCLKMA_04875	crt	Short-chain-enoyl-CoA hydratase	1	1	1	1	1	1	1	1	1	1	1	11	CORE	COG1024_I#	42.5	197	5.45E-50	NA	42.5	197	1.46E-49
5732	Prokka:CIODPCDJ_02465	-	hypothetical protein	1	1	1	1	1	1	1	1	1	1	1	11	CORE	COG4587_R#	35.3	131	3.67E-30	K01992	35.3	131	9.81E-30
5733	Prokka:CIODPCDJ_02413	xynR_2	HTH-type transcriptional regulator XynR	1	1	1	1	1	1	1	1	1	1	1	11	CORE					NA	26.4	62	5.6E-09
5734	WP_122216279.1	-	ABC transporter ATP-binding protein	1	1	1	1	1	1	1	1	1	1	1	11	CORE	COG1120_PH#	50.2	236	1.06E-61	K02013	39.7	185	5.73E-46
5735	OSC57556.1	-	hypothetical protein	0	1	0	0	0	0	0	0	0	0	0	1	Unique								
5736	Prokka:CIODPCDJ_05131	rfbF	Glucose-1-phosphate cytidylyltransferase	1	0	1	1	1	1	1	1	1	1	1	10	Accessory	COG1208_MJ#	44.3	225	1.87E-58	K00978	34.7	169	5.55E-41
5737	OSC65722.1	-	hypothetical protein	0	1	0	0	0	0	0	0	0	0	0	1	Unique								
5738	WP_048460160.1	-	inositol phosphorylceramide synthase	1	1	1	0	1	1	1	1	1	1	1	10	Accessory								
5739	PWE09624.1	-	ABC transporter permease	1	1	1	1	1	1	1	1	1	1	1	11	CORE	COG1177_E#	38.5	188	3.31E-47	K11070	38.8	173	2.94E-42
5740	OSC62271.1	-	thioesterase	0	1	0	0	0	0	0	0	0	0	0	1	Unique								
5741	Prokka:JAPCLKMA_02055	-	hypothetical protein	1	0	0	0	0	1	1	1	1	1	1	7	Accessory	COG0454_KR#	27.9	45	0.000346				

cluster	GI	gene	Annotation	Streptomyces_thermocarboxydus_BPSAC147	Streptomyces_sp._4F	Streptomyces_sp._BSE7F	Streptomyces_sp._ETH9427	Streptomyces_sp._FxanaD5	Streptomyces_sp._UNC401CLCol	Actinospica_acidiphila_NRRL_B_24431	Streptomyces_sp._HNS054	Streptomyces_sp._SMS_SU21	Streptomyces_sp._ZS0098	Streptomyces_thermocarboxydus_K155	Addition	Core-Accessory-Unique	COG	%Similarity_COG	Score_COG	Evalue_COG	KEGG	%Similarity_KEGG	Score_KEGG	Evalue_KEGG
5742	OSC72476.1	-	hypothetical protein	1	1	1	1	1	1	1	1	1	1	1	11	CORE								
5743	AXI85320.1	-	hypothetical protein	1	0	0	1	0	0	0	0	0	0	0	2	Accessory								
5744	OSC57257.1	-	methyltransferase	0	1	0	0	0	0	0	0	0	0	0	1	Unique	COG0500_QR#	27.2	50	0.00000824	NA	35	64	2.51E-09
5745	WP_086020133.1	-	serine/threonine protein kinase	0	0	0	0	1	1	0	0	0	0	1	3	Accessory	COG0515_RTKL#	50.5	92	3.23E-18	K08884	50.5	92	8.62E-18
5746	OSC73384.1	-	rhamnogalacturonan acetylesterase	1	1	0	0	1	1	1	0	0	0	1	6	Accessory	COG2755_E#	45.1	167	6.04E-41	NA	43.5	164	1.37E-39
5747	AXI84795.1	-	methyltransferase	0	1	1	1	1	1	1	1	1	1	1	10	Accessory	COG0500_QR#	37.4	154	5.29E-37				
5748	WP_102641321.1	-	sugar phosphate isomerase/epimerase	1	0	0	0	1	1	1	0	1	1	1	7	Accessory	COG1082_G#	37.6	181	4.04E-45	NA	36.9	173	3.84E-42
5749	OSC70827.1	-	hypothetical protein	0	1	0	0	0	0	0	0	0	0	0	1	Unique	COG2267_I#	38.1	66	1.45E-10	K11275	31.8	61	1.25E-08
5750	Prokka:JAPCLKMA_03243	-	hypothetical protein	1	0	1	1	1	1	1	1	1	1	1	10	Accessory	COG1477_H#	34.7	136	1.14E-31	K03734	33.9	94	1.74E-18
5751	WP_102641914.1	-	succinate dehydrogenase/fumarate reductase iron-sulfur subunit	1	1	1	1	1	1	1	1	1	1	1	11	CORE	COG0479_C#	74.6	403	6.54E-112	K00240	74.6	403	1.75E-111
5752	OSC69908.1	-	hypothetical protein	0	1	0	0	0	0	0	0	0	0	0	1	Unique								
5753	OSC61397.1	-	oxidoreductase	1	1	1	1	1	1	1	1	1	1	1	11	CORE	COG1028_IQR#	45.6	176	1.7E-43	NA	38.1	166	4.69E-40

cluster	GI	gene	Annotation	Streptomyces_thermocarboxydus_BPSAC147	Streptomyces_sp._4F	Streptomyces_sp._BSE7F	Streptomyces_sp._ETH9427	Streptomyces_sp._FxanaD5	Streptomyces_sp._UNC401CLCol	Actinospica_acidiphila_NRRL_B_24431	Streptomyces_sp._HNS054	Streptomyces_sp._SMS_SU21	Streptomyces_sp._ZS0098	Streptomyces_thermocarboxydus_K155	Addition	Core-Accessory-Unique	COG	%Similarity_COG	Score_COG	Evalue_COG	KEGG	%Similarity_KEGG	Score_KEGG	Evalue_KEGG
5754	WP_078611577.1	-	FadR family transcriptional regulator	0	0	0	0	1	1	0	0	0	0	1	3	Accessory	COG2186_K#	31	84	6.73E-16	K03710	27.3	47	0.000243
5755	OSC56636.1	-	sugar phosphate isomerase	0	1	0	0	0	0	0	0	0	0	0	1	Unique					NA	24.3	65	6.61E-10
5756	OSC69645.1	-	NmrA family protein	0	1	0	0	0	0	0	0	0	0	0	1	Unique	COG0702_MG#	32.2	107	9.69E-23				
5757	AXI91273.1	-	AAA family ATPase	0	0	0	1	0	0	0	0	0	0	0	1	Unique	COG1484_L#	39.4	186	1.26E-46	NA	32	127	2.42E-28
5758	Prokka:JAPCLKMA_05250	btuD_15	Vitamin B12 import ATP-binding protein BtuD	1	1	1	1	1	1	1	1	1	1	1	11	CORE	COG1116_P#	45.6	216	8.65E-56	K02049	45.6	216	2.31E-55
5759	WP_020699955.1	-	hypothetical protein	0	0	0	0	1	1	0	0	0	0	1	3	Accessory								
5760	Prokka:JAPCLKMA_02390	cmoM_3	tRNA 5-carboxymethoxyuridine methyltransferase	1	0	0	0	1	1	0	0	0	0	1	4	Accessory	COG0500_QR#	30.5	99	2.63E-20	K06219	30.5	99	7.02E-20
5761	WP_102638787.1	-	metallophosphoesterase	1	1	1	1	1	1	1	1	1	1	1	11	CORE								
5762	OSC57382.1	-	protein tyrosine phosphatase	1	1	1	1	1	1	1	1	1	1	1	11	CORE	COG2365_T#	29.4	118	4.19E-26	K01104	32	111	1.05E-23
5763	Prokka:JAPCLKMA_04592	-	hypothetical protein	1	1	1	1	1	1	1	1	1	1	1	11	CORE					NA	39	164	1.78E-39

cluster	GI	gene	Annotation	Streptomyces_thermocarboxydus_BPSAC147	Streptomyces_sp._4F	Streptomyces_sp._BSE7F	Streptomyces_sp._ETH9427	Streptomyces_sp._FxanaD5	Streptomyces_sp._UNC401CLCol	Actinospica_acidiphila_NRRL_B_24431	Streptomyces_sp._HNS054	Streptomyces_sp._SMS_SU21	Streptomyces_sp._ZS0098	Streptomyces_thermocarboxydus_K155	Addition	Core-Accessory-Unique	COG	%Similarity_COG	Score_COG	Evalue_COG	KEGG	%Similarity_KEGG	Score_KEGG	Evalue_KEGG
5764	WP_033274941.1	-	type III pantothenate kinase	1	1	1	1	1	1	1	1	1	1	1	11	CORE	COG1521_K#	50.9	270	6.59E-72	K03525	50.2	265	4.34E-70
5765	WP_028960334.1	-	thioesterase family protein	1	0	1	1	1	1	1	1	1	1	1	10	Accessory								
5766	AXI87529.1	-	hypothetical protein	1	1	1	1	1	1	1	1	1	1	1	11	CORE					NA	48.4	105	7.5E-22
5767	OSC57457.1	-	carbon-nitrogen hydrolase	1	1	1	1	1	1	1	1	1	1	1	11	CORE	COG0388_R#	45.1	206	8.95E-53	NA	32	140	2.1E-32
5768	OSC66248.1	-	ABC transporter	0	1	0	0	0	0	0	0	0	0	0	1	Unique								
5769	PWE11973.1	-	GNAT family N-acetyltransferase	1	0	1	1	1	1	1	1	1	1	1	10	Accessory					NA	39.2	160	2.57E-38
5770	WP_024887131.1	-	ABC transporter ATP-binding protein	1	1	1	1	1	1	1	1	1	1	1	11	CORE	COG1119_P#	53.7	270	6.59E-72	K02013	53.7	270	1.76E-71
5771	OSC69423.1	-	carbon-nitrogen hydrolase	1	1	1	1	1	1	1	1	1	1	1	11	CORE	COG0388_R#	37.2	169	2.07E-41	K13566	36.6	158	7.47E-38
5772	WP_033273406.1	-	alpha/beta hydrolase	0	0	0	0	0	0	1	0	0	0	0	1	Unique	COG0596_R#	39.1	81	5.67E-15	K13703	30.8	85	1.05E-15
5773	Prokka:CIODPCDJ_05213	-	hypothetical protein	0	0	0	0	0	0	0	0	0	0	1	1	Unique								
5774	WP_019527081.1	-	type II secretion system F family protein	1	1	1	1	1	1	1	1	1	1	1	11	CORE	COG2064_NU#	38.5	101	4.06E-21	NA	38.5	101	1.08E-20
5775	PWE09292.1	-	hypothetical protein	0	0	1	0	0	0	0	0	0	0	0	1	Unique								

cluster	GI	gene	Annotation	Streptomyces_thermocarboxydus_BPSAC147	Streptomyces_sp._4F	Streptomyces_sp._BSE7F	Streptomyces_sp._ETH9427	Streptomyces_sp._FxanaD5	Streptomyces_sp._UNC401CLCol	Actinospica_acidiphila_NRRL_B_24431	Streptomyces_sp._HNS054	Streptomyces_sp._SMS_SU21	Streptomyces_sp._ZS0098	Streptomyces_thermocarboxydus_K155	Addition	Core-Accessory-Unique	COG	%Similarity_COG	Score_COG	Evalue_COG	KEGG	%Similarity_KEGG	Score_KEGG	Evalue_KEGG
5776	OSC62187.1	-	hydroxylase	1	1	1	1	1	1	1	1	1	1	1	11	CORE	COG3324_R#	31.7	128	4.05E-29	K06996	31.9	119	3.84E-26
5777	Prokka:CIODPCDJ_00515	panB_1	3-methyl-2-oxobutanoate hydroxymethyltransferase	0	0	0	0	1	1	0	0	0	0	1	3	Accessory	COG0413_H#	35	142	2.07E-33	K00606	30.8	121	1.73E-26
5778	Prokka:CIODPCDJ_05980	-	hypothetical protein	1	0	0	0	1	1	0	0	0	0	1	4	Accessory	COG1853_R#	24.6	44	0.000588	NA	21.7	47	0.000317
5779	OSC71674.1	-	transcriptional regulator	0	1	0	0	0	0	0	0	0	0	0	1	Unique	COG1167_KE#	40.4	51	0.00000481				
5780	OSC61338.1	-	hypothetical protein	1	1	1	1	1	1	1	1	1	1	1	11	CORE	COG3300_T#	42.2	129	1.82E-29	K11527	35.5	104	1.67E-21
5781	OSC65714.1	-	enhanced serine sensitivity protein SseB	1	1	1	1	1	1	1	1	1	1	1	11	CORE								
5782	AXI85552.1	-	GDSL family lipase	1	1	1	1	1	1	1	1	1	1	1	11	CORE	COG2755_E#	34.9	138	2.99E-32				
5783	AXI91388.1	-	hypothetical protein	0	0	0	1	0	0	0	0	0	0	0	1	Unique								
5784	OSC73263.1	-	ABC transporter permease	0	1	0	0	0	0	0	0	0	0	0	1	Unique	COG1177_E#	46.8	235	2.35E-61	K11070	28.7	109	6.79E-23
5785	WP_122216922.1	-	FtsQ-type POTRA domain-containing protein	1	1	1	1	1	1	1	1	1	1	1	11	CORE	COG1589_M#	28.1	95	2.9E-19	K03589	28.1	95	7.73E-19

cluster	GI	gene	Annotation	Streptomyces_thermocarboxydus_BPSAC147	Streptomyces_sp._4F	Streptomyces_sp._BSE7F	Streptomyces_sp._ETH9427	Streptomyces_sp._FxanaD5	Streptomyces_sp._UNC401CLCol	Actinospica_acidiphila_NRRL_B_24431	Streptomyces_sp._HNS054	Streptomyces_sp._SMS_SU21	Streptomyces_sp._ZS0098	Streptomyces_thermocarboxydus_K155	Addition	Core-Accessory-Unique	COG	%Similarity_COG	Score_COG	Evalue_COG	KEGG	%Similarity_KEGG	Score_KEGG	Evalue_KEGG
5786	WP_033277346.1	-	GntR family transcriptional regulator	0	0	0	0	1	0	1	0	0	0	0	2	Accessory	COG2188_K#	24.4	60	1.03E-08	NA	25.7	50	0.0000285
5787	WP_102641881.1	-	thioesterase	0	0	1	0	0	0	0	0	1	0	0	2	Accessory	COG3208_Q#	41.1	169	1.58E-41	NA	32.3	143	3.23E-33
5788	Prokka:JAPCLKMA_06456	-	hypothetical protein	1	0	1	1	1	1	1	1	1	0	1	9	Accessory	COG1396_K#	28.9	44	0.000766				
5789	AXI88389.1	-	TetR family transcriptional regulator	1	1	1	1	1	1	1	1	1	1	1	11	CORE	COG1309_K#	28.7	60	7.91E-09	NA	33.3	55	6.79E-07
5790	WP_026243788.1	-	membrane protein	1	1	1	1	1	1	1	1	1	1	1	11	CORE								
5791	WP_122213040.1	-	VOC family protein	1	0	1	1	1	1	0	1	1	1	1	9	Accessory	COG3324_R#	34.3	164	5.08E-40	K06996	34.3	164	1.36E-39
5792	WP_019526976.1	-	ATP-binding protein	0	0	0	0	1	0	0	0	0	1	0	2	Accessory	COG1484_L#	43.9	196	9.23E-50	NA	32.7	126	5.34E-28
5793	WP_122216496.1	-	SDR family oxidoreductase	1	1	1	0	1	1	1	1	1	1	1	10	Accessory	COG1028_IQR#	56.3	275	2.04E-73	NA	49.2	239	3.32E-62
5794	PWE08061.1	-	ABC transporter permease	1	1	1	1	1	1	1	1	1	1	1	11	CORE	COG0842_V#	40.9	198	3.17E-50	K01992	23.7	57	2.33E-07
5795	WP_019527906.1	-	class II aldolase/adducin family protein	1	1	1	1	1	1	1	1	1	1	1	11	CORE	COG0235_G#	57.3	316	7.99E-86	K18622	34.5	121	1.72E-26
5796	Prokka:JAPCLKMA_01269	-	hypothetical protein	1	1	1	0	1	1	1	1	1	1	1	10	Accessory	COG1985_H#	40.7	161	5.61E-39	K00082	40.7	161	1.5E-38

cluster	GI	gene	Annotation	Streptomyces_thermocarboxydus_BPSAC147	Streptomyces_sp._4F	Streptomyces_sp._BSE7F	Streptomyces_sp._ETH9427	Streptomyces_sp._FxanaD5	Streptomyces_sp._UNC401CLCol	Actinospica_acidiphila_NRRL_B_24431	Streptomyces_sp._HNS054	Streptomyces_sp._SMS_SU21	Streptomyces_sp._ZS0098	Streptomyces_thermocarboxydus_K155	Addition	Core-Accessory-Unique	COG	%Similarity_COG	Score_COG	Evalue_COG	KEGG	%Similarity_KEGG	Score_KEGG	Evalue_KEGG
5797	WP_026243819.1	-	SDR family NAD(P)-dependent oxidoreductase	0	1	0	0	1	1	1	0	0	0	1	5	Accessory	COG1028_IQR#	41.4	171	4.15E-42	K00059	39.5	157	2.16E-37
5798	WP_102642027.1	-	aquaporin family protein	1	1	1	1	1	1	1	1	1	1	1	11	CORE	COG0580_G#	52.7	258	2.58E-68	K02440	50.4	251	8.43E-66
5799	OSC70660.1	-	hypothetical protein	0	1	0	0	0	0	0	0	0	0	0	1	Unique								
5800	WP_102639514.1	-	site-2 protease family protein	1	1	1	1	1	1	1	1	1	1	1	11	CORE	COG1994_R#	41.5	189	1.47E-47	NA	41.5	189	3.94E-47
5801	Prokka:JAPCLKMA_00637	cbiM_1	Cobalt transport protein CbiM	1	1	1	1	1	1	1	1	1	1	1	11	CORE	COG0310_P#	56.3	250	5.39E-66	K02007	50.5	206	2.38E-52
5802	AXI86303.1	-	thiazole synthase	1	0	1	1	1	1	1	1	1	1	1	10	Accessory	COG2022_H#	71.7	341	3.96E-93	K03149	71.3	337	1.53E-91
5803	OSC61313.1	-	IclR family transcriptional regulator	1	1	0	1	1	0	1	0	0	1	0	6	Accessory	COG1414_K#	33.7	114	4.61E-25	K13641	33.7	114	1.23E-24
5804	WP_102641758.1	prcA	proteasome subunit alpha	1	1	0	1	1	1	1	1	1	1	1	10	Accessory	COG0638_O#	54.2	248	2.67E-65	K03432	54.2	248	7.14E-65
5805	WP_122214811.1	-	hypothetical protein	0	0	1	1	0	0	0	1	1	1	0	5	Accessory								
5806	OSC61173.1	-	SAM-dependent methyltransferase	0	1	0	0	0	0	0	0	0	0	0	1	Unique	COG2226_H#	36.8	64	7.15E-10	NA	29	62	7.26E-09
5807	OSC61349.1	-	glycosyltransferase	0	1	0	0	0	0	0	0	0	0	0	1	Unique	COG0463_M#	34.7	45	0.000263				
5808	OSC61410.1	-	hypothetical protein	1	1	1	0	1	1	1	1	1	1	1	10	Accessory								

cluster	GI	gene	Annotation	Streptomyces_thermocarboxydus_BPSAC147	Streptomyces_sp._4F	Streptomyces_sp._BSE7F	Streptomyces_sp._ETH9427	Streptomyces_sp._FxanaD5	Streptomyces_sp._UNC401CLCol	Actinospica_acidiphila_NRRL_B_24431	Streptomyces_sp._HNS054	Streptomyces_sp._SMS_SU21	Streptomyces_sp._ZS0098	Streptomyces_thermocarboxydus_K155	Addition	Core-Accessory-Unique	COG	%Similarity_COG	Score_COG	Evalue_COG	KEGG	%Similarity_KEGG	Score_KEGG	Evalue_KEGG
5809	WP_051724620.1	-	urease accessory protein UreD	1	0	1	1	1	1	1	1	1	1	1	10	Accessory					K03190	33.3	73	4.11E-12
5810	OSC54620.1	-	hypothetical protein	0	1	0	0	0	0	0	0	0	0	0	1	Unique								
5811	OSC65732.1	-	hypothetical protein	0	1	0	0	0	0	0	0	0	0	0	1	Unique	COG3409_M#	58.3	50	0.000014	K01449	40.9	47	0.000315
5812	Prokka:CIODPCDJ_03066	-	hypothetical protein	0	1	1	1	0	1	1	1	1	1	1	9	Accessory								
5813	WP_019525143.1	ehuA	ectoine/hydroxyectoine ABC transporter ATP-binding protein EhuA	1	1	1	1	1	1	1	1	1	1	1	11	CORE	COG1126_E#	59.9	290	7.98E-78	K02028	50.4	242	3.9E-63
5814	WP_048459572.1	-	ABC transporter ATP-binding protein	1	1	1	1	1	1	1	1	1	1	1	11	CORE	COG1131_V#	53	238	2.11E-62	K01990	53	238	5.64E-62
5815	WP_019522157.1	-	amino acid ABC transporter ATP-binding protein	1	1	1	1	1	1	1	1	1	1	1	11	CORE	COG1126_E#	62.5	310	4.37E-84	K02028	61.1	295	3.88E-79
5816	PWE08572.1	-	radical SAM protein	0	0	1	0	0	0	0	0	0	0	0	1	Unique	COG0535_R#	24.5	47	0.0000901				
5817	OSC57249.1	-	hypothetical protein	0	1	0	0	0	0	0	0	0	0	0	1	Unique								

cluster	GI	gene	Annotation	Streptomyces_thermocarboxydus_BPSAC147	Streptomyces_sp._4F	Streptomyces_sp._BSE7F	Streptomyces_sp._ETH9427	Streptomyces_sp._FxanaD5	Streptomyces_sp._UNC401CLCol	Actinospica_acidiphila_NRRL_B_24431	Streptomyces_sp._HNS054	Streptomyces_sp._SMS_SU21	Streptomyces_sp._ZS0098	Streptomyces_thermocarboxydus_K155	Addition	Core-Accessory-Unique	COG	%Similarity_COG	Score_COG	Evalue_COG	KEGG	%Similarity_KEGG	Score_KEGG	Evalue_KEGG
5818	WP_033274480.1	-	enoyl-CoA hydratase/isomerase family protein	1	0	1	1	1	1	1	1	1	1	1	10	Accessory	COG1024_I#	34.1	107	7.34E-23	K01661	31.7	93	3.82E-18
5819	PWE10110.1	-	transcriptional regulator	1	0	1	1	1	1	1	1	1	1	1	10	Accessory	COG1396_K#	51.3	240	7.25E-63				
5820	Prokka:CIODPCDJ_01794	ydjF	Phage shock protein A	1	1	1	1	1	1	1	1	1	1	1	11	CORE	COG1842_KT#	55.4	253	1.08E-66	K03969	31.4	116	5.51E-25
5821	PWE08415.1	-	hypothetical protein	0	0	1	0	0	0	0	0	1	0	0	2	Accessory								
5822	WP_028960900.1	-	N-formylglutamate amidohydrolase	1	0	1	1	1	1	1	1	1	1	1	10	Accessory								
5823	WP_122212673.1	-	hypothetical protein	1	1	0	1	1	1	1	1	1	1	1	10	Accessory	COG3480_T#	54.6	120	8.37E-27	K07177	39.8	119	6.51E-26
5824	WP_048460637.1	-	ABC transporter ATP-binding protein	1	1	1	1	0	1	1	1	1	1	1	10	Accessory	COG1134_GM#	44.6	186	9.52E-47	K09693	44.6	188	6.69E-47
5825	OSC65319.1	-	hypothetical protein	1	1	1	1	1	1	1	1	1	1	1	11	CORE	COG1357_S#	35.5	138	3.88E-32	NA	28.4	56	5.18E-07
5826	WP_033272640.1	-	redox-sensing transcriptional repressor Rex	1	1	1	1	1	1	1	1	1	1	1	11	CORE	COG2344_R#	42.6	168	3.5E-41	K01926	39.9	156	4.8E-37
5827	Prokka:CIODPCDJ_05232	frcA_1	Fructose import ATP-binding protein FrcA	1	1	1	1	1	1	1	1	1	1	1	11	CORE	COG1129_G#	65.2	316	1.04E-85	K02056	39.6	179	5.3E-44

cluster	GI	gene	Annotation	Streptomyces_thermocarboxydus_BPSAC147	Streptomyces_sp._4F	Streptomyces_sp._BSE7F	Streptomyces_sp._ETH9427	Streptomyces_sp._FxanaD5	Streptomyces_sp._UNC401CLCol	Actinospica_acidiphila_NRRL_B_24431	Streptomyces_sp._HNS054	Streptomyces_sp._SMS_SU21	Streptomyces_sp._ZS0098	Streptomyces_thermocarboxydus_K155	Addition	Core-Accessory-Unique	COG	%Similarity_COG	Score_COG	Evalue_COG	KEGG	%Similarity_KEGG	Score_KEGG	Evalue_KEGG
5828	OSC61736.1	-	short chain dehydrogenase	0	1	0	0	0	0	0	0	0	0	0	1	Unique	COG1028_IQR#	48.9	228	2.85E-59	K00059	34.8	127	2.39E-28
5829	OSC62641.1	-	ABC transporter ATP-binding protein	1	1	1	1	1	1	1	1	1	1	1	11	CORE	COG0410_E#	49	230	5.75E-60	K01996	47.6	221	9.31E-57
5830	AXI91250.1	-	GntR family transcriptional regulator	0	0	0	1	0	0	0	0	0	0	0	1	Unique	COG2188_K#	29.6	69	1.7E-11	NA	49.2	57	1.78E-07
5831	OSC64137.1	-	hypothetical protein	1	1	0	1	1	1	1	1	1	1	1	10	Accessory								
5832	WP_033275368.1	-	DUF4111 domain-containing protein	1	0	1	0	1	1	1	1	1	1	1	9	Accessory								
5833	WP_019522721.1	-	ferritin-like domain-containing protein	1	1	1	1	1	1	1	1	1	1	1	11	CORE								
5834	OSC71389.1	-	carbon-nitrogen hydrolase family protein	0	1	0	0	0	0	0	0	0	0	0	1	Unique	COG0388_R#	30.6	47	0.0000687				
5835	WP_048456436.1	-	electron transfer flavoprotein subunit beta/FixA family protein	1	1	1	1	1	1	1	1	1	1	1	11	CORE	COG2086_C#	51.9	258	1.96E-68	K03521	51.9	258	5.24E-68
5836	WP_019524275.1	-	aldose 1-epimerase	1	1	1	1	1	1	1	1	1	1	1	11	CORE								

cluster	GI	gene	Annotation	Streptomyces_thermocarboxydus_BPSAC147	Streptomyces_sp._4F	Streptomyces_sp._BSE7F	Streptomyces_sp._ETH9427	Streptomyces_sp._FxanaD5	Streptomyces_sp._UNC401CLCol	Actinospica_acidiphila_NRRL_B_24431	Streptomyces_sp._HNS054	Streptomyces_sp._SMS_SU21	Streptomyces_sp._ZS0098	Streptomyces_thermocarboxydus_K155	Addition	Core-Accessory-Unique	COG	%Similarity_COG	Score_COG	Evalue_COG	KEGG	%Similarity_KEGG	Score_KEGG	Evalue_KEGG
5837	AXI86835.1	-	phosphatase	1	1	1	1	1	1	1	1	1	1	1	11	CORE								
5838	WP_026243812.1	-	TVP38/TMEM64 family protein	1	1	1	1	1	1	1	1	1	1	1	11	CORE	COG0398_S#	32.2	101	4.01E-21	NA	32.2	101	1.07E-20
5839	WP_033277997.1	-	methyltransferase domain-containing protein	1	1	1	1	1	1	1	1	1	1	1	11	CORE	COG0500_QR#	37.3	139	1.33E-32	NA	37.3	139	3.55E-32
5840	OSC71586.1	-	hypothetical protein	0	1	0	0	0	0	0	0	0	0	0	1	Unique					NA	44.7	193	2.7E-48
5841	WP_028960748.1	-	hypothetical protein	1	1	1	1	1	1	1	1	1	1	1	11	CORE								
5842	WP_086704612.1	-	DUF2344 domain-containing protein	1	1	1	1	1	1	1	1	1	1	1	11	CORE	COG5011_S#	28.7	78	3.64E-14	NA	29	80	1.96E-14
5843	PWE10542.1	-	hypothetical protein	0	0	1	0	0	0	0	0	1	0	0	2	Accessory	COG0664_T#	28.3	89	2.69E-17	NA	27.7	87	2.73E-16
5844	WP_122212965.1	-	acetoacetate decarboxylase	1	0	0	0	1	1	1	0	0	1	1	6	Accessory								
5845	WP_028961474.1	-	hypothetical protein	1	0	1	1	1	1	1	1	1	1	1	10	Accessory								
5846	WP_122216163.1	-	hypothetical protein	0	0	0	0	0	0	0	0	0	1	1	2	Accessory								
5847	WP_019524590.1	cas6e	type I-E CRISPR-associated protein Cas6/Cse3/CasE	0	0	0	0	1	0	0	0	0	0	0	1	Unique					K19126	47.2	47	0.000313
5848	WP_033274203.1	-	SDR family oxidoreductase	0	1	0	0	0	0	1	0	0	0	0	2	Accessory	COG1028_IQR#	40.7	168	4.56E-41	K00540	40.2	151	9.02E-36

cluster	GI	gene	Annotation	Streptomyces_thermocarboxydus_BPSAC147	Streptomyces_sp._4F	Streptomyces_sp._BSE7F	Streptomyces_sp._ETH9427	Streptomyces_sp._FxanaD5	Streptomyces_sp._UNC401CLCol	Actinospica_acidiphila_NRRL_B_24431	Streptomyces_sp._HNS054	Streptomyces_sp._SMS_SU21	Streptomyces_sp._ZS0098	Streptomyces_thermocarboxydus_K155	Addition	Core-Accessory-Unique	COG	%Similarity_COG	Score_COG	Evalue_COG	KEGG	%Similarity_KEGG	Score_KEGG	Evalue_KEGG
5849	OSC62611.1	-	CPBP family intramembrane metalloprotease	0	1	0	0	0	0	0	0	0	0	0	1	Unique	COG1266_R#	25.7	98	4.43E-20	K07052	28.6	62	7.2E-09
5850	Prokka:JAPCLKMA_01624	pcm_1	Protein-L-isoaspartate O-methyltransferase	1	0	0	0	0	0	0	0	0	0	0	1	Unique	COG2518_O#	37.4	109	1.47E-23	K00573	38.1	104	1.65E-21
5851	WP_102639934.1	-	class I SAM-dependent methyltransferase	0	1	0	0	0	0	0	0	1	0	0	2	Accessory	COG0500_QR#	28.7	55	2.52E-07	NA	28.7	55	6.74E-07
5852	OSC62144.1	-	hypothetical protein	1	1	1	1	1	1	1	1	1	1	1	11	CORE								
5853	WP_122215891.1	-	SDR family oxidoreductase	0	0	0	0	0	0	0	0	0	1	0	1	Unique	COG1028_IQR#	45.1	205	2.58E-52	K00038	45.1	205	6.88E-52
5854	Prokka:JAPCLKMA_02180	-	hypothetical protein	1	0	0	0	1	1	1	0	0	0	1	5	Accessory								
5855	AXI91329.1	-	hypothetical protein	0	0	0	1	0	0	0	0	0	0	0	1	Unique								
5856	AXI88191.1	-	ADP-ribose pyrophosphatase	0	0	0	1	1	0	0	0	0	0	0	2	Accessory	COG0494_LR#	32.7	73	1.53E-12	NA	46.6	58	1.04E-07
5857	WP_033277589.1	pcaH	protocatechuate 3,4-dioxygenase subunit beta	1	1	0	0	1	1	1	0	0	0	1	6	Accessory	COG3485_Q#	53.4	238	2.75E-62				
5858	Prokka:JAPCLKMA_00578	srtE2	Sortase SrtE2	1	1	1	1	1	1	1	1	1	1	1	11	CORE	COG3764_M#	32.9	79	2.78E-14				

cluster	GI	gene	Annotation	Streptomyces_thermocarboxydus_BPSAC147	Streptomyces_sp._4F	Streptomyces_sp._BSE7F	Streptomyces_sp._ETH9427	Streptomyces_sp._FxanaD5	Streptomyces_sp._UNC401CLCol	Actinospica_acidiphila_NRRL_B_24431	Streptomyces_sp._HNS054	Streptomyces_sp._SMS_SU21	Streptomyces_sp._ZS0098	Streptomyces_thermocarboxydus_K155	Addition	Core-Accessory-Unique	COG	%Similarity_COG	Score_COG	Evalue_COG	KEGG	%Similarity_KEGG	Score_KEGG	Evalue_KEGG
5859	Prokka:JAPCLKMA_05570	-	hypothetical protein	1	0	0	0	1	1	0	0	0	0	1	4	Accessory								
5860	OSC61215.1	-	ABC transporter ATP-binding protein	0	1	0	0	0	0	0	0	0	0	0	1	Unique	COG1123_R#	46.3	217	6.55E-56	K02032	48.7	213	2.53E-54
5861	OSC71900.1	-	oxidoreductase	1	1	1	1	1	1	1	1	1	1	1	11	CORE	COG1028_IQR#	57.2	261	3.02E-69	K19336	44.9	193	2.69E-48
5862	WP_102639494.1	-	META domain-containing protein	1	1	1	1	1	1	1	1	1	1	0	10	Accessory	COG3187_O#	33.3	44	0.00058				
5863	Prokka:CIODPCDJ_04671	-	hypothetical protein	0	0	0	0	0	0	0	0	0	0	1	1	Unique								
5864	PWE09336.1	nagB	glucosamine-6-phosphate deaminase	1	1	1	1	1	1	1	1	1	1	1	11	CORE	COG0363_G#	45	211	3.58E-54	K02564	43.1	203	2.6E-51
5865	Prokka:JAPCLKMA_00823	nagR_1	HTH-type transcriptional repressor NagR	1	1	1	1	1	1	1	1	1	1	1	11	CORE	COG2188_K#	49.6	233	1.15E-60	K03710	32.2	136	5.11E-31
5866	Prokka:JAPCLKMA_04882	-	hypothetical protein	1	0	0	0	0	0	0	0	0	0	0	1	Unique								
5867	AXI90901.1	-	ABC transporter	0	0	1	1	0	0	0	1	1	1	0	5	Accessory	COG0842_V#	35.4	147	8.29E-35	K01992	31.6	93	2.91E-18
5868	Prokka:CIODPCDJ_02788	ygiD	4,5-DOPA dioxygenase extradiol	1	1	1	1	1	1	1	1	1	1	1	11	CORE	COG3384_S#	46.4	199	1.84E-50	NA	37.7	166	3.53E-40

cluster	GI	gene	Annotation	Streptomyces_thermocarboxydus_BPSAC147	Streptomyces_sp._4F	Streptomyces_sp._BSE7F	Streptomyces_sp._ETH9427	Streptomyces_sp._FxanaD5	Streptomyces_sp._UNC401CLCol	Actinospica_acidiphila_NRRL_B_24431	Streptomyces_sp._HNS054	Streptomyces_sp._SMS_SU21	Streptomyces_sp._ZS0098	Streptomyces_thermocarboxydus_K155	Addition	Core-Accessory-Unique	COG	%Similarity_COG	Score_COG	Evalue_COG	KEGG	%Similarity_KEGG	Score_KEGG	Evalue_KEGG
5869	PWE10433.1	-	SGNH/GDSL hydrolase family protein	0	1	1	1	1	1	1	1	1	1	0	9	Accessory	COG2755_E#	34.5	144	7.02E-34				
5870	Prokka:JAPCLKMA_00900	walR_1	Transcriptional regulatory protein WalR	1	0	0	0	1	1	0	0	0	0	1	4	Accessory	COG0745_TK#	40.2	170	7E-42	K07668	38.3	164	1.03E-39
5871	WP_102640849.1	-	carboxypeptidase regulatory-like domain-containing protein	1	0	1	1	1	1	1	1	1	1	1	10	Accessory								
5872	OSC59786.1	-	histidinol phosphatase	0	1	0	0	0	0	0	0	0	0	0	1	Unique	COG1120_PH#	52.5	238	2.73E-62	K02013	42	196	4.16E-49
5873	WP_048458063.1	-	Crp/Fnr family transcriptional regulator	0	0	0	1	0	0	0	1	0	1	0	3	Accessory	COG0664_T#	32.1	89	1.57E-17	NA	32.1	89	4.2E-17
5874	OSC66904.1	-	2-hydroxyhepta-2,4-diene-1,7-dioate isomerase	1	1	1	1	1	1	1	1	1	1	1	11	CORE	COG0179_Q#	53.1	247	7.69E-65	NA	53.1	247	2.05E-64
5875	WP_019524920.1	cas5e	type I-E CRISPR-associated protein Cas5/CasD	0	0	0	0	1	0	0	0	0	0	1	2	Accessory								
5876	WP_019525420.1	-	recombinase family protein	0	0	0	0	1	0	0	0	0	0	0	1	Unique								

cluster	GI	gene	Annotation	Streptomyces_thermocarboxydus_BPSAC147	Streptomyces_sp._4F	Streptomyces_sp._BSE7F	Streptomyces_sp._ETH9427	Streptomyces_sp._FxanaD5	Streptomyces_sp._UNC401CLCol	Actinospica_acidiphila_NRRL_B_24431	Streptomyces_sp._HNS054	Streptomyces_sp._SMS_SU21	Streptomyces_sp._ZS0098	Streptomyces_thermocarboxydus_K155	Addition	Core-Accessory-Unique	COG	%Similarity_COG	Score_COG	Evalue_COG	KEGG	%Similarity_KEGG	Score_KEGG	Evalue_KEGG
5877	Prokka:CIODPCDJ_02994	nagR_3	HTH-type transcriptional repressor NagR	0	0	0	0	0	1	0	0	0	0	1	2	Accessory	COG2188_K#	30.6	97	7.53E-20	K03710	31.8	92	4.96E-18
5878	Prokka:JAPCLKMA_06323	glpR_3	Glycerol-3-phosphate regulon repressor	1	0	1	1	1	1	1	1	1	0	1	9	Accessory	COG1349_KG#	36.4	128	3.05E-29	K03436	29.2	111	1.35E-23
5879	WP_033274248.1	-	HAD family phosphatase	0	1	1	1	1	1	1	1	1	1	1	10	Accessory	COG0637_R#	26.7	73	1.52E-12	NA	29.1	58	1.04E-07
5880	WP_033277626.1	-	enoyl-CoA hydratase	1	1	1	1	1	1	1	1	1	1	1	11	CORE	COG1024_I#	36.7	138	2.95E-32	K15016	33.3	136	3.91E-31
5881	OSC71220.1	-	hypothetical protein	1	1	0	0	0	0	0	0	0	0	0	2	Accessory								
5882	PWE10534.1	-	DUF1963 domain-containing protein	0	0	1	1	0	0	0	1	1	1	0	5	Accessory								
5883	WP_028960427.1	-	crotonase	1	0	0	0	1	1	0	0	0	0	1	4	Accessory	COG1024_I#	40.6	182	2.33E-45	K08299	38.5	182	6.21E-45
5884	WP_048455618.1	-	isocitrate lyase/phosphoenolpyruvate mutase family protein	1	1	0	1	1	1	1	1	1	1	1	10	Accessory	COG2513_G#	46.2	186	9.44E-47	NA	46.2	186	2.52E-46
5885	Prokka:JAPCLKMA_02499	yfcA_1	putative membrane transporter protein YfcA	1	1	1	1	1	1	1	1	1	1	1	11	CORE	COG0730_R#	41.7	189	1.45E-47	K07090	33.9	151	1.53E-35

cluster	GI	gene	Annotation	Streptomyces_thermocarboxydus_BPSAC147	Streptomyces_sp._4F	Streptomyces_sp._BSE7F	Streptomyces_sp._ETH9427	Streptomyces_sp._FxanaD5	Streptomyces_sp._UNC401CLCol	Actinospica_acidiphila_NRRL_B_24431	Streptomyces_sp._HNS054	Streptomyces_sp._SMS_SU21	Streptomyces_sp._ZS0098	Streptomyces_thermocarboxydus_K155	Addition	Core-Accessory-Unique	COG	%Similarity_COG	Score_COG	Evalue_COG	KEGG	%Similarity_KEGG	Score_KEGG	Evalue_KEGG
5886	AXI86466.1	-	peroxide stress protein YaaA	1	1	1	1	1	1	1	1	1	1	1	11	CORE	COG3022_S#	41.2	148	2.84E-35				
5887	WP_019525015.1	-	sulfite exporter TauE/SafE family protein	1	0	1	0	1	1	1	0	1	0	1	7	Accessory	COG0730_R#	30.3	112	2.94E-24	NA	33.3	48	0.000139
5888	PWE08194.1	-	ABC transporter permease	0	0	1	0	0	0	0	0	1	1	0	3	Accessory								
5889	WP_019521591.1	-	3-oxoacyl-ACP reductase	1	1	1	1	1	1	1	1	1	1	1	11	CORE	COG1028_IQR#	72.5	369	1.02E-101	NA	72.5	369	2.73E-101
5890	WP_019521469.1	-	CoA transferase subunit A	1	1	0	0	1	1	0	0	0	0	1	5	Accessory	COG1788_I#	69.1	334	4.76E-91	K01028	69.1	334	1.27E-90
5891	AXI86008.1	-	sporulation protein	1	1	1	1	1	1	1	1	1	1	1	11	CORE	COG4326_R#	33.3	125	2.57E-28	K06377	34.3	129	3.64E-29
5892	Prokka:CIODPCDJ_06050	-	hypothetical protein	1	1	0	0	1	1	0	0	0	0	1	5	Accessory	COG0489_D#	30.1	48	0.00004	NA	30.3	107	1.94E-22
5893	WP_028958962.1	-	hypothetical protein	1	1	1	1	1	1	1	1	1	1	1	11	CORE								
5894	Prokka:JAPCLKMA_02396	-	hypothetical protein	1	0	0	0	0	0	0	0	0	0	0	1	Unique	COG1511_S#	33.3	40	0.00834	NA	31.3	77	2.8E-13
5895	WP_033276755.1	-	ABC transporter permease	1	1	1	0	0	0	1	0	1	1	1	7	Accessory	COG0842_V#	29.1	133	9.45E-31				
5896	AXI89181.1	-	antagonist protein	0	0	1	1	0	0	0	1	1	1	0	5	Accessory								
5897	OSC56637.1	-	triose-phosphate isomerase	0	1	0	0	0	0	0	0	0	0	0	1	Unique	COG0149_G#	38.1	133	1.23E-30	K01803	38.1	133	3.3E-30

cluster	GI	gene	Annotation	Streptomyces_thermocarboxydus_BPSAC147	Streptomyces_sp._4F	Streptomyces_sp._BSE7F	Streptomyces_sp._ETH9427	Streptomyces_sp._FxanaD5	Streptomyces_sp._UNC401CLCol	Actinospica_acidiphila_NRRL_B_24431	Streptomyces_sp._HNS054	Streptomyces_sp._SMS_SU21	Streptomyces_sp._ZS0098	Streptomyces_thermocarboxydus_K155	Addition	Core-Accessory-Unique	COG	%Similarity_COG	Score_COG	Evalue_COG	KEGG	%Similarity_KEGG	Score_KEGG	Evalue_KEGG
5898	WP_033273230.1	-	DUF881 domain-containing protein	1	1	1	1	1	1	1	1	1	1	1	11	CORE	COG3879_S#	39	167	7.71E-41	NA	39	167	2.06E-40
5899	WP_086020209.1	-	hypothetical protein	1	1	1	1	1	1	1	1	1	1	1	11	CORE								
5900	AXI91357.1	-	hypothetical protein	0	0	0	1	0	0	0	0	0	0	0	1	Unique								
5901	OSC66603.1	-	ABC transporter ATP-binding protein	0	1	0	0	0	0	0	0	0	0	0	1	Unique	COG1131_V#	48.2	220	7.68E-57	K01990	43.5	204	1.16E-51
5902	Prokka:JAPCLKMA_01235	-	hypothetical protein	1	0	0	0	0	0	0	0	0	0	0	1	Unique								
5903	OSC70776.1	-	multidrug ABC transporter permease	1	1	0	0	0	0	1	0	0	0	1	4	Accessory	COG0842_V#	28.1	127	8.85E-29				
5904	Prokka:CIODPCDJ_02621	-	hypothetical protein	0	0	0	0	0	0	0	0	0	0	1	1	Unique					NA	28.8	54	0.00000195
5905	Prokka:CIODPCDJ_01693	-	hypothetical protein	0	0	0	0	0	0	0	0	0	0	1	1	Unique								
5906	OSC61957.1	-	NADPH-dependent ferric siderophore reductase	0	1	0	0	0	0	0	0	0	0	0	1	Unique	COG2375_P#	28.7	108	4.25E-23	NA	28.4	83	3.91E-15
5907	WP_033272609.1	-	SDR family NAD(P)-dependent oxidoreductase	1	1	1	1	1	1	1	1	1	1	1	11	CORE	COG4221_R#	56.1	250	9.01E-66	NA	56.1	250	2.41E-65

cluster	GI	gene	Annotation	Streptomyces_thermocarboxydus_BPSAC147	Streptomyces_sp._4F	Streptomyces_sp._BSE7F	Streptomyces_sp._ETH9427	Streptomyces_sp._FxanaD5	Streptomyces_sp._UNC401CLCol	Actinospica_acidiphila_NRRL_B_24431	Streptomyces_sp._HNS054	Streptomyces_sp._SMS_SU21	Streptomyces_sp._ZS0098	Streptomyces_thermocarboxydus_K155	Addition	Core-Accessory-Unique	COG	%Similarity_COG	Score_COG	Evalue_COG	KEGG	%Similarity_KEGG	Score_KEGG	Evalue_KEGG
5908	WP_093767169.1	-	HAD family hydrolase	1	1	1	1	1	1	1	1	1	1	1	11	CORE	COG0647_G#	64.7	331	2.36E-90	K02566	43.1	218	5.94E-56
5909	WP_028961827.1	-	SseB family protein	1	1	1	1	1	1	1	1	1	1	1	11	CORE								
5910	Prokka:CIODPCDJ_02688	-	hypothetical protein	1	1	1	1	1	1	1	1	1	1	1	11	CORE					NA	39.7	153	2.35E-36
5911	WP_084207028.1	-	FkbM family methyltransferase	0	0	1	0	0	0	1	1	1	1	0	5	Accessory	COG0500_QR#	36.1	68	3.72E-11	NA	36.1	68	9.93E-11
5912	WP_102641337.1	-	(d)CMP kinase	1	1	1	1	1	1	1	1	1	1	1	11	CORE	COG0283_F#	49.1	194	5.87E-49	K00945	46.4	174	1.29E-42
5913	PWE09506.1	-	alpha/beta hydrolase	1	1	1	1	1	1	1	1	1	1	1	11	CORE	COG0596_R#	41.9	195	2.63E-49				
5914	Prokka:JAPCLKMA_06337	-	hypothetical protein	1	1	1	1	1	1	1	1	1	1	1	11	CORE	COG0745_TK#	58.8	289	1.03E-77	NA	58.8	289	2.74E-77
5915	OSC71321.1	-	hypothetical protein	0	1	0	0	0	0	0	0	0	0	0	1	Unique								
5916	WP_102637713.1	xth	exodeoxyribonuclease III	1	1	1	1	1	1	1	1	1	1	1	11	CORE	COG0708_L#	52.1	267	7.13E-71	K01142	52.1	267	1.9E-70
5917	WP_107080980.1	-	ABC transporter permease subunit	1	1	1	1	1	1	1	1	1	1	1	11	CORE								
5918	Prokka:CIODPCDJ_04672	-	hypothetical protein	0	0	0	0	0	0	0	0	0	0	1	1	Unique								
5919	OSC71997.1	-	RNA polymerase subunit sigma	1	1	1	1	1	1	1	1	1	1	1	11	CORE								

cluster	GI	gene	Annotation	Streptomyces_thermocarboxydus_BPSAC147	Streptomyces_sp._4F	Streptomyces_sp._BSE7F	Streptomyces_sp._ETH9427	Streptomyces_sp._FxanaD5	Streptomyces_sp._UNC401CLCol	Actinospica_acidiphila_NRRL_B_24431	Streptomyces_sp._HNS054	Streptomyces_sp._SMS_SU21	Streptomyces_sp._ZS0098	Streptomyces_thermocarboxydus_K155	Addition	Core-Accessory-Unique	COG	%Similarity_COG	Score_COG	Evalue_COG	KEGG	%Similarity_KEGG	Score_KEGG	Evalue_KEGG
5920	WP_048459008.1	-	GntR family transcriptional regulator	0	0	1	0	1	0	0	1	0	0	0	3	Accessory	COG2188_K#	30.9	98	3.36E-20	K03710	30.9	98	8.96E-20
5921	WP_033277001.1	lexA	transcriptional repressor LexA	1	1	1	1	1	1	1	1	1	1	1	11	CORE	COG1974_KT#	70.5	311	3.29E-84	K01356	66.5	311	8.8E-84
5922	WP_048460819.1	sdhB	succinate dehydrogenase iron-sulfur subunit	1	1	1	1	1	1	1	1	1	1	1	11	CORE	COG0479_C#	63.3	334	2.78E-91	K00240	63.3	334	7.43E-91
5923	Prokka:JAPCLKMA_06505	-	hypothetical protein	1	0	0	0	0	0	0	0	0	0	0	1	Unique	COG3064_M#	29.1	43	0.00167				
5924	OSC69943.1	-	aldo/keto reductase	0	1	0	0	0	0	0	0	0	0	0	1	Unique	COG0667_C#	42.7	201	3.68E-51	NA	42.2	189	5.04E-47
5925	AXI91278.1	-	hypothetical protein	0	0	0	1	0	0	0	0	0	0	0	1	Unique								
5926	Prokka:CIODPCDJ_06746	-	hypothetical protein	0	0	0	0	0	0	0	0	0	0	1	1	Unique								
5927	OSC71474.1	-	GntR family transcriptional regulator	0	1	0	0	0	0	0	0	0	0	0	1	Unique	COG2188_K#	32.8	99	1.51E-20	K03710	28.7	82	8.68E-15
5928	Prokka:CIODPCDJ_00070	-	hypothetical protein	0	0	0	0	1	1	0	0	0	0	1	3	Accessory								
5929	OSC63293.1	-	SAM-dependent methyltransferase	1	1	1	1	1	1	1	1	1	1	1	11	CORE	COG0500_QR#	39.1	162	1.89E-39	NA	34.9	52	0.00000962

cluster	GI	gene	Annotation	Streptomyces_thermocarboxydus_BPSAC147	Streptomyces_sp._4F	Streptomyces_sp._BSE7F	Streptomyces_sp._ETH9427	Streptomyces_sp._FxanaD5	Streptomyces_sp._UNC401CLCol	Actinospica_acidiphila_NRRL_B_24431	Streptomyces_sp._HNS054	Streptomyces_sp._SMS_SU21	Streptomyces_sp._ZS0098	Streptomyces_thermocarboxydus_K155	Addition	Core-Accessory-Unique	COG	%Similarity_COG	Score_COG	Evalue_COG	KEGG	%Similarity_KEGG	Score_KEGG	Evalue_KEGG
5930	WP_033273811.1	map	type I methionyl aminopeptidase	1	1	1	1	1	1	1	1	1	1	1	11	CORE	COG0024_J#	53.5	251	4.05E-66	K01265	53.5	251	1.08E-65
5931	WP_122215688.1	-	polysaccharide deacetylase family protein	1	1	0	0	1	1	1	0	0	1	1	7	Accessory	COG0726_G#	38.2	150	7.44E-36	NA	36.3	147	1.68E-34
5932	OSC61171.1	-	short-chain dehydrogenase	0	1	1	1	0	0	1	1	1	1	1	8	Accessory	COG1028_IQR#	44.3	190	6.49E-48	K00059	42.8	155	8.07E-37
5933	WP_107503755.1	-	hypothetical protein	0	0	1	0	0	0	0	1	1	0	0	3	Accessory								
5934	Prokka:JAPCLKMA_01030	-	hypothetical protein	1	0	0	0	0	0	0	0	0	0	0	1	Unique								
5935	PWE09792.1	-	polysaccharide lyase family 7 protein	0	0	1	0	0	0	0	0	0	0	0	1	Unique								
5936	WP_102638151.1	-	helix-turn-helix domain-containing protein	1	1	1	1	1	1	0	1	1	1	1	10	Accessory								
5937	Prokka:JAPCLKMA_02487	-	hypothetical protein	1	1	1	1	1	1	1	1	1	1	1	11	CORE								
5938	WP_019525268.1	-	triose-phosphate isomerase	1	1	1	1	1	1	1	1	1	1	1	11	CORE	COG0149_G#	67.2	346	1.2E-94	K01803	67.2	346	3.21E-94
5939	WP_102637468.1	-	N(G),N(G)-dimethylarginine dimethylaminohydrolase	1	1	0	1	1	1	1	1	1	1	1	10	Accessory	COG1834_E#	35.8	144	4.07E-34	K01482	32.1	117	2.43E-25

cluster	GI	gene	Annotation	Streptomyces_thermocarboxydus_BPSAC147	Streptomyces_sp._4F	Streptomyces_sp._BSE7F	Streptomyces_sp._ETH9427	Streptomyces_sp._FxanaD5	Streptomyces_sp._UNC401CLCol	Actinospica_acidiphila_NRRL_B_24431	Streptomyces_sp._HNS054	Streptomyces_sp._SMS_SU21	Streptomyces_sp._ZS0098	Streptomyces_thermocarboxydus_K155	Addition	Core-Accessory-Unique	COG	%Similarity_COG	Score_COG	Evalue_COG	KEGG	%Similarity_KEGG	Score_KEGG	Evalue_KEGG
5940	WP_019521586.1	-	SDR family oxidoreductase	1	1	1	1	1	1	1	1	1	1	1	11	CORE	COG1028_IQR#	48.2	235	1.75E-61	K00059	34.4	131	1.62E-29
5941	Prokka:JAPCLKMA_04811	-	hypothetical protein	1	1	1	1	1	1	1	1	1	1	1	11	CORE								
5942	WP_048460690.1	-	SDR family oxidoreductase	0	1	1	1	0	0	1	1	1	1	0	7	Accessory	COG1028_IQR#	51.4	234	5.1E-61	K00059	39.2	163	3.85E-39
5943	WP_019527575.1	-	hypothetical protein	1	0	1	1	1	1	1	1	1	1	1	10	Accessory								
5944	PWE08000.1	-	ABC transporter ATP-binding protein	1	1	1	1	1	1	1	1	1	1	1	11	CORE	COG1131_V#	51.4	242	1.43E-63	K01990	38.7	167	1.57E-40
5945	WP_026244027.1	-	sigma-70 family RNA polymerase sigma factor	1	1	0	1	1	1	1	1	1	1	1	10	Accessory	COG1595_K#	32.5	92	1.83E-18	K03088	32.5	92	4.9E-18
5946	AXI90453.1	-	MerR family transcriptional regulator	0	0	1	1	0	0	0	1	1	1	0	5	Accessory	COG0789_K#	28.8	72	2.57E-12	K06042	34.1	59	7.84E-08
5947	Prokka:JAPCLKMA_05520	pstB1	Phosphate import ATP-binding protein PstB 1	1	1	1	1	1	1	1	1	1	1	1	11	CORE	COG1117_P#	74.8	403	4.86E-112	K02036	74.8	401	4.93E-111
5948	AXI89913.1	cobF	precorrin-6A synthase (deacetylating)	1	1	1	1	1	1	1	1	1	1	1	11	CORE	COG2243_H#	50.6	255	2.79E-67				

cluster	GI	gene	Annotation	Streptomyces_thermocarboxydus_BPSAC147	Streptomyces_sp._4F	Streptomyces_sp._BSE7F	Streptomyces_sp._ETH9427	Streptomyces_sp._FxanaD5	Streptomyces_sp._UNC401CLCol	Actinospica_acidiphila_NRRL_B_24431	Streptomyces_sp._HNS054	Streptomyces_sp._SMS_SU21	Streptomyces_sp._ZS0098	Streptomyces_thermocarboxydus_K155	Addition	Core-Accessory-Unique	COG	%Similarity_COG	Score_COG	Evalue_COG	KEGG	%Similarity_KEGG	Score_KEGG	Evalue_KEGG
5949	PWE08106.1	-	2-C-methyl-D-erythritol 4-phosphate cytidylyltransferase	1	1	1	1	1	1	1	1	1	1	1	11	CORE	COG1211_I#	42.8	166	1.31E-40	K00991	43.6	166	3.49E-40
5950	Prokka:CIODPCDJ_03870	menG_2	Demethylmenaquinone methyltransferase	1	1	1	1	1	1	1	1	1	1	1	11	CORE	COG2226_H#	65.5	298	2.2E-80	K03183	65.5	298	5.88E-80
5951	Prokka:JAPCLKMA_02475	catD_1	3-oxoadipate enol-lactonase 2	1	0	0	0	1	0	0	0	0	0	1	3	Accessory	COG0596_R#	32.6	124	4.36E-28	NA	23.9	74	1.38E-12
5952	Prokka:JAPCLKMA_01501	-	hypothetical protein	1	0	0	1	0	0	0	0	0	0	0	2	Accessory								
5953	WP_122215324.1	-	hypothetical protein	0	0	0	0	0	0	0	0	1	1	0	2	Accessory								
5954	WP_048458922.1	-	ABC transporter permease	0	0	0	1	0	0	0	1	0	0	0	2	Accessory								
5955	OSC53463.1	-	hypothetical protein	0	1	0	0	0	0	0	0	0	0	0	1	Unique								
5956	WP_048456176.1	-	alpha/beta hydrolase	0	0	0	0	0	0	0	1	0	0	0	1	Unique	COG0596_R#	30.5	83	1.11E-15	NA	29.1	73	4.02E-12
5957	WP_122216608.1	-	IclR family transcriptional regulator	0	0	0	0	0	0	0	0	0	1	0	1	Unique	COG1414_K#	28.5	111	4.98E-24	NA	35.3	107	1.92E-22
5958	OSC63344.1	-	hypothetical protein	0	1	0	0	0	0	0	0	0	0	0	1	Unique					NA	41.2	46	0.000526

cluster	GI	gene	Annotation	Streptomyces_thermocarboxydus_BPSAC147	Streptomyces_sp._4F	Streptomyces_sp._BSE7F	Streptomyces_sp._ETH9427	Streptomyces_sp._FxanaD5	Streptomyces_sp._UNC401CLCol	Actinospica_acidiphila_NRRL_B_24431	Streptomyces_sp._HNS054	Streptomyces_sp._SMS_SU21	Streptomyces_sp._ZS0098	Streptomyces_thermocarboxydus_K155	Addition	Core-Accessory-Unique	COG	%Similarity_COG	Score_COG	Evalue_COG	KEGG	%Similarity_KEGG	Score_KEGG	Evalue_KEGG
5959	WP_051724702.1	-	class I SAM-dependent methyltransferase	0	0	0	0	0	0	1	0	0	0	0	1	Unique	COG0500_QR#	34.4	116	2.02E-25	NA	28.2	77	1.63E-13
5960	OSC54129.1	-	DEAD/DEAH box helicase	0	1	0	0	0	0	0	0	0	0	0	1	Unique	COG1204_R#	27.5	70	1.27E-11	NA	32.3	68	1.29E-10
5961	OSC72152.1	-	DNA-binding protein	1	1	1	1	1	1	1	1	1	1	1	11	CORE	COG1272_R#	36.5	160	7.16E-39	K11068	36.5	160	1.91E-38
5962	AXI85317.1	-	DNA ligase	0	0	0	1	0	0	0	0	0	0	0	1	Unique	COG1793_L#	32.3	101	3.95E-21	NA	32.3	101	1.05E-20
5963	PWE11822.1	-	uracil-DNA glycosylase	1	1	1	1	1	1	1	1	1	1	1	11	CORE	COG0692_L#	72.2	339	1.13E-92	K03648	72.2	339	3.01E-92
5964	Prokka:CIODPCDJ_04028	-	hypothetical protein	0	0	0	0	0	0	0	0	0	0	1	1	Unique	COG0732_V#	34.2	49	0.0000304	NA	27.3	58	1.34E-07
5965	AXI89637.1	-	glycosyl hydrolase	0	0	0	1	0	0	0	0	0	0	0	1	Unique								
5966	OSC73342.1	-	signal peptidase I	1	1	1	1	1	1	1	1	1	1	1	11	CORE	COG0681_U#	43	79	2.1E-14	K03100	43	79	5.61E-14
5967	Prokka:JAPCLKMA_01329	-	hypothetical protein	1	0	0	0	0	0	0	0	0	0	0	1	Unique								
5968	WP_019522358.1	-	isoprenyl transferase	1	0	1	1	1	1	1	1	1	1	1	10	Accessory	COG0020_I#	58.5	258	1.93E-68	K12503	51.4	251	1.07E-65
5969	WP_033277797.1	-	CDP-alcohol phosphatidyltransferase family protein	1	0	1	1	1	1	1	1	1	1	1	10	Accessory	COG0558_I#	34.3	81	4.21E-15	K00993	30.7	46	0.000401

cluster	GI	gene	Annotation	Streptomyces_thermocarboxydus_BPSAC147	Streptomyces_sp._4F	Streptomyces_sp._BSE7F	Streptomyces_sp._ETH9427	Streptomyces_sp._FxanaD5	Streptomyces_sp._UNC401CLCol	Actinospica_acidiphila_NRRL_B_24431	Streptomyces_sp._HNS054	Streptomyces_sp._SMS_SU21	Streptomyces_sp._ZS0098	Streptomyces_thermocarboxydus_K155	Addition	Core-Accessory-Unique	COG	%Similarity_COG	Score_COG	Evalue_COG	KEGG	%Similarity_KEGG	Score_KEGG	Evalue_KEGG
5970	Prokka:CIODPCDJ_02052	xynR_1	HTH-type transcriptional regulator XynR	1	1	1	1	1	1	1	1	1	1	1	11	CORE	COG1414_K#	34.4	154	6.68E-37	NA	35.1	139	4.55E-32
5971	WP_019522746.1	-	SDR family oxidoreductase	1	1	1	1	1	1	1	1	1	1	1	11	CORE	COG1028_IQR#	40.3	181	2.99E-45	NA	40.3	181	7.99E-45
5972	WP_033272593.1	-	cytochrome C biogenesis protein CcdA	1	1	1	1	1	1	1	1	1	1	1	11	CORE	COG0785_O#	44.4	206	1.48E-52	K06196	44.4	206	3.96E-52
5973	Prokka:CIODPCDJ_00531	lldR_1	Putative L-lactate dehydrogenase operon regulatory protein	0	0	0	0	1	1	0	0	0	0	1	3	Accessory	COG2186_K#	32.3	109	1.89E-23	K05799	29.4	102	4.72E-21
5974	AXI88310.1	-	SDR family NAD(P)-dependent oxidoreductase	1	1	1	1	1	1	1	1	1	1	1	11	CORE	COG0300_R#	38	169	1.54E-41	K07124	38.2	148	1.28E-34
5975	OSC61079.1	-	ABC transporter	1	1	0	1	1	1	1	1	1	1	1	10	Accessory	COG0842_V#	43.5	198	4.03E-50	K01992	43.5	198	1.08E-49
5976	Prokka:JAPCLKMA_04747	fabG_11	3-oxoacyl-[acyl-carrier-protein] reductase FabG	1	0	0	0	1	1	0	0	0	0	1	4	Accessory	COG1028_IQR#	53.9	252	1.38E-66	K00059	39.1	149	3.36E-35

cluster	GI	gene	Annotation	Streptomyces_thermocarboxydus_BPSAC147	Streptomyces_sp._4F	Streptomyces_sp._BSE7F	Streptomyces_sp._ETH9427	Streptomyces_sp._FxanaD5	Streptomyces_sp._UNC401CLCol	Actinospica_acidiphila_NRRL_B_24431	Streptomyces_sp._HNS054	Streptomyces_sp._SMS_SU21	Streptomyces_sp._ZS0098	Streptomyces_thermocarboxydus_K155	Addition	Core-Accessory-Unique	COG	%Similarity_COG	Score_COG	Evalue_COG	KEGG	%Similarity_KEGG	Score_KEGG	Evalue_KEGG
5977	WP_019527042.1	-	TetR/AcrR family transcriptional regulator	1	1	1	1	1	1	1	1	1	1	1	11	CORE	COG1309_K#	30.5	94	6.28E-19	NA	28.3	81	1.47E-14
5978	WP_033276266.1	-	aldolase	0	0	0	0	1	1	1	1	0	1	1	6	Accessory	COG3836_G#	32.4	113	1.31E-24	K01630	31.8	104	1.24E-21
5979	Prokka:CIODPCDJ_05602	-	hypothetical protein	1	1	1	1	1	1	1	1	1	1	1	11	CORE								
5980	WP_026243963.1	-	methyltransferase domain-containing protein	1	0	1	1	1	1	0	1	1	1	1	9	Accessory	COG2226_H#	33.2	124	4.34E-28	NA	33.2	124	1.16E-27
5981	Prokka:JAPCLKMA_00773	-	hypothetical protein	1	1	1	1	1	1	1	1	1	1	1	11	CORE	COG0739_M#	34.7	97	5.68E-20	NA	37.8	89	7.05E-17
5982	WP_102639961.1	-	GvpL/GvpF family gas vesicle protein	1	1	1	1	1	1	1	1	1	1	1	11	CORE								
5983	OSC58876.1	-	beta-galactosidase	0	1	0	0	0	0	0	0	0	0	0	1	Unique	COG1874_G#	34.2	116	1.54E-25	K12309	40.4	139	3.48E-32
5984	WP_033277755.1	-	dienelactone hydrolase family protein	1	1	1	1	1	1	1	1	1	1	1	11	CORE	COG0412_Q#	45	188	4.17E-47	K01061	45	188	1.11E-46
5985	WP_019526067.1	-	hypothetical protein	0	0	0	0	1	0	0	0	0	0	0	1	Unique	COG2319_R#	24	44	0.000973				
5986	AXI84714.1	-	thioesterase	0	0	1	1	0	0	0	1	1	1	0	5	Accessory	COG3208_Q#	38	159	1.59E-38	NA	36.7	158	9.46E-38
5987	Prokka:JAPCLKMA_05756	-	hypothetical protein	1	1	1	1	1	1	1	1	1	1	1	11	CORE	COG1595_K#	68.4	251	4.01E-66	K03088	63.1	247	1.19E-64

cluster	GI	gene	Annotation	Streptomyces_thermocarboxydus_BPSAC147	Streptomyces_sp._4F	Streptomyces_sp._BSE7F	Streptomyces_sp._ETH9427	Streptomyces_sp._FxanaD5	Streptomyces_sp._UNC401CLCol	Actinospica_acidiphila_NRRL_B_24431	Streptomyces_sp._HNS054	Streptomyces_sp._SMS_SU21	Streptomyces_sp._ZS0098	Streptomyces_thermocarboxydus_K155	Addition	Core-Accessory-Unique	COG	%Similarity_COG	Score_COG	Evalue_COG	KEGG	%Similarity_KEGG	Score_KEGG	Evalue_KEGG
5988	OSC62865.1	-	short-chain dehydrogenase	0	1	0	1	0	0	0	1	0	0	0	3	Accessory	COG1028_IQR#	55.3	268	1.86E-71	K00059	39	136	3.85E-31
5989	WP_048458467.1	-	IclR family transcriptional regulator	0	0	1	0	0	1	0	1	1	0	1	5	Accessory	COG1414_K#	32.3	127	5.13E-29	K13641	32.3	127	1.37E-28
5990	PWE08636.1	-	hypothetical protein	0	0	1	0	0	0	0	0	0	0	0	1	Unique								
5991	OSC60083.1	-	SAM-dependent methyltransferase	0	1	0	0	0	0	0	0	0	0	0	1	Unique	COG0500_QR#	29.4	106	2.09E-22	NA	34.1	75	6.17E-13
5992	WP_102640668.1	-	transcriptional regulator	0	0	0	0	0	0	0	0	1	0	0	1	Unique								
5993	OSC63091.1	-	aminotransferase	0	1	0	0	0	0	0	0	0	0	0	1	Unique	COG0115_EH#	39.6	132	1.59E-30				
5994	PWE07374.1	-	SAM-dependent methyltransferase	0	0	1	1	0	0	1	1	1	1	0	6	Accessory	COG0500_QR#	42.4	166	9.96E-41	NA	42.4	166	2.66E-40
5995	Prokka:JAPCLKMA_01998	-	hypothetical protein	1	0	0	0	0	0	0	0	0	0	0	1	Unique								
5996	WP_159020939.1	-	hypothetical protein	0	0	0	1	0	0	0	1	0	0	0	2	Accessory								
5997	Prokka:CIODPCDJ_02027	-	hypothetical protein	0	0	1	1	1	1	1	1	1	1	1	9	Accessory								
5998	WP_122213571.1	-	GTP cyclohydrolase I FolE2	0	0	0	0	0	0	0	0	0	1	0	1	Unique	COG1469_S#	38.4	181	2.99E-45	K09007	38.4	181	7.99E-45

cluster	GI	gene	Annotation	Streptomyces_thermocarboxydus_BPSAC147	Streptomyces_sp._4F	Streptomyces_sp._BSE7F	Streptomyces_sp._ETH9427	Streptomyces_sp._FxanaD5	Streptomyces_sp._UNC401CLCol	Actinospica_acidiphila_NRRL_B_24431	Streptomyces_sp._HNS054	Streptomyces_sp._SMS_SU21	Streptomyces_sp._ZS0098	Streptomyces_thermocarboxydus_K155	Addition	Core-Accessory-Unique	COG	%Similarity_COG	Score_COG	Evalue_COG	KEGG	%Similarity_KEGG	Score_KEGG	Evalue_KEGG
5999	WP_122213371.1	-	glucose 1-dehydrogenase	0	0	0	0	0	0	0	0	0	1	0	1	Unique	COG1028_IQR#	38.9	155	3E-37	K00034	38.9	155	8.01E-37
6000	OSC72092.1	-	hypothetical protein	0	1	0	0	0	0	0	0	0	0	0	1	Unique	COG2771_K#	41.9	60	1.31E-08	K11618	38.5	60	2.06E-08
6001	WP_048457391.1	-	ABC transporter ATP-binding protein	1	0	1	1	1	1	1	1	1	1	1	10	Accessory	COG1122_P#	47.9	224	4.01E-58	K02006	45.4	217	1.71E-55
6002	Prokka:CIODPCDJ_06212	-	hypothetical protein	1	0	0	0	1	1	1	0	0	0	1	5	Accessory	COG1234_R#	32.8	131	6.03E-30	K00784	30.6	99	3.98E-20
6003	WP_086020124.1	-	hypothetical protein	1	1	1	1	1	1	1	1	1	1	1	11	CORE								
6004	WP_019527021.1	-	glucose 1-dehydrogenase	1	1	1	1	1	1	1	1	1	1	1	11	CORE	COG1028_IQR#	43.8	175	2.79E-43	K00059	40.1	161	1.45E-38
6005	WP_102638636.1	-	TetR/AcrR family transcriptional regulator	1	1	0	1	1	1	1	1	1	1	1	10	Accessory	COG1309_K#	34	86	2.23E-16	NA	31.9	50	0.0000361
6006	WP_102638783.1	-	methyltransferase domain-containing protein	1	1	1	1	1	1	1	1	1	1	1	11	CORE	COG0500_QR#	33.2	103	1.03E-21	K06219	30.6	98	8.86E-20
6007	WP_019522754.1	-	maleylpyruvate isomerase family mycothiol-dependent enzyme	1	0	0	0	1	1	0	0	0	0	1	4	Accessory								
6008	AXI86642.1	-	IS5/IS1182 family transposase	1	1	0	1	1	1	0	0	0	0	1	6	Accessory								

cluster	GI	gene	Annotation	Streptomyces_thermocarboxydus_BPSAC147	Streptomyces_sp._4F	Streptomyces_sp._BSE7F	Streptomyces_sp._ETH9427	Streptomyces_sp._FxanaD5	Streptomyces_sp._UNC401CLCol	Actinospica_acidiphila_NRRL_B_24431	Streptomyces_sp._HNS054	Streptomyces_sp._SMS_SU21	Streptomyces_sp._ZS0098	Streptomyces_thermocarboxydus_K155	Addition	Core-Accessory-Unique	COG	%Similarity_COG	Score_COG	Evalue_COG	KEGG	%Similarity_KEGG	Score_KEGG	Evalue_KEGG
6009	Prokka:JAPCLKMA_04779	hypB	Hydrogenase maturation factor HypB	1	0	0	1	1	1	0	1	0	1	1	7	Accessory	COG0378_OK#	48.3	175	2.14E-43	K04652	48.3	175	5.7E-43
6010	WP_048457032.1	-	GntR family transcriptional regulator	1	1	1	1	1	1	1	1	1	1	1	11	CORE	COG1802_K#	32.9	92	2.38E-18	NA	32.4	84	1.32E-15
6011	PWE06939.1	-	DeoR/GlpR transcriptional regulator	1	1	1	1	1	1	1	1	1	1	1	11	CORE	COG1349_KG#	33.1	145	2.37E-34	K03436	33.1	145	6.32E-34
6012	WP_019524732.1	-	response regulator transcription factor	1	1	1	1	1	1	1	1	1	1	1	11	CORE	COG3279_KT#	34.8	128	2.99E-29	K07705	29.7	108	1.12E-22
6013	WP_019526974.1	-	ATP-binding protein	1	0	0	1	1	0	0	0	0	0	0	3	Accessory	COG1484_L#	41.6	220	9.87E-57	NA	38.1	173	3.7E-42
6014	OSC71406.1	-	AraC family transcriptional regulator	0	1	0	0	0	0	0	0	0	0	0	1	Unique	COG2207_K#	30.4	131	4.62E-30	NA	23.7	54	0.0000025
6015	WP_033274239.1	-	metal ABC transporter ATP-binding protein	0	1	1	1	1	1	1	1	1	1	1	10	Accessory	COG1121_P#	48.3	182	1.75E-45	K11710	38.5	138	7.73E-32
6016	OSC60992.1	-	hypothetical protein	0	1	0	0	0	0	0	0	0	0	0	1	Unique								
6017	WP_033278123.1	-	ABC transporter ATP-binding protein	1	1	1	1	1	1	1	1	1	1	1	11	CORE	COG1136_V#	51.8	236	7.8E-62	K02003	44.1	203	2.55E-51

cluster	GI	gene	Annotation	Streptomyces_thermocarboxydus_BPSAC147	Streptomyces_sp._4F	Streptomyces_sp._BSE7F	Streptomyces_sp._ETH9427	Streptomyces_sp._FxanaD5	Streptomyces_sp._UNC401CLCol	Actinospica_acidiphila_NRRL_B_24431	Streptomyces_sp._HNS054	Streptomyces_sp._SMS_SU21	Streptomyces_sp._ZS0098	Streptomyces_thermocarboxydus_K155	Addition	Core-Accessory-Unique	COG	%Similarity_COG	Score_COG	Evalue_COG	KEGG	%Similarity_KEGG	Score_KEGG	Evalue_KEGG
6018	WP_102637317.1	-	4'-phosphopantetheinyl transferase superfamily protein	0	0	1	0	0	0	0	0	1	0	0	2	Accessory	COG2091_H#	36.8	67	6.27E-11	K06133	28	53	0.00000327
6019	Prokka:JAPCLKMA_04830	-	hypothetical protein	1	0	0	0	1	0	0	0	0	0	0	2	Accessory								
6020	Prokka:JAPCLKMA_01523	-	hypothetical protein	1	0	0	1	0	0	0	0	0	0	0	2	Accessory	COG0463_M#	29.4	46	0.000195				
6021	OSC70934.1	-	hypothetical protein	1	1	0	0	0	1	0	0	0	0	1	4	Accessory	COG1404_O#	33.8	43	0.00165	NA	45.6	50	0.0000361
6022	Prokka:JAPCLKMA_01039	-	IS5 family transposase ISStsp4	1	1	0	0	0	0	0	0	0	0	0	2	Accessory								
6023	OSC70072.1	-	Clp protease	1	1	1	1	1	1	1	1	1	1	1	11	CORE	COG0542_O#	50	145	2.37E-34	NA	58.6	277	1.07E-73
6024	Prokka:CIODPCDJ_05734	mftE	Putative mycofactocin system creatinine amidohydrolase family protein MftE	1	1	1	1	1	1	1	1	1	1	1	11	CORE	COG1402_R#	37.2	138	4.91E-32	NA	33.2	102	4.68E-21
6025	Prokka:JAPCLKMA_03563	-	hypothetical protein	1	1	1	1	1	1	1	1	1	1	1	11	CORE								
6026	WP_033274115.1	-	GntR family transcriptional regulator	1	1	1	1	1	1	1	1	1	1	1	11	CORE	COG2188_K#	38.9	163	8.36E-40	K03710	38.9	163	2.23E-39

cluster	GI	gene	Annotation	Streptomyces_thermocarboxydus_BPSAC147	Streptomyces_sp._4F	Streptomyces_sp._BSE7F	Streptomyces_sp._ETH9427	Streptomyces_sp._FxanaD5	Streptomyces_sp._UNC401CLCol	Actinospica_acidiphila_NRRL_B_24431	Streptomyces_sp._HNS054	Streptomyces_sp._SMS_SU21	Streptomyces_sp._ZS0098	Streptomyces_thermocarboxydus_K155	Addition	Core-Accessory-Unique	COG	%Similarity_COG	Score_COG	Evalue_COG	KEGG	%Similarity_KEGG	Score_KEGG	Evalue_KEGG
6027	WP_019522100.1	fabI	enoyl-ACP reductase FabI	1	1	1	1	1	1	1	1	1	1	1	11	CORE	COG0623_I#	49.4	262	1.32E-69	K11611	47.7	260	1.34E-68
6028	AXI87891.1	-	DNA-binding response regulator	1	1	1	1	1	1	1	1	1	1	1	11	CORE	COG2197_TK#	29.7	110	1.1E-23	NA	33.5	106	5.53E-22
6029	WP_019524804.1	-	hypothetical protein	1	0	0	0	1	1	0	0	0	0	1	4	Accessory	COG0412_Q#	28.8	99	1.48E-20				
6030	Prokka:CIODPCDJ_05326	dinG_4	3'-5' exonuclease DinG	1	1	1	1	1	1	1	1	1	1	1	11	CORE	COG0847_L#	39.9	159	2.06E-38				
6031	WP_122214887.1	-	glycerophosphodiester phosphodiesterase	1	0	1	1	1	1	1	1	0	1	1	9	Accessory	COG0584_C#	42.2	187	7.06E-47	K01126	42.2	187	1.89E-46
6032	WP_019524348.1	-	hypothetical protein	1	1	1	1	1	1	1	1	1	1	1	11	CORE	COG1024_I#	47.6	205	1.92E-52	K01692	45.1	200	1.65E-50
6033	OSC69720.1	-	SAM-dependent methyltransferase	0	1	0	0	0	0	0	0	0	0	0	1	Unique	COG0500_QR#	30.9	87	7.62E-17	NA	30.9	87	2.03E-16
6034	WP_033276631.1	-	SUMF1/EgtB/PvdO family nonheme iron enzyme	0	0	0	1	0	0	1	1	0	0	0	3	Accessory	COG1262_S#	32.1	80	7.13E-15				
6035	WP_048458491.1	-	permease	1	1	1	1	1	1	1	1	1	1	1	11	CORE					K14711	29.7	57	1.73E-07
6036	WP_028961285.1	-	phosphotransferase	0	0	0	0	0	1	0	0	0	0	0	1	Unique								
6037	OSC73029.1	-	nucleotidyltransferase	0	1	0	0	0	0	0	0	0	0	0	1	Unique	COG1708_R#	42	185	2.68E-46				

cluster	GI	gene	Annotation	Streptomyces_thermocarboxydus_BPSAC147	Streptomyces_sp._4F	Streptomyces_sp._BSE7F	Streptomyces_sp._ETH9427	Streptomyces_sp._FxanaD5	Streptomyces_sp._UNC401CLCol	Actinospica_acidiphila_NRRL_B_24431	Streptomyces_sp._HNS054	Streptomyces_sp._SMS_SU21	Streptomyces_sp._ZS0098	Streptomyces_thermocarboxydus_K155	Addition	Core-Accessory-Unique	COG	%Similarity_COG	Score_COG	Evalue_COG	KEGG	%Similarity_KEGG	Score_KEGG	Evalue_KEGG
6038	Prokka:CIODPCDJ_02299	pgaB	Poly-beta-1,6-N-acetyl-D-glucosamine N-deacetylase	1	1	1	1	1	1	1	1	1	1	1	11	CORE	COG0726_G#	28.6	84	8.42E-16	NA	24.6	80	2.49E-14
6039	OSC67446.1	-	hypothetical protein	0	1	0	0	0	0	0	0	0	0	0	1	Unique								
6040	Prokka:JAPCLKMA_06044	-	hypothetical protein	1	0	0	0	0	0	0	0	0	0	0	1	Unique								
6041	Prokka:JAPCLKMA_01820	-	hypothetical protein	1	0	1	1	1	1	1	1	1	1	1	10	Accessory								
6042	OSC69687.1	-	XRE family transcriptional regulator	0	1	0	0	0	0	0	0	0	0	0	1	Unique								
6043	PWE09788.1	-	oxidoreductase	0	0	1	0	0	0	0	0	0	0	0	1	Unique	COG1028_IQR#	44	201	2.77E-51	K00059	41.2	176	3.33E-43
6044	OSC69660.1	-	hypothetical protein	0	1	0	0	0	0	0	0	0	0	0	1	Unique	COG3440_V#	55.3	152	1.93E-36	K07454	55.3	152	5.15E-36
6045	OSC54841.1	-	alpha/beta hydrolase	0	1	0	0	0	0	0	0	0	0	0	1	Unique	COG0596_R#	50.8	267	7.02E-71	NA	32.5	99	3.96E-20
6046	OSC59569.1	-	urease subunit gamma	1	1	0	1	1	1	1	1	0	1	1	9	Accessory	COG0831_E#	60	114	7.6E-25	K14048	51.1	210	1.59E-53
6047	OSC70269.1	-	cation-binding protein	1	1	0	1	1	0	1	1	0	1	1	8	Accessory								
6048	OSC68202.1	-	hydrolase	1	1	1	1	1	1	1	1	1	1	1	11	CORE	COG3324_R#	29.1	100	8.7E-21				
6049	Prokka:JAPCLKMA_01995	-	hypothetical protein	1	0	0	0	0	0	0	0	0	0	0	1	Unique								
6050	PWE11451.1	-	enoyl-CoA hydratase	0	0	1	0	0	0	0	0	1	0	0	2	Accessory	COG1024_I#	38	112	2.21E-24	K01692	38	112	5.91E-24

cluster	GI	gene	Annotation	Streptomyces_thermocarboxydus_BPSAC147	Streptomyces_sp._4F	Streptomyces_sp._BSE7F	Streptomyces_sp._ETH9427	Streptomyces_sp._FxanaD5	Streptomyces_sp._UNC401CLCol	Actinospica_acidiphila_NRRL_B_24431	Streptomyces_sp._HNS054	Streptomyces_sp._SMS_SU21	Streptomyces_sp._ZS0098	Streptomyces_thermocarboxydus_K155	Addition	Core-Accessory-Unique	COG	%Similarity_COG	Score_COG	Evalue_COG	KEGG	%Similarity_KEGG	Score_KEGG	Evalue_KEGG
6051	OSC70395.1	-	hypothetical protein	0	1	0	0	0	0	0	0	0	0	0	1	Unique								
6052	OSC62296.1	-	hypothetical protein	1	1	1	1	1	1	1	1	1	1	1	11	CORE								
6053	AXI86557.1	-	ABC transporter permease	0	0	1	1	1	1	1	1	1	1	1	9	Accessory	COG0767_Q#	51.2	265	1.56E-70	K02066	51.2	265	4.16E-70
6054	Prokka:CIODPCDJ_03758	menH_3	2-succinyl-6-hydroxy-2, 4-cyclohexadiene-1-carboxylate synthase	1	1	1	1	1	1	1	1	1	1	1	11	CORE	COG0596_R#	29.6	78	3.52E-14	NA	31.4	76	3.58E-13
6055	WP_033275705.1	sufC	Fe-S cluster assembly ATPase SufC	1	1	1	1	1	1	1	1	1	1	1	11	CORE	COG0396_O#	76.6	380	4.34E-105	K09013	76.6	380	1.16E-104
6056	WP_033276702.1	-	IclR family transcriptional regulator	1	1	1	1	1	1	1	1	1	1	1	11	CORE	COG1414_K#	34.7	136	1.86E-31	NA	32.3	127	1.35E-28
6057	PWE09273.1	-	tRNA 2-methylthio-N6-isopentenyl adenosine(37) hydroxylase MiaE-like protein	1	1	1	1	1	1	1	1	1	1	1	11	CORE					NA	39.6	184	1.59E-45

cluster	GI	gene	Annotation	Streptomyces_thermocarboxydus_BPSAC147	Streptomyces_sp._4F	Streptomyces_sp._BSE7F	Streptomyces_sp._ETH9427	Streptomyces_sp._FxanaD5	Streptomyces_sp._UNC401CLCol	Actinospica_acidiphila_NRRL_B_24431	Streptomyces_sp._HNS054	Streptomyces_sp._SMS_SU21	Streptomyces_sp._ZS0098	Streptomyces_thermocarboxydus_K155	Addition	Core-Accessory-Unique	COG	%Similarity_COG	Score_COG	Evalue_COG	KEGG	%Similarity_KEGG	Score_KEGG	Evalue_KEGG
6058	WP_122216676.1	-	helix-turn-helix domain-containing protein	1	1	1	1	1	1	1	1	1	1	1	11	CORE	COG3355_K#	29.2	49	0.0000229				
6059	Prokka:JAPCLKMA_04979	-	hypothetical protein	1	0	0	0	1	1	0	0	0	0	1	4	Accessory								
6060	WP_102638432.1	-	sulfite exporter TauE/SafE family protein	1	1	1	1	1	1	1	1	1	1	1	11	CORE	COG0730_R#	44.6	213	9.17E-55	K07090	26.1	89	4.08E-17
6061	WP_033276341.1	-	crotonase/enoyl-CoA hydratase family protein	1	0	1	1	1	1	1	1	1	1	1	10	Accessory	COG1024_I#	74.2	358	2.3E-98	K01692	74.2	358	6.15E-98
6062	OSC62030.1	-	pyrroline-5-carboxylate reductase	0	1	0	0	0	0	0	0	0	0	0	1	Unique					K00286	25.4	67	1.66E-10
6063	WP_033277105.1	-	SDR family oxidoreductase	0	0	0	0	0	0	1	0	0	1	0	2	Accessory	COG1028_IQR#	41.6	168	4.42E-41	K00540	37.9	159	5.48E-38
6064	Prokka:JAPCLKMA_02217	-	hypothetical protein	1	0	0	0	0	0	0	0	0	0	0	1	Unique								
6065	Prokka:CIODPCDJ_06813	-	hypothetical protein	0	0	0	0	0	0	0	0	0	0	1	1	Unique								
6066	OSC61924.1	-	3-alpha-hydroxysteroid dehydrogenase	0	1	0	0	0	0	0	0	0	0	0	1	Unique	COG1028_IQR#	50.8	230	5.55E-60	K00038	50.8	230	1.48E-59
6067	OSC48023.1	-	hypothetical protein	0	1	0	0	0	0	0	0	0	0	0	1	Unique	COG3321_Q#	48	199	1.37E-50	K12430	48	199	3.66E-50

cluster	GI	gene	Annotation	Streptomyces_thermocarboxydus_BPSAC147	Streptomyces_sp._4F	Streptomyces_sp._BSE7F	Streptomyces_sp._ETH9427	Streptomyces_sp._FxanaD5	Streptomyces_sp._UNC401CLCol	Actinospica_acidiphila_NRRL_B_24431	Streptomyces_sp._HNS054	Streptomyces_sp._SMS_SU21	Streptomyces_sp._ZS0098	Streptomyces_thermocarboxydus_K155	Addition	Core-Accessory-Unique	COG	%Similarity_COG	Score_COG	Evalue_COG	KEGG	%Similarity_KEGG	Score_KEGG	Evalue_KEGG
6068	AXI89513.1	-	DUF1365 domain-containing protein	0	0	0	1	0	0	0	1	1	1	0	4	Accessory	COG3496_S#	46.4	207	6.57E-53	K09701	46.4	193	2.62E-48
6069	OSC73220.1	-	IclR family transcriptional regulator	1	1	1	1	1	1	1	1	1	1	1	11	CORE	COG1414_K#	37	169	1.52E-41	NA	33.9	143	3.11E-33
6070	Prokka:CIODPCDJ_00884	-	hypothetical protein	0	0	0	0	0	0	0	0	0	0	1	1	Unique								
6071	OSC65504.1	-	ABC transporter ATP-binding protein	1	1	1	1	1	1	1	1	1	1	1	11	CORE	COG1136_V#	46.5	201	2.76E-51	K02003	45.9	192	5.84E-48
6072	OSC59413.1	-	NmrA family transcriptional regulator	0	1	0	0	0	0	0	0	0	0	0	1	Unique	COG0702_MG#	37.2	133	1.21E-30	K19267	30.6	85	1.01E-15
6073	WP_033276946.1	-	GntR family transcriptional regulator	1	1	1	1	1	1	1	1	1	1	1	11	CORE	COG2188_K#	35.3	92	3.07E-18	K03710	35.3	92	8.19E-18
6074	WP_122215960.1	-	MerR family transcriptional regulator	1	1	1	1	1	1	1	1	1	1	1	11	CORE	COG0789_K#	40	186	1.56E-46	NA	40	186	4.17E-46
6075	OSC55749.1	-	DNA-binding response regulator	0	1	0	0	0	0	0	0	0	0	0	1	Unique	COG0745_TK#	45.9	173	1.37E-42	K07776	45.9	173	3.65E-42
6076	OSC67538.1	-	hypothetical protein	0	1	0	0	1	0	0	0	0	0	0	2	Accessory								

cluster	GI	gene	Annotation	Streptomyces_thermocarboxydus_BPSAC147	Streptomyces_sp._4F	Streptomyces_sp._BSE7F	Streptomyces_sp._ETH9427	Streptomyces_sp._FxanaD5	Streptomyces_sp._UNC401CLCol	Actinospica_acidiphila_NRRL_B_24431	Streptomyces_sp._HNS054	Streptomyces_sp._SMS_SU21	Streptomyces_sp._ZS0098	Streptomyces_thermocarboxydus_K155	Addition	Core-Accessory-Unique	COG	%Similarity_COG	Score_COG	Evalue_COG	KEGG	%Similarity_KEGG	Score_KEGG	Evalue_KEGG
6077	WP_033276224.1	-	TIGR03089 family protein	1	1	1	1	1	1	1	1	1	1	1	11	CORE	COG0365_I#	43.3	44	0.000562	NA	39.6	142	5.29E-33
6078	WP_019523965.1	-	phosphoglyceromutase	1	1	1	1	1	1	1	1	1	1	1	11	CORE	COG0588_G#	71.5	352	9.64E-97	K01834	67.5	341	7.76E-93
6079	Prokka:JAPCLKMA_04766	-	hypothetical protein	1	0	1	1	0	0	1	1	1	1	0	7	Accessory	COG2513_G#	51.6	232	1.45E-60				
6080	WP_048460331.1	-	thioesterase	0	0	0	1	0	0	0	1	1	1	0	4	Accessory	COG3208_Q#	42.9	176	1.62E-43	K04788	40.8	163	3.78E-39
6081	WP_019526099.1	-	SDR family oxidoreductase	1	1	1	1	1	1	1	1	1	1	1	11	CORE	COG1028_IQR#	47.4	213	9.13E-55	K00059	45.2	200	2.79E-50
6082	WP_019524165.1	-	DeoR/GlpR transcriptional regulator	1	1	1	0	1	1	1	1	1	1	1	10	Accessory	COG1349_KG#	42.6	183	1.01E-45	K03436	33.3	140	2.62E-32
6083	OSC59423.1	-	GNAT family N-acetyltransferase	0	1	0	0	0	0	0	0	0	0	0	1	Unique	COG3393_R#	41.9	164	3.73E-40				
6084	WP_019524210.1	cbiQ	cobalt ECF transporter T component CbiQ	1	0	1	1	1	1	1	1	1	1	1	10	Accessory	COG0619_P#	32.9	107	9.22E-23	K02008	27.5	80	3.22E-14
6085	PWE09809.1	-	hypothetical protein	0	1	1	1	1	1	1	1	1	1	1	10	Accessory								
6086	AXI89610.1	-	phosphotransferase	0	0	0	1	0	0	0	0	0	0	0	1	Unique								
6087	AXI89579.1	-	hypothetical protein	0	0	0	1	0	0	0	0	0	0	0	1	Unique								
6088	AXI88647.1	-	phage tail protein	1	1	1	1	1	1	1	1	1	1	1	11	CORE								

cluster	GI	gene	Annotation	Streptomyces_thermocarboxydus_BPSAC147	Streptomyces_sp._4F	Streptomyces_sp._BSE7F	Streptomyces_sp._ETH9427	Streptomyces_sp._FxanaD5	Streptomyces_sp._UNC401CLCol	Actinospica_acidiphila_NRRL_B_24431	Streptomyces_sp._HNS054	Streptomyces_sp._SMS_SU21	Streptomyces_sp._ZS0098	Streptomyces_thermocarboxydus_K155	Addition	Core-Accessory-Unique	COG	%Similarity_COG	Score_COG	Evalue_COG	KEGG	%Similarity_KEGG	Score_KEGG	Evalue_KEGG
6089	AXI91038.1	-	branched-chain amino acid ABC transporter permease	1	1	1	1	1	1	1	1	1	1	1	11	CORE	COG1296_E#	34.9	116	1.52E-25	NA	34.9	116	4.06E-25
6090	OSC71386.1	-	ABC transporter	0	1	0	0	0	0	0	0	0	0	0	1	Unique	COG0842_V#	25.8	97	9.54E-20	K01992	23.4	48	0.000104
6091	PWE09900.1	-	hypothetical protein	0	0	1	1	1	1	0	1	1	1	0	7	Accessory								
6092	Prokka:CIODPCDJ_06811	-	hypothetical protein	0	0	0	0	0	0	0	0	0	0	1	1	Unique								
6093	OSC65882.1	-	Na(+)/H(+) antiporter subunit C	0	1	0	0	0	0	0	0	0	0	0	1	Unique	COG1006_P#	44.7	110	8.34E-24	K05567	38.5	77	2.73E-13
6094	OSC69143.1	-	alpha/beta hydrolase	1	1	1	1	1	1	1	1	1	1	1	11	CORE								
6095	OSC71515.1	-	hypothetical protein	0	1	0	0	0	0	0	0	0	0	0	1	Unique								
6096	WP_159020915.1	-	hypothetical protein	0	0	0	0	0	0	0	1	0	0	0	1	Unique	COG0265_O#	26.3	64	8.95E-10	NA	26.3	64	2.39E-09
6097	OSC68613.1	-	cupin	0	1	0	1	0	0	0	1	1	0	1	5	Accessory								
6098	OSC64127.1	-	3-oxoacyl-ACP reductase	1	1	1	1	1	1	1	1	1	1	1	11	CORE	COG1028_IQR#	45.8	223	6.76E-58	NA	45.8	223	1.8E-57
6099	OSC61142.1	-	GntR family transcriptional regulator	0	1	0	0	0	0	0	0	0	0	0	1	Unique	COG2186_K#	31.8	118	3.06E-26	K05799	29	114	1.54E-24

cluster	GI	gene	Annotation	Streptomyces_thermocarboxydus_BPSAC147	Streptomyces_sp._4F	Streptomyces_sp._BSE7F	Streptomyces_sp._ETH9427	Streptomyces_sp._FxanaD5	Streptomyces_sp._UNC401CLCol	Actinospica_acidiphila_NRRL_B_24431	Streptomyces_sp._HNS054	Streptomyces_sp._SMS_SU21	Streptomyces_sp._ZS0098	Streptomyces_thermocarboxydus_K155	Addition	Core-Accessory-Unique	COG	%Similarity_COG	Score_COG	Evalue_COG	KEGG	%Similarity_KEGG	Score_KEGG	Evalue_KEGG
6100	Prokka:JAPCLKMA_01592	dsbD_2	Thiol:disulfide interchange protein DsbD	1	0	0	0	0	0	0	0	0	0	0	1	Unique	COG0785_O#	41	185	2.66E-46	K06196	39.8	178	8.69E-44
6101	OSC70188.1	-	3-ketoacyl-ACP reductase	0	1	0	0	0	0	0	0	0	0	0	1	Unique	COG1028_IQR#	43.5	197	5.19E-50	NA	40.4	165	5.83E-40
6102	Prokka:CIODPCDJ_00001	-	hypothetical protein	0	0	0	0	0	0	0	0	0	0	1	1	Unique								
6103	OSC67739.1	-	MerR family transcriptional regulator	1	1	1	1	1	1	1	1	1	1	1	11	CORE	COG0789_K#	33	89	1.99E-17	K11923	27.3	62	6.95E-09
6104	PWE07458.1	-	MBL fold metallo-hydrolase	1	1	1	0	1	1	1	1	1	1	1	10	Accessory	COG0491_R#	42.2	158	3.49E-38	NA	42.2	158	9.31E-38
6105	AXI88999.1	-	UMP kinase	1	1	1	1	1	1	1	1	1	1	1	11	CORE	COG0528_F#	75.4	335	1.22E-91	K09903	72.7	327	1.15E-88
6106	WP_102641206.1	-	TIGR00730 family Rossman fold protein	1	1	1	1	1	1	0	1	1	1	1	10	Accessory	COG1611_R#	62.8	300	4.33E-81	K06966	51.5	196	4.01E-49
6107	WP_122212987.1	-	GntR family transcriptional regulator	1	0	1	0	1	1	1	1	0	1	1	8	Accessory	COG2188_K#	29.7	67	6.17E-11	NA	49.2	56	4.96E-07
6108	WP_033272964.1	-	endonuclease	1	1	1	1	1	1	1	1	1	1	1	11	CORE								
6109	Prokka:JAPCLKMA_00373	fabG_2	3-oxoacyl-[acyl-carrier-protein] reductase FabG	1	1	1	1	1	1	1	1	1	1	1	11	CORE	COG1028_IQR#	43.7	198	3.95E-50	K00059	36.5	143	2.36E-33

cluster	GI	gene	Annotation	Streptomyces_thermocarboxydus_BPSAC147	Streptomyces_sp._4F	Streptomyces_sp._BSE7F	Streptomyces_sp._ETH9427	Streptomyces_sp._FxanaD5	Streptomyces_sp._UNC401CLCol	Actinospica_acidiphila_NRRL_B_24431	Streptomyces_sp._HNS054	Streptomyces_sp._SMS_SU21	Streptomyces_sp._ZS0098	Streptomyces_thermocarboxydus_K155	Addition	Core-Accessory-Unique	COG	%Similarity_COG	Score_COG	Evalue_COG	KEGG	%Similarity_KEGG	Score_KEGG	Evalue_KEGG
6110	WP_048456751.1	-	hypothetical protein	1	1	0	1	1	1	1	1	1	1	1	10	Accessory					NA	41.6	178	6.63E-44
6111	WP_048457575.1	-	class I SAM-dependent methyltransferase	0	0	1	1	0	0	1	1	1	1	0	6	Accessory	COG0500_QR#	32.7	69	1.62E-11	NA	32.7	69	4.34E-11
6112	Prokka:JAPCLKMA_01183	-	hypothetical protein	1	1	1	1	1	1	1	1	1	1	1	11	CORE	COG1309_K#	31.8	90	1.16E-17	NA	32.1	97	1.94E-19
6113	OSC69121.1	-	short-chain dehydrogenase	0	1	0	0	0	0	0	0	0	0	0	1	Unique	COG1028_IQR#	41.3	187	6.98E-47	K00059	37.7	156	4.6E-37
6114	OSC63053.1	-	TetR family transcriptional regulator	0	1	0	0	0	0	0	0	0	0	0	1	Unique	COG1309_K#	27.8	64	8.91E-10				
6115	AXI88234.1	-	hypothetical protein	1	1	1	1	1	1	1	1	1	1	1	11	CORE	COG3361_S#	49.2	217	4.82E-56	K09166	49.2	217	1.29E-55
6116	WP_078894923.1	-	SDR family oxidoreductase	0	1	1	1	1	0	1	1	0	0	0	6	Accessory	COG1028_IQR#	48.6	231	4.22E-60	K00059	39.9	147	1.64E-34
6117	WP_033275160.1	-	LamB/YcsF family protein	1	1	1	1	1	1	1	1	1	1	1	11	CORE	COG1540_R#	54.8	282	1.22E-75	K07160	42.1	208	1.02E-52
6118	WP_053076978.1	-	ATP-binding protein	0	0	0	0	0	0	0	1	0	0	0	1	Unique								
6119	AXI85365.1	-	hypothetical protein	0	0	0	1	0	0	0	0	0	0	0	1	Unique								
6120	AXI91258.1	-	hypothetical protein	0	0	0	1	0	0	0	0	0	0	0	1	Unique								
6121	AXI85356.1	-	hypothetical protein	1	0	0	1	0	0	0	0	0	0	0	2	Accessory								

cluster	GI	gene	Annotation	Streptomyces_thermocarboxydus_BPSAC147	Streptomyces_sp._4F	Streptomyces_sp._BSE7F	Streptomyces_sp._ETH9427	Streptomyces_sp._FxanaD5	Streptomyces_sp._UNC401CLCol	Actinospica_acidiphila_NRRL_B_24431	Streptomyces_sp._HNS054	Streptomyces_sp._SMS_SU21	Streptomyces_sp._ZS0098	Streptomyces_thermocarboxydus_K155	Addition	Core-Accessory-Unique	COG	%Similarity_COG	Score_COG	Evalue_COG	KEGG	%Similarity_KEGG	Score_KEGG	Evalue_KEGG
6122	Prokka:JAPCLKMA_03955	rppH_5	RNA pyrophosphohydrolase	1	1	1	1	1	1	1	1	1	1	1	11	CORE	COG1051_F#	41.9	148	4.69E-35	NA	41.9	148	1.25E-34
6123	Prokka:CIODPCDJ_06778	-	hypothetical protein	0	0	0	0	0	0	0	0	0	0	1	1	Unique								
6124	WP_033272075.1	-	GntR family transcriptional regulator	0	0	1	0	0	1	1	0	0	0	0	3	Accessory	COG2188_K#	28.8	75	3.87E-13	K00375	31.1	47	0.000231
6125	OSC53976.1	-	LuxR family transcriptional regulator	0	1	1	1	0	0	1	1	1	1	0	7	Accessory	COG2771_K#	37.1	40	0.00808				
6126	OSC63245.1	-	hypothetical protein	1	1	1	1	1	1	1	1	1	1	1	11	CORE								
6127	WP_037954407.1	-	TnsA-like heteromeric transposase endonuclease subunit	0	0	0	0	0	1	0	0	0	0	0	1	Unique								
6128	OSC62973.1	-	DNA-binding response regulator	1	1	1	1	1	1	1	1	1	1	1	11	CORE	COG2197_TK#	45.8	180	6.53E-45	NA	43.2	180	2.28E-44
6129	OSC66760.1	-	hypothetical protein	1	1	1	1	1	1	1	1	1	1	1	11	CORE								
6130	WP_102641125.1	-	ABC transporter permease	0	0	1	1	1	1	1	1	1	1	1	9	Accessory	COG0842_V#	44.7	194	5.69E-49	K01992	44.7	194	1.52E-48
6131	OSC62858.1	-	hypothetical protein	0	1	1	1	0	0	1	1	1	1	0	7	Accessory	COG1891_S#	58.2	261	2.9E-69	K09733	57.9	256	3.26E-67

cluster	GI	gene	Annotation	Streptomyces_thermocarboxydus_BPSAC147	Streptomyces_sp._4F	Streptomyces_sp._BSE7F	Streptomyces_sp._ETH9427	Streptomyces_sp._FxanaD5	Streptomyces_sp._UNC401CLCol	Actinospica_acidiphila_NRRL_B_24431	Streptomyces_sp._HNS054	Streptomyces_sp._SMS_SU21	Streptomyces_sp._ZS0098	Streptomyces_thermocarboxydus_K155	Addition	Core-Accessory-Unique	COG	%Similarity_COG	Score_COG	Evalue_COG	KEGG	%Similarity_KEGG	Score_KEGG	Evalue_KEGG
6132	Prokka:JAPCLKMA_00951	kipR	HTH-type transcriptional regulator KipR	1	0	0	0	1	1	0	0	0	0	1	4	Accessory	COG1414_K#	29.8	98	3.25E-20	K19333	26.4	86	5.83E-16
6133	WP_019521345.1	egtC	ergothioneine biosynthesis protein EgtC	1	0	1	1	1	1	1	1	1	1	1	10	Accessory	COG0121_R#	44.3	195	3.33E-49	K07008	44.3	195	8.91E-49
6134	WP_122214687.1	-	SIS domain-containing protein	1	0	1	1	1	1	1	1	1	1	1	10	Accessory	COG4821_R#	37.1	152	1.9E-36				
6135	WP_102637329.1	-	AIM24 family protein	1	1	1	1	1	1	1	1	1	1	1	11	CORE	COG2013_S#	36	105	3.48E-22				
6136	Prokka:CIODPCDJ_06497	hmp_4	Flavohemoprotein	1	0	1	1	1	1	1	1	1	1	1	10	Accessory	COG1018_C#	27.3	73	1.46E-12				
6137	WP_019521305.1	-	(Fe-S)-binding protein	1	1	1	1	1	1	1	1	1	1	1	11	CORE	COG0247_C#	63	303	6.66E-82	K18928	63	303	1.78E-81
6138	WP_033272354.1	-	decaprenylphospho-beta-D-erythro-pentofuranosid- 2-ulose 2-reductase	1	1	1	0	1	1	1	1	1	1	1	10	Accessory	COG1028_IQR#	46.6	232	1.88E-60	K16652	47.8	227	1.62E-58
6139	WP_028959481.1	-	hypothetical protein	1	1	1	1	1	1	1	1	1	1	1	11	CORE								
6140	WP_122212990.1	-	oxidoreductase	1	1	1	1	1	1	1	1	1	1	1	11	CORE	COG1018_C#	31.4	86	1.28E-16	K15983	28	81	1.44E-14

cluster	GI	gene	Annotation	Streptomyces_thermocarboxydus_BPSAC147	Streptomyces_sp._4F	Streptomyces_sp._BSE7F	Streptomyces_sp._ETH9427	Streptomyces_sp._FxanaD5	Streptomyces_sp._UNC401CLCol	Actinospica_acidiphila_NRRL_B_24431	Streptomyces_sp._HNS054	Streptomyces_sp._SMS_SU21	Streptomyces_sp._ZS0098	Streptomyces_thermocarboxydus_K155	Addition	Core-Accessory-Unique	COG	%Similarity_COG	Score_COG	Evalue_COG	KEGG	%Similarity_KEGG	Score_KEGG	Evalue_KEGG
6141	AXI86245.1	-	imidazole glycerol phosphate synthase subunit HisF	1	1	1	1	1	1	1	1	1	1	1	11	CORE	COG0107_E#	73.7	357	3.88E-98	K02500	73.7	357	1.04E-97
6142	WP_048456602.1	-	MBL fold metallo-hydrolase	1	1	1	1	1	1	1	1	1	1	1	11	CORE	COG0491_R#	43.4	216	1.07E-55	NA	43.4	216	2.86E-55
6143	Prokka:JAPCLKMA_04946	-	hypothetical protein	1	1	0	0	1	1	0	0	0	0	1	5	Accessory								
6144	Prokka:JAPCLKMA_01537	rsrIM	Modification methylase RsrI	1	0	0	1	0	0	0	0	0	0	0	2	Accessory	COG0863_L#	43.7	203	1.22E-51	K00571	41.5	49	0.0000463
6145	WP_019523975.1	-	hypothetical protein	1	0	0	0	1	1	0	0	0	0	1	4	Accessory								
6146	WP_109288625.1	-	GntR family transcriptional regulator	0	1	0	0	0	1	0	1	1	0	0	4	Accessory	COG2188_K#	27.1	86	1.67E-16	K03710	27.1	86	4.46E-16
6147	Prokka:JAPCLKMA_04754	serB	Phosphoserine phosphatase	1	0	0	0	0	0	0	0	0	0	0	1	Unique	COG0561_R#	24.5	41	0.00616				
6148	WP_033274626.1	-	hypothetical protein	0	0	0	0	0	0	1	0	0	0	0	1	Unique								
6149	OSC71259.1	-	NADH-quinone oxidoreductase subunit C	1	1	1	1	1	1	1	1	1	1	1	11	CORE	COG0852_C#	69.1	343	7.58E-94	K00332	69.1	343	2.02E-93

cluster	GI	gene	Annotation	Streptomyces_thermocarboxydus_BPSAC147	Streptomyces_sp._4F	Streptomyces_sp._BSE7F	Streptomyces_sp._ETH9427	Streptomyces_sp._FxanaD5	Streptomyces_sp._UNC401CLCol	Actinospica_acidiphila_NRRL_B_24431	Streptomyces_sp._HNS054	Streptomyces_sp._SMS_SU21	Streptomyces_sp._ZS0098	Streptomyces_thermocarboxydus_K155	Addition	Core-Accessory-Unique	COG	%Similarity_COG	Score_COG	Evalue_COG	KEGG	%Similarity_KEGG	Score_KEGG	Evalue_KEGG
6150	OSC70364.1	-	peptidase propeptide and YpeB domain protein	0	1	0	0	0	0	0	0	0	0	0	1	Unique					NA	46.7	49	0.0000789
6151	WP_019527578.1	-	phage Gp37/Gp68 family protein	1	0	0	0	1	0	0	0	0	0	1	3	Accessory	COG4422_S#	73.7	393	4.89E-109	NA	73.7	393	1.31E-108
6152	OSC66109.1	-	oxidoreductase	0	1	0	0	0	0	0	0	0	0	0	1	Unique	COG1028_IQR#	39	156	1.72E-37	K00059	39	156	4.58E-37
6153	AXI89865.1	-	nucleotide sugar-1-phosphate transferase	1	1	1	1	1	1	1	1	1	1	1	11	CORE	COG1208_MJ#	26.8	71	4.24E-12	K00966	30.2	54	0.00000187
6154	WP_048459153.1	-	nucleotidyltransferase	0	0	0	1	0	0	0	1	0	0	0	2	Accessory	COG3541_R#	45.6	127	6.51E-29				
6155	WP_048460743.1	-	4-hydroxy-tetrahydrodipicolinate reductase	1	1	1	1	1	1	1	1	1	1	1	11	CORE	COG0289_E#	62.8	310	7.08E-84	K00215	62.8	310	1.89E-83
6156	WP_019523423.1	-	class I SAM-dependent methyltransferase	1	0	1	0	1	1	1	1	1	1	1	9	Accessory	COG0500_QR#	37.5	47	0.000112	NA	37.5	47	0.000299
6157	WP_019522320.1	-	GntR family transcriptional regulator	1	1	1	1	1	1	1	1	1	1	1	11	CORE	COG2188_K#	31.4	87	5.72E-17	K03710	31.4	87	1.53E-16
6158	WP_102638437.1	-	IclR family transcriptional regulator	1	0	1	1	1	1	1	1	1	1	1	10	Accessory	COG1414_K#	25.5	93	1.04E-18	NA	25.9	72	5.08E-12

cluster	GI	gene	Annotation	Streptomyces_thermocarboxydus_BPSAC147	Streptomyces_sp._4F	Streptomyces_sp._BSE7F	Streptomyces_sp._ETH9427	Streptomyces_sp._FxanaD5	Streptomyces_sp._UNC401CLCol	Actinospica_acidiphila_NRRL_B_24431	Streptomyces_sp._HNS054	Streptomyces_sp._SMS_SU21	Streptomyces_sp._ZS0098	Streptomyces_thermocarboxydus_K155	Addition	Core-Accessory-Unique	COG	%Similarity_COG	Score_COG	Evalue_COG	KEGG	%Similarity_KEGG	Score_KEGG	Evalue_KEGG
6159	Prokka:CIODPCDJ_03442	mprA_3	Response regulator MprA	1	1	0	0	1	1	0	0	0	0	1	5	Accessory	COG0745_TK#	51.5	222	1.49E-57	K07672	50.2	216	3.72E-55
6160	WP_019525087.1	-	MBL fold metallo-hydrolase	1	1	1	1	1	1	1	1	1	1	1	11	CORE	COG1234_R#	44.5	204	5.47E-52	NA	43.4	199	3.6E-50
6161	Prokka:JAPCLKMA_06746	-	putative membrane transporter protein	1	1	1	1	1	1	1	1	1	1	1	11	CORE	COG0730_R#	42.7	168	2.55E-41	K07090	37.2	136	4.89E-31
6162	AXI84818.1	-	TetR family transcriptional regulator	0	1	1	1	0	1	1	1	1	1	1	9	Accessory	COG1309_K#	43.7	63	1.15E-09	NA	43.7	63	3.08E-09
6163	PWE08644.1	-	ABC transporter permease	0	0	1	1	0	0	1	1	1	1	0	6	Accessory								
6164	AXI84793.1	-	TIGR03943 family protein	0	1	1	1	0	0	1	1	1	1	0	7	Accessory								
6165	PWE09770.1	-	DUF3710 domain-containing protein	1	1	1	1	1	1	1	1	1	1	1	11	CORE					NA	30.3	89	6.85E-17
6166	WP_122217251.1	-	hypothetical protein	1	1	1	1	1	1	1	1	1	1	1	11	CORE								
6167	WP_033276046.1	-	ABC transporter permease	1	1	1	1	1	1	1	0	1	1	1	10	Accessory	COG0842_V#	26.8	69	2.1E-11	K01992	26.9	59	7.6E-08
6168	Prokka:JAPCLKMA_05617	-	putative transcriptional regulatory protein	1	1	1	1	1	1	1	1	1	1	1	11	CORE	COG0217_S#	79.5	404	2.76E-112	NA	79.5	404	7.37E-112

cluster	GI	gene	Annotation	Streptomyces_thermocarboxydus_BPSAC147	Streptomyces_sp._4F	Streptomyces_sp._BSE7F	Streptomyces_sp._ETH9427	Streptomyces_sp._FxanaD5	Streptomyces_sp._UNC401CLCol	Actinospica_acidiphila_NRRL_B_24431	Streptomyces_sp._HNS054	Streptomyces_sp._SMS_SU21	Streptomyces_sp._ZS0098	Streptomyces_thermocarboxydus_K155	Addition	Core-Accessory-Unique	COG	%Similarity_COG	Score_COG	Evalue_COG	KEGG	%Similarity_KEGG	Score_KEGG	Evalue_KEGG
6169	WP_033273794.1	-	FadR family transcriptional regulator	0	1	0	0	0	0	1	0	0	0	0	2	Accessory	COG2186_K#	41	169	1.95E-41	NA	28.2	87	2.6E-16
6170	WP_048460847.1	-	arylmalonate decarboxylase	0	0	0	1	0	0	0	1	0	0	0	2	Accessory								
6171	OSC61925.1	-	short-chain dehydrogenase	0	1	0	0	0	0	0	0	0	0	0	1	Unique	COG1028_IQR#	37.7	158	2.64E-38	K00540	35.6	137	2.19E-31
6172	OSC64055.1	-	TetR family transcriptional regulator	0	1	0	0	0	0	0	0	0	0	0	1	Unique	COG1309_K#	26	67	1.04E-10	NA	26	67	2.79E-10
6173	OSC65917.1	-	methyltransferase	0	1	0	0	0	0	0	0	0	0	0	1	Unique								
6174	WP_019526043.1	-	helix-turn-helix transcriptional regulator	0	0	0	0	1	0	0	0	0	0	0	1	Unique								
6175	OSC64497.1	-	hypothetical protein	0	1	0	0	0	0	0	0	0	0	0	1	Unique	COG3321_Q#	70.7	276	8.68E-74	K12435	70.7	276	2.32E-73
6176	OSC71862.1	-	3-oxoacyl-ACP reductase	0	1	0	0	0	0	0	0	0	0	0	1	Unique	COG1028_IQR#	39.5	158	3.45E-38	K00038	39.5	158	9.2E-38
6177	WP_033273996.1	-	response regulator transcription factor	1	1	1	1	1	1	1	1	1	1	1	11	CORE	COG0745_TK#	44.5	209	1.7E-53	K07658	44.5	200	2.76E-50
6178	OSC61735.1	-	short-chain dehydrogenase	0	1	0	0	0	0	0	0	0	0	0	1	Unique	COG1028_IQR#	51.2	233	6.45E-61	K00059	39.8	161	1.42E-38

cluster	GI	gene	Annotation	Streptomyces_thermocarboxydus_BPSAC147	Streptomyces_sp._4F	Streptomyces_sp._BSE7F	Streptomyces_sp._ETH9427	Streptomyces_sp._FxanaD5	Streptomyces_sp._UNC401CLCol	Actinospica_acidiphila_NRRL_B_24431	Streptomyces_sp._HNS054	Streptomyces_sp._SMS_SU21	Streptomyces_sp._ZS0098	Streptomyces_thermocarboxydus_K155	Addition	Core-Accessory-Unique	COG	%Similarity_COG	Score_COG	Evalue_COG	KEGG	%Similarity_KEGG	Score_KEGG	Evalue_KEGG
6179	WP_122215932.1	-	helix-turn-helix transcriptional regulator	0	0	0	0	0	0	0	0	0	1	0	1	Unique								
6180	WP_033277859.1	-	TetR/AcrR family transcriptional regulator	1	0	0	0	1	1	1	0	0	0	1	5	Accessory	COG1309_K#	35.5	57	6.34E-08	NA	35.5	57	1.69E-07
6181	WP_019523811.1	-	hypothetical protein	1	0	0	0	1	1	0	0	0	0	1	4	Accessory								
6182	Prokka:CIODPCDJ_03166	irtA	Iron import ATP-binding/permease protein IrtA	1	1	1	1	1	0	1	1	1	1	1	10	Accessory								
6183	WP_122213487.1	-	hypothetical protein	1	1	1	1	1	1	1	1	1	1	1	11	CORE								
6184	WP_048459311.1	-	ATP-binding cassette domain-containing protein	1	1	1	1	1	1	1	1	1	1	1	11	CORE	COG4181_Q#	42.4	149	2.08E-35	K09810	43.4	148	7.26E-35
6185	AXI89509.1	-	anti-sigma factor	0	0	0	1	0	0	0	1	1	1	0	4	Accessory					NA	28.7	78	1.21E-13
6186	WP_102636953.1	-	DUF4142 domain-containing protein	1	1	1	1	1	1	1	1	1	1	1	11	CORE								
6187	WP_048455043.1	-	class I SAM-dependent methyltransferase	1	0	0	1	1	1	1	1	0	1	1	8	Accessory	COG0500_QR#	34.3	116	1.5E-25	NA	25.2	75	7.81E-13

cluster	GI	gene	Annotation	Streptomyces_thermocarboxydus_BPSAC147	Streptomyces_sp._4F	Streptomyces_sp._BSE7F	Streptomyces_sp._ETH9427	Streptomyces_sp._FxanaD5	Streptomyces_sp._UNC401CLCol	Actinospica_acidiphila_NRRL_B_24431	Streptomyces_sp._HNS054	Streptomyces_sp._SMS_SU21	Streptomyces_sp._ZS0098	Streptomyces_thermocarboxydus_K155	Addition	Core-Accessory-Unique	COG	%Similarity_COG	Score_COG	Evalue_COG	KEGG	%Similarity_KEGG	Score_KEGG	Evalue_KEGG
6188	WP_033276751.1	-	GntR family transcriptional regulator	1	1	1	1	1	1	1	1	1	1	1	11	CORE	COG2188_K#	28.3	89	2.56E-17	K03710	26.7	84	1.68E-15
6189	WP_048455632.1	-	IS5/IS1182 family transposase	0	0	0	0	0	0	1	1	0	0	0	2	Accessory								
6190	Prokka:JAPCLKMA_04938	-	hypothetical protein	1	0	0	0	0	0	0	0	0	0	0	1	Unique								
6191	WP_122213135.1	-	metallophosphoesterase	1	1	1	1	1	1	1	1	1	1	1	11	CORE	COG2129_R#	44.2	182	2.22E-45	NA	29.3	75	7.81E-13
6192	Prokka:JAPCLKMA_06085	frdB_2	Fumarate reductase iron-sulfur subunit	1	1	0	1	1	1	1	1	1	1	1	10	Accessory	COG0479_C#	52.6	266	1.17E-70	K00240	56.9	298	5.67E-80
6193	OSC57337.1	-	DNA-binding response regulator	1	1	1	1	1	1	1	1	1	1	1	11	CORE	COG0745_TK#	40.9	173	1.03E-42	K07669	42.4	166	4.39E-40
6194	WP_048458364.1	-	SDR family oxidoreductase	1	0	1	1	1	1	1	1	0	1	1	9	Accessory	COG0702_MG#	55.7	280	4.58E-75				
6195	PWE11011.1	-	hypothetical protein	0	0	1	0	0	0	0	0	1	0	0	2	Accessory								
6196	WP_033275792.1	recO	DNA repair protein RecO	1	1	1	1	1	1	1	1	1	1	1	11	CORE	COG1381_L#	51.6	253	1.02E-66	K03584	51.6	253	2.73E-66
6197	OSC61535.1	-	short-chain dehydrogenase	0	1	0	1	0	0	1	1	0	1	0	5	Accessory	COG1028_IQR#	55.6	254	3.52E-67	NA	50.4	235	5.9E-61
6198	OSC71675.1	-	hypothetical protein	0	1	0	0	0	0	0	0	0	0	0	1	Unique	COG0463_M#	27.4	53	0.00000155				

cluster	GI	gene	Annotation	Streptomyces_thermocarboxydus_BPSAC147	Streptomyces_sp._4F	Streptomyces_sp._BSE7F	Streptomyces_sp._ETH9427	Streptomyces_sp._FxanaD5	Streptomyces_sp._UNC401CLCol	Actinospica_acidiphila_NRRL_B_24431	Streptomyces_sp._HNS054	Streptomyces_sp._SMS_SU21	Streptomyces_sp._ZS0098	Streptomyces_thermocarboxydus_K155	Addition	Core-Accessory-Unique	COG	%Similarity_COG	Score_COG	Evalue_COG	KEGG	%Similarity_KEGG	Score_KEGG	Evalue_KEGG
6199	Prokka:JAPCLKMA_00261	-	hypothetical protein	1	1	1	1	1	1	1	1	1	1	1	11	CORE	COG2345_K#	43.4	178	3.2E-44	NA	43.4	178	8.56E-44
6200	OSC66604.1	-	multidrug ABC transporter permease	0	1	0	0	0	0	0	0	0	0	0	1	Unique	COG0842_V#	30.6	112	2.16E-24	K01992	26.9	69	7.31E-11
6201	OSC71426.1	-	nucleopolyhedrovirus P10 family protein	0	1	0	0	0	0	0	0	0	0	0	1	Unique								
6202	WP_102640544.1	-	hypothetical protein	0	0	1	1	0	0	0	0	1	0	0	3	Accessory								
6203	OSC71415.1	-	hypothetical protein	0	1	0	0	0	0	0	0	0	0	0	1	Unique	COG4912_L#	30.3	114	5.68E-25				
6204	WP_102637768.1	-	SDR family oxidoreductase	1	1	1	1	1	1	1	1	1	1	1	11	CORE	COG1028_IQR#	44.9	187	6.9E-47	NA	44.9	187	1.84E-46
6205	Prokka:JAPCLKMA_05684	-	hypothetical protein	1	0	0	0	0	0	0	0	0	0	0	1	Unique								
6206	Prokka:CIODPCDJ_06037	-	hypothetical protein	0	0	0	0	0	0	0	0	0	0	1	1	Unique								
6207	WP_033272284.1	-	PH domain-containing protein	1	1	1	1	1	1	1	1	1	1	1	11	CORE					NA	38.5	49	0.0000459
6208	WP_033272697.1	-	FHA domain-containing protein	0	1	0	0	0	0	1	0	0	0	0	2	Accessory								
6209	OSC61494.1	-	alpha/beta hydrolase	0	1	0	0	0	0	0	0	0	0	0	1	Unique	COG1073_R#	34.8	97	9.39E-20	K06889	32.1	109	3.74E-23

cluster	GI	gene	Annotation	Streptomyces_thermocarboxydus_BPSAC147	Streptomyces_sp._4F	Streptomyces_sp._BSE7F	Streptomyces_sp._ETH9427	Streptomyces_sp._FxanaD5	Streptomyces_sp._UNC401CLCol	Actinospica_acidiphila_NRRL_B_24431	Streptomyces_sp._HNS054	Streptomyces_sp._SMS_SU21	Streptomyces_sp._ZS0098	Streptomyces_thermocarboxydus_K155	Addition	Core-Accessory-Unique	COG	%Similarity_COG	Score_COG	Evalue_COG	KEGG	%Similarity_KEGG	Score_KEGG	Evalue_KEGG
6210	OSC61321.1	-	16S rRNA (uracil(1498)-N(3))-methyltransferase	1	1	1	1	1	1	1	1	1	1	1	11	CORE	COG1385_S#	45.2	214	5.27E-55	K09761	43.9	188	6.33E-47
6211	WP_053076976.1	-	HNH endonuclease	0	0	0	0	0	0	0	1	0	0	0	1	Unique					NA	29.6	86	3.39E-16
6212	Prokka:JAPCLKMA_01611	-	hypothetical protein	1	0	0	0	0	0	0	0	0	0	0	1	Unique					NA	40.8	42	0.00561
6213	OSC59937.1	-	hypothetical protein	0	1	0	0	0	0	0	0	0	0	0	1	Unique								
6214	WP_159020782.1	-	hypothetical protein	0	0	0	1	0	0	0	1	0	0	0	2	Accessory								
6215	AXI89641.1	-	hypothetical protein	0	0	0	1	0	0	0	0	0	0	0	1	Unique								
6216	WP_122212785.1	-	zinc ribbon domain-containing protein	0	0	0	0	0	0	0	1	1	1	0	3	Accessory								
6217	PWE09146.1	-	toxic cation resistance protein	0	1	1	1	0	0	1	1	0	1	0	6	Accessory					NA	34.5	126	2.94E-28
6218	WP_019521741.1	-	glycerophosphoryl diester phosphodiesterase	1	0	1	1	1	1	1	1	1	1	1	10	Accessory	COG0584_C#	27.8	64	6.72E-10	K01126	25.6	64	1.79E-09
6219	WP_048460348.1	-	thioesterase	0	0	1	1	0	0	0	1	1	1	0	5	Accessory	COG3208_Q#	35.3	121	6.05E-27	NA	30.8	106	3.15E-22
6220	WP_107685352.1	-	GAF and ANTAR domain-containing protein	1	1	1	1	1	1	1	1	1	1	1	11	CORE								

cluster	GI	gene	Annotation	Streptomyces_thermocarboxydus_BPSAC147	Streptomyces_sp._4F	Streptomyces_sp._BSE7F	Streptomyces_sp._ETH9427	Streptomyces_sp._FxanaD5	Streptomyces_sp._UNC401CLCol	Actinospica_acidiphila_NRRL_B_24431	Streptomyces_sp._HNS054	Streptomyces_sp._SMS_SU21	Streptomyces_sp._ZS0098	Streptomyces_thermocarboxydus_K155	Addition	Core-Accessory-Unique	COG	%Similarity_COG	Score_COG	Evalue_COG	KEGG	%Similarity_KEGG	Score_KEGG	Evalue_KEGG
6221	WP_102641753.1	-	alpha/beta hydrolase	1	1	1	1	1	1	1	1	1	1	1	11	CORE								
6222	PWE07941.1	-	hypothetical protein	1	1	1	1	1	1	1	1	1	1	1	11	CORE								
6223	OSC56495.1	-	DNA-binding response regulator	1	1	1	1	1	1	1	1	1	1	1	11	CORE	COG2197_TK#	46	176	1.58E-43	NA	44.2	172	6.11E-42
6224	WP_048456503.1	-	SDR family oxidoreductase	1	0	1	1	1	1	1	1	1	1	1	10	Accessory	COG1028_IQR#	37	150	9.31E-36	NA	33.7	102	7.76E-21
6225	WP_033274624.1	-	aminoglycoside phosphotransferase family protein	0	1	0	0	0	0	1	0	0	0	0	2	Accessory								
6226	Prokka:CIODPCDJ_06008	fabG_10	3-oxoacyl-[acyl-carrier-protein] reductase FabG	0	0	0	0	1	1	1	1	0	1	1	6	Accessory	COG1028_IQR#	49.4	215	1.8E-55	K00059	39.4	129	4.54E-29
6227	PWE12203.1	pflA	pyruvate formate lyase-activating protein	0	0	1	1	0	0	0	1	1	1	0	5	Accessory	COG1180_O#	45.2	227	4.59E-59	K04069	45.2	223	1.77E-57
6228	WP_033276647.1	-	class I SAM-dependent methyltransferase	0	0	0	0	0	0	1	0	0	0	1	2	Accessory	COG0500_QR#	27.2	69	2.73E-11	NA	27.2	69	7.28E-11
6229	OSC73376.1	-	hypothetical protein	0	1	0	0	0	0	0	0	0	0	0	1	Unique	COG3246_S#	41.8	162	2.37E-39	NA	39.9	143	3.97E-33

cluster	GI	gene	Annotation	Streptomyces_thermocarboxydus_BPSAC147	Streptomyces_sp._4F	Streptomyces_sp._BSE7F	Streptomyces_sp._ETH9427	Streptomyces_sp._FxanaD5	Streptomyces_sp._UNC401CLCol	Actinospica_acidiphila_NRRL_B_24431	Streptomyces_sp._HNS054	Streptomyces_sp._SMS_SU21	Streptomyces_sp._ZS0098	Streptomyces_thermocarboxydus_K155	Addition	Core-Accessory-Unique	COG	%Similarity_COG	Score_COG	Evalue_COG	KEGG	%Similarity_KEGG	Score_KEGG	Evalue_KEGG
6230	OSC64817.1	-	DNA-binding response regulator	1	1	1	1	1	1	1	1	1	1	1	11	CORE	COG2197_TK#	58.5	245	2.78E-64	K07684	37	138	1.28E-31
6231	Prokka:JAPCLKMA_04385	-	hypothetical protein	1	0	0	0	1	1	1	0	0	0	1	5	Accessory								
6232	WP_033277898.1	-	SDR family NAD(P)-dependent oxidoreductase	1	1	1	1	1	1	1	1	1	1	1	11	CORE	COG4221_R#	24.2	50	0.00001	K03331	25.2	57	2.19E-07
6233	Prokka:CIODPCDJ_06462	-	hypothetical protein	1	0	1	1	1	1	1	1	1	1	1	10	Accessory								
6234	WP_122215632.1	-	ROK family protein	1	1	1	1	1	1	1	1	1	1	1	11	CORE	COG1940_KG#	58	286	1.09E-76	K00886	60.4	285	6.47E-76
6235	WP_037778353.1	-	glycosyltransferase family 2 protein	1	0	1	0	1	1	1	1	1	1	1	9	Accessory	COG0463_M#	53	205	2.44E-52	NA	52.6	201	1.23E-50
6236	WP_033277568.1	-	DUF4129 domain-containing protein	1	1	1	0	1	1	1	1	1	1	1	10	Accessory								
6237	OSC54630.1	-	hypothetical protein	0	1	0	0	0	0	0	0	0	0	0	1	Unique								
6238	Prokka:JAPCLKMA_06566	-	hypothetical protein	1	0	0	0	0	0	0	0	1	0	0	2	Accessory								
6239	OSC62029.1	-	alpha/beta hydrolase	0	1	0	0	0	0	0	0	0	0	0	1	Unique	COG0657_I#	35.8	113	1.26E-24	NA	36.4	103	2.67E-21

cluster	GI	gene	Annotation	Streptomyces_thermocarboxydus_BPSAC147	Streptomyces_sp._4F	Streptomyces_sp._BSE7F	Streptomyces_sp._ETH9427	Streptomyces_sp._FxanaD5	Streptomyces_sp._UNC401CLCol	Actinospica_acidiphila_NRRL_B_24431	Streptomyces_sp._HNS054	Streptomyces_sp._SMS_SU21	Streptomyces_sp._ZS0098	Streptomyces_thermocarboxydus_K155	Addition	Core-Accessory-Unique	COG	%Similarity_COG	Score_COG	Evalue_COG	KEGG	%Similarity_KEGG	Score_KEGG	Evalue_KEGG
6240	OSC67018.1	-	SAM-dependent methyltransferase	0	1	0	0	0	0	0	0	0	0	0	1	Unique	COG0500_QR#	65.3	348	1.78E-95	NA	65.3	348	4.76E-95
6241	WP_033275246.1	-	response regulator transcription factor	1	1	1	1	1	1	1	1	1	1	1	11	CORE	COG0745_TK#	40.8	176	1.58E-43	K07775	40.8	176	3.24E-43
6242	OSC56427.1	-	dienelactone hydrolase	0	1	0	0	0	0	1	0	0	0	0	2	Accessory	COG0412_Q#	41	172	1.75E-42	K01061	41	172	4.68E-42
6243	Prokka:JAPCLKMA_01721	mqnB	Futalosine hydrolase	1	1	1	1	1	1	1	1	1	1	1	11	CORE	COG0775_F#	43.8	166	1.64E-40	K11783	45.2	132	4.11E-30
6244	Prokka:CIODPCDJ_06708	-	hypothetical protein	0	0	0	0	0	0	0	0	0	0	1	1	Unique								
6245	WP_102639839.1	-	HNH endonuclease	0	0	0	0	0	0	0	0	1	0	0	1	Unique	COG3183_V#	34.4	107	9.04E-23				
6246	WP_102637987.1	-	HAD-IA family hydrolase	0	0	0	0	0	0	0	0	1	0	0	1	Unique	COG0546_R#	29.1	60	7.43E-09	NA	26.8	49	0.0000597
6247	OSC72002.1	-	beta-ketoacyl-ACP reductase	0	1	0	0	0	0	0	0	0	0	0	1	Unique	COG1028_IQR#	50.2	241	4.01E-63	K00059	50.2	241	1.07E-62
6248	OSC68788.1	-	hypothetical protein	0	1	0	0	0	0	0	0	0	0	0	1	Unique								
6249	Prokka:JAPCLKMA_02135	narX	Nitrate reductase-like protein NarX	1	0	0	0	1	1	0	0	0	0	1	4	Accessory	COG2181_C#	43	200	7.81E-51	K00374	43	200	2.09E-50

cluster	GI	gene	Annotation	Streptomyces_thermocarboxydus_BPSAC147	Streptomyces_sp._4F	Streptomyces_sp._BSE7F	Streptomyces_sp._ETH9427	Streptomyces_sp._FxanaD5	Streptomyces_sp._UNC401CLCol	Actinospica_acidiphila_NRRL_B_24431	Streptomyces_sp._HNS054	Streptomyces_sp._SMS_SU21	Streptomyces_sp._ZS0098	Streptomyces_thermocarboxydus_K155	Addition	Core-Accessory-Unique	COG	%Similarity_COG	Score_COG	Evalue_COG	KEGG	%Similarity_KEGG	Score_KEGG	Evalue_KEGG
6250	AXI86267.1	-	SDR family NAD(P)-dependent oxidoreductase	1	0	1	1	1	1	1	1	1	1	1	10	Accessory	COG1028_IQR#	45	187	5.24E-47	K00059	34.3	114	1.15E-24
6251	WP_078895344.1	-	CDP-alcohol phosphatidyltransferase family protein	1	1	1	1	1	1	1	1	1	1	1	11	CORE	COG0558_I#	30.5	59	2.81E-08				
6252	PWE09260.1	-	hypothetical protein	1	1	1	1	1	1	1	1	1	1	1	11	CORE								
6253	WP_102641704.1	-	SLATT domain-containing protein	1	1	1	1	1	1	1	1	1	1	1	11	CORE								
6254	WP_033278133.1	-	acetyltransferase-like protein	0	1	0	0	0	0	1	0	0	0	0	2	Accessory								
6255	OSC60084.1	-	oxidoreductase	0	1	0	0	0	0	0	0	0	0	0	1	Unique	COG0778_C#	37.3	131	5.82E-30				
6256	OSC72815.1	-	RNA polymerase sigma factor SigM	1	1	1	1	1	1	1	1	1	1	1	11	CORE	COG1595_K#	48.9	156	1.69E-37	K03088	49.4	158	1.19E-37
6257	Prokka:JAPCLKMA_06143	mca_2	Mycothiol S-conjugate amidase	1	1	1	1	1	1	1	1	1	1	1	11	CORE	COG2120_S#	43.1	179	1.09E-44	NA	23.7	44	0.0025

cluster	GI	gene	Annotation	Streptomyces_thermocarboxydus_BPSAC147	Streptomyces_sp._4F	Streptomyces_sp._BSE7F	Streptomyces_sp._ETH9427	Streptomyces_sp._FxanaD5	Streptomyces_sp._UNC401CLCol	Actinospica_acidiphila_NRRL_B_24431	Streptomyces_sp._HNS054	Streptomyces_sp._SMS_SU21	Streptomyces_sp._ZS0098	Streptomyces_thermocarboxydus_K155	Addition	Core-Accessory-Unique	COG	%Similarity_COG	Score_COG	Evalue_COG	KEGG	%Similarity_KEGG	Score_KEGG	Evalue_KEGG
6258	OSC70669.1	-	lantibiotic ABC transporter ATP-binding protein	0	1	0	0	0	0	0	0	0	0	0	1	Unique	COG1131_V#	46.7	183	1.29E-45	K01990	46.7	183	3.45E-45
6259	AXI84916.1	-	diguanylate cyclase	1	1	1	1	1	1	1	1	1	1	1	11	CORE								
6260	Prokka:CIODPCDJ_00953	-	putative HTH-type transcriptional regulator	1	1	1	1	1	1	1	1	1	1	1	11	CORE	COG0789_K#	45.9	183	9.88E-46	NA	45.9	183	2.64E-45
6261	Prokka:JAPCLKMA_01469	-	hypothetical protein	1	0	0	0	0	0	0	0	0	0	0	1	Unique	COG0513_LKJ#	40.2	49	0.0000223	K14563	45.1	59	5.75E-08
6262	WP_102639441.1	-	glyoxalase	0	0	0	0	0	0	0	0	1	1	0	2	Accessory								
6263	WP_158688206.1	-	hypothetical protein	0	0	0	0	1	1	0	0	0	0	0	2	Accessory								
6264	WP_033275423.1	-	hypothetical protein	0	0	0	0	1	1	1	0	0	0	1	4	Accessory								
6265	OSC56172.1	-	hypothetical protein	0	1	0	0	0	0	0	0	0	0	0	1	Unique	COG1473_R#	45.7	191	2.78E-48	NA	45.7	191	7.42E-48
6266	WP_122216017.1	-	thioesterase	0	0	1	1	0	0	0	1	1	1	0	5	Accessory	COG3208_Q#	35.8	117	5.1E-26	NA	29	108	1.08E-22
6267	Prokka:CIODPCDJ_04251	cypM_1	Cypemycin N-terminal methyltransferase	0	0	0	0	0	0	0	0	0	0	1	1	Unique	COG0500_QR#	31.5	52	0.00000344	NA	27	79	7.01E-14
6268	WP_033275641.1	cobI	precorrin-2 C(20)-methyltransferase	1	1	1	1	1	1	1	1	1	1	1	11	CORE	COG2243_H#	32.6	115	2.53E-25	K03394	32.2	103	2.66E-21

cluster	GI	gene	Annotation	Streptomyces_thermocarboxydus_BPSAC147	Streptomyces_sp._4F	Streptomyces_sp._BSE7F	Streptomyces_sp._ETH9427	Streptomyces_sp._FxanaD5	Streptomyces_sp._UNC401CLCol	Actinospica_acidiphila_NRRL_B_24431	Streptomyces_sp._HNS054	Streptomyces_sp._SMS_SU21	Streptomyces_sp._ZS0098	Streptomyces_thermocarboxydus_K155	Addition	Core-Accessory-Unique	COG	%Similarity_COG	Score_COG	Evalue_COG	KEGG	%Similarity_KEGG	Score_KEGG	Evalue_KEGG
6269	WP_122214770.1	-	helix-turn-helix transcriptional regulator	1	0	0	0	0	0	0	0	0	1	1	3	Accessory								
6270	WP_048455253.1	-	class I SAM-dependent methyltransferase	1	1	1	1	1	1	1	1	1	1	1	11	CORE	COG0500_QR#	41.1	78	4.48E-14	K10770	31.9	54	0.00000241
6271	WP_078895109.1	-	HEAT repeat domain-containing protein	0	0	0	0	0	0	0	1	0	0	0	1	Unique	COG1413_C#	46.4	43	0.00122				
6272	AXI85979.1	-	nucleotidyltransferase	1	1	1	1	1	1	1	1	1	1	1	11	CORE	COG3541_R#	29.5	74	6.46E-13				
6273	WP_033275659.1	-	DsbA family oxidoreductase	1	1	1	1	1	1	1	1	1	1	1	11	CORE	COG2761_Q#	46.7	198	2.97E-50	NA	46.7	198	7.93E-50
6274	OSC64993.1	-	ABC transporter permease	0	1	0	0	0	0	0	0	0	0	0	1	Unique	COG0390_R#	33.1	101	6.45E-21	K02069	29.3	103	3.47E-21
6275	OSC69553.1	-	NADPH oxidoreductase	0	1	0	0	0	0	0	0	0	0	0	1	Unique	COG2249_R#	42.3	138	2.79E-32	NA	39.8	116	3.96E-25
6276	OSC61825.1	-	hypothetical protein	0	1	0	0	0	0	0	0	0	0	0	1	Unique	COG3889_R#	36.2	43	0.0016	NA	33.3	47	0.000295
6277	WP_106959469.1	-	DUF3043 domain-containing protein	1	1	0	1	1	1	1	1	1	1	1	10	Accessory					NA	33.7	117	1.78E-25

cluster	GI	gene	Annotation	Streptomyces_thermocarboxydus_BPSAC147	Streptomyces_sp._4F	Streptomyces_sp._BSE7F	Streptomyces_sp._ETH9427	Streptomyces_sp._FxanaD5	Streptomyces_sp._UNC401CLCol	Actinospica_acidiphila_NRRL_B_24431	Streptomyces_sp._HNS054	Streptomyces_sp._SMS_SU21	Streptomyces_sp._ZS0098	Streptomyces_thermocarboxydus_K155	Addition	Core-Accessory-Unique	COG	%Similarity_COG	Score_COG	Evalue_COG	KEGG	%Similarity_KEGG	Score_KEGG	Evalue_KEGG
6278	OSC72641.1	-	carboxyvinyl-carboxyphosphonate phosphorylmutase	0	1	0	0	0	0	0	0	0	0	0	1	Unique	COG2513_G#	39.2	136	1.39E-31	NA	36.5	116	3.03E-25
6279	WP_019524589.1	cas5e	type I-E CRISPR-associated protein Cas5/CasD	0	0	0	0	1	0	0	0	0	0	0	1	Unique					K19125	35.5	96	4.24E-19
6280	OSC72206.1	-	hypothetical protein	0	1	0	0	0	0	0	0	0	0	0	1	Unique	COG3694_R#	31.3	128	2.89E-29				
6281	OSC64624.1	-	hypothetical protein	0	1	0	0	0	0	0	0	0	0	0	1	Unique					NA	33.5	57	2.18E-07
6282	PWE08159.1	-	DUF2993 domain-containing protein	1	1	1	1	1	1	1	1	1	1	1	11	CORE								
6283	AXI87374.1	-	DUF3566 domain-containing protein	1	1	1	1	1	1	1	1	1	1	1	11	CORE					NA	31.4	109	6.3E-23
6284	WP_028959369.1	-	ABC transporter permease	1	0	0	0	1	1	1	0	0	0	1	5	Accessory	COG0842_V#	28.3	118	2.98E-26	K01992	28.3	118	7.95E-26
6285	WP_122214100.1	-	TIGR04222 domain-containing membrane protein	1	0	0	0	1	1	1	1	1	1	1	8	Accessory	COG2931_Q#	42.6	47	0.0000843	NA	65.7	57	2.84E-07
6286	AXI88729.1	-	DNA-binding response regulator	1	1	0	1	1	1	1	1	1	1	1	10	Accessory	COG0745_TK#	45.2	184	4.42E-46	K07658	43	181	1.3E-44

cluster	GI	gene	Annotation	Streptomyces_thermocarboxydus_BPSAC147	Streptomyces_sp._4F	Streptomyces_sp._BSE7F	Streptomyces_sp._ETH9427	Streptomyces_sp._FxanaD5	Streptomyces_sp._UNC401CLCol	Actinospica_acidiphila_NRRL_B_24431	Streptomyces_sp._HNS054	Streptomyces_sp._SMS_SU21	Streptomyces_sp._ZS0098	Streptomyces_thermocarboxydus_K155	Addition	Core-Accessory-Unique	COG	%Similarity_COG	Score_COG	Evalue_COG	KEGG	%Similarity_KEGG	Score_KEGG	Evalue_KEGG
6287	AXI89691.1	-	ABC transporter ATP-binding protein	1	0	1	1	1	1	1	1	1	1	1	10	Accessory	COG0410_E#	43.3	167	7.3E-41	K01996	41	163	2.81E-39
6288	WP_028960070.1	-	TetR/AcrR family transcriptional regulator	0	0	0	0	1	1	0	0	0	0	1	3	Accessory	COG1309_K#	26.1	58	4.77E-08	NA	33.3	55	0.00000108
6289	WP_019527150.1	-	FAD-dependent thymidylate synthase	1	1	1	1	1	1	1	1	1	1	1	11	CORE	COG1351_F#	40.9	136	1.38E-31	K03465	33.2	108	8.23E-23
6290	Prokka:CIODPCDJ_03392	-	hypothetical protein	0	0	0	0	0	0	0	0	0	0	1	1	Unique								
6291	WP_048455891.1	-	hypothetical protein	0	0	0	0	0	0	0	1	0	0	0	1	Unique	COG0532_J#	29.8	56	1.81E-07	NA	31.2	70	3.24E-11
6292	AXI89692.1	-	ABC transporter ATP-binding protein	1	0	1	1	1	1	1	1	1	1	1	10	Accessory	COG0411_E#	43.2	188	2.34E-47	K01995	42.4	183	2.63E-45
6293	OSC72207.1	-	hypothetical protein	0	1	0	0	0	0	0	0	0	0	0	1	Unique	COG4587_R#	31.2	97	5.44E-20	K01992	31.2	97	1.45E-19
6294	WP_102638029.1	-	class I SAM-dependent methyltransferase	1	1	1	1	1	1	1	1	1	1	1	11	CORE	COG0500_QR#	31.1	100	1.1E-20	K00559	34.3	70	3.24E-11
6295	WP_102638905.1	-	LON peptidase substrate-binding domain-containing protein	1	1	1	1	1	1	1	1	1	1	1	11	CORE	COG2802_R#	32.3	95	2.7E-19	K07157	55.2	59	5.72E-08

cluster	GI	gene	Annotation	Streptomyces_thermocarboxydus_BPSAC147	Streptomyces_sp._4F	Streptomyces_sp._BSE7F	Streptomyces_sp._ETH9427	Streptomyces_sp._FxanaD5	Streptomyces_sp._UNC401CLCol	Actinospica_acidiphila_NRRL_B_24431	Streptomyces_sp._HNS054	Streptomyces_sp._SMS_SU21	Streptomyces_sp._ZS0098	Streptomyces_thermocarboxydus_K155	Addition	Core-Accessory-Unique	COG	%Similarity_COG	Score_COG	Evalue_COG	KEGG	%Similarity_KEGG	Score_KEGG	Evalue_KEGG
6296	WP_048459953.1	-	class I SAM-dependent methyltransferase	1	1	0	1	1	1	1	1	1	1	1	10	Accessory	COG0500_QR#	27.4	82	2.36E-15	NA	29.5	81	1.41E-14
6297	OSC55796.1	-	hypothetical protein	1	1	1	1	1	1	1	1	1	1	1	11	CORE								
6298	PWE10589.1	-	AraC family transcriptional regulator	0	0	1	0	0	0	0	0	1	1	0	3	Accessory	COG2207_K#	50.6	240	8.86E-63	NA	50.2	239	3.09E-62
6299	WP_019527047.1	-	GntR family transcriptional regulator	1	0	1	0	1	0	0	1	0	0	0	4	Accessory	COG2188_K#	29	89	2.53E-17	K11922	33.3	79	4.09E-14
6300	Prokka:JAPCLKMA_01852	ygfF	putative oxidoreductase YgfF	1	1	1	1	1	1	1	1	1	1	1	11	CORE	COG1028_IQR#	49.4	235	1.67E-61	NA	46.9	217	1.26E-55
6301	Prokka:CIODPCDJ_03804	-	hypothetical protein	1	1	0	0	1	1	1	0	0	1	1	7	Accessory					NA	43.5	52	0.00000536
6302	Prokka:JAPCLKMA_01541	-	hypothetical protein	1	0	0	0	0	0	0	0	0	0	0	1	Unique								
6303	WP_063833274.1	-	TetR family transcriptional regulator	1	0	1	1	1	1	1	1	1	1	1	10	Accessory	COG1309_K#	28	54	6.89E-07	NA	28	54	0.00000184
6304	PWE11242.1	-	hypothetical protein	0	0	1	0	0	0	0	0	0	0	0	1	Unique								
6305	OSC73131.1	-	DNA-binding response regulator	1	1	1	1	1	1	1	1	1	1	1	11	CORE	COG0745_TK#	62.9	283	9.13E-76	K07669	62.6	283	2.44E-75

cluster	GI	gene	Annotation	Streptomyces_thermocarboxydus_BPSAC147	Streptomyces_sp._4F	Streptomyces_sp._BSE7F	Streptomyces_sp._ETH9427	Streptomyces_sp._FxanaD5	Streptomyces_sp._UNC401CLCol	Actinospica_acidiphila_NRRL_B_24431	Streptomyces_sp._HNS054	Streptomyces_sp._SMS_SU21	Streptomyces_sp._ZS0098	Streptomyces_thermocarboxydus_K155	Addition	Core-Accessory-Unique	COG	%Similarity_COG	Score_COG	Evalue_COG	KEGG	%Similarity_KEGG	Score_KEGG	Evalue_KEGG
6306	Prokka:CIODPCDJ_05334	cbiX_2	Sirohydrochlorin cobaltochelatase	1	1	1	1	1	1	1	1	1	1	1	11	CORE	COG2138_S#	41	149	2.06E-35	NA	41	149	5.49E-35
6307	WP_109288708.1	-	hypothetical protein	1	0	0	0	1	1	1	0	1	0	0	5	Accessory								
6308	OSC72225.1	-	NADH-quinone oxidoreductase subunit I	1	1	1	1	1	1	1	1	1	1	1	11	CORE	COG1143_C#	33.9	84	8.13E-16	K00338	33.9	84	2.17E-15
6309	Prokka:JAPCLKMA_01912	-	hypothetical protein	1	1	1	1	1	1	1	1	1	1	1	11	CORE								
6310	OSC69753.1	-	hypothetical protein	1	1	1	1	1	1	1	1	1	1	1	11	CORE	COG0637_R#	56.7	264	3.36E-70	NA	56.7	264	8.97E-70
6311	WP_102641599.1	-	rhomboid family intramembrane serine protease	1	1	1	1	1	1	1	1	1	1	1	11	CORE	COG0705_R#	35	114	4.28E-25	NA	35	114	1.14E-24
6312	WP_122214331.1	paaC	phenylacetate-CoA oxygenase subunit PaaC	1	0	1	1	1	1	1	1	1	1	1	10	Accessory	COG3396_S#	50.6	216	1.04E-55	K02611	50.6	216	2.79E-55
6313	WP_086701787.1	-	ribonuclease PH	1	1	1	1	1	1	1	1	1	1	1	11	CORE	COG0689_J#	64.9	295	1.77E-79	K00989	64.9	295	4.73E-79
6314	WP_078504634.1	cofC	2-phospho-L-lactate guanylyltransferase	0	0	0	0	1	1	0	0	0	0	1	3	Accessory								
6315	Prokka:JAPCLKMA_03800	-	hypothetical protein	1	0	0	0	0	0	0	0	0	0	0	1	Unique								

cluster	GI	gene	Annotation	Streptomyces_thermocarboxydus_BPSAC147	Streptomyces_sp._4F	Streptomyces_sp._BSE7F	Streptomyces_sp._ETH9427	Streptomyces_sp._FxanaD5	Streptomyces_sp._UNC401CLCol	Actinospica_acidiphila_NRRL_B_24431	Streptomyces_sp._HNS054	Streptomyces_sp._SMS_SU21	Streptomyces_sp._ZS0098	Streptomyces_thermocarboxydus_K155	Addition	Core-Accessory-Unique	COG	%Similarity_COG	Score_COG	Evalue_COG	KEGG	%Similarity_KEGG	Score_KEGG	Evalue_KEGG
6316	WP_048455949.1	-	SigE family RNA polymerase sigma factor	1	1	1	1	1	1	1	1	1	1	1	11	CORE	COG1595_K#	33.5	76	1.29E-13	K03088	32.3	76	3.45E-13
6317	Prokka:JAPCLKMA_03449	yidC_2	Membrane protein insertase YidC	1	1	1	1	1	1	1	1	1	1	1	11	CORE	COG0706_U#	26.3	80	8.95E-15	NA	41.7	74	2.24E-12
6318	WP_033272543.1	-	prepilin peptidase	1	1	1	1	1	1	1	1	1	1	1	11	CORE	COG1989_NOU#	32.6	91	5.07E-18	K02654	32.6	91	1.35E-17
6319	WP_122216809.1	-	nucleotidyltransferase	1	0	1	1	1	1	0	1	1	1	1	9	Accessory								
6320	AXI85136.1	-	TetR family transcriptional regulator	1	0	1	1	1	1	1	1	0	1	1	9	Accessory	COG1309_K#	32.5	49	0.0000288				
6321	WP_114873522.1	pgeF	peptidoglycan editing factor PgeF	1	1	1	1	1	1	1	1	1	1	1	11	CORE	COG1496_S#	50.9	209	1.67E-53	K05810	51.1	206	2.21E-52
6322	WP_122215983.1	-	M23 family metallopeptidase	1	1	1	1	1	1	1	1	1	1	1	11	CORE	COG0739_M#	34.8	114	5.59E-25	NA	45.1	99	3.81E-20
6323	WP_033273683.1	-	EboA domain-containing protein	1	0	1	1	1	1	1	1	1	1	1	10	Accessory								
6324	WP_122213824.1	-	hypothetical protein	0	0	0	0	0	0	0	0	0	1	0	1	Unique								
6325	WP_048458342.1	-	peptidase M15	1	1	1	1	1	1	1	1	1	1	1	11	CORE	COG3409_M#	49.2	58	4.76E-08	K01447	49.2	58	1.27E-07
6326	Prokka:JAPCLKMA_01621	-	hypothetical protein	1	1	1	1	0	0	1	1	0	0	0	6	Accessory	COG0053_P#	23.8	55	4.03E-07	NA	34.2	109	3.68E-23

cluster	GI	gene	Annotation	Streptomyces_thermocarboxydus_BPSAC147	Streptomyces_sp._4F	Streptomyces_sp._BSE7F	Streptomyces_sp._ETH9427	Streptomyces_sp._FxanaD5	Streptomyces_sp._UNC401CLCol	Actinospica_acidiphila_NRRL_B_24431	Streptomyces_sp._HNS054	Streptomyces_sp._SMS_SU21	Streptomyces_sp._ZS0098	Streptomyces_thermocarboxydus_K155	Addition	Core-Accessory-Unique	COG	%Similarity_COG	Score_COG	Evalue_COG	KEGG	%Similarity_KEGG	Score_KEGG	Evalue_KEGG
6327	PWE07316.1	-	Tellurium resistance	1	1	1	1	1	1	1	1	1	1	1	11	CORE	COG4110_R#	36.4	131	5.77E-30	NA	36.4	131	1.54E-29
6328	AXI90070.1	-	oxidoreductase	0	0	0	1	0	0	1	1	0	0	0	3	Accessory	COG4221_R#	51.6	226	1.01E-58	NA	47.3	220	1.48E-56
6329	Prokka:JAPCLKMA_03784	-	hypothetical protein	1	0	0	0	0	0	0	0	0	0	1	2	Accessory								
6330	Prokka:CIODPCDJ_00254	-	hypothetical protein	1	0	0	1	1	1	0	0	0	0	1	5	Accessory								
6331	Prokka:CIODPCDJ_03496	-	hypothetical protein	0	0	1	1	0	0	1	0	0	0	1	4	Accessory	COG1484_L#	32.9	69	1.58E-11	K02315	32.9	69	4.22E-11
6332	WP_051724473.1	-	adenosylcobinamide amidohydrolase	1	1	0	1	1	1	1	1	1	1	1	10	Accessory	COG1865_S#	33.7	86	1.25E-16	NA	30.8	67	2.73E-10
6333	Prokka:JAPCLKMA_05579	iphP	Tyrosine-protein phosphatase	1	0	0	0	0	0	0	0	0	0	0	1	Unique	COG2365_T#	30.2	111	6.18E-24	NA	33	92	4.65E-18
6334	WP_033273624.1	-	hypothetical protein	1	0	1	1	0	0	1	1	1	1	1	8	Accessory								
6335	WP_019524953.1	-	hypothetical protein	0	0	0	0	1	0	0	0	0	0	0	1	Unique								
6336	Prokka:JAPCLKMA_05950	-	putative HTH-type transcriptional regulator	1	0	1	1	1	1	1	1	1	1	1	10	Accessory	COG1309_K#	42.4	149	1.2E-35	NA	42.4	149	3.21E-35
6337	OSC58831.1	-	guanyltransferase	1	1	1	1	1	1	1	1	1	1	1	11	CORE	COG1208_MJ#	41.1	163	1.37E-39	K00973	38.7	156	3.43E-37
6338	OSC71048.1	-	ABC transporter ATP-binding protein	0	1	0	0	0	0	0	0	0	0	0	1	Unique	COG1131_V#	49.5	204	4.11E-52	K01990	40.9	168	8.71E-41

cluster	GI	gene	Annotation	Streptomyces_thermocarboxydus_BPSAC147	Streptomyces_sp._4F	Streptomyces_sp._BSE7F	Streptomyces_sp._ETH9427	Streptomyces_sp._FxanaD5	Streptomyces_sp._UNC401CLCol	Actinospica_acidiphila_NRRL_B_24431	Streptomyces_sp._HNS054	Streptomyces_sp._SMS_SU21	Streptomyces_sp._ZS0098	Streptomyces_thermocarboxydus_K155	Addition	Core-Accessory-Unique	COG	%Similarity_COG	Score_COG	Evalue_COG	KEGG	%Similarity_KEGG	Score_KEGG	Evalue_KEGG
6339	Prokka:CIODPCDJ_01135	metP	Methionine import system permease protein MetP	1	1	1	1	1	1	1	1	1	1	1	11	CORE	COG2011_P#	54.4	241	3.03E-63	K02072	59.2	235	4.44E-61
6340	OSC71930.1	-	hypothetical protein	0	1	0	0	0	0	0	0	0	0	0	1	Unique					NA	28	71	1.11E-11
6341	Prokka:JAPCLKMA_02216	-	hypothetical protein	1	0	0	0	0	0	0	0	0	0	0	1	Unique								
6342	WP_019526378.1	-	fibronectin type III domain-containing protein	0	0	0	0	1	1	0	0	0	0	0	2	Accessory					NA	27.4	47	0.000224
6343	Prokka:JAPCLKMA_03801	-	hypothetical protein	1	0	0	0	0	0	0	0	0	0	0	1	Unique								
6344	WP_019524549.1	-	enoyl-CoA hydratase family protein	1	1	1	1	1	1	1	1	1	1	1	11	CORE	COG1024_I#	56	243	7.95E-64	K01692	56	243	2.12E-63
6345	Prokka:CIODPCDJ_01205	lldR_2	Putative L-lactate dehydrogenase operon regulatory protein	1	1	1	1	1	1	1	1	1	1	1	11	CORE	COG2186_K#	34	91	5.05E-18	K14348	28.3	86	5.66E-16
6346	WP_122215575.1	-	Mut7-C ubiquitin/RNAse domain-containing protein	1	1	1	1	1	1	1	1	1	1	1	11	CORE	COG1656_S#	42.9	169	1.9E-41	K09122	42.9	169	5.09E-41
6347	WP_122213219.1	-	alpha/beta hydrolase	0	0	1	1	0	0	1	1	1	1	0	6	Accessory	COG0596_R#	56.3	248	1.89E-65				

cluster	GI	gene	Annotation	Streptomyces_thermocarboxydus_BPSAC147	Streptomyces_sp._4F	Streptomyces_sp._BSE7F	Streptomyces_sp._ETH9427	Streptomyces_sp._FxanaD5	Streptomyces_sp._UNC401CLCol	Actinospica_acidiphila_NRRL_B_24431	Streptomyces_sp._HNS054	Streptomyces_sp._SMS_SU21	Streptomyces_sp._ZS0098	Streptomyces_thermocarboxydus_K155	Addition	Core-Accessory-Unique	COG	%Similarity_COG	Score_COG	Evalue_COG	KEGG	%Similarity_KEGG	Score_KEGG	Evalue_KEGG
6348	PWE08469.1	-	translation initiation factor IF-2	1	0	1	1	1	1	0	1	1	1	1	9	Accessory								
6349	WP_019527722.1	-	hypothetical protein	0	0	0	0	1	0	0	0	0	1	1	3	Accessory								
6350	PWE07247.1	-	DNA primase	1	1	1	1	1	1	1	1	1	1	1	11	CORE								
6351	WP_048459733.1	-	NAD(P)H-binding protein	0	0	1	1	0	0	1	1	1	1	0	6	Accessory	COG0702_MG#	34.7	156	9.78E-38				
6352	WP_122213372.1	-	FadR family transcriptional regulator	0	1	0	0	0	0	0	0	0	1	0	2	Accessory	COG2186_K#	37.4	148	3.48E-35	K05799	32.7	92	6.05E-18
6353	WP_102640910.1	-	transglycosylase SLT domain-containing protein	1	1	1	1	1	1	1	1	1	1	1	11	CORE	COG3953_R#	30.9	81	4E-15	NA	32.8	89	6.69E-17
6354	Prokka:JAPCLKMA_00950	pcaR	Pca regulon regulatory protein	1	0	0	0	1	1	0	0	0	0	1	4	Accessory	COG1414_K#	44.7	197	5E-50	NA	33.9	108	1.07E-22
6355	OSC71737.1	-	transglycosylase	1	1	1	1	1	1	1	1	1	1	1	11	CORE	COG3583_S#	44.2	48	0.000049	NA	57	147	1.59E-34
6356	WP_102638118.1	-	hypothetical protein	0	0	1	1	0	0	0	1	1	0	0	4	Accessory								
6357	AXI87132.1	-	DNA-binding response regulator	0	0	0	1	0	0	0	1	1	0	0	3	Accessory	COG0745_TK#	42.1	181	3.71E-45	K07670	39.2	174	9.27E-43
6358	WP_048460341.1	-	thioesterase	0	0	0	1	0	0	0	1	1	1	0	4	Accessory	COG3208_Q#	36.2	117	5.04E-26	NA	26.3	87	1.95E-16
6359	OSC66906.1	-	hydrolase	1	1	1	1	1	1	1	1	1	1	1	11	CORE	COG1011_R#	28.8	76	1.68E-13				

cluster	GI	gene	Annotation	Streptomyces_thermocarboxydus_BPSAC147	Streptomyces_sp._4F	Streptomyces_sp._BSE7F	Streptomyces_sp._ETH9427	Streptomyces_sp._FxanaD5	Streptomyces_sp._UNC401CLCol	Actinospica_acidiphila_NRRL_B_24431	Streptomyces_sp._HNS054	Streptomyces_sp._SMS_SU21	Streptomyces_sp._ZS0098	Streptomyces_thermocarboxydus_K155	Addition	Core-Accessory-Unique	COG	%Similarity_COG	Score_COG	Evalue_COG	KEGG	%Similarity_KEGG	Score_KEGG	Evalue_KEGG
6360	WP_033276666.1	-	LysM peptidoglycan-binding domain-containing protein	0	1	0	0	0	0	1	0	0	0	0	2	Accessory	COG1388_M#	52.1	48	0.0000375	NA	52.1	48	0.0001
6361	PWE08823.1	-	hypothetical protein	1	1	1	1	1	1	1	1	1	1	1	11	CORE								
6362	Prokka:CIODPCDJ_05710	-	hypothetical protein	1	1	1	1	1	1	1	1	1	1	1	11	CORE	COG0789_K#	57.1	45	0.000415				
6363	WP_028960725.1	-	class II aldolase/adducin family protein	1	1	1	1	1	1	1	1	1	1	1	11	CORE	COG0235_G#	40.1	146	1.72E-34	K01628	39.3	142	6.63E-33
6364	OSC71517.1	-	AraC family transcriptional regulator	0	1	0	0	0	0	0	0	0	0	0	1	Unique	COG0693_R#	40.9	141	4.24E-33	NA	36.1	46	0.000495
6365	WP_019521878.1	-	SDR family oxidoreductase	1	0	0	0	1	1	1	0	0	0	1	5	Accessory	COG1028_IQR#	49.4	204	5.32E-52	NA	37.7	102	7.61E-21
6366	OSC70600.1	-	MerR family transcriptional regulator	0	1	0	0	0	0	0	0	0	0	0	1	Unique	COG0789_K#	34.6	70	7.03E-12	NA	38.4	55	8.16E-07
6367	WP_102640537.1	-	decarboxylase	1	1	1	1	1	1	1	1	1	1	1	11	CORE	COG3473_Q#	33.9	96	2.04E-19				
6368	AXI86229.1	-	hypothetical protein	1	1	1	1	1	1	1	1	1	1	1	11	CORE	COG1814_S#	44.6	171	2.93E-42	NA	43.5	155	5.8E-37
6369	WP_122215634.1	-	HEAT repeat domain-containing protein	1	0	0	1	0	1	0	1	0	1	0	5	Accessory	COG1413_C#	50	44	0.00092				

cluster	GI	gene	Annotation	Streptomyces_thermocarboxydus_BPSAC147	Streptomyces_sp._4F	Streptomyces_sp._BSE7F	Streptomyces_sp._ETH9427	Streptomyces_sp._FxanaD5	Streptomyces_sp._UNC401CLCol	Actinospica_acidiphila_NRRL_B_24431	Streptomyces_sp._HNS054	Streptomyces_sp._SMS_SU21	Streptomyces_sp._ZS0098	Streptomyces_thermocarboxydus_K155	Addition	Core-Accessory-Unique	COG	%Similarity_COG	Score_COG	Evalue_COG	KEGG	%Similarity_KEGG	Score_KEGG	Evalue_KEGG
6370	WP_019523452.1	-	chlorite dismutase family protein	1	1	1	1	1	1	1	1	1	1	1	11	CORE	COG3253_S#	61.7	275	1.88E-73	K09162	61.7	275	5.02E-73
6371	WP_033272833.1	-	hypothetical protein	1	0	1	1	1	1	1	1	1	1	1	10	Accessory								
6372	OSC71478.1	-	GntR family transcriptional regulator	0	1	0	0	0	0	0	1	1	0	0	3	Accessory	COG2188_K#	27.3	83	1.37E-15	K03710	27.3	83	3.66E-15
6373	WP_102641434.1	-	acyltransferase domain-containing protein	0	0	1	1	0	0	0	1	1	1	0	5	Accessory	COG3321_Q#	38.2	86	1.24E-16	K12430	38.2	86	3.31E-16
6374	Prokka:JAPCLKMA_06545	-	hypothetical protein	1	0	0	0	1	0	0	0	1	0	0	3	Accessory								
6375	WP_158688230.1	-	hypothetical protein	0	0	0	0	1	0	0	0	0	0	0	1	Unique								
6376	Prokka:JAPCLKMA_05835	-	hypothetical protein	1	1	1	1	1	1	1	1	1	1	1	11	CORE								
6377	Prokka:JAPCLKMA_03793	-	hypothetical protein	1	0	0	0	0	0	0	0	0	0	1	2	Accessory	COG0463_M#	27.9	68	3.49E-11	NA	43	65	1.03E-09
6378	OSC61731.1	-	extradiol ring-cleavage dioxygenase	0	1	0	0	0	0	0	0	0	0	0	1	Unique	COG0346_E#	30.3	73	1.08E-12				
6379	WP_158688256.1	-	hypothetical protein	0	0	0	0	1	0	0	0	0	0	0	1	Unique								
6380	Prokka:JAPCLKMA_03335	-	hypothetical protein	1	0	0	0	0	0	0	0	0	0	0	1	Unique					K01528	40.9	49	0.0000764

cluster	GI	gene	Annotation	Streptomyces_thermocarboxydus_BPSAC147	Streptomyces_sp._4F	Streptomyces_sp._BSE7F	Streptomyces_sp._ETH9427	Streptomyces_sp._FxanaD5	Streptomyces_sp._UNC401CLCol	Actinospica_acidiphila_NRRL_B_24431	Streptomyces_sp._HNS054	Streptomyces_sp._SMS_SU21	Streptomyces_sp._ZS0098	Streptomyces_thermocarboxydus_K155	Addition	Core-Accessory-Unique	COG	%Similarity_COG	Score_COG	Evalue_COG	KEGG	%Similarity_KEGG	Score_KEGG	Evalue_KEGG
6381	WP_122212641.1	-	ATP-binding protein	1	0	1	1	1	0	1	0	0	1	0	6	Accessory								
6382	WP_019526478.1	-	response regulator transcription factor	1	1	1	0	1	1	1	1	1	1	1	10	Accessory	COG0745_TK#	52.7	238	3.31E-62	K07669	53.2	238	8.84E-62
6383	Prokka:JAPCLKMA_05942	-	hypothetical protein	1	1	1	1	1	1	1	1	1	1	1	11	CORE								
6384	WP_033276920.1	-	DNA alkylation repair protein	1	0	1	1	1	1	1	1	1	1	1	10	Accessory	COG4912_L#	42.4	153	8.21E-37				
6385	OSC70177.1	-	SAM-dependent methyltransferase	0	1	0	0	0	0	0	0	0	0	0	1	Unique								
6386	WP_019521598.1	-	ABC transporter substrate-binding protein	1	1	0	1	1	1	1	1	1	1	1	10	Accessory	COG0614_P#	31.1	102	2.84E-21	K02016	32.8	74	2.21E-12
6387	WP_033277895.1	-	1-acyl-sn-glycerol-3-phosphate acyltransferase	1	1	1	1	1	1	1	1	1	1	1	11	CORE	COG0204_I#	49.6	229	1.54E-59	NA	49.6	229	4.11E-59
6388	Prokka:JAPCLKMA_00761	pytH	Pyrethroid hydrolase	1	0	1	1	1	1	1	1	0	1	1	9	Accessory	COG0596_R#	32.4	42	0.00348	K08233	25.3	74	1.3E-12
6389	PWE08411.1	-	hypothetical protein	0	0	1	0	0	0	0	0	1	0	0	2	Accessory	COG1502_I#	30.7	60	9.46E-09	NA	35.4	44	0.00245
6390	WP_048457111.1	-	helix-turn-helix transcriptional regulator	1	0	1	1	1	1	1	1	1	1	1	10	Accessory	COG1309_K#	38.6	106	1.15E-22	NA	37.1	89	6.64E-17

cluster	GI	gene	Annotation	Streptomyces_thermocarboxydus_BPSAC147	Streptomyces_sp._4F	Streptomyces_sp._BSE7F	Streptomyces_sp._ETH9427	Streptomyces_sp._FxanaD5	Streptomyces_sp._UNC401CLCol	Actinospica_acidiphila_NRRL_B_24431	Streptomyces_sp._HNS054	Streptomyces_sp._SMS_SU21	Streptomyces_sp._ZS0098	Streptomyces_thermocarboxydus_K155	Addition	Core-Accessory-Unique	COG	%Similarity_COG	Score_COG	Evalue_COG	KEGG	%Similarity_KEGG	Score_KEGG	Evalue_KEGG
6391	WP_019524725.1	-	glutamine amidotransferase	1	1	1	1	1	1	1	1	1	1	1	11	CORE	COG3442_R#	45.1	188	3.93E-47	K07009	45.1	188	1.05E-46
6392	OSC52801.1	-	hypothetical protein	1	1	1	1	1	1	1	1	1	1	1	11	CORE								
6393	WP_048458401.1	-	hypothetical protein	0	0	1	1	0	0	1	1	1	1	0	6	Accessory								
6394	AXI85543.1	-	dethiobiotin synthase	1	0	1	1	1	1	1	1	1	1	1	10	Accessory	COG0132_H#	46.7	162	2.31E-39	K01935	46.7	161	8.06E-39
6395	Prokka:JAPCLKMA_03765	phnF	putative transcriptional regulator PhnF	1	0	1	0	1	0	0	1	0	0	0	4	Accessory	COG2188_K#	25.2	90	1.12E-17	K03710	33	81	1.06E-14
6396	Prokka:CIODPCDJ_01199	-	hypothetical protein	0	0	0	0	0	0	0	0	0	0	1	1	Unique					NA	25.6	46	0.000493
6397	OSC69079.1	-	TetR family transcriptional regulator	1	1	1	1	1	1	1	1	1	1	1	11	CORE	COG1309_K#	30	89	1.46E-17	NA	31.1	64	1.75E-09
6398	Prokka:JAPCLKMA_00188	udgB	Type-5 uracil-DNA glycosylase	1	1	1	1	1	1	1	1	1	1	1	11	CORE	COG1573_L#	57.1	262	1.26E-69	NA	57.1	262	3.35E-69
6399	WP_146217797.1	-	substrate-binding domain-containing protein	0	0	1	0	0	0	0	0	1	0	0	2	Accessory	COG1609_K#	44.2	58	3.6E-08	K03604	34.3	52	0.00000527
6400	WP_102640887.1	-	hypothetical protein	1	0	0	1	1	1	1	1	1	1	1	9	Accessory					NA	34.2	86	5.62E-16
6401	WP_048458311.1	-	methyltransferase domain-containing protein	1	0	1	0	1	1	1	1	1	1	1	9	Accessory	COG0500_QR#	33.3	67	1.01E-10	NA	33.1	66	3.53E-10

cluster	GI	gene	Annotation	Streptomyces_thermocarboxydus_BPSAC147	Streptomyces_sp._4F	Streptomyces_sp._BSE7F	Streptomyces_sp._ETH9427	Streptomyces_sp._FxanaD5	Streptomyces_sp._UNC401CLCol	Actinospica_acidiphila_NRRL_B_24431	Streptomyces_sp._HNS054	Streptomyces_sp._SMS_SU21	Streptomyces_sp._ZS0098	Streptomyces_thermocarboxydus_K155	Addition	Core-Accessory-Unique	COG	%Similarity_COG	Score_COG	Evalue_COG	KEGG	%Similarity_KEGG	Score_KEGG	Evalue_KEGG
6402	PWE08809.1	-	branched-chain amino acid ABC transporter permease	1	0	1	1	1	1	0	1	1	1	1	9	Accessory	COG1296_E#	43.6	181	3.68E-45	NA	31.1	79	5.26E-14
6403	WP_019521289.1	-	DUF2470 domain-containing protein	1	1	1	1	1	1	1	1	1	1	1	11	CORE								
6404	OSC62256.1	-	short-chain dehydrogenase	0	1	0	0	1	0	1	0	0	1	1	5	Accessory	COG1028_IQR#	42.5	175	2.64E-43	NA	42.5	175	7.04E-43
6405	WP_048457001.1	-	DUF2625 domain-containing protein	0	0	0	0	0	0	0	1	0	0	0	1	Unique								
6406	OSC70434.1	-	4-hydroxythreonine-4-phosphate dehydrogenase PdxA	0	1	0	0	0	0	0	0	0	0	0	1	Unique	COG1995_H#	53.7	242	1.34E-63	K00097	45.5	187	1.79E-46
6407	AXI89639.1	-	hypothetical protein	0	0	0	1	0	0	0	0	0	0	0	1	Unique								
6408	AXI84687.1	-	peptidase E	0	0	0	1	0	0	0	1	0	0	0	2	Accessory	COG3340_E#	34.2	142	1.89E-33	NA	35.8	128	9.87E-29
6409	WP_048456157.1	-	Crp/Fnr family transcriptional regulator	1	0	0	1	1	1	0	1	0	1	1	7	Accessory	COG0664_T#	26.9	73	1.41E-12	NA	26.9	73	3.77E-12
6410	OSC60989.1	-	hypothetical protein	0	1	0	0	0	0	0	0	0	0	0	1	Unique								

cluster	GI	gene	Annotation	Streptomyces_thermocarboxydus_BPSAC147	Streptomyces_sp._4F	Streptomyces_sp._BSE7F	Streptomyces_sp._ETH9427	Streptomyces_sp._FxanaD5	Streptomyces_sp._UNC401CLCol	Actinospica_acidiphila_NRRL_B_24431	Streptomyces_sp._HNS054	Streptomyces_sp._SMS_SU21	Streptomyces_sp._ZS0098	Streptomyces_thermocarboxydus_K155	Addition	Core-Accessory-Unique	COG	%Similarity_COG	Score_COG	Evalue_COG	KEGG	%Similarity_KEGG	Score_KEGG	Evalue_KEGG
6411	WP_078493100.1	-	N-acyl homoserine lactonase family protein	0	0	0	0	0	0	1	0	0	1	0	2	Accessory	COG0491_R#	32	80	6.77E-15	NA	32	80	1.81E-14
6412	OSC55844.1	-	aspartate aminotransferase family protein	0	1	0	0	0	0	0	0	0	0	0	1	Unique	COG0160_E#	37.7	183	1.26E-45	K00823	37.7	183	3.38E-45
6413	Prokka:JAPCLKMA_05794	-	hypothetical protein	1	0	0	0	0	0	0	0	0	0	0	1	Unique								
6414	WP_048458870.1	-	DUF4839 domain-containing protein	0	0	0	0	0	0	0	1	0	0	0	1	Unique								
6415	WP_019525222.1	rplA	50S ribosomal protein L1	1	0	1	1	1	1	1	1	1	1	1	10	Accessory	COG0081_J#	73.6	352	1.2E-96	K02863	73.2	349	2.71E-95
6416	WP_033275250.1	-	class I SAM-dependent methyltransferase	1	1	1	1	1	1	1	1	1	1	1	11	CORE	COG0500_QR#	42.3	172	1.7E-42	NA	42.3	172	4.54E-42
6417	PWE11531.1	-	hypothetical protein	0	0	1	1	0	0	0	1	1	1	0	5	Accessory								
6418	WP_033275189.1	-	CDP-alcohol phosphatidyltransferase family protein	1	0	1	1	1	1	1	1	1	1	1	10	Accessory	COG0558_I#	45.2	171	3.79E-42	K00995	44.4	162	4.7E-39

cluster	GI	gene	Annotation	Streptomyces_thermocarboxydus_BPSAC147	Streptomyces_sp._4F	Streptomyces_sp._BSE7F	Streptomyces_sp._ETH9427	Streptomyces_sp._FxanaD5	Streptomyces_sp._UNC401CLCol	Actinospica_acidiphila_NRRL_B_24431	Streptomyces_sp._HNS054	Streptomyces_sp._SMS_SU21	Streptomyces_sp._ZS0098	Streptomyces_thermocarboxydus_K155	Addition	Core-Accessory-Unique	COG	%Similarity_COG	Score_COG	Evalue_COG	KEGG	%Similarity_KEGG	Score_KEGG	Evalue_KEGG
6419	PWE08652.1	-	disulfide bond formation protein DsbA	0	0	1	1	0	0	0	1	1	1	0	5	Accessory								
6420	WP_048455311.1	-	NAD-dependent deacylase	1	1	1	1	1	1	1	1	1	1	1	11	CORE	COG0846_K#	40.8	177	6.91E-44	K12410	42.9	155	5.75E-37
6421	WP_033277675.1	-	MBL fold metallo-hydrolase	1	1	0	0	0	1	1	0	0	0	1	5	Accessory	COG0491_R#	31.7	86	2.09E-16	NA	33.3	74	1.29E-12
6422	WP_122214959.1	-	3'-5' exonuclease	1	1	1	1	1	1	1	1	1	1	1	11	CORE	COG0847_L#	38.9	140	7.17E-33				
6423	WP_048456854.1	priA	bifunctional 1-(5-phosphoribosyl)-5-((5-phosphoribosylamino)methylideneamino)imidazole-4-carboxamide isomerase/phosphoribosylanthranilate isomerase PriA	1	1	1	1	1	1	1	1	1	1	1	11	CORE	COG0106_E#	68.7	333	7.53E-91	K01817	68.7	333	2.01E-90
6424	AXI84680.1	-	dienelactone hydrolase	0	1	0	1	0	0	1	1	0	0	0	4	Accessory	COG0412_Q#	34	139	1.6E-32				
6425	OSC68520.1	-	hypothetical protein	0	1	0	0	0	0	0	1	0	0	0	2	Accessory								

cluster	GI	gene	Annotation	Streptomyces_thermocarboxydus_BPSAC147	Streptomyces_sp._4F	Streptomyces_sp._BSE7F	Streptomyces_sp._ETH9427	Streptomyces_sp._FxanaD5	Streptomyces_sp._UNC401CLCol	Actinospica_acidiphila_NRRL_B_24431	Streptomyces_sp._HNS054	Streptomyces_sp._SMS_SU21	Streptomyces_sp._ZS0098	Streptomyces_thermocarboxydus_K155	Addition	Core-Accessory-Unique	COG	%Similarity_COG	Score_COG	Evalue_COG	KEGG	%Similarity_KEGG	Score_KEGG	Evalue_KEGG
6426	OSC69752.1	-	nucleoside/nucleotide kinase family protein	0	1	0	0	0	0	0	0	0	0	0	1	Unique	COG1072_H#	35.7	115	2.47E-25	K02173	29.8	77	1.99E-13
6427	OSC73005.1	-	transcriptional regulator	1	1	1	1	1	1	1	1	1	1	1	11	CORE								
6428	OSC63815.1	-	hypothetical protein	0	1	0	0	0	0	0	0	0	0	0	1	Unique					NA	32.8	142	5.04E-33
6429	OSC64046.1	-	sugar ABC transporter permease	0	1	0	0	0	0	0	0	0	0	0	1	Unique	COG0395_G#	38.6	165	2.08E-40	K02026	35.2	143	2.26E-33
6430	OSC62352.1	-	Asp/Glu/hydantoin racemase	0	1	0	0	0	0	0	0	0	0	0	1	Unique	COG4126_E#	41.6	175	2.01E-43				
6431	OSC65834.1	-	cytochrome	0	1	0	0	0	0	0	0	0	0	0	1	Unique	COG2124_Q#	37.8	151	5.3E-36	NA	37.8	151	1.42E-35
6432	Prokka:CIODPCDJ_01966	-	hypothetical protein	1	1	1	1	1	1	1	1	1	1	1	11	CORE	COG2119_S#	32	91	4.99E-18	NA	32.5	77	1.99E-13
6433	PWE08895.1	-	hypothetical protein	1	0	1	1	1	1	0	1	1	1	1	9	Accessory								
6434	OSC59935.1	-	hypothetical protein	1	1	1	1	1	1	1	1	1	1	1	11	CORE								
6435	OSC72621.1	-	hypothetical protein	1	1	0	1	1	1	1	1	1	1	1	10	Accessory	COG0671_I#	28.2	55	3.03E-07				
6436	OSC56634.1	-	dipeptidase E	0	1	0	0	0	0	0	0	0	0	0	1	Unique	COG3340_E#	44.3	181	3.66E-45	K05995	53.4	238	8.81E-62
6437	OSC62723.1	-	SAM-dependent methyltransferase	0	1	0	0	0	0	0	0	0	0	0	1	Unique	COG0500_QR#	32.5	129	1.65E-29				

cluster	GI	gene	Annotation	Streptomyces_thermocarboxydus_BPSAC147	Streptomyces_sp._4F	Streptomyces_sp._BSE7F	Streptomyces_sp._ETH9427	Streptomyces_sp._FxanaD5	Streptomyces_sp._UNC401CLCol	Actinospica_acidiphila_NRRL_B_24431	Streptomyces_sp._HNS054	Streptomyces_sp._SMS_SU21	Streptomyces_sp._ZS0098	Streptomyces_thermocarboxydus_K155	Addition	Core-Accessory-Unique	COG	%Similarity_COG	Score_COG	Evalue_COG	KEGG	%Similarity_KEGG	Score_KEGG	Evalue_KEGG
6438	WP_106432542.1	-	sigma-70 family RNA polymerase sigma factor	1	0	1	1	1	1	1	1	1	1	1	10	Accessory	COG1595_K#	33.2	86	1.23E-16	K03088	33.2	86	3.28E-16
6439	OSC61100.1	-	hypothetical protein	0	1	0	0	0	0	0	0	0	0	0	1	Unique	COG1309_K#	33.7	72	2.4E-12	NA	33.7	72	6.4E-12
6440	AXI85288.1	-	NUDIX hydrolase	0	0	0	1	0	0	0	0	0	0	0	1	Unique	COG0494_LR#	45.7	44	0.000913				
6441	OSC65794.1	-	short-chain dehydrogenase	0	1	0	0	0	0	0	0	0	0	0	1	Unique	COG1028_IQR#	31.1	73	1.08E-12	NA	29.7	70	2.43E-11
6442	OSC62303.1	-	peptidyl-tRNA hydrolase	1	1	1	1	1	1	1	1	1	1	1	11	CORE	COG1990_S#	41.8	119	1.71E-26				
6443	OSC70034.1	-	hydrolase	1	1	1	1	1	1	1	1	1	1	1	11	CORE								
6444	WP_020700212.1	-	hypothetical protein	0	0	0	0	1	0	0	0	0	0	0	1	Unique								
6445	Prokka:CIODPCDJ_04484	-	hypothetical protein	1	1	1	1	1	1	1	1	1	1	1	11	CORE	COG3571_R#	34.1	98	3.11E-20	K07020	32.4	77	1.52E-13
6446	Prokka:JAPCLKMA_03889	-	hypothetical protein	1	1	1	1	1	1	1	1	1	1	1	11	CORE								
6447	WP_019526834.1	-	CBS domain-containing protein	1	0	1	1	1	1	1	1	1	1	1	10	Accessory	COG0517_R#	32.9	95	3.44E-19	NA	28.7	69	4.13E-11
6448	Prokka:CIODPCDJ_01299	-	hypothetical protein	1	1	1	1	1	1	1	1	1	1	1	11	CORE	COG0640_K#	48.4	176	8.99E-44	NA	40.8	170	1.72E-41
6449	OSC70061.1	-	EamA family transporter	0	1	0	0	0	0	0	0	0	0	0	1	Unique	COG0697_GER#	28.9	101	3.67E-21	NA	26.6	61	1.47E-08

cluster	GI	gene	Annotation	Streptomyces_thermocarboxydus_BPSAC147	Streptomyces_sp._4F	Streptomyces_sp._BSE7F	Streptomyces_sp._ETH9427	Streptomyces_sp._FxanaD5	Streptomyces_sp._UNC401CLCol	Actinospica_acidiphila_NRRL_B_24431	Streptomyces_sp._HNS054	Streptomyces_sp._SMS_SU21	Streptomyces_sp._ZS0098	Streptomyces_thermocarboxydus_K155	Addition	Core-Accessory-Unique	COG	%Similarity_COG	Score_COG	Evalue_COG	KEGG	%Similarity_KEGG	Score_KEGG	Evalue_KEGG
6450	WP_158688229.1	-	hypothetical protein	0	0	0	0	1	0	0	0	0	0	0	1	Unique								
6451	Prokka:CIODPCDJ_04715	-	hypothetical protein	1	0	1	1	1	1	1	1	1	1	1	10	Accessory								
6452	OSC68929.1	-	respiratory nitrate reductase subunit gamma	0	1	0	0	0	0	0	0	0	0	0	1	Unique	COG2181_C#	54	275	1.86E-73	K00370	54	275	4.96E-73
6453	WP_122212773.1	-	LysM peptidoglycan-binding domain-containing protein	0	0	0	1	0	0	0	1	1	1	0	4	Accessory	COG1652_S#	29.5	77	7.42E-14	NA	49	48	0.000129
6454	WP_033273890.1	-	SDR family oxidoreductase	0	0	0	0	0	0	1	0	0	0	0	1	Unique	COG1028_IQR#	40.8	161	2.99E-39	NA	33.6	112	5.56E-24
6455	Prokka:JAPCLKMA_01577	fabG_5	3-oxoacyl-[acyl-carrier-protein] reductase FabG	1	1	1	1	1	1	1	1	1	1	1	11	CORE	COG1028_IQR#	55.4	255	2.6E-67	K11610	51.7	235	7.43E-61
6456	OSC57360.1	-	hypothetical protein	0	1	0	0	0	0	0	0	0	0	0	1	Unique	COG2730_G#	40.8	74	6.28E-13	NA	31.9	82	8.04E-15
6457	AXI89500.1	-	hypothetical protein	0	0	0	1	0	0	0	0	0	0	0	1	Unique								
6458	WP_084206959.1	-	MFS transporter	0	0	0	0	0	0	1	0	0	0	0	1	Unique	COG0477_GEPR#	33.2	140	9.32E-33	K07001	35.2	137	1.61E-31
6459	OSC69180.1	-	hypothetical protein	1	1	0	1	1	1	1	1	1	1	1	10	Accessory								

cluster	GI	gene	Annotation	Streptomyces_thermocarboxydus_BPSAC147	Streptomyces_sp._4F	Streptomyces_sp._BSE7F	Streptomyces_sp._ETH9427	Streptomyces_sp._FxanaD5	Streptomyces_sp._UNC401CLCol	Actinospica_acidiphila_NRRL_B_24431	Streptomyces_sp._HNS054	Streptomyces_sp._SMS_SU21	Streptomyces_sp._ZS0098	Streptomyces_thermocarboxydus_K155	Addition	Core-Accessory-Unique	COG	%Similarity_COG	Score_COG	Evalue_COG	KEGG	%Similarity_KEGG	Score_KEGG	Evalue_KEGG
6460	WP_019527642.1	-	ABC transporter ATP-binding protein	1	0	0	0	1	1	1	0	0	0	1	5	Accessory	COG1116_P#	66	301	2.41E-81	K15555	52	213	2.3E-54
6461	WP_019521943.1	-	YggS family pyridoxal phosphate-dependent enzyme	1	1	1	1	1	1	1	1	1	1	1	11	CORE	COG0325_R#	41.9	156	9.58E-38	K06997	41.9	156	2.56E-37
6462	WP_122213186.1	-	FAD dependent dehydrogenase	1	1	1	1	1	1	1	1	1	1	1	11	CORE								
6463	PWE08345.1	-	16S rRNA (guanine(527)-N(7))-methyltransferase RsmG	1	1	1	1	1	1	1	1	1	1	1	11	CORE	COG0357_M#	51	210	9.54E-54	K03501	47.7	199	3.44E-50
6464	Prokka:JAPCLKMA_00969	-	hypothetical protein	1	1	0	0	1	1	0	0	0	0	1	5	Accessory	COG3819_S#	56	232	1.37E-60				
6465	WP_033273304.1	-	ABC transporter permease	1	1	1	1	1	1	1	1	1	1	1	11	CORE	COG1277_R#	25.2	47	0.0000819	K01992	25.2	47	0.000219
6466	PWE08359.1	-	transcriptional regulator	1	1	1	1	1	1	1	1	1	1	1	11	CORE								
6467	OSC69267.1	-	DNA primase	0	1	0	0	0	0	0	0	0	0	0	1	Unique	COG3064_M#	28.4	54	5.13E-07	NA	27.2	63	3.85E-09
6468	WP_122216223.1	-	ABC transporter permease	0	0	1	1	0	0	0	0	1	1	0	4	Accessory								

cluster	GI	gene	Annotation	Streptomyces_thermocarboxydus_BPSAC147	Streptomyces_sp._4F	Streptomyces_sp._BSE7F	Streptomyces_sp._ETH9427	Streptomyces_sp._FxanaD5	Streptomyces_sp._UNC401CLCol	Actinospica_acidiphila_NRRL_B_24431	Streptomyces_sp._HNS054	Streptomyces_sp._SMS_SU21	Streptomyces_sp._ZS0098	Streptomyces_thermocarboxydus_K155	Addition	Core-Accessory-Unique	COG	%Similarity_COG	Score_COG	Evalue_COG	KEGG	%Similarity_KEGG	Score_KEGG	Evalue_KEGG
6469	OSC72632.1	-	glycerophosphodiester phosphodiesterase	1	1	1	1	1	1	1	1	1	1	1	11	CORE	COG0584_C#	29.1	79	2.54E-14	K01126	22.8	72	8.29E-12
6470	WP_033273787.1	-	SGNH/GDSL hydrolase family protein	0	0	0	0	0	0	1	0	0	0	0	1	Unique								
6471	WP_147474378.1	-	hypothetical protein	0	0	0	0	0	0	0	0	0	1	0	1	Unique					NA	41.4	162	6.09E-39
6472	Prokka:CIODPCDJ_04630	copR	Transcriptional activator protein CopR	1	1	1	1	1	1	1	1	1	1	1	11	CORE	COG0745_TK#	47.1	191	4.59E-48	K02483	47.1	191	1.22E-47
6473	Prokka:CIODPCDJ_06724	-	IS6 family transposase ISRH1	0	0	0	0	0	0	0	0	0	0	1	1	Unique	COG3316_L#	55.2	262	2.12E-69				
6474	WP_033276510.1	-	hypothetical protein	0	0	1	0	0	0	1	0	0	0	0	2	Accessory								
6475	OSC68619.1	-	phosphatase PAP2 family protein	1	1	1	1	1	1	1	1	1	1	1	11	CORE	COG0671_I#	39.3	65	2.91E-10	K19302	29.3	59	5.56E-08
6476	OSC71221.1	-	2-keto-4-pentenoate hydratase	0	1	0	0	0	0	0	0	0	0	0	1	Unique	COG3971_Q#	63.1	252	1.28E-66	K02554	53.7	239	3E-62
6477	Prokka:CIODPCDJ_06719	-	hypothetical protein	0	0	0	0	0	1	0	0	0	0	1	2	Accessory	COG0494_LR#	28	69	2.63E-11				
6478	WP_051724622.1	-	histidine phosphatase family protein	1	1	1	1	1	1	1	1	1	1	1	11	CORE					NA	48.6	201	1.18E-50

cluster	GI	gene	Annotation	Streptomyces_thermocarboxydus_BPSAC147	Streptomyces_sp._4F	Streptomyces_sp._BSE7F	Streptomyces_sp._ETH9427	Streptomyces_sp._FxanaD5	Streptomyces_sp._UNC401CLCol	Actinospica_acidiphila_NRRL_B_24431	Streptomyces_sp._HNS054	Streptomyces_sp._SMS_SU21	Streptomyces_sp._ZS0098	Streptomyces_thermocarboxydus_K155	Addition	Core-Accessory-Unique	COG	%Similarity_COG	Score_COG	Evalue_COG	KEGG	%Similarity_KEGG	Score_KEGG	Evalue_KEGG
6479	WP_019521479.1	-	ABC transporter ATP-binding protein	0	1	0	0	1	1	1	1	1	0	1	7	Accessory	COG1136_V#	44.3	195	2.42E-49	K02003	41.6	179	4.8E-44
6480	AXI89849.1	-	MerR family DNA-binding transcriptional regulator	1	0	0	1	1	1	1	1	1	1	1	9	Accessory	COG0789_K#	36.8	49	0.000028	NA	36.8	49	0.0000748
6481	WP_048456883.1	-	ABC transporter ATP-binding protein	1	1	1	1	1	1	1	1	1	1	1	11	CORE	COG0410_E#	57.7	281	3.35E-75	K01996	58.5	278	5.81E-74
6482	WP_086701191.1	npdG	NADPH-dependent F420 reductase	1	1	1	1	1	1	1	1	1	1	1	11	CORE	COG2085_R#	38.3	133	1.13E-30	K00263	41	44	0.00184
6483	AXI88009.1	-	hypothetical protein	1	0	1	1	1	1	1	1	1	1	1	10	Accessory	COG3382_S#	40.7	178	4E-44				
6484	OSC69717.1	-	hypothetical protein	0	1	0	0	0	0	0	0	0	0	0	1	Unique								
6485	AXI90899.1	-	hypothetical protein	0	0	0	1	0	0	1	0	0	0	0	2	Accessory								
6486	WP_102638875.1	-	hypothetical protein	1	0	1	1	1	1	0	1	1	1	1	9	Accessory								
6487	OSC61047.1	-	thiamine biosynthesis protein	0	1	0	0	0	0	0	0	0	0	0	1	Unique	COG1477_H#	35.5	154	4.74E-37				
6488	WP_102640351.1	-	helix-turn-helix domain-containing protein	0	0	1	0	1	1	0	0	1	0	1	5	Accessory	COG3409_M#	27.5	58	4.62E-08	K01449	36	47	0.000284
6489	AXI88529.1	-	hypothetical protein	1	1	1	1	1	1	1	1	1	1	1	11	CORE	COG3064_M#	31.8	62	3.2E-09	K03646	31.8	62	8.54E-09

cluster	GI	gene	Annotation	Streptomyces_thermocarboxydus_BPSAC147	Streptomyces_sp._4F	Streptomyces_sp._BSE7F	Streptomyces_sp._ETH9427	Streptomyces_sp._FxanaD5	Streptomyces_sp._UNC401CLCol	Actinospica_acidiphila_NRRL_B_24431	Streptomyces_sp._HNS054	Streptomyces_sp._SMS_SU21	Streptomyces_sp._ZS0098	Streptomyces_thermocarboxydus_K155	Addition	Core-Accessory-Unique	COG	%Similarity_COG	Score_COG	Evalue_COG	KEGG	%Similarity_KEGG	Score_KEGG	Evalue_KEGG
6490	WP_028960528.1	-	toll/interleukin-1 receptor domain-containing protein	0	0	0	0	0	1	0	0	0	0	0	1	Unique								
6491	WP_019524830.1	-	response regulator transcription factor	0	0	1	1	1	1	0	1	1	1	1	8	Accessory	COG0745_TK#	50.6	225	2.85E-58	K02483	50.6	225	7.62E-58
6492	WP_102638142.1	-	NUDIX domain-containing protein	0	0	0	0	0	0	0	1	1	1	0	3	Accessory								
6493	Prokka:CIODPCDJ_00400	cpnA_1	Cyclopentanol dehydrogenase	1	0	0	0	1	1	1	0	0	0	1	5	Accessory	COG1028_IQR#	45.8	167	5.41E-41	K00059	32.3	93	3.46E-18
6494	Prokka:JAPCLKMA_01495	-	hypothetical protein	1	0	0	1	0	0	0	0	0	0	0	2	Accessory								
6495	WP_078894957.1	-	DUF421 domain-containing protein	0	0	0	1	0	0	0	1	0	1	0	3	Accessory	COG2323_S#	27.9	79	1.94E-14	NA	27.9	79	5.17E-14
6496	Prokka:JAPCLKMA_01570	-	hypothetical protein	1	0	0	1	0	0	0	0	0	0	0	2	Accessory								
6497	OSC64075.1	-	hypothetical protein	0	1	0	0	0	0	0	0	0	0	0	1	Unique					NA	32.6	53	0.00000304
6498	OSC72224.1	-	proton-conducting membrane transporter	1	1	1	1	1	1	1	1	1	1	1	11	CORE	COG0839_C#	37.5	96	1.53E-19	K05578	37.5	96	4.09E-19

cluster	GI	gene	Annotation	Streptomyces_thermocarboxydus_BPSAC147	Streptomyces_sp._4F	Streptomyces_sp._BSE7F	Streptomyces_sp._ETH9427	Streptomyces_sp._FxanaD5	Streptomyces_sp._UNC401CLCol	Actinospica_acidiphila_NRRL_B_24431	Streptomyces_sp._HNS054	Streptomyces_sp._SMS_SU21	Streptomyces_sp._ZS0098	Streptomyces_thermocarboxydus_K155	Addition	Core-Accessory-Unique	COG	%Similarity_COG	Score_COG	Evalue_COG	KEGG	%Similarity_KEGG	Score_KEGG	Evalue_KEGG
6499	WP_122215716.1	-	NADPH-dependent F420 reductase	0	0	0	0	0	0	0	0	0	1	0	1	Unique	COG2085_R#	46.7	179	1.05E-44				
6500	OSC66907.1	-	IclR family transcriptional regulator	1	1	1	1	1	1	1	1	1	1	1	11	CORE	COG1414_K#	53	215	2.95E-55	NA	52.6	215	4.62E-55
6501	WP_048459937.1	-	TetR/AcrR family transcriptional regulator	0	0	1	0	0	0	1	1	1	0	1	5	Accessory	COG1309_K#	29.6	77	5.61E-14	NA	29.6	77	1.5E-13
6502	WP_122216678.1	-	NAD(P)H-binding protein	0	0	1	1	0	0	0	1	0	1	0	4	Accessory					K07118	29.6	42	0.00698
6503	OSC70563.1	-	hypothetical protein	0	1	0	0	0	0	0	0	0	0	0	1	Unique								
6504	WP_019523324.1	-	hypothetical protein	1	1	1	1	1	1	0	1	1	1	1	10	Accessory					NA	25.3	57	0.00000021
6505	Prokka:JAPCLKMA_02228	-	hypothetical protein	1	0	1	1	1	1	1	1	1	1	1	10	Accessory								
6506	OSC71621.1	-	MFS transporter	0	1	0	0	0	0	0	0	0	0	0	1	Unique	COG0477_GEPR#	42.1	209	1.61E-53	K03761	39.6	176	3.09E-43
6507	AXI87116.1	-	DNA-binding response regulator	0	0	1	1	0	0	0	1	1	1	0	5	Accessory	COG2197_TK#	31.9	113	1.2E-24	K07692	31.4	113	3.22E-24
6508	WP_033276638.1	-	hypothetical protein	0	0	0	0	0	0	1	0	0	0	0	1	Unique								
6509	PWE06781.1	-	TetR/AcrR family transcriptional regulator	0	0	1	0	0	0	1	0	1	1	0	4	Accessory	COG1309_K#	33.7	94	5.79E-19	NA	37.6	60	3.23E-08

cluster	GI	gene	Annotation	Streptomyces_thermocarboxydus_BPSAC147	Streptomyces_sp._4F	Streptomyces_sp._BSE7F	Streptomyces_sp._ETH9427	Streptomyces_sp._FxanaD5	Streptomyces_sp._UNC401CLCol	Actinospica_acidiphila_NRRL_B_24431	Streptomyces_sp._HNS054	Streptomyces_sp._SMS_SU21	Streptomyces_sp._ZS0098	Streptomyces_thermocarboxydus_K155	Addition	Core-Accessory-Unique	COG	%Similarity_COG	Score_COG	Evalue_COG	KEGG	%Similarity_KEGG	Score_KEGG	Evalue_KEGG
6510	Prokka:JAPCLKMA_05133	-	hypothetical protein	1	1	1	1	1	1	1	1	1	1	1	11	CORE								
6511	WP_026243783.1	-	HAD family phosphatase	1	0	1	1	1	1	1	1	1	1	1	10	Accessory	COG0637_R#	37.5	106	1.92E-22	K00861	36.3	109	4.65E-23
6512	WP_086020245.1	-	hypothetical protein	0	0	0	0	1	1	0	0	0	0	1	3	Accessory								
6513	AXI85291.1	-	hypothetical protein	0	0	0	1	0	0	0	0	0	0	0	1	Unique								
6514	WP_122215714.1	-	SDR family oxidoreductase	0	0	0	0	0	0	0	0	0	1	0	1	Unique	COG1028_IQR#	61.6	291	3.23E-78	NA	32.8	129	4.34E-29
6515	OSC72012.1	-	thioesterase	0	1	0	0	0	0	0	0	0	0	0	1	Unique	COG3208_Q#	42.6	182	1.62E-45	NA	35.4	131	1.14E-29
6516	Prokka:JAPCLKMA_03720	-	hypothetical protein	1	0	0	1	0	1	1	0	0	0	0	4	Accessory								
6517	OSC72532.1	-	nucleoside-diphosphate-sugar pyrophosphorylase	0	1	0	0	0	0	0	0	0	0	0	1	Unique	COG1208_MJ#	58.6	287	3.57E-77	K00966	58.6	287	9.53E-77
6518	PWE06582.1	-	MBL fold metallo-hydrolase	1	1	1	1	1	1	1	1	1	1	1	11	CORE	COG0491_R#	44.7	192	1.56E-48	NA	44.7	192	4.16E-48
6519	WP_048455520.1	-	phosphoadenylyl-sulfate reductase	1	1	1	1	1	1	1	1	1	1	1	11	CORE	COG0175_EH#	50.8	259	1.04E-68	K00390	50.8	259	2.77E-68
6520	OSC61234.1	-	GntR family transcriptional regulator	0	1	0	0	0	0	0	0	0	0	0	1	Unique	COG1802_K#	38.3	148	2.58E-35	NA	33	127	1.26E-28
6521	WP_147474340.1	-	hypothetical protein	0	0	0	0	0	0	0	0	0	1	0	1	Unique								

cluster	GI	gene	Annotation	Streptomyces_thermocarboxydus_BPSAC147	Streptomyces_sp._4F	Streptomyces_sp._BSE7F	Streptomyces_sp._ETH9427	Streptomyces_sp._FxanaD5	Streptomyces_sp._UNC401CLCol	Actinospica_acidiphila_NRRL_B_24431	Streptomyces_sp._HNS054	Streptomyces_sp._SMS_SU21	Streptomyces_sp._ZS0098	Streptomyces_thermocarboxydus_K155	Addition	Core-Accessory-Unique	COG	%Similarity_COG	Score_COG	Evalue_COG	KEGG	%Similarity_KEGG	Score_KEGG	Evalue_KEGG
6522	OSC71667.1	-	hypothetical protein	0	1	0	0	0	0	0	0	0	0	0	1	Unique								
6523	WP_019521417.1	-	(2Fe-2S)-binding protein	1	0	0	1	1	1	1	1	0	1	1	8	Accessory					NA	64.9	84	1.59E-15
6524	WP_019521538.1	-	TetR/AcrR family transcriptional regulator	1	0	0	0	1	1	1	0	0	0	1	5	Accessory	COG1309_K#	27.2	71	4E-12	NA	27.6	68	1.18E-10
6525	Prokka:CIODPCDJ_03431	-	hypothetical protein	1	0	1	0	1	1	1	1	1	1	1	9	Accessory								
6526	Prokka:CIODPCDJ_00026	-	hypothetical protein	1	0	0	0	1	1	0	0	0	0	1	4	Accessory	COG2180_C#	42.1	141	5.37E-33	K00373	42.1	141	1.43E-32
6527	OSC56430.1	-	2-phosphosulfolactate phosphatase	1	1	1	1	1	1	1	1	1	1	1	11	CORE	COG2045_HR#	29.7	96	1.16E-19	K05979	29.7	96	3.1E-19
6528	WP_122216569.1	-	helix-turn-helix domain-containing protein	0	0	1	1	0	0	1	1	1	1	0	6	Accessory								
6529	WP_033277944.1	-	hypothetical protein	1	1	1	0	1	1	1	1	1	1	1	10	Accessory								
6530	WP_037954337.1	cas5c	type I-C CRISPR-associated protein Cas5	0	0	0	1	0	1	0	0	0	0	0	2	Accessory								
6531	Prokka:JAPCLKMA_06248	udk_2	Uridine kinase	1	0	1	1	1	1	1	1	1	1	1	10	Accessory	COG1072_H#	43.3	143	1.41E-33	K02173	32.5	91	1.3E-17
6532	Prokka:CIODPCDJ_03578	-	hypothetical protein	1	0	0	0	1	1	1	0	0	0	1	5	Accessory	COG1309_K#	30.6	92	2.19E-18	NA	33.1	56	3.56E-07

cluster	GI	gene	Annotation	Streptomyces_thermocarboxydus_BPSAC147	Streptomyces_sp._4F	Streptomyces_sp._BSE7F	Streptomyces_sp._ETH9427	Streptomyces_sp._FxanaD5	Streptomyces_sp._UNC401CLCol	Actinospica_acidiphila_NRRL_B_24431	Streptomyces_sp._HNS054	Streptomyces_sp._SMS_SU21	Streptomyces_sp._ZS0098	Streptomyces_thermocarboxydus_K155	Addition	Core-Accessory-Unique	COG	%Similarity_COG	Score_COG	Evalue_COG	KEGG	%Similarity_KEGG	Score_KEGG	Evalue_KEGG
6533	WP_122212620.1	ehuC	ectoine/hydroxyectoine ABC transporter permease subunit EhuC	1	1	1	1	1	1	1	1	1	1	1	11	CORE	COG0765_E#	49.3	199	1.66E-50	K02029	34.6	131	1.48E-29
6534	WP_122215892.1	-	maleylpyruvate isomerase family mycothiol-dependent enzyme	0	0	0	0	0	0	0	0	0	1	0	1	Unique								
6535	OSC61801.1	-	DUF461 domain-containing protein	1	1	1	1	1	1	1	1	1	1	1	11	CORE								
6536	WP_048456837.1	-	membrane protein	1	1	1	1	1	1	1	1	1	1	0	10	Accessory								
6537	WP_078504578.1	-	response regulator transcription factor	1	0	1	1	1	1	1	1	1	1	1	10	Accessory	COG2197_TK#	49.5	176	8.84E-44	K07693	45.9	169	2.88E-41
6538	Prokka:JAPCLKMA_05678	-	hypothetical protein	1	0	0	0	0	0	0	0	0	0	0	1	Unique								
6539	OSC72610.1	-	hypothetical protein	1	1	1	1	1	1	1	1	1	1	1	11	CORE								
6540	OSC72971.1	-	TetR family transcriptional regulator	1	1	1	1	1	1	1	1	1	1	1	11	CORE	COG1309_K#	30.2	65	2.2E-10	NA	35.7	50	0.0000195
6541	OSC72544.1	-	flagellar biosynthesis protein FlgA	0	1	0	0	1	0	0	0	0	0	1	3	Accessory								

cluster	GI	gene	Annotation	Streptomyces_thermocarboxydus_BPSAC147	Streptomyces_sp._4F	Streptomyces_sp._BSE7F	Streptomyces_sp._ETH9427	Streptomyces_sp._FxanaD5	Streptomyces_sp._UNC401CLCol	Actinospica_acidiphila_NRRL_B_24431	Streptomyces_sp._HNS054	Streptomyces_sp._SMS_SU21	Streptomyces_sp._ZS0098	Streptomyces_thermocarboxydus_K155	Addition	Core-Accessory-Unique	COG	%Similarity_COG	Score_COG	Evalue_COG	KEGG	%Similarity_KEGG	Score_KEGG	Evalue_KEGG
6542	OSC66317.1	-	permease	0	1	0	0	0	0	0	0	0	0	0	1	Unique	COG0730_R#	50.4	225	2.83E-58				
6543	WP_048458930.1	-	phosphatase PAP2 family protein	1	1	1	1	1	1	1	1	1	1	1	11	CORE	COG0671_I#	28.5	83	1.01E-15	K19302	28.5	83	2.71E-15
6544	WP_019527925.1	-	ester cyclase	1	0	1	1	1	1	0	1	1	1	1	9	Accessory	COG5485_R#	29.9	57	7.78E-08				
6545	WP_033273177.1	-	SDR family oxidoreductase	0	0	0	0	1	1	1	0	0	0	1	4	Accessory	COG1028_IQR#	48.1	217	4.5E-56	NA	42.9	103	2.53E-21
6546	Prokka:JAPCLKMA_04731	clpP2_2	ATP-dependent Clp protease proteolytic subunit 2	1	1	1	1	1	1	1	1	1	1	1	11	CORE	COG0740_OU#	69.5	282	1.94E-75	K01358	67.3	277	9.78E-74
6547	PWE08774.1	-	ABC transporter	0	0	1	1	0	0	1	1	1	1	0	6	Accessory	COG3910_R#	36.9	136	1.01E-31				
6548	OSC65823.1	-	GntR family transcriptional regulator	1	1	1	0	1	1	1	0	0	1	1	8	Accessory	COG2186_K#	35.6	125	2.33E-28	K13637	33.3	116	4.92E-25
6549	PWE08736.1	-	SAM-dependent methyltransferase	0	0	1	0	0	0	0	0	0	1	0	2	Accessory								
6550	WP_033276435.1	-	ribonuclease HI	1	1	1	1	1	1	1	1	1	1	1	11	CORE	COG0328_L#	37.2	103	1.24E-21	K03469	35.9	96	3.09E-19
6551	WP_019522580.1	-	hypothetical protein	1	0	1	1	1	1	1	1	1	1	1	10	Accessory	COG3959_G#	41	149	1.5E-35	K00615	34.6	108	7.86E-23
6552	OSC71821.1	-	hypothetical protein	1	1	1	1	1	1	1	1	1	1	1	11	CORE								

cluster	GI	gene	Annotation	Streptomyces_thermocarboxydus_BPSAC147	Streptomyces_sp._4F	Streptomyces_sp._BSE7F	Streptomyces_sp._ETH9427	Streptomyces_sp._FxanaD5	Streptomyces_sp._UNC401CLCol	Actinospica_acidiphila_NRRL_B_24431	Streptomyces_sp._HNS054	Streptomyces_sp._SMS_SU21	Streptomyces_sp._ZS0098	Streptomyces_thermocarboxydus_K155	Addition	Core-Accessory-Unique	COG	%Similarity_COG	Score_COG	Evalue_COG	KEGG	%Similarity_KEGG	Score_KEGG	Evalue_KEGG
6553	WP_033272503.1	-	NADH-quinone oxidoreductase subunit B	1	1	1	1	0	0	1	1	1	1	1	9	Accessory	COG0377_C#	58.7	177	5.16E-44	K03940	51.2	181	7.3E-45
6554	WP_033276830.1	-	ABC transporter ATP-binding protein	1	1	1	1	1	1	1	1	1	1	1	11	CORE	COG1136_V#	47.9	179	1.36E-44	K09810	47.9	179	3.63E-44
6555	PWE10715.1	-	TetR/AcrR family transcriptional regulator	1	0	1	1	1	1	1	1	1	1	1	10	Accessory								
6556	WP_019526477.1	-	hypothetical protein	1	1	0	1	1	1	0	0	0	0	1	6	Accessory	COG0431_R#	59.5	136	1.72E-31				
6557	WP_157842182.1	-	hypothetical protein	0	0	0	0	0	0	1	0	0	0	0	1	Unique								
6558	WP_109288790.1	-	protein rep	0	0	0	0	0	0	0	0	1	0	0	1	Unique	COG5655_L#	59.2	292	1.1E-78				
6559	Prokka:CIODPCDJ_02928	-	hypothetical protein	1	1	1	1	1	1	1	1	1	1	1	11	CORE	COG4339_S#	40.1	130	7.23E-30				
6560	WP_019526843.1	-	4-carboxy-4-hydroxy-2-oxoadipate aldolase/oxaloacetate decarboxylase	1	0	1	0	1	1	1	0	1	0	1	7	Accessory	COG0684_H#	64.2	285	1.34E-76	K08093	32.7	87	1.87E-16
6561	OSC73281.1	-	GntR family transcriptional regulator	0	1	0	0	0	0	0	0	0	0	0	1	Unique	COG2188_K#	32.3	124	3.97E-28	K03710	32.3	124	1.06E-27

cluster	GI	gene	Annotation	Streptomyces_thermocarboxydus_BPSAC147	Streptomyces_sp._4F	Streptomyces_sp._BSE7F	Streptomyces_sp._ETH9427	Streptomyces_sp._FxanaD5	Streptomyces_sp._UNC401CLCol	Actinospica_acidiphila_NRRL_B_24431	Streptomyces_sp._HNS054	Streptomyces_sp._SMS_SU21	Streptomyces_sp._ZS0098	Streptomyces_thermocarboxydus_K155	Addition	Core-Accessory-Unique	COG	%Similarity_COG	Score_COG	Evalue_COG	KEGG	%Similarity_KEGG	Score_KEGG	Evalue_KEGG
6562	AXI89603.1	-	HAD family hydrolase	0	0	0	1	0	0	0	0	0	0	0	1	Unique	COG0546_R#	31.2	83	1.32E-15	K06019	28.2	64	1.7E-09
6563	PWE11162.1	-	class F sortase	1	1	1	1	1	1	1	1	1	1	1	11	CORE								
6564	OSC64790.1	-	EamA family transporter	0	1	0	0	0	0	0	0	0	0	0	1	Unique	COG0697_GER#	33.9	111	3.48E-24	NA	33.9	111	9.28E-24
6565	OSC70867.1	-	coenzyme A pyrophosphatase	1	1	1	1	1	1	1	1	1	1	1	11	CORE	COG0494_LR#	41.8	164	3.46E-40	NA	41.8	164	9.24E-40
6566	PWE09074.1	-	2-oxo-4-hydroxy-4-carboxy-5-ureidoimidazoline decarboxylase	1	1	1	1	1	1	1	1	1	1	1	11	CORE								
6567	OSC70424.1	-	Flp pilus assembly protein CpaB	1	1	1	1	1	1	1	1	1	1	1	11	CORE								
6568	WP_122216109.1	-	endonuclease V	1	1	1	1	1	1	1	1	1	1	1	11	CORE	COG1515_L#	45.6	190	5.86E-48	K05982	38.4	145	5.78E-34
6569	AXI85819.1	-	hypothetical protein	1	1	0	1	1	1	1	1	0	1	1	9	Accessory	COG3786_S#	27.1	96	1.15E-19	NA	27.1	96	3.08E-19
6570	Prokka:JAPCLKMA_06408	-	hypothetical protein	1	0	0	0	0	0	0	0	0	0	0	1	Unique					NA	34.3	121	1.17E-26
6571	PWE07192.1	-	DUF4191 domain-containing protein	1	1	1	1	1	1	1	1	1	1	1	11	CORE					NA	43.3	166	2.42E-40
6572	Prokka:JAPCLKMA_01583	-	hypothetical protein	1	0	0	0	0	0	0	0	0	0	0	1	Unique					NA	29.9	107	2.28E-22

cluster	GI	gene	Annotation	Streptomyces_thermocarboxydus_BPSAC147	Streptomyces_sp._4F	Streptomyces_sp._BSE7F	Streptomyces_sp._ETH9427	Streptomyces_sp._FxanaD5	Streptomyces_sp._UNC401CLCol	Actinospica_acidiphila_NRRL_B_24431	Streptomyces_sp._HNS054	Streptomyces_sp._SMS_SU21	Streptomyces_sp._ZS0098	Streptomyces_thermocarboxydus_K155	Addition	Core-Accessory-Unique	COG	%Similarity_COG	Score_COG	Evalue_COG	KEGG	%Similarity_KEGG	Score_KEGG	Evalue_KEGG
6573	PWE07434.1	-	hypothetical protein	1	1	1	1	1	1	1	1	1	1	1	11	CORE								
6574	WP_019524940.1	-	hypothetical protein	0	0	0	0	1	0	0	0	0	0	0	1	Unique								
6575	WP_102639007.1	-	SDR family NAD(P)-dependent oxidoreductase	1	1	1	1	1	1	1	1	1	1	1	11	CORE	COG4221_R#	36.7	124	5.16E-28	NA	33.2	123	3.07E-27
6576	WP_146217839.1	-	YSIRK-type signal peptide-containing protein	0	0	0	0	0	0	0	0	1	0	0	1	Unique	COG0513_LKJ#	29.2	62	1.84E-09	NA	33.3	79	6.64E-14
6577	OSC73297.1	-	FkbM family methyltransferase	0	1	0	0	0	0	0	0	0	0	0	1	Unique	COG0500_QR#	34.5	77	5.54E-14	NA	29.9	44	0.00181
6578	WP_048458356.1	-	response regulator transcription factor	1	1	1	1	1	1	1	1	1	1	1	11	CORE	COG2197_TK#	60.5	260	7.87E-69	K07695	60.5	260	2.1E-68
6579	Prokka:JAPCLKMA_00772	gntR_2	putative D-xylose utilization operon transcriptional repressor	1	1	1	1	1	1	1	1	1	1	1	11	CORE	COG1802_K#	33.7	96	1.5E-19	NA	31.4	78	8.67E-14
6580	WP_122212776.1	-	DUF4255 domain-containing protein	0	0	0	1	0	0	0	1	1	1	0	4	Accessory								
6581	OSC73260.1	-	haloacid dehalogenase	0	1	0	0	0	0	0	0	0	0	0	1	Unique	COG0546_R#	30	56	1.32E-07				

cluster	GI	gene	Annotation	Streptomyces_thermocarboxydus_BPSAC147	Streptomyces_sp._4F	Streptomyces_sp._BSE7F	Streptomyces_sp._ETH9427	Streptomyces_sp._FxanaD5	Streptomyces_sp._UNC401CLCol	Actinospica_acidiphila_NRRL_B_24431	Streptomyces_sp._HNS054	Streptomyces_sp._SMS_SU21	Streptomyces_sp._ZS0098	Streptomyces_thermocarboxydus_K155	Addition	Core-Accessory-Unique	COG	%Similarity_COG	Score_COG	Evalue_COG	KEGG	%Similarity_KEGG	Score_KEGG	Evalue_KEGG
6582	PWE08580.1	-	nucleotidyltransferase	0	0	1	0	0	0	0	0	0	0	0	1	Unique								
6583	Prokka:JAPCLKMA_00187	-	hypothetical protein	1	1	1	0	1	1	1	1	1	1	1	10	Accessory	COG1188_J#	59	147	7.43E-35	K04762	40.3	83	3.52E-15
6584	PWE07396.1	-	hypothetical protein	1	1	1	1	1	1	1	1	1	1	1	11	CORE	COG2949_S#	40.4	137	7.69E-32	K03748	38.7	141	8.34E-33
6585	WP_109288757.1	-	hypothetical protein	1	0	1	1	1	1	1	1	1	1	1	10	Accessory					NA	38.6	86	5.43E-16
6586	OSC72314.1	-	TetR family transcriptional regulator	1	1	1	1	1	1	1	1	1	1	1	11	CORE	COG1309_K#	40.4	132	1.45E-30	NA	40.4	132	3.87E-30
6587	OSC66319.1	-	permease	0	1	0	0	0	0	0	0	0	0	0	1	Unique	COG2233_F#	52.5	236	7.13E-62	NA	39.2	159	2.96E-38
6588	WP_033273318.1	-	hypothetical protein	1	0	1	1	1	1	1	1	1	1	1	10	Accessory								
6589	Prokka:CIODPCDJ_06804	-	hypothetical protein	0	0	0	0	1	0	0	0	0	0	1	2	Accessory								
6590	OSC55280.1	-	hypothetical protein	0	1	0	0	0	0	0	0	0	0	0	1	Unique	COG3321_Q#	45.3	178	3.93E-44	K12430	45.3	178	1.05E-43
6591	Prokka:JAPCLKMA_04750	-	hypothetical protein	1	0	0	0	1	1	0	0	0	0	1	4	Accessory								
6592	Prokka:CIODPCDJ_02446	-	hypothetical protein	0	0	0	0	0	1	0	1	0	0	1	3	Accessory								
6593	OSC61048.1	-	ABC transporter permease	1	1	1	1	1	1	1	1	1	1	1	11	CORE	COG1174_E#	36.3	132	1.89E-30	K05846	36.3	132	5.06E-30
6594	WP_158688232.1	-	hypothetical protein	0	0	0	0	1	0	0	0	0	0	0	1	Unique								

cluster	GI	gene	Annotation	Streptomyces_thermocarboxydus_BPSAC147	Streptomyces_sp._4F	Streptomyces_sp._BSE7F	Streptomyces_sp._ETH9427	Streptomyces_sp._FxanaD5	Streptomyces_sp._UNC401CLCol	Actinospica_acidiphila_NRRL_B_24431	Streptomyces_sp._HNS054	Streptomyces_sp._SMS_SU21	Streptomyces_sp._ZS0098	Streptomyces_thermocarboxydus_K155	Addition	Core-Accessory-Unique	COG	%Similarity_COG	Score_COG	Evalue_COG	KEGG	%Similarity_KEGG	Score_KEGG	Evalue_KEGG
6595	OSC70202.1	-	nicotinic acid mononucleotide adenylyltransferase	1	1	1	1	1	1	1	1	1	1	1	11	CORE	COG1057_H#	68.5	278	2.14E-74	K00969	66.5	261	9.43E-69
6596	OSC71383.1	-	SAM-dependent methyltransferase	0	1	0	0	0	0	0	0	0	0	0	1	Unique	COG0500_QR#	24.2	68	4.39E-11	NA	24.2	68	1.17E-10
6597	OSC68289.1	-	hypothetical protein	0	1	0	0	0	0	0	0	0	0	0	1	Unique								
6598	Prokka:CIODPCDJ_01778	ctaE	Cytochrome c oxidase subunit 3	1	1	1	1	1	1	1	1	1	1	1	11	CORE	COG1845_C#	57.4	234	4.6E-61	K02276	57.4	234	1.23E-60
6599	OSC73099.1	-	antitermination regulator	0	1	0	0	0	0	0	0	0	0	0	1	Unique								
6600	OSC70179.1	-	ABC transporter ATP-binding protein	0	1	0	0	0	0	0	0	0	0	0	1	Unique	COG1132_V#	29.2	97	5.15E-20	K14698	34.1	104	1.13E-21
6601	Prokka:JAPCLKMA_06199	-	hypothetical protein	1	0	0	0	1	1	1	0	1	0	1	6	Accessory	COG3427_S#	41.7	132	2.46E-30	NA	41.8	103	3.27E-21
6602	AXI87642.1	phoU	phosphate transport system regulatory protein PhoU	1	1	1	1	1	1	1	1	1	1	1	11	CORE	COG0704_P#	51.4	209	1.21E-53	K02039	51.4	209	3.24E-53
6603	WP_028961280.1	-	hypothetical protein	0	0	0	1	1	1	0	0	0	0	0	3	Accessory								
6604	WP_033278068.1	-	hypothetical protein	0	0	0	0	0	0	1	0	0	0	0	1	Unique								

cluster	GI	gene	Annotation	Streptomyces_thermocarboxydus_BPSAC147	Streptomyces_sp._4F	Streptomyces_sp._BSE7F	Streptomyces_sp._ETH9427	Streptomyces_sp._FxanaD5	Streptomyces_sp._UNC401CLCol	Actinospica_acidiphila_NRRL_B_24431	Streptomyces_sp._HNS054	Streptomyces_sp._SMS_SU21	Streptomyces_sp._ZS0098	Streptomyces_thermocarboxydus_K155	Addition	Core-Accessory-Unique	COG	%Similarity_COG	Score_COG	Evalue_COG	KEGG	%Similarity_KEGG	Score_KEGG	Evalue_KEGG
6605	Prokka:JAPCLKMA_01028	-	hypothetical protein	1	0	0	0	1	1	0	0	0	0	0	3	Accessory								
6606	Prokka:CIODPCDJ_05352	-	hypothetical protein	1	1	1	1	1	1	1	1	1	1	1	11	CORE	COG2207_K#	35	115	2.39E-25				
6607	WP_019521734.1	-	peptidase E	1	0	0	0	1	1	1	0	1	1	1	7	Accessory	COG3340_E#	46.9	199	1.26E-50	NA	47.4	201	6.76E-51
6608	Prokka:CIODPCDJ_01248	gph_2	Phosphoglycolate phosphatase	1	1	1	1	1	1	1	1	1	1	1	11	CORE	COG0637_R#	34.8	115	3.12E-25	K00861	35.5	106	2.96E-22
6609	OSC69914.1	-	SAM-dependent methyltransferase	1	1	1	1	1	1	1	1	1	1	1	11	CORE	COG0500_QR#	31	99	2.31E-20	K06219	29.5	88	8.34E-17
6610	WP_019522732.1	-	FadR family transcriptional regulator	1	0	1	1	1	1	1	1	1	1	1	10	Accessory	COG2186_K#	39.2	146	1.65E-34				
6611	OSC70167.1	-	hypothetical protein	0	1	0	0	0	0	0	0	0	0	0	1	Unique								
6612	Prokka:JAPCLKMA_06823	-	hypothetical protein	1	0	0	0	0	0	0	0	0	0	0	1	Unique								
6613	OSC73318.1	-	two-component system response regulator	1	1	1	1	1	1	1	1	1	1	1	11	CORE	COG4565_KT#	38.5	145	2.81E-34	K11692	33.5	127	2.12E-28
6614	WP_033272739.1	-	hypothetical protein	0	0	1	1	1	1	1	1	1	1	1	9	Accessory								

cluster	GI	gene	Annotation	Streptomyces_thermocarboxydus_BPSAC147	Streptomyces_sp._4F	Streptomyces_sp._BSE7F	Streptomyces_sp._ETH9427	Streptomyces_sp._FxanaD5	Streptomyces_sp._UNC401CLCol	Actinospica_acidiphila_NRRL_B_24431	Streptomyces_sp._HNS054	Streptomyces_sp._SMS_SU21	Streptomyces_sp._ZS0098	Streptomyces_thermocarboxydus_K155	Addition	Core-Accessory-Unique	COG	%Similarity_COG	Score_COG	Evalue_COG	KEGG	%Similarity_KEGG	Score_KEGG	Evalue_KEGG
6615	Prokka:CIODPCDJ_00788	COQ5_4	2-methoxy-6-polyprenyl-1,4-benzoquinol methylase, mitochondrial	1	0	1	1	1	1	0	1	1	1	1	9	Accessory	COG0500_QR#	47.6	156	1.22E-37	NA	47.6	156	3.26E-37
6616	Prokka:CIODPCDJ_00851	-	putative membrane protein	1	1	1	1	1	1	1	1	1	1	1	11	CORE	COG0586_S#	55.3	248	3.08E-65	NA	55.3	248	8.23E-65
6617	WP_106432504.1	-	TnsA-like heteromeric transposase endonuclease subunit	0	0	0	0	1	0	0	0	0	0	0	1	Unique								
6618	Prokka:JAPCLKMA_01844	ung_1	Uracil-DNA glycosylase	1	1	1	1	1	1	1	1	1	1	1	11	CORE	COG0692_L#	59.7	277	3.63E-74	K03648	59.7	277	9.7E-74
6619	WP_033273025.1	-	hypothetical protein	1	1	1	1	1	1	1	1	1	1	1	11	CORE								
6620	WP_019526968.1	-	hypothetical protein	0	0	0	0	1	0	0	0	0	0	0	1	Unique								
6621	WP_033276216.1	-	ABC transporter ATP-binding protein	0	0	0	0	0	0	1	0	0	0	0	1	Unique	COG1136_V#	49.8	221	2.36E-57	K02003	50	220	2.4E-56
6622	OSC69015.1	-	GNAT family N-acetyltransferase	0	1	0	0	0	0	0	0	0	0	0	1	Unique	COG1670_J#	39.3	109	1.71E-23	NA	33.3	97	1.38E-19

cluster	GI	gene	Annotation	Streptomyces_thermocarboxydus_BPSAC147	Streptomyces_sp._4F	Streptomyces_sp._BSE7F	Streptomyces_sp._ETH9427	Streptomyces_sp._FxanaD5	Streptomyces_sp._UNC401CLCol	Actinospica_acidiphila_NRRL_B_24431	Streptomyces_sp._HNS054	Streptomyces_sp._SMS_SU21	Streptomyces_sp._ZS0098	Streptomyces_thermocarboxydus_K155	Addition	Core-Accessory-Unique	COG	%Similarity_COG	Score_COG	Evalue_COG	KEGG	%Similarity_KEGG	Score_KEGG	Evalue_KEGG
6623	WP_158688214.1	-	hypothetical protein	0	0	0	0	1	0	0	0	0	0	0	1	Unique								
6624	OSC64981.1	-	glyoxalase/bleomycin resistance/extradiol dioxygenase family protein	0	1	0	0	0	0	1	0	0	0	0	2	Accessory								
6625	WP_078895276.1	-	SDR family oxidoreductase	1	1	1	1	1	1	1	1	1	1	1	11	CORE	COG1028_IQR#	53.6	216	1.29E-55	NA	53.6	216	3.45E-55
6626	WP_051724594.1	-	hypothetical protein	1	0	0	0	0	1	1	0	0	0	1	4	Accessory								
6627	WP_078894975.1	-	O-methyltransferase	1	1	1	1	1	1	1	1	1	1	1	11	CORE	COG4122_R#	49.5	194	4.03E-49	NA	49.5	194	1.08E-48
6628	WP_102640908.1	-	SDR family oxidoreductase	1	0	1	1	1	1	1	1	1	1	1	10	Accessory	COG1028_IQR#	38.1	124	3.92E-28	K00540	35.6	97	1.37E-19
6629	Prokka:JAPCLKMA_02164	-	hypothetical protein	1	0	1	1	1	1	0	1	1	1	1	9	Accessory					NA	61.4	228	5.14E-59
6630	WP_159020976.1	-	AAA family ATPase	0	0	0	0	0	0	0	1	0	0	0	1	Unique					NA	84.1	406	1.8E-112
6631	Prokka:CIODPCDJ_03527	-	hypothetical protein	1	0	1	1	1	1	1	1	1	1	1	10	Accessory								
6632	Prokka:CIODPCDJ_04862	-	hypothetical protein	1	1	1	1	1	1	1	1	1	1	1	11	CORE					NA	30.4	48	0.000124
6633	Prokka:CIODPCDJ_03014	-	hypothetical protein	1	0	0	0	1	1	0	0	0	0	1	4	Accessory	COG0664_T#	25.5	79	1.89E-14	NA	23.9	64	2.19E-09
6634	Prokka:JAPCLKMA_00989	-	hypothetical protein	1	1	1	1	1	1	1	1	1	1	1	11	CORE	COG0406_G#	58.6	238	3.17E-62	NA	58.6	238	8.48E-62

cluster	GI	gene	Annotation	Streptomyces_thermocarboxydus_BPSAC147	Streptomyces_sp._4F	Streptomyces_sp._BSE7F	Streptomyces_sp._ETH9427	Streptomyces_sp._FxanaD5	Streptomyces_sp._UNC401CLCol	Actinospica_acidiphila_NRRL_B_24431	Streptomyces_sp._HNS054	Streptomyces_sp._SMS_SU21	Streptomyces_sp._ZS0098	Streptomyces_thermocarboxydus_K155	Addition	Core-Accessory-Unique	COG	%Similarity_COG	Score_COG	Evalue_COG	KEGG	%Similarity_KEGG	Score_KEGG	Evalue_KEGG
6635	OSC60209.1	-	GNAT family N-acetyltransferase	1	1	1	1	1	1	1	1	1	1	1	11	CORE	COG1670_J#	28.1	64	8.21E-10				
6636	Prokka:JAPCLKMA_02998	-	hypothetical protein	1	0	0	0	1	1	1	0	0	0	1	5	Accessory								
6637	Prokka:JAPCLKMA_02976	purN	Phosphoribosylglycinamide formyltransferase	1	1	1	1	1	1	1	1	1	1	1	11	CORE	COG0299_F#	58.7	226	1.25E-58	K11175	58.5	223	1.65E-57
6638	Prokka:JAPCLKMA_01813	-	hypothetical protein	1	0	0	0	0	0	0	0	0	0	1	2	Accessory								
6639	WP_033273085.1	-	class I SAM-dependent methyltransferase	1	0	1	1	1	1	1	1	1	1	1	10	Accessory	COG0500_QR#	25.5	53	0.00000145				
6640	WP_019527223.1	-	AAC(3) family N-acetyltransferase	1	0	0	0	1	1	0	0	0	0	1	4	Accessory	COG2746_V#	34.7	121	5.66E-27	K00662	36.2	101	1.24E-20
6641	AXI91402.1	-	hypothetical protein	0	0	0	1	0	0	0	0	0	0	0	1	Unique	COG1595_K#	33.8	89	2.38E-17	K03088	33.8	89	6.36E-17
6642	Prokka:JAPCLKMA_04818	-	hypothetical protein	1	0	1	1	1	1	1	1	1	1	1	10	Accessory	COG2860_S#	41.3	144	6.24E-34	NA	35.2	122	6.78E-27
6643	OSC63020.1	-	hypothetical protein	0	1	0	0	0	0	0	0	0	0	0	1	Unique								
6644	OSC69681.1	-	DNA methylase	0	1	0	0	0	0	0	0	0	0	0	1	Unique								
6645	OSC66115.1	-	hypothetical protein	1	1	1	1	1	1	1	1	1	1	1	11	CORE	COG3467_R#	51	208	2.69E-53				
6646	OSC70240.1	-	aminotransferase	0	1	0	0	0	0	0	0	0	0	0	1	Unique	COG0518_F#	36.8	117	6.25E-26	NA	31.5	84	1.2E-15

cluster	GI	gene	Annotation	Streptomyces_thermocarboxydus_BPSAC147	Streptomyces_sp._4F	Streptomyces_sp._BSE7F	Streptomyces_sp._ETH9427	Streptomyces_sp._FxanaD5	Streptomyces_sp._UNC401CLCol	Actinospica_acidiphila_NRRL_B_24431	Streptomyces_sp._HNS054	Streptomyces_sp._SMS_SU21	Streptomyces_sp._ZS0098	Streptomyces_thermocarboxydus_K155	Addition	Core-Accessory-Unique	COG	%Similarity_COG	Score_COG	Evalue_COG	KEGG	%Similarity_KEGG	Score_KEGG	Evalue_KEGG
6647	OSC63119.1	-	AraC family transcriptional regulator	0	1	0	0	0	0	0	0	0	0	0	1	Unique	COG2207_K#	34	76	1.6E-13	NA	32.1	48	0.0000953
6648	WP_158693608.1	-	helix-turn-helix domain-containing protein	0	0	0	0	0	1	0	0	0	0	0	1	Unique								
6649	OSC70334.1	-	hypothetical protein	0	1	0	0	0	0	0	0	0	0	0	1	Unique								
6650	OSC64141.1	-	spermidine synthase	1	1	1	1	1	1	1	1	1	1	1	11	CORE	COG0421_E#	37.9	125	2.3E-28	NA	39	121	1.51E-26
6651	PWE09543.1	-	hypothetical protein	0	0	1	0	0	0	0	0	1	0	0	2	Accessory								
6652	OSC72630.1	-	DUF4190 domain-containing protein	0	1	0	0	0	0	0	0	0	0	0	1	Unique								
6653	WP_102638513.1	-	GntR family transcriptional regulator	1	1	1	1	1	1	1	1	1	1	1	11	CORE	COG1802_K#	41.1	154	4.6E-37	NA	27.1	74	2.11E-12
6654	WP_019522339.1	-	DUF1707 and DUF2154 domain-containing protein	1	1	0	1	1	1	1	1	1	1	1	10	Accessory					NA	29.1	90	2.18E-17
6655	WP_051724604.1	-	hypothetical protein	0	0	0	0	0	0	1	0	0	0	0	1	Unique								
6656	OSC63422.1	-	cholesterol esterase	0	1	0	0	0	0	0	0	0	0	0	1	Unique								

cluster	GI	gene	Annotation	Streptomyces_thermocarboxydus_BPSAC147	Streptomyces_sp._4F	Streptomyces_sp._BSE7F	Streptomyces_sp._ETH9427	Streptomyces_sp._FxanaD5	Streptomyces_sp._UNC401CLCol	Actinospica_acidiphila_NRRL_B_24431	Streptomyces_sp._HNS054	Streptomyces_sp._SMS_SU21	Streptomyces_sp._ZS0098	Streptomyces_thermocarboxydus_K155	Addition	Core-Accessory-Unique	COG	%Similarity_COG	Score_COG	Evalue_COG	KEGG	%Similarity_KEGG	Score_KEGG	Evalue_KEGG
6657	AXI84686.1	-	4-phosphopantetheinyl transferase	0	0	0	1	0	0	0	1	0	0	0	2	Accessory	COG2091_H#	42.3	116	1.06E-25	K06133	27.4	64	2.18E-09
6658	WP_102638273.1	-	L,D-transpeptidase	1	0	1	1	1	1	1	1	1	1	1	10	Accessory	COG1376_S#	33.9	67	7.39E-11				
6659	Prokka:CIODPCDJ_03034	lldR_3	Putative L-lactate dehydrogenase operon regulatory protein	1	0	1	1	1	1	1	1	1	1	1	10	Accessory	COG2186_K#	35.4	116	1.06E-25	K05799	31.6	105	8.54E-22
6660	Prokka:JAPCLKMA_03070	afsQ1_1	Transcriptional regulatory protein AfsQ1	1	1	1	1	1	1	1	1	1	1	1	11	CORE	COG0745_TK#	46.6	208	2.68E-53	K07670	46.6	208	7.16E-53
6661	WP_078492802.1	-	TetR/AcrR family transcriptional regulator	1	1	1	1	1	1	1	1	1	1	1	11	CORE	COG1309_K#	34.2	92	2.8E-18	NA	34.1	89	6.33E-17
6662	OSC64030.1	-	hypothetical protein	0	1	0	0	0	0	0	0	0	0	0	1	Unique								
6663	OSC72244.1	-	hypothetical protein	0	1	0	0	0	0	0	0	0	0	0	1	Unique								
6664	OSC60510.1	-	GntR family transcriptional regulator	1	1	1	0	1	1	1	1	1	1	1	10	Accessory	COG1802_K#	27.5	97	8.71E-20	K13654	31.3	80	2.25E-14

cluster	GI	gene	Annotation	Streptomyces_thermocarboxydus_BPSAC147	Streptomyces_sp._4F	Streptomyces_sp._BSE7F	Streptomyces_sp._ETH9427	Streptomyces_sp._FxanaD5	Streptomyces_sp._UNC401CLCol	Actinospica_acidiphila_NRRL_B_24431	Streptomyces_sp._HNS054	Streptomyces_sp._SMS_SU21	Streptomyces_sp._ZS0098	Streptomyces_thermocarboxydus_K155	Addition	Core-Accessory-Unique	COG	%Similarity_COG	Score_COG	Evalue_COG	KEGG	%Similarity_KEGG	Score_KEGG	Evalue_KEGG
6665	WP_033273593.1	-	type 1 glutamine amidotransferase domain-containing protein	0	0	0	0	1	1	1	0	0	0	1	4	Accessory	COG0693_R#	43.5	165	1.99E-40	NA	39.2	97	2.33E-19
6666	WP_122213572.1	queC	7-cyano-7-deazaguanine synthase QueC	0	0	0	0	0	0	0	0	0	1	0	1	Unique	COG0603_R#	64.3	286	7.75E-77	K06920	51.1	237	1.44E-61
6667	WP_033274447.1	-	3-oxoacid CoA-transferase subunit A	0	0	0	0	0	0	1	0	0	0	0	1	Unique	COG1788_I#	63.8	293	8.28E-79	K01028	48.9	220	1.39E-56
6668	WP_048460855.1	-	HAD-IB family hydrolase	0	0	0	1	0	0	0	1	0	0	0	2	Accessory	COG0560_E#	32.2	74	4.63E-13				
6669	OSC67741.1	-	hypothetical protein	0	1	0	0	0	0	0	0	0	0	0	1	Unique								
6670	AXI87874.1	-	SMI1/KNR4 family protein	0	0	0	1	0	0	0	0	0	0	0	1	Unique								
6671	WP_019526992.1	-	hypothetical protein	0	0	0	0	1	0	0	0	0	0	0	1	Unique								
6672	WP_122215723.1	-	type 1 glutamine amidotransferase domain-containing protein	0	0	0	0	0	0	0	0	0	1	0	1	Unique	COG0693_R#	44.3	174	5.61E-43	NA	35.6	146	4.37E-34
6673	WP_048458568.1	-	restriction endonuclease	0	0	0	0	0	0	0	1	0	0	0	1	Unique								
6674	OSC69674.1	-	SpdA protein	1	1	1	0	1	1	0	1	1	0	1	8	Accessory	COG0515_RTKL#	38.2	50	0.00000935	K08884	38.2	50	0.000025

cluster	GI	gene	Annotation	Streptomyces_thermocarboxydus_BPSAC147	Streptomyces_sp._4F	Streptomyces_sp._BSE7F	Streptomyces_sp._ETH9427	Streptomyces_sp._FxanaD5	Streptomyces_sp._UNC401CLCol	Actinospica_acidiphila_NRRL_B_24431	Streptomyces_sp._HNS054	Streptomyces_sp._SMS_SU21	Streptomyces_sp._ZS0098	Streptomyces_thermocarboxydus_K155	Addition	Core-Accessory-Unique	COG	%Similarity_COG	Score_COG	Evalue_COG	KEGG	%Similarity_KEGG	Score_KEGG	Evalue_KEGG
6675	AXI91276.1	-	XRE family transcriptional regulator	0	0	0	1	0	0	0	0	0	0	0	1	Unique								
6676	WP_122214508.1	-	hypothetical protein	0	0	0	0	0	0	0	0	0	1	0	1	Unique								
6677	WP_028961307.1	pqqC	pyrroloquinoline-quinone synthase PqqC	0	0	0	0	1	1	0	0	0	0	0	2	Accessory	COG5424_H#	52.4	252	2.12E-66				
6678	PWE11028.1	ureG	urease accessory protein UreG	1	1	1	1	1	1	1	1	1	1	1	11	CORE	COG0378_OK#	64.7	265	1.42E-70	K03189	64.7	265	3.78E-70
6679	WP_102639937.1	-	hypothetical protein	0	0	0	0	0	0	0	0	1	0	0	1	Unique								
6680	Prokka:JAPCLKMA_01540	-	hypothetical protein	1	0	0	0	0	0	0	0	0	0	0	1	Unique								
6681	WP_048456124.1	urtE	urea ABC transporter ATP-binding subunit UrtE	1	0	1	1	1	1	1	1	1	1	1	10	Accessory	COG0410_E#	56.2	253	7.24E-67	K11963	46.2	193	2.37E-48
6682	AXI84616.1	-	hypothetical protein	0	1	1	1	0	0	1	1	1	1	0	7	Accessory								
6683	Prokka:CIODPCDJ_06475	lutR_4	HTH-type transcriptional regulator LutR	1	0	0	0	1	1	1	0	0	0	1	5	Accessory	COG1802_K#	33.2	109	2.21E-23	NA	31.4	92	5.7E-18
6684	WP_026243943.1	-	GAF and ANTAR domain-containing protein	1	1	1	1	1	1	1	1	1	1	1	11	CORE								

cluster	GI	gene	Annotation	Streptomyces_thermocarboxydus_BPSAC147	Streptomyces_sp._4F	Streptomyces_sp._BSE7F	Streptomyces_sp._ETH9427	Streptomyces_sp._FxanaD5	Streptomyces_sp._UNC401CLCol	Actinospica_acidiphila_NRRL_B_24431	Streptomyces_sp._HNS054	Streptomyces_sp._SMS_SU21	Streptomyces_sp._ZS0098	Streptomyces_thermocarboxydus_K155	Addition	Core-Accessory-Unique	COG	%Similarity_COG	Score_COG	Evalue_COG	KEGG	%Similarity_KEGG	Score_KEGG	Evalue_KEGG
6685	AXI87754.1	-	metal-dependent transcriptional regulator	1	1	1	1	1	1	1	1	1	1	1	11	CORE	COG1321_K#	62.5	258	2.94E-68	K03709	62.5	258	7.85E-68
6686	WP_019526368.1	-	ABC transporter ATP-binding protein	1	1	1	0	1	1	1	0	1	1	1	9	Accessory	COG1136_V#	51.1	220	6.79E-57	K16920	45.7	202	3.91E-51
6687	WP_019525995.1	-	hypothetical protein	1	0	1	1	1	1	1	1	1	1	1	10	Accessory								
6688	WP_020700151.1	-	hypothetical protein	0	1	1	1	1	1	1	1	1	1	1	10	Accessory								
6689	Prokka:CIODPCDJ_01402	lutR_2	HTH-type transcriptional regulator LutR	1	1	1	1	1	1	1	1	1	1	1	11	CORE	COG2186_K#	39.2	158	3.17E-38	K05799	31	99	4.67E-20
6690	OSC62972.1	-	ribosomal subunit interface protein	1	1	1	1	1	1	1	1	1	1	1	11	CORE	COG1544_J#	46.9	171	2.77E-42	NA	46.9	171	7.4E-42
6691	OSC57411.1	-	hypothetical protein	1	1	1	1	1	1	1	1	1	1	1	11	CORE								
6692	PWE11840.1	-	cytoplasmic protein	0	0	1	1	0	0	0	1	1	1	0	5	Accessory	COG3382_S#	48.2	186	8.32E-47	NA	37.3	136	3.44E-31
6693	OSC71684.1	-	di-trans,poly-cis-decaprenylcistransferase	0	1	0	0	0	0	0	0	0	0	0	1	Unique	COG0020_I#	45.7	210	7.03E-54	K12503	38.5	151	7.92E-36
6694	Prokka:CIODPCDJ_04146	vraR_2	Response regulator protein VraR	1	1	1	1	1	1	1	1	1	1	1	11	CORE	COG2197_TK#	43.8	167	5.22E-41	K02479	37.8	159	2.91E-38

cluster	GI	gene	Annotation	Streptomyces_thermocarboxydus_BPSAC147	Streptomyces_sp._4F	Streptomyces_sp._BSE7F	Streptomyces_sp._ETH9427	Streptomyces_sp._FxanaD5	Streptomyces_sp._UNC401CLCol	Actinospica_acidiphila_NRRL_B_24431	Streptomyces_sp._HNS054	Streptomyces_sp._SMS_SU21	Streptomyces_sp._ZS0098	Streptomyces_thermocarboxydus_K155	Addition	Core-Accessory-Unique	COG	%Similarity_COG	Score_COG	Evalue_COG	KEGG	%Similarity_KEGG	Score_KEGG	Evalue_KEGG
6695	OSC71785.1	-	hypothetical protein	0	1	0	0	0	0	0	0	0	1	0	2	Accessory								
6696	WP_019522662.1	-	response regulator transcription factor	0	1	1	1	1	1	1	1	1	1	1	10	Accessory	COG2197_TK#	42.8	170	8.06E-42	K02479	42.8	170	2.15E-41
6697	PWE07925.1	-	hypothetical protein	0	0	1	0	0	0	0	0	0	1	0	2	Accessory								
6698	WP_048459436.1	-	response regulator transcription factor	0	0	0	1	0	0	0	1	1	1	0	4	Accessory	COG2197_TK#	31.5	106	1.43E-22	NA	30.9	103	2.47E-21
6699	WP_051724484.1	-	hypothetical protein	0	0	1	0	0	0	1	0	1	0	0	3	Accessory								
6700	Prokka:CIODPCDJ_04261	degU_10	Transcriptional regulatory protein DegU	0	0	0	0	0	0	0	0	0	0	1	1	Unique	COG2197_TK#	29.8	80	6.43E-15	K07684	29.8	73	2.74E-12
6701	Prokka:CIODPCDJ_05130	-	hypothetical protein	1	0	1	1	1	1	1	1	1	1	1	10	Accessory	COG2120_S#	42.6	159	1.42E-38				
6702	WP_122216002.1	-	HAD hydrolase-like protein	0	0	0	0	0	0	0	0	0	1	0	1	Unique	COG0546_R#	28.7	64	8.14E-10				
6703	WP_051724584.1	-	response regulator transcription factor	1	1	1	1	1	1	1	1	1	1	1	11	CORE	COG2197_TK#	40.1	169	1.05E-41	NA	40.1	169	2.81E-41
6704	OSC52866.1	-	cellulose-binding protein	1	1	1	1	1	1	1	1	1	1	1	11	CORE	COG3397_S#	34.5	118	2.78E-26				
6705	AXI91387.1	-	hypothetical protein	0	0	0	1	0	0	0	0	0	0	0	1	Unique								
6706	AXI85313.1	-	hypothetical protein	1	0	0	1	0	0	0	0	0	0	0	2	Accessory								
6707	OSC72425.1	-	hypothetical protein	1	1	1	1	1	1	1	1	1	0	1	10	Accessory								

cluster	GI	gene	Annotation	Streptomyces_thermocarboxydus_BPSAC147	Streptomyces_sp._4F	Streptomyces_sp._BSE7F	Streptomyces_sp._ETH9427	Streptomyces_sp._FxanaD5	Streptomyces_sp._UNC401CLCol	Actinospica_acidiphila_NRRL_B_24431	Streptomyces_sp._HNS054	Streptomyces_sp._SMS_SU21	Streptomyces_sp._ZS0098	Streptomyces_thermocarboxydus_K155	Addition	Core-Accessory-Unique	COG	%Similarity_COG	Score_COG	Evalue_COG	KEGG	%Similarity_KEGG	Score_KEGG	Evalue_KEGG
6708	AXI85336.1	-	hypothetical protein	1	0	0	1	0	0	0	0	0	0	0	2	Accessory								
6709	OSC69133.1	-	hypothetical protein	0	1	0	0	0	0	0	0	0	0	0	1	Unique								
6710	Prokka:JAPCLKMA_05682	tam_6	Trans-aconitate 2-methyltransferase	1	1	0	0	0	0	0	0	0	0	0	2	Accessory	COG0500_QR#	29.3	52	0.00000188	K05928	29.3	52	0.00000501
6711	WP_146217813.1	-	hypothetical protein	0	0	0	0	0	0	0	0	1	0	0	1	Unique								
6712	OSC72091.1	-	peptidase M15	0	1	0	0	0	0	0	0	0	0	0	1	Unique	COG1876_M#	28.2	52	0.00000245	NA	28.2	52	0.00000654
6713	WP_033275680.1	-	GntR family transcriptional regulator	1	1	1	1	1	1	1	1	1	1	1	11	CORE	COG1802_K#	29.8	101	5.98E-21	NA	30.2	89	4.81E-17
6714	AXI86423.1	-	phenazine biosynthesis protein PhzC/PhzF	1	1	1	1	1	1	1	1	1	1	1	11	CORE	COG0384_R#	42.1	156	1.57E-37	NA	42.1	156	4.18E-37
6715	OSC71398.1	-	SAM-dependent methyltransferase	0	1	0	0	0	0	0	0	0	0	0	1	Unique	COG0500_QR#	30.3	77	9.25E-14	NA	35.6	67	1.96E-10
6716	WP_028960631.1	-	copper homeostasis protein CutC	1	1	0	1	1	1	1	1	1	1	1	10	Accessory	COG3142_P#	32.1	80	8.36E-15	K06201	30.3	85	6.94E-16
6717	Prokka:CIODPCDJ_06548	-	hypothetical protein	0	0	0	0	1	1	0	0	0	0	1	3	Accessory								

cluster	GI	gene	Annotation	Streptomyces_thermocarboxydus_BPSAC147	Streptomyces_sp._4F	Streptomyces_sp._BSE7F	Streptomyces_sp._ETH9427	Streptomyces_sp._FxanaD5	Streptomyces_sp._UNC401CLCol	Actinospica_acidiphila_NRRL_B_24431	Streptomyces_sp._HNS054	Streptomyces_sp._SMS_SU21	Streptomyces_sp._ZS0098	Streptomyces_thermocarboxydus_K155	Addition	Core-Accessory-Unique	COG	%Similarity_COG	Score_COG	Evalue_COG	KEGG	%Similarity_KEGG	Score_KEGG	Evalue_KEGG
6718	WP_006132401.1	ftsE	cell division ATP-binding protein FtsE	1	1	1	1	1	1	1	1	1	1	1	11	CORE	COG2884_D#	63.3	280	7.19E-75	K09812	62.4	278	5.59E-74
6719	WP_033274389.1	-	glutamine amidotransferase	0	1	1	1	0	0	1	1	1	1	0	7	Accessory	COG0693_R#	36.1	101	4.58E-21	NA	34.2	59	4.08E-08
6720	Prokka:CIODPCDJ_05211	-	hypothetical protein	0	0	0	0	0	0	0	0	0	0	1	1	Unique	COG1309_K#	29.3	77	9.25E-14				
6721	AXI89981.1	-	hypothetical protein	0	0	0	1	0	0	0	1	1	1	0	4	Accessory								
6722	OSC57344.1	-	hypothetical protein	1	1	1	1	1	1	1	1	1	1	1	11	CORE								
6723	WP_033273094.1	-	DUF899 domain-containing protein	1	1	0	1	1	1	1	0	0	0	1	7	Accessory	COG4312_S#	49.4	208	3.47E-53				
6724	WP_033273117.1	-	maleylpyruvate isomerase family mycothiol-dependent enzyme	1	1	1	1	1	1	1	1	1	1	1	11	CORE								
6725	OSC60973.1	-	AAA family ATPase	0	1	0	0	0	0	0	0	0	0	0	1	Unique	COG1511_S#	38.3	60	8.96E-09	NA	32.3	72	7.94E-12
6726	WP_122215173.1	-	TrkA family potassium uptake protein	0	0	1	1	0	0	0	1	1	1	0	5	Accessory	COG0569_P#	38.8	158	3.16E-38	K03499	36.2	152	4.62E-36

cluster	GI	gene	Annotation	Streptomyces_thermocarboxydus_BPSAC147	Streptomyces_sp._4F	Streptomyces_sp._BSE7F	Streptomyces_sp._ETH9427	Streptomyces_sp._FxanaD5	Streptomyces_sp._UNC401CLCol	Actinospica_acidiphila_NRRL_B_24431	Streptomyces_sp._HNS054	Streptomyces_sp._SMS_SU21	Streptomyces_sp._ZS0098	Streptomyces_thermocarboxydus_K155	Addition	Core-Accessory-Unique	COG	%Similarity_COG	Score_COG	Evalue_COG	KEGG	%Similarity_KEGG	Score_KEGG	Evalue_KEGG
6727	WP_102637519.1	-	ABC transporter ATP-binding protein	0	0	0	1	0	0	0	1	1	0	0	3	Accessory	COG1136_V#	53.4	216	1.28E-55	K02003	51.5	211	8.39E-54
6728	Prokka:JAPCLKMA_02419	-	hypothetical protein	1	1	1	1	1	1	1	1	1	1	1	11	CORE					NA	26.3	63	2.83E-09
6729	Prokka:CIODPCDJ_00897	svp_1	4'-phosphopantetheinyl transferase Svp	0	0	0	0	1	1	0	0	0	0	1	3	Accessory	COG2977_Q#	40.9	132	1.85E-30	NA	40.9	131	8.45E-30
6730	Prokka:JAPCLKMA_04907	yedK_1	SOS response-associated protein YedK	1	0	0	0	0	0	0	0	0	0	0	1	Unique	COG2135_S#	35.7	133	8.32E-31	NA	35.7	133	2.22E-30
6731	Prokka:CIODPCDJ_03909	degU_8	Transcriptional regulatory protein DegU	1	1	1	1	1	1	1	1	1	1	1	11	CORE	COG2197_TK#	35.1	112	1.52E-24	K07692	35.1	112	4.06E-24
6732	WP_028960572.1	-	hypothetical protein	0	0	0	0	0	1	0	0	0	0	0	1	Unique								
6733	AXI86447.1	-	DNA-binding response regulator	0	0	0	1	0	0	0	1	0	0	0	2	Accessory	COG0745_TK#	39.5	148	4.27E-35	K07776	39.5	148	1.14E-34
6734	WP_033274680.1	-	response regulator transcription factor	1	1	1	1	1	1	1	1	1	0	1	10	Accessory	COG2197_TK#	46.6	181	3.48E-45	K02479	41.1	168	8.14E-41
6735	OSC62003.1	-	hypothetical protein	0	1	0	0	0	0	0	0	0	0	0	1	Unique								
6736	OSC66751.1	-	hypothetical protein	0	1	0	0	0	0	0	0	0	0	0	1	Unique								

cluster	GI	gene	Annotation	Streptomyces_thermocarboxydus_BPSAC147	Streptomyces_sp._4F	Streptomyces_sp._BSE7F	Streptomyces_sp._ETH9427	Streptomyces_sp._FxanaD5	Streptomyces_sp._UNC401CLCol	Actinospica_acidiphila_NRRL_B_24431	Streptomyces_sp._HNS054	Streptomyces_sp._SMS_SU21	Streptomyces_sp._ZS0098	Streptomyces_thermocarboxydus_K155	Addition	Core-Accessory-Unique	COG	%Similarity_COG	Score_COG	Evalue_COG	KEGG	%Similarity_KEGG	Score_KEGG	Evalue_KEGG
6737	Prokka:JAPCLKMA_04885	-	hypothetical protein	1	0	0	0	0	0	0	0	0	0	0	1	Unique								
6738	Prokka:JAPCLKMA_04884	-	hypothetical protein	1	0	0	0	0	0	0	0	0	0	0	1	Unique								
6739	OSC64125.1	-	hypothetical protein	0	1	0	0	0	0	0	0	0	0	0	1	Unique					NA	53.8	67	2.56E-10
6740	OSC58622.1	-	glycosyl transferase	0	1	0	0	0	0	0	0	0	0	0	1	Unique	COG0438_M#	42.6	164	4.4E-40	NA	42.6	164	1.18E-39
6741	WP_048454912.1	-	methyltransferase	1	1	1	1	1	1	1	1	1	1	1	11	CORE	COG2890_J#	43	135	2.18E-31	K02493	32.8	110	2.62E-23
6742	AXI89096.1	-	DNA-binding response regulator	1	1	1	1	1	1	1	1	1	1	1	11	CORE	COG0745_TK#	43.3	180	5.91E-45	K07776	43.6	171	7.33E-42
6743	WP_033275114.1	rpe	ribulose-phosphate 3-epimerase	1	1	1	1	1	1	1	1	1	1	1	11	CORE	COG0036_G#	62.2	289	1.54E-77	K01783	62.2	289	4.12E-77
6744	OSC62353.1	-	GntR family transcriptional regulator	0	1	0	0	0	0	0	0	0	0	0	1	Unique	COG1802_K#	34.7	109	1.67E-23	NA	32.3	94	1.14E-18
6745	Prokka:CIODPCDJ_01025	-	hypothetical protein	1	1	1	1	1	1	1	1	1	1	1	11	CORE								
6746	PWE06691.1	-	gamma-glutamyl-gamma-aminobutyrate hydrolase	1	1	1	1	1	1	1	1	1	1	1	11	CORE	COG2071_R#	48.9	219	1.5E-56	K07010	48.9	219	4.01E-56
6747	AXI87301.1	snpA	snapalysin	1	0	1	1	1	1	1	1	1	1	1	10	Accessory								

cluster	GI	gene	Annotation	Streptomyces_thermocarboxydus_BPSAC147	Streptomyces_sp._4F	Streptomyces_sp._BSE7F	Streptomyces_sp._ETH9427	Streptomyces_sp._FxanaD5	Streptomyces_sp._UNC401CLCol	Actinospica_acidiphila_NRRL_B_24431	Streptomyces_sp._HNS054	Streptomyces_sp._SMS_SU21	Streptomyces_sp._ZS0098	Streptomyces_thermocarboxydus_K155	Addition	Core-Accessory-Unique	COG	%Similarity_COG	Score_COG	Evalue_COG	KEGG	%Similarity_KEGG	Score_KEGG	Evalue_KEGG
6748	WP_019522719.1	-	MSMEG_4193 family putative phosphomutase	1	1	1	1	1	1	1	1	1	1	1	11	CORE	COG0406_G#	50.4	226	7.19E-59	NA	50.4	226	1.92E-58
6749	OSC73478.1	-	MOSC domain-containing protein	1	1	1	1	1	1	1	1	1	1	1	11	CORE	COG2258_S#	56.3	223	1.04E-57	NA	56.3	223	2.77E-57
6750	WP_102638846.1	-	UbiX family flavin prenyltransferase	1	1	1	1	1	1	1	1	1	1	1	11	CORE	COG0163_H#	40.3	140	8.85E-33	K03186	40.3	140	2.36E-32
6751	WP_028960010.1	-	class I SAM-dependent methyltransferase	1	1	1	1	1	1	1	1	1	1	1	11	CORE	COG0500_QR#	32.8	99	1.33E-20	NA	33.5	63	2.81E-09
6752	OSC65693.1	-	aminocyclopropane-1-carboxylate deaminase/D-cysteine desulfhydrase family protein	0	1	0	0	0	0	0	0	0	0	0	1	Unique	COG2515_E#	66.8	303	6.05E-82	K05396	38.5	129	5.45E-29
6753	Prokka:JAPCLKMA_02821	-	hypothetical protein	1	1	0	1	1	1	1	1	1	1	1	10	Accessory	COG0789_K#	30.6	49	0.0000157	NA	30.6	49	0.000042
6754	WP_102639469.1	-	uracil-DNA glycosylase	0	0	0	0	0	0	0	0	1	1	0	2	Accessory					NA	42.2	139	5.27E-32

cluster	GI	gene	Annotation	Streptomyces_thermocarboxydus_BPSAC147	Streptomyces_sp._4F	Streptomyces_sp._BSE7F	Streptomyces_sp._ETH9427	Streptomyces_sp._FxanaD5	Streptomyces_sp._UNC401CLCol	Actinospica_acidiphila_NRRL_B_24431	Streptomyces_sp._HNS054	Streptomyces_sp._SMS_SU21	Streptomyces_sp._ZS0098	Streptomyces_thermocarboxydus_K155	Addition	Core-Accessory-Unique	COG	%Similarity_COG	Score_COG	Evalue_COG	KEGG	%Similarity_KEGG	Score_KEGG	Evalue_KEGG
6755	Prokka:JAPCLKMA_00618	rspR_1	HTH-type transcriptional repressor RspR	1	1	1	1	1	1	1	1	1	1	1	11	CORE	COG1802_K#	32.9	107	4.87E-23	NA	31.2	82	7.64E-15
6756	Prokka:JAPCLKMA_00118	-	hypothetical protein	1	0	0	0	1	1	1	0	0	0	1	5	Accessory	COG1309_K#	33.2	96	1.12E-19	NA	36	49	0.000042
6757	OSC55578.1	-	hypothetical protein	1	1	1	1	1	1	1	1	1	1	1	11	CORE					NA	29.1	64	1.65E-09
6758	AXI85294.1	-	methyltransferase	0	0	0	1	0	0	0	0	0	0	0	1	Unique								
6759	OSC72601.1	-	nucleotide exchange factor GrpE	1	1	1	1	1	1	1	1	1	1	1	11	CORE	COG0576_O#	37.4	129	1.2E-29	K03687	37.4	129	3.2E-29
6760	WP_019524115.1	-	hypothetical protein	0	0	0	0	1	0	0	0	0	0	0	1	Unique								
6761	WP_033274782.1	-	response regulator transcription factor	0	1	0	1	0	0	1	1	0	0	0	4	Accessory	COG2197_TK#	34.4	110	9.81E-24	NA	34.1	108	7.62E-23
6762	Prokka:JAPCLKMA_03472	ddpX	D-alanyl-D-alanine dipeptidase	1	1	0	1	1	0	0	0	0	0	0	4	Accessory	COG2173_M#	31	99	2.26E-20	K08641	31	99	6.04E-20
6763	WP_028961515.1	-	alpha/beta fold hydrolase	0	0	0	0	0	1	0	0	0	0	1	2	Accessory	COG0596_R#	27.6	103	1.2E-21	K13703	31.1	88	1.07E-16
6764	WP_078895242.1	-	MerR family transcriptional regulator	1	1	1	1	1	1	1	1	1	1	1	11	CORE	COG0789_K#	58.2	216	7.41E-56	NA	58.2	216	1.98E-55
6765	WP_102636974.1	-	glycerophosphodiester phosphodiesterase	1	1	1	1	1	1	1	1	1	1	1	11	CORE	COG0584_C#	33.2	132	2.4E-30	K01126	32.5	119	5.62E-26

cluster	GI	gene	Annotation	Streptomyces_thermocarboxydus_BPSAC147	Streptomyces_sp._4F	Streptomyces_sp._BSE7F	Streptomyces_sp._ETH9427	Streptomyces_sp._FxanaD5	Streptomyces_sp._UNC401CLCol	Actinospica_acidiphila_NRRL_B_24431	Streptomyces_sp._HNS054	Streptomyces_sp._SMS_SU21	Streptomyces_sp._ZS0098	Streptomyces_thermocarboxydus_K155	Addition	Core-Accessory-Unique	COG	%Similarity_COG	Score_COG	Evalue_COG	KEGG	%Similarity_KEGG	Score_KEGG	Evalue_KEGG
6766	OSC66186.1	-	hypothetical protein	1	1	1	0	1	1	1	1	1	1	1	10	Accessory								
6767	WP_033277149.1	-	phosphatase PAP2 family protein	1	0	1	1	1	1	1	1	1	1	1	10	Accessory								
6768	WP_122215989.1	-	phosphatase PAP2 family protein	1	1	0	0	1	1	1	1	0	1	1	8	Accessory	COG0671_I#	39.9	74	5.94E-13	NA	34.4	65	9.63E-10
6769	OSC68207.1	-	oxidase	1	1	1	1	1	1	1	1	1	1	1	11	CORE	COG0259_H#	41.7	149	1.9E-35	K00275	37.9	131	1.09E-29
6770	WP_109288795.1	-	phage tail protein	0	0	0	0	0	0	0	0	1	0	0	1	Unique					NA	67	286	2.66E-76
6771	Prokka:JAPCLKMA_05358	-	hypothetical protein	1	0	1	1	1	1	1	1	1	1	1	10	Accessory								
6772	PWE09814.1	-	DNA-binding response regulator	1	1	1	1	1	1	1	1	1	1	1	11	CORE	COG0745_TK#	63.5	280	5.46E-75	K07667	63.5	280	1.46E-74
6773	WP_033273264.1	-	PadR family transcriptional regulator	1	1	1	1	1	1	1	1	1	1	1	11	CORE	COG1695_K#	50	181	3.45E-45	NA	50	181	9.22E-45
6774	OSC68158.1	-	hypothetical protein	0	1	0	0	0	0	0	0	0	0	0	1	Unique								
6775	PWE09913.1	-	hypothetical protein	1	0	1	1	1	1	1	1	1	1	1	10	Accessory								
6776	WP_033272968.1	-	response regulator transcription factor	1	1	1	1	1	1	1	1	1	1	1	11	CORE	COG2197_TK#	38.7	151	2.93E-36	NA	39.9	147	1.47E-34
6777	Prokka:JAPCLKMA_06294	-	hypothetical protein	1	0	0	0	1	1	0	0	0	0	1	4	Accessory								

cluster	GI	gene	Annotation	Streptomyces_thermocarboxydus_BPSAC147	Streptomyces_sp._4F	Streptomyces_sp._BSE7F	Streptomyces_sp._ETH9427	Streptomyces_sp._FxanaD5	Streptomyces_sp._UNC401CLCol	Actinospica_acidiphila_NRRL_B_24431	Streptomyces_sp._HNS054	Streptomyces_sp._SMS_SU21	Streptomyces_sp._ZS0098	Streptomyces_thermocarboxydus_K155	Addition	Core-Accessory-Unique	COG	%Similarity_COG	Score_COG	Evalue_COG	KEGG	%Similarity_KEGG	Score_KEGG	Evalue_KEGG
6778	WP_019524795.1	-	SGNH/GDSL hydrolase family protein	1	0	0	0	1	1	0	0	0	0	1	4	Accessory								
6779	WP_051724495.1	-	TetR/AcrR family transcriptional regulator	0	1	1	0	1	1	1	0	1	0	1	7	Accessory	COG1309_K#	35.2	101	4.54E-21	NA	34.2	91	1.64E-17
6780	OSC73390.1	-	hypothetical protein	1	1	1	0	1	1	1	1	1	1	1	10	Accessory								
6781	WP_019521488.1	-	TetR/AcrR family transcriptional regulator	0	0	0	0	1	1	1	1	0	1	1	6	Accessory	COG1309_K#	29.7	75	2.67E-13	NA	29.7	75	7.12E-13
6782	OSC65104.1	-	hypothetical protein	0	1	0	0	0	0	0	0	0	0	0	1	Unique	COG0778_C#	36.7	145	2.74E-34	NA	36.8	135	7.57E-31
6783	OSC71304.1	-	translation initiation factor IF-2	0	1	0	0	0	0	0	0	0	0	0	1	Unique	COG0532_J#	40.2	163	1.27E-39	K02519	42.7	145	7.32E-34
6784	OSC71873.1	-	N-acetylmuramoyl-L-alanine amidase	0	1	0	0	0	0	0	0	0	0	0	1	Unique								
6785	OSC73028.1	-	hypothetical protein	1	1	1	1	1	1	1	1	1	1	1	11	CORE								
6786	OSC63421.1	-	Tat pathway signal sequence domain protein	0	1	0	0	0	0	0	0	0	0	0	1	Unique								
6787	WP_026243695.1	-	response regulator transcription factor	1	1	1	1	1	1	1	1	1	1	1	11	CORE	COG0745_TK#	75.2	338	1.68E-92	K07776	75.2	338	4.49E-92

cluster	GI	gene	Annotation	Streptomyces_thermocarboxydus_BPSAC147	Streptomyces_sp._4F	Streptomyces_sp._BSE7F	Streptomyces_sp._ETH9427	Streptomyces_sp._FxanaD5	Streptomyces_sp._UNC401CLCol	Actinospica_acidiphila_NRRL_B_24431	Streptomyces_sp._HNS054	Streptomyces_sp._SMS_SU21	Streptomyces_sp._ZS0098	Streptomyces_thermocarboxydus_K155	Addition	Core-Accessory-Unique	COG	%Similarity_COG	Score_COG	Evalue_COG	KEGG	%Similarity_KEGG	Score_KEGG	Evalue_KEGG
6788	WP_019524092.1	-	low molecular weight phosphatase family protein	1	1	1	1	1	1	1	1	1	1	1	11	CORE								
6789	WP_121721039.1	-	hyaluronoglucosaminidase	0	0	0	0	0	0	0	0	0	1	0	1	Unique								
6790	WP_019523578.1	-	TetR/AcrR family transcriptional regulator	1	1	0	0	1	1	1	0	0	0	1	6	Accessory	COG1309_K#	33.8	67	9.44E-11	NA	25.7	62	8.11E-09
6791	Prokka:JAPCLKMA_03945	-	hypothetical protein	1	1	1	1	1	1	1	1	1	1	1	11	CORE								
6792	Prokka:JAPCLKMA_00783	citT_1	Transcriptional regulatory protein CitT	1	1	1	1	1	1	1	1	1	1	1	11	CORE	COG4565_KT#	39.7	150	8.48E-36	K11692	33.8	140	1.79E-32
6793	Prokka:CIODPCDJ_03138	purQ	Phosphoribosylformylglycinamidine synthase subunit PurQ	1	1	1	1	1	1	1	1	1	1	1	11	CORE	COG0047_F#	64.2	281	2.44E-75	K01952	64.2	281	6.51E-75
6794	WP_102638386.1	-	response regulator transcription factor	0	1	0	0	0	0	0	0	1	0	0	2	Accessory	COG2197_TK#	39.3	153	1.31E-36	K02479	39.3	153	3.49E-36
6795	WP_122217387.1	-	RloB domain-containing protein	0	0	0	0	0	0	0	0	0	1	0	1	Unique								

cluster	GI	gene	Annotation	Streptomyces_thermocarboxydus_BPSAC147	Streptomyces_sp._4F	Streptomyces_sp._BSE7F	Streptomyces_sp._ETH9427	Streptomyces_sp._FxanaD5	Streptomyces_sp._UNC401CLCol	Actinospica_acidiphila_NRRL_B_24431	Streptomyces_sp._HNS054	Streptomyces_sp._SMS_SU21	Streptomyces_sp._ZS0098	Streptomyces_thermocarboxydus_K155	Addition	Core-Accessory-Unique	COG	%Similarity_COG	Score_COG	Evalue_COG	KEGG	%Similarity_KEGG	Score_KEGG	Evalue_KEGG
6796	WP_026243367.1	-	response regulator transcription factor	1	1	1	1	1	1	1	1	1	1	1	11	CORE	COG2197_TK#	44	165	2.55E-40	NA	40.2	155	7.04E-37
6797	WP_048455718.1	-	AIM24 family protein	1	1	1	1	1	1	1	1	1	1	1	11	CORE	COG2013_S#	35	130	9.08E-30	NA	35	130	2.43E-29
6798	AXI89646.1	-	hypothetical protein	0	0	0	1	0	0	0	0	0	0	0	1	Unique								
6799	OSC70135.1	-	hypothetical protein	0	1	0	0	0	0	0	0	0	0	0	1	Unique								
6800	OSC61300.1	-	class F sortase	1	1	1	1	1	1	1	1	1	1	1	11	CORE								
6801	PWE07142.1	-	hypothetical protein	1	1	1	1	1	1	1	1	1	1	1	11	CORE								
6802	Prokka:CIODPCDJ_01719	-	hypothetical protein	1	1	1	1	1	1	1	1	1	1	1	11	CORE	COG0737_F#	29.5	49	0.0000266	NA	32.9	89	4.74E-17
6803	OSC71608.1	-	4'-phosphopantetheinyl transferase	1	1	1	1	1	1	1	1	1	1	1	11	CORE	COG2977_Q#	46	200	7.15E-51	NA	46	200	1.91E-50
6804	OSC61658.1	-	TetR family transcriptional regulator	0	1	0	0	0	0	0	0	0	0	0	1	Unique	COG1309_K#	31.9	89	2.32E-17	NA	31	59	4.03E-08
6805	Prokka:CIODPCDJ_06412	-	hypothetical protein	1	0	1	1	0	1	0	1	0	1	1	7	Accessory								
6806	OSC72287.1	-	hypothetical protein	1	1	1	1	1	1	1	1	1	1	1	11	CORE								
6807	WP_102637230.1	-	class F sortase	0	0	1	0	0	0	0	1	1	1	0	4	Accessory								
6808	WP_157842173.1	-	hypothetical protein	0	0	0	0	0	0	1	0	0	0	0	1	Unique								

cluster	GI	gene	Annotation	Streptomyces_thermocarboxydus_BPSAC147	Streptomyces_sp._4F	Streptomyces_sp._BSE7F	Streptomyces_sp._ETH9427	Streptomyces_sp._FxanaD5	Streptomyces_sp._UNC401CLCol	Actinospica_acidiphila_NRRL_B_24431	Streptomyces_sp._HNS054	Streptomyces_sp._SMS_SU21	Streptomyces_sp._ZS0098	Streptomyces_thermocarboxydus_K155	Addition	Core-Accessory-Unique	COG	%Similarity_COG	Score_COG	Evalue_COG	KEGG	%Similarity_KEGG	Score_KEGG	Evalue_KEGG
6809	WP_028959754.1	-	O-methyltransferase	1	1	1	1	1	1	1	1	1	1	1	11	CORE	COG4122_R#	59	254	3.18E-67	NA	60.3	234	1.19E-60
6810	WP_033273695.1	-	GntR family transcriptional regulator	1	1	1	1	1	1	1	1	1	1	1	11	CORE	COG1802_K#	33	110	9.68E-24	NA	33.5	101	1.2E-20
6811	Prokka:CIODPCDJ_01519	-	hypothetical protein	1	0	0	0	1	1	0	0	0	0	1	4	Accessory	COG3427_S#	40.1	127	7.65E-29	NA	40.5	98	7.79E-20
6812	WP_106959439.1	-	DegT/DnrJ/EryC1/StrS aminotransferase	1	1	1	1	1	1	1	1	1	1	1	11	CORE	COG0399_M#	31.2	88	3.94E-17	K13010	43.4	84	1.52E-15
6813	WP_159020879.1	-	helix-turn-helix domain containing protein	0	0	0	0	0	0	0	1	0	0	0	1	Unique								
6814	Prokka:JAPCLKMA_01785	-	hypothetical protein	1	1	1	1	1	1	1	1	1	1	1	11	CORE								
6815	Prokka:JAPCLKMA_04120	regX3_3	Sensory transduction protein regX3	1	1	1	1	1	1	1	1	1	1	1	11	CORE	COG0745_TK#	47.4	215	2.79E-55	K07776	48	211	1.08E-53
6816	OSC61495.1	-	hypothetical protein	0	1	0	0	0	0	0	0	0	0	0	1	Unique	COG3391_S#	30.2	41	0.00423	K10939	39.4	49	0.0000542
6817	WP_048456288.1	-	response regulator	1	1	1	1	1	1	1	1	1	1	1	11	CORE	COG0784_T#	59.9	159	1.39E-38	K11527	36.5	95	8.61E-19
6818	Prokka:CIODPCDJ_00444	-	hypothetical protein	1	1	1	1	1	1	1	1	1	1	1	11	CORE	COG2013_S#	37.6	143	1.35E-33	NA	37.6	143	3.6E-33
6819	WP_019522061.1	-	response regulator transcription factor	1	0	1	1	1	1	1	1	1	0	1	9	Accessory	COG2197_TK#	46.4	177	4.94E-44	NA	42.2	172	5.54E-42

cluster	GI	gene	Annotation	Streptomyces_thermocarboxydus_BPSAC147	Streptomyces_sp._4F	Streptomyces_sp._BSE7F	Streptomyces_sp._ETH9427	Streptomyces_sp._FxanaD5	Streptomyces_sp._UNC401CLCol	Actinospica_acidiphila_NRRL_B_24431	Streptomyces_sp._HNS054	Streptomyces_sp._SMS_SU21	Streptomyces_sp._ZS0098	Streptomyces_thermocarboxydus_K155	Addition	Core-Accessory-Unique	COG	%Similarity_COG	Score_COG	Evalue_COG	KEGG	%Similarity_KEGG	Score_KEGG	Evalue_KEGG
6820	WP_122216778.1	-	N-acetylmuramoyl-L-alanine amidase	1	0	0	0	0	0	0	0	1	1	0	3	Accessory								
6821	WP_122216259.1	-	GAP family protein	0	0	1	0	0	0	0	0	1	1	0	3	Accessory								
6822	WP_157842215.1	-	hypothetical protein	0	0	0	0	0	0	1	0	0	0	0	1	Unique								
6823	OSC62143.1	-	potassium transporter TrkA	1	1	1	1	1	1	1	1	1	1	1	11	CORE	COG0569_P#	58.3	265	1.8E-70	K03499	58.3	265	4.81E-70
6824	OSC71397.1	-	hypothetical protein	0	1	0	0	0	0	0	0	0	0	0	1	Unique								
6825	AXI86443.1	-	TetR/AcrR family transcriptional regulator	0	0	0	1	0	0	0	1	0	0	0	2	Accessory	COG1309_K#	38.4	69	1.89E-11	NA	35.2	59	6.84E-08
6826	Prokka:CIODPCDJ_04831	-	hypothetical protein	1	1	1	1	1	1	1	1	1	1	1	11	CORE								
6827	WP_122214854.1	-	hypothetical protein	0	0	1	0	0	0	1	1	0	1	0	4	Accessory								
6828	Prokka:CIODPCDJ_02661	-	hypothetical protein	0	0	0	0	1	1	1	0	0	0	1	4	Accessory	COG1309_K#	32.1	86	1.5E-16	NA	32.1	86	4E-16
6829	Prokka:CIODPCDJ_04310	-	hypothetical protein	1	0	0	0	1	1	0	0	0	0	1	4	Accessory	COG1674_D#	40.2	67	9.4E-11	NA	38.1	81	1.29E-14
6830	AXI91233.1	-	GGDEF domain-containing protein	0	0	0	1	0	0	0	0	0	0	0	1	Unique	COG2199_T#	41.3	113	8.76E-25	NA	39.7	101	1.2E-20
6831	WP_028960602.1	-	hypothetical protein	0	0	0	0	0	1	0	0	0	0	0	1	Unique								

cluster	GI	gene	Annotation	Streptomyces_thermocarboxydus_BPSAC147	Streptomyces_sp._4F	Streptomyces_sp._BSE7F	Streptomyces_sp._ETH9427	Streptomyces_sp._FxanaD5	Streptomyces_sp._UNC401CLCol	Actinospica_acidiphila_NRRL_B_24431	Streptomyces_sp._HNS054	Streptomyces_sp._SMS_SU21	Streptomyces_sp._ZS0098	Streptomyces_thermocarboxydus_K155	Addition	Core-Accessory-Unique	COG	%Similarity_COG	Score_COG	Evalue_COG	KEGG	%Similarity_KEGG	Score_KEGG	Evalue_KEGG
6832	PWE09793.1	-	2-dehydro-3-deoxyphosphogluconate aldolase	0	0	1	0	0	0	0	0	0	0	0	1	Unique	COG0800_G#	36.4	139	1.14E-32	K01625	39	132	6.35E-30
6833	Prokka:JAPCLKMA_03951	-	hypothetical protein	1	1	1	1	1	1	1	1	1	1	1	11	CORE					K00934	30.2	48	0.000121
6834	OSC60962.1	-	hypothetical protein	0	1	0	0	0	0	0	0	0	0	0	1	Unique	COG1357_S#	27.7	68	4.22E-11	NA	29.2	67	1.47E-10
6835	WP_086020134.1	-	transposase	0	0	0	0	1	1	0	0	0	0	0	2	Accessory								
6836	WP_109288793.1	-	HlyC/CorC family transporter	0	0	0	0	0	0	0	0	1	0	0	1	Unique	COG1253_R#	63.2	282	1.09E-75	NA	63.2	282	2.91E-75
6837	WP_033274202.1	-	GntR family transcriptional regulator	0	1	0	0	0	0	1	0	0	0	0	2	Accessory	COG1802_K#	28.2	105	2.39E-22	K11476	29	89	6.17E-17
6838	WP_122213818.1	-	phage prohead protease	0	0	0	0	0	0	0	0	0	1	0	1	Unique								
6839	OSC71199.1	-	sulfatase modifying factor 1 (C-alpha-formyglycine-generating enzyme 1)	0	1	0	0	0	0	0	0	0	0	0	1	Unique	COG1262_S#	48.7	217	4.31E-56	K13444	57.1	277	1.22E-73
6840	OSC70421.1	-	Crp/Fnr family transcriptional regulator	1	1	1	1	1	1	1	1	1	1	1	11	CORE	COG0664_T#	55.4	265	1.37E-70	NA	54.9	264	8.17E-70

cluster	GI	gene	Annotation	Streptomyces_thermocarboxydus_BPSAC147	Streptomyces_sp._4F	Streptomyces_sp._BSE7F	Streptomyces_sp._ETH9427	Streptomyces_sp._FxanaD5	Streptomyces_sp._UNC401CLCol	Actinospica_acidiphila_NRRL_B_24431	Streptomyces_sp._HNS054	Streptomyces_sp._SMS_SU21	Streptomyces_sp._ZS0098	Streptomyces_thermocarboxydus_K155	Addition	Core-Accessory-Unique	COG	%Similarity_COG	Score_COG	Evalue_COG	KEGG	%Similarity_KEGG	Score_KEGG	Evalue_KEGG
6841	WP_019526577.1	-	HAD family hydrolase	1	1	1	1	1	1	1	1	1	1	1	11	CORE	COG0546_R#	31.6	105	3.1E-22	NA	31.6	104	1.41E-21
6842	OSC57298.1	-	helix-turn-helix transcriptional regulator	1	1	1	1	1	1	1	1	1	1	1	11	CORE	COG2197_TK#	35.6	111	5.65E-24	K07684	35.6	111	1.51E-23
6843	WP_086702697.1	-	TetR/AcrR family transcriptional regulator	1	1	1	1	1	1	1	1	1	1	1	11	CORE	COG1309_K#	64.5	257	3.74E-68	NA	64.4	253	1.44E-66
6844	WP_048458774.1	-	CBS domain-containing protein	0	0	0	1	0	0	0	1	1	1	0	4	Accessory	COG0517_R#	35.4	95	3.21E-19	NA	36.2	94	1.12E-18
6845	Prokka:JAPCLKMA_01943	ureF	Urease accessory protein UreF	1	0	1	1	1	1	1	1	1	1	1	10	Accessory	COG0830_O#	40.3	140	6.66E-33	K03188	40.3	140	1.78E-32
6846	WP_109288596.1	-	peptidoglycan-binding protein	1	0	1	0	1	1	1	1	1	1	1	9	Accessory								
6847	WP_048460809.1	-	RNA polymerase sigma factor	1	1	1	1	1	1	1	1	1	1	1	11	CORE	COG1595_K#	25.7	50	0.0000118				
6848	WP_033273927.1	-	restriction endonuclease	0	0	0	0	0	0	1	1	0	1	0	3	Accessory								
6849	WP_106963208.1	-	type VII secretion system-associated protein	0	0	0	1	1	1	1	1	1	1	1	8	Accessory								
6850	WP_033272798.1	-	hypothetical protein	1	1	1	1	1	1	1	1	1	1	1	11	CORE								

cluster	GI	gene	Annotation	Streptomyces_thermocarboxydus_BPSAC147	Streptomyces_sp._4F	Streptomyces_sp._BSE7F	Streptomyces_sp._ETH9427	Streptomyces_sp._FxanaD5	Streptomyces_sp._UNC401CLCol	Actinospica_acidiphila_NRRL_B_24431	Streptomyces_sp._HNS054	Streptomyces_sp._SMS_SU21	Streptomyces_sp._ZS0098	Streptomyces_thermocarboxydus_K155	Addition	Core-Accessory-Unique	COG	%Similarity_COG	Score_COG	Evalue_COG	KEGG	%Similarity_KEGG	Score_KEGG	Evalue_KEGG
6851	WP_102641338.1	-	1-acyl-sn-glycerol-3-phosphate acyltransferase	1	1	1	1	1	1	1	1	1	1	1	11	CORE	COG0204_I#	37.1	94	5.47E-19	NA	33.9	92	7.26E-18
6852	Prokka:JAPCLKMA_04059	-	hypothetical protein	1	1	0	0	1	1	1	0	0	0	1	6	Accessory								
6853	WP_028959401.1	-	hypothetical protein	1	0	1	1	1	1	1	1	1	1	1	10	Accessory								
6854	PWE09934.1	-	DUF3000 domain-containing protein	1	1	1	1	1	1	1	1	1	1	1	11	CORE					NA	46.2	159	2.83E-38
6855	WP_028959544.1	-	hypothetical protein	0	0	0	0	1	1	0	0	0	0	0	2	Accessory								
6856	OSC53111.1	-	hypothetical protein	0	1	0	0	0	0	0	0	0	0	0	1	Unique	COG0500_QR#	39.4	110	9.64E-24				
6857	Prokka:JAPCLKMA_01045	-	hypothetical protein	1	0	0	0	0	0	0	0	0	0	0	1	Unique								
6858	AXI88856.1	-	DNA-binding response regulator	1	1	1	1	1	1	1	0	1	1	1	10	Accessory	COG2197_TK#	52.3	192	1.48E-48	NA	47.1	182	4.08E-45
6859	PWE10913.1	-	TetR family transcriptional regulator	0	0	1	0	1	1	1	0	1	0	1	6	Accessory	COG1309_K#	27.1	68	4.2E-11	NA	27.1	68	1.12E-10
6860	PWE08785.1	-	redoxin domain-containing protein	1	1	1	1	1	1	1	1	1	1	1	11	CORE	COG0526_OC#	39.2	128	3.42E-29	NA	39.2	128	9.14E-29
6861	WP_028961443.1	-	hypothetical protein	1	0	1	1	1	1	1	1	1	1	1	10	Accessory	COG3064_M#	31.3	62	3.01E-09	K03646	31.3	62	8.04E-09

cluster	GI	gene	Annotation	Streptomyces_thermocarboxydus_BPSAC147	Streptomyces_sp._4F	Streptomyces_sp._BSE7F	Streptomyces_sp._ETH9427	Streptomyces_sp._FxanaD5	Streptomyces_sp._UNC401CLCol	Actinospica_acidiphila_NRRL_B_24431	Streptomyces_sp._HNS054	Streptomyces_sp._SMS_SU21	Streptomyces_sp._ZS0098	Streptomyces_thermocarboxydus_K155	Addition	Core-Accessory-Unique	COG	%Similarity_COG	Score_COG	Evalue_COG	KEGG	%Similarity_KEGG	Score_KEGG	Evalue_KEGG
6862	WP_033273790.1	-	putative N-acetylmannosamine-6-phosphate 2-epimerase	0	0	0	0	0	0	1	0	0	0	0	1	Unique	COG3010_G#	45.5	169	1.34E-41	K01788	44.9	142	6.11E-33
6863	WP_033277297.1	-	hypothetical protein	0	0	0	0	0	0	1	0	0	0	0	1	Unique								
6864	WP_026243770.1	casB	type I-E CRISPR-associated protein Cse2/CasB	0	0	0	0	1	0	0	0	0	0	0	1	Unique								
6865	WP_102639935.1	-	response regulator transcription factor	0	0	0	0	0	0	0	0	1	0	0	1	Unique	COG2197_TK#	42.3	154	4.46E-37	NA	42.3	154	1.19E-36
6866	AXI90054.1	-	succinate dehydrogenase	1	1	1	1	1	1	1	1	1	1	1	11	CORE					K00241	26.8	82	5.72E-15
6867	WP_102640761.1	-	GntR family transcriptional regulator	1	0	1	0	1	1	1	0	1	0	1	7	Accessory	COG1802_K#	32.1	101	4.46E-21	NA	26.3	77	1.41E-13
6868	AXI87474.1	-	hypothetical protein	0	0	0	1	0	0	0	1	0	0	0	2	Accessory								
6869	OSC72608.1	-	GNAT family N-acetyltransferase	1	1	1	1	1	1	1	1	1	1	1	11	CORE	COG0454_KR#	31.5	109	2.14E-23	NA	32.2	89	4.68E-17
6870	Prokka:JAPCLKMA_01589	-	hypothetical protein	1	1	1	1	1	1	1	1	1	1	1	11	CORE	COG3544_S#	36.3	50	0.00000902	NA	36.3	50	0.0000241

cluster	GI	gene	Annotation	Streptomyces_thermocarboxydus_BPSAC147	Streptomyces_sp._4F	Streptomyces_sp._BSE7F	Streptomyces_sp._ETH9427	Streptomyces_sp._FxanaD5	Streptomyces_sp._UNC401CLCol	Actinospica_acidiphila_NRRL_B_24431	Streptomyces_sp._HNS054	Streptomyces_sp._SMS_SU21	Streptomyces_sp._ZS0098	Streptomyces_thermocarboxydus_K155	Addition	Core-Accessory-Unique	COG	%Similarity_COG	Score_COG	Evalue_COG	KEGG	%Similarity_KEGG	Score_KEGG	Evalue_KEGG
6871	WP_019526374.1	-	TrkA family potassium uptake protein	1	1	1	1	1	1	1	1	1	1	1	11	CORE	COG0569_P#	53.6	238	2.34E-62	K03499	53.2	236	1.82E-61
6872	OSC69596.1	-	hypothetical protein	1	1	1	0	1	1	1	1	1	1	1	10	Accessory								
6873	Prokka:JAPCLKMA_06115	-	hypothetical protein	1	1	1	1	1	1	1	1	1	1	1	11	CORE	COG1309_K#	49.7	158	4.01E-38	NA	49.7	158	1.07E-37
6874	WP_053076908.1	-	DUF4232 domain-containing protein	0	0	0	1	0	0	0	1	0	0	0	2	Accessory								
6875	Prokka:JAPCLKMA_01618	-	hypothetical protein	1	0	0	0	0	0	0	0	0	0	0	1	Unique								
6876	AXI85296.1	-	thymidylate kinase	0	0	0	1	0	0	0	0	0	0	0	1	Unique								
6877	WP_048456180.1	-	response regulator transcription factor	0	1	0	0	0	0	0	1	0	0	0	2	Accessory	COG2197_TK#	39.3	157	6.85E-38	K02479	39.3	157	1.83E-37
6878	OSC70199.1	-	histidine phosphatase family protein	1	1	1	1	1	1	1	1	1	1	1	11	CORE	COG0406_G#	41.3	134	3.64E-31	K15634	41.3	134	9.72E-31
6879	Prokka:CIODPCDJ_00887	mupP	N-acetylmuramic acid 6-phosphate phosphatase	0	0	0	0	1	1	0	0	0	0	1	3	Accessory	COG0546_R#	28.8	106	1.39E-22	K01091	30.1	97	2.25E-19
6880	WP_033275507.1	nucS	endonuclease NucS	1	1	1	1	1	1	1	1	1	1	1	11	CORE	COG1637_L#	76.2	347	2.73E-95	K07503	75.8	345	3.62E-94
6881	WP_048457441.1	-	enoyl-CoA hydratase	1	0	0	1	1	1	1	1	1	1	1	9	Accessory	COG1024_I#	54.3	216	7.28E-56	NA	37.6	136	3.34E-31

cluster	GI	gene	Annotation	Streptomyces_thermocarboxydus_BPSAC147	Streptomyces_sp._4F	Streptomyces_sp._BSE7F	Streptomyces_sp._ETH9427	Streptomyces_sp._FxanaD5	Streptomyces_sp._UNC401CLCol	Actinospica_acidiphila_NRRL_B_24431	Streptomyces_sp._HNS054	Streptomyces_sp._SMS_SU21	Streptomyces_sp._ZS0098	Streptomyces_thermocarboxydus_K155	Addition	Core-Accessory-Unique	COG	%Similarity_COG	Score_COG	Evalue_COG	KEGG	%Similarity_KEGG	Score_KEGG	Evalue_KEGG
6882	Prokka:CIODPCDJ_03044	-	hypothetical protein	1	1	1	1	1	1	1	1	1	1	1	11	CORE								
6883	Prokka:JAPCLKMA_05895	-	hypothetical protein	1	0	1	1	1	1	1	1	1	1	1	10	Accessory	COG3707_T#	31.7	50	0.00000691				
6884	WP_102638362.1	-	vitamin K epoxide reductase family protein	1	1	1	1	1	1	1	1	1	1	1	11	CORE	COG4243_S#	37.9	153	9.89E-37	NA	37.4	146	4.21E-34
6885	OSC55690.1	-	hypothetical protein	0	1	0	0	0	0	0	0	0	0	0	1	Unique	COG0517_R#	31.4	80	1.06E-14	NA	27.1	66	4.24E-10
6886	OSC63076.1	-	GntR family transcriptional regulator	0	1	0	0	0	0	0	0	0	0	0	1	Unique	COG2186_K#	32.2	114	6.65E-25	K05799	32.2	114	1.77E-24
6887	OSC68284.1	-	rod shape-determining protein MreD	1	1	1	1	1	1	1	1	1	1	1	11	CORE								
6888	OSC57635.1	-	hypothetical protein	0	1	0	0	0	0	0	0	0	0	0	1	Unique								
6889	WP_048458592.1	-	HNH endonuclease	1	1	0	1	1	0	0	1	0	0	1	6	Accessory					NA	28.6	92	7.22E-18
6890	OSC71288.1	-	DNA primase	1	1	1	1	1	1	1	1	1	1	1	11	CORE								
6891	WP_107685346.1	-	hypothetical protein	1	0	0	0	0	0	1	0	0	0	1	3	Accessory								
6892	WP_084206927.1	-	hypothetical protein	1	0	1	1	1	1	1	1	1	1	1	10	Accessory								
6893	WP_158693616.1	-	hypothetical protein	0	0	0	0	0	1	0	0	0	0	0	1	Unique								
6894	WP_102641630.1	-	hypothetical protein	1	0	0	1	1	1	0	1	1	0	1	7	Accessory								

cluster	GI	gene	Annotation	Streptomyces_thermocarboxydus_BPSAC147	Streptomyces_sp._4F	Streptomyces_sp._BSE7F	Streptomyces_sp._ETH9427	Streptomyces_sp._FxanaD5	Streptomyces_sp._UNC401CLCol	Actinospica_acidiphila_NRRL_B_24431	Streptomyces_sp._HNS054	Streptomyces_sp._SMS_SU21	Streptomyces_sp._ZS0098	Streptomyces_thermocarboxydus_K155	Addition	Core-Accessory-Unique	COG	%Similarity_COG	Score_COG	Evalue_COG	KEGG	%Similarity_KEGG	Score_KEGG	Evalue_KEGG
6895	Prokka:CIODPCDJ_06027	scoB	putative succinyl-CoA:3-ketoacid coenzyme A transferase subunit B	1	1	0	0	1	1	1	0	0	0	1	6	Accessory	COG2057_I#	72.8	310	4.82E-84	K01029	72.8	310	1.29E-83
6896	WP_028961712.1	-	AAA family ATPase	1	1	1	1	1	1	1	1	1	1	1	11	CORE								
6897	PWE07224.1	-	DUF3105 domain-containing protein	1	1	1	1	1	1	1	1	1	1	1	11	CORE								
6898	WP_019521818.1	-	hypothetical protein	1	1	1	1	1	1	1	1	1	1	1	11	CORE								
6899	WP_048455130.1	-	pyridoxamine 5'-phosphate oxidase	1	0	1	1	1	1	1	1	0	1	1	9	Accessory	COG3576_R#	46.4	174	3.16E-43	K07006	29.9	106	4.81E-22
6900	Prokka:CIODPCDJ_01965	hxpA	Hexitol phosphatase A	1	1	1	1	1	1	1	1	1	1	1	11	CORE	COG0637_R#	44.5	161	3.61E-39	K19270	38.9	159	3.67E-38
6901	WP_033275396.1	-	DUF1275 family protein	1	0	0	0	1	1	1	0	0	0	1	5	Accessory	COG3619_S#	31.7	75	2E-13	NA	31.7	75	5.33E-13
6902	OSC61652.1	-	DNA-binding response regulator	1	1	1	1	1	1	1	1	1	1	1	11	CORE	COG0745_TK#	40.4	158	2.34E-38	K02483	40.4	158	6.26E-38
6903	WP_033276526.1	pdxH	pyridoxamine 5'-phosphate oxidase	1	1	1	1	1	1	1	1	1	1	1	11	CORE	COG0259_H#	49.1	215	2.75E-55	K00275	47.7	208	8.99E-53
6904	OSC48375.1	-	hypothetical protein	0	1	0	0	0	0	0	0	0	0	0	1	Unique	COG1020_Q#	43.1	180	5.76E-45	K15654	39.3	156	3.1E-37

cluster	GI	gene	Annotation	Streptomyces_thermocarboxydus_BPSAC147	Streptomyces_sp._4F	Streptomyces_sp._BSE7F	Streptomyces_sp._ETH9427	Streptomyces_sp._FxanaD5	Streptomyces_sp._UNC401CLCol	Actinospica_acidiphila_NRRL_B_24431	Streptomyces_sp._HNS054	Streptomyces_sp._SMS_SU21	Streptomyces_sp._ZS0098	Streptomyces_thermocarboxydus_K155	Addition	Core-Accessory-Unique	COG	%Similarity_COG	Score_COG	Evalue_COG	KEGG	%Similarity_KEGG	Score_KEGG	Evalue_KEGG
6905	WP_122214571.1	-	hypothetical protein	1	0	1	1	1	1	1	1	1	1	1	10	Accessory	COG2319_R#	29	47	0.0000582	NA	36.5	53	0.0000037
6906	AXI90523.1	-	hypothetical protein	0	0	0	1	0	0	0	0	0	0	0	1	Unique								
6907	AXI89289.1	-	hypothetical protein	0	0	0	1	0	0	0	1	1	1	0	4	Accessory								
6908	WP_122212586.1	-	potassium-transporting ATPase subunit C	0	1	1	1	0	0	1	1	1	1	0	7	Accessory	COG2156_P#	46	152	1.68E-36	K01548	42.1	141	7.91E-33
6909	WP_051724607.1	-	hypothetical protein	0	0	0	0	0	0	1	0	0	0	1	2	Accessory								
6910	WP_102638775.1	-	thiamine phosphate synthase	1	1	1	1	1	1	1	1	1	1	1	11	CORE	COG0352_H#	61.7	254	3.14E-67	K00788	61.7	254	8.38E-67
6911	WP_019526610.1	-	hypothetical protein	1	0	0	1	1	0	1	1	1	0	1	7	Accessory					NA	45.1	119	5.49E-26
6912	OSC72283.1	-	hypothetical protein	1	1	0	0	1	1	1	0	0	0	1	6	Accessory								
6913	Prokka:JAPCLKMA_00813	-	hypothetical protein	1	1	1	1	1	1	1	1	1	1	1	11	CORE					NA	46.2	156	2.38E-37
6914	AXI91396.1	-	hypothetical protein	0	0	0	1	0	0	0	0	0	0	0	1	Unique								
6915	AXI88086.1	-	hypothetical protein	0	0	0	1	0	0	0	0	0	0	0	1	Unique								
6916	AXI89635.1	-	hypothetical protein	0	0	0	1	0	0	0	0	0	0	0	1	Unique								
6917	OSC64621.1	-	DUF305 domain-containing protein	1	1	1	1	1	1	1	1	1	1	1	11	CORE	COG3544_S#	36.1	123	1.09E-27	NA	36.4	101	9.08E-21
6918	Prokka:JAPCLKMA_03502	-	hypothetical protein	1	1	1	1	1	1	1	1	1	1	1	11	CORE								
6919	Prokka:CIODPCDJ_02473	-	hypothetical protein	1	1	1	1	1	1	1	0	1	1	1	10	Accessory								

cluster	GI	gene	Annotation	Streptomyces_thermocarboxydus_BPSAC147	Streptomyces_sp._4F	Streptomyces_sp._BSE7F	Streptomyces_sp._ETH9427	Streptomyces_sp._FxanaD5	Streptomyces_sp._UNC401CLCol	Actinospica_acidiphila_NRRL_B_24431	Streptomyces_sp._HNS054	Streptomyces_sp._SMS_SU21	Streptomyces_sp._ZS0098	Streptomyces_thermocarboxydus_K155	Addition	Core-Accessory-Unique	COG	%Similarity_COG	Score_COG	Evalue_COG	KEGG	%Similarity_KEGG	Score_KEGG	Evalue_KEGG
6920	OSC66251.1	-	DNA-binding response regulator	1	1	1	1	1	1	1	1	1	1	1	11	CORE	COG2197_TK#	43.1	176	8.31E-44	NA	39.8	161	1.26E-38
6921	Prokka:JAPCLKMA_01615	-	hypothetical protein	1	0	0	0	0	0	0	0	0	0	0	1	Unique	COG1651_O#	32.4	92	2.69E-18				
6922	WP_158693593.1	-	helix-turn-helix domain-containing protein	0	0	0	0	0	1	0	0	0	0	0	1	Unique	COG0664_T#	27.9	93	9.25E-19	NA	25.1	57	0.00000015
6923	WP_033278002.1	-	TetR/AcrR family transcriptional regulator	1	1	0	1	1	1	1	1	1	1	1	10	Accessory	COG1309_K#	37.4	143	1.33E-33	NA	37.4	143	3.55E-33
6924	OSC59836.1	-	hypothetical protein	0	1	0	0	0	0	0	0	0	0	0	1	Unique								
6925	WP_109288769.1	-	HAD hydrolase-like protein	1	1	1	1	1	1	1	1	1	1	1	11	CORE								
6926	WP_033278061.1	-	hypothetical protein	0	1	0	0	0	0	1	0	0	0	0	2	Accessory								
6927	PWE10598.1	-	class F sortase	1	1	1	1	1	1	1	1	1	1	1	11	CORE								
6928	WP_122213598.1	-	1,4-alpha-glucan branching protein	1	0	1	1	1	1	1	1	1	1	1	10	Accessory								
6929	WP_048456269.1	-	amino acid ABC transporter permease	1	1	1	1	1	1	1	1	1	1	1	11	CORE	COG0765_E#	56	238	2.32E-62	K02029	38	116	4.63E-25
6930	WP_019526586.1	-	hypothetical protein	1	0	1	1	1	1	1	1	1	1	1	10	Accessory								

cluster	GI	gene	Annotation	Streptomyces_thermocarboxydus_BPSAC147	Streptomyces_sp._4F	Streptomyces_sp._BSE7F	Streptomyces_sp._ETH9427	Streptomyces_sp._FxanaD5	Streptomyces_sp._UNC401CLCol	Actinospica_acidiphila_NRRL_B_24431	Streptomyces_sp._HNS054	Streptomyces_sp._SMS_SU21	Streptomyces_sp._ZS0098	Streptomyces_thermocarboxydus_K155	Addition	Core-Accessory-Unique	COG	%Similarity_COG	Score_COG	Evalue_COG	KEGG	%Similarity_KEGG	Score_KEGG	Evalue_KEGG
6931	Prokka:CIODPCDJ_00033	nicS	HTH-type transcriptional repressor NicS	1	1	0	0	1	1	0	0	0	0	1	5	Accessory	COG1309_K#	52.3	220	8.52E-57	NA	26.8	61	1.35E-08
6932	WP_019525965.1	-	DNA primase	1	0	1	1	1	1	1	1	1	1	1	10	Accessory								
6933	WP_086702543.1	-	ABC transporter permease	1	1	1	1	1	1	1	1	1	1	1	11	CORE	COG1174_E#	49.5	204	3.71E-52	K05846	49.5	204	9.9E-52
6934	AXI84703.1	-	MerR family DNA-binding transcriptional regulator	0	0	1	1	0	0	0	1	1	1	0	5	Accessory	COG0789_K#	42.5	57	7.32E-08				
6935	PWE08721.1	-	hydrolase	1	1	1	1	1	1	1	1	1	1	1	11	CORE	COG0637_R#	43.8	163	1.24E-39	NA	32.5	108	9.65E-23
6936	WP_033275153.1	-	membrane protein	1	1	1	1	1	1	1	1	1	1	1	11	CORE	COG2364_S#	44.4	147	5.38E-35	K07149	44.4	147	1.44E-34
6937	WP_122216852.1	-	ATP-binding protein	1	1	0	1	1	1	1	1	1	1	1	10	Accessory								
6938	WP_019527388.1	-	hypothetical protein	1	0	0	0	1	1	0	0	0	0	1	4	Accessory								
6939	WP_019521650.1	-	VWA domain-containing protein	0	0	1	0	1	1	1	0	0	0	0	4	Accessory	COG4245_R#	27	77	5.23E-14				
6940	AXI91372.1	-	hypothetical protein	0	0	0	1	0	0	0	0	0	0	0	1	Unique								
6941	OSC61464.1	-	hypothetical protein	0	1	0	0	0	0	0	0	0	0	0	1	Unique	COG0517_R#	30.8	95	2.42E-19	NA	29.9	70	1.71E-11
6942	OSC60092.1	-	hypothetical protein	1	1	1	1	1	1	0	1	1	1	1	10	Accessory					NA	34.7	109	5.66E-23

cluster	GI	gene	Annotation	Streptomyces_thermocarboxydus_BPSAC147	Streptomyces_sp._4F	Streptomyces_sp._BSE7F	Streptomyces_sp._ETH9427	Streptomyces_sp._FxanaD5	Streptomyces_sp._UNC401CLCol	Actinospica_acidiphila_NRRL_B_24431	Streptomyces_sp._HNS054	Streptomyces_sp._SMS_SU21	Streptomyces_sp._ZS0098	Streptomyces_thermocarboxydus_K155	Addition	Core-Accessory-Unique	COG	%Similarity_COG	Score_COG	Evalue_COG	KEGG	%Similarity_KEGG	Score_KEGG	Evalue_KEGG
6943	PWE11055.1	-	transcriptional regulator	0	0	1	0	0	0	0	0	0	0	0	1	Unique								
6944	WP_122215619.1	-	hypothetical protein	0	1	0	1	0	0	0	1	1	1	0	5	Accessory								
6945	WP_122215913.1	-	TetR family transcriptional regulator	0	1	1	1	0	0	1	1	0	1	0	6	Accessory	COG1309_K#	33.9	102	2.59E-21	K16137	26.8	52	0.00000481
6946	OSC73186.1	-	DNA-binding response regulator	0	1	0	0	0	0	0	0	0	0	0	1	Unique	COG0745_TK#	64.1	283	4.81E-76	K02483	48	198	5.43E-50
6947	OSC61436.1	-	hypothetical protein	0	1	0	0	0	0	0	0	0	0	0	1	Unique								
6948	PWE10280.1	-	two-component system response regulator	0	0	1	0	0	0	0	0	0	0	0	1	Unique	COG2197_TK#	31.9	107	4.72E-23	NA	35.4	102	6.92E-21
6949	WP_037953217.1	-	hypothetical protein	1	0	0	0	0	1	1	1	0	0	1	5	Accessory								
6950	WP_019527430.1	-	YdcF family protein	0	0	0	0	1	0	1	0	0	0	0	2	Accessory	COG1434_S#	40.3	142	1.73E-33				
6951	AXI89624.1	-	hypothetical protein	0	0	0	1	0	0	0	0	0	0	0	1	Unique								
6952	WP_102640598.1	-	DUF4276 family protein	0	0	0	0	0	0	0	0	1	0	0	1	Unique								
6953	OSC57517.1	-	DNA-binding response regulator	1	1	1	1	1	1	1	1	1	1	1	11	CORE	COG2197_TK#	46.9	178	2.17E-44	K02479	43.3	174	8.36E-43
6954	WP_122216354.1	-	SDR family oxidoreductase	1	1	1	1	1	1	1	1	1	1	1	11	CORE	COG0702_MG#	40.6	143	1.01E-33	NA	33.9	124	1.3E-27

cluster	GI	gene	Annotation	Streptomyces_thermocarboxydus_BPSAC147	Streptomyces_sp._4F	Streptomyces_sp._BSE7F	Streptomyces_sp._ETH9427	Streptomyces_sp._FxanaD5	Streptomyces_sp._UNC401CLCol	Actinospica_acidiphila_NRRL_B_24431	Streptomyces_sp._HNS054	Streptomyces_sp._SMS_SU21	Streptomyces_sp._ZS0098	Streptomyces_thermocarboxydus_K155	Addition	Core-Accessory-Unique	COG	%Similarity_COG	Score_COG	Evalue_COG	KEGG	%Similarity_KEGG	Score_KEGG	Evalue_KEGG
6955	OSC55866.1	-	polyisoprenoid-binding protein	1	1	0	0	1	1	0	0	0	0	1	5	Accessory	COG2353_S#	47.8	170	7.71E-42	NA	30.3	78	8.15E-14
6956	WP_048456632.1	-	transcriptional regulator	1	0	1	1	1	1	1	1	1	1	1	10	Accessory								
6957	AXI89327.1	-	DNA-binding response regulator	1	1	1	1	1	1	1	1	1	1	1	11	CORE	COG2197_TK#	32.4	76	1.16E-13	K07684	32.4	76	3.1E-13
6958	WP_122212894.1	-	DUF305 domain-containing protein	1	1	1	1	1	1	1	1	1	1	1	11	CORE	COG3544_S#	28.9	97	4.86E-20	NA	32.7	81	9.63E-15
6959	Prokka:CIODPCDJ_06130	-	hypothetical protein	1	1	1	1	1	1	1	1	1	1	1	11	CORE	COG1595_K#	44.4	112	2.49E-24	K03088	44.4	112	6.65E-24
6960	PWE10680.1	-	DUF4230 domain-containing protein	1	1	1	1	1	1	1	1	1	1	1	11	CORE								
6961	WP_033277374.1	-	GNAT family N-acetyltransferase	1	1	1	1	1	1	1	1	1	1	1	11	CORE	COG1670_J#	47.3	171	4.52E-42	K03790	47.3	171	1.21E-41
6962	Prokka:JAPCLKMA_03786	gph_6	Phosphoglycolate phosphatase	1	0	0	0	0	0	0	0	0	0	1	2	Accessory	COG1011_R#	27.3	82	2.76E-15	K07025	27.7	50	0.0000238
6963	PWE11031.1	-	urease subunit beta	1	1	1	1	1	1	1	1	1	1	1	11	CORE	COG0831_E#	63.6	128	2.57E-29	K14048	51	198	9.22E-50
6964	WP_102638034.1	-	hypothetical protein	1	1	1	1	1	1	1	1	1	1	1	11	CORE								
6965	OSC63617.1	-	hypothetical protein	0	1	0	0	0	0	0	0	0	0	0	1	Unique								
6966	OSC69006.1	-	hypothetical protein	0	1	0	0	0	0	0	0	0	0	0	1	Unique								

cluster	GI	gene	Annotation	Streptomyces_thermocarboxydus_BPSAC147	Streptomyces_sp._4F	Streptomyces_sp._BSE7F	Streptomyces_sp._ETH9427	Streptomyces_sp._FxanaD5	Streptomyces_sp._UNC401CLCol	Actinospica_acidiphila_NRRL_B_24431	Streptomyces_sp._HNS054	Streptomyces_sp._SMS_SU21	Streptomyces_sp._ZS0098	Streptomyces_thermocarboxydus_K155	Addition	Core-Accessory-Unique	COG	%Similarity_COG	Score_COG	Evalue_COG	KEGG	%Similarity_KEGG	Score_KEGG	Evalue_KEGG
6967	WP_122215930.1	-	class I SAM-dependent methyltransferase	0	0	0	0	0	0	0	0	0	1	0	1	Unique								
6968	OSC71702.1	-	hypothetical protein	1	1	1	0	1	1	1	1	1	1	1	10	Accessory	COG0586_S#	28.9	45	0.000286				
6969	OSC63928.1	-	SMC-Scp complex subunit ScpB	1	1	1	1	1	1	1	1	1	1	1	11	CORE	COG1386_K#	50.7	194	4.99E-49	K06024	50.7	194	1.33E-48
6970	AXI91140.1	-	GPP34 family phosphoprotein	0	0	0	1	0	0	0	0	0	0	0	1	Unique								
6971	OSC72726.1	-	GPP34 family phosphoprotein	1	1	1	1	1	1	1	1	1	1	1	11	CORE								
6972	OSC69510.1	-	enterochelin esterase	0	1	0	0	0	0	0	0	0	0	0	1	Unique	COG2382_P#	31.7	90	7.76E-18	K07214	31.7	90	2.07E-17
6973	Prokka:JAPCLKMA_01566	-	hypothetical protein	1	0	0	0	0	0	1	0	0	0	1	3	Accessory								
6974	WP_102637907.1	-	NUDIX hydrolase	0	0	1	0	0	0	0	0	1	0	0	2	Accessory	COG0494_LR#	30.1	78	3.99E-14				
6975	OSC69231.1	-	hypothetical protein	0	1	0	0	0	0	0	0	0	0	0	1	Unique								
6976	OSC62683.1	-	hypothetical protein	0	1	0	0	0	0	0	0	0	0	0	1	Unique								
6977	Prokka:JAPCLKMA_04800	-	hypothetical protein	1	1	1	1	1	1	1	1	1	1	1	11	CORE								
6978	OSC68517.1	-	haloacid dehalogenase	0	1	0	0	0	0	0	0	0	0	0	1	Unique	COG1011_R#	34.1	67	9.19E-11	K07025	34.1	67	2.45E-10

cluster	GI	gene	Annotation	Streptomyces_thermocarboxydus_BPSAC147	Streptomyces_sp._4F	Streptomyces_sp._BSE7F	Streptomyces_sp._ETH9427	Streptomyces_sp._FxanaD5	Streptomyces_sp._UNC401CLCol	Actinospica_acidiphila_NRRL_B_24431	Streptomyces_sp._HNS054	Streptomyces_sp._SMS_SU21	Streptomyces_sp._ZS0098	Streptomyces_thermocarboxydus_K155	Addition	Core-Accessory-Unique	COG	%Similarity_COG	Score_COG	Evalue_COG	KEGG	%Similarity_KEGG	Score_KEGG	Evalue_KEGG
6979	Prokka:CIODPCDJ_02796	-	hypothetical protein	0	1	0	0	0	1	0	0	0	0	1	3	Accessory								
6980	OSC69938.1	-	hypothetical protein	0	1	0	0	0	0	0	0	0	0	0	1	Unique								
6981	OSC70544.1	-	hypothetical protein	0	1	0	0	0	0	0	0	0	0	0	1	Unique								
6982	Prokka:JAPCLKMA_01638	-	hypothetical protein	1	1	1	1	1	1	1	1	1	1	1	11	CORE	COG2229_R#	53.6	191	3.23E-48	K06945	53.6	191	8.63E-48
6983	WP_033276036.1	-	NAD(P)-binding domain-containing protein	1	0	1	1	1	1	1	1	1	1	1	10	Accessory	COG2085_R#	53.6	221	2.92E-57				
6984	OSC71928.1	-	hypothetical protein	0	1	0	0	0	0	0	0	0	0	0	1	Unique								
6985	OSC72538.1	-	hypothetical protein	0	1	0	0	0	0	0	0	0	0	0	1	Unique								
6986	AXI91376.1	-	hypothetical protein	0	0	0	1	0	0	0	0	0	0	0	1	Unique								
6987	PWE11058.1	-	phosphatase	0	0	1	0	0	0	0	0	1	1	0	3	Accessory	COG0394_T#	50.8	196	1E-49	K03325	48.6	131	1.06E-29
6988	Prokka:JAPCLKMA_00631	oppF	Oligopeptide transport ATP-binding protein OppF	1	1	0	1	1	1	1	1	1	1	1	10	Accessory	COG4608_E#	39.3	149	1.07E-35	K02032	36	135	5.59E-31
6989	WP_122213267.1	-	TrkA family potassium uptake protein	0	0	0	0	0	1	0	0	1	1	1	4	Accessory	COG0569_P#	33	121	5.34E-27	K03499	32.6	119	4.15E-26
6990	Prokka:JAPCLKMA_05112	-	hypothetical protein	1	0	1	1	1	1	1	1	1	1	1	10	Accessory								

cluster	GI	gene	Annotation	Streptomyces_thermocarboxydus_BPSAC147	Streptomyces_sp._4F	Streptomyces_sp._BSE7F	Streptomyces_sp._ETH9427	Streptomyces_sp._FxanaD5	Streptomyces_sp._UNC401CLCol	Actinospica_acidiphila_NRRL_B_24431	Streptomyces_sp._HNS054	Streptomyces_sp._SMS_SU21	Streptomyces_sp._ZS0098	Streptomyces_thermocarboxydus_K155	Addition	Core-Accessory-Unique	COG	%Similarity_COG	Score_COG	Evalue_COG	KEGG	%Similarity_KEGG	Score_KEGG	Evalue_KEGG
6991	WP_048458042.1	rplD	50S ribosomal protein L4	1	1	1	1	1	1	1	1	1	1	1	11	CORE	COG0088_J#	64.8	279	1.17E-74	K02926	64.8	279	3.13E-74
6992	WP_033273066.1	-	response regulator	1	1	1	1	1	1	1	1	1	1	1	11	CORE	COG3707_T#	64.4	263	6.66E-70	K07183	64.4	263	1.78E-69
6993	WP_102641706.1	-	DUF5063 domain-containing protein	1	1	1	1	1	1	1	1	1	1	1	11	CORE								
6994	AXI89515.1	-	fasciclin	0	0	0	1	0	0	0	1	1	1	0	4	Accessory	COG2335_M#	39.2	141	2.92E-33	NA	39.2	141	7.81E-33
6995	WP_033276867.1	-	ATP-dependent Clp protease proteolytic subunit	1	1	1	1	1	1	1	1	1	1	1	11	CORE	COG0740_OU#	64.5	250	4.47E-66	K01358	59.2	224	9.16E-58
6996	WP_028958927.1	-	TrkA family potassium uptake protein	0	0	0	0	0	1	0	0	1	1	1	4	Accessory	COG0569_P#	36.9	139	1.89E-32	K03499	36.9	139	5.06E-32
6997	WP_019524471.1	-	hypothetical protein	1	0	0	0	1	1	0	0	0	0	0	3	Accessory								
6998	AXI91261.1	-	hypothetical protein	0	0	0	1	0	0	0	0	0	0	0	1	Unique								
6999	Prokka:JAPCLKMA_00971	-	hypothetical protein	1	1	1	1	1	1	1	1	1	1	1	11	CORE	COG2049_E#	47.4	183	1.14E-45	NA	43.3	146	2.43E-34
7000	OSC73246.1	-	peptidase M23	1	1	1	1	1	1	1	1	1	1	1	11	CORE	COG0739_M#	45.4	103	1.15E-21	NA	45.4	103	3.08E-21
7001	OSC67736.1	-	keto-deoxy-phosphogluconate aldolase	1	1	1	1	1	1	1	1	1	1	1	11	CORE	COG0800_G#	59.1	239	1.03E-62	K01625	54.5	227	1.41E-58
7002	WP_033276068.1	-	hypothetical protein	1	1	1	1	1	1	1	1	1	1	1	11	CORE	COG2267_I#	39.2	51	0.0000052	K11275	31.6	52	0.00000813

cluster	GI	gene	Annotation	Streptomyces_thermocarboxydus_BPSAC147	Streptomyces_sp._4F	Streptomyces_sp._BSE7F	Streptomyces_sp._ETH9427	Streptomyces_sp._FxanaD5	Streptomyces_sp._UNC401CLCol	Actinospica_acidiphila_NRRL_B_24431	Streptomyces_sp._HNS054	Streptomyces_sp._SMS_SU21	Streptomyces_sp._ZS0098	Streptomyces_thermocarboxydus_K155	Addition	Core-Accessory-Unique	COG	%Similarity_COG	Score_COG	Evalue_COG	KEGG	%Similarity_KEGG	Score_KEGG	Evalue_KEGG
7003	WP_033273138.1	-	cupin domain-containing protein	1	1	1	1	1	1	1	1	1	1	1	11	CORE	COG1741_R#	40.2	163	1.23E-39	K06911	40.2	163	3.27E-39
7004	WP_146217817.1	-	suppressor of fused domain protein	0	0	0	0	0	0	0	0	1	0	0	1	Unique								
7005	OSC72714.1	-	hypothetical protein	1	1	1	1	1	1	1	1	1	1	1	11	CORE								
7006	OSC58527.1	-	hypothetical protein	0	1	0	0	0	0	0	0	0	0	0	1	Unique	COG3247_S#	32.4	97	4.84E-20	NA	32.4	97	1.29E-19
7007	OSC61435.1	-	hypothetical protein	0	1	0	0	0	0	0	0	0	0	0	1	Unique								
7008	PWE08821.1	-	hypothetical protein	0	0	1	0	0	0	0	0	0	1	0	2	Accessory								
7009	WP_048455643.1	-	hypothetical protein	1	0	1	0	1	1	1	1	1	1	1	9	Accessory								
7010	OSC66124.1	-	PadR family transcriptional regulator	1	1	1	1	1	1	1	1	1	1	1	11	CORE	COG1695_K#	48.4	157	6.69E-38	NA	47.1	81	1.25E-14
7011	AXI90117.1	-	HAD family hydrolase	1	1	1	1	1	1	1	1	1	1	1	11	CORE	COG0546_R#	36.3	115	2.92E-25	NA	36.3	115	7.79E-25
7012	WP_033273311.1	-	SCO family protein	1	1	1	1	1	1	1	1	1	1	1	11	CORE	COG1999_R#	35.2	102	1.96E-21	K07152	35.2	102	5.22E-21
7013	WP_026243357.1	-	phosphatidylserine decarboxylase	1	1	1	1	1	1	1	1	1	1	1	11	CORE	COG0688_I#	44.2	154	4.34E-37	K01613	41.4	117	2.05E-25
7014	OSC59665.1	-	SAM-dependent methyltransferase	0	1	0	0	0	0	0	0	0	0	0	1	Unique								

cluster	GI	gene	Annotation	Streptomyces_thermocarboxydus_BPSAC147	Streptomyces_sp._4F	Streptomyces_sp._BSE7F	Streptomyces_sp._ETH9427	Streptomyces_sp._FxanaD5	Streptomyces_sp._UNC401CLCol	Actinospica_acidiphila_NRRL_B_24431	Streptomyces_sp._HNS054	Streptomyces_sp._SMS_SU21	Streptomyces_sp._ZS0098	Streptomyces_thermocarboxydus_K155	Addition	Core-Accessory-Unique	COG	%Similarity_COG	Score_COG	Evalue_COG	KEGG	%Similarity_KEGG	Score_KEGG	Evalue_KEGG
7015	Prokka:JAPCLKMA_03980	-	hypothetical protein	1	1	1	1	1	1	1	1	1	1	1	11	CORE	COG1309_K#	33.8	77	6.74E-14	NA	33.8	77	1.8E-13
7016	WP_048455013.1	-	zf-HC2 domain-containing protein	1	1	1	1	1	1	1	1	1	1	1	11	CORE								
7017	AXI86170.1	-	Zn-dependent hydrolase	1	1	1	1	1	1	1	1	1	1	1	11	CORE	COG0491_R#	52.5	216	9.3E-56	NA	52.5	216	2.48E-55
7018	WP_048460798.1	-	TetR/AcrR family transcriptional regulator	0	0	1	0	0	0	0	1	1	1	0	4	Accessory	COG1309_K#	24.1	62	2.24E-09	K18939	31.6	57	2.52E-07
7019	WP_048456704.1	-	hydrolase	0	0	1	1	0	0	0	1	1	0	0	4	Accessory	COG1335_Q#	64.8	285	1.25E-76	NA	42.7	138	1.12E-31
7020	PWE07964.1	-	hypothetical protein	1	0	1	1	1	1	1	1	1	1	1	10	Accessory								
7021	WP_122215218.1	-	hypothetical protein	1	0	1	0	1	0	0	0	0	1	1	5	Accessory								
7022	OSC66861.1	-	hypothetical protein	0	1	0	0	0	0	0	0	0	0	0	1	Unique	COG2120_S#	34.9	100	7.43E-21				
7023	PWE10331.1	-	hypothetical protein	0	0	1	0	0	0	0	0	0	0	0	1	Unique								
7024	Prokka:JAPCLKMA_06147	slmA_3	Nucleoid occlusion factor SlmA	1	1	1	1	1	1	1	1	1	1	1	11	CORE	COG1309_K#	37.4	135	2.09E-31	NA	37.4	135	5.57E-31
7025	Prokka:JAPCLKMA_04167	-	hypothetical protein	1	1	1	1	1	1	1	1	1	1	1	11	CORE	COG1309_K#	32	102	2.56E-21	NA	31.5	89	3.5E-17
7026	OSC61655.1	-	hypothetical protein	0	1	0	0	0	0	0	0	0	0	0	1	Unique								
7027	WP_033273904.1	-	HAD family hydrolase	1	1	1	0	1	1	1	1	1	1	1	10	Accessory	COG0241_E#	34.6	103	1.15E-21	K03273	36.1	93	2.43E-18

cluster	GI	gene	Annotation	Streptomyces_thermocarboxydus_BPSAC147	Streptomyces_sp._4F	Streptomyces_sp._BSE7F	Streptomyces_sp._ETH9427	Streptomyces_sp._FxanaD5	Streptomyces_sp._UNC401CLCol	Actinospica_acidiphila_NRRL_B_24431	Streptomyces_sp._HNS054	Streptomyces_sp._SMS_SU21	Streptomyces_sp._ZS0098	Streptomyces_thermocarboxydus_K155	Addition	Core-Accessory-Unique	COG	%Similarity_COG	Score_COG	Evalue_COG	KEGG	%Similarity_KEGG	Score_KEGG	Evalue_KEGG
7028	AXI89611.1	-	histidine phosphatase family protein	0	0	0	1	0	0	0	0	0	0	0	1	Unique								
7029	WP_019523362.1	-	hypothetical protein	0	0	0	0	1	0	0	0	0	0	0	1	Unique								
7030	OSC70895.1	-	IclR family transcriptional regulator	1	1	1	1	1	1	1	1	1	1	1	11	CORE	COG1414_K#	28.2	61	3.83E-09				
7031	OSC66911.1	-	DNA-binding protein	1	1	1	1	1	1	1	1	1	1	1	11	CORE	COG0776_L#	47.5	161	3.55E-39	K03530	47.5	161	9.48E-39
7032	OSC71620.1	-	transcriptional regulator	1	1	1	0	1	1	1	1	1	1	1	10	Accessory								
7033	OSC58482.1	-	hypothetical protein	0	1	0	0	0	0	0	0	0	0	0	1	Unique								
7034	OSC71166.1	-	TetR family transcriptional regulator	1	1	1	1	1	1	1	1	1	1	1	11	CORE	COG1309_K#	27.8	84	5.51E-16	NA	27.8	84	1.47E-15
7035	OSC60429.1	-	TetR family transcriptional regulator	0	1	0	0	0	0	0	0	0	0	0	1	Unique	COG1309_K#	32.9	65	2.03E-10				
7036	Prokka:JAPCLKMA_00015	-	hypothetical protein	1	0	0	0	0	0	0	0	0	0	0	1	Unique								
7037	AXI88268.1	-	adenylate kinase	1	1	0	1	1	1	1	1	1	1	1	10	Accessory	COG0563_F#	57.3	255	1.38E-67	K00939	53.7	231	5.68E-60

cluster	GI	gene	Annotation	Streptomyces_thermocarboxydus_BPSAC147	Streptomyces_sp._4F	Streptomyces_sp._BSE7F	Streptomyces_sp._ETH9427	Streptomyces_sp._FxanaD5	Streptomyces_sp._UNC401CLCol	Actinospica_acidiphila_NRRL_B_24431	Streptomyces_sp._HNS054	Streptomyces_sp._SMS_SU21	Streptomyces_sp._ZS0098	Streptomyces_thermocarboxydus_K155	Addition	Core-Accessory-Unique	COG	%Similarity_COG	Score_COG	Evalue_COG	KEGG	%Similarity_KEGG	Score_KEGG	Evalue_KEGG
7038	PWE08974.1	tsaB	tRNA (adenosine(37)-N6)-threonylcarbamoyltransferase complex dimerization subunit type 1 TsaB	1	1	1	1	1	1	1	1	1	1	1	11	CORE	COG1214_O#	43.1	145	2.62E-34	NA	43.1	145	7E-34
7039	PWE11989.1	-	ATP-dependent Clp protease proteolytic subunit	1	0	1	1	1	1	1	1	1	1	1	10	Accessory	COG0740_OU#	65.1	234	3.28E-61	K01358	60.7	215	7.19E-55
7040	WP_102638129.1	-	N-acetylmuramoyl-L-alanine amidase	1	0	1	1	1	1	1	1	1	1	1	10	Accessory								
7041	WP_028961626.1	-	hypothetical protein	1	0	0	0	1	1	0	0	0	0	1	4	Accessory								
7042	WP_028961665.1	-	hypothetical protein	0	0	0	0	1	1	0	1	0	0	1	4	Accessory								
7043	WP_033275651.1	-	hypothetical protein	1	0	0	0	1	1	1	0	0	0	1	5	Accessory								
7044	OSC63326.1	-	hypothetical protein	1	1	1	1	1	1	1	1	1	1	1	11	CORE								
7045	OSC71524.1	-	hypothetical protein	0	1	0	0	0	0	0	0	0	0	0	1	Unique								
7046	OSC62096.1	-	dienelactone hydrolase	1	1	1	1	1	1	1	1	1	1	1	11	CORE	COG0412_Q#	45	149	1.06E-35				

cluster	GI	gene	Annotation	Streptomyces_thermocarboxydus_BPSAC147	Streptomyces_sp._4F	Streptomyces_sp._BSE7F	Streptomyces_sp._ETH9427	Streptomyces_sp._FxanaD5	Streptomyces_sp._UNC401CLCol	Actinospica_acidiphila_NRRL_B_24431	Streptomyces_sp._HNS054	Streptomyces_sp._SMS_SU21	Streptomyces_sp._ZS0098	Streptomyces_thermocarboxydus_K155	Addition	Core-Accessory-Unique	COG	%Similarity_COG	Score_COG	Evalue_COG	KEGG	%Similarity_KEGG	Score_KEGG	Evalue_KEGG
7047	WP_048455654.1	-	TetR/AcrR family transcriptional regulator	1	1	1	1	1	1	1	1	1	1	0	10	Accessory	COG1309_K#	33.6	69	2.39E-11	NA	31.9	65	9.2E-10
7048	Prokka:CIODPCDJ_05601	-	putative membrane protein	1	1	1	1	1	1	1	1	1	1	1	11	CORE								
7049	Prokka:JAPCLKMA_03485	-	hypothetical protein	1	0	1	1	1	1	1	0	1	1	1	9	Accessory								
7050	WP_102636946.1	-	hypothetical protein	0	0	1	1	0	0	1	1	1	1	0	6	Accessory								
7051	WP_102640665.1	-	recombinase family protein	0	0	0	0	0	0	0	0	1	0	0	1	Unique								
7052	OSC69103.1	-	TetR family transcriptional regulator	0	1	1	1	0	0	1	1	1	1	0	7	Accessory	COG1309_K#	31.1	77	8.76E-14	NA	31.1	77	2.34E-13
7053	WP_122215831.1	-	hypothetical protein	0	0	0	0	0	0	0	0	0	1	0	1	Unique								
7054	AXI91397.1	-	hypothetical protein	0	0	0	1	0	0	0	0	0	0	0	1	Unique								
7055	OSC64692.1	-	translation initiation factor IF-3	1	1	1	1	1	1	1	1	1	1	1	11	CORE	COG0290_J#	66	269	9.21E-72	K02520	66	269	2.46E-71
7056	WP_019524053.1	-	AIM24 family protein	1	1	1	1	1	1	1	1	1	1	1	11	CORE	COG2013_S#	37.9	138	3.19E-32	NA	37.9	138	8.51E-32
7057	WP_019522891.1	def	peptide deformylase	1	1	1	1	1	1	1	1	1	1	1	11	CORE	COG0242_J#	51.1	161	2.69E-39	K01462	46.6	142	4.51E-33
7058	Prokka:JAPCLKMA_03555	cseA	Lipoprotein CseA	1	1	1	1	1	1	1	1	1	1	1	11	CORE								

cluster	GI	gene	Annotation	Streptomyces_thermocarboxydus_BPSAC147	Streptomyces_sp._4F	Streptomyces_sp._BSE7F	Streptomyces_sp._ETH9427	Streptomyces_sp._FxanaD5	Streptomyces_sp._UNC401CLCol	Actinospica_acidiphila_NRRL_B_24431	Streptomyces_sp._HNS054	Streptomyces_sp._SMS_SU21	Streptomyces_sp._ZS0098	Streptomyces_thermocarboxydus_K155	Addition	Core-Accessory-Unique	COG	%Similarity_COG	Score_COG	Evalue_COG	KEGG	%Similarity_KEGG	Score_KEGG	Evalue_KEGG
7059	WP_033274749.1	ehuD	ectoine/hydroxyectoine ABC transporter permease subunit EhuD	1	1	1	1	1	1	1	1	1	1	1	11	CORE	COG0765_E#	51.4	221	2.19E-57	K10009	38.7	131	7.97E-30
7060	WP_019526345.1	-	thymidine kinase	1	1	1	1	1	1	1	1	1	1	1	11	CORE	COG1435_F#	39.4	133	1.03E-30	K00857	36.4	123	2.83E-27
7061	WP_033274830.1	-	hypothetical protein	1	1	1	1	1	1	1	1	1	1	1	11	CORE								
7062	WP_048456441.1	-	hypothetical protein	0	0	1	1	0	0	0	1	1	1	0	5	Accessory								
7063	Prokka:JAPCLKMA_03260	vldH_1	Validoxylamine A 7'-phosphate phosphatase	1	0	1	1	1	1	1	1	1	1	1	10	Accessory	COG0637_R#	44.9	161	2.69E-39	K19270	38.4	131	7.97E-30
7064	WP_019524368.1	-	TetR family transcriptional regulator	1	0	1	1	1	1	1	1	1	1	1	10	Accessory	COG1309_K#	35.3	107	7.87E-23	NA	31.9	98	7.47E-20
7065	PWE07286.1	-	TetR family transcriptional regulator	1	1	1	1	1	1	1	1	1	1	1	11	CORE	COG1309_K#	42	147	5.25E-35	NA	42.9	146	2.39E-34
7066	AXI85332.1	-	DUF4913 domain-containing protein	1	0	0	1	0	0	0	0	0	0	0	2	Accessory								
7067	Prokka:CIODPCDJ_06054	pcp	Pyrrolidone-carboxylate peptidase	1	0	0	0	1	1	0	0	0	0	1	4	Accessory	COG2039_O#	50.7	229	1.37E-59	K01304	52.1	225	5.3E-58

cluster	GI	gene	Annotation	Streptomyces_thermocarboxydus_BPSAC147	Streptomyces_sp._4F	Streptomyces_sp._BSE7F	Streptomyces_sp._ETH9427	Streptomyces_sp._FxanaD5	Streptomyces_sp._UNC401CLCol	Actinospica_acidiphila_NRRL_B_24431	Streptomyces_sp._HNS054	Streptomyces_sp._SMS_SU21	Streptomyces_sp._ZS0098	Streptomyces_thermocarboxydus_K155	Addition	Core-Accessory-Unique	COG	%Similarity_COG	Score_COG	Evalue_COG	KEGG	%Similarity_KEGG	Score_KEGG	Evalue_KEGG
7068	WP_026243499.1	-	hypothetical protein	1	0	0	0	1	1	0	0	0	0	1	4	Accessory								
7069	WP_028960175.1	leuE	leucine efflux protein LeuE	1	0	1	1	1	1	1	1	1	1	1	10	Accessory	COG1280_E#	61.7	262	1.46E-69	K11250	41.9	170	1.55E-41
7070	WP_019527821.1	-	TetR family transcriptional regulator	1	1	0	0	1	1	1	0	0	0	1	6	Accessory	COG1309_K#	37	124	6.22E-28	NA	29.9	70	2.85E-11
7071	WP_019522921.1	-	CGNR zinc finger domain-containing protein	1	1	0	0	1	1	1	1	1	1	1	9	Accessory								
7072	AXI85280.1	-	hypothetical protein	1	0	0	1	0	0	0	0	0	0	0	2	Accessory								
7073	Prokka:CIODPCDJ_05505	-	hypothetical protein	1	0	1	1	1	1	1	1	1	1	1	10	Accessory	COG0739_M#	32.3	40	0.00905				
7074	OSC72301.1	-	uracil-DNA glycosylase	0	1	0	0	0	0	1	0	0	0	0	2	Accessory	COG1573_L#	50.3	182	1.47E-45	K02334	41.7	124	1.27E-27
7075	Prokka:CIODPCDJ_00926	petC_1	Cytochrome b6-f complex iron-sulfur subunit	1	1	1	0	1	1	1	1	1	1	1	10	Accessory	COG2146_PR#	36.3	70	8.16E-12	K15878	40.6	61	1.01E-08
7076	WP_122217350.1	-	lipopolysaccharide biosynthesis protein	1	1	1	1	1	1	1	1	1	1	0	10	Accessory								
7077	WP_158688231.1	-	hypothetical protein	0	0	0	0	1	0	0	0	0	0	0	1	Unique								
7078	OSC60093.1	-	hypothetical protein	0	1	0	0	0	0	0	0	0	0	0	1	Unique								
7079	OSC58520.1	-	hypothetical protein	0	1	0	0	0	0	0	0	0	0	0	1	Unique								

cluster	GI	gene	Annotation	Streptomyces_thermocarboxydus_BPSAC147	Streptomyces_sp._4F	Streptomyces_sp._BSE7F	Streptomyces_sp._ETH9427	Streptomyces_sp._FxanaD5	Streptomyces_sp._UNC401CLCol	Actinospica_acidiphila_NRRL_B_24431	Streptomyces_sp._HNS054	Streptomyces_sp._SMS_SU21	Streptomyces_sp._ZS0098	Streptomyces_thermocarboxydus_K155	Addition	Core-Accessory-Unique	COG	%Similarity_COG	Score_COG	Evalue_COG	KEGG	%Similarity_KEGG	Score_KEGG	Evalue_KEGG
7080	OSC55867.1	-	hypothetical protein	0	1	0	0	0	0	0	0	0	0	0	1	Unique								
7081	OSC66065.1	-	imidazole glycerol phosphate synthase subunit HisH	1	1	1	1	1	1	1	1	1	1	1	11	CORE	COG0118_E#	62.6	273	4.86E-73	K02501	60.2	263	1.75E-69
7082	AXI89573.1	-	MBL fold metallo-hydrolase	1	1	1	1	1	1	1	1	1	1	1	11	CORE	COG2220_R#	43.7	158	2.28E-38	NA	43.7	158	6.09E-38
7083	OSC57273.1	-	alpha-ketoglutarate-dependent dioxygenase AlkB	0	1	0	0	0	0	0	0	0	0	0	1	Unique	COG3145_L#	43.8	168	2.2E-41	NA	33.2	112	6.53E-24
7084	OSC73391.1	-	TetR family transcriptional regulator	0	1	0	0	0	0	0	0	0	0	0	1	Unique	COG1309_K#	36.6	128	3.3E-29	NA	36.6	128	8.81E-29
7085	WP_078504695.1	-	hypothetical protein	0	0	0	0	1	0	0	0	0	0	0	1	Unique								
7086	WP_084207084.1	-	SnoaL-like domain-containing protein	0	0	0	0	1	0	1	0	0	0	1	3	Accessory								
7087	WP_019524921.1	cas6e	type I-E CRISPR-associated protein Cas6/Cse3/CasE	0	0	0	0	1	0	0	0	0	0	1	2	Accessory								
7088	OSC72832.1	-	hypothetical protein	0	1	0	0	0	0	0	0	0	0	0	1	Unique								
7089	WP_102640767.1	-	hypothetical protein	1	0	0	1	0	0	1	1	1	0	0	5	Accessory								

cluster	GI	gene	Annotation	Streptomyces_thermocarboxydus_BPSAC147	Streptomyces_sp._4F	Streptomyces_sp._BSE7F	Streptomyces_sp._ETH9427	Streptomyces_sp._FxanaD5	Streptomyces_sp._UNC401CLCol	Actinospica_acidiphila_NRRL_B_24431	Streptomyces_sp._HNS054	Streptomyces_sp._SMS_SU21	Streptomyces_sp._ZS0098	Streptomyces_thermocarboxydus_K155	Addition	Core-Accessory-Unique	COG	%Similarity_COG	Score_COG	Evalue_COG	KEGG	%Similarity_KEGG	Score_KEGG	Evalue_KEGG
7090	WP_033276850.1	-	DSBA oxidoreductase	1	1	1	1	1	1	1	1	1	1	1	11	CORE					NA	53.3	233	1.48E-60
7091	AXI89367.1	-	TetR/AcrR family transcriptional regulator	1	1	1	1	1	1	1	1	1	1	1	11	CORE	COG1309_K#	33.3	71	3.65E-12	NA	33.3	71	9.74E-12
7092	Prokka:CIODPCDJ_04593	-	Putative threonylcarbamoyl-AMP synthase	1	1	1	1	1	1	1	1	1	1	1	11	CORE	COG0009_J#	57.2	237	3.84E-62	NA	57.2	237	1.03E-61
7093	AXI87829.1	-	TetR/AcrR family transcriptional regulator	1	1	0	1	1	1	1	1	1	1	1	10	Accessory	COG1309_K#	37.4	92	2.61E-18	NA	34.9	85	6.52E-16
7094	Prokka:JAPCLKMA_02699	hmuO	Heme oxygenase	1	1	1	1	1	1	1	1	1	1	1	11	CORE	COG5398_P#	39.5	146	1.17E-34	K00510	33.8	136	4.21E-31
7095	AXI86595.1	-	class F sortase	1	1	1	1	1	1	1	1	1	1	1	11	CORE								
7096	WP_033276782.1	-	LysM peptidoglycan-binding domain-containing protein	1	0	1	1	0	1	1	1	1	1	1	9	Accessory	COG3583_S#	53.3	42	0.00309	NA	40.4	132	3.56E-30
7097	WP_122216332.1	-	phosphoribosyltransferase	1	1	1	0	1	1	1	1	1	1	1	10	Accessory	COG1926_R#	50	187	4.56E-47	NA	50	187	1.22E-46
7098	WP_019525066.1	-	ABC transporter permease	1	1	1	1	1	1	1	1	1	1	1	11	CORE	COG1174_E#	37.1	160	7.8E-39	K05845	35.4	147	1.82E-34
7099	WP_086699961.1	-	hypothetical protein	1	1	1	1	1	1	1	1	1	1	1	11	CORE								

cluster	GI	gene	Annotation	Streptomyces_thermocarboxydus_BPSAC147	Streptomyces_sp._4F	Streptomyces_sp._BSE7F	Streptomyces_sp._ETH9427	Streptomyces_sp._FxanaD5	Streptomyces_sp._UNC401CLCol	Actinospica_acidiphila_NRRL_B_24431	Streptomyces_sp._HNS054	Streptomyces_sp._SMS_SU21	Streptomyces_sp._ZS0098	Streptomyces_thermocarboxydus_K155	Addition	Core-Accessory-Unique	COG	%Similarity_COG	Score_COG	Evalue_COG	KEGG	%Similarity_KEGG	Score_KEGG	Evalue_KEGG
7100	WP_122214569.1	-	hypothetical protein	1	1	1	1	1	1	1	1	1	1	1	11	CORE								
7101	WP_122212645.1	-	LysE family translocator	1	0	1	1	1	1	1	0	1	1	1	9	Accessory	COG1280_E#	39.1	138	3.17E-32	NA	33.3	115	7.68E-25
7102	WP_122216940.1	-	sulfite oxidase-like oxidoreductase	1	1	1	1	1	1	1	1	1	1	1	11	CORE	COG2041_R#	40.1	171	2.59E-42	K07147	41.6	165	6.47E-40
7103	WP_157842174.1	-	hypothetical protein	0	0	0	0	0	0	1	0	0	0	0	1	Unique								
7104	WP_102637514.1	-	hypothetical protein	0	0	1	0	0	0	0	0	1	1	0	3	Accessory								
7105	WP_051101715.1	-	chaplin	1	0	1	1	1	1	1	1	1	1	1	10	Accessory								
7106	OSC65694.1	-	GntR family transcriptional regulator	0	1	0	0	0	0	0	0	0	0	0	1	Unique	COG1802_K#	34.4	102	1.93E-21	NA	28.8	80	1.61E-14
7107	Prokka:CIODPCDJ_02834	fabR_1	HTH-type transcriptional repressor FabR	1	1	1	1	1	1	1	1	1	1	1	11	CORE	COG1309_K#	25.6	45	0.00028	NA	25.6	45	0.000747
7108	WP_122216038.1	-	regulator	0	0	1	1	0	0	0	1	1	1	0	5	Accessory								
7109	WP_033276364.1	-	response regulator transcription factor	0	0	0	0	0	0	1	0	0	0	0	1	Unique	COG0745_TK#	41.7	156	1.13E-37	NA	41.7	156	3.01E-37
7110	OSC60605.1	-	asparagine synthase	0	1	0	0	0	0	0	0	0	0	0	1	Unique								
7111	AXI85357.1	-	hypothetical protein	1	0	0	1	0	0	0	0	0	0	0	2	Accessory								

cluster	GI	gene	Annotation	Streptomyces_thermocarboxydus_BPSAC147	Streptomyces_sp._4F	Streptomyces_sp._BSE7F	Streptomyces_sp._ETH9427	Streptomyces_sp._FxanaD5	Streptomyces_sp._UNC401CLCol	Actinospica_acidiphila_NRRL_B_24431	Streptomyces_sp._HNS054	Streptomyces_sp._SMS_SU21	Streptomyces_sp._ZS0098	Streptomyces_thermocarboxydus_K155	Addition	Core-Accessory-Unique	COG	%Similarity_COG	Score_COG	Evalue_COG	KEGG	%Similarity_KEGG	Score_KEGG	Evalue_KEGG
7112	AXI91253.1	-	HAD family hydrolase	0	0	0	1	0	0	0	0	0	0	0	1	Unique	COG1011_R#	33.6	79	2.28E-14	K08723	31.8	66	3.13E-10
7113	OSC69636.1	-	deaminase	1	1	1	1	1	1	1	1	1	1	1	11	CORE	COG0262_H#	48.4	187	5.96E-47				
7114	AXI91390.1	-	hypothetical protein	0	0	0	1	0	0	0	0	0	0	0	1	Unique								
7115	Prokka:JAPCLKMA_03180	-	hypothetical protein	1	0	1	1	1	1	1	1	1	1	1	10	Accessory								
7116	OSC72671.1	-	50S ribosomal protein L3	1	1	1	1	1	1	1	1	1	1	1	11	CORE	COG0087_J#	71	299	1.07E-80	K02906	71	299	2.86E-80
7117	Prokka:CIODPCDJ_01896	-	hypothetical protein	1	1	1	1	1	1	1	1	1	1	1	11	CORE	COG1309_K#	29.1	87	4.89E-17	NA	29.1	87	1.31E-16
7118	WP_033274275.1	-	MBL fold metallo-hydrolase	1	0	1	1	1	1	1	1	1	0	1	9	Accessory	COG2220_R#	38.9	134	3.49E-31	NA	38.9	134	9.32E-31
7119	WP_048459351.1	-	DUF177 domain-containing protein	1	1	1	1	1	1	1	1	1	1	1	11	CORE	COG1399_R#	42.6	156	1.46E-37	K07040	42.6	156	3.91E-37
7120	PWE11316.1	-	TetR/AcrR family transcriptional regulator	1	1	1	1	1	1	1	1	1	1	1	11	CORE	COG1309_K#	45.6	190	5.36E-48	NA	45.6	190	1.43E-47
7121	WP_102638320.1	-	cholesterol esterase	1	1	1	1	1	1	1	1	1	1	1	11	CORE								
7122	OSC71518.1	-	thiamine biosynthesis protein ThiJ	0	1	0	0	0	0	0	0	0	0	0	1	Unique	COG0693_R#	38.7	139	1.42E-32	NA	33.9	101	1.14E-20
7123	WP_048457654.1	-	HNH endonuclease	1	1	0	1	1	1	1	1	1	1	1	10	Accessory					NA	35.4	111	1.44E-23

cluster	GI	gene	Annotation	Streptomyces_thermocarboxydus_BPSAC147	Streptomyces_sp._4F	Streptomyces_sp._BSE7F	Streptomyces_sp._ETH9427	Streptomyces_sp._FxanaD5	Streptomyces_sp._UNC401CLCol	Actinospica_acidiphila_NRRL_B_24431	Streptomyces_sp._HNS054	Streptomyces_sp._SMS_SU21	Streptomyces_sp._ZS0098	Streptomyces_thermocarboxydus_K155	Addition	Core-Accessory-Unique	COG	%Similarity_COG	Score_COG	Evalue_COG	KEGG	%Similarity_KEGG	Score_KEGG	Evalue_KEGG
7124	WP_048460354.1	-	FMN-binding negative transcriptional regulator	0	0	1	1	0	0	0	1	1	1	0	5	Accessory	COG2808_K#	32.1	128	3.27E-29	K07734	29.1	92	5.31E-18
7125	WP_028961742.1	-	MerR family transcriptional regulator	1	0	0	0	1	1	1	0	0	0	1	5	Accessory	COG0789_K#	28.4	51	0.00000508	NA	24.1	48	0.000115
7126	WP_063823435.1	-	ATP-binding protein	1	1	1	1	1	1	1	1	1	1	1	11	CORE								
7127	OSC70491.1	-	hypothetical protein	1	1	1	1	1	1	1	1	1	1	1	11	CORE	COG3201_H#	40.9	166	8.29E-41	K03811	26.3	82	4.21E-15
7128	OSC52802.1	-	riboflavin biosynthesis protein RibD	0	1	1	1	0	0	0	1	1	1	0	6	Accessory	COG0262_H#	48.6	188	2.66E-47				
7129	WP_019526240.1	-	DUF4232 domain-containing protein	1	0	0	0	1	1	1	0	0	1	1	6	Accessory								
7130	WP_102640167.1	-	hydrolase	1	1	1	1	1	1	1	1	1	1	1	11	CORE	COG1926_R#	56.5	209	1.12E-53	K07100	56.5	209	2.98E-53
7131	WP_026243911.1	-	hypothetical protein	0	1	0	0	1	0	0	0	0	0	1	3	Accessory								
7132	OSC70926.1	-	GNAT family N-acetyltransferase	0	1	0	0	0	0	0	0	0	0	0	1	Unique	COG1670_J#	34.5	100	9.53E-21	NA	24.7	65	9.07E-10
7133	Prokka:JAPCLKMA_02212	-	hypothetical protein	1	0	0	0	0	0	0	0	0	0	0	1	Unique					NA	34.5	97	2.15E-19
7134	WP_028959036.1	-	peptide deformylase	1	1	1	1	1	1	1	1	1	1	1	11	CORE	COG0242_J#	36.9	104	3.87E-22	K01462	41.5	137	1.44E-31

cluster	GI	gene	Annotation	Streptomyces_thermocarboxydus_BPSAC147	Streptomyces_sp._4F	Streptomyces_sp._BSE7F	Streptomyces_sp._ETH9427	Streptomyces_sp._FxanaD5	Streptomyces_sp._UNC401CLCol	Actinospica_acidiphila_NRRL_B_24431	Streptomyces_sp._HNS054	Streptomyces_sp._SMS_SU21	Streptomyces_sp._ZS0098	Streptomyces_thermocarboxydus_K155	Addition	Core-Accessory-Unique	COG	%Similarity_COG	Score_COG	Evalue_COG	KEGG	%Similarity_KEGG	Score_KEGG	Evalue_KEGG
7135	WP_033274250.1	-	CBS domain-containing protein	0	0	0	0	0	0	1	0	0	0	0	1	Unique	COG0517_R#	35.7	87	6.39E-17	NA	32.7	82	5.49E-15
7136	OSC70716.1	-	aldolase	0	1	0	0	0	0	0	0	0	1	0	2	Accessory	COG0800_G#	36.5	127	7.28E-29	K01625	34.1	121	8.17E-27
7137	Prokka:CIODPCDJ_05472	-	hypothetical protein	1	0	1	1	1	1	1	1	1	1	1	10	Accessory	COG1733_K#	49	94	4E-19	NA	49	94	1.07E-18
7138	OSC70207.1	-	hypothetical protein	1	1	1	1	1	1	1	1	1	1	1	11	CORE								
7139	OSC69651.1	-	DNA-binding response regulator	0	1	0	0	0	0	0	0	0	0	0	1	Unique	COG2197_TK#	42.4	163	9.17E-40	K02479	38.9	155	5.1E-37
7140	OSC50478.1	-	polyketide synthase	0	1	0	0	0	0	0	0	0	0	0	1	Unique								
7141	Prokka:JAPCLKMA_05983	-	hypothetical protein	1	0	1	1	1	1	1	1	1	1	1	10	Accessory								
7142	WP_033272545.1	-	hypothetical protein	1	1	0	0	1	1	1	0	0	0	1	6	Accessory								
7143	OSC62341.1	-	type III effector protein	0	1	0	0	0	0	0	0	0	0	0	1	Unique								
7144	WP_026243421.1	-	hypothetical protein	1	1	1	1	1	1	1	1	1	1	1	11	CORE								
7145	Prokka:JAPCLKMA_01637	-	hypothetical protein	1	0	0	1	0	0	0	0	0	0	0	2	Accessory								
7146	WP_019522756.1	-	TetR/AcrR family transcriptional regulator	1	1	1	1	1	1	1	1	1	1	1	11	CORE	COG1309_K#	28.4	87	6.36E-17	NA	28.4	87	1.7E-16
7147	WP_048456959.1	sepF	cell division protein SepF	1	1	1	1	1	1	1	1	1	1	1	11	CORE	COG1799_S#	38.2	131	3.84E-30	K09772	38.2	131	1.03E-29

cluster	GI	gene	Annotation	Streptomyces_thermocarboxydus_BPSAC147	Streptomyces_sp._4F	Streptomyces_sp._BSE7F	Streptomyces_sp._ETH9427	Streptomyces_sp._FxanaD5	Streptomyces_sp._UNC401CLCol	Actinospica_acidiphila_NRRL_B_24431	Streptomyces_sp._HNS054	Streptomyces_sp._SMS_SU21	Streptomyces_sp._ZS0098	Streptomyces_thermocarboxydus_K155	Addition	Core-Accessory-Unique	COG	%Similarity_COG	Score_COG	Evalue_COG	KEGG	%Similarity_KEGG	Score_KEGG	Evalue_KEGG
7148	AXI86016.1	-	DNA-binding response regulator	1	1	1	1	1	1	1	1	1	1	1	11	CORE	COG2197_TK#	49.3	196	1.27E-49	K11618	50.7	194	1.29E-48
7149	Prokka:JAPCLKMA_00138	-	hypothetical protein	1	1	1	1	1	1	1	1	1	1	1	11	CORE								
7150	AXI86271.1	-	signal peptidase II	1	1	1	1	1	1	1	1	1	1	1	11	CORE	COG0597_MU#	43	146	1.51E-34	K03101	43	146	4.03E-34
7151	WP_122213435.1	-	carbonic anhydrase	0	0	1	1	1	1	1	1	1	1	1	9	Accessory	COG0288_P#	57.3	216	6.95E-56	K01673	57.3	216	1.86E-55
7152	WP_028959416.1	-	hypothetical protein	0	0	0	0	0	1	0	1	1	0	1	4	Accessory								
7153	OSC70659.1	-	hypothetical protein	0	1	0	0	0	0	0	0	0	0	0	1	Unique								
7154	WP_102637507.1	-	sigma-70 family RNA polymerase sigma factor	0	1	1	1	1	1	1	1	1	1	1	10	Accessory	COG1595_K#	50.6	156	1.12E-37	K03088	47.3	151	9.58E-36
7155	WP_033275431.1	-	superoxide dismutase	0	1	1	1	1	1	1	1	1	1	1	10	Accessory	COG0605_P#	54.2	235	2.47E-61	K04564	54.2	235	6.59E-61
7156	AXI88124.1	-	NADH-quinone oxidoreductase subunit NuoI	1	1	1	1	1	1	1	1	1	1	1	11	CORE	COG1143_C#	67.4	257	6.06E-68	K00338	67.4	257	1.62E-67
7157	WP_028960943.1	-	hypothetical protein	1	1	1	1	1	1	1	1	1	1	1	11	CORE								
7158	WP_048459248.1	-	PepSY domain-containing protein	1	0	1	1	1	1	1	1	1	1	1	10	Accessory								

cluster	GI	gene	Annotation	Streptomyces_thermocarboxydus_BPSAC147	Streptomyces_sp._4F	Streptomyces_sp._BSE7F	Streptomyces_sp._ETH9427	Streptomyces_sp._FxanaD5	Streptomyces_sp._UNC401CLCol	Actinospica_acidiphila_NRRL_B_24431	Streptomyces_sp._HNS054	Streptomyces_sp._SMS_SU21	Streptomyces_sp._ZS0098	Streptomyces_thermocarboxydus_K155	Addition	Core-Accessory-Unique	COG	%Similarity_COG	Score_COG	Evalue_COG	KEGG	%Similarity_KEGG	Score_KEGG	Evalue_KEGG
7159	WP_102640572.1	-	methyltransferase domain-containing protein	1	1	1	1	1	1	1	1	1	1	1	11	CORE	COG0500_QR#	27	60	6.38E-09	K18534	27	60	1.7E-08
7160	WP_033274910.1	-	PadR family transcriptional regulator	1	1	1	1	0	0	1	1	1	1	0	8	Accessory								
7161	WP_019522079.1	-	DNA-3-methyladenine glycosylase	1	1	1	1	1	1	1	1	1	1	1	11	CORE	COG2094_L#	42.2	136	1.19E-31	K03652	42.2	136	3.19E-31
7162	AXI85306.1	-	WhiB family transcriptional regulator	1	0	0	1	0	0	0	0	0	0	0	2	Accessory					K18955	41.3	55	5.48E-07
7163	WP_122215297.1	-	hypothetical protein	0	0	0	1	0	0	0	0	0	1	0	2	Accessory								
7164	Prokka:CIODPCDJ_04263	fadR	Fatty acid metabolism regulator protein	1	0	1	1	1	1	1	1	1	1	1	10	Accessory	COG1309_K#	33.5	70	1.05E-11	NA	33.5	70	2.81E-11
7165	AXI85310.1	-	plasmid mobilization relaxosome protein MobC	0	0	0	1	0	0	0	0	0	0	0	1	Unique								
7166	OSC69644.1	-	TetR family transcriptional regulator	0	1	0	0	0	0	0	0	0	0	0	1	Unique	COG1309_K#	35.6	101	3.26E-21	NA	33.3	87	1.7E-16

cluster	GI	gene	Annotation	Streptomyces_thermocarboxydus_BPSAC147	Streptomyces_sp._4F	Streptomyces_sp._BSE7F	Streptomyces_sp._ETH9427	Streptomyces_sp._FxanaD5	Streptomyces_sp._UNC401CLCol	Actinospica_acidiphila_NRRL_B_24431	Streptomyces_sp._HNS054	Streptomyces_sp._SMS_SU21	Streptomyces_sp._ZS0098	Streptomyces_thermocarboxydus_K155	Addition	Core-Accessory-Unique	COG	%Similarity_COG	Score_COG	Evalue_COG	KEGG	%Similarity_KEGG	Score_KEGG	Evalue_KEGG
7167	AXI90653.1	-	hypothetical protein	0	0	0	1	0	0	0	0	0	0	0	1	Unique								
7168	WP_102641633.1	-	hypothetical protein	0	0	0	1	0	0	0	1	1	1	1	5	Accessory								
7169	OSC60090.1	-	hypothetical protein	0	1	0	0	0	0	0	0	0	0	0	1	Unique					NA	34.2	106	3.54E-22
7170	AXI91359.1	-	beta-carotene 15,15'-monooxygenase	0	0	0	1	0	0	0	0	0	1	0	2	Accessory								
7171	Prokka:JAPCLKMA_01795	-	hypothetical protein	1	1	1	1	1	1	1	1	1	1	1	11	CORE								
7172	WP_033274392.1	-	response regulator transcription factor	0	0	0	0	0	0	1	0	0	0	0	1	Unique	COG2197_TK#	32.4	91	4.41E-18	K02479	28.2	79	3.54E-14
7173	WP_122217369.1	-	pentapeptide repeat-containing protein	0	0	0	1	0	0	0	1	0	1	0	3	Accessory	COG1357_S#	34.9	72	2.12E-12	NA	34.9	72	5.66E-12
7174	OSC65793.1	-	hypothetical protein	0	1	1	1	0	0	0	1	1	1	0	6	Accessory								
7175	WP_019526018.1	-	TetR/AcrR family transcriptional regulator	1	0	0	1	1	1	1	1	0	1	1	8	Accessory	COG1309_K#	29.4	83	1.2E-15	NA	31.7	81	1.22E-14
7176	Prokka:JAPCLKMA_02545	-	hypothetical protein	1	0	0	1	1	1	1	1	0	0	0	6	Accessory								
7177	OSC66602.1	-	TetR family transcriptional regulator	0	1	0	0	0	0	0	0	0	0	0	1	Unique	COG1309_K#	28.8	73	1.24E-12	NA	35.6	62	4.48E-09

cluster	GI	gene	Annotation	Streptomyces_thermocarboxydus_BPSAC147	Streptomyces_sp._4F	Streptomyces_sp._BSE7F	Streptomyces_sp._ETH9427	Streptomyces_sp._FxanaD5	Streptomyces_sp._UNC401CLCol	Actinospica_acidiphila_NRRL_B_24431	Streptomyces_sp._HNS054	Streptomyces_sp._SMS_SU21	Streptomyces_sp._ZS0098	Streptomyces_thermocarboxydus_K155	Addition	Core-Accessory-Unique	COG	%Similarity_COG	Score_COG	Evalue_COG	KEGG	%Similarity_KEGG	Score_KEGG	Evalue_KEGG
7178	Prokka:CIODPCDJ_00435	-	hypothetical protein	1	1	1	1	1	1	1	1	1	0	0	9	Accessory	COG3145_L#	58.3	241	3.43E-63	NA	58.3	241	9.15E-63
7179	WP_122216541.1	-	hypothetical protein	1	0	1	1	1	1	1	1	1	1	1	10	Accessory								
7180	AXI84676.1	-	DUF4291 domain-containing protein	0	1	0	1	1	1	0	0	1	1	0	6	Accessory								
7181	WP_102640920.1	-	aminodeoxychorismate/anthranilate synthase component II	1	1	1	1	1	1	1	1	1	1	1	11	CORE	COG0512_EH#	62.7	257	6.04E-68	K01664	65.6	251	8.84E-66
7182	WP_026243965.1	-	DUF998 domain-containing protein	1	1	1	1	1	1	1	1	1	1	1	11	CORE								
7183	OSC68205.1	-	lysine transporter LysE	0	1	0	0	0	0	0	0	0	0	0	1	Unique	COG1280_E#	33.5	97	6.12E-20	NA	32.2	87	1.3E-16
7184	Prokka:JAPCLKMA_05327	-	hypothetical protein	1	1	1	1	1	1	1	1	1	1	1	11	CORE	COG1396_K#	31.3	81	3.48E-15	NA	25	70	1.64E-11
7185	WP_019523233.1	cofC	2-phospho-L-lactate guanylyltransferase	1	1	1	1	1	1	0	1	1	1	1	10	Accessory								
7186	WP_028960731.1	-	hypothetical protein	1	1	1	1	1	1	1	1	1	1	1	11	CORE								
7187	Prokka:CIODPCDJ_05921	-	hypothetical protein	1	1	1	1	1	1	1	1	1	1	1	11	CORE	COG1595_K#	22.2	42	0.00179				
7188	AXI90129.1	-	lysine transporter LysE	1	0	0	1	1	1	1	1	1	1	1	9	Accessory	COG1280_E#	28.5	53	0.00000132				

cluster	GI	gene	Annotation	Streptomyces_thermocarboxydus_BPSAC147	Streptomyces_sp._4F	Streptomyces_sp._BSE7F	Streptomyces_sp._ETH9427	Streptomyces_sp._FxanaD5	Streptomyces_sp._UNC401CLCol	Actinospica_acidiphila_NRRL_B_24431	Streptomyces_sp._HNS054	Streptomyces_sp._SMS_SU21	Streptomyces_sp._ZS0098	Streptomyces_thermocarboxydus_K155	Addition	Core-Accessory-Unique	COG	%Similarity_COG	Score_COG	Evalue_COG	KEGG	%Similarity_KEGG	Score_KEGG	Evalue_KEGG
7189	WP_122215726.1	-	RNA polymerase sigma factor	0	0	0	0	0	0	0	1	0	1	0	2	Accessory	COG1595_K#	36.4	89	2.18E-17	K03088	33.9	84	1.87E-15
7190	WP_033276253.1	-	hypothetical protein	0	0	0	0	0	0	1	0	0	0	0	1	Unique								
7191	OSC66752.1	-	hypothetical protein	0	1	0	0	0	0	0	0	0	0	0	1	Unique								
7192	Prokka:JAPCLKMA_00820	-	hypothetical protein	1	1	1	0	1	1	1	1	1	1	1	10	Accessory								
7193	WP_028961344.1	-	hypothetical protein	0	0	0	0	0	1	0	0	0	0	1	2	Accessory								
7194	WP_028959372.1	-	Uma2 family endonuclease	1	0	1	1	1	1	1	1	1	1	1	10	Accessory								
7195	WP_048458862.1	-	hypothetical protein	0	0	0	0	0	0	0	1	0	0	0	1	Unique								
7196	WP_048460194.1	-	glutamine amidotransferase	0	1	0	0	0	0	0	1	1	0	0	3	Accessory	COG0693_R#	44.4	156	1.45E-37	NA	42.3	129	2.97E-29
7197	WP_146217780.1	-	hypothetical protein	0	0	0	0	0	0	0	0	1	0	0	1	Unique					NA	36.4	72	5.63E-12
7198	WP_020700248.1	-	hypothetical protein	0	0	0	0	1	0	0	0	0	0	0	1	Unique								
7199	WP_106959433.1	-	hypothetical protein	1	1	0	1	1	1	1	1	1	1	1	10	Accessory								
7200	WP_063823445.1	-	hypothetical protein	0	0	0	0	1	0	0	0	0	0	0	1	Unique								
7201	OSC69388.1	-	arylsulfatase	0	1	0	0	0	0	0	0	0	0	0	1	Unique								
7202	WP_078611360.1	-	hypothetical protein	0	1	1	1	1	1	1	0	1	1	0	8	Accessory	COG0515_RTKL#	38.3	82	1.56E-15	NA	50.9	122	4.74E-27
7203	WP_033274781.1	-	DUF4255 domain-containing protein	0	1	0	0	0	0	1	0	0	0	0	2	Accessory								

cluster	GI	gene	Annotation	Streptomyces_thermocarboxydus_BPSAC147	Streptomyces_sp._4F	Streptomyces_sp._BSE7F	Streptomyces_sp._ETH9427	Streptomyces_sp._FxanaD5	Streptomyces_sp._UNC401CLCol	Actinospica_acidiphila_NRRL_B_24431	Streptomyces_sp._HNS054	Streptomyces_sp._SMS_SU21	Streptomyces_sp._ZS0098	Streptomyces_thermocarboxydus_K155	Addition	Core-Accessory-Unique	COG	%Similarity_COG	Score_COG	Evalue_COG	KEGG	%Similarity_KEGG	Score_KEGG	Evalue_KEGG
7204	OSC69344.1	-	TetR family transcriptional regulator	0	1	1	1	0	0	0	1	1	0	0	5	Accessory	COG1309_K#	42.7	144	3.34E-34	NA	33.8	123	2.13E-27
7205	WP_019527288.1	-	TetR/AcrR family transcriptional regulator	1	0	1	1	1	1	1	1	1	1	1	10	Accessory	COG1309_K#	40.9	130	8.48E-30	NA	40.9	130	2.26E-29
7206	WP_019527028.1	-	uracil phosphoribosyltransferase	1	0	1	1	1	1	1	1	1	1	1	10	Accessory	COG0035_F#	54.3	218	2.37E-56	K00761	54.3	218	6.32E-56
7207	WP_122212749.1	-	FMN-binding negative transcriptional regulator	1	1	1	1	1	1	1	1	1	1	1	11	CORE								
7208	WP_031117445.1	queE	7-carboxy-7-deazaguanine synthase	0	0	0	0	0	0	0	0	0	1	0	1	Unique	COG0602_O#	67.1	299	1.06E-80	NA	42.7	146	2.34E-34
7209	AXI86243.1	-	TIGR03085 family protein	1	1	1	1	1	1	1	1	1	1	1	11	CORE					NA	39.7	125	5.58E-28
7210	Prokka:JAPCLKMA_03420	-	hypothetical protein	1	1	1	1	1	1	1	1	1	1	1	11	CORE	COG1309_K#	27.4	79	1.31E-14	NA	27.4	79	3.51E-14
7211	WP_102641480.1	-	5-formyltetrahydrofolate cyclo-ligase	1	1	1	1	1	1	1	1	1	1	1	11	CORE	COG0212_H#	36.8	124	3.56E-28	NA	40.4	108	7.06E-23
7212	OSC70067.1	-	dTMP kinase	0	1	0	0	0	0	0	0	0	0	0	1	Unique								

cluster	GI	gene	Annotation	Streptomyces_thermocarboxydus_BPSAC147	Streptomyces_sp._4F	Streptomyces_sp._BSE7F	Streptomyces_sp._ETH9427	Streptomyces_sp._FxanaD5	Streptomyces_sp._UNC401CLCol	Actinospica_acidiphila_NRRL_B_24431	Streptomyces_sp._HNS054	Streptomyces_sp._SMS_SU21	Streptomyces_sp._ZS0098	Streptomyces_thermocarboxydus_K155	Addition	Core-Accessory-Unique	COG	%Similarity_COG	Score_COG	Evalue_COG	KEGG	%Similarity_KEGG	Score_KEGG	Evalue_KEGG
7213	Prokka:JAPCLKMA_01202	-	hypothetical protein	1	0	0	0	1	1	0	0	0	0	0	3	Accessory	COG1309_K#	43.9	145	2.55E-34	NA	37.3	100	2.51E-20
7214	WP_102637618.1	-	TetR family transcriptional regulator	0	1	0	1	0	0	1	1	1	1	0	6	Accessory								
7215	OSC66658.1	-	hypothetical protein	0	1	0	0	0	0	0	0	0	0	0	1	Unique								
7216	OSC55609.1	-	hypothetical protein	0	1	0	0	0	0	0	0	0	0	0	1	Unique								
7217	WP_122215807.1	-	hypothetical protein	0	0	0	0	0	0	0	0	0	1	0	1	Unique								
7218	WP_051101655.1	-	TetR family transcriptional regulator	0	0	1	1	1	1	1	1	1	1	0	8	Accessory	COG1309_K#	31.2	69	1.78E-11	NA	27.1	65	8.95E-10
7219	WP_107080805.1	-	hypothetical protein	1	1	1	1	1	1	1	1	1	1	1	11	CORE								
7220	OSC72050.1	-	TetR family transcriptional regulator	1	1	1	1	1	1	1	1	1	1	1	11	CORE	COG1309_K#	51.4	169	1.65E-41	NA	51.4	169	4.4E-41
7221	WP_033275430.1	-	ATP-binding cassette domain-containing protein	0	0	0	0	1	1	1	0	0	0	1	4	Accessory	COG1120_PH#	35	106	1E-22	K02003	36	106	4.57E-22
7222	Prokka:JAPCLKMA_05406	-	hypothetical protein	1	0	0	0	0	0	0	0	0	0	0	1	Unique								
7223	Prokka:JAPCLKMA_03068	-	hypothetical protein	1	1	1	1	1	1	1	1	1	1	1	11	CORE								

cluster	GI	gene	Annotation	Streptomyces_thermocarboxydus_BPSAC147	Streptomyces_sp._4F	Streptomyces_sp._BSE7F	Streptomyces_sp._ETH9427	Streptomyces_sp._FxanaD5	Streptomyces_sp._UNC401CLCol	Actinospica_acidiphila_NRRL_B_24431	Streptomyces_sp._HNS054	Streptomyces_sp._SMS_SU21	Streptomyces_sp._ZS0098	Streptomyces_thermocarboxydus_K155	Addition	Core-Accessory-Unique	COG	%Similarity_COG	Score_COG	Evalue_COG	KEGG	%Similarity_KEGG	Score_KEGG	Evalue_KEGG
7224	WP_147474359.1	-	hypothetical protein	0	0	0	0	0	0	0	0	0	1	0	1	Unique								
7225	WP_106959455.1	-	dirigent protein	1	0	0	0	1	1	0	0	0	1	1	5	Accessory								
7226	OSC71629.1	-	SAM-dependent methyltransferase	0	1	0	0	0	0	0	0	0	0	0	1	Unique	COG0500_QR#	39	95	3.02E-19	NA	34.3	63	3.4E-09
7227	AXI91406.1	-	hypothetical protein	0	0	0	1	0	0	0	0	0	0	0	1	Unique								
7228	WP_122214502.1	-	alpha/beta hydrolase	0	0	0	0	0	0	0	0	0	1	0	1	Unique								
7229	OSC71482.1	-	hypothetical protein	0	1	0	0	0	0	0	0	0	0	0	1	Unique								
7230	AXI91104.1	-	hypothetical protein	0	0	0	1	0	0	0	0	0	0	0	1	Unique								
7231	OSC57885.1	-	ADP-ribose pyrophosphatase	1	1	1	1	1	1	1	1	1	1	1	11	CORE	COG0494_LR#	44.3	155	2.46E-37	K01515	41.5	154	1.46E-36
7232	OSC69733.1	-	aminoacyl-tRNA hydrolase	1	1	1	1	1	1	1	1	1	1	1	11	CORE	COG0193_J#	52.1	203	6.03E-52	K01056	52.1	203	1.61E-51
7233	Prokka:JAPCLKMA_00206	-	hypothetical protein	1	1	1	0	1	1	1	1	1	1	1	10	Accessory								
7234	Prokka:JAPCLKMA_05688	-	hypothetical protein	1	0	0	0	0	0	0	0	0	0	0	1	Unique								
7235	Prokka:CIODPCDJ_03649	-	hypothetical protein	1	1	1	1	1	1	1	1	1	1	1	11	CORE								
7236	OSC63111.1	-	RNA polymerase subunit sigma-70	0	1	0	0	0	0	0	0	0	0	0	1	Unique	COG1595_K#	38	92	2.55E-18	K03088	38	92	6.8E-18

cluster	GI	gene	Annotation	Streptomyces_thermocarboxydus_BPSAC147	Streptomyces_sp._4F	Streptomyces_sp._BSE7F	Streptomyces_sp._ETH9427	Streptomyces_sp._FxanaD5	Streptomyces_sp._UNC401CLCol	Actinospica_acidiphila_NRRL_B_24431	Streptomyces_sp._HNS054	Streptomyces_sp._SMS_SU21	Streptomyces_sp._ZS0098	Streptomyces_thermocarboxydus_K155	Addition	Core-Accessory-Unique	COG	%Similarity_COG	Score_COG	Evalue_COG	KEGG	%Similarity_KEGG	Score_KEGG	Evalue_KEGG
7237	WP_033272986.1	-	TetR/AcrR family transcriptional regulator	1	1	1	1	1	1	1	1	1	1	1	11	CORE	COG1309_K#	32.1	87	8.19E-17	NA	23.8	72	7.28E-12
7238	WP_053076920.1	-	TetR/AcrR family transcriptional regulator	0	0	1	1	0	0	0	1	1	1	0	5	Accessory	COG1309_K#	27.8	62	2.16E-09	NA	37	45	0.000559
7239	WP_048459253.1	-	AIM24 family protein	1	1	0	1	1	1	1	1	1	1	1	10	Accessory	COG2013_S#	36.9	140	6.24E-33	NA	36.9	140	1.67E-32
7240	WP_033275335.1	-	LUD domain-containing protein	1	1	1	1	1	1	1	1	1	1	1	11	CORE	COG1556_S#	43.6	154	5.46E-37	K00782	43.6	154	1.46E-36
7241	WP_033272729.1	-	hypothetical protein	0	0	1	1	1	1	1	1	1	1	1	9	Accessory								
7242	WP_102637398.1	-	hypothetical protein	0	0	0	0	0	0	0	0	1	0	0	1	Unique								
7243	WP_048459616.1	-	hypothetical protein	0	0	0	0	0	0	0	1	0	0	0	1	Unique								
7244	WP_051101614.1	-	NADAR family protein	1	1	1	1	1	1	1	1	1	1	1	11	CORE	COG3236_S#	57.7	204	3.52E-52	K09935	40.3	118	6.79E-26
7245	WP_147474375.1	-	hypothetical protein	0	0	0	0	0	0	0	1	0	1	0	2	Accessory								
7246	WP_102637403.1	-	hypothetical protein	0	0	0	0	0	0	0	0	1	0	0	1	Unique								
7247	OSC55544.1	-	hypothetical protein	0	1	0	0	0	0	0	0	0	0	0	1	Unique	COG1309_K#	25.8	52	0.00000171	NA	30.9	50	0.0000174
7248	WP_033284751.1	-	hypothetical protein	0	0	0	0	0	0	0	0	1	0	0	1	Unique								
7249	OSC58526.1	-	metal transporter	0	1	0	0	0	0	0	0	0	0	0	1	Unique	COG1226_P#	32.5	55	3.45E-07	NA	28.8	48	0.000113
7250	OSC64659.1	-	epimerase	0	1	0	0	0	0	0	0	0	0	0	1	Unique	COG2910_R#	33.2	86	1.83E-16	K05901	34.8	93	3.05E-18

cluster	GI	gene	Annotation	Streptomyces_thermocarboxydus_BPSAC147	Streptomyces_sp._4F	Streptomyces_sp._BSE7F	Streptomyces_sp._ETH9427	Streptomyces_sp._FxanaD5	Streptomyces_sp._UNC401CLCol	Actinospica_acidiphila_NRRL_B_24431	Streptomyces_sp._HNS054	Streptomyces_sp._SMS_SU21	Streptomyces_sp._ZS0098	Streptomyces_thermocarboxydus_K155	Addition	Core-Accessory-Unique	COG	%Similarity_COG	Score_COG	Evalue_COG	KEGG	%Similarity_KEGG	Score_KEGG	Evalue_KEGG
7251	Prokka:JAPCLKMA_04937	-	hypothetical protein	1	0	0	0	0	0	0	0	0	0	0	1	Unique								
7252	WP_122215669.1	-	hypothetical protein	0	0	0	0	0	0	0	0	0	1	0	1	Unique								
7253	OSC62614.1	-	siderophore biosynthesis protein	1	1	1	1	1	1	1	1	1	1	1	11	CORE	COG1670_J#	33.5	90	9.68E-18				
7254	WP_107080822.1	-	hypothetical protein	0	0	0	1	0	0	0	1	1	0	0	3	Accessory								
7255	OSC69346.1	-	ABC transporter ATP-binding protein	0	1	0	0	0	0	0	0	0	0	0	1	Unique	COG3842_E#	31.1	91	5.67E-18	K10004	30.5	91	1.16E-17
7256	AXI88037.1	-	glyoxalase	1	1	1	1	1	1	1	1	1	1	1	11	CORE								
7257	Prokka:JAPCLKMA_01622	-	hypothetical protein	1	0	0	0	0	0	0	0	0	0	0	1	Unique	COG2020_O#	50.8	123	1.03E-27	NA	50.8	123	2.74E-27
7258	WP_147474348.1	-	hypothetical protein	1	0	1	1	1	1	0	1	1	1	1	9	Accessory								
7259	PWE08142.1	-	TetR family transcriptional regulator	1	1	1	1	0	1	1	1	1	1	1	10	Accessory	COG1309_K#	32.2	71	3.55E-12	NA	35.6	50	0.0000226
7260	WP_048454891.1	-	MBL fold metallo-hydrolase	1	1	1	1	1	1	1	1	1	1	1	11	CORE	COG0491_R#	57.7	266	9.81E-71	NA	58.2	264	7.62E-70
7261	WP_019526053.1	-	hypothetical protein	0	0	0	0	1	0	0	0	0	1	0	2	Accessory								
7262	AXI87619.1	-	hypothetical protein	1	0	1	1	1	1	1	1	1	1	1	10	Accessory								
7263	Prokka:JAPCLKMA_02746	-	hypothetical protein	1	0	0	1	0	0	0	0	0	0	0	2	Accessory	COG0671_I#	25.3	52	0.0000017	NA	25.3	52	0.00000455

cluster	GI	gene	Annotation	Streptomyces_thermocarboxydus_BPSAC147	Streptomyces_sp._4F	Streptomyces_sp._BSE7F	Streptomyces_sp._ETH9427	Streptomyces_sp._FxanaD5	Streptomyces_sp._UNC401CLCol	Actinospica_acidiphila_NRRL_B_24431	Streptomyces_sp._HNS054	Streptomyces_sp._SMS_SU21	Streptomyces_sp._ZS0098	Streptomyces_thermocarboxydus_K155	Addition	Core-Accessory-Unique	COG	%Similarity_COG	Score_COG	Evalue_COG	KEGG	%Similarity_KEGG	Score_KEGG	Evalue_KEGG
7264	AXI85322.1	-	SAM-dependent methyltransferase	0	0	0	1	0	0	0	0	0	0	0	1	Unique	COG0500_QR#	32.3	59	1.82E-08	NA	30.6	54	0.00000204
7265	WP_086020183.1	-	Tat pathway signal sequence domain protein	1	0	1	1	1	1	1	0	1	1	1	9	Accessory								
7266	OSC73286.1	-	beta-N-acetylhexosaminidase	0	1	0	0	0	0	0	0	0	0	0	1	Unique								
7267	Prokka:JAPCLKMA_01552	gph_1	Phosphoglycolate phosphatase	1	0	0	1	0	0	0	0	0	0	0	2	Accessory								
7268	Prokka:CIODPCDJ_02934	-	hypothetical protein	1	0	0	0	1	1	1	0	0	0	1	5	Accessory								
7269	WP_122216538.1	-	hypothetical protein	1	0	0	1	1	1	1	1	1	1	1	9	Accessory								
7270	WP_102640666.1	-	class I SAM-dependent methyltransferase	0	0	0	0	0	0	0	0	1	0	0	1	Unique					NA	32.7	49	0.0000503
7271	WP_020700116.1	-	3-hexulose-6-phosphate synthase	0	0	0	0	1	0	0	0	0	0	0	1	Unique	COG0269_G#	40.2	161	3.4E-39	K08093	39.6	148	7.96E-35
7272	Prokka:JAPCLKMA_02523	-	hypothetical protein	1	0	0	0	1	1	0	0	0	0	1	4	Accessory								

cluster	GI	gene	Annotation	Streptomyces_thermocarboxydus_BPSAC147	Streptomyces_sp._4F	Streptomyces_sp._BSE7F	Streptomyces_sp._ETH9427	Streptomyces_sp._FxanaD5	Streptomyces_sp._UNC401CLCol	Actinospica_acidiphila_NRRL_B_24431	Streptomyces_sp._HNS054	Streptomyces_sp._SMS_SU21	Streptomyces_sp._ZS0098	Streptomyces_thermocarboxydus_K155	Addition	Core-Accessory-Unique	COG	%Similarity_COG	Score_COG	Evalue_COG	KEGG	%Similarity_KEGG	Score_KEGG	Evalue_KEGG
7273	OSC71723.1	-	TetR family transcriptional regulator	1	1	1	1	1	1	1	1	1	1	1	11	CORE	COG1309_K#	28.6	64	4.33E-10	NA	34.2	60	1.67E-08
7274	OSC67359.1	-	SAM-dependent methyltransferase	1	1	1	1	1	1	1	1	1	1	1	11	CORE	COG0500_QR#	44.6	135	2.61E-31	NA	37.9	80	2.66E-14
7275	WP_084207078.1	-	ATP-binding protein	1	1	1	1	1	1	1	1	1	1	1	11	CORE								
7276	OSC59905.1	-	SAM-dependent methyltransferase	0	1	0	0	0	0	0	0	0	0	0	1	Unique	COG0500_QR#	32.5	84	5.29E-16	K18534	31.2	65	6.79E-10
7277	OSC63559.1	-	MFS transporter	0	1	0	0	0	0	0	0	0	0	0	1	Unique	COG0477_GEPR#	30.2	86	1.39E-16	NA	31.4	82	4.11E-15
7278	PWE11712.1	-	YigZ family protein	1	1	1	1	1	1	1	1	1	1	1	11	CORE	COG1739_S#	47.3	201	3.86E-51	NA	47.3	201	1.03E-50
7279	Prokka:JAPCLKMA_06497	-	hypothetical protein	1	0	0	0	0	0	0	0	0	0	0	1	Unique								
7280	Prokka:JAPCLKMA_00893	betI_4	HTH-type transcriptional regulator BetI	1	0	0	0	1	1	1	0	0	0	1	5	Accessory	COG1309_K#	31	58	3.09E-08	NA	28.1	54	0.00000203
7281	WP_019526710.1	-	hypothetical protein	1	1	1	0	1	1	1	1	1	1	1	10	Accessory	COG0810_M#	32.9	53	0.0000013	NA	43.1	46	0.000424
7282	AXI91351.1	-	hypothetical protein	0	0	0	1	0	0	0	0	0	0	0	1	Unique								
7283	Prokka:JAPCLKMA_02468	gmhA	Phosphoheptose isomerase	1	1	1	1	1	1	1	1	1	1	1	11	CORE	COG0279_G#	47.1	144	5.59E-34	K03271	47.1	144	1.49E-33

cluster	GI	gene	Annotation	Streptomyces_thermocarboxydus_BPSAC147	Streptomyces_sp._4F	Streptomyces_sp._BSE7F	Streptomyces_sp._ETH9427	Streptomyces_sp._FxanaD5	Streptomyces_sp._UNC401CLCol	Actinospica_acidiphila_NRRL_B_24431	Streptomyces_sp._HNS054	Streptomyces_sp._SMS_SU21	Streptomyces_sp._ZS0098	Streptomyces_thermocarboxydus_K155	Addition	Core-Accessory-Unique	COG	%Similarity_COG	Score_COG	Evalue_COG	KEGG	%Similarity_KEGG	Score_KEGG	Evalue_KEGG
7284	PWE10892.1	-	dihydrofolate reductase	1	0	1	1	1	1	1	1	1	1	1	10	Accessory	COG0262_H#	36.9	120	6.62E-27	NA	24	57	1.41E-07
7285	Prokka:CIODPCDJ_06739	-	hypothetical protein	0	0	0	0	1	0	0	0	0	0	1	2	Accessory								
7286	WP_033273659.1	-	class I SAM-dependent methyltransferase	0	1	1	1	0	0	1	1	1	1	0	7	Accessory	COG0500_QR#	25.9	55	2.62E-07	K16868	30.2	53	0.00000347
7287	WP_122215813.1	-	HD domain-containing protein	1	0	1	1	1	1	1	1	1	1	1	10	Accessory					K06950	35.8	90	2.56E-17
7288	WP_158688255.1	-	hypothetical protein	0	0	0	0	1	1	0	0	0	0	0	2	Accessory								
7289	WP_019524682.1	-	response regulator transcription factor	1	0	0	0	1	1	0	0	0	0	1	4	Accessory	COG2197_TK#	34.1	118	3.29E-26	K11624	29.7	115	7.43E-25
7290	WP_078611435.1	-	DUF393 domain-containing protein	1	1	1	1	1	1	1	1	1	1	1	11	CORE								
7291	OSC69005.1	-	hypothetical protein	0	1	0	0	0	0	0	0	0	0	0	1	Unique	COG2197_TK#	35.1	72	2.07E-12	NA	31	66	3.03E-10
7292	AXI90592.1	-	hypothetical protein	0	0	1	1	0	0	0	1	1	1	0	5	Accessory								
7293	WP_048458585.1	-	hypothetical protein	0	0	0	0	0	0	0	1	0	0	0	1	Unique								
7294	PWE11296.1	-	hypothetical protein	1	1	1	1	1	1	1	1	1	1	1	11	CORE								
7295	OSC70189.1	-	TetR family transcriptional regulator	0	1	0	0	0	0	0	0	0	0	0	1	Unique	COG1309_K#	29.5	59	1.39E-08	NA	29.5	59	3.71E-08

cluster	GI	gene	Annotation	Streptomyces_thermocarboxydus_BPSAC147	Streptomyces_sp._4F	Streptomyces_sp._BSE7F	Streptomyces_sp._ETH9427	Streptomyces_sp._FxanaD5	Streptomyces_sp._UNC401CLCol	Actinospica_acidiphila_NRRL_B_24431	Streptomyces_sp._HNS054	Streptomyces_sp._SMS_SU21	Streptomyces_sp._ZS0098	Streptomyces_thermocarboxydus_K155	Addition	Core-Accessory-Unique	COG	%Similarity_COG	Score_COG	Evalue_COG	KEGG	%Similarity_KEGG	Score_KEGG	Evalue_KEGG
7296	OSC72202.1	-	hypothetical protein	0	1	0	0	0	0	0	0	0	0	0	1	Unique	COG2771_K#	29.5	46	0.000159				
7297	WP_102639840.1	-	ATP-binding protein	0	0	0	0	0	0	0	0	1	0	0	1	Unique								
7298	WP_033274554.1	-	hypothetical protein	0	0	0	0	0	0	1	0	0	0	0	1	Unique								
7299	OSC57417.1	-	hypothetical protein	0	1	0	0	0	0	0	0	0	0	0	1	Unique								
7300	OSC71757.1	-	hypothetical protein	0	1	0	0	0	0	0	0	0	0	0	1	Unique	COG0662_G#	33	42	0.00229				
7301	WP_051724552.1	-	hypothetical protein	0	0	0	0	0	0	1	0	0	0	0	1	Unique								
7302	AXI86364.1	-	GntR family transcriptional regulator	1	1	1	1	1	1	1	1	1	1	1	11	CORE	COG1802_K#	33.3	99	2.05E-20	NA	34	94	1.76E-18
7303	WP_102637360.1	-	GNAT family N-acetyltransferase	1	1	1	1	1	1	1	1	1	1	1	11	CORE	COG0454_KR#	35.7	90	9.54E-18	K00680	35.7	90	2.55E-17
7304	WP_048455948.1	-	hypothetical protein	1	0	0	1	1	0	0	1	0	0	1	5	Accessory								
7305	WP_086020170.1	-	hypothetical protein	0	0	0	0	1	0	0	0	0	0	0	1	Unique								
7306	WP_102637201.1	-	TetR/AcrR family transcriptional regulator	1	1	1	1	1	1	1	1	1	1	1	11	CORE	COG1309_K#	33.3	109	1.98E-23	NA	33.3	109	5.3E-23
7307	WP_028959642.1	-	hypothetical protein	1	0	1	1	1	1	1	1	1	1	1	10	Accessory								
7308	AXI91407.1	-	hypothetical protein	0	0	0	1	0	0	0	0	0	0	0	1	Unique								
7309	WP_019521559.1	-	hypothetical protein	1	1	1	1	1	1	1	1	1	1	1	11	CORE								

cluster	GI	gene	Annotation	Streptomyces_thermocarboxydus_BPSAC147	Streptomyces_sp._4F	Streptomyces_sp._BSE7F	Streptomyces_sp._ETH9427	Streptomyces_sp._FxanaD5	Streptomyces_sp._UNC401CLCol	Actinospica_acidiphila_NRRL_B_24431	Streptomyces_sp._HNS054	Streptomyces_sp._SMS_SU21	Streptomyces_sp._ZS0098	Streptomyces_thermocarboxydus_K155	Addition	Core-Accessory-Unique	COG	%Similarity_COG	Score_COG	Evalue_COG	KEGG	%Similarity_KEGG	Score_KEGG	Evalue_KEGG
7310	Prokka:CIODPCDJ_00533	dhaL-2	PEP-dependent dihydroxyacetone kinase 2, ADP-binding subunit DhaL	1	1	1	1	1	1	1	1	1	1	1	11	CORE	COG2376_G#	48.7	152	2.04E-36	K05879	44.6	152	4.18E-36
7311	WP_086020109.1	-	hypothetical protein	1	1	1	1	1	1	1	1	1	1	1	11	CORE								
7312	Prokka:CIODPCDJ_01278	-	hypothetical protein	1	0	1	1	1	1	1	1	1	1	1	10	Accessory	COG2864_C#	28.9	65	3.29E-10				
7313	Prokka:JAPCLKMA_02748	-	hypothetical protein	1	0	1	0	0	0	0	0	0	0	0	2	Accessory								
7314	Prokka:CIODPCDJ_06758	-	hypothetical protein	0	0	0	0	0	0	0	0	0	0	1	1	Unique								
7315	Prokka:JAPCLKMA_02014	phzD2	Phenazine biosynthesis protein PhzD2	1	0	0	0	0	0	0	0	0	0	0	1	Unique	COG1535_Q#	63.8	282	1.31E-75	K01252	48.8	210	2.21E-53
7316	WP_078895358.1	-	DDE endonuclease	0	0	0	0	0	0	0	1	0	0	0	1	Unique	COG3335_L#	31.8	79	2.2E-14	K07494	31.5	79	3.44E-14
7317	WP_122216598.1	-	(2Fe-2S)-binding protein	1	1	1	1	1	1	1	1	1	1	1	11	CORE	COG2080_C#	63.6	203	1.01E-51	K13483	53.8	190	2.36E-47
7318	Prokka:CIODPCDJ_05457	-	hypothetical protein	0	0	0	0	0	0	0	0	0	0	1	1	Unique	COG0454_KR#	40.6	79	1.29E-14				
7319	WP_051724396.1	-	deaminase	0	1	1	1	0	0	1	1	1	1	0	7	Accessory	COG0262_H#	39.1	116	1.62E-25	NA	25.6	67	1.77E-10

cluster	GI	gene	Annotation	Streptomyces_thermocarboxydus_BPSAC147	Streptomyces_sp._4F	Streptomyces_sp._BSE7F	Streptomyces_sp._ETH9427	Streptomyces_sp._FxanaD5	Streptomyces_sp._UNC401CLCol	Actinospica_acidiphila_NRRL_B_24431	Streptomyces_sp._HNS054	Streptomyces_sp._SMS_SU21	Streptomyces_sp._ZS0098	Streptomyces_thermocarboxydus_K155	Addition	Core-Accessory-Unique	COG	%Similarity_COG	Score_COG	Evalue_COG	KEGG	%Similarity_KEGG	Score_KEGG	Evalue_KEGG
7320	OSC61247.1	-	histidine phosphatase family protein	0	1	0	0	0	0	0	0	0	0	0	1	Unique	COG0406_G#	36	51	0.00000376				
7321	AXI91098.1	-	hypothetical protein	0	0	0	1	0	0	0	0	0	0	0	1	Unique								
7322	WP_109288797.1	-	hypothetical protein	0	0	0	0	0	0	0	0	1	0	0	1	Unique								
7323	WP_033273658.1	-	helix-turn-helix domain-containing protein	1	1	0	1	1	1	1	1	1	1	1	10	Accessory	COG1396_K#	31.2	74	4.15E-13	NA	25.5	66	3.02E-10
7324	AXI85316.1	-	hypothetical protein	0	0	0	1	0	0	0	0	0	0	0	1	Unique								
7325	OSC71870.1	-	hypothetical protein	0	1	0	0	0	0	0	0	0	0	0	1	Unique								
7326	PWE07241.1	-	ATP-binding protein	0	0	1	0	0	0	0	0	1	0	0	2	Accessory								
7327	Prokka:JAPCLKMA_04922	-	hypothetical protein	1	0	0	0	0	0	0	0	0	0	1	2	Accessory								
7328	Prokka:JAPCLKMA_05605	-	hypothetical protein	1	1	1	1	1	1	1	1	1	1	1	11	CORE								
7329	Prokka:CIODPCDJ_02862	-	hypothetical protein	1	1	0	1	1	1	1	1	0	0	1	8	Accessory								
7330	WP_122215023.1	-	ATP-binding protein	1	1	1	1	1	1	1	1	1	1	1	11	CORE	COG2229_R#	51.3	191	2.32E-48	K06945	51.3	191	6.19E-48
7331	WP_102641680.1	-	septum formation inhibitor Maf	1	1	1	1	1	1	1	1	1	1	1	11	CORE	COG0424_D#	47.9	173	1.11E-42	K06287	47.4	173	1.74E-42

cluster	GI	gene	Annotation	Streptomyces_thermocarboxydus_BPSAC147	Streptomyces_sp._4F	Streptomyces_sp._BSE7F	Streptomyces_sp._ETH9427	Streptomyces_sp._FxanaD5	Streptomyces_sp._UNC401CLCol	Actinospica_acidiphila_NRRL_B_24431	Streptomyces_sp._HNS054	Streptomyces_sp._SMS_SU21	Streptomyces_sp._ZS0098	Streptomyces_thermocarboxydus_K155	Addition	Core-Accessory-Unique	COG	%Similarity_COG	Score_COG	Evalue_COG	KEGG	%Similarity_KEGG	Score_KEGG	Evalue_KEGG
7332	WP_109288680.1	-	DUF2064 domain-containing protein	1	0	1	0	1	1	1	1	1	1	0	8	Accessory	COG3222_S#	41.7	119	1.46E-26	K09931	41.7	119	3.9E-26
7333	PWE08183.1	-	DUF47 domain-containing protein	1	1	1	1	1	1	1	1	1	1	1	11	CORE	COG1392_P#	30.5	117	7.25E-26	K07220	33.2	131	9.92E-30
7334	OSC71128.1	-	lysine transporter LysE	1	1	1	1	1	1	1	1	1	1	1	11	CORE	COG1280_E#	63.1	263	8.18E-70	NA	37.3	128	6.43E-29
7335	Prokka:CIODPCDJ_01308	slmA_1	Nucleoid occlusion factor SlmA	1	1	1	1	1	1	1	1	1	1	1	11	CORE	COG1309_K#	36.4	86	1.05E-16	NA	40.3	53	0.00000263
7336	PWE12016.1	-	amino acid transporter	1	1	1	1	1	1	1	1	1	1	1	11	CORE	COG1279_R#	53.5	195	1.6E-49	K06895	51	192	4.74E-48
7337	OSC62255.1	-	deaminase	0	1	0	0	0	0	0	0	0	0	0	1	Unique	COG0262_H#	42.5	163	6.76E-40	NA	25.9	51	0.00000999
7338	OSC57385.1	-	TetR family transcriptional regulator	0	1	0	0	0	0	0	0	0	0	0	1	Unique	COG1309_K#	30.3	66	1.47E-10	NA	25.8	48	0.0000846
7339	Prokka:JAPCLKMA_03877	-	putative HTH-type transcriptional regulator	1	1	1	1	1	1	1	1	1	1	1	11	CORE	COG1309_K#	34.7	109	1.51E-23	NA	34.7	109	4.04E-23
7340	WP_033273135.1	-	helix-turn-helix transcriptional regulator	0	0	1	0	0	0	1	0	1	0	0	3	Accessory	COG1309_K#	30.6	79	2.19E-14	NA	26.8	62	5.66E-09
7341	WP_107080953.1	-	CBS domain-containing protein	0	0	1	1	0	0	0	1	1	1	0	5	Accessory	COG0517_R#	31	87	8.04E-17	NA	31.5	72	4.19E-12

cluster	GI	gene	Annotation	Streptomyces_thermocarboxydus_BPSAC147	Streptomyces_sp._4F	Streptomyces_sp._BSE7F	Streptomyces_sp._ETH9427	Streptomyces_sp._FxanaD5	Streptomyces_sp._UNC401CLCol	Actinospica_acidiphila_NRRL_B_24431	Streptomyces_sp._HNS054	Streptomyces_sp._SMS_SU21	Streptomyces_sp._ZS0098	Streptomyces_thermocarboxydus_K155	Addition	Core-Accessory-Unique	COG	%Similarity_COG	Score_COG	Evalue_COG	KEGG	%Similarity_KEGG	Score_KEGG	Evalue_KEGG
7342	WP_158693583.1	-	hypothetical protein	0	0	0	0	0	1	0	0	0	0	0	1	Unique								
7343	OSC69587.1	-	TetR family transcriptional regulator	0	1	0	0	0	0	0	0	0	0	0	1	Unique	COG1309_K#	25	50	0.0000109	NA	38.3	44	0.0016
7344	Prokka:JAPCLKMA_02011	phzG	Phenazine biosynthesis protein PhzG	1	0	0	0	0	0	0	0	0	0	0	1	Unique	COG0259_H#	49	184	3.7E-46	K00275	38.5	127	1.87E-28
7345	WP_078895278.1	-	snapalysin family zinc-dependent metalloprotease	0	0	1	1	1	1	1	1	1	0	1	8	Accessory								
7346	Prokka:JAPCLKMA_04352	-	hypothetical protein	1	1	1	0	1	1	1	1	1	1	1	10	Accessory								
7347	WP_028958760.1	-	threonylcarbamoyl-AMP synthase	1	1	1	1	1	1	1	1	1	1	1	11	CORE	COG0009_J#	56.6	252	1.11E-66	NA	56.6	252	2.95E-66
7348	WP_019524910.1	-	hypothetical protein	0	0	0	1	1	0	0	1	0	0	0	3	Accessory								
7349	WP_051724672.1	-	hypothetical protein	0	0	0	0	0	0	1	0	0	0	0	1	Unique								
7350	WP_122216273.1	-	sigma-70 family RNA polymerase sigma factor	0	0	0	0	0	0	0	0	0	1	0	1	Unique	COG1595_K#	31.2	63	1.24E-09	K03088	28.1	55	0.00000053
7351	WP_086020146.1	-	flavin reductase	1	1	1	1	1	1	1	1	1	1	1	11	CORE	COG1853_R#	37.7	111	3.98E-24	NA	37.7	111	1.06E-23
7352	WP_106432481.1	-	HNH endonuclease	0	0	0	0	1	0	0	0	0	0	0	1	Unique	COG1403_V#	40.9	44	0.000597				

cluster	GI	gene	Annotation	Streptomyces_thermocarboxydus_BPSAC147	Streptomyces_sp._4F	Streptomyces_sp._BSE7F	Streptomyces_sp._ETH9427	Streptomyces_sp._FxanaD5	Streptomyces_sp._UNC401CLCol	Actinospica_acidiphila_NRRL_B_24431	Streptomyces_sp._HNS054	Streptomyces_sp._SMS_SU21	Streptomyces_sp._ZS0098	Streptomyces_thermocarboxydus_K155	Addition	Core-Accessory-Unique	COG	%Similarity_COG	Score_COG	Evalue_COG	KEGG	%Similarity_KEGG	Score_KEGG	Evalue_KEGG
7353	WP_033274858.1	-	TetR/AcrR family transcriptional regulator	0	0	0	0	0	0	1	0	0	0	0	1	Unique	COG1309_K#	37	75	3.16E-13	NA	37	75	8.44E-13
7354	OSC61462.1	-	GNAT family N-acetyltransferase	0	1	0	0	0	0	0	0	0	0	0	1	Unique	COG1042_C#	35	117	4.25E-26	K09181	31.6	84	1.07E-15
7355	OSC70271.1	-	cyclase	1	1	1	1	1	1	1	1	1	1	1	11	CORE								
7356	WP_026243808.1	-	sigma-70 family RNA polymerase sigma factor	1	0	0	0	1	1	0	0	0	0	1	4	Accessory								
7357	WP_102639421.1	-	hypothetical protein	0	0	1	0	0	0	0	0	1	0	0	2	Accessory								
7358	Prokka:CIODPCDJ_00523	-	hypothetical protein	0	0	0	0	1	1	0	0	0	0	1	3	Accessory	COG3435_Q#	32.1	42	0.00227				
7359	WP_033274841.1	-	ankyrin repeat domain-containing protein	1	0	0	0	0	0	1	1	1	1	1	6	Accessory	COG0666_R#	37.3	84	5.18E-16	NA	35.8	82	6.87E-15
7360	AXI87127.1	-	TetR family transcriptional regulator	0	0	1	1	0	0	0	0	1	0	0	3	Accessory	COG1309_K#	33.7	90	9.45E-18	NA	31.1	60	2.14E-08
7361	WP_102638880.1	-	hypothetical protein	0	1	1	1	0	0	1	1	1	1	0	7	Accessory								
7362	Prokka:JAPCLKMA_02016	-	hypothetical protein	1	0	0	0	0	0	0	0	0	0	0	1	Unique								
7363	WP_158688244.1	-	hypothetical protein	0	0	0	0	1	1	0	0	0	0	0	2	Accessory								

cluster	GI	gene	Annotation	Streptomyces_thermocarboxydus_BPSAC147	Streptomyces_sp._4F	Streptomyces_sp._BSE7F	Streptomyces_sp._ETH9427	Streptomyces_sp._FxanaD5	Streptomyces_sp._UNC401CLCol	Actinospica_acidiphila_NRRL_B_24431	Streptomyces_sp._HNS054	Streptomyces_sp._SMS_SU21	Streptomyces_sp._ZS0098	Streptomyces_thermocarboxydus_K155	Addition	Core-Accessory-Unique	COG	%Similarity_COG	Score_COG	Evalue_COG	KEGG	%Similarity_KEGG	Score_KEGG	Evalue_KEGG
7364	AXI84832.1	-	DNA-binding protein	1	1	0	1	1	1	0	1	1	1	1	9	Accessory	COG0262_H#	34.4	96	1.01E-19				
7365	WP_019525376.1	-	GNAT family N-acetyltransferase	1	1	1	1	1	1	1	1	1	1	1	11	CORE					NA	38.6	148	5.98E-35
7366	WP_033275990.1	-	TetR/AcrR family transcriptional regulator	1	0	0	0	1	1	1	0	1	1	1	7	Accessory	COG1309_K#	28.8	79	1.28E-14	NA	28.8	79	3.41E-14
7367	WP_051724429.1	-	carboxymuconolactone decarboxylase family protein	0	0	0	0	0	0	1	0	0	0	0	1	Unique								
7368	WP_052183282.1	-	Uma2 family endonuclease	1	0	1	1	1	1	1	1	1	0	1	9	Accessory								
7369	WP_019526910.1	-	L,D-transpeptidase	0	0	0	0	1	1	0	0	0	0	1	3	Accessory								
7370	WP_048458800.1	-	TetR family transcriptional regulator	0	1	0	1	0	0	0	1	0	0	0	3	Accessory	COG1309_K#	24.2	59	1.37E-08				
7371	WP_102641632.1	-	hypothetical protein	0	0	0	1	0	0	0	0	1	0	0	2	Accessory								
7372	WP_026243319.1	-	TetR/AcrR family transcriptional regulator	1	0	0	0	1	1	1	0	0	0	1	5	Accessory	COG1309_K#	47.1	51	0.00000486	NA	41.7	42	0.00603

cluster	GI	gene	Annotation	Streptomyces_thermocarboxydus_BPSAC147	Streptomyces_sp._4F	Streptomyces_sp._BSE7F	Streptomyces_sp._ETH9427	Streptomyces_sp._FxanaD5	Streptomyces_sp._UNC401CLCol	Actinospica_acidiphila_NRRL_B_24431	Streptomyces_sp._HNS054	Streptomyces_sp._SMS_SU21	Streptomyces_sp._ZS0098	Streptomyces_thermocarboxydus_K155	Addition	Core-Accessory-Unique	COG	%Similarity_COG	Score_COG	Evalue_COG	KEGG	%Similarity_KEGG	Score_KEGG	Evalue_KEGG
7373	WP_048460796.1	-	TetR/AcrR family transcriptional regulator	0	0	0	1	0	0	0	1	1	1	0	4	Accessory	COG1309_K#	25.9	64	7.25E-10	NA	43.8	45	0.000931
7374	WP_122215708.1	-	TetR/AcrR family transcriptional regulator	0	0	0	0	0	0	0	0	0	1	0	1	Unique	COG1309_K#	29.2	94	3.84E-19	K16137	26.6	74	1.43E-12
7375	WP_033275352.1	-	TetR/AcrR family transcriptional regulator	1	1	1	1	1	1	1	1	1	1	1	11	CORE	COG1309_K#	38.3	127	6.97E-29	NA	34.2	93	2.28E-18
7376	OSC72614.1	-	hypothetical protein	0	1	0	0	0	0	0	0	0	0	0	1	Unique								
7377	WP_122215697.1	-	hypothetical protein	0	0	0	0	0	0	0	0	0	1	0	1	Unique	COG5485_R#	31.3	74	7.01E-13				
7378	OSC57541.1	-	TetR family transcriptional regulator	1	1	1	1	1	1	1	1	1	1	1	11	CORE	COG1309_K#	35.9	98	3.47E-20	NA	35.9	98	9.27E-20
7379	OSC54419.1	-	hypothetical protein	0	1	1	1	0	0	1	1	1	1	0	7	Accessory					NA	26.5	68	7.86E-11
7380	OSC71487.1	-	TetR family transcriptional regulator	0	1	0	0	0	0	0	0	0	0	0	1	Unique	COG1309_K#	30.8	85	2.33E-16	NA	30.8	85	6.21E-16
7381	OSC71198.1	-	hypothetical protein	0	1	0	0	0	0	0	0	0	0	0	1	Unique								
7382	WP_167338232.1	-	hypothetical protein	1	1	1	1	1	1	1	1	1	1	1	11	CORE								
7383	WP_122214998.1	grpE	nucleotide exchange factor GrpE	0	0	0	0	0	0	0	0	1	1	0	2	Accessory	COG0576_O#	33	104	4.84E-22	K03687	33.8	75	6.43E-13

cluster	GI	gene	Annotation	Streptomyces_thermocarboxydus_BPSAC147	Streptomyces_sp._4F	Streptomyces_sp._BSE7F	Streptomyces_sp._ETH9427	Streptomyces_sp._FxanaD5	Streptomyces_sp._UNC401CLCol	Actinospica_acidiphila_NRRL_B_24431	Streptomyces_sp._HNS054	Streptomyces_sp._SMS_SU21	Streptomyces_sp._ZS0098	Streptomyces_thermocarboxydus_K155	Addition	Core-Accessory-Unique	COG	%Similarity_COG	Score_COG	Evalue_COG	KEGG	%Similarity_KEGG	Score_KEGG	Evalue_KEGG
7384	OSC62258.1	-	hypothetical protein	0	1	0	0	0	0	0	0	0	0	0	1	Unique								
7385	Prokka:JAPCLKMA_01982	-	hypothetical protein	1	0	0	0	0	0	0	0	0	0	0	1	Unique								
7386	AXI91271.1	-	hypothetical protein	0	0	0	1	0	0	0	0	0	0	0	1	Unique								
7387	Prokka:CIODPCDJ_06503	-	hypothetical protein	1	0	1	1	0	1	1	1	1	1	1	9	Accessory								
7388	WP_033273445.1	-	TetR family transcriptional regulator	1	0	0	0	1	1	1	0	0	0	1	5	Accessory	COG1309_K#	29.1	82	1.97E-15	NA	30.5	59	4.77E-08
7389	WP_102637523.1	-	TetR/AcrR family transcriptional regulator	0	0	0	1	0	0	0	1	1	0	0	3	Accessory	COG1309_K#	34.7	91	3.23E-18	NA	34.7	91	8.63E-18
7390	WP_122214518.1	-	hypothetical protein	1	1	0	1	1	1	1	1	1	1	1	10	Accessory								
7391	Prokka:JAPCLKMA_03622	-	hypothetical protein	1	1	1	1	1	1	1	1	1	1	1	11	CORE								
7392	WP_122215616.1	-	OmpA family protein	1	1	1	1	1	1	1	1	1	1	1	11	CORE	COG2885_M#	32	83	8.8E-16	K02557	26.4	63	3.29E-09
7393	Prokka:JAPCLKMA_00838	-	hypothetical protein	1	0	1	1	1	1	1	1	1	1	1	10	Accessory	COG3548_S#	33.9	85	3.02E-16				
7394	WP_019523761.1	rpsD	30S ribosomal protein S4	1	1	1	1	1	1	1	1	1	1	1	11	CORE	COG0522_J#	44	163	1.14E-39	K02986	44.6	158	7.51E-38
7395	WP_048459538.1	-	TmrB	1	0	1	1	1	1	1	1	1	1	1	10	Accessory					NA	43.4	164	1.37E-39

cluster	GI	gene	Annotation	Streptomyces_thermocarboxydus_BPSAC147	Streptomyces_sp._4F	Streptomyces_sp._BSE7F	Streptomyces_sp._ETH9427	Streptomyces_sp._FxanaD5	Streptomyces_sp._UNC401CLCol	Actinospica_acidiphila_NRRL_B_24431	Streptomyces_sp._HNS054	Streptomyces_sp._SMS_SU21	Streptomyces_sp._ZS0098	Streptomyces_thermocarboxydus_K155	Addition	Core-Accessory-Unique	COG	%Similarity_COG	Score_COG	Evalue_COG	KEGG	%Similarity_KEGG	Score_KEGG	Evalue_KEGG
7396	Prokka:CIODPCDJ_00379	ligD_1	Multifunctional non-homologous end joining protein LigD	1	0	0	0	1	1	1	0	0	0	1	5	Accessory	COG1793_L#	44.7	178	3.43E-44	K01971	41.2	119	2.96E-26
7397	Prokka:CIODPCDJ_06705	-	IS66 family transposase ISPre3	0	0	0	0	0	1	0	0	0	0	1	2	Accessory								
7398	WP_102639706.1	-	hypothetical protein	0	0	1	1	0	0	1	1	1	1	1	7	Accessory								
7399	WP_019523713.1	-	riboflavin synthase	1	1	1	1	1	1	1	1	1	1	1	11	CORE	COG0307_H#	70.4	260	5.25E-69	K00793	70.4	260	1.4E-68
7400	WP_048456697.1	-	TetR/AcrR family transcriptional regulator	1	0	1	1	1	1	1	1	1	1	1	10	Accessory	COG1309_K#	30.6	91	3.23E-18	NA	30.6	91	8.63E-18
7401	Prokka:CIODPCDJ_04254	cypD	Cypemycin cysteine dehydrogenase (decarboxylating)	0	0	0	0	0	0	0	0	0	0	1	1	Unique	COG0452_H#	35.5	106	1.66E-22	K13038	31.7	69	5.99E-11
7402	Prokka:CIODPCDJ_01427	-	hypothetical protein	1	0	1	1	1	1	1	1	1	1	1	10	Accessory	COG1595_K#	41.9	65	2.48E-10	K03088	32.6	47	0.000244
7403	Prokka:CIODPCDJ_06735	betI_16	HTH-type transcriptional regulator BetI	0	0	0	0	0	0	0	0	0	0	1	1	Unique	COG1309_K#	32.8	98	3.45E-20	NA	32.8	98	9.22E-20
7404	Prokka:JAPCLKMA_03795	tmk_2	Thymidylate kinase	1	0	0	0	0	0	0	0	0	0	1	2	Accessory								

cluster	GI	gene	Annotation	Streptomyces_thermocarboxydus_BPSAC147	Streptomyces_sp._4F	Streptomyces_sp._BSE7F	Streptomyces_sp._ETH9427	Streptomyces_sp._FxanaD5	Streptomyces_sp._UNC401CLCol	Actinospica_acidiphila_NRRL_B_24431	Streptomyces_sp._HNS054	Streptomyces_sp._SMS_SU21	Streptomyces_sp._ZS0098	Streptomyces_thermocarboxydus_K155	Addition	Core-Accessory-Unique	COG	%Similarity_COG	Score_COG	Evalue_COG	KEGG	%Similarity_KEGG	Score_KEGG	Evalue_KEGG
7405	WP_102639502.1	-	response regulator transcription factor	1	1	1	1	1	1	1	1	1	1	1	11	CORE	COG2197_TK#	56.2	204	4.47E-52	K07693	52	184	9.78E-46
7406	WP_019526319.1	-	hypothetical protein	1	1	1	1	1	1	1	1	1	1	1	11	CORE								
7407	PWE10379.1	-	TetR family transcriptional regulator	1	1	1	0	1	1	1	1	1	1	1	10	Accessory	COG1309_K#	30.4	69	1.32E-11	NA	30.4	69	3.51E-11
7408	WP_033276548.1	-	GNAT family N-acetyltransferase	1	1	0	1	1	1	1	0	0	1	1	8	Accessory	COG1670_J#	37	64	7.22E-10	K03817	37	64	1.93E-09
7409	AXI84688.1	-	DNA-binding response regulator	0	0	0	1	0	0	0	1	0	0	0	2	Accessory	COG2197_TK#	33.6	102	2.39E-21	NA	33.6	102	6.39E-21
7410	WP_167338243.1	paaJ	phenylacetate-CoA oxygenase subunit PaaJ	0	1	1	1	0	0	1	1	1	1	0	7	Accessory	COG2151_R#	39.8	126	9.06E-29	K02612	39.8	126	2.42E-28
7411	WP_019524805.1	-	TetR/AcrR family transcriptional regulator	1	0	0	0	1	1	0	0	0	0	1	4	Accessory	COG1309_K#	37.7	106	9.71E-23	NA	36.5	97	1.2E-19
7412	OSC68652.1	-	TetR family transcriptional regulator	0	1	0	0	0	0	0	0	0	0	0	1	Unique	COG1309_K#	35.1	92	1.45E-18	NA	38	80	1.99E-14
7413	WP_019527951.1	-	SMI1/KNR4 family protein	1	0	0	1	1	0	0	0	0	1	0	4	Accessory								

cluster	GI	gene	Annotation	Streptomyces_thermocarboxydus_BPSAC147	Streptomyces_sp._4F	Streptomyces_sp._BSE7F	Streptomyces_sp._ETH9427	Streptomyces_sp._FxanaD5	Streptomyces_sp._UNC401CLCol	Actinospica_acidiphila_NRRL_B_24431	Streptomyces_sp._HNS054	Streptomyces_sp._SMS_SU21	Streptomyces_sp._ZS0098	Streptomyces_thermocarboxydus_K155	Addition	Core-Accessory-Unique	COG	%Similarity_COG	Score_COG	Evalue_COG	KEGG	%Similarity_KEGG	Score_KEGG	Evalue_KEGG
7414	WP_102637153.1	-	helix-turn-helix transcriptional regulator	0	1	1	1	0	0	1	1	1	1	0	7	Accessory	COG1396_K#	29.2	63	1.23E-09				
7415	AXI89419.1	-	TetR family transcriptional regulator	1	1	1	1	1	1	1	1	1	1	1	11	CORE	COG1309_K#	45.6	170	5.47E-42				
7416	WP_157842166.1	-	DUF3592 domain-containing protein	1	0	0	0	1	1	1	0	0	0	1	5	Accessory								
7417	WP_146217826.1	-	HNH endonuclease	0	0	0	0	0	0	0	0	1	0	0	1	Unique								
7418	WP_033272751.1	-	LysE family transporter	0	0	0	0	0	0	1	0	0	0	0	1	Unique	COG1280_E#	55.9	211	2.8E-54	NA	30.5	105	5.78E-22
7419	WP_122216687.1	-	transposase	1	0	0	0	0	0	0	0	0	1	0	2	Accessory	COG3293_L#	47.6	71	3.46E-12	K07492	47.6	71	9.24E-12
7420	PWE08570.1	-	GTP pyrophosphokinase	0	0	1	0	0	0	0	0	0	0	0	1	Unique	COG0317_TK#	31.4	69	2.24E-11	K00951	32.3	62	4.29E-09
7421	OSC63315.1	-	ATP-binding protein	1	1	0	1	1	1	1	1	1	1	1	10	Accessory								
7422	Prokka:CIODPCDJ_05661	-	hypothetical protein	0	0	0	0	1	1	0	0	0	0	1	3	Accessory	COG2203_T#	31.2	72	2.03E-12				
7423	WP_033277101.1	-	FMN reductase	0	0	0	0	0	0	1	0	0	0	0	1	Unique	COG0431_R#	57.4	127	6.94E-29				
7424	WP_051724513.1	-	helix-turn-helix transcriptional regulator	1	1	1	1	1	1	1	1	1	1	1	11	CORE	COG1309_K#	32.8	92	2.47E-18				

cluster	GI	gene	Annotation	Streptomyces_thermocarboxydus_BPSAC147	Streptomyces_sp._4F	Streptomyces_sp._BSE7F	Streptomyces_sp._ETH9427	Streptomyces_sp._FxanaD5	Streptomyces_sp._UNC401CLCol	Actinospica_acidiphila_NRRL_B_24431	Streptomyces_sp._HNS054	Streptomyces_sp._SMS_SU21	Streptomyces_sp._ZS0098	Streptomyces_thermocarboxydus_K155	Addition	Core-Accessory-Unique	COG	%Similarity_COG	Score_COG	Evalue_COG	KEGG	%Similarity_KEGG	Score_KEGG	Evalue_KEGG
7425	AXI91327.1	-	thymidylate kinase	0	0	0	1	0	0	0	0	0	0	0	1	Unique								
7426	WP_102641257.1	-	dephospho-CoA kinase	1	1	1	1	1	1	1	1	1	1	1	11	CORE	COG0237_H#	51.8	193	7.85E-49	K00859	51.8	193	2.1E-48
7427	PWE08674.1	folK	2-amino-4-hydroxy-6-hydroxymethyldihydropteridine diphosphokinase	1	1	1	1	1	1	1	1	1	1	1	11	CORE	COG0801_H#	44.2	126	9.02E-29	K00950	43	126	3.15E-28
7428	WP_048455086.1	-	TetR/AcrR family transcriptional regulator	0	0	0	0	1	1	1	1	0	1	1	6	Accessory	COG1309_K#	29.3	47	0.0000695	NA	31.6	49	0.0000489
7429	WP_114874992.1	-	TetR/AcrR family transcriptional regulator	0	0	0	1	0	0	0	1	1	1	0	4	Accessory	COG1309_K#	31.4	50	0.00000629	NA	31.4	50	0.0000168
7430	PWE11239.1	-	hypothetical protein	0	1	1	1	0	0	0	1	1	1	0	6	Accessory								
7431	AXI85930.1	-	TetR/AcrR family transcriptional regulator	0	0	1	1	0	0	0	1	1	1	0	5	Accessory	COG1309_K#	37.1	125	2.01E-28	NA	30.2	59	3.62E-08
7432	AXI85396.1	-	hypothetical protein	1	1	0	1	1	1	1	1	1	1	1	10	Accessory								
7433	OSC73481.1	-	DNA-binding response regulator	1	1	1	1	1	1	1	1	1	1	1	11	CORE	COG2197_TK#	35.5	119	1.88E-26	K02479	35.5	119	5.02E-26

cluster	GI	gene	Annotation	Streptomyces_thermocarboxydus_BPSAC147	Streptomyces_sp._4F	Streptomyces_sp._BSE7F	Streptomyces_sp._ETH9427	Streptomyces_sp._FxanaD5	Streptomyces_sp._UNC401CLCol	Actinospica_acidiphila_NRRL_B_24431	Streptomyces_sp._HNS054	Streptomyces_sp._SMS_SU21	Streptomyces_sp._ZS0098	Streptomyces_thermocarboxydus_K155	Addition	Core-Accessory-Unique	COG	%Similarity_COG	Score_COG	Evalue_COG	KEGG	%Similarity_KEGG	Score_KEGG	Evalue_KEGG
7434	WP_019522879.1	-	TetR/AcrR family transcriptional regulator	1	1	1	1	1	1	1	1	1	1	1	11	CORE	COG1309_K#	34.8	74	6.94E-13	NA	34.8	74	1.85E-12
7435	WP_051101621.1	-	TetR/AcrR family transcriptional regulator	1	0	1	1	1	1	1	1	1	1	1	10	Accessory	COG1309_K#	41	110	6.69E-24	NA	34.3	90	2.5E-17
7436	WP_159020909.1	-	hypothetical protein	0	0	0	0	0	0	0	1	0	0	0	1	Unique								
7437	WP_048455649.1	-	SH3 domain-containing protein	0	0	1	1	0	0	1	1	1	1	0	6	Accessory	COG5651_N#	32.2	52	0.00000166	NA	40.9	69	4.56E-11
7438	OSC56374.1	-	TetR family transcriptional regulator	0	1	0	0	0	0	0	0	0	0	0	1	Unique	COG1309_K#	25.5	67	6.49E-11				
7439	OSC72947.1	-	hypothetical protein	0	1	1	1	1	1	1	1	1	1	0	9	Accessory								
7440	OSC66142.1	-	hypothetical protein	0	1	0	0	0	0	0	0	0	0	0	1	Unique	COG2850_S#	34.1	48	0.0000408	NA	34.1	48	0.000109
7441	OSC56159.1	-	collagen alpha-1(I) chain	0	1	0	0	0	0	0	0	0	0	0	1	Unique								
7442	WP_078895359.1	-	DUF4253 domain-containing protein	0	0	0	0	0	0	0	1	0	1	0	2	Accessory								
7443	WP_033274407.1	-	TetR/AcrR family transcriptional regulator	0	0	0	0	0	0	1	0	0	1	0	2	Accessory	COG1309_K#	28	47	0.0000695	NA	28	47	0.000186

cluster	GI	gene	Annotation	Streptomyces_thermocarboxydus_BPSAC147	Streptomyces_sp._4F	Streptomyces_sp._BSE7F	Streptomyces_sp._ETH9427	Streptomyces_sp._FxanaD5	Streptomyces_sp._UNC401CLCol	Actinospica_acidiphila_NRRL_B_24431	Streptomyces_sp._HNS054	Streptomyces_sp._SMS_SU21	Streptomyces_sp._ZS0098	Streptomyces_thermocarboxydus_K155	Addition	Core-Accessory-Unique	COG	%Similarity_COG	Score_COG	Evalue_COG	KEGG	%Similarity_KEGG	Score_KEGG	Evalue_KEGG
7444	OSC71030.1	-	TetR family transcriptional regulator	1	1	1	1	1	1	1	1	1	1	1	11	CORE	COG1309_K#	30.3	81	4.35E-15	NA	30.3	81	1.16E-14
7445	WP_086020198.1	-	hypothetical protein	1	1	1	1	1	1	1	1	1	1	1	11	CORE								
7446	Prokka:JAPCLKMA_00422	-	Putative 3-methyladenine DNA glycosylase	1	0	1	0	0	0	0	0	1	1	0	4	Accessory	COG2094_L#	50.8	176	1.3E-43	K03652	49	173	2.93E-42
7447	OSC69864.1	-	glutamine amidotransferase	0	1	1	1	0	0	1	1	1	1	0	7	Accessory	COG0693_R#	35	127	4.05E-29	NA	36.1	120	1.73E-26
7448	PWE11506.1	-	glyoxalase	0	0	1	0	0	0	0	0	1	0	0	2	Accessory	COG0346_E#	38.3	101	5.3E-21				
7449	WP_033273798.1	-	class I SAM-dependent methyltransferase	0	1	0	0	0	0	1	0	0	0	0	2	Accessory	COG0500_QR#	33.8	113	7.9E-25	NA	25.3	54	0.00000198
7450	Prokka:JAPCLKMA_04782	-	hypothetical protein	1	0	0	0	0	0	0	0	0	0	0	1	Unique								
7451	WP_084207095.1	-	SDR family oxidoreductase	0	0	0	0	0	0	1	0	0	0	0	1	Unique	COG4221_R#	38.1	152	1.54E-36	NA	37.3	122	4.54E-27
7452	OSC69001.1	-	deaminase	0	1	0	0	0	0	0	0	0	0	0	1	Unique	COG0262_H#	39.4	143	9.31E-34	NA	27.3	74	1.42E-12
7453	Prokka:JAPCLKMA_03379	-	hypothetical protein	1	0	0	0	0	0	0	0	0	0	0	1	Unique								
7454	WP_019527769.1	-	GGDEF domain-containing protein	0	0	0	0	1	0	0	0	0	0	0	1	Unique	COG2199_T#	42	108	2.54E-23	NA	42	108	6.79E-23

cluster	GI	gene	Annotation	Streptomyces_thermocarboxydus_BPSAC147	Streptomyces_sp._4F	Streptomyces_sp._BSE7F	Streptomyces_sp._ETH9427	Streptomyces_sp._FxanaD5	Streptomyces_sp._UNC401CLCol	Actinospica_acidiphila_NRRL_B_24431	Streptomyces_sp._HNS054	Streptomyces_sp._SMS_SU21	Streptomyces_sp._ZS0098	Streptomyces_thermocarboxydus_K155	Addition	Core-Accessory-Unique	COG	%Similarity_COG	Score_COG	Evalue_COG	KEGG	%Similarity_KEGG	Score_KEGG	Evalue_KEGG
7455	WP_102638296.1	-	flavodoxin family protein	1	1	1	1	1	1	1	1	1	1	1	11	CORE	COG0655_R#	44.7	163	1.13E-39	NA	42	161	1.15E-38
7456	WP_028961254.1	-	CDP-alcohol phosphatidyltransferase family protein	1	1	1	1	1	1	1	1	1	1	1	11	CORE	COG0558_I#	44.6	159	1.63E-38	K00995	40.9	147	1.31E-34
7457	WP_086020139.1	pdxT	pyridoxal 5'-phosphate synthase glutaminase subunit PdxT	1	1	1	1	1	1	1	1	1	1	1	11	CORE	COG0311_H#	57.1	214	4.27E-55	K08681	55.8	210	1.65E-53
7458	Prokka:CIODPCDJ_04067	-	hypothetical protein	0	0	0	0	0	0	0	0	0	0	1	1	Unique								
7459	Prokka:JAPCLKMA_05762	-	hypothetical protein	1	1	1	1	1	1	1	1	1	1	1	11	CORE	COG4420_S#	54	154	4.02E-37	NA	54	154	1.07E-36
7460	AXI89291.1	-	dihydrofolate reductase	1	0	1	1	1	1	1	1	1	1	1	10	Accessory	COG0262_H#	33.2	116	1.58E-25	NA	29.7	85	6.12E-16
7461	AXI89804.1	-	hypothetical protein	1	0	1	1	1	1	0	1	1	1	0	8	Accessory								
7462	WP_019522143.1	cobO	cob(I)yrinic acid a,c-diamide adenosyltransferase	1	1	1	1	1	1	1	1	1	1	1	11	CORE	COG2109_H#	61.8	270	3.85E-72	K19221	61.8	270	1.03E-71
7463	WP_048458127.1	-	hypothetical protein	1	1	1	1	1	1	1	1	1	1	1	11	CORE								
7464	PWE06915.1	-	DUF1349 domain-containing protein	1	1	1	1	1	1	1	1	1	1	1	11	CORE	COG3506_S#	28.2	41	0.00496				

cluster	GI	gene	Annotation	Streptomyces_thermocarboxydus_BPSAC147	Streptomyces_sp._4F	Streptomyces_sp._BSE7F	Streptomyces_sp._ETH9427	Streptomyces_sp._FxanaD5	Streptomyces_sp._UNC401CLCol	Actinospica_acidiphila_NRRL_B_24431	Streptomyces_sp._HNS054	Streptomyces_sp._SMS_SU21	Streptomyces_sp._ZS0098	Streptomyces_thermocarboxydus_K155	Addition	Core-Accessory-Unique	COG	%Similarity_COG	Score_COG	Evalue_COG	KEGG	%Similarity_KEGG	Score_KEGG	Evalue_KEGG
7465	WP_019526847.1	-	cupin domain-containing protein	0	0	0	0	1	1	0	0	0	0	1	3	Accessory								
7466	WP_019523861.1	-	SseB family protein	0	0	0	1	1	1	1	1	1	1	1	8	Accessory								
7467	WP_102638529.1	-	transposase	0	0	0	0	0	0	1	0	1	0	0	2	Accessory	COG2801_L#	53.6	95	2.89E-19	NA	53.6	95	7.73E-19
7468	WP_146217840.1	-	aminotransferase class I/II-fold pyridoxal phosphate-dependent enzyme	0	0	0	0	0	0	0	0	1	0	0	1	Unique	COG0156_H#	49.7	183	1.06E-45	NA	58.9	247	1.22E-64
7469	OSC69417.1	-	hypothetical protein	0	1	0	0	0	0	0	0	0	0	0	1	Unique								
7470	WP_019527495.1	-	membrane protein	0	0	0	0	1	1	0	0	0	0	0	2	Accessory								
7471	Prokka:CIODPCDJ_00727	COQ5_3	2-methoxy-6-polyprenyl-1,4-benzoquinol methylase, mitochondrial	1	1	1	0	1	1	1	1	1	1	1	10	Accessory	COG2226_H#	35.1	69	2.22E-11	NA	32.7	65	5.02E-10
7472	OSC65098.1	-	6-phospho 3-hexuloisomerase	0	1	0	0	0	0	0	0	0	0	0	1	Unique	COG0794_M#	39.1	119	1.87E-26	K08094	38.8	107	1.97E-22
7473	AXI88560.1	-	hypothetical protein	0	0	0	1	0	0	0	1	0	0	0	2	Accessory								
7474	WP_028961381.1	-	hypothetical protein	0	0	0	0	0	1	0	0	0	0	0	1	Unique								

cluster	GI	gene	Annotation	Streptomyces_thermocarboxydus_BPSAC147	Streptomyces_sp._4F	Streptomyces_sp._BSE7F	Streptomyces_sp._ETH9427	Streptomyces_sp._FxanaD5	Streptomyces_sp._UNC401CLCol	Actinospica_acidiphila_NRRL_B_24431	Streptomyces_sp._HNS054	Streptomyces_sp._SMS_SU21	Streptomyces_sp._ZS0098	Streptomyces_thermocarboxydus_K155	Addition	Core-Accessory-Unique	COG	%Similarity_COG	Score_COG	Evalue_COG	KEGG	%Similarity_KEGG	Score_KEGG	Evalue_KEGG
7475	OSC68486.1	-	hypothetical protein	0	1	0	0	0	0	0	0	0	0	0	1	Unique								
7476	WP_033272584.1	-	rhomboid family intramembrane serine protease	1	0	1	1	1	1	1	1	1	1	1	10	Accessory	COG0705_R#	43.6	146	1.43E-34	NA	43.6	146	3.82E-34
7477	Prokka:CIODPCDJ_06557	sutR_4	HTH-type transcriptional regulator SutR	1	0	0	0	1	1	0	0	0	0	1	4	Accessory	COG1396_K#	38.4	117	5.45E-26	NA	30.2	84	1.04E-15
7478	OSC61536.1	-	TetR family transcriptional regulator	0	1	1	1	0	0	1	1	0	1	0	6	Accessory	COG1309_K#	41.1	148	3.76E-35	NA	36.2	112	6.11E-24
7479	OSC70751.1	-	TetR family transcriptional regulator	0	1	0	0	0	0	0	0	0	0	0	1	Unique	COG1309_K#	42.2	100	9E-21	NA	42.2	100	2.4E-20
7480	PWE09597.1	-	DNA mismatch repair protein MutT	1	1	1	1	1	1	1	1	1	1	1	11	CORE	COG0494_LR#	44.2	101	4.04E-21	NA	43.3	104	1.27E-21
7481	WP_122215720.1	wrbA	NAD(P)H:quinone oxidoreductase	0	0	0	0	0	0	0	0	0	1	0	1	Unique	COG0655_R#	53	207	4E-53	K03809	50	193	1.6E-48
7482	Prokka:CIODPCDJ_04253	-	hypothetical protein	0	0	0	0	0	0	0	0	0	0	1	1	Unique								
7483	AXI91249.1	-	hypothetical protein	0	0	0	1	0	0	0	0	0	0	0	1	Unique								

cluster	GI	gene	Annotation	Streptomyces_thermocarboxydus_BPSAC147	Streptomyces_sp._4F	Streptomyces_sp._BSE7F	Streptomyces_sp._ETH9427	Streptomyces_sp._FxanaD5	Streptomyces_sp._UNC401CLCol	Actinospica_acidiphila_NRRL_B_24431	Streptomyces_sp._HNS054	Streptomyces_sp._SMS_SU21	Streptomyces_sp._ZS0098	Streptomyces_thermocarboxydus_K155	Addition	Core-Accessory-Unique	COG	%Similarity_COG	Score_COG	Evalue_COG	KEGG	%Similarity_KEGG	Score_KEGG	Evalue_KEGG
7484	WP_033278070.1	-	malonic semialdehyde reductase	1	1	1	1	1	1	1	1	1	1	1	11	CORE	COG0778_C#	59.6	247	3.49E-65	K09019	39.7	138	1.04E-31
7485	WP_028959782.1	-	ATP-binding protein	0	1	0	0	1	1	0	0	1	1	1	6	Accessory								
7486	PWE09795.1	-	GNAT family N-acetyltransferase	0	0	1	0	0	0	0	0	0	0	0	1	Unique	COG1670_J#	32.3	90	9.31E-18	NA	32.3	90	2.49E-17
7487	AXI86737.1	-	TetR/AcrR family transcriptional regulator	0	1	1	1	0	0	1	1	1	0	0	6	Accessory	COG1309_K#	34.1	68	3.77E-11	NA	38.9	53	0.00000335
7488	WP_102638239.1	-	TetR family transcriptional regulator	1	1	1	0	1	1	1	1	1	1	1	10	Accessory	COG1309_K#	32.6	57	6.65E-08	K03577	27.9	54	0.00000115
7489	WP_019521790.1	-	adenylosuccinate lyase	1	0	1	1	1	1	1	1	1	1	1	10	Accessory								
7490	PWE12284.1	-	DNA invertase	0	0	1	0	0	0	0	0	0	0	0	1	Unique	COG1961_L#	44	145	1.86E-34	NA	48	139	3.56E-32
7491	WP_122216152.1	-	deaminase	1	1	1	1	1	1	1	1	1	1	1	11	CORE	COG0262_H#	31.3	79	1.64E-14				
7492	OSC68117.1	-	Holliday junction branch migration protein RuvA	1	1	1	1	1	1	1	1	1	1	1	11	CORE	COG0632_L#	46.6	166	1.02E-40	K03550	46.6	166	2.72E-40
7493	OSC65791.1	-	hypothetical protein	0	1	0	0	0	0	0	0	0	0	0	1	Unique								

cluster	GI	gene	Annotation	Streptomyces_thermocarboxydus_BPSAC147	Streptomyces_sp._4F	Streptomyces_sp._BSE7F	Streptomyces_sp._ETH9427	Streptomyces_sp._FxanaD5	Streptomyces_sp._UNC401CLCol	Actinospica_acidiphila_NRRL_B_24431	Streptomyces_sp._HNS054	Streptomyces_sp._SMS_SU21	Streptomyces_sp._ZS0098	Streptomyces_thermocarboxydus_K155	Addition	Core-Accessory-Unique	COG	%Similarity_COG	Score_COG	Evalue_COG	KEGG	%Similarity_KEGG	Score_KEGG	Evalue_KEGG
7494	WP_122216694.1	-	TetR/AcrR family transcriptional regulator	0	0	1	1	0	0	0	1	0	1	0	4	Accessory	COG1309_K#	24.7	76	1.06E-13	NA	27	42	0.00453
7495	Prokka:JAPCLKMA_02561	-	hypothetical protein	1	1	1	1	1	1	1	1	1	1	1	11	CORE								
7496	AXI91060.1	-	class I SAM-dependent methyltransferase	0	0	1	1	0	0	0	1	1	1	0	5	Accessory	COG0500_QR#	36.7	113	1.02E-24	NA	34.4	104	1.66E-21
7497	Prokka:CIODPCDJ_02416	gbpA_2	GlcNAc-binding protein A	1	1	1	1	1	1	1	1	1	1	1	11	CORE	COG3397_S#	43.4	153	8.91E-37	K03933	36.8	134	1.49E-30
7498	WP_048459526.1	folE	GTP cyclohydrolase I FolE	1	1	1	1	1	1	1	1	1	1	1	11	CORE	COG0302_H#	67.2	264	2.74E-70	K01495	67.2	264	7.33E-70
7499	WP_009190144.1	rpsE	30S ribosomal protein S5	1	1	1	1	1	1	1	1	1	1	1	11	CORE	COG0098_J#	71.5	287	5.16E-77	K02988	71.5	287	1.38E-76
7500	WP_102639142.1	-	hypothetical protein	1	0	1	1	1	1	1	1	1	1	1	10	Accessory								
7501	PWE12357.1	-	NAD(P)H dehydrogenase	0	1	1	1	0	0	1	1	1	0	0	6	Accessory	COG2249_R#	48.8	186	9.5E-47	K00355	30	82	5.16E-15
7502	OSC61657.1	-	deaminase	0	1	0	0	0	0	0	0	0	0	0	1	Unique	COG0262_H#	43.8	176	1.28E-43	NA	33	97	1.55E-19
7503	WP_033277588.1	pcaG	protocatechuate 3,4-dioxygenase subunit alpha	1	1	0	0	1	1	1	0	0	0	1	6	Accessory	COG3485_Q#	37.9	114	4.59E-25				

cluster	GI	gene	Annotation	Streptomyces_thermocarboxydus_BPSAC147	Streptomyces_sp._4F	Streptomyces_sp._BSE7F	Streptomyces_sp._ETH9427	Streptomyces_sp._FxanaD5	Streptomyces_sp._UNC401CLCol	Actinospica_acidiphila_NRRL_B_24431	Streptomyces_sp._HNS054	Streptomyces_sp._SMS_SU21	Streptomyces_sp._ZS0098	Streptomyces_thermocarboxydus_K155	Addition	Core-Accessory-Unique	COG	%Similarity_COG	Score_COG	Evalue_COG	KEGG	%Similarity_KEGG	Score_KEGG	Evalue_KEGG
7504	AXI85534.1	-	ATP-dependent Clp protease proteolytic subunit	1	0	1	1	0	1	1	1	1	1	1	9	Accessory	COG0740_OU#	48.4	189	1.12E-47	K01358	47.1	186	2.54E-46
7505	WP_102637027.1	-	sulfite oxidase-like oxidoreductase	1	1	1	1	1	1	1	1	1	1	1	11	CORE	COG2041_R#	49	194	4.56E-49	K07147	47.8	169	3.21E-41
7506	WP_019522251.1	-	MarC family protein	1	1	1	1	1	1	1	1	1	1	1	11	CORE	COG2095_U#	35.6	140	5.98E-33	K05595	35.6	140	1.6E-32
7507	OSC63118.1	-	TetR family transcriptional regulator	0	1	0	0	0	0	0	0	0	0	0	1	Unique	COG1309_K#	27.3	62	2.07E-09	NA	33.3	46	0.00041
7508	PWE11067.1	-	hypothetical protein	0	0	1	0	0	0	0	0	0	0	0	1	Unique								
7509	OSC55175.1	-	TetR family transcriptional regulator	0	1	0	0	0	0	0	0	0	0	0	1	Unique	COG1309_K#	39.9	135	2.51E-31	NA	35.2	92	3.82E-18
7510	OSC61460.1	-	hypothetical protein	0	1	0	0	0	0	0	0	0	0	0	1	Unique	COG1926_R#	42.3	129	1.38E-29	K07100	42.3	129	3.68E-29
7511	OSC65421.1	-	oligoribonuclease	1	1	1	1	1	1	1	1	1	1	1	11	CORE	COG1949_A#	60.5	247	3.47E-65	K13288	60.5	247	9.27E-65
7512	WP_033275979.1	-	TetR family transcriptional regulator	1	1	0	1	1	1	1	1	0	1	1	9	Accessory	COG1309_K#	26.3	63	1.21E-09	NA	33.3	49	0.0000371
7513	AXI85346.1	-	hypothetical protein	1	0	0	1	0	0	0	0	0	0	0	2	Accessory								

cluster	GI	gene	Annotation	Streptomyces_thermocarboxydus_BPSAC147	Streptomyces_sp._4F	Streptomyces_sp._BSE7F	Streptomyces_sp._ETH9427	Streptomyces_sp._FxanaD5	Streptomyces_sp._UNC401CLCol	Actinospica_acidiphila_NRRL_B_24431	Streptomyces_sp._HNS054	Streptomyces_sp._SMS_SU21	Streptomyces_sp._ZS0098	Streptomyces_thermocarboxydus_K155	Addition	Core-Accessory-Unique	COG	%Similarity_COG	Score_COG	Evalue_COG	KEGG	%Similarity_KEGG	Score_KEGG	Evalue_KEGG
7514	Prokka:JAPCLKMA_01612	-	hypothetical protein	1	0	0	0	0	0	0	0	0	0	0	1	Unique								
7515	WP_033278063.1	-	hypothetical protein	0	0	0	0	0	0	1	0	0	0	0	1	Unique								
7516	WP_159020893.1	-	DNA-binding response regulator	0	1	0	1	0	0	0	1	0	0	0	3	Accessory	COG2197_TK#	40.7	126	1.17E-28	K07693	31.7	98	9.08E-20
7517	OSC72875.1	-	hypothetical protein	0	1	0	0	0	0	0	0	0	0	0	1	Unique								
7518	OSC63765.1	-	TIGR03086 family protein	1	1	1	1	1	1	1	1	1	1	1	11	CORE					NA	24.6	54	0.00000115
7519	OSC71759.1	-	NADPH-dependent FMN reductase	0	1	0	0	0	0	0	0	0	0	0	1	Unique	COG0431_R#	46.2	159	1.24E-38	NA	38.1	91	8.5E-18
7520	OSC70988.1	-	hypothetical protein	0	1	0	0	0	0	0	0	0	0	0	1	Unique								
7521	AXI88228.1	-	SMI1/KNR4 family protein	0	0	0	1	0	0	0	1	0	0	0	2	Accessory								
7522	OSC69695.1	-	hypothetical protein	0	1	0	0	0	0	0	0	0	0	0	1	Unique								
7523	OSC55379.1	-	hypothetical protein	0	1	0	0	0	0	0	0	0	0	0	1	Unique								
7524	WP_102642156.1	-	histidine phosphatase family protein	1	1	1	1	1	1	1	1	1	1	1	11	CORE	COG0406_G#	49.7	178	2.57E-44	K15634	42.6	144	1.44E-33
7525	PWE06768.1	-	NADP oxidoreductase	0	0	1	1	0	0	0	1	1	1	0	5	Accessory	COG2085_R#	43.8	154	5.2E-37				
7526	WP_019523002.1	-	hypothetical protein	1	0	0	1	1	1	1	1	1	1	1	9	Accessory								
7527	WP_122217047.1	-	hypothetical protein	1	1	1	1	1	1	1	1	1	1	1	11	CORE								

cluster	GI	gene	Annotation	Streptomyces_thermocarboxydus_BPSAC147	Streptomyces_sp._4F	Streptomyces_sp._BSE7F	Streptomyces_sp._ETH9427	Streptomyces_sp._FxanaD5	Streptomyces_sp._UNC401CLCol	Actinospica_acidiphila_NRRL_B_24431	Streptomyces_sp._HNS054	Streptomyces_sp._SMS_SU21	Streptomyces_sp._ZS0098	Streptomyces_thermocarboxydus_K155	Addition	Core-Accessory-Unique	COG	%Similarity_COG	Score_COG	Evalue_COG	KEGG	%Similarity_KEGG	Score_KEGG	Evalue_KEGG
7528	AXI89967.1	-	DNA-binding response regulator	0	0	1	1	0	0	0	0	1	1	0	4	Accessory	COG2197_TK#	40.9	153	6.79E-37	K07693	42.6	145	6.45E-34
7529	AXI89965.1	-	GNAT family N-acetyltransferase	1	0	1	1	1	1	1	1	1	1	1	10	Accessory								
7530	AXI85366.1	-	hypothetical protein	0	0	0	1	0	0	0	0	0	0	0	1	Unique								
7531	Prokka:JAPCLKMA_01502	-	hypothetical protein	1	0	0	1	0	0	0	0	0	0	0	2	Accessory								
7532	WP_159020815.1	-	hypothetical protein	0	0	0	0	0	0	0	1	0	0	0	1	Unique								
7533	WP_122214616.1	-	TetR/AcrR family transcriptional regulator	1	1	1	1	1	1	1	1	1	1	1	11	CORE	COG1309_K#	36.8	106	1.62E-22	NA	36.8	106	4.34E-22
7534	Prokka:CIODPCDJ_00266	-	hypothetical protein	1	1	1	1	1	1	1	1	1	1	1	11	CORE	COG2229_R#	52	185	2.11E-46	K06945	52	185	5.62E-46
7535	WP_048456500.1	-	hypothetical protein	1	0	1	1	1	1	1	1	1	1	1	10	Accessory								
7536	Prokka:JAPCLKMA_04497	-	hypothetical protein	1	1	1	1	1	1	1	1	1	1	1	11	CORE	COG2353_S#	48.6	181	2.33E-45	NA	32.2	114	1.22E-24
7537	WP_086699834.1	-	hypothetical protein	0	0	1	1	0	0	1	1	1	1	0	6	Accessory								
7538	AXI87503.1	-	Fe-S cluster assembly protein HesB	1	1	1	1	1	1	1	1	1	1	1	11	CORE					NA	58.5	221	5.42E-57
7539	Prokka:CIODPCDJ_02475	-	dITP/XTP pyrophosphatase	1	1	1	1	1	1	1	1	1	1	1	11	CORE	COG0127_F#	51.8	193	1.01E-48	K02428	51.8	193	2.7E-48

cluster	GI	gene	Annotation	Streptomyces_thermocarboxydus_BPSAC147	Streptomyces_sp._4F	Streptomyces_sp._BSE7F	Streptomyces_sp._ETH9427	Streptomyces_sp._FxanaD5	Streptomyces_sp._UNC401CLCol	Actinospica_acidiphila_NRRL_B_24431	Streptomyces_sp._HNS054	Streptomyces_sp._SMS_SU21	Streptomyces_sp._ZS0098	Streptomyces_thermocarboxydus_K155	Addition	Core-Accessory-Unique	COG	%Similarity_COG	Score_COG	Evalue_COG	KEGG	%Similarity_KEGG	Score_KEGG	Evalue_KEGG
7540	Prokka:CIODPCDJ_05996	rutE_2	malonic semialdehyde reductase RutE	1	0	0	0	1	1	1	0	0	0	1	5	Accessory	COG0778_C#	42	139	1.73E-32	NA	28.8	65	4.97E-10
7541	AXI85920.1	-	hypothetical protein	1	1	1	1	1	1	1	1	1	1	1	11	CORE								
7542	OSC73553.1	-	hypothetical protein	0	1	0	0	0	0	0	0	0	0	0	1	Unique								
7543	WP_033274397.1	-	hypothetical protein	0	0	0	0	0	0	1	0	0	0	0	1	Unique								
7544	OSC69941.1	-	DNA polymerase III subunit epsilon	0	1	0	0	0	0	0	0	0	0	0	1	Unique	COG0847_L#	34.1	73	1.17E-12	K02342	29.4	58	1.04E-07
7545	AXI90499.1	-	hypothetical protein	0	0	0	1	0	0	0	1	0	0	0	2	Accessory								
7546	Prokka:CIODPCDJ_00003	-	hypothetical protein	0	0	1	0	1	1	1	0	1	1	1	7	Accessory	COG1309_K#	43.6	146	1.42E-34	NA	37.1	106	2.54E-22
7547	WP_048460477.1	-	nucleotidyltransferase family protein	1	0	1	1	1	0	1	1	0	1	1	8	Accessory	COG3575_S#	45.8	183	1.04E-45	K09962	43.3	141	7.13E-33
7548	OSC60098.1	-	ArsR family transcriptional regulator	0	1	0	0	0	0	0	0	0	0	0	1	Unique	COG0607_P#	53.6	118	3.16E-26	NA	56	208	8.1E-53
7549	OSC73332.1	-	hypothetical protein	0	1	0	0	0	0	0	0	0	0	0	1	Unique					NA	31.1	50	0.0000166
7550	Prokka:CIODPCDJ_06716	-	hypothetical protein	0	0	0	0	0	0	0	0	0	0	1	1	Unique	COG1203_R#	24.9	63	9.24E-10				
7551	OSC67906.1	-	DDE endonuclease	0	1	0	0	0	0	0	0	0	0	0	1	Unique	COG3335_L#	34.7	99	1.98E-20	K07494	34.7	99	5.3E-20

cluster	GI	gene	Annotation	Streptomyces_thermocarboxydus_BPSAC147	Streptomyces_sp._4F	Streptomyces_sp._BSE7F	Streptomyces_sp._ETH9427	Streptomyces_sp._FxanaD5	Streptomyces_sp._UNC401CLCol	Actinospica_acidiphila_NRRL_B_24431	Streptomyces_sp._HNS054	Streptomyces_sp._SMS_SU21	Streptomyces_sp._ZS0098	Streptomyces_thermocarboxydus_K155	Addition	Core-Accessory-Unique	COG	%Similarity_COG	Score_COG	Evalue_COG	KEGG	%Similarity_KEGG	Score_KEGG	Evalue_KEGG
7552	OSC60334.1	-	precorrin-8X methylmutase	1	1	1	1	1	1	1	1	1	1	1	11	CORE	COG2082_H#	41.7	122	1.68E-27	K06042	37.9	120	2.22E-26
7553	OSC57147.1	-	hypothetical protein	0	1	0	0	0	0	0	0	0	0	0	1	Unique								
7554	Prokka:JAPCLKMA_02747	-	hypothetical protein	1	0	0	0	0	0	0	0	0	0	0	1	Unique								
7555	Prokka:JAPCLKMA_03327	-	hypothetical protein	1	0	0	1	0	0	0	1	0	1	1	5	Accessory								
7556	WP_122215721.1	-	TetR family transcriptional regulator	0	0	0	0	0	0	0	0	0	1	0	1	Unique	COG1309_K#	34.5	127	6.8E-29	K18939	29.1	75	4.8E-13
7557	OSC61940.1	-	hypothetical protein	0	1	0	0	0	0	0	0	0	0	0	1	Unique								
7558	AXI87190.1	-	recombination protein RecR	1	1	1	1	1	1	1	1	1	1	1	11	CORE	COG0353_L#	68.3	288	1.76E-77	K06187	68.3	288	4.69E-77
7559	WP_048460756.1	-	hypothetical protein	1	0	1	0	1	1	1	1	1	1	1	9	Accessory	COG3170_NU#	35.5	54	4.27E-07	NA	30.2	57	1.35E-07
7560	AXI91338.1	-	hypothetical protein	0	0	0	1	0	0	0	0	0	0	0	1	Unique								
7561	OSC62045.1	-	hypothetical protein	1	1	1	1	1	1	1	1	1	1	1	11	CORE								
7562	PWE10095.1	-	4-diphosphocytidyl-2C-methyl-D-erythritol synthase	1	1	1	1	1	1	1	1	1	1	1	11	CORE	COG2068_R#	37.5	95	2.18E-19				
7563	OSC69665.1	-	hypothetical protein	0	1	0	0	0	0	0	0	0	0	0	1	Unique	COG4916_S#	26	42	0.00219				

cluster	GI	gene	Annotation	Streptomyces_thermocarboxydus_BPSAC147	Streptomyces_sp._4F	Streptomyces_sp._BSE7F	Streptomyces_sp._ETH9427	Streptomyces_sp._FxanaD5	Streptomyces_sp._UNC401CLCol	Actinospica_acidiphila_NRRL_B_24431	Streptomyces_sp._HNS054	Streptomyces_sp._SMS_SU21	Streptomyces_sp._ZS0098	Streptomyces_thermocarboxydus_K155	Addition	Core-Accessory-Unique	COG	%Similarity_COG	Score_COG	Evalue_COG	KEGG	%Similarity_KEGG	Score_KEGG	Evalue_KEGG
7564	OSC70060.1	-	LysR family transcriptional regulator	0	1	0	0	0	0	0	0	0	0	0	1	Unique	COG0583_K#	31.3	96	9.8E-20	NA	31.8	86	2.71E-16
7565	WP_033272077.1	-	hypothetical protein	0	0	0	0	0	0	1	0	0	0	0	1	Unique								
7566	WP_028959923.1	-	PadR family transcriptional regulator	1	0	0	0	1	1	0	0	0	0	1	4	Accessory								
7567	OSC57569.1	-	SWF or SNF family helicase	0	1	0	0	0	0	0	0	0	0	0	1	Unique	COG4279_S#	32.4	91	3.15E-18	NA	31.4	78	9.63E-14
7568	OSC69942.1	-	hypothetical protein	0	1	0	0	0	0	0	0	0	0	0	1	Unique								
7569	OSC71643.1	-	transglutaminase	1	1	1	1	1	1	1	1	1	1	1	11	CORE	COG1305_E#	44.6	158	2.74E-38				
7570	WP_084206916.1	-	hypothetical protein	0	0	0	0	0	1	1	1	1	0	0	4	Accessory								
7571	WP_019526070.1	-	hypothetical protein	0	0	0	0	1	0	0	0	0	0	0	1	Unique								
7572	Prokka:JAPCLKMA_02635	-	hypothetical protein	1	0	0	0	0	0	0	0	0	0	0	1	Unique								
7573	AXI91101.1	-	DUF4913 domain-containing protein	0	0	0	1	0	0	0	0	0	0	0	1	Unique								
7574	OSC71271.1	-	hypothetical protein	1	1	1	1	0	0	0	0	1	0	0	5	Accessory								
7575	OSC69734.1	-	50S ribosomal protein L25/general stress protein Ctc	1	1	1	1	1	1	1	1	1	1	1	11	CORE	COG1825_J#	45.5	156	7.98E-38	K02897	45.5	156	2.13E-37

cluster	GI	gene	Annotation	Streptomyces_thermocarboxydus_BPSAC147	Streptomyces_sp._4F	Streptomyces_sp._BSE7F	Streptomyces_sp._ETH9427	Streptomyces_sp._FxanaD5	Streptomyces_sp._UNC401CLCol	Actinospica_acidiphila_NRRL_B_24431	Streptomyces_sp._HNS054	Streptomyces_sp._SMS_SU21	Streptomyces_sp._ZS0098	Streptomyces_thermocarboxydus_K155	Addition	Core-Accessory-Unique	COG	%Similarity_COG	Score_COG	Evalue_COG	KEGG	%Similarity_KEGG	Score_KEGG	Evalue_KEGG
7576	PWE06621.1	-	methyltransferase	0	0	1	0	0	0	0	0	0	0	0	1	Unique	COG4725_TK#	30	49	0.0000234				
7577	OSC71565.1	-	N-acetyltransferase	0	1	0	0	0	0	0	0	0	0	0	1	Unique	COG0454_KR#	34	107	4.25E-23	K00680	34	107	1.14E-22
7578	Prokka:CIODPCDJ_06791	-	hypothetical protein	0	1	0	0	1	0	0	0	0	0	1	3	Accessory								
7579	WP_102637672.1	-	TetR/AcrR family transcriptional regulator	0	0	1	0	0	0	1	0	1	0	0	3	Accessory	COG1309_K#	44.4	157	6.11E-38	NA	39	63	2.46E-09
7580	WP_157842177.1	-	hypothetical protein	0	0	0	0	0	0	1	0	0	0	0	1	Unique								
7581	Prokka:JAPCLKMA_05180	-	hypothetical protein	1	0	0	0	0	0	0	0	0	0	0	1	Unique								
7582	Prokka:JAPCLKMA_01191	-	hypothetical protein	1	1	0	0	1	1	0	0	0	0	1	5	Accessory								
7583	OSC65880.1	-	Na+/H+ antiporter subunit E	0	1	0	0	0	0	0	0	0	0	0	1	Unique	COG1863_P#	31.4	83	1.12E-15	K05569	29.4	61	9.33E-09
7584	PWE09301.1	-	M48 family peptidase	1	1	1	1	1	1	1	1	1	1	1	11	CORE	COG1451_R#	47.7	146	1.07E-34	K07043	34.6	63	3.19E-09
7585	WP_033275661.1	-	hypothetical protein	1	0	1	0	1	1	1	0	1	0	1	7	Accessory								
7586	WP_102641423.1	-	response regulator transcription factor	1	0	1	0	0	0	0	0	1	0	0	3	Accessory	COG2197_TK#	33.7	96	9.75E-20	NA	33.7	96	2.6E-19
7587	WP_037952887.1	-	hypothetical protein	1	0	1	1	1	1	1	1	1	1	1	10	Accessory								

cluster	GI	gene	Annotation	Streptomyces_thermocarboxydus_BPSAC147	Streptomyces_sp._4F	Streptomyces_sp._BSE7F	Streptomyces_sp._ETH9427	Streptomyces_sp._FxanaD5	Streptomyces_sp._UNC401CLCol	Actinospica_acidiphila_NRRL_B_24431	Streptomyces_sp._HNS054	Streptomyces_sp._SMS_SU21	Streptomyces_sp._ZS0098	Streptomyces_thermocarboxydus_K155	Addition	Core-Accessory-Unique	COG	%Similarity_COG	Score_COG	Evalue_COG	KEGG	%Similarity_KEGG	Score_KEGG	Evalue_KEGG
7588	WP_053076993.1	-	TetR/AcrR family transcriptional regulator	0	0	0	0	0	0	0	1	0	0	0	1	Unique	COG1309_K#	28.4	62	2.04E-09	K18939	23.6	60	2.07E-08
7589	PWE08655.1	-	hypothetical protein	0	0	1	0	0	0	0	1	0	0	0	2	Accessory								
7590	Prokka:CIODPCDJ_03842	-	hypothetical protein	1	1	0	0	1	1	1	0	0	0	1	6	Accessory								
7591	WP_122215910.1	-	DUF2461 family protein	0	0	0	0	0	0	0	0	0	1	0	1	Unique								
7592	WP_048455407.1	-	hypothetical protein	1	1	1	1	1	1	1	1	1	1	1	11	CORE								
7593	OSC68437.1	-	hypothetical protein	0	1	0	0	0	0	0	0	0	0	0	1	Unique	COG3583_S#	41	59	2.25E-08	NA	40.9	58	7.86E-08
7594	WP_122213821.1	-	phage gp6-like head-tail connector protein	0	0	0	0	0	0	0	0	0	1	0	1	Unique								
7595	Prokka:JAPCLKMA_06811	-	hypothetical protein	1	0	0	0	0	0	1	1	0	0	0	3	Accessory								
7596	WP_019523875.1	-	hydrogenase maturation protease	1	0	0	1	1	1	0	1	0	1	1	7	Accessory	COG0680_C#	30.7	59	2.25E-08				
7597	Prokka:JAPCLKMA_06414	-	hypothetical protein	1	0	1	1	0	1	1	1	1	1	1	9	Accessory	COG1309_K#	34.4	106	1.23E-22	NA	32	77	1.63E-13
7598	Prokka:JAPCLKMA_06413	gpm2_2	Acid phosphatase	1	1	1	1	1	1	1	1	1	1	1	11	CORE	COG0406_G#	49	181	3.93E-45	K15634	41.1	146	2.87E-34
7599	WP_048459880.1	-	hypothetical protein	1	0	0	1	1	1	0	1	0	1	1	7	Accessory								

cluster	GI	gene	Annotation	Streptomyces_thermocarboxydus_BPSAC147	Streptomyces_sp._4F	Streptomyces_sp._BSE7F	Streptomyces_sp._ETH9427	Streptomyces_sp._FxanaD5	Streptomyces_sp._UNC401CLCol	Actinospica_acidiphila_NRRL_B_24431	Streptomyces_sp._HNS054	Streptomyces_sp._SMS_SU21	Streptomyces_sp._ZS0098	Streptomyces_thermocarboxydus_K155	Addition	Core-Accessory-Unique	COG	%Similarity_COG	Score_COG	Evalue_COG	KEGG	%Similarity_KEGG	Score_KEGG	Evalue_KEGG
7600	WP_078611411.1	-	response regulator transcription factor	1	0	0	0	0	1	0	0	0	0	1	3	Accessory	COG2197_TK#	53.5	191	2.23E-48	K07693	53.5	191	5.95E-48
7601	WP_033274288.1	-	TetR family transcriptional regulator	1	0	0	0	1	1	1	0	0	0	1	5	Accessory	COG1309_K#	29	67	8.27E-11	NA	28.1	66	3.77E-10
7602	Prokka:CIODPCDJ_03696	-	hypothetical protein	1	1	0	1	1	1	1	1	1	1	1	10	Accessory	COG1309_K#	35.4	67	8.27E-11	NA	35.4	67	2.21E-10
7603	Prokka:JAPCLKMA_02608	wbpD	UDP-2-acetamido-3-amino-2,3-dideoxy-D-glucuronate N-acetyltransferase	1	0	0	0	0	0	0	0	0	0	0	1	Unique	COG0110_R#	42.8	149	1.27E-35	K00674	34.4	76	3.64E-13
7604	Prokka:JAPCLKMA_01586	-	hypothetical protein	1	0	0	0	0	0	0	0	0	0	0	1	Unique	COG2020_O#	47	121	2.83E-27	NA	47	121	7.56E-27
7605	OSC72768.1	-	Suppressor of fused domain protein	1	1	1	1	1	1	1	1	1	1	1	11	CORE								
7606	Prokka:CIODPCDJ_04376	-	hypothetical protein	1	0	1	1	1	1	1	1	1	1	1	10	Accessory								
7607	WP_019524859.1	-	TetR/AcrR family transcriptional regulator	0	0	0	0	1	1	0	0	0	0	1	3	Accessory	COG1309_K#	44.9	116	9.1E-26	NA	44.9	116	2.43E-25

cluster	GI	gene	Annotation	Streptomyces_thermocarboxydus_BPSAC147	Streptomyces_sp._4F	Streptomyces_sp._BSE7F	Streptomyces_sp._ETH9427	Streptomyces_sp._FxanaD5	Streptomyces_sp._UNC401CLCol	Actinospica_acidiphila_NRRL_B_24431	Streptomyces_sp._HNS054	Streptomyces_sp._SMS_SU21	Streptomyces_sp._ZS0098	Streptomyces_thermocarboxydus_K155	Addition	Core-Accessory-Unique	COG	%Similarity_COG	Score_COG	Evalue_COG	KEGG	%Similarity_KEGG	Score_KEGG	Evalue_KEGG
7608	OSC69583.1	-	TetR family transcriptional regulator	0	1	0	0	0	0	0	0	0	0	0	1	Unique	COG1309_K#	24.9	71	3.36E-12	NA	26.4	63	2.44E-09
7609	OSC65795.1	-	hypothetical protein	0	1	0	0	0	0	0	0	0	0	0	1	Unique								
7610	WP_102641975.1	-	hypothetical protein	0	0	0	0	0	0	1	1	1	1	0	4	Accessory								
7611	WP_048458588.1	-	helix-turn-helix domain-containing protein	0	0	0	0	0	0	0	1	0	0	0	1	Unique								
7612	OSC67128.1	-	hypothetical protein	0	1	0	0	0	0	0	0	0	0	0	1	Unique								
7613	OSC55325.1	-	TetR family transcriptional regulator	0	1	0	0	0	0	0	0	0	0	0	1	Unique	COG1309_K#	26	55	3.25E-07	NA	27.3	54	0.00000148
7614	OSC63263.1	-	polysaccharide deacetylase	0	1	0	0	0	0	0	0	0	0	0	1	Unique	COG0726_G#	32.7	77	7.99E-14	NA	31	66	3.77E-10
7615	Prokka:JAPCLKMA_03681	-	hypothetical protein	1	1	1	1	1	1	1	1	1	1	1	11	CORE	COG1595_K#	30.4	72	1.51E-12	K03090	35.4	49	0.0000477
7616	OSC69666.1	-	hypothetical protein	0	1	0	0	0	0	0	0	0	0	0	1	Unique								
7617	WP_102640741.1	-	hypothetical protein	1	0	1	1	1	1	1	1	1	1	1	10	Accessory								
7618	WP_122212646.1	-	TetR family transcriptional regulator	1	1	1	1	0	1	1	1	1	1	1	10	Accessory	COG3226_S#	42.5	144	4.08E-34	NA	42.5	143	2.43E-33
7619	OSC71195.1	-	hypothetical protein	0	1	0	0	0	0	0	0	0	0	0	1	Unique								

cluster	GI	gene	Annotation	Streptomyces_thermocarboxydus_BPSAC147	Streptomyces_sp._4F	Streptomyces_sp._BSE7F	Streptomyces_sp._ETH9427	Streptomyces_sp._FxanaD5	Streptomyces_sp._UNC401CLCol	Actinospica_acidiphila_NRRL_B_24431	Streptomyces_sp._HNS054	Streptomyces_sp._SMS_SU21	Streptomyces_sp._ZS0098	Streptomyces_thermocarboxydus_K155	Addition	Core-Accessory-Unique	COG	%Similarity_COG	Score_COG	Evalue_COG	KEGG	%Similarity_KEGG	Score_KEGG	Evalue_KEGG
7620	OSC62503.1	-	LuxR family transcriptional regulator	0	1	0	0	0	0	0	0	0	0	0	1	Unique	COG2771_K#	32.4	63	1.19E-09	K02479	34.3	51	0.0000125
7621	OSC70543.1	-	TetR family transcriptional regulator	0	1	0	0	0	0	0	1	0	0	0	2	Accessory	COG1309_K#	29.7	72	2.57E-12	K03577	34.9	52	0.00000431
7622	OSC72280.1	-	hypothetical protein	0	1	0	0	0	0	0	0	0	0	0	1	Unique								
7623	OSC72084.1	-	deaminase	0	1	0	0	0	0	0	0	0	0	0	1	Unique								
7624	WP_019525013.1	-	winged helix-turn-helix transcriptional regulator	1	0	1	0	1	1	1	1	1	1	1	9	Accessory	COG0745_TK#	50.5	89	1.55E-17	NA	50.5	89	4.14E-17
7625	WP_033273032.1	hisB	imidazoleglycerol-phosphate dehydratase HisB	1	1	1	1	1	1	1	1	1	1	1	11	CORE	COG0131_E#	57.9	234	3.89E-61	K01693	57.9	234	1.04E-60
7626	Prokka:CIODPCDJ_04514	-	hypothetical protein	1	1	1	1	1	1	1	1	1	1	1	11	CORE								
7627	WP_019524965.1	-	isopentenyl-diphosphate Delta-isomerase	1	1	1	1	1	1	1	1	1	1	1	11	CORE	COG1443_I#	37.6	96	1.27E-19	K01823	37.6	94	1.68E-18
7628	WP_106432552.1	-	D-alanyl-alanine synthetase A	0	0	0	0	1	0	0	0	0	0	0	1	Unique	COG1181_M#	33.3	94	4.81E-19	K01921	34.8	92	4.89E-18
7629	AXI89529.1	-	hypothetical protein	0	0	0	1	0	0	0	1	0	0	0	2	Accessory								

cluster	GI	gene	Annotation	Streptomyces_thermocarboxydus_BPSAC147	Streptomyces_sp._4F	Streptomyces_sp._BSE7F	Streptomyces_sp._ETH9427	Streptomyces_sp._FxanaD5	Streptomyces_sp._UNC401CLCol	Actinospica_acidiphila_NRRL_B_24431	Streptomyces_sp._HNS054	Streptomyces_sp._SMS_SU21	Streptomyces_sp._ZS0098	Streptomyces_thermocarboxydus_K155	Addition	Core-Accessory-Unique	COG	%Similarity_COG	Score_COG	Evalue_COG	KEGG	%Similarity_KEGG	Score_KEGG	Evalue_KEGG
7630	Prokka:JAPCLKMA_03083	-	hypothetical protein	1	1	1	1	1	1	1	1	1	1	1	11	CORE	COG0412_Q#	37.9	121	2.82E-27				
7631	WP_147474358.1	-	hypothetical protein	0	0	0	0	0	0	0	0	0	1	0	1	Unique								
7632	WP_033274823.1	-	Uma2 family endonuclease	0	0	0	0	0	0	1	0	0	0	0	1	Unique								
7633	OSC61405.1	-	DNA starvation/stationary phase protection protein	0	1	0	0	0	0	0	0	0	0	0	1	Unique	COG0783_P#	34.2	91	4.08E-18	K04047	31.4	62	7.07E-09
7634	WP_033275879.1	leuD	3-isopropylmalate dehydratase small subunit	1	1	1	1	1	1	1	1	1	1	1	11	CORE	COG0066_E#	63.1	244	3.76E-64	K01704	60.9	236	2.74E-61
7635	WP_051724516.1	gmk	guanylate kinase	1	1	1	1	1	1	1	1	1	1	1	11	CORE	COG0194_F#	49	182	1.34E-45	K00942	50	181	8E-45
7636	AXI90948.1	-	TetR family transcriptional regulator	1	0	1	1	1	1	1	1	1	1	1	10	Accessory	COG1309_K#	42.9	137	6.48E-32	NA	36.5	96	3.38E-19
7637	WP_019526294.1	-	hypothetical protein	1	0	0	1	1	1	1	1	1	1	1	9	Accessory								
7638	OSC73358.1	-	TetR family transcriptional regulator	1	1	1	1	1	1	1	1	1	1	1	11	CORE	COG1309_K#	32.8	109	1.11E-23	NA	32.8	109	2.96E-23
7639	WP_048459984.1	-	phosphoglycerate mutase	1	1	1	1	1	1	1	1	1	1	1	11	CORE	COG0406_G#	33.2	103	1.04E-21	K15634	29.5	53	0.00000252

cluster	GI	gene	Annotation	Streptomyces_thermocarboxydus_BPSAC147	Streptomyces_sp._4F	Streptomyces_sp._BSE7F	Streptomyces_sp._ETH9427	Streptomyces_sp._FxanaD5	Streptomyces_sp._UNC401CLCol	Actinospica_acidiphila_NRRL_B_24431	Streptomyces_sp._HNS054	Streptomyces_sp._SMS_SU21	Streptomyces_sp._ZS0098	Streptomyces_thermocarboxydus_K155	Addition	Core-Accessory-Unique	COG	%Similarity_COG	Score_COG	Evalue_COG	KEGG	%Similarity_KEGG	Score_KEGG	Evalue_KEGG
7640	AXI84678.1	-	TetR/AcrR family transcriptional regulator	0	0	0	1	0	0	0	0	0	0	0	1	Unique	COG1309_K#	34.3	104	3.56E-22	NA	34.8	90	1.86E-17
7641	OSC68617.1	-	hypothetical protein	0	1	0	0	0	0	0	0	0	0	0	1	Unique								
7642	WP_048458348.1	-	CGNR zinc finger domain-containing protein	1	0	1	1	1	1	1	1	1	1	1	10	Accessory	COG5516_R#	31.7	57	8.52E-08				
7643	Prokka:JAPCLKMA_04944	-	hypothetical protein	1	0	0	0	1	1	0	0	0	0	1	4	Accessory								
7644	AXI87179.1	-	electron transporter	0	1	1	1	0	0	1	1	1	1	0	7	Accessory								
7645	WP_033274110.1	-	hypothetical protein	1	1	1	1	1	1	1	1	1	1	1	11	CORE	COG4430_S#	54.5	203	7.36E-52	NA	30.1	82	3.87E-15
7646	WP_102639764.1	-	PH domain-containing protein	1	1	1	1	1	1	1	1	1	1	1	11	CORE	COG3402_S#	42.1	113	7.67E-25	K09167	33	54	0.00000147
7647	PWE11504.1	-	ABC transporter	0	0	1	0	0	0	0	0	0	0	0	1	Unique	COG1131_V#	33.5	111	3.81E-24	K05650	35.5	109	5.04E-23
7648	OSC60326.1	-	hypothetical protein	1	1	1	1	1	1	1	1	1	1	1	11	CORE								
7649	WP_019526045.1	-	hypothetical protein	0	0	0	0	1	0	0	0	0	0	0	1	Unique								
7650	OSC60085.1	-	TetR family transcriptional regulator	0	1	0	0	0	0	0	0	0	0	0	1	Unique	COG1309_K#	43.8	150	7.39E-36	NA	43.8	150	1.97E-35
7651	OSC62996.1	-	oxidoreductase	1	1	1	1	1	1	1	1	1	1	1	11	CORE	COG2080_C#	54.9	98	2.55E-20	K03518	44.4	92	6.38E-18

cluster	GI	gene	Annotation	Streptomyces_thermocarboxydus_BPSAC147	Streptomyces_sp._4F	Streptomyces_sp._BSE7F	Streptomyces_sp._ETH9427	Streptomyces_sp._FxanaD5	Streptomyces_sp._UNC401CLCol	Actinospica_acidiphila_NRRL_B_24431	Streptomyces_sp._HNS054	Streptomyces_sp._SMS_SU21	Streptomyces_sp._ZS0098	Streptomyces_thermocarboxydus_K155	Addition	Core-Accessory-Unique	COG	%Similarity_COG	Score_COG	Evalue_COG	KEGG	%Similarity_KEGG	Score_KEGG	Evalue_KEGG
7652	OSC69491.1	-	bifunctional pyr operon transcriptional regulator/uracil phosphoribosyltransferase	1	1	1	1	1	1	1	1	1	1	1	11	CORE	COG2065_F#	56.9	210	7.86E-54	K02825	56.9	210	2.1E-53
7653	WP_107080959.1	-	ATP-binding protein	0	0	0	0	0	0	0	1	1	0	0	2	Accessory								
7654	Prokka:JAPCLKMA_06125	-	hypothetical protein	1	0	0	0	0	0	0	0	0	0	0	1	Unique								
7655	Prokka:JAPCLKMA_06538	-	hypothetical protein	1	1	0	0	0	0	1	0	0	0	1	4	Accessory								
7656	OSC57029.1	-	DNA polymerase III	0	1	0	0	0	0	0	0	0	0	0	1	Unique	COG5018_R#	37.4	99	1.15E-20				
7657	OSC70185.1	-	TetR family transcriptional regulator	0	1	0	0	0	0	0	0	0	0	0	1	Unique	COG1309_K#	30.4	67	8.23E-11	NA	30.3	60	1.58E-08
7658	Prokka:CIODPCDJ_05987	-	hypothetical protein	1	1	0	0	1	1	1	0	0	0	1	6	Accessory	COG0454_KR#	55	179	1.49E-44	K03829	35.1	53	0.00000328
7659	OSC61375.1	-	hypothetical protein	0	1	0	0	0	0	0	0	0	0	0	1	Unique								
7660	WP_019524912.1	-	hypothetical protein	0	0	0	0	1	0	0	0	0	0	0	1	Unique								
7661	WP_146217841.1	-	hypothetical protein	0	0	0	0	0	0	0	0	1	0	0	1	Unique					NA	61.1	144	1.08E-33
7662	WP_147474332.1	-	hypothetical protein	0	0	0	0	0	0	0	0	0	1	0	1	Unique								

cluster	GI	gene	Annotation	Streptomyces_thermocarboxydus_BPSAC147	Streptomyces_sp._4F	Streptomyces_sp._BSE7F	Streptomyces_sp._ETH9427	Streptomyces_sp._FxanaD5	Streptomyces_sp._UNC401CLCol	Actinospica_acidiphila_NRRL_B_24431	Streptomyces_sp._HNS054	Streptomyces_sp._SMS_SU21	Streptomyces_sp._ZS0098	Streptomyces_thermocarboxydus_K155	Addition	Core-Accessory-Unique	COG	%Similarity_COG	Score_COG	Evalue_COG	KEGG	%Similarity_KEGG	Score_KEGG	Evalue_KEGG
7663	WP_019523619.1	-	hemerythrin domain-containing protein	0	1	0	0	1	1	1	0	0	0	1	5	Accessory								
7664	WP_033276641.1	-	hypothetical protein	1	0	0	0	0	0	1	0	0	0	1	3	Accessory								
7665	OSC61213.1	-	TetR family transcriptional regulator	0	1	0	0	0	0	0	0	0	0	0	1	Unique	COG1309_K#	30.4	99	1.14E-20	NA	30.4	99	3.04E-20
7666	PWE09250.1	-	DNA-3-methyladenine glycosylase I	1	1	1	1	1	1	1	1	1	1	1	11	CORE	COG2818_L#	56.7	208	2.97E-53	K01246	56.7	208	7.94E-53
7667	PWE06955.1	-	TetR family transcriptional regulator	0	1	1	1	1	1	1	1	1	1	0	9	Accessory	COG3226_S#	43.1	121	4.78E-27	NA	43.1	121	1.28E-26
7668	Prokka:JAPCLKMA_04408	-	hypothetical protein	1	0	1	1	1	1	1	1	1	1	1	10	Accessory	COG1846_K#	33.6	82	1.88E-15	NA	36.5	97	1.16E-19
7669	OSC66607.1	-	hypothetical protein	1	1	1	1	1	1	1	1	1	1	1	11	CORE					NA	34.7	123	2.57E-27
7670	PWE06733.1	-	DUF3090 domain-containing protein	1	1	1	1	1	1	1	1	1	1	1	11	CORE					NA	55.7	198	4.82E-50
7671	WP_019523694.1	-	FxsA family protein	1	0	0	1	1	1	1	1	1	1	1	9	Accessory	COG3030_R#	27.1	59	2.23E-08	K07113	31.4	55	6.58E-07
7672	WP_086700107.1	-	single-stranded DNA-binding protein	1	1	1	1	1	1	1	1	1	1	1	11	CORE	COG0629_L#	54.9	217	4.9E-56	K03111	63	200	1.66E-50

cluster	GI	gene	Annotation	Streptomyces_thermocarboxydus_BPSAC147	Streptomyces_sp._4F	Streptomyces_sp._BSE7F	Streptomyces_sp._ETH9427	Streptomyces_sp._FxanaD5	Streptomyces_sp._UNC401CLCol	Actinospica_acidiphila_NRRL_B_24431	Streptomyces_sp._HNS054	Streptomyces_sp._SMS_SU21	Streptomyces_sp._ZS0098	Streptomyces_thermocarboxydus_K155	Addition	Core-Accessory-Unique	COG	%Similarity_COG	Score_COG	Evalue_COG	KEGG	%Similarity_KEGG	Score_KEGG	Evalue_KEGG
7673	AXI91204.1	-	hypothetical protein	0	0	0	1	0	0	0	0	0	0	0	1	Unique								
7674	Prokka:JAPCLKMA_01994	-	hypothetical protein	1	0	0	0	0	0	0	0	0	0	0	1	Unique								
7675	OSC67939.1	-	RNA polymerase subunit sigma-70	1	1	1	1	1	1	1	1	1	1	1	11	CORE	COG1595_K#	24.2	60	5.87E-09				
7676	AXI85064.1	-	hypothetical protein	1	0	1	1	1	1	1	1	1	1	1	10	Accessory								
7677	WP_048457494.1	-	Uma2 family endonuclease	0	0	0	0	0	0	0	1	0	1	0	2	Accessory								
7678	WP_102637553.1	-	hypothetical protein	0	0	0	1	0	0	0	1	1	0	0	3	Accessory								
7679	AXI86439.1	-	TetR/AcrR family transcriptional regulator	0	0	0	1	0	0	0	1	0	0	0	2	Accessory	COG1309_K#	27.5	56	1.11E-07	NA	29.6	53	0.00000327
7680	AXI91310.1	-	hypothetical protein	0	0	0	1	0	0	0	0	0	0	0	1	Unique								
7681	AXI88139.1	-	hypothetical protein	0	0	0	1	0	0	0	0	0	0	0	1	Unique								
7682	WP_078895337.1	ectA	diaminobutyrate acetyltransferase	0	1	1	1	0	0	0	1	1	1	0	6	Accessory	COG0454_KR#	38.5	90	6.92E-18				
7683	OSC71999.1	-	hypothetical protein	1	1	1	1	1	1	1	1	1	1	1	11	CORE	COG5516_R#	32.9	66	1.07E-10				
7684	Prokka:JAPCLKMA_01617	-	hypothetical protein	1	0	0	0	0	0	0	0	0	0	0	1	Unique	COG2372_R#	38.9	87	7.65E-17	K07156	32	66	2.86E-10
7685	OSC64764.1	-	cysteine hydrolase	0	1	0	0	0	0	0	0	0	0	0	1	Unique	COG1335_Q#	39.4	127	6.67E-29	NA	35.6	105	7.25E-22

cluster	GI	gene	Annotation	Streptomyces_thermocarboxydus_BPSAC147	Streptomyces_sp._4F	Streptomyces_sp._BSE7F	Streptomyces_sp._ETH9427	Streptomyces_sp._FxanaD5	Streptomyces_sp._UNC401CLCol	Actinospica_acidiphila_NRRL_B_24431	Streptomyces_sp._HNS054	Streptomyces_sp._SMS_SU21	Streptomyces_sp._ZS0098	Streptomyces_thermocarboxydus_K155	Addition	Core-Accessory-Unique	COG	%Similarity_COG	Score_COG	Evalue_COG	KEGG	%Similarity_KEGG	Score_KEGG	Evalue_KEGG
7686	WP_033277661.1	-	tripartite tricarboxylate transporter TctB family protein	1	1	1	1	1	1	1	1	1	1	1	11	CORE								
7687	Prokka:CIODPCDJ_03196	-	hypothetical protein	1	0	0	0	1	1	0	1	0	1	1	6	Accessory								
7688	PWE08034.1	-	Uma2 family endonuclease	0	1	1	1	0	0	0	1	1	1	0	6	Accessory								
7689	WP_102637878.1	rsmD	16S rRNA (guanine(966)-N(2))-methyltransferase RsmD	1	1	0	1	1	1	1	1	1	1	1	10	Accessory	COG0742_L#	48.6	156	7.82E-38	NA	48.6	156	2.09E-37
7690	WP_048456425.1	-	MarR family transcriptional regulator	1	1	0	1	1	1	1	1	1	1	1	10	Accessory	COG1846_K#	34	96	9.6E-20	NA	35.5	81	1.11E-14
7691	WP_033275186.1	-	pyridoxamine 5'-phosphate oxidase family protein	1	1	1	1	1	1	1	1	1	1	1	11	CORE								
7692	WP_102638119.1	-	hypothetical protein	0	0	0	0	0	0	0	0	1	0	0	1	Unique								
7693	Prokka:JAPCLKMA_02098	-	hypothetical protein	1	0	0	0	1	1	0	0	0	0	1	4	Accessory								

cluster	GI	gene	Annotation	Streptomyces_thermocarboxydus_BPSAC147	Streptomyces_sp._4F	Streptomyces_sp._BSE7F	Streptomyces_sp._ETH9427	Streptomyces_sp._FxanaD5	Streptomyces_sp._UNC401CLCol	Actinospica_acidiphila_NRRL_B_24431	Streptomyces_sp._HNS054	Streptomyces_sp._SMS_SU21	Streptomyces_sp._ZS0098	Streptomyces_thermocarboxydus_K155	Addition	Core-Accessory-Unique	COG	%Similarity_COG	Score_COG	Evalue_COG	KEGG	%Similarity_KEGG	Score_KEGG	Evalue_KEGG
7694	WP_030221188.1	-	sigma-70 family RNA polymerase sigma factor	1	1	1	1	1	1	1	1	1	1	1	11	CORE	COG1595_K#	46.7	161	3.17E-39	K03088	46.7	161	8.48E-39
7695	WP_019525076.1	-	isochorismatase family protein	1	1	1	1	1	1	1	1	1	1	1	11	CORE	COG1335_Q#	66.8	255	1.62E-67	K08281	66.8	255	4.31E-67
7696	PWE09790.1	-	hypothetical protein	0	0	1	0	0	0	0	0	0	0	0	1	Unique								
7697	OSC70645.1	-	hypothetical protein	0	1	0	0	0	0	0	0	0	0	0	1	Unique					NA	24.4	48	0.0000801
7698	OSC71055.1	-	dTDP-4-dehydrorhamnose 3,5-epimerase	0	1	0	0	0	0	0	0	0	0	0	1	Unique	COG1898_M#	43.7	158	3.51E-38	K01790	43.7	158	9.37E-38
7699	WP_102638109.1	-	hypothetical protein	1	0	0	0	1	1	1	0	1	1	1	7	Accessory								
7700	WP_026244201.1	-	SIS domain-containing protein	0	0	0	0	1	1	0	0	0	0	0	2	Accessory	COG0794_M#	40.6	109	1.1E-23	K08094	36.3	99	3.03E-20
7701	Prokka:CIODPCDJ_02095	-	hypothetical protein	1	0	1	1	1	1	1	1	1	1	1	10	Accessory	COG1309_K#	32	79	2.07E-14	NA	32	79	5.53E-14
7702	Prokka:CIODPCDJ_06672	-	hypothetical protein	1	0	0	0	1	1	0	0	0	0	1	4	Accessory	COG3247_S#	40.1	122	1.25E-27				
7703	WP_019526828.1	-	rhodanese-like domain-containing protein	1	1	1	1	1	1	1	1	1	1	1	11	CORE	COG0607_P#	38.3	107	4.17E-23	NA	35	92	3.7E-18

cluster	GI	gene	Annotation	Streptomyces_thermocarboxydus_BPSAC147	Streptomyces_sp._4F	Streptomyces_sp._BSE7F	Streptomyces_sp._ETH9427	Streptomyces_sp._FxanaD5	Streptomyces_sp._UNC401CLCol	Actinospica_acidiphila_NRRL_B_24431	Streptomyces_sp._HNS054	Streptomyces_sp._SMS_SU21	Streptomyces_sp._ZS0098	Streptomyces_thermocarboxydus_K155	Addition	Core-Accessory-Unique	COG	%Similarity_COG	Score_COG	Evalue_COG	KEGG	%Similarity_KEGG	Score_KEGG	Evalue_KEGG
7704	WP_102641251.1	-	GNAT family N-acetyltransferase	1	0	1	1	1	1	1	1	1	1	1	10	Accessory	COG1670_J#	46.4	161	4.15E-39	K03790	33.1	87	2.03E-16
7705	OSC73072.1	-	PadR family transcriptional regulator	0	1	0	0	0	0	0	0	0	0	0	1	Unique	COG1695_K#	35.6	106	1.58E-22	NA	28.7	90	2.4E-17
7706	PWE10278.1	-	TetR family transcriptional regulator	0	0	1	0	0	0	0	0	0	0	0	1	Unique	COG1309_K#	28.4	70	9.62E-12	NA	28.4	70	2.57E-11
7707	WP_028961725.1	-	hypothetical protein	1	1	1	1	1	1	1	1	1	1	1	11	CORE								
7708	PWE07485.1	-	hypothetical protein	0	0	1	0	0	0	0	0	1	0	0	2	Accessory	COG2812_L#	34.5	62	2.01E-09	NA	34.1	69	3.36E-11
7709	OSC66412.1	-	hypothetical protein	0	1	0	0	0	0	0	0	0	0	0	1	Unique								
7710	OSC71430.1	-	hypothetical protein	1	1	1	1	1	1	1	1	1	1	1	11	CORE								
7711	WP_159020945.1	-	hypothetical protein	0	0	0	0	0	0	0	1	0	0	0	1	Unique								
7712	AXI87620.1	-	hypothetical protein	1	0	0	1	1	1	0	1	0	0	1	6	Accessory								
7713	WP_048460258.1	-	hypothetical protein	0	0	0	1	0	0	0	1	0	0	0	2	Accessory								
7714	AXI91223.1	-	hypothetical protein	0	0	0	1	0	0	0	0	0	0	0	1	Unique								
7715	WP_146217842.1	-	DUF3450 domain-containing protein	0	0	0	0	0	0	0	0	1	0	0	1	Unique	COG1511_S#	41	129	1.34E-29				
7716	Prokka:JAPCLKMA_06731	rppH_10	RNA pyrophosphohydrolase	1	0	1	1	1	1	1	1	1	1	1	10	Accessory	COG0494_LR#	32.7	51	0.00000354	K03574	32.7	51	0.00000946

cluster	GI	gene	Annotation	Streptomyces_thermocarboxydus_BPSAC147	Streptomyces_sp._4F	Streptomyces_sp._BSE7F	Streptomyces_sp._ETH9427	Streptomyces_sp._FxanaD5	Streptomyces_sp._UNC401CLCol	Actinospica_acidiphila_NRRL_B_24431	Streptomyces_sp._HNS054	Streptomyces_sp._SMS_SU21	Streptomyces_sp._ZS0098	Streptomyces_thermocarboxydus_K155	Addition	Core-Accessory-Unique	COG	%Similarity_COG	Score_COG	Evalue_COG	KEGG	%Similarity_KEGG	Score_KEGG	Evalue_KEGG
7717	OSC69214.1	-	hypothetical protein	0	1	0	0	0	0	0	0	0	0	0	1	Unique	COG5635_T#	40.7	113	9.91E-25	NA	51.8	109	2.93E-23
7718	PWE12178.1	-	TetR/AcrR family transcriptional regulator	0	0	1	1	0	0	0	1	1	0	0	4	Accessory	COG1309_K#	33.9	110	6.43E-24	NA	33.9	110	1.72E-23
7719	AXI90975.1	-	restriction endonuclease	0	0	0	1	0	0	0	1	0	1	0	3	Accessory								
7720	OSC57678.1	-	hypothetical protein	1	1	1	1	1	1	1	1	1	1	1	11	CORE								
7721	OSC62696.1	-	hypothetical protein	0	1	0	0	0	0	0	0	0	0	0	1	Unique								
7722	OSC66147.1	-	hypothetical protein	0	1	0	0	0	0	0	0	0	0	0	1	Unique								
7723	WP_122215929.1	-	CYTH domain-containing protein	0	0	0	0	0	0	0	0	0	1	0	1	Unique								
7724	OSC57386.1	-	carboxymuconolactone decarboxylase family protein	0	1	0	0	0	0	0	0	0	0	0	1	Unique	COG2128_S#	37.7	122	1.25E-27	K07486	36.7	123	2.56E-27
7725	OSC55109.1	-	TetR family transcriptional regulator	0	1	0	0	0	0	0	0	0	0	0	1	Unique	COG1309_K#	32	63	1.18E-09	NA	29.9	62	7E-09
7726	PWE07239.1	-	hypothetical protein	0	0	1	0	0	0	0	0	1	0	0	2	Accessory								
7727	OSC71197.1	-	hypothetical protein	0	1	0	0	0	0	0	0	0	0	0	1	Unique								
7728	OSC70310.1	-	stress protein	1	1	1	1	1	1	1	1	1	1	1	11	CORE	COG2310_T#	32.4	87	4.44E-17	K05791	32.4	87	1.19E-16
7729	OSC61134.1	-	phosphodiesterase	1	1	1	1	1	1	1	1	1	1	1	11	CORE								

cluster	GI	gene	Annotation	Streptomyces_thermocarboxydus_BPSAC147	Streptomyces_sp._4F	Streptomyces_sp._BSE7F	Streptomyces_sp._ETH9427	Streptomyces_sp._FxanaD5	Streptomyces_sp._UNC401CLCol	Actinospica_acidiphila_NRRL_B_24431	Streptomyces_sp._HNS054	Streptomyces_sp._SMS_SU21	Streptomyces_sp._ZS0098	Streptomyces_thermocarboxydus_K155	Addition	Core-Accessory-Unique	COG	%Similarity_COG	Score_COG	Evalue_COG	KEGG	%Similarity_KEGG	Score_KEGG	Evalue_KEGG
7730	WP_026244054.1	-	PaaI family thioesterase	1	1	1	1	1	1	1	1	1	1	1	11	CORE					NA	29.3	54	0.00000145
7731	Prokka:JAPCLKMA_04285	yyaP_2	putative protein YyaP	1	0	0	0	1	1	0	0	0	0	1	4	Accessory								
7732	WP_086020204.1	-	hypothetical protein	0	0	0	0	1	1	0	0	0	0	0	2	Accessory								
7733	WP_048460079.1	-	hypothetical protein	0	0	1	0	0	0	0	1	0	0	0	2	Accessory								
7734	PWE07287.1	-	hypothetical protein	0	0	1	0	0	0	0	0	1	1	0	3	Accessory								
7735	WP_122215251.1	-	flavodoxin family protein	0	0	1	0	0	0	1	1	1	1	0	5	Accessory	COG0655_R#	34.1	110	6.39E-24	NA	33	102	4.65E-21
7736	WP_033276858.1	-	biotin transporter BioY	1	1	1	1	1	1	1	1	1	1	1	11	CORE	COG1268_R#	44.4	115	1.99E-25	K03523	28.6	75	4.66E-13
7737	WP_078492847.1	-	sigma-70 family RNA polymerase sigma factor	1	1	1	1	1	1	1	1	1	1	1	11	CORE	COG1595_K#	42.8	129	1.33E-29	K03088	42.8	129	3.55E-29
7738	OSC72323.1	-	photosystem reaction center subunit H	0	1	0	0	0	0	0	0	0	0	0	1	Unique								
7739	OSC54508.1	-	tellurium resistance protein	0	1	1	1	1	0	0	1	1	1	0	7	Accessory	COG3793_P#	41.1	117	6.83E-26	K05793	41.1	117	1.82E-25
7740	Prokka:JAPCLKMA_03057	-	hypothetical protein	1	0	1	0	1	1	1	0	1	0	1	7	Accessory								
7741	Prokka:JAPCLKMA_00899	-	hypothetical protein	1	0	0	0	1	1	0	0	0	0	1	4	Accessory	COG2939_E#	24.3	46	0.000113	K14775	29.6	62	5.33E-09

cluster	GI	gene	Annotation	Streptomyces_thermocarboxydus_BPSAC147	Streptomyces_sp._4F	Streptomyces_sp._BSE7F	Streptomyces_sp._ETH9427	Streptomyces_sp._FxanaD5	Streptomyces_sp._UNC401CLCol	Actinospica_acidiphila_NRRL_B_24431	Streptomyces_sp._HNS054	Streptomyces_sp._SMS_SU21	Streptomyces_sp._ZS0098	Streptomyces_thermocarboxydus_K155	Addition	Core-Accessory-Unique	COG	%Similarity_COG	Score_COG	Evalue_COG	KEGG	%Similarity_KEGG	Score_KEGG	Evalue_KEGG
7742	WP_106959428.1	-	acyl-CoA thioesterase	1	1	1	1	1	1	1	1	1	1	1	11	CORE	COG1607_I#	41	121	4.73E-27	NA	45.2	111	7.66E-24
7743	OSC64791.1	-	XRE family transcriptional regulator	0	1	0	0	0	0	0	0	0	0	0	1	Unique	COG1396_K#	35.9	132	2.05E-30	NA	27.8	75	6.09E-13
7744	Prokka:JAPCLKMA_03711	-	hypothetical protein	1	0	0	0	0	0	0	0	0	0	0	1	Unique								
7745	PWE10939.1	-	hypothetical protein	0	0	1	0	0	0	0	0	0	0	0	1	Unique								
7746	Prokka:JAPCLKMA_03067	-	hypothetical protein	1	1	1	1	1	1	1	1	1	1	1	11	CORE								
7747	WP_102637678.1	-	sigma-70 family RNA polymerase sigma factor	1	1	1	1	1	1	1	1	1	1	1	11	CORE	COG1595_K#	45.9	159	1.57E-38	K03088	45.9	159	4.19E-38
7748	AXI91292.1	-	hypothetical protein	0	0	0	1	0	0	0	0	0	0	0	1	Unique								
7749	OSC70458.1	-	GNAT family N-acetyltransferase	1	1	1	1	1	1	1	1	1	1	1	11	CORE	COG1670_J#	30.8	83	1.09E-15	K03790	30.8	83	2.92E-15
7750	OSC72968.1	-	DUF5134 domain-containing protein	1	1	1	1	1	1	1	1	1	1	1	11	CORE								
7751	OSC69123.1	-	TetR family transcriptional regulator	0	1	0	0	0	0	0	0	0	0	0	1	Unique	COG1309_K#	37.9	131	2.68E-30	K16137	38.5	132	3.21E-30
7752	OSC54911.1	-	lipase	0	1	0	0	0	0	0	0	0	0	0	1	Unique	COG2755_E#	36	97	4.29E-20				

cluster	GI	gene	Annotation	Streptomyces_thermocarboxydus_BPSAC147	Streptomyces_sp._4F	Streptomyces_sp._BSE7F	Streptomyces_sp._ETH9427	Streptomyces_sp._FxanaD5	Streptomyces_sp._UNC401CLCol	Actinospica_acidiphila_NRRL_B_24431	Streptomyces_sp._HNS054	Streptomyces_sp._SMS_SU21	Streptomyces_sp._ZS0098	Streptomyces_thermocarboxydus_K155	Addition	Core-Accessory-Unique	COG	%Similarity_COG	Score_COG	Evalue_COG	KEGG	%Similarity_KEGG	Score_KEGG	Evalue_KEGG
7753	OSC71844.1	-	hypothetical protein	1	1	1	1	1	1	1	1	1	1	1	11	CORE								
7754	AXI91378.1	-	hypothetical protein	0	0	0	1	0	0	0	0	0	0	0	1	Unique								
7755	Prokka:JAPCLKMA_05374	yfcD	putative Nudix hydrolase YfcD	1	1	1	1	1	1	1	1	1	1	1	11	CORE	COG0494_LR#	38.9	102	2.27E-21	NA	38.9	102	6.07E-21
7756	OSC62768.1	-	hypothetical protein	0	1	0	0	0	0	0	0	0	0	0	1	Unique								
7757	WP_048455349.1	-	hypothetical protein	0	0	0	1	0	0	0	1	0	0	0	2	Accessory								
7758	Prokka:JAPCLKMA_04920	-	hypothetical protein	1	0	0	0	0	0	0	0	0	0	0	1	Unique								
7759	WP_048460196.1	-	HDED protein	1	0	1	1	1	1	1	1	1	1	1	10	Accessory	COG3247_S#	31.3	89	1.16E-17				
7760	WP_048460652.1	-	sugar O-acetyltransferase	0	1	1	1	0	0	1	1	1	1	0	7	Accessory	COG0110_R#	53	200	7.97E-51	K00661	48.1	170	1.38E-41
7761	OSC70580.1	-	hypothetical protein	0	1	0	0	0	0	0	0	0	0	0	1	Unique								
7762	WP_026244123.1	-	TetR family transcriptional regulator	1	0	0	0	1	1	0	0	0	0	1	4	Accessory	COG3226_S#	31.4	66	1.38E-10	NA	31.4	65	6.27E-10
7763	WP_086020189.1	-	YqgE/AlgH family protein	0	1	1	1	1	1	1	1	1	1	1	10	Accessory	COG1678_K#	45.4	154	5.02E-37	K07735	45.4	154	1.34E-36
7764	WP_102638399.1	-	TetR/AcrR family transcriptional regulator	1	0	1	1	1	1	1	1	1	1	1	10	Accessory	COG1309_K#	40.7	105	2.67E-22	NA	37	82	3.79E-15
7765	Prokka:CIODPCDJ_06702	cynT	Carbonic anhydrase 1	0	1	1	0	0	1	0	0	0	0	1	4	Accessory	COG0288_P#	45.5	159	1.56E-38	K01673	40.4	146	3.65E-34

cluster	GI	gene	Annotation	Streptomyces_thermocarboxydus_BPSAC147	Streptomyces_sp._4F	Streptomyces_sp._BSE7F	Streptomyces_sp._ETH9427	Streptomyces_sp._FxanaD5	Streptomyces_sp._UNC401CLCol	Actinospica_acidiphila_NRRL_B_24431	Streptomyces_sp._HNS054	Streptomyces_sp._SMS_SU21	Streptomyces_sp._ZS0098	Streptomyces_thermocarboxydus_K155	Addition	Core-Accessory-Unique	COG	%Similarity_COG	Score_COG	Evalue_COG	KEGG	%Similarity_KEGG	Score_KEGG	Evalue_KEGG
7766	WP_102637062.1	-	rhodanese-like domain-containing protein	0	0	1	1	0	0	0	1	1	1	0	5	Accessory	COG0607_P#	47.2	163	1.08E-39	NA	41.5	124	8.7E-28
7767	OSC65553.1	-	hypothetical protein	0	1	0	0	0	0	0	0	0	0	0	1	Unique								
7768	OSC58248.1	-	hypothetical protein	0	1	0	0	0	0	0	0	0	0	0	1	Unique	COG0859_M#	29.4	55	3.17E-07				
7769	PWE10450.1	-	transcriptional regulator	1	1	1	1	1	1	1	1	1	1	1	11	CORE	COG1396_K#	41	134	3.15E-31	NA	27.6	59	4.49E-08
7770	WP_048455987.1	-	hypothetical protein	1	1	1	1	1	1	1	1	1	1	1	11	CORE								
7771	OSC46875.1	-	chromosome partitioning protein ParB	0	1	0	0	0	0	0	0	0	0	0	1	Unique	COG1475_K#	31.2	75	2.27E-13				
7772	OSC70120.1	-	hypothetical protein	1	1	1	1	1	1	1	1	1	1	1	11	CORE								
7773	PWE11848.1	-	hypothetical protein	0	0	1	0	0	0	0	0	0	0	0	1	Unique	COG0346_E#	39.4	59	1.29E-08				
7774	WP_122213858.1	-	hypothetical protein	0	0	0	0	0	0	0	0	0	1	0	1	Unique								
7775	OSC71227.1	-	hypothetical protein	0	1	0	0	0	0	0	0	0	0	0	1	Unique								
7776	OSC71047.1	-	ABC transporter permease	0	1	0	0	0	0	0	0	0	0	0	1	Unique								
7777	Prokka:JAPCLKMA_03133	-	hypothetical protein	1	0	0	0	0	0	0	0	0	0	0	1	Unique								
7778	OSC63999.1	-	hypothetical protein	0	1	0	0	0	0	0	0	0	0	0	1	Unique								

cluster	GI	gene	Annotation	Streptomyces_thermocarboxydus_BPSAC147	Streptomyces_sp._4F	Streptomyces_sp._BSE7F	Streptomyces_sp._ETH9427	Streptomyces_sp._FxanaD5	Streptomyces_sp._UNC401CLCol	Actinospica_acidiphila_NRRL_B_24431	Streptomyces_sp._HNS054	Streptomyces_sp._SMS_SU21	Streptomyces_sp._ZS0098	Streptomyces_thermocarboxydus_K155	Addition	Core-Accessory-Unique	COG	%Similarity_COG	Score_COG	Evalue_COG	KEGG	%Similarity_KEGG	Score_KEGG	Evalue_KEGG
7779	OSC57248.1	-	TetR family transcriptional regulator	0	1	0	0	0	0	0	0	0	0	0	1	Unique	COG1309_K#	32.5	106	1.56E-22	K16137	33	107	1.87E-22
7780	OSC55845.1	-	PucR family transcriptional regulator	0	1	0	0	0	0	0	0	0	0	0	1	Unique	COG2508_TQ#	34.6	88	2.57E-17	NA	22.9	57	0.00000017
7781	WP_122214961.1	-	hypothetical protein	1	1	1	1	1	1	1	1	1	1	1	11	CORE								
7782	WP_019524448.1	-	hypothetical protein	1	1	0	0	1	1	1	0	0	0	1	6	Accessory								
7783	WP_019527859.1	-	2'-5' RNA ligase family protein	1	1	1	1	1	1	1	1	1	1	1	11	CORE								
7784	AXI88399.1	-	hypothetical protein	1	1	1	1	1	1	1	1	1	1	1	11	CORE								
7785	OSC69685.1	-	phosphohydrolase	0	1	0	0	0	0	0	0	0	1	0	2	Accessory	COG1418_R#	31	41	0.00471				
7786	OSC57952.1	-	TetR family transcriptional regulator	0	1	0	0	0	0	0	0	0	0	0	1	Unique	COG1309_K#	43	136	1.41E-31	NA	38.7	109	4.92E-23
7787	OSC56511.1	-	hypothetical protein	0	1	0	0	0	0	0	0	0	0	0	1	Unique	COG2091_H#	28.7	50	0.0000101	K06133	28.7	49	0.0000462
7788	WP_078894979.1	-	TetR/AcrR family transcriptional regulator	0	0	1	1	0	0	0	1	1	0	0	4	Accessory	COG1309_K#	26.9	62	1.51E-09	NA	29.9	57	2.22E-07

cluster	GI	gene	Annotation	Streptomyces_thermocarboxydus_BPSAC147	Streptomyces_sp._4F	Streptomyces_sp._BSE7F	Streptomyces_sp._ETH9427	Streptomyces_sp._FxanaD5	Streptomyces_sp._UNC401CLCol	Actinospica_acidiphila_NRRL_B_24431	Streptomyces_sp._HNS054	Streptomyces_sp._SMS_SU21	Streptomyces_sp._ZS0098	Streptomyces_thermocarboxydus_K155	Addition	Core-Accessory-Unique	COG	%Similarity_COG	Score_COG	Evalue_COG	KEGG	%Similarity_KEGG	Score_KEGG	Evalue_KEGG
7789	WP_033274350.1	-	DNA starvation/stationary phase protection protein	1	1	1	1	1	1	1	1	1	1	1	11	CORE	COG0783_P#	53.1	169	8.79E-42	K04047	34.4	83	2.89E-15
7790	WP_078895023.1	-	G/U mismatch-specific DNA glycosylase	1	1	1	1	1	1	1	1	1	1	1	11	CORE	COG3663_L#	48.1	158	3.46E-38	K03649	48.1	158	9.23E-38
7791	WP_157842180.1	-	hypothetical protein	0	0	0	0	1	0	1	0	0	0	0	2	Accessory								
7792	OSC62682.1	-	TetR family transcriptional regulator	0	1	0	0	0	0	0	0	0	0	0	1	Unique	COG1309_K#	31.2	81	3.15E-15	NA	25.4	53	0.0000032
7793	WP_024882439.1	-	Tellurium resistance protein terZ	1	1	1	1	1	1	1	1	1	1	1	11	CORE	COG2310_T#	50.5	192	1.27E-48	K05791	50.5	192	3.38E-48
7794	Prokka:CIODPCDJ_02389	-	hypothetical protein	1	1	1	1	1	1	1	1	1	1	1	11	CORE								
7795	WP_028961210.1	ruvC	crossover junction endodeoxyribonuclease RuvC	1	1	1	0	1	1	1	1	1	1	1	10	Accessory	COG0817_L#	61.8	220	4.34E-57	K01159	61.8	220	1.16E-56
7796	AXI87174.1	-	hypothetical protein	0	0	0	1	0	0	0	1	0	0	0	2	Accessory								
7797	OSC58753.1	-	TetR family transcriptional regulator	0	1	0	0	0	0	0	0	0	0	0	1	Unique	COG1309_K#	32.8	101	5.01E-21	K16137	32.5	102	4.6E-21

cluster	GI	gene	Annotation	Streptomyces_thermocarboxydus_BPSAC147	Streptomyces_sp._4F	Streptomyces_sp._BSE7F	Streptomyces_sp._ETH9427	Streptomyces_sp._FxanaD5	Streptomyces_sp._UNC401CLCol	Actinospica_acidiphila_NRRL_B_24431	Streptomyces_sp._HNS054	Streptomyces_sp._SMS_SU21	Streptomyces_sp._ZS0098	Streptomyces_thermocarboxydus_K155	Addition	Core-Accessory-Unique	COG	%Similarity_COG	Score_COG	Evalue_COG	KEGG	%Similarity_KEGG	Score_KEGG	Evalue_KEGG
7798	Prokka:CIODPCDJ_00923	-	hypothetical protein	1	1	1	1	1	1	1	0	1	1	1	10	Accessory	COG0515_RTKL#	35.3	66	1.37E-10	NA	32	59	4.47E-08
7799	WP_048458634.1	-	sigma-70 family RNA polymerase sigma factor	0	0	1	1	0	0	0	1	0	1	0	4	Accessory	COG1595_K#	34.2	103	7.73E-22	K03088	34.2	103	2.07E-21
7800	PWE06618.1	-	hypothetical protein	0	0	1	0	0	0	0	0	0	0	0	1	Unique								
7801	WP_078895176.1	-	lamin tail domain-containing protein	0	0	0	0	0	0	0	1	0	0	0	1	Unique								
7802	Prokka:JAPCLKMA_05470	-	hypothetical protein	1	0	0	0	1	1	0	0	0	0	1	4	Accessory	COG0454_KR#	44.7	112	1.66E-24	NA	44.7	112	4.45E-24
7803	OSC62150.1	-	deoxyuridine 5'-triphosphate nucleotidohydrolase	1	1	1	1	1	1	1	1	1	1	1	11	CORE	COG0756_F#	55.8	164	4.82E-40	K01520	55.7	161	8.35E-39
7804	WP_146217776.1	-	hypothetical protein	0	0	0	0	0	0	0	0	1	0	0	1	Unique								
7805	OSC68511.1	-	deaminase	1	1	1	1	1	1	1	1	1	1	1	11	CORE	COG0262_H#	31.5	70	5.56E-12				
7806	WP_048459028.1	-	TerD family protein	1	1	1	1	1	1	1	1	1	1	1	11	CORE	COG2310_T#	64.9	267	5.25E-71	K05795	63.9	266	2.39E-70
7807	Prokka:JAPCLKMA_03139	-	hypothetical protein	1	0	0	0	1	1	0	0	1	0	1	5	Accessory								
7808	AXI89892.1	-	histidine kinase	1	1	1	1	1	1	1	1	1	1	1	11	CORE	COG2203_T#	30.4	75	2.93E-13	NA	26.9	64	1.8E-09
7809	Prokka:JAPCLKMA_01633	-	hypothetical protein	1	0	0	1	0	0	0	0	0	0	0	2	Accessory								

cluster	GI	gene	Annotation	Streptomyces_thermocarboxydus_BPSAC147	Streptomyces_sp._4F	Streptomyces_sp._BSE7F	Streptomyces_sp._ETH9427	Streptomyces_sp._FxanaD5	Streptomyces_sp._UNC401CLCol	Actinospica_acidiphila_NRRL_B_24431	Streptomyces_sp._HNS054	Streptomyces_sp._SMS_SU21	Streptomyces_sp._ZS0098	Streptomyces_thermocarboxydus_K155	Addition	Core-Accessory-Unique	COG	%Similarity_COG	Score_COG	Evalue_COG	KEGG	%Similarity_KEGG	Score_KEGG	Evalue_KEGG
7810	WP_019524841.1	-	dTDP-4-dehydrorhamnose 3,5-epimerase family protein	0	0	0	0	1	1	0	0	0	0	1	3	Accessory	COG1898_M#	44.2	155	2.23E-37	K01790	44.2	155	5.95E-37
7811	WP_122212901.1	-	GNAT family N-acetyltransferase	1	1	1	1	1	1	1	1	1	1	1	11	CORE	COG0454_KR#	48	152	1.44E-36				
7812	WP_106432548.1	-	ATP-binding protein	1	0	0	1	1	1	1	1	1	1	1	9	Accessory								
7813	Prokka:JAPCLKMA_03752	dcd	dCTP deaminase, dUMP-forming	1	1	1	1	1	1	1	1	1	1	1	11	CORE	COG0717_F#	74.6	296	8.09E-80	K01494	74.6	296	2.16E-79
7814	WP_026243913.1	-	hypothetical protein	0	1	0	0	1	0	0	0	0	0	1	3	Accessory								
7815	WP_122216595.1	-	CGNR zinc finger domain-containing protein	0	0	1	1	0	0	0	1	1	1	0	5	Accessory								
7816	WP_122215825.1	-	hypothetical protein	0	0	0	0	0	0	0	0	0	1	0	1	Unique	COG3464_L#	38.2	57	4.84E-08				
7817	PWE11322.1	-	hypothetical protein	0	0	1	0	0	0	1	0	0	0	0	2	Accessory								
7818	Prokka:JAPCLKMA_05893	-	hypothetical protein	1	1	1	1	1	1	1	1	1	1	1	11	CORE								
7819	OSC64654.1	-	TetR family transcriptional regulator	0	1	0	0	0	0	0	0	0	0	0	1	Unique	COG1309_K#	29.3	52	0.00000156	NA	25.6	43	0.0033
7820	OSC60087.1	-	hypothetical protein	0	1	0	0	0	0	0	0	0	0	0	1	Unique								

cluster	GI	gene	Annotation	Streptomyces_thermocarboxydus_BPSAC147	Streptomyces_sp._4F	Streptomyces_sp._BSE7F	Streptomyces_sp._ETH9427	Streptomyces_sp._FxanaD5	Streptomyces_sp._UNC401CLCol	Actinospica_acidiphila_NRRL_B_24431	Streptomyces_sp._HNS054	Streptomyces_sp._SMS_SU21	Streptomyces_sp._ZS0098	Streptomyces_thermocarboxydus_K155	Addition	Core-Accessory-Unique	COG	%Similarity_COG	Score_COG	Evalue_COG	KEGG	%Similarity_KEGG	Score_KEGG	Evalue_KEGG
7821	Prokka:JAPCLKMA_02191	betI_6	HTH-type transcriptional regulator BetI	1	0	1	1	1	1	1	1	1	1	1	10	Accessory	COG1309_K#	29.9	80	6.98E-15	NA	29.9	80	1.86E-14
7822	WP_019524011.1	-	FABP family protein	1	1	1	1	1	1	1	1	1	1	1	11	CORE					NA	37.3	105	7.06E-22
7823	Prokka:JAPCLKMA_05690	-	hypothetical protein	1	0	1	1	1	1	0	1	1	1	1	9	Accessory								
7824	WP_102637525.1	-	TetR/AcrR family transcriptional regulator	0	1	0	0	0	0	0	1	1	0	0	3	Accessory	COG1309_K#	31	70	5.53E-12	NA	31	70	1.48E-11
7825	Prokka:JAPCLKMA_05630	-	hypothetical protein	1	0	0	0	0	0	0	0	0	0	0	1	Unique								
7826	OSC70571.1	-	hypothetical protein	0	1	0	0	0	0	0	0	0	0	0	1	Unique								
7827	OSC70164.1	-	tyrosinase	0	1	0	0	0	0	0	0	0	0	0	1	Unique								
7828	Prokka:JAPCLKMA_02783	-	hypothetical protein	1	0	0	0	0	0	0	0	0	0	0	1	Unique								
7829	Prokka:JAPCLKMA_04918	-	hypothetical protein	1	0	0	0	0	0	0	0	0	0	0	1	Unique								
7830	OSC65567.1	-	hypothetical protein	0	1	0	0	0	0	0	0	0	0	0	1	Unique	COG0513_LKJ#	35.5	46	0.000112	NA	43.6	59	4.44E-08
7831	WP_033275367.1	-	TetR/AcrR family transcriptional regulator	0	0	0	1	0	0	1	1	0	0	0	3	Accessory	COG1309_K#	44.8	160	5.3E-39	NA	36	102	5.98E-21
7832	PWE06620.1	-	hypothetical protein	0	0	1	0	0	0	0	0	0	0	0	1	Unique								

cluster	GI	gene	Annotation	Streptomyces_thermocarboxydus_BPSAC147	Streptomyces_sp._4F	Streptomyces_sp._BSE7F	Streptomyces_sp._ETH9427	Streptomyces_sp._FxanaD5	Streptomyces_sp._UNC401CLCol	Actinospica_acidiphila_NRRL_B_24431	Streptomyces_sp._HNS054	Streptomyces_sp._SMS_SU21	Streptomyces_sp._ZS0098	Streptomyces_thermocarboxydus_K155	Addition	Core-Accessory-Unique	COG	%Similarity_COG	Score_COG	Evalue_COG	KEGG	%Similarity_KEGG	Score_KEGG	Evalue_KEGG
7833	Prokka:JAPCLKMA_02081	arsC_1	Arsenate reductase	1	1	1	1	1	1	1	1	1	1	1	11	CORE	COG0394_T#	68	178	1.88E-44	K03325	68	178	5.03E-44
7834	WP_048454772.1	-	GNAT family N-acetyltransferase	1	0	1	1	1	1	1	1	1	1	1	10	Accessory								
7835	WP_122216001.1	-	hypothetical protein	0	0	0	0	0	0	0	0	0	1	0	1	Unique								
7836	WP_033278186.1	-	DUF742 domain-containing protein	1	1	1	1	1	1	1	1	1	1	1	11	CORE					NA	39.5	71	1.12E-11
7837	Prokka:JAPCLKMA_01212	-	hypothetical protein	1	1	1	1	1	1	1	1	1	1	1	11	CORE	COG0454_KR#	32.3	74	6.49E-13				
7838	Prokka:JAPCLKMA_01810	-	hypothetical protein	1	0	0	0	0	1	1	0	0	0	1	4	Accessory								
7839	Prokka:JAPCLKMA_06415	-	hypothetical protein	1	0	1	1	0	0	1	1	1	1	0	7	Accessory								
7840	WP_102639062.1	-	cob(I)yrinic acid a,c-diamide adenosyltransferase	1	1	1	1	1	1	1	1	1	1	1	11	CORE	COG2096_S#	68.3	254	2.68E-67	K00798	68.3	254	7.17E-67
7841	WP_158688235.1	-	hypothetical protein	0	0	0	0	1	0	0	0	0	0	0	1	Unique								
7842	OSC69218.1	-	hypothetical protein	1	1	0	1	1	1	1	1	1	1	1	10	Accessory	COG5512_R#	41.3	122	1.59E-27	NA	41.3	122	4.25E-27
7843	WP_019521599.1	-	XRE family transcriptional regulator	1	1	1	1	1	1	1	1	1	1	1	11	CORE	COG1396_K#	24.6	68	2.73E-11	NA	38.1	48	0.000078
7844	Prokka:CIODPCDJ_02890	ytlP	RNA 2',3'-cyclic phosphodiesterase	1	1	1	1	1	1	1	1	1	1	1	11	CORE	COG1514_J#	40.1	107	4.06E-23				

cluster	GI	gene	Annotation	Streptomyces_thermocarboxydus_BPSAC147	Streptomyces_sp._4F	Streptomyces_sp._BSE7F	Streptomyces_sp._ETH9427	Streptomyces_sp._FxanaD5	Streptomyces_sp._UNC401CLCol	Actinospica_acidiphila_NRRL_B_24431	Streptomyces_sp._HNS054	Streptomyces_sp._SMS_SU21	Streptomyces_sp._ZS0098	Streptomyces_thermocarboxydus_K155	Addition	Core-Accessory-Unique	COG	%Similarity_COG	Score_COG	Evalue_COG	KEGG	%Similarity_KEGG	Score_KEGG	Evalue_KEGG
7845	WP_102639332.1	-	helix-turn-helix domain-containing protein	1	1	1	1	1	1	1	1	1	1	1	11	CORE	COG1396_K#	39.1	146	7.88E-35	NA	29.4	87	1.16E-16
7846	OSC46523.1	-	ammonia channel protein	0	1	0	0	0	0	0	0	0	0	0	1	Unique	COG0004_P#	48.6	188	3.09E-47	K03320	48.6	188	8.24E-47
7847	OSC73526.1	-	RNA polymerase subunit sigma-24	0	1	0	0	0	0	0	0	0	0	0	1	Unique	COG1595_K#	31	78	2.64E-14	K03088	31.2	80	2.42E-14
7848	OSC67244.1	-	hypothetical protein	1	1	1	1	1	1	1	1	1	1	1	11	CORE	COG2246_S#	29.4	75	1.71E-13	NA	33.1	74	1.02E-12
7849	WP_107080983.1	-	HXXEE domain-containing protein	1	0	1	1	1	1	1	1	1	1	1	10	Accessory								
7850	OSC73275.1	-	hypothetical protein	0	1	0	0	0	0	0	0	0	0	0	1	Unique								
7851	WP_122213204.1	-	hypothetical protein	1	0	0	0	1	1	1	0	1	1	1	7	Accessory								
7852	WP_033274663.1	-	CGNR zinc finger domain-containing protein	0	0	0	0	0	0	1	0	0	0	0	1	Unique	COG5516_R#	32.8	76	1.31E-13				
7853	OSC57636.1	-	amidase	0	1	0	0	0	0	0	0	0	0	0	1	Unique	COG0154_J#	36.8	100	6.48E-21	K01426	36.8	100	1.73E-20
7854	WP_084206942.1	-	SMI1/KNR4 family protein	0	0	0	0	0	0	1	0	0	0	0	1	Unique								
7855	OSC71167.1	-	peptide deformylase	1	1	1	1	1	1	1	1	1	1	1	11	CORE	COG0242_J#	48.3	148	2.07E-35	K01462	45.3	132	3.15E-30

cluster	GI	gene	Annotation	Streptomyces_thermocarboxydus_BPSAC147	Streptomyces_sp._4F	Streptomyces_sp._BSE7F	Streptomyces_sp._ETH9427	Streptomyces_sp._FxanaD5	Streptomyces_sp._UNC401CLCol	Actinospica_acidiphila_NRRL_B_24431	Streptomyces_sp._HNS054	Streptomyces_sp._SMS_SU21	Streptomyces_sp._ZS0098	Streptomyces_thermocarboxydus_K155	Addition	Core-Accessory-Unique	COG	%Similarity_COG	Score_COG	Evalue_COG	KEGG	%Similarity_KEGG	Score_KEGG	Evalue_KEGG
7856	OSC71193.1	-	TetR family transcriptional regulator	0	1	0	0	0	0	0	0	0	0	0	1	Unique	COG1309_K#	30.7	72	2.47E-12	NA	25	54	0.00000109
7857	WP_033272123.1	-	nuclear transport factor 2 family protein	0	0	1	0	0	0	1	0	1	0	0	3	Accessory								
7858	AXI84752.1	-	hypothetical protein	0	0	1	1	0	0	1	1	1	1	0	6	Accessory								
7859	WP_019523922.1	-	hypothetical protein	1	1	1	1	1	1	1	1	1	1	1	11	CORE								
7860	WP_102637788.1	-	hypothetical protein	1	1	1	1	1	1	1	1	1	1	1	11	CORE								
7861	WP_019523378.1	cysC	adenylyl-sulfate kinase	1	1	1	1	1	1	1	1	1	1	1	11	CORE	COG0529_P#	50.9	174	2.69E-43	K00860	50.9	174	7.18E-43
7862	WP_102637294.1	-	NADH-quinone oxidoreductase subunit J	0	0	0	1	0	0	0	1	1	1	0	4	Accessory								
7863	AXI87027.1	-	GNAT family N-acetyltransferase	1	1	1	1	1	1	1	1	1	1	1	11	CORE	COG1670_J#	25.3	72	2.45E-12	K03817	33	69	3.25E-11
7864	OSC61493.1	-	ArsR family transcriptional regulator	0	1	0	0	0	0	0	0	0	0	0	1	Unique								
7865	OSC67189.1	-	hypothetical protein	1	1	1	1	1	1	1	1	1	1	1	11	CORE								

cluster	GI	gene	Annotation	Streptomyces_thermocarboxydus_BPSAC147	Streptomyces_sp._4F	Streptomyces_sp._BSE7F	Streptomyces_sp._ETH9427	Streptomyces_sp._FxanaD5	Streptomyces_sp._UNC401CLCol	Actinospica_acidiphila_NRRL_B_24431	Streptomyces_sp._HNS054	Streptomyces_sp._SMS_SU21	Streptomyces_sp._ZS0098	Streptomyces_thermocarboxydus_K155	Addition	Core-Accessory-Unique	COG	%Similarity_COG	Score_COG	Evalue_COG	KEGG	%Similarity_KEGG	Score_KEGG	Evalue_KEGG
7866	AXI90366.1	-	PadR family transcriptional regulator	1	0	1	1	1	1	1	1	1	1	1	10	Accessory	COG1695_K#	31.7	92	2.29E-18	NA	31.4	91	8E-18
7867	WP_122214779.1	-	hypothetical protein	0	0	0	0	0	0	1	1	0	1	0	3	Accessory	COG5516_R#	33	64	3.92E-10				
7868	PWE11493.1	-	hypothetical protein	0	1	1	1	0	0	0	1	1	1	0	6	Accessory								
7869	OSC55515.1	-	hypothetical protein	0	1	0	0	0	0	0	0	0	0	0	1	Unique								
7870	OSC65315.1	-	hypothetical protein	0	1	0	0	0	0	0	0	0	0	0	1	Unique	COG3193_R#	37.5	96	9.31E-20				
7871	OSC56425.1	-	peptidase	1	1	1	1	1	1	1	1	1	1	1	11	CORE								
7872	OSC69603.1	-	OHCU decarboxylase	1	1	1	0	1	1	1	1	1	1	1	10	Accessory	COG3195_S#	30.5	71	3.21E-12	K13484	33.1	87	1.51E-16
7873	Prokka:JAPCLKMA_00911	-	hypothetical protein	1	1	1	1	1	1	1	1	1	1	1	11	CORE								
7874	AXI91339.1	-	hypothetical protein	0	0	0	1	0	0	0	0	0	0	0	1	Unique					NA	29.8	53	0.00000241
7875	AXI91251.1	-	guanylate kinase	0	0	0	1	0	0	0	0	0	0	0	1	Unique								
7876	PWE10854.1	-	hypothetical protein	0	0	1	0	0	0	0	0	1	1	0	3	Accessory								
7877	OSC57281.1	-	hypothetical protein	1	1	1	1	1	1	1	1	1	1	1	11	CORE	COG3162_S#	38	72	1.44E-12				
7878	OSC56271.1	-	chloramphenicol phosphotransferase	1	1	1	1	1	1	1	0	1	1	1	10	Accessory	COG3896_V#	39.2	107	4.04E-23				
7879	AXI91401.1	-	hypothetical protein	0	0	0	1	0	0	0	0	0	0	0	1	Unique								
7880	OSC72104.1	-	hypothetical protein	0	1	0	0	0	0	0	0	0	0	0	1	Unique								
7881	WP_048456488.1	-	hypothetical protein	0	1	1	1	0	0	0	1	1	1	0	6	Accessory								

cluster	GI	gene	Annotation	Streptomyces_thermocarboxydus_BPSAC147	Streptomyces_sp._4F	Streptomyces_sp._BSE7F	Streptomyces_sp._ETH9427	Streptomyces_sp._FxanaD5	Streptomyces_sp._UNC401CLCol	Actinospica_acidiphila_NRRL_B_24431	Streptomyces_sp._HNS054	Streptomyces_sp._SMS_SU21	Streptomyces_sp._ZS0098	Streptomyces_thermocarboxydus_K155	Addition	Core-Accessory-Unique	COG	%Similarity_COG	Score_COG	Evalue_COG	KEGG	%Similarity_KEGG	Score_KEGG	Evalue_KEGG
7882	Prokka:JAPCLKMA_06600	-	hypothetical protein	1	1	1	1	1	1	1	1	1	1	1	11	CORE	COG1846_K#	27.5	61	4.33E-09	NA	27.5	61	1.16E-08
7883	PWE08991.1	-	hypothetical protein	0	0	1	0	0	0	0	0	0	0	0	1	Unique								
7884	OSC52281.1	-	hypothetical protein	0	1	0	0	0	0	0	0	0	0	0	1	Unique	COG4264_Q#	65.4	114	5.63E-25	K15654	27.6	49	0.0000594
7885	WP_102638862.1	-	helix-turn-helix transcriptional regulator	1	0	1	1	1	1	0	1	1	1	1	9	Accessory	COG1396_K#	54.5	192	1.24E-48	NA	30.9	89	3.95E-17
7886	WP_019522206.1	-	(2Fe-2S)-binding protein	1	0	0	0	1	1	0	0	0	0	1	4	Accessory	COG2080_C#	48.7	157	4.42E-38	K03518	44.8	151	6.48E-36
7887	WP_122215881.1	-	NifU family protein	1	0	0	1	1	1	0	1	0	1	1	7	Accessory								
7888	WP_122216342.1	-	NAD(P)H-dependent oxidoreductase	1	0	1	1	1	1	1	1	1	1	1	10	Accessory	COG0431_R#	53.9	194	3.26E-49	NA	53.9	194	8.71E-49
7889	PWE06570.1	efp	elongation factor P	1	1	1	1	1	1	1	1	1	1	1	11	CORE	COG0231_J#	66.5	255	2.03E-67	K02356	66.5	255	5.43E-67
7890	WP_028959283.1	-	PIN domain-containing protein	1	0	0	0	0	1	0	0	0	0	1	3	Accessory	COG1569_R#	28.6	40	0.00603				
7891	AXI89041.1	-	GNAT family N-acetyltransferase	1	1	1	1	1	1	1	1	1	1	1	11	CORE	COG0456_R#	33.8	80	5.26E-15	NA	35	76	2.65E-13
7892	WP_048456809.1	-	hypothetical protein	1	1	0	1	1	1	1	1	1	1	1	10	Accessory								
7893	WP_102637163.1	-	GNAT family N-acetyltransferase	0	0	1	0	0	0	1	0	1	0	0	3	Accessory								

cluster	GI	gene	Annotation	Streptomyces_thermocarboxydus_BPSAC147	Streptomyces_sp._4F	Streptomyces_sp._BSE7F	Streptomyces_sp._ETH9427	Streptomyces_sp._FxanaD5	Streptomyces_sp._UNC401CLCol	Actinospica_acidiphila_NRRL_B_24431	Streptomyces_sp._HNS054	Streptomyces_sp._SMS_SU21	Streptomyces_sp._ZS0098	Streptomyces_thermocarboxydus_K155	Addition	Core-Accessory-Unique	COG	%Similarity_COG	Score_COG	Evalue_COG	KEGG	%Similarity_KEGG	Score_KEGG	Evalue_KEGG
7894	WP_102641100.1	-	GNAT family N-acetyltransferase	1	1	1	1	1	1	1	0	1	1	1	10	Accessory	COG0454_KR#	39.4	104	4.44E-22	NA	34.5	91	1.04E-17
7895	WP_157842169.1	-	hypothetical protein	0	0	0	0	0	0	1	0	0	0	0	1	Unique								
7896	AXI91414.1	-	hypothetical protein	0	0	0	1	0	0	0	0	0	0	0	1	Unique								
7897	AXI87806.1	-	hypothetical protein	0	1	1	1	0	0	0	1	1	0	0	5	Accessory								
7898	OSC52815.1	-	resuscitation-promoting factor	0	1	0	0	0	0	0	0	0	0	0	1	Unique								
7899	PWE10644.1	-	type 1 glutamine amidotransferase	1	1	1	1	1	1	1	1	1	1	1	11	CORE	COG0693_R#	51.4	197	3.85E-50	K05520	51.4	197	1.03E-49
7900	WP_019525426.1	-	hypothetical protein	0	0	0	0	1	0	0	0	0	0	0	1	Unique								
7901	AXI85821.1	-	arginine repressor	1	1	1	1	1	1	1	1	1	1	1	11	CORE	COG1438_K#	53.8	163	8.05E-40	K03402	56.9	162	4.79E-39
7902	PWE08564.1	-	hypothetical protein	0	0	1	0	0	0	0	0	0	0	0	1	Unique								
7903	Prokka:CIODPCDJ_06373	-	hypothetical protein	1	1	1	1	1	1	1	1	1	1	1	11	CORE								
7904	Prokka:JAPCLKMA_02735	-	hypothetical protein	1	0	0	0	0	0	0	0	0	0	0	1	Unique								
7905	Prokka:JAPCLKMA_01614	cynT_2	Carbonic anhydrase 1	1	0	0	0	0	0	0	0	0	0	0	1	Unique	COG0288_P#	36.5	110	8.09E-24	K01673	35.4	107	1.4E-22

cluster	GI	gene	Annotation	Streptomyces_thermocarboxydus_BPSAC147	Streptomyces_sp._4F	Streptomyces_sp._BSE7F	Streptomyces_sp._ETH9427	Streptomyces_sp._FxanaD5	Streptomyces_sp._UNC401CLCol	Actinospica_acidiphila_NRRL_B_24431	Streptomyces_sp._HNS054	Streptomyces_sp._SMS_SU21	Streptomyces_sp._ZS0098	Streptomyces_thermocarboxydus_K155	Addition	Core-Accessory-Unique	COG	%Similarity_COG	Score_COG	Evalue_COG	KEGG	%Similarity_KEGG	Score_KEGG	Evalue_KEGG
7906	Prokka:CIODPCDJ_00867	tgnA	Flavin-dependent trigonelline monooxygenase, reductase component	0	0	0	0	1	1	0	0	0	0	1	3	Accessory	COG1853_R#	39.9	122	1.21E-27	K16048	34.1	101	7.69E-21
7907	OSC72173.1	-	hypothetical protein	1	1	1	1	1	1	1	1	1	1	1	11	CORE	COG4762_S#	43.1	121	4.58E-27	NA	43.1	121	1.22E-26
7908	OSC62718.1	-	GNAT family N-acetyltransferase	1	1	1	1	1	1	1	1	1	1	1	11	CORE	COG1670_J#	29.9	80	8.97E-15				
7909	OSC65615.1	-	hypothetical protein	1	1	1	1	1	1	1	1	1	1	1	11	CORE					NA	33.8	93	2.09E-18
7910	OSC62971.1	-	competence protein F phosphoribosyltransferase	0	1	0	0	0	0	0	0	0	0	0	1	Unique	COG1040_R#	41.7	72	1.87E-12				
7911	AXI89622.1	-	WhiB family transcriptional regulator	0	0	0	1	0	0	0	0	0	0	0	1	Unique					K18958	46.8	47	0.000225
7912	OSC63775.1	-	SigE family RNA polymerase sigma factor	0	1	0	0	0	0	0	0	0	0	0	1	Unique	COG1595_K#	30.5	65	2.98E-10	K03088	27.7	48	0.000101
7913	WP_048458924.1	-	GNAT family N-acetyltransferase	1	1	1	1	1	1	1	1	1	1	1	11	CORE	COG0454_KR#	27.9	58	3.63E-08	NA	31.2	51	0.00000907

cluster	GI	gene	Annotation	Streptomyces_thermocarboxydus_BPSAC147	Streptomyces_sp._4F	Streptomyces_sp._BSE7F	Streptomyces_sp._ETH9427	Streptomyces_sp._FxanaD5	Streptomyces_sp._UNC401CLCol	Actinospica_acidiphila_NRRL_B_24431	Streptomyces_sp._HNS054	Streptomyces_sp._SMS_SU21	Streptomyces_sp._ZS0098	Streptomyces_thermocarboxydus_K155	Addition	Core-Accessory-Unique	COG	%Similarity_COG	Score_COG	Evalue_COG	KEGG	%Similarity_KEGG	Score_KEGG	Evalue_KEGG
7914	WP_048460676.1	-	hypothetical protein	1	0	1	1	1	1	1	1	1	1	1	10	Accessory								
7915	Prokka:JAPCLKMA_00449	sigE_1	RNA polymerase sigma-E factor	1	1	1	1	1	1	1	1	1	1	1	11	CORE	COG1595_K#	31.6	69	1.57E-11	NA	26.1	69	3.22E-11
7916	Prokka:CIODPCDJ_06810	-	hypothetical protein	0	0	0	0	0	0	0	0	0	0	1	1	Unique								
7917	WP_028961751.1	rimM	ribosome maturation factor RimM	1	1	1	1	1	1	1	1	1	1	1	11	CORE	COG0806_J#	46.3	140	5.56E-33	K02860	46.3	140	1.48E-32
7918	WP_122212770.1	-	phage tail protein	0	0	0	1	0	0	0	1	1	1	0	4	Accessory								
7919	Prokka:JAPCLKMA_02298	-	hypothetical protein	1	0	0	0	0	0	1	0	0	1	1	4	Accessory					NA	49.2	47	0.000131
7920	WP_102641217.1	-	deaminase	1	1	0	1	1	1	1	1	1	1	1	10	Accessory	COG0262_H#	43.3	139	1.62E-32	NA	27.1	90	2.3E-17
7921	PWE10329.1	-	hypothetical protein	0	0	1	0	0	0	1	0	0	0	0	2	Accessory								
7922	WP_051101670.1	-	GNAT family N-acetyltransferase	0	1	1	1	1	1	1	1	1	1	1	10	Accessory								
7923	PWE11001.1	-	GNAT family N-acetyltransferase	0	0	1	1	0	0	0	1	0	1	0	4	Accessory								
7924	AXI91341.1	-	hypothetical protein	0	0	0	1	0	0	0	0	0	0	0	1	Unique								
7925	AXI86454.1	-	GMP synthase	0	0	0	1	0	0	0	1	0	0	0	2	Accessory	COG0518_F#	27.3	79	1.52E-14	K01951	27.3	79	4.06E-14
7926	OSC67443.1	-	peptidase	1	1	1	1	1	1	1	1	1	1	1	11	CORE								
7927	WP_033276931.1	-	hypothetical protein	1	1	1	1	1	1	1	1	1	1	1	11	CORE	COG2606_S#	43.8	113	9.51E-25				

cluster	GI	gene	Annotation	Streptomyces_thermocarboxydus_BPSAC147	Streptomyces_sp._4F	Streptomyces_sp._BSE7F	Streptomyces_sp._ETH9427	Streptomyces_sp._FxanaD5	Streptomyces_sp._UNC401CLCol	Actinospica_acidiphila_NRRL_B_24431	Streptomyces_sp._HNS054	Streptomyces_sp._SMS_SU21	Streptomyces_sp._ZS0098	Streptomyces_thermocarboxydus_K155	Addition	Core-Accessory-Unique	COG	%Similarity_COG	Score_COG	Evalue_COG	KEGG	%Similarity_KEGG	Score_KEGG	Evalue_KEGG
7928	Prokka:CIODPCDJ_01858	-	hypothetical protein	0	0	1	0	1	1	0	0	1	1	1	6	Accessory								
7929	PWE10332.1	-	ATP-binding protein	1	0	1	1	1	1	0	1	1	1	0	8	Accessory								
7930	Prokka:CIODPCDJ_00156	-	hypothetical protein	1	0	1	1	1	1	1	1	1	1	1	10	Accessory								
7931	OSC72838.1	-	hypothetical protein	0	1	0	0	0	0	0	0	0	0	0	1	Unique								
7932	AXI91408.1	-	hypothetical protein	0	0	0	1	0	0	0	0	0	0	0	1	Unique								
7933	Prokka:JAPCLKMA_06231	-	hypothetical protein	1	0	0	0	1	1	0	0	0	0	1	4	Accessory								
7934	OSC58245.1	-	hemerythrin	0	1	0	0	0	0	0	0	0	0	0	1	Unique								
7935	OSC56392.1	-	hypothetical protein	0	1	0	0	0	0	0	0	0	0	0	1	Unique								
7936	OSC57882.1	-	hypothetical protein	1	1	0	1	1	1	1	1	0	0	0	7	Accessory								
7937	OSC70302.1	-	hypothetical protein	0	1	0	0	0	0	0	0	0	0	0	1	Unique								
7938	OSC58270.1	-	hypothetical protein	0	1	0	0	0	0	0	0	0	0	0	1	Unique	COG3576_R#	44.4	151	3.15E-36	K07006	38	112	3.32E-24
7939	WP_028960328.1	-	PadR family transcriptional regulator	1	1	1	1	1	1	1	1	1	1	1	11	CORE	COG1695_K#	53.5	175	1.55E-43	NA	42.2	129	2.61E-29
7940	Prokka:JAPCLKMA_04679	-	hypothetical protein	1	1	1	1	1	1	1	1	1	1	1	11	CORE								
7941	WP_033274920.1	hpt	hypoxanthine phosphoribosyltransferase	1	1	1	1	1	1	1	1	1	1	1	11	CORE	COG0634_F#	59.9	221	3.22E-57	K00760	59.7	221	8.6E-57

cluster	GI	gene	Annotation	Streptomyces_thermocarboxydus_BPSAC147	Streptomyces_sp._4F	Streptomyces_sp._BSE7F	Streptomyces_sp._ETH9427	Streptomyces_sp._FxanaD5	Streptomyces_sp._UNC401CLCol	Actinospica_acidiphila_NRRL_B_24431	Streptomyces_sp._HNS054	Streptomyces_sp._SMS_SU21	Streptomyces_sp._ZS0098	Streptomyces_thermocarboxydus_K155	Addition	Core-Accessory-Unique	COG	%Similarity_COG	Score_COG	Evalue_COG	KEGG	%Similarity_KEGG	Score_KEGG	Evalue_KEGG
7942	WP_078634972.1	-	HDIG domain-containing protein	1	0	0	0	1	0	0	0	1	0	0	3	Accessory								
7943	WP_033277097.1	-	NAD(P)H-dependent oxidoreductase	0	1	0	0	0	0	1	0	0	1	0	3	Accessory	COG0431_R#	54.8	204	3.12E-52	NA	35.5	85	5.64E-16
7944	AXI89223.1	rfbC	dTDP-4-dehydrorhamnose 3,5-epimerase	1	0	1	1	1	1	1	1	1	1	1	10	Accessory	COG1898_M#	39.9	133	8.83E-31	K01790	38	126	2.88E-28
7945	WP_033275877.1	-	DUF4188 domain-containing protein	1	0	0	0	1	1	1	0	0	0	1	5	Accessory								
7946	WP_102639887.1	-	hypothetical protein	0	0	0	0	0	0	0	0	1	0	0	1	Unique								
7947	OSC71754.1	-	hypothetical protein	0	1	0	0	0	0	0	0	0	0	0	1	Unique								
7948	WP_019523796.1	-	CGNR zinc finger domain-containing protein	1	1	1	1	1	1	1	1	1	1	1	11	CORE	COG5516_R#	48.3	53	8.89E-07				
7949	PWE10531.1	-	DUF2243 domain-containing protein	0	0	1	1	0	0	0	1	1	0	0	4	Accessory	COG4329_S#	50	158	2.56E-38				
7950	OSC72598.1	-	hypothetical protein	0	1	0	0	0	0	0	0	0	0	0	1	Unique								
7951	OSC56581.1	-	Tat pathway signal sequence domain protein	0	1	0	0	0	0	0	0	0	0	0	1	Unique								

cluster	GI	gene	Annotation	Streptomyces_thermocarboxydus_BPSAC147	Streptomyces_sp._4F	Streptomyces_sp._BSE7F	Streptomyces_sp._ETH9427	Streptomyces_sp._FxanaD5	Streptomyces_sp._UNC401CLCol	Actinospica_acidiphila_NRRL_B_24431	Streptomyces_sp._HNS054	Streptomyces_sp._SMS_SU21	Streptomyces_sp._ZS0098	Streptomyces_thermocarboxydus_K155	Addition	Core-Accessory-Unique	COG	%Similarity_COG	Score_COG	Evalue_COG	KEGG	%Similarity_KEGG	Score_KEGG	Evalue_KEGG
7952	OSC59904.1	-	GDP-mannose pyrophosphatase	0	1	0	0	0	0	0	0	0	0	0	1	Unique	COG0494_LR#	55.4	179	1.07E-44	K12945	55.4	177	1.09E-43
7953	OSC73445.1	-	F0F1 ATP synthase subunit B	1	1	1	1	1	1	1	1	1	1	1	11	CORE	COG0711_C#	41.6	133	6.76E-31	K02109	30.9	79	5.28E-14
7954	OSC72096.1	-	quercetin 2,3-dioxygenase	0	1	0	0	0	0	0	0	0	0	0	1	Unique	COG1917_S#	32.9	87	7.26E-17	K07155	32.9	86	3.31E-16
7955	OSC57377.1	-	hypothetical protein	0	1	0	0	0	0	0	0	0	0	0	1	Unique	COG3321_Q#	48.1	170	6.52E-42	K12434	48.1	170	1.74E-41
7956	WP_078504581.1	-	RICIN domain-containing protein	0	0	0	0	1	0	0	0	0	0	0	1	Unique								
7957	Prokka:CIODPCDJ_02284	-	hypothetical protein	1	0	0	0	0	0	0	0	0	0	1	2	Accessory								
7958	WP_033277870.1	-	hypothetical protein	1	0	0	0	1	0	1	0	0	0	1	4	Accessory								
7959	OSC71610.1	-	toxin-antitoxin system, toxin component	1	1	1	0	1	1	1	1	1	1	1	10	Accessory								
7960	PWE09602.1	-	TetR/AcrR family transcriptional regulator	1	1	1	1	1	1	1	1	1	1	1	11	CORE	COG1309_K#	28.9	54	5.18E-07	NA	33.3	47	0.000169
7961	Prokka:CIODPCDJ_03987	rplE	50S ribosomal protein L5	1	1	1	1	1	1	1	1	1	1	1	11	CORE	COG0094_J#	77	286	6.21E-77	K02931	73.7	278	3.46E-74
7962	AXI90161.1	-	hypothetical protein	0	0	1	1	0	1	1	1	1	1	0	7	Accessory								

cluster	GI	gene	Annotation	Streptomyces_thermocarboxydus_BPSAC147	Streptomyces_sp._4F	Streptomyces_sp._BSE7F	Streptomyces_sp._ETH9427	Streptomyces_sp._FxanaD5	Streptomyces_sp._UNC401CLCol	Actinospica_acidiphila_NRRL_B_24431	Streptomyces_sp._HNS054	Streptomyces_sp._SMS_SU21	Streptomyces_sp._ZS0098	Streptomyces_thermocarboxydus_K155	Addition	Core-Accessory-Unique	COG	%Similarity_COG	Score_COG	Evalue_COG	KEGG	%Similarity_KEGG	Score_KEGG	Evalue_KEGG
7963	WP_048456946.1	-	DinB family protein	1	1	1	1	1	1	1	1	1	1	1	11	CORE								
7964	OSC62190.1	-	DNA-binding protein	0	1	1	1	0	0	1	1	0	0	0	5	Accessory								
7965	Prokka:JAPCLKMA_04960	rimI	N-alpha-acetyltransferase RimI	1	1	1	1	1	1	1	1	1	1	1	11	CORE	COG0456_R#	37.7	98	3.13E-20	K03789	33.8	82	6.2E-15
7966	WP_006136598.1	-	ribosome recycling factor	1	1	1	1	1	1	1	1	1	1	1	11	CORE	COG0233_J#	64.9	245	2.07E-64	K02838	63.8	243	1.23E-63
7967	WP_122216697.1	-	XRE family transcriptional regulator	0	0	0	0	1	0	0	0	0	1	0	2	Accessory								
7968	Prokka:JAPCLKMA_03941	nrdR	Transcriptional repressor NrdR	1	1	1	1	1	1	1	1	1	1	1	11	CORE	COG1327_K#	72.4	218	2.08E-56	K07738	72.4	218	5.54E-56
7969	AXI85299.1	-	hypothetical protein	0	0	0	1	0	0	0	0	0	0	0	1	Unique								
7970	OSC52949.1	-	LacI family transcriptional regulator	0	1	0	0	0	0	0	0	0	0	0	1	Unique	COG1609_K#	36.3	85	2.74E-16	K02529	29.8	69	5.43E-11
7971	WP_122215868.1	-	deaminase	0	0	0	0	0	0	0	0	0	1	0	1	Unique	COG0262_H#	40.1	138	2.73E-32				
7972	AXI90038.1	-	hypothetical protein	0	0	0	1	0	0	0	0	0	0	0	1	Unique								
7973	OSC72702.1	-	hypothetical protein	0	1	0	0	0	0	0	0	0	0	0	1	Unique								
7974	OSC61765.1	-	hypothetical protein	0	1	0	0	0	0	0	0	0	0	0	1	Unique	COG3786_S#	32.6	50	0.00000749				

cluster	GI	gene	Annotation	Streptomyces_thermocarboxydus_BPSAC147	Streptomyces_sp._4F	Streptomyces_sp._BSE7F	Streptomyces_sp._ETH9427	Streptomyces_sp._FxanaD5	Streptomyces_sp._UNC401CLCol	Actinospica_acidiphila_NRRL_B_24431	Streptomyces_sp._HNS054	Streptomyces_sp._SMS_SU21	Streptomyces_sp._ZS0098	Streptomyces_thermocarboxydus_K155	Addition	Core-Accessory-Unique	COG	%Similarity_COG	Score_COG	Evalue_COG	KEGG	%Similarity_KEGG	Score_KEGG	Evalue_KEGG
7975	WP_033272895.1	-	winged helix family transcriptional regulator	1	0	0	0	1	1	1	0	0	0	1	5	Accessory	COG0745_TK#	56.5	97	4.09E-20	NA	56.5	97	1.09E-19
7976	WP_033276035.1	-	glycoside hydrolase family 25 protein	1	1	1	1	1	1	1	1	1	1	1	11	CORE	COG3757_M#	31.8	75	2.17E-13	K07273	31.8	75	5.8E-13
7977	WP_048459689.1	-	hypothetical protein	0	0	0	0	0	0	0	1	0	0	0	1	Unique								
7978	OSC58913.1	-	TetR family transcriptional regulator	0	1	0	0	0	0	0	0	0	0	0	1	Unique	COG1309_K#	43.4	125	1.83E-28	NA	28.2	80	1.38E-14
7979	AXI86747.1	-	PadR family transcriptional regulator	0	1	0	1	0	0	1	0	1	0	0	4	Accessory	COG1695_K#	34.3	70	5.35E-12	NA	24.9	55	6.22E-07
7980	OSC70830.1	-	DNA repair protein	0	1	0	0	0	0	0	0	0	0	0	1	Unique								
7981	WP_033277624.1	-	putative glycolipid-binding domain-containing protein	0	0	0	0	0	0	1	0	0	0	0	1	Unique	COG3554_S#	33.9	87	4.23E-17				
7982	OSC70657.1	-	RNA polymerase subunit sigma-24	0	1	0	0	0	0	0	0	0	0	0	1	Unique	COG1595_K#	40.8	125	1.83E-28	K03088	32.6	83	2.13E-15
7983	Prokka:JAPCLKMA_04346	-	hypothetical protein	1	0	0	0	0	0	0	0	0	0	0	1	Unique								

cluster	GI	gene	Annotation	Streptomyces_thermocarboxydus_BPSAC147	Streptomyces_sp._4F	Streptomyces_sp._BSE7F	Streptomyces_sp._ETH9427	Streptomyces_sp._FxanaD5	Streptomyces_sp._UNC401CLCol	Actinospica_acidiphila_NRRL_B_24431	Streptomyces_sp._HNS054	Streptomyces_sp._SMS_SU21	Streptomyces_sp._ZS0098	Streptomyces_thermocarboxydus_K155	Addition	Core-Accessory-Unique	COG	%Similarity_COG	Score_COG	Evalue_COG	KEGG	%Similarity_KEGG	Score_KEGG	Evalue_KEGG
7984	OSC55874.1	-	FMN reductase (NADPH)	1	1	0	0	1	1	1	0	0	0	1	6	Accessory	COG0431_R#	53.8	169	1.11E-41	K00299	43.1	128	7.55E-29
7985	OSC58493.1	-	hypothetical protein	1	1	1	1	1	1	1	1	1	1	1	11	CORE								
7986	WP_106432559.1	-	sigma-70 family RNA polymerase sigma factor	0	0	0	0	1	0	0	0	0	0	0	1	Unique								
7987	AXI86179.1	-	cysteine methyltransferase	1	1	1	1	1	1	1	1	1	1	1	11	CORE	COG0350_L#	39.9	111	3.55E-24	K00567	39.6	109	2.76E-23
7988	OSC71827.1	-	alkyl hydroperoxide reductase	1	1	1	1	1	1	1	1	1	1	1	11	CORE	COG0450_O#	61.5	233	8.1E-61	K03386	61.5	233	2.16E-60
7989	WP_147474421.1	-	hypothetical protein	0	0	0	0	0	0	0	0	0	1	0	1	Unique								
7990	OSC65008.1	-	flavoprotein	1	1	1	1	1	1	1	1	1	1	1	11	CORE	COG0452_H#	31.4	66	1E-10	K13038	41.3	51	0.00000893
7991	WP_078493103.1	-	flavin reductase family protein	0	0	0	0	0	0	1	0	0	0	0	1	Unique	COG1853_R#	38.6	94	4.5E-19	NA	32.9	75	4.42E-13
7992	OSC63368.1	-	hypothetical protein	1	1	1	1	1	1	1	1	1	1	1	11	CORE								
7993	AXI85302.1	-	secondary metabolite protein	0	0	0	1	0	0	0	0	0	0	0	1	Unique								
7994	Prokka:JAPCLKMA_01078	nuoB	NADH-quinone oxidoreductase subunit B	1	1	1	1	1	1	1	1	1	1	1	11	CORE	COG0377_C#	78.8	299	9.2E-81	K00331	78.8	299	2.46E-80
7995	OSC57423.1	-	hypothetical protein	0	1	0	0	0	0	0	0	0	0	0	1	Unique								

cluster	GI	gene	Annotation	Streptomyces_thermocarboxydus_BPSAC147	Streptomyces_sp._4F	Streptomyces_sp._BSE7F	Streptomyces_sp._ETH9427	Streptomyces_sp._FxanaD5	Streptomyces_sp._UNC401CLCol	Actinospica_acidiphila_NRRL_B_24431	Streptomyces_sp._HNS054	Streptomyces_sp._SMS_SU21	Streptomyces_sp._ZS0098	Streptomyces_thermocarboxydus_K155	Addition	Core-Accessory-Unique	COG	%Similarity_COG	Score_COG	Evalue_COG	KEGG	%Similarity_KEGG	Score_KEGG	Evalue_KEGG
7996	WP_053077025.1	-	hypothetical protein	0	0	1	1	0	0	0	1	1	1	0	5	Accessory								
7997	Prokka:CIODPCDJ_02958	-	hypothetical protein	1	0	1	1	1	1	1	1	0	1	1	9	Accessory	COG0456_R#	30	40	0.00771				
7998	AXI87221.1	-	DUF2617 domain-containing protein	1	1	0	1	1	1	1	1	1	1	1	10	Accessory					NA	33.7	58	7.3E-08
7999	OSC61143.1	-	Tat pathway signal sequence domain protein	0	1	0	0	0	0	0	0	0	0	0	1	Unique	COG1409_R#	33.8	63	8.5E-10				
8000	OSC69404.1	-	hypothetical protein	1	1	1	1	1	1	1	1	1	1	1	11	CORE								
8001	OSC46771.1	-	hypothetical protein	0	1	0	0	0	0	0	0	0	0	0	1	Unique								
8002	WP_048456652.1	-	hypothetical protein	0	0	0	0	0	0	0	1	0	0	0	1	Unique								
8003	WP_102637598.1	-	ATP-binding protein	1	1	1	1	1	1	1	1	1	1	1	11	CORE								
8004	WP_019525445.1	-	hypothetical protein	0	0	0	0	1	0	0	0	0	0	1	2	Accessory								
8005	Prokka:JAPCLKMA_01514	-	hypothetical protein	1	0	0	1	0	0	0	0	0	0	0	2	Accessory								
8006	WP_033277796.1	-	hypothetical protein	1	0	1	1	1	1	1	1	1	1	1	10	Accessory								
8007	OSC68998.1	-	hypothetical protein	0	1	0	0	0	0	0	0	0	0	0	1	Unique								
8008	OSC63051.1	-	multidrug ABC transporter permease	0	1	0	0	0	0	0	0	0	0	0	1	Unique	COG0842_V#	40.4	151	3.1E-36				

cluster	GI	gene	Annotation	Streptomyces_thermocarboxydus_BPSAC147	Streptomyces_sp._4F	Streptomyces_sp._BSE7F	Streptomyces_sp._ETH9427	Streptomyces_sp._FxanaD5	Streptomyces_sp._UNC401CLCol	Actinospica_acidiphila_NRRL_B_24431	Streptomyces_sp._HNS054	Streptomyces_sp._SMS_SU21	Streptomyces_sp._ZS0098	Streptomyces_thermocarboxydus_K155	Addition	Core-Accessory-Unique	COG	%Similarity_COG	Score_COG	Evalue_COG	KEGG	%Similarity_KEGG	Score_KEGG	Evalue_KEGG
8009	OSC72043.1	-	acetyltransferase	1	1	1	1	1	1	1	1	1	1	1	11	CORE	COG1670_J#	40.4	140	5.47E-33				
8010	AXI91307.1	-	hypothetical protein	0	0	0	1	0	0	0	0	0	0	0	1	Unique								
8011	WP_019525857.1	-	SigE family RNA polymerase sigma factor	1	0	1	1	1	1	1	1	1	1	1	10	Accessory	COG1595_K#	27	62	1.44E-09	K03088	27	59	3.26E-08
8012	AXI87579.1	-	RNA polymerase subunit sigma-24	1	1	1	1	1	1	1	1	1	1	1	11	CORE	COG1595_K#	37.4	82	1.76E-15	K03088	37.9	84	1.24E-15
8013	WP_084207003.1	-	NUDIX hydrolase	1	1	1	1	1	1	1	1	1	1	1	11	CORE	COG0494_LR#	32.6	75	2.15E-13	K01515	28.7	62	5.03E-09
8014	WP_048455536.1	-	FHA domain-containing protein	1	1	1	1	1	1	1	1	1	1	1	11	CORE	COG1716_T#	40.5	53	8.75E-07	NA	37.1	52	0.00000399
8015	OSC71301.1	-	ribosome maturation factor RimP	1	1	1	1	1	1	1	1	1	1	1	11	CORE	COG0779_S#	37.7	85	2.08E-16	K09748	37.7	85	5.55E-16
8016	Prokka:CIODPCDJ_01600	-	hypothetical protein	1	1	1	1	1	1	1	1	1	1	1	11	CORE	COG5500_S#	43.7	131	2.53E-30				
8017	OSC68653.1	-	cysteine hydrolase	0	1	0	0	0	0	0	0	0	0	0	1	Unique	COG1335_Q#	41.2	92	2.22E-18	NA	30.8	66	3.48E-10
8018	Prokka:JAPCLKMA_06392	-	hypothetical protein	1	0	0	0	1	1	0	0	0	0	1	4	Accessory								
8019	OSC65074.1	-	Ku protein	0	1	0	0	0	0	0	0	0	0	0	1	Unique	COG1273_S#	33.5	89	1.88E-17	K10979	27.6	84	1.61E-15
8020	WP_078894973.1	-	hypothetical protein	0	0	0	0	0	0	0	1	0	0	0	1	Unique								
8021	PWE08520.1	-	Uma2 family endonuclease	0	0	1	0	0	0	0	0	0	0	0	1	Unique								

cluster	GI	gene	Annotation	Streptomyces_thermocarboxydus_BPSAC147	Streptomyces_sp._4F	Streptomyces_sp._BSE7F	Streptomyces_sp._ETH9427	Streptomyces_sp._FxanaD5	Streptomyces_sp._UNC401CLCol	Actinospica_acidiphila_NRRL_B_24431	Streptomyces_sp._HNS054	Streptomyces_sp._SMS_SU21	Streptomyces_sp._ZS0098	Streptomyces_thermocarboxydus_K155	Addition	Core-Accessory-Unique	COG	%Similarity_COG	Score_COG	Evalue_COG	KEGG	%Similarity_KEGG	Score_KEGG	Evalue_KEGG
8022	OSC72208.1	-	TmrB-like protein	0	1	0	0	0	0	0	0	0	0	0	1	Unique					NA	40.8	106	3.04E-22
8023	Prokka:JAPCLKMA_01330	-	hypothetical protein	1	0	0	0	0	0	0	0	0	0	1	2	Accessory								
8024	OSC57484.1	-	hypothetical protein	0	1	0	0	0	0	0	0	0	0	0	1	Unique								
8025	PWE08927.1	-	hypothetical protein	0	0	1	1	0	0	0	1	1	1	0	5	Accessory								
8026	WP_102637911.1	-	GGDEF domain-containing protein	1	1	0	0	1	0	0	0	1	0	0	4	Accessory	COG2199_T#	39.8	99	1.39E-20	NA	39.8	99	3.71E-20
8027	WP_026244199.1	-	membrane protein	0	0	0	0	1	1	0	0	0	0	0	2	Accessory	COG1971_S#	31.5	100	6.24E-21				
8028	WP_078504538.1	-	hypothetical protein	0	0	0	0	1	0	0	0	0	0	0	1	Unique	COG2931_Q#	34	45	0.000182	K13219	37.4	58	9.49E-08
8029	PWE06555.1	-	hypothetical protein	0	1	1	1	1	1	1	1	1	1	1	10	Accessory					NA	39.2	42	0.00412
8030	WP_033273217.1	paaJ	phenylacetate-CoA oxygenase subunit PaaJ	1	0	0	1	1	1	1	1	1	1	1	9	Accessory	COG2151_R#	51.2	160	6.64E-39	K02612	43.3	139	2.48E-32
8031	AXI91237.1	-	hypothetical protein	0	0	0	1	0	0	0	0	0	0	0	1	Unique								
8032	OSC71226.1	-	osmotically inducible protein OsmC	0	1	0	0	0	0	0	0	0	0	0	1	Unique								
8033	OSC72135.1	-	hypothetical protein	0	1	0	0	0	0	0	0	0	0	0	1	Unique								
8034	AXI91240.1	-	hypothetical protein	0	0	0	1	0	0	0	0	0	0	0	1	Unique								
8035	OSC71698.1	-	GNAT family N-acetyltransferase	0	1	0	0	0	0	0	0	0	0	0	1	Unique								

cluster	GI	gene	Annotation	Streptomyces_thermocarboxydus_BPSAC147	Streptomyces_sp._4F	Streptomyces_sp._BSE7F	Streptomyces_sp._ETH9427	Streptomyces_sp._FxanaD5	Streptomyces_sp._UNC401CLCol	Actinospica_acidiphila_NRRL_B_24431	Streptomyces_sp._HNS054	Streptomyces_sp._SMS_SU21	Streptomyces_sp._ZS0098	Streptomyces_thermocarboxydus_K155	Addition	Core-Accessory-Unique	COG	%Similarity_COG	Score_COG	Evalue_COG	KEGG	%Similarity_KEGG	Score_KEGG	Evalue_KEGG
8036	Prokka:JAPCLKMA_05641	-	hypothetical protein	1	0	0	1	1	1	1	0	0	1	1	7	Accessory								
8037	Prokka:JAPCLKMA_02634	-	hypothetical protein	1	0	0	0	0	0	0	0	0	0	0	1	Unique								
8038	Prokka:JAPCLKMA_04345	cheB	Protein-glutamate methylesterase/protein-glutamine glutaminase	1	1	1	1	1	1	1	1	1	1	1	11	CORE	COG0784_T#	41.4	98	3.1E-20	K10697	40.2	97	1.08E-19
8039	Prokka:JAPCLKMA_05144	-	hypothetical protein	1	0	0	1	1	0	0	0	0	1	0	4	Accessory								
8040	WP_048457050.1	-	orotate phosphoribosyltransferase	1	1	1	1	1	1	1	1	1	1	1	11	CORE	COG0461_F#	59.7	209	1.24E-53	K00762	59.6	204	1.06E-51
8041	AXI87441.1	-	RNA 2'-phosphotransferase	1	1	1	1	1	1	1	1	1	1	1	11	CORE	COG1859_J#	53.3	173	9.84E-43	K07559	46.7	148	6.93E-35
8042	WP_033273380.1	-	hypothetical protein	1	0	1	1	1	1	1	1	1	1	1	10	Accessory					NA	38.7	52	0.00000518
8043	Prokka:JAPCLKMA_05059	apt	Adenine phosphoribosyltransferase	1	1	1	1	1	1	1	1	1	1	1	11	CORE	COG0503_F#	51.2	174	2.59E-43	K00759	51.2	174	6.92E-43
8044	OSC71187.1	-	hypothetical protein	0	1	0	0	0	0	0	0	0	0	0	1	Unique								
8045	OSC65370.1	-	hypothetical protein	0	1	0	0	0	0	1	0	0	0	0	2	Accessory	COG4332_S#	33.1	85	2.07E-16	NA	29.4	85	7.21E-16
8046	WP_102641696.1	-	HIT domain-containing protein	1	1	1	1	1	1	1	1	1	1	1	11	CORE	COG0537_FGR#	55.8	202	1.16E-51	NA	55.8	202	3.1E-51

cluster	GI	gene	Annotation	Streptomyces_thermocarboxydus_BPSAC147	Streptomyces_sp._4F	Streptomyces_sp._BSE7F	Streptomyces_sp._ETH9427	Streptomyces_sp._FxanaD5	Streptomyces_sp._UNC401CLCol	Actinospica_acidiphila_NRRL_B_24431	Streptomyces_sp._HNS054	Streptomyces_sp._SMS_SU21	Streptomyces_sp._ZS0098	Streptomyces_thermocarboxydus_K155	Addition	Core-Accessory-Unique	COG	%Similarity_COG	Score_COG	Evalue_COG	KEGG	%Similarity_KEGG	Score_KEGG	Evalue_KEGG
8047	Prokka:JAPCLKMA_01223	-	hypothetical protein	1	1	1	1	1	1	1	1	1	1	1	11	CORE	COG3797_S#	39.2	121	4.44E-27				
8048	Prokka:CIODPCDJ_03319	-	hypothetical protein	1	1	0	1	1	1	1	1	1	1	1	10	Accessory								
8049	OSC64315.1	-	carbonic anhydrase	1	1	1	1	1	1	1	1	1	1	1	11	CORE	COG0288_P#	52.8	175	1.98E-43	K01673	52.8	175	5.29E-43
8050	WP_028959723.1	-	membrane protein	0	0	1	1	1	1	1	1	1	1	1	9	Accessory								
8051	WP_033275420.1	-	GNAT family N-acetyltransferase	0	0	0	0	0	0	1	0	0	0	0	1	Unique	COG0454_KR#	31.8	57	4.61E-08	K03789	26.9	50	0.0000151
8052	WP_146217844.1	-	HNH endonuclease	0	0	0	0	0	0	0	0	1	0	0	1	Unique								
8053	PWE08543.1	-	MarR family transcriptional regulator	0	0	1	1	0	0	1	1	1	1	0	6	Accessory								
8054	WP_033276575.1	-	hypothetical protein	1	0	0	0	0	1	1	0	0	0	0	3	Accessory								
8055	OSC66252.1	-	hypothetical protein	0	1	0	0	0	0	0	0	0	0	0	1	Unique								
8056	WP_019526413.1	-	SDR family oxidoreductase	0	0	0	1	1	1	0	1	0	0	1	5	Accessory	COG1028_IQR#	37.8	111	2.69E-24	NA	41.7	54	0.00000104
8057	AXI89134.1	-	hypothetical protein	0	0	0	1	0	1	0	1	0	1	0	4	Accessory								
8058	Prokka:JAPCLKMA_04895	-	hypothetical protein	1	0	0	0	0	0	0	0	0	0	0	1	Unique								
8059	PWE09317.1	-	HNH endonuclease	0	0	1	0	0	0	1	0	0	0	0	2	Accessory								

cluster	GI	gene	Annotation	Streptomyces_thermocarboxydus_BPSAC147	Streptomyces_sp._4F	Streptomyces_sp._BSE7F	Streptomyces_sp._ETH9427	Streptomyces_sp._FxanaD5	Streptomyces_sp._UNC401CLCol	Actinospica_acidiphila_NRRL_B_24431	Streptomyces_sp._HNS054	Streptomyces_sp._SMS_SU21	Streptomyces_sp._ZS0098	Streptomyces_thermocarboxydus_K155	Addition	Core-Accessory-Unique	COG	%Similarity_COG	Score_COG	Evalue_COG	KEGG	%Similarity_KEGG	Score_KEGG	Evalue_KEGG
8060	WP_106959508.1	-	TIGR00730 family Rossman fold protein	1	1	1	1	1	1	1	1	1	1	1	11	CORE	COG1611_R#	37.8	106	1.48E-22	NA	36.5	100	1.66E-20
8061	OSC70049.1	-	hypothetical protein	0	1	0	0	0	0	0	0	0	0	0	1	Unique								
8062	OSC61741.1	-	MarR family transcriptional regulator	0	1	0	0	0	0	0	0	0	0	0	1	Unique	COG1846_K#	44.4	132	1.92E-30	K15973	29.1	68	6.98E-11
8063	AXI91145.1	-	copper chaperone PCu(A)C	0	0	0	1	0	0	0	1	0	1	0	3	Accessory	COG2847_S#	29.1	50	0.00000962	K09796	27.6	45	0.000826
8064	OSC55806.1	-	cysteine methyltransferase	1	1	1	1	1	1	1	1	1	1	1	11	CORE	COG0350_L#	53.5	150	6.83E-36	K00567	53.5	150	1.82E-35
8065	WP_122215885.1	-	NIPSNAP family protein	0	0	0	0	0	0	0	0	0	1	0	1	Unique								
8066	OSC63222.1	-	hypothetical protein	0	1	0	0	0	0	0	0	0	0	0	1	Unique								
8067	Prokka:CIODPCDJ_06345	-	hypothetical protein	1	1	1	1	1	1	1	1	1	1	1	11	CORE								
8068	AXI90210.1	-	TetR family transcriptional regulator	0	0	1	1	0	0	1	1	1	1	0	6	Accessory	COG1309_K#	38	43	0.00117				
8069	WP_019523200.1	-	hypothetical protein	1	1	0	1	1	1	1	1	1	1	1	10	Accessory								
8070	WP_102637242.1	-	CinA family protein	1	1	1	1	1	1	1	1	1	1	1	11	CORE	COG1546_R#	39.3	112	1.2E-24	K03742	43.8	104	1.14E-21
8071	WP_028961291.1	-	hypothetical protein	0	0	0	0	0	1	0	0	0	0	0	1	Unique								

cluster	GI	gene	Annotation	Streptomyces_thermocarboxydus_BPSAC147	Streptomyces_sp._4F	Streptomyces_sp._BSE7F	Streptomyces_sp._ETH9427	Streptomyces_sp._FxanaD5	Streptomyces_sp._UNC401CLCol	Actinospica_acidiphila_NRRL_B_24431	Streptomyces_sp._HNS054	Streptomyces_sp._SMS_SU21	Streptomyces_sp._ZS0098	Streptomyces_thermocarboxydus_K155	Addition	Core-Accessory-Unique	COG	%Similarity_COG	Score_COG	Evalue_COG	KEGG	%Similarity_KEGG	Score_KEGG	Evalue_KEGG
8072	AXI86907.1	-	ArsR family transcriptional regulator	1	0	1	1	1	1	1	0	1	1	1	9	Accessory	COG1733_K#	43.8	138	2.05E-32	NA	40.1	107	1.76E-22
8073	WP_106959438.1	-	ATP-binding protein	0	0	0	0	0	1	0	0	0	0	0	1	Unique								
8074	PWE06908.1	-	hypothetical protein	1	1	1	0	0	0	0	0	1	0	0	4	Accessory								
8075	Prokka:JAPCLKMA_05681	-	hypothetical protein	1	0	0	0	0	0	0	0	0	0	0	1	Unique								
8076	WP_033277024.1	-	cysteine dioxygenase	1	1	0	0	1	1	1	0	0	0	1	6	Accessory								
8077	WP_019525921.1	-	bacterial proteasome activator family protein	1	1	1	1	1	1	1	1	1	1	1	11	CORE					NA	67.3	216	1.58E-55
8078	AXI91346.1	-	hypothetical protein	0	0	0	1	0	0	0	0	0	0	0	1	Unique								
8079	PWE12306.1	-	pyridoxamine 5'-phosphate oxidase family protein	0	1	1	0	0	0	1	0	1	1	0	5	Accessory								
8080	WP_102638077.1	-	hypothetical protein	0	0	1	0	0	0	0	0	1	0	0	2	Accessory								
8081	WP_033277127.1	-	flavin reductase family protein	0	0	0	0	0	0	1	0	0	0	0	1	Unique	COG1853_R#	45	153	8.02E-37	K16048	45	153	2.14E-36
8082	OSC64453.1	-	HNH endonuclease	1	1	1	1	1	1	1	1	1	1	1	11	CORE	COG1403_V#	45.7	122	1.16E-27	NA	45.7	122	3.1E-27

cluster	GI	gene	Annotation	Streptomyces_thermocarboxydus_BPSAC147	Streptomyces_sp._4F	Streptomyces_sp._BSE7F	Streptomyces_sp._ETH9427	Streptomyces_sp._FxanaD5	Streptomyces_sp._UNC401CLCol	Actinospica_acidiphila_NRRL_B_24431	Streptomyces_sp._HNS054	Streptomyces_sp._SMS_SU21	Streptomyces_sp._ZS0098	Streptomyces_thermocarboxydus_K155	Addition	Core-Accessory-Unique	COG	%Similarity_COG	Score_COG	Evalue_COG	KEGG	%Similarity_KEGG	Score_KEGG	Evalue_KEGG
8083	WP_048460651.1	-	DUF2267 domain-containing protein	1	0	1	0	1	1	1	1	1	1	1	9	Accessory								
8084	WP_122215282.1	-	hypothetical protein	0	0	1	0	0	0	0	1	1	1	0	4	Accessory								
8085	Prokka:JAPCLKMA_06620	-	hypothetical protein	1	0	0	0	0	0	0	0	0	0	0	1	Unique								
8086	AXI88760.1	-	hypothetical protein	0	0	0	1	0	0	0	0	0	0	0	1	Unique								
8087	OSC69933.1	-	GNAT family N-acetyltransferase	0	1	0	0	0	0	0	0	0	0	0	1	Unique								
8088	OSC57458.1	-	Repetin	0	1	0	0	0	0	0	0	0	0	0	1	Unique								
8089	OSC68330.1	-	hypothetical protein	1	1	1	1	1	1	1	1	1	1	1	11	CORE								
8090	AXI91089.1	-	Na+/H+ antiporter subunit E	0	0	0	1	0	0	0	0	0	0	0	1	Unique	COG1863_P#	33.6	82	1.74E-15	K05569	31.7	60	1.89E-08
8091	Prokka:JAPCLKMA_05614	-	hypothetical protein	1	1	1	1	1	1	1	1	1	1	1	11	CORE					NA	42.3	96	2.37E-19
8092	WP_033272356.1	-	RNA polymerase sigma factor	1	0	0	0	1	1	1	0	0	0	1	5	Accessory	COG1595_K#	35.4	85	2.04E-16	K03088	33.7	82	6.03E-15
8093	WP_122216882.1	-	response regulator	1	0	1	1	1	1	1	1	1	1	1	10	Accessory	COG0784_T#	37.4	93	9.8E-19	NA	31	64	9.97E-10
8094	Prokka:JAPCLKMA_03812	-	hypothetical protein	1	0	0	0	0	0	0	0	0	0	0	1	Unique								
8095	OSC69708.1	-	hypothetical protein	0	1	0	0	0	0	0	0	0	0	0	1	Unique								

cluster	GI	gene	Annotation	Streptomyces_thermocarboxydus_BPSAC147	Streptomyces_sp._4F	Streptomyces_sp._BSE7F	Streptomyces_sp._ETH9427	Streptomyces_sp._FxanaD5	Streptomyces_sp._UNC401CLCol	Actinospica_acidiphila_NRRL_B_24431	Streptomyces_sp._HNS054	Streptomyces_sp._SMS_SU21	Streptomyces_sp._ZS0098	Streptomyces_thermocarboxydus_K155	Addition	Core-Accessory-Unique	COG	%Similarity_COG	Score_COG	Evalue_COG	KEGG	%Similarity_KEGG	Score_KEGG	Evalue_KEGG
8096	OSC62700.1	-	RNA polymerase subunit sigma-70	0	1	0	0	0	0	0	0	0	0	0	1	Unique	COG1595_K#	38.7	127	4.69E-29	K03088	35.8	103	2.53E-21
8097	Prokka:JAPCLKMA_05446	-	hypothetical protein	1	1	1	1	1	1	1	1	1	1	1	11	CORE								
8098	OSC67246.1	-	5-(carboxyamino)imidazole ribonucleotide mutase	1	1	1	1	1	1	1	1	1	1	1	11	CORE	COG0041_F#	64.3	206	1.04E-52	K01588	65.9	205	3.62E-52
8099	OSC61641.1	-	alkylhydroperoxidase	0	1	0	0	0	0	0	0	0	0	0	1	Unique	COG2128_S#	39	98	3.05E-20	NA	46.8	60	1.88E-08
8100	Prokka:CIODPCDJ_04466	-	hypothetical protein	1	1	1	1	1	1	1	1	1	1	1	11	CORE	COG0314_H#	45.5	116	8.28E-26	K03635	45.5	116	2.21E-25
8101	AXI90647.1	-	hypothetical protein	1	0	0	1	0	0	0	0	0	0	0	2	Accessory								
8102	AXI87957.1	-	hypothetical protein	0	0	0	1	0	0	0	0	1	0	0	2	Accessory								
8103	WP_146217798.1	-	hypothetical protein	0	0	1	0	0	0	1	0	1	0	0	3	Accessory								
8104	PWE12163.1	-	RpiR family transcriptional regulator	1	1	1	0	1	1	1	1	1	1	1	10	Accessory	COG2259_S#	49.6	111	4.54E-24	K15977	46.8	108	7.86E-23
8105	Prokka:JAPCLKMA_04921	-	hypothetical protein	1	0	0	0	0	0	0	0	0	0	0	1	Unique								
8106	OSC53076.1	-	ABC transporter	0	1	0	0	0	0	0	0	0	0	0	1	Unique	COG1132_V#	39.3	118	2.18E-26	K06148	33	89	4.94E-17
8107	OSC66755.1	-	hypothetical protein	0	1	0	0	0	0	0	0	0	0	0	1	Unique								

cluster	GI	gene	Annotation	Streptomyces_thermocarboxydus_BPSAC147	Streptomyces_sp._4F	Streptomyces_sp._BSE7F	Streptomyces_sp._ETH9427	Streptomyces_sp._FxanaD5	Streptomyces_sp._UNC401CLCol	Actinospica_acidiphila_NRRL_B_24431	Streptomyces_sp._HNS054	Streptomyces_sp._SMS_SU21	Streptomyces_sp._ZS0098	Streptomyces_thermocarboxydus_K155	Addition	Core-Accessory-Unique	COG	%Similarity_COG	Score_COG	Evalue_COG	KEGG	%Similarity_KEGG	Score_KEGG	Evalue_KEGG
8108	WP_028959650.1	-	hypothetical protein	0	0	1	1	1	1	0	1	1	0	0	6	Accessory								
8109	WP_053077027.1	-	hypothetical protein	0	0	1	1	0	0	1	1	1	1	0	6	Accessory								
8110	OSC72203.1	-	GNAT family N-acetyltransferase	0	1	0	0	0	0	0	0	0	0	0	1	Unique								
8111	OSC57555.1	-	hypothetical protein	0	1	0	0	0	0	0	0	0	0	0	1	Unique								
8112	Prokka:JAPCLKMA_02784	-	hypothetical protein	1	0	0	0	0	0	0	0	0	0	0	1	Unique								
8113	WP_106432484.1	-	hypothetical protein	0	0	0	0	1	0	0	0	0	0	0	1	Unique								
8114	OSC56749.1	-	hypothetical protein	1	1	1	1	1	1	1	1	1	1	1	11	CORE					NA	46.1	128	5.59E-29
8115	WP_086700703.1	-	GNAT family N-acetyltransferase	1	1	1	1	1	1	1	1	1	1	1	11	CORE	COG0456_R#	28.1	57	7.74E-08	K03789	31.6	50	0.0000253
8116	WP_114873391.1	-	YbhB/YbcL family Raf kinase inhibitor-like protein	1	1	1	1	1	1	1	1	1	1	1	11	CORE	COG1881_R#	54.1	177	3.93E-44	K06910	54.1	177	1.05E-43
8117	Prokka:JAPCLKMA_05549	rplF	50S ribosomal protein L6	1	1	1	1	1	1	1	1	1	1	1	11	CORE	COG0097_J#	72.1	277	3.64E-74	K02933	72.1	277	9.73E-74
8118	AXI90652.1	-	hypothetical protein	1	0	0	1	0	0	0	0	0	0	0	2	Accessory								
8119	WP_122214256.1	-	DinB family protein	1	0	1	1	1	1	1	1	1	1	1	10	Accessory								

cluster	GI	gene	Annotation	Streptomyces_thermocarboxydus_BPSAC147	Streptomyces_sp._4F	Streptomyces_sp._BSE7F	Streptomyces_sp._ETH9427	Streptomyces_sp._FxanaD5	Streptomyces_sp._UNC401CLCol	Actinospica_acidiphila_NRRL_B_24431	Streptomyces_sp._HNS054	Streptomyces_sp._SMS_SU21	Streptomyces_sp._ZS0098	Streptomyces_thermocarboxydus_K155	Addition	Core-Accessory-Unique	COG	%Similarity_COG	Score_COG	Evalue_COG	KEGG	%Similarity_KEGG	Score_KEGG	Evalue_KEGG
8120	OSC67642.1	-	molybdenum cofactor biosynthesis protein	1	1	1	1	1	1	1	1	1	1	1	11	CORE	COG0521_H#	61.5	189	1.3E-47	NA	61.5	189	3.48E-47
8121	Prokka:CIODPCDJ_02960	-	hypothetical protein	1	1	1	1	1	1	1	1	1	1	1	11	CORE								
8122	WP_086699195.1	-	MarR family transcriptional regulator	0	1	1	1	0	0	0	1	1	1	0	6	Accessory	COG1846_K#	54.8	158	2.47E-38	NA	54.8	158	6.59E-38
8123	Prokka:JAPCLKMA_02743	cas1	CRISPR-associated endonuclease Cas1	1	0	0	0	0	0	0	0	0	0	0	1	Unique	COG1518_L#	41.3	143	1.07E-33	K15342	29.2	85	7.09E-16
8124	WP_048459557.1	-	SigE family RNA polymerase sigma factor	1	1	1	1	1	1	1	1	1	1	1	11	CORE	COG1595_K#	34.5	69	1.51E-11	K03088	34.5	69	4.02E-11
8125	OSC69057.1	-	hypothetical protein	1	1	1	1	1	1	1	1	1	1	1	11	CORE								
8126	WP_028959854.1	-	histidine phosphatase family protein	1	0	1	0	1	1	1	1	1	1	1	9	Accessory	COG2062_T#	46.4	141	4.08E-33	K08296	44.2	110	2.06E-23
8127	Prokka:CIODPCDJ_06745	-	hypothetical protein	0	0	0	0	0	0	0	0	0	0	1	1	Unique								
8128	WP_028961113.1	-	hypothetical protein	1	0	0	0	0	1	0	0	0	0	1	3	Accessory								
8129	OSC61044.1	-	hypothetical protein	0	1	0	0	0	0	0	0	0	0	0	1	Unique								
8130	AXI86920.1	-	hypothetical protein	0	0	0	1	0	0	1	1	0	0	0	3	Accessory								
8131	OSC61396.1	-	hypothetical protein	0	1	0	0	0	0	0	0	0	0	0	1	Unique								

cluster	GI	gene	Annotation	Streptomyces_thermocarboxydus_BPSAC147	Streptomyces_sp._4F	Streptomyces_sp._BSE7F	Streptomyces_sp._ETH9427	Streptomyces_sp._FxanaD5	Streptomyces_sp._UNC401CLCol	Actinospica_acidiphila_NRRL_B_24431	Streptomyces_sp._HNS054	Streptomyces_sp._SMS_SU21	Streptomyces_sp._ZS0098	Streptomyces_thermocarboxydus_K155	Addition	Core-Accessory-Unique	COG	%Similarity_COG	Score_COG	Evalue_COG	KEGG	%Similarity_KEGG	Score_KEGG	Evalue_KEGG
8132	WP_106432491.1	-	hypothetical protein	1	0	1	1	1	1	1	1	1	1	1	10	Accessory								
8133	OSC71739.1	-	phosphatase	1	1	1	1	1	1	1	1	1	1	1	11	CORE	COG1507_S#	61.1	195	2.38E-49	K09009	62	189	2.67E-47
8134	OSC71566.1	-	hypothetical protein	0	1	0	0	0	0	0	0	0	0	0	1	Unique								
8135	OSC67905.1	-	transposase	0	1	0	0	0	0	0	0	0	0	0	1	Unique	COG3415_L#	36.8	82	2.25E-15	K07499	36.8	82	6E-15
8136	OSC72590.1	-	YbjN domain-containing protein	1	1	1	1	1	1	1	1	1	1	1	11	CORE								
8137	WP_157842214.1	-	hypothetical protein	0	0	0	0	0	0	1	0	0	0	0	1	Unique								
8138	OSC73273.1	-	hypothetical protein	0	1	0	0	0	0	0	0	0	0	0	1	Unique	COG1846_K#	28	51	0.00000422				
8139	OSC63558.1	-	GNAT family N-acetyltransferase	0	1	0	0	0	0	0	0	0	0	0	1	Unique	COG1670_J#	29.5	60	9.09E-09	K03817	29.5	60	2.43E-08
8140	WP_086016100.1	-	DUF4916 domain-containing protein	1	1	1	1	1	1	1	1	1	1	1	11	CORE								
8141	WP_019524937.1	-	hypothetical protein	0	0	0	0	1	0	0	0	0	0	0	1	Unique								
8142	WP_048457461.1	-	hypothetical protein	0	0	0	0	0	0	0	1	0	1	0	2	Accessory								
8143	WP_028960679.1	-	hypothetical protein	0	0	0	1	1	1	1	1	1	1	1	8	Accessory								
8144	Prokka:CIODPCDJ_02255	-	hypothetical protein	1	1	1	1	1	1	1	1	1	1	1	11	CORE	COG1403_V#	53.1	176	8.7E-44	NA	53.1	176	2.32E-43
8145	Prokka:JAPCLKMA_06098	nicF	Maleamate amidohydrolase	1	1	1	1	1	1	1	1	1	1	1	11	CORE	COG1335_Q#	31.3	114	4.06E-25	NA	31.3	114	1.08E-24

cluster	GI	gene	Annotation	Streptomyces_thermocarboxydus_BPSAC147	Streptomyces_sp._4F	Streptomyces_sp._BSE7F	Streptomyces_sp._ETH9427	Streptomyces_sp._FxanaD5	Streptomyces_sp._UNC401CLCol	Actinospica_acidiphila_NRRL_B_24431	Streptomyces_sp._HNS054	Streptomyces_sp._SMS_SU21	Streptomyces_sp._ZS0098	Streptomyces_thermocarboxydus_K155	Addition	Core-Accessory-Unique	COG	%Similarity_COG	Score_COG	Evalue_COG	KEGG	%Similarity_KEGG	Score_KEGG	Evalue_KEGG
8146	OSC62502.1	-	helix-turn-helix transcriptional regulator	0	1	0	0	0	0	0	0	0	0	0	1	Unique								
8147	Prokka:JAPCLKMA_05686	rppH_8	RNA pyrophosphohydrolase	1	0	0	0	0	0	0	0	0	0	0	1	Unique	COG1051_F#	30.3	65	2.83E-10	K03574	30.3	65	7.55E-10
8148	WP_102642044.1	-	copper chaperone PCu(A)C	0	0	0	0	0	0	0	0	1	0	0	1	Unique	COG2847_S#	27.8	55	2.92E-07	K09796	27.8	55	7.81E-07
8149	PWE11308.1	-	hypothetical protein	0	0	1	1	0	0	0	1	1	1	0	5	Accessory	COG3196_S#	48.3	190	5.82E-48	K09925	48.3	190	1.56E-47
8150	WP_019521494.1	-	hypothetical protein	1	0	0	0	1	1	0	0	0	0	1	4	Accessory								
8151	AXI91416.1	-	hypothetical protein	1	0	0	1	1	1	0	0	0	0	0	4	Accessory								
8152	OSC71828.1	-	alkyl hydroperoxide reductase	1	1	1	1	1	1	1	1	1	1	1	11	CORE	COG2128_S#	56.3	186	6.44E-47	K04756	56.3	186	1.72E-46
8153	WP_102639843.1	-	GNAT family N-acetyltransferase	1	0	0	1	1	1	1	1	1	1	1	9	Accessory	COG1670_J#	33.6	65	1.66E-10	NA	32.9	65	4.42E-10
8154	OSC71196.1	-	deaminase	0	1	0	0	0	0	0	0	0	0	0	1	Unique	COG0262_H#	27.8	66	9.71E-11	NA	26.5	60	1.42E-08
8155	WP_048459700.1	-	hypothetical protein	0	0	0	0	0	0	1	1	0	1	0	3	Accessory								
8156	OSC71714.1	-	gamma carbonic anhydrase family protein	1	1	1	1	1	1	1	1	1	1	1	11	CORE	COG0663_R#	47.5	166	1.18E-40	NA	46.6	163	2.66E-39
8157	WP_033272737.1	-	hypothetical protein	0	0	1	0	1	1	1	1	1	1	1	8	Accessory								
8158	OSC47059.1	-	histidine kinase	0	1	0	0	0	0	0	0	0	0	0	1	Unique	COG0642_T#	39.5	95	1.95E-19	NA	39.5	95	5.21E-19

cluster	GI	gene	Annotation	Streptomyces_thermocarboxydus_BPSAC147	Streptomyces_sp._4F	Streptomyces_sp._BSE7F	Streptomyces_sp._ETH9427	Streptomyces_sp._FxanaD5	Streptomyces_sp._UNC401CLCol	Actinospica_acidiphila_NRRL_B_24431	Streptomyces_sp._HNS054	Streptomyces_sp._SMS_SU21	Streptomyces_sp._ZS0098	Streptomyces_thermocarboxydus_K155	Addition	Core-Accessory-Unique	COG	%Similarity_COG	Score_COG	Evalue_COG	KEGG	%Similarity_KEGG	Score_KEGG	Evalue_KEGG
8159	WP_028959317.1	-	hypothetical protein	1	0	0	0	1	1	1	0	0	0	1	5	Accessory								
8160	OSC67907.1	-	hypothetical protein	0	1	0	0	0	0	0	0	0	0	0	1	Unique								
8161	OSC73227.1	-	hypothetical protein	0	1	0	0	0	0	0	0	0	0	0	1	Unique								
8162	OSC63021.1	-	transcriptional regulator	1	1	1	1	1	1	1	1	1	1	1	11	CORE	COG1396_K#	50.4	126	7.91E-29	NA	50.4	126	2.11E-28
8163	OSC58736.1	-	NUDIX hydrolase	1	1	1	1	1	1	1	1	1	1	1	11	CORE	COG1051_F#	30.7	54	4.99E-07	NA	38.9	62	4.89E-09
8164	OSC65397.1	-	hypothetical protein	1	1	1	1	1	1	1	1	1	1	1	11	CORE								
8165	WP_020700044.1	-	hypothetical protein	1	0	1	1	1	1	0	1	1	1	1	9	Accessory								
8166	WP_026243653.1	-	GNAT family N-acetyltransferase	1	0	1	1	1	1	1	1	1	1	1	10	Accessory	COG1670_J#	27.6	69	1.95E-11	NA	31.2	55	7.77E-07
8167	PWE09725.1	-	TerD-family protein	1	1	1	1	1	1	1	1	1	1	1	11	CORE								
8168	AXI89406.1	-	hypothetical protein	1	0	1	1	1	1	1	1	1	1	1	10	Accessory					NA	30.5	62	3.72E-09
8169	WP_048459548.1	-	amino-acid N-acetyltransferase	1	1	1	1	1	1	1	1	1	1	1	11	CORE	COG1246_E#	62.2	202	1.47E-51	K00619	61.8	202	3.93E-51
8170	Prokka:JAPCLKMA_03783	-	hypothetical protein	1	0	0	1	0	0	1	1	0	0	1	5	Accessory								
8171	OSC64591.1	-	TetR family transcriptional regulator	0	1	0	0	0	0	0	0	0	0	0	1	Unique	COG1309_K#	34.5	79	1.88E-14	NA	34.5	79	5.02E-14

cluster	GI	gene	Annotation	Streptomyces_thermocarboxydus_BPSAC147	Streptomyces_sp._4F	Streptomyces_sp._BSE7F	Streptomyces_sp._ETH9427	Streptomyces_sp._FxanaD5	Streptomyces_sp._UNC401CLCol	Actinospica_acidiphila_NRRL_B_24431	Streptomyces_sp._HNS054	Streptomyces_sp._SMS_SU21	Streptomyces_sp._ZS0098	Streptomyces_thermocarboxydus_K155	Addition	Core-Accessory-Unique	COG	%Similarity_COG	Score_COG	Evalue_COG	KEGG	%Similarity_KEGG	Score_KEGG	Evalue_KEGG
8172	WP_019527630.1	-	sigma-70 family RNA polymerase sigma factor	1	1	1	1	1	1	1	1	1	1	1	11	CORE	COG1595_K#	35.2	72	2.3E-12	K03088	35.2	72	6.14E-12
8173	Prokka:JAPCLKMA_01073	-	Acetyltransferase	1	1	1	1	1	1	1	1	1	1	1	11	CORE	COG1670_J#	39.9	82	1.3E-15	NA	32.6	68	8.87E-11
8174	WP_048460444.1	-	calcium-binding protein	0	1	1	1	0	0	1	1	1	1	0	7	Accessory								
8175	WP_122212718.1	-	hypothetical protein	0	0	1	1	0	0	0	1	1	1	0	5	Accessory								
8176	WP_019525589.1	-	DoxX family protein	1	1	1	1	1	1	1	1	1	1	1	11	CORE	COG2259_S#	38.1	96	8.72E-20				
8177	Prokka:JAPCLKMA_03005	-	Putative decaprenylphosphoryl-5-phosphoribose phosphatase	1	1	1	1	1	1	1	1	1	1	1	11	CORE	COG0671_I#	50.7	121	2.53E-27	NA	41.1	101	9.45E-21
8178	WP_019522577.1	-	GNAT family N-acetyltransferase	1	0	1	0	1	1	1	0	1	0	1	7	Accessory	COG0454_KR#	51	143	1.06E-33				
8179	OSC71300.1	-	hypothetical protein	0	1	0	0	0	0	0	0	0	0	0	1	Unique								
8180	OSC67422.1	-	6-O-methylguanine DNA methyltransferase	1	1	1	1	1	1	1	1	1	1	1	11	CORE	COG3695_L#	41.7	54	0.00000038	NA	41.7	54	0.00000101
8181	Prokka:JAPCLKMA_02213	-	hypothetical protein	1	0	0	0	0	0	0	0	0	0	0	1	Unique								

cluster	GI	gene	Annotation	Streptomyces_thermocarboxydus_BPSAC147	Streptomyces_sp._4F	Streptomyces_sp._BSE7F	Streptomyces_sp._ETH9427	Streptomyces_sp._FxanaD5	Streptomyces_sp._UNC401CLCol	Actinospica_acidiphila_NRRL_B_24431	Streptomyces_sp._HNS054	Streptomyces_sp._SMS_SU21	Streptomyces_sp._ZS0098	Streptomyces_thermocarboxydus_K155	Addition	Core-Accessory-Unique	COG	%Similarity_COG	Score_COG	Evalue_COG	KEGG	%Similarity_KEGG	Score_KEGG	Evalue_KEGG
8182	WP_102638385.1	-	hypothetical protein	0	0	0	0	0	0	0	0	1	0	0	1	Unique								
8183	AXI91375.1	-	hypothetical protein	0	0	0	1	0	0	0	0	0	0	0	1	Unique								
8184	AXI87974.1	-	cysteine dioxygenase	1	1	0	1	1	1	1	1	1	1	1	10	Accessory					NA	40.7	83	2.66E-15
8185	OSC72419.1	-	GNAT family N-acetyltransferase	0	1	0	0	0	0	0	0	0	0	0	1	Unique	COG1670_J#	32.2	90	8.16E-18	NA	32.5	91	1.28E-17
8186	OSC72501.1	-	hypothetical protein	1	1	0	1	0	0	1	0	0	1	0	5	Accessory	COG3196_S#	48.6	157	4.16E-38	K09925	48.6	157	1.11E-37
8187	AXI84935.1	-	hypothetical protein	0	0	0	1	0	0	0	0	0	0	0	1	Unique								
8188	WP_146217846.1	-	helix-turn-helix transcriptional regulator	0	0	0	0	0	0	0	0	1	0	0	1	Unique	COG2207_K#	48.6	173	7.33E-43	NA	34.6	64	9.8E-10
8189	WP_122213805.1	-	VUT family protein	0	0	0	0	0	0	0	0	0	1	0	1	Unique								
8190	WP_158688233.1	-	hypothetical protein	0	0	0	0	1	0	0	0	0	0	0	1	Unique								
8191	OSC62259.1	-	NUDIX hydrolase	1	1	1	1	1	1	1	1	1	1	1	11	CORE	COG1051_F#	35.7	65	2.81E-10	NA	30.3	60	2.41E-08
8192	OSC57750.1	-	hypothetical protein	0	1	0	0	0	0	0	0	0	0	0	1	Unique								
8193	OSC72481.1	-	hypothetical protein	0	1	0	0	0	0	0	0	0	0	0	1	Unique								
8194	OSC70125.1	-	50S ribosomal protein L10	1	1	1	1	1	1	1	1	1	1	1	11	CORE	COG0244_J#	58	186	8.32E-47	K02864	58	186	2.22E-46
8195	WP_102639922.1	-	hypothetical protein	1	0	0	1	1	1	1	1	1	1	1	9	Accessory								
8196	Prokka:JAPCLKMA_05703	-	hypothetical protein	1	1	0	0	1	1	0	0	0	0	1	5	Accessory	COG1695_K#	33.7	81	3.77E-15	NA	33.7	81	1.01E-14

cluster	GI	gene	Annotation	Streptomyces_thermocarboxydus_BPSAC147	Streptomyces_sp._4F	Streptomyces_sp._BSE7F	Streptomyces_sp._ETH9427	Streptomyces_sp._FxanaD5	Streptomyces_sp._UNC401CLCol	Actinospica_acidiphila_NRRL_B_24431	Streptomyces_sp._HNS054	Streptomyces_sp._SMS_SU21	Streptomyces_sp._ZS0098	Streptomyces_thermocarboxydus_K155	Addition	Core-Accessory-Unique	COG	%Similarity_COG	Score_COG	Evalue_COG	KEGG	%Similarity_KEGG	Score_KEGG	Evalue_KEGG
8197	Prokka:CIODPCDJ_05361	-	hypothetical protein	1	0	1	1	1	1	1	1	1	1	1	10	Accessory								
8198	WP_063837959.1	-	DUF1707 and DUF4190 domain-containing protein	1	0	1	1	1	1	1	1	1	1	1	10	Accessory					NA	40	43	0.00233
8199	WP_102641354.1	-	hypothetical protein	0	0	1	0	0	0	0	0	1	0	0	2	Accessory								
8200	WP_048454906.1	-	SRPBCC family protein	0	0	1	1	0	0	0	1	1	1	0	5	Accessory								
8201	Prokka:JAPCLKMA_03126	-	hypothetical protein	1	0	1	1	1	1	1	1	1	1	1	10	Accessory								
8202	OSC63333.1	-	toxin	1	1	0	0	1	1	0	0	0	0	1	5	Accessory								
8203	WP_122215555.1	-	hypothetical protein	0	0	0	0	0	0	0	0	0	1	0	1	Unique	COG1511_S#	28.5	51	0.0000032	NA	36.5	77	1.45E-13
8204	WP_026243477.1	-	hypothetical protein	1	0	1	1	1	1	1	1	1	1	1	10	Accessory								
8205	OSC69634.1	-	SMI1/KNR4 family protein	0	1	0	0	0	0	0	0	0	0	0	1	Unique					NA	37.6	104	1.45E-21
8206	WP_159020974.1	-	hypothetical protein	0	0	0	0	0	0	0	1	0	0	0	1	Unique	COG4451_C#	38.7	97	5.08E-20	K01602	69.5	263	1.09E-69
8207	OSC72098.1	-	polyketide cyclase	0	1	0	0	0	0	0	0	0	0	0	1	Unique								
8208	WP_102637149.1	-	MarR family transcriptional regulator	0	0	0	1	0	0	1	1	1	0	0	4	Accessory	COG1846_K#	35.8	55	2.89E-07				

cluster	GI	gene	Annotation	Streptomyces_thermocarboxydus_BPSAC147	Streptomyces_sp._4F	Streptomyces_sp._BSE7F	Streptomyces_sp._ETH9427	Streptomyces_sp._FxanaD5	Streptomyces_sp._UNC401CLCol	Actinospica_acidiphila_NRRL_B_24431	Streptomyces_sp._HNS054	Streptomyces_sp._SMS_SU21	Streptomyces_sp._ZS0098	Streptomyces_thermocarboxydus_K155	Addition	Core-Accessory-Unique	COG	%Similarity_COG	Score_COG	Evalue_COG	KEGG	%Similarity_KEGG	Score_KEGG	Evalue_KEGG
8209	WP_122216177.1	-	PPOX class F420-dependent oxidoreductase	1	0	0	0	1	1	0	0	0	1	1	5	Accessory					K07005	32.4	86	4.09E-16
8210	WP_020700229.1	-	GNAT family N-acetyltransferase	1	0	0	0	1	0	1	0	0	0	1	4	Accessory								
8211	OSC71510.1	-	hypothetical protein	0	1	0	0	0	0	0	0	0	0	0	1	Unique								
8212	WP_033273233.1	-	peptidylprolyl isomerase	1	1	1	1	1	1	1	1	1	1	1	11	CORE	COG0652_O#	65.7	219	8.81E-57	K03767	63.9	218	6.85E-56
8213	WP_048457758.1	-	universal stress protein	1	0	1	1	1	1	1	1	1	1	1	10	Accessory								
8214	WP_122213511.1	-	NUDIX hydrolase	1	1	1	1	1	1	1	1	1	1	1	11	CORE								
8215	PWE10308.1	-	hypothetical protein	0	0	1	0	0	0	0	0	0	0	0	1	Unique								
8216	WP_051101659.1	-	methyltransferase	0	0	0	0	1	0	0	0	0	0	0	1	Unique	COG2518_O#	35.7	89	1.8E-17	K00573	33.7	75	4.2E-13
8217	AXI86399.1	-	pyridoxamine 5'-phosphate oxidase	1	0	1	1	1	1	0	1	1	1	1	9	Accessory								
8218	Prokka:JAPCLKMA_04670	-	hypothetical protein	1	0	0	0	1	1	0	0	0	0	1	4	Accessory								
8219	OSC59210.1	-	thioesterase	0	1	0	0	0	0	0	0	0	0	0	1	Unique	COG3208_Q#	29.6	67	5.6E-11				
8220	WP_033272632.1	-	hypothetical protein	0	0	1	1	0	0	1	1	0	1	0	5	Accessory								
8221	Prokka:CIODPCDJ_06582	-	hypothetical protein	1	0	0	0	1	1	0	0	0	0	1	4	Accessory	COG3409_M#	40.7	45	0.000228				

cluster	GI	gene	Annotation	Streptomyces_thermocarboxydus_BPSAC147	Streptomyces_sp._4F	Streptomyces_sp._BSE7F	Streptomyces_sp._ETH9427	Streptomyces_sp._FxanaD5	Streptomyces_sp._UNC401CLCol	Actinospica_acidiphila_NRRL_B_24431	Streptomyces_sp._HNS054	Streptomyces_sp._SMS_SU21	Streptomyces_sp._ZS0098	Streptomyces_thermocarboxydus_K155	Addition	Core-Accessory-Unique	COG	%Similarity_COG	Score_COG	Evalue_COG	KEGG	%Similarity_KEGG	Score_KEGG	Evalue_KEGG
8222	OSC69208.1	-	hypothetical protein	0	1	0	0	0	0	0	0	0	0	0	1	Unique					NA	32.5	53	0.00000292
8223	Prokka:JAPCLKMA_02869	-	hypothetical protein	1	1	1	1	1	1	1	1	1	1	1	11	CORE	COG1846_K#	42.9	121	3.27E-27	NA	23.7	53	0.00000292
8224	WP_037779537.1	-	hypothetical protein	0	0	0	0	1	0	0	0	0	0	1	2	Accessory								
8225	OSC60030.1	-	hypothetical protein	1	1	1	1	1	1	1	1	1	1	1	11	CORE								
8226	Prokka:JAPCLKMA_06544	rppH_9	RNA pyrophosphohydrolase	1	1	1	0	1	0	0	1	1	1	1	8	Accessory	COG1051_F#	39.8	75	2.68E-13	NA	33.8	72	6.07E-12
8227	OSC65797.1	-	hypothetical protein	0	1	0	0	0	0	0	0	0	0	0	1	Unique								
8228	OSC71738.1	-	acyl-phosphate glycerol 3-phosphate acyltransferase	1	1	1	1	1	1	1	1	1	1	1	11	CORE	COG2919_D#	35.3	80	8.35E-15	NA	35.3	80	2.23E-14
8229	Prokka:JAPCLKMA_05499	-	hypothetical protein	1	0	0	0	0	0	0	0	0	0	0	1	Unique								
8230	OSC63556.1	-	transferase	0	1	0	0	0	0	0	0	0	0	0	1	Unique	COG0110_R#	45.7	154	2.67E-37	K00661	42.4	126	3.54E-28
8231	OSC70576.1	-	hypothetical protein	0	1	0	0	0	0	0	0	0	0	0	1	Unique								
8232	PWE10920.1	-	DUF488 domain-containing protein	1	0	1	1	1	1	1	1	1	1	1	10	Accessory	COG5483_S#	32.2	86	8.87E-17	NA	32.7	87	1.06E-16
8233	PWE08797.1	-	N-acetyltransferase	1	1	1	1	1	1	1	1	1	1	1	11	CORE	COG0454_KR#	31.1	62	2.34E-09				

cluster	GI	gene	Annotation	Streptomyces_thermocarboxydus_BPSAC147	Streptomyces_sp._4F	Streptomyces_sp._BSE7F	Streptomyces_sp._ETH9427	Streptomyces_sp._FxanaD5	Streptomyces_sp._UNC401CLCol	Actinospica_acidiphila_NRRL_B_24431	Streptomyces_sp._HNS054	Streptomyces_sp._SMS_SU21	Streptomyces_sp._ZS0098	Streptomyces_thermocarboxydus_K155	Addition	Core-Accessory-Unique	COG	%Similarity_COG	Score_COG	Evalue_COG	KEGG	%Similarity_KEGG	Score_KEGG	Evalue_KEGG
8234	WP_019524397.1	ilvN	acetolactate synthase small subunit	1	0	1	1	1	1	1	1	1	1	1	10	Accessory	COG0440_E#	64.8	209	9.07E-54	K01653	64.8	209	2.42E-53
8235	AXI90987.1	-	PadR family transcriptional regulator	1	1	1	1	1	1	1	1	1	1	1	11	CORE	COG1695_K#	29	65	2.12E-10	NA	25.4	59	5.29E-08
8236	Prokka:JAPCLKMA_01340	-	hypothetical protein	1	0	0	0	0	0	0	0	0	0	0	1	Unique								
8237	WP_019526031.1	-	hypothetical protein	1	0	0	0	1	0	0	0	0	1	0	3	Accessory								
8238	OSC60988.1	-	hypothetical protein	0	1	0	0	0	0	0	0	0	0	0	1	Unique								
8239	WP_019526988.1	-	hypothetical protein	0	0	0	0	1	0	0	0	0	0	1	2	Accessory								
8240	Prokka:JAPCLKMA_02855	mscL	Large-conductance mechanosensitive channel	1	1	1	1	1	1	1	1	1	1	1	11	CORE	COG1970_M#	43.3	100	7.75E-21	K03282	39.9	87	1.06E-16
8241	WP_048457136.1	-	hypothetical protein	0	0	0	1	0	0	0	1	0	1	0	3	Accessory								
8242	OSC57434.1	-	hypothetical protein	0	1	0	1	0	0	1	1	0	1	0	5	Accessory								
8243	Prokka:JAPCLKMA_00711	-	hypothetical protein	1	0	1	0	1	1	0	0	0	0	1	5	Accessory	COG1366_T#	32.6	57	7.52E-08	K04749	32.6	57	2.01E-07
8244	AXI85790.1	-	disulfide bond formation protein DsbA	1	1	1	1	1	1	1	1	1	1	1	11	CORE	COG1651_O#	28.9	69	1.46E-11	NA	27.8	54	0.0000013
8245	PWE08577.1	-	diguanylate cyclase	0	0	1	0	0	0	0	0	0	0	0	1	Unique								

cluster	GI	gene	Annotation	Streptomyces_thermocarboxydus_BPSAC147	Streptomyces_sp._4F	Streptomyces_sp._BSE7F	Streptomyces_sp._ETH9427	Streptomyces_sp._FxanaD5	Streptomyces_sp._UNC401CLCol	Actinospica_acidiphila_NRRL_B_24431	Streptomyces_sp._HNS054	Streptomyces_sp._SMS_SU21	Streptomyces_sp._ZS0098	Streptomyces_thermocarboxydus_K155	Addition	Core-Accessory-Unique	COG	%Similarity_COG	Score_COG	Evalue_COG	KEGG	%Similarity_KEGG	Score_KEGG	Evalue_KEGG
8246	OSC64590.1	-	hypothetical protein	0	1	0	0	0	0	0	0	0	1	0	2	Accessory								
8247	WP_051747681.1	-	hypothetical protein	1	0	1	1	1	1	1	1	1	1	1	10	Accessory					NA	46.7	43	0.0023
8248	WP_158688213.1	-	hypothetical protein	0	0	0	0	1	0	0	0	0	0	0	1	Unique								
8249	OSC60985.1	-	hypothetical protein	0	1	0	0	0	0	0	0	0	0	0	1	Unique								
8250	Prokka:JAPCLKMA_04900	-	hypothetical protein	1	0	0	0	0	0	0	0	0	0	0	1	Unique								
8251	WP_033273847.1	-	DUF3291 domain-containing protein	0	0	0	0	0	0	1	0	0	0	0	1	Unique								
8252	OSC64982.1	-	hypothetical protein	0	1	0	0	0	0	0	0	0	0	0	1	Unique								
8253	OSC62801.1	-	hypothetical protein	1	1	1	1	1	1	1	1	1	1	1	11	CORE								
8254	OSC70837.1	-	hypothetical protein	1	1	1	1	1	1	1	1	1	1	1	11	CORE	COG2018_R#	29.1	57	5.76E-08	K07131	29.1	57	1.54E-07
8255	WP_048458609.1	-	hypothetical protein	0	0	0	1	0	0	0	1	0	0	0	2	Accessory								
8256	WP_048456741.1	-	DUF4240 domain-containing protein	1	0	1	1	1	1	1	1	1	1	1	10	Accessory								
8257	PWE08129.1	-	YbjN domain-containing protein	1	1	1	1	1	1	1	1	1	1	1	11	CORE					NA	47.3	161	5.76E-39
8258	WP_019527904.1	-	DoxX family protein	1	1	1	1	1	1	1	1	1	1	1	11	CORE	COG2259_S#	30.7	57	4.39E-08				
8259	WP_048458809.1	-	NUDIX domain-containing protein	1	0	0	1	1	1	1	1	1	1	1	9	Accessory	COG0494_LR#	31	62	1.36E-09	NA	30.6	69	3.89E-11

cluster	GI	gene	Annotation	Streptomyces_thermocarboxydus_BPSAC147	Streptomyces_sp._4F	Streptomyces_sp._BSE7F	Streptomyces_sp._ETH9427	Streptomyces_sp._FxanaD5	Streptomyces_sp._UNC401CLCol	Actinospica_acidiphila_NRRL_B_24431	Streptomyces_sp._HNS054	Streptomyces_sp._SMS_SU21	Streptomyces_sp._ZS0098	Streptomyces_thermocarboxydus_K155	Addition	Core-Accessory-Unique	COG	%Similarity_COG	Score_COG	Evalue_COG	KEGG	%Similarity_KEGG	Score_KEGG	Evalue_KEGG
8260	WP_122214150.1	-	nitroreductase family deazaflavin-dependent oxidoreductase	0	0	1	1	0	0	0	1	1	1	0	5	Accessory								
8261	WP_122214149.1	-	hypothetical protein	0	0	1	1	0	0	0	1	1	1	0	5	Accessory								
8262	WP_028959122.1	-	PepSY domain-containing protein	1	0	0	0	1	1	0	0	0	0	1	4	Accessory	COG5277_Z#	38.6	49	0.0000156	NA	51.9	60	2.36E-08
8263	WP_122213271.1	-	DUF1918 domain-containing protein	0	0	0	0	0	0	0	0	1	1	0	2	Accessory					NA	37.9	42	0.00509
8264	WP_019527540.1	-	hypothetical protein	1	0	0	0	1	0	0	0	0	0	1	3	Accessory								
8265	Prokka:JAPCLKMA_04663	-	hypothetical protein	1	0	0	0	0	1	0	0	0	0	1	3	Accessory								
8266	Prokka:CIODPCDJ_05093	-	hypothetical protein	1	0	0	1	1	1	0	1	0	1	1	7	Accessory								
8267	OSC66764.1	-	glutathione peroxidase	1	1	1	1	1	1	1	1	1	1	1	11	CORE	COG0386_O#	59.2	194	3.92E-49	K00432	47.3	167	1.05E-40
8268	WP_078493171.1	-	ATP-binding protein	0	0	1	0	0	1	1	0	0	0	0	3	Accessory	COG0645_R#	35.6	77	6.99E-14	K07028	35.6	77	1.87E-13
8269	Prokka:JAPCLKMA_04902	-	hypothetical protein	1	0	0	0	0	0	0	0	0	0	0	1	Unique								
8270	OSC61734.1	-	cupin	0	1	0	0	0	0	0	0	0	0	0	1	Unique								

cluster	GI	gene	Annotation	Streptomyces_thermocarboxydus_BPSAC147	Streptomyces_sp._4F	Streptomyces_sp._BSE7F	Streptomyces_sp._ETH9427	Streptomyces_sp._FxanaD5	Streptomyces_sp._UNC401CLCol	Actinospica_acidiphila_NRRL_B_24431	Streptomyces_sp._HNS054	Streptomyces_sp._SMS_SU21	Streptomyces_sp._ZS0098	Streptomyces_thermocarboxydus_K155	Addition	Core-Accessory-Unique	COG	%Similarity_COG	Score_COG	Evalue_COG	KEGG	%Similarity_KEGG	Score_KEGG	Evalue_KEGG
8271	AXI85383.1	-	hypothetical protein	0	0	0	1	0	0	0	0	0	0	0	1	Unique								
8272	Prokka:CIODPCDJ_00924	-	hypothetical protein	1	1	1	1	1	1	1	1	1	1	1	11	CORE					NA	31.9	76	2.44E-13
8273	WP_033272403.1	-	pyridoxamine 5'-phosphate oxidase	1	1	1	1	1	1	1	1	1	1	1	11	CORE					NA	37.8	50	0.0000187
8274	WP_033276644.1	-	hypothetical protein	1	1	0	0	0	0	1	0	0	0	1	4	Accessory								
8275	Prokka:JAPCLKMA_01197	-	hypothetical protein	1	0	1	1	1	1	1	1	1	1	1	10	Accessory								
8276	OSC65156.1	-	hypothetical protein	0	1	0	0	0	0	0	0	0	0	0	1	Unique								
8277	AXI85279.1	-	DNA-binding protein	1	0	0	1	0	0	0	0	0	0	0	2	Accessory								
8278	OSC67643.1	-	cyclic pyranopterin monophosphate synthase MoaC	1	1	1	1	1	1	1	1	1	1	1	11	CORE	COG0315_H#	60.1	188	2.81E-47	K03637	60.1	188	7.5E-47
8279	OSC69759.1	-	DUF4440 domain-containing protein	1	1	1	1	1	1	1	1	1	1	1	11	CORE								
8280	OSC63823.1	-	hypothetical protein	1	1	0	0	1	1	1	0	0	0	1	6	Accessory								
8281	OSC70336.1	-	hypothetical protein	0	1	0	0	0	0	0	0	0	0	0	1	Unique								
8282	Prokka:JAPCLKMA_02201	-	hypothetical protein	1	1	1	1	1	1	1	1	1	1	1	11	CORE	COG3682_K#	38.1	57	5.73E-08				

cluster	GI	gene	Annotation	Streptomyces_thermocarboxydus_BPSAC147	Streptomyces_sp._4F	Streptomyces_sp._BSE7F	Streptomyces_sp._ETH9427	Streptomyces_sp._FxanaD5	Streptomyces_sp._UNC401CLCol	Actinospica_acidiphila_NRRL_B_24431	Streptomyces_sp._HNS054	Streptomyces_sp._SMS_SU21	Streptomyces_sp._ZS0098	Streptomyces_thermocarboxydus_K155	Addition	Core-Accessory-Unique	COG	%Similarity_COG	Score_COG	Evalue_COG	KEGG	%Similarity_KEGG	Score_KEGG	Evalue_KEGG
8283	OSC70242.1	-	TetR family transcriptional regulator	0	1	0	0	0	0	0	0	0	0	0	1	Unique								
8284	PWE09616.1	-	Lrp/AsnC family transcriptional regulator	1	1	1	1	1	1	1	1	1	1	1	11	CORE	COG1522_K#	27.6	84	3.33E-16	K03718	28.7	76	2.42E-13
8285	WP_048457706.1	-	hypothetical protein	0	0	1	1	0	0	1	1	1	1	0	6	Accessory								
8286	Prokka:CIODPCDJ_02787	bar	Phosphinothricin N-acetyltransferase	1	1	1	1	1	1	1	1	1	1	1	11	CORE	COG1247_M#	34.5	116	1.03E-25	K03823	34.4	97	1.73E-19
8287	WP_048455660.1	-	hypothetical protein	1	1	0	1	1	1	1	1	1	1	1	10	Accessory					K07131	35.4	82	3.38E-15
8288	OSC69514.1	-	FHA domain-containing protein	1	1	1	1	1	1	1	1	1	1	1	11	CORE	COG1716_T#	45	141	2.3E-33	NA	45	141	6.13E-33
8289	OSC64595.1	-	chitin-binding protein	0	1	0	0	0	0	0	0	0	0	0	1	Unique	COG3397_S#	31.4	92	1.6E-18	K03933	31.4	92	4.27E-18
8290	WP_048460448.1	-	RNA polymerase sigma factor	0	1	1	1	0	0	1	1	1	1	0	7	Accessory	COG1595_K#	33.5	86	1.5E-16	K03088	33.5	86	3.99E-16
8291	WP_033276723.1	-	gluconokinase	1	1	1	1	1	1	1	1	1	1	1	11	CORE	COG3265_G#	56.7	182	1.17E-45	K00851	56.7	182	3.13E-45
8292	WP_158693612.1	-	hypothetical protein	0	0	0	0	0	1	0	0	0	0	1	2	Accessory								
8293	WP_122217432.1	-	single-stranded DNA-binding protein	1	1	1	1	1	1	1	1	1	1	1	11	CORE								

cluster	GI	gene	Annotation	Streptomyces_thermocarboxydus_BPSAC147	Streptomyces_sp._4F	Streptomyces_sp._BSE7F	Streptomyces_sp._ETH9427	Streptomyces_sp._FxanaD5	Streptomyces_sp._UNC401CLCol	Actinospica_acidiphila_NRRL_B_24431	Streptomyces_sp._HNS054	Streptomyces_sp._SMS_SU21	Streptomyces_sp._ZS0098	Streptomyces_thermocarboxydus_K155	Addition	Core-Accessory-Unique	COG	%Similarity_COG	Score_COG	Evalue_COG	KEGG	%Similarity_KEGG	Score_KEGG	Evalue_KEGG
8294	WP_019521978.1	bfr	bacterioferritin	1	1	1	1	1	1	1	1	1	1	1	11	CORE	COG2193_P#	63.5	204	2.88E-52	K03594	63.5	204	7.7E-52
8295	OSC64625.1	-	hypothetical protein	0	1	0	0	0	0	0	0	0	0	0	1	Unique								
8296	WP_048458532.1	-	histidine phosphatase family protein	1	1	1	1	1	1	1	1	1	1	1	11	CORE	COG2062_T#	41.1	121	4.19E-27	K08296	40.1	111	8.87E-24
8297	Prokka:CIODPCDJ_04234	-	hypothetical protein	1	1	0	1	1	1	1	1	1	1	1	10	Accessory	COG1846_K#	34.3	80	6.28E-15	NA	32.4	61	1.05E-08
8298	OSC73365.1	-	redox-sensitive transcriptional activator SoxR	1	1	1	1	1	1	1	1	1	1	1	11	CORE	COG0789_K#	64.1	183	5.27E-46	K13639	60.1	171	5.53E-42
8299	WP_122213871.1	-	tryptophan-rich sensory protein	0	0	1	1	0	0	1	1	1	1	0	6	Accessory	COG3476_T#	46.7	137	5.65E-32	K07185	34	104	1.42E-21
8300	Prokka:JAPCLKMA_05629	-	hypothetical protein	1	0	0	0	0	0	0	0	0	0	0	1	Unique								
8301	OSC73530.1	-	hypothetical protein	1	1	1	1	1	1	1	1	1	1	1	11	CORE								
8302	OSC57527.1	-	hypothetical protein	0	1	0	0	0	0	0	0	0	0	0	1	Unique								
8303	AXI89602.1	-	ATP-binding protein	0	0	0	1	0	0	0	0	0	0	0	1	Unique					NA	30.2	49	0.0000414
8304	WP_122213460.1	-	hypothetical protein	1	0	1	1	1	1	1	1	1	1	1	10	Accessory	COG1755_S#	65.4	219	1.13E-56	K16168	65.4	219	3.02E-56
8305	OSC54969.1	-	TIGR03086 family protein	0	1	0	0	0	0	0	0	0	0	0	1	Unique					NA	33.1	68	6.6E-11

cluster	GI	gene	Annotation	Streptomyces_thermocarboxydus_BPSAC147	Streptomyces_sp._4F	Streptomyces_sp._BSE7F	Streptomyces_sp._ETH9427	Streptomyces_sp._FxanaD5	Streptomyces_sp._UNC401CLCol	Actinospica_acidiphila_NRRL_B_24431	Streptomyces_sp._HNS054	Streptomyces_sp._SMS_SU21	Streptomyces_sp._ZS0098	Streptomyces_thermocarboxydus_K155	Addition	Core-Accessory-Unique	COG	%Similarity_COG	Score_COG	Evalue_COG	KEGG	%Similarity_KEGG	Score_KEGG	Evalue_KEGG
8306	Prokka:JAPCLKMA_04704	rppH_7	RNA pyrophosphohydrolase	1	1	1	1	1	1	1	1	1	1	1	11	CORE	COG0494_LR#	30.2	54	0.00000063	K03574	30.2	54	0.00000168
8307	OSC52248.1	-	serine/threonine protein kinase	0	1	0	0	0	0	0	0	0	0	0	1	Unique								
8308	WP_102640153.1	-	hypothetical protein	0	0	1	0	0	0	0	0	1	0	0	2	Accessory								
8309	WP_033276313.1	-	DUF4334 domain-containing protein	1	0	0	0	1	1	1	0	0	0	1	5	Accessory					NA	40	48	0.0000706
8310	PWE12393.1	-	hypothetical protein	0	0	1	0	0	0	0	0	0	0	0	1	Unique								
8311	AXI86917.1	-	GNAT family N-acetyltransferase	0	1	1	1	0	0	0	1	1	1	0	6	Accessory	COG0454_KR#	28.2	75	1.55E-13				
8312	OSC69399.1	-	hypothetical protein	0	1	0	0	0	0	0	0	0	0	0	1	Unique								
8313	PWE10275.1	-	hypothetical protein	0	0	1	0	0	0	0	0	0	0	0	1	Unique	COG2020_O#	36.8	75	1.55E-13				
8314	WP_109288799.1	-	IS3 family transposase	0	0	0	0	0	0	0	0	1	0	0	1	Unique	COG2801_L#	95.2	242	1.63E-63	K07497	39.6	124	1.01E-27
8315	PWE07423.1	-	hypothetical protein	0	0	1	0	0	0	0	1	0	0	0	2	Accessory								
8316	OSC60430.1	-	hypothetical protein	0	1	0	0	0	0	0	0	0	0	0	1	Unique								
8317	PWE11425.1	-	hypothetical protein	0	0	1	0	0	0	0	0	0	0	0	1	Unique								
8318	OSC71951.1	-	hypothetical protein	0	1	0	0	0	0	0	0	0	0	0	1	Unique								

cluster	GI	gene	Annotation	Streptomyces_thermocarboxydus_BPSAC147	Streptomyces_sp._4F	Streptomyces_sp._BSE7F	Streptomyces_sp._ETH9427	Streptomyces_sp._FxanaD5	Streptomyces_sp._UNC401CLCol	Actinospica_acidiphila_NRRL_B_24431	Streptomyces_sp._HNS054	Streptomyces_sp._SMS_SU21	Streptomyces_sp._ZS0098	Streptomyces_thermocarboxydus_K155	Addition	Core-Accessory-Unique	COG	%Similarity_COG	Score_COG	Evalue_COG	KEGG	%Similarity_KEGG	Score_KEGG	Evalue_KEGG
8319	WP_122214191.1	-	O-acetyl-ADP-ribose deacetylase	1	1	1	1	1	1	1	1	1	1	1	11	CORE	COG2110_R#	53.1	159	1.06E-38	NA	48.3	141	1.04E-32
8320	AXI86151.1	-	PadR family transcriptional regulator	1	1	1	1	1	1	1	1	1	1	1	11	CORE	COG1695_K#	27.1	75	2.01E-13	NA	26.1	62	3.6E-09
8321	AXI84855.1	-	GNAT family N-acetyltransferase	0	0	0	1	1	0	1	0	0	0	0	3	Accessory	COG1670_J#	40.7	49	0.0000118				
8322	WP_122213922.1	-	hypothetical protein	1	0	1	1	1	1	1	1	1	1	1	10	Accessory								
8323	OSC58135.1	-	hypothetical protein	1	1	1	1	1	1	1	1	1	1	1	11	CORE								
8324	OSC72330.1	-	hypothetical protein	1	1	1	1	1	1	1	1	1	1	1	11	CORE					NA	50.3	171	9.38E-42
8325	AXI85328.1	-	regulator	1	0	0	1	0	0	0	0	0	0	0	2	Accessory								
8326	OSC66110.1	-	MarR family transcriptional regulator	0	1	0	0	0	0	0	0	0	0	0	1	Unique	COG1846_K#	37.6	89	1.34E-17	NA	28.8	63	2.11E-09
8327	Prokka:JAPCLKMA_06564	-	hypothetical protein	1	0	0	0	0	0	0	0	0	0	0	1	Unique								
8328	Prokka:CIODPCDJ_03719	-	hypothetical protein	1	1	1	1	1	1	1	1	1	1	1	11	CORE								
8329	OSC63649.1	-	Tat pathway signal sequence domain protein	0	1	0	0	0	0	0	0	0	0	0	1	Unique								

cluster	GI	gene	Annotation	Streptomyces_thermocarboxydus_BPSAC147	Streptomyces_sp._4F	Streptomyces_sp._BSE7F	Streptomyces_sp._ETH9427	Streptomyces_sp._FxanaD5	Streptomyces_sp._UNC401CLCol	Actinospica_acidiphila_NRRL_B_24431	Streptomyces_sp._HNS054	Streptomyces_sp._SMS_SU21	Streptomyces_sp._ZS0098	Streptomyces_thermocarboxydus_K155	Addition	Core-Accessory-Unique	COG	%Similarity_COG	Score_COG	Evalue_COG	KEGG	%Similarity_KEGG	Score_KEGG	Evalue_KEGG
8330	AXI88765.1	-	hypothetical protein	0	0	0	1	0	1	0	0	0	0	0	2	Accessory								
8331	OSC51709.1	-	hypothetical protein	0	1	0	0	0	0	0	0	0	0	0	1	Unique	COG2409_R#	38.5	121	3.19E-27	K11625	38.5	121	8.53E-27
8332	WP_159020841.1	-	hypothetical protein	0	0	0	0	0	0	0	1	0	0	0	1	Unique								
8333	AXI88492.1	-	hypothetical protein	0	0	0	1	0	0	0	0	0	0	0	1	Unique								
8334	WP_033275564.1	-	SigE family RNA polymerase sigma factor	1	0	0	1	1	1	1	1	1	1	1	9	Accessory	COG1595_K#	38.1	67	4.19E-11	K03088	32.1	64	1.24E-09
8335	WP_051724676.1	-	hypothetical protein	0	0	0	0	0	0	1	0	0	0	0	1	Unique								
8336	WP_167338236.1	-	ATP-binding protein	1	1	1	1	1	1	1	1	1	1	1	11	CORE	COG0642_T#	32.4	54	6.26E-07				
8337	PWE11424.1	-	tellurium resistance protein	0	1	1	0	1	1	0	0	0	0	0	4	Accessory	COG4110_R#	37.3	104	3.09E-22	K05792	37.3	104	8.26E-22
8338	OSC62228.1	-	pep a2	1	1	1	1	1	1	1	1	1	1	1	11	CORE								
8339	WP_048458618.1	-	hypothetical protein	0	0	0	1	0	0	0	1	0	0	0	2	Accessory								
8340	WP_048459277.1	-	hypothetical protein	0	0	0	1	0	0	0	1	1	0	0	3	Accessory								
8341	AXI85275.1	-	hypothetical protein	1	0	0	1	0	0	0	0	0	0	0	2	Accessory	COG2018_R#	32.8	62	1.35E-09	K07131	32.8	62	3.6E-09
8342	WP_020700056.1	-	hypothetical protein	0	0	0	0	1	0	0	0	0	0	0	1	Unique								
8343	Prokka:CIODPCDJ_02218	-	hypothetical protein	0	0	0	0	1	1	1	0	0	0	1	4	Accessory								
8344	Prokka:JAPCLKMA_01846	-	hypothetical protein	1	0	1	0	1	1	1	1	1	1	1	9	Accessory					NA	32.4	45	0.000455
8345	PWE11026.1	-	hypothetical protein	0	0	1	0	0	0	0	0	0	0	0	1	Unique	COG2801_L#	48.6	77	4.05E-14	NA	45.3	77	1.08E-13

cluster	GI	gene	Annotation	Streptomyces_thermocarboxydus_BPSAC147	Streptomyces_sp._4F	Streptomyces_sp._BSE7F	Streptomyces_sp._ETH9427	Streptomyces_sp._FxanaD5	Streptomyces_sp._UNC401CLCol	Actinospica_acidiphila_NRRL_B_24431	Streptomyces_sp._HNS054	Streptomyces_sp._SMS_SU21	Streptomyces_sp._ZS0098	Streptomyces_thermocarboxydus_K155	Addition	Core-Accessory-Unique	COG	%Similarity_COG	Score_COG	Evalue_COG	KEGG	%Similarity_KEGG	Score_KEGG	Evalue_KEGG
8346	OSC62623.1	-	shikimate kinase	1	1	1	1	1	1	1	1	1	1	1	11	CORE	COG0703_E#	52.1	155	1.99E-37	K00891	52.1	155	5.33E-37
8347	OSC57648.1	-	MarR family transcriptional regulator	1	1	1	1	1	1	1	1	1	1	1	11	CORE	COG1846_K#	49.1	144	2.68E-34	K15973	25.4	52	0.00000631
8348	WP_033276909.1	-	DinB family protein	1	0	1	1	1	1	1	1	1	1	1	10	Accessory								
8349	Prokka:CIODPCDJ_03299	-	hypothetical protein	1	1	1	1	1	1	1	1	1	1	1	11	CORE	COG1847_R#	62.1	184	3.99E-46	K06346	62.1	184	1.06E-45
8350	WP_086705158.1	-	MaoC family dehydratase	1	1	1	1	1	1	1	1	1	1	1	11	CORE	COG2030_I#	43.2	125	2.2E-28	NA	39.1	105	4.81E-22
8351	WP_019526966.1	rpsI	30S ribosomal protein S9	1	1	1	1	1	1	1	1	1	1	1	11	CORE	COG0103_J#	60.7	159	8.05E-39	K02996	60.7	159	2.15E-38
8352	OSC73129.1	-	hypothetical protein	0	1	0	0	0	0	0	0	0	0	0	1	Unique								
8353	WP_102639342.1	-	hypothetical protein	1	0	1	1	1	1	1	1	1	1	1	10	Accessory								
8354	AXI90247.1	msrA	peptide-methionine (S)-S-oxide reductase	1	1	1	1	1	1	1	1	1	1	1	11	CORE	COG0225_O#	78.8	300	4.99E-81	K07304	78.8	300	1.33E-80
8355	WP_033277365.1	-	YbaK/EbsC family protein	1	1	0	1	1	1	1	1	1	1	1	10	Accessory	COG2606_S#	42.5	124	3.75E-28	NA	37.2	78	6.3E-14
8356	PWE09262.1	-	hypothetical protein	1	1	1	1	1	1	1	1	1	1	1	11	CORE								
8357	WP_033274079.1	-	hypothetical protein	1	0	0	1	1	1	1	1	1	1	1	9	Accessory								
8358	WP_147474392.1	-	hypothetical protein	0	0	1	1	0	0	0	1	1	1	0	5	Accessory								

cluster	GI	gene	Annotation	Streptomyces_thermocarboxydus_BPSAC147	Streptomyces_sp._4F	Streptomyces_sp._BSE7F	Streptomyces_sp._ETH9427	Streptomyces_sp._FxanaD5	Streptomyces_sp._UNC401CLCol	Actinospica_acidiphila_NRRL_B_24431	Streptomyces_sp._HNS054	Streptomyces_sp._SMS_SU21	Streptomyces_sp._ZS0098	Streptomyces_thermocarboxydus_K155	Addition	Core-Accessory-Unique	COG	%Similarity_COG	Score_COG	Evalue_COG	KEGG	%Similarity_KEGG	Score_KEGG	Evalue_KEGG
8359	Prokka:CIODPCDJ_01141	-	hypothetical protein	1	1	1	1	1	1	1	1	1	1	1	11	CORE								
8360	WP_026243687.1	-	hypothetical protein	1	0	0	0	1	1	0	0	0	1	1	5	Accessory								
8361	OSC69952.1	-	hypothetical protein	0	1	0	0	0	0	0	0	0	0	0	1	Unique								
8362	WP_122216236.1	-	flavodoxin	0	0	0	1	0	0	0	1	1	1	0	4	Accessory								
8363	OSC71753.1	-	hydrolase	0	1	0	1	0	0	0	1	0	1	0	4	Accessory								
8364	WP_106963372.1	-	transposase	0	0	0	0	0	0	1	0	0	0	0	1	Unique								
8365	OSC61461.1	-	flavodoxin	0	1	0	1	0	0	0	1	1	1	0	5	Accessory								
8366	OSC57398.1	-	hypothetical protein	0	1	0	0	0	0	0	0	0	0	0	1	Unique								
8367	Prokka:JAPCLKMA_01576	-	hypothetical protein	1	0	0	0	0	0	0	0	0	0	0	1	Unique								
8368	AXI91370.1	-	hypothetical protein	0	0	0	1	0	0	0	0	0	0	0	1	Unique								
8369	WP_122215443.1	-	hypothetical protein	0	0	1	1	0	0	1	0	1	1	0	5	Accessory					NA	40	56	2.56E-07
8370	OSC72642.1	-	alkylhydroperoxidase	1	1	1	1	1	1	1	1	1	1	1	11	CORE	COG2128_S#	46.5	131	4.01E-30	NA	40.3	112	3.01E-24
8371	WP_048455375.1	-	Na(+)/H(+) antiporter subunit C	0	0	1	1	0	0	0	1	1	1	0	5	Accessory	COG1006_P#	41.9	113	6.62E-25	K05567	39.1	86	2.31E-16
8372	OSC63976.1	-	GNAT family N-acetyltransferase	0	1	0	0	0	0	0	0	0	0	0	1	Unique	COG3153_R#	35.9	87	6.63E-17	NA	35.3	77	1.4E-13
8373	OSC62472.1	-	alcohol dehydrogenase	0	1	0	0	0	0	0	0	0	0	0	1	Unique	COG1064_R#	38.2	112	1.47E-24	K13953	38.7	111	6.72E-24
8374	OSC57627.1	-	protein kinase	0	1	0	0	0	0	0	0	0	0	0	1	Unique								

cluster	GI	gene	Annotation	Streptomyces_thermocarboxydus_BPSAC147	Streptomyces_sp._4F	Streptomyces_sp._BSE7F	Streptomyces_sp._ETH9427	Streptomyces_sp._FxanaD5	Streptomyces_sp._UNC401CLCol	Actinospica_acidiphila_NRRL_B_24431	Streptomyces_sp._HNS054	Streptomyces_sp._SMS_SU21	Streptomyces_sp._ZS0098	Streptomyces_thermocarboxydus_K155	Addition	Core-Accessory-Unique	COG	%Similarity_COG	Score_COG	Evalue_COG	KEGG	%Similarity_KEGG	Score_KEGG	Evalue_KEGG
8375	Prokka:JAPCLKMA_02590	rsfS	Ribosomal silencing factor RsfS	1	1	1	1	1	1	1	1	1	1	1	11	CORE	COG0799_S#	53.9	137	5.59E-32	K09710	53.9	137	1.49E-31
8376	AXI85436.1	-	flavin reductase	1	1	1	1	1	1	1	1	1	1	1	11	CORE	COG1853_R#	36.8	96	1.09E-19	NA	36.8	96	2.9E-19
8377	WP_102638325.1	-	MarR family transcriptional regulator	1	1	1	1	1	1	1	1	1	1	1	11	CORE	COG1846_K#	49.7	141	2.26E-33	NA	49.7	141	6.02E-33
8378	WP_122216593.1	-	peptidoglycan-binding protein	0	0	1	0	0	0	0	0	1	1	0	3	Accessory								
8379	WP_028959543.1	-	phosphoribosyltransferase	1	1	1	1	1	1	1	1	1	1	1	11	CORE	COG2236_R#	64.6	208	1.5E-53	K07101	36.4	96	2.22E-19
8380	WP_122213812.1	-	sporulation protein SsgA	0	0	0	0	0	0	0	0	0	1	0	1	Unique								
8381	WP_102638377.1	-	DinB family protein	1	0	1	1	1	1	1	1	1	0	1	9	Accessory								
8382	OSC64387.1	-	hypothetical protein	0	1	0	0	0	0	0	0	0	0	0	1	Unique								
8383	WP_033273448.1	-	hypothetical protein	0	0	0	0	0	0	1	0	0	0	0	1	Unique								
8384	WP_102639571.1	-	SigE family RNA polymerase sigma factor	1	1	1	1	1	1	0	1	1	1	1	10	Accessory	COG1595_K#	35	62	2.27E-09	K03088	25.8	50	0.000014
8385	Prokka:CIODPCDJ_04107	-	hypothetical protein	1	1	1	1	1	1	1	1	1	1	1	11	CORE								
8386	WP_102637621.1	-	hypothetical protein	1	1	0	1	1	1	1	1	1	1	1	10	Accessory								
8387	OSC72938.1	-	hypothetical protein	0	1	0	0	0	0	0	0	0	0	0	1	Unique	COG3250_G#	38	99	1.28E-20				

cluster	GI	gene	Annotation	Streptomyces_thermocarboxydus_BPSAC147	Streptomyces_sp._4F	Streptomyces_sp._BSE7F	Streptomyces_sp._ETH9427	Streptomyces_sp._FxanaD5	Streptomyces_sp._UNC401CLCol	Actinospica_acidiphila_NRRL_B_24431	Streptomyces_sp._HNS054	Streptomyces_sp._SMS_SU21	Streptomyces_sp._ZS0098	Streptomyces_thermocarboxydus_K155	Addition	Core-Accessory-Unique	COG	%Similarity_COG	Score_COG	Evalue_COG	KEGG	%Similarity_KEGG	Score_KEGG	Evalue_KEGG
8388	OSC71065.1	-	hypothetical protein	0	1	0	0	0	0	0	0	0	0	0	1	Unique					NA	39.1	44	0.001
8389	Prokka:CIODPCDJ_06106	-	hypothetical protein	0	0	0	0	1	1	1	0	0	0	1	4	Accessory								
8390	AXI89608.1	-	topology modulation protein	0	0	0	1	0	0	0	0	0	0	0	1	Unique	COG0563_F#	37.8	130	5.2E-30	NA	25.1	84	1.49E-15
8391	Prokka:JAPCLKMA_04833	-	hypothetical protein	1	0	0	1	0	0	0	0	0	1	0	3	Accessory								
8392	WP_122215915.1	-	GNAT family N-acetyltransferase	1	0	0	0	1	1	0	0	0	1	1	5	Accessory	COG0454_KR#	48.7	145	2.04E-34	K00657	36.7	102	4.05E-21
8393	Prokka:CIODPCDJ_04806	coaD	Phosphopantetheine adenylyltransferase	1	1	1	1	1	1	1	1	1	1	1	11	CORE	COG0669_H#	54.5	175	1.84E-43	K00954	54.5	175	4.92E-43
8394	Prokka:JAPCLKMA_01582	lspA_2	Lipoprotein signal peptidase	1	0	0	0	0	0	0	0	0	0	0	1	Unique	COG0597_MU#	34	68	2.43E-11				
8395	OSC61897.1	-	hypothetical protein	0	1	0	0	0	0	0	0	0	0	0	1	Unique	COG0457_R#	52.1	146	9.16E-35	NA	52.1	146	2.45E-34
8396	OSC72103.1	-	phosphotransferase	0	1	0	0	0	0	0	0	0	0	0	1	Unique								
8397	AXI91246.1	-	hypothetical protein	0	0	0	1	0	0	0	0	0	0	0	1	Unique								
8398	AXI84839.1	-	DUF2199 domain-containing protein	0	0	0	1	0	0	0	0	0	0	0	1	Unique	COG4899_S#	35.3	74	5.78E-13				
8399	Prokka:JAPCLKMA_04913	-	hypothetical protein	1	0	0	0	0	0	0	0	0	0	0	1	Unique								
8400	WP_051724411.1	-	hypothetical protein	0	0	0	0	0	0	1	0	0	0	0	1	Unique								

cluster	GI	gene	Annotation	Streptomyces_thermocarboxydus_BPSAC147	Streptomyces_sp._4F	Streptomyces_sp._BSE7F	Streptomyces_sp._ETH9427	Streptomyces_sp._FxanaD5	Streptomyces_sp._UNC401CLCol	Actinospica_acidiphila_NRRL_B_24431	Streptomyces_sp._HNS054	Streptomyces_sp._SMS_SU21	Streptomyces_sp._ZS0098	Streptomyces_thermocarboxydus_K155	Addition	Core-Accessory-Unique	COG	%Similarity_COG	Score_COG	Evalue_COG	KEGG	%Similarity_KEGG	Score_KEGG	Evalue_KEGG
8401	Prokka:CIODPCDJ_03772	-	hypothetical protein	0	0	1	1	1	1	0	1	1	1	1	8	Accessory								
8402	OSC72085.1	-	hypothetical protein	0	1	0	0	0	0	0	0	0	0	0	1	Unique								
8403	WP_033277747.1	-	MarR family transcriptional regulator	1	0	1	1	1	1	1	1	1	1	1	10	Accessory	COG1846_K#	29.6	68	3.15E-11	NA	29.2	52	0.00000366
8404	WP_019527169.1	-	CGNR zinc finger domain-containing protein	1	1	1	1	1	1	1	1	1	1	1	11	CORE	COG5516_R#	34.9	67	5.37E-11				
8405	WP_048458037.1	rplQ	50S ribosomal protein L17	1	1	1	1	1	1	1	1	1	1	1	11	CORE	COG0203_J#	58.4	192	1.11E-48	K02879	58.4	192	2.96E-48
8406	Prokka:CIODPCDJ_00493	sigE_1	RNA polymerase sigma-E factor	1	0	1	1	1	1	1	1	1	1	1	10	Accessory	COG1595_K#	33.1	62	1.32E-09	K03088	31.5	55	5.64E-07
8407	OSC71317.1	-	hypothetical protein	1	1	0	1	1	1	1	1	1	1	1	10	Accessory								
8408	Prokka:JAPCLKMA_03606	-	hypothetical protein	1	1	1	1	1	1	1	1	1	1	1	11	CORE								
8409	Prokka:JAPCLKMA_04323	-	hypothetical protein	1	1	1	1	1	1	1	1	1	1	1	11	CORE					K01110	27.1	45	0.000763
8410	WP_026243410.1	ectA	diaminobutyrate acetyltransferase	1	0	1	1	1	1	1	1	1	1	1	10	Accessory	COG0454_KR#	38.4	119	1.56E-26				
8411	WP_048457085.1	-	hypothetical protein	0	0	0	1	0	0	0	1	0	1	0	3	Accessory								

cluster	GI	gene	Annotation	Streptomyces_thermocarboxydus_BPSAC147	Streptomyces_sp._4F	Streptomyces_sp._BSE7F	Streptomyces_sp._ETH9427	Streptomyces_sp._FxanaD5	Streptomyces_sp._UNC401CLCol	Actinospica_acidiphila_NRRL_B_24431	Streptomyces_sp._HNS054	Streptomyces_sp._SMS_SU21	Streptomyces_sp._ZS0098	Streptomyces_thermocarboxydus_K155	Addition	Core-Accessory-Unique	COG	%Similarity_COG	Score_COG	Evalue_COG	KEGG	%Similarity_KEGG	Score_KEGG	Evalue_KEGG
8412	WP_107080923.1	ruvX	Holliday junction resolvase RuvX	1	1	1	1	1	1	1	1	1	1	1	11	CORE	COG0816_L#	56.1	144	2.65E-34	K07447	55.6	138	8.65E-32
8413	Prokka:JAPCLKMA_01851	-	hypothetical protein	1	0	1	1	1	1	1	1	1	1	1	10	Accessory								
8414	WP_122213151.1	-	SRPBCC family protein	1	1	1	1	1	1	1	1	1	1	1	11	CORE								
8415	Prokka:JAPCLKMA_06345	-	hypothetical protein	1	1	1	1	1	1	1	1	1	1	1	11	CORE								
8416	AXI85271.1	-	ATP-binding protein	0	0	0	1	0	0	0	0	0	0	0	1	Unique								
8417	Prokka:JAPCLKMA_06510	-	hypothetical protein	1	0	0	0	0	0	0	0	0	0	0	1	Unique								
8418	WP_147474408.1	-	XdhC family protein	0	0	0	0	0	0	0	0	0	1	0	1	Unique	COG1975_O#	41.5	93	9.15E-19	K07402	41.5	93	2.44E-18
8419	OSC69424.1	-	hypothetical protein	1	1	1	1	1	1	1	1	1	1	1	11	CORE	COG4894_S#	41.5	140	5E-33				
8420	OSC69342.1	-	GNAT family N-acetyltransferase	1	1	1	1	1	1	1	1	1	1	1	11	CORE	COG0454_KR#	34.6	53	0.00000105	NA	34.6	53	0.0000028
8421	WP_033275339.1	-	hypothetical protein	0	1	0	0	0	0	1	0	0	0	0	2	Accessory								
8422	OSC64622.1	-	hypothetical protein	1	1	1	1	1	1	1	1	1	1	1	11	CORE								
8423	OSC72243.1	-	hypothetical protein	0	1	0	0	0	0	0	0	0	0	0	1	Unique	COG5472_S#	45.2	114	3.83E-25				
8424	OSC66038.1	-	hypothetical protein	1	1	1	1	1	1	1	1	1	1	1	11	CORE								
8425	WP_033274763.1	-	hypothetical protein	1	0	0	1	1	1	1	1	0	1	1	8	Accessory								

cluster	GI	gene	Annotation	Streptomyces_thermocarboxydus_BPSAC147	Streptomyces_sp._4F	Streptomyces_sp._BSE7F	Streptomyces_sp._ETH9427	Streptomyces_sp._FxanaD5	Streptomyces_sp._UNC401CLCol	Actinospica_acidiphila_NRRL_B_24431	Streptomyces_sp._HNS054	Streptomyces_sp._SMS_SU21	Streptomyces_sp._ZS0098	Streptomyces_thermocarboxydus_K155	Addition	Core-Accessory-Unique	COG	%Similarity_COG	Score_COG	Evalue_COG	KEGG	%Similarity_KEGG	Score_KEGG	Evalue_KEGG
8426	AXI91244.1	-	hypothetical protein	0	0	0	1	0	0	0	0	0	0	0	1	Unique								
8427	AXI91236.1	-	hypothetical protein	0	0	0	1	0	0	0	0	0	0	0	1	Unique								
8428	OSC69490.1	-	transcriptional regulator	1	1	1	1	1	1	1	1	1	1	1	11	CORE								
8429	WP_122215422.1	-	NUDIX hydrolase	1	1	0	1	1	1	1	1	1	1	1	10	Accessory	COG0494_LR#	44	50	0.00000883				
8430	OSC47862.1	-	MFS transporter	0	1	0	0	0	0	0	0	0	0	0	1	Unique	COG0477_GEPR#	32.1	91	4.51E-18	K03762	32.1	91	1.21E-17
8431	WP_033276635.1	-	lamin tail domain-containing protein	1	1	0	1	1	1	1	1	1	1	1	10	Accessory								
8432	WP_086020173.1	-	DUF3830 family protein	1	1	1	1	1	1	1	1	1	0	1	10	Accessory								
8433	WP_106432543.1	-	hypothetical protein	0	0	0	0	1	0	0	0	0	0	0	1	Unique								
8434	Prokka:CIODPCDJ_03378	-	hypothetical protein	1	1	1	1	1	1	1	1	1	1	1	11	CORE								
8435	AXI89660.1	-	hypothetical protein	0	0	0	1	0	0	0	0	0	0	0	1	Unique								
8436	Prokka:CIODPCDJ_00759	asnC_2	Regulatory protein AsnC	1	1	0	1	1	1	1	1	1	1	1	10	Accessory	COG1522_K#	50	142	1.71E-33	NA	50	142	4.56E-33
8437	WP_122212527.1	-	hypothetical protein	1	0	1	1	1	1	1	1	1	1	1	10	Accessory								
8438	WP_102639450.1	-	Hsp20/alpha crystallin family protein	1	0	0	0	1	1	0	0	1	1	1	6	Accessory	COG0071_O#	31.9	86	1.11E-16	K13993	28.8	75	4.01E-13
8439	OSC43649.1	-	hypothetical protein	0	1	0	0	0	0	0	0	0	0	0	1	Unique								

cluster	GI	gene	Annotation	Streptomyces_thermocarboxydus_BPSAC147	Streptomyces_sp._4F	Streptomyces_sp._BSE7F	Streptomyces_sp._ETH9427	Streptomyces_sp._FxanaD5	Streptomyces_sp._UNC401CLCol	Actinospica_acidiphila_NRRL_B_24431	Streptomyces_sp._HNS054	Streptomyces_sp._SMS_SU21	Streptomyces_sp._ZS0098	Streptomyces_thermocarboxydus_K155	Addition	Core-Accessory-Unique	COG	%Similarity_COG	Score_COG	Evalue_COG	KEGG	%Similarity_KEGG	Score_KEGG	Evalue_KEGG
8440	OSC65369.1	-	tRNA-specific adenosine deaminase	0	1	0	0	0	0	0	0	0	0	0	1	Unique	COG0590_FJ#	44.8	141	2.91E-33	NA	52.3	156	2.34E-37
8441	OSC72390.1	-	RNA polymerase subunit sigma-70	1	1	0	1	0	1	1	1	0	1	1	8	Accessory								
8442	WP_019522490.1	-	GNAT family N-acetyltransferase	1	1	1	1	1	1	1	1	1	1	1	11	CORE	COG0454_KR#	33.1	50	0.00000517	NA	45.3	44	0.00129
8443	WP_122213826.1	-	hypothetical protein	0	0	0	0	0	0	0	0	0	1	0	1	Unique								
8444	Prokka:JAPCLKMA_06697	-	hypothetical protein	1	1	1	1	1	1	1	1	1	1	1	11	CORE	COG1396_K#	45.7	75	2.56E-13	NA	45.7	75	6.84E-13
8445	WP_026244295.1	tsaE	tRNA (adenosine(37)-N6)-threonylcarbamoyltransferase complex ATPase subunit type 1 TsaE	1	1	1	1	1	1	1	1	1	1	1	11	CORE	COG0802_R#	48.5	118	2.02E-26	K06925	48.6	117	1.2E-25
8446	Prokka:JAPCLKMA_01605	-	hypothetical protein	1	0	0	0	0	0	0	0	0	0	0	1	Unique								
8447	Prokka:JAPCLKMA_01558	-	hypothetical protein	1	0	0	0	0	0	0	0	0	0	0	1	Unique								
8448	OSC69662.1	-	hypothetical protein	0	1	0	0	0	0	0	0	0	0	0	1	Unique								
8449	WP_033274199.1	-	hypothetical protein	0	0	0	0	0	0	1	0	0	0	0	1	Unique								

cluster	GI	gene	Annotation	Streptomyces_thermocarboxydus_BPSAC147	Streptomyces_sp._4F	Streptomyces_sp._BSE7F	Streptomyces_sp._ETH9427	Streptomyces_sp._FxanaD5	Streptomyces_sp._UNC401CLCol	Actinospica_acidiphila_NRRL_B_24431	Streptomyces_sp._HNS054	Streptomyces_sp._SMS_SU21	Streptomyces_sp._ZS0098	Streptomyces_thermocarboxydus_K155	Addition	Core-Accessory-Unique	COG	%Similarity_COG	Score_COG	Evalue_COG	KEGG	%Similarity_KEGG	Score_KEGG	Evalue_KEGG
8450	OSC71029.1	-	AsnC family transcriptional regulator	1	1	1	1	1	1	1	1	1	1	1	11	CORE	COG1522_K#	32.6	84	4.22E-16	NA	30.8	72	4.44E-12
8451	WP_084206987.1	-	helix-turn-helix transcriptional regulator	0	0	0	0	0	0	1	0	0	0	0	1	Unique	COG1733_K#	42.3	113	8.49E-25	NA	36.5	94	8.35E-19
8452	AXI84565.1	-	DUF1877 domain-containing protein	0	0	0	1	0	0	0	0	0	0	0	1	Unique								
8453	WP_084207012.1	-	ATP-binding protein	1	0	0	0	0	1	1	1	1	1	1	7	Accessory	COG3920_T#	45.2	44	0.000484				
8454	OSC62110.1	-	hypothetical protein	0	1	0	0	0	0	0	0	0	0	0	1	Unique								
8455	AXI91356.1	-	hypothetical protein	0	0	0	1	0	0	0	0	0	0	0	1	Unique								
8456	OSC72570.1	-	MarR family transcriptional regulator	1	1	1	1	1	1	1	1	1	1	1	11	CORE	COG1846_K#	28.9	57	5.53E-08	K03712	25.9	51	0.0000106
8457	Prokka:JAPCLKMA_05580	-	Inorganic triphosphatase	1	0	0	0	0	0	0	0	0	0	0	1	Unique	COG2954_S#	38.4	79	1.77E-14				
8458	WP_019523076.1	-	hypothetical protein	1	0	1	1	1	1	1	1	1	1	1	10	Accessory								
8459	WP_122216906.1	ybaK	Cys-tRNA(Pro) deacylase	1	1	1	1	1	1	1	1	1	1	1	11	CORE	COG2606_S#	61	171	2.61E-42	K03976	50	163	2.48E-39
8460	AXI89619.1	-	XRE family transcriptional regulator	0	0	0	1	0	0	0	0	0	0	0	1	Unique					NA	43.8	108	7.25E-23

cluster	GI	gene	Annotation	Streptomyces_thermocarboxydus_BPSAC147	Streptomyces_sp._4F	Streptomyces_sp._BSE7F	Streptomyces_sp._ETH9427	Streptomyces_sp._FxanaD5	Streptomyces_sp._UNC401CLCol	Actinospica_acidiphila_NRRL_B_24431	Streptomyces_sp._HNS054	Streptomyces_sp._SMS_SU21	Streptomyces_sp._ZS0098	Streptomyces_thermocarboxydus_K155	Addition	Core-Accessory-Unique	COG	%Similarity_COG	Score_COG	Evalue_COG	KEGG	%Similarity_KEGG	Score_KEGG	Evalue_KEGG
8461	Prokka:JAPCLKMA_00368	-	Alkaline shock protein 23	1	1	1	1	1	1	1	1	1	1	1	11	CORE	COG1302_S#	41.7	92	1.18E-18	NA	41.7	86	2.26E-16
8462	WP_122216400.1	-	metallophosphoesterase	1	1	1	1	1	1	1	1	1	1	1	11	CORE	COG0622_R#	60.7	206	9.57E-53	K07095	34.2	79	4.71E-14
8463	WP_048459359.1	-	DUF3515 domain-containing protein	1	0	1	1	1	1	1	1	1	1	1	10	Accessory								
8464	WP_167338178.1	-	GNAT family N-acetyltransferase	1	1	1	1	1	1	1	1	1	1	1	11	CORE	COG0454_KR#	29.2	79	1.35E-14	NA	29.2	79	3.61E-14
8465	WP_019521349.1	-	nucleotidase	1	0	1	1	1	1	1	1	1	1	1	10	Accessory								
8466	WP_157842165.1	-	hypothetical protein	0	0	0	1	0	0	1	1	0	1	0	4	Accessory								
8467	Prokka:JAPCLKMA_05443	nudC_3	NADH pyrophosphatase	1	1	1	1	1	1	1	1	1	1	1	11	CORE	COG0494_LR#	33.7	82	2.08E-15	K03574	33.7	82	5.56E-15
8468	Prokka:JAPCLKMA_04198	-	hypothetical protein	1	1	1	1	1	1	1	1	1	1	1	11	CORE								
8469	PWE11373.1	-	hypothetical protein	1	0	1	1	1	1	0	0	1	1	1	8	Accessory								
8470	Prokka:JAPCLKMA_05861	-	hypothetical protein	1	1	1	1	1	1	1	1	1	1	1	11	CORE								
8471	Prokka:JAPCLKMA_01814	-	hypothetical protein	1	0	0	0	0	1	1	0	0	0	1	4	Accessory								
8472	WP_026243333.1	-	hemerythrin domain-containing protein	1	0	0	0	1	0	0	0	0	0	1	3	Accessory								
8473	PWE10990.1	-	hypothetical protein	0	0	1	1	0	0	0	1	1	0	0	4	Accessory								

cluster	GI	gene	Annotation	Streptomyces_thermocarboxydus_BPSAC147	Streptomyces_sp._4F	Streptomyces_sp._BSE7F	Streptomyces_sp._ETH9427	Streptomyces_sp._FxanaD5	Streptomyces_sp._UNC401CLCol	Actinospica_acidiphila_NRRL_B_24431	Streptomyces_sp._HNS054	Streptomyces_sp._SMS_SU21	Streptomyces_sp._ZS0098	Streptomyces_thermocarboxydus_K155	Addition	Core-Accessory-Unique	COG	%Similarity_COG	Score_COG	Evalue_COG	KEGG	%Similarity_KEGG	Score_KEGG	Evalue_KEGG
8474	WP_048460257.1	-	hypothetical protein	0	0	0	0	0	0	0	1	0	0	0	1	Unique								
8475	WP_033273642.1	-	hypothetical protein	0	0	0	0	0	0	1	0	0	0	0	1	Unique								
8476	OSC64404.1	-	antibiotic biosynthesis monooxygenase	0	1	0	0	0	0	0	0	0	0	0	1	Unique								
8477	Prokka:JAPCLKMA_05675	-	hypothetical protein	1	0	0	0	0	0	0	0	0	0	0	1	Unique								
8478	WP_033273660.1	-	DinB family protein	0	1	0	0	0	0	1	0	0	0	0	2	Accessory								
8479	OSC64579.1	-	hypothetical protein	1	1	1	1	1	1	1	1	1	1	1	11	CORE								
8480	WP_122214775.1	-	cupin domain-containing protein	0	0	0	0	0	0	0	0	0	1	0	1	Unique	COG0662_G#	38.5	54	4.65E-07	NA	25.5	47	0.000152
8481	OSC63249.1	-	hypothetical protein	1	1	1	1	1	1	1	1	1	1	1	11	CORE	COG2847_S#	32.6	63	7.67E-10				
8482	Prokka:JAPCLKMA_06357	-	hypothetical protein	1	0	1	1	1	1	1	1	1	1	1	10	Accessory								
8483	WP_102641043.1	-	hypothetical protein	1	1	1	1	1	1	1	1	1	1	1	11	CORE								
8484	OSC52483.1	-	amidase	0	1	0	0	0	0	0	0	0	0	0	1	Unique	COG0154_J#	41.7	107	3.55E-23	K01426	41.7	107	9.47E-23
8485	OSC61305.1	-	hypothetical protein	1	1	1	1	1	1	1	1	1	1	1	11	CORE								
8486	OSC69934.1	-	hypothetical protein	0	1	0	0	0	0	0	0	0	0	0	1	Unique								
8487	OSC68524.1	-	very short patch repair endonuclease	0	1	0	0	0	0	0	1	1	0	1	4	Accessory	COG3727_L#	39	98	2.15E-20				

cluster	GI	gene	Annotation	Streptomyces_thermocarboxydus_BPSAC147	Streptomyces_sp._4F	Streptomyces_sp._BSE7F	Streptomyces_sp._ETH9427	Streptomyces_sp._FxanaD5	Streptomyces_sp._UNC401CLCol	Actinospica_acidiphila_NRRL_B_24431	Streptomyces_sp._HNS054	Streptomyces_sp._SMS_SU21	Streptomyces_sp._ZS0098	Streptomyces_thermocarboxydus_K155	Addition	Core-Accessory-Unique	COG	%Similarity_COG	Score_COG	Evalue_COG	KEGG	%Similarity_KEGG	Score_KEGG	Evalue_KEGG
8488	OSC50630.1	-	ABC transporter substrate-binding protein	0	1	0	0	0	0	0	0	0	0	0	1	Unique	COG0747_E#	41.3	101	4.33E-21				
8489	Prokka:CIODPCDJ_04030	-	hypothetical protein	0	0	0	0	0	0	0	0	0	0	1	1	Unique								
8490	OSC72316.1	-	serine/threonine protein kinase	0	1	0	0	0	0	0	0	0	0	0	1	Unique	COG3291_R#	33.7	58	2.47E-08	NA	30.5	60	1.73E-08
8491	WP_106963390.1	-	ATP-binding protein	1	1	1	1	1	1	1	1	1	1	1	11	CORE								
8492	OSC64402.1	-	hypothetical protein	0	1	0	0	0	0	0	0	0	0	0	1	Unique								
8493	WP_019524630.1	-	DUF2975 domain-containing protein	1	1	1	1	1	1	1	1	1	1	1	11	CORE								
8494	PWE06650.1	-	GNAT family N-acetyltransferase	1	1	1	1	1	1	1	1	1	1	1	11	CORE	COG0454_KR#	37.8	96	1.06E-19				
8495	WP_122216234.1	-	hypothetical protein	0	0	0	1	0	0	0	0	0	1	0	2	Accessory								
8496	AXI84605.1	-	transmembrane transport protein	0	0	1	1	0	0	0	1	0	1	0	4	Accessory								
8497	PWE07446.1	-	rRNA maturation RNase YbeY	1	1	1	1	1	1	1	1	1	1	1	11	CORE	COG0319_R#	55.2	179	9.53E-45	K07042	55.2	179	2.55E-44
8498	OSC72423.1	-	cation-binding protein	1	1	1	1	1	1	1	1	1	1	1	11	CORE								

cluster	GI	gene	Annotation	Streptomyces_thermocarboxydus_BPSAC147	Streptomyces_sp._4F	Streptomyces_sp._BSE7F	Streptomyces_sp._ETH9427	Streptomyces_sp._FxanaD5	Streptomyces_sp._UNC401CLCol	Actinospica_acidiphila_NRRL_B_24431	Streptomyces_sp._HNS054	Streptomyces_sp._SMS_SU21	Streptomyces_sp._ZS0098	Streptomyces_thermocarboxydus_K155	Addition	Core-Accessory-Unique	COG	%Similarity_COG	Score_COG	Evalue_COG	KEGG	%Similarity_KEGG	Score_KEGG	Evalue_KEGG
8499	WP_019522616.1	-	MarR family transcriptional regulator	1	1	0	1	1	1	1	1	1	1	1	10	Accessory	COG1846_K#	51.6	159	7.82E-39	K03712	36.8	56	2.49E-07
8500	WP_051724480.1	-	DUF1360 domain-containing protein	0	0	0	0	0	0	1	0	0	0	0	1	Unique								
8501	Prokka:CIODPCDJ_01997	-	hypothetical protein	1	1	1	1	1	1	1	1	1	1	1	11	CORE	COG0454_KR#	40.2	48	0.0000331	NA	40.2	48	0.0000885
8502	Prokka:JAPCLKMA_04664	-	hypothetical protein	1	0	0	0	0	1	0	0	0	0	1	3	Accessory								
8503	WP_086700321.1	-	hypothetical protein	1	1	1	1	1	1	1	1	1	1	1	11	CORE								
8504	Prokka:CIODPCDJ_04238	greA	Transcription elongation factor GreA	1	1	1	1	1	1	1	1	1	1	1	11	CORE	COG0782_K#	54.5	175	1.8E-43	K03624	54.5	175	4.8E-43
8505	Prokka:JAPCLKMA_03100	-	hypothetical protein	1	1	1	1	1	1	1	1	1	1	1	11	CORE	COG1846_K#	36.2	67	6.89E-11	NA	36.4	75	6.76E-13
8506	OSC66766.1	-	hypothetical protein	0	1	0	0	0	0	0	0	0	0	0	1	Unique								
8507	Prokka:JAPCLKMA_01645	trmO	tRNA (adenine(37)-N6)-methyltransferase	1	0	0	1	0	0	0	0	0	0	0	2	Accessory	COG1720_S#	39.7	114	4.92E-25	NA	33.6	68	8.26E-11
8508	Prokka:JAPCLKMA_01643	-	hypothetical protein	1	0	0	1	0	0	0	0	0	0	0	2	Accessory								
8509	OSC50261.1	-	hypothetical protein	0	1	0	0	0	0	0	0	0	0	0	1	Unique								
8510	WP_122213042.1	-	hypothetical protein	1	1	1	1	1	1	1	1	1	1	1	11	CORE								

cluster	GI	gene	Annotation	Streptomyces_thermocarboxydus_BPSAC147	Streptomyces_sp._4F	Streptomyces_sp._BSE7F	Streptomyces_sp._ETH9427	Streptomyces_sp._FxanaD5	Streptomyces_sp._UNC401CLCol	Actinospica_acidiphila_NRRL_B_24431	Streptomyces_sp._HNS054	Streptomyces_sp._SMS_SU21	Streptomyces_sp._ZS0098	Streptomyces_thermocarboxydus_K155	Addition	Core-Accessory-Unique	COG	%Similarity_COG	Score_COG	Evalue_COG	KEGG	%Similarity_KEGG	Score_KEGG	Evalue_KEGG
8511	OSC62453.1	-	growth inhibitor PemK	1	1	1	1	1	1	1	1	1	1	1	11	CORE					NA	43.2	119	3.13E-26
8512	WP_028960320.1	-	hypothetical protein	0	0	0	0	1	1	0	0	0	0	1	3	Accessory								
8513	OSC62338.1	-	peptidylprolyl isomerase	0	1	0	0	0	0	0	0	0	0	0	1	Unique	COG0652_O#	72.8	248	2.18E-65	K01802	72.8	248	5.83E-65
8514	OSC56576.1	-	hypothetical protein	0	1	0	0	0	0	0	0	0	0	0	1	Unique								
8515	OSC71583.1	-	hypothetical protein	0	1	0	0	0	0	0	0	0	0	0	1	Unique								
8516	OSC71564.1	-	hypothetical protein	0	1	0	0	0	0	0	0	0	0	0	1	Unique								
8517	WP_019527717.1	-	hypothetical protein	0	0	0	0	1	0	0	0	0	0	0	1	Unique								
8518	OSC62937.1	-	hypothetical protein	0	1	0	0	0	0	0	0	0	0	0	1	Unique								
8519	OSC48189.1	-	aldo/keto reductase	0	1	0	0	0	0	0	0	0	0	0	1	Unique	COG0667_C#	35.6	85	2.45E-16	K15303	38.1	96	2.83E-19
8520	Prokka:JAPCLKMA_04226	-	hypothetical protein	1	0	0	0	1	1	0	0	0	1	1	5	Accessory								
8521	WP_122213548.1	-	MarR family transcriptional regulator	1	1	1	1	1	1	1	1	1	1	1	11	CORE	COG1846_K#	27.4	54	0.00000046	NA	26.4	50	0.0000136
8522	WP_102637279.1	-	carboxymuconolactone decarboxylase family protein	0	0	0	1	0	0	0	1	1	1	0	4	Accessory	COG2128_S#	41.9	115	2.19E-25	NA	40.7	79	3.56E-14
8523	AXI87014.1	-	MarR family transcriptional regulator	1	1	1	1	1	1	1	1	1	1	1	11	CORE	COG1846_K#	54.6	135	1.57E-31	NA	46.3	124	9.66E-28

cluster	GI	gene	Annotation	Streptomyces_thermocarboxydus_BPSAC147	Streptomyces_sp._4F	Streptomyces_sp._BSE7F	Streptomyces_sp._ETH9427	Streptomyces_sp._FxanaD5	Streptomyces_sp._UNC401CLCol	Actinospica_acidiphila_NRRL_B_24431	Streptomyces_sp._HNS054	Streptomyces_sp._SMS_SU21	Streptomyces_sp._ZS0098	Streptomyces_thermocarboxydus_K155	Addition	Core-Accessory-Unique	COG	%Similarity_COG	Score_COG	Evalue_COG	KEGG	%Similarity_KEGG	Score_KEGG	Evalue_KEGG
8524	WP_033273346.1	-	nucleoside deaminase	1	1	1	1	1	1	1	1	1	1	1	11	CORE	COG0590_FJ#	63.1	162	1.2E-39	K11991	63.1	162	3.2E-39
8525	AXI91382.1	-	hypothetical protein	0	0	0	1	0	0	0	0	0	0	0	1	Unique								
8526	WP_019523957.1	-	2-C-methyl-D-erythritol 2,4-cyclodiphosphate synthase	1	1	1	1	1	1	1	1	1	1	1	11	CORE	COG0245_I#	55.5	176	1.05E-43	K01770	55.5	176	2.8E-43
8527	PWE07337.1	-	DUF3145 domain-containing protein	1	1	1	1	1	1	1	1	1	1	1	11	CORE					NA	43.6	151	7.38E-36
8528	OSC57725.1	-	protein tyrosine phosphatase	1	1	1	1	1	1	1	1	1	1	1	11	CORE	COG0394_T#	46.8	131	3.86E-30	K01104	46.8	131	1.03E-29
8529	AXI91381.1	-	hypothetical protein	0	0	0	1	0	0	0	0	0	0	0	1	Unique								
8530	WP_048459431.1	-	hypothetical protein	0	1	0	1	0	0	1	1	1	1	0	6	Accessory								
8531	Prokka:JAPCLKMA_06317	-	hypothetical protein	1	0	0	0	0	0	0	0	0	0	0	1	Unique								
8532	WP_019525947.1	-	hypothetical protein	1	1	1	1	1	1	1	1	1	1	1	11	CORE								
8533	WP_048455894.1	-	hypothetical protein	0	0	0	0	0	0	0	1	0	0	0	1	Unique								
8534	Prokka:CIODPCDJ_00258	rppH_1	RNA pyrophosphohydrolase	1	1	1	1	1	1	1	1	1	1	1	11	CORE	COG1051_F#	39.4	77	5.07E-14	K03574	33.8	60	2.24E-08
8535	PWE07643.1	-	hypothetical protein	0	0	1	0	0	0	0	0	1	0	0	2	Accessory	COG4278_S#	29	50	0.00000867				

cluster	GI	gene	Annotation	Streptomyces_thermocarboxydus_BPSAC147	Streptomyces_sp._4F	Streptomyces_sp._BSE7F	Streptomyces_sp._ETH9427	Streptomyces_sp._FxanaD5	Streptomyces_sp._UNC401CLCol	Actinospica_acidiphila_NRRL_B_24431	Streptomyces_sp._HNS054	Streptomyces_sp._SMS_SU21	Streptomyces_sp._ZS0098	Streptomyces_thermocarboxydus_K155	Addition	Core-Accessory-Unique	COG	%Similarity_COG	Score_COG	Evalue_COG	KEGG	%Similarity_KEGG	Score_KEGG	Evalue_KEGG
8536	PWE06920.1	-	GNAT family N-acetyltransferase	1	1	1	1	1	1	1	1	1	1	1	11	CORE	COG1670_J#	32.7	69	1.38E-11	NA	30.6	62	4.51E-09
8537	OSC57674.1	-	MarR family transcriptional regulator	0	1	0	0	0	0	0	0	0	0	0	1	Unique	COG1846_K#	36.4	82	2.06E-15	NA	31.1	58	8.5E-08
8538	Prokka:CIODPCDJ_06728	-	hypothetical protein	0	0	0	0	0	0	0	0	0	0	1	1	Unique								
8539	OSC54721.1	-	amidohydrolase	0	1	0	0	0	0	0	0	0	0	0	1	Unique	COG1574_R#	31.7	59	1.87E-08	K07047	31	57	1.45E-07
8540	OSC61417.1	-	hypothetical protein	0	1	0	0	0	0	0	0	0	0	0	1	Unique	COG0399_M#	29.2	70	6.2E-12	K19430	28.2	49	0.0000516
8541	WP_102638086.1	-	hypothetical protein	0	0	0	0	0	0	0	0	1	0	0	1	Unique								
8542	WP_033274263.1	-	hypothetical protein	0	0	0	1	0	0	1	1	0	1	0	4	Accessory								
8543	WP_051724667.1	-	hypothetical protein	0	0	0	0	0	0	1	0	0	0	0	1	Unique								
8544	WP_033275077.1	yajC	preprotein translocase subunit YajC	0	1	1	1	1	1	1	1	1	1	1	10	Accessory	COG1862_U#	37.5	56	9.26E-08	K03210	34.1	56	3.23E-07
8545	OSC57243.1	-	hypothetical protein	1	1	1	1	1	1	1	1	1	1	1	11	CORE								
8546	WP_033276976.1	-	hypothetical protein	0	0	0	0	0	0	1	0	0	0	0	1	Unique								
8547	OSC68915.1	-	nitrate reductase molybdenum cofactor assembly chaperone	0	1	0	0	0	0	0	0	0	0	0	1	Unique	COG2180_C#	38.8	111	2.43E-24	K00373	38.8	111	6.48E-24
8548	OSC69548.1	-	hypothetical protein	0	1	0	0	0	0	0	0	0	0	0	1	Unique								

cluster	GI	gene	Annotation	Streptomyces_thermocarboxydus_BPSAC147	Streptomyces_sp._4F	Streptomyces_sp._BSE7F	Streptomyces_sp._ETH9427	Streptomyces_sp._FxanaD5	Streptomyces_sp._UNC401CLCol	Actinospica_acidiphila_NRRL_B_24431	Streptomyces_sp._HNS054	Streptomyces_sp._SMS_SU21	Streptomyces_sp._ZS0098	Streptomyces_thermocarboxydus_K155	Addition	Core-Accessory-Unique	COG	%Similarity_COG	Score_COG	Evalue_COG	KEGG	%Similarity_KEGG	Score_KEGG	Evalue_KEGG
8549	WP_122214782.1	-	hypothetical protein	0	0	1	1	0	0	1	1	1	1	0	6	Accessory								
8550	OSC60970.1	-	hypothetical protein	0	1	1	1	1	0	1	1	0	1	0	7	Accessory								
8551	WP_102640306.1	-	hypothetical protein	0	0	1	1	0	0	1	1	1	1	0	6	Accessory								
8552	PWE10530.1	-	hypothetical protein	0	0	1	0	0	0	0	0	1	0	0	2	Accessory	COG1309_K#	52.1	50	0.00000505	NA	52.1	50	0.0000135
8553	PWE10514.1	-	alkylhydroperoxidase	1	1	1	1	1	1	1	1	1	1	1	11	CORE	COG2128_S#	50.8	124	2.75E-28	NA	33.1	92	5.28E-18
8554	Prokka:JAPCLKMA_06485	slyA_3	Transcriptional regulator SlyA	1	0	0	0	1	1	1	0	0	0	1	5	Accessory	COG1846_K#	35.2	67	5.21E-11				
8555	PWE08585.1	-	mobilization protein	0	0	1	0	0	0	0	0	0	0	0	1	Unique								
8556	PWE11255.1	-	inorganic diphosphatase	1	1	1	1	1	1	1	1	1	1	1	11	CORE	COG0221_C#	66.9	233	7.18E-61	K01507	66.9	233	1.92E-60
8557	Prokka:JAPCLKMA_03447	-	hypothetical protein	1	1	1	1	1	1	1	1	1	1	1	11	CORE	COG4702_S#	46.6	132	1.01E-30				
8558	WP_019523648.1	-	RNA polymerase sigma factor	1	1	1	1	1	1	1	1	1	1	1	11	CORE	COG1595_K#	27.3	61	2.86E-09				
8559	WP_048457401.1	-	cupin domain-containing protein	0	0	1	0	0	0	0	1	1	0	0	3	Accessory								
8560	WP_026243649.1	-	DUF2243 domain-containing protein	1	0	1	1	1	1	1	1	1	1	1	10	Accessory	COG4329_S#	44.6	148	2.32E-35				

cluster	GI	gene	Annotation	Streptomyces_thermocarboxydus_BPSAC147	Streptomyces_sp._4F	Streptomyces_sp._BSE7F	Streptomyces_sp._ETH9427	Streptomyces_sp._FxanaD5	Streptomyces_sp._UNC401CLCol	Actinospica_acidiphila_NRRL_B_24431	Streptomyces_sp._HNS054	Streptomyces_sp._SMS_SU21	Streptomyces_sp._ZS0098	Streptomyces_thermocarboxydus_K155	Addition	Core-Accessory-Unique	COG	%Similarity_COG	Score_COG	Evalue_COG	KEGG	%Similarity_KEGG	Score_KEGG	Evalue_KEGG
8561	WP_048459461.1	-	Lrp/AsnC family transcriptional regulator	1	1	0	1	1	1	1	1	1	1	1	10	Accessory	COG1522_K#	48.3	148	3.04E-35	NA	40.4	107	1.21E-22
8562	OSC69870.1	-	hypothetical protein	0	1	0	0	0	0	0	0	0	0	0	1	Unique								
8563	WP_109288796.1	-	Ig domain-containing protein	0	0	0	0	0	0	0	0	1	0	0	1	Unique	COG5492_N#	98.2	312	1.22E-84	NA	34.5	46	0.000332
8564	Prokka:JAPCLKMA_03588	-	hypothetical protein	1	1	1	1	1	1	1	1	1	1	1	11	CORE								
8565	OSC61199.1	-	hypothetical protein	0	1	0	0	0	0	0	0	0	0	0	1	Unique					NA	37.3	72	3.31E-12
8566	WP_019522636.1	-	DUF485 domain-containing protein	1	1	1	1	1	1	0	1	1	1	1	10	Accessory					NA	38.6	50	0.000023
8567	Prokka:CIODPCDJ_06351	-	hypothetical protein	1	0	0	0	1	1	1	0	0	0	1	5	Accessory								
8568	Prokka:JAPCLKMA_02009	phzB2	Phenazine biosynthesis protein PhzB2	1	0	0	0	0	0	0	0	0	0	0	1	Unique								
8569	Prokka:JAPCLKMA_01895	lrpC_2	HTH-type transcriptional regulator LrpC	1	1	1	1	1	1	1	1	1	1	1	11	CORE	COG1522_K#	43.5	124	2.75E-28	K03719	45.1	99	4.32E-20
8570	WP_037953051.1	-	hypothetical protein	0	0	0	0	0	1	0	0	0	0	0	1	Unique								
8571	WP_159020943.1	-	hypothetical protein	0	0	0	0	0	0	0	1	0	0	0	1	Unique								
8572	OSC55804.1	-	hypothetical protein	1	1	1	1	1	1	1	1	1	1	1	11	CORE								

cluster	GI	gene	Annotation	Streptomyces_thermocarboxydus_BPSAC147	Streptomyces_sp._4F	Streptomyces_sp._BSE7F	Streptomyces_sp._ETH9427	Streptomyces_sp._FxanaD5	Streptomyces_sp._UNC401CLCol	Actinospica_acidiphila_NRRL_B_24431	Streptomyces_sp._HNS054	Streptomyces_sp._SMS_SU21	Streptomyces_sp._ZS0098	Streptomyces_thermocarboxydus_K155	Addition	Core-Accessory-Unique	COG	%Similarity_COG	Score_COG	Evalue_COG	KEGG	%Similarity_KEGG	Score_KEGG	Evalue_KEGG
8573	PWE10853.1	-	cytidine deaminase	1	1	1	1	1	1	1	1	1	0	1	10	Accessory					K01489	26.9	47	0.000195
8574	OSC72418.1	-	GNAT family N-acetyltransferase	0	1	0	0	0	0	0	0	0	0	0	1	Unique								
8575	OSC62656.1	-	hypothetical protein	1	1	1	1	1	1	1	1	1	1	1	11	CORE								
8576	Prokka:CIODPCDJ_03836	-	hypothetical protein	0	0	0	0	0	0	0	0	1	0	1	2	Accessory								
8577	AXI87106.1	-	DoxX family protein	0	0	1	1	0	0	0	1	1	1	0	5	Accessory								
8578	AXI89596.1	-	hypothetical protein	0	0	0	1	0	0	0	0	0	0	0	1	Unique					NA	32.1	43	0.00215
8579	OSC62615.1	-	cupin	1	1	0	1	1	1	1	1	1	1	1	10	Accessory	COG3542_S#	40.3	107	4.55E-23	K09705	40.3	107	1.21E-22
8580	OSC71299.1	-	hypothetical protein	1	1	1	1	1	1	1	1	1	1	1	11	CORE					NA	40	78	6.04E-14
8581	WP_033275980.1	-	hypothetical protein	0	0	0	0	0	0	1	0	0	0	0	1	Unique								
8582	OSC56038.1	-	zf-CGNR multi-domain protein	0	1	0	0	0	0	0	0	0	0	0	1	Unique								
8583	AXI88177.1	-	YajQ family cyclic di-GMP-binding protein	1	1	1	1	1	1	1	1	1	1	1	11	CORE	COG1666_S#	65.6	219	8.16E-57	K09767	65.6	219	2.18E-56
8584	WP_048458889.1	-	hypothetical protein	0	0	0	1	0	0	0	1	0	0	0	2	Accessory								
8585	WP_086703670.1	-	phage holin family protein	1	1	1	1	1	1	1	1	1	1	1	11	CORE								

cluster	GI	gene	Annotation	Streptomyces_thermocarboxydus_BPSAC147	Streptomyces_sp._4F	Streptomyces_sp._BSE7F	Streptomyces_sp._ETH9427	Streptomyces_sp._FxanaD5	Streptomyces_sp._UNC401CLCol	Actinospica_acidiphila_NRRL_B_24431	Streptomyces_sp._HNS054	Streptomyces_sp._SMS_SU21	Streptomyces_sp._ZS0098	Streptomyces_thermocarboxydus_K155	Addition	Core-Accessory-Unique	COG	%Similarity_COG	Score_COG	Evalue_COG	KEGG	%Similarity_KEGG	Score_KEGG	Evalue_KEGG
8586	AXI89631.1	-	hypothetical protein	0	0	0	1	0	0	0	0	0	0	0	1	Unique								
8587	WP_019524687.1	-	MarR family transcriptional regulator	1	1	1	1	1	1	1	1	1	1	1	11	CORE	COG1846_K#	36.2	74	3.25E-13	NA	36.2	74	8.67E-13
8588	WP_019525943.1	-	two pore domain potassium channel family protein	1	1	1	1	1	1	1	1	1	1	1	11	CORE	COG1226_P#	35.8	54	5.93E-07	K10716	28.5	51	0.0000103
8589	WP_019524673.1	-	hypothetical protein	1	0	1	1	1	1	1	1	1	1	1	10	Accessory								
8590	Prokka:JAPCLKMA_03141	-	hypothetical protein	1	0	1	1	1	1	1	1	1	1	1	10	Accessory	COG2764_S#	43.4	124	3.57E-28	K04750	40.6	100	1.48E-20
8591	PWE10170.1	-	cysteine methyltransferase	1	0	1	1	1	1	0	1	1	1	1	9	Accessory	COG0350_L#	55.1	174	3.01E-43	K00567	55.1	174	8.04E-43
8592	Prokka:JAPCLKMA_01554	-	hypothetical protein	1	0	0	1	0	0	0	0	0	0	0	2	Accessory								
8593	OSC70617.1	-	hypothetical protein	0	1	0	0	0	0	0	0	0	0	0	1	Unique								
8594	WP_019525443.1	-	hypothetical protein	0	0	0	0	1	0	0	0	0	0	1	2	Accessory								
8595	WP_078504445.1	-	N-acetyltransferase	1	1	1	0	1	1	1	1	1	1	1	10	Accessory	COG2388_R#	35.4	41	0.00305	K06975	41.7	53	0.00000207
8596	OSC62924.1	-	thioesterase	1	1	1	1	1	1	1	1	1	1	1	11	CORE	COG2050_Q#	50	109	1.19E-23	NA	50	109	3.18E-23
8597	WP_028959398.1	-	DUF4265 domain-containing protein	0	0	0	0	0	1	0	0	0	0	0	1	Unique								
8598	OSC70875.1	-	baseplate protein	0	1	0	1	0	0	1	1	1	1	0	6	Accessory	COG3628_R#	47.2	117	5.7E-26				

cluster	GI	gene	Annotation	Streptomyces_thermocarboxydus_BPSAC147	Streptomyces_sp._4F	Streptomyces_sp._BSE7F	Streptomyces_sp._ETH9427	Streptomyces_sp._FxanaD5	Streptomyces_sp._UNC401CLCol	Actinospica_acidiphila_NRRL_B_24431	Streptomyces_sp._HNS054	Streptomyces_sp._SMS_SU21	Streptomyces_sp._ZS0098	Streptomyces_thermocarboxydus_K155	Addition	Core-Accessory-Unique	COG	%Similarity_COG	Score_COG	Evalue_COG	KEGG	%Similarity_KEGG	Score_KEGG	Evalue_KEGG
8599	WP_033277139.1	-	MarR family transcriptional regulator	1	0	1	1	1	1	1	1	1	1	1	10	Accessory	COG1846_K#	37.4	62	1.28E-09	NA	37.4	62	3.41E-09
8600	WP_122214999.1	-	hypothetical protein	0	0	0	0	0	0	0	0	1	1	1	3	Accessory					NA	40.4	99	3.29E-20
8601	OSC57881.1	-	hypothetical protein	0	1	0	0	0	0	0	0	0	0	0	1	Unique								
8602	OSC67908.1	-	transposase	1	1	0	0	0	0	0	0	0	0	0	2	Accessory	COG3293_L#	53.5	144	4.36E-34				
8603	Prokka:JAPCLKMA_01057	-	hypothetical protein	1	1	1	1	1	1	1	1	1	1	1	11	CORE								
8604	AXI91355.1	-	hypothetical protein	0	0	0	1	0	0	0	0	0	0	0	1	Unique								
8605	Prokka:CIODPCDJ_03583	khtT_2	K(+)/H(+) antiporter subunit KhtT	1	1	1	1	1	1	1	1	1	1	1	11	CORE	COG0490_P#	45.7	119	8.8E-27	K07228	45.7	119	2.35E-26
8606	WP_033276436.1	-	VOC family protein	1	1	1	1	1	1	1	1	1	1	1	11	CORE	COG3865_S#	55.4	179	7.17E-45	NA	41.3	119	2.35E-26
8607	AXI85312.1	-	hypothetical protein	1	0	0	1	0	0	0	0	0	0	0	2	Accessory								
8608	OSC50809.1	-	polysaccharide deacetylase	0	1	0	0	0	0	0	0	0	0	0	1	Unique	COG0726_G#	32.6	74	5.54E-13	NA	30.7	48	0.0000665
8609	Prokka:JAPCLKMA_05602	-	hypothetical protein	1	1	1	1	1	1	1	1	1	1	1	11	CORE								
8610	WP_033272422.1	-	hypothetical protein	1	0	1	0	0	0	1	0	0	0	0	3	Accessory								
8611	AXI90646.1	-	hypothetical protein	0	0	0	1	0	0	0	0	0	0	0	1	Unique								
8612	OSC63097.1	-	hypothetical protein	1	1	1	1	1	1	1	1	1	1	1	11	CORE								

cluster	GI	gene	Annotation	Streptomyces_thermocarboxydus_BPSAC147	Streptomyces_sp._4F	Streptomyces_sp._BSE7F	Streptomyces_sp._ETH9427	Streptomyces_sp._FxanaD5	Streptomyces_sp._UNC401CLCol	Actinospica_acidiphila_NRRL_B_24431	Streptomyces_sp._HNS054	Streptomyces_sp._SMS_SU21	Streptomyces_sp._ZS0098	Streptomyces_thermocarboxydus_K155	Addition	Core-Accessory-Unique	COG	%Similarity_COG	Score_COG	Evalue_COG	KEGG	%Similarity_KEGG	Score_KEGG	Evalue_KEGG
8613	OSC61237.1	-	GNAT family N-acetyltransferase	0	1	0	0	0	0	0	0	0	0	0	1	Unique	COG0456_R#	30.8	45	0.000161				
8614	WP_033277408.1	-	hypothetical protein	1	0	1	0	1	1	1	1	1	1	1	9	Accessory								
8615	OSC63909.1	-	succinate dehydrogenase	1	1	1	1	1	1	1	1	1	1	1	11	CORE	COG2142_C#	59.6	170	5.68E-42	K00242	58.9	169	3.38E-41
8616	OSC65080.1	-	hypothetical protein	0	1	0	0	0	0	0	0	0	0	0	1	Unique								
8617	OSC56692.1	-	flavin oxidoreductase	0	1	0	0	0	0	0	0	0	0	0	1	Unique	COG1853_R#	43.3	118	2.56E-26	NA	43.3	118	6.84E-26
8618	WP_106959449.1	-	VOC family protein	1	0	1	1	1	1	1	1	1	0	1	9	Accessory	COG0346_E#	54.1	163	5.31E-40				
8619	WP_048455570.1	-	SRPBCC domain-containing protein	1	1	1	1	1	1	0	1	1	1	1	10	Accessory	COG3832_S#	27.9	60	4.82E-09				
8620	Prokka:JAPCLKMA_02432	-	hypothetical protein	1	0	1	1	1	1	1	1	1	1	0	9	Accessory								
8621	WP_078611349.1	-	GNAT family N-acetyltransferase	1	0	0	1	1	1	1	1	1	1	1	9	Accessory	COG0454_KR#	50.8	110	5.31E-24				
8622	WP_019527648.1	-	ribose-5-phosphate isomerase	1	1	1	1	1	1	1	1	1	1	1	11	CORE	COG0698_G#	63.5	216	5.26E-56	K01808	63.5	216	1.4E-55
8623	WP_106963283.1	-	hypothetical protein	1	0	1	1	1	1	1	1	1	1	1	10	Accessory								
8624	OSC68245.1	-	Appr-1-p processing protein	1	1	1	1	1	1	1	1	1	1	1	11	CORE					NA	57.4	183	1.32E-45

cluster	GI	gene	Annotation	Streptomyces_thermocarboxydus_BPSAC147	Streptomyces_sp._4F	Streptomyces_sp._BSE7F	Streptomyces_sp._ETH9427	Streptomyces_sp._FxanaD5	Streptomyces_sp._UNC401CLCol	Actinospica_acidiphila_NRRL_B_24431	Streptomyces_sp._HNS054	Streptomyces_sp._SMS_SU21	Streptomyces_sp._ZS0098	Streptomyces_thermocarboxydus_K155	Addition	Core-Accessory-Unique	COG	%Similarity_COG	Score_COG	Evalue_COG	KEGG	%Similarity_KEGG	Score_KEGG	Evalue_KEGG
8625	WP_048458134.1	-	6,7-dimethyl-8-ribityllumazine synthase	1	1	1	1	1	1	1	1	1	1	1	11	CORE	COG0054_H#	55.5	173	8.7E-43	K00794	55.5	173	2.32E-42
8626	WP_019525368.1	-	hypothetical protein	1	0	1	1	1	1	1	1	1	1	1	10	Accessory								
8627	WP_037643062.1	-	hypothetical protein	0	0	0	0	0	0	1	0	0	0	0	1	Unique								
8628	OSC62667.1	-	tRNA-specific adenosine deaminase	1	1	1	1	1	1	1	1	1	1	1	11	CORE	COG0590_FJ#	43.4	153	5.46E-37				
8629	WP_033276473.1	-	hypothetical protein	1	0	1	1	1	1	1	1	1	1	1	10	Accessory					K13909	34.4	52	0.00000598
8630	WP_147474330.1	-	hypothetical protein	0	0	1	0	0	0	0	0	1	1	0	3	Accessory								
8631	OSC57644.1	-	MFS transporter	0	1	0	0	0	0	0	0	0	0	0	1	Unique	COG0477_GEPR#	46.9	160	5.84E-39				
8632	WP_048455347.1	-	hypothetical protein	1	0	0	1	1	1	0	1	0	0	1	6	Accessory					NA	48	131	1.01E-29
8633	WP_019526380.1	-	DUF1877 family protein	0	0	0	0	1	1	0	0	0	0	0	2	Accessory								
8634	WP_086020098.1	-	transglycosylase SLT domain-containing protein	1	1	1	1	1	1	1	1	1	1	1	11	CORE								
8635	WP_147474373.1	-	hypothetical protein	0	0	0	0	0	0	0	0	0	1	0	1	Unique								
8636	WP_051101638.1	-	ATP-binding protein	1	0	1	0	1	0	0	1	0	1	0	5	Accessory								

cluster	GI	gene	Annotation	Streptomyces_thermocarboxydus_BPSAC147	Streptomyces_sp._4F	Streptomyces_sp._BSE7F	Streptomyces_sp._ETH9427	Streptomyces_sp._FxanaD5	Streptomyces_sp._UNC401CLCol	Actinospica_acidiphila_NRRL_B_24431	Streptomyces_sp._HNS054	Streptomyces_sp._SMS_SU21	Streptomyces_sp._ZS0098	Streptomyces_thermocarboxydus_K155	Addition	Core-Accessory-Unique	COG	%Similarity_COG	Score_COG	Evalue_COG	KEGG	%Similarity_KEGG	Score_KEGG	Evalue_KEGG
8637	OSC70560.1	-	MarR family transcriptional regulator	0	1	0	0	0	0	0	0	0	0	0	1	Unique								
8638	Prokka:JAPCLKMA_01539	-	hypothetical protein	1	0	0	1	0	0	0	0	0	0	0	2	Accessory								
8639	OSC70618.1	-	CrcB protein	1	1	1	1	1	1	1	1	1	1	1	11	CORE	COG0239_D#	39.3	84	5.32E-16	K06199	38.7	81	9.21E-15
8640	AXI91231.1	-	hypothetical protein	0	0	0	1	0	0	0	0	0	0	0	1	Unique								
8641	AXI91392.1	-	hypothetical protein	0	0	0	1	0	0	0	0	0	0	0	1	Unique								
8642	OSC69669.1	-	NUDIX hydrolase	0	1	0	0	0	0	0	0	0	0	0	1	Unique	COG1051_F#	27.9	52	0.00000131	NA	33.3	52	0.00000458
8643	OSC66689.1	-	hypothetical protein	1	1	0	0	1	1	0	0	0	0	1	5	Accessory								
8644	OSC49656.1	-	YfcE family phosphodiesterase	0	1	0	0	0	0	0	0	0	0	0	1	Unique	COG0639_T#	28.6	56	1.19E-07	NA	32.6	48	0.0000661
8645	WP_102640667.1	-	hypothetical protein	0	0	0	0	0	0	0	0	1	0	0	1	Unique	COG4278_S#	36.4	44	0.000467				
8646	Prokka:JAPCLKMA_00983	-	hypothetical protein	1	0	0	0	0	0	0	0	0	0	0	1	Unique								
8647	OSC64237.1	-	GNAT family N-acetyltransferase	1	1	1	1	1	1	1	1	1	1	1	11	CORE	COG0454_KR#	37.3	75	1.45E-13	NA	31.4	70	1.24E-11
8648	Prokka:JAPCLKMA_02006	-	hypothetical protein	1	0	0	0	0	0	0	0	0	0	0	1	Unique								
8649	AXI91395.1	-	hypothetical protein	0	0	0	1	0	0	0	0	0	0	0	1	Unique								
8650	WP_028960527.1	-	TIR domain-containing protein	0	0	0	0	0	1	0	0	0	0	0	1	Unique								

cluster	GI	gene	Annotation	Streptomyces_thermocarboxydus_BPSAC147	Streptomyces_sp._4F	Streptomyces_sp._BSE7F	Streptomyces_sp._ETH9427	Streptomyces_sp._FxanaD5	Streptomyces_sp._UNC401CLCol	Actinospica_acidiphila_NRRL_B_24431	Streptomyces_sp._HNS054	Streptomyces_sp._SMS_SU21	Streptomyces_sp._ZS0098	Streptomyces_thermocarboxydus_K155	Addition	Core-Accessory-Unique	COG	%Similarity_COG	Score_COG	Evalue_COG	KEGG	%Similarity_KEGG	Score_KEGG	Evalue_KEGG
8651	AXI91399.1	-	hypothetical protein	0	0	0	1	0	0	0	0	0	0	0	1	Unique								
8652	AXI85239.1	-	polyketide cyclase	1	1	1	1	1	1	1	1	1	1	1	11	CORE								
8653	OSC62098.1	-	GNAT family N-acetyltransferase	1	1	1	1	1	1	1	1	1	1	1	11	CORE	COG0454_KR#	42.9	114	3.65E-25	NA	35.8	91	8.84E-18
8654	WP_048454828.1	-	hypothetical protein	1	1	1	1	1	1	1	1	1	1	1	11	CORE								
8655	WP_028960550.1	-	hypothetical protein	1	0	0	0	0	1	0	0	0	0	1	3	Accessory								
8656	WP_019523251.1	-	hypothetical protein	0	0	0	0	1	0	0	0	0	0	0	1	Unique								
8657	WP_003953493.1	-	CarD family transcriptional regulator	1	1	1	1	1	1	1	1	1	1	1	11	CORE	COG1329_K#	85	271	1.79E-72	K07736	85	271	4.77E-72
8658	AXI85330.1	-	hypothetical protein	1	0	0	1	0	0	0	0	0	0	0	2	Accessory								
8659	WP_033273279.1	-	NUDIX hydrolase	1	1	1	1	1	1	1	1	1	1	1	11	CORE								
8660	Prokka:JAPCLKMA_03348	-	hypothetical protein	1	1	1	1	1	1	1	1	1	1	1	11	CORE								
8661	WP_048455229.1	-	DNA starvation/stationary phase protection protein	1	1	1	1	1	1	1	1	1	1	1	11	CORE	COG0783_P#	34.6	87	4.78E-17	K04047	28.5	67	1.05E-10
8662	WP_102639497.1	-	helix-turn-helix transcriptional regulator	0	0	1	1	0	0	1	1	1	1	0	6	Accessory								

cluster	GI	gene	Annotation	Streptomyces_thermocarboxydus_BPSAC147	Streptomyces_sp._4F	Streptomyces_sp._BSE7F	Streptomyces_sp._ETH9427	Streptomyces_sp._FxanaD5	Streptomyces_sp._UNC401CLCol	Actinospica_acidiphila_NRRL_B_24431	Streptomyces_sp._HNS054	Streptomyces_sp._SMS_SU21	Streptomyces_sp._ZS0098	Streptomyces_thermocarboxydus_K155	Addition	Core-Accessory-Unique	COG	%Similarity_COG	Score_COG	Evalue_COG	KEGG	%Similarity_KEGG	Score_KEGG	Evalue_KEGG
8663	OSC56638.1	-	ribose-5-phosphate isomerase	0	1	0	0	0	0	0	0	0	0	0	1	Unique	COG0698_G#	45.9	122	1.75E-27	K01808	38.9	109	2.4E-23
8664	WP_122215701.1	-	helix-turn-helix transcriptional regulator	0	0	0	0	0	0	0	0	0	1	0	1	Unique	COG1733_K#	40.7	118	2.53E-26	NA	41.1	94	1.36E-18
8665	WP_028960803.1	-	hypothetical protein	0	0	0	0	0	1	0	0	0	0	0	1	Unique								
8666	WP_026243918.1	-	hypothetical protein	0	0	0	0	1	0	0	0	0	0	0	1	Unique								
8667	WP_019524870.1	-	hypothetical protein	0	0	0	0	1	1	0	0	0	0	1	3	Accessory								
8668	WP_019524888.1	-	hypothetical protein	0	0	0	0	1	0	0	0	0	0	0	1	Unique								
8669	WP_078493123.1	-	LysR family transcriptional regulator	0	0	0	0	0	0	1	0	0	0	0	1	Unique	COG0583_K#	59.3	68	3E-11	NA	57.6	65	5.19E-10
8670	Prokka:CIODPCDJ_03503	-	hypothetical protein	0	0	0	0	0	0	0	0	0	0	1	1	Unique								
8671	OSC62536.1	-	hypothetical protein	0	1	0	0	0	0	0	0	0	0	0	1	Unique								
8672	WP_048455385.1	-	hypothetical protein	0	0	0	1	0	0	0	1	0	1	0	3	Accessory								
8673	WP_033275408.1	-	GNAT family N-acetyltransferase	1	1	1	1	1	1	1	1	1	1	1	11	CORE	COG0454_KR#	40.4	121	3.9E-27				
8674	OSC70898.1	-	AsnC family transcriptional regulator	1	1	1	1	1	1	1	1	1	1	1	11	CORE	COG1522_K#	36.2	92	1.49E-18	K03718	27.5	72	3.25E-12
8675	OSC61379.1	-	hypothetical protein	0	1	0	0	0	0	0	0	0	0	0	1	Unique								

cluster	GI	gene	Annotation	Streptomyces_thermocarboxydus_BPSAC147	Streptomyces_sp._4F	Streptomyces_sp._BSE7F	Streptomyces_sp._ETH9427	Streptomyces_sp._FxanaD5	Streptomyces_sp._UNC401CLCol	Actinospica_acidiphila_NRRL_B_24431	Streptomyces_sp._HNS054	Streptomyces_sp._SMS_SU21	Streptomyces_sp._ZS0098	Streptomyces_thermocarboxydus_K155	Addition	Core-Accessory-Unique	COG	%Similarity_COG	Score_COG	Evalue_COG	KEGG	%Similarity_KEGG	Score_KEGG	Evalue_KEGG
8676	OSC69083.1	-	MerR family transcriptional regulator	1	1	1	1	1	1	1	1	1	1	1	11	CORE	COG0789_K#	39.3	96	1.03E-19	NA	39.3	96	2.75E-19
8677	Prokka:CIODPCDJ_05447	-	hypothetical protein	1	1	1	1	1	1	1	1	1	1	1	11	CORE	COG0640_K#	53.5	118	2.51E-26	K03892	53.5	118	6.71E-26
8678	OSC48529.1	-	amidohydrolase	0	1	0	0	0	0	0	0	0	0	0	1	Unique	COG1574_R#	41.3	109	8.94E-24	K07047	41.3	109	2.39E-23
8679	AXI91361.1	-	hypothetical protein	1	0	0	1	0	0	0	0	0	0	0	2	Accessory								
8680	WP_093767074.1	smpB	SsrA-binding protein SmpB	1	1	1	1	1	1	1	1	1	1	1	11	CORE	COG0691_O#	52.6	178	1.57E-44	K03664	52.9	173	1.76E-42
8681	WP_102638593.1	-	ASCH domain-containing protein	1	0	1	1	1	1	1	1	1	0	1	9	Accessory	COG4405_S#	30.8	66	1.13E-10				
8682	WP_048459708.1	-	GNAT family N-acetyltransferase	1	0	1	1	1	1	1	1	1	1	1	10	Accessory	COG0454_KR#	42.1	107	5.79E-23				
8683	Prokka:JAPCLKMA_05050	-	hypothetical protein	1	1	0	1	1	1	1	1	1	1	1	10	Accessory								
8684	OSC69061.1	-	anti-anti-sigma factor	1	1	1	1	1	1	1	1	1	1	1	11	CORE								
8685	WP_102640517.1	-	cation:proton antiporter regulatory subunit	0	0	0	1	0	0	0	1	1	1	0	4	Accessory	COG0490_P#	70.4	233	7E-61	K07228	70.4	233	1.87E-60
8686	WP_033275810.1	-	hypothetical protein	1	0	1	1	1	1	1	1	1	1	1	10	Accessory								

cluster	GI	gene	Annotation	Streptomyces_thermocarboxydus_BPSAC147	Streptomyces_sp._4F	Streptomyces_sp._BSE7F	Streptomyces_sp._ETH9427	Streptomyces_sp._FxanaD5	Streptomyces_sp._UNC401CLCol	Actinospica_acidiphila_NRRL_B_24431	Streptomyces_sp._HNS054	Streptomyces_sp._SMS_SU21	Streptomyces_sp._ZS0098	Streptomyces_thermocarboxydus_K155	Addition	Core-Accessory-Unique	COG	%Similarity_COG	Score_COG	Evalue_COG	KEGG	%Similarity_KEGG	Score_KEGG	Evalue_KEGG
8687	WP_122216612.1	-	diadenosine tetraphosphate hydrolase	0	0	0	0	1	0	0	1	0	1	0	3	Accessory	COG0537_FGR#	34.2	81	3.4E-15				
8688	AXI91299.1	-	hypothetical protein	0	0	0	1	0	0	0	0	0	0	0	1	Unique								
8689	AXI91316.1	-	hypothetical protein	0	0	0	1	0	0	0	0	0	0	0	1	Unique								
8690	Prokka:CIODPCDJ_02909	-	hypothetical protein	1	0	1	1	1	1	1	1	1	1	1	10	Accessory								
8691	OSC57202.1	-	hypothetical protein	0	1	0	0	0	0	0	0	0	0	0	1	Unique								
8692	OSC70572.1	-	replication initiation protein, RepL1	0	1	0	0	0	0	0	0	0	0	0	1	Unique								
8693	WP_026243836.1	-	ATP-binding protein	1	1	1	1	1	1	1	1	1	1	1	11	CORE								
8694	PWE11046.1	-	transcriptional regulator	0	0	1	0	0	0	0	0	0	0	0	1	Unique	COG1846_K#	31.2	74	4.16E-13	NA	30.3	57	1.84E-07
8695	WP_048456406.1	-	Lrp/AsnC family transcriptional regulator	1	1	1	1	1	1	1	1	1	1	1	11	CORE	COG1522_K#	36.2	83	8.96E-16	K03718	27.8	75	4.99E-13
8696	WP_102640445.1	-	hypothetical protein	0	0	0	0	0	0	0	1	1	0	0	2	Accessory								
8697	PWE10867.1	-	hypothetical protein	1	0	1	1	1	1	1	1	0	0	1	8	Accessory					NA	32	48	0.0000653
8698	OSC73528.1	-	hypothetical protein	0	1	0	0	0	0	0	0	0	0	0	1	Unique					NA	38.2	62	4.37E-09
8699	OSC66078.1	-	hypothetical protein	1	1	1	1	1	1	1	1	1	1	1	11	CORE					NA	33.1	66	3.03E-10
8700	OSC69073.1	-	hypothetical protein	1	1	1	1	1	1	1	1	1	1	1	11	CORE					NA	50.3	142	4.34E-33

cluster	GI	gene	Annotation	Streptomyces_thermocarboxydus_BPSAC147	Streptomyces_sp._4F	Streptomyces_sp._BSE7F	Streptomyces_sp._ETH9427	Streptomyces_sp._FxanaD5	Streptomyces_sp._UNC401CLCol	Actinospica_acidiphila_NRRL_B_24431	Streptomyces_sp._HNS054	Streptomyces_sp._SMS_SU21	Streptomyces_sp._ZS0098	Streptomyces_thermocarboxydus_K155	Addition	Core-Accessory-Unique	COG	%Similarity_COG	Score_COG	Evalue_COG	KEGG	%Similarity_KEGG	Score_KEGG	Evalue_KEGG
8701	OSC71442.1	-	hypothetical protein	1	1	1	1	1	1	1	1	1	1	1	11	CORE								
8702	OSC72833.1	-	hypothetical protein	0	1	0	0	0	0	0	0	0	0	0	1	Unique								
8703	WP_019526991.1	-	hypothetical protein	0	0	0	0	1	0	0	0	0	0	0	1	Unique								
8704	WP_006130189.1	-	MarR family transcriptional regulator	1	1	1	1	1	1	1	1	1	1	1	11	CORE	COG1846_K#	33.9	84	5.22E-16	NA	24.1	45	0.000549
8705	Prokka:CIODPCDJ_00817	glpE_2	Thiosulfate sulfurtransferase GlpE	1	1	1	1	1	1	1	1	1	1	1	11	CORE	COG0607_P#	51.1	106	1.28E-22	NA	32.1	55	5.31E-07
8706	Prokka:CIODPCDJ_01329	-	hypothetical protein	1	1	1	1	1	1	1	1	1	1	1	11	CORE	COG0454_KR#	40.6	102	1.85E-21				
8707	WP_122216554.1	-	glyoxalase	1	0	1	1	1	1	1	1	1	1	1	10	Accessory	COG0346_E#	56	174	2.25E-43				
8708	Prokka:CIODPCDJ_00086	iorA	Isoquinoline 1-oxidoreductase subunit alpha	1	0	0	0	1	1	0	0	0	0	1	4	Accessory	COG2080_C#	51	159	9.77E-39	K13483	41.4	107	9.01E-23
8709	Prokka:CIODPCDJ_02439	-	hypothetical protein	0	0	0	0	0	1	0	0	0	0	1	2	Accessory	COG3727_L#	38.6	100	5.39E-21				
8710	WP_106963299.1	-	hypothetical protein	0	0	0	0	0	0	1	0	0	0	0	1	Unique								
8711	WP_033273333.1	-	MarR family transcriptional regulator	0	0	0	0	0	0	1	0	0	0	0	1	Unique	COG1846_K#	28.4	58	3.07E-08	NA	31.5	76	2.91E-13
8712	AXI89636.1	-	hypothetical protein	0	0	0	1	0	0	0	0	0	0	0	1	Unique								

cluster	GI	gene	Annotation	Streptomyces_thermocarboxydus_BPSAC147	Streptomyces_sp._4F	Streptomyces_sp._BSE7F	Streptomyces_sp._ETH9427	Streptomyces_sp._FxanaD5	Streptomyces_sp._UNC401CLCol	Actinospica_acidiphila_NRRL_B_24431	Streptomyces_sp._HNS054	Streptomyces_sp._SMS_SU21	Streptomyces_sp._ZS0098	Streptomyces_thermocarboxydus_K155	Addition	Core-Accessory-Unique	COG	%Similarity_COG	Score_COG	Evalue_COG	KEGG	%Similarity_KEGG	Score_KEGG	Evalue_KEGG
8713	OSC70211.1	-	hypothetical protein	1	1	1	1	1	1	1	1	1	1	1	11	CORE								
8714	PWE11016.1	-	hypothetical protein	0	0	1	0	0	0	0	0	1	0	0	2	Accessory	COG1846_K#	28.1	42	0.00227	NA	28.1	42	0.00607
8715	AXI84853.1	-	cyclase	1	1	1	1	1	1	1	1	1	1	1	11	CORE								
8716	WP_019524616.1	-	nitroreductase family deazaflavin-dependent oxidoreductase	1	0	1	1	1	1	1	1	1	1	1	10	Accessory					NA	35.1	45	0.000421
8717	WP_078895178.1	-	hypothetical protein	0	0	0	0	0	0	0	1	0	0	0	1	Unique								
8718	WP_026243766.1	-	ATP-binding protein	0	0	0	1	1	1	0	0	0	0	0	3	Accessory								
8719	Prokka:JAPCLKMA_00948	tphA3I	Terephthalate 1,2-dioxygenase, terminal oxygenase component subunit beta 1	1	0	0	0	1	1	0	0	0	0	1	4	Accessory	COG5517_Q#	28.2	82	1.52E-15				
8720	WP_026243372.1	-	GNAT family N-acetyltransferase	1	0	0	0	1	1	1	0	0	0	1	5	Accessory								
8721	WP_051724629.1	-	MarR family transcriptional regulator	0	0	0	0	0	0	1	0	0	1	0	2	Accessory	COG1846_K#	32.7	89	9.51E-18				

cluster	GI	gene	Annotation	Streptomyces_thermocarboxydus_BPSAC147	Streptomyces_sp._4F	Streptomyces_sp._BSE7F	Streptomyces_sp._ETH9427	Streptomyces_sp._FxanaD5	Streptomyces_sp._UNC401CLCol	Actinospica_acidiphila_NRRL_B_24431	Streptomyces_sp._HNS054	Streptomyces_sp._SMS_SU21	Streptomyces_sp._ZS0098	Streptomyces_thermocarboxydus_K155	Addition	Core-Accessory-Unique	COG	%Similarity_COG	Score_COG	Evalue_COG	KEGG	%Similarity_KEGG	Score_KEGG	Evalue_KEGG
8722	OSC64031.1	-	GNAT family N-acetyltransferase	0	1	0	0	0	0	0	0	0	0	0	1	Unique	COG1670_J#	53.2	149	1.32E-35	NA	41.5	115	4.32E-25
8723	WP_033274341.1	-	hypothetical protein	0	0	0	0	0	0	1	0	0	0	0	1	Unique	COG5637_S#	39.3	47	0.0000707				
8724	Prokka:JAPCLKMA_03873	-	hypothetical protein	1	0	1	0	0	0	0	0	0	0	0	2	Accessory								
8725	Prokka:JAPCLKMA_06009	-	hypothetical protein	1	0	0	0	0	0	0	0	0	0	0	1	Unique					NA	33.7	42	0.00607
8726	AXI87241.1	-	hypothetical protein	0	0	0	1	0	0	0	0	0	0	0	1	Unique								
8727	WP_102639046.1	aroQ	type II 3-dehydroquinate dehydratase	1	1	1	1	1	1	1	1	1	1	1	11	CORE	COG0757_E#	68.8	185	1.65E-46	K03786	48.6	138	4.74E-32
8728	Prokka:CIODPCDJ_06271	cutS	Carbon monoxide dehydrogenase small chain	1	0	0	0	1	1	1	0	0	0	1	5	Accessory	COG2080_C#	60.6	199	8.47E-51	K03518	58	191	4.72E-48
8729	WP_004934251.1	-	bifunctional nuclease family protein	1	1	1	1	1	1	1	1	1	1	1	11	CORE	COG1259_S#	61.5	205	1.54E-52	K08999	60.2	202	2.67E-51
8730	WP_028961390.1	-	hypothetical protein	1	0	1	1	1	1	1	1	1	1	1	10	Accessory								
8731	OSC61776.1	-	hypothetical protein	0	1	0	0	0	0	0	0	0	0	0	1	Unique								
8732	WP_033277432.1	-	hypothetical protein	0	0	1	0	0	0	1	0	1	0	0	3	Accessory								

cluster	GI	gene	Annotation	Streptomyces_thermocarboxydus_BPSAC147	Streptomyces_sp._4F	Streptomyces_sp._BSE7F	Streptomyces_sp._ETH9427	Streptomyces_sp._FxanaD5	Streptomyces_sp._UNC401CLCol	Actinospica_acidiphila_NRRL_B_24431	Streptomyces_sp._HNS054	Streptomyces_sp._SMS_SU21	Streptomyces_sp._ZS0098	Streptomyces_thermocarboxydus_K155	Addition	Core-Accessory-Unique	COG	%Similarity_COG	Score_COG	Evalue_COG	KEGG	%Similarity_KEGG	Score_KEGG	Evalue_KEGG
8733	OSC63367.1	-	MarR family transcriptional regulator	1	1	0	0	1	1	1	0	0	0	1	6	Accessory								
8734	Prokka:CIODPCDJ_06615	-	hypothetical protein	0	0	1	1	1	1	1	1	1	1	1	9	Accessory								
8735	WP_033278035.1	-	hypothetical protein	1	1	1	1	1	1	1	1	1	1	1	11	CORE								
8736	WP_019527247.1	-	membrane protein	1	1	1	1	1	1	1	1	1	1	1	11	CORE								
8737	OSC72251.1	-	nitroreductase	1	1	1	1	1	1	1	1	1	1	1	11	CORE					NA	58	138	8.08E-32
8738	WP_048456336.1	-	C40 family peptidase	1	1	1	1	1	1	1	1	1	1	1	11	CORE	COG0791_M#	44.6	99	1.19E-20	NA	40.4	91	1.13E-17
8739	OSC66348.1	-	MxaD family protein	1	1	1	1	1	1	1	1	1	1	1	11	CORE								
8740	PWE07982.1	-	hypothetical protein	0	0	1	1	0	0	0	0	1	1	0	4	Accessory								
8741	OSC52614.1	-	hypothetical protein	1	1	1	1	1	1	1	1	1	1	1	11	CORE								
8742	WP_122215255.1	-	hypothetical protein	1	1	1	1	1	1	1	1	1	1	1	11	CORE								
8743	OSC72750.1	-	ABC transporter	0	1	0	0	0	0	0	0	0	0	0	1	Unique	COG1132_V#	49	137	5.16E-32	K06148	49	137	1.38E-31
8744	WP_019525198.1	-	hypothetical protein	1	0	0	1	1	1	1	1	1	1	1	9	Accessory								
8745	Prokka:JAPCLKMA_04409	-	hypothetical protein	1	0	1	1	1	1	1	1	1	1	1	10	Accessory	COG1742_S#	72.5	181	2.39E-45	K09771	72.5	181	6.37E-45
8746	Prokka:JAPCLKMA_01268	-	hypothetical protein	1	1	1	1	1	1	1	1	1	1	1	11	CORE								
8747	WP_122213802.1	-	hypothetical protein	0	0	0	0	0	0	0	0	0	1	0	1	Unique								

cluster	GI	gene	Annotation	Streptomyces_thermocarboxydus_BPSAC147	Streptomyces_sp._4F	Streptomyces_sp._BSE7F	Streptomyces_sp._ETH9427	Streptomyces_sp._FxanaD5	Streptomyces_sp._UNC401CLCol	Actinospica_acidiphila_NRRL_B_24431	Streptomyces_sp._HNS054	Streptomyces_sp._SMS_SU21	Streptomyces_sp._ZS0098	Streptomyces_thermocarboxydus_K155	Addition	Core-Accessory-Unique	COG	%Similarity_COG	Score_COG	Evalue_COG	KEGG	%Similarity_KEGG	Score_KEGG	Evalue_KEGG
8748	OSC56246.1	-	hypothetical protein	1	1	0	0	0	0	0	0	0	0	0	2	Accessory								
8749	PWE09860.1	-	hypothetical protein	1	1	1	1	1	1	1	1	1	1	1	11	CORE								
8750	OSC61236.1	-	mannitol dehydrogenase	0	1	0	0	0	0	0	0	0	0	0	1	Unique	COG0246_G#	35.5	101	2.4E-21	K00540	41.6	99	2.44E-20
8751	OSC58528.1	-	hypothetical protein	1	1	1	1	1	1	1	1	1	1	1	11	CORE								
8752	WP_122216610.1	-	peptidoglycan-binding protein	0	0	0	0	0	0	0	0	0	1	0	1	Unique	COG3409_M#	37.1	59	1.79E-08	K01447	32.3	50	0.000017
8753	WP_033272377.1	-	hypothetical protein	0	1	0	0	0	0	1	0	0	0	0	2	Accessory								
8754	WP_048460715.1	-	ATP-binding protein	0	0	1	1	0	0	0	1	1	1	0	5	Accessory								
8755	OSC61166.1	-	hypothetical protein	1	1	1	1	1	1	1	1	1	1	1	11	CORE								
8756	OSC70190.1	-	hypothetical protein	0	1	0	0	0	0	0	0	0	0	0	1	Unique	COG2315_S#	44.1	60	8.02E-09	NA	40.7	55	6.89E-07
8757	PWE07264.1	-	hypothetical protein	0	0	1	0	0	0	0	0	0	0	0	1	Unique								
8758	Prokka:CIODPCDJ_05065	-	hypothetical protein	1	1	1	1	1	1	1	1	1	1	1	11	CORE					NA	36.2	77	1.68E-13
8759	AXI85590.1	-	transcriptional regulator	1	0	1	1	1	1	1	1	1	1	1	10	Accessory	COG1733_K#	46.9	134	2.55E-31	NA	45.8	129	3.73E-29
8760	Prokka:CIODPCDJ_00158	-	hypothetical protein	1	1	0	0	1	1	1	0	1	0	1	7	Accessory								
8761	WP_048458848.1	-	hypothetical protein	0	0	0	1	0	0	0	1	0	0	0	2	Accessory								
8762	Prokka:JAPCLKMA_01153	rpsG	30S ribosomal protein S7	1	1	1	1	1	1	1	1	1	1	1	11	CORE	COG0049_J#	80.8	263	3.63E-70	K02992	80.8	263	9.7E-70

cluster	GI	gene	Annotation	Streptomyces_thermocarboxydus_BPSAC147	Streptomyces_sp._4F	Streptomyces_sp._BSE7F	Streptomyces_sp._ETH9427	Streptomyces_sp._FxanaD5	Streptomyces_sp._UNC401CLCol	Actinospica_acidiphila_NRRL_B_24431	Streptomyces_sp._HNS054	Streptomyces_sp._SMS_SU21	Streptomyces_sp._ZS0098	Streptomyces_thermocarboxydus_K155	Addition	Core-Accessory-Unique	COG	%Similarity_COG	Score_COG	Evalue_COG	KEGG	%Similarity_KEGG	Score_KEGG	Evalue_KEGG
8763	WP_033276265.1	-	SnoaL-like domain-containing protein	0	0	0	0	1	1	1	1	0	1	1	6	Accessory								
8764	AXI87163.1	-	RidA family protein	1	1	1	1	1	1	1	1	1	1	1	11	CORE	COG0251_J#	65.3	195	1.59E-49	NA	66.4	190	1.36E-47
8765	Prokka:JAPCLKMA_01836	-	hypothetical protein	1	1	1	1	1	1	1	1	1	1	1	11	CORE								
8766	AXI85295.1	-	NUDIX hydrolase	0	0	0	1	0	0	0	0	0	0	0	1	Unique	COG1051_F#	36.6	60	4.67E-09	NA	35.9	60	1.63E-08
8767	WP_033275898.1	-	GNAT family N-acetyltransferase	1	0	0	0	1	1	1	0	1	1	1	7	Accessory	COG0454_KR#	35.8	65	1.45E-10	NA	34.9	47	0.000143
8768	Prokka:CIODPCDJ_03183	-	hypothetical protein	1	1	0	1	1	1	1	1	0	1	1	9	Accessory					K07005	39.5	83	1.8E-15
8769	Prokka:JAPCLKMA_02835	-	hypothetical protein	1	0	0	0	1	1	0	0	0	0	1	4	Accessory								
8770	OSC48960.1	-	Tat pathway signal sequence domain protein	0	1	0	0	0	0	0	0	0	0	0	1	Unique								
8771	WP_106959434.1	-	multicopper oxidase domain-containing protein	0	0	0	0	1	1	0	0	0	0	1	3	Accessory								

cluster	GI	gene	Annotation	Streptomyces_thermocarboxydus_BPSAC147	Streptomyces_sp._4F	Streptomyces_sp._BSE7F	Streptomyces_sp._ETH9427	Streptomyces_sp._FxanaD5	Streptomyces_sp._UNC401CLCol	Actinospica_acidiphila_NRRL_B_24431	Streptomyces_sp._HNS054	Streptomyces_sp._SMS_SU21	Streptomyces_sp._ZS0098	Streptomyces_thermocarboxydus_K155	Addition	Core-Accessory-Unique	COG	%Similarity_COG	Score_COG	Evalue_COG	KEGG	%Similarity_KEGG	Score_KEGG	Evalue_KEGG
8772	OSC69200.1	-	SUF system NifU family Fe-S cluster assembly protein	1	1	1	1	1	1	1	1	1	1	1	11	CORE	COG0822_C#	57.6	178	2.62E-44	K04488	57.6	178	7E-44
8773	OSC73293.1	-	ribosome-binding factor A	1	1	1	1	1	1	1	1	1	1	1	11	CORE	COG0858_J#	51	138	3.01E-32	K02834	51	138	8.03E-32
8774	WP_122214337.1	-	hypothetical protein	0	0	0	0	0	0	1	1	0	1	0	3	Accessory								
8775	WP_106432487.1	-	ATP-binding protein	1	0	0	0	1	1	1	0	0	0	1	5	Accessory								
8776	AXI87195.1	-	MarR family transcriptional regulator	0	1	1	1	0	0	1	1	1	1	0	7	Accessory	COG1846_K#	44.9	85	1.77E-16	NA	41	74	8.35E-13
[truncated: 479,932 more chars]
